# Red blood cells release microparticles containing human argonaute 2 and miRNAs to target genes of *Plasmodium falciparum*

**DOI:** 10.1038/emi.2017.63

**Published:** 2017-08-23

**Authors:** Zhensheng Wang, Juemin Xi, Xiao Hao, Weiwei Deng, Juan Liu, Chunyan Wei, Yuhui Gao, Lianhui Zhang, Heng Wang

**Affiliations:** 1 Department of Microbiology and Parasitology, Institute of Basic Medical Sciences/Chinese Academy of Medical Sciences, School of Basic Medicine/Peking Union Medical College, Beijing 100005, China

**Keywords:** human argonaute 2, malaria, microparticle, miRNA, *Plasmodium falciparum*

## Abstract

Red blood cells (RBCs) are known to function as a refuge for providing food resources and as a shelter against the host’s immune system after malaria parasite (*Plasmodium*) infection. Recent studies have reported significant production of extracellular vesicles (microparticles, MPs) in the circulation of malaria patients. However, it is unclear how these extracellular vesicles are generated and what their biological functions are. In this study, we isolated the MPs from a culture medium of normal RBCs and malaria parasite-infected RBCs (iRBCs), compared their quantity and origins, and profiled their miRNAs by deep sequencing. We found a much larger number of MPs released in the culture of iRBCs than in the culture of normal RBCs. Further investigation indicated that, in these MPs, human argonaute 2 (hAgo2) was found to bind to hundreds of miRNAs. These hAgo2-miRNA complexes were transferred into the parasites, and the expression of an essential malaria antigen, PfEMP1, was downregulated by miR-451/140 through its binding to the A and B subgroups of *var* genes, a family of genes encoding PfEMP1. Our data suggest for the first time that, through the release of MPs, mature RBCs present an innate resistance to malaria infection. These studies also shed new light on the reason why RBCs’ genetic mutation occurs mainly in populations living in intensive malaria endemic areas and on the possibility of using miRNAs as novel medicines for malaria patients.

## INTRODUCTION

Red blood cells (RBCs) are essential in vertebrates to deliver oxygen (O_2_) to the body tissues through the circulatory system. Mature RBCs have no nuclei and lack most organelles, and their cytoplasm is filled with hemoglobin (Hb). Pathogens rarely invade RBCs as a host cell. However, hemosporina parasites, especially *Plasmodium*, the malaria parasite, exclusively target RBCs after leaving the hepatocytes and entering the bloodstream. RBCs have long been recognized as an ideal refuge for these parasite invaders, providing them not only with abundant food resources, but also with shelter from host immune attacks. One reason behind this phenomenon is that mature RBCs, lacking major histocompatibility complex I,^1, 2^ are therefore unable to present any pathogen-derived epitopes to immune cells for a protective immune response.

Malaria is still the most prevalent and devastating parasitic disease in tropical and subtropical areas. Among the five species of *Plasmodium* that cause human malaria, *Plasmodium falciparum* (*P. falciparum*) infection caused 99% death in human malaria cases worldwide in 2015 (World Malaria Report 2015, WHO). In malaria endemic areas where *Plasmodium* infection is thought to have a history of longer than 10 thousand years, some populations, by way of increasing resistance to malaria, have developed protective genetic mutations that lead to altered protein expression in RBCs, such as hemoglobin S (HbS) (the basis of sickle cell anemia)^3, 4, 5, 6^ and glucose-6-phosphate dehydrogenase deficiency.^7, 8^ Such genetic resistance is inheritable, suggesting that RBCs are at the forefront of responding to the selective pressure imposed by malaria.

Extracellular vesicles have been suggested to play essential roles in the transportation of molecules among cells for signal transduction, transcriptional regulation during immune responses^9^ and inflammatory reactions.^10^ In recent years, elevated levels of circulating microparticles (MPs) have been detected in patients with malaria, and higher levels of MPs seemed to be linked to the progress of immunopathological lesions in cerebral malaria.^11, 12, 13^ Mirroring *in vivo* findings, data from *in vitro* culture have shown that exosome-like vesicles derived from *P. falciparum*-infected RBCs (iRBCs) act as messengers, setting up communication between parasites during asexual blood stages and helping direct the parasites to develop into sexual stages.^14^ Data from mouse models also show that reticulocyte-derived vesicles that contain parasite protein are involved in modulating the immune response during *Plasmodium yoelii* (*P. yoelii*) infection and provide immunoprotection for the host against the attack of a lethal *P. yoelii* strain.^15^ Most importantly, all these studies provide evidence that, during the blood stage of *Plasmodium* infection, MPs are involved in cell-cell communication and host immunoregulation and that iRBCs can release MPs to communicate with other host cells, especially immune cells, for protection against parasite infection.^12^ In addition, it has been discovered that, in RBCs of sickle cell anemia patients, human miR-451 and let-7i are highly enriched, both of which are capable of targeting the mRNAs of malaria parasites to inhibit their translation.^16^ Interestingly, both exosome-like vesicles and human miRNAs were separately demonstrated as the regulatory factors involved in the sexual form differentiation of *Plasmodium* parasites. It is unclear whether there is an intrinsic connection among these vesicles and human miRNAs during malaria infection.

The functions of RBC-derived MPs have been rarely illustrated previously due to the diversity of MPs in the *in vivo* system. In this study, we investigated the cargo transfer ability of RBC-derived MPs during *in vitro* culture of the asexual erythrocytic stages of *P. falciparum*, attempted to identify the miRNA complexes within the MPs, and studied the effects of two miRNAs, miR-451 and miR-140, on the expression of *P. falciparum var* genes. Our data demonstrated that MPs that are abundantly released from RBCs during the blood stage of malaria infection are able to transfer hAgo2-miRNA complexes into the parasites within iRBCs. Among these miRNAs, miR-451 and miR-140 targeted the mRNAs of a critical parasite antigen, *P. falciparum* erythrocyte membrane protein-1 (PfEMP1), and downregulated its expression.

## MATERIALS AND METHODS

### Ethics statement

All animal work was approved by the Ethical Inspection Committee of the Institute of Basic Medical Sciences/Chinese Academy of Medical Sciences (Permission number: ACUC-A02-2013-038) and was performed in accordance with the Regulations for the Administration of Affairs Concerning Experimental Animals in China (14 November 1988).

### Maintenance, synchronization and collection of *Plasmodium* parasites

The *P. falciparum* 3D7 strain was obtained from ATCC (Manassas, VA, USA) and was maintained in fresh human RBCs at 5% hematocrit using RPMI-HEPES supplemented with 0.5% (w/v) Albumax II (Invitrogen, Carlsbad, CA, USA) as described previously.^17^ The *P. falciparum* parasites were synchronized at the ring stage using 5% sorbitol following a previously described protocol.^18^ Late-stage infected RBCs were enriched with 50% Nycodenz (Sigma-Aldrich, Shanghai, China). The *P. yoelii* 17XL strain was maintained in female Balb/c mice aged 6–8 weeks. Infections were initiated by intraperitoneal injection of 2 × 10^5^ *P. yoelii*-infected RBCs. Parasitemia was monitored by Giemsa-stained blood smears.

### Purification and isolation of MPs

Generally, a total of 5 × 10^9^ RBCs, including 3%–5% iRBCs, were used to release MPs in the parasite-exposed medium (referred to as iMPs hereafter), and these RBCs were cultured for 24 h in 15 mL of medium before iMP isolation. By contrast, MPs in the medium culturing an equal number of normal RBCs (referred to as nMPs hereafter) under the same conditions were set as controls. MPs were isolated from 15 mL of culture supernatant as follows. The collected supernatant was centrifuged at 800*g* to remove cells and again at 3000*g* to remove the remaining debris, followed by filtration through a 3-μm sterilized filter (Millipore Co., Billerica, MA, USA), removing all particles >3 μm, such as cell debris and apoptotic bodies. MPs were pelleted by centrifugation for 2 h at 20 000*g* at 4 °C and were then resuspended in 150 μL PBS or fresh parasite culture medium for storage.

### RBC labeling, live fluorescence microscopy and indirect immunofluorescence assay

Fluorescein isothiocyanate (FITC) labeling of mature normal RBCs (nRBCs) was performed as described previously.^19^ The FITC-labeled nRBCs (FITC-nRBCs) were cultured in *P. falciparum* complete culture medium before they were used in FITC-Dextran transfer assays. For light and fluorescence microscopy, parasite-infected RBCs were stained with 4′,6-diamidino-2-phenylindole (DAPI, Sigma-Aldrich) in PBS and were examined at the indicated time points using a Leica DM2000 fluorescence microscope (Leica, Bensheim, Germany). For indirect immunofluorescence assay, thin blood smears were incubated with a mixture of anti-Ago2 mouse monoclonal antibody (ab 57113, Abcam, Cambriage, MA, USA) and anti-parasite cytoplasm rat monoclonal antibody 5H2C6V (maintained in our lab). Normal mouse IgG (Santa Cruz Biotechnology, Inc., Santa Cruz, CA, USA) was used as a negative control. FITC-conjugated goat anti-mouse IgG and AlexaFluor 594-conjugated donkey anti-rat IgG were used as secondary antibodies. The slides were mounted with mounting medium containing DAPI (ZSGB-bio, Beijing, China) and were analyzed using a confocal Leica TCS-SP2 fluorescence microscope (Leica). The transfer potential of FITC-Dextran directly toward RBCs was evaluated with 5 × 10^7^ nRBCs cultured in medium containing 0.5 mg/mL FITC-Dextran for 24 or 72 h and then analyzed by flow cytometry (Supplementary Figure S1).

### Flow cytometry

The RBCs labeled with FITC-Dextran were harvested by centrifugation at the indicated time points and were washed twice in PBS. iRBCs were fixed and stained with propidium iodide (PI, BD Biosciences, PharMingen, San Diego, CA, USA) to visualize the parasites. Isolated MPs were suspended in PBS before cytometric analysis. An Accuri C6 Flow Cytometer (BD Biosciences, Franklin Lakes, NJ, USA) was used for data acquisition and analysis. Generally, more than 10 000 events were collected in each sample.

### Comparison of FITC-labeled MPs in different conditions

To count the MPs in different culture conditions, a total of 5 × 10^7^ FITC-nRBCs were separately incubated with 2.5 × 10^6^ synchronized ring stage parasites, medium exchanged from iRBCs-cultured supernatant and fresh *P. falciparum* complete medium for 30 h. Supernatants (400 μL) were collected from each condition at 0, 6, 12, 18, 24 and 30 h and the MP levels in the supernatants were determined by flow cytometry.

### RNA isolation, stem-loop RT-PCR and quantitative real-time PCR

Total RNA was extracted from mature nRBCs, iRBCs, MPs using Trizol reagent (Invitrogen, CA, USA) following the manufacturer’s instructions. Sampled RNAs were quantified by the absorbance at 260 nm. For stem-loop RT-PCR, total RNA templates were first reverse-transcribed into cDNAs using miRNA-specific stem-loop primers and were then amplified by PCR. The primers were designed as described previously^20, 21^ and are listed in Supplementary Table S1. For detection of miRNAs by quantitative real-time PCR (RT-qPCR), synthesized *Arabidopsis thaliana* miR-404 RNA oligos (ath-miR-404) were added to counted cells (2 nmol/10^8^ RBCs) before total RNA extraction and were used as an exogenous reference for data normalization. TaqMan MicroRNA Assays (Applied Biosystems, Foster City, CA, USA) were used to determine the relative expression of hsa/mmu-miR-451 (Assay ID 001105), hsa-miR-486 (Assay ID 001278), hsa-miR-181a (Assay ID 000480) and ath-miR-404 (Assay ID 001420) according to the manufacturer’s instructions. For RT-qPCR to detect *var* mRNAs, random hexamer primers were used in reverse transcription, and TransStart Tip Green qPCR superMix kits (Transgene, Beijing, China) with SYBR green dye were used in qPCR according to the product manual. The qPCR primers are listed in Supplementary Table S1. RT-qPCR was carried out using the StepOne Plus real-time PCR System (Applied Biosystems). The relative expression of miRNAs was determined by the 2^−ΔΔCt^ method and was presented as the fold change.

### Northern blotting

The nRBCs and iRBCs were counted before total RNA extraction with synthesized *A. thaliana* miR-404 RNA oligos (100 nmol/10^8^ × RBCs) added as a loading control. Total RNA (5–8 μg) was run on a 15% urea denatured acrylamide gel and was transferred onto Hybond-N+ membrane (Amersham Biosciences Corp., Uppsala, Sweden). Reagents from the DIG High Prime DNA Labeling and Detection Starter Kit II (Roche Applied Science, Mannheim, Germany) were used according to the manufacturer’s instructions. The sequences of the digoxigenin-conjugated DNA oligonucleotide probes are listed in Supplementary Table S2. After hybridization, the membrane was incubated with anti-digoxigenin-AP and covered with Chloro-5-substituted adamantyl-1,2-dioxetane phosphate (CSPD) for chemiluminescence detection. The membrane was exposed to X-ray films (X-Omat, Kodak, Rochester, NY, USA) for 20 min or longer.

### Protein extraction, western blot and immunoprecipitation

Protein lysates (10 μg each) made with 0.05% saponin from parasite pellets, nRBCs or MPs were separated by 10% SDS-PAGE and were wet-transferred to a PVDF membrane (Amersham Biosciences Corp.). The membrane was then incubated in anti-Ago2 monoclonal antibody, followed by HRP-conjugated goat anti-mouse IgG secondary antibody. To compare the expression levels of hAgo2 in iRBCs, the protein lysates were collected from 1.5 × 10^7^ iRBCs, nRBCs or nRBC ghosts (negative control), and 200 ng of purified recombinant His-tagged protein M.RCAg-1 was added. The His-tagged M.RCAg-1 was detected by His-Tag (2A8) mouse monoclonal antibody (M20001, Abmart, Shanghai, China) as a loading control. To detect hAgo2 in MPs, the isolated MPs were treated with or without 20 μg/mL proteinase K in the presence or absence of 0.1% v/v Triton X-100. An enhanced chemiluminescence detection system (ECL, Pierce, Invitrogen; USA) was used following the manufacturer’s instructions. The membrane was exposed to X-ray films (X-Omat, Kodak) for 20 min or longer. For immunoprecipitation (IP), parasite pellets and nRBCs were lysed in lysis buffer. The supernatant was collected by centrifugation and was then divided into two aliquots. One aliquot was incubated with anti-Ago2 monoclonal antibody (ab 57113, Abcam) and the other was incubated without antibody as a control. Protein G-Sepharose beads were added to pull down the mouse antibody. The immunoprecipitated proteins were resuspended in SDS-PAGE loading buffer for further analysis.

### Immunodepletion of hAgo2 in mature nRBCs

Immunodepletion was carried out as described previously.^22^ Mock-treated RBCs incubated with protein G-sepharose beads without antibody were used as a control. The efficiency of hAgo2 immunodepletion in mature RBCs was determined by western blot (Supplementary Figure S2).

### Effects of miRNA overexpression/inhibition and effects of MPs on *var* expression

nRBCs were washed twice with RPMI1640 and were resuspended in complete cytomix. Approximately 200 μL of cells at 50% hematocrit were electroporated with 10 μg of synthesized FAM-conjugated miR-451/-140 mimics, inhibitors or negative control oligonucleotides as described previously.^16^ Transfected RBCs were infected with synchronized schizont parasites with 4% parasitemia in 24-well plates and were incubated for 48 h. To assess the effects of MPs on the expression of *var*, the same concentration of nMPs or iMPs was added to synchronized ring stage parasite cultures with 0.5% parasitemia and 5% hematocrit in 6-well plates in triplicate. Parasite culture without MPs were used as a negative control. The total RNAs were extracted from all samples after 48 h using Trizol reagent (Invitrogen, CA, USA), and the mRNA levels of *var* group A/B/C genes were detected by RT-qPCR.

### miRNA IP and illumina HiSeq miRNA sequencing

IP of hAgo2-binding small RNAs was carried out as described previously.^23^ The total RNAs pulled down were extracted using Trizol reagent (Invitrogen, Shanghai, China). Two small RNA libraries were then constructed and quality evaluated (Supplementary Figure S3) and were designated as nRBCs-RIP and Pf-RIP. RNAs between 18 and 30 nt were sequenced using a HiSeq2000 deep sequencing system, and the raw data were analyzed by Beijing Genomics Institute (BGI, Shenzhen, China). The data from clean reads of the two RIP-Seq sets are listed in Table 1.

### Immune electron microscopy

RBCs, including 3%–4% iRBCs, were fixed in 4% paraformaldehyde and 1.25% glutaraldehyde, followed by four steps of dehydration. Dehydrated cells were imbedded in LR-White (London Resin Company Ltd, Hampshire, UK) and were cut into ultrathin sections. The sections were incubated in mouse anti-Ago2 monoclonal antibody (ab 57113, Abcam), and normal mouse IgG multiple antibody (ab 188776, Abcam) was set as a control (Supplementary Figure S4). The gold-conjugated anti-mouse IgG secondary antibody (Sigma-Aldrich) was then applied. The sections were stained with uranyl acetate and were examined under a JEM-1010 transmission electron microscope (TEM, JEOL, Japan).

### Fluorescence *in situ* hybridization

iRBCs were seeded on poly-lysine-coated cover slips, then fixed and permeabilized on ice. The cover slips were incubated overnight with hybridization buffer containing 5′ FAM-labeled miR-451 probe (Genepharma, Shanghai, China) complementary to miR-451. A scramble probe was used as a negative control. The slides were mounted with mounting medium containing DAPI (ZSGB-bio) and were analyzed by fluorescence microscopy (Leica DM2000B).

### Construction of firefly luciferase reporter vectors and dual-luciferase reporter assay

Total RNA from the *P. falciparum* 3D7 parasite was reverse transcribed using the DBL1α reverse primer. PCR primers were designed for the 5′UTRs of *var* groups A, B and C according to previously published sequences.^24^ The PCR products were digested with *Eco*RI and *Hin*dIII and were cloned into a pCDNA3.1 luciferase vector. The resulting constructs were Luc-A, Luc-B and Luc-C. The 3′UTR of *var* group A was amplified from the genomic DNA of the *P. falciparum* 3D7 strain using published primers^25^ and was then cloned into the pCDNA3.1 luciferase vector (Down-A). The primers used for amplification of *var* UTRs are listed in Supplementary Table S3.

The human embryonic kidney 293ET cell line (kindly provided by the Cell Center of Peking Union Medical College, Beijing, China) was applied for further study. For luciferase assay, one constructed vector (Luc-A/-B/-C/Down-A) or a site-mutated vector (Luc-A^mut^/-B^mut^/Down-A^mut^) mixed with Renilla luciferase vector was added to the wells in triplicate and was co-transfected into 293ET cells with/without miR-451/-140 mimics or inhibitors (synthesized by Invitrogen Corp., China) using Lipofectamine TM2000 (Invitrogen, Shanghai, China) as instructed by the manufacturer. The sequences of miR-451 and miR-140 mimics or inhibitors are listed in Supplementary Table S4. The cells were harvested after 48 h and were lysed with 1 × passive lysis buffer. Luciferase activity was assayed using the Dual-Luciferase Reporter Assay System (#E1910, Promega, Madison, WI, USA) and was detected with a luminometer (LB960, Berthold, Pforzheim, Germany). The ratio of firefly to Renilla luciferase activity in each well was normalized against the negative control.

The site-mutated vectors Luc-A^mut^/-B^mut^ and Down-A^mut^ were constructed using a site-directed mutagenesis kit (Yeasen, Shanghai, China) with mutation primers. The sequences of UTRs and mutation primers for target site mutation are listed in Supplementary Table S5.

### Prediction of target genes in *P. falciparum*

The Miranda algorithm^26^ and PITA algorithm^27^ were used to predict the target genes of hmiRNAs in *P. falciparum*. UTR data from the *P. falciparum* 3D7 strain in *Plasmo*DB release-27 were analyzed thoroughly. The prediction results are provided in Table 2 and Supplementary Table S5.

### Statistical analysis

All error bars shown in figures are standard errors of the mean (sem), and the data are given as the mean±s.e.m. Statistical differences were analyzed in SPSS by one-way analysis of variance (ANOVA). A *P*-value less than 0.05 was considered to be significant. *N*-values for biological replicates are shown.

## RESULTS

### Microparticle release and cargo transfer

**Figure 1:**
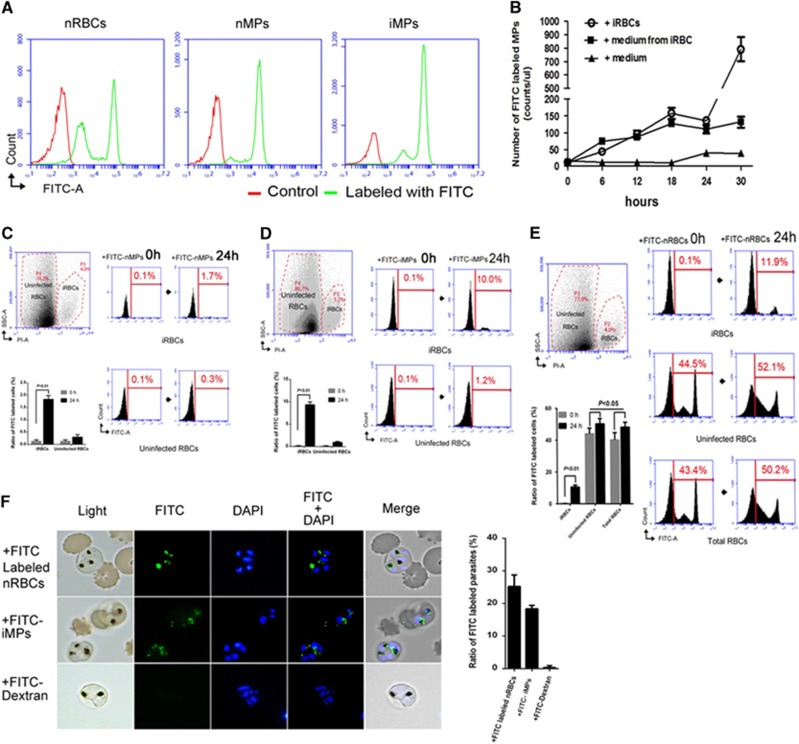
*P. falciparum* infection intensively enhances RBC release of MPs to transfer their cargo into parasites within iRBCs. (**A**) Flow cytometric analysis distinguished FITC-labeled (green) normal RBCs (FITC-nRBCs; left) from cells without labeling (all in red). The MPs released by FITC-nRBCs (nMPs; middle) or FITC-nRBCs mixed with iRBCs (iMPs; right) were also labeled. (**B**) Origination and quantity analysis of MPs by adding cell culture medium (filled triangle), medium exchanged from the iRBC culture (filled box) or iRBCs only (sphere) into each FITC-nRBCs culture at 0 h. (**C**–**E**) Flow cytometric analysis of cargo transfer from nMPs (**C**), iMPs (**D**) and nRBCs (**E**) into iRBCs and uninfected RBCs. iRBCs were isolated and gated by PI staining (left, top, **C**–**E**). Cargo transference by nMPs (**C**), iMPs (**D**) and nRBCs (**E**) into iRBCs (**C**–**E**, right, top), uninfected RBCs (**C**, **D**, right, bottom; **E**, right, middle), and among total RBCs (**E**, right, bottom) was analyzed at 0 and 24 h. The average ratio of FITC-iRBCs and FITC-labeled uninfected RBCs from three repeats was also analyzed (**C**–**E**, left, bottom). (**F**) Images (left) and ratios (right) of FITC-iRBCs in fluorescence microscopy. Synchronized ring stage iRBCs were co-cultured with FITC-nRBCs (left, top), FITC-iMPs (left, middle) or 0.5 mg/mL FITC-Dextran alone (left, bottom) for 24 h. The FITC-iRBC ratios (right) were counted under fluorescence microscope.

First, we established the method for labeling nRBCs with FITC-Dextran *in vitro* and confirmed its feasibility by flow cytometric analysis (Figure 1A, left). Next, we analyzed nMPs (Figure 1A, middle) and iMPs (Figure 1A, right); 80%–90% of both types of MPs were labeled with FITC after the cells were cultured for 24 h (Figure 1A, middle right). To exclude the possibility that FITC-Dextran randomly binds to the membranes of any cells, we performed tests by adding FITC-Dextran directly to the culture medium and incubating for 24 or 72 h, finding no FITC-Dextran-labeled RBCs (Supplementary Figure S1). These results confirmed that the FITC labeling technique could be used to closely follow changes in the quantity of MPs and RBCs under different experimental conditions. For the purpose of understanding the origin of the MPs, we separated the FITC-nRBCs equally into three cell cultures and then added different components into each at 0 h. We found that the level of FITC-labeled MPs (FITC-MPs) in the FITC-nRBCs plus unlabeled, synchronized ring stage iRBCs culture and the level of FITC-MPs in the culture for which ordinary medium was exchanged by the supernatant of the iRBC culture had similar levels within 24 h, and significantly fewer FITC-MPs were detected in nRBC culture with ordinary culture medium (Figure 1B). Interestingly, the level of FITC-MPs in the medium added with unlabeled iRBCs was dramatically elevated at 30 h when the parasites developed from ring stages into the schizont stages and started the next generation. This indicates that FITC-MPs were dominantly produced by nRBCs when the parasites developed at their early phase in RBCs. We speculate that messages released from the parasite or triggered by the invasion of RBCs by merozoites might exist in the medium, which could stimulate the nRBCs to release more MPs (e.g., the result of adding the iRBC-conditioned medium to the nRBC culture).

We also wanted to know what type of RBC, nRBCs or iRBCs is the main target of the MPs. First, we isolated FITC-labeled iMPs (FITC-iMPs) from the iRBC culture (FITC-nRBCs co-cultured with synchronized ring stage iRBCs at 3%–5% parasitemia) and FITC-labeled nMPs (FITC-nMPs) from the FITC-nRBCs culture. After incubation of the FITC-nMPs or -iMPs with the unlabeled RBCs (including 3%–5% iRBCs) for 24 h, there were significantly more FITC-labeled iRBCs (FITC-iRBCs) (Figures 1C and 1D, right top) than FITC-nRBCs (Figures 1C and 1D, right bottom). On the other hand, FITC-iMPs could increase the labeled cell ratio much more (from 0.1% to 10% for iRBCs, from 0.1% to 1.2% for nRBCs; Figure 1D, right) than FITC-nMPs (from 0.1% to 1.7% for iRBCs, from 0.1% to 0.3% for nRBCs; Figure 1C, right). These results suggest that when RBCs are invaded by malaria parasites, MPs prefer to enter iRBCs instead of nRBCs. To confirm these discoveries, we directly added FITC-nRBCs to the unlabeled RBCs (including 3%–5% iRBCs) and incubated for 24 h. A significant increase in FITC-iRBCs was measured (from 0.1% to 11.9% Figure 1E, right top), in contrast to a moderate increase of FITC-nRBCs (from 44.5% to 52.1% Figure 1E, right middle) and FITC-labeled cells in total (43.4% to 50.2% Figure 1E, right bottom). All of the data suggest that the invasion of RBCs by malaria parasites could trigger nRBCs to release iMPs, which could establish cell-cell communication between uninfected and infected RBCs, and that the iMPs mostly target the iRBCs. To visualize the process of cargo transfer by the MPs, we incubated the unlabeled iRBCs with FITC-nRBCs, FITC-iMPs and FITC-Dextran (control) for 24 h. Surprisingly, under the live fluorescence microscope, the green fluorescence of FITC-nRBCs or FITC-iMPs was observed to enter into the iRBCs and potentially localize in the cytoplasm of the parasites (Figure 1F).

### HAgo2 affects parasite development

**Figure 2:**
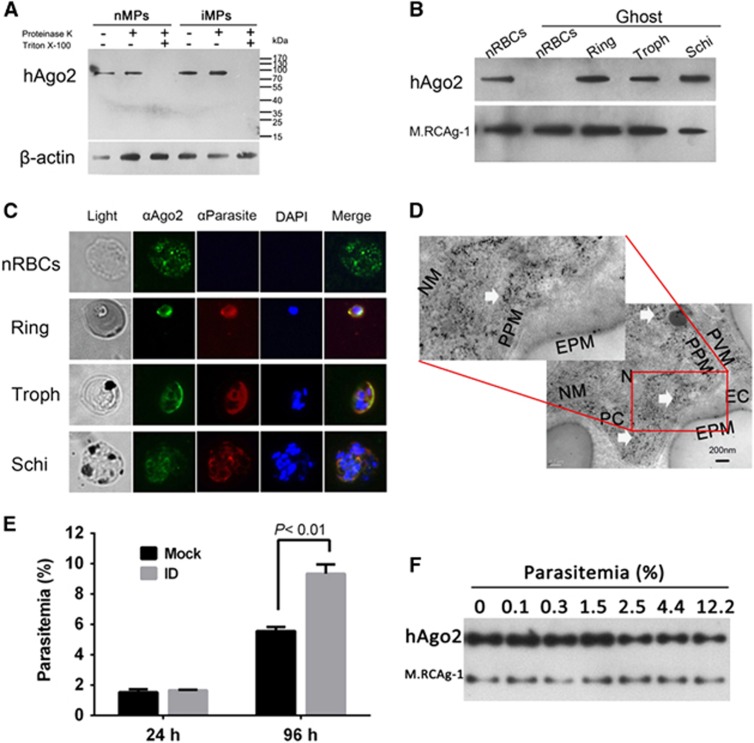
Cargo of MPs identified in *P. falciparum* parasites and hAgo2 detected in both nRBCs and *P. falciparum* parasites. (**A**) Western blot analysis of hAgo2 in nMPs and iMPs. Proteinase K in the presence or absence of Triton X-100 was used to test the localization of Ago2. β-actin was used as a loading control. trophozoite, Troph; schizont, Schi. (**B**) Western blot detecting hAgo2 in nRBCs and synchronized parasites at different stages. The parasites were purified from 0.05% saponin-treated iRBCs, creating ghost-like RBCs to avoid the interference of hAgo2 in the cytoplasm of RBCs. (**C**) Immunofluorescence assays of the localization of hAgo2 in nRBCs and parasites. The samples were labeled with FITC (green), Alexa Fluor 594 (Red) and DAPI (blue). (**D**) Immune electricity microscopy to observe hAgo2 inside the parasite. The hAgo2 is indicated by anti-hAgo2 antibody (white arrows). The control used normal mouse antibody (Supplementary Figure S4). Erythrocyte/parasite cytoplasm, EC/PC; erythrocyte/parasite plasma membrane, EPM/PPM; parasitophorous vacuole membrane, PVM; nucleus/nuclear membrane, N/NM. (**E**) The impact of hAgo2 depletion on parasitemia. iRBCs were co-cultured with hAgo2-immunodepleted nRBCs (ID) or with mock-treated RBCs (Mock) for 24 or 96 h (*n*=3). (**F**) Western blot detecting hAgo2 in iRBCs during *P. falciparum* infection. The equal numbers of iRBCs at trophozoite stages with different parasitemia were sampled to extract whole protein mix. M.RCAg-1 was used as a loading control.

We performed western blots and demonstrated hAgo2 in both nMPs and iMPs (Figure 2A). In addition, hAgo2 was present in nRBCs and in parasites at different stages of development (Figure 2B), which was confirmed by immunofluorescence assay (Figure 2C) and immune electron microscopy, which revealed hAgo2 in the cytoplasm of the parasite (Figure 2D). These data imply the involvement of cargo transfer of MPs in hAgo2 translocation. Next, parasites were cultured with hAgo2-depleted nRBCs (Supplementary Figure S2) for 96 h. Compared with the nRBCs, hAgo2-depleted RBCs had significantly higher parasitemia, implying that hAgo2 plays an inhibitory role during parasite development (Figure 2E). To examine the potential correlation between the level of hAgo2 in iRBCs and the degree of parasitemia, we used western blots. The level of hAgo2 in iRBCs decreased when the parasitemia increased from 1.5% to 12.2% in culture (Figure 2F), suggesting that hAgo2 is consumed when parasites continue to develop and is mainly involved at the beginning of *P. falciparum* infection or after a mild infection in a malaria patient.

### Human miRNAs complex with MP-transferred Ago2 *in vitro* and *in vivo*

**Figure 3:**
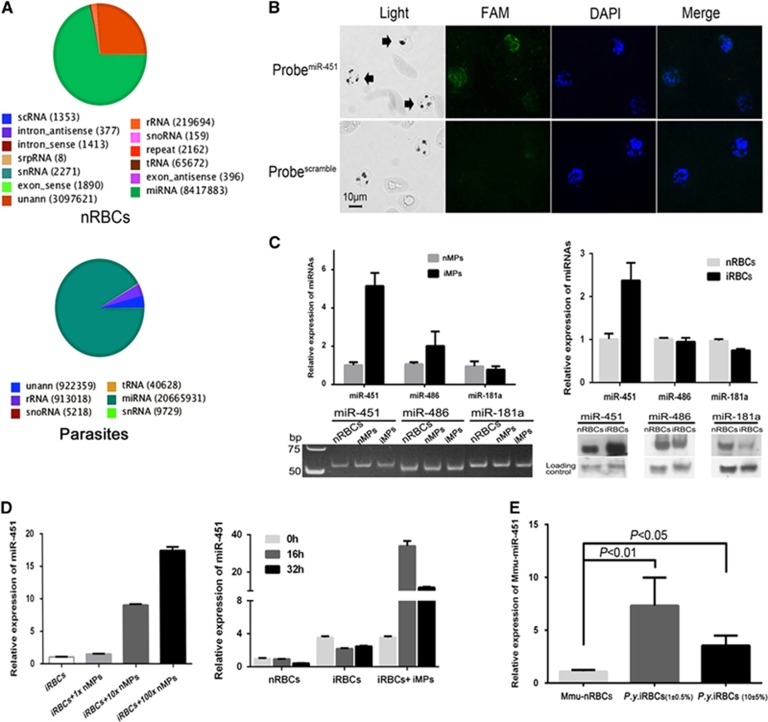
The hAgo2-miRNA complex transferred by MPs into *Plasmodium* parasite *in vitro* and *in vivo.* (**A**) Annotations of high-throughput sequencing data of small RNAs bound by hAgo2. The read counts of each RNA class are listed in round brackets. (**B**) Fluorescence *in situ* hybridization detection of miR-451 in iRBCs. Black arrows in light fields indicate the parasites. miR-451 was labeled with 5′ carboxyfluorescein (FAM)/scramble probes (green), and the parasite nuclei were labeled with DAPI (blue). (**C**) RT-qPCR (left top) and stem-loop RT-PCR (left bottom) analysis of miR-451, miR-486 and miR-181a within nMPs and iMPs; RT-qPCR (right top) and northern blot (right bottom) analysis of these miRNAs in nRBCs and iRBCs. (**D**) RT-qPCR analysis of miR-451 in iRBCs treated with nMPs (left) and iMPs (right). Synchronized ring stage iRBCs were incubated with different concentrations of nMPs (1 ×, 10 ×, 100 ×) or iMPs at separate times (16, 32 h). The level of miR-451 in iRBCs/nRBCs was given as a relative value of 1.0 (*n*=3). (**E**) RT-qPCR analysis of Mmu-miR-451 in the nRBCs (Mmu-nRBCs) and iRBCs of *P. yoelii*-infected mice (*P.y.*iRBCs) at different parasitemia ratios (1%±0.5% and 10%±5%) (i.e., three mice in each group) (*n*=3).

Given that hAgo2 directly interacts with mature miRNAs in the miRNA pathway of RNA interference, we isolated and identified the miRNAs that bind to hAgo2 in the cytoplasm of *P. falciparum* parasites using RNA immunoprecipitation (RIP). A sharp peak of RNAs of ~25 nt was observed (Supplementary Figure S3). The isolated small RNAs were then analyzed by Illumina HiSeq2000 sequencing (RIP-Seq). A total of 22 556 883 clean reads (mostly 22 nt in length) were obtained from the parasite small RNA library, and 11 810 899 clean reads were obtained from the nRBC small RNA library (Table 1). Clean reads (91.6%) identified by RIP-Seq were mapped to known miRNA families, of which 373 hmiRNAs came from parasites and 488 from nRBCs (Figure 3A and Supplementary Table S6). To further examine the localization of hmiRNAs, we used miR-451, one of the most abundant hmiRNAs identified by RIP-Seq with high read counts (Supplementary Table S6), as a marker to perform fluorescence *in situ* hybridization in the parasite. Our results confirm the localization of miR-451 in parasites (Figure 3B), which is consistent with the findings of recent studies.^16, 28^ Thus, hAgo2 complexes with abundant hmiRNAs in the parasites, indicating a functional involvement in parasite development during the blood stage.

Furthermore, in both nMPs and iMPs, we identified mature human miRNAs, such as miR-451, miR-486 and miR-181a, by RT-qPCR and stem-loop RT-PCR (Figure 3C, left). These three hmiRNAs have been previously demonstrated to translocate into the parasites at different levels during the blood stage.^16^ The miR-451 levels appeared to be higher in iMPs than in nMPs (Figure 3C, left). Surprisingly, the results from RT-qPCR and northern blot analysis showed that the level of miR-451 was at least twofold higher in iRBCs than in nRBCs (Figure 3C, right), which are the same patterns as those in MPs; however, no such significant changes were seen for miR-486 and miR-181, which provided evidence that miR-451 is apparently exchanged between nRBCs and iRBCs via some specific conveyors. Therefore, we speculated that such components may be the effectors of cell-cell communication mediated by MPs. After co-culturing nMPs or iMPs with synchronized ring stage iRBCs for 24 h, both nMPs and iMPs could increase the level of miR-451 in iRBCs in a dosage-dependent manner (Figure 3D, left); the highest level was from the group of iMPs plus iRBCs when incubating for 16 h (Figure 3D, right). To confirm the quantitative variation of miR-451 *in vivo*, we infected mice with *P. yoelii* parasites. RT-qPCR showed that, after infection, mouse miR-451 (Mmu-miR-451) levels were significantly higher in iRBCs than in nRBCs, even at different levels of parasitemia (Figure 3E). Taken together, these results demonstrate that mature hmiRNAs were transferred from nRBCs to iRBCs by MPs during the blood stage of malaria infection.

### Host miRNAs affect *P. falciparum var* gene expression

**Figure 4:**
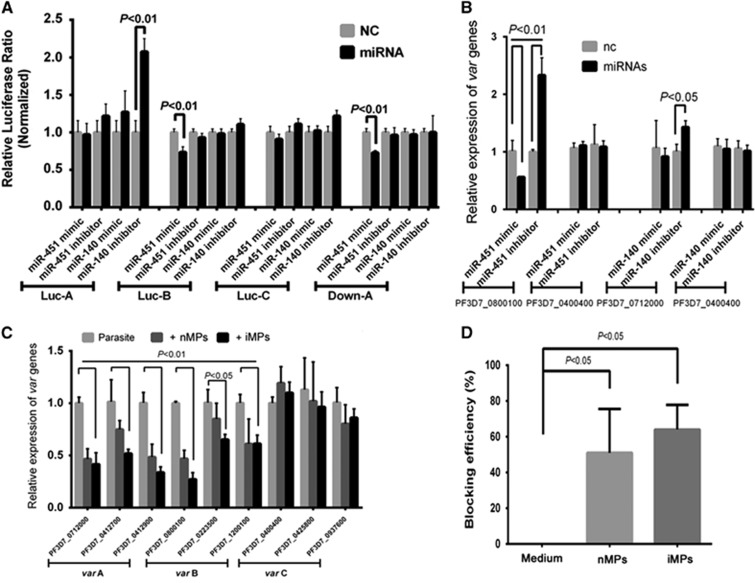
Human miRNAs transferred by MPs downregulate the expression of *P. falciparum var*, and MPs block merozoites from invading RBCs. (**A**) Dual-luciferase assay of interactions between the UTRs of *var* and hmiRNAs. Firefly luciferase constructs containing 5′UTRs from *var* group A/B/C (Luc-A/-B/-C) or 3′UTRs from *var* group A (Down-A) were co-transfected with miR-451/-140 mimics or inhibitors (miRNA) or with control miRNAs (NC) (*n*=3). (**B**) RT-qPCR analysis of the expression of two *var* genes in parasites transfected with miR-451/-140 mimics or inhibitors (miRNAs) or with control miRNAs (nc) (*n*=3). (**C**) RT-qPCR analysis of MPs effects on the expression of three groups of *var* genes. Three genes in each group were selected and quantitatively evaluated by adding nMPs or iMPs to the parasite cultures for 24 h (*n*=3). (**D**) Invasion blocking assays to assess the invasion blocking efficiency of MPs. nMPs or iMPs isolated from the equal volume of supernatants were applied to co-culture with schizont stage parasites (*n*=3). Cultures with no adding MPs were used as negative controls, the block efficiency of which was set as zero percent.

A bioinformatics analysis of precipitated hAgo2 binding of hmiRNAs predicted that many identified miRNAs target parasite mRNAs, several of which encode important *P. falciparum* proteins, including PfEMP1, Rifin and Stevor (Table 2). Among these miRNAs, we chose miR-451 and miR-140 for further studies because they were predicted to target the UTR of *var* genes (Supplementary Table S5), which encodes 60 homologs of PfEMP1 protein, a critical molecule in the adherence of iRBCs to the endothelium of host blood vessels in malaria patients.^29, 30^ To examine whether these miRNAs could regulate the expression of PfEMP1, we performed a dual-luciferase assay in 293ET cells. Luc-A exhibited significantly increased luciferase activity when co-transfected with a miR-140 inhibitor, whereas Luc-B and Down-A exhibited significantly decreased luciferase activity when co-transfected with a miR-451 mimic, in spite of inconsistent effects with miR-140 mimic and miR-451 inhibitors, possibly due to the interference of endogenous miRNAs in 293ET cells (Figure 4A). By contrast, when we mutated the target sites in the UTRs of Luc-A, -B or Down-A into scramble sequences (Supplementary Table S5), the efficiency of regulation was greatly diminished (Supplementary Figure S5). These data indicate that miR-140 and miR-451 were able to downregulate luciferase expression by targeting the UTR sequences of *var* and therefore imply that both miR-140 and miR-451 can downregulate the expression of *var* group A genes and that miR-451 can also downregulate the expression of *var* group B genes. Taken together, our data suggest that, by mediating the transfer of hAgo2-miRNA complexes into iRBCs, host-derived miR-451 and miR-140 play a crucial role in regulating *var* gene expression during the blood stage of malaria infection.

To further verify the effects of hmiRNAs on the parasites, we transfected synthesized small RNAs into the parasites to overexpress mimic miRNAs or knockdown miRNAs with their inhibitors and then quantitatively detected the expression of their targets, including a *var* group A gene and a *var* group B gene. In contrast to a control *var* group C gene PF3D7_0400400, both PF3D7_0712000 (*var* group A gene) and PF3D7_0800100 (*var* group B gene) could be downregulated by miR-140 and miR-451, respectively, although the regulation efficiency of the miR-140 mimic did not reach significance (Figure 4B). We also added isolated nMPs or iMPs to the parasite culture medium and demonstrated that expression of both *var* group A and B genes can be downregulated at different levels. However, the *var* group C genes were not affected (Figure 4C). For the purpose of evaluating the function of MPs in parasite development, we performed an invasion-blocking assay and observed both nMPs and iMPs inhibited the invasion of merozoites into RBCs (Figure 4D). Therefore, the host-originated miRNAs and MPs not only affect the expression of the parasite’s genes, but also could protect the host RBCs from further parasite invasion.

## DISCUSSION

After malaria parasites turn into merozoites and break away from the hepatocytes, RBCs are the only target cells in the circulation. Although all malaria clinical symptoms appear at this stage, including severe pathogenic damage and even death, the potential importance of the interaction between infected and uninfected (i.e., normal) RBCs is unknown. In this study, we methodically studied nRBC-derived MPs for their role in malaria infection. The MPs were obtained from *in vitro* RBC cultures to ensure homogeneity. Our data demonstrate that after some of the RBCs were infected by malaria parasites, abundant MPs were released from the uninfected RBCs at an early phase of parasite development, and they mainly targeted the iRBCs. The cargo of the MPs was found near the nuclei of the parasite cells within the iRBCs. HAgo2 in both MPs and parasite cells inhibits the development of the parasites. The miRNAs that complex with hAgo2 target several essential *P. falciparum* genes that are involved in malaria pathogenesis. Among these genes, miR-451 and miR-140 target the mRNA of a critical parasite antigen, PfEMP1, and downregulate its expression. Pierre-Yves *et al* recently showed that host miRNAs complexed with hAgo2 derived from iRBCs is capable of specifically silencing gene expression in host endothelial cells and altering their barrier properties.^31^ Even so, the origin of the RISC complex still needs to be traced. By labeling the iMPs, we found that iRBCs were translocating the hAgo2-miRNAs complexes, most likely from the nRBCs, during the development of blood stage. The fact that the MPs isolated from the parasite-infected RBCs were conditionally targeted to the iRBCs much more than to the nRBCs and the observation that the cargo of MPs translocated into the malaria parasite cells supports our hypothesis and suggests that MPs act as conveyors of cell information between nRBCs and malaria parasites, which further suggests that RBCs can have a resistant action during the parasite infection. Furthermore, the presence of host-derived hAgo2 in not only RBCs and MPs, but also the cytoplasm of *Plasmodium* parasites at different stages of intraerythrocytic development strongly supports its involvement in the regulation of parasite genes. Our work also showed that the depletion of hAgo2 from RBCs can dramatically accelerate the development of *Plasmodium* parasite in culture, implying that the crucial role of host miRNAs, a canonical regulator generally complexed with hAgo2, is to inhibit parasite activities. Based on our *in vitro* results that the highest level of hAgo2 occurs at 0% to 1.5% parasitemia but decreased when the parasitemia was higher, hAgo2 must translocate via MPs into the parasites either at the beginning of the infection or when the extent of infection is mild; however, hAgo2 is consumed if the parasite growth is not controlled, and it invades more RBCs.

MPs and their miRNA content have inspired many investigations of their pathological and disease-resistant effects.^32, 33, 34, 35^ Here we demonstrated that the miRNAs identified from the RIP-Seq of *P. falciparum* parasites were different from the miRNAs in nRBCs. The bioinformatics data predict that the former targets to an abundance of parasite mRNAs, in which essential parasite antigens, especially the virulence genes PfEMP1, are involved. These data indicate the crucial functions of these miRNAs because of the important role of PfEMP1 in the adherence of iRBCs.^29, 30^ As miR-451 had the highest read count, we chose to use it as a marker, finding that it was present inside iRBCs, iMPs and nMPs. However, one of the most notable observations in this study was that iMPs include much more miR-451 than nMPs. Also, much higher miR-451 levels were found when iMPs were added to the iRBCs than when they were added to the nRBCs. In an *in vivo* rodent model, we detected increased levels of miR-451 in the RBCs of *P. yoelii-*infected mice. Taken together, MPs were further shown as a quicker approach that was selected by nRBCs to transfer hAgo2-miRNA complexes into iRBCs to further play an important role in parasite infection.

The *P. falciparum* antigen PfEMP1 has been a major focus of malaria research since it was first reported.^36^ The protein is expressed by as many as 60 *var* variations with a single copy in each parasite and can be classified into five main groups (A, B, C, D and E) on the basis of the upstream sequence (Ups). This mutually exclusive expression of the genes helps the parasites to escape from immune attacks and to adhere to the deep capillary vessels of organs, allowing them the opportunity and time to produce gametocytes to continue their life cycle. Despite the translational and posttranscriptional regulation reported for some subgroups of *var* genes,^37, 38^ the clonal variation of *var* is mainly regulated at the transcriptional level by epigenetic factors, such as histone modification, nuclear architecture and *cis*-regulatory elements in the upstream or intron regions.^39, 40, 41, 42, 43^ Our data demonstrated for the first time that human miR-451 and miR-140 can recognize the 5′ or 3′UTR of *var* genes at the transcriptional level, effectively downregulating the expression of the luciferase reporter. Although the experimental data attest to several targeted A and B subgroups of *var* genes, these two miRNAs might be involved in the broad transcriptional regulation of all A and B subgroups of *var* genes due to the high UTR conservation of these *var* genes. The bioinformatics data also confirmed this supposition. Interestingly, the same results were obtained when iMPs were added to the parasite culture medium (Figure 4C). Our data showed different efficacy of miR-451 in hemoglobin (Hb) AA RBCs from a previous report,^16^ in which miR-451 in heterozygous HbAS or HbSS erythrocytes was covalently integrated into *P. falciparum* mRNAs essential for parasite growth, suppressing the translation of one of these transcripts, a cAMP-dependent protein kinase PKA-R, and inhibiting the parasite blood stage development. Since the expression of PfEMP1 offers the parasite advantages *in vivo*, but not *in vitro*, the downregulation of PfEMP1 caused by miR-451 and miR-140 is unlikely to affect parasite development during *in vitro* culture, indicating that the function of human miRNAs against parasites is more complicated. Based on our data from bioinformatics analysis, an abundance of host-derived miRNAs appears to target the UTRs of different genes in parasites; however, the mechanism underlying host miRNAs in parasite gene regulation still needs to be further elucidated.

Parasite-derived exosome-like vesicles were demonstrated to prompt the differentiation of sexual forms of parasites,^14, 44^ and host platelet- and white blood cell-derived MPs were shown to have the functions of enhancing cerebral malaria,^45, 46^ indicating that MPs originating from different parent cells might be involved in various pathological processes during malaria parasite infection. Focusing on nRBC-derived MPs, the release of which was initiated at the time when uninfected RBCs received some kind of signals from the iRBCs, we found that more iMPs containing Ago2 and miRNAs entered the parasite cells within iRBCs. We conclude that the MPs should be involved in parasite development, although the detailed mechanisms underlying cell-cell communication and the signals received by the nRBCs require further illumination. However, as *P. falciparum* invades and grows inside mature RBCs, which lack nuclei, Ago2 and miRNAs must exist within the cytoplasm of RBCs before erythroid denucleation occurs; the detailed mechanism of this process also needs to be explored. Considering that a variety of extracellular vesicles have been observed in the circulation of malaria patients, the *in vivo* situation, in which iRBCs receive other substances from different cell sources and are thus affected, may prove to be much more complicated.

In mammalian cells, some miRNAs negatively regulate gene expression by degrading mRNAs or by inhibiting the translation of target RNAs after binding to the 3′UTR region of target mRNAs and are involved in cell proliferation, development, differentiation and death. miRNAs have been shown to play important roles in normal erythropoiesis by regulating differentiation at most stages.^47^ MiR-451, an erythroid-specific miRNA, is an example in both erythropoiesis and in anemia linked to malaria. This miRNA is significantly lower in beta-thalassemia patients than in normal individuals, but it is significantly overexpressed in sickle cell anemia patients.^48, 49, 50^ In studies of the mechanism of miRNA expression in human erythropoiesis, an abundant amount of miR-451 has been demonstrated in enucleated normal RBCs and 10^4^-fold more miR-451 is expressed in RBCs than in granulocytes.^51, 52^ However, the reason why a large amount of miR-451 exists in RBCs has not yet been clearly determined. One can venture that RBCs may prepare for prevention against infection by the malaria parasite. Given that the co-evolution of *Plasmodium* parasites with great apes and humans has been estimated to have been continuing for more than a million years,^53, 54^ it is tempting to speculate that this miR-451 inhibition is an inherited genetic feature passed down from primate ancestors as a genetic memory.

As a critical regulator in various diseases, miRNAs have the potential to be used as therapeutic agents. Quite a few groups and pharmaceutical companies around the world are conducting research and development to explore miRNA-based therapies and to build a new area of miRNA therapeutics. By virtue of our experiments confirming that miR-451 is involved in the resistance of blood stage malaria infection and the results from Chi’s group that transfection of HbAA erythrocytes with miR-451 markedly reduces parasitemia,^16^ we think that miR-451 has the potential to be developed as a new therapeutic drug for malaria infection, especially in the treatment of cerebral malaria.

In conclusion, our results demonstrate for the first time that normal RBCs display an innate ability to resist infection by *P. falciparum* parasite by releasing Ago2-miRNA complexes via MPs into iRBCs. In addition, miR-451 downregulates the *var* gene of parasite virulence factor PfEMP1, which could moderate pathological injury due to the disease. Therefore, we propose that the use of therapeutic miRNAs may unveil new avenues for malaria control.

## Figures and Tables

**Table 1:** Data cleaning for the two RIP-seq sets

**Reads type**	**nRBCs**		**Parasites**	
	**Count**	**Ratio**	**Count**	**Ratio**
Total reads	12 280 094		22 905 773	
High quality	12 262 275	100%	22 612 387	100%
3′adapter null	84 103	0.69%	7760	0.03%
Insert null	6185	0.05%	5583	0.02%
5′adapter contaminants	21 569	0.18%	3699	0.02%
Smaller than 18 nt	339 487	2.77%	38 449	0.17%
Poly A	32	0.00%	13	0.00%
Clean reads	11 810 899	96.32%	22 556 883	99.75%

The total reads were sorted by getting rid of the low-quality reads, 3′ adaptor null reads, insert null reads, 5′ adaptor contaminants, reads shorter than 18 nt, and reads with a polyA tail.

**Table 2:** Target genes predicting human miRNAs against *P. falciparum* parasite using Miranda algorithm and PITA algorithm

**hmiRNAs**	**The target** ***P. falciparum*** **genes (ID)**	**RPM in parasites**
miR-451	PfEMP1 (PF11_0008)/Cg1 protein (PF07_0035)/protein phosphatase, putative (MAL8P1.108)/translation initiation factor SUI1, putative (PF08_0079)	36705.11
miR-140	PfEMP1 (PF11_0008)	68932.83
miR-185	PfEMP1 (PFL1970w/PFL1955w)	78516.42
miR-503	rifin (PF14_0770)	44.1372
miR-629	PfEMP1 (PFD1235w/MAL8P1.207)/merozoite surface protein (PF10_0352)	53.5313
miR-330	Sporozoite invasion-associated protein 1 (PFD0425w)/early transcribed membrane protein 10.3 (PF10_0164)	15.9053
let-7b	rifin (PFD0045c)	79978.26
miR-4732	rifin (MAL13P1.2)	1253.835
let-7f	rifin (psu|PFD0045c)/regulator of nonsense transcripts, putative (PF10_0057)	469839.4
miR-182	DNA-directed RNA polymerase 3 largest subunit (PF13_0150)	92.5987

Abbreviation: reads per million, RPM.
