# Supplementary material for: Red blood cells release microparticles containing human argonaute 2 and miRNAs to target genes of Plasmodium falciparum
Source: Emerg Microbes Infect. 2017 Aug 23;6(8):e75–. doi: 10.1038/emi.2017.63 (PMC5583671; doi:10.1038/emi.2017.63)
Supplement: Supplementary Figure S1 [file emi201763x1.pdf]

Supplementary Figure S1 Flow cytometric analysis of the transfer potential of FITC-Dextran towards nRBCs.

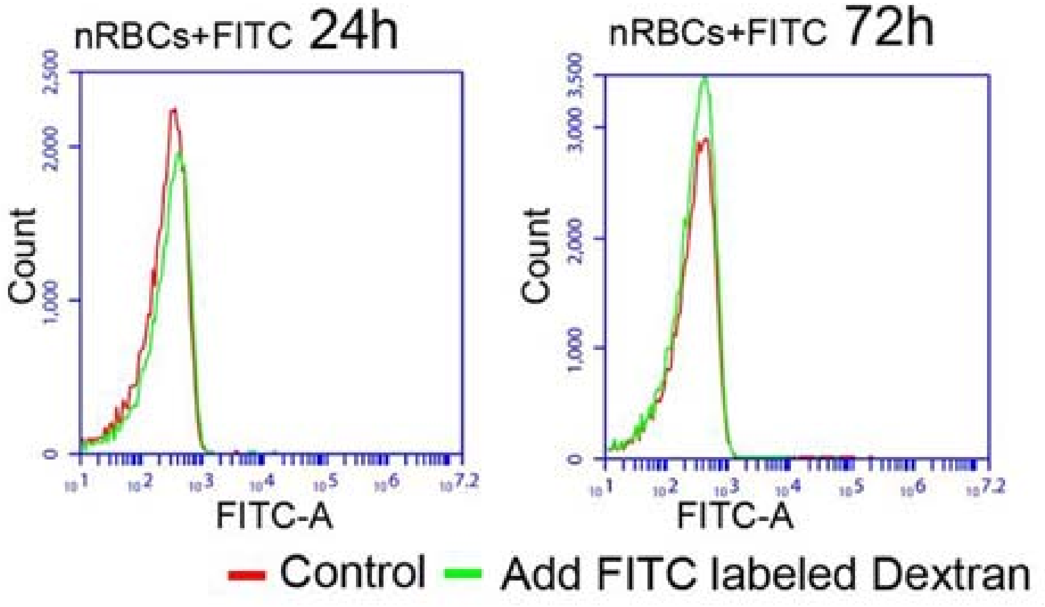

5×10^7 nRBCs were cultured in medium containing 0.5 mg/ml FITC-Dextran for 24 and 72 h. The FITC-nRBCs were analyzed by flow cytometry at 24 and 72 h ($n=3$). Red lines and green lines indicate control nRBCs and FITC-nRBCs, respectively.
