# Supplementary material for: Red blood cells release microparticles containing human argonaute 2 and miRNAs to target genes of Plasmodium falciparum
Source: Emerg Microbes Infect. 2017 Aug 23;6(8):e75–. doi: 10.1038/emi.2017.63 (PMC5583671; doi:10.1038/emi.2017.63)
Supplement: Supplementary Figure S2 [file emi201763x2.pdf]

Supplementary Figure S2 Western blot analysis of hAgo2 immunodepletion.

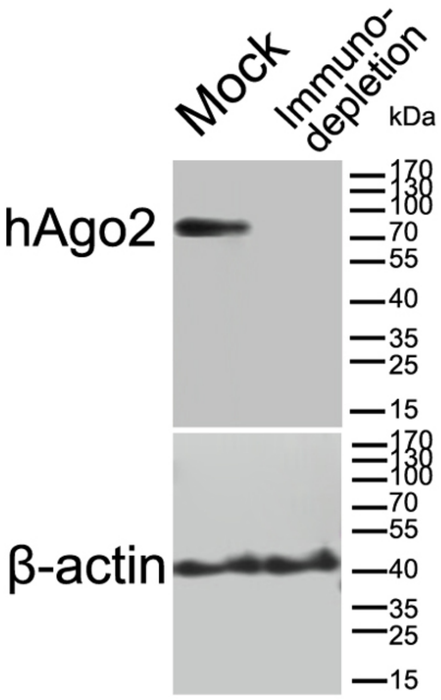

Two groups of nRBCs were immunodepleted using hAgo2 monoclonal antibody (Immuno-depletion) and control antibody (Mock). β-actin was used as a loading control.
