# Supplementary material for: Red blood cells release microparticles containing human argonaute 2 and miRNAs to target genes of Plasmodium falciparum
Source: Emerg Microbes Infect. 2017 Aug 23;6(8):e75–. doi: 10.1038/emi.2017.63 (PMC5583671; doi:10.1038/emi.2017.63)
Supplement: Supplementary Figure S3 [file emi201763x3.pdf]

Supplementary Figure S3 Quality evaluation of small RNA samples extracted from RIP.

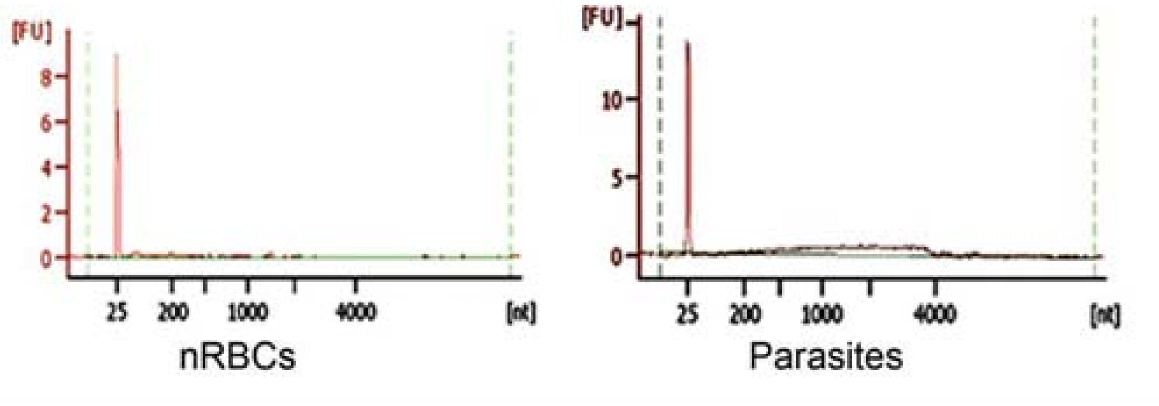

Red curves show the distribution of length and quantity of RNA segments in the RNA samples purified from RIP samples of nRBCs (left) and parasite lysates (right).
