# Supplementary material for: Red blood cells release microparticles containing human argonaute 2 and miRNAs to target genes of Plasmodium falciparum
Source: Emerg Microbes Infect. 2017 Aug 23;6(8):e75–. doi: 10.1038/emi.2017.63 (PMC5583671; doi:10.1038/emi.2017.63)
Supplement: Supplementary Figure S4 [file emi201763x4.pdf]

Supplementary Figure S4 IEM control for localization of hAgo2 in the P. falciparum parasite.

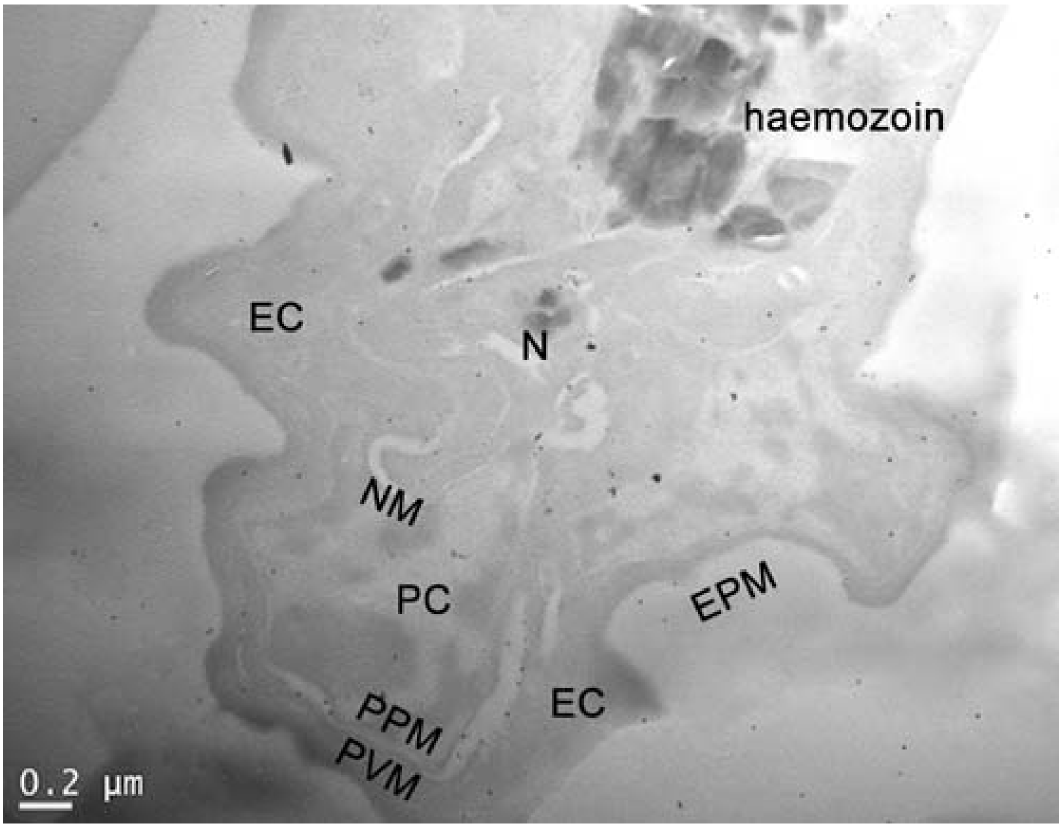

Normal mouse antibody was used. EC: erythrocyte cytoplasm, EPM: erythrocyte plasma membrane, PVM: parasitophorous vacuole membrane, PPM: parasite plasma membrane, PC: parasite cytoplasm, NM: nuclear membrane, N: nucleus of parasite.
