# Supplementary material for: Red blood cells release microparticles containing human argonaute 2 and miRNAs to target genes of Plasmodium falciparum
Source: Emerg Microbes Infect. 2017 Aug 23;6(8):e75–. doi: 10.1038/emi.2017.63 (PMC5583671; doi:10.1038/emi.2017.63)
Supplement: Supplementary Figure S5 [file emi201763x5.pdf]

Supplementary Figure S5 Dual-luciferase assay of the interactions between site-mutated UTRs from var and hmiRNAs.

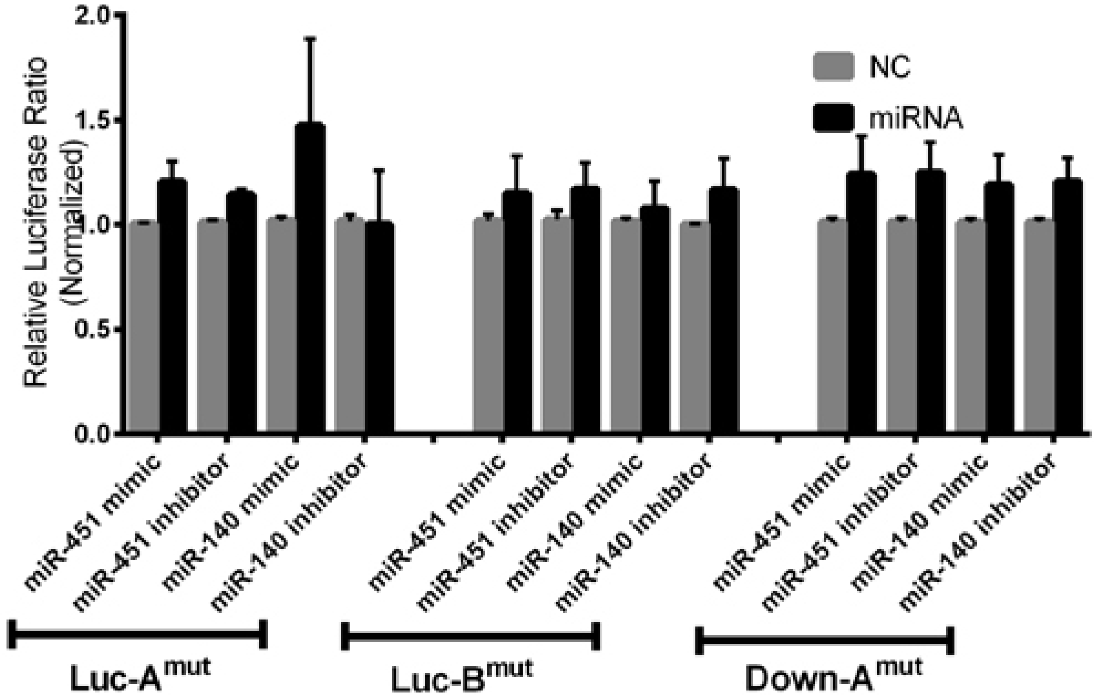

Luc-A^{mut}/-B^{mut} and Down-A^{mut} represent firefly luciferase constructs containing site-mutated 5'UTRs from *var* group A/B and site-mutated 3'UTRs from *var* group A, respectively. miRNA: luciferase reporter constructs co-transfected with miR-451 or miR-140 mimics or inhibitors; NC: negative control without co-transfection of miRNAs (*n=3*).
