# Supplementary material for: Red blood cells release microparticles containing human argonaute 2 and miRNAs to target genes of Plasmodium falciparum
Source: Emerg Microbes Infect. 2017 Aug 23;6(8):e75–. doi: 10.1038/emi.2017.63 (PMC5583671; doi:10.1038/emi.2017.63)
Supplement: Supplementary Table S1 [file emi201763x6.pdf]

Supplementary Table S1 Sequences of reverse transcription primers and qRT-PCR primers*

Primer ID	Sequences
For human miRNAs	
miR-451 RT	GTCGTATCCAGTGCAGGGTCCGAGGTATTCGCAC TGGATACGACAACTCA
miR-451 forward	CACGCAAAACCGTTACCATTACT
miR-486 RT	GTCGTATCCAGTGCAGGGTCCGAGGTATTCGCAC TGGATACGACACTCGGG
miR-486 forward	CACGCATCCTGTACTGAGCTGCCC
miR-181a RT	GTCGTATCCAGTGCAGGGTCCGAGGTATTCGCAC TGGATACGACAAACTCA
miR-181a forward	CACGCAAACATTCAACGCTGTCGG
General Reverse**	CCAGTGCAGGGTCCGAGGTA
For *var* genes	
PF3D7_0712000-up	ATATGGGAAGGGATGCTCTG
PF3D7_0712000-down	TGAACCATCGAAGGAATTGA
PF3D7_0412700-up	AAACACGTTGAATGGCGATA
PF3D7_0412700-down	GACGCCGAGGAGGTAAATAG
PF3D7_0412900-up	TGACCAAGACGAAGTATGGAA
PF3D7_0412900-down	TTGATCTCTGTTCGCTGTCC
PF3D7_0800100-up	GTCGTGGAAAAACGAAAGGT
PF3D7_0800100-down	TATCTATCCAGGGCCCAAAG
PF3D7_0223500-up	CAATTTTGGGTGTGGAATCA
PF3D7_0223500-down	CACTGGCCACCAAGTGTATC
PF3D7_1200100-up	CGGAGGAGGAAAAACAAGAG
PF3D7_1200100-down	TGCCGTATTTGAGACCACAT
PF3D7_0400400-up	GTTGAGTCTGCGGCAATAGA
PF3D7_0400400-down	CTGGGGTTTGTTCAACACTG
PF3D7_0425800-up	TAAAAGACGCCAACAGATGC
PF3D7_0425800-down	TCATCGTCTTCGTCTTCGTC
PF3D7_0937600-up	ACTTTCTGGTGGGGAATCAG
PF3D7_0937600-down	TTCACCGCCACTTACTTCAG
serine tRNA ligase-up	AAGTAGCAGGTCATCGTGGTT
serinetRNA ligase-down	TTCGGCACATTCTTCCATAA
arginine tRNA ligase-up	AAGAGATGCATGTTGGTC
arginine tRNA ligase-down	GTACCCCAATCACCTACA

*Due to the stem-loop structure designed in reverse transcription primers, the lengths of PCR products were extended to 69 base pairs.

**This primer is universal for PCR amplification of miR-451, miR-486, and miR-181a.
