# Supplementary material for: Red blood cells release microparticles containing human argonaute 2 and miRNAs to target genes of Plasmodium falciparum
Source: Emerg Microbes Infect. 2017 Aug 23;6(8):e75–. doi: 10.1038/emi.2017.63 (PMC5583671; doi:10.1038/emi.2017.63)
Supplement: Supplementary Table S2 [file emi201763x7.pdf]

Supplementary Table S2 Oligonucleotide probe sequences used in Northern blot and fluorescence *in situ* hybridyzation

Probe ID	Sequences and modification
miR-451 DNA probe	DIG*-AACTCAGTAATGGTAACGGTTT
miR-486 DNA probe	DIG-CTCGGGGCAGCTCAGTACAGGA
miR-181a DNA probe	DIG-ACTCACCGACAGCGTTGAATGTT
miR-451 probe	FAM**-AACTCAGTAATGGTAACGGTTT
Scramble probe	FAM-GTGTAACACGTCTATACGCCCA

*Digoxigenin labeling.

**Carboxyfluorescein labeling.
