# Supplementary material for: Red blood cells release microparticles containing human argonaute 2 and miRNAs to target genes of Plasmodium falciparum
Source: Emerg Microbes Infect. 2017 Aug 23;6(8):e75–. doi: 10.1038/emi.2017.63 (PMC5583671; doi:10.1038/emi.2017.63)
Supplement: Supplementary Table S3 [file emi201763x8.pdf]

Supplementary Table S3 Primer sequences related to the amplification of *var*

Primers ID	Sequences
DBL1α-EcoR I reverse	CGGAATTCCGCCAATATCAGCAAAACTTCGTGC
*var*A forward	CCAAGCTTGGAACTTACCATAAATTATCATCAAA
*var*A reverse	CGGAATTCCGTCACCTACAACAAATGTAATAAA
*var*B forward	CCAAGCTTGGCTCATTTATAATTTTACAAAATAAATAAAAC
*var*B reverse	CGGAATTCCGTTATGGGAGTATAGTGATATGGTAGAAT
*var*C forward	CCAAGCTTGGAATATTCATATTCCCACATTGTCATATAT
*var*C reverse	CGGAATTCCGATTATGTGGTAATATCATGTAATGG
DownA forward	CCGCTCGAGCGGCGGATGTATGGAATATAT
DownA reverse	GCTCTAGAGCTACTATTACATAATACATTC
