# Supplementary material for: Red blood cells release microparticles containing human argonaute 2 and miRNAs to target genes of Plasmodium falciparum
Source: Emerg Microbes Infect. 2017 Aug 23;6(8):e75–. doi: 10.1038/emi.2017.63 (PMC5583671; doi:10.1038/emi.2017.63)
Supplement: Supplementary Table S4 [file emi201763x9.pdf]

Supplementary Table S4 Sequences of miRNA mimics and inhibitors in dual-luciferase reporter assay

ID	Sequences and modifications (5' labeled)
miR-451 mimic	FAM*-5'-AAACCGUUACCAUUACUGAGUU-3'<br>3'-CCCAGUAAUGGUAACGGUUUUU-5'-FAM
miR-451 inhibitor	FAM-5'-AACUCAGUAAUGGUAACGGUUU-3
miR-140 mimic	FAM-5'-UACCACAGGGUAGAACCACGG-3'<br>3'-GUGGUUCUACCCUGUGGUAUU-5' -FAM
miR-140 inhibitor	FAM-5'-CCGUGGUUCUACCCUGUGGUA-3'
Negative control	FAM-5'-UUCUCCGAACGUGUCACGUTT-3'<br>3'-ACGUGACACGUUCGGAGAATT-5'-FAM

*Carboxyfluorescein labeling.
