# Supplementary material for: Red blood cells release microparticles containing human argonaute 2 and miRNAs to target genes of Plasmodium falciparum
Source: Emerg Microbes Infect. 2017 Aug 23;6(8):e75–. doi: 10.1038/emi.2017.63 (PMC5583671; doi:10.1038/emi.2017.63)
Supplement: Supplementary Table S5 [file emi201763x10.pdf]

Supplementary Table S5 The target site prediction of human miRNAs in UTR of *var* genes

Seq ID	length	Squence
5'UTR of var group A	147	AACTTACCATAAATTATCATCAAATTATATATATATATA*CACTACTT*AGCACTATTGAATAAAATATAGCATAAAAAAATTATACATATAATGGCAAACCTTTGGTATCGAAAAAAATATTTAATTTATTACATTTGTTGTAGGTGA

Note: the target sites are shown in italic

Target Prediction:

microRNA	Position in UTR	Seed	dGduplex	dGopen	ddG	Seq	Target site	Num of Matches	Mutated seq	Mutation primers
hsa-miR-140-5p	39	8:1:1	-7.87	-7.08	-0.78	CAGUGGUUUUACCCUAUGGUAG	CACTACTT	8	AATTGCAT	Up:ATCATCAAATTATATATATATATAAATTGCATAGCACTATTG;Down:ATGCTATATTTTATTCAATAGTGCTATGCAATTTATATATA
hsa-miR-574-5p	37	8:1:1	-16.7	-6.21	-10.48	UGAGUGUGUGUGUGUGAGUGUGU	TACACTACTTAGCAC	8		
hsa-miR-20a	48	8:1:0	-14.7	-5.6	-9.09	UAAAGUGCUUAUAGUGCAGGUAG	GCACTATT	8		
hsa-miR-525-5p	99	8:1:1	-16.4	-7.58	-8.81	CUCCAGAGGGAUGCACUUUCU	CTTTGGTATCGAAAA	8		
hsa-miR-106b	48	8:1:0	-14.2	-5.6	-8.59	UAAAGUGCUGACAGUGCAGAU	GCACTATTGAATAAAAT	8		
hsa-miR-20b	48	8:1:0	-14	-5.6	-8.39	CAAAGUGCUCAUAGUGCAGGUAG	GCACTATTGAATAAAAT	8		
hsa-miR-221	63	8:1:0	-13.5	-5.81	-7.68	ACCUGGCAUACAAUGUAGAUUU	ATATAGCATAAAAAAAT	8		
hsa-miR-17	48	8:1:0	-13	-5.6	-7.39	CAAAGUGCUUACAGUGCAGGUAG	GCACTATTG	8		
hsa-miR-106a	48	8:1:0	-13	-5.6	-7.39	AAAAGUGCUUACAGUGCAGGUAG	GCACTATTGAATA	8		
hsa-miR-1272	16	8:1:0	-9.5	-2.29	-7.2	GAUGAUGAUGGCAGCAAAUUCUGAAA	TCATCAAAT	8		
hsa-miR-93	48	8:1:0	-12.6	-5.6	-6.99	CAAAGUGCUGUUCGUGCAGGUAG	GCACTATTGAATAAAAT	8		
hsa-miR-200c	49	8:1:0	-12	-5.61	-6.38	CGUCUUACCCAGCAGUGUUUGG	CACTATTGAA	8		

hsa-miR-512-3p	46	8:1:1	-11.8	-5.64	-6.15	AAGUGCUGUCAUAGCUGAGGUC	TAGCACTATT	8
hsa-let-7a	41	8:1:1	-11.2	-5.17	-6.02	UGAGGUAGUAGGUUGUAUAGUU	CTACTTAGCACTATTGA	8
hsa-let-7b	41	8:1:1	-10.9	-5.17	-5.72	UGAGGUAGUAGGUUGUGUGGUU	CTACTTAGC	8
hsa-let-7c	41	8:1:1	-10.9	-5.17	-5.72	UGAGGUAGUAGGUUGUAUGGUU	CTACTTAG	8
hsa-miR-142-3p	38	8:1:0	-12.77	-7.08	-5.68	UGUAGUGUUUCCUACUUUAUGGA	ACACTACTTAGCACTAT	8
hsa-miR-1282	93	8:1:0	-13.26	-7.79	-5.46	UCGUUUGCCUUUUUCUGCUU	GCAAACCTTTGGTAT	8
hsa-miR-155	67	8:1:0	-9.4	-4.03	-5.36	UUAAUGCUAAUCGUGAUAGGGGU	AGCATAAAAAAATT	8
hsa-miR-433	15	8:1:1	-7.5	-2.19	-5.3	UACGGUGAGCCUGUCAUUAUUC	ATCATCAAATTATA	8
hsa-miR-129-5p	109	8:1:0	-11.7	-6.43	-5.26	CUUUUUGCGGUCUGGGCUUGC	GAAAAAAATATTTAAT	8
hsa-let-7f	41	8:1:1	-10.3	-5.17	-5.12	UGAGGUAGUAGAUUGUAUAGUU	CTACTTAGC	8
hsa-miR-1270	15	8:1:1	-7.3	-2.19	-5.1	CUGGAGAUAUGGAAGAGCUGUGU	ATCATCAAAT	8
hsa-let-7g	41	8:1:1	-10	-5.17	-4.82	UGAGGUAGUAGUUUGUACAGUU	CTACTTAG	8
hsa-let-7i	41	8:1:1	-9.7	-5.17	-4.52	UGAGGUAGUAGUUUGUGCUGUU	CTACTTAGCACTATTG	8
hsa-miR-200b	49	8:1:0	-10.1	-5.61	-4.48	CAUCUUACUGGGCAGCAUUGGA	CACTATTG	8
hsa-miR-372	47	8:1:1	-9.8	-5.67	-4.12	CCUCAAAUGUGGAGCACUAUUCU	AGCACTATTGAA	8
hsa-miR-520a-5p	99	8:1:1	-11.5	-7.58	-3.91	CUCCAGAGGGAAGUACUUUCU	CTTTGGTATCGAAA	8
hsa-miR-199a-3p	50	8:1:1	-9.5	-5.6	-3.89	ACAGUAGUCUGCACAUUGGUUA	ACTATTGAAT	8

hsa-miR-199b-3p	50	8:1:1	-9.5	-5.6	-3.89	ACAGUAGUCUGCACAUUGGUUA	ACTATTGAAT	8
hsa-miR-568	34	8:1:0	-10	-6.2	-3.79	AUGUAUAAAUGUAUACACAC	ATATACACT	8
hsa-miR-409-3p	49	8:1:1	-9.4	-5.61	-3.78	GAAUGUUGCUCGGUGAACCCCU	CACTATTGAATAAAATA	8
hsa-miR-155	47	8:1:0	-9.43	-5.67	-3.75	UUAAUGCUAAUCGUGAUAGGGGU	AGCACTATTG	8
hsa-miR-578	70	8:1:1	-8.86	-5.28	-3.57	CUUCUUGUGCUCUAGGAUUGU	ATAAAAAAATTATACAT	8
hsa-miR-548i	128	8:1:0	-9.65	-6.12	-3.52	AAAAGUAAUUGCGGAUUUUGCC	TTACATTTGTTG	8
hsa-miR-140-3p	99	8:1:0	-10.9	-7.58	-3.31	UACCACAGGGUAGAACCACGG	CTTTGGTATCGAAAAAA	8
hsa-miR-587	52	8:1:0	-8.7	-5.6	-3.09	UUUCCAUAGGUGAUGAGUCAC	TATTGAAT	8
hsa-miR-520d-5p	100	8:1:1	-10.6	-7.55	-3.04	CUACAAAGGGAAGCCCUUUC	TTTGGTATCG	8
hsa-miR-98	41	8:1:1	-8.2	-5.17	-3.02	UGAGGUAGUAAGUUGUAUUGUU	CTACTTAGCAC	8
hsa-miR-491-3p	67	8:1:0	-7	-4.03	-2.96	CUUAUGCAAGAUUCCCUUCUAC	AGCATAAAAA	8
hsa-miR-620	15	8:1:1	-5.1	-2.19	-2.9	AUGGAGAUAGAUAUAGAAAU	ATCATCAAATTATAT	8
hsa-miR-190	114	8:1:1	-9.7	-6.83	-2.86	UGAUAUGUUUGAUAUAUUAGGU	AAATATTTA	8
hsa-miR-577	120	8:1:1	-10	-7.15	-2.84	UAGAUAAAAUAUUGGUACCUG	TTAATTTATTAC	8
hsa-miR-380	23	8:1:1	-6.3	-3.55	-2.74	UGGUUGACCAUAGAACAUGCGC	ATTATATATATATATAC	8
hsa-miR-936	50	8:1:1	-8.2	-5.6	-2.59	ACAGUAGAGGGAGGAAUCGCAG	ACTATTGAATAAAAT	8
hsa-miR-548d-5p	128	8:1:0	-8.71	-6.12	-2.58	AAAAGUAAUUGUGGUUUUUGCC	TTACATTTGTTGT	8

hsa-let-7d	41	8:1:1	-7.5	-5.17	-2.32	AGAGGUAGUAGGUUGCAUAGUU	CTACTTAGCACTATTGA	8
hsa-miR-548l	116	8:1:1	-9	-6.74	-2.25	AAAAGUAUUUGCGGGUUUUGUC	ATATTTAATTTA	8
hsa-miR-190b	114	8:1:1	-8.9	-6.83	-2.06	UGAUAUGUUUGAUAUUGGGUU	AAATATTTAAT	8
hsa-miR-548b-5p	128	8:1:0	-8.11	-6.12	-1.98	AAAAGUAAUUGUGGUUUUGGCC	TTACATTTGTT	8
hsa-miR-548c-5p	128	8:1:0	-8.05	-6.12	-1.92	AAAAGUAAUUGCGGUUUUUGCC	TTACATTTGTTGTAG	8
hsa-miR-568	32	8:1:1	-8	-6.17	-1.82	AUGUAUAAAUGUAUACACAC	ATATATAC	8
hsa-miR-548j	128	8:1:0	-7.91	-6.12	-1.78	AAAAGUAAUUGCGGUCUUUGGU	TTACATTTGTTGTAGGT	8
hsa-miR-579	20	8:1:1	-5.1	-3.46	-1.63	UCGCGGUUUGUGCCAGAUGACG	CAAATTATA	8
hsa-miR-380	127	8:0:0	-7.79	-6.17	-1.61	UGGUUGACCAUAGAACAUGCGC	ATTACATTTGTTGTAGG	8
hsa-miR-299-5p	93	8:1:0	-9.3	-7.79	-1.5	UGGUUUACCGUCCCACAUACAU	GCAAACCTTTGGT	8
hsa-miR-590-3p	59	8:1:0	-7.7	-6.19	-1.5	UAAUUUUAUGUAUAAGCUAGU	TAAAATATAGCATA	8
hsa-miR-196a	40	8:1:1	-7.19	-5.77	-1.41	UAGGUAGUUUCAUGUUGUUGGG	ACTACTTAGCA	8
hsa-miR-429	49	8:1:1	-7	-5.61	-1.38	UAAUACUGUCUGGUAAAACCGU	CACTATTGA	8
hsa-miR-493	96	8:1:1	-9.2	-8.13	-1.06	UUGUACAUGGUAGGCUUUCAUU	AACCTTTGGTAT	8
hsa-miR-1259	24	8:0:1	-5	-4.06	-0.93	UCCCUGAGACCCUUUAACCUGUGA	TTATATATATATATACA	8
hsa-miR-196b	40	8:1:1	-6.7	-5.77	-0.92	UAGGUAGUUUCCUGUUGUUGGG	ACTACTTAGCACTATTG	8
hsa-miR-122	38	8:1:1	-8.01	-7.08	-0.92	UGGAGUGUGACAAUGGUGUUUG	ACACTACTTAGCACT	8

hsa-miR-206	130	8:1:1	-8.6	-7.87	-0.72	UGGAAUGUAAGGAAGUGUGUGG	ACATTTGTTGTA	8
hsa-miR-524-5p	100	8:1:1	-8.2	-7.55	-0.64	CUACAAAGGGAAGCACUUUCUC	TTTGGTATCGAAAAA	8
hsa-miR-1	130	8:1:1	-8.5	-7.87	-0.62	ACAUACUUCUUUAUAUGCCCAU	ACATTTGTTGTAGG	8
hsa-miR-449b	39	8:1:1	-7.7	-7.08	-0.61	AGGCAGUGUAUUGUUAGCUGGC	CACTACTTAGC	8
hsa-miR-1826	16	8:1:0	-2.9	-2.29	-0.6	UUUGGCAAUGGUAGAACUCACACU	TCATCAAAT	8
hsa-miR-590-3p	19	8:1:0	-4.1	-3.5	-0.59	UAAUUUUAUGUAUAAGCUAGU	TCAAATTATATA	8
hsa-miR-584	61	8:1:1	-6.7	-6.15	-0.54	UUAUGGUUUGCCUGGGACUGAG	AAATATAGCAT	8
hsa-miR-454	128	8:1:1	-6.65	-6.12	-0.52	ACCCUAUCAAUAUUGUCUCUGC	TTACATTTGTTG	8
hsa-miR-449a	39	8:1:1	-7.6	-7.08	-0.51	UGGCAGUGUAUUGUUAGCUGGU	CACTACTTAGCACTA	8
hsa-miR-1290	113	8:1:1	-7.3	-6.81	-0.48	UGGAUUUUUGGAUCAGGGA	AAAATATTTAA	8
hsa-miR-448	34	8:1:1	-6.5	-6.2	-0.29	UUGCAUAUGUAGGAUGUCCCAU	ATATACACTACTTAG	8
hsa-miR-539	123	8:1:1	-6.93	-6.7	-0.22	GGAGAAAUUAUCCUUGGUGUGU	ATTTATTA	8
hsa-miR-34c-5p	39	8:1:1	-7.3	-7.08	-0.21	AGGCAGUGUAGUUAGCUGAUUGC	CACTACTTA	8
hsa-miR-450a	91	8:1:0	-7.4	-7.31	-0.084	UUUUGCGAUGUGUUCCUAAUAU	TGGCAAACCTT	8
hsa-miR-568	79	8:1:0	-5.81	-5.74	-0.065	AUGUAUAAAUGUAUACACAC	TTATACATA	8
hsa-miR-203	117	8:1:1	-6.8	-6.74	-0.056	AGUGGUUCUUAACAGUUCAACAGUU	TATTTAATT	8
hsa-miR-190	63	8:1:1	-5.84	-5.81	-0.025	UGAUAUGUUUGAUAUAUUAGGU	ATATAGCA	8

hsa-miR-648	36	8:1:1	-6.3	-6.27	-0.02	AAGUGUGCAGGGCACUGGU	ATACACTACTT	8
hsa-miR-659	56	8:1:1	-6.1	-6.08	-0.012	AGGACCUUCCCUGAACCAAGGA	GAATAAAATAT	8
hsa-miR-130b	128	8:1:1	-6.05	-6.12	0.072	ACUCUUUCCCUGUUGCACUAC	TTACATTTGTTGTAG	8
hsa-miR-633	50	8:1:1	-5.5	-5.6	0.1	CUAAUAGUAUCUACCACAAUAAA	ACTATTGAATAAAATAT	8
hsa-miR-568	24	8:1:1	-3.9	-4.06	0.16	AUGUAUAAAUGUAUACACAC	TTATATATATATATA	8
hsa-miR-568	30	8:1:1	-5.8	-6.07	0.27	AUGUAUAAAUGUAUACACAC	ATATATATACACTAC	8
hsa-miR-548m	96	8:1:0	-7.79	-8.13	0.34	CAAAGGUAUUUGUGGUUUUUG	AACCTTTGGTATCGAAA	8
hsa-miR-559	120	8:1:0	-6.8	-7.15	0.35	UAAAGUAAAUAUGCACCAAAA	TTAATTTA	8
hsa-miR-1322	48	8:1:1	-5.21	-5.6	0.39	GAUGAUGCUGCUGAUGCUG	GCACTATTGAATAAA	8
hsa-miR-301b	128	8:1:1	-5.7	-6.12	0.42	GCUCUGACGAGGUUGCACUACU	TTACATTT	8
hsa-miR-590-3p	74	8:1:0	-4.9	-5.47	0.57	UAAUUUUAUGUAUAAGCUAGU	AAAAATTATACA	8
hsa-miR-380	65	8:1:1	-4.5	-5.07	0.57	UGGUUGACCAUAGAACAUGCGC	ATAGCATAA	8
hsa-miR-1278	39	8:1:0	-6.5	-7.08	0.58	UAGUACUGUGCAUAUCAUCUAU	CACTACTTAGC	8
hsa-miR-190b	63	8:1:1	-5.2	-5.81	0.61	UGAUAUGUUUGAUAUUGGGUU	ATATAGCATAAA	8
hsa-miR-337-3p	64	8:1:0	-4.3	-5.09	0.79	CUCCUAUAUGAUGCCUUUCUUC	TATAGCATAAA	8
hsa-miR-301a	128	8:1:1	-5.3	-6.12	0.82	GCUCUGACUUUAUUGCACUACU	TTACATTTGTTGTA	8
hsa-miR-331-5p	81	8:1:1	-5	-5.84	0.84	CUAGGUAUGGUCCCAGGGAUCC	ATACATATAATGGCAAA	8

hsa-miR-28-3p	123	8:1:1	-5.8	-6.7	0.9	CACUAGAUUGUGAGCUCCUGGA	ATTTATTACATTTGTT	8
hsa-miR-548a-5p	128	8:1:0	-5.21	-6.12	0.91	AAAAGUAAUUGCGAGUUUUACC	TTACATTTGTTGTAGGT	8
hsa-miR-140-5p	96	8:1:1	-7.2	-8.13	0.93	CAGUGGUUUUACCCUAUGGUAG	AACCTTTGGTATC	8
hsa-miR-1259	79	8:1:1	-4.8	-5.74	0.94	UCCCUGAGACCCUUUAACCUGUGA	TTATACATATAA	8
hsa-miR-130a	128	8:1:1	-4.77	-6.12	1.35	UUCACAUUGUGCUACUGUCUGC	TTACATTTGTT	8
hsa-miR-548h	128	8:1:0	-4.61	-6.12	1.51	AAAAGUAAUCGCGGUUUUUGUC	TTACATTTG	8
hsa-miR-450a	107	8:1:0	-4.5	-6.08	1.58	UUUUGCGAUGUGUUCCUAAUAU	TCGAAAAAAA	8
hsa-miR-372	40	8:1:1	-4.17	-5.77	1.6	CCUCAAAUGUGGAGCACUAUUCU	ACTACTTA	8
hsa-miR-511	61	8:1:1	-4.4	-6.15	1.75	GUGUCUUUUGCUCUGCAGUCA	AAATATAGCA	8
hsa-miR-885-5p	87	8:1:0	-5.4	-7.22	1.82	UCCAUUACACUACCCUGCCUCU	ATAATGGCAAACCTT	8
hsa-miR-34a	39	8:1:1	-5.1	-7.08	1.98	UGGCAGUGUCUUAGCUGGUUGU	CACTACTTAGCACTATT	8
hsa-miR-643	85	8:1:1	-4.8	-6.81	2.01	ACUUGUAUGCUAGCUCAGGUAG	ATATAATGGCAAACCTT	8
hsa-miR-410	136	8:1:1	-7.9	-10.06	2.16	AGGUUGUCUGUGAUGAGUUCG	GTTGTAGGT	8
hsa-miR-450b-5p	91	8:1:0	-5.1	-7.31	2.21	UUUUGCAAUAUGUUCCUGAAUA	TGGCAAACCTTTGGTAT	8
hsa-miR-1290	75	8:1:1	-3.4	-5.62	2.22	UGGAUUUUUGGAUCAGGGA	AAAATTATACA	8
hsa-miR-153	79	8:1:1	-3.5	-5.74	2.24	UCAUUUUUGUGAUGUUGCAGCU	TTATACATATAAT	8
hsa-miR-629	94	8:1:1	-5.7	-8.1	2.4	UGGGUUUACGUUGGGAGAACU	CAAACCTTTGGTA	8

hsa-miR-568	26	8:1:1	-3	-5.62	2.62	AUGUAUAAAUGUAUACACAC	ATATATATA	8
hsa-miR-296-3p	95	8:1:1	-5.5	-8.14	2.64	GAGGGUUGGGUGGAGGCUCUCC	AAACCTTTGG	8
hsa-miR-1283	136	8:1:1	-7.3	-10.06	2.76	UCUACAAAGGAAAGCGCUUUCU	GTTGTAGGTGA	8
hsa-miR-568	28	8:1:1	-3.1	-5.95	2.85	AUGUAUAAAUGUAUACACAC	ATATATATATACACT	8
hsa-miR-512-3p	39	8:1:1	-4.2	-7.08	2.88	AAGUGCUGUCAUAGCUGAGGUC	CACTACTTA	8
hsa-miR-655	124	8:1:0	-3.15	-6.04	2.89	AGAGGUUAUCCGUGUUAUGUUC	TTTATTACATTTGTTGT	8
hsa-miR-656	65	8:1:1	-2	-5.07	3.07	AGGUUGCCUGUGAGGUGUUCA	ATAGCATAAAAA	8
hsa-miR-548n	129	8:1:0	-4.5	-7.6	3.1	CAAAAGUAAUUGUGGAUUUUGU	TACATTTGTTGTAGGT	8
hsa-miR-656	60	8:1:0	-3.1	-6.21	3.11	AGGUUGCCUGUGAGGUGUUCA	AAAATATAGCATA	8
hsa-miR-129-5p	70	8:1:1	-1.72	-5.28	3.56	CUUUUUGCGGUCUGGGCUUGC	ATAAAAAAATTA	8
hsa-miR-656	87	8:1:1	-3.5	-7.22	3.72	AGGUUGCCUGUGAGGUGUUCA	ATAATGGCAAACCTTTG	8
hsa-miR-491-3p	55	8:1:0	-2.1	-6.07	3.97	CUUAUGCAAGAUUCCCUUCUAC	TGAATAAAATAT	8
hsa-miR-380	53	8:1:1	-1.6	-5.67	4.07	UGGUUGACCAUAGAACAUGCGC	ATTGAATAAAA	8
hsa-miR-421	134	8:1:1	-6.2	-10.33	4.13	AUCAACAGACAUUAAUUGGGCGC	TTGTTGTAGGT	8
hsa-miR-545	133	8:1:1	-6	-10.15	4.15	UCAGUAAAUGUUUAUUAGAUGA	TTTGTTGTAGG	8
hsa-miR-511	112	8:1:1	-2.2	-6.81	4.61	GUGUCUUUUGCUCUGCAGUCA	AAAAATATTT	8
hsa-miR-522	115	8:1:1	-2.01	-6.76	4.75	CUCUAGAGGGAAGCGCUUUCUG	AATATTTAATTTAT	8

hsa-miR-142-5p	122	8:1:0	-2.1	-6.95	4.85	CAUAAAGUAGAAAGCACUACU	AATTTATTA	8		
hsa-miR-186	123	8:1:1	-1.71	-6.7	4.99	CAAAGAAUUCUCCUUUUGGGCU	ATTTATTACATTTGTTG	8		
hsa-miR-142-5p	118	8:1:1	-1.6	-6.73	5.13	CAUAAAGUAGAAAGCACUACU	ATTTAATTTATT	8		

Seq ID	length	Squence
5'UTR of var group B	374	CTCATTTATAATTTACAAAATAAATAAAACATAAAAAAATAATATATATAATTAAATATTTAAATTAAG GAATACATGAAATATATAATATTTTTCATAAAATGT*AATTGTTG*TTTTTTTTTTTGTTAGAATATTTAAA TTTATTATAAAAGTATTAATATATATATTTTTTTTTTAAAAATATATATATAAAACTAATAAGTATTATTA TATACATATTAAATATTATTTATTAATATATATTATATATATATTATAATATTACAACTATTATAATTACT ATATATATATAAATATATATATAATACTTATATATATATATTTCAACAAATACAATATTATCATTATTCT ACCATATCACTATACTCCCATAA

Note: the target sites are shown in italic

Target Prediction:

microRNA	Position in UTR	Seed	dGduplex	dGopen	ddG	Seq	Target site	Num of Matches	Mutated seq	Mutation primers
hsa-miR-451	105	8:1:1	-6.2	-7.49	1.29	AAACCGUUACCAUUACUGAGUU	AATTGTTG	8	GATATGAT	Up:AATATATAATATTTTTCATAAAATGTGATATGATTTTTTTTTTTTGT; Down:AATTTAAATATTCTAACAAAAAAAAAAAATCATATCACATTTTATGA
hsa-miR-140-5p	355	8:1:1	-9	-1.03	-7.96	CAGUGGUUUUACCCUAUGGUAG	TATCACTA	8		
hsa-miR-140-5p	192	8:1:1	-6	-15.7	9.7	CAGUGGUUUUACCCUAUGGUAG	AACTAATA	8		
hsa-miR-1270	356	8:1:1	-16.6	-1.39	-15.2	CUGGAGAUAUGGAAGAGCUGUGU	ATCACTAT	8		
hsa-miR-202	362	8:1:1	-14.9	-1.19	-13.7	UUCCUAUGCAUAUACUUCUUUG	ATACTCCCA	8		
hsa-miR-206	362	8:1:1	-12.6	-1.19	-11.4	UGGAAUGUAAGGAAGUGUGUGG	ATACTCCCAT	8		
hsa-miR-34c-5p	363	8:1:1	-12.2	-1.15	-11.04	AGGCAGUGUAGUUAGCUGAUUGC	TACTCCCATAA	8		
hsa-miR-449a	363	8:1:1	-12.2	-1.15	-11.04	UGGCAGUGUAUUGUUAGCUGGU	TACTCCCATAA	8		

hsa-miR-576-3p	353	8:1:1	-11.5	-0.8	-10.69	AAGAUGUGGAAAAAUUGGAAUC	CATATCACTATACTC	8
hsa-miR-449b	363	8:1:1	-11.4	-1.15	-10.24	AGGCAGUGUAUUGUUAGCUGGC	TACTCCCATAA	8
hsa-miR-573	358	8:1:1	-11.7	-1.83	-9.86	CUGAAGUGAUGUGUAACUGAUCAG	CACTATACTCCCATAA	8
hsa-miR-585	363	8:1:0	-11	-1.15	-9.84	CUAGCACACAGAUACGCCCAGA	TACTCCCATA	8
hsa-miR-1275	366	8:1:1	-10.3	-0.94	-9.35	GUGGGGGAGAGGCUGUC	TCCCATAA	8
hsa-miR-921	356	8:1:1	-10.4	-1.39	-9	CUAGUGAGGGACAGAACCAGGAUUC	ATCACTAT	8
hsa-miR-190b	352	8:1:0	-10.34	-1.82	-8.51	UGAUAUGUUUGAUAUAUUAGGU	CCATATCACTATA	8
hsa-miR-34a	363	8:1:1	-9.22	-1.15	-8.06	UGGCAGUGUCUUAGCUGGUUGU	TACTCCCATAA	8
hsa-miR-379	347	8:1:0	-11.2	-4.03	-7.16	UGGUAGACUAUGGAACGUAGG	TTCTACCATATCACT	8
hsa-miR-508-3p	361	8:1:1	-8.3	-1.22	-7.07	UGAUUGUAGCCUUUUGGAGUAGA	TATACTCCCATA	8
hsa-miR-620	356	8:1:1	-8.1	-1.39	-6.7	AUGGAGAUAGAUAUAGAAAU	ATCACTATACTCC	8
hsa-miR-190	352	8:1:0	-8	-1.82	-6.17	UGAUAUGUUUGAUAUAUUAGGU	CCATATCACTATACTC	8
hsa-miR-142-3p	357	8:1:1	-7.72	-2.1	-5.61	UGUAGUGUUUCCUACUUUAUGGA	TCACTATACTCCCATA	8
hsa-miR-1	362	8:1:1	-6.5	-1.19	-5.3	ACAUACUUCUUUAUAUGCCCAU	ATACTCCCAT	8
hsa-miR-1245	355	8:1:0	-6.1	-1.03	-5.06	AAGUGAUCUAAAGGCCUACAU	TATCACTATACTCCC	8
hsa-miR-299-3p	351	8:1:1	-7.7	-2.79	-4.9	UAUGUGGGAUGGUAAACCGCUU	ACCATATCACTATACT	8
hsa-miR-512-3p	355	8:1:1	-5.5	-1.03	-4.46	AAGUGCUGUCAUAGCUGAGGUC	TATCACTATACT	8

hsa-miR-1272	357	8:1:1	-6	-2.1	-3.89	GAUGAUGAUGGCAGCAAAUUCUGAAA	TCACTATACTC	8
hsa-miR-1206	74	8:1:0	-11.8	-8.46	-3.33	UGUUCAUGUAGAUGUUUAAGC	CATGAAATATATAA	8
hsa-miR-1272	338	8:0:1	-9	-6.26	-2.73	GAUGAUGAUGGCAGCAAAUUCUGAAA	TTATCATTATT	8
hsa-miR-1267	323	8:1:0	-13.2	-10.5	-2.69	CCUGUUGAAGUGUAAUCCCCA	TCAACAAATACAATATT	8
hsa-miR-517c	72	8:1:1	-10.2	-8.31	-1.88	CCUCUAGAUGGAAGCACUGUCU	TACATGAAATATATA	8
hsa-miR-1258	347	8:1:1	-5.9	-4.03	-1.86	AGUUAGGAUUAGGUCGUGGAA	TTCTACCAT	8
hsa-miR-517a	72	8:1:1	-10.1	-8.31	-1.78	CCUCUAGAUGGAAGCACUGUCU	TACATGAAATA	8
hsa-miR-1272	341	8:0:1	-7.85	-6.13	-1.71	GAUGAUGAUGGCAGCAAAUUCUGAAA	TCATTATT	8
hsa-miR-590-3p	76	8:1:1	-9.9	-8.18	-1.71	UAAUUUUAUGUAUAAGCUAGU	TGAAATATATAATAT	8
hsa-miR-502-5p	64	8:1:1	-6.4	-4.68	-1.71	AUCCUUGCUAUCUGGGUGCUA	TTAAGGAATACAT	8
hsa-miR-203	56	8:1:1	-6.1	-4.52	-1.57	AGUGGUUCUUAACAGUUCAACAGUU	TATTTAAATTAAGG	8
hsa-miR-539	89	8:1:1	-9.95	-8.52	-1.42	GGAGAAAUUAUCCUUGGUGUGU	ATTTTTCATAA	8
hsa-miR-581	71	8:1:1	-9.5	-8.13	-1.36	UCUUGUGUUCUCUAGAUCAGU	ATACATGAAATAT	8
hsa-miR-581	28	8:1:1	-4.7	-3.4	-1.29	UCUUGUGUUCUCUAGAUCAGU	ACATAAAAAAAT	8
hsa-miR-651	346	8:1:1	-6.8	-5.58	-1.21	UUUAGGAUAAGCUUGACUUUUG	ATTCTACCA	8
hsa-miR-590-3p	46	8:1:0	-5.4	-4.3	-1.09	UAAUUUUAUGUAUAAGCUAGU	TATAATTAA	8

hsa-miR-548l	55	8:1:1	-5.61	-4.57	-1.03	AAAAGUAUUUGCGGGUUUUGUC	ATATTTAAAT	8
hsa-miR-532-5p	66	8:1:1	-7.3	-6.35	-0.94	CAUGCCUUGAGUGUAGGACCGU	AAGGAATACAT	8
hsa-miR-630	68	8:1:1	-8.3	-7.53	-0.76	AGUAUUCUGUACCAGGGAAGGU	GGAATACAT	8
hsa-miR-548p	114	8:1:1	-12.7	-12.41	-0.28	UAGCAAAAACUGCAGUUACUUU	TTTTTTTTTTG	8
hsa-miR-409-3p	333	8:1:1	-6.2	-5.95	-0.24	GAAUGUUGCUCGGUGAACCCCU	CAATATTATC	8
hsa-miR-578	73	8:1:1	-8.7	-8.47	-0.22	CUUCUUGUGCUCUAGGAUUGU	ACATGAAATAT	8
hsa-miR-500	66	8:1:1	-6.5	-6.35	-0.14	UAAUCCUUGCUACCUGGGUGAGA	AAGGAATACATGAA	8
hsa-miR-549	105	8:1:1	-7.4	-7.49	0.09	UGACAACUAUGGAUGAGCUCU	AATTGTTGTTTTT	8
hsa-miR-384	65	8:1:1	-5.5	-5.65	0.15	AUUCCUAGAAAUUGUUCAUA	TAAGGAATACATG	8
hsa-miR-548b-5p	344	8:1:1	-7	-7.23	0.23	AAAAGUAAUUGUGGUUUUGGCC	TTATTCTACCA	8
hsa-miR-584	26	8:1:0	-2.9	-3.37	0.47	UUAUGGUUUGCCUGGGACUGAG	AAACATAAAAAAATAAT	8
hsa-miR-548d-5p	276	8:1:0	-12.5	-13.1	0.6	AAAAGUAAUUGUGGUUUUUGCC	TTACTATATATATAT	8
hsa-miR-433	340	8:1:1	-5.77	-6.41	0.64	UACGGUGAGCCUGUCAUUAUUC	ATCATTATTC	8
hsa-miR-587	65	8:1:1	-5	-5.65	0.65	UUUCCAUAGGUGAUGAGUCAC	TAAGGAATACAT	8
hsa-miR-568	43	8:1:1	-3.2	-4.09	0.89	AUGUAUAAAUGUAUACACAC	ATATATAATTAAATAT	8
hsa-miR-548b-5p	276	8:1:0	-12.2	-13.1	0.9	AAAAGUAAUUGUGGUUUUGGCC	TTACTATAT	8
hsa-miR-578	30	8:1:1	-2.6	-3.54	0.94	CUUCUUGUGCUCUAGGAUUGU	ATAAAAAA	8

hsa-miR-664	18	8:1:0	0	-1.01	1.01	ACUGGCUAGGGAAAAUGAUUGGAU	AATAAATA	8
hsa-miR-513a-3p	135	8:1:0	-7.9	-8.94	1.04	UAAAUUUCACCUUUCUGAGAAGG	TAAATTTATTA	8
hsa-miR-190	80	8:1:1	-7.9	-8.94	1.04	UGAUAUGUUUGAUAUAUUAGGU	ATATATAATATTTT	8
hsa-miR-621	123	8:1:1	-11.5	-12.56	1.06	GGCUAGCAACAGCGCUUACCU	TGTTAGAATATT	8
hsa-miR-137	67	8:1:0	-6.3	-7.36	1.06	UUAUUGCUUAAGAAUACGCGUAG	AGGAATACATGAA	8
hsa-miR-155	340	8:1:0	-5.3	-6.41	1.11	UUAAUGCUAAUCGUGAUAGGGGU	ATCATTATTCTACC	8
hsa-miR-205	63	8:1:0	-3.6	-4.71	1.11	UCCUUCAUUCCACCGGAGUCUG	ATTAAGGA	8
hsa-miR-1244	265	8:0:1	-11.1	-12.33	1.23	AAGUAGUUGGUUUGUAUGAGAUGGUU	AACTATTATAATTAC	8
hsa-miR-580	337	8:1:1	-4	-5.31	1.31	UAAUGAUUCAUCAGACUCAGAU	ATTATCATTATT	8
hsa-miR-1322	338	8:1:1	-4.9	-6.26	1.36	GAUGAUGCUGCUGAUGCUG	TTATCATTATTCTAC	8
hsa-miR-190b	41	8:1:1	-3.61	-5.04	1.43	UGAUAUGUUUGAUAUUGGGUU	ATATATATAAT	8
hsa-miR-548n	88	8:1:1	-6.9	-8.43	1.53	CAAAAGUAAUUGUGGAUUUUGU	TATTTTTCATAAAATG	8
hsa-miR-219-2-3p	343	8:1:1	-5.3	-6.84	1.54	UGAUUGUCCAAACGCAAUUCU	ATTATTCTACCATATCA	8
hsa-miR-188-5p	66	8:1:1	-4.8	-6.35	1.55	CAUCCCUUGCAUGGUGGAGGG	AAGGAATA	8
hsa-miR-569	342	8:1:1	-4.6	-6.16	1.56	AGUUAAUGAAUCCUGGAAAGU	CATTATTCTACCAT	8
hsa-miR-548p	120	8:0:1	-11.3	-12.9	1.6	UAGCAAAAACUGCAGUUACUUU	TTTTGTTAGAA	8
hsa-miR-876-5p	68	8:1:1	-5.8	-7.53	1.73	UGGAUUUCUUUGUGAAUCACCA	GGAATACATGAAATATA	8

hsa-miR-190	41	8:1:1	-3.1	-5.04	1.94	UGAUAUGUUUGAUAUAUUAGGU	ATATATATAATTAA	8
hsa-miR-129-5p	30	8:1:1	-1.6	-3.54	1.94	CUUUUUGCGGUCUGGGCUUGC	ATAAAAAAATAATATAT	8
hsa-miR-561	53	8:1:1	-2.4	-4.55	2.15	AUCAAGGAUCUUAAACUUUGCC	AAATATTTA	8
hsa-miR-513a-3p	77	8:1:0	-6.3	-8.52	2.22	UAAAUUUCACCUUUCUGAGAAGG	GAAATATATAATAT	8
hsa-miR-548l	222	8:1:1	-6.01	-8.24	2.23	AAAAGUAUUUGCGGGUUUUGUC	ATATTATTTAT	8
hsa-miR-511	26	8:1:1	-1.1	-3.37	2.27	GUGUCUUUUGCUCUGCAGUCA	AAACATAAAAAAATAAT	8
hsa-miR-297	328	8:1:0	-7.3	-9.6	2.3	AUGUAUGUGUGCAUGUGCAUG	AAATACAATAT	8
hsa-miR-374a	240	8:1:0	-7.6	-9.92	2.32	UUAUAAUACAACCUGAUAAGUG	TATTATATATATATTA	8
hsa-miR-548d-5p	344	8:1:1	-4.9	-7.23	2.33	AAAAGUAAUUGUGGUUUUUGCC	TTATTCTA	8
hsa-miR-548l	87	8:0:1	-6	-8.34	2.34	AAAAGUAUUUGCGGGUUUUGUC	ATATTTTTCATA	8
hsa-miR-1259	41	8:1:1	-2.7	-5.04	2.34	UCCCUGAGACCCUUUAACCUGUGA	ATATATATAATTAAAT	8
hsa-miR-656	38	8:1:0	-3.7	-6.07	2.37	AGGUUGCCUGUGAGGUGUUCA	ATAATATATAT	8
hsa-miR-656	84	8:0:0	-6	-8.41	2.41	AGGUUGCCUGUGAGGUGUUCA	ATAATATTT	8
hsa-miR-1278	333	8:1:1	-3.5	-5.95	2.45	UAGUACUGUGCAUAUCAUCUAU	CAATATTA	8
hsa-miR-548d-3p	111	8:1:0	-8.9	-11.42	2.52	CAAAAACCACAGUUUCUUUUGC	TGTTTTTTTTTTTG	8
hsa-miR-653	322	8:0:0	-8.2	-10.85	2.65	GUGUUGAAACAAUCUCUACUG	TTCAACAAATACAAT	8
hsa-miR-633	47	8:1:1	-1.6	-4.29	2.69	CUAAUAGUAUCUACCACAAUAAA	ATAATTAAA	8

hsa-miR-190b	80	8:1:1	-6.2	-8.94	2.74	UGAUAUGUUUGAUAUUGGGUU	ATATATAATATTTT	8
hsa-miR-380	241	8:0:1	-7.1	-9.86	2.76	UGGUUGACCAUAGAACAUGCGC	ATTATATA	8
hsa-miR-633	337	8:1:1	-2.5	-5.31	2.81	CUAAUAGUAUCUACCACAAUAAA	ATTATCATT	8
hsa-miR-1270	320	8:1:1	-8.33	-11.23	2.9	CUGGAGAUAUGGAAGAGCUGUGU	ATTTCAACAAATAC	8
hsa-miR-548j	276	8:1:0	-10.2	-13.1	2.9	AAAAGUAAUUGCGGUCUUUGGU	TTACTATATAT	8
hsa-miR-935	103	8:1:1	-6.1	-9.05	2.95	CCAGUUACCGCUUCCGCUACCGC	GTAATTGTTG	8
hsa-miR-548l	335	8:1:1	-2.8	-5.78	2.98	AAAAGUAUUUGCGGGUUUUGUC	ATATTATCATTATTCT	8
hsa-miR-1322	341	8:1:1	-3	-6.13	3.13	GAUGAUGCUGCUGAUGCUG	TCATTATTCTACC	8
hsa-miR-380	40	8:1:1	-2.3	-5.45	3.15	UGGUUGACCAUAGAACAUGCGC	AATATATATAATT	8
hsa-miR-548c-5p	276	8:1:0	-9.9	-13.1	3.2	AAAAGUAAUUGCGGUUUUUGCC	TTACTATATA	8
hsa-miR-620	320	8:1:1	-8	-11.23	3.23	AUGGAGAUAGAUAUAGAAAU	ATTTCAACAAATACAAT	8
hsa-miR-548b-5p	225	8:1:1	-5.95	-9.18	3.23	AAAAGUAAUUGUGGUUUUGGCC	TTATTTATTAATATA	8
hsa-miR-190	293	8:1:1	-10.9	-14.2	3.3	UGAUAUGUUUGAUAUAUUAGGU	ATATATAT	8
hsa-miR-374a	141	8:1:0	-5.29	-8.59	3.3	UUAUAAUACAACCUGAUAAGUG	TATTATAAAAGTAT	8
hsa-miR-155	150	8:1:1	-9	-12.32	3.32	UUAAUGCUAAUCGUGAUAGGGGU	AGTATTAATAT	8
hsa-miR-889	334	8:1:0	-2.6	-5.93	3.33	AAUGGCUGUCCGUAGUAUGGUC	AATATTATCATTATTCT	8
hsa-miR-429	333	8:1:0	-2.6	-5.95	3.35	UAAUACUGUCUGGUAAAACCGU	CAATATTATCATTATTC	8

hsa-miR-568	242	8:1:1	-7	-10.44	3.44	AUGUAUAAAUGUAUACACAC	TTATATATATATTATA	8
hsa-miR-590-3p	85	8:1:0	-4.8	-8.28	3.48	UAAUUUUAUGUAUAAGCUAGU	TAATATTTTTCATA	8
hsa-miR-1259	51	8:1:1	-0.9	-4.5	3.6	UCCCUGAGACCCUUUAACCUGUGA	TTAAATATT	8
hsa-miR-380	84	8:1:1	-4.8	-8.41	3.61	UGGUUGACCAUAGAACAUGCGC	ATAATATT	8
hsa-miR-548a-5p	225	8:1:1	-5.55	-9.18	3.63	AAAAGUAAUUGCGAGUUUUACC	TTATTTATTAATA	8
hsa-miR-522	54	8:1:1	-0.9	-4.57	3.67	CUCUAGAGGGAAGCGCUUUCUG	AATATTTAAATTAAGG	8
hsa-miR-549	108	8:1:1	-6.1	-9.81	3.71	UGACAACUAUGGAUGAGCUCU	TGTTGTTTTTTTT	8
hsa-miR-1826	48	8:1:1	-0.7	-4.44	3.74	UUUGGCAAUGGUAGAACUCACACU	TAATTAAAT	8
hsa-miR-548h	344	8:1:1	-3.4	-7.23	3.83	AAAAGUAAUCGCGGUUUUUGUC	TTATTCTACCAT	8
hsa-miR-187	71	8:1:1	-4.3	-8.13	3.83	GGCUACAACACAGGACCCGGGC	ATACATGAAA	8
hsa-miR-429	149	8:1:0	-7.5	-11.35	3.85	UAAUACUGUCUGGUAAAACCGU	AAGTATTAATA	8
hsa-miR-590-3p	146	8:1:0	-4.3	-8.16	3.86	UAAUUUUAUGUAUAAGCUAGU	TAAAAGTATTA	8
hsa-miR-151-3p	345	8:1:1	-3	-6.87	3.87	UCGAGGAGCUCACAGUCUAGU	TATTCTACCATATCACT	8
hsa-miR-1259	143	8:1:1	-4.8	-8.67	3.87	UCCCUGAGACCCUUUAACCUGUGA	TTATAAAAGTAT	8
hsa-miR-548p	115	8:1:1	-8.9	-12.8	3.9	UAGCAAAAACUGCAGUUACUUU	TTTTTTTTTGT	8
hsa-miR-1259	39	8:1:0	-1.6	-5.54	3.94	UCCCUGAGACCCUUUAACCUGUGA	TAATATATAT	8
hsa-miR-190	213	8:1:1	-10.9	-14.88	3.98	UGAUAUGUUUGAUAUAUUAGGU	ACATATTAAA	8

hsa-miR-548j	344	8:1:1	-3.1	-7.23	4.13	AAAAGUAAUUGCGGUCUUUGGU	TTATTCTACCATATCA	8
hsa-miR-633	142	8:1:1	-4.5	-8.65	4.15	CUAAUAGUAUCUACCACAAUAAA	ATTATAAAA	8
hsa-miR-944	86	8:1:1	-4	-8.17	4.17	AAAUUAUUGUACAUCGGAUGAG	AATATTTTTCATAA	8
hsa-miR-548p	108	8:1:1	-5.5	-9.81	4.31	UAGCAAAAACUGCAGUUACUUU	TGTTGTTTTTTTTTTG	8
hsa-miR-581	330	8:1:1	-4.5	-8.82	4.32	UCUUGUGUUCUCUAGAUCAGU	ATACAATATTA	8
hsa-miR-548i	344	8:1:1	-2.9	-7.23	4.33	AAAAGUAAUUGCGGAUUUUGCC	TTATTCTACCA	8
hsa-miR-1259	242	8:0:1	-6.1	-10.44	4.34	UCCCUGAGACCCUUUAACCUGUGA	TTATATATATATTATAA	8
hsa-miR-561	136	8:1:1	-4.5	-8.84	4.34	AUCAAGGAUCUUAAACUUUGCC	AAATTTATTAT	8
hsa-miR-511	34	8:1:1	-0.3	-4.71	4.41	GUGUCUUUUGCUCUGCAGUCA	AAAAATAATATAT	8
hsa-miR-367	101	8:1:1	-6.2	-10.67	4.47	ACUGUUGCUAAUAUGCAACUCU	ATGTAATTGTTGTTTT	8
hsa-miR-643	145	8:1:1	-3.56	-8.09	4.53	ACUUGUAUGCUAGCUCAGGUAG	ATAAAAGTA	8
hsa-miR-1246	70	8:1:1	-3.4	-8.06	4.66	AAUGGAUUUUUGGAGCAGG	AATACATGAAAT	8
hsa-miR-548p	116	8:1:1	-7.9	-12.69	4.79	UAGCAAAAACUGCAGUUACUUU	TTTTTTTTG	8
hsa-miR-548j	87	8:1:1	-3.54	-8.34	4.8	AAAAGUAAUUGCGGUCUUUGGU	ATATTTTT	8
hsa-miR-519c-3p	102	8:1:1	-5.4	-10.22	4.82	AAAGUGCAUCUUUUUAGAGGAU	TGTAATTGTTGT	8
hsa-miR-190	180	8:1:1	-8.29	-13.12	4.83	UGAUAUGUUUGAUAUAUUAGGU	ATATATATATAA	8
hsa-miR-380	142	8:1:1	-3.8	-8.65	4.85	UGGUUGACCAUAGAACAUGCGC	ATTATAAA	8

hsa-miR-651	142	8:1:1	-3.8	-8.65	4.85	UUUAGGAUAAGCUUGACUUUUG	ATTATAAAAGTATTAA	8
hsa-miR-374b	141	8:1:0	-3.7	-8.59	4.89	AUAUAAUACAACCUGCUAAGUG	TATTATAAAAGTATT	8
hsa-miR-548c-5p	344	8:1:1	-2.3	-7.23	4.93	AAAAGUAAUUGCGGUUUUUGCC	TTATTCTACCAT	8
hsa-miR-548l	130	8:1:1	-5.3	-10.25	4.95	AAAAGUAUUUGCGGGUUUUGUC	ATATTTAAATTTATT	8
hsa-miR-144	250	8:1:1	-8.7	-13.66	4.96	GGAUAUCAUCAUAUACUGUAAG	ATATTATAATATTACA	8
hsa-miR-190b	213	8:1:1	-9.9	-14.88	4.98	UGAUAUGUUUGAUAUUGGGUU	ACATATTAAATATT	8
hsa-miR-144	239	8:1:1	-5.1	-10.09	4.99	GGAUAUCAUCAUAUACUGUAAG	ATATTATATATAT	8
hsa-miR-218	328	8:1:1	-4.6	-9.6	5	UUGUGCUUGAUCUAACCAUGU	AAATACAATATTATCA	8
hsa-miR-203	131	8:1:1	-5.2	-10.2	5	AGUGGUUCUUAACAGUUCAACAGUU	TATTTAAATTT	8
hsa-miR-519a	102	8:1:1	-5.22	-10.22	5	CUCUAGAGGGAAGCGCUUUCUG	TGTAATTGTTGTTTT	8
hsa-miR-548d-5p	225	8:1:1	-4.15	-9.18	5.03	AAAAGUAAUUGUGGUUUUUGCC	TTATTTATTAATAT	8
hsa-miR-548b-5p	87	8:1:1	-3.3	-8.34	5.04	AAAAGUAAUUGUGGUUUUGGCC	ATATTTTTCATAAA	8
hsa-miR-548h	87	8:1:1	-3.3	-8.34	5.04	AAAAGUAAUCGCGGUUUUUGUC	ATATTTTTCA	8
hsa-miR-137	37	8:1:1	-0.8	-5.88	5.08	UUAUUGCUUAAGAAUACGCGUAG	AATAATATAT	8
hsa-miR-380	260	8:1:0	-7	-12.2	5.2	UGGUUGACCAUAGAACAUGCGC	ATTACAACTATTATAA	8
hsa-miR-491-3p	93	8:1:0	-4.66	-9.9	5.24	CUUAUGCAAGAUUCCCUUCUAC	TTCATAAAAT	8
hsa-miR-548d-5p	87	8:1:1	-3.1	-8.34	5.24	AAAAGUAAUUGUGGUUUUUGCC	ATATTTTTCA	8

hsa-miR-584	37	8:1:1	-0.6	-5.88	5.28	UUAUGGUUUGCCUGGGACUGAG	AATAATATATAT	8
hsa-miR-548n	226	8:1:1	-4.4	-9.73	5.33	CAAAAGUAAUUGUGGAUUUUGU	TATTTATT	8
hsa-miR-577	224	8:1:1	-3.4	-8.75	5.35	UAGAUAAAAUAUUGGUACCUG	ATTATTTATTAA	8
hsa-miR-885-5p	103	8:1:0	-3.7	-9.05	5.35	UCCAUUACACUACCCUGCCUCU	GTAATTGTTGTTTTT	8
hsa-miR-559	140	8:1:1	-3.1	-8.46	5.36	UAAAGUAAAUAUGCACCAAAA	TTATTATAAAAG	8
hsa-miR-28-3p	138	8:1:1	-2.79	-8.16	5.37	CACUAGAUUGUGAGCUCCUGGA	ATTTATTATA	8
hsa-miR-548a-5p	276	8:1:0	-7.7	-13.1	5.4	AAAAGUAAUUGCGAGUUUUACC	TTACTATATAT	8
hsa-miR-548p	117	8:1:1	-7.2	-12.64	5.44	UAGCAAAAACUGCAGUUACUUU	TTTTTTTGTT	8
hsa-miR-380	224	8:1:1	-3.3	-8.75	5.45	UGGUUGACCAUAGAACAUGCGC	ATTATTTATT	8
hsa-miR-633	224	8:1:1	-3.3	-8.75	5.45	CUAAUAGUAUCUACCACAAUAAA	ATTATTTATTAATA	8
hsa-miR-369-3p	239	8:1:0	-4.6	-10.09	5.49	AAUAAUACAUGGUUGAUCUUU	ATATTATATAT	8
hsa-miR-203	319	8:0:1	-6.6	-12.1	5.5	AGUGGUUCUUAACAGUUCAACAGUU	TATTTCAAC	8
hsa-miR-590-3p	189	8:1:0	-8	-13.54	5.54	UAAUUUUAUGUAUAAGCUAGU	TAAAACTAATAAGTATT	8
hsa-miR-590-3p	97	8:1:0	-5.4	-10.94	5.54	UAAUUUUAUGUAUAAGCUAGU	TAAAATGTAATT	8
hsa-miR-568	285	8:1:1	-5.9	-11.5	5.6	AUGUAUAAAUGUAUACACAC	ATATATAAATATATATA	8
hsa-miR-450b-5p	143	8:1:1	-3	-8.67	5.67	UUUUGCAAUAUGUUCCUGAAUA	TTATAAAAGTATTAA	8

hsa-miR-203	90	8:1:1	-2.8	-8.48	5.68	AGUGGUUCUUAACAGUUCAACAGUU	TTTTTCATAAA	8
hsa-miR-548h	276	8:1:0	-7.4	-13.1	5.7	AAAAGUAAUCGCGGUUUUUGUC	TTACTATAT	8
hsa-miR-633	340	8:1:1	-0.7	-6.41	5.71	CUAAUAGUAUCUACCACAAUAAA	ATCATTATTCTAC	8
hsa-miR-494	89	8:1:0	-2.8	-8.52	5.72	AGGUUGUCCGUGUUGUCUUCUCU	ATTTTTCATAAAATGT	8
hsa-miR-548i	225	8:1:1	-3.4	-9.18	5.78	AAAAGUAAUUGCGGAUUUUGCC	TTATTTATTA	8
hsa-miR-429	223	8:1:1	-2.8	-8.62	5.82	UAAUACUGUCUGGUAAAACCGU	TATTATTTATTA	8
hsa-miR-628-5p	124	8:1:1	-6.2	-12.03	5.83	AUGCUGACAUAUUUACUAGAGG	GTTAGAATATTTAAATT	8
hsa-miR-221	101	8:1:1	-4.8	-10.67	5.87	ACCUGGCAUACAAUGUAGAUUU	ATGTAATTGTTGTT	8
hsa-miR-190	235	8:1:1	-5.82	-11.7	5.88	UGAUAUGUUUGAUAUAUUAGGU	ATATATATT	8
hsa-miR-548g	163	8:1:1	-5.8	-11.69	5.89	UGCAAAAGUAAUUGCAGUUUUUG	ATATTTTTTTT	8
hsa-miR-548j	225	8:1:1	-3.25	-9.18	5.93	AAAAGUAAUUGCGGUCUUUGGU	TTATTTATTAATATATA	8
hsa-miR-144	163	8:1:1	-5.7	-11.69	5.99	GGAUAUCAUCAUAUACUGUAAG	ATATTTTTTTTTTAAA	8
hsa-miR-1179	128	8:1:1	-5	-11.03	6.03	AAGCAUUCUUUCAUUGGUUGG	GAATATTTAAATTT	8
hsa-miR-1259	80	8:1:1	-2.9	-8.94	6.04	UCCCUGAGACCCUUUAACCUGUGA	ATATATAATATTTTTC	8
hsa-miR-519b-3p	102	8:1:1	-4.1	-10.22	6.12	AAAGUGCAUCCUUUUAGAGGUU	TGTAATTGT	8
hsa-miR-374b	240	8:1:0	-3.79	-9.92	6.13	AUAUAAUACAACCUGCUAAGUG	TATTATATATATATTAT	8
hsa-miR-302f	102	8:1:1	-4.09	-10.22	6.13	UAAUUGCUUCCAUGUUU	TGTAATTGTT	8

hsa-miR-548c-5p	87	8:1:1	-2.2	-8.34	6.14	AAAAGUAAUUGCGGUUUUUGCC	ATATTTTTCATA	8
hsa-miR-369-3p	140	8:1:0	-2.3	-8.46	6.16	AAUAAUACAUGGUUGAUCUUU	TTATTATAAAA	8
hsa-miR-186	138	8:1:1	-2	-8.16	6.16	CAAAGAAUUCUCCUUUUGGGCU	ATTTATTAT	8
hsa-miR-633	266	8:1:0	-5.5	-11.69	6.19	CUAAUAGUAUCUACCACAAUAAA	ACTATTATAA	8
hsa-miR-548a-5p	344	8:1:1	-1	-7.23	6.23	AAAAGUAAUUGCGAGUUUUACC	TTATTCTACCATATCA	8
hsa-miR-1272	270	8:1:1	-5	-11.27	6.27	GAUGAUGAUGGCAGCAAAUUCUGAAA	TTATAATT	8
hsa-miR-548i	276	8:1:0	-6.8	-13.1	6.3	AAAAGUAAUUGCGGAUUUUGCC	TTACTATATATATATA	8
hsa-miR-600	122	8:1:1	-6.8	-13.12	6.32	ACUUACAGACAAGAGCCUUGCUC	TTGTTAGAATA	8
hsa-miR-802	111	8:1:1	-5.1	-11.42	6.32	CAGUAACAAAGAUUCAUCCUUGU	TGTTTTTTTTTT	8
hsa-miR-548a-5p	87	8:1:1	-2.02	-8.34	6.32	AAAAGUAAUUGCGAGUUUUACC	ATATTTTTCATAAAATG	8
hsa-miR-656	332	8:1:0	-0.7	-7.1	6.4	AGGUUGCCUGUGAGGUGUUCA	ACAATATTATCATTATT	8
hsa-miR-33a	99	8:1:1	-4.5	-10.92	6.42	GUGCAUUGUAGUUGCAUUGCA	AAATGTAATTGTTGTT	8
hsa-miR-548k	87	8:1:1	-1.92	-8.34	6.42	AAAAGUACUUGCGGAUUUUGCU	ATATTTTTCATA	8
hsa-miR-548i	87	8:1:1	-1.92	-8.34	6.42	AAAAGUAAUUGCGGAUUUUGCC	ATATTTTTCATAAAATG	8
hsa-miR-374a	268	8:1:0	-4.4	-10.85	6.45	UUAUAAUACAACCUGAUAAGUG	TATTATAATTACTA	8
hsa-miR-514	151	8:1:1	-6.6	-13.06	6.46	UACUCUGGAGAGUGACAAUCAUG	GTATTAATA	8
hsa-miR-190b	293	8:1:1	-7.7	-14.2	6.5	UGAUAUGUUUGAUAUUGGGUU	ATATATATATAAT	8

hsa-miR-656	77	8:1:1	-2	-8.52	6.52	AGGUUGCCUGUGAGGUGUUCA	GAAATATATA	8
hsa-miR-548d-3p	112	8:1:0	-5	-11.54	6.54	CAAAAACCACAGUUUCUUUUGC	GTTTTTTTT	8
hsa-miR-190b	235	8:1:1	-5.1	-11.7	6.6	UGAUAUGUUUGAUAUUGGGUU	ATATATATTATAT	8
hsa-miR-568	244	8:1:1	-5.1	-11.73	6.63	AUGUAUAAAUGUAUACACAC	ATATATATATTA	8
hsa-miR-495	112	8:1:0	-4.9	-11.54	6.64	GAAGUUGCCCAUGUUAUUUUCG	GTTTTTTTTTTGTT	8
hsa-miR-655	139	8:1:0	-1.3	-7.97	6.67	AGAGGUUAUCCGUGUUAUGUUC	TTTATTATAAAGTAT	8
hsa-miR-496	303	8:0:1	-7.5	-14.18	6.68	UGAGUAUUACAUGGCCAAUCUC	AATACTTA	8
hsa-miR-522	266	8:1:1	-5	-11.69	6.69	CUCUAGAGGGAAGCGCUUUCUG	ACTATTATAA	8
hsa-miR-499-5p	150	8:1:0	-5.6	-12.32	6.72	UUAAGACUUGCAGUGAUGUUU	AGTATTAATATATAT	8
hsa-miR-548h	225	8:1:1	-2.43	-9.18	6.75	AAAAGUAAUCGCGGUUUUUGUC	TTATTTAT	8
hsa-miR-1264	86	8:1:1	-1.4	-8.17	6.77	CAAGUCUUAUUUGAGCACCUGUU	AATATTTTT	8
hsa-miR-548c-5p	225	8:1:1	-2.4	-9.18	6.78	AAAAGUAAUUGCGGUUUUUGCC	TTATTTATTAATA	8
hsa-miR-190b	157	8:1:1	-6.7	-13.48	6.78	UGAUAUGUUUGAUAUUGGGUU	ATATATATATTTTTTT	8
hsa-miR-1259	207	8:0:1	-8.7	-15.54	6.84	UCCCUGAGACCCUUUAACCUGUGA	TTATATACATATT	8
hsa-miR-655	228	8:1:0	-3.2	-10.06	6.86	AGAGGUUAUCCGUGUUAUGUUC	TTTATTAATATAT	8
hsa-miR-380	217	8:1:0	-6	-12.93	6.93	UGGUUGACCAUAGAACAUGCGC	ATTAAATATT	8
hsa-miR-576-5p	124	8:1:1	-5.1	-12.03	6.93	AUUCUAAUUUCUCCACGUCUUU	GTTAGAATAT	8

hsa-miR-1278	149	8:1:1	-4.4	-11.35	6.95	UAGUACUGUGCAUAUCAUCUAU	AAGTATTAATA	8
hsa-miR-380	252	8:1:1	-6.2	-13.17	6.97	UGGUUGACCAUAGAACAUGCGC	ATTATAATATTACAAC	8
hsa-miR-25	101	8:1:1	-3.7	-10.67	6.97	AGGCGGAGACUUGGGCAAUUG	ATGTAATTGTTGTTTT	8
hsa-miR-1259	225	8:1:1	-2.2	-9.18	6.98	UCCCUGAGACCCUUUAACCUGUGA	TTATTTATTAAT	8
hsa-miR-944	271	8:1:1	-3.9	-10.93	7.03	AAAUUAUUGUACAUCGGAUGAG	TATAATTACTATA	8
hsa-miR-92a	101	8:1:1	-3.6	-10.67	7.07	UAUUGCACUUGUCCCGGCCUGU	ATGTAATTGTTGT	8
hsa-miR-92b	101	8:1:1	-3.6	-10.67	7.07	AGGGACGGGACGCGGUGCAGUG	ATGTAATTGTTG	8
hsa-miR-410	109	8:1:1	-3.7	-10.78	7.08	AGGUUGUCUGUGAUGAGUUCG	GTTGTTTTTT	8
hsa-miR-380	269	8:1:1	-3.7	-10.79	7.09	UGGUUGACCAUAGAACAUGCGC	ATTATAATTACTATA	8
hsa-miR-655	150	8:1:0	-5.2	-12.32	7.12	AGAGGUUAUCCGUGUUAUGUUC	AGTATTAATATATAT	8
hsa-miR-1246	79	8:1:1	-1.7	-8.85	7.15	AAUGGAUUUUUGGAGCAGG	AATATATAATA	8
hsa-miR-374b	268	8:1:0	-3.6	-10.85	7.25	AUAUAAUACAACCUGCUAAGUG	TATTATAATTACT	8
hsa-miR-568	209	8:1:0	-7.6	-14.94	7.34	AUGUAUAAAUGUAUACACAC	ATATACATATTAAATAT	8
hsa-miR-539	138	8:1:1	-0.8	-8.16	7.36	GGAGAAAUUAUCCUUGGUGUGU	ATTTATTA	8
hsa-miR-190	157	8:1:1	-6.1	-13.48	7.38	UGAUAUGUUUGAUAUAUUAGGU	ATATATATATT	8
hsa-miR-568	308	8:1:1	-5.8	-13.22	7.42	AUGUAUAAAUGUAUACACAC	TTATATATATA	8
hsa-miR-410	241	8:1:0	-2.4	-9.86	7.46	AGGUUGUCUGUGAUGAGUUCG	ATTATATATATATTATA	8

hsa-miR-577	167	8:1:1	-4.3	-11.86	7.56	UAGAUAAAAUAUUGGUACCUG	TTTTTTTTTAAAAATAT	8
hsa-miR-568	283	8:1:1	-3.9	-11.47	7.57	AUGUAUAAAUGUAUACACAC	ATATATATAAA	8
hsa-miR-548c-3p	164	8:1:0	-3.83	-11.44	7.61	CAAAAAUCUCAAUUACUUUUGC	TATTTTTTT	8
hsa-miR-568	310	8:1:1	-5.5	-13.24	7.74	AUGUAUAAAUGUAUACACAC	ATATATATATAT	8
hsa-miR-548m	305	8:1:1	-6.6	-14.34	7.74	CAAAGGUAUUUGUGGUUUUUG	TACTTATATATAT	8
hsa-miR-190b	180	8:1:1	-5.37	-13.12	7.75	UGAUAUGUUUGAUAUUGGGUU	ATATATAT	8
hsa-miR-559	163	8:1:1	-3.9	-11.69	7.79	UAAAGUAAAUAUGCACCAAAA	ATATTTTTT	8
hsa-miR-568	159	8:1:1	-5.7	-13.52	7.82	AUGUAUAAAUGUAUACACAC	ATATATATTT	8
hsa-miR-548n	164	8:1:1	-3.6	-11.44	7.84	CAAAAGUAAUUGUGGAUUUUGU	TATTTTTTTTTTAAA	8
hsa-miR-561	220	8:1:1	-2.4	-10.26	7.86	AUCAAGGAUCUUAAACUUUGCC	AAATATTATT	8
hsa-miR-568	184	8:1:1	-4.9	-12.81	7.91	AUGUAUAAAUGUAUACACAC	ATATATAAA	8
hsa-miR-656	127	8:1:0	-2.1	-10.13	8.03	AGGUUGCCUGUGAGGUGUUCA	AGAATATTTAAATTTA	8
hsa-miR-655	238	8:1:0	-2.7	-10.74	8.04	AGAGGUUAUCCGUGUUAUGUUC	TATATTATATATATA	8
hsa-miR-577	169	8:1:1	-4.4	-12.49	8.09	UAGAUAAAAUAUUGGUACCUG	TTTTTTTA	8
hsa-miR-548c-3p	88	8:1:0	-0.3	-8.43	8.13	CAAAAAUCUCAAUUACUUUUGC	TATTTTTCATA	8
hsa-miR-429	85	8:1:1	-0.1	-8.28	8.18	UAAUACUGUCUGGUAAAACCGU	TAATATTTT	8
hsa-miR-577	168	8:1:1	-3.91	-12.17	8.26	UAGAUAAAAUAUUGGUACCUG	TTTTTTTTAAAAATA	8

hsa-miR-568	314	8:1:1	-5.8	-14.16	8.36	AUGUAUAAAUGUAUACACAC	ATATATATTTCAACA	8
hsa-miR-656	255	8:0:0	-4.1	-12.53	8.43	AGGUUGCCUGUGAGGUGUUCA	ATAATATTACAAC	8
hsa-miR-1259	157	8:1:1	-5	-13.48	8.48	UCCCUGAGACCCUUUAACCUGUGA	ATATATAT	8
hsa-miR-633	269	8:1:1	-2.3	-10.79	8.49	CUAAUAGUAUCUACCACAAUAAA	ATTATAAT	8
hsa-miR-656	239	8:1:0	-1.6	-10.09	8.49	AGGUUGCCUGUGAGGUGUUCA	ATATTATATATA	8
hsa-miR-548a-5p	204	8:1:1	-7.5	-16.03	8.53	AAAAGUAAUUGCGAGUUUUACC	TTATTATATACATATTA	8
hsa-miR-1246	179	8:1:1	-5.1	-13.63	8.53	AAUGGAUUUUUGGAGCAGG	AATATATATATAA	8
hsa-miR-630	301	8:1:1	-5.1	-13.67	8.57	AGUAUUCUGUACCAGGGAAGGU	ATAATACT	8
hsa-miR-548b-5p	204	8:1:1	-7.4	-16.03	8.63	AAAAGUAAUUGUGGUUUUGGCC	TTATTATATACATAT	8
hsa-miR-331-5p	304	8:1:1	-5.2	-13.88	8.68	CUAGGUAUGGUCCCAGGGAUCC	ATACTTATATATAT	8
hsa-miR-495	120	8:1:0	-4.22	-12.9	8.68	GAAGUUGCCCAUGUUAUUUUCG	TTTTGTTAGAATATTT	8
hsa-miR-1259	235	8:1:1	-3	-11.7	8.7	UCCCUGAGACCCUUUAACCUGUGA	ATATATATTATAT	8
hsa-miR-1290	177	8:1:1	-5	-13.7	8.7	UGGAUUUUUGGAUCAGGGA	AAAATATATATATAAAA	8
hsa-miR-568	281	8:1:1	-3.9	-12.63	8.73	AUGUAUAAAUGUAUACACAC	ATATATATATAAATA	8
hsa-miR-548f	88	8:1:1	0.3	-8.43	8.73	UGCAAAAGUAAUCACAGUUUUU	TATTTTTCA	8
hsa-miR-186	87	8:1:1	0.41	-8.34	8.75	CAAAGAAUUCUCCUUUUGGGCU	ATATTTTTCATAAAAT	8
hsa-miR-142-5p	239	8:1:1	-1.3	-10.09	8.79	CAUAAAGUAGAAAGCACUACU	ATATTATAT	8

hsa-miR-137	150	8:1:1	-3.5	-12.32	8.82	UUAUUGCUUAAGAAUACGCGUAG	AGTATTAATAT	8
hsa-miR-142-5p	241	8:1:1	-1	-9.86	8.86	CAUAAAGUAGAAAGCACUACU	ATTATATATATATTAT	8
hsa-miR-559	167	8:1:1	-3	-11.86	8.86	UAAAGUAAAUAUGCACCAAAA	TTTTTTTTTAA	8
hsa-miR-548l	304	8:1:0	-4.89	-13.88	8.99	AAAAGUAUUUGCGGGUUUUGUC	ATACTTATAT	8
hsa-miR-568	297	8:1:1	-4.9	-13.94	9.04	AUGUAUAAAUGUAUACACAC	ATATATAATACTTATA	8
hsa-miR-186	165	8:1:1	-2.4	-11.44	9.04	CAAAGAAUUCUCCUUUUGGGCU	ATTTTTTTT	8
hsa-miR-559	117	8:1:1	-3.6	-12.64	9.04	UAAAGUAAAUAUGCACCAAAA	TTTTTTTGTTAG	8
hsa-miR-656	241	8:1:0	-0.8	-9.86	9.06	AGGUUGCCUGUGAGGUGUUCA	ATTATATATA	8
hsa-miR-651	269	8:1:1	-1.7	-10.79	9.09	UUUAGGAUAAGCUUGACUUUUG	ATTATAATTA	8
hsa-miR-496	257	8:1:1	-3.4	-12.5	9.1	UGAGUAUUACAUGGCCAAUCUC	AATATTACAACTATT	8
hsa-miR-568	233	8:1:1	-3.3	-12.42	9.12	AUGUAUAAAUGUAUACACAC	TAATATATATTATATA	8
hsa-miR-548f	164	8:1:1	-2.3	-11.44	9.14	UGCAAAAGUAAUCACAGUUUUU	TATTTTTTTTTTAAAAA	8
hsa-miR-361-5p	92	8:1:1	-0.45	-9.6	9.15	UUAUCAGAAUCUCCAGGGGUAC	TTTCATAAAAT	8
hsa-miR-1278	302	8:1:1	-4.6	-13.81	9.21	UAGUACUGUGCAUAUCAUCUAU	TAATACTTATATATATA	8
hsa-miR-548n	114	8:1:1	-3.2	-12.41	9.21	CAAAAGUAAUUGUGGAUUUUGU	TTTTTTTTTTG	8
hsa-miR-374a	251	8:1:0	-4.2	-13.55	9.35	UUAUAAUACAACCUGAUAAGUG	TATTATAATAT	8
hsa-miR-568	246	8:1:1	-4.4	-13.77	9.37	AUGUAUAAAUGUAUACACAC	ATATATAT	8

hsa-miR-577	114	8:1:1	-3	-12.41	9.41	UAGAUAAAAUAUUGGUACCUG	TTTTTTTTTTGTTA	8
hsa-miR-548l	163	8:1:1	-2.26	-11.69	9.43	AAAAGUAUUUGCGGGUUUUGUC	ATATTTTTTTTTTA	8
hsa-miR-1261	308	8:1:1	-3.7	-13.22	9.52	AUGGAUAAGGCUUUGGCUU	TTATATATATA	8
hsa-miR-142-5p	278	8:1:0	-3.9	-13.42	9.52	CAUAAAGUAGAAAGCACUACU	ACTATATATATAT	8
hsa-miR-548k	201	8:1:1	-6.7	-16.22	9.52	AAAAGUACUUGCGGAUUUUGCU	GTATTATTATATACA	8
hsa-miR-590-3p	256	8:1:0	-3	-12.53	9.53	UAAUUUUAUGUAUAAGCUAGU	TAATATTACAAC	8
hsa-miR-568	207	8:1:1	-6	-15.54	9.54	AUGUAUAAAUGUAUACACAC	TTATATACATATT	8
hsa-miR-568	295	8:1:1	-4.4	-13.99	9.59	AUGUAUAAAUGUAUACACAC	ATATATATAATACTTA	8
hsa-miR-1259	308	8:1:1	-3.6	-13.22	9.62	UCCCUGAGACCCUUUAACCUGUGA	TTATATATATATATTC	8
hsa-miR-656	98	8:1:1	-1.3	-10.95	9.65	AGGUUGCCUGUGAGGUGUUCA	AAAATGTAA	8
hsa-miR-142-5p	165	8:1:1	-1.77	-11.44	9.67	CAUAAAGUAGAAAGCACUACU	ATTTTTTTTTT	8
hsa-miR-494	318	8:1:0	-2.8	-12.48	9.68	AGGUUGUCCGUGUUGUCUUCUCU	ATATTTCAACAA	8
hsa-miR-1259	218	8:1:1	-2.31	-12.03	9.72	UCCCUGAGACCCUUUAACCUGUGA	TTAAATATTATTTAT	8
hsa-miR-374b	251	8:1:0	-3.8	-13.55	9.75	AUAUAAUACAACCUGCUAAGUG	TATTATAAT	8
hsa-miR-633	231	8:1:1	-1.4	-11.2	9.8	CUAAUAGUAUCUACCACAAUAAA	ATTAATATATA	8
hsa-miR-369-3p	201	8:1:0	-6.4	-16.22	9.82	AAUAAUACAUGGUUGAUCUUU	GTATTATTATATA	8
hsa-miR-548d-5p	166	8:1:1	-1.7	-11.53	9.83	AAAAGUAAUUGUGGUUUUUGCC	TTTTTTTT	8

hsa-miR-568	312	8:1:1	-4.2	-14.05	9.85	AUGUAUAAAUGUAUACACAC	ATATATATATTTCAA	8
hsa-miR-568	182	8:1:1	-3	-12.88	9.88	AUGUAUAAAUGUAUACACAC	ATATATATAA	8
hsa-miR-203	259	8:1:1	-2.5	-12.4	9.9	AGUGGUUCUUAACAGUUCAACAGUU	TATTACAACTATTATA	8
hsa-miR-1272	253	8:1:1	-2.6	-12.55	9.95	GAUGAUGAUGGCAGCAAAUUCUGAAA	TTATAATATTACAACTA	8
hsa-miR-633	252	8:1:1	-3.1	-13.17	10.07	CUAAUAGUAUCUACCACAAUAAA	ATTATAAT	8
hsa-miR-559	116	8:1:1	-2.6	-12.69	10.09	UAAAGUAAAUAUGCACCAAAA	TTTTTTTTG	8
hsa-miR-590-3p	102	8:1:1	-0.1	-10.22	10.12	UAAUUUUAUGUAUAAGCUAGU	TGTAATTGTTGTTT	8
hsa-miR-548c-5p	166	8:1:1	-1.4	-11.53	10.13	AAAAGUAAUUGCGGUUUUUGCC	TTTTTTTTTTAAA	8
hsa-miR-369-3p	250	8:1:0	-3.5	-13.66	10.16	AAUAAUACAUGGUUGAUCUUU	ATATTATAA	8
hsa-miR-656	301	8:1:0	-3.5	-13.67	10.17	AGGUUGCCUGUGAGGUGUUCA	ATAATACTTA	8
hsa-miR-559	114	8:1:1	-2.2	-12.41	10.21	UAAAGUAAAUAUGCACCAAAA	TTTTTTTTTTGTT	8
hsa-miR-548m	162	8:1:1	-1.7	-11.99	10.29	CAAAGGUAUUUGUGGUUUUUG	TATATTTTTTT	8
hsa-miR-361-5p	121	8:1:1	-3	-13.33	10.33	UUAUCAGAAUCUCCAGGGGUAC	TTTGTTAGA	8
hsa-miR-548m	317	8:1:1	-2.8	-13.22	10.42	CAAAGGUAUUUGUGGUUUUUG	TATATTTCAACAAATAC	8
hsa-miR-1259	180	8:1:1	-2.7	-13.12	10.42	UCCCUGAGACCCUUUAACCUGUGA	ATATATATATA	8
hsa-miR-548d-5p	204	8:1:1	-5.6	-16.03	10.43	AAAAGUAAUUGUGGUUUUUGCC	TTATTATA	8

hsa-miR-1259	279	8:1:1	-3	-13.5	10.5	UCCCUGAGACCCUUUAACCUGUGA	CTATATATATATAAAT	8
hsa-miR-548b-5p	166	8:1:1	-0.98	-11.53	10.55	AAAAGUAAUUGUGGUUUUGGCC	TTTTTTTTTTAAAAAT	8
hsa-miR-153	279	8:1:1	-2.9	-13.5	10.6	UCAUUUUUGUGAUGUUGCAGCU	CTATATAT	8
hsa-miR-380	234	8:1:1	-1.7	-12.33	10.63	UGGUUGACCAUAGAACAUGCGC	AATATATATTATA	8
hsa-miR-641	165	8:1:1	-0.8	-11.44	10.64	AAAGACAUAGGAUAGAGUCACCUC	ATTTTTTTTTTA	8
hsa-miR-633	203	8:0:1	-5.4	-16.07	10.67	CUAAUAGUAUCUACCACAAUAAA	ATTATTATATACATA	8
hsa-miR-155	200	8:1:1	-5.6	-16.27	10.67	UUAAUGCUAAUCGUGAUAGGGGU	AGTATTATTATATAC	8
hsa-miR-374a	202	8:1:0	-5.5	-16.21	10.71	UUAUAAUACAACCUGAUAAGUG	TATTATTAT	8
hsa-miR-374b	202	8:1:0	-5.5	-16.21	10.71	AUAUAAUACAACCUGCUAAGUG	TATTATTATATACATA	8
hsa-miR-590-5p	197	8:1:1	-5	-15.73	10.73	GAGCUUAUUCAUAAAAGUGCAG	ATAAGTATTAT	8
hsa-miR-568	155	8:1:1	-2.7	-13.43	10.73	AUGUAUAAAUGUAUACACAC	TAATATATATATT	8
hsa-miR-613	258	8:1:1	-1.8	-12.55	10.75	AGGAAUGUUCCUUCUUUGCC	ATATTACAA	8
hsa-miR-944	303	8:1:1	-3.4	-14.18	10.78	AAAUUAUUGUACAUCGGAUGAG	AATACTTATATA	8
hsa-miR-889	257	8:1:1	-1.7	-12.5	10.8	AAUGGCUGUCCGUAGUAUGGUC	AATATTACAACTATT	8
hsa-miR-548j	204	8:1:1	-5.2	-16.03	10.83	AAAAGUAAUUGCGGUCUUUGGU	TTATTATATACATATT	8
hsa-miR-186	166	8:1:1	-0.7	-11.53	10.83	CAAAGAAUUCUCCUUUUGGGCU	TTTTTTTTT	8
hsa-miR-1264	303	8:1:0	-3.3	-14.18	10.88	CAAGUCUUAUUUGAGCACCUGUU	AATACTTAT	8

hsa-miR-331-5p	211	8:1:1	-3.8	-14.8	11	CUAGGUAUGGUCCCAGGGAUCC	ATACATATTAAATATTA	8
hsa-miR-203	214	8:1:1	-3.8	-14.83	11.03	AGUGGUUCUUAACAGUUCAACAGUU	CATATTAAATATTA	8
hsa-miR-655	200	8:1:0	-5.2	-16.27	11.07	AGAGGUUAUCCGUGUUAUGUUC	AGTATTATTATAT	8
hsa-miR-548n	168	8:1:1	-1.1	-12.17	11.07	CAAAAGUAAUUGUGGAUUUUGU	TTTTTTTTA	8
hsa-miR-633	272	8:1:1	-0.6	-11.7	11.1	CUAAUAGUAUCUACCACAAUAAA	ATAATTACTATA	8
hsa-miR-429	256	8:1:1	-1.3	-12.53	11.23	UAAUACUGUCUGGUAAAACCGU	TAATATTACAACT	8
hsa-miR-380	206	8:0:1	-4.7	-15.98	11.28	UGGUUGACCAUAGAACAUGCGC	ATTATATA	8
hsa-miR-641	163	8:1:1	-0.4	-11.69	11.29	AAAGACAUAGGAUAGAGUCACCUC	ATATTTTTTTTTTA	8
hsa-miR-633	257	8:1:0	-1.2	-12.5	11.3	CUAAUAGUAUCUACCACAAUAAA	AATATTACAACTAT	8
hsa-miR-548n	117	8:1:1	-1.31	-12.64	11.33	CAAAAGUAAUUGUGGAUUUUGU	TTTTTTTGTT	8
hsa-miR-548n	167	8:1:1	-0.5	-11.86	11.36	CAAAAGUAAUUGUGGAUUUUGU	TTTTTTTTTAA	8
hsa-miR-380	156	8:1:1	-1.7	-13.11	11.41	UGGUUGACCAUAGAACAUGCGC	AATATATATATTT	8
hsa-miR-374a	205	8:1:0	-4.6	-16.02	11.42	UUAUAAUACAACCUGAUAAGUG	TATTATATACATATAA	8
hsa-miR-448	209	8:1:1	-3.5	-14.94	11.44	UUGCAUAUGUAGGAUGUCCCAU	ATATACATATTA	8
hsa-miR-499-5p	200	8:1:0	-4.8	-16.27	11.47	UUAAGACUUGCAGUGAUGUUU	AGTATTATTA	8
hsa-miR-548n	115	8:1:1	-1.33	-12.8	11.47	CAAAAGUAAUUGUGGAUUUUGU	TTTTTTTTTGTTAGAAT	8
hsa-miR-1259	293	8:1:1	-2.7	-14.2	11.5	UCCCUGAGACCCUUUAACCUGUGA	ATATATATA	8

hsa-miR-137	200	8:1:1	-4.7	-16.27	11.57	UUAUUGCUUAAGAAUACGCGUAG	AGTATTATTAT		8
hsa-miR-653	173	8:1:1	-1.6	-13.17	11.57	GUGUUGAAACAAUCUCUACUG	TTTAAAAATATATATA		8
hsa-miR-633	153	8:1:1	-2.72	-14.31	11.59	CUAAUAGUAUCUACCACAAUAAA	ATTAATATATATAT		8
hsa-miR-494	165	8:1:1	0.2	-11.44	11.64	AGGUUGUCCGUGUUGUCUUCUCU	ATTTTTTTT		8
hsa-miR-548p	167	8:1:1	-0.21	-11.86	11.65	UAGCAAAAACUGCAGUUACUUU	TTTTTTTTTAAAAA		8
hsa-miR-548n	169	8:1:1	-0.8	-12.49	11.69	CAAAAGUAAUUGUGGAUUUUGU	TTTTTTTAAAAATATA		8
hsa-miR-561	163	8:1:1	0	-11.69	11.69	AUCAAGGAUCUUAAACUUUGCC	ATATTTTTTTTTAA		8
hsa-miR-559	115	8:1:1	-1.1	-12.8	11.7	UAAAGUAAAUAUGCACCAAAA	TTTTTTTTTGTTAGAAT		8
hsa-miR-101	201	8:1:1	-4.5	-16.22	11.72	CAGUUAUCACAGUGCUGAUGCU	GTATTATTATATAC		8
hsa-miR-155	257	8:1:1	-0.7	-12.5	11.8	UUAAUGCUAAUCGUGAUAGGGGU	AATATTACAA		8
hsa-miR-655	249	8:1:0	-1.9	-13.7	11.8	AGAGGUUAUCCGUGUUAUGUUC	TATATTATAATATTA		8
hsa-miR-374b	205	8:1:0	-4.2	-16.02	11.82	AUAUAAUACAACCUGCUAAGUG	TATTATATACATAT		8
hsa-miR-494	163	8:1:1	0.2	-11.69	11.89	AGGUUGUCCGUGUUGUCUUCUCU	ATATTTTTT		8
hsa-miR-380	278	8:1:1	-1.5	-13.42	11.92	UGGUUGACCAUAGAACAUGCGC	ACTATATATATAT		8
hsa-miR-548n	116	8:1:1	-0.77	-12.69	11.92	CAAAAGUAAUUGUGGAUUUUGU	TTTTTTTTGTTAGAAT		8
hsa-miR-548i	166	8:1:1	0.4	-11.53	11.93	AAAAGUAAUUGCGGAUUUUGCC	TTTTTTTTTTAAA		8
hsa-miR-656	250	8:1:0	-1.6	-13.66	12.06	AGGUUGCCUGUGAGGUGUUCA	ATATTATAATATTAC		8

hsa-miR-651	252	8:1:1	-1	-13.17	12.17	UUUAGGAUAAGCUUGACUUUUG	ATTATAATAT	8
hsa-miR-548h	204	8:1:1	-3.8	-16.03	12.23	AAAAGUAAUCGCGGUUUUUGUC	TTATTATATA	8
hsa-miR-380	255	8:1:1	-0.2	-12.53	12.33	UGGUUGACCAUAGAACAUGCGC	ATAATATTACA	8
hsa-miR-548c-5p	204	8:1:1	-3.7	-16.03	12.33	AAAAGUAAUUGCGGUUUUUGCC	TTATTATATACA	8
hsa-miR-142-5p	250	8:1:1	-1.3	-13.66	12.36	CAUAAAGUAGAAAGCACUACU	ATATTATAATATTA	8
hsa-miR-548p	168	8:1:1	0.35	-12.17	12.52	UAGCAAAAACUGCAGUUACUUU	TTTTTTTTAAAAATA	8
hsa-miR-577	115	8:1:1	-0.2	-12.8	12.6	UAGAUAAAAUAUUGGUACCUG	TTTTTTTTTG	8
hsa-miR-223	192	8:1:1	-3	-15.7	12.7	CGUGUAUUUGACAAGCUGAGUU	AACTAATAAGTATTATT	8
hsa-miR-548i	204	8:1:1	-3.3	-16.03	12.73	AAAAGUAAUUGCGGAUUUUGCC	TTATTATATACATATTA	8
hsa-miR-548a-5p	166	8:1:1	1.2	-11.53	12.73	AAAAGUAAUUGCGAGUUUUACC	TTTTTTTTT	8
hsa-miR-559	169	8:1:1	0.3	-12.49	12.79	UAAAGUAAAUAUGCACCAAAA	TTTTTTTAAAA	8
hsa-miR-548j	166	8:1:1	1.3	-11.53	12.83	AAAAGUAAUUGCGGUCUUUGGU	TTTTTTTTTTAAAAA	8
hsa-miR-577	117	8:1:1	0.2	-12.64	12.84	UAGAUAAAAUAUUGGUACCUG	TTTTTTTGTTAGAAT	8
hsa-miR-548p	169	8:1:1	0.4	-12.49	12.89	UAGCAAAAACUGCAGUUACUUU	TTTTTTTAA	8
hsa-miR-559	168	8:1:1	0.8	-12.17	12.97	UAAAGUAAAUAUGCACCAAAA	TTTTTTTTAAA	8
hsa-miR-200b	202	8:1:1	-3.2	-16.21	13.01	CAUCUUACUGGGCAGCAUUGGA	TATTATTATATACATAT	8
hsa-miR-548h	166	8:1:1	1.5	-11.53	13.03	AAAAGUAAUCGCGGUUUUUGUC	TTTTTTTTTTAAAAATA	8

hsa-miR-577	116	8:1:1	0.4	-12.69	13.09	UAGAUAAAAUAUUGGUACCUG	TTTTTTTTGTTAG	8
hsa-miR-200c	202	8:1:1	-2.9	-16.21	13.31	CGUCUUACCCAGCAGUGUUUGG	TATTATTATATACATA	8
hsa-miR-584	178	8:1:1	-0.3	-13.74	13.44	UUAUGGUUUGCCUGGGACUGAG	AAATATATA	8
hsa-miR-410	206	8:1:0	-2.4	-15.98	13.58	AGGUUGUCUGUGAUGAGUUCG	ATTATATACA	8
hsa-miR-375	194	8:1:1	-2.5	-16.13	13.63	UUUGUUCGUUCGGCUCGCGUGA	CTAATAAGTATTATT	8
hsa-miR-511	178	8:1:1	-0.1	-13.74	13.64	GUGUCUUUUGCUCUGCAGUCA	AAATATATATATA	8
hsa-miR-155	203	8:1:1	-2.4	-16.07	13.67	UUAAUGCUAAUCGUGAUAGGGGU	ATTATTATATACAT	8
hsa-miR-1246	292	8:1:1	0.2	-13.95	14.15	AAUGGAUUUUUGGAGCAGG	AATATATA	8
hsa-miR-944	196	8:1:1	-1.9	-16.07	14.17	AAAUUAUUGUACAUCGGAUGAG	AATAAGTATT	8
hsa-miR-500	199	8:1:1	-1.5	-15.87	14.37	UAAUCCUUGCUACCUGGGUGAGA	AAGTATTATTATATACA	8
hsa-miR-142-5p	206	8:1:1	-1.6	-15.98	14.38	CAUAAAGUAGAAAGCACUACU	ATTATATACATATT	8
hsa-miR-656	206	8:1:0	-0.7	-15.98	15.28	AGGUUGCCUGUGAGGUGUUCA	ATTATATACATATTA	8
hsa-miR-137	193	8:1:1	-0.8	-16.11	15.31	UUAUUGCUUAAGAAUACGCGUAG	ACTAATAAGTAT	8

Seq ID	length	Squence
5'UTR of var group C	230	AATATTCATATTCCCACATTGTCATATATTTATAATATATATAAATTAAAATATATATATATATTCTTCAAATATCTACATAAATATATATGTATAACATGCATGTATTATTTTATACAACACATCGATTACATTTT*AGCGTTTTTTTCCATCACATAT*AATACGACTAAGAAACAAAATAACATCACAACAAACATAGTGACTACCATTACATGATATTACCACATAAT

Note: the target sites are shown in italic

Target Prediction:

microRNA	Position in UTR	Seed	dGduplex	dGopen	ddG	Seq	Target site	Num of Matches
hsa-miR-451	137	8:1:1	-3.8	-9.52	5.72	AAACCGUUACCAUUACUGAGUU	AGCGTTTTTTTCCATCACATAT	8
hsa-let-7b	218	8:1:1	-16.6	-4.99	-11.6	UGAGGUAGUAGGUUGUGUGGUU	TTACCACATAAT	8

hsa-let-7c	218	8:1:1	-16.6	-4.99	-11.6	UGAGGUAGUAGGUUGUAUGGUU	TTACCACA	8
hsa-miR-196a	201	8:1:1	-19	-7.61	-11.38	UAGGUAGUUUCAUGUUGUUGGG	ACTACCATTACA	8
hsa-miR-299-3p	152	8:1:1	-16.8	-6.21	-10.58	UAUGUGGGAUGGUAAACCGCUU	CACATATAAT	8
hsa-miR-196b	201	8:1:1	-17.3	-7.61	-9.68	UAGGUAGUUUCCUGUUGUUGGG	ACTACCATTACA	8
hsa-let-7b	202	8:1:1	-16.5	-7.32	-9.17	UGAGGUAGUAGGUUGUGUGGUU	CTACCATTACA	8
hsa-miR-1270	217	8:1:1	-14	-5.06	-8.93	CUGGAGAUAUGGAAGAGCUGUGU	ATTACCACATAA	8
hsa-let-7a	218	8:1:1	-13.9	-4.99	-8.9	UGAGGUAGUAGGUUGUAUAGUU	TTACCACATA	8
hsa-let-7f	218	8:1:1	-13.9	-4.99	-8.9	UGAGGUAGUAGAUUGUAUAGUU	TTACCACATAA	8
hsa-miR-1294	181	8:1:0	-12.4	-3.56	-8.83	UGUGAGGUUGGCAUUGUUGUCU	ACATCACAACAAA	8
hsa-miR-1265	15	8:1:1	-10.6	-2.11	-8.48	CAGGAUGUGGUCAAGUGUUGUU	ACATTGTCATATAT	8
hsa-miR-299-3p	220	8:1:0	-13.26	-4.99	-8.26	UAUGUGGGAUGGUAAACCGCUU	ACCACATAAT	8
hsa-miR-182	18	8:1:1	-11.2	-3.01	-8.19	UUUGGCAAUGGUAGAACUCACACU	TTGTCATATATTTAT	8
hsa-let-7i	218	8:1:1	-12.9	-4.99	-7.9	UGAGGUAGUAGUUUGUGCUGUU	TTACCACATAAT	8
hsa-miR-98	218	8:1:1	-12.6	-4.99	-7.6	UGAGGUAGUAAGUUGUAUUGUU	TTACCACATAAT	8
hsa-miR-1293	201	8:1:1	-15.2	-7.61	-7.58	UGGGUGGUCUGGAGAUUUGUGC	ACTACCATTAC	8
hsa-let-7c	202	8:1:1	-14.6	-7.32	-7.27	UGAGGUAGUAGGUUGUAUGGUU	CTACCATTACATG	8
hsa-miR-382	178	8:1:1	-12.4	-5.2	-7.19	GAAGUUGUUCGUGGUGGAUUCG	ATAACATCACAACA	8

hsa-let-7g	218	8:1:1	-12.1	-4.99	-7.1	UGAGGUAGUAGUUUGUACAGUU	TTACCACATA	8
hsa-let-7d	218	8:1:1	-11.9	-4.99	-6.9	AGAGGUAGUAGGUUGCAUAGUU	TTACCACATAAT	8
hsa-miR-98	202	8:1:1	-14.2	-7.32	-6.87	UGAGGUAGUAAGUUGUAUUGUU	CTACCATTACATGAT	8
hsa-let-7i	202	8:1:1	-14	-7.32	-6.67	UGAGGUAGUAGUUUGUGCUGUU	CTACCATTACATGATAT	8
hsa-miR-380	207	8:1:0	-11.9	-5.41	-6.48	UGGUUGACCAUAGAACAUGCGC	ATTACATGATAT	8
hsa-miR-183	18	8:1:1	-9.3	-3.01	-6.29	UAUGGCACUGGUAGAAUUCACU	TTGTCATATATTTA	8
hsa-let-7g	202	8:1:1	-13.4	-7.32	-6.07	UGAGGUAGUAGUUUGUACAGUU	CTACCATTACATGATAT	8
hsa-miR-592	19	8:1:1	-9.3	-3.26	-6.03	UUGUGUCAAUAUGCGAUGAUGU	TGTCATATATTTATAAT	8
hsa-miR-1277	74	8:1:0	-14.11	-8.33	-5.77	AAAUAUAUAUAUAUAUGUACGUAU	TCTACATAAAT	8
hsa-miR-147	221	8:1:1	-10.7	-4.99	-5.7	GUGUGUGGAAAUGCUUCUGC	CCACATAAT	8
hsa-miR-224	198	8:1:0	-13.1	-7.47	-5.62	CAAGUCACUAGUGGUUCCGUU	GTGACTACCA	8
hsa-miR-323-5p	200	8:1:1	-13.4	-7.83	-5.56	AGGUGGUCCGUGGCGCGUUCGC	GACTACCATTA	8
hsa-let-7a	202	8:1:1	-12.6	-7.32	-5.27	UGAGGUAGUAGGUUGUAUAGUU	CTACCATTACAT	8
hsa-miR-1206	78	8:1:1	-14.4	-9.12	-5.27	UGUUCAUGUAGAUGUUUAAGC	CATAAATATA	8
hsa-miR-580	17	8:1:0	-7.71	-2.55	-5.15	UAAUGAUUCAUCAGACUCAGAU	ATTGTCATATATTTATA	8
hsa-miR-297	96	8:1:1	-16.3	-11.46	-4.83	AUGUAUGUGUGCAUGUGCAUG	ACATGCATGTATTA	8
hsa-miR-1259	21	8:0:1	-8.4	-3.78	-4.61	UCCCUGAGACCCUUUAACCUGUGA	TCATATATTTATA	8

hsa-miR-1270	73	8:1:0	-13.1	-8.53	-4.56	CUGGAGAUAUGGAAGAGCUGUGU	ATCTACATAAAT	8
hsa-miR-585	161	8:1:1	-9.9	-5.64	-4.25	CUAGCACACAGAUACGCCCAGA	TACGACTAAGAAA	8
hsa-miR-630	157	8:1:1	-9.9	-5.7	-4.19	AGUAUUCUGUACCAGGGAAGGU	ATAATACGACTAAGA	8
hsa-let-7f	202	8:1:1	-11.5	-7.32	-4.17	UGAGGUAGUAGAUUGUAUAGUU	CTACCATTACA	8
hsa-miR-541	200	8:1:1	-12	-7.83	-4.16	AAAGGAUUCUGCUGUCGGUCCCAC[illegible]	GACTACCATTA	8
hsa-miR-620	144	8:1:1	-10.4	-6.28	-4.11	AUGGAGAUAGAUAUAGAAAU	TTTTCCATCACAT	8
hsa-let-7d	202	8:1:1	-11.39	-7.32	-4.06	AGAGGUAGUAGGUUGCAUAGUU	CTACCATTA	8
hsa-miR-142-3p	205	8:1:1	-8.5	-4.47	-4.02	UGUAGUGUUUCCUACUUUAUGGA	CCATTACAT	8
hsa-miR-190b	34	8:1:1	-10.4	-6.49	-3.9	UGAUAUGUUUGAUAUUGGGUU	ATATATATA	8
hsa-miR-892a	193	8:1:1	-10.7	-7.22	-3.47	CACUGUGUCCUUUCUGCGUAG	ACATAGTGACTAC	8
hsa-miR-27a	17	8:1:1	-6	-2.55	-3.44	AGGGCUUAGCUGCUUGUGAGCA	ATTGTCATATATTTATA	8
hsa-miR-27b	17	8:1:1	-6	-2.55	-3.44	AGAGCUUAGCUGAUUGGUGAAC	ATTGTCATATATTTAT	8
hsa-miR-297	153	8:0:1	-9.71	-6.27	-3.43	AUGUAUGUGUGCAUGUGCAUG	ACATATAATACGA	8
hsa-miR-518c	138	8:0:1	-12.01	-8.62	-3.38	UCUCUGGAGGGAAGCACUUUCUG	GCGTTTTTTTCCATCA	8
hsa-miR-208b	19	8:1:1	-6.4	-3.26	-3.13	AAGCUUUUUGCUCGAAUUAUGU	TGTCATATATTTAT	8
hsa-miR-576-3p	152	8:1:1	-9.1	-6.21	-2.88	AAGAUGUGGAAAAAUUGGAAUC	CACATATAATACGACTA	8
hsa-miR-548b-5p	128	8:1:0	-10.4	-7.61	-2.78	AAAAGUAAUUGUGGUUUUGGCC	TTACATTTTAGC	8

hsa-miR-548h	128	8:1:0	-10.4	-7.61	-2.78	AAAAGUAAUCGCGGUUUUUGUC	TTACATTTTA	8
hsa-miR-26a	209	8:1:0	-8.3	-5.57	-2.72	UUCAAGUAAUCCAGGAUAGGCU	TACATGATATTACC	8
hsa-miR-26b	209	8:1:0	-8.3	-5.57	-2.72	UUCAAGUAAUUCAGGAUAGGU	TACATGATA	8
hsa-miR-1270	144	8:1:1	-9	-6.28	-2.71	CUGGAGAUAUGGAAGAGCUGUGU	TTTTCCATCACATATAA	8
hsa-miR-590-3p	213	8:1:1	-7.9	-5.3	-2.59	UAAUUUUAUGUAUAAGCUAGU	TGATATTACCA	8
hsa-miR-208a	19	8:1:1	-5.7	-3.26	-2.43	GAGCUUUUGGCCCGGGUUAUAC	TGTCATATATTTATA	8
hsa-miR-155	137	8:1:1	-11.9	-9.52	-2.37	UUAAUGCUAAUCGUGAUAGGGGU	AGCGTTTTTTCCAT	8
hsa-miR-142-3p	181	8:1:1	-5.92	-3.56	-2.35	UGUAGUGUUUCCUACUUUAUGGA	ACATCACA	8
hsa-miR-1252	146	8:1:0	-8.3	-6.25	-2.04	AGAAGGAAAUUGAAUUCAUUUA	TTCCATCAC	8
hsa-miR-142-3p	160	8:1:1	-7.59	-5.65	-1.93	UGUAGUGUUUCCUACUUUAUGGA	ATACGACTAAGAA	8
hsa-miR-1297	209	8:1:0	-7.5	-5.57	-1.92	UUCAAGUAAUUCAGGUG	TACATGATATTACC	8
hsa-miR-625	146	8:1:1	-8	-6.25	-1.74	AGGGGGAAAGUUCUAUAGUCC	TTCCATCACAT	8
hsa-miR-190	193	8:1:1	-8.7	-7.22	-1.47	UGAUAUGUUUGAUAUAUUAGGU	ACATAGTGAC	8
hsa-miR-190b	193	8:1:1	-8.7	-7.22	-1.47	UGAUAUGUUUGAUAUUGGGUU	ACATAGTGACTACC	8
hsa-miR-548d-5p	128	8:1:0	-9	-7.61	-1.38	AAAAGUAAUUGUGGUUUUUGCC	TTACATTTTAGC	8
hsa-miR-568	112	8:0:0	-11.3	-9.98	-1.31	AUGUAUAAAUGUAUACACAC	TTATACAACACATCGA	8
hsa-miR-548m	24	8:1:1	-6.2	-4.89	-1.3	CAAAGGUAUUUGUGGUUUUUG	TATATTTATAATATA	8

hsa-miR-433	195	8:1:1	-8.69	-7.42	-1.26	UACGGUGAGCCUGUCAUUAUUC	ATAGTGACTACCA	8
hsa-miR-548n	129	8:1:0	-8.8	-7.53	-1.26	CAAAAGUAAUUGUGGAUUUUGU	TACATTTTAGCGTT	8
hsa-miR-190	34	8:1:1	-7.7	-6.49	-1.2	UGAUAUGUUUGAUAUAUUAGGU	ATATATATAAATT	8
hsa-miR-610	135	8:1:1	-11.3	-10.16	-1.13	UGAGCUAAAUGUGUGCUGGGA	TTAGCGTTTT	8
hsa-miR-410	20	8:1:0	-4.7	-3.6	-1.09	AGGUUGUCUGUGAUGAGUUCG	GTCATATATTTA	8
hsa-miR-155	204	8:1:0	-5.6	-4.51	-1.08	UUAAUGCUAAUCGUGAUAGGGGU	ACCATTACAT	8
hsa-miR-889	214	8:0:1	-6.2	-5.14	-1.05	AAUGGCUGUCCGUAGUAUGGUC	GATATTACCACATAAT	8
hsa-miR-30e	26	8:1:1	-6.7	-5.85	-0.84	UGUAAACAUCCUUGACUGGAAG	TATTTATAATATA	8
hsa-miR-518a-3p	138	8:0:1	-9.34	-8.62	-0.71	GAAAGCGCUUCCCUUUGCUGGA	GCGTTTTTTTCCATCAC	8
hsa-miR-548a-5p	128	8:1:0	-8.15	-7.61	-0.53	AAAAGUAAUUGCGAGUUUUACC	TTACATTT	8
hsa-miR-548j	128	8:1:0	-8.1	-7.61	-0.48	AAAAGUAAUUGCGGUCUUUGGU	TTACATTTTAGC	8
hsa-miR-548a-5p	107	8:0:1	-11	-10.51	-0.48	AAAAGUAAUUGCGAGUUUUACC	TTATTTTATACAACA	8
hsa-miR-375	163	8:1:1	-6.09	-5.63	-0.45	UUUGUUCGUUCGGCUCGCGUGA	CGACTAAGAAAC	8
hsa-miR-518d-3p	138	8:0:1	-9	-8.62	-0.37	CAAAGCGCUUCCCUUUGGAGC	GCGTTTTTTT	8
hsa-miR-508-3p	203	8:1:1	-7.1	-6.79	-0.3	UGAUUGUAGCCUUUUGGAGUAGA	TACCATTACAT	8
hsa-miR-559	107	8:1:1	-10.8	-10.51	-0.28	UAAAGUAAAUAUGCACCAAAA	TTATTTTATACAACAC	8
hsa-miR-1261	144	8:1:1	-6.5	-6.28	-0.21	AUGGAUAAGGCUUUGGCUU	TTTTCCATC	8

hsa-miR-518e	137	8:1:1	-9.61	-9.52	-0.08	CUCUAGAGGGAAGCGCUUUCUG	AGCGTTTT	8
hsa-miR-1259	151	8:1:0	-6.24	-6.21	-0.029	UCCCUGAGACCCUUUAACCUGUGA	TCACATATAATACG	8
hsa-miR-1277	86	8:1:1	-10.9	-10.87	-0.023	AAAUAUAUAUAUAUAUGUACGUAU	TATATGTAT	8
hsa-miR-105	138	8:1:1	-8.6	-8.62	0.027	UCAAAUGCUCAGACUCCUGUGGU	GCGTTTTTTTC	8
hsa-miR-518f	138	8:0:1	-8.5	-8.62	0.12	CUCUAGAGGGAAGCACUUUCUC	GCGTTTTTT	8
hsa-miR-190b	50	8:1:1	-9.31	-9.51	0.2	UGAUAUGUUUGAUAUUGGGUU	ATATATATATA	8
hsa-miR-518b	138	8:0:1	-8.4	-8.62	0.22	CAAAGCGCUCCCCUUUAGAGGU	GCGTTTTTTCC	8
hsa-miR-620	217	8:1:1	-4.8	-5.06	0.26	AUGGAGAUAGAUAUAGAAAU	ATTACCACA	8
hsa-miR-369-3p	104	8:0:0	-11	-11.26	0.26	AAUAAUACAUGGUUGAUCUUU	GTATTATTTTAT	8
hsa-miR-643	155	8:1:1	-5.73	-6.07	0.34	ACUUGUAUGCUAGCUCAGGUAG	ATATAATA	8
hsa-miR-1178	198	8:1:1	-7.1	-7.47	0.37	CAGGGUCAGCUGAGCAUG	GTGACTACC	8
hsa-miR-656	212	8:1:1	-5.1	-5.48	0.38	AGGUUGCCUGUGAGGUGUUCA	ATGATATT	8
hsa-miR-548c-5p	128	8:1:0	-7.2	-7.61	0.41	AAAAGUAAUUGCGGUUUUUGCC	TTACATTTTAGCG	8
hsa-miR-27a	195	8:1:1	-7	-7.42	0.42	AGGGCUUAGCUGCUUGUGAGCA	ATAGTGACTA	8
hsa-miR-27b	195	8:1:1	-7	-7.42	0.42	AGAGCUUAGCUGAUUGGUGAAC	ATAGTGACT	8
hsa-miR-620	73	8:1:0	-8.1	-8.53	0.43	AUGGAGAUAGAUAUAGAAAU	ATCTACATAAATATAT	8
hsa-miR-592	112	8:1:1	-9.5	-9.98	0.48	UUGUGUCAAUAUGCGAUGAUGU	TTATACAACACATC	8

hsa-miR-511	169	8:1:1	-5	-5.6	0.6	GUGUCUUUUGCUCUGCAGUCA	AGAAACAAAATAA	8
hsa-miR-34b	197	8:1:0	-6.9	-7.69	0.79	UAGGCAGUGUCAUUAGCUGAUUG	AGTGACTACCA	8
hsa-miR-633	204	8:1:0	-3.6	-4.51	0.91	CUAAUAGUAUCUACCACAAUAAA	ACCATTACATGATATT	8
hsa-miR-489	214	8:1:1	-4.2	-5.14	0.94	GGUCGUAUGUGUGACGCCAUUU	GATATTACCACATAAT	8
hsa-miR-548i	128	8:1:0	-6.67	-7.61	0.94	AAAAGUAAUUGCGGAUUUUGCC	TTACATTTTA	8
hsa-miR-578	186	8:1:0	-4.64	-5.68	1.04	CUUCUUGUGCUCUAGGAUUGU	ACAACAAA	8
hsa-miR-655	103	8:0:0	-10.2	-11.31	1.11	AGAGGUUAUCCGUGUUAUGUUC	TGTATTATTTTATACA	8
hsa-miR-553	137	8:1:1	-8.4	-9.52	1.12	AAAACGGUGAGAUUUUGUUUU	AGCGTTTTTTT	8
hsa-miR-190	50	8:1:1	-8.3	-9.51	1.21	UGAUAUGUUUGAUAUAUUAGGU	ATATATATATATAT	8
hsa-miR-548n	108	8:1:1	-9.16	-10.43	1.27	CAAAAGUAAUUGUGGAUUUUGU	TATTTTATACA	8
hsa-miR-181d	99	8:1:0	-10.1	-11.37	1.27	AACAUUCAUUGUUGUCGGUGGGU	TGCATGTATT	8
hsa-miR-130b	128	8:1:1	-6.3	-7.61	1.31	ACUCUUUCCCUGUUGCACUAC	TTACATTT	8
hsa-miR-548c-5p	107	8:0:1	-9.2	-10.51	1.31	AAAAGUAAUUGCGGUUUUUGCC	TTATTTTATACA	8
hsa-miR-553	123	8:1:1	-6.5	-7.82	1.32	AAAACGGUGAGAUUUUGUUUU	ATCGATTAC	8
hsa-miR-30a	26	8:1:1	-4.3	-5.85	1.55	UGUAAACAUCCUCGACUGGAAG	TATTTATAAT	8
hsa-miR-30d	26	8:1:1	-4.3	-5.85	1.55	UGUAAACAUCCCCGACUGGAAG	TATTTATAATATATATA	8
hsa-miR-656	31	8:0:0	-4.7	-6.38	1.68	AGGUUGCCUGUGAGGUGUUCA	ATAATATA	8

hsa-miR-1290	70	8:1:1	-7.3	-9.02	1.72	UGGAUUUUUGGAUCAGGGA	AATATCTAC	8
hsa-miR-190	119	8:1:0	-7	-8.76	1.76	UGAUAUGUUUGAUAUAUUAGGU	ACACATCGA	8
hsa-miR-570	142	8:1:1	-5.5	-7.35	1.85	AAAGGUAAUUGCAGUUUUUCCC	TTTTTTCCAT	8
hsa-miR-548i	107	8:0:1	-8.6	-10.51	1.91	AAAAGUAAUUGCGGAUUUUGCC	TTATTTTATA	8
hsa-miR-331-5p	71	8:1:1	-6.9	-9.08	2.18	CUAGGUAUGGUCCCAGGGAUCC	ATATCTACATAAAT	8
hsa-miR-656	157	8:1:0	-3.5	-5.7	2.2	AGGUUGCCUGUGAGGUGUUCA	ATAATACGACTAAGA	8
hsa-miR-1224-5p	63	8:1:1	-6.2	-8.4	2.2	GUGAGGACUCGGGAGGUGG	TTCTTCAAA	8
hsa-miR-380	127	8:1:0	-6.1	-8.33	2.23	UGGUUGACCAUAGAACAUGCGC	ATTACATTT	8
hsa-miR-523	138	8:1:1	-6.21	-8.62	2.41	CUCUAGAGGGAAGCGCUUUCUG	GCGTTTTTTCCATCAC	8
hsa-miR-1252	62	8:1:1	-6.6	-9.06	2.46	AGAAGGAAAUUGAAUUCAUUUA	ATTCTTCAAATA	8
hsa-miR-491-3p	209	8:1:1	-3.07	-5.57	2.5	CUUAUGCAAGAUUCCCUUCUAC	TACATGATATT	8
hsa-miR-568	56	8:1:1	-7.1	-9.63	2.53	AUGUAUAAAUGUAUACACAC	ATATATATTCTTCAA	8
hsa-miR-190b	119	8:1:0	-6.2	-8.76	2.56	UGAUAUGUUUGAUAUUGGGUU	ACACATCGATTA	8
hsa-miR-548g	130	8:1:0	-7.3	-9.98	2.68	UGCAAAAGUAAUUGCAGUUUUUG	ACATTTTAGCGTTT	8
hsa-miR-768-5p	65	8:1:1	-5.4	-8.11	2.71	UGCACCAUGGUUGUCUGAGCAUG	CTTCAAATATCTAC	8
hsa-miR-656	20	8:1:1	-0.8	-3.6	2.8	AGGUUGCCUGUGAGGUGUUCA	GTCATATATTTATAA	8
hsa-miR-218	191	8:1:1	-4.4	-7.27	2.87	UUGUGCUUGAUCUAACCAUGU	AAACATAG	8

hsa-miR-380	33	8:1:1	-3.6	-6.52	2.92	UGGUUGACCAUAGAACAUGCGC	AATATATATAAATTAAA	8
hsa-miR-1206	97	8:1:1	-8.3	-11.23	2.93	UGUUCAUGUAGAUGUUUAAGC	CATGCATGTAT	8
hsa-miR-548b-5p	107	8:0:1	-7.56	-10.51	2.95	AAAAGUAAUUGUGGUUUUGGCC	TTATTTTATACAAC	8
hsa-miR-568	36	8:1:1	-3.5	-6.46	2.96	AUGUAUAAAUGUAUACACAC	ATATATAAA	8
hsa-miR-374b	105	8:1:0	-8.2	-11.17	2.97	AUAUAAUACAACCUGCUAAGUG	TATTATTTTA	8
hsa-miR-1308	98	8:1:1	-8.3	-11.35	3.05	UUCACAUUGUGCUACUGUCUGC	ATGCATGTATTATT	8
hsa-miR-517b	98	8:1:1	-8	-11.35	3.35	CCUCUAGAUGGAAGCACUGUCU	ATGCATGTATTATTTT	8
hsa-miR-643	114	8:1:1	-6.6	-9.96	3.36	ACUUGUAUGCUAGCUCAGGUAG	ATACAACACATCGATTA	8
hsa-miR-517a	99	8:1:1	-8	-11.37	3.37	CCUCUAGAUGGAAGCACUGUCU	TGCATGTA	8
hsa-miR-517c	99	8:1:1	-8	-11.37	3.37	CCUCUAGAUGGAAGCACUGUCU	TGCATGTATT	8
hsa-miR-1259	32	8:1:0	-3	-6.52	3.52	UCCCUGAGACCCUUUAACCUGUGA	TAATATATAT	8
hsa-miR-548h	107	8:0:1	-6.9	-10.51	3.61	AAAAGUAAUCGCGGUUUUUGUC	TTATTTTAT	8
hsa-miR-643	98	8:1:1	-7.7	-11.35	3.65	ACUUGUAUGCUAGCUCAGGUAG	ATGCATGTATT	8
hsa-miR-548j	107	8:0:1	-6.8	-10.51	3.71	AAAAGUAAUUGCGGUCUUUGGU	TTATTTTAT	8
hsa-miR-548k	107	8:1:1	-6.8	-10.51	3.71	AAAAGUACUUGCGGAUUUUGCU	TTATTTTATACAACACA	8
hsa-miR-548d-5p	107	8:0:1	-6.76	-10.51	3.75	AAAAGUAAUUGUGGUUUUUGCC	TTATTTTATACAA	8
hsa-miR-1259	34	8:1:1	-2.7	-6.49	3.79	UCCCUGAGACCCUUUAACCUGUGA	ATATATATAAATTAAAA	8

hsa-miR-203	131	8:0:1	-6.4	-10.21	3.81	AGUGGUUCUUAACAGUUCAACAGUU	CATTTTAGCGT	8
hsa-miR-633	214	8:1:1	-1.2	-5.14	3.94	CUAAUAGUAUCUACCACAAUAAA	GATATTACCACATAA	8
hsa-miR-142-5p	109	8:0:1	-6.3	-10.25	3.95	CAUAAAGUAGAAAGCACUACU	ATTTTATACA	8
hsa-miR-548l	107	8:1:1	-6.5	-10.51	4.01	AAAAGUAUUUGCGGGUUUUGUC	TTATTTTATACAAC	8
hsa-miR-190b	83	8:1:1	-7.2	-11.24	4.04	UGAUAUGUUUGAUAUUGGGUU	ATATATATGTATAACA	8
hsa-miR-301b	128	8:1:1	-3.5	-7.61	4.11	GCUCUGACGAGGUUGCACUACU	TTACATTT	8
hsa-miR-297	77	8:1:1	-5	-9.12	4.12	AUGUAUGUGUGCAUGUGCAUG	ACATAAATA	8
hsa-miR-567	95	8:1:1	-7.4	-11.57	4.17	AGUAUGUUCUUCCAGGACAGAAC	AACATGCA	8
hsa-miR-655	90	8:1:0	-5.8	-10	4.2	AGAGGUUAUCCGUGUUAUGUUC	TGTATAACAT	8
hsa-miR-301a	128	8:1:1	-3.4	-7.61	4.21	GCUCUGACUUUAUUGCACUACU	TTACATTTTAGCGT	8
hsa-miR-656	93	8:1:0	-5.7	-9.94	4.24	AGGUUGCCUGUGAGGUGUUCA	ATAACATGCATGTA	8
hsa-miR-202	62	8:1:1	-4.8	-9.06	4.26	UUCCUAUGCAUAUACUUCUUUG	ATTCTTCA	8
hsa-miR-519c-3p	99	8:1:1	-7.05	-11.37	4.32	AAAGUGCAUCUUUUUAGAGGAU	TGCATGTATTATTTTA	8
hsa-miR-491-3p	90	8:1:1	-5.5	-10	4.5	CUUAUGCAAGAUUCCCUUCUAC	TGTATAACATGCATGTA	8
hsa-miR-144	60	8:1:1	-4.7	-9.22	4.52	GGAUAUCAUCAUAUACUGUAAG	ATATTCTT	8
hsa-miR-30c	26	8:1:1	-1.3	-5.85	4.55	UGUAAACAUCCUACACUCUCAGC	TATTTATAATATATA	8
hsa-miR-30b	26	8:1:1	-1.3	-5.85	4.55	UGUAAACAUCCUACACUCAGCU	TATTTATAATATA	8

hsa-miR-454	128	8:1:1	-2.9	-7.61	4.71	ACCCUAUCAAUAUUGUCUCUGC	TTACATTTT	8
hsa-miR-1259	110	8:1:1	-5.4	-10.15	4.75	UCCCUGAGACCCUUUAACCUGUGA	TTTTATACAACACA	8
hsa-miR-374a	105	8:1:0	-6.3	-11.17	4.87	UUAUAAUACAACCUGAUAAGUG	TATTATTTTA	8
hsa-miR-568	54	8:1:1	-4.7	-9.62	4.92	AUGUAUAAAUGUAUACACAC	ATATATATAT	8
hsa-miR-181b	99	8:1:0	-6.43	-11.37	4.94	AACAUUCAUUGCUGUCGGUGGGU	TGCATGTATTATTTTAT	8
hsa-miR-519b-3p	99	8:1:1	-6.4	-11.37	4.97	AAAGUGCAUCCUUUUAGAGGUU	TGCATGTATTATTTT	8
hsa-miR-519a	99	8:1:1	-6.4	-11.37	4.97	CUCUAGAGGGAAGCGCUUUCUG	TGCATGTATT	8
hsa-miR-190	83	8:1:1	-6.1	-11.24	5.14	UGAUAUGUUUGAUAUAUUAGGU	ATATATATGTATAA	8
hsa-miR-380	31	8:1:1	-0.9	-6.38	5.48	UGGUUGACCAUAGAACAUGCGC	ATAATATAT	8
hsa-miR-130a	128	8:1:1	-2.1	-7.61	5.51	UUCACAUUGUGCUACUGUCUGC	TTACATTTTA	8
hsa-miR-1297	108	8:1:1	-4.8	-10.43	5.63	UUCAAGUAAUUCAGGUG	TATTTTATAC	8
hsa-miR-380	93	8:1:0	-4.2	-9.94	5.74	UGGUUGACCAUAGAACAUGCGC	ATAACATGCAT	8
hsa-miR-196a	70	8:1:1	-3.2	-9.02	5.82	UAGGUAGUUUCAUGUUGUUGGG	AATATCTACATAAA	8
hsa-miR-196b	70	8:1:1	-3.2	-9.02	5.82	UAGGUAGUUUCCUGUUGUUGGG	AATATCTACATAA	8
hsa-miR-26a	108	8:1:1	-4.4	-10.43	6.03	UUCAAGUAAUCCAGGAUAGGCU	TATTTTATACAA	8
hsa-miR-139-5p	132	8:1:1	-4.37	-10.43	6.06	UCUACAGUGCACGUGUCUCCAGU	ATTTTAGCGTTTTTTT	8
hsa-miR-340	110	8:1:1	-4	-10.15	6.15	UUAUAAAGCAAUGAGACUGAUU	TTTTATACAACACATCG	8

hsa-miR-599	113	8:1:1	-3.5	-9.98	6.48	GUUGUGUCAGUUUAUCAAAC	TATACAACACA	8
hsa-miR-26b	108	8:1:1	-3.9	-10.43	6.53	UUCAAGUAAUUCAGGAUAGGU	TATTTTATACAACAC	8
hsa-miR-584	48	8:1:1	-2.61	-9.26	6.65	UUAUGGUUUGCCUGGGACUGAG	AAATATATATATAT	8
hsa-miR-568	52	8:1:1	-3	-9.74	6.74	AUGUAUAAAUGUAUACACAC	ATATATATATA	8
hsa-miR-1259	50	8:1:1	-2.7	-9.51	6.81	UCCCUGAGACCCUUUAACCUGUGA	ATATATATAT	8
hsa-miR-200b	105	8:1:1	-4.3	-11.17	6.87	CAUCUUACUGGGCAGCAUUGGA	TATTATTTTATA	8
hsa-miR-487b	104	8:1:1	-4.3	-11.26	6.96	GUGGUUAUCCCUGUCCUGUUCG	GTATTATTTTA	8
hsa-miR-200c	105	8:1:1	-4.1	-11.17	7.07	CGUCUUACCCAGCAGUGUUUGG	TATTATTTT	8
hsa-miR-487a	104	8:1:0	-4	-11.26	7.26	GUGGUUAUCCCUGCUGUGUUCG	GTATTATTTTATACAAC	8
hsa-miR-544	90	8:1:1	-2.7	-10	7.3	AUUCUGCAUUUUUAGCAAGUUC	TGTATAACATG	8
hsa-miR-1261	112	8:1:1	-2.62	-9.98	7.36	AUGGAUAAGGCUUUGGCUU	TTATACAACACATCGAT	8
hsa-miR-380	106	8:1:1	-3.5	-10.9	7.4	UGGUUGACCAUAGAACAUGCGC	ATTATTTTATAC	8
hsa-miR-633	106	8:1:1	-3.5	-10.9	7.4	CUAAUAGUAUCUACCACAAUAAA	ATTATTTTA	8
hsa-miR-576-5p	44	8:1:0	-1.2	-8.77	7.57	AUUCUAAUUUCUCCACGUCUUU	ATTAAAAT	8
hsa-miR-577	106	8:1:1	-3.2	-10.9	7.7	UAGAUAAAAUAUUGGUACCUG	ATTATTTTATA	8
hsa-miR-656	47	8:1:0	-1.3	-9.12	7.82	AGGUUGCCUGUGAGGUGUUCA	AAAATATA	8
hsa-miR-561	130	8:1:1	-2.1	-9.98	7.88	AUCAAGGAUCUUAAACUUUGCC	ACATTTTAGCG	8

hsa-miR-511	48	8:1:1	-0.8	-9.26	8.46	GUGUCUUUUGCUCUGCAGUCA	AAATATATATA	8
hsa-miR-1259	83	8:1:1	-2.7	-11.24	8.54	UCCCUGAGACCCUUUAACCUGUGA	ATATATATGTATA	8
hsa-miR-1246	82	8:1:1	-2.4	-11.25	8.85	AAUGGAUUUUUGGAGCAGG	AATATATATGTATA	8
hsa-miR-1246	49	8:1:1	-0.4	-9.27	8.87	AAUGGAUUUUUGGAGCAGG	AATATATA	8
hsa-miR-380	109	8:1:1	-0.3	-10.25	9.95	UGGUUGACCAUAGAACAUGCGC	ATTTTATACAAC	8
hsa-miR-656	104	8:1:1	-1	-11.26	10.26	AGGUUGCCUGUGAGGUGUUCA	GTATTATTTTATA	8

Seq ID	length	Squence
3'UTR of var group A	1291	CGGATGTATGGAATATATAAGAAAAAAAAAAATATATTATTTTACTTGTTTATTTTTTTTTTTTTTTTTTTTGTTATATTTTATTTGTATATTCATTTGTATAATATAAATATATATATTCATAGTATATATATATTTTTATTTTGATATATTTGTATAATAATATTTGTATATATATATATATAATTTGTTTTTTTGGCTTTTATATAATATTTGTAAATATATATATATATATATAATTTGTTTTTTTGTTTTTGTATAATATTTCTAAATATATTCGATTCATAATTTTTTGAGTAATATTTTTATTTTTTTTTCATTTTATTTTTGATTTTTTTTTTAAATGTCATTGTTCATAATTTTAATATATAATTTTTATTTTGTTTGTATAATATATATAAATTCGTTTTATTTTGTTTCTATTTGACACATGTGATTATTTGTATATATAAATTTGTTTTATTTGTATAATATATATATATGTTTTATTTTGTGTTTATTTGACACATTTGATTATTTGTATATATATTTTGTTTGAATTTTTTTATTAATTTGTTTTTGGATATTTGTATATATAAATTTGTTTTATTTGTATAATATGTATGTGATTAAATAGAAAAAAAAAGAAAAAAAAAATAGAAAAATATACCATACCATATAAATTATATTCATAGTAGTATATTATATGGTTCCATATTAAAATAAGCTAATATAATACACTAAAATATTTCTTATTATATATATATATATATATATTTAATATAGTAACAACGAAAATGTTTTCAACACCATATACGGATATATCAACACATTATAATATAATAATAATGAATTAATCTAATCTTACAATAAGTATTATTATAAAATAGTAACAATGTAATATATCATATATAATAATTGTATTGGAATAACAATATTTATATGATATCCTTTTTTTTTTTTTTTTTTTTTTTGTATTCATACATACTATCATCATCAATATAGGAAATAAATTCTTTTTAAGAAATATGAAGAATTTTTTTTTT*AACGCTTC*TAATTACGTATAAAATAAAAATAAATGATTCCTTGTTTTTCCATAGTATATATAATGTCGCGTTATTTGTTTATTGTATAATAATATATTTATTAAAATATGTAGTAATTAATTATTTTGTTACAACATGTAATAATAAAATTAATGTATCATAAGTTGTAAAATACCATAACTTCTATTTTAAGAAATATATATATAATAATACACGTTATGATTATAATATATTTGAATAACATAAAACAATAGAATGTATTATGTAATAGTA

Note: the target sites are shown in italic

Target Prediction:

microRNA	Position in UTR	Seed	dGduplex	dGopen	ddG	Seq	Target site	Num of Matches	Mutated seq	Mutation primers
hsa-miR-451	1017	8:1:0	-3.6	-11.27	7.67	AAACCGUUACCAUUACUGAGUU	AACGCTTC	8	TTATATTA	Up:AAGAAATATGAAGAATTTTTTTTTTATATTATAATTACGTAT;Down:TCATTTATTTTTATTTTTATACGTAATTATAATAT
hsa-miR-297	954	8:1:1	-15.1	-1.75	-13.34	AUGUAUGUGUGCAUGUGCAUG	TACATACTATCAT	8		
hsa-miR-1272	965	8:1:0	-14.52	-2.44	-12.07	GAUGAUGAUGGCAGCAAAUUCUGAAA	ATCATCAATATAGGAAA	8		

hsa-miR-574-5p	955	8:1:1	-13.8	-1.79	-12	UGAGUGUGUGUGUGUGAGUGUGU	ACATACTATCATCAT	8
hsa-miR-1272	962	8:0:0	-12.2	-1.64	-10.55	GAUGAUGAUGGCAGCAAAUUCUGAAA	ATCATCATCA	8
hsa-miR-574-5p	801	8:1:1	-17.3	-7.31	-9.98	UGAGUGUGUGUGUGUGAGUGUGU	ACACATTATAAT	8
hsa-miR-299-3p	648	8:1:1	-14.7	-5.32	-9.37	UAUGUGGGAUGGUAAACCGCUU	TACCATATAA	8
hsa-miR-1	270	8:1:1	-12.4	-4.8	-7.59	ACAUACUUCUUUAUAUGCCCAU	TATATTCGATTCATAA	8
hsa-miR-620	961	8:1:1	-9.3	-1.73	-7.56	AUGGAGAUAGAUAUAGAAAU	TATCATCATCAATATAGG	8
hsa-miR-103	1079	8:1:1	-17.9	-10.72	-7.17	UCAUAGCCCUGUACAAUGCUGCU	AATGTCGCGTTATTTG	8
hsa-miR-206	270	8:1:1	-11.9	-4.8	-7.09	UGGAAUGUAAGGAAGUGUGUGG	TATATTCGATTCATAAT	8
hsa-miR-508-3p	960	8:1:1	-8.64	-1.77	-6.86	UGAUUGUAGCCUUUUGGAGUAGA	CTATCATCATCAATATA	8
hsa-miR-539	722	8:1:1	-13.9	-7.05	-6.84	GGAGAAAUUAUCCUUGGUGUGU	TATTTCTTATTA	8
hsa-miR-206	660	8:1:1	-14.4	-7.77	-6.62	UGGAAUGUAAGGAAGUGUGUGG	TATATTCATAGTAGTATAT	8
hsa-miR-337-3p	972	8:0:0	-13.3	-7.33	-5.96	CUCCUAUAUGAUGCCUUUCUUC	ATATAGGAAATAAATTC	8
hsa-miR-433	964	8:1:0	-8.14	-2.26	-5.87	UACGGUGAGCCUGUCAUUAUUC	CATCATCAATATAGGAA	8
hsa-miR-433	961	8:1:1	-7.4	-1.73	-5.66	UACGGUGAGCCUGUCAUUAUUC	TATCATCATCAATATA	8
hsa-miR-107	1079	8:1:1	-16.36	-10.72	-5.63	AGCAGCAUUGUACAGGGCUAUCA	AATGTCGCGT	8
hsa-miR-608	644	8:1:1	-11.4	-5.82	-5.57	AGGGGUGGUGUUGGGACAGCUCCGU	ACCATACCATATAAATT	8
hsa-miR-221	868	8:1:1	-12.5	-7.2	-5.29	ACCUGGCAUACAAUGUAGAUUU	AATGTAATATATCAT	8

hsa-miR-206	954	8:1:1	-6.98	-1.75	-5.22	UGGAAUGUAAGGAAGUGUGUGG	TACATACTAT	8
hsa-miR-331-5p	952	8:1:0	-9.1	-4.02	-5.07	CUAGGUAUGGUCCCAGGGAUCC	CATACATACTATCA	8
hsa-miR-1265	918	8:1:1	-16.3	-11.29	-5	CAGGAUGUGGUCAAGUGUUGUU	GATATCCTTTTTT	8
hsa-miR-648	954	8:1:1	-6.6	-1.75	-4.84	AAGUGUGCAGGGCACUGGU	TACATACTAT	8
hsa-miR-888	288	8:0:0	-14.7	-9.96	-4.73	UACUCAAAAAGCUGUCAGUCA	TTTTGAGTAATATT	8
hsa-miR-190	800	8:1:1	-10.8	-6.33	-4.46	UGAUAUGUUUGAUAUAUUAGGU	AACACATTAT	8
hsa-miR-1322	962	8:1:0	-6.1	-1.64	-4.45	GAUGAUGCUGCUGAUGCUG	ATCATCATCAATA	8
hsa-miR-548h	433	8:1:1	-13	-8.63	-4.36	AAAAGUAAUCGCGGUUUUUGUC	ATTATTTGTATATATAAAT	8
hsa-miR-567	638	8:1:1	-7.4	-3.05	-4.34	AGUAUGUUCUUCCAGGACAGAAC	AAATATACCA	8
hsa-miR-539	983	8:1:1	-12.61	-8.33	-4.27	GGAGAAAUUAUCCUUGGUGUGU	AAATTCTTTTT	8
hsa-miR-1	954	8:1:1	-5.95	-1.75	-4.19	ACAUACUUCUUUAUAUGCCCAU	TACATACTATCATCATC	8
hsa-miR-592	421	8:0:0	-12.3	-8.3	-3.99	UUGUGUCAAUAUGCGAUGAUGU	TTGACACATGTGATT	8
hsa-miR-1303	413	8:1:1	-11.7	-7.73	-3.96	UUUAGAGACGGGGUCUUGCUCU	GTTTCTATTTGACACATGT	8
hsa-miR-548b-5p	658	8:1:1	-10.5	-6.56	-3.93	AAAAGUAAUUGUGGUUUUGGCC	ATTATATTCAT	8
hsa-miR-128	344	8:1:1	-7.1	-3.17	-3.92	CGGGGCCGUAGCACUGUCUGAGA	TCATTGTTCAT	8
hsa-miR-548a-5p	405	8:1:1	-10.6	-6.74	-3.85	AAAAGUAAUUGCGAGUUUUACC	TTTATTTTGTTTC	8
hsa-miR-297	950	8:1:1	-11.3	-7.46	-3.83	AUGUAUGUGUGCAUGUGCAUG	TTCATACATA	8

hsa-miR-495	379	8:1:0	-8.7	-4.88	-3.81	GAAGUUGCCCAUGUUAUUUUCG	TGTTTGTATAA	8
hsa-miR-147	798	8:1:0	-9.5	-5.74	-3.75	GUGUGUGGAAAUGCUUCUGC	TCAACACATTATAATATA	8
hsa-miR-499-5p	368	8:1:1	-8.3	-4.91	-3.38	UUAAGACUUGCAGUGAUGUUU	AATTTTTATT	8
hsa-miR-548i	405	8:1:1	-10.1	-6.74	-3.35	AAAAGUAAUUGCGGAUUUUGCC	TTTATTTTGTTTCTATTT	8
hsa-miR-539	720	8:1:1	-9.8	-6.49	-3.3	GGAGAAAUUAUCCUUGGUGUGU	AATATTTCTTATTATATAT	8
hsa-miR-934	1069	8:1:1	-12.7	-9.43	-3.26	UGUCUACUACUGGAGACACUGG	TAGTATATATAATGTCGC	8
hsa-miR-1256	338	8:1:1	-6.4	-3.15	-3.24	AGGCAUUGACUUCUCACUAGCU	TAAATGTCATTGTTCATAA	8
hsa-miR-183	340	8:1:1	-6.32	-3.11	-3.2	UAUGGCACUGGUAGAAUUCACU	AATGTCATTGTTCATAATT	8
hsa-miR-486-5p	971	8:1:1	-9.6	-6.46	-3.13	UCCUGUACUGAGCUGCCCCGAG	AATATAGGAAATAAATTCT	8
hsa-miR-1322	959	8:1:1	-4.9	-1.77	-3.12	GAUGAUGCUGCUGAUGCUG	ACTATCATCATC	8
hsa-miR-633	958	8:1:0	-4.9	-1.77	-3.12	CUAAUAGUAUCUACCACAAUAAA	TACTATCATCATCAAT	8
hsa-miR-33a	866	8:0:1	-9.5	-6.48	-3.01	GUGCAUUGUAGUUGCAUUGCA	ACAATGTAATATATCAT	8
hsa-miR-142-5p	304	8:1:1	-7	-4.03	-2.96	CAUAAAGUAGAAAGCACUACU	TATTTTTTTTTCA	8
hsa-miR-1262	950	8:1:1	-10.35	-7.46	-2.88	AUGGGUGAAUUUGUAGAAGGAU	TTCATACATACTAT	8
hsa-miR-221	1079	8:1:0	-13.6	-10.72	-2.87	ACCUGGCAUACAAUGUAGAUUU	AATGTCGCGTTATTT	8
hsa-miR-383	1234	8:1:1	-11.3	-8.46	-2.83	AGAUCAGAAGGUGAUUGUGGCU	TTATGATTATAATATATTT	8
hsa-miR-656	385	8:0:0	-11.5	-8.69	-2.8	AGGUUGCCUGUGAGGUGUUCA	TATAATATATAT	8

hsa-miR-548n	373	8:1:1	-7.59	-4.8	-2.78	CAAAAGUAAUUGUGGAUUUUGU	TTATTTTGTTTGTATAAT	8
hsa-miR-1265	802	8:1:1	-10.2	-7.44	-2.75	CAGGAUGUGGUCAAGUGUUGUU	CACATTATAATATAAT	8
hsa-miR-1265	954	8:1:1	-4.5	-1.75	-2.74	CAGGAUGUGGUCAAGUGUUGUU	TACATACTATCATCATCA	8
hsa-miR-1302	1080	8:1:1	-13.6	-10.86	-2.73	UUGGGACAUACUUAUGCUAAA	ATGTCGCGTTATTTGT	8
hsa-miR-599	422	8:1:0	-11	-8.26	-2.73	GUUGUGUCAGUUUAUCAAAC	TGACACATGTGATT	8
hsa-miR-1826	965	8:1:0	-5.09	-2.44	-2.64	UUUGGCAAUGGUAGAACUCACACU	ATCATCAATATAGG	8
hsa-miR-574-5p	711	8:1:1	-12.8	-10.15	-2.64	UGAGUGUGUGUGUGUGAGUGUGU	ATACACTAAAA	8
hsa-miR-380	34	8:1:1	-10.55	-7.92	-2.62	UGGUUGACCAUAGAACAUGCGC	TATTATTTTACTTGTTTAT	8
hsa-miR-450b-3p	1051	8:1:1	-11.5	-9	-2.49	UUGGGAUCAUUUUGCAUCCAUA	TGATTCCTTGTTTT	8
hsa-miR-875-3p	773	8:1:1	-12	-9.5	-2.49	CCUGGAAACACUGAGGUUGUG	GTTTTCAACACCA	8
hsa-miR-548j	433	8:1:1	-11.1	-8.63	-2.46	AAAAGUAAUUGCGGUCUUUGGU	ATTATTTGTATA	8
hsa-miR-1270	1061	8:1:1	-11.05	-8.6	-2.44	CUGGAGAUAUGGAAGAGCUGUGU	TTTTTCCATA	8
hsa-miR-33b	866	8:1:1	-8.8	-6.48	-2.31	GUGCAUUGCUGUUGCAUUGC	ACAATGTAATATATCATAT	8
hsa-miR-524-5p	379	8:1:1	-7.2	-4.88	-2.31	CUACAAAGGGAAGCACUUUCUC	TGTTTGTATAATA	8
hsa-miR-520d-5p	379	8:1:1	-7.2	-4.88	-2.31	CUACAAAGGGAAGCCCUUUC	TGTTTGTATAATATA	8
hsa-miR-203	721	8:1:1	-9.01	-6.71	-2.29	AGUGGUUCUUAACAGUUCAACAGUU	ATATTTCTTATT	8
hsa-miR-584	647	8:1:0	-7.5	-5.22	-2.27	UUAUGGUUUGCCUGGGACUGAG	ATACCATATAAA	8

hsa-miR-643	651	8:1:1	-8.1	-5.85	-2.24	ACUUGUAUGCUAGCUCAGGUAG	CATATAAATTATAT	8
hsa-miR-1272	959	8:1:1	-4	-1.77	-2.22	GAUGAUGAUGGCAGCAAAUUCUGAAA	ACTATCATCATCAAT	8
hsa-miR-586	867	8:1:1	-8.9	-6.68	-2.21	UAUGCAUUGUAUUUUUAGGUCC	CAATGTAATATATC	8
hsa-miR-548b-5p	372	8:1:1	-7.09	-4.99	-2.09	AAAAGUAAUUGUGGUUUUGGCC	TTTATTTTGTTTGTAT	8
hsa-miR-1270	961	8:1:1	-3.8	-1.73	-2.06	CUGGAGAUAUGGAAGAGCUGUGU	TATCATCATCAATAT	8
hsa-miR-190b	800	8:1:1	-8.4	-6.33	-2.06	UGAUAUGUUUGAUAUUGGGUU	AACACATTATAA	8
hsa-miR-208a	401	8:0:1	-8.7	-6.63	-2.06	GAGCUUUUGGCCCGGGUUAUAC	TCGTTTTATTTTGTTTCTA	8
hsa-miR-26a	434	8:1:1	-11.5	-9.45	-2.04	UUCAAGUAAUCCAGGAUAGGCU	TTATTTGTATATATA	8
hsa-miR-208b	401	8:0:1	-8.6	-6.63	-1.96	AAGCUUUUUGCUCGAAUUAUGU	TCGTTTTATTTTGTT	8
hsa-miR-186	984	8:0:0	-10.5	-8.54	-1.95	CAAAGAAUUCUCCUUUUGGGCU	AATTCTTTTT	8
hsa-miR-383	725	8:1:1	-9.51	-7.59	-1.91	AGAUCAGAAGGUGAUUGUGGCU	TTCTTATTATATATAT	8
hsa-miR-200c	670	8:1:1	-12.8	-11.06	-1.73	CGUCUUACCCAGCAGUGUUUGG	GTAGTATATTAT	8
hsa-miR-944	351	8:1:1	-5.4	-3.77	-1.62	AAAUUAUUGUACAUCGGAUGAG	TCATAATTTTAATATAT	8
hsa-miR-190	720	8:1:1	-8.1	-6.49	-1.6	UGAUAUGUUUGAUAUAUUAGGU	AATATTTCTTATTATAT	8
hsa-miR-548n	303	8:0:1	-6.72	-5.12	-1.59	CAAAAGUAAUUGUGGAUUUUGU	TTATTTTTTTTTCATTTT	8
hsa-miR-369-3p	1274	8:0:0	-11.6	-10.03	-1.56	AAUAAUACAUGGUUGAUCUUU	TGTATTATGTAATAG	8
hsa-miR-1259	730	8:0:1	-9.55	-7.98	-1.56	UCCCUGAGACCCUUUAACCUGUGA	ATTATATATAT	8

hsa-miR-548j	658	8:1:1	-8.1	-6.56	-1.53	AAAAGUAAUUGCGGUCUUUGGU	ATTATATTCAT	8
hsa-miR-1	660	8:1:1	-9.3	-7.77	-1.52	ACAUACUUCUUUAUAUGCCCAU	TATATTCATAGTA	8
hsa-miR-944	814	8:1:1	-8.3	-6.78	-1.51	AAAUUAUUGUACAUCGGAUGAG	TAATAATAATGAATTA	8
hsa-miR-581	423	8:1:1	-9.5	-8	-1.49	UCUUGUGUUCUCUAGAUCAGU	GACACATGTGATTATTT	8
hsa-miR-155	845	8:1:1	-9.7	-8.24	-1.45	UUAAUGCUAAUCGUGAUAGGGGU	AAGTATTATTATAAAAT	8
hsa-miR-1283	943	8:1:1	-8.5	-7.08	-1.41	UCUACAAAGGAAAGCGCUUUCU	TTTTGTATTCA	8
hsa-miR-409-3p	863	8:1:1	-7.6	-6.21	-1.38	GAAUGUUGCUCGGUGAACCCCU	GTAACAATGTAATATA	8
hsa-miR-494	410	8:1:0	-8.3	-6.94	-1.35	AGGUUGUCCGUGUUGUCUUCUCU	TTTGTTTCTATTTGACACA	8
hsa-miR-452	857	8:1:1	-7.4	-6.07	-1.32	AACUGUUUGCAGAGGAAACUGA	AAAATAGTAACAATGTAAT	8
hsa-miR-1206	795	8:1:1	-7.5	-6.23	-1.26	UGUUCAUGUAGAUGUUUAAGC	ATATCAACACATTATAAT	8
hsa-miR-1272	968	8:1:1	-6.4	-5.17	-1.22	GAUGAUGAUGGCAGCAAAUUCUGAAA	ATCAATATAGGAAA	8
hsa-miR-190b	720	8:1:1	-7.7	-6.49	-1.2	UGAUAUGUUUGAUAUUGGGUU	AATATTTCTTATTATAT	8
hsa-miR-548l	268	8:1:1	-5.8	-4.61	-1.18	AAAAGUAUUUGCGGGUUUUGUC	AATATATTCGAT	8
hsa-miR-202	641	8:1:1	-7.6	-6.42	-1.17	UUCCUAUGCAUAUACUUCUUUG	TATACCATACCATATA	8
hsa-miR-548a-5p	433	8:1:1	-9.8	-8.63	-1.16	AAAAGUAAUUGCGAGUUUUACC	ATTATTTGTAT	8
hsa-miR-576-5p	630	8:1:0	-5	-3.89	-1.1	AUUCUAAUUUCUCCACGUCUUU	AAATAGAAAAAT	8
hsa-miR-429	1068	8:1:1	-10.1	-9.01	-1.08	UAAUACUGUCUGGUAAAACCGU	ATAGTATATATAATGTCGC	8

hsa-miR-135b	340	8:1:1	-4.19	-3.11	-1.07	UAUGGCUUUUCAUUCCUAUGUGA	AATGTCATTGTTCATA	8
hsa-miR-26b	417	8:1:1	-10.4	-9.35	-1.04	UUCAAGUAAUUCAGGAUAGGU	CTATTTGACACATGT	8
hsa-miR-190b	361	8:1:1	-6.43	-5.42	-1	UGAUAUGUUUGAUAUUGGGUU	AATATATAATT	8
hsa-miR-206	982	8:1:1	-9.31	-8.31	-0.99	UGGAAUGUAAGGAAGUGUGUGG	TAAATTCTTTTTAAGA	8
hsa-miR-135a	340	8:1:1	-4.1	-3.11	-0.98	UAUGGCUUUUUAUUCCUAUGUGA	AATGTCATTGTTCAT	8
hsa-miR-653	796	8:1:0	-7.2	-6.24	-0.95	GUGUUGAAACAAUCUCUACUG	TATCAACACATTATAATA	8
hsa-miR-26a	417	8:1:1	-10.3	-9.35	-0.94	UUCAAGUAAUCCAGGAUAGGCU	CTATTTGACACATGTG	8
hsa-miR-613	639	8:1:1	-6.4	-5.49	-0.9	AGGAAUGUUCCUUCUUUGCC	AATATACCATACCAT	8
hsa-miR-1183	837	8:1:1	-8.7	-7.81	-0.88	CACUGUAGGUGAUGGUGAGAGUGGGCA	CTTACAATAAGTATT	8
hsa-miR-28-3p	829	8:1:0	-6.52	-5.63	-0.88	CACUAGAUUGUGAGCUCCUGGA	AATCTAATCTTACAATAAG	8
hsa-miR-944	1104	8:1:1	-13.5	-12.62	-0.87	AAAUUAUUGUACAUCGGAUGAG	TAATAATATATTTAT	8
hsa-miR-758	349	8:1:1	-4.2	-3.32	-0.87	GAUGGUUGACCAGAGAGCACAC	GTTCATAATTTTAATATA	8
hsa-miR-656	1102	8:1:0	-12	-11.19	-0.8	AGGUUGCCUGUGAGGUGUUCA	TATAATAATAT	8
hsa-miR-144	1095	8:1:1	-10.8	-10.01	-0.78	GGAUAUCAUCAUAUACUGUAAG	TTTATTGTATAATAAT	8
hsa-miR-944	841	8:1:0	-8.8	-8.02	-0.77	AAAUUAUUGUACAUCGGAUGAG	CAATAAGTATTA	8
hsa-miR-105	433	8:1:1	-9.4	-8.63	-0.76	UCAAAUGCUCAGACUCCUGUGGU	ATTATTTGTATATATAAAT	8
hsa-miR-600	212	8:1:1	-10.7	-9.94	-0.75	ACUUACAGACAAGAGCCUUGCUC	TTTGTAAATATATATAT	8

hsa-miR-628-3p	727	8:1:1	-8.5	-7.75	-0.74	UCUAGUAAGAGUGGCAGUCGA	CTTATTATATATATATATA	8
hsa-miR-548n	367	8:1:1	-5.44	-4.69	-0.74	CAAAAGUAAUUGUGGAUUUUGU	TAATTTTTATTT	8
hsa-miR-144	374	8:1:1	-5.7	-4.96	-0.73	GGAUAUCAUCAUAUACUGUAAG	TATTTTGTTTGTATAATAT	8
hsa-miR-433	804	8:1:1	-8.62	-7.89	-0.72	UACGGUGAGCCUGUCAUUAUUC	CATTATAATATAATA	8
hsa-miR-568	390	8:1:1	-8.1	-7.38	-0.71	AUGUAUAAAUGUAUACACAC	TATATATAAATTCGTTT	8
hsa-miR-1284	382	8:1:1	-9.2	-8.54	-0.65	UCUAUACAGACCCUGGCUUUUC	TTGTATAATATATATAAAT	8
hsa-miR-30a	1092	8:1:1	-11.31	-10.7	-0.6	UGUAAACAUCCUCGACUGGAAG	TTGTTTATTGTA	8
hsa-miR-542-3p	420	8:1:1	-9.4	-8.8	-0.59	UGUGACAGAUUGAUAACUGAAA	TTTGACACATGT	8
hsa-miR-1297	434	8:1:1	-10	-9.45	-0.54	UUCAAGUAAUUCAGGUG	TTATTTGTATATATAAAT	8
hsa-miR-30d	1092	8:1:1	-11.21	-10.7	-0.5	UGUAAACAUCCCCGACUGGAAG	TTGTTTATTG	8
hsa-miR-1	982	8:1:1	-8.81	-8.31	-0.49	ACAUACUUCUUUAUAUGCCCAU	TAAATTCTTTTTAAGA	8
hsa-miR-876-3p	1186	8:1:1	-12.3	-11.82	-0.47	UGGUGGUUUACAAAGUAAUUCA	AAAATACCATAACTT	8
hsa-miR-190b	873	8:0:1	-11.3	-10.85	-0.44	UGAUAUGUUUGAUAUUGGGUU	AATATATCATATATAATA	8
hsa-miR-548d-5p	658	8:1:1	-7	-6.56	-0.43	AAAAGUAAUUGUGGUUUUUGCC	ATTATATTCA	8
hsa-miR-508-3p	838	8:1:1	-8.2	-7.8	-0.39	UGAUUGUAGCCUUUUGGAGUAGA	TTACAATAAGTAT	8
hsa-miR-382	865	8:1:1	-6.65	-6.28	-0.36	GAAGUUGUUCGUGGUGGAUUCG	AACAATGTAATATATC	8
hsa-miR-190	361	8:1:1	-5.73	-5.42	-0.3	UGAUAUGUUUGAUAUAUUAGGU	AATATATAATTTT	8

hsa-miR-592	498	8:0:0	-13.7	-13.4	-0.29	UUGUGUCAAUAUGCGAUGAUGU	TTGACACATTTGATTATT	8
hsa-miR-190	718	8:1:1	-6.2	-5.95	-0.24	UGAUAUGUUUGAUAUAUUAGGU	AAAATATTTCTTATTATAT	8
hsa-miR-206	87	8:1:1	-10.7	-10.51	-0.18	UGGAAUGUAAGGAAGUGUGUGG	TATATTCATTTGTAT	8
hsa-miR-875-3p	413	8:1:1	-7.9	-7.73	-0.16	CCUGGAAACACUGAGGUUGUG	GTTTCTATTT	8
hsa-miR-641	304	8:1:1	-4.2	-4.03	-0.16	AAAGACAUAGGAUAGAGUCACCUC	TATTTTTTTTTCATT	8
hsa-miR-548h	405	8:1:1	-6.9	-6.74	-0.15	AAAAGUAAUCGCGGUUUUUGUC	TTTATTTTGTTTCT	8
hsa-miR-208b	314	8:1:1	-5.9	-5.77	-0.12	AAGCUUUUUGCUCGAAUUAUGU	TCATTTTATT	8
hsa-miR-153	866	8:1:1	-6.6	-6.48	-0.11	UCAUUUUUGUGAUGUUGCAGCU	ACAATGTAAT	8
hsa-miR-142-5p	1202	8:1:1	-8.7	-8.6	-0.099	CAUAAAGUAGAAAGCACUACU	TATTTTAAGA	8
hsa-miR-496	659	8:1:1	-6.6	-6.51	-0.084	UGAGUAUUACAUGGCCAAUCUC	TTATATTCATAGTAGTA	8
hsa-miR-568	742	8:1:1	-8.2	-8.15	-0.045	AUGUAUAAAUGUAUACACAC	TATATATATTTAATATA	8
hsa-miR-584	1189	8:1:0	-10.3	-10.26	-0.039	UUAUGGUUUGCCUGGGACUGAG	ATACCATAACT	8
hsa-miR-548n	297	8:1:1	-8	-7.97	-0.029	CAAAAGUAAUUGUGGAUUUUGU	ATATTTTTATTTTT	8
hsa-miR-1272	1236	8:1:1	-8.5	-8.48	-0.017	GAUGAUGAUGGCAGCAAAUUCUGAAA	ATGATTATAATA	8
hsa-miR-1276	197	8:1:1	-12.7	-12.69	-0.00039	UAAAGAGCCCUGUGGAGACA	GGCTTTTATATAATAT	8
hsa-miR-33a	819	8:1:1	-6.9	-6.91	0.012	GUGCAUUGUAGUUGCAUUGCA	ATAATGAATTAATCTAATC	8
hsa-miR-489	1078	8:1:1	-10.9	-10.92	0.023	GGUCGUAUGUGUGACGCCAUUU	TAATGTCGCGT	8

hsa-miR-548p	375	8:1:1	-4.59	-4.62	0.037	UAGCAAAAACUGCAGUUACUUU	ATTTTGTTTGTATA	8
hsa-miR-181a	337	8:1:0	-3.13	-3.16	0.037	AACAUUCAACGCUGUCGGUGAGU	TTAAATGTCAT	8
hsa-miR-297	651	8:1:1	-5.8	-5.85	0.056	AUGUAUGUGUGCAUGUGCAUG	CATATAAATTATATTCATA	8
hsa-miR-1290	636	8:1:1	-2.15	-2.22	0.072	UGGAUUUUUGGAUCAGGGA	AAAAATATACC	8
hsa-miR-656	255	8:0:0	-10	-10.07	0.077	AGGUUGCCUGUGAGGUGUUCA	TATAATATTTCTAAATA	8
hsa-miR-624	1054	8:1:0	-8.9	-8.98	0.086	UAGUACCAGUACCUUGUGUUCA	TTCCTTGTTTTTC	8
hsa-miR-302c	1004	8:1:1	-10.8	-10.89	0.09	UUUAACAUGGGGGUACCUGCUG	AAGAATTTTTTTT	8
hsa-miR-190b	639	8:1:1	-5.4	-5.49	0.092	UGAUAUGUUUGAUAUUGGGUU	AATATACCATACC	8
hsa-miR-548c-5p	433	8:1:1	-8.5	-8.63	0.13	AAAAGUAAUUGCGGUUUUUGCC	ATTATTTGTATAT	8
hsa-miR-548i	433	8:1:1	-8.5	-8.63	0.13	AAAAGUAAUUGCGGAUUUUGCC	ATTATTTGTATATAT	8
hsa-miR-570	771	8:1:0	-7.91	-8.07	0.16	AAAGGUAAUUGCAGUUUUUCCC	ATGTTTTCAA	8
hsa-miR-573	275	8:1:1	-6.2	-6.36	0.16	CUGAAGUGAUGUGUAACUGAUCAG	TCGATTCATAATTTTTT	8
hsa-miR-569	966	8:1:1	-3.2	-3.37	0.17	AGUUAAUGAAUCCUGGAAAGU	TCATCAATAT	8
hsa-miR-30a	771	8:1:0	-7.9	-8.07	0.17	UGUAAACAUCCUCGACUGGAAG	ATGTTTTCAACACCATAT	8
hsa-miR-584	637	8:1:1	-1.9	-2.14	0.24	UUAUGGUUUGCCUGGGACUGAG	AAAATATACCATACCAT	8
hsa-miR-548c-5p	405	8:1:1	-6.5	-6.74	0.24	AAAAGUAAUUGCGGUUUUUGCC	TTTATTTTGTTTCTATTTG	8
hsa-miR-586	276	8:1:1	-6.8	-7.06	0.26	UAUGCAUUGUAUUUUUAGGUCC	CGATTCATAATTTTTTG	8

hsa-miR-369-3p	846	8:1:0	-7.8	-8.07	0.27	AAUAAUACAUGGUUGAUCUUU	AGTATTATTATAAAAT	8
hsa-miR-548l	720	8:1:1	-6.2	-6.49	0.29	AAAAGUAUUUGCGGGUUUUGUC	AATATTTCTTATTATA	8
hsa-miR-147	421	8:1:1	-8	-8.3	0.3	GUGUGUGGAAAUGCUUCUGC	TTGACACATGTGAT	8
hsa-miR-380	657	8:1:1	-6.4	-6.71	0.31	UGGUUGACCAUAGAACAUGCGC	AATTATATTCATA	8
hsa-miR-380	1069	8:1:1	-9.1	-9.43	0.33	UGGUUGACCAUAGAACAUGCGC	TAGTATATAT	8
hsa-miR-664	820	8:1:0	-6.51	-6.84	0.33	ACUGGCUAGGGAAAAUGAUUGGAU	TAATGAATTAATCT	8
hsa-miR-584	642	8:1:0	-5.9	-6.24	0.34	UUAUGGUUUGCCUGGGACUGAG	ATACCATACC	8
hsa-miR-548j	405	8:1:1	-6.4	-6.74	0.34	AAAAGUAAUUGCGGUCUUUGGU	TTTATTTTGTT	8
hsa-miR-559	405	8:0:1	-6.39	-6.74	0.35	UAAAGUAAAUAUGCACCAAAA	TTTATTTTGTTTCTATTTG	8
hsa-miR-656	155	8:1:0	-9.8	-10.17	0.37	AGGUUGCCUGUGAGGUGUUCA	TATAATAATAT	8
hsa-miR-616	1025	8:1:1	-11.5	-11.91	0.41	ACUCAAAACCCUUCAGUGACUU	CTAATTACGTATA	8
hsa-miR-520d-5p	210	8:1:1	-9.6	-10.01	0.41	CUACAAAGGGAAGCCCUUUC	TATTTGTAAATATAT	8
hsa-miR-539	368	8:1:1	-4.49	-4.91	0.42	GGAGAAAUUAUCCUUGGUGUGU	AATTTTTATTTTGTTTGTA	8
hsa-miR-181c	337	8:1:0	-2.69	-3.16	0.47	AACAUUCAACCUGUCGGUGAGU	TTAAATGTCAT	8
hsa-miR-490-5p	277	8:1:1	-7.4	-7.88	0.48	CCAUGGAUCUCCAGGUGGGU	GATTCATAATTTT	8
hsa-miR-190	639	8:1:1	-5	-5.49	0.49	UGAUAUGUUUGAUAUAUUAGGU	AATATACCAT	8
hsa-miR-548n	319	8:0:1	-7.2	-7.69	0.49	CAAAAGUAAUUGUGGAUUUUGU	TTATTTTTGAT	8

hsa-miR-590-3p	206	8:1:0	-12.1	-12.59	0.49	UAAUUUUAUGUAUAAGCUAGU	ATAATATTTGTA	8
hsa-miR-1257	951	8:1:1	-5.43	-5.93	0.5	AGUGAAUGAUGGGUUCUGACC	TCATACATACTAT	8
hsa-miR-548n	406	8:1:1	-6.3	-6.8	0.5	CAAAAGUAAUUGUGGAUUUUGU	TTATTTTGTTTCTATTTG	8
hsa-miR-583	922	8:0:1	-10.22	-10.75	0.53	CAAAGAGGAAGGUCCCAUUAC	TCCTTTTTTTTT	8
hsa-miR-190	388	8:1:1	-6.85	-7.39	0.54	UGAUAUGUUUGAUAUAUUAGGU	AATATATATAAATTCGT	8
hsa-miR-1826	1236	8:1:1	-7.9	-8.48	0.58	UUUGGCAAUGGUAGAACUCACACU	ATGATTATAATATA	8
hsa-miR-186	1007	8:0:1	-8.56	-9.19	0.63	CAAAGAAUUCUCCUUUUGGGCU	AATTTTTTTTTA	8
hsa-miR-1272	805	8:1:1	-8.01	-8.64	0.63	GAUGAUGAUGGCAGCAAAUUCUGAAA	ATTATAATATAATA	8
hsa-miR-511	637	8:1:1	-1.5	-2.14	0.64	GUGUCUUUUGCUCUGCAGUCA	AAAATATACCA	8
hsa-miR-559	302	8:0:1	-5.6	-6.25	0.65	UAAAGUAAAUAUGCACCAAAA	TTTATTTTTTTTTCA	8
hsa-miR-208a	314	8:1:1	-5.1	-5.77	0.67	GAGCUUUUGGCCCGGGUUAUAC	TCATTTTATTTTTGATTTT	8
hsa-miR-126	1231	8:1:1	-8.4	-9.13	0.73	CAUUAUUACUUUUGGUACGCG	ACGTTATGATTATAATATA	8
hsa-miR-203	793	8:1:1	-5	-5.73	0.73	AGUGGUUCUUAACAGUUCAACAGUU	ATATATCAACACAT	8
hsa-miR-577	306	8:1:1	-3.9	-4.63	0.73	UAGAUAAAAUAUUGGUACCUG	TTTTTTTTTCA	8
hsa-miR-891b	1178	8:0:1	-13.5	-14.24	0.74	UGCAACUUACCUGAGUCAUUGA	TAAGTTGTAAAATACC	8
hsa-miR-944	257	8:1:1	-9.1	-9.84	0.74	AAAUUAUUGUACAUCGGAUGAG	TAATATTTCTAAATA	8

hsa-miR-568	740	8:1:1	-8.2	-8.95	0.75	AUGUAUAAAUGUAUACACAC	TATATATATAT	8
hsa-miR-26b	434	8:1:1	-8.7	-9.45	0.75	UUCAAGUAAUUCAGGAUAGGU	TTATTTGTATAT	8
hsa-miR-568	738	8:1:1	-8.1	-8.9	0.8	AUGUAUAAAUGUAUACACAC	TATATATATAT	8
hsa-miR-653	775	8:0:0	-9	-9.81	0.81	GUGUUGAAACAAUCUCUACUG	TTTCAACACC	8
hsa-miR-23b	867	8:1:0	-5.81	-6.68	0.87	UGGGUUCCUGGCAUGCUGAUUU	CAATGTAATATATC	8
hsa-miR-944	817	8:1:1	-6.04	-6.91	0.87	AAAUUAUUGUACAUCGGAUGAG	TAATAATGAATT	8
hsa-miR-1278	667	8:1:1	-9	-9.87	0.87	UAGUACUGUGCAUAUCAUCUAU	ATAGTAGTATATT	8
hsa-miR-568	175	8:1:1	-12.4	-13.28	0.88	AUGUAUAAAUGUAUACACAC	TATATATATAATTTGT	8
hsa-miR-1259	649	8:1:0	-4.8	-5.7	0.9	UCCCUGAGACCCUUUAACCUGUGA	ACCATATAAAT	8
hsa-miR-200c	847	8:1:1	-6.9	-7.81	0.91	CGUCUUACCCAGCAGUGUUUGG	GTATTATTATAAAA	8
hsa-miR-1	87	8:1:1	-9.6	-10.51	0.91	ACAUACUUCUUUAUAUGCCCAU	TATATTCATTTGT	8
hsa-miR-1252	410	8:1:1	-6	-6.94	0.94	AGAAGGAAAUUGAAUUCAUUUA	TTTGTTTCTATTTGACAC	8
hsa-miR-380	1243	8:1:1	-7.3	-8.26	0.96	UGGUUGACCAUAGAACAUGCGC	TAATATATTTGA	8
hsa-miR-203	314	8:1:1	-4.8	-5.77	0.97	AGUGGUUCUUAACAGUUCAACAGUU	TCATTTTATTTTTG	8
hsa-miR-384	973	8:1:0	-7.1	-8.08	0.98	AUUCCUAGAAAUUGUUCAUA	TATAGGAAATAAA	8
hsa-miR-23a	867	8:1:0	-5.7	-6.68	0.98	GGGGUUCCUGGGGAUGGGAUUU	CAATGTAATATATC	8
hsa-miR-944	157	8:1:1	-10.2	-11.2	1	AAAUUAUUGUACAUCGGAUGAG	TAATAATATT	8

hsa-miR-890	263	8:1:1	-5.6	-6.61	1.01	UACUUGGAAAGGCAUCAGUUG	TTCTAAATATATTCGAT	8
hsa-miR-374a	1275	8:0:1	-9.1	-10.12	1.02	UUAUAAUACAACCUGAUAAGUG	GTATTATGTAATAGTA	8
hsa-miR-511	619	8:1:0	-7.9	-8.92	1.02	GUGUCUUUUGCUCUGCAGUCA	AAAAGAAAAAAAAATA	8
hsa-miR-19b	211	8:1:1	-7.8	-8.84	1.04	AGUUUUGCAUAGUUGCACUACA	ATTTGTAAATAT	8
hsa-miR-559	40	8:1:0	-9.05	-10.09	1.04	UAAAGUAAAUAUGCACCAAAA	TTTACTTGTTTATTTTT	8
hsa-miR-548b-5p	302	8:1:1	-5.2	-6.25	1.05	AAAAGUAAUUGUGGUUUUGGCC	TTTATTTTTT	8
hsa-miR-381	1056	8:1:0	-8	-9.07	1.07	AGCGAGGUUGCCCUUUGUAUAU	CCTTGTTTTTCCATAGT	8
hsa-miR-300	1056	8:1:0	-8	-9.07	1.07	UAUACAAGGGCAGACUCUCUCU	CCTTGTTTTTCCATAGTAT	8
hsa-miR-523	1082	8:1:1	-10.33	-11.43	1.1	CUCUAGAGGGAAGCGCUUUCUG	GTCGCGTTATTT	8
hsa-miR-570	308	8:1:1	-3.2	-4.31	1.11	AAAGGUAAUUGCAGUUUUUCCC	TTTTTTTCATTTTATT	8
hsa-miR-548b-5p	433	8:1:1	-7.5	-8.63	1.13	AAAAGUAAUUGUGGUUUUGGCC	ATTATTTGTATATA	8
hsa-miR-487a	253	8:1:0	-10.6	-11.73	1.13	GUGGUUAUCCCUGCUGUGUUCG	TGTATAATATTTCT	8
hsa-miR-300	380	8:1:1	-5.4	-6.54	1.14	UAUACAAGGGCAGACUCUCUCU	GTTTGTATAATATA	8
hsa-miR-934	671	8:1:1	-10.2	-11.35	1.15	UGUCUACUACUGGAGACACUGG	TAGTATATTATATGGT	8
hsa-miR-144	407	8:1:1	-5.6	-6.75	1.15	GGAUAUCAUCAUAUACUGUAAG	TATTTTGTTTC	8
hsa-miR-592	950	8:1:1	-6.3	-7.46	1.16	UUGUGUCAAUAUGCGAUGAUGU	TTCATACATA	8
hsa-miR-655	869	8:1:0	-7	-8.18	1.18	AGAGGUUAUCCGUGUUAUGUUC	ATGTAATATA	8

hsa-miR-190	1212	8:1:1	-6.6	-7.79	1.19	UGAUAUGUUUGAUAUAUUAGGU	AATATATATATAA	8
hsa-miR-26a	494	8:0:1	-12.4	-13.59	1.19	UUCAAGUAAUCCAGGAUAGGCU	TTATTTGACACAT	8
hsa-miR-26b	494	8:0:1	-12.4	-13.59	1.19	UUCAAGUAAUUCAGGAUAGGU	TTATTTGACACATTTGA	8
hsa-miR-369-3p	870	8:1:0	-7.3	-8.51	1.21	AAUAAUACAUGGUUGAUCUUU	TGTAATATATC	8
hsa-miR-374b	1275	8:0:1	-8.9	-10.12	1.22	AUAUAAUACAACCUGCUAAGUG	GTATTATGTAAT	8
hsa-miR-200c	294	8:1:1	-7.1	-8.34	1.24	CGUCUUACCCAGCAGUGUUUGG	GTAATATTTTTATT	8
hsa-miR-649	1228	8:1:1	-9.1	-10.36	1.26	AAACCUGUGUUGUUCAAGAGUC	TACACGTTATGATTATA	8
hsa-miR-1259	852	8:1:1	-6.6	-7.86	1.26	UCCCUGAGACCCUUUAACCUGUGA	ATTATAAAATAGTA	8
hsa-miR-186	328	8:1:1	-2.8	-4.06	1.26	CAAAGAAUUCUCCUUUUGGGCU	ATTTTTTTTTTA	8
hsa-miR-548k	846	8:1:1	-6.8	-8.07	1.27	AAAAGUACUUGCGGAUUUUGCU	AGTATTATTATAAAA	8
hsa-miR-548b-5p	366	8:1:1	-3.3	-4.59	1.29	AAAAGUAAUUGUGGUUUUGGCC	ATAATTTTTATTTTGTT	8
hsa-miR-142-5p	315	8:1:1	-5.3	-6.6	1.3	CAUAAAGUAGAAAGCACUACU	CATTTTATTTTTGATTTTT	8
hsa-miR-548p	306	8:1:1	-3.3	-4.63	1.33	UAGCAAAAACUGCAGUUACUUU	TTTTTTTTTCATTTTAT	8
hsa-miR-599	499	8:1:0	-11	-12.34	1.34	GUUGUGUCAGUUUAUCAAAC	TGACACATTTGA	8
hsa-miR-656	1241	8:0:0	-7.2	-8.56	1.36	AGGUUGCCUGUGAGGUGUUCA	TATAATATATTTGAAT	8
hsa-miR-944	280	8:1:1	-7.4	-8.76	1.36	AAAUUAUUGUACAUCGGAUGAG	TCATAATTTTT	8
hsa-miR-891a	400	8:1:1	-4.9	-6.33	1.43	UGCAACGAACCUGAGCCACUGA	TTCGTTTTATTTT	8

hsa-miR-382	1254	8:1:1	-9.1	-10.54	1.44	GAAGUUGUUCGUGGUGGAUUCG	AATAACATAAAACAATAG	8
hsa-miR-891a	410	8:1:1	-5.5	-6.94	1.44	UGCAACGAACCUGAGCCACUGA	TTTGTTTCTATTT	8
hsa-miR-381	380	8:1:1	-5.1	-6.54	1.44	AGCGAGGUUGCCCUUUGUAUAU	GTTTGTATAATATATAT	8
hsa-miR-1279	294	8:1:1	-6.9	-8.34	1.44	UCAUAUUGCUUCUUUCU	GTAATATTTTTAT	8
hsa-miR-151-3p	314	8:1:1	-4.32	-5.77	1.45	UCGAGGAGCUCACAGUCUAGU	TCATTTTATTTTTGATT	8
hsa-miR-548h	328	8:1:1	-2.6	-4.06	1.46	AAAAGUAAUCGCGGUUUUUGUC	ATTTTTTTTTTAAATGTC	8
hsa-miR-30e	1092	8:1:1	-9.21	-10.7	1.49	UGUAAACAUCCUUGACUGGAAG	TTGTTTATTGTATAATAA	8
hsa-miR-559	372	8:0:1	-3.5	-4.99	1.49	UAAAGUAAAUAUGCACCAAAA	TTTATTTTGTTTGTAT	8
hsa-miR-548d-5p	372	8:1:1	-3.5	-4.99	1.49	AAAAGUAAUUGUGGUUUUUGCC	TTTATTTTGTTT	8
hsa-miR-491-3p	382	8:0:1	-7	-8.54	1.54	CUUAUGCAAGAUUCCCUUCUAC	TTGTATAATAT	8
hsa-miR-577	371	8:0:1	-3.6	-5.14	1.54	UAGAUAAAAUAUUGGUACCUG	TTTTATTTTGTTTGTA	8
hsa-miR-376c	1199	8:1:1	-7	-8.56	1.56	GGUGGAUAUUCCUUCUAUGUU	TTCTATTTTAAGAAAT	8
hsa-miR-190b	718	8:1:1	-4.39	-5.95	1.56	UGAUAUGUUUGAUAUUGGGUU	AAAATATTTCTTATT	8
hsa-miR-559	35	8:1:1	-6.87	-8.44	1.57	UAAAGUAAAUAUGCACCAAAA	ATTATTTTACTTGTTTA	8
hsa-miR-583	1055	8:1:1	-7.4	-8.98	1.58	CAAAGAGGAAGGUCCCAUUAC	TCCTTGTTTTT	8
hsa-miR-590-3p	855	8:1:0	-6.3	-7.88	1.58	UAAUUUUAUGUAUAAGCUAGU	ATAAAATAGTA	8
hsa-miR-200b	670	8:1:1	-9.47	-11.06	1.59	CAUCUUACUGGGCAGCAUUGGA	GTAGTATATTATATG	8

hsa-miR-943	859	8:1:1	-4.6	-6.2	1.6	CUGACUGUUGCCGUCCUCCAG	AATAGTAACAATGTAAT	8
hsa-miR-548p	307	8:1:1	-2.72	-4.34	1.62	UAGCAAAAACUGCAGUUACUUU	TTTTTTTTCATTTTA	8
hsa-miR-568	1216	8:1:1	-4.9	-6.53	1.63	AUGUAUAAAUGUAUACACAC	TATATATAATAATACACG	8
hsa-miR-299-3p	951	8:1:1	-4.3	-5.93	1.63	UAUGUGGGAUGGUAAACCGCUU	TCATACATACTAT	8
hsa-miR-1244	1195	8:1:1	-7.4	-9.04	1.64	AAGUAGUUGGUUUGUAUGAGAUGGUU	TAACTTCTATTTTA	8
hsa-miR-19a	211	8:1:1	-7.2	-8.84	1.64	AGUUUUGCAUAGUUGCACUACA	ATTTGTAAATATATATAT	8
hsa-miR-548c-3p	326	8:1:0	-4.1	-5.75	1.65	CAAAAAUCUCAAUUACUUUUGC	TGATTTTTTTTTAAATGT	8
hsa-miR-1267	1064	8:1:1	-5.1	-6.77	1.67	CCUGUUGAAGUGUAAUCCCCA	TTCCATAGTATATAT	8
hsa-miR-802	1058	8:1:1	-7.4	-9.07	1.67	CAGUAACAAAGAUUCAUCCUUGU	TTGTTTTTCCAT	8
hsa-miR-155	1083	8:1:1	-10.12	-11.81	1.69	UUAAUGCUAAUCGUGAUAGGGGU	TCGCGTTATTTGTTTATT	8
hsa-miR-190	218	8:1:1	-9.6	-11.3	1.7	UGAUAUGUUUGAUAUAUUAGGU	AATATATATATAT	8
hsa-miR-374a	847	8:1:0	-6.1	-7.81	1.71	UUAUAAUACAACCUGAUAAGUG	GTATTATTATAAAATAGTA	8
hsa-miR-374b	847	8:1:0	-6.1	-7.81	1.71	AUAUAAUACAACCUGCUAAGUG	GTATTATTATAAAATAG	8
hsa-miR-577	301	8:0:1	-5.3	-7.01	1.71	UAGAUAAAAUAUUGGUACCUG	TTTTATTTTTTTTTCA	8
hsa-miR-578	839	8:1:1	-6.2	-7.92	1.72	CUUCUUGUGCUCUAGGAUUGU	TACAATAAGTATTATT	8
hsa-miR-1252	312	8:1:1	-3.5	-5.22	1.72	AGAAGGAAAUUGAAUUCAUUUA	TTTCATTTTAT	8
hsa-miR-410	1097	8:1:1	-8.6	-10.35	1.75	AGGUUGUCUGUGAUGAGUUCG	TATTGTATAATAATA	8

hsa-miR-548a-5p	305	8:1:1	-2.7	-4.49	1.79	AAAAGUAAUUGCGAGUUUUACC	ATTTTTTTTTC	8
hsa-miR-511	627	8:1:1	-3.7	-5.53	1.83	GUGUCUUUUGCUCUGCAGUCA	AAAAAATAGAAA	8
hsa-miR-548d-5p	35	8:0:1	-6.6	-8.44	1.84	AAAAGUAAUUGUGGUUUUUGCC	ATTATTTTACTT	8
hsa-miR-641	722	8:1:0	-5.2	-7.05	1.85	AAAGACAUAGGAUAGAGUCACCUC	TATTTCTTATTATATAT	8
hsa-miR-548a-5p	302	8:1:1	-4.4	-6.25	1.85	AAAAGUAAUUGCGAGUUUUACC	TTTATTTTTTTTT	8
hsa-miR-586	820	8:1:1	-4.96	-6.84	1.88	UAUGCAUUGUAUUUUUAGGUCC	TAATGAATTAA	8
hsa-miR-186	304	8:1:1	-2.15	-4.03	1.88	CAAAGAAUUCUCCUUUUGGGCU	TATTTTTTTTTCATTTT	8
hsa-miR-200b	847	8:1:1	-5.9	-7.81	1.91	CAUCUUACUGGGCAGCAUUGGA	GTATTATTATAAA	8
hsa-miR-606	668	8:1:1	-7.9	-9.83	1.93	AAACUACUGAAAAUCAAAGAU	TAGTAGTATAT	8
hsa-miR-545	1094	8:1:1	-8.7	-10.64	1.94	UCAGUAAAUGUUUAUUAGAUGA	GTTTATTGTATAATA	8
hsa-miR-802	411	8:1:0	-5.6	-7.55	1.95	CAGUAACAAAGAUUCAUCCUUGU	TTGTTTCTATT	8
hsa-miR-515-5p	555	8:1:0	-8.05	-10.01	1.96	UUCUCCAAAAGAAAGCACUUUCUG	TTTGGATATTTGTATA	8
hsa-miR-491-3p	96	8:0:1	-7.1	-9.07	1.97	CUUAUGCAAGAUUCCCUUCUAC	TTGTATAATATAAA	8
hsa-miR-302a	1004	8:1:1	-8.91	-10.89	1.98	ACUUAAACGUGGAUGUACUUGCU	AAGAATTTTTTTTAAC	8
hsa-miR-1259	878	8:0:0	-8.3	-10.31	2.01	UCCCUGAGACCCUUUAACCUGUGA	ATCATATATA	8
hsa-miR-186	368	8:1:1	-2.9	-4.91	2.01	CAAAGAAUUCUCCUUUUGGGCU	AATTTTTATTT	8
hsa-miR-548n	307	8:1:1	-2.33	-4.34	2.01	CAAAAGUAAUUGUGGAUUUUGU	TTTTTTTTCATTT	8

hsa-miR-656	590	8:0:0	-9.7	-11.72	2.02	AGGUUGCCUGUGAGGUGUUCA	TATAATATGTATG	8
hsa-miR-548c-5p	35	8:0:1	-6.4	-8.44	2.04	AAAAGUAAUUGCGGUUUUUGCC	ATTATTTTACT	8
hsa-miR-568	439	8:1:1	-8.2	-10.25	2.05	AUGUAUAAAUGUAUACACAC	TGTATATATAAATTTG	8
hsa-miR-340	403	8:1:1	-5	-7.05	2.05	UUAUAAAGCAAUGAGACUGAUU	GTTTTATTTTG	8
hsa-miR-548d-5p	302	8:1:1	-4.2	-6.25	2.05	AAAAGUAAUUGUGGUUUUUGCC	TTTATTTTTTTTTCATTT	8
hsa-miR-1179	1270	8:1:1	-9.2	-11.26	2.06	AAGCAUUCUUUCAUUGGUUGG	AGAATGTATTATGTAA	8
hsa-miR-382	1193	8:1:1	-7.4	-9.46	2.06	GAAGUUGUUCGUGGUGGAUUCG	CATAACTTCTATTT	8
hsa-miR-448	593	8:1:1	-10.15	-12.21	2.06	UUGCAUAUGUAGGAUGUCCCAU	AATATGTATGTGATTA	8
hsa-miR-548p	408	8:1:1	-4.7	-6.78	2.08	UAGCAAAAACUGCAGUUACUUU	ATTTTGTTTCTATTTG	8
hsa-miR-577	331	8:1:1	-2.2	-4.3	2.1	UAGAUAAAAUAUUGGUACCUG	TTTTTTTTAAATGTCATT	8
hsa-miR-548n	329	8:1:1	-1.9	-4	2.1	CAAAAGUAAUUGUGGAUUUUGU	TTTTTTTTTTAAAT	8
hsa-miR-374b	871	8:1:0	-7.3	-9.41	2.11	AUAUAAUACAACCUGCUAAGUG	GTAATATATC	8
hsa-miR-768-5p	836	8:1:1	-4.85	-6.97	2.12	UGCACCAUGGUUGUCUGAGCAUG	TCTTACAATAAGTATTATT	8
hsa-miR-548e	303	8:1:1	-3	-5.12	2.12	CAAAAGCAAUCGCGGUUUUUGC	TTATTTTTTTT	8
hsa-miR-944	295	8:1:1	-6.1	-8.25	2.15	AAAUUAUUGUACAUCGGAUGAG	TAATATTTTTATTT	8
hsa-miR-361-5p	323	8:1:1	-5.55	-7.71	2.16	UUAUCAGAAUCUCCAGGGGUAC	TTTTGATTTTTTTTTTA	8
hsa-miR-577	404	8:0:1	-4.6	-6.78	2.18	UAGAUAAAAUAUUGGUACCUG	TTTTATTTTGTTTCTATT	8

hsa-miR-337-3p	15	8:1:0	-6.2	-8.38	2.18	CUCCUAUAUGAUGCCUUUCUUC	ATATAAGAAAAAA	8
hsa-miR-190b	1212	8:1:1	-5.6	-7.79	2.19	UGAUAUGUUUGAUAUUGGGUU	AATATATATATAATAATA	8
hsa-miR-548i	305	8:1:1	-2.3	-4.49	2.19	AAAAGUAAUUGCGGAUUUUGCC	ATTTTTTTTCATTTTATT	8
hsa-miR-620	1061	8:1:1	-6.4	-8.6	2.2	AUGGAGAUAGAUAUAGAAAU	TTTTTCCATAGTAT	8
hsa-miR-548i	372	8:1:1	-2.79	-4.99	2.2	AAAAGUAAUUGCGGAUUUUGCC	TTTATTTTGTTTGTA	8
hsa-miR-656	99	8:0:0	-9.7	-11.92	2.22	AGGUUGCCUGUGAGGUGUUCA	TATAATATAAATATATAT	8
hsa-miR-548d-5p	433	8:1:1	-6.4	-8.63	2.23	AAAAGUAAUUGUGGUUUUUGCC	ATTATTTGTA	8
hsa-miR-499-5p	845	8:1:0	-6	-8.24	2.24	UUAAGACUUGCAGUGAUGUUU	AAGTATTATTATAAAAT	8
hsa-miR-300	1097	8:1:1	-8.1	-10.35	2.25	UAUACAAGGGCAGACUCUCUCU	TATTGTATAATAATAT	8
hsa-miR-548i	302	8:1:1	-4	-6.25	2.25	AAAAGUAAUUGCGGAUUUUGCC	TTTATTTTTTTTTCATTTT	8
hsa-miR-548c-5p	658	8:1:1	-4.3	-6.56	2.26	AAAAGUAAUUGCGGUUUUUGCC	ATTATATTCATAGTA	8
hsa-miR-548n	306	8:1:1	-2.37	-4.63	2.26	CAAAAGUAAUUGUGGAUUUUGU	TTTTTTTTTCATTTT	8
hsa-miR-101	846	8:1:1	-5.8	-8.07	2.27	CAGUUAUCACAGUGCUGAUGCU	AGTATTATTATAAAATA	8
hsa-miR-30e	771	8:1:0	-5.8	-8.07	2.27	UGUAAACAUCCUUGACUGGAAG	ATGTTTTCAACACCATAT	8
hsa-miR-374a	384	8:1:0	-7.2	-9.47	2.27	UUAUAAUACAACCUGAUAAGUG	GTATAATATATATAATT	8
hsa-miR-651	851	8:1:1	-5.4	-7.68	2.28	UUUAGGAUAAGCUUGACUUUUG	TATTATAAAATA	8
hsa-miR-219-1-3p	776	8:1:1	-7.6	-9.88	2.28	UGAUUGUCCAAACGCAAUUCU	TTCAACACCATATAC	8

hsa-miR-1303	261	8:1:1	-6.3	-8.58	2.28	UUUAGAGACGGGGUCUUGCUCU	ATTTCTAAATATAT	8
hsa-miR-374a	1101	8:1:0	-9	-11.29	2.29	UUAUAAUACAACCUGAUAAGUG	GTATAATAATATATTTATT	8
hsa-miR-548a-5p	372	8:1:1	-2.7	-4.99	2.29	AAAAGUAAUUGCGAGUUUUACC	TTTATTTTGTTTGTATAA	8
hsa-miR-548c-5p	372	8:1:1	-2.7	-4.99	2.29	AAAAGUAAUUGCGGUUUUUGCC	TTTATTTTGTTTGTA	8
hsa-miR-548j	372	8:1:1	-2.7	-4.99	2.29	AAAAGUAAUUGCGGUCUUUGGU	TTTATTTTGTTTGTATA	8
hsa-miR-548h	372	8:1:1	-2.7	-4.99	2.29	AAAAGUAAUCGCGGUUUUUGUC	TTTATTTTGTTT	8
hsa-miR-586	1271	8:1:1	-8.4	-10.7	2.3	UAUGCAUUGUAUUUUUAGGUCC	GAATGTATTATGTAATAG	8
hsa-miR-1185	919	8:1:1	-8.9	-11.2	2.3	AGAGGAUACCCUUUGUAUGUU	ATATCCTTTTTTTTT	8
hsa-miR-374a	1096	8:1:1	-8.2	-10.51	2.31	UUAUAAUACAACCUGAUAAGUG	TTATTGTATAATAATAT	8
hsa-miR-425	402	8:1:1	-4.8	-7.12	2.32	AAUGACACGAUCACUCCCGUUGA	CGTTTTATTTTGTTT	8
hsa-miR-1259	361	8:1:1	-3.1	-5.42	2.32	UCCCUGAGACCCUUUAACCUGUGA	AATATATAATTTTTAT	8
hsa-miR-494	1059	8:1:1	-6.7	-9.06	2.36	AGGUUGUCCGUGUUGUCUUCUCU	TGTTTTTCCA	8
hsa-miR-151-3p	827	8:1:1	-3.3	-5.66	2.36	UCGAGGAGCUCACAGUCUAGU	TTAATCTAATC	8
hsa-miR-1267	776	8:1:0	-7.5	-9.88	2.38	CCUGUUGAAGUGUAAUCCCCA	TTCAACACCATA	8
hsa-miR-548p	331	8:1:1	-1.92	-4.3	2.38	UAGCAAAAACUGCAGUUACUUU	TTTTTTTTAAATGTCATT	8
hsa-miR-203	259	8:1:1	-7.1	-9.5	2.4	AGUGGUUCUUAACAGUUCAACAGUU	ATATTTCTAAATATATT	8
hsa-miR-570	945	8:1:1	-4.6	-7.03	2.43	AAAGGUAAUUGCAGUUUUUCCC	TTGTATTCATA	8

hsa-miR-548p	942	8:1:1	-4.8	-7.24	2.44	UAGCAAAAACUGCAGUUACUUU	TTTTTGTATTCA	8
hsa-miR-187	1226	8:1:0	-7.6	-10.05	2.45	GGCUACAACACAGGACCCGGGC	AATACACGTTATGATTAT	8
hsa-miR-520f	1003	8:1:1	-8.2	-10.65	2.45	CCUCUAAAGGGAAGCGCUUUCU	GAAGAATTTTTTT	8
hsa-miR-633	848	8:0:1	-5.4	-7.86	2.46	CUAAUAGUAUCUACCACAAUAAA	TATTATTATAA	8
hsa-miR-944	1222	8:1:1	-5.7	-8.17	2.47	AAAUUAUUGUACAUCGGAUGAG	TAATAATACACGTT	8
hsa-miR-376c	415	8:1:1	-6.3	-8.78	2.48	GGUGGAUAUUCCUUCUAUGUU	TTCTATTTGACACA	8
hsa-miR-1259	1242	8:1:0	-6	-8.51	2.51	UCCCUGAGACCCUUUAACCUGUGA	ATAATATATTT	8
hsa-miR-137	1208	8:1:0	-6.1	-8.61	2.51	UUAUUGCUUAAGAAUACGCGUAG	AAGAAATATATA	8
hsa-miR-590-3p	364	8:1:0	-2.4	-4.95	2.55	UAAUUUUAUGUAUAAGCUAGU	ATATAATTTTTATTTTG	8
hsa-miR-584	876	8:1:1	-7.6	-10.17	2.57	UUAUGGUUUGCCUGGGACUGAG	ATATCATATATAATAAT	8
hsa-miR-1284	96	8:1:1	-6.5	-9.07	2.57	UCUAUACAGACCCUGGCUUUUC	TTGTATAATATA	8
hsa-miR-302b	1004	8:1:1	-8.3	-10.89	2.59	ACUUUAACAUGGAAGUGCUUUC	AAGAATTTTTTT	8
hsa-miR-548b-5p	849	8:1:1	-5.2	-7.79	2.59	AAAAGUAAUUGUGGUUUUGGCC	ATTATTATAAAATAG	8
hsa-miR-92a	868	8:1:1	-4.6	-7.2	2.6	UAUUGCACUUGUCCCGGCCUGU	AATGTAATAT	8
hsa-miR-92b	868	8:1:1	-4.6	-7.2	2.6	AGGGACGGGACGCGGUGCAGUG	AATGTAATATATCA	8
hsa-miR-494	770	8:1:1	-5.7	-8.3	2.6	AGGUUGUCCGUGUUGUCUUCUCU	AATGTTTTCAACACCA	8
hsa-miR-656	812	8:1:0	-3.8	-6.41	2.61	AGGUUGCCUGUGAGGUGUUCA	TATAATAATAA	8

hsa-miR-584	780	8:1:0	-7.35	-9.96	2.61	UUAUGGUUUGCCUGGGACUGAG	ACACCATATACGGATA	8
hsa-miR-1297	457	8:1:1	-8.9	-11.51	2.61	UUCAAGUAAUUCAGGUG	TTATTTGTATAATATATAT	8
hsa-miR-26a	41	8:1:0	-8.38	-11	2.62	UUCAAGUAAUCCAGGAUAGGCU	TTACTTGTTTATTTTTTT	8
hsa-miR-1277	474	8:1:1	-9.7	-12.34	2.64	AAAUAUAUAUAUAUAUGUACGUAU	ATATATGTTTTATTTTGT	8
hsa-miR-548b-5p	405	8:1:1	-4.1	-6.74	2.64	AAAAGUAAUUGUGGUUUUGGCC	TTTATTTTGTTTCTAT	8
hsa-miR-548p	330	8:1:1	-1.62	-4.26	2.64	UAGCAAAAACUGCAGUUACUUU	TTTTTTTTTAAATG	8
hsa-miR-137	1280	8:1:1	-7.8	-10.45	2.65	UUAUUGCUUAAGAAUACGCGUAG	ATGTAATAGTA	8
hsa-miR-381	1097	8:1:1	-7.7	-10.35	2.65	AGCGAGGUUGCCCUUUGUAUAU	TATTGTATAATA	8
hsa-miR-337-3p	785	8:1:1	-5.5	-8.17	2.67	CUCCUAUAUGAUGCCUUUCUUC	ATATACGGATATATCAA	8
hsa-miR-520c-3p	1004	8:1:1	-8.2	-10.89	2.69	AAAGUGCUUCCUUUUAGAGGGU	AAGAATTTTTTTTAACGC	8
hsa-miR-491-3p	564	8:1:1	-9.5	-12.19	2.69	CUUAUGCAAGAUUCCCUUCUAC	TTGTATATATAAATTTGT	8
hsa-miR-191	398	8:1:1	-4.7	-7.39	2.69	CAACGGAAUCCCAAAAGCAGCUG	AATTCGTTTTATTTT	8
hsa-miR-367	868	8:1:1	-4.5	-7.2	2.7	ACUGUUGCUAAUAUGCAACUCU	AATGTAATAT	8
hsa-miR-576-5p	608	8:1:0	-6.6	-9.32	2.72	AUUCUAAUUUCUCCACGUCUUU	AAATAGAAAAAAAAAGAA	8
hsa-miR-655	822	8:1:0	-4.4	-7.13	2.73	AGAGGUUAUCCGUGUUAUGUUC	ATGAATTAATCTAATCTTA	8
hsa-miR-1259	782	8:1:0	-5.7	-8.44	2.74	UCCCUGAGACCCUUUAACCUGUGA	ACCATATACGGA	8
hsa-miR-576-3p	501	8:1:1	-9.1	-11.84	2.74	AAGAUGUGGAAAAAUUGGAAUC	ACACATTTGATTATT	8

hsa-miR-548b-5p	35	8:0:1	-5.7	-8.44	2.74	AAAAGUAAUUGUGGUUUUGGCC	ATTATTTTACT	8
hsa-miR-548h	35	8:0:1	-5.7	-8.44	2.74	AAAAGUAAUCGCGGUUUUUGUC	ATTATTTTACT	8
hsa-miR-641	549	8:1:1	-7.1	-9.87	2.77	AAAGACAUAGGAUAGAGUCACCUC	TTTGTTTTTG	8
hsa-miR-300	94	8:1:1	-6.8	-9.57	2.77	UAUACAAGGGCAGACUCUCUCU	ATTTGTATAATATAAATA	8
hsa-miR-651	828	8:1:1	-3.1	-5.88	2.78	UUUAGGAUAAGCUUGACUUUUG	TAATCTAATCTTACA	8
hsa-miR-337-5p	398	8:1:1	-4.61	-7.39	2.78	GAACGGCUUCAUACAGGAGUU	AATTCGTTTTATTTT	8
hsa-miR-548p	329	8:1:1	-1.22	-4	2.78	UAGCAAAAACUGCAGUUACUUU	TTTTTTTTTTAAATGTC	8
hsa-miR-382	1004	8:1:1	-8.1	-10.89	2.79	GAAGUUGUUCGUGGUGGAUUCG	AAGAATTTTTTTTT	8
hsa-miR-374b	728	8:1:0	-5	-7.79	2.79	AUAUAAUACAACCUGCUAAGUG	TTATTATATATA	8
hsa-miR-25	868	8:1:1	-4.4	-7.2	2.8	AGGCGGAGACUUGGGCAAUUG	AATGTAATAT	8
hsa-miR-561	982	8:1:1	-5.5	-8.31	2.81	AUCAAGGAUCUUAAACUUUGCC	TAAATTCTTTT	8
hsa-miR-524-5p	210	8:1:1	-7.2	-10.01	2.81	CUACAAAGGGAAGCACUUUCUC	TATTTGTAAATATATAT	8
hsa-miR-568	177	8:1:1	-10.9	-13.71	2.81	AUGUAUAAAUGUAUACACAC	TATATATAATTTG	8
hsa-miR-1270	87	8:1:1	-7.7	-10.51	2.81	CUGGAGAUAUGGAAGAGCUGUGU	TATATTCATTT	8
hsa-miR-524-5p	435	8:1:1	-7	-9.83	2.83	CUACAAAGGGAAGCACUUUCUC	TATTTGTATATAT	8
hsa-miR-380	729	8:0:1	-5.1	-7.94	2.84	UGGUUGACCAUAGAACAUGCGC	TATTATATAT	8
hsa-miR-655	1273	8:0:0	-7.2	-10.05	2.85	AGAGGUUAUCCGUGUUAUGUUC	ATGTATTATGTAATAGT	8

hsa-miR-374b	675	8:1:0	-9.2	-12.05	2.85	AUAUAAUACAACCUGCUAAGUG	ATATTATATGGTTC	8
hsa-miR-424	411	8:1:1	-4.7	-7.55	2.85	CAGCAGCAAUUCAUGUUUUGAA	TTGTTTCTATTTGA	8
hsa-miR-154	1193	8:1:1	-6.6	-9.46	2.86	UAGGUUAUCCGUGUUGCCUUCG	CATAACTTCTATTTTAAGA	8
hsa-miR-568	950	8:1:0	-4.6	-7.46	2.86	AUGUAUAAAUGUAUACACAC	TTCATACATACT	8
hsa-miR-590-5p	842	8:1:1	-5.1	-7.96	2.86	GAGCUUAUUCAUAAAAGUGCAG	AATAAGTATTATT	8
hsa-miR-876-5p	395	8:1:1	-4.6	-7.47	2.87	UGGAUUUCUUUGUGAAUCACCA	ATAAATTCGT	8
hsa-miR-375	1031	8:1:1	-8.26	-11.14	2.88	UUUGUUCGUUCGGCUCGCGUGA	ACGTATAAAATAAAA	8
hsa-miR-374a	728	8:1:0	-4.9	-7.79	2.89	UUAUAAUACAACCUGAUAAGUG	TTATTATATATATA	8
hsa-miR-181c	1269	8:1:0	-8.5	-11.4	2.9	AACAUUCAACCUGUCGGUGAGU	TAGAATGTATTATGTAA	8
hsa-miR-1261	1061	8:1:1	-5.7	-8.6	2.9	AUGGAUAAGGCUUUGGCUU	TTTTTCCATAGTATATAT	8
hsa-miR-568	736	8:1:1	-5.8	-8.71	2.91	AUGUAUAAAUGUAUACACAC	TATATATATATAT	8
hsa-miR-888	323	8:1:0	-4.8	-7.71	2.91	UACUCAAAAAGCUGUCAGUCA	TTTTGATTTTT	8
hsa-miR-641	451	8:1:1	-8.2	-11.12	2.92	AAAGACAUAGGAUAGAGUCACCUC	TTTGTTTTATTTGT	8
hsa-miR-548i	658	8:1:1	-3.6	-6.56	2.96	AAAAGUAAUUGCGGAUUUUGCC	ATTATATTCATAGT	8
hsa-miR-302f	1128	8:1:1	-9.5	-12.47	2.97	UAAUUGCUUCCAUGUUU	TAGTAATTAATT	8
hsa-miR-548b-5p	353	8:1:1	-2.4	-5.37	2.97	AAAAGUAAUUGUGGUUUUGGCC	ATAATTTTAATATATAA	8
hsa-miR-548a-5p	353	8:1:1	-2.4	-5.37	2.97	AAAAGUAAUUGCGAGUUUUACC	ATAATTTTAATAT	8

hsa-miR-301b	38	8:1:1	-7	-9.97	2.97	GCUCUGACGAGGUUGCACUACU	ATTTTACTTGTT	8
hsa-miR-154	918	8:1:0	-8.3	-11.29	2.99	UAGGUUAUCCGUGUUGCCUUCG	GATATCCTTTTTTTT	8
hsa-miR-656	365	8:1:0	-1.7	-4.7	3	AGGUUGCCUGUGAGGUGUUCA	TATAATTTTTATTTTG	8
hsa-miR-656	358	8:1:0	-2.4	-5.4	3	AGGUUGCCUGUGAGGUGUUCA	TTTAATATATAA	8
hsa-miR-577	329	8:1:1	-1	-4	3	UAGAUAAAAUAUUGGUACCUG	TTTTTTTTTT	8
hsa-miR-323-3p	870	8:1:0	-5.5	-8.51	3.01	AGGUGGUCCGUGGCGCGUUCGC	TGTAATATATC	8
hsa-miR-656	464	8:0:0	-9.7	-12.71	3.01	AGGUUGCCUGUGAGGUGUUCA	TATAATATATATATAT	8
hsa-miR-496	709	8:1:0	-8.4	-11.42	3.02	UGAGUAUUACAUGGCCAAUCUC	TAATACACTAAATA	8
hsa-miR-590-5p	697	8:0:0	-10.3	-13.36	3.06	GAGCUUAUUCAUAAAAGUGCAG	AATAAGCTAATA	8
hsa-miR-548i	328	8:1:1	-1	-4.06	3.06	AAAAGUAAUUGCGGAUUUUGCC	ATTTTTTTTTTAAAT	8
hsa-miR-548l	296	8:0:1	-5	-8.06	3.06	AAAAGUAUUUGCGGGUUUUGUC	AATATTTTTATTTTT	8
hsa-miR-1	114	8:1:1	-8	-11.06	3.06	ACAUACUUCUUUAUAUGCCCAU	TATATTCATAGTATATA	8
hsa-miR-206	114	8:1:1	-8	-11.06	3.06	UGGAAUGUAAGGAAGUGUGUGG	TATATTCATA	8
hsa-miR-30d	771	8:1:0	-5	-8.07	3.07	UGUAAACAUCCCCGACUGGAAG	ATGTTTTCAA	8
hsa-miR-548c-5p	353	8:1:1	-2.3	-5.37	3.07	AAAAGUAAUUGCGGUUUUUGCC	ATAATTTTAAT	8
hsa-miR-548j	353	8:1:1	-2.3	-5.37	3.07	AAAAGUAAUUGCGGUCUUUGGU	ATAATTTTAATA	8
hsa-miR-301a	38	8:1:1	-6.9	-9.97	3.07	GCUCUGACUUUAUUGCACUACU	ATTTTACTTGTTT	8

hsa-miR-579	1046	8:1:1	-7.1	-10.18	3.08	UCGCGGUUUGUGCCAGAUGACG	ATAAATGATT	8
hsa-miR-568	359	8:1:1	-2.5	-5.59	3.09	AUGUAUAAAUGUAUACACAC	TTAATATATAA	8
hsa-miR-186	305	8:1:1	-1.4	-4.49	3.09	CAAAGAAUUCUCCUUUUGGGCU	ATTTTTTTTTCATT	8
hsa-miR-26a	1247	8:1:1	-6	-9.11	3.11	UUCAAGUAAUCCAGGAUAGGCU	ATATTTGAATAAC	8
hsa-miR-30e	1236	8:1:1	-5.37	-8.48	3.11	UGUAAACAUCCUUGACUGGAAG	ATGATTATAATATATTTG	8
hsa-miR-137	869	8:1:1	-5.05	-8.18	3.13	UUAUUGCUUAAGAAUACGCGUAG	ATGTAATATATCATATA	8
hsa-miR-548c-5p	302	8:1:1	-3.1	-6.25	3.15	AAAAGUAAUUGCGGUUUUUGCC	TTTATTTTTTTTTCATTT	8
hsa-miR-548j	302	8:1:1	-3.1	-6.25	3.15	AAAAGUAAUUGCGGUCUUUGGU	TTTATTTTTTTTTC	8
hsa-miR-381	436	8:1:1	-6.8	-9.96	3.16	AGCGAGGUUGCCCUUUGUAUAU	ATTTGTATATATA	8
hsa-miR-548a-5p	328	8:1:1	-0.9	-4.06	3.16	AAAAGUAAUUGCGAGUUUUACC	ATTTTTTTTTTAAATGTCA	8
hsa-miR-381	94	8:1:1	-6.4	-9.57	3.17	AGCGAGGUUGCCCUUUGUAUAU	ATTTGTATAAT	8
hsa-miR-607	1248	8:1:0	-7.5	-10.69	3.19	GUUCAAAUCCAGAUCUAUAAC	TATTTGAATAACAT	8
hsa-miR-548g	365	8:1:1	-1.5	-4.7	3.2	UGCAAAAGUAAUUGCAGUUUUUG	TATAATTTTTA	8
hsa-miR-429	667	8:1:1	-6.66	-9.87	3.21	UAAUACUGUCUGGUAAAACCGU	ATAGTAGTATAT	8
hsa-miR-892a	500	8:1:0	-8.6	-11.81	3.21	CACUGUGUCCUUUCUGCGUAG	GACACATTTGAT	8
hsa-miR-1279	1275	8:1:1	-6.9	-10.12	3.22	UCAUAUUGCUUCUUUCU	GTATTATGTAATAGTA	8
hsa-miR-655	845	8:1:0	-5	-8.24	3.24	AGAGGUUAUCCGUGUUAUGUUC	AAGTATTATTATAAAAT	8

hsa-miR-190	873	8:0:1	-7.6	-10.85	3.25	UGAUAUGUUUGAUAUAUUAGGU	AATATATCAT	8
hsa-miR-548n	434	8:1:1	-6.2	-9.45	3.25	CAAAAGUAAUUGUGGAUUUUGU	TTATTTGTAT	8
hsa-miR-548n	326	8:1:1	-2.5	-5.75	3.25	CAAAAGUAAUUGUGGAUUUUGU	TGATTTTTTTT	8
hsa-miR-155	848	8:1:1	-4.6	-7.86	3.26	UUAAUGCUAAUCGUGAUAGGGGU	TATTATTATAAAATA	8
hsa-miR-30b	299	8:1:1	-4.54	-7.8	3.26	UGUAAACAUCCUACACUCAGCU	ATTTTTATTTTTTTTTCA	8
hsa-miR-448	93	8:1:1	-7.1	-10.36	3.26	UUGCAUAUGUAGGAUGUCCCAU	CATTTGTATAATATAAA	8
hsa-miR-500	844	8:1:1	-5	-8.27	3.27	UAAUCCUUGCUACCUGGGUGAGA	TAAGTATTATTA	8
hsa-miR-548b-5p	318	8:1:1	-4.4	-7.68	3.28	AAAAGUAAUUGUGGUUUUGGCC	TTTATTTTTGATTTT	8
hsa-miR-548d-5p	318	8:1:1	-4.4	-7.68	3.28	AAAAGUAAUUGUGGUUUUUGCC	TTTATTTTTG	8
hsa-miR-520b	1004	8:1:1	-7.6	-10.89	3.29	AAAGUGCUUCCUUUUAGAGGG	AAGAATTTTTTTTTAACGC	8
hsa-miR-1324	416	8:1:1	-5.85	-9.17	3.32	CCAGACAGAAUUCUAUGCACUUUC	TCTATTTGACACATG	8
hsa-miR-383	141	8:1:1	-8.21	-11.54	3.33	AGAUCAGAAGGUGAUUGUGGCU	TTTTGATATATTTGTAT	8
hsa-miR-577	1199	8:1:1	-5.22	-8.56	3.34	UAGAUAAAAUAUUGGUACCUG	TTCTATTTTAAGAAAT	8
hsa-miR-200b	1127	8:1:1	-9.5	-12.84	3.34	CAUCUUACUGGGCAGCAUUGGA	GTAGTAATTAATT	8
hsa-miR-656	1276	8:1:1	-7.4	-10.75	3.35	AGGUUGCCUGUGAGGUGUUCA	TATTATGTAATAGTA	8
hsa-miR-1264	295	8:1:1	-4.9	-8.25	3.35	CAAGUCUUAUUUGAGCACCUGUU	TAATATTTTT	8
hsa-miR-609	1187	8:1:1	-7.8	-11.18	3.38	AGGGUGUUUCUCUCAUCUCU	AAATACCATAAC	8

hsa-miR-577	317	8:0:1	-4.3	-7.68	3.38	UAGAUAAAAUAUUGGUACCUG	TTTTATTTTTGAT	8
hsa-miR-548d-5p	305	8:1:1	-1.1	-4.49	3.39	AAAAGUAAUUGUGGUUUUUGCC	ATTTTTTTTT	8
hsa-miR-26b	1247	8:1:1	-5.7	-9.11	3.41	UUCAAGUAAUUCAGGAUAGGU	ATATTTGAATAACA	8
hsa-miR-1297	1247	8:1:1	-5.7	-9.11	3.41	UUCAAGUAAUUCAGGUG	ATATTTGAATAACA	8
hsa-miR-1284	438	8:1:1	-6.7	-10.12	3.42	UCUAUACAGACCCUGGCUUUUC	TTGTATATATAAATTT	8
hsa-miR-507	1181	8:1:1	-8.4	-11.83	3.43	UUUUGCACCUUUUGGAGUGAA	GTTGTAAAATACC	8
hsa-miR-190b	388	8:1:1	-3.95	-7.39	3.44	UGAUAUGUUUGAUAUUGGGUU	AATATATATAAATT	8
hsa-miR-1246	267	8:1:1	-1.49	-4.94	3.45	AAUGGAUUUUUGGAGCAGG	AAATATATTC	8
hsa-miR-1259	658	8:1:1	-3.1	-6.56	3.46	UCCCUGAGACCCUUUAACCUGUGA	ATTATATTCATAGTA	8
hsa-miR-548h	353	8:1:1	-1.9	-5.37	3.47	AAAAGUAAUCGCGGUUUUUGUC	ATAATTTTAATATAT	8
hsa-miR-548i	353	8:1:1	-1.9	-5.37	3.47	AAAAGUAAUUGCGGAUUUUGCC	ATAATTTTAATATATAA	8
hsa-miR-1277	596	8:1:1	-8.45	-11.93	3.48	AAAUAUAUAUAUAUAUGUACGUAU	ATGTATGTGATTAAAT	8
hsa-miR-590-3p	572	8:1:0	-7.1	-10.58	3.48	UAAUUUUAUGUAUAAGCUAGU	ATAAATTTGTTTTATTTG	8
hsa-miR-548f	366	8:1:1	-1.1	-4.59	3.49	UGCAAAAGUAAUCACAGUUUUU	ATAATTTTTATTTTGTTT	8
hsa-miR-181a	1269	8:1:0	-7.9	-11.4	3.5	AACAUUCAACGCUGUCGGUGAGU	TAGAATGTATT	8
hsa-miR-876-5p	995	8:1:1	-6.8	-10.3	3.5	UGGAUUUCUUUGUGAAUCACCA	AGAAATATGAAGA	8
hsa-miR-1323	320	8:1:1	-4.2	-7.7	3.5	UCAAAACUGAGGGGCAUUUUCU	TATTTTTGATTTTT	8

hsa-miR-26b	41	8:1:0	-7.5	-11	3.5	UUCAAGUAAUUCAGGAUAGGU	TTACTTGTTTATTTTTTT	8
hsa-miR-876-5p	981	8:1:1	-4.95	-8.47	3.52	UGGAUUUCUUUGUGAAUCACCA	ATAAATTCTTTTTAA	8
hsa-miR-548e	400	8:1:1	-2.8	-6.33	3.53	CAAAAGCAAUCGCGGUUUUUGC	TTCGTTTTATTTTGTTTCT	8
hsa-miR-137	845	8:1:1	-4.7	-8.24	3.54	UUAUUGCUUAAGAAUACGCGUAG	AAGTATTATT	8
hsa-miR-374b	850	8:1:0	-4.2	-7.75	3.55	AUAUAAUACAACCUGCUAAGUG	TTATTATAAA	8
hsa-miR-522	719	8:1:1	-2.86	-6.41	3.55	CUCUAGAGGGAAGCGCUUUCUG	AAATATTTCTTATTATA	8
hsa-miR-137	994	8:1:0	-6.6	-10.16	3.56	UUAUUGCUUAAGAAUACGCGUAG	AAGAAATATGAAGAATTT	8
hsa-miR-944	886	8:0:1	-8.5	-12.06	3.56	AAAUUAUUGUACAUCGGAUGAG	TAATAATTGTATTG	8
hsa-miR-568	471	8:1:1	-8.6	-12.16	3.56	AUGUAUAAAUGUAUACACAC	TATATATATGTT	8
hsa-miR-491-3p	1064	8:1:1	-3.2	-6.77	3.57	CUUAUGCAAGAUUCCCUUCUAC	TTCCATAGTATATATAATG	8
hsa-miR-548d-5p	353	8:1:1	-1.8	-5.37	3.57	AAAAGUAAUUGUGGUUUUUGCC	ATAATTTTAA	8
hsa-miR-130b	38	8:1:1	-6.4	-9.97	3.57	ACUCUUUCCCUGUUGCACUAC	ATTTTACTTGTTTATTTTT	8
hsa-miR-374a	589	8:1:0	-8.2	-11.78	3.58	UUAUAAUACAACCUGAUAAGUG	GTATAATATGTATGTGATT	8
hsa-miR-548h	849	8:1:1	-4.2	-7.79	3.59	AAAAGUAAUCGCGGUUUUUGUC	ATTATTATAAAAT	8
hsa-miR-1253	725	8:1:1	-4	-7.59	3.59	AGAGAAGAAGAUCAGCCUGCA	TTCTTATTATATA	8
hsa-miR-491-3p	152	8:0:1	-7.1	-10.7	3.6	CUUAUGCAAGAUUCCCUUCUAC	TTGTATAATAAT	8
hsa-miR-32	291	8:1:1	-4.9	-8.51	3.61	UAUUGCACAUUACUAAGUUGCA	TGAGTAATAT	8

hsa-miR-487a	913	8:1:0	-7.82	-11.45	3.63	GUGGUUAUCCCUGCUGUGUUCG	TATATGATATCC	8
hsa-miR-559	306	8:1:1	-1	-4.63	3.63	UAAAGUAAAUAUGCACCAAAA	TTTTTTTTTCATTTTAT	8
hsa-miR-656	293	8:1:1	-4.8	-8.43	3.63	AGGUUGCCUGUGAGGUGUUCA	AGTAATATTT	8
hsa-miR-448	1122	8:1:1	-7.3	-10.94	3.64	UUGCAUAUGUAGGAUGUCCCAU	AATATGTAGTA	8
hsa-miR-548p	249	8:1:1	-8.3	-11.94	3.64	UAGCAAAAACUGCAGUUACUUU	TTTTTGTATAATATTTCT	8
hsa-miR-655	1280	8:1:0	-6.8	-10.45	3.65	AGAGGUUAUCCGUGUUAUGUUC	ATGTAATAGTA	8
hsa-miR-590-3p	1035	8:1:0	-7.7	-11.36	3.66	UAAUUUUAUGUAUAAGCUAGU	ATAAAATAAAAATAAAT	8
hsa-miR-876-5p	1209	8:1:1	-5.3	-8.97	3.67	UGGAUUUCUUUGUGAAUCACCA	AGAAATATATATATA	8
hsa-miR-576-3p	1192	8:1:1	-6.2	-9.87	3.67	AAGAUGUGGAAAAAUUGGAAUC	CCATAACTTCTATTTTAA	8
hsa-miR-487b	153	8:1:1	-6.6	-10.28	3.68	GUGGUUAUCCCUGUCCUGUUCG	TGTATAATAA	8
hsa-miR-544	1001	8:1:0	-6.3	-10	3.7	AUUCUGCAUUUUUAGCAAGUUC	ATGAAGAATTTTTTTTT	8
hsa-miR-656	205	8:0:0	-8.5	-12.2	3.7	AGGUUGCCUGUGAGGUGUUCA	TATAATATTTGTAAATATA	8
hsa-miR-559	1095	8:1:1	-6.3	-10.01	3.71	UAAAGUAAAUAUGCACCAAAA	TTTATTGTATAATA	8
hsa-miR-656	870	8:0:1	-4.8	-8.51	3.71	AGGUUGCCUGUGAGGUGUUCA	TGTAATATATCATATATAA	8
hsa-miR-26a	457	8:1:1	-7.8	-11.51	3.71	UUCAAGUAAUCCAGGAUAGGCU	TTATTTGTAT	8
hsa-miR-656	807	8:0:0	-4.7	-8.42	3.72	AGGUUGCCUGUGAGGUGUUCA	TATAATATAATAATAAT	8
hsa-miR-361-5p	357	8:1:1	-1.6	-5.32	3.72	UUAUCAGAAUCUCCAGGGGUAC	TTTTAATATATA	8

hsa-miR-26a	511	8:1:1	-11.1	-14.84	3.74	UUCAAGUAAUCCAGGAUAGGCU	TTATTTGTATAT	8
hsa-miR-374a	850	8:1:0	-4	-7.75	3.75	UUAUAAUACAACCUGAUAAGUG	TTATTATAAA	8
hsa-miR-1297	417	8:1:1	-5.6	-9.35	3.75	UUCAAGUAAUUCAGGUG	CTATTTGACACATG	8
hsa-miR-300	459	8:1:1	-7.7	-11.46	3.76	UAUACAAGGGCAGACUCUCUCU	ATTTGTATAA	8
hsa-miR-1179	768	8:1:1	-4.7	-8.47	3.77	AAGCAUUCUUUCAUUGGUUGG	AAAATGTTTTC	8
hsa-miR-143	91	8:1:1	-7.3	-11.07	3.77	GGUGCAGUGCUGCAUCUCUGGU	TTCATTTGTA	8
hsa-miR-380	360	8:1:1	-1.7	-5.48	3.78	UGGUUGACCAUAGAACAUGCGC	TAATATATAA	8
hsa-miR-568	173	8:1:1	-8.1	-11.88	3.78	AUGUAUAAAUGUAUACACAC	TATATATATA	8
hsa-miR-302d	1004	8:1:1	-7.1	-10.89	3.79	ACUUUAACAUGGAGGCACUUGC	AAGAATTTTTTTTAA	8
hsa-miR-548j	849	8:1:1	-4	-7.79	3.79	AAAAGUAAUUGCGGUCUUUGGU	ATTATTATAAAATA	8
hsa-miR-1259	388	8:1:1	-3.6	-7.39	3.79	UCCCUGAGACCCUUUAACCUGUGA	AATATATATA	8
hsa-miR-1238	1001	8:1:1	-6.2	-10	3.8	GUGAGUGGGAGCCCCAGUGUGUG	ATGAAGAATTTTTTT	8
hsa-miR-641	770	8:1:1	-4.5	-8.3	3.8	AAAGACAUAGGAUAGAGUCACCUC	AATGTTTTCAACAC	8
hsa-miR-491-3p	438	8:1:1	-6.3	-10.12	3.82	CUUAUGCAAGAUUCCCUUCUAC	TTGTATATATAAATTTG	8
hsa-miR-508-5p	288	8:1:1	-6.14	-9.96	3.82	UACUCCAGAGGGCGUCACUCAUG	TTTTGAGTAATATTTTTAT	8
hsa-miR-577	34	8:1:1	-4.1	-7.92	3.82	UAGAUAAAAUAUUGGUACCUG	TATTATTTTACTTG	8
hsa-miR-548a-3p	1057	8:1:1	-5.2	-9.03	3.83	CAAAACUGGCAAUUACUUUUGC	CTTGTTTTTCCATAGT	8

hsa-miR-802	550	8:1:1	-6	-9.84	3.84	CAGUAACAAAGAUUCAUCCUUGU	TTGTTTTTGGATATTTGTA	8
hsa-miR-548d-5p	405	8:1:1	-2.9	-6.74	3.84	AAAAGUAAUUGUGGUUUUUGCC	TTTATTTTGTTTCTA	8
hsa-miR-548p	322	8:1:0	-3.89	-7.73	3.84	UAGCAAAAACUGCAGUUACUUU	TTTTTGATTTT	8
hsa-miR-664	1048	8:1:0	-5.97	-9.82	3.85	ACUGGCUAGGGAAAAUGAUUGGAU	AAATGATTCCTTGTTTTTC	8
hsa-miR-708	1019	8:1:1	-8.3	-12.15	3.85	AAGGAGCUUACAAUCUAGCUGGG	ACGCTTCTAATTAC	8
hsa-miR-548b-5p	328	8:1:1	-0.2	-4.06	3.86	AAAAGUAAUUGUGGUUUUGGCC	ATTTTTTTTTTAA	8
hsa-miR-548c-5p	328	8:1:1	-0.2	-4.06	3.86	AAAAGUAAUUGCGGUUUUUGCC	ATTTTTTTTTTAA	8
hsa-miR-548d-5p	328	8:1:1	-0.2	-4.06	3.86	AAAAGUAAUUGUGGUUUUUGCC	ATTTTTTTTTTA	8
hsa-miR-548j	328	8:1:1	-0.2	-4.06	3.86	AAAAGUAAUUGCGGUCUUUGGU	ATTTTTTTTTTAAATG	8
hsa-miR-548m	353	8:1:1	-1.5	-5.37	3.87	CAAAGGUAUUUGUGGUUUUUG	ATAATTTTAA	8
hsa-miR-1272	1239	8:1:1	-4.8	-8.68	3.88	GAUGAUGAUGGCAGCAAAUUCUGAAA	ATTATAATATATTT	8
hsa-miR-380	851	8:1:1	-3.8	-7.68	3.88	UGGUUGACCAUAGAACAUGCGC	TATTATAAAATAG	8
hsa-miR-548a-5p	35	8:0:1	-4.56	-8.44	3.88	AAAAGUAAUUGCGAGUUUUACC	ATTATTTTACTTGTTTATT	8
hsa-miR-376a	1232	8:1:1	-4.6	-8.49	3.89	GUAGAUUCUCCUUCUAUGAGUA	CGTTATGATTATAATAT	8
hsa-miR-376b	1232	8:1:1	-4.6	-8.49	3.89	CGUGGAUAUUCCUUCUAUGUUU	CGTTATGATTATAATAT	8
hsa-miR-1297	583	8:1:1	-8.9	-12.79	3.89	UUCAAGUAAUUCAGGUG	TTATTTGTATAA	8
hsa-miR-641	477	8:0:1	-7.4	-11.29	3.89	AAAGACAUAGGAUAGAGUCACCUC	TATGTTTTATTT	8

hsa-miR-548p	300	8:1:1	-3.6	-7.5	3.9	UAGCAAAAACUGCAGUUACUUU	TTTTTATTTTTTTTTCAT	8
hsa-miR-1279	871	8:1:1	-5.5	-9.41	3.91	UCAUAUUGCUUCUUUCU	GTAATATATCAT	8
hsa-miR-1246	661	8:1:1	-4.72	-8.63	3.91	AAUGGAUUUUUGGAGCAGG	ATATTCATAG	8
hsa-miR-26b	457	8:1:1	-7.6	-11.51	3.91	UUCAAGUAAUUCAGGAUAGGU	TTATTTGTATA	8
hsa-miR-620	87	8:1:1	-6.6	-10.51	3.91	AUGGAGAUAGAUAUAGAAAU	TATATTCATTTGT	8
hsa-miR-548n	325	8:1:1	-2.4	-6.33	3.93	CAAAAGUAAUUGUGGAUUUUGU	TTGATTTTTTTTTT	8
hsa-miR-548i	35	8:0:1	-4.5	-8.44	3.94	AAAAGUAAUUGCGGAUUUUGCC	ATTATTTTAC	8
hsa-miR-382	980	8:1:1	-4.8	-8.75	3.95	GAAGUUGUUCGUGGUGGAUUCG	AATAAATTCTTTTTAAGAA	8
hsa-miR-770-5p	893	8:1:1	-8.8	-12.78	3.98	UCCAGUACCACGUGUCAGGGCCA	TGTATTGGAA	8
hsa-miR-1259	1212	8:1:1	-3.8	-7.79	3.99	UCCCUGAGACCCUUUAACCUGUGA	AATATATATATAATAATA	8
hsa-miR-208a	987	8:1:1	-4.84	-8.83	3.99	GAGCUUUUGGCCCGGGUUAUAC	TCTTTTTAAGAAAT	8
hsa-miR-374a	254	8:1:0	-6.6	-10.59	3.99	UUAUAAUACAACCUGAUAAGUG	GTATAATATTTCTAAA	8
hsa-miR-1299	685	8:1:1	-10.1	-14.1	4	UUCUGGAAUUCUGUGUGAGGGA	GTTCCATATTAAAATA	8
hsa-miR-190b	218	8:1:1	-7.3	-11.3	4	UGAUAUGUUUGAUAUUGGGUU	AATATATATATATATAT	8
hsa-miR-26b	286	8:1:1	-5.9	-9.92	4.02	UUCAAGUAAUUCAGGAUAGGU	TTTTTTGAGT	8
hsa-miR-105	510	8:1:1	-10.9	-14.93	4.03	UCAAAUGCUCAGACUCCUGUGGU	ATTATTTGTATATATAT	8
hsa-miR-548c-5p	305	8:1:1	-0.46	-4.49	4.03	AAAAGUAAUUGCGGUUUUUGCC	ATTTTTTTTTC	8

hsa-miR-491-3p	252	8:0:1	-8.1	-12.13	4.03	CUUAUGCAAGAUUCCCUUCUAC	TTGTATAATATTTCTA	8
hsa-miR-32	1279	8:1:1	-6.7	-10.74	4.04	UAUUGCACAUUACUAAGUUGCA	TATGTAATAGTA	8
hsa-miR-524-5p	458	8:1:1	-7.2	-11.24	4.04	CUACAAAGGGAAGCACUUUCUC	TATTTGTATAATATAT	8
hsa-miR-200b	294	8:1:1	-4.3	-8.34	4.04	CAUCUUACUGGGCAGCAUUGGA	GTAATATTTTTAT	8
hsa-miR-568	212	8:1:1	-5.9	-9.94	4.04	AUGUAUAAAUGUAUACACAC	TTTGTAAATAT	8
hsa-miR-361-5p	141	8:0:1	-7.5	-11.54	4.04	UUAUCAGAAUCUCCAGGGGUAC	TTTTGATATATTTGTATAA	8
hsa-miR-590-3p	231	8:1:0	-8.21	-12.26	4.05	UAAUUUUAUGUAUAAGCUAGU	ATATAATTTGTTTTTTTG	8
hsa-miR-656	1220	8:1:0	-3.8	-7.86	4.06	AGGUUGCCUGUGAGGUGUUCA	TATAATAATACA	8
hsa-miR-373	1004	8:1:1	-6.83	-10.89	4.06	ACUCAAAAUGGGGGCGCUUUCC	AAGAATTTTT	8
hsa-miR-548a-5p	658	8:1:1	-2.5	-6.56	4.06	AAAAGUAAUUGCGAGUUUUACC	ATTATATTCATAGTAGTAT	8
hsa-miR-548p	370	8:1:1	-1.3	-5.36	4.06	UAGCAAAAACUGCAGUUACUUU	TTTTTATTTTGTTTGTAT	8
hsa-miR-577	330	8:1:1	-0.2	-4.26	4.06	UAGAUAAAAUAUUGGUACCUG	TTTTTTTTTAAATGTCATT	8
hsa-miR-203	803	8:1:1	-3.6	-7.67	4.07	AGUGGUUCUUAACAGUUCAACAGUU	ACATTATAATATAATAATA	8
hsa-miR-1290	395	8:1:1	-3.4	-7.47	4.07	UGGAUUUUUGGAUCAGGGA	ATAAATTCGTTTTAT	8
hsa-miR-374b	384	8:1:0	-5.4	-9.47	4.07	AUAUAAUACAACCUGCUAAGUG	GTATAATATA	8
hsa-miR-664	824	8:1:1	-1.3	-5.38	4.08	ACUGGCUAGGGAAAAUGAUUGGAU	GAATTAATCT	8
hsa-miR-568	730	8:1:1	-3.9	-7.98	4.08	AUGUAUAAAUGUAUACACAC	ATTATATATA	8

hsa-miR-487a	153	8:1:0	-6.2	-10.28	4.08	GUGGUUAUCCCUGCUGUGUUCG	TGTATAATAATAT	8
hsa-miR-410	729	8:1:0	-3.85	-7.94	4.09	AGGUUGUCUGUGAUGAGUUCG	TATTATATATATATAT	8
hsa-miR-1284	564	8:1:1	-8.1	-12.19	4.09	UCUAUACAGACCCUGGCUUUUC	TTGTATATATAAA	8
hsa-miR-561	655	8:1:1	-2.1	-6.2	4.1	AUCAAGGAUCUUAAACUUUGCC	TAAATTATATTCATA	8
hsa-miR-487a	383	8:1:0	-5.2	-9.3	4.1	GUGGUUAUCCCUGCUGUGUUCG	TGTATAATAT	8
hsa-miR-548n	331	8:1:1	-0.2	-4.3	4.1	CAAAAGUAAUUGUGGAUUUUGU	TTTTTTTTAAA	8
hsa-miR-559	329	8:1:1	0.1	-4	4.1	UAAAGUAAAUAUGCACCAAAA	TTTTTTTTTTAAATGTCAT	8
hsa-miR-1259	1068	8:1:1	-4.9	-9.01	4.11	UCCCUGAGACCCUUUAACCUGUGA	ATAGTATATATAAT	8
hsa-miR-487a	588	8:1:0	-7.76	-11.87	4.11	GUGGUUAUCCCUGCUGUGUUCG	TGTATAATATGTATGT	8
hsa-miR-590-3p	446	8:1:0	-7	-11.11	4.11	UAAUUUUAUGUAUAAGCUAGU	ATAAATTTGTTTTATTT	8
hsa-miR-548l	1244	8:1:1	-4	-8.13	4.13	AAAAGUAUUUGCGGGUUUUGUC	AATATATTTGAATAACATA	8
hsa-miR-652	684	8:1:1	-10	-14.14	4.14	CAACCCUAGGAGAGGGUGCCAUUC[illegible]	GGTTCCATATTAAAAT	8
hsa-miR-1297	511	8:1:1	-10.7	-14.84	4.14	UUCAAGUAAUUCAGGUG	TTATTTGTATATATA	8
hsa-miR-450b-5p	212	8:1:1	-5.8	-9.94	4.14	UUUUGCAAUAUGUUCCUGAAUA	TTTGTAAATATAT	8
hsa-miR-548h	302	8:1:1	-2.1	-6.25	4.15	AAAAGUAAUCGCGGUUUUUGUC	TTTATTTTTTTTT	8
hsa-miR-196a	1027	8:1:1	-7.6	-11.76	4.16	UAGGUAGUUUCAUGUUGUUGGG	AATTACGTATAAAATAA	8
hsa-miR-196b	1027	8:1:1	-7.6	-11.76	4.16	UAGGUAGUUUCCUGUUGUUGGG	AATTACGTATAAAATAA	8

hsa-miR-381	459	8:1:1	-7.3	-11.46	4.16	AGCGAGGUUGCCCUUUGUAUAU	ATTTGTATAAT	8
hsa-miR-300	436	8:1:1	-5.8	-9.96	4.16	UAUACAAGGGCAGACUCUCUCU	ATTTGTATATATA	8
hsa-miR-548m	1245	8:1:1	-3.8	-7.99	4.19	CAAAGGUAUUUGUGGUUUUUG	ATATATTTGAATAACAT	8
hsa-miR-520d-3p	1004	8:1:1	-6.7	-10.89	4.19	AAAGUGCUUCUCUUUGGUGGGU	AAGAATTTTTT	8
hsa-miR-1284	152	8:1:1	-6.5	-10.7	4.2	UCUAUACAGACCCUGGCUUUUC	TTGTATAATAA	8
hsa-miR-802	1143	8:1:0	-9.5	-13.71	4.21	CAGUAACAAAGAUUCAUCCUUGU	TTGTTACAAC	8
hsa-miR-26a	1088	8:1:1	-7.6	-11.81	4.21	UUCAAGUAAUCCAGGAUAGGCU	TTATTTGTTTATTGTATAA	8
hsa-miR-568	224	8:1:1	-8.3	-12.51	4.21	AUGUAUAAAUGUAUACACAC	TATATATATATAAT	8
hsa-miR-643	784	8:1:1	-4.2	-8.42	4.22	ACUUGUAUGCUAGCUCAGGUAG	CATATACGGATATAT	8
hsa-miR-653	1146	8:1:0	-10.06	-14.29	4.23	GUGUUGAAACAAUCUCUACUG	TTACAACATG	8
hsa-miR-568	751	8:1:1	-4.2	-8.43	4.23	AUGUAUAAAUGUAUACACAC	TTAATATAGTAACAACGA	8
hsa-miR-448	880	8:1:1	-6.2	-10.44	4.24	UUGCAUAUGUAGGAUGUCCCAU	CATATATAATAAT	8
hsa-miR-577	307	8:1:1	-0.1	-4.34	4.24	UAGAUAAAAUAUUGGUACCUG	TTTTTTTTCATTTTATTT	8
hsa-miR-194	1142	8:1:1	-9.02	-13.27	4.25	UGUAACAGCAACUCCAUGUGGA	TTTGTTACAACATGTAA	8
hsa-miR-568	222	8:1:1	-8.3	-12.55	4.25	AUGUAUAAAUGUAUACACAC	TATATATATATATAAT	8
hsa-miR-548m	985	8:1:1	-5	-9.26	4.26	CAAAGGUAUUUGUGGUUUUUG	ATTCTTTTTAAGAA	8
hsa-miR-548h	658	8:1:1	-2.3	-6.56	4.26	AAAAGUAAUCGCGGUUUUUGUC	ATTATATTCA	8

hsa-miR-380	387	8:1:1	-3.3	-7.56	4.26	UGGUUGACCAUAGAACAUGCGC	TAATATATATAAATTCGT	8
hsa-miR-495	1093	8:1:0	-6.7	-10.97	4.27	GAAGUUGCCCAUGUUAUUUUCG	TGTTTATTGT	8
hsa-miR-633	851	8:1:1	-3.4	-7.68	4.28	CUAAUAGUAUCUACCACAAUAAA	TATTATAAAA	8
hsa-miR-380	807	8:1:1	-4.14	-8.42	4.28	UGGUUGACCAUAGAACAUGCGC	TATAATATAATAA	8
hsa-miR-1259	386	8:1:0	-3.7	-7.98	4.28	UCCCUGAGACCCUUUAACCUGUGA	ATAATATATATAAATT	8
hsa-miR-559	318	8:0:1	-3.4	-7.68	4.28	UAAAGUAAAUAUGCACCAAAA	TTTATTTTTG	8
hsa-miR-520e	1004	8:1:1	-6.6	-10.89	4.29	AAAGUGCUUCCUUUUUGAGGG	AAGAATTTTTT	8
hsa-miR-548m	366	8:1:1	-0.3	-4.59	4.29	CAAAGGUAUUUGUGGUUUUUG	ATAATTTTTATTTTGT	8
hsa-miR-300	150	8:1:1	-6.5	-10.79	4.29	UAUACAAGGGCAGACUCUCUCU	ATTTGTATAAT	8
hsa-miR-548n	36	8:1:1	-4.8	-9.09	4.29	CAAAAGUAAUUGUGGAUUUUGU	TTATTTTACT	8
hsa-miR-30c	1092	8:1:1	-6.4	-10.7	4.3	UGUAAACAUCCUACACUCUCAGC	TTGTTTATTGT	8
hsa-miR-30b	1092	8:1:1	-6.4	-10.7	4.3	UGUAAACAUCCUACACUCAGCU	TTGTTTATTGT	8
hsa-miR-1252	920	8:1:1	-6.9	-11.2	4.3	AGAAGGAAAUUGAAUUCAUUUA	TATCCTTTTTTTTTTT	8
hsa-miR-337-3p	754	8:1:0	-4.2	-8.5	4.3	CUCCUAUAUGAUGCCUUUCUUC	ATATAGTAACAACG	8
hsa-miR-487b	383	8:1:1	-5	-9.3	4.3	GUGGUUAUCCCUGUCCUGUUCG	TGTATAATAT	8
hsa-miR-548l	30	8:1:1	-4.7	-9	4.3	AAAAGUAUUUGCGGGUUUUGUC	AATATATTATT	8
hsa-miR-153	593	8:1:1	-7.9	-12.21	4.31	UCAUUUUUGUGAUGUUGCAGCU	AATATGTATGTGATTAA	8

hsa-miR-380	676	8:0:1	-7.7	-12.02	4.32	UGGUUGACCAUAGAACAUGCGC	TATTATATGGTTCC	8
hsa-miR-633	298	8:1:1	-3.6	-7.92	4.32	CUAAUAGUAUCUACCACAAUAAA	TATTTTTATTTTTTT	8
hsa-miR-494	304	8:1:1	0.3	-4.03	4.33	AGGUUGUCCGUGUUGUCUUCUCU	TATTTTTTTTTCATTTT	8
hsa-miR-384	991	8:1:1	-4.3	-8.65	4.35	AUUCCUAGAAAUUGUUCAUA	TTTAAGAAATATGAAGAA	8
hsa-miR-944	906	8:1:0	-7.7	-12.06	4.36	AAAUUAUUGUACAUCGGAUGAG	CAATATTTATATGATA	8
hsa-miR-30a	299	8:1:1	-3.44	-7.8	4.36	UGUAAACAUCCUCGACUGGAAG	ATTTTTATTTTTT	8
hsa-miR-30d	299	8:1:1	-3.44	-7.8	4.36	UGUAAACAUCCCCGACUGGAAG	ATTTTTATTTT	8
hsa-miR-30e	299	8:1:1	-3.44	-7.8	4.36	UGUAAACAUCCUUGACUGGAAG	ATTTTTATTTT	8
hsa-miR-568	441	8:1:1	-6.1	-10.47	4.37	AUGUAUAAAUGUAUACACAC	TATATATAAATTT	8
hsa-miR-186	398	8:1:0	-3	-7.39	4.39	CAAAGAAUUCUCCUUUUGGGCU	AATTCGTTTT	8
hsa-miR-561	367	8:1:1	-0.3	-4.69	4.39	AUCAAGGAUCUUAAACUUUGCC	TAATTTTTATTT	8
hsa-miR-653	838	8:1:1	-3.4	-7.8	4.4	GUGUUGAAACAAUCUCUACUG	TTACAATAAGTAT	8
hsa-miR-568	1214	8:1:1	-3	-7.41	4.41	AUGUAUAAAUGUAUACACAC	TATATATATAATAATA	8
hsa-miR-548p	1140	8:1:1	-8.9	-13.31	4.41	UAGCAAAAACUGCAGUUACUUU	ATTTTGTTACAACAT	8
hsa-miR-448	87	8:1:1	-6.1	-10.51	4.41	UUGCAUAUGUAGGAUGUCCCAU	TATATTCATT	8
hsa-miR-221	1272	8:1:1	-5.7	-10.12	4.42	ACCUGGCAUACAAUGUAGAUUU	AATGTATTATGTA	8
hsa-miR-181a	556	8:1:1	-6.6	-11.03	4.43	AACAUUCAACGCUGUCGGUGAGU	TTGGATATTTGTATATAT	8

hsa-miR-656	1281	8:1:1	-5.1	-9.54	4.44	AGGUUGCCUGUGAGGUGUUCA	TGTAATAGTA	8
hsa-miR-200c	1127	8:1:1	-8.4	-12.84	4.44	CGUCUUACCCAGCAGUGUUUGG	GTAGTAATTAATTATT	8
hsa-miR-142-5p	729	8:1:1	-3.5	-7.94	4.44	CAUAAAGUAGAAAGCACUACU	TATTATATATATAT	8
hsa-miR-548l	258	8:1:1	-5.3	-9.75	4.45	AAAAGUAUUUGCGGGUUUUGUC	AATATTTCTAAATAT	8
hsa-miR-21	697	8:1:0	-8.9	-13.36	4.46	UAGCUUAUCAGACUGAUGUUGA	AATAAGCTAATATAA	8
hsa-miR-524-5p	93	8:1:0	-5.9	-10.36	4.46	CUACAAAGGGAAGCACUUUCUC	CATTTGTATAATATA	8
hsa-miR-30c	771	8:1:0	-3.6	-8.07	4.47	UGUAAACAUCCUACACUCUCAGC	ATGTTTTCAACACC	8
hsa-miR-30b	771	8:1:0	-3.6	-8.07	4.47	UGUAAACAUCCUACACUCAGCU	ATGTTTTCAACACCATAT	8
hsa-miR-559	582	8:1:1	-7.7	-12.17	4.47	UAAAGUAAAUAUGCACCAAAA	TTTATTTGTATAA	8
hsa-miR-568	469	8:1:1	-7.6	-12.07	4.47	AUGUAUAAAUGUAUACACAC	TATATATATAT	8
hsa-miR-487a	1100	8:1:0	-6.81	-11.3	4.49	GUGGUUAUCCCUGCUGUGUUCG	TGTATAATAATATA	8
hsa-miR-142-3p	1018	8:1:1	-7.09	-11.58	4.49	UGUAGUGUUUCCUACUUUAUGGA	AACGCTTCTAATTACGT	8
hsa-miR-548a-5p	849	8:1:1	-3.3	-7.79	4.49	AAAAGUAAUUGCGAGUUUUACC	ATTATTATAAA	8
hsa-miR-548c-5p	849	8:1:1	-3.3	-7.79	4.49	AAAAGUAAUUGCGGUUUUUGCC	ATTATTATAAAA	8
hsa-miR-548i	849	8:1:1	-3.3	-7.79	4.49	AAAAGUAAUUGCGGAUUUUGCC	ATTATTATAAAATAGTAA	8
hsa-miR-548a-5p	366	8:1:1	-0.1	-4.59	4.49	AAAAGUAAUUGCGAGUUUUACC	ATAATTTTTATTTT	8
hsa-miR-548c-5p	366	8:1:1	-0.1	-4.59	4.49	AAAAGUAAUUGCGGUUUUUGCC	ATAATTTTTATTTTG	8

hsa-miR-548d-5p	366	8:1:1	-0.1	-4.59	4.49	AAAAGUAAUUGUGGUUUUUGCC	ATAATTTTTATTTTGTTTG	8
hsa-miR-548j	366	8:1:1	-0.1	-4.59	4.49	AAAAGUAAUUGCGGUCUUUGGU	ATAATTTTTATTT	8
hsa-miR-548h	366	8:1:1	-0.1	-4.59	4.49	AAAAGUAAUCGCGGUUUUUGUC	ATAATTTTTAT	8
hsa-miR-548i	366	8:1:1	-0.1	-4.59	4.49	AAAAGUAAUUGCGGAUUUUGCC	ATAATTTTTA	8
hsa-miR-26a	912	8:1:1	-7	-11.51	4.51	UUCAAGUAAUCCAGGAUAGGCU	TTATATGATAT	8
hsa-miR-142-3p	779	8:1:1	-5.1	-9.62	4.52	UGUAGUGUUUCCUACUUUAUGGA	AACACCATATACGGATAT	8
hsa-miR-425	1084	8:1:1	-7.8	-12.33	4.53	AAUGACACGAUCACUCCCGUUGA	CGCGTTATTTGTTTAT	8
hsa-miR-487a	97	8:1:0	-5.1	-9.65	4.55	GUGGUUAUCCCUGCUGUGUUCG	TGTATAATATAAATA	8
hsa-miR-487b	97	8:1:1	-5.1	-9.65	4.55	GUGGUUAUCCCUGUCCUGUUCG	TGTATAATATAAATA	8
hsa-miR-33a	1270	8:1:1	-6.7	-11.26	4.56	GUGCAUUGUAGUUGCAUUGCA	AGAATGTATTATGTAAT	8
hsa-miR-520d-5p	93	8:1:0	-5.8	-10.36	4.56	CUACAAAGGGAAGCCCUUUC	CATTTGTATA	8
hsa-miR-518a-5p	93	8:1:1	-5.8	-10.36	4.56	CUGCAAAGGGAAGCCCUUUC	CATTTGTATAATA	8
hsa-miR-527	93	8:1:1	-5.8	-10.36	4.56	CUGCAAAGGGAAGCCCUUUC	CATTTGTATAATA	8
hsa-miR-448	660	8:1:1	-3.2	-7.77	4.57	UUGCAUAUGUAGGAUGUCCCAU	TATATTCATA	8
hsa-miR-182	261	8:1:1	-4	-8.58	4.58	UUUGGCAAUGGUAGAACUCACACU	ATTTCTAAATATATTC	8
hsa-miR-221	753	8:1:1	-3.9	-8.5	4.6	ACCUGGCAUACAAUGUAGAUUU	AATATAGTAACAACGAAAA	8
hsa-miR-1297	41	8:1:0	-6.4	-11	4.6	UUCAAGUAAUUCAGGUG	TTACTTGTTTATTTTT	8

hsa-miR-137	976	8:1:1	-5.1	-9.71	4.61	UUAUUGCUUAAGAAUACGCGUAG	AGGAAATAAATTCT	8
hsa-miR-155	668	8:1:1	-5.2	-9.83	4.63	UUAAUGCUAAUCGUGAUAGGGGU	TAGTAGTATAT	8
hsa-miR-524-5p	561	8:1:1	-6.5	-11.14	4.64	CUACAAAGGGAAGCACUUUCUC	TATTTGTATATATAAA	8
hsa-miR-548o	320	8:1:1	-3.05	-7.7	4.65	AAAAGUAAUUGCGGUUUUUGCC	TATTTTTGATTTTTTT	8
hsa-miR-656	755	8:1:1	-3.8	-8.46	4.66	AGGUUGCCUGUGAGGUGUUCA	TATAGTAACAACGAAA	8
hsa-miR-1270	660	8:1:1	-3.11	-7.77	4.66	CUGGAGAUAUGGAAGAGCUGUGU	TATATTCATAGTAGTA	8
hsa-miR-190b	108	8:1:1	-7.7	-12.36	4.66	UGAUAUGUUUGAUAUUGGGUU	AATATATATATTCA	8
hsa-miR-620	660	8:1:1	-3.1	-7.77	4.67	AUGGAGAUAGAUAUAGAAAU	TATATTCATAGTAGTATA	8
hsa-miR-374a	463	8:1:0	-8.2	-12.87	4.67	UUAUAAUACAACCUGAUAAGUG	GTATAATATAT	8
hsa-miR-494	451	8:1:1	-6.45	-11.12	4.67	AGGUUGUCCGUGUUGUCUUCUCU	TTTGTTTTATT	8
hsa-miR-548p	243	8:0:1	-7.2	-11.87	4.67	UAGCAAAAACUGCAGUUACUUU	TTTTTGTTTTTGTAT	8
hsa-miR-421	1091	8:1:1	-6.2	-10.88	4.68	AUCAACAGACAUUAAUUGGGCGC	TTTGTTTATT	8
hsa-miR-548n	929	8:1:1	-4.44	-9.13	4.69	CAAAAGUAAUUGUGGAUUUUGU	TTTTTTTTTTTTTT	8
hsa-miR-139-5p	666	8:1:1	-5.1	-9.79	4.69	UCUACAGUGCACGUGUCUCCAGU	CATAGTAGTATAT	8
hsa-miR-381	150	8:1:1	-6.1	-10.79	4.69	AGCGAGGUUGCCCUUUGUAUAU	ATTTGTATAATAATATT	8
hsa-miR-802	300	8:1:1	-2.8	-7.5	4.7	CAGUAACAAAGAUUCAUCCUUGU	TTTTTATTTTTTT	8
hsa-miR-300	562	8:1:1	-6.71	-11.42	4.71	UAUACAAGGGCAGACUCUCUCU	ATTTGTATATATAAATT	8

hsa-miR-491-3p	1099	8:0:1	-6.5	-11.22	4.72	CUUAUGCAAGAUUCCCUUCUAC	TTGTATAATA	8
hsa-miR-633	34	8:1:1	-3.2	-7.92	4.72	CUAAUAGUAUCUACCACAAUAAA	TATTATTTTACTT	8
hsa-miR-520d-5p	435	8:1:1	-5.1	-9.83	4.73	CUACAAAGGGAAGCCCUUUC	TATTTGTATATAT	8
hsa-miR-1283	250	8:1:1	-7.4	-12.13	4.73	UCUACAAAGGAAAGCGCUUUCU	TTTTGTATAATATTT	8
hsa-miR-548l	984	8:1:0	-3.8	-8.54	4.74	AAAAGUAUUUGCGGGUUUUGUC	AATTCTTTTTAAGAA	8
hsa-miR-595	501	8:1:1	-7.1	-11.84	4.74	GAAGUGUGCCGUGGUGUGUCU	ACACATTTGATTATTTGT	8
hsa-miR-30e	369	8:1:1	-0.61	-5.35	4.74	UGUAAACAUCCUUGACUGGAAG	ATTTTTATTTTGTTTGTA	8
hsa-miR-655	892	8:1:1	-7.4	-12.15	4.75	AGAGGUUAUCCGUGUUAUGUUC	TTGTATTGGAATAA	8
hsa-miR-369-3p	97	8:1:0	-4.9	-9.65	4.75	AAUAAUACAUGGUUGAUCUUU	TGTATAATATAAAT	8
hsa-miR-30a	1236	8:1:1	-3.71	-8.48	4.77	UGUAAACAUCCUCGACUGGAAG	ATGATTATAATATATT	8
hsa-miR-496	1225	8:1:0	-4.9	-9.67	4.77	UGAGUAUUACAUGGCCAAUCUC	TAATACACGTTATGATTA	8
hsa-miR-374b	1096	8:1:1	-5.74	-10.51	4.77	AUAUAAUACAACCUGCUAAGUG	TTATTGTATAATAATATAT	8
hsa-miR-374b	1101	8:1:0	-6.5	-11.29	4.79	AUAUAAUACAACCUGCUAAGUG	GTATAATAATATATTTAT	8
hsa-miR-491-3p	587	8:0:1	-7.1	-11.9	4.8	CUUAUGCAAGAUUCCCUUCUAC	TTGTATAATATGTATGT	8
hsa-miR-802	539	8:1:1	-5.8	-10.6	4.8	CAGUAACAAAGAUUCAUCCUUGU	TTTTTATTAATTTGTT	8
hsa-miR-559	331	8:1:1	0.5	-4.3	4.8	UAAAGUAAAUAUGCACCAAAA	TTTTTTTTAAATGTCA	8
hsa-miR-491-3p	290	8:1:1	-3.8	-8.6	4.8	CUUAUGCAAGAUUCCCUUCUAC	TTGAGTAATATTTTT	8

hsa-miR-568	226	8:1:1	-8.3	-13.1	4.8	AUGUAUAAAUGUAUACACAC	TATATATATAATT	8
hsa-miR-548n	1088	8:1:1	-7	-11.81	4.81	CAAAAGUAAUUGUGGAUUUUGU	TTATTTGTTTAT	8
hsa-miR-381	562	8:1:1	-6.61	-11.42	4.81	AGCGAGGUUGCCCUUUGUAUAU	ATTTGTATATATAAATT	8
hsa-miR-548n	330	8:1:1	0.55	-4.26	4.81	CAAAAGUAAUUGUGGAUUUUGU	TTTTTTTTTAA	8
hsa-miR-1246	983	8:1:1	-3.51	-8.33	4.82	AAUGGAUUUUUGGAGCAGG	AAATTCTTTTTAAG	8
hsa-miR-33a	708	8:1:1	-7.2	-12.02	4.82	GUGCAUUGUAGUUGCAUUGCA	ATAATACACTA	8
hsa-miR-487b	1274	8:1:1	-5.2	-10.03	4.83	GUGGUUAUCCCUGUCCUGUUCG	TGTATTATGTAA	8
hsa-miR-384	1205	8:1:1	-3.4	-8.23	4.83	AUUCCUAGAAAUUGUUCAUA	TTTAAGAAATATATATATA	8
hsa-miR-549	1089	8:1:1	-6.85	-11.68	4.83	UGACAACUAUGGAUGAGCUCU	TATTTGTTTATTGT	8
hsa-miR-129-5p	1032	8:1:1	-6.3	-11.15	4.85	CUUUUUGCGGUCUGGGCUUGC	CGTATAAAATAAAA	8
hsa-miR-1206	999	8:1:1	-5.9	-10.76	4.86	UGUUCAUGUAGAUGUUUAAGC	ATATGAAGAATTTTT	8
hsa-miR-656	1067	8:1:1	-4	-8.87	4.87	AGGUUGCCUGUGAGGUGUUCA	CATAGTATATAT	8
hsa-miR-340	580	8:1:1	-6.95	-11.84	4.89	UUAUAAAGCAAUGAGACUGAUU	GTTTTATTTGTATAATA	8
hsa-miR-494	477	8:0:1	-6.4	-11.29	4.89	AGGUUGUCCGUGUUGUCUUCUCU	TATGTTTTAT	8
hsa-miR-548b-5p	305	8:1:1	0.4	-4.49	4.89	AAAAGUAAUUGUGGUUUUGGCC	ATTTTTTTTTCA	8
hsa-miR-28-3p	753	8:1:0	-3.6	-8.5	4.9	CACUAGAUUGUGAGCUCCUGGA	AATATAGTAACAACGAA	8
hsa-miR-1284	587	8:1:1	-7	-11.9	4.9	UCUAUACAGACCCUGGCUUUUC	TTGTATAATAT	8

hsa-miR-561	573	8:1:1	-5.52	-10.42	4.9	AUCAAGGAUCUUAAACUUUGCC	TAAATTTGTTTT	8
hsa-miR-26b	1088	8:1:1	-6.9	-11.81	4.91	UUCAAGUAAUUCAGGAUAGGU	TTATTTGTTTA	8
hsa-miR-802	246	8:1:1	-7	-11.93	4.93	CAGUAACAAAGAUUCAUCCUUGU	TTGTTTTTGTATAATATT	8
hsa-miR-300	585	8:1:1	-7.7	-12.64	4.94	UAUACAAGGGCAGACUCUCUCU	ATTTGTATAATATGTATGT	8
hsa-miR-30a	369	8:1:1	-0.4	-5.35	4.95	UGUAAACAUCCUCGACUGGAAG	ATTTTTATTTTGTTTGTAT	8
hsa-miR-30c	369	8:1:1	-0.4	-5.35	4.95	UGUAAACAUCCUACACUCUCAGC	ATTTTTATTTTGTT	8
hsa-miR-30d	369	8:1:1	-0.4	-5.35	4.95	UGUAAACAUCCCCGACUGGAAG	ATTTTTATTTTGTT	8
hsa-miR-30b	369	8:1:1	-0.4	-5.35	4.95	UGUAAACAUCCUACACUCAGCU	ATTTTTATTT	8
hsa-miR-559	313	8:1:1	-0.6	-5.56	4.96	UAAAGUAAAUAUGCACCAAAA	TTCATTTTATTTTT	8
hsa-miR-30c	299	8:1:1	-2.84	-7.8	4.96	UGUAAACAUCCUACACUCUCAGC	ATTTTTATTTTTTTT	8
hsa-miR-542-3p	497	8:1:1	-9.1	-14.07	4.97	UGUGACAGAUUGAUAACUGAAA	TTTGACACATTTGATT	8
hsa-miR-1279	996	8:1:0	-5.3	-10.28	4.98	UCAUAUUGCUUCUUUCU	GAAATATGAAGAAT	8
hsa-miR-1259	808	8:1:0	-3.3	-8.28	4.98	UCCCUGAGACCCUUUAACCUGUGA	ATAATATAATAA	8
hsa-miR-548i	318	8:1:1	-2.7	-7.68	4.98	AAAAGUAAUUGCGGAUUUUGCC	TTTATTTTTGATTT	8
hsa-miR-548j	35	8:0:1	-3.46	-8.44	4.98	AAAAGUAAUUGCGGUCUUUGGU	ATTATTTTACTTG	8
hsa-miR-26a	583	8:1:1	-7.8	-12.79	4.99	UUCAAGUAAUCCAGGAUAGGCU	TTATTTGTATAATATG	8
hsa-miR-374a	871	8:1:0	-4.4	-9.41	5.01	UUAUAAUACAACCUGAUAAGUG	GTAATATATCATATATAAT	8

hsa-miR-340	454	8:1:1	-6.3	-11.31	5.01	UUAUAAAGCAAUGAGACUGAUU	GTTTTATTTGTATA	8
hsa-miR-448	784	8:1:1	-3.4	-8.42	5.02	UUGCAUAUGUAGGAUGUCCCAU	CATATACGGA	8
hsa-miR-374b	154	8:1:0	-5	-10.02	5.02	AUAUAAUACAACCUGCUAAGUG	GTATAATAAT	8
hsa-miR-568	732	8:1:1	-3	-8.06	5.06	AUGUAUAAAUGUAUACACAC	TATATATATATATATATA	8
hsa-miR-548f	353	8:1:1	-0.3	-5.37	5.07	UGCAAAAGUAAUCACAGUUUUU	ATAATTTTAATATATAATT	8
hsa-miR-577	455	8:0:1	-6.1	-11.18	5.08	UAGAUAAAAUAUUGGUACCUG	TTTTATTTGTATAATATAT	8
hsa-miR-454	38	8:1:1	-4.87	-9.97	5.1	ACCCUAUCAAUAUUGUCUCUGC	ATTTTACTTG	8
hsa-miR-548p	193	8:1:0	-6.7	-11.81	5.11	UAGCAAAAACUGCAGUUACUUU	TTTTGGCTTTTAT	8
hsa-miR-421	286	8:1:1	-4.8	-9.92	5.12	AUCAACAGACAUUAAUUGGGCGC	TTTTTTGAGTAA	8
hsa-miR-374a	154	8:1:0	-4.9	-10.02	5.12	UUAUAAUACAACCUGAUAAGUG	GTATAATAAT	8
hsa-miR-30d	1236	8:1:1	-3.35	-8.48	5.13	UGUAAACAUCCCCGACUGGAAG	ATGATTATAA	8
hsa-miR-600	989	8:1:1	-3.4	-8.54	5.14	ACUUACAGACAAGAGCCUUGCUC	TTTTTAAGAAATATGAAGA	8
hsa-miR-487b	588	8:1:1	-6.73	-11.87	5.14	GUGGUUAUCCCUGUCCUGUUCG	TGTATAATATGTATGTGAT	8
hsa-miR-576-5p	1238	8:1:1	-3.4	-8.55	5.15	AUUCUAAUUUCUCCACGUCUUU	GATTATAATATAT	8
hsa-miR-548d-3p	326	8:1:0	-0.6	-5.75	5.15	CAAAAACCACAGUUUCUUUUGC	TGATTTTTTTTTT	8
hsa-miR-548n	928	8:1:1	-4.02	-9.18	5.16	CAAAAGUAAUUGUGGAUUUUGU	TTTTTTTTTTTT	8
hsa-miR-802	370	8:1:1	-0.2	-5.36	5.16	CAGUAACAAAGAUUCAUCCUUGU	TTTTTATTTTGTTTG	8

hsa-miR-1246	1211	8:1:1	-3	-8.18	5.18	AAUGGAUUUUUGGAGCAGG	AAATATATATATAATAA	8
hsa-miR-577	943	8:1:1	-1.9	-7.08	5.18	UAGAUAAAAUAUUGGUACCUG	TTTTGTATTCATACATA	8
hsa-miR-568	734	8:1:1	-3.1	-8.28	5.18	AUGUAUAAAUGUAUACACAC	TATATATATATATATATTT	8
hsa-miR-369-3p	153	8:1:0	-5.1	-10.28	5.18	AAUAAUACAUGGUUGAUCUUU	TGTATAATAATATTTGTA	8
hsa-miR-26b	583	8:1:1	-7.6	-12.79	5.19	UUCAAGUAAUUCAGGAUAGGU	TTATTTGTATAA	8
hsa-miR-153	1122	8:1:1	-5.74	-10.94	5.2	UCAUUUUUGUGAUGUUGCAGCU	AATATGTAGTA	8
hsa-miR-548n	41	8:1:0	-5.8	-11	5.2	CAAAAGUAAUUGUGGAUUUUGU	TTACTTGTTTATTTTTTTT	8
hsa-miR-511	26	8:1:1	-3.26	-8.46	5.2	GUGUCUUUUGCUCUGCAGUCA	AAAAAATATATTATTTT	8
hsa-miR-369-3p	1095	8:1:1	-4.8	-10.01	5.21	AAUAAUACAUGGUUGAUCUUU	TTTATTGTATAA	8
hsa-miR-142-3p	1223	8:1:1	-3.85	-9.07	5.22	UGUAGUGUUUCCUACUUUAUGGA	AATAATACACGTTATGATT	8
hsa-miR-548n	931	8:1:1	-3.29	-8.52	5.23	CAAAAGUAAUUGUGGAUUUUGU	TTTTTTTTTT	8
hsa-miR-559	307	8:1:1	0.89	-4.34	5.23	UAAAGUAAAUAUGCACCAAAA	TTTTTTTTCATT	8
hsa-miR-885-5p	1281	8:1:0	-4.3	-9.54	5.24	UCCAUUACACUACCCUGCCUCU	TGTAATAGTA	8
hsa-miR-548a-5p	318	8:1:1	-2.44	-7.68	5.24	AAAAGUAAUUGCGAGUUUUACC	TTTATTTTTGATTTTTT	8
hsa-miR-522	90	8:1:1	-6.19	-11.43	5.24	CUCUAGAGGGAAGCGCUUUCUG	ATTCATTTGTAT	8
hsa-miR-487b	913	8:1:1	-6.2	-11.45	5.25	GUGGUUAUCCCUGUCCUGUUCG	TATATGATATCCTTT	8
hsa-miR-491-3p	166	8:1:1	-6.3	-11.55	5.25	CUUAUGCAAGAUUCCCUUCUAC	TTGTATATAT	8

hsa-miR-1284	166	8:1:1	-6.3	-11.55	5.25	UCUAUACAGACCCUGGCUUUUC	TTGTATATATA	8
hsa-miR-144	131	8:1:1	-7.8	-13.05	5.25	GGAUAUCAUCAUAUACUGUAAG	TATATTTTTATTTT	8
hsa-miR-656	717	8:1:0	-0.7	-5.97	5.27	AGGUUGCCUGUGAGGUGUUCA	TAAAATATTTCTTA	8
hsa-miR-641	245	8:1:1	-6.6	-11.87	5.27	AAAGACAUAGGAUAGAGUCACCUC	TTTGTTTTTGTATAATAT	8
hsa-miR-891a	1054	8:1:1	-3.7	-8.98	5.28	UGCAACGAACCUGAGCCACUGA	TTCCTTGTTT	8
hsa-miR-380	385	8:1:1	-3.4	-8.69	5.29	UGGUUGACCAUAGAACAUGCGC	TATAATATATATAAAT	8
hsa-miR-374b	254	8:1:0	-5.3	-10.59	5.29	AUAUAAUACAACCUGCUAAGUG	GTATAATATTTCTAA	8
hsa-miR-147	498	8:1:1	-8.1	-13.4	5.3	GUGUGUGGAAAUGCUUCUGC	TTGACACATTTG	8
hsa-miR-577	312	8:1:1	0.1	-5.22	5.32	UAGAUAAAAUAUUGGUACCUG	TTTCATTTTATTTTTGA	8
hsa-miR-181d	596	8:1:0	-6.6	-11.93	5.33	AACAUUCAUUGUUGUCGGUGGGU	ATGTATGTGATTAA	8
hsa-miR-1284	252	8:1:1	-6.8	-12.13	5.33	UCUAUACAGACCCUGGCUUUUC	TTGTATAATATTTCTAAA	8
hsa-miR-331-5p	880	8:1:1	-5.1	-10.44	5.34	CUAGGUAUGGUCCCAGGGAUCC	CATATATAATA	8
hsa-miR-381	585	8:1:1	-7.3	-12.64	5.34	AGCGAGGUUGCCCUUUGUAUAU	ATTTGTATAATAT	8
hsa-miR-569	1013	8:1:1	-6.03	-11.39	5.36	AGUUAAUGAAUCCUGGAAAGU	TTTTTAACGCTTCTAATTA	8
hsa-miR-410	943	8:1:1	-1.7	-7.08	5.38	AGGUUGUCUGUGAUGAGUUCG	TTTTGTATTCAT	8
hsa-miR-628-3p	40	8:1:1	-4.7	-10.09	5.39	UCUAGUAAGAGUGGCAGUCGA	TTTACTTGTTTAT	8
hsa-miR-548n	930	8:1:1	-3.86	-9.28	5.42	CAAAAGUAAUUGUGGAUUUUGU	TTTTTTTTTT	8

hsa-miR-491-3p	461	8:0:1	-7.1	-12.52	5.42	CUUAUGCAAGAUUCCCUUCUAC	TTGTATAATATATATAT	8
hsa-miR-26a	286	8:1:1	-4.5	-9.92	5.42	UUCAAGUAAUCCAGGAUAGGCU	TTTTTTGAGTAATATTT	8
hsa-miR-548n	927	8:1:1	-3.6	-9.03	5.43	CAAAAGUAAUUGUGGAUUUUGU	TTTTTTTTTTTTTTT	8
hsa-miR-487b	253	8:1:1	-6.3	-11.73	5.43	GUGGUUAUCCCUGUCCUGUUCG	TGTATAATATTTCT	8
hsa-miR-410	1276	8:1:1	-5.3	-10.75	5.45	AGGUUGUCUGUGAUGAGUUCG	TATTATGTAATAGTA	8
hsa-miR-568	201	8:0:1	-8.06	-13.52	5.46	AUGUAUAAAUGUAUACACAC	TTTATATAAT	8
hsa-miR-544	1125	8:1:1	-8.1	-13.57	5.47	AUUCUGCAUUUUUAGCAAGUUC	ATGTAGTAATTAATTA	8
hsa-miR-203	874	8:1:1	-5.46	-10.93	5.47	AGUGGUUCUUAACAGUUCAACAGUU	ATATATCATA	8
hsa-miR-548d-5p	849	8:1:1	-2.3	-7.79	5.49	AAAAGUAAUUGUGGUUUUUGCC	ATTATTATAAAA	8
hsa-miR-32	1100	8:1:1	-5.8	-11.3	5.5	UAUUGCACAUUACUAAGUUGCA	TGTATAATAATATAT	8
hsa-miR-548m	919	8:1:1	-5.7	-11.2	5.5	CAAAGGUAUUUGUGGUUUUUG	ATATCCTTTTTTTTTT	8
hsa-miR-410	870	8:1:0	-3	-8.51	5.51	AGGUUGUCUGUGAUGAGUUCG	TGTAATATATCATA	8
hsa-miR-1284	461	8:1:1	-7	-12.52	5.52	UCUAUACAGACCCUGGCUUUUC	TTGTATAATATAT	8
hsa-miR-520d-5p	458	8:1:1	-5.7	-11.24	5.54	CUACAAAGGGAAGCCCUUUC	TATTTGTATAATA	8
hsa-miR-548m	745	8:1:1	-2.8	-8.35	5.55	CAAAGGUAUUUGUGGUUUUUG	ATATATTTAAT	8
hsa-miR-539	258	8:1:1	-4.2	-9.75	5.55	GGAGAAAUUAUCCUUGGUGUGU	AATATTTCTAAATA	8
hsa-miR-374a	98	8:1:0	-5.6	-11.15	5.55	UUAUAAUACAACCUGAUAAGUG	GTATAATATAAATATATA	8

hsa-miR-1290	981	8:1:1	-2.9	-8.47	5.57	UGGAUUUUUGGAUCAGGGA	ATAAATTCTTTTTAAG	8
hsa-miR-524-5p	584	8:1:1	-7.2	-12.77	5.57	CUACAAAGGGAAGCACUUUCUC	TATTTGTATAATA	8
hsa-miR-548p	1089	8:1:1	-6.1	-11.68	5.58	UAGCAAAAACUGCAGUUACUUU	TATTTGTTTA	8
hsa-miR-548c-5p	318	8:1:1	-2.1	-7.68	5.58	AAAAGUAAUUGCGGUUUUUGCC	TTTATTTTTGATTTTT	8
hsa-miR-548j	318	8:1:1	-2.1	-7.68	5.58	AAAAGUAAUUGCGGUCUUUGGU	TTTATTTTTGATTTTTTTT	8
hsa-miR-548h	318	8:1:1	-2.1	-7.68	5.58	AAAAGUAAUCGCGGUUUUUGUC	TTTATTTTTGA	8
hsa-miR-375	1001	8:1:1	-4.4	-10	5.6	UUUGUUCGUUCGGCUCGCGUGA	ATGAAGAATTTTTTTTA	8
hsa-miR-337-3p	894	8:1:1	-7.4	-13	5.6	CUCCUAUAUGAUGCCUUUCUUC	GTATTGGAATAACAATA	8
hsa-miR-186	284	8:0:1	-4.2	-9.81	5.61	CAAAGAAUUCUCCUUUUGGGCU	AATTTTTTGAGTA	8
hsa-miR-450b-5p	1098	8:1:1	-5.4	-11.02	5.62	UUUUGCAAUAUGUUCCUGAAUA	ATTGTATAATAATATATT	8
hsa-miR-767-5p	681	8:1:1	-8.4	-14.02	5.62	UGCACCAUGGUUGUCUGAGCAUG	TATGGTTCCATATT	8
hsa-miR-380	877	8:1:1	-4.6	-10.23	5.63	UGGUUGACCAUAGAACAUGCGC	TATCATATATAAT	8
hsa-miR-548l	35	8:1:1	-2.81	-8.44	5.63	AAAAGUAUUUGCGGGUUUUGUC	ATTATTTTACTTGTTTAT	8
hsa-miR-557	1032	8:1:1	-5.5	-11.15	5.65	GUUUGCACGGGUGGGCCUUGUCU	CGTATAAAAT	8
hsa-miR-559	330	8:1:1	1.4	-4.26	5.66	UAAAGUAAAUAUGCACCAAAA	TTTTTTTTTAAAT	8
hsa-miR-186	296	8:1:1	-2.4	-8.06	5.66	CAAAGAAUUCUCCUUUUGGGCU	AATATTTTTATTTTTTTT	8
hsa-miR-190	108	8:1:1	-6.7	-12.36	5.66	UGAUAUGUUUGAUAUAUUAGGU	AATATATATATTCATAGT	8

hsa-miR-548f	297	8:1:1	-2.3	-7.97	5.67	UGCAAAAGUAAUCACAGUUUUU	ATATTTTTATTTTTT	8
hsa-miR-511	28	8:1:1	-3.13	-8.8	5.67	GUGUCUUUUGCUCUGCAGUCA	AAAATATATTA	8
hsa-miR-30c	1236	8:1:1	-2.8	-8.48	5.68	UGUAAACAUCCUACACUCUCAGC	ATGATTATAAT	8
hsa-miR-30b	1236	8:1:1	-2.8	-8.48	5.68	UGUAAACAUCCUACACUCAGCU	ATGATTATAATATATT	8
hsa-miR-369-3p	893	8:1:1	-7.1	-12.78	5.68	AAUAAUACAUGGUUGAUCUUU	TGTATTGGAATAACA	8
hsa-miR-885-5p	893	8:1:0	-7.1	-12.78	5.68	UCCAUUACACUACCCUGCCUCU	TGTATTGGAATAACAATA	8
hsa-miR-1264	207	8:1:1	-6.4	-12.09	5.69	CAAGUCUUAUUUGAGCACCUGUU	TAATATTTGTAAATA	8
hsa-miR-26a	36	8:1:1	-3.4	-9.09	5.69	UUCAAGUAAUCCAGGAUAGGCU	TTATTTTACTTGTTTATT	8
hsa-miR-656	1105	8:1:0	-7.5	-13.2	5.7	AGGUUGCCUGUGAGGUGUUCA	AATAATATATTTATTAAAA	8
hsa-miR-568	228	8:1:1	-8.4	-14.1	5.7	AUGUAUAAAUGUAUACACAC	TATATATAATTTGTTTTTT	8
hsa-miR-1297	1088	8:1:1	-6.1	-11.81	5.71	UUCAAGUAAUUCAGGUG	TTATTTGTTTA	8
hsa-miR-369-3p	32	8:1:0	-3.2	-8.91	5.71	AAUAAUACAUGGUUGAUCUUU	TATATTATTTTA	8
hsa-miR-1284	1099	8:1:1	-5.5	-11.22	5.72	UCUAUACAGACCCUGGCUUUUC	TTGTATAATAATATATTT	8
hsa-miR-524-5p	987	8:1:1	-3.1	-8.83	5.73	CUACAAAGGGAAGCACUUUCUC	TCTTTTTAAGA	8
hsa-miR-208b	987	8:1:1	-3.1	-8.83	5.73	AAGCUUUUUGCUCGAAUUAUGU	TCTTTTTAAGAAATATGA	8
hsa-miR-556-3p	668	8:1:1	-4.1	-9.83	5.73	AUAUUACCAUUAGCUCAUCUUU	TAGTAGTATATTATA	8
hsa-miR-144	32	8:1:1	-3.17	-8.91	5.74	GGAUAUCAUCAUAUACUGUAAG	TATATTATTTTAC	8

hsa-miR-548n	932	8:1:1	-2.73	-8.48	5.75	CAAAAGUAAUUGUGGAUUUUGU	TTTTTTTTTTTT	8
hsa-miR-577	581	8:0:1	-6.1	-11.85	5.75	UAGAUAAAAUAUUGGUACCUG	TTTTATTTGTATAAT	8
hsa-miR-539	235	8:1:1	-6.5	-12.25	5.75	GGAGAAAUUAUCCUUGGUGUGU	AATTTGTTTTTTGTTT	8
hsa-miR-548j	985	8:1:1	-3.5	-9.26	5.76	AAAAGUAAUUGCGGUCUUUGGU	ATTCTTTTTAAGAA	8
hsa-miR-19a	1249	8:1:1	-5.2	-10.97	5.77	AGUUUUGCAUAGUUGCACUACA	ATTTGAATAAC	8
hsa-miR-548d-3p	1058	8:1:0	-3.3	-9.07	5.77	CAAAAACCACAGUUUCUUUUGC	TTGTTTTTCCAT	8
hsa-miR-1179	1005	8:1:1	-5.2	-10.97	5.77	AAGCAUUCUUUCAUUGGUUGG	AGAATTTTTT	8
hsa-miR-583	415	8:1:1	-3	-8.78	5.78	CAAAGAGGAAGGUCCCAUUAC	TTCTATTTGACACA	8
hsa-miR-509-5p	1124	8:1:1	-7.67	-13.46	5.79	UACUGCAGACAGUGGCAAUCA	TATGTAGTAATTAA	8
hsa-miR-548e	319	8:1:1	-1.9	-7.69	5.79	CAAAAGCAAUCGCGGUUUUUGC	TTATTTTTGATT	8
hsa-miR-581	1257	8:1:1	-5.22	-11.04	5.82	UCUUGUGUUCUCUAGAUCAGU	AACATAAAACAATA	8
hsa-miR-548n	986	8:1:1	-3.2	-9.02	5.82	CAAAAGUAAUUGUGGAUUUUGU	TTCTTTTTAAGA	8
hsa-miR-181c	556	8:1:1	-5.2	-11.03	5.83	AACAUUCAACCUGUCGGUGAGU	TTGGATATTTGT	8
hsa-miR-548n	132	8:1:1	-7.01	-12.84	5.83	CAAAAGUAAUUGUGGAUUUUGU	ATATTTTTATTTTGATA	8
hsa-miR-559	522	8:1:1	-9	-14.84	5.84	UAAAGUAAAUAUGCACCAAAA	TATATTTTGTTTGAATTTT	8
hsa-miR-1264	257	8:1:1	-4	-9.84	5.84	CAAGUCUUAUUUGAGCACCUGUU	TAATATTTCTAAA	8
hsa-miR-494	549	8:1:1	-4.02	-9.87	5.85	AGGUUGUCCGUGUUGUCUUCUCU	TTTGTTTTTGG	8

hsa-miR-589	1059	8:1:1	-3.2	-9.06	5.86	UGAGAACCACGUCUGCUCUGAG	TGTTTTTCCATAGTATAT	8
hsa-miR-20b	1005	8:1:1	-5.1	-10.97	5.87	CAAAGUGCUCAUAGUGCAGGUAG	AGAATTTTTTTTAACG	8
hsa-miR-568	565	8:1:1	-5.8	-11.67	5.87	AUGUAUAAAUGUAUACACAC	TGTATATATAAATTTGT	8
hsa-miR-548c-3p	297	8:1:0	-2.1	-7.97	5.87	CAAAAAUCUCAAUUACUUUUGC	ATATTTTTATTT	8
hsa-miR-300	164	8:1:1	-6.6	-12.48	5.88	UAUACAAGGGCAGACUCUCUCU	ATTTGTATATATA	8
hsa-miR-548n	926	8:1:1	-3.2	-9.09	5.89	CAAAAGUAAUUGUGGAUUUUGU	TTTTTTTTTTT	8
hsa-miR-494	489	8:1:1	-7.9	-13.79	5.89	AGGUUGUCCGUGUUGUCUUCUCU	TGTGTTTATTTGACACA	8
hsa-miR-1290	27	8:1:1	-2.5	-8.39	5.89	UGGAUUUUUGGAUCAGGGA	AAAAATATATTATT	8
hsa-miR-374a	894	8:1:1	-7.1	-13	5.9	UUAUAAUACAACCUGAUAAGUG	GTATTGGAATAA	8
hsa-miR-369-3p	383	8:1:0	-3.4	-9.3	5.9	AAUAAUACAUGGUUGAUCUUU	TGTATAATATATATA	8
hsa-miR-568	167	8:1:1	-5.54	-11.44	5.9	AUGUAUAAAUGUAUACACAC	TGTATATATA	8
hsa-miR-548n	925	8:1:1	-2.96	-8.87	5.91	CAAAAGUAAUUGUGGAUUUUGU	TTTTTTTTTTT	8
hsa-miR-656	1034	8:1:0	-5.3	-11.22	5.92	AGGUUGCCUGUGAGGUGUUCA	TATAAAATAAAAAT	8
hsa-miR-548n	933	8:1:1	-2.5	-8.43	5.93	CAAAAGUAAUUGUGGAUUUUGU	TTTTTTTTTT	8
hsa-miR-520d-5p	561	8:1:1	-5.2	-11.14	5.94	CUACAAAGGGAAGCCCUUUC	TATTTGTATATAT	8
hsa-miR-221	1124	8:1:1	-7.5	-13.46	5.96	ACCUGGCAUACAAUGUAGAUUU	TATGTAGTAATTAATTAT	8
hsa-miR-222	1124	8:1:1	-7.5	-13.46	5.96	CUCAGUAGCCAGUGUAGAUCCU	TATGTAGTAATT	8

hsa-miR-548n	934	8:1:1	-2.45	-8.41	5.96	CAAAAGUAAUUGUGGAUUUUGU	TTTTTTTTTTT	8
hsa-miR-21	1176	8:1:1	-8.51	-14.48	5.97	UAGCUUAUCAGACUGAUGUUGA	CATAAGTTGTAAAA	8
hsa-miR-568	110	8:1:1	-6.1	-12.07	5.97	AUGUAUAAAUGUAUACACAC	TATATATATTCATAGTA	8
hsa-miR-142-5p	37	8:1:1	-3.8	-9.77	5.97	CAUAAAGUAGAAAGCACUACU	TATTTTACTTGTTTAT	8
hsa-miR-181d	1166	8:1:0	-6.5	-12.48	5.98	AACAUUCAUUGUUGUCGGUGGGU	ATTAATGTATCATAAG	8
hsa-miR-495	1089	8:1:0	-5.7	-11.68	5.98	GAAGUUGCCCAUGUUAUUUUCG	TATTTGTTTA	8
hsa-miR-1297	494	8:0:1	-7.6	-13.59	5.99	UUCAAGUAAUUCAGGUG	TTATTTGACACATTT	8
hsa-miR-548j	305	8:1:1	1.5	-4.49	5.99	AAAAGUAAUUGCGGUCUUUGGU	ATTTTTTTTTCATTTTA	8
hsa-miR-548h	305	8:1:1	1.5	-4.49	5.99	AAAAGUAAUCGCGGUUUUUGUC	ATTTTTTTTTCAT	8
hsa-miR-340	480	8:1:1	-8.06	-14.07	6.01	UUAUAAAGCAAUGAGACUGAUU	GTTTTATTTTGTG	8
hsa-miR-383	1023	8:1:1	-6.8	-12.82	6.02	AGAUCAGAAGGUGAUUGUGGCU	TTCTAATTACGTATAAAAT	8
hsa-miR-410	579	8:1:0	-5.8	-11.82	6.02	AGGUUGUCUGUGAUGAGUUCG	TGTTTTATTTGTATA	8
hsa-miR-32	97	8:1:1	-3.63	-9.65	6.02	UAUUGCACAUUACUAAGUUGCA	TGTATAATATA	8
hsa-miR-653	1204	8:1:1	-2.1	-8.13	6.03	GUGUUGAAACAAUCUCUACUG	TTTTAAGAAATAT	8
hsa-miR-32	253	8:1:1	-5.7	-11.73	6.03	UAUUGCACAUUACUAAGUUGCA	TGTATAATATTTC	8
hsa-miR-656	750	8:1:0	-2.4	-8.44	6.04	AGGUUGCCUGUGAGGUGUUCA	TTTAATATAGTAA	8
hsa-miR-633	726	8:1:1	-1.5	-7.54	6.04	CUAAUAGUAUCUACCACAAUAAA	TCTTATTATATATATATA	8

hsa-miR-203	747	8:1:1	-2.33	-8.38	6.05	AGUGGUUCUUAACAGUUCAACAGUU	ATATTTAATAT	8
hsa-miR-190	258	8:1:1	-3.7	-9.75	6.05	UGAUAUGUUUGAUAUAUUAGGU	AATATTTCTAAATAT	8
hsa-miR-583	1199	8:1:1	-2.5	-8.56	6.06	CAAAGAGGAAGGUCCCAUUAC	TTCTATTTTAAGA	8
hsa-miR-548a-5p	296	8:1:1	-2	-8.06	6.06	AAAAGUAAUUGCGAGUUUUACC	AATATTTTTAT	8
hsa-miR-448	114	8:1:1	-5	-11.06	6.06	UUGCAUAUGUAGGAUGUCCCAU	TATATTCATAGTATATAT	8
hsa-miR-590-3p	256	8:1:0	-3.9	-9.97	6.07	UAAUUUUAUGUAUAAGCUAGU	ATAATATTTCT	8
hsa-miR-380	255	8:1:1	-4	-10.07	6.07	UGGUUGACCAUAGAACAUGCGC	TATAATATTT	8
hsa-miR-1322	1171	8:1:1	-8.3	-14.38	6.08	GAUGAUGCUGCUGAUGCUG	TGTATCATAAGTTGTAAAA	8
hsa-miR-200c	1085	8:1:1	-6.6	-12.68	6.08	CGUCUUACCCAGCAGUGUUUGG	GCGTTATTTG	8
hsa-miR-944	543	8:1:1	-4	-10.12	6.12	AAAUUAUUGUACAUCGGAUGAG	TATTAATTTGTTTTTG	8
hsa-miR-1277	1027	8:1:1	-5.6	-11.76	6.16	AAAUAUAUAUAUAUAUGUACGUAU	AATTACGTATA	8
hsa-miR-548k	296	8:1:1	-1.9	-8.06	6.16	AAAAGUACUUGCGGAUUUUGCU	AATATTTTTATTTTTTTTT	8
hsa-miR-548i	296	8:1:1	-1.9	-8.06	6.16	AAAAGUAAUUGCGGAUUUUGCC	AATATTTTTATTTTTTT	8
hsa-miR-1303	1021	8:1:1	-6.7	-12.88	6.18	UUUAGAGACGGGGUCUUGCUCU	GCTTCTAATTACGTATA	8
hsa-miR-548n	196	8:1:1	-6.4	-12.58	6.18	CAAAAGUAAUUGUGGAUUUUGU	TGGCTTTTATATAATAT	8
hsa-miR-548n	457	8:1:1	-5.3	-11.51	6.21	CAAAAGUAAUUGUGGAUUUUGU	TTATTTGTATA	8
hsa-miR-559	325	8:1:1	-0.1	-6.33	6.23	UAAAGUAAAUAUGCACCAAAA	TTGATTTTTTTTTAAA	8

hsa-miR-877	1020	8:1:1	-6.6	-12.84	6.24	GUAGAGGAGAUGGCGCAGGG	CGCTTCTAATTACGTATA	8
hsa-miR-183	684	8:1:1	-7.9	-14.14	6.24	UAUGGCACUGGUAGAAUUCACU	GGTTCCATATTAA	8
hsa-miR-499-5p	478	8:1:1	-4.79	-11.03	6.24	UUAAGACUUGCAGUGAUGUUU	ATGTTTTATTTTGTG	8
hsa-miR-380	1027	8:1:1	-5.5	-11.76	6.26	UGGUUGACCAUAGAACAUGCGC	AATTACGTAT	8
hsa-miR-548c-5p	985	8:1:1	-3	-9.26	6.26	AAAAGUAAUUGCGGUUUUUGCC	ATTCTTTTTAAGAAAT	8
hsa-miR-548d-5p	985	8:1:1	-3	-9.26	6.26	AAAAGUAAUUGUGGUUUUUGCC	ATTCTTTTTAAGAAAT	8
hsa-miR-539	449	8:1:1	-4.83	-11.09	6.26	GGAGAAAUUAUCCUUGGUGUGU	AATTTGTTTTATTTGTA	8
hsa-miR-381	164	8:1:1	-6.2	-12.48	6.28	AGCGAGGUUGCCCUUUGUAUAU	ATTTGTATATATATATA	8
hsa-miR-802	725	8:1:1	-1.3	-7.59	6.29	CAGUAACAAAGAUUCAUCCUUGU	TTCTTATTATATAT	8
hsa-miR-548n	935	8:1:1	-2.41	-8.71	6.3	CAAAAGUAAUUGUGGAUUUUGU	TTTTTTTTTTT	8
hsa-miR-32	383	8:1:1	-3	-9.3	6.3	UAUUGCACAUUACUAAGUUGCA	TGTATAATATAT	8
hsa-miR-376b	912	8:1:1	-5.2	-11.51	6.31	CGUGGAUAUUCCUUCUAUGUUU	TTATATGATAT	8
hsa-miR-1185	86	8:1:1	-5.1	-11.41	6.31	AGAGGAUACCCUUUGUAUGUU	GTATATTCATTTGT	8
hsa-miR-655	1153	8:1:0	-8.6	-14.92	6.32	AGAGGUUAUCCGUGUUAUGUUC	ATGTAATAATAAAATTA	8
hsa-miR-889	688	8:1:0	-7.7	-14.02	6.32	AAUGGCUGUCCGUAGUAUGGUC	CCATATTAAA	8
hsa-miR-374b	98	8:1:0	-4.8	-11.15	6.35	AUAUAAUACAACCUGCUAAGUG	GTATAATATAAATATA	8
hsa-miR-217	1124	8:1:1	-7.1	-13.46	6.36	UACUGCAUCAGGAACUGAUUGGA	TATGTAGTAATTAAT	8

hsa-miR-548k	672	8:1:1	-5.3	-11.67	6.37	AAAAGUACUUGCGGAUUUUGCU	AGTATATTATATGGTT	8
hsa-miR-26b	511	8:1:1	-8.46	-14.84	6.38	UUCAAGUAAUUCAGGAUAGGU	TTATTTGTATATATATT	8
hsa-miR-577	39	8:1:1	-3.7	-10.08	6.38	UAGAUAAAAUAUUGGUACCUG	TTTTACTTGTTTATTTT	8
hsa-miR-599	1256	8:1:1	-4.7	-11.11	6.41	GUUGUGUCAGUUUAUCAAAC	TAACATAAAACAATAGA	8
hsa-miR-376a	912	8:1:1	-5.1	-11.51	6.41	GUAGAUUCUCCUUCUAUGAGUA	TTATATGATATCCTT	8
hsa-miR-888	554	8:1:1	-4	-10.41	6.41	UACUCAAAAAGCUGUCAGUCA	TTTTGGATAT	8
hsa-miR-768-3p	1275	8:1:1	-3.7	-10.12	6.42	UGCACCAUGGUUGUCUGAGCAUG	GTATTATGTAATAGT	8
hsa-miR-520d-5p	987	8:1:1	-2.4	-8.83	6.43	CUACAAAGGGAAGCCCUUUC	TCTTTTTAAGAAAT	8
hsa-miR-144	1076	8:1:0	-4.7	-11.15	6.45	GGAUAUCAUCAUAUACUGUAAG	TATAATGTCGCGTTATT	8
hsa-miR-559	493	8:1:1	-6.6	-13.05	6.45	UAAAGUAAAUAUGCACCAAAA	TTTATTTGACACATTT	8
hsa-miR-548c-5p	296	8:1:1	-1.6	-8.06	6.46	AAAAGUAAUUGCGGUUUUUGCC	AATATTTTTATTTTTT	8
hsa-miR-548j	296	8:1:1	-1.6	-8.06	6.46	AAAAGUAAUUGCGGUCUUUGGU	AATATTTTTATT	8
hsa-miR-548h	296	8:1:1	-1.6	-8.06	6.46	AAAAGUAAUCGCGGUUUUUGUC	AATATTTTTA	8
hsa-miR-584	1263	8:1:0	-3	-9.47	6.47	UUAUGGUUUGCCUGGGACUGAG	AAACAATAGAATGTATTA	8
hsa-miR-548k	1005	8:1:1	-4.5	-10.97	6.47	AAAAGUACUUGCGGAUUUUGCU	AGAATTTTTTTTTAA	8
hsa-miR-300	513	8:1:1	-7.3	-13.77	6.47	UAUACAAGGGCAGACUCUCUCU	ATTTGTATATATATTTT	8
hsa-miR-137	1263	8:1:0	-2.99	-9.47	6.48	UUAUUGCUUAAGAAUACGCGUAG	AAACAATAGA	8

hsa-miR-590-3p	1040	8:1:0	-4.12	-10.6	6.48	UAAUUUUAUGUAUAAGCUAGU	ATAAAAATAAATG	8
hsa-miR-548l	161	8:1:1	-7.4	-13.88	6.48	AAAAGUAUUUGCGGGUUUUGUC	AATATTTGTA	8
hsa-miR-548l	1007	8:1:1	-2.7	-9.19	6.49	AAAAGUAUUUGCGGGUUUUGUC	AATTTTTTTTA	8
hsa-miR-487b	1100	8:1:1	-4.8	-11.3	6.5	GUGGUUAUCCCUGUCCUGUUCG	TGTATAATAA	8
hsa-miR-1297	286	8:1:1	-3.4	-9.92	6.52	UUCAAGUAAUUCAGGUG	TTTTTTGAGTAATAT	8
hsa-miR-548l	522	8:1:1	-8.3	-14.84	6.54	AAAAGUAUUUGCGGGUUUUGUC	TATATTTTGTTTGAATTTT	8
hsa-miR-581	1226	8:1:1	-3.5	-10.05	6.55	UCUUGUGUUCUCUAGAUCAGU	AATACACGTTATGATTA	8
hsa-miR-142-5p	1097	8:1:1	-3.8	-10.35	6.55	CAUAAAGUAGAAAGCACUACU	TATTGTATAATAATAT	8
hsa-miR-548b-5p	296	8:1:1	-1.5	-8.06	6.56	AAAAGUAAUUGUGGUUUUGGCC	AATATTTTTATTTTTTTT	8
hsa-miR-548d-5p	296	8:1:1	-1.5	-8.06	6.56	AAAAGUAAUUGUGGUUUUUGCC	AATATTTTTATTTT	8
hsa-miR-1270	114	8:1:1	-4.5	-11.06	6.56	CUGGAGAUAUGGAAGAGCUGUGU	TATATTCATAGTA	8
hsa-miR-582-5p	93	8:1:1	-3.8	-10.36	6.56	UUACAGUUGUUCAACCAGUUACU	CATTTGTATAATA	8
hsa-miR-577	136	8:0:1	-5.7	-12.27	6.57	UAGAUAAAAUAUUGGUACCUG	TTTTATTTTG	8
hsa-miR-656	854	8:1:0	-1.3	-7.88	6.58	AGGUUGCCUGUGAGGUGUUCA	TATAAAATAGTAACAATGT	8
hsa-miR-653	990	8:1:1	-2	-8.59	6.59	GUGUUGAAACAAUCUCUACUG	TTTTAAGAAATATG	8
hsa-miR-548n	939	8:1:1	-2.85	-9.45	6.6	CAAAAGUAAUUGUGGAUUUUGU	TTTTTTTTGTA	8
hsa-miR-561	283	8:1:1	-3.2	-9.8	6.6	AUCAAGGAUCUUAAACUUUGCC	TAATTTTTTGAGTAAT	8

hsa-miR-548d-5p	137	8:1:1	-5.8	-12.41	6.61	AAAAGUAAUUGUGGUUUUUGCC	TTTATTTTGAT	8
hsa-miR-186	1052	8:1:1	-2.4	-9.05	6.65	CAAAGAAUUCUCCUUUUGGGCU	GATTCCTTGTTTTTCCA	8
hsa-miR-509-3-5p	1124	8:1:1	-6.8	-13.46	6.66	UACUGCAGACGUGGCAAUCAUG	TATGTAGTAATTAA	8
hsa-miR-548b-5p	985	8:1:1	-2.6	-9.26	6.66	AAAAGUAAUUGUGGUUUUGGCC	ATTCTTTTTAAGA	8
hsa-miR-1259	1070	8:1:1	-2.79	-9.46	6.67	UCCCUGAGACCCUUUAACCUGUGA	AGTATATATA	8
hsa-miR-548n	936	8:1:1	-2.37	-9.04	6.67	CAAAAGUAAUUGUGGAUUUUGU	TTTTTTTTTT	8
hsa-miR-340	199	8:1:1	-7.6	-14.27	6.67	UUAUAAAGCAAUGAGACUGAUU	CTTTTATATAAT	8
hsa-miR-584	28	8:1:1	-2.13	-8.8	6.67	UUAUGGUUUGCCUGGGACUGAG	AAAATATATTA	8
hsa-miR-548a-5p	282	8:1:1	-3	-9.69	6.69	AAAAGUAAUUGCGAGUUUUACC	ATAATTTTTT	8
hsa-miR-374b	894	8:1:1	-6.3	-13	6.7	AUAUAAUACAACCUGCUAAGUG	GTATTGGAATAACAA	8
hsa-miR-485-3p	598	8:1:1	-5.6	-12.3	6.7	GUCAUACACGGCUCUCCUCUCU	GTATGTGATTAAAT	8
hsa-miR-653	1063	8:1:1	-2.1	-8.82	6.72	GUGUUGAAACAAUCUCUACUG	TTTCCATAGTATATA	8
hsa-miR-380	915	8:1:1	-4.6	-11.32	6.72	UGGUUGACCAUAGAACAUGCGC	TATGATATCCTTTTTTTTT	8
hsa-miR-142-5p	538	8:1:1	-3.8	-10.52	6.72	CAUAAAGUAGAAAGCACUACU	TTTTTTATTAATTTGTTTT	8
hsa-miR-568	1072	8:1:1	-3.2	-9.93	6.73	AUGUAUAAAUGUAUACACAC	TATATATAATGTCGCGT	8
hsa-miR-548k	35	8:1:1	-1.7	-8.44	6.74	AAAAGUACUUGCGGAUUUUGCU	ATTATTTTACTTGTTTATT	8
hsa-miR-142-3p	904	8:1:1	-5.8	-12.55	6.75	UGUAGUGUUUCCUACUUUAUGGA	AACAATATTT	8

hsa-miR-450b-5p	852	8:1:1	-1.1	-7.86	6.76	UUUUGCAAUAUGUUCCUGAAUA	ATTATAAAATAGTAACA	8
hsa-miR-620	114	8:1:1	-4.29	-11.06	6.77	AUGGAGAUAGAUAUAGAAAU	TATATTCATAGTAT	8
hsa-miR-10b	679	8:1:1	-6.2	-12.98	6.78	UACCCUGUAGAACCGAAUUUGUG	TATATGGTTC	8
hsa-miR-655	31	8:1:0	-2.2	-8.98	6.78	AGAGGUUAUCCGUGUUAUGUUC	ATATATTATTTTACTTGT	8
hsa-miR-380	205	8:1:1	-5.4	-12.2	6.8	UGGUUGACCAUAGAACAUGCGC	TATAATATTTGTAAA	8
hsa-miR-203	1247	8:1:1	-2.3	-9.11	6.81	AGUGGUUCUUAACAGUUCAACAGUU	ATATTTGAATAACATAA	8
hsa-miR-570	452	8:1:1	-4.3	-11.11	6.81	AAAGGUAAUUGCAGUUUUUCCC	TTGTTTTATTT	8
hsa-miR-548c-5p	1137	8:0:1	-7.1	-13.92	6.82	AAAAGUAAUUGCGGUUUUUGCC	ATTATTTTGTTACAAC	8
hsa-miR-548d-5p	1137	8:0:1	-7.1	-13.92	6.82	AAAAGUAAUUGUGGUUUUUGCC	ATTATTTTGTTACAACATG	8
hsa-miR-630	707	8:1:1	-5.6	-12.44	6.84	AGUAUUCUGUACCAGGGAAGGU	TATAATACACTAAAATATT	8
hsa-miR-548d-3p	550	8:1:0	-3	-9.84	6.84	CAAAAACCACAGUUUCUUUUGC	TTGTTTTTGGATATTTGT	8
hsa-miR-203	503	8:1:0	-6.1	-12.96	6.86	AGUGGUUCUUAACAGUUCAACAGUU	ACATTTGATTATTTGTATA	8
hsa-miR-519a	90	8:1:1	-4.57	-11.43	6.86	CUCUAGAGGGAAGCGCUUUCUG	ATTCATTTGTATA	8
hsa-miR-381	513	8:1:1	-6.9	-13.77	6.87	AGCGAGGUUGCCCUUUGUAUAU	ATTTGTATATATATTTGT	8
hsa-miR-144	679	8:1:1	-6.1	-12.98	6.88	GGAUAUCAUCAUAUACUGUAAG	TATATGGTTCCATATTAAA	8
hsa-miR-539	547	8:1:1	-3	-9.88	6.88	GGAGAAAUUAUCCUUGGUGUGU	AATTTGTTTTTGGATA	8

hsa-miR-1267	606	8:1:1	-4.1	-10.99	6.89	CCUGUUGAAGUGUAAUCCCCA	TTAAATAGAAAA	8
hsa-miR-577	540	8:1:1	-3.8	-10.7	6.9	UAGAUAAAAUAUUGGUACCUG	TTTTATTAATTTGTTT	8
hsa-miR-944	1048	8:1:1	-2.9	-9.82	6.92	AAAUUAUUGUACAUCGGAUGAG	AAATGATTCCTTGTTTTT	8
hsa-miR-487a	1274	8:1:0	-3.1	-10.03	6.93	GUGGUUAUCCCUGCUGUGUUCG	TGTATTATGTAATAGTA	8
hsa-miR-494	577	8:1:1	-4.9	-11.83	6.93	AGGUUGUCCGUGUUGUCUUCUCU	TTTGTTTTATTTGTATAAT	8
hsa-miR-656	1076	8:0:1	-4.2	-11.15	6.95	AGGUUGCCUGUGAGGUGUUCA	TATAATGTCGCGTTA	8
hsa-miR-374b	589	8:1:0	-4.8	-11.78	6.98	AUAUAAUACAACCUGCUAAGUG	GTATAATATGTATGTGAT	8
hsa-miR-487a	462	8:1:0	-5.9	-12.88	6.98	GUGGUUAUCCCUGCUGUGUUCG	TGTATAATATATATA	8
hsa-miR-488	197	8:1:1	-5.7	-12.69	6.99	CCCAGAUAAUGGCACUCUCAA	GGCTTTTATATAA	8
hsa-miR-524-5p	149	8:1:1	-4.2	-11.19	6.99	CUACAAAGGGAAGCACUUUCUC	TATTTGTATAA	8
hsa-miR-656	915	8:0:1	-4.3	-11.32	7.02	AGGUUGCCUGUGAGGUGUUCA	TATGATATCC	8
hsa-miR-142-5p	676	8:1:1	-5	-12.02	7.02	CAUAAAGUAGAAAGCACUACU	TATTATATGGTTCC	8
hsa-miR-524-5p	512	8:1:1	-7.5	-14.52	7.02	CUACAAAGGGAAGCACUUUCUC	TATTTGTATATATATTTTG	8
hsa-miR-655	478	8:1:0	-4	-11.03	7.03	AGAGGUUAUCCGUGUUAUGUUC	ATGTTTTATTTT	8
hsa-miR-559	456	8:1:1	-4.6	-11.64	7.04	UAAAGUAAAUAUGCACCAAAA	TTTATTTGTATAATATAT	8
hsa-miR-191	985	8:1:1	-2.2	-9.26	7.06	CAACGGAAUCCCAAAAGCAGCUG	ATTCTTTTTA	8
hsa-miR-1259	871	8:1:1	-2.35	-9.41	7.06	UCCCUGAGACCCUUUAACCUGUGA	GTAATATATC	8

hsa-miR-499-5p	1179	8:1:1	-7	-14.07	7.07	UUAAGACUUGCAGUGAUGUUU	AAGTTGTAAAAT	8
hsa-miR-520d-5p	584	8:1:1	-5.7	-12.77	7.07	CUACAAAGGGAAGCCCUUUC	TATTTGTATAATATGTAT	8
hsa-miR-520a-3p	1004	8:1:1	-3.8	-10.89	7.09	AAAGUGCUUCCCUUUGGACUGU	AAGAATTTTTTTTAA	8
hsa-miR-520d-5p	149	8:1:1	-4.1	-11.19	7.09	CUACAAAGGGAAGCCCUUUC	TATTTGTATAATAATA	8
hsa-miR-548n	937	8:1:1	-2.33	-9.43	7.1	CAAAAGUAAUUGUGGAUUUUGU	TTTTTTTTTTGTATTC	8
hsa-miR-361-5p	555	8:1:1	-2.9	-10.01	7.11	UUAUCAGAAUCUCCAGGGGUAC	TTTGGATATTTGTATATAT	8
hsa-miR-559	137	8:0:1	-5.3	-12.41	7.11	UAAAGUAAAUAUGCACCAAAA	TTTATTTTGATATATTTGT	8
hsa-miR-410	453	8:1:0	-4.05	-11.18	7.13	AGGUUGUCUGUGAUGAGUUCG	TGTTTTATTTGTATAATA	8
hsa-miR-548b-5p	544	8:1:0	-3	-10.14	7.14	AAAAGUAAUUGUGGUUUUGGCC	ATTAATTTGTTTTTGGATA	8
hsa-miR-548l	208	8:1:1	-4.37	-11.51	7.14	AAAAGUAUUUGCGGGUUUUGUC	AATATTTGTAAAT	8
hsa-miR-548l	1108	8:1:1	-4.62	-11.77	7.15	AAAAGUAUUUGCGGGUUUUGUC	AATATATTTATTAAA	8
hsa-miR-429	33	8:1:1	-1.15	-8.33	7.18	UAAUACUGUCUGGUAAAACCGU	ATATTATTTTACTTGTTTA	8
hsa-miR-302e	1004	8:1:1	-3.7	-10.89	7.19	UAAGUGCUUCCAUGCUU	AAGAATTTTTTTTAA	8
hsa-miR-548i	282	8:1:1	-2.5	-9.69	7.19	AAAAGUAAUUGCGGAUUUUGCC	ATAATTTTTTGAGTAA	8
hsa-miR-590-3p	1161	8:0:0	-5.8	-13	7.2	UAAUUUUAUGUAUAAGCUAGU	ATAAAATTAAT	8
hsa-miR-548n	283	8:1:1	-2.6	-9.8	7.2	CAAAAGUAAUUGUGGAUUUUGU	TAATTTTTTGAGTAA	8
hsa-miR-548a-5p	1137	8:0:1	-6.71	-13.92	7.21	AAAAGUAAUUGCGAGUUUUACC	ATTATTTTGTTACAAC	8

hsa-miR-1259	218	8:1:1	-4.09	-11.3	7.21	UCCCUGAGACCCUUUAACCUGUGA	AATATATATATATA	8
hsa-miR-1259	201	8:1:1	-6.3	-13.52	7.22	UCCCUGAGACCCUUUAACCUGUGA	TTTATATAATATTTGTAA	8
hsa-miR-656	729	8:1:0	-0.7	-7.94	7.24	AGGUUGCCUGUGAGGUGUUCA	TATTATATATATATATAT	8
hsa-miR-570	578	8:1:1	-4.6	-11.84	7.24	AAAGGUAAUUGCAGUUUUUCCC	TTGTTTTATT	8
hsa-miR-568	171	8:1:1	-5.1	-12.35	7.25	AUGUAUAAAUGUAUACACAC	TATATATATATATAATTT	8
hsa-miR-380	1241	8:1:1	-1.3	-8.56	7.26	UGGUUGACCAUAGAACAUGCGC	TATAATATATTTGA	8
hsa-miR-1259	605	8:1:1	-4.6	-11.86	7.26	UCCCUGAGACCCUUUAACCUGUGA	ATTAAATAGA	8
hsa-miR-641	577	8:1:1	-4.56	-11.83	7.27	AAAGACAUAGGAUAGAGUCACCUC	TTTGTTTTATTTGTA	8
hsa-miR-539	184	8:1:1	-6.5	-13.77	7.27	GGAGAAAUUAUCCUUGGUGUGU	AATTTGTTTTTTTGGC	8
hsa-miR-130a	38	8:1:1	-2.7	-9.97	7.27	UUCACAUUGUGCUACUGUCUGC	ATTTTACTTGTTTATT	8
hsa-miR-1246	29	8:1:1	-1.7	-8.97	7.27	AAUGGAUUUUUGGAGCAGG	AAATATATTATT	8
hsa-miR-570	924	8:1:1	-1.39	-8.67	7.28	AAAGGUAAUUGCAGUUUUUCCC	CTTTTTTTTTTTTTT	8
hsa-miR-10a	679	8:1:1	-5.7	-12.98	7.28	UACCCUGUAGAUCCGAAUUUGUG	TATATGGTTCCAT	8
hsa-miR-26b	36	8:1:1	-1.8	-9.09	7.29	UUCAAGUAAUUCAGGAUAGGU	TTATTTTACTTGTTTATT	8
hsa-miR-1297	36	8:1:1	-1.8	-9.09	7.29	UUCAAGUAAUUCAGGUG	TTATTTTACTTGTTTA	8
hsa-miR-501-5p	1047	8:1:1	-2.61	-9.91	7.3	AAUCCUUUGUCCCUGGGUGAGA	TAAATGATTCCTTG	8
hsa-miR-1279	591	8:1:1	-4.4	-11.7	7.3	UCAUAUUGCUUCUUUCU	ATAATATGTATGTGATTA	8

hsa-miR-548n	938	8:1:1	-2.29	-9.6	7.31	CAAAAGUAAUUGUGGAUUUUGU	TTTTTTTTTGTATTC	8
hsa-miR-409-3p	905	8:1:1	-4.9	-12.21	7.31	GAAUGUUGCUCGGUGAACCCCU	ACAATATTTATAT	8
hsa-miR-890	1023	8:1:1	-5.5	-12.82	7.32	UACUUGGAAAGGCAUCAGUUG	TTCTAATTACGTATAA	8
hsa-miR-380	590	8:1:1	-4.39	-11.72	7.33	UGGUUGACCAUAGAACAUGCGC	TATAATATGTAT	8
hsa-miR-548h	510	8:1:1	-7.6	-14.93	7.33	AAAAGUAAUCGCGGUUUUUGUC	ATTATTTGTATA	8
hsa-miR-549	42	8:1:1	-4.6	-11.93	7.33	UGACAACUAUGGAUGAGCUCU	TACTTGTTTATTTTT	8
hsa-miR-656	674	8:1:0	-5.4	-12.74	7.34	AGGUUGCCUGUGAGGUGUUCA	TATATTATATGGTTC	8
hsa-miR-548d-5p	544	8:1:0	-2.8	-10.14	7.34	AAAAGUAAUUGUGGUUUUUGCC	ATTAATTTGT	8
hsa-miR-559	324	8:1:1	0	-7.35	7.35	UAAAGUAAAUAUGCACCAAAA	TTTGATTTTTTTTT	8
hsa-miR-190b	258	8:1:1	-2.4	-9.75	7.35	UGAUAUGUUUGAUAUUGGGUU	AATATTTCTAAATATATT	8
hsa-miR-758	1144	8:1:1	-6.6	-13.96	7.36	GAUGGUUGACCAGAGAGCACAC	TGTTACAACATGTA	8
hsa-miR-374b	670	8:1:0	-3.7	-11.06	7.36	AUAUAAUACAACCUGCUAAGUG	GTAGTATATT	8
hsa-miR-548b-5p	137	8:1:1	-5.02	-12.41	7.39	AAAAGUAAUUGUGGUUUUGGCC	TTTATTTTGATAT	8
hsa-miR-33a	1224	8:1:1	-2	-9.4	7.4	GUGCAUUGUAGUUGCAUUGCA	ATAATACACGT	8
hsa-miR-26b	912	8:1:1	-4.1	-11.51	7.41	UUCAAGUAAUUCAGGAUAGGU	TTATATGATA	8
hsa-miR-548p	925	8:1:1	-1.45	-8.87	7.42	UAGCAAAAACUGCAGUUACUUU	TTTTTTTTTTTTTTTTTTT	8
hsa-miR-186	547	8:1:1	-2.46	-9.88	7.42	CAAAGAAUUCUCCUUUUGGGCU	AATTTGTTTTTGGATA	8

hsa-miR-656	1274	8:1:1	-2.6	-10.03	7.43	AGGUUGCCUGUGAGGUGUUCA	TGTATTATGTA	8
hsa-miR-559	1200	8:1:1	-1.8	-9.23	7.43	UAAAGUAAAUAUGCACCAAAA	TCTATTTTAAG	8
hsa-miR-323-3p	1076	8:1:0	-3.7	-11.15	7.45	AGGUGGUCCGUGGCGCGUUCGC	TATAATGTCGC	8
hsa-miR-142-5p	748	8:1:1	-1.2	-8.65	7.45	CAUAAAGUAGAAAGCACUACU	TATTTAATATAGTA	8
hsa-miR-568	567	8:1:1	-5.1	-12.55	7.45	AUGUAUAAAUGUAUACACAC	TATATATAAAT	8
hsa-miR-32	153	8:1:1	-2.83	-10.28	7.45	UAUUGCACAUUACUAAGUUGCA	TGTATAATAATATTTGT	8
hsa-miR-374a	670	8:1:0	-3.6	-11.06	7.46	UUAUAAUACAACCUGAUAAGUG	GTAGTATATTA	8
hsa-miR-568	516	8:1:1	-6.4	-13.86	7.46	AUGUAUAAAUGUAUACACAC	TGTATATATAT	8
hsa-miR-190	467	8:1:1	-4.6	-12.06	7.46	UGAUAUGUUUGAUAUAUUAGGU	AATATATATATATGTTTTA	8
hsa-miR-186	535	8:0:1	-6	-13.47	7.47	CAAAGAAUUCUCCUUUUGGGCU	AATTTTTTTATTAA	8
hsa-miR-491-3p	515	8:1:1	-6.3	-13.78	7.48	CUUAUGCAAGAUUCCCUUCUAC	TTGTATATATATT	8
hsa-miR-1284	515	8:1:1	-6.3	-13.78	7.48	UCUAUACAGACCCUGGCUUUUC	TTGTATATATATTTTG	8
hsa-miR-548n	583	8:1:1	-5.3	-12.79	7.49	CAAAAGUAAUUGUGGAUUUUGU	TTATTTGTATAAT	8
hsa-miR-548e	451	8:1:1	-3.63	-11.12	7.49	CAAAAGCAAUCGCGGUUUUUGC	TTTGTTTTATTTGT	8
hsa-miR-568	143	8:1:1	-4.87	-12.38	7.51	AUGUAUAAAUGUAUACACAC	TTGATATATTTGTA	8
hsa-miR-491-3p	84	8:1:1	-6.3	-13.81	7.51	CUUAUGCAAGAUUCCCUUCUAC	TTGTATATTCATTTGTA	8
hsa-miR-1284	84	8:1:1	-6.3	-13.81	7.51	UCUAUACAGACCCUGGCUUUUC	TTGTATATTCATTTGT	8

hsa-miR-181b	596	8:1:0	-4.4	-11.93	7.53	AACAUUCAUUGCUGUCGGUGGGU	ATGTATGTGATTAAATAG	8
hsa-miR-144	1139	8:1:1	-6	-13.55	7.55	GGAUAUCAUCAUAUACUGUAAG	TATTTTGTTACAACA	8
hsa-miR-656	1097	8:1:1	-2.8	-10.35	7.55	AGGUUGCCUGUGAGGUGUUCA	TATTGTATAA	8
hsa-miR-548i	985	8:1:1	-1.7	-9.26	7.56	AAAAGUAAUUGCGGAUUUUGCC	ATTCTTTTTAAGAAATA	8
hsa-miR-934	122	8:1:1	-5.8	-13.36	7.56	UGUCUACUACUGGAGACACUGG	TAGTATATATATATT	8
hsa-miR-494	245	8:1:1	-4.3	-11.87	7.57	AGGUUGUCCGUGUUGUCUUCUCU	TTTGTTTTTGTATAATATT	8
hsa-miR-548n	138	8:1:1	-5.01	-12.58	7.57	CAAAAGUAAUUGUGGAUUUUGU	TTATTTTGATATATT	8
hsa-miR-633	1235	8:1:1	-0.9	-8.49	7.59	CUAAUAGUAUCUACCACAAUAAA	TATGATTATAATAT	8
hsa-miR-1297	912	8:1:1	-3.9	-11.51	7.61	UUCAAGUAAUUCAGGUG	TTATATGATATCCTT	8
hsa-miR-569	1115	8:1:1	-4.7	-12.32	7.62	AGUUAAUGAAUCCUGGAAAGU	TTATTAAAATATGT	8
hsa-miR-548m	476	8:1:1	-4.3	-11.92	7.62	CAAAGGUAUUUGUGGUUUUUG	ATATGTTTTATTT	8
hsa-miR-656	158	8:1:0	-4.7	-12.32	7.62	AGGUUGCCUGUGAGGUGUUCA	AATAATATTT	8
hsa-miR-380	122	8:1:1	-5.7	-13.36	7.66	UGGUUGACCAUAGAACAUGCGC	TAGTATATATAT	8
hsa-miR-93	1005	8:1:1	-3.3	-10.97	7.67	CAAAGUGCUGUUCGUGCAGGUAG	AGAATTTTTTTTTAACGCT	8
hsa-miR-548p	926	8:1:1	-1.41	-9.09	7.68	UAGCAAAAACUGCAGUUACUUU	TTTTTTTTTTTT	8
hsa-miR-186	770	8:1:1	-0.62	-8.3	7.68	CAAAGAAUUCUCCUUUUGGGCU	AATGTTTTCAACAC	8
hsa-miR-624	546	8:1:1	-2.2	-9.89	7.69	UAGUACCAGUACCUUGUGUUCA	TAATTTGTTTTT	8

hsa-miR-568	169	8:1:1	-4.5	-12.19	7.69	AUGUAUAAAUGUAUACACAC	TATATATATATATAT	8
hsa-miR-587	1278	8:1:1	-3.1	-10.8	7.7	UUUCCAUAGGUGAUGAGUCAC	TTATGTAATA	8
hsa-miR-144	484	8:1:1	-6.9	-14.65	7.75	GGAUAUCAUCAUAUACUGUAAG	TATTTTGTGTTT	8
hsa-miR-641	237	8:1:1	-5.81	-13.56	7.75	AAAGACAUAGGAUAGAGUCACCUC	TTTGTTTTTTTGTTTTTG	8
hsa-miR-221	1169	8:1:0	-5.6	-13.38	7.78	ACCUGGCAUACAAUGUAGAUUU	AATGTATCATA	8
hsa-miR-487b	462	8:1:1	-5.1	-12.88	7.78	GUGGUUAUCCCUGUCCUGUUCG	TGTATAATAT	8
hsa-miR-577	415	8:1:1	-1	-8.78	7.78	UAGAUAAAAUAUUGGUACCUG	TTCTATTTGACA	8
hsa-miR-656	884	8:1:0	-3.8	-11.59	7.79	AGGUUGCCUGUGAGGUGUUCA	TATAATAATTGTATTGGAA	8
hsa-miR-548i	1137	8:0:1	-6.11	-13.92	7.81	AAAAGUAAUUGCGGAUUUUGCC	ATTATTTTGTTACAACAT	8
hsa-miR-888	193	8:1:1	-4	-11.81	7.81	UACUCAAAAAGCUGUCAGUCA	TTTTGGCTTTTATATA	8
hsa-miR-19b	1249	8:1:1	-3.1	-10.97	7.87	UGUGCAAAUCCAUGCAAAACUGA	ATTTGAATAACATAA	8
hsa-miR-590-3p	1268	8:1:1	-3.7	-11.58	7.88	UAAUUUUAUGUAUAAGCUAGU	ATAGAATGTATTATGTAA	8
hsa-miR-568	220	8:1:1	-4.6	-12.49	7.89	AUGUAUAAAUGUAUACACAC	TATATATATATATAT	8
hsa-miR-369-3p	1100	8:1:0	-3.4	-11.3	7.9	AAUAAUACAUGGUUGAUCUUU	TGTATAATAATATAT	8
hsa-miR-548d-5p	1008	8:1:1	-2.9	-10.8	7.9	AAAAGUAAUUGUGGUUUUUGCC	ATTTTTTTTT	8
hsa-miR-429	121	8:1:1	-5.8	-13.71	7.91	UAAUACUGUCUGGUAAAACCGU	ATAGTATATATATAT	8
hsa-miR-656	32	8:1:0	-1	-8.91	7.91	AGGUUGCCUGUGAGGUGUUCA	TATATTATTTTA	8

hsa-miR-548b-5p	1137	8:0:1	-6	-13.92	7.92	AAAAGUAAUUGUGGUUUUGGCC	ATTATTTTGTTACAACAT	8
hsa-miR-577	929	8:1:1	-1.2	-9.13	7.93	UAGAUAAAAUAUUGGUACCUG	TTTTTTTTTTT	8
hsa-miR-410	250	8:1:1	-4.2	-12.13	7.93	AGGUUGUCUGUGAUGAGUUCG	TTTTGTATAATATTT	8
hsa-miR-548a-5p	544	8:1:0	-2.2	-10.14	7.94	AAAAGUAAUUGCGAGUUUUACC	ATTAATTTGTTTT	8
hsa-miR-548c-5p	544	8:1:0	-2.2	-10.14	7.94	AAAAGUAAUUGCGGUUUUUGCC	ATTAATTTGTTTTTG	8
hsa-miR-590-5p	980	8:1:1	-0.8	-8.75	7.95	GAGCUUAUUCAUAAAAGUGCAG	AATAAATTCTTTTTAAGAA	8
hsa-miR-559	131	8:1:1	-5.1	-13.05	7.95	UAAAGUAAAUAUGCACCAAAA	TATATTTTTATTTTGATA	8
hsa-miR-548a-5p	985	8:1:1	-1.3	-9.26	7.96	AAAAGUAAUUGCGAGUUUUACC	ATTCTTTTTA	8
hsa-miR-548e	549	8:1:1	-1.9	-9.87	7.97	CAAAAGCAAUCGCGGUUUUUGC	TTTGTTTTTGGATA	8
hsa-miR-891b	546	8:1:1	-1.9	-9.89	7.99	UGCAACUUACCUGAGUCAUUGA	TAATTTGTTTTTGGATATT	8
hsa-miR-664	979	8:1:0	-1.1	-9.1	8	ACUGGCUAGGGAAAAUGAUUGGAU	AAATAAATTCTTT	8
hsa-miR-429	905	8:1:0	-4.2	-12.21	8.01	UAAUACUGUCUGGUAAAACCGU	ACAATATTTATATGAT	8
hsa-miR-186	432	8:1:1	0.4	-7.63	8.03	CAAAGAAUUCUCCUUUUGGGCU	GATTATTTGTATA	8
hsa-miR-944	160	8:1:1	-5.5	-13.53	8.03	AAAUUAUUGUACAUCGGAUGAG	TAATATTTGTATATAT	8
hsa-miR-548i	544	8:1:0	-2.1	-10.14	8.04	AAAAGUAAUUGCGGAUUUUGCC	ATTAATTTGTTTTTG	8
hsa-miR-577	481	8:0:1	-6.6	-14.64	8.04	UAGAUAAAAUAUUGGUACCUG	TTTTATTTTGTGTTT	8
hsa-miR-590-3p	294	8:1:1	-0.3	-8.34	8.04	UAAUUUUAUGUAUAAGCUAGU	GTAATATTTTTATT	8

hsa-miR-590-3p	180	8:1:0	-6.2	-14.24	8.04	UAAUUUUAUGUAUAAGCUAGU	ATATAATTTGTTT	8
hsa-miR-1284	892	8:1:1	-4.1	-12.15	8.05	UCUAUACAGACCCUGGCUUUUC	TTGTATTGGAATA	8
hsa-miR-577	324	8:1:1	0.7	-7.35	8.05	UAGAUAAAAUAUUGGUACCUG	TTTGATTTTT	8
hsa-miR-380	1144	8:1:1	-5.9	-13.96	8.06	UGGUUGACCAUAGAACAUGCGC	TGTTACAACAT	8
hsa-miR-633	540	8:1:1	-2.64	-10.7	8.06	CUAAUAGUAUCUACCACAAUAAA	TTTTATTAATTTGTTT	8
hsa-miR-190b	467	8:1:1	-4	-12.06	8.06	UGAUAUGUUUGAUAUUGGGUU	AATATATATATATGTTT	8
hsa-miR-374b	463	8:1:0	-4.8	-12.87	8.07	AUAUAAUACAACCUGCUAAGUG	GTATAATATATATA	8
hsa-miR-561	447	8:1:1	-3	-11.08	8.08	AUCAAGGAUCUUAAACUUUGCC	TAAATTTGTTTTA	8
hsa-miR-1259	1174	8:1:1	-6.4	-14.49	8.09	UCCCUGAGACCCUUUAACCUGUGA	ATCATAAGTTGT	8
hsa-miR-548i	536	8:1:1	-3	-11.1	8.1	AAAAGUAAUUGCGGAUUUUGCC	ATTTTTTTATTAA	8
hsa-miR-144	674	8:1:1	-4.6	-12.74	8.14	GGAUAUCAUCAUAUACUGUAAG	TATATTATATGGTT	8
hsa-miR-1246	88	8:1:1	-3.3	-11.46	8.16	AAUGGAUUUUUGGAGCAGG	ATATTCATTT	8
hsa-miR-17	1005	8:1:1	-2.8	-10.97	8.17	CAAAGUGCUUACAGUGCAGGUAG	AGAATTTTTTTTAACGC	8
hsa-miR-20a	1005	8:1:1	-2.8	-10.97	8.17	UAAAGUGCUUAUAGUGCAGGUAG	AGAATTTTTTTTA	8
hsa-miR-106a	1005	8:1:1	-2.8	-10.97	8.17	AAAAGUGCUUACAGUGCAGGUAG	AGAATTTTTTTT	8
hsa-miR-548c-3p	1005	8:1:0	-2.8	-10.97	8.17	CAAAAAUCUCAAUUACUUUUGC	AGAATTTTTTTTAAC	8
hsa-miR-548e	245	8:1:1	-3.7	-11.87	8.17	CAAAAGCAAUCGCGGUUUUUGC	TTTGTTTTTGTAT	8

hsa-miR-181b	1166	8:1:0	-4.3	-12.48	8.18	AACAUUCAUUGCUGUCGGUGGGU	ATTAATGTATCATAA	8
hsa-miR-577	928	8:1:1	-1	-9.18	8.18	UAGAUAAAAUAUUGGUACCUG	TTTTTTTTTTTTT	8
hsa-miR-656	707	8:1:0	-4.26	-12.44	8.18	AGGUUGCCUGUGAGGUGUUCA	TATAATACACTAAAAT	8
hsa-miR-548j	282	8:1:1	-1.5	-9.69	8.19	AAAAGUAAUUGCGGUCUUUGGU	ATAATTTTTTGAGTAA	8
hsa-miR-664	1271	8:1:1	-2.5	-10.7	8.2	ACUGGCUAGGGAAAAUGAUUGGAU	GAATGTATTATGTAATA	8
hsa-miR-548e	577	8:1:1	-3.63	-11.83	8.2	CAAAAGCAAUCGCGGUUUUUGC	TTTGTTTTATTTG	8
hsa-miR-1259	121	8:1:1	-5.5	-13.71	8.21	UCCCUGAGACCCUUUAACCUGUGA	ATAGTATATA	8
hsa-miR-142-5p	32	8:1:1	-0.7	-8.91	8.21	CAUAAAGUAGAAAGCACUACU	TATATTATTTT	8
hsa-miR-548b-5p	510	8:1:1	-6.7	-14.93	8.23	AAAAGUAAUUGUGGUUUUGGCC	ATTATTTGTATAT	8
hsa-miR-382	904	8:1:1	-4.3	-12.55	8.25	GAAGUUGUUCGUGGUGGAUUCG	AACAATATTTATAT	8
hsa-miR-337-3p	1125	8:1:1	-5.3	-13.57	8.27	CUCCUAUAUGAUGCCUUUCUUC	ATGTAGTAATTAATTA	8
hsa-miR-656	1119	8:1:0	-1.3	-9.58	8.28	AGGUUGCCUGUGAGGUGUUCA	TAAAATATGTAG	8
hsa-miR-380	872	8:1:1	-2.3	-10.58	8.28	UGGUUGACCAUAGAACAUGCGC	TAATATATCA	8
hsa-miR-487a	679	8:1:1	-4.7	-12.98	8.28	GUGGUUAUCCCUGCUGUGUUCG	TATATGGTTCCATATTAAA	8
hsa-miR-26b	80	8:1:1	-6.8	-15.08	8.28	UUCAAGUAAUUCAGGAUAGGU	TTATTTGTATATTCA	8
hsa-miR-429	206	8:1:1	-4.3	-12.59	8.29	UAAUACUGUCUGGUAAAACCGU	ATAATATTTGTAAATATAT	8
hsa-miR-548l	284	8:1:1	-1.5	-9.81	8.31	AAAAGUAUUUGCGGGUUUUGUC	AATTTTTTGAG	8

hsa-miR-548j	1137	8:0:1	-5.6	-13.92	8.32	AAAAGUAAUUGCGGUCUUUGGU	ATTATTTTGTTACAACATG	8
hsa-miR-487a	599	8:1:1	-4.1	-12.43	8.33	GUGGUUAUCCCUGCUGUGUUCG	TATGTGATTAAATAGAAAA	8
hsa-miR-369-3p	253	8:1:0	-3.4	-11.73	8.33	AAUAAUACAUGGUUGAUCUUU	TGTATAATAT	8
hsa-miR-410	144	8:1:0	-3.4	-11.75	8.35	AGGUUGUCUGUGAUGAGUUCG	TGATATATTTGTA	8
hsa-miR-369-3p	453	8:1:0	-2.8	-11.18	8.38	AAUAAUACAUGGUUGAUCUUU	TGTTTTATTTGTATAATA	8
hsa-miR-26a	80	8:1:1	-6.7	-15.08	8.38	UUCAAGUAAUCCAGGAUAGGCU	TTATTTGTATATTCA	8
hsa-miR-194	198	8:1:1	-5.4	-13.8	8.4	UGUAACAGCAACUCCAUGUGGA	GCTTTTATATAATA	8
hsa-miR-548l	907	8:1:1	-3.6	-12.02	8.42	AAAAGUAUUUGCGGGUUUUGUC	AATATTTATATGATA	8
hsa-miR-576-5p	895	8:1:1	-4.5	-12.92	8.42	AUUCUAAUUUCUCCACGUCUUU	TATTGGAATAACA	8
hsa-miR-539	575	8:1:1	-3.21	-11.63	8.42	GGAGAAAUUAUCCUUGGUGUGU	AATTTGTTTTATTTGTATA	8
hsa-miR-491-3p	1251	8:1:0	-2.4	-10.83	8.43	CUUAUGCAAGAUUCCCUUCUAC	TTGAATAACATAA	8
hsa-miR-577	927	8:1:1	-0.6	-9.03	8.43	UAGAUAAAAUAUUGGUACCUG	TTTTTTTTTTTT	8
hsa-miR-495	491	8:1:0	-5.3	-13.75	8.45	GAAGUUGCCCAUGUUAUUUUCG	TGTTTATTTG	8
hsa-miR-548h	985	8:1:1	-0.8	-9.26	8.46	AAAAGUAAUCGCGGUUUUUGUC	ATTCTTTTTAAG	8
hsa-miR-369-3p	588	8:1:0	-3.4	-11.87	8.47	AAUAAUACAUGGUUGAUCUUU	TGTATAATATGTATGTG	8
hsa-miR-190	1150	8:1:1	-7.5	-15.99	8.49	UGAUAUGUUUGAUAUAUUAGGU	AACATGTAATA	8
hsa-miR-548b-5p	1008	8:1:1	-2.3	-10.8	8.5	AAAAGUAAUUGUGGUUUUGGCC	ATTTTTTTTTAACG	8

hsa-miR-577	931	8:1:1	0	-8.52	8.52	UAGAUAAAAUAUUGGUACCUG	TTTTTTTTTT	8
hsa-miR-559	195	8:1:1	-4.02	-12.54	8.52	UAAAGUAAAUAUGCACCAAAA	TTGGCTTTTATATAATAT	8
hsa-miR-548d-5p	510	8:1:1	-6.4	-14.93	8.53	AAAAGUAAUUGUGGUUUUUGCC	ATTATTTGTATATATATT	8
hsa-miR-590-3p	1184	8:1:1	-3.6	-12.15	8.55	UAAUUUUAUGUAUAAGCUAGU	GTAAAATACCATAACT	8
hsa-miR-548n	1138	8:1:1	-5.7	-14.25	8.55	CAAAAGUAAUUGUGGAUUUUGU	TTATTTTGTTACAA	8
hsa-miR-374a	675	8:1:0	-3.5	-12.05	8.55	UUAUAAUACAACCUGAUAAGUG	ATATTATATGGTTCCA	8
hsa-miR-429	256	8:1:1	-1.4	-9.97	8.57	UAAUACUGUCUGGUAAAACCGU	ATAATATTTCTAA	8
hsa-miR-568	125	8:1:1	-4.75	-13.33	8.58	AUGUAUAAAUGUAUACACAC	TATATATATATTTT	8
hsa-miR-944	207	8:1:1	-3.5	-12.09	8.59	AAAUUAUUGUACAUCGGAUGAG	TAATATTTGTA	8
hsa-miR-369-3p	1171	8:1:0	-5.77	-14.38	8.61	AAUAAUACAUGGUUGAUCUUU	TGTATCATAAGTTG	8
hsa-miR-105	208	8:1:1	-2.9	-11.51	8.61	UCAAAUGCUCAGACUCCUGUGGU	AATATTTGTAAATATATA	8
hsa-miR-380	99	8:1:1	-3.3	-11.92	8.62	UGGUUGACCAUAGAACAUGCGC	TATAATATAAATA	8
hsa-miR-30a	134	8:1:1	-2.71	-11.34	8.63	UGUAAACAUCCUCGACUGGAAG	ATTTTTATTTTGATATAT	8
hsa-miR-30d	134	8:1:1	-2.71	-11.34	8.63	UGUAAACAUCCCCGACUGGAAG	ATTTTTATTTTGATAT	8
hsa-miR-30e	134	8:1:1	-2.71	-11.34	8.63	UGUAAACAUCCUUGACUGGAAG	ATTTTTATTTTGATATATT	8
hsa-miR-577	925	8:1:1	-0.23	-8.87	8.64	UAGAUAAAAUAUUGGUACCUG	TTTTTTTTTTTTTTTTTT	8
hsa-miR-548n	511	8:1:1	-6.2	-14.84	8.64	CAAAAGUAAUUGUGGAUUUUGU	TTATTTGTATATATATT	8

hsa-miR-548c-3p	558	8:1:1	-2.8	-11.45	8.65	CAAAAAUCUCAAUUACUUUUGC	GGATATTTGTATAT	8
hsa-miR-653	1250	8:1:1	-2.2	-10.86	8.66	GUGUUGAAACAAUCUCUACUG	TTTGAATAACATAAAA	8
hsa-miR-590-5p	1176	8:1:1	-5.8	-14.48	8.68	GAGCUUAUUCAUAAAAGUGCAG	CATAAGTTGTAAAATACCA	8
hsa-miR-577	930	8:1:1	-0.6	-9.28	8.68	UAGAUAAAAUAUUGGUACCUG	TTTTTTTTTTTTTTTT	8
hsa-miR-1246	115	8:1:1	-3.41	-12.09	8.68	AAUGGAUUUUUGGAGCAGG	ATATTCATAGTATATAT	8
hsa-miR-491-3p	1182	8:1:1	-3.5	-12.19	8.69	CUUAUGCAAGAUUCCCUUCUAC	TTGTAAAATAC	8
hsa-miR-190b	1150	8:1:1	-7.3	-15.99	8.69	UGAUAUGUUUGAUAUUGGGUU	AACATGTAAT	8
hsa-miR-655	1113	8:1:0	-2.4	-11.09	8.69	AGAGGUUAUCCGUGUUAUGUUC	ATTTATTAAAA	8
hsa-miR-577	933	8:1:1	0.26	-8.43	8.69	UAGAUAAAAUAUUGGUACCUG	TTTTTTTTTTTTTGTA	8
hsa-miR-577	932	8:1:1	0.21	-8.48	8.69	UAGAUAAAAUAUUGGUACCUG	TTTTTTTTTTTTTTGTAT	8
hsa-miR-1305	1180	8:1:0	-5.1	-13.8	8.7	UUUUCAACUCUAAUGGGAGAGA	AGTTGTAAAATACCATAAC	8
hsa-miR-1246	217	8:1:1	-2.69	-11.39	8.7	AAUGGAUUUUUGGAGCAGG	AAATATATATATATATATA	8
hsa-miR-577	934	8:1:1	0.3	-8.41	8.71	UAGAUAAAAUAUUGGUACCUG	TTTTTTTTTTT	8
hsa-miR-548j	510	8:1:1	-6.2	-14.93	8.73	AAAAGUAAUUGCGGUCUUUGGU	ATTATTTGTATA	8
hsa-miR-548m	921	8:1:1	-2.4	-11.16	8.76	CAAAGGUAUUUGUGGUUUUUG	ATCCTTTTTTTTTTTT	8
hsa-miR-655	1125	8:1:0	-4.8	-13.57	8.77	AGAGGUUAUCCGUGUUAUGUUC	ATGTAGTAATTAA	8
hsa-miR-429	891	8:1:1	-3	-11.77	8.77	UAAUACUGUCUGGUAAAACCGU	ATTGTATTGGAA	8

hsa-miR-577	926	8:1:1	-0.3	-9.09	8.79	UAGAUAAAAUAUUGGUACCUG	TTTTTTTTTTTTTTTT	8
hsa-miR-380	466	8:1:1	-3.3	-12.09	8.79	UGGUUGACCAUAGAACAUGCGC	TAATATATATA	8
hsa-miR-592	1143	8:1:1	-4.9	-13.71	8.81	UUGUGUCAAUAUGCGAUGAUGU	TTGTTACAACATGTAAT	8
hsa-miR-548p	934	8:1:1	0.4	-8.41	8.81	UAGCAAAAACUGCAGUUACUUU	TTTTTTTTTTTTTGT	8
hsa-miR-548c-5p	137	8:1:1	-3.6	-12.41	8.81	AAAAGUAAUUGCGGUUUUUGCC	TTTATTTTGATATA	8
hsa-miR-548p	933	8:1:1	0.4	-8.43	8.83	UAGCAAAAACUGCAGUUACUUU	TTTTTTTTTTTTT	8
hsa-miR-568	518	8:1:1	-5.5	-14.33	8.83	AUGUAUAAAUGUAUACACAC	TATATATATT	8
hsa-miR-495	243	8:1:0	-3	-11.87	8.87	GAAGUUGCCCAUGUUAUUUUCG	TTTTTGTTTTTG	8
hsa-miR-568	127	8:1:1	-4.24	-13.11	8.87	AUGUAUAAAUGUAUACACAC	TATATATATTTTTATTTTG	8
hsa-miR-548p	932	8:1:1	0.4	-8.48	8.88	UAGCAAAAACUGCAGUUACUUU	TTTTTTTTTTTTTTT	8
hsa-miR-548a-5p	536	8:1:1	-2.2	-11.1	8.9	AAAAGUAAUUGCGAGUUUUACC	ATTTTTTTATTAATTTG	8
hsa-miR-548p	931	8:1:1	0.4	-8.52	8.92	UAGCAAAAACUGCAGUUACUUU	TTTTTTTTTTT	8
hsa-miR-32	462	8:1:1	-3.95	-12.88	8.93	UAUUGCACAUUACUAAGUUGCA	TGTATAATATATATAT	8
hsa-miR-548d-3p	246	8:1:0	-3	-11.93	8.93	CAAAAACCACAGUUUCUUUUGC	TTGTTTTTGTATAA	8
hsa-miR-186	1008	8:1:1	-1.86	-10.8	8.94	CAAAGAAUUCUCCUUUUGGGCU	ATTTTTTTTTAACGCTTCT	8
hsa-miR-624	234	8:1:1	-3.3	-12.24	8.94	UAGUACCAGUACCUUGUGUUCA	TAATTTGTTTTTTT	8
hsa-miR-410	676	8:1:0	-3.05	-12.02	8.97	AGGUUGUCUGUGAUGAGUUCG	TATTATATGGTTCCATAT	8

hsa-miR-548d-3p	196	8:1:0	-3.6	-12.58	8.98	CAAAAACCACAGUUUCUUUUGC	TGGCTTTTATATAA	8
hsa-miR-577	78	8:0:1	-6.6	-15.59	8.99	UAGAUAAAAUAUUGGUACCUG	TTTTATTTGTATATT	8
hsa-miR-548a-5p	1008	8:1:1	-1.8	-10.8	9	AAAAGUAAUUGCGAGUUUUACC	ATTTTTTTTTAAC	8
hsa-miR-380	464	8:1:1	-3.71	-12.71	9	UGGUUGACCAUAGAACAUGCGC	TATAATATAT	8
hsa-miR-548i	137	8:1:1	-3.4	-12.41	9.01	AAAAGUAAUUGCGGAUUUUGCC	TTTATTTTGATATA	8
hsa-miR-369-3p	579	8:1:0	-2.8	-11.82	9.02	AAUAAUACAUGGUUGAUCUUU	TGTTTTATTTGTA	8
hsa-miR-369-3p	674	8:1:0	-3.7	-12.74	9.04	AAUAAUACAUGGUUGAUCUUU	TATATTATATGGTTC	8
hsa-miR-142-5p	546	8:1:1	-0.85	-9.89	9.04	CAUAAAGUAGAAAGCACUACU	TAATTTGTTTTTGGATA	8
hsa-miR-607	529	8:1:1	-6.7	-15.74	9.04	GUUCAAAUCCAGAUCUAUAAC	TGTTTGAATT	8
hsa-miR-599	1144	8:1:1	-4.9	-13.96	9.06	GUUGUGUCAGUUUAUCAAAC	TGTTACAACATGTAATA	8
hsa-miR-577	935	8:1:1	0.35	-8.71	9.06	UAGAUAAAAUAUUGGUACCUG	TTTTTTTTTTTTGTATTCA	8
hsa-miR-369-3p	479	8:1:0	-4.6	-13.66	9.06	AAUAAUACAUGGUUGAUCUUU	TGTTTTATTTTGTGTT	8
hsa-miR-802	70	8:1:1	-8.3	-17.36	9.06	CAGUAACAAAGAUUCAUCCUUGU	TTGTTATATTTTATTTGTA	8
hsa-miR-606	1128	8:1:1	-3.4	-12.47	9.07	AAACUACUGAAAAUCAAAGAU	TAGTAATTAATTA	8
hsa-miR-548p	927	8:1:1	0.04	-9.03	9.07	UAGCAAAAACUGCAGUUACUUU	TTTTTTTTTTTTTT	8
hsa-miR-548a-5p	482	8:1:1	-5.47	-14.55	9.08	AAAAGUAAUUGCGAGUUUUACC	TTTATTTTGT	8
hsa-miR-548c-5p	282	8:1:1	-0.6	-9.69	9.09	AAAAGUAAUUGCGGUUUUUGCC	ATAATTTTTT	8

hsa-miR-548h	282	8:1:1	-0.6	-9.69	9.09	AAAAGUAAUCGCGGUUUUUGUC	ATAATTTTTTG	8
hsa-miR-548p	935	8:1:1	0.4	-8.71	9.11	UAGCAAAAACUGCAGUUACUUU	TTTTTTTTTTTTGTAT	8
hsa-miR-581	695	8:1:1	-4.27	-13.38	9.11	UCUUGUGUUCUCUAGAUCAGU	AAAATAAGCTAATATAATA	8
hsa-miR-144	524	8:1:1	-7	-16.11	9.11	GGAUAUCAUCAUAUACUGUAAG	TATTTTGTTTGAATTTTT	8
hsa-miR-1264	1017	8:1:1	-2.1	-11.27	9.17	CAAGUCUUAUUUGAGCACCUGUU	TAACGCTTCTAATTACG	8
hsa-miR-548i	482	8:1:1	-5.37	-14.55	9.18	AAAAGUAAUUGCGGAUUUUGCC	TTTATTTTGTGTTTATT	8
hsa-miR-377	528	8:1:0	-6.6	-15.79	9.19	AGAGGUUGCCCUUGGUGAAUUC	TTGTTTGAATTTTT	8
hsa-miR-548f	282	8:1:1	-0.5	-9.69	9.19	UGCAAAAGUAAUCACAGUUUUU	ATAATTTTTTGAGT	8
hsa-miR-186	536	8:1:1	-1.9	-11.1	9.2	CAAAGAAUUCUCCUUUUGGGCU	ATTTTTTTATTAATTTG	8
hsa-miR-630	1185	8:1:1	-2.8	-12.01	9.21	AGUAUUCUGUACCAGGGAAGGU	TAAAATACCAT	8
hsa-miR-649	1176	8:1:1	-5.25	-14.48	9.23	AAACCUGUGUUGUUCAAGAGUC	CATAAGTTGTAAAATACCA	8
hsa-miR-548m	146	8:1:1	-2.1	-11.34	9.24	CAAAGGUAUUUGUGGUUUUUG	ATATATTTGTATAAT	8
hsa-miR-548p	929	8:1:1	0.12	-9.13	9.25	UAGCAAAAACUGCAGUUACUUU	TTTTTTTTTT	8
hsa-miR-548p	928	8:1:1	0.08	-9.18	9.26	UAGCAAAAACUGCAGUUACUUU	TTTTTTTTTT	8
hsa-miR-505	490	8:1:1	-4.4	-13.66	9.26	GGGAGCCAGGAAGUAUUGAUGU	GTGTTTATTTGAC	8
hsa-miR-569	1131	8:1:1	-2.2	-11.47	9.27	AGUUAAUGAAUCCUGGAAAGU	TAATTAATTATTTTGTTA	8
hsa-miR-524-5p	163	8:1:1	-4.1	-13.37	9.27	CUACAAAGGGAAGCACUUUCUC	TATTTGTATATATATATAT	8

hsa-miR-520d-5p	163	8:1:1	-4.1	-13.37	9.27	CUACAAAGGGAAGCCCUUUC	TATTTGTATATATA	8
hsa-miR-590-3p	159	8:1:0	-3.6	-12.87	9.27	UAAUUUUAUGUAUAAGCUAGU	ATAATATTTG	8
hsa-miR-548c-5p	1008	8:1:1	-1.5	-10.8	9.3	AAAAGUAAUUGCGGUUUUUGCC	ATTTTTTTTTAA	8
hsa-miR-548i	1008	8:1:1	-1.5	-10.8	9.3	AAAAGUAAUUGCGGAUUUUGCC	ATTTTTTTTTAACGCTT	8
hsa-miR-548a-5p	137	8:1:1	-3.1	-12.41	9.31	AAAAGUAAUUGCGAGUUUUACC	TTTATTTTGATATA	8
hsa-miR-494	74	8:1:1	-5.1	-14.41	9.31	AGGUUGUCCGUGUUGUCUUCUCU	TATATTTTATT	8
hsa-miR-548l	74	8:1:1	-5.1	-14.41	9.31	AAAAGUAUUUGCGGGUUUUGUC	TATATTTTATTTGTAT	8
hsa-miR-656	877	8:1:0	-0.9	-10.23	9.33	AGGUUGCCUGUGAGGUGUUCA	TATCATATATAAT	8
hsa-miR-1259	100	8:1:0	-2.8	-12.13	9.33	UCCCUGAGACCCUUUAACCUGUGA	ATAATATAAA	8
hsa-miR-498	529	8:1:1	-6.4	-15.74	9.34	UUUCAAGCCAGGGGGCGUUUUUC	TGTTTGAATTTTTTT	8
hsa-miR-380	671	8:1:1	-2	-11.35	9.35	UGGUUGACCAUAGAACAUGCGC	TAGTATATTATATGGT	8
hsa-miR-1259	467	8:1:1	-2.7	-12.06	9.36	UCCCUGAGACCCUUUAACCUGUGA	AATATATATATATGTTT	8
hsa-miR-577	939	8:1:1	-0.08	-9.45	9.37	UAGAUAAAAUAUUGGUACCUG	TTTTTTTTGTATT	8
hsa-miR-302f	1132	8:1:1	-2.6	-11.98	9.38	UAAUUGCUUCCAUGUUU	AATTAATTATT	8
hsa-miR-300	82	8:1:1	-6	-15.38	9.38	UAUACAAGGGCAGACUCUCUCU	ATTTGTATATT	8
hsa-miR-1259	591	8:1:1	-2.3	-11.7	9.4	UCCCUGAGACCCUUUAACCUGUGA	ATAATATGTATGTGATT	8
hsa-miR-1278	905	8:1:1	-2.8	-12.21	9.41	UAGUACUGUGCAUAUCAUCUAU	ACAATATTTATA	8

hsa-miR-559	482	8:0:1	-5.13	-14.55	9.42	UAAAGUAAAUAUGCACCAAAA	TTTATTTTGTGTTT	8
hsa-miR-577	936	8:1:1	0.39	-9.04	9.43	UAGAUAAAAUAUUGGUACCUG	TTTTTTTTTTTGTAT	8
hsa-miR-369-3p	462	8:1:0	-3.45	-12.88	9.43	AAUAAUACAUGGUUGAUCUUU	TGTATAATATATA	8
hsa-miR-142-5p	234	8:1:1	-2.81	-12.24	9.43	CAUAAAGUAGAAAGCACUACU	TAATTTGTTTTTTTGT	8
hsa-miR-548p	936	8:1:1	0.4	-9.04	9.44	UAGCAAAAACUGCAGUUACUUU	TTTTTTTTTTTG	8
hsa-miR-142-5p	76	8:0:1	-5.7	-15.15	9.45	CAUAAAGUAGAAAGCACUACU	TATTTTATTTGTAT	8
hsa-miR-410	200	8:1:0	-4.6	-14.06	9.46	AGGUUGUCUGUGAUGAGUUCG	TTTTATATAATATTTGT	8
hsa-miR-32	588	8:1:1	-2.4	-11.87	9.47	UAUUGCACAUUACUAAGUUGCA	TGTATAATATGTAT	8
hsa-miR-200b	1085	8:1:1	-3.2	-12.68	9.48	CAUCUUACUGGGCAGCAUUGGA	GCGTTATTTGTTTATTGT	8
hsa-miR-519c-3p	90	8:1:1	-1.9	-11.43	9.53	AAAGUGCAUCUUUUUAGAGGAU	ATTCATTTGTATA	8
hsa-miR-519b-3p	90	8:1:1	-1.9	-11.43	9.53	AAAGUGCAUCCUUUUAGAGGUU	ATTCATTTGT	8
hsa-miR-524-5p	81	8:1:1	-5.8	-15.33	9.53	CUACAAAGGGAAGCACUUUCUC	TATTTGTATATTCATTTGT	8
hsa-miR-520d-5p	81	8:1:1	-5.8	-15.33	9.53	CUACAAAGGGAAGCCCUUUC	TATTTGTATATTCATTTGT	8
hsa-miR-1259	465	8:1:0	-2.6	-12.15	9.55	UCCCUGAGACCCUUUAACCUGUGA	ATAATATATATAT	8
hsa-miR-656	142	8:1:1	-2.5	-12.05	9.55	AGGUUGCCUGUGAGGUGUUCA	TTTGATATATTTGTATAA	8
hsa-miR-106b	1005	8:1:1	-1.4	-10.97	9.57	UAAAGUGCUGACAGUGCAGAU	AGAATTTTTTTTAACGC	8
hsa-miR-548b-5p	282	8:1:1	-0.1	-9.69	9.59	AAAAGUAAUUGUGGUUUUGGCC	ATAATTTTTTGAGTAA	8

hsa-miR-548d-5p	282	8:1:1	-0.1	-9.69	9.59	AAAAGUAAUUGUGGUUUUUGCC	ATAATTTTTTGA	8
hsa-miR-548m	282	8:1:1	-0.1	-9.69	9.59	CAAAGGUAUUUGUGGUUUUUG	ATAATTTTTTGAGTA	8
hsa-miR-548j	1008	8:1:1	-1.2	-10.8	9.6	AAAAGUAAUUGCGGUCUUUGGU	ATTTTTTTTTAACGC	8
hsa-miR-641	186	8:1:1	-4	-13.61	9.61	AAAGACAUAGGAUAGAGUCACCUC	TTTGTTTTTTTGGC	8
hsa-miR-548a-5p	510	8:1:1	-5.3	-14.93	9.63	AAAAGUAAUUGCGAGUUUUACC	ATTATTTGTA	8
hsa-miR-548l	131	8:1:1	-3.4	-13.05	9.65	AAAAGUAUUUGCGGGUUUUGUC	TATATTTTTATT	8
hsa-miR-1259	108	8:1:1	-2.7	-12.36	9.66	UCCCUGAGACCCUUUAACCUGUGA	AATATATATATTCATAGTA	8
hsa-miR-891a	1142	8:1:1	-3.6	-13.27	9.67	UGCAACGAACCUGAGCCACUGA	TTTGTTACAACATGTA	8
hsa-miR-34b	1128	8:1:0	-2.8	-12.47	9.67	UAGGCAGUGUCAUUAGCUGAUUG	TAGTAATTAATTATTTTG	8
hsa-miR-548p	930	8:1:1	0.4	-9.28	9.68	UAGCAAAAACUGCAGUUACUUU	TTTTTTTTTTTTTTTTTG	8
hsa-miR-548a-5p	48	8:1:1	-7.3	-16.98	9.68	AAAAGUAAUUGCGAGUUUUACC	TTTATTTTTTTTTTTTT	8
hsa-miR-581	1174	8:1:1	-4.8	-14.49	9.69	UCUUGUGUUCUCUAGAUCAGU	ATCATAAGTTGTA	8
hsa-miR-433	1173	8:1:1	-4.8	-14.49	9.69	UACGGUGAGCCUGUCAUUAUUC	TATCATAAGTTGT	8
hsa-miR-1259	1150	8:1:1	-6.3	-15.99	9.69	UCCCUGAGACCCUUUAACCUGUGA	AACATGTAAT	8
hsa-miR-577	492	8:1:1	-3.7	-13.39	9.69	UAGAUAAAAUAUUGGUACCUG	GTTTATTTGACACATTTG	8
hsa-miR-548j	137	8:1:1	-2.7	-12.41	9.71	AAAAGUAAUUGCGGUCUUUGGU	TTTATTTTGATA	8
hsa-miR-548h	137	8:1:1	-2.7	-12.41	9.71	AAAAGUAAUCGCGGUUUUUGUC	TTTATTTTGATATATT	8

hsa-miR-142-5p	674	8:1:1	-3.01	-12.74	9.73	CAUAAAGUAGAAAGCACUACU	TATATTATATGGTTCCATA	8
hsa-miR-548n	1009	8:1:1	-3.2	-12.96	9.76	CAAAAGUAAUUGUGGAUUUUGU	TTTTTTTTTAACG	8
hsa-miR-363	1152	8:1:1	-5.2	-14.98	9.78	CGGGUGGAUCACGAUGCAAUUU	CATGTAATAATAA	8
hsa-miR-381	82	8:1:1	-5.6	-15.38	9.78	AGCGAGGUUGCCCUUUGUAUAU	ATTTGTATAT	8
hsa-miR-548j	544	8:1:0	-0.34	-10.14	9.8	AAAAGUAAUUGCGGUCUUUGGU	ATTAATTTGTTTTTGGA	8
hsa-miR-548p	937	8:1:1	0.4	-9.43	9.83	UAGCAAAAACUGCAGUUACUUU	TTTTTTTTTTGTATTC	8
hsa-miR-374b	1172	8:1:0	-4.5	-14.34	9.84	AUAUAAUACAACCUGCUAAGUG	GTATCATAAGTTGT	8
hsa-miR-641	522	8:1:1	-5	-14.84	9.84	AAAGACAUAGGAUAGAGUCACCUC	TATATTTTGTTTGAAT	8
hsa-miR-548m	1109	8:1:1	-1.65	-11.5	9.85	CAAAGGUAUUUGUGGUUUUUG	ATATATTTATTAAAAT	8
hsa-miR-548p	939	8:1:1	0.4	-9.45	9.85	UAGCAAAAACUGCAGUUACUUU	TTTTTTTTGTATTC	8
hsa-miR-495	188	8:1:0	-3.37	-13.22	9.85	GAAGUUGCCCAUGUUAUUUUCG	TGTTTTTTTG	8
hsa-miR-380	200	8:1:1	-4.2	-14.06	9.86	UGGUUGACCAUAGAACAUGCGC	TTTTATATAATATTTGTAA	8
hsa-miR-30a	45	8:1:1	-4.8	-14.66	9.86	UGUAAACAUCCUCGACUGGAAG	TTGTTTATTTTTTTTTT	8
hsa-miR-30c	45	8:1:1	-4.8	-14.66	9.86	UGUAAACAUCCUACACUCUCAGC	TTGTTTATTTTTTTTTTT	8
hsa-miR-30d	45	8:1:1	-4.8	-14.66	9.86	UGUAAACAUCCCCGACUGGAAG	TTGTTTATTTTT	8
hsa-miR-30b	45	8:1:1	-4.8	-14.66	9.86	UGUAAACAUCCUACACUCAGCU	TTGTTTATTTTTTT	8
hsa-miR-30e	45	8:1:1	-4.8	-14.66	9.86	UGUAAACAUCCUUGACUGGAAG	TTGTTTATTTTTTTTTTTT	8

hsa-miR-577	937	8:1:1	0.44	-9.43	9.87	UAGAUAAAAUAUUGGUACCUG	TTTTTTTTTTGTATTCAT	8
hsa-miR-425	1171	8:1:1	-4.5	-14.38	9.88	AAUGACACGAUCACUCCCGUUGA	TGTATCATAAGT	8
hsa-miR-30a	1111	8:1:1	-1.01	-10.89	9.88	UGUAAACAUCCUCGACUGGAAG	ATATTTATTAAAATATG	8
hsa-miR-944	1156	8:1:1	-3.46	-13.35	9.89	AAAUUAUUGUACAUCGGAUGAG	TAATAATAAAATT	8
hsa-miR-548h	1008	8:1:1	-0.9	-10.8	9.9	AAAAGUAAUCGCGGUUUUUGUC	ATTTTTTTTTAACGCTTC	8
hsa-miR-548p	539	8:1:1	-0.7	-10.6	9.9	UAGCAAAAACUGCAGUUACUUU	TTTTTATTAATTTG	8
hsa-miR-1246	1164	8:1:1	-2.8	-12.71	9.91	AAUGGAUUUUUGGAGCAGG	AAATTAATGTATCATA	8
hsa-miR-559	934	8:1:1	1.5	-8.41	9.91	UAAAGUAAAUAUGCACCAAAA	TTTTTTTTTTTT	8
hsa-miR-559	933	8:1:1	1.5	-8.43	9.93	UAAAGUAAAUAUGCACCAAAA	TTTTTTTTTTTTTTG	8
hsa-miR-1264	160	8:1:1	-3.6	-13.53	9.93	CAAGUCUUAUUUGAGCACCUGUU	TAATATTTGTA	8
hsa-miR-374a	1172	8:1:0	-4.4	-14.34	9.94	UUAUAAUACAACCUGAUAAGUG	GTATCATAAGTTG	8
hsa-miR-559	932	8:1:1	1.5	-8.48	9.98	UAAAGUAAAUAUGCACCAAAA	TTTTTTTTTTTT	8
hsa-miR-891b	234	8:1:1	-2.25	-12.24	9.99	UGCAACUUACCUGAGUCAUUGA	TAATTTGTTTTTTT	8
hsa-miR-491-3p	202	8:1:1	-2.6	-12.59	9.99	CUUAUGCAAGAUUCCCUUCUAC	TTATATAATATT	8
hsa-miR-656	1269	8:1:1	-1.4	-11.4	10	AGGUUGCCUGUGAGGUGUUCA	TAGAATGTATTATGTAATA	8
hsa-miR-548p	938	8:1:1	0.4	-9.6	10	UAGCAAAAACUGCAGUUACUUU	TTTTTTTTTGTATT	8
hsa-miR-548c-3p	1006	8:1:1	-0.4	-10.41	10.01	CAAAAAUCUCAAUUACUUUUGC	GAATTTTTTTTTAACGC	8

hsa-miR-802	238	8:1:1	-3.3	-13.31	10.01	CAGUAACAAAGAUUCAUCCUUGU	TTGTTTTTTTGTTT	8
hsa-miR-559	931	8:1:1	1.5	-8.52	10.02	UAAAGUAAAUAUGCACCAAAA	TTTTTTTTTTTTTTTTGTA	8
hsa-miR-548f	132	8:1:1	-2.8	-12.84	10.04	UGCAAAAGUAAUCACAGUUUUU	ATATTTTTATT	8
hsa-miR-656	120	8:1:1	-3.7	-13.74	10.04	AGGUUGCCUGUGAGGUGUUCA	CATAGTATAT	8
hsa-miR-496	886	8:1:1	-2	-12.06	10.06	UGAGUAUUACAUGGCCAAUCUC	TAATAATTGTAT	8
hsa-miR-137	1153	8:1:1	-4.85	-14.92	10.07	UUAUUGCUUAAGAAUACGCGUAG	ATGTAATAATAAAA	8
hsa-miR-577	938	8:1:1	0.48	-9.6	10.08	UAGAUAAAAUAUUGGUACCUG	TTTTTTTTTGTATT	8
hsa-miR-624	183	8:1:1	-3.6	-13.7	10.1	UAGUACCAGUACCUUGUGUUCA	TAATTTGTTTTTTTGGCT	8
hsa-miR-548l	1137	8:1:1	-3.8	-13.92	10.12	AAAAGUAUUUGCGGGUUUUGUC	ATTATTTTGTTACA	8
hsa-miR-1259	1106	8:1:0	-2.96	-13.08	10.12	UCCCUGAGACCCUUUAACCUGUGA	ATAATATATTTAT	8
hsa-miR-494	237	8:1:1	-3.4	-13.56	10.16	AGGUUGUCCGUGUUGUCUUCUCU	TTTGTTTTTTTGT	8
hsa-miR-559	935	8:1:1	1.5	-8.71	10.21	UAAAGUAAAUAUGCACCAAAA	TTTTTTTTTTTTGTATTCA	8
hsa-miR-494	186	8:1:1	-3.4	-13.61	10.21	AGGUUGUCCGUGUUGUCUUCUCU	TTTGTTTTTTTGGCTTT	8
hsa-miR-548c-3p	132	8:1:0	-2.62	-12.84	10.22	CAAAAAUCUCAAUUACUUUUGC	ATATTTTTATTT	8
hsa-miR-577	250	8:1:1	-1.9	-12.13	10.23	UAGAUAAAAUAUUGGUACCUG	TTTTGTATAATATTT	8
hsa-miR-548p	135	8:1:1	-1.26	-11.52	10.26	UAGCAAAAACUGCAGUUACUUU	TTTTTATTTTGA	8
hsa-miR-664	1044	8:1:0	0	-10.27	10.27	ACUGGCUAGGGAAAAUGAUUGGAU	AAATAAATGAT	8

hsa-miR-186	575	8:1:1	-1.36	-11.63	10.27	CAAAGAAUUCUCCUUUUGGGCU	AATTTGTTTTATTTGTAT	8
hsa-miR-524-5p	199	8:1:1	-4	-14.27	10.27	CUACAAAGGGAAGCACUUUCUC	CTTTTATATAAT	8
hsa-miR-548c-5p	536	8:1:1	-0.8	-11.1	10.3	AAAAGUAAUUGCGGUUUUUGCC	ATTTTTTTATTAATTTGT	8
hsa-miR-548j	536	8:1:1	-0.8	-11.1	10.3	AAAAGUAAUUGCGGUCUUUGGU	ATTTTTTTATTAATTTGT	8
hsa-miR-655	1170	8:1:0	-4.1	-14.42	10.32	AGAGGUUAUCCGUGUUAUGUUC	ATGTATCATAAGTTG	8
hsa-miR-1302	1170	8:1:1	-4.1	-14.42	10.32	UUGGGACAUACUUAUGCUAAA	ATGTATCATAAGTTG	8
hsa-miR-559	1137	8:1:1	-3.6	-13.92	10.32	UAAAGUAAAUAUGCACCAAAA	ATTATTTTGT	8
hsa-miR-559	925	8:1:1	1.45	-8.87	10.32	UAAAGUAAAUAUGCACCAAAA	TTTTTTTTTTT	8
hsa-miR-494	681	8:1:0	-3.7	-14.02	10.32	AGGUUGUCCGUGUUGUCUUCUCU	TATGGTTCCAT	8
hsa-miR-559	939	8:1:1	0.9	-9.45	10.35	UAAAGUAAAUAUGCACCAAAA	TTTTTTTTGTAT	8
hsa-miR-181a	532	8:1:0	-5.46	-15.81	10.35	AACAUUCAACGCUGUCGGUGAGU	TTGAATTTTTTTATTAA	8
hsa-miR-548c-5p	482	8:1:1	-4.2	-14.55	10.35	AAAAGUAAUUGCGGUUUUUGCC	TTTATTTTGTGTTTA	8
hsa-miR-548j	482	8:1:1	-4.2	-14.55	10.35	AAAAGUAAUUGCGGUCUUUGGU	TTTATTTTGTGTTTA	8
hsa-miR-30d	1111	8:1:1	-0.51	-10.89	10.38	UGUAAACAUCCCCGACUGGAAG	ATATTTATTAAAATATGTA	8
hsa-miR-30c	1111	8:1:1	-0.5	-10.89	10.39	UGUAAACAUCCUACACUCUCAGC	ATATTTATTAAA	8
hsa-miR-30b	1111	8:1:1	-0.5	-10.89	10.39	UGUAAACAUCCUACACUCAGCU	ATATTTATTAAAATATGTA	8
hsa-miR-30e	1111	8:1:1	-0.5	-10.89	10.39	UGUAAACAUCCUUGACUGGAAG	ATATTTATTAAAATATGTA	8

hsa-miR-559	926	8:1:1	1.31	-9.09	10.4	UAAAGUAAAUAUGCACCAAAA	TTTTTTTTTTTTTTT	8
hsa-miR-361-5p	1141	8:1:1	-2.76	-13.17	10.41	UUAUCAGAAUCUCCAGGGGUAC	TTTTGTTACAAC	8
hsa-miR-520d-5p	512	8:1:1	-4.1	-14.52	10.42	CUACAAAGGGAAGCCCUUUC	TATTTGTATAT	8
hsa-miR-655	673	8:1:1	-2	-12.43	10.43	AGAGGUUAUCCGUGUUAUGUUC	GTATATTATA	8
hsa-miR-495	239	8:1:0	-2.2	-12.63	10.43	GAAGUUGCCCAUGUUAUUUUCG	TGTTTTTTTGTTTTTGTA	8
hsa-miR-802	187	8:1:1	-3.3	-13.76	10.46	CAGUAACAAAGAUUCAUCCUUGU	TTGTTTTTTTGGCTTTT	8
hsa-miR-548m	130	8:1:1	-2.7	-13.18	10.48	CAAAGGUAUUUGUGGUUUUUG	ATATATTTTTA	8
hsa-miR-410	71	8:0:0	-6.3	-16.78	10.48	AGGUUGUCUGUGAUGAGUUCG	TGTTATATTTTATT	8
hsa-miR-548b-5p	536	8:1:1	-0.6	-11.1	10.5	AAAAGUAAUUGUGGUUUUGGCC	ATTTTTTTATTA	8
hsa-miR-548d-5p	536	8:1:1	-0.6	-11.1	10.5	AAAAGUAAUUGUGGUUUUUGCC	ATTTTTTTATTAA	8
hsa-miR-548k	1137	8:1:1	-3.4	-13.92	10.52	AAAAGUACUUGCGGAUUUUGCU	ATTATTTTGTT	8
hsa-miR-548h	1137	8:0:1	-3.4	-13.92	10.52	AAAAGUAAUCGCGGUUUUUGUC	ATTATTTTGT	8
hsa-miR-559	927	8:1:1	1.5	-9.03	10.53	UAAAGUAAAUAUGCACCAAAA	TTTTTTTTTTTTT	8
hsa-miR-30a	908	8:1:1	-1.1	-11.63	10.53	UGUAAACAUCCUCGACUGGAAG	ATATTTATATGA	8
hsa-miR-30c	908	8:1:1	-1.1	-11.63	10.53	UGUAAACAUCCUACACUCUCAGC	ATATTTATATGATATCCTT	8
hsa-miR-30d	908	8:1:1	-1.1	-11.63	10.53	UGUAAACAUCCCCGACUGGAAG	ATATTTATATGATATCC	8
hsa-miR-30b	908	8:1:1	-1.1	-11.63	10.53	UGUAAACAUCCUACACUCAGCU	ATATTTATATGATA	8

hsa-miR-30e	908	8:1:1	-1.1	-11.63	10.53	UGUAAACAUCCUUGACUGGAAG	ATATTTATATGAT	8
hsa-miR-559	936	8:1:1	1.5	-9.04	10.54	UAAAGUAAAUAUGCACCAAAA	TTTTTTTTTTTGTATTCA	8
hsa-miR-548h	544	8:1:0	0.4	-10.14	10.54	AAAAGUAAUCGCGGUUUUUGUC	ATTAATTTGTTT	8
hsa-miR-380	1107	8:1:1	-2.2	-12.75	10.55	UGGUUGACCAUAGAACAUGCGC	TAATATATTTATTAAAA	8
hsa-miR-656	232	8:1:0	-1.7	-12.25	10.55	AGGUUGCCUGUGAGGUGUUCA	TATAATTTGTTTTT	8
hsa-miR-186	449	8:1:1	-0.5	-11.09	10.59	CAAAGAAUUCUCCUUUUGGGCU	AATTTGTTTTATTTGTATA	8
hsa-miR-559	929	8:1:1	1.5	-9.13	10.63	UAAAGUAAAUAUGCACCAAAA	TTTTTTTTTT	8
hsa-miR-548c-5p	510	8:1:1	-4.3	-14.93	10.63	AAAAGUAAUUGCGGUUUUUGCC	ATTATTTGTATATATAT	8
hsa-miR-548i	510	8:1:1	-4.3	-14.93	10.63	AAAAGUAAUUGCGGAUUUUGCC	ATTATTTGTAT	8
hsa-miR-577	486	8:1:1	-4.3	-14.96	10.66	UAGAUAAAAUAUUGGUACCUG	TTTTGTGTTTATTTGACAC	8
hsa-miR-559	928	8:1:1	1.5	-9.18	10.68	UAAAGUAAAUAUGCACCAAAA	TTTTTTTTTTTTTTTT	8
hsa-miR-651	1173	8:1:0	-3.8	-14.49	10.69	UUUAGGAUAAGCUUGACUUUUG	TATCATAAGTTGTAAAA	8
hsa-miR-495	46	8:1:0	-4.8	-15.52	10.72	GAAGUUGCCCAUGUUAUUUUCG	TGTTTATTTTTTTTT	8
hsa-miR-181c	532	8:1:0	-5.06	-15.81	10.75	AACAUUCAACCUGUCGGUGAGU	TTGAATTTTT	8
hsa-miR-559	79	8:1:1	-4.2	-14.97	10.77	UAAAGUAAAUAUGCACCAAAA	TTTATTTGTA	8
hsa-miR-487a	1171	8:1:0	-3.6	-14.38	10.78	GUGGUUAUCCCUGCUGUGUUCG	TGTATCATAAGTTGTAAA	8
hsa-miR-559	930	8:1:1	1.5	-9.28	10.78	UAAAGUAAAUAUGCACCAAAA	TTTTTTTTTTTTTTTT	8

hsa-miR-1259	123	8:1:1	-2.6	-13.39	10.79	UCCCUGAGACCCUUUAACCUGUGA	AGTATATATATATTT	8
hsa-miR-410	479	8:1:0	-2.86	-13.66	10.8	AGGUUGUCUGUGAUGAGUUCG	TGTTTTATTTT	8
hsa-miR-548g	131	8:1:1	-2.25	-13.05	10.8	UGCAAAAGUAAUUGCAGUUUUUG	TATATTTTTATT	8
hsa-miR-656	1183	8:1:1	-1.4	-12.22	10.82	AGGUUGCCUGUGAGGUGUUCA	TGTAAAATACCATAA	8
hsa-miR-548h	482	8:1:1	-3.7	-14.55	10.85	AAAAGUAAUCGCGGUUUUUGUC	TTTATTTTGTGT	8
hsa-miR-548d-3p	238	8:1:0	-2.4	-13.31	10.91	CAAAAACCACAGUUUCUUUUGC	TTGTTTTTTTGTTTT	8
hsa-miR-656	1154	8:1:1	-3.8	-14.72	10.92	AGGUUGCCUGUGAGGUGUUCA	TGTAATAATAAAA	8
hsa-miR-559	937	8:1:1	1.5	-9.43	10.93	UAAAGUAAAUAUGCACCAAAA	TTTTTTTTTTGT	8
hsa-miR-30c	134	8:1:1	-0.4	-11.34	10.94	UGUAAACAUCCUACACUCUCAGC	ATTTTTATTT	8
hsa-miR-30b	134	8:1:1	-0.4	-11.34	10.94	UGUAAACAUCCUACACUCAGCU	ATTTTTATTTTGA	8
hsa-miR-561	131	8:1:1	-2.1	-13.05	10.95	AUCAAGGAUCUUAAACUUUGCC	TATATTTTTATTTTG	8
hsa-miR-487b	1171	8:1:1	-3.4	-14.38	10.98	GUGGUUAUCCCUGUCCUGUUCG	TGTATCATAAGTTGTAAAA	8
hsa-miR-105	161	8:1:1	-2.9	-13.88	10.98	UCAAAUGCUCAGACUCCUGUGGU	AATATTTGTATATATATAT	8
hsa-miR-1297	80	8:1:1	-4.1	-15.08	10.98	UUCAAGUAAUUCAGGUG	TTATTTGTATATTCATT	8
hsa-miR-1246	107	8:1:1	-1.6	-12.63	11.03	AAUGGAUUUUUGGAGCAGG	AAATATATATATTCAT	8
hsa-miR-633	133	8:1:1	-1.2	-12.25	11.05	CUAAUAGUAUCUACCACAAUAAA	TATTTTTATTTT	8
hsa-miR-944	1135	8:1:1	-1.9	-12.98	11.08	AAAUUAUUGUACAUCGGAUGAG	TAATTATTTTG	8

hsa-miR-559	938	8:1:1	1.5	-9.6	11.1	UAAAGUAAAUAUGCACCAAAA	TTTTTTTTTGTATTCATAC	8
hsa-miR-577	193	8:1:1	-0.7	-11.81	11.11	UAGAUAAAAUAUUGGUACCUG	TTTTGGCTTTTATATAA	8
hsa-miR-656	676	8:1:0	-0.8	-12.02	11.22	AGGUUGCCUGUGAGGUGUUCA	TATTATATGGTTCCATA	8
hsa-miR-144	522	8:1:1	-3.6	-14.84	11.24	GGAUAUCAUCAUAUACUGUAAG	TATATTTTGTTTG	8
hsa-miR-577	1009	8:1:1	-1.7	-12.96	11.26	UAGAUAAAAUAUUGGUACCUG	TTTTTTTTTAACG	8
hsa-miR-548p	67	8:0:1	-5.3	-16.6	11.3	UAGCAAAAACUGCAGUUACUUU	TTTTTGTTAT	8
hsa-miR-548n	483	8:1:1	-3.2	-14.52	11.32	CAAAAGUAAUUGUGGAUUUUGU	TTATTTTGTGTTTATTTGA	8
hsa-miR-802	135	8:1:1	-0.2	-11.52	11.32	CAGUAACAAAGAUUCAUCCUUGU	TTTTTATTTTGATATATT	8
hsa-miR-888	486	8:1:0	-3.6	-14.96	11.36	UACUCAAAAAGCUGUCAGUCA	TTTTGTGTTTATT	8
hsa-miR-548d-3p	187	8:1:0	-2.4	-13.76	11.36	CAAAAACCACAGUUUCUUUUGC	TTGTTTTTTT	8
hsa-miR-548b-5p	482	8:1:1	-3.1	-14.55	11.45	AAAAGUAAUUGUGGUUUUGGCC	TTTATTTTGTGTTTA	8
hsa-miR-548d-5p	482	8:1:1	-3.1	-14.55	11.45	AAAAGUAAUUGUGGUUUUUGCC	TTTATTTTGTGTT	8
hsa-miR-548g	232	8:1:1	-0.8	-12.25	11.45	UGCAAAAGUAAUUGCAGUUUUUG	TATAATTTGTTTT	8
hsa-miR-548p	525	8:1:1	-4.4	-15.93	11.53	UAGCAAAAACUGCAGUUACUUU	ATTTTGTTTGAATTT	8
hsa-miR-548e	237	8:1:1	-1.95	-13.56	11.61	CAAAAGCAAUCGCGGUUUUUGC	TTTGTTTTTTTG	8
hsa-miR-548g	181	8:1:1	-2.39	-14.03	11.64	UGCAAAAGUAAUUGCAGUUUUUG	TATAATTTGTTTTTTT	8
hsa-miR-548n	49	8:0:1	-5.7	-17.36	11.66	CAAAAGUAAUUGUGGAUUUUGU	TTATTTTTTTTTTTTTTTT	8

hsa-miR-559	48	8:0:1	-5.3	-16.98	11.68	UAAAGUAAAUAUGCACCAAAA	TTTATTTTTTTTT	8
hsa-miR-548h	536	8:1:1	0.6	-11.1	11.7	AAAAGUAAUCGCGGUUUUUGUC	ATTTTTTTATTA	8
hsa-miR-891b	183	8:1:1	-2	-13.7	11.7	UGCAACUUACCUGAGUCAUUGA	TAATTTGTTTTTTTGG	8
hsa-miR-548e	186	8:1:1	-1.9	-13.61	11.71	CAAAAGCAAUCGCGGUUUUUGC	TTTGTTTTTTTG	8
hsa-miR-520d-5p	199	8:1:1	-2.5	-14.27	11.77	CUACAAAGGGAAGCCCUUUC	CTTTTATATA	8
hsa-miR-144	74	8:1:1	-2.6	-14.41	11.81	GGAUAUCAUCAUAUACUGUAAG	TATATTTTATTTGTATAT	8
hsa-miR-374a	70	8:1:1	-5.53	-17.36	11.83	UUAUAAUACAACCUGAUAAGUG	TTGTTATATTT	8
hsa-miR-186	235	8:1:1	-0.4	-12.25	11.85	CAAAGAAUUCUCCUUUUGGGCU	AATTTGTTTTTTTG	8
hsa-miR-548n	80	8:1:1	-3.2	-15.08	11.88	CAAAAGUAAUUGUGGAUUUUGU	TTATTTGTATATTCATTT	8
hsa-miR-494	50	8:1:1	-5.6	-17.48	11.88	AGGUUGUCCGUGUUGUCUUCUCU	TATTTTTTTTT	8
hsa-miR-548m	521	8:1:1	-2.6	-14.61	12.01	CAAAGGUAUUUGUGGUUUUUG	ATATATTTTGTTTGAA	8
hsa-miR-548d-3p	239	8:1:0	-0.6	-12.63	12.03	CAAAAACCACAGUUUCUUUUGC	TGTTTTTTTGT	8
hsa-miR-577	47	8:1:1	-4.3	-16.37	12.07	UAGAUAAAAUAUUGGUACCUG	GTTTATTTTTTTTTTTT	8
hsa-miR-548g	74	8:1:1	-2.3	-14.41	12.11	UGCAAAAGUAAUUGCAGUUUUUG	TATATTTTATTTG	8
hsa-miR-641	74	8:1:1	-2.2	-14.41	12.21	AAAGACAUAGGAUAGAGUCACCUC	TATATTTTATTTGTATA	8
hsa-miR-548a-5p	51	8:1:1	-5.1	-17.31	12.21	AAAAGUAAUUGCGAGUUUUACC	ATTTTTTTTTTTTTTTTT	8
hsa-miR-577	1010	8:1:1	-1.1	-13.32	12.22	UAGAUAAAAUAUUGGUACCUG	TTTTTTTTAACGC	8

hsa-miR-577	200	8:1:0	-1.8	-14.06	12.26	UAGAUAAAAUAUUGGUACCUG	TTTTATATAATATTTGTA	8
hsa-miR-548l	535	8:1:1	-1.2	-13.47	12.27	AAAAGUAUUUGCGGGUUUUGUC	AATTTTTTTATTAATTT	8
hsa-miR-656	181	8:1:0	-1.7	-14.03	12.33	AGGUUGCCUGUGAGGUGUUCA	TATAATTTGTTTTTTTGG	8
hsa-miR-641	131	8:1:1	-0.7	-13.05	12.35	AAAGACAUAGGAUAGAGUCACCUC	TATATTTTTATTTTGATAT	8
hsa-miR-559	74	8:1:1	-2	-14.41	12.41	UAAAGUAAAUAUGCACCAAAA	TATATTTTATTTG	8
hsa-miR-561	74	8:1:1	-1.9	-14.41	12.51	AUCAAGGAUCUUAAACUUUGCC	TATATTTTATT	8
hsa-miR-548p	487	8:1:1	-2.06	-14.67	12.61	UAGCAAAAACUGCAGUUACUUU	TTTGTGTTTATTTGACA	8
hsa-miR-548d-3p	188	8:1:0	-0.6	-13.22	12.62	CAAAAACCACAGUUUCUUUUGC	TGTTTTTTTGG	8
hsa-miR-548g	522	8:1:1	-2.2	-14.84	12.64	UGCAAAAGUAAUUGCAGUUUUUG	TATATTTTGTTTGAATTT	8
hsa-miR-494	522	8:1:1	-2.15	-14.84	12.69	AGGUUGUCCGUGUUGUCUUCUCU	TATATTTTGTTT	8
hsa-miR-664	1164	8:1:0	0	-12.71	12.71	ACUGGCUAGGGAAAAUGAUUGGAU	AAATTAATGTATCATAAGT	8
hsa-miR-380	76	8:1:1	-2.3	-15.15	12.85	UGGUUGACCAUAGAACAUGCGC	TATTTTATTTGTAT	8
hsa-miR-548n	55	8:1:1	-3.4	-16.3	12.9	CAAAAGUAAUUGUGGAUUUUGU	TTTTTTTTTTTTTTTT	8
hsa-miR-494	131	8:1:1	-0.1	-13.05	12.95	AGGUUGUCCGUGUUGUCUUCUCU	TATATTTTTATTTT	8
hsa-miR-548p	1009	8:1:1	0	-12.96	12.96	UAGCAAAAACUGCAGUUACUUU	TTTTTTTTTAACGCTT	8
hsa-miR-548n	1010	8:1:1	-0.35	-13.32	12.97	CAAAAGUAAUUGUGGAUUUUGU	TTTTTTTTAACGCT	8
hsa-miR-543	533	8:1:1	-2.64	-15.61	12.97	AAACAUUCGCGGUGCACUUCUU	TGAATTTTTTT	8

hsa-miR-429	159	8:1:1	0.1	-12.87	12.97	UAAUACUGUCUGGUAAAACCGU	ATAATATTTG	8
hsa-miR-495	67	8:1:0	-3.6	-16.6	13	GAAGUUGCCCAUGUUAUUUUCG	TTTTTGTTATATTTTA	8
hsa-miR-142-5p	183	8:1:1	-0.6	-13.7	13.1	CAUAAAGUAGAAAGCACUACU	TAATTTGTTTTTT	8
hsa-miR-559	1009	8:1:1	0.2	-12.96	13.16	UAAAGUAAAUAUGCACCAAAA	TTTTTTTTTAACGCT	8
hsa-miR-186	184	8:1:1	-0.4	-13.77	13.37	CAAAGAAUUCUCCUUUUGGGCU	AATTTGTTTTTT	8
hsa-miR-548b-5p	48	8:1:1	-3.5	-16.98	13.48	AAAAGUAAUUGUGGUUUUGGCC	TTTATTTTTTTTTTT	8
hsa-miR-548d-5p	48	8:1:1	-3.5	-16.98	13.48	AAAAGUAAUUGUGGUUUUUGCC	TTTATTTTTTTTT	8
hsa-miR-577	73	8:1:1	-1.9	-15.44	13.54	UAGAUAAAAUAUUGGUACCUG	TTATATTTTA	8
hsa-miR-641	50	8:1:1	-3.9	-17.48	13.58	AAAGACAUAGGAUAGAGUCACCUC	TATTTTTTTTTTTT	8
hsa-miR-548p	1010	8:1:1	0.3	-13.32	13.62	UAGCAAAAACUGCAGUUACUUU	TTTTTTTTAACGCT	8
hsa-miR-513a-3p	533	8:1:0	-1.9	-15.61	13.71	UAAAUUUCACCUUUCUGAGAAGG	TGAATTTTTTTATT	8
hsa-miR-548c-3p	534	8:1:1	-0.4	-14.17	13.77	CAAAAAUCUCAAUUACUUUUGC	GAATTTTTTTA	8
hsa-miR-142-5p	50	8:1:1	-3.7	-17.48	13.78	CAUAAAGUAGAAAGCACUACU	TATTTTTTTTTTTT	8
hsa-miR-577	52	8:1:1	-4	-17.8	13.8	UAGAUAAAAUAUUGGUACCUG	TTTTTTTTTTTTTTTTTT	8
hsa-miR-374b	70	8:1:1	-3.5	-17.36	13.86	AUAUAAUACAACCUGCUAAGUG	TTGTTATATTTTA	8
hsa-miR-548n	73	8:1:1	-1.5	-15.44	13.94	CAAAAGUAAUUGUGGAUUUUGU	TTATATTTTATTT	8
hsa-miR-548n	56	8:1:1	-1.9	-15.86	13.96	CAAAAGUAAUUGUGGAUUUUGU	TTTTTTTTTTTT	8

hsa-miR-548h	48	8:1:1	-3	-16.98	13.98	AAAAGUAAUCGCGGUUUUUGUC	TTTATTTTTTTT	8
hsa-miR-559	52	8:1:1	-3.7	-17.8	14.1	UAAAGUAAAUAUGCACCAAAA	TTTTTTTTTTTTTT	8
hsa-miR-561	522	8:1:1	-0.7	-14.84	14.14	AUCAAGGAUCUUAAACUUUGCC	TATATTTTGTTTG	8
hsa-miR-548i	48	8:1:1	-2.8	-16.98	14.18	AAAAGUAAUUGCGGAUUUUGCC	TTTATTTTTTTTTTTTT	8
hsa-miR-559	1010	8:1:1	0.9	-13.32	14.22	UAAAGUAAAUAUGCACCAAAA	TTTTTTTTAA	8
hsa-miR-548c-5p	48	8:1:1	-2.6	-16.98	14.38	AAAAGUAAUUGCGGUUUUUGCC	TTTATTTTTTTTTTTTTT	8
hsa-miR-548j	48	8:1:1	-2.6	-16.98	14.38	AAAAGUAAUUGCGGUCUUUGGU	TTTATTTTTTTTTTTTTTT	8
hsa-miR-548n	57	8:1:1	-1.3	-15.71	14.41	CAAAAGUAAUUGUGGAUUUUGU	TTTTTTTTTTTTTTTGTTA	8
hsa-miR-186	1136	8:1:0	0.8	-13.64	14.44	CAAAGAAUUCUCCUUUUGGGCU	AATTATTTTGTTACAA	8
hsa-miR-369-3p	69	8:1:1	-2.3	-17.24	14.94	AAUAAUACAUGGUUGAUCUUU	TTTGTTATATTTT	8
hsa-miR-548n	54	8:1:1	-2.1	-17.24	15.14	CAAAAGUAAUUGUGGAUUUUGU	TTTTTTTTTTTT	8
hsa-miR-513a-3p	532	8:1:1	-0.6	-15.81	15.21	UAAAUUUCACCUUUCUGAGAAGG	TTGAATTTTT	8
hsa-miR-361-5p	68	8:1:1	-1.8	-17.05	15.25	UUAUCAGAAUCUCCAGGGGUAC	TTTTGTTATATTTTATT	8
hsa-miR-548n	58	8:1:1	-0.72	-16.18	15.46	CAAAAGUAAUUGUGGAUUUUGU	TTTTTTTTTTTTT	8
hsa-miR-186	509	8:1:1	0.4	-15.26	15.66	CAAAGAAUUCUCCUUUUGGGCU	GATTATTTGTATATAT	8
hsa-miR-548p	52	8:1:1	-1.8	-17.8	16	UAGCAAAAACUGCAGUUACUUU	TTTTTTTTTTT	8
hsa-miR-548n	64	8:1:1	0	-16.08	16.08	CAAAAGUAAUUGUGGAUUUUGU	TTTTTTTTGTTAT	8

hsa-miR-548p	57	8:1:1	0.4	-15.71	16.11	UAGCAAAAACUGCAGUUACUUU	TTTTTTTTTTTTTTTGTTA	8
hsa-miR-548n	53	8:1:1	-1.7	-17.87	16.17	CAAAAGUAAUUGUGGAUUUUGU	TTTTTTTTTTTTTTTTTTT	8
hsa-miR-186	50	8:1:1	-1.3	-17.48	16.18	CAAAGAAUUCUCCUUUUGGGCU	TATTTTTTTTTTTTT	8
hsa-miR-577	57	8:1:1	0.5	-15.71	16.21	UAGAUAAAAUAUUGGUACCUG	TTTTTTTTTTTTTTTGTT	8
hsa-miR-548p	56	8:1:1	0.4	-15.86	16.26	UAGCAAAAACUGCAGUUACUUU	TTTTTTTTTTT	8
hsa-miR-577	58	8:1:1	0.1	-16.18	16.28	UAGAUAAAAUAUUGGUACCUG	TTTTTTTTTTT	8
hsa-miR-548n	59	8:1:1	-0.14	-16.47	16.33	CAAAAGUAAUUGUGGAUUUUGU	TTTTTTTTTTTTTGTT	8
hsa-miR-577	64	8:1:1	0.3	-16.08	16.38	UAGAUAAAAUAUUGGUACCUG	TTTTTTTTGTTA	8
hsa-miR-548p	64	8:1:1	0.4	-16.08	16.48	UAGCAAAAACUGCAGUUACUUU	TTTTTTTTGT	8
hsa-miR-548p	55	8:1:1	0.2	-16.3	16.5	UAGCAAAAACUGCAGUUACUUU	TTTTTTTTTTTT	8
hsa-miR-548p	58	8:1:1	0.4	-16.18	16.58	UAGCAAAAACUGCAGUUACUUU	TTTTTTTTTT	8
hsa-miR-186	51	8:1:1	-0.7	-17.31	16.61	CAAAGAAUUCUCCUUUUGGGCU	ATTTTTTTTTTTTTTTT	8
hsa-miR-548p	63	8:1:1	0.4	-16.25	16.65	UAGCAAAAACUGCAGUUACUUU	TTTTTTTTTG	8
hsa-miR-577	56	8:1:1	0.8	-15.86	16.66	UAGAUAAAAUAUUGGUACCUG	TTTTTTTTTT	8
hsa-miR-656	71	8:1:1	-0.1	-16.78	16.68	AGGUUGCCUGUGAGGUGUUCA	TGTTATATTTTATTTGT	8
hsa-miR-577	55	8:1:1	0.4	-16.3	16.7	UAGAUAAAAUAUUGGUACCUG	TTTTTTTTTTTTTTTTTG	8
hsa-miR-548i	51	8:1:1	-0.6	-17.31	16.71	AAAAGUAAUUGCGGAUUUUGCC	ATTTTTTTTTTTTTTTT	8

hsa-miR-548p	62	8:1:1	0.4	-16.36	16.76	UAGCAAAAACUGCAGUUACUUU	TTTTTTTTTTGTT	8
hsa-miR-548p	53	8:1:1	-1.1	-17.87	16.77	UAGCAAAAACUGCAGUUACUUU	TTTTTTTTTTT	8
hsa-miR-548p	61	8:1:1	0.4	-16.4	16.8	UAGCAAAAACUGCAGUUACUUU	TTTTTTTTTTTG	8
hsa-miR-548p	54	8:1:1	-0.4	-17.24	16.84	UAGCAAAAACUGCAGUUACUUU	TTTTTTTTTT	8
hsa-miR-548n	63	8:1:1	0.6	-16.25	16.85	CAAAAGUAAUUGUGGAUUUUGU	TTTTTTTTTGTT	8
hsa-miR-548p	59	8:1:1	0.4	-16.47	16.87	UAGCAAAAACUGCAGUUACUUU	TTTTTTTTTTT	8
hsa-miR-577	53	8:1:1	-1	-17.87	16.87	UAGAUAAAAUAUUGGUACCUG	TTTTTTTTTT	8
hsa-miR-548c-5p	51	8:1:1	-0.4	-17.31	16.91	AAAAGUAAUUGCGGUUUUUGCC	ATTTTTTTTTTTTTTTT	8
hsa-miR-548j	51	8:1:1	-0.4	-17.31	16.91	AAAAGUAAUUGCGGUCUUUGGU	ATTTTTTTTTT	8
hsa-miR-548p	60	8:1:1	0.4	-16.54	16.94	UAGCAAAAACUGCAGUUACUUU	TTTTTTTTTTTGTTATAT	8
hsa-miR-577	54	8:1:1	-0.3	-17.24	16.94	UAGAUAAAAUAUUGGUACCUG	TTTTTTTTTTTTTTTT	8
hsa-miR-548n	62	8:1:1	0.6	-16.36	16.96	CAAAAGUAAUUGUGGAUUUUGU	TTTTTTTTTTGTTATATTT	8
hsa-miR-559	56	8:1:1	1.1	-15.86	16.96	UAAAGUAAAUAUGCACCAAAA	TTTTTTTTTTTTTTTTG	8
hsa-miR-559	64	8:1:1	0.9	-16.08	16.98	UAAAGUAAAUAUGCACCAAAA	TTTTTTTTGTTATATT	8
hsa-miR-548n	60	8:1:1	0.44	-16.54	16.98	CAAAAGUAAUUGUGGAUUUUGU	TTTTTTTTTTTGTTAT	8
hsa-miR-548n	61	8:1:1	0.6	-16.4	17	CAAAAGUAAUUGUGGAUUUUGU	TTTTTTTTTTTGTT	8
hsa-miR-548n	52	8:1:1	-0.7	-17.8	17.1	CAAAAGUAAUUGUGGAUUUUGU	TTTTTTTTTTTTT	8

hsa-miR-559	54	8:1:1	-0.1	-17.24	17.14	UAAAGUAAAUAUGCACCAAAA	TTTTTTTTTTTTT	8
hsa-miR-577	63	8:1:1	0.9	-16.25	17.15	UAGAUAAAAUAUUGGUACCUG	TTTTTTTTTGTTATATTT	8
hsa-miR-548e	49	8:1:1	-0.2	-17.36	17.16	CAAAAGCAAUCGCGGUUUUUGC	TTATTTTTTTTTTTTT	8
hsa-miR-577	59	8:1:1	0.7	-16.47	17.17	UAGAUAAAAUAUUGGUACCUG	TTTTTTTTTTTTTGT	8
hsa-miR-559	55	8:1:1	0.9	-16.3	17.2	UAAAGUAAAUAUGCACCAAAA	TTTTTTTTTTT	8
hsa-miR-559	57	8:1:1	1.5	-15.71	17.21	UAAAGUAAAUAUGCACCAAAA	TTTTTTTTTTTTTT	8
hsa-miR-577	62	8:1:1	0.9	-16.36	17.26	UAGAUAAAAUAUUGGUACCUG	TTTTTTTTTTGTTATA	8
hsa-miR-577	61	8:1:1	0.9	-16.4	17.3	UAGAUAAAAUAUUGGUACCUG	TTTTTTTTTTTGTTAT	8
hsa-miR-577	60	8:1:1	0.9	-16.54	17.44	UAGAUAAAAUAUUGGUACCUG	TTTTTTTTTTTTGTT	8
hsa-miR-559	53	8:1:1	-0.3	-17.87	17.57	UAAAGUAAAUAUGCACCAAAA	TTTTTTTTTTTTTTTTTTT	8
hsa-miR-559	58	8:1:1	1.5	-16.18	17.68	UAAAGUAAAUAUGCACCAAAA	TTTTTTTTTTTT	8
hsa-miR-559	63	8:1:1	1.5	-16.25	17.75	UAAAGUAAAUAUGCACCAAAA	TTTTTTTTTGTTAT	8
hsa-miR-559	62	8:1:1	1.5	-16.36	17.86	UAAAGUAAAUAUGCACCAAAA	TTTTTTTTTTGTT	8
hsa-miR-559	61	8:1:1	1.5	-16.4	17.9	UAAAGUAAAUAUGCACCAAAA	TTTTTTTTTTTGTTAT	8
hsa-miR-548h	51	8:1:1	0.6	-17.31	17.91	AAAAGUAAUCGCGGUUUUUGUC	ATTTTTTTTT	8
hsa-miR-559	59	8:1:1	1.5	-16.47	17.97	UAAAGUAAAUAUGCACCAAAA	TTTTTTTTTTTTTGT	8
hsa-miR-548b-5p	51	8:1:1	0.7	-17.31	18.01	AAAAGUAAUUGUGGUUUUGGCC	ATTTTTTTTTTT	8

hsa-miR-548d-5p	51	8:1:1	0.7	-17.31	18.01	AAAAGUAAUUGUGGUUUUUGCC	ATTTTTTTTTTTTTTTTTT	8
hsa-miR-559	60	8:1:1	1.5	-16.54	18.04	UAAAGUAAAUAUGCACCAAA	TTTTTTTTTTTT	8
