# Supplementary material for: Red blood cells release microparticles containing human argonaute 2 and miRNAs to target genes of Plasmodium falciparum
Source: Emerg Microbes Infect. 2017 Aug 23;6(8):e75–. doi: 10.1038/emi.2017.63 (PMC5583671; doi:10.1038/emi.2017.63)
Supplement: Supplementary Table S6 [file emi201763x11.pdf]

Supplementary Table S6 Annotations of human miRNAs in high throughput sequencing
Parasites

Seq ID	Length	Read Count	Seq	Category	Annotation
t0000001	22	233247	AAACCGTTACCATTACTGAGTT	miRNA	miR-451
t0000002	21	231838	AAACCGTTACCATTACTGAGT	miRNA	miR-451
t0000003	21	215314	TGGAGAGAAAGGCAGTTCCTG	miRNA	miR-185
t0000004	22	189897	AAAAGCTGGGTTGAGAGGGCGA	miRNA	miR-320a
t0000005	22	181534	TGAGGGGCAGAGAGCGAGACTT	miRNA	miR-423-5p
t0000006	22	178960	TGAGGTAGTAGTTTGTACAGTT	miRNA	let-7g
t0000007	23	172858	TACCACAGGGTAGAACCACGGAC	miRNA	miR-140
t0000008	20	161954	TAGCAGCACATCATGGTTTA	miRNA	miR-15b
t0000009	22	155565	TCCTGTACTGAGCTGCCCCGAG	miRNA	miR-486-5p
t0000010	21	139043	TCCTGTACTGAGCTGCCCCGA	miRNA	miR-486-5p
t0000011	22	133544	AGCAGCATTGTACAGGGCTATG	miRNA	miR-103a
t0000012	20	129187	TGAGGTAGTAGGTTGTATAG	miRNA	let-7
t0000013	22	113850	AGAGGTAGTAGGTTGCATAGTT	miRNA	let-7d
t0000014	23	107039	TGGAGAGAAAGGCAGTTCCTGAA	miRNA	miR-185
t0000015	20	106741	AAACCGTTACCATTACTGAG	miRNA	miR-451
t0000016	23	103910	AGCAGCATTGTACAGGGCTATGA	miRNA	miR-103a
t0000017	23	100163	TACCACAGGGTAGAACCACGGAA	miRNA	miR-140
t0000018	22	96622	CATTGCACTTGTCTCGGTCTGA	miRNA	miR-25
t0000019	21	96107	AGCAGCATTGTACAGGGCTAT	miRNA	miR-103a
t0000020	20	85942	TAGCAGCACATAATGGTTTG	miRNA	miR-15a
t0000021	23	83980	AAACCGTTACCATTACTGAGTTT	miRNA	miR-451
t0000022	24	83607	TACCACAGGGTAGAACCACGGACA	miRNA	miR-140
t0000023	21	82545	TACAGTACTGTGATAACTGAA	miRNA	miR-101
t0000024	19	67329	TGAGGTAGTAGATTGTATA	miRNA	let-7f
t0000025	23	64574	AAAAGCTGGGTTGAGAGGGCGAA	miRNA	miR-320a
t0000027	22	62677	TAGCAGCACATCATGGTTTACA	miRNA	miR-15b
t0000028	20	62631	AGCAGCATTGTACAGGGCTA	miRNA	miR-103a
t0000029	19	61224	TAGCAGCACATCATGGTTT	miRNA	miR-15b
t0000030	20	59735	TAGCAGCACGTAAATATTGG	miRNA	miR-16
t0000031	22	57242	AAAAGCTGGGTTGAGAGGGCGT	miRNA	miR-320d
t0000032	21	56974	TAGCAGCACATAATGGTTTGT	miRNA	miR-15a
t0000033	22	54332	ACCACAGGGTAGAACCACGGAC	miRNA	miR-140
t0000035	20	48633	TGAGGTAGTAGGTTGTGTGG	miRNA	let-7b
t0000036	22	48032	GTACAGTACTGTGATAACTGAA	miRNA	miR-101
t0000037	21	45445	AGAGGTAGTAGGTTGCATAGT	miRNA	let-7d
t0000038	22	45353	ACCACAGGGTAGAACCACGGAA	miRNA	miR-140
t0000039	22	42540	CAACGGAATCCCAAAAGCAGCT	miRNA	miR-191
t0000040	23	40778	AAAAGCTGGGTTGAGAGGGCGTA	miRNA	miR-320d
t0000041	22	40061	TGAGGTAGTAGTTTGTGCTGTT	miRNA	let-7i
t0000042	23	39150	TGAGGTAGTAGATTGTATAGTTA	miRNA	let-7f
t0000043	23	38215	TCCTGTACTGAGCTGCCCCGAGA	miRNA	miR-486-5p
t0000044	21	37910	CATTGCACTTGTCTCGGTCTG	miRNA	miR-25
t0000046	23	36856	TGAGGTAGTAGATTGTATAGTTG	miRNA	let-7f
t0000047	22	36486	TGAGGTAGTAGGTTGTGTGGTA	miRNA	let-7b
t0000048	21	36388	TGAGGTAGTAGTTTGTACAGT	miRNA	let-7g
t0000050	23	31992	TGGAGAGAAAGGCAGTTCCTGAT	miRNA	miR-185
t0000051	23	31745	AAAAGCTGGGTTGAGAGGGCGAT	miRNA	miR-320a
t0000052	23	29970	TGAGGTAGTAGGTTGTGTGGTTA	miRNA	let-7b
t0000053	23	29284	TACCACAGGGTAGAACCACGGAT	miRNA	miR-140
t0000054	20	28620	AGAGGTAGTAGGTTGCATAG	miRNA	let-7d
t0000057	23	28203	TGAGGGGCAGAGAGCGAGACTTA	miRNA	miR-423-5p
t0000058	20	27904	TACAGTACTGTGATAACTGA	miRNA	miR-101
t0000059	22	24615	ACCACAGGGTAGAACCACGGAT	miRNA	miR-140
t0000060	23	22675	AAAAGCTGGGTTGAGAGGGCGTT	miRNA	miR-320d
t0000062	21	22536	GTACAGTACTGTGATAACTGA	miRNA	miR-101

t0000063	22	22438	TGAGGTAGTAGATTGTATAGTA	miRNA	let-7f
t0000064	23	21953	CAACGGAATCCCAAAAGCAGCTG	miRNA	miR-191
t0000065	20	21314	TGGAGAGAAAGGCAGTTCCT	miRNA	miR-185
t0000068	23	19476	ACCACAGGGTAGAACCACGGACA	miRNA	miR-140
t0000069	24	18885	TGAGGGGCAGAGAGCGAGACTTTT	miRNA	miR-423-5p
t0000070	23	17735	TGAGGTAGTAGATTGTATAGTTT	miRNA	let-7f
t0000071	22	17578	TTCAAGTAATCCAGGATAGGCT	miRNA	miR-26a
t0000072	19	17090	TGAGGTAGTAGGTTGTATA	miRNA	let-7
t0000073	23	16862	TGAGGTAGTAGGTTGTGTGGTTT	miRNA	let-7b
t0000074	21	16231	AAAAGCTGGGTTGAGAGGGCG	miRNA	miR-320a
t0000077	23	15561	TAGCAGCACGTAAATATTGGCGA	miRNA	miR-16
t0000078	23	14898	TGGAGAGAAAGGCAGTTCCTGAG	miRNA	miR-185
t0000079	23	14754	TGTAGAGCAGGGAGCAGGAAGCT	miRNA	miR-4732-5p
t0000080	22	13897	TACAGTACTGTGATAACTGAAG	miRNA	miR-101
t0000081	19	13413	AAACCGTTACCATTACTGA	miRNA	miR-451
t0000082	20	13186	TGAGGTAGTAGTTTGTGCTG	miRNA	let-7i
t0000083	22	12124	TGAGGGAGTAGATTGTATAGTT	miRNA	miR-1827
t0000085	20	12041	TAAAGTGCTGACAGTGCAGA	miRNA	miR-106b
t0000086	21	12025	TGAGGTAGTAGTTTGTGCTGT	miRNA	let-7i
t0000087	19	11847	TAGCAGCACGTAAATATTG	miRNA	miR-16
t0000088	20	11709	TCCTGTACTGAGCTGCCCCG	miRNA	miR-486-5p
t0000089	19	11611	TGAGGGGCAGAGAGCGAGA	miRNA	miR-423-5p
t0000090	24	11435	AAACCGTTACCATTACTGAGTTTA	miRNA	miR-451
t0000091	19	11343	TGAGGTAGTAGGTTGTGTG	miRNA	let-7b
t0000092	22	11180	TAGCAGCACATAATGGTTTGTG	miRNA	miR-15a
t0000093	25	11066	AAACCGTTACCATTACTGAGTTTAG	miRNA	miR-451
t0000095	23	10487	TAGCAGCACGTAAATATTGGCGT	miRNA	miR-16
t0000096	20	10466	CCCATAAAGTAGAAAGCACT	miRNA	miR-142
t0000097	22	10086	TATTGCACTTGTCCCGGCCTGT	miRNA	miR-92a
t0000098	24	9701	TGAGGTAGTAGGTTGTGTGGTTAA	miRNA	let-7b
t0000099	21	9389	TTCAAGTAATTCAGGATAGGT	miRNA	miR-26b
t0000100	18	8784	TACAGTATAGATGATGTA	miRNA	miR-144
t0000101	22	8682	TTCAAGTAATTCAGGATAGGTT	miRNA	miR-26b
t0000102	19	8425	ATCACATTGCCAGGGATTT	miRNA	miR-23a
t0000103	22	8231	TCTCACACAGAAATCGCACCCG	miRNA	miR-342-3p
t0000104	21	7940	TAAAGTGCTGACAGTGCAGAT	miRNA	miR-106b
t0000105	20	7897	TGAGGGGCAGAGAGCGAGAC	miRNA	miR-423-5p
t0000106	22	7865	TGAGGTAGTAGATTGTGTAGTT	miRNA	let-7f
t0000107	21	7696	TACCACAGGGTAGAACCACGG	miRNA	miR-140
t0000108	20	7478	ACCACAGGGTAGAACCACGG	miRNA	miR-140
t0000109	23	7074	TCCTGTACTGAGCTGCCCCGAGT	miRNA	miR-486-5p
t0000110	18	6915	TAGCAGCACATCATGGTT	miRNA	miR-15b
t0000113	21	6881	GAGGTAGTAGATTGTATAGTT	miRNA	let-7f
t0000115	28	6816	AACTTTGAAGACTGAAGTGGAGAAGGGT	miRNA	miR-3526
t0000117	23	6770	TCTCACACAGAAATCGCACCCGT	miRNA	miR-342-3p
t0000118	21	6745	TATTGCACTTGTCCCGGCCTG	miRNA	miR-92a
t0000120	23	6658	TGAGATGAAGCACTGTAGCTATC	miRNA	miR-143
t0000121	21	6629	AGGTAGATAGAACAGGTCTTG	miRNA	miR-1839
t0000124	22	6582	AACGGAATCCCAAAAGCAGCTG	miRNA	miR-191
t0000126	22	6506	AGCAGCATTGTACAGGGCTATT	miRNA	miR-107
t0000127	21	6453	CTGACCTATGAATTGACAGCC	miRNA	miR-192
t0000128	19	6388	TAGCAGCACATAATGGTTT	miRNA	miR-15a
t0000129	22	6379	AAGGTAGATAGAACAGGTCTTG	miRNA	miR-1839
t0000131	23	6357	TGAAGACTGAAGTGGAGAAGGGT	miRNA	miR-3526
t0000133	23	6281	ACTGGACTTGGAGTCAGAAGGCA	miRNA	miR-378
t0000134	23	6179	AGCAGCATTGTACAGGGCTATGT	miRNA	miR-103a
t0000135	22	6062	TAGCAGCACGTAAATATTGTCG	miRNA	miR-16
t0000136	19	6005	CTACAGTATAGATGATGTA	miRNA	miR-144

t0000137	22	5670	ATATAATACAACCTGCTAAGTG	miRNA	miR-374
t0000138	21	5651	TAGCAGCACATCATGGTTTAC	miRNA	miR-15b
t0000139	22	5630	TAGCAGCACGTAAATATTGGCA	miRNA	miR-16b
t0000140	24	5620	TACCACAGGGTAGAACCACGGACG	miRNA	miR-140
t0000141	23	5519	TGAGGTAGTAGGTTGTGTGGTAA	miRNA	let-7b
t0000143	21	5417	TGTAGAGCAGGGAGCAGGAAG	miRNA	miR-4732-5p
t0000144	24	5351	CAACGGAATCCCAAAAGCAGCTGA	miRNA	miR-191
t0000145	23	5211	TGAGGTAGTAGGTTGTGTGGTTG	miRNA	let-7b
t0000146	22	5200	CAAAGTGCTGTTCGTGCAGGTA	miRNA	miR-93
t0000148	22	5090	AAACCTTTACCATTACTGAGTT	miRNA	miR-451
t0000150	27	5066	AACTTTGAAGACTGAAGTGGAGAAGGG	miRNA	miR-3526
t0000151	20	5046	TGAGGTAGTAGATTGTAGTT	miRNA	let-7f-5p
t0000153	26	4989	AAACCGTTACCATTACTGAGTTTAGT	miRNA	miR-451
t0000155	20	4876	CAACGGAATCCCAAAAGCAG	miRNA	miR-191
t0000156	19	4732	TGAGGTAGTAGTTTGTGCT	miRNA	let-7i
t0000157	24	4634	TGAGGTAGTAGGTTGTGTGGTTAT	miRNA	let-7b
t0000158	25	4528	TGAGGTAGTAGGTTGTATGGTTATC	miRNA	let-7c
t0000159	22	4203	TGGAGAGAAAGGAAGTTCCTGA	miRNA	miR-185
t0000160	22	4178	TGAGGTAGTAGATTGTATACTT	miRNA	let-7f
t0000161	22	4116	AAAAGCTGGGTTGAGAGGGCAA	miRNA	miR-320b
t0000163	23	4114	CAAAGTGCTGTTCGTGCAGGTAG	miRNA	miR-93
t0000164	21	4050	TAGCTTATCAGACTGATGTTG	miRNA	miR-21
t0000167	22	3980	TGAGGTAGTAGATTGTATAGGT	miRNA	let-7f
t0000168	21	3944	CCCATAAAGTAGAAAGCACTA	miRNA	miR-142
t0000169	21	3932	AAACCTTTACCATTACTGAGT	miRNA	miR-451
t0000170	21	3771	TGAGGTAGTAGGTTGTGTGGA	miRNA	let-7b
t0000171	22	3690	TACAGTACTGTGATAACTGAAA	miRNA	miR-101
t0000172	23	3657	ACCACAGGGTAGAACCACGGACT	miRNA	miR-140
t0000174	22	3647	CATTGCACTTGTCTCGGTCTGT	miRNA	miR-25
t0000177	21	3631	ACCACAGGGTAGAACTACGGA	miRNA	miR-140
t0000180	22	3606	TCCTGTACTGAGCTGCCCCGAA	miRNA	miR-486-5p
t0000182	24	3591	TGAGGGGCAGAGAGCGAGACTTTA	miRNA	miR-423-5p
t0000184	19	3569	AGAGGTAGTAGGTTGCATA	miRNA	let-7d
t0000185	21	3531	TGAGGGAGTAGATTGTATAGT	miRNA	miR-1827
t0000186	22	3514	TACCACAGGGTAGAACTACGGA	miRNA	miR-140
t0000187	23	3491	TACCACAGGGTAGAACCACGGAG	miRNA	miR-140
t0000188	23	3484	TGAGGTAGTAGGTTGTATAGTTA	miRNA	let-7
t0000189	22	3482	TAGCAGCACGTAAATATTGCCG	miRNA	miR-16
t0000191	22	3458	TGAGGTGGTAGATTGTATAGTT	miRNA	let-7f
t0000193	22	3446	TGAGGTAGTAGATTGTATATTT	miRNA	let-7f
t0000194	23	3440	ACCACAGGGTAGAACCACGGACG	miRNA	miR-140
t0000195	20	3436	AAAGCTGGGTTGAGAGGGCG	miRNA	miR-320a
t0000196	22	3373	TGGAGAGAAAGGCAGTTCCTGT	miRNA	miR-185
t0000198	22	3371	TGGAGAGAAAGGCAGTTCCTGC	miRNA	miR-185
t0000199	23	3348	TATTGCACTTGTCCCGGCCTGTA	miRNA	miR-92a
t0000201	20	3343	TGAGGTAGTAGATTGTATTT	miRNA	let-7f-5p
t0000203	22	3279	TCTGGGCACAGGCGGATGGACA	miRNA	miR-5107
t0000204	22	3190	GGATATCATCATATACTGTAAG	miRNA	miR-144*
t0000206	23	3169	AAAAGCTGGGTTGAGAGGGCGAG	miRNA	miR-320a
t0000207	22	3168	TACCACAGGGTAGAACCCCGGA	miRNA	miR-140
t0000208	23	3124	TGAGGTAGTAGTTTGTGCTGTTT	miRNA	let-7i
t0000209	24	3120	TGAAGACTGAAGTGGAGAAGGGTT	miRNA	miR-3526
t0000210	22	3117	ACCACAGGGTAGAACCACGGAG	miRNA	miR-140
t0000211	21	3105	TGACCTATGAATTGACAGCCA	miRNA	miR-192
t0000213	21	3098	AACGGAATCCCAAAAGCAGCT	miRNA	miR-191
t0000214	21	3031	CAACGGAATCCCAAAAGCAGC	miRNA	miR-191
t0000215	20	3015	CCACAGGGTAGAACCACGGA	miRNA	miR-140
t0000216	18	3015	TGAGGTAGTAGATTGTAT	miRNA	let-7f

t0000217	23	2980	TGAGGTAGTAGGTTGTATAGTTG	miRNA	let-7
t0000219	22	2974	AGCAGCATTGTACAGGGCTATA	miRNA	miR-107
t0000220	21	2971	CAAAGTGCTGTTCGTGCAGGT	miRNA	miR-93
t0000221	20	2950	TACAGTATAGATGATGTACT	miRNA	miR-144
t0000222	21	2943	TGAGGTAGTAGATTGTATTGT	miRNA	let-7f
t0000224	21	2920	TCAGTGCACTACAGAACTTTG	miRNA	miR-148a
t0000225	22	2906	TAGCAGCACGTAAATATTGGTG	miRNA	miR-16
t0000226	24	2889	TACCACAGGGTAGAACCACGGAAA	miRNA	miR-140
t0000227	21	2806	AGCAGCACGTAAATATTGGCG	miRNA	miR-16
t0000228	22	2786	CAGTGCAATGTTAAAAGGGCAT	miRNA	miR-130a
t0000230	22	2781	CCTCCCACACCCAAGGCTTGCA	miRNA	miR-532-3p
t0000233	23	2707	GGATATCATCATATACTGTAAGT	miRNA	miR-144*
t0000234	22	2687	TGAGGTAGTAGATTGTATAGTG	miRNA	let-7f
t0000235	22	2569	TAGCAGTACGTAAATATTGGCG	miRNA	miR-16
t0000236	22	2559	TAGCAGCATGTAAATATTGGCG	miRNA	miR-16
t0000237	21	2544	TGAGGTAGTAGGTTGTATGGT	miRNA	let-7c
t0000239	22	2528	TGAGGTAGTAGGTTGTATAGTA	miRNA	let-7-5p
t0000240	25	2526	TGAGATGAAGCACTGTAGCTCTATC	miRNA	miR-143
t0000241	21	2523	TTCAAGTAATCCAGGATAGGC	miRNA	miR-26a
t0000242	23	2513	AACGGAATCCCAAAAGCAGCTGA	miRNA	miR-191
t0000243	23	2513	TGAGGTAGTAGGTTGTATAGTTT	miRNA	let-7
t0000245	19	2488	AGCAGCATTGTACAGGGCT	miRNA	miR-103a
t0000246	19	2484	TAGCAGCACATCATGGTTA	miRNA	miR-15b
t0000247	24	2482	TGAGGTAGTAGGTTGTGTGGTTAG	miRNA	let-7b
t0000249	21	2474	TGAGGTAGTAGATTGTATAGA	miRNA	let-7f
t0000250	23	2454	TGAGGTAGTAGGTTGTGTGGTAT	miRNA	let-7b
t0000253	22	2389	TGAGGTAGTAGGTTGTATGGTT	miRNA	let-7c
t0000254	21	2351	AGGCGGAGACTTGGGCAATTG	miRNA	miR-25*
t0000256	22	2334	TGAGGGAGTAGGTTGTGTGGTT	miRNA	miR-4510
t0000257	22	2314	AAAAGCTGGGTTGAGAGGGCAT	miRNA	miR-320d
t0000258	22	2292	TGAGGAAGTAGATTGTATAGTT	miRNA	let-7f
t0000259	22	2276	TGAGGTAGTAGATTGTACAGTT	miRNA	let-7f
t0000261	23	2238	AATCTGAGAAGGCGCACAAGGTT	miRNA	miR-3200-5p
t0000262	21	2229	TAGCAGCACGTAAATATTGCG	miRNA	miR-16
t0000263	25	2224	ACTGGACTTGGAGTCAGAAGGCATC	miRNA	miR-378
t0000264	23	2189	TGAGGGGCAGAGAGCGAGACTTG	miRNA	miR-423-5p
t0000265	21	2188	ACTGGACTTGGAGTCAGAAGG	miRNA	miR-378
t0000267	21	2181	TAGCAGCACGTAAATATTGGC	miRNA	miR-16
t0000269	20	2151	GAGGTAGTAGATTGTATAGT	miRNA	let-7f
t0000271	22	2116	TACAGTACTGTGATAACTGACT	miRNA	miR-101c
t0000273	21	2115	AAAGCTGGGTTGAGAGGGCGA	miRNA	miR-320a
t0000275	22	2105	TAGCTTATCAGACTGATGTTGA	miRNA	miR-21
t0000277	23	2101	TGGCTCAGTTCAGCAGGAACAGT	miRNA	miR-24
t0000278	22	2092	TGAGGCAGTAGATTGTATAGTT	miRNA	let-7f
t0000279	23	2077	TGAGGTAGTAGTTTGTACAGTTA	miRNA	let-7g
t0000282	20	2055	TGACCTATGAATTGACAGCC	miRNA	miR-192
t0000283	20	2011	TATTGCACTTGTCCCGGCCT	miRNA	miR-92a
t0000284	19	2007	TGGAGAGAAAGGCAGTTGA	miRNA	miR-185
t0000285	22	1982	TGAGGTAGTAGATTGTATTGTT	miRNA	let-7f
t0000286	24	1979	CAACGGAATCCCAAAAGCAGCTGT	miRNA	miR-191
t0000287	25	1924	TGGAATGTAAGGAAGTGTGTGGATC	miRNA	miR-206
t0000288	20	1923	CCGCACTGTGGGTACTTGCT	miRNA	miR-106b*
t0000289	21	1914	AAACCGTTACCATTATTGAGT	miRNA	miR-451
t0000290	21	1907	TGAGGTAGTAGATTGTGTAGT	miRNA	let-7f
t0000291	21	1888	GGATATCATCATATACTGTAA	miRNA	miR-144*
t0000292	22	1881	CGAGGTAGTAGATTGTATAGTT	miRNA	let-7f
t0000293	20	1880	TGAGGGAGTAGATTGTATAG	miRNA	miR-1827
t0000295	20	1874	TGAGGTAGTAGTTTGTACAG	miRNA	let-7g

t0000296	22	1869	TGGGGTAGTAGATTGTATAGTT	miRNA	let-7f
t0000297	21	1841	TTATAAAGCAATGAGACTGAT	miRNA	miR-340-5p
t0000298	23	1836	AAACCTTTACCATTACTGAGTTT	miRNA	miR-451
t0000299	20	1826	TGAGGTAGTAGATTGTGTAG	miRNA	let-7f
t0000300	22	1806	TGAGGTAGTAGATTGCATAGTT	miRNA	let-7f
t0000301	23	1794	TGAGGTAGTAGTTTGTGCTGTTA	miRNA	let-7i
t0000303	20	1789	ATCACATTGCCAGGGATTTC	miRNA	miR-23a
t0000304	22	1786	TAGCAGCACGTAAATATTGGAG	miRNA	miR-16
t0000305	24	1774	TCTCACACAGAAATCGCACCCGTC	miRNA	miR-342-3p
t0000307	22	1767	TGAGGTAGTAGATTGTATAGCT	miRNA	let-7f
t0000308	23	1764	ACCACAGGGTAGAACCACGGAAA	miRNA	miR-140
t0000309	24	1742	TAGCAGCACGTAAATATTGGCGAA	miRNA	miR-16
t0000310	22	1733	TACAGTACTGTGATAACTGAAT	miRNA	miR-101
t0000311	21	1710	TCAGTGCATCACAGAACTTTG	miRNA	miR-148b-3p
t0000313	20	1710	GGATATCATCATATACTGTA	miRNA	miR-144*
t0000314	22	1709	TGAGGTAGTAGGTTGTGTGGAA	miRNA	let-7b
t0000316	22	1692	AACATTCAACGCTGTCGGTGAG	miRNA	miR-181a
t0000317	22	1688	TAAAGTGCTGACAGTGCAGATA	miRNA	miR-106b
t0000319	21	1678	AGCAGCATTGTACAGGGCTAA	miRNA	miR-103a
t0000321	24	1672	AGCAGCATTGTACAGGGCTATGAT	miRNA	miR-103a
t0000322	22	1666	TTAGGTAGTAGATTGTATAGTT	miRNA	let-7f
t0000323	22	1659	TAGCAGCACGTAAATATTGGCT	miRNA	miR-16b
t0000324	24	1645	TTGAAGACTGAAGTGGAGAAGGGT	miRNA	miR-3526
t0000325	22	1645	TTCAAGTAATCCAGGATAGGCA	miRNA	miR-26a
t0000326	23	1644	ATAGCAGCACGTAAATATTGGCG	miRNA	miR-16
t0000327	22	1641	TGAGGTAGCAGATTGTATAGTT	miRNA	let-7f
t0000329	23	1627	TGACCTATGAATTGACAGCCAGA	miRNA	miR-192
t0000330	22	1614	TGAGGTAGGAGATTGTATAGTT	miRNA	let-7e
t0000331	19	1611	TGAGGTAGTAGATTGTAGT	miRNA	let-7f
t0000334	22	1610	AAACCGTTACCATTATTGAGTT	miRNA	miR-451
t0000336	22	1609	TGGAGAGAAAGGCAGTTTCTGA	miRNA	miR-185
t0000337	25	1580	TGAGGTAGTAGGTTGTGTGGTTATC	miRNA	let-7b
t0000338	22	1564	TGAGGTAGTAGTTTGTATAGTT	miRNA	let-7f
t0000339	24	1554	TACCACAGGGTAGAACCACGGACT	miRNA	miR-140
t0000340	29	1503	AACTTTGAAGACTGAAGTGGAGAAGGGT	miRNA	miR-3526
t0000341	24	1501	AAAAGCTGGGTTGAGAGGGCGAAA	miRNA	miR-320a
t0000342	22	1493	TGAGGTAGTGGATTGTATAGTT	miRNA	let-7f-5p
t0000343	22	1490	TGAAGACTGAAGTGGAGAAGGG	miRNA	miR-3526
t0000344	19	1485	TGGAGAGAAAGGCAGTTCC	miRNA	miR-185
t0000346	25	1472	TGAAGACTGAAGTGGAGAAGGGTTT	miRNA	miR-3526
t0000347	18	1470	TGAGGTAGTAGGTTGTGT	miRNA	let-7b
t0000348	24	1445	TGGAGAGAAAGGCAGTTCCTGAAA	miRNA	miR-185
t0000349	18	1438	TGAGGTAGTAGATTGTAG	miRNA	let-7f
t0000350	23	1434	CAAAGTGCTTACAGTGCAGGTAG	miRNA	miR-17
t0000353	26	1433	AACTTTGAAGACTGAAGTGGAGAAGG	miRNA	miR-3526
t0000354	21	1424	TGAGGGGCAGAGAGCGAGACA	miRNA	miR-423-5p
t0000356	22	1422	AGAGGTAGTAGGTTGCATAGTA	miRNA	let-7d
t0000358	22	1421	TGGCTCAGTTCAGCAGGAACAG	miRNA	miR-24
t0000360	21	1415	CAGCAGCAATTCATGTTTTGA	miRNA	miR-424
t0000361	22	1415	TGAGGTAGTAGATTGTATGGTT	miRNA	let-7f
t0000362	21	1384	TGAGGTGGTAGATTGTATAGT	miRNA	let-7f
t0000364	21	1381	AAAAGTGCTTACAGTGCAGGT	miRNA	miR-106
t0000366	21	1375	TGAGGGAGTAGGTTGTGTGGT	miRNA	miR-4510
t0000368	22	1374	CTGACCTATGAATTGACAGCCA	miRNA	miR-192
t0000369	23	1373	TGAGGTAGTAGATTGTATAGTAA	miRNA	let-7f
t0000370	21	1368	TGAGGTAGTAGGTTGTGTGTT	miRNA	let-7b
t0000371	21	1364	AACTTTGAAGACTGAAGTGGA	miRNA	miR-3526
t0000372	18	1353	TAGCAGCACATAATGGTT	miRNA	miR-15a

t0000375	24	1349	TGAGGTAGTAGATTGTATAGTTAA	miRNA	let-7f
t0000376	22	1347	TGAGGTAGTAGGTTGTGTAGTT	miRNA	let-7b
t0000377	24	1331	AAAAGCTGGGTTGAGAGGGCGAAT	miRNA	miR-320a
t0000379	23	1327	TACCACAGGGTAGAACCCCGGAC	miRNA	miR-140
t0000378	21	1327	ACCACAGGGTAGAATCACGGA	miRNA	miR-140
t0000381	24	1317	TGAGGGGCAGAGAGCGAGACTTAA	miRNA	miR-423-5p
t0000382	18	1312	AAACCGTTACCATTACTG	miRNA	miR-451
t0000384	18	1308	TGAGGTAGTAGGTTGTAT	miRNA	let-7
t0000385	22	1307	TCTTGTACTGAGCTGCCCCGAG	miRNA	miR-486-5p
t0000386	23	1305	TGAGGGGCAGAGAGCGAGAATTT	miRNA	miR-423-5p
t0000387	21	1291	TGAGGTAGTAGATTGTATATT	miRNA	let-7f
t0000388	22	1290	TACCACAGGGTAGAACCACGGC	miRNA	miR-140
t0000389	21	1287	TAGCAGCACGTAAATATTGGA	miRNA	miR-16
t0000391	21	1283	AAAAGCTGGGTTGAGAGGGCA	miRNA	miR-320b
t0000392	22	1283	AAAAGTGCTTACAGTGCAGGTA	miRNA	miR-106
t0000394	21	1278	TCTCCCAACCCTTGTACCAGT	miRNA	miR-150
t0000395	22	1276	CAGTGCAATGATGAAAGGGCAT	miRNA	miR-130b
t0000396	20	1273	AAACCTTTACCATTACTGAG	miRNA	miR-451
t0000398	21	1263	AAAGCTGGGTTGAGAGGGCGT	miRNA	miR-320d
t0000399	22	1261	TACGTCATCGTTGTCATCGTCA	miRNA	miR-598
t0000400	21	1243	CAGTGCAATGTTAAAAGGGCA	miRNA	miR-130a
t0000401	26	1236	TAGCTTATCAGACTGATGTTGACATC	miRNA	miR-21
t0000402	24	1234	TGAGGTAGTAGGTTGTATGGTATC	miRNA	let-7-5p
t0000403	23	1209	GTACAGTACTGTGATAACTGAAA	miRNA	miR-101
t0000404	22	1189	TGAGGTAGTAGATTGTTTAGTT	miRNA	let-7f
t0000405	22	1187	TGTAGAGCAGGGAGCAGGAAGC	miRNA	miR-4732-5p
t0000406	22	1178	AGGTAGATAGAACAGGTCTTGT	miRNA	miR-1839
t0000407	23	1177	TACCACAGGGTAGAACCACGGCC	miRNA	miR-140
t0000408	22	1176	TGATGTAGTAGATTGTATAGTT	miRNA	let-7f
t0000409	22	1174	TCTCCCAACCCTTGTACCAGTG	miRNA	miR-150
t0000412	21	1173	ATATAATACAACCTGCTAAGT	miRNA	miR-374
t0000413	22	1170	TACCGCACTGTGGGTACTTGCT	miRNA	miR-106b*
t0000414	22	1168	TGAGGTAGTAGCTTGTATAGTT	miRNA	let-7f
t0000415	18	1166	AGCAGCATTGTACAGGGC	miRNA	miR-103a
t0000416	23	1161	GAAGACTGAAGTGGAGAAGGGTT	miRNA	miR-739
t0000417	22	1155	TCCTGTACTGAGCTGTCCCGAG	miRNA	miR-486-5p
t0000419	22	1140	TGAGGTAGTAGATCGTATAGTT	miRNA	let-7f
t0000420	24	1135	AGCAGCATTGTACAGGGCTATGAA	miRNA	miR-103a
t0000421	20	1122	CTGACCTATGAATTGACAGC	miRNA	miR-192
t0000423	21	1121	CAAAGTGCTTACAGTGCAGGT	miRNA	miR-17
t0000424	21	1120	AAACCGTTACCATTACTTAGT	miRNA	miR-451
t0000426	20	1116	TAGCAGCACGTAAATATTCG	miRNA	miR-16
t0000427	21	1106	TGAGGTAGTAGATTGTACAGT	miRNA	let-7f
t0000428	22	1104	CAAAGTGCTTACAGTGCAGGTA	miRNA	miR-17
t0000429	23	1103	TGAGGGGCAGAGAGCGAGACTAA	miRNA	miR-423-5p
t0000430	20	1099	ATCACATTGCCAGGGATTTA	miRNA	miR-23a
t0000432	23	1080	CATTGCACTTGTCTCGGTCTGAA	miRNA	miR-25
t0000433	22	1076	TGAGGTAGTAGGTTGTGGGGTT	miRNA	let-7b
t0000434	22	1070	TATTGCACTTGTCCCGGCCTGA	miRNA	miR-92b-3p
t0000435	23	1069	AGAGGTAGTAGGTTGCATAGTTA	miRNA	let-7d
t0000436	25	1066	TGGAATGTAAAGAAGTGTGTATATC	miRNA	miR-1
t0000439	19	1064	TGTGAGGTTGGCATTGTTG	miRNA	miR-1294
t0000440	24	1060	TCCTGTACTGAGCTGCCCCGAGAA	miRNA	miR-486-5p
t0000441	22	1059	TGAGGTACTAGATTGTATAGTT	miRNA	let-7f
t0000442	26	1052	TGTAAACATCCTCGACTGGAAGCATC	miRNA	miR-30a-5p
t0000443	21	1052	GTAGTGTTTCCTACTTTATGG	miRNA	miR-142-3p
t0000444	22	1049	TCCTGTACTGAGCTGCCCCGAT	miRNA	miR-486-5p
t0000445	22	1048	TAAGGTAGTAGATTGTATAGTT	miRNA	let-7f

t0000447	23	1039	AGAGGTAGTAGGTTGCATAGTTT	miRNA	let-7d
t0000450	20	1027	AGGGACGGGACGCGGTGCAG	miRNA	miR-92b*
t0000451	21	1025	TAAAGTGCTGACAGTGCAGAA	miRNA	miR-106b
t0000453	22	1015	TGAGGTAGTAGTTTGTACAGTA	miRNA	let-7g
t0000454	22	1002	GTAGTGTTTCCTACTTTATGGA	miRNA	miR-142-3p
t0000455	22	993	TGAGGTAGTAGACTGTATAGTT	miRNA	let-7f
t0000456	21	992	TGGCTCAGTTCAGCAGGAACA	miRNA	miR-24
t0000458	23	989	TGAGGGGCAGAGAGCGAGATTTT	miRNA	miR-423-5p
t0000459	21	985	TTGAGGTAGTAGATTGTATAG	miRNA	let-7f
t0000461	22	984	AAAAGCTGGGTTGAGAGGGCGG	miRNA	miR-320d
t0000462	22	983	TACCACAGGGTAGAACCACGGT	miRNA	miR-140
t0000463	23	975	AAACCGTTACCATTACTGAGTTA	miRNA	miR-451
t0000464	23	974	AAACCGTTACCATTACTGAGTAA	miRNA	miR-451
t0000465	18	973	TGGAGAGAAAGGCAGTTG	miRNA	miR-185
t0000466	23	970	TGACCTATGAATTGACAGCCAGT	miRNA	miR-192
t0000467	21	970	TAGCAGCACATCATGGTTTAA	miRNA	miR-15b
t0000468	21	969	TTATGGTTTGCCTGGGACTGA	miRNA	miR-584
t0000470	23	966	TGTAGAGCAGGGAGCAGGAAGCA	miRNA	miR-4732-5p
t0000473	20	965	TGAGGTAGTAGATTGTATGT	miRNA	let-7f-5p
t0000475	20	964	CATTGCACTTGTCTCGGTCT	miRNA	miR-25
t0000476	22	961	GAAGACTGAAGTGGAGAAGGGT	miRNA	miR-739
t0000478	22	958	AATTGCACGGTATCCATCTGTA	miRNA	miR-363
t0000479	19	958	TACAGTATAGATGATGTAA	miRNA	miR-144
t0000480	22	953	AGGGACGGGACGCGGTGCAGTG	miRNA	miR-92b*
t0000481	23	952	AATGACACGATCACTCCCGTTGA	miRNA	miR-425
t0000482	22	949	TGTAAACATCCTACACTCAGCT	miRNA	miR-30b
t0000483	22	948	TTATAAAGCAATGAGACTGATT	miRNA	miR-340-5p
t0000485	24	947	TGAGGTAGTAGGTTGTGTGGTTGA	miRNA	let-7b
t0000484	18	945	TGAGGGGCAGAGAGCGAG	miRNA	miR-423-5p
t0000486	21	944	AGCTACATCTGGCTACTGGGT	miRNA	miR-222
t0000487	22	939	TGAGGGAGTAGGTTGTATAGTT	miRNA	miR-1827
t0000488	23	937	TGAGGGGCAGATAGCGAGACTTT	miRNA	miR-423-5p
t0000489	25	934	TGGAATGTAAAGAAGTGTGTACATC	miRNA	miR-1
t0000491	22	927	TTTGGCAATGGTAGAACTCACA	miRNA	miR-182
t0000493	24	924	TGTAGAGCAGGGAGCAGGAAGCTG	miRNA	miR-4732-5p
t0000492	22	924	AAACCGTTACCATTACTTAGTT	miRNA	miR-451
t0000494	24	920	CAAAGTGCTTACAGTGCAGGTAGA	miRNA	miR-17
t0000496	22	919	TGGAGAGAAAGGCAGTTACTGA	miRNA	miR-185
t0000498	20	917	AACATTCAACGCTGTCGGTG	miRNA	miR-181a
t0000499	20	917	TAGCAGCACATCATGGGTTA	miRNA	miR-15b
t0000500	24	911	TGTAAACATCCCCGACTGGAAGCT	miRNA	miR-30d
t0000503	22	907	TAGCAGCACGTAAATATTGGCC	miRNA	miR-16b
t0000504	22	904	AAAAGCTGGGTTGAGAGGGCGC	miRNA	miR-320d
t0000505	22	903	TGGAGAGAAAGGCAGTTCCTTA	miRNA	miR-185
t0000506	21	899	CTGACCTATGAATTGACAGCA	miRNA	miR-192
t0000507	22	893	TGCGGTAGTAGATTGTATAGTT	miRNA	let-7f
t0000510	21	893	TGAGGTAGTAGATTGCATAGT	miRNA	let-7f
t0000512	22	886	AAACCGTTACCATTACTCAGTT	miRNA	miR-451
t0000511	25	882	TTGAAGACTGAAGTGGAGAAGGGTT	miRNA	miR-3526
t0000513	22	881	TCCTGTACTGAGCTGCCCCGCG	miRNA	miR-486-5p
t0000514	21	875	TACAGTATAGATGATGTACTA	miRNA	miR-144
t0000515	22	875	TGAGGTAGTAGATTGAATAGTT	miRNA	let-7k
t0000516	21	873	TGAGGTAGTAGGTTGTGGGGT	miRNA	let-7b
t0000517	22	871	TGAGGTAGTAGATTATATAGTT	miRNA	let-7f
t0000519	22	866	TGGAGAGAAAGGCAGTTCTTGA	miRNA	miR-185
t0000520	20	866	ACTCGGCGTGGCGTCGGTCG	miRNA	miR-1307
t0000521	19	861	CATAAAGTAGAAAGCACTA	miRNA	miR-142-5p
t0000524	22	859	TAGCACCATTTGAAATCGGTTA	miRNA	miR-29c

t0000528	21	856	ACCACAGGGTAGAACCACGGC	miRNA	miR-140
t0000529	21	853	TCTTGTACTGAGCTGCCCCGA	miRNA	miR-486-5p
t0000532	23	852	ACCACAGGGTAGAACCACGGATA	miRNA	miR-140
t0000534	23	852	ACCACAGGGTAGAACCACGGAAG	miRNA	miR-140
t0000535	21	852	TGAGGTAGTAGATTGTATAGG	miRNA	let-7f
t0000538	22	848	TGAGGTAGTAGATTGTATAGAT	miRNA	let-7f
t0000537	19	843	ATCACATTGCCAGGGATTA	miRNA	miR-23b
t0000540	22	843	ATCACATTGCCAGGGATTTCCA	miRNA	miR-23a
t0000541	24	843	TACCACAGGGTAGAACCACGGAAT	miRNA	miR-140
t0000544	25	834	TGAGGTAGTAGGTTGTATAGTTATC	miRNA	let-7
t0000543	19	829	TATTGCACTTGTCCCGGCC	miRNA	miR-92a
t0000545	23	829	AAAAGCTGGGTTGAGAGGGCGGA	miRNA	miR-320d
t0000548	22	827	TGAGGTAGAAGATTGTATAGTT	miRNA	let-7f
t0000549	19	824	TAGCAGCACATAATGGTTG	miRNA	miR-15a
t0000550	20	816	TTCAAGTAATCCAGGATAGG	miRNA	miR-26a
t0000552	21	816	TGAGGTAGTAGATTGTATGTT	miRNA	let-7f-5p
t0000553	19	815	TACAGTACTGTGATAACTG	miRNA	miR-101
t0000551	22	811	TGAGGTAGTAGATTCTATAGTT	miRNA	let-7f
t0000554	23	805	AAAAGTGCTTACAGTGCAGGTAG	miRNA	miR-106
t0000557	22	803	TAAAGTGCTTATAGTGCAGGTA	miRNA	miR-20
t0000556	22	801	TAGAAGCACGTAAATATTGGCG	miRNA	miR-16
t0000558	22	799	CTGGACTTGGAGTCAGAAGGCA	miRNA	miR-378
t0000561	22	797	TAGTAGCACGTAAATATTGGCG	miRNA	miR-16
t0000563	25	790	ATGACCTATGATTTGACAGACAATC	miRNA	miR-215
t0000564	22	776	ACTGGACTTGGAGTCAGAAGGC	miRNA	miR-378
t0000567	23	775	TGAGGGGCAGAGAGAGAGACTTT	miRNA	miR-423-5p
t0000568	24	773	TAGCAGCACGTAAATATTGGCGTA	miRNA	miR-16
t0000569	23	771	TGAGGTAGTAGGTTGTGTGGTAG	miRNA	let-7b
t0000570	21	768	CGAGGTAGTAGATTGTATAGT	miRNA	let-7f
t0000571	21	768	TGAGGCAGTAGATTGTATAGT	miRNA	let-7f
t0000573	22	765	TGAGGTAGTAGATTGTATAGTC	miRNA	let-7f
t0000576	22	754	AAAGCTGGGTTGAGAGGGCGAA	miRNA	miR-320a
t0000577	23	750	TGAGGGGCAGAGAGCGAGCCTTT	miRNA	miR-423-5p
t0000578	22	746	TAGCAGCACGTAAATATTTGCG	miRNA	miR-16
t0000579	22	745	TGAGGGGCAGAGAGCGAGACTA	miRNA	miR-423-5p
t0000580	22	739	TAGCAGAACGTAAATATTGGCG	miRNA	miR-16
t0000583	21	733	TGAGGGAGTAGGTTGTATAGT	miRNA	miR-1827
t0000589	21	731	TGAGGTAGTAGGTTGTGTAGT	miRNA	let-7b
t0000590	24	730	AAAAGCTGGGTTGAGAGGGCGATT	miRNA	miR-320a
t0000591	21	729	TGAGGAAGTAGATTGTATAGT	miRNA	let-7f
t0000593	21	723	TGGAGAGAAAGGAAGTTCCTG	miRNA	miR-185
t0000594	20	722	TTATAAAGCAATGAGACTGA	miRNA	miR-340-5p
t0000595	21	721	AAACCGTTACCATTACTCAGT	miRNA	miR-451
t0000597	22	721	TGGAGAGAAAGGCATTTCCTGA	miRNA	miR-185
t0000599	24	717	TGAGGTAGTAGATTGTATAGTTGA	miRNA	let-7f
t0000600	21	708	ACCACAGTGTAGAACCACGGA	miRNA	miR-140
t0000601	22	707	TGAGGTAGTAGGGTGTGTGGTT	miRNA	let-7b
t0000602	23	704	TGAGGGGCAGAGAGCTAGACTTT	miRNA	miR-423-5p
t0000603	22	704	TCTGGGAGGTTGTAGCAGTGGA	miRNA	miR-3192
t0000604	21	702	CAGTGCAATGATGAAAGGGCA	miRNA	miR-130b
t0000605	22	702	TGAGGTAGTAGGTTGTGCGGTT	miRNA	let-7b
t0000606	23	701	GAAAAGCTGGGTTGAGAGGGCGA	miRNA	miR-320a
t0000607	20	700	AAACCGTTACCATTATTGAG	miRNA	miR-451
t0000611	26	699	TGTAAACATCCCCGACTGGAAGCATC	miRNA	miR-30a-5p
t0000612	23	699	TACCACAGGGTAGAACCCCGGAA	miRNA	miR-140
t0000613	21	696	AAACCTTACCATTACTGAGTT	miRNA	miR-451
t0000614	25	695	TGGAATGTAAAGAAGTATGTATATC	miRNA	miR-1a
t0000615	22	690	TGAGGTATTAGATTGTATAGTT	miRNA	let-7f

t0000616	23	685	TGAGGTAGTAGTTTGTACAGTTG	miRNA	let-7g
t0000617	19	682	AACATTCAACGCTGTCGGT	miRNA	miR-181a
t0000618	22	679	AAAGCTGGGTTGAGAGGGCGTA	miRNA	miR-320d
t0000619	22	676	TAGCATCACGTAAATATTGGCG	miRNA	miR-16
t0000620	22	676	TGAGGTAGTAGATTGTAAAGTT	miRNA	let-7f
t0000621	22	676	TGAGGTAGTAGGTTGTGTGTTT	miRNA	let-7b
t0000622	22	675	TTGAGGTAGTAGATTGTATAGT	miRNA	let-7f
t0000623	24	675	TGAGGTAGTAGGTTGTGTGGTATC	miRNA	let-7b
t0000624	19	671	TGAGGTAGTAGATTGTATT	miRNA	let-7f
t0000625	24	670	TACCACAGGGTAGAACCCCGGACA	miRNA	miR-140
t0000626	21	668	ACCACAGGGTAGAACAACGGA	miRNA	miR-140
t0000627	22	665	TCCTGTACTGAGCTGTTCCGAG	miRNA	miR-486-5p
t0000630	22	661	ACCACAGGGTAGAATCACGGAC	miRNA	miR-140
t0000633	22	661	TGAGGTAGTAGATTGTATCGTT	miRNA	let-7f
t0000634	22	660	AAAGCTGGGTTGAGAGGGCGAT	miRNA	miR-320a
t0000635	23	658	AGAGGTAGTAGGTTGCATAGTTG	miRNA	let-7d
t0000637	20	657	TATTGCACTCGTCCCGGCCA	miRNA	miR-92
t0000638	22	654	TTGGGGAAACGGCCGCTGAGTG	miRNA	miR-2110
t0000636	23	652	TATTGCACTTGTCCCGGCCTGTT	miRNA	miR-92a
t0000639	21	652	TGAGGTAGTAGATCGTATAGT	miRNA	let-7f
t0000640	22	649	AAAAGTTGGGTTGAGAGGGCGA	miRNA	miR-320a
t0000644	23	648	TGAGGTAGTAGGTTGTATGGATC	miRNA	let-7b
t0000645	21	647	TGAGGTAGTAGTTTGTATAGT	miRNA	let-7f
t0000646	20	646	TGAGGTAGTAGGTTGTGTGA	miRNA	let-7b
t0000649	24	641	TCCTGTACTGAGCTGCCCCGAGAT	miRNA	miR-486-5p
t0000652	21	641	TGAGGGGCAGAGAGAGAGACT	miRNA	miR-423-5p
t0000650	21	640	TACAGTACTGTGATAACTGAT	miRNA	miR-101c
t0000654	21	631	TCCTGTACTGAGCTGCTCCGA	miRNA	miR-486-5p
t0000656	22	630	TACCACAGGGTAGAATCACGGA	miRNA	miR-140
t0000657	23	629	TGAGGTAGTAGTTTGTGCTGTTG	miRNA	let-7i
t0000659	25	629	TGAGGTAGTAGGTTGTGTGGTTAGA	miRNA	let-7b
t0000661	23	626	TGAGGGGCAGACAGCGAGACTTT	miRNA	miR-423-5p
t0000660	22	625	AAACCCTTACCATTACTGAGTT	miRNA	miR-451
t0000664	19	622	CATTGCACTTGTCTCGGTC	miRNA	miR-25
t0000665	20	621	TAGCAGCATATCATGGTTTA	miRNA	miR-15b
t0000666	23	618	TACAGTACTGTGATAACTGAAGA	miRNA	miR-101
t0000668	22	617	TGAGGTAATAGATTGTATAGTT	miRNA	let-7f
t0000671	19	616	TCCTGTACTGAGCTGCCCC	miRNA	miR-486-5p
t0000672	21	614	AGGGGCAGAGAGCGAGACTTT	miRNA	miR-423-5p
t0000673	21	614	TAGCAGCACATCATGGTTACA	miRNA	miR-15b
t0000677	22	614	TGAGTTAGTAGTTTGTACAGTT	miRNA	let-7g
t0000678	23	613	AGGCGGAGACTTGGGCAATTGCT	miRNA	miR-25*
t0000681	22	611	TAGCAGCACGCAAATATTGGCG	miRNA	miR-16
t0000680	22	611	TGAGGTAGTATATTGTATAGTT	miRNA	let-7f
t0000682	18	608	TGGAGAGAAAGGCAGTTC	miRNA	miR-185
t0000684	22	607	ACCACAGGGTAGAATTACGGAC	miRNA	miR-140
t0000689	22	604	TGAGATAGTAGATTGTATAGTT	miRNA	let-7f
t0000691	22	603	ACTGCAGTGAAGGCACTTGTAG	miRNA	miR-17*
t0000692	21	601	GAAGACTGAAGTGGAGAAGGG	miRNA	miR-739
t0000694	22	601	TACCACAGGGTAGAACAACGGA	miRNA	miR-140
t0000695	23	599	CAAAGTGCTCATAGTGCAGGTAG	miRNA	miR-20b
t0000698	22	599	TGAGGTAGTAGATTGTGTGGTT	miRNA	let-7b
t0000699	20	598	TAGCAGCACATAATGGGTTG	miRNA	miR-15a
t0000696	21	597	TCCTGTACTGAGCTGCCTCGA	miRNA	miR-486-5p
t0000701	22	592	AATGACACGATCACTCCCGTTG	miRNA	miR-425
t0000703	21	589	TCCTGTACTGAGCTGCCCCGC	miRNA	miR-486-5p
t0000702	22	588	TGAGGAAGTAGGTTGTGTGGTT	miRNA	let-7b
t0000704	21	587	TATTGCACTCGTCCCGGCCTA	miRNA	miR-92

t0000705	24	585	TGGAGAGAAAGGCAGTTCCTGAGA	miRNA	miR-185
t0000706	19	581	CAACGGAATCCCAAAAGCA	miRNA	miR-191
t0000707	21	581	TGGGGTAGTAGATTGTATAGT	miRNA	let-7f
t0000708	22	580	TGAGGTAGTAGTTTGTGTGGTT	miRNA	let-7b
t0000709	23	579	CTGAGGTAGTAGATTGTATAGTT	miRNA	let-7f
t0000711	23	573	TACCACAGGGTAGAACTACGGAA	miRNA	miR-140
t0000710	24	570	AAAAGCTGGGTTGAGAGGGCGATA	miRNA	miR-320a
t0000712	21	568	TGAGGGGCAGATAGCGAGACT	miRNA	miR-423-5p
t0000713	20	568	ACCACTGACCGTTGACTGTA	miRNA	miR-181a-2*
t0000716	22	568	TAGCAGCACGTAAAAATTGGCG	miRNA	miR-16
t0000718	22	567	TGAGGTGGTAGGTTGTGTGGTT	miRNA	let-7b
t0000720	20	565	TATTGCACTCGTCCCGGCCT	miRNA	miR-92
t0000721	23	562	AAAAGCTGGGTTGAGAGGGCGTG	miRNA	miR-320d
t0000723	21	562	ACCACAGGGTAGAACCACGGT	miRNA	miR-140
t0000727	21	562	TTAGGTAGTAGATTGTATAGT	miRNA	let-7f
t0000726	25	561	TCTCACACAGAAATCGCACCCGTCT	miRNA	miR-342-3p
t0000728	24	560	TACCACAGGGTAGAACCACGGATA	miRNA	miR-140
t0000729	25	558	TGGAATGTAAAGAAGTATGTACATC	miRNA	miR-1a
t0000731	22	558	TGAGGTAGTAGGTTCTGTGGTT	miRNA	let-7b
t0000732	21	556	TAGCAGCACGTAAATATTGGT	miRNA	miR-16
t0000733	24	556	TAGCAGCACATAATGGTTTGTGGA	miRNA	miR-15a
t0000735	19	555	TTGTACTGAGCTGCCCCGA	miRNA	miR-486-5p
t0000736	22	554	TAGCAGCACGTAAATATTGACG	miRNA	miR-16
t0000738	20	553	AATTGCACGGTATCCATCTG	miRNA	miR-363
t0000739	22	553	CGGATGAGCAAAGAAAGTGGTT	miRNA	miR-1255b
t0000742	21	553	TGAGGTAGGAGATTGTATAGT	miRNA	let-7e
t0000740	21	550	AGGGACGGGACGCGGTGCAGT	miRNA	miR-92b*
t0000741	23	550	AAAAGCTGGGTTGAGAGGGCGCA	miRNA	miR-320d
t0000743	21	546	ACCGCACTGTGGGTACTTGCT	miRNA	miR-106b*
t0000744	21	545	ACCACAGGGTAGAATTACGGA	miRNA	miR-140
t0000745	23	544	AAGGTAGATAGAACAGGTCTTGA	miRNA	miR-1839
t0000746	25	544	TAGCAGCACGTAAATATTGGCGAAA	miRNA	miR-16
t0000749	20	543	GTGAGGACTCGGGAGGTGGA	miRNA	miR-1224
t0000748	22	542	TGAGTTAGTAGATTGTATAGTT	miRNA	let-7f
t0000750	22	541	TCCTGTACTGAGCTGCCCTGAG	miRNA	miR-486-5p
t0000751	19	540	TGAGGTAGATTGTATAGTT	miRNA	miR-1961
t0000752	22	540	TGGAGGGAAAGGCAGTTCCTGA	miRNA	miR-185
t0000753	22	540	TACAGTATAGATGATGTACTAT	miRNA	miR-144
t0000754	22	537	TGGAGAGAAAGGCAGTTCCTGG	miRNA	miR-185
t0000756	22	534	GTACAGTACTGTGATAACTGAT	miRNA	miR-101c
t0000755	21	534	TGAGGTAGTGGATTGTATAGT	miRNA	let-7f-5p
t0000761	21	532	TGAGGGGCAGAGAGCGAGATT	miRNA	miR-423-5p
t0000762	22	531	AGGTAGATAGAACAGGTCTTGA	miRNA	miR-1839
t0000763	21	529	CCACAGGGTAGAACCACGGAA	miRNA	miR-140
t0000764	21	528	TGAGGTAGTAGATTGTATGGT	miRNA	let-7f
t0000766	22	527	TGAGGTAGTAGATTGGATAGTT	miRNA	let-7f
t0000767	22	525	TGGAGAGAAAGGCAGTTCTCGA	miRNA	miR-185
t0000768	20	524	CAGCAGCAATTCATGTTTTG	miRNA	miR-424
t0000769	21	524	TGAGGTATTAGATTGTATAGT	miRNA	let-7f
t0000770	22	523	TGAGGGGCAGAGAGCGAGATTT	miRNA	miR-423-5p
t0000771	21	521	TGAGGTAGCAGATTGTATAGT	miRNA	let-7f
t0000773	21	520	TGAAGACTGAAGTGGAGAAGG	miRNA	miR-3526
t0000776	22	520	TGGAGAGAAAGGCAGTTCATGA	miRNA	miR-185
t0000775	21	516	ACTACAGGGTAGAACCACGGA	miRNA	miR-140
t0000778	22	512	TGCGGGGCTAGGGCTAACAGCA	miRNA	miR-744
t0000779	22	510	TGGAGAGAAAGGTAGTTCCTGA	miRNA	miR-185
t0000782	19	508	CAGTACTGTGATAACTGAA	miRNA	miR-101c
t0000783	22	507	TGAGGTAGTAGTTTGTGCTGTA	miRNA	let-7i

t0000785	20	503	AAGCTGCCAGTTGAAGAACT	miRNA	miR-22-3p
t0000788	23	502	TGAGGGGCAGAGAGTGAGACTTT	miRNA	miR-423-5p
t0000790	22	499	TTCTGTACTGAGCTGCCCCGAG	miRNA	miR-486-5p
t0000791	18	499	ATCACATTGCCAGGGATT	miRNA	miR-23a
t0000792	21	499	ACCACAGGGGAGAACCACGGA	miRNA	miR-140
t0000793	21	498	TGAGGTAGTAGATTGTTTAGT	miRNA	let-7f
t0000794	22	498	TGAGCTAGTAGATTGTATAGTT	miRNA	let-7f
t0000795	22	495	TCCTGTACTGAGCTGCTCCGAG	miRNA	miR-486-5p
t0000796	21	495	TGAGGGGCAGAGAGCGAGCCT	miRNA	miR-423-5p
t0000800	22	495	TGAGGTCGTAGATTGTATAGTT	miRNA	let-7f
t0000801	20	494	AGGCGGAGACTTGGGCAATT	miRNA	miR-25*
t0000802	21	494	CTCGGCGTGGCGTCGGTCGTG	miRNA	miR-1307
t0000803	22	493	TGAGGTAGTAGATTTTATAGTT	miRNA	let-7f
t0000806	22	489	ACCACAGGGTAGAACTACGGAA	miRNA	miR-140
t0000807	21	489	AGGTAGTAGATTGTATAGTTG	miRNA	let-7f
t0000809	22	488	TGAGGGAGTAGTTTGTACAGTT	miRNA	let-7g
t0000810	22	488	TGAGGTAGTAGGTTGTGTGGTG	miRNA	let-7b
t0000811	21	486	TGGAGAGAAAGGCATTTCCTG	miRNA	miR-185
t0000812	22	486	TACCACAGTGTAGAACCACGGA	miRNA	miR-140
t0000814	21	485	ATCACATTGCCAGGGATTTCA	miRNA	miR-23a
t0000818	21	485	TAGGTAGTAGATTGTATAGTT	miRNA	let-7f
t0000819	21	482	TGAGGTAGTAGCTTGTATAGT	miRNA	let-7f
t0000817	22	481	TACAGTATAGATGATGTACTAA	miRNA	miR-144
t0000820	21	480	CCACAGGGTAGAACCACGGAC	miRNA	miR-140
t0000821	22	479	TCGAATCCGAGTCACGGCACCA	miRNA	miR-4454
t0000822	24	477	TAGCTTATCAGACTGATGTTGATC	miRNA	miR-21
t0000824	22	476	CATTGCATTTGTCTCGGTCTGA	miRNA	miR-25
t0000825	23	475	ATGAGGTAGTAGATTGTATAGTT	miRNA	let-7f
t0000826	19	474	CCCATAAAGTAGAAAGCAC	miRNA	miR-142
t0000827	22	472	TGGAGAGAAAGGGAGTTCCTGA	miRNA	miR-185
t0000832	21	469	TAGCACCATTTGAAATCGGTT	miRNA	miR-29c
t0000831	19	468	ACTGCAGTGAAGGCACTTG	miRNA	miR-17*
t0000834	22	467	TACCACATGGTAGAACCACGGA	miRNA	miR-140
t0000835	23	465	AACGGAATCCCAAAAGCAGCTGT	miRNA	miR-191
t0000836	23	465	ACTGCAGTGAAGGCACTTGTAGA	miRNA	miR-17*
t0000837	21	464	TGAGGGGCAGAGAGTGAGACT	miRNA	miR-423-5p
t0000838	21	464	ATTGCACTTGTCTCGGTCTGA	miRNA	miR-25
t0000839	20	464	TGAGGTAGTAGATTGTATAA	miRNA	let-7f
t0000840	22	464	TGAGGTAGTAGGGTGTATAGTT	miRNA	let-7
t0000841	24	463	TGGCTCAGTTCAGCAGGAACAGTT	miRNA	miR-24
t0000842	22	461	TGGAGATAAAGGCAGTTCCTGA	miRNA	miR-185
t0000844	21	460	CATAAAGTAGAAAGCACTACT	miRNA	miR-142-5p
t0000845	21	457	TCCTGTACTGAGCTGCCTTGA	miRNA	miR-486-5p
t0000849	22	457	TGAGGTAGTCGATTGTATAGTT	miRNA	let-7f
t0000850	21	454	TGGAGAGAAAGGCAGTTCTTG	miRNA	miR-185
t0000851	22	452	TCCTGTACTGAGCTGCCTCGAG	miRNA	miR-486-5p
t0000852	23	451	TGAGGTAGTAGTTTGTACAGTTT	miRNA	let-7g
t0000853	19	450	TGGGTTTACGTTGGGAGAA	miRNA	miR-629
t0000857	18	450	CCCATAAAGTAGAAAGCA	miRNA	miR-142
t0000856	22	449	TAGCAGCACGTAAATATTAGCG	miRNA	miR-16
t0000854	23	445	ACTGGACTTGGAGTCAGAAGGCT	miRNA	miR-378
t0000855	24	443	TGAGGGGCAGAGAGCGAGACTTGA	miRNA	miR-423-5p
t0000861	24	443	TGGACGGAGAACTGATAAGGGATC	miRNA	miR-184
t0000862	19	441	TAGCACGTAAATATTGGCG	miRNA	miR-16c
t0000863	21	440	TCCTGTACTGAGTTGCCCCGA	miRNA	miR-486-5p
t0000866	21	440	TACAGTATAGATGATGTACTT	miRNA	miR-144
t0000867	22	439	TGAGGTAGTAGGTTGTTTGGTT	miRNA	let-7b
t0000868	20	436	TGAAGACTGAAGTGGAGAAG	miRNA	miR-3526

t0000869	18	435	CATTGCACTTGTCTCGGT	miRNA	miR-25
t0000871	22	435	CCGCACTGTGGGTACTTGCTGA	miRNA	miR-106b*
t0000874	21	434	TAAAGTGCTTATAGTGCAGGT	miRNA	miR-20
t0000873	21	433	TGAGGGGCAGAGAGCTAGACT	miRNA	miR-423-5p
t0000876	20	430	AGCAGCATTGTACAGGGCTT	miRNA	miR-107b
t0000882	22	429	CAACGGAATCCCAAAAGCAGCA	miRNA	miR-191
t0000886	19	429	TAGCAGCACATTATGGTTT	miRNA	miR-15b
t0000887	23	426	TGAGTGGCAGAGAGCGAGACTTT	miRNA	miR-423-5p
t0000889	20	424	TAGCAGCACATAATGGTTGT	miRNA	miR-15a
t0000890	21	424	TGAGGTAGTAGGTTGTTTGGT	miRNA	let-7b
t0000892	25	421	TGGACGGAGAACTGATAAGGGCATC	miRNA	miR-184-3p
t0000893	20	421	TGAGGTAGTAGGTTGTGTGT	miRNA	let-7b
t0000896	24	420	TGACCTATGAATTGACAGCCAGTA	miRNA	miR-192
t0000897	22	420	TGACCTATGAATTGACAGCCAG	miRNA	miR-192
t0000898	21	419	TGAGGTACTAGATTGTATAGT	miRNA	let-7f
t0000900	21	418	TTCTGTACTGAGCTGCCCCGA	miRNA	miR-486-5p
t0000901	22	418	TAGCACCATCTGAAATCGGTTA	miRNA	miR-29c
t0000904	19	417	AATTGCACGGTATCCATCT	miRNA	miR-363
t0000905	21	417	TGTAAACATCCTACACTCTCA	miRNA	miR-30c
t0000906	22	417	CTGAGGTAGTAGATTGTATAGT	miRNA	let-7f
t0000908	23	416	TGAGGGGCAGAGGGCGAGACTTT	miRNA	miR-423-5p
t0000912	22	416	TACCATAGGGTAGAACCACGGA	miRNA	miR-140
t0000910	22	415	TACCACAGGGTAGAAACACGGA	miRNA	miR-140
t0000917	23	413	TACCATAGGGTAGAACCACGGAC	miRNA	miR-140
t0000914	22	413	TGAGTTAGTAGGTTGTGTGGTT	miRNA	let-7b
t0000918	22	411	CTGGACTTGGAGTCAGAAGGCT	miRNA	miR-378
t0000919	24	411	TAAAGTGCTTATAGTGCAGGTAGA	miRNA	miR-20
t0000920	24	411	TGGAGTGTGACAATGGTGTTTATC	miRNA	miR-122
t0000923	22	411	TGAGGTAGTAGATTGTCTAGTT	miRNA	let-7f
t0000924	22	411	TGAGGCAGTAGGTTGTGTGGTT	miRNA	let-7b
t0000928	22	408	TGAAGTAGTAGATTGTATAGTT	miRNA	let-7f
t0000929	21	407	TGAGGGGCAGAGAGCGAGAAT	miRNA	miR-423-5p
t0000927	22	407	TAGCAGCACTTAAATATTGGCG	miRNA	miR-16
t0000930	20	407	AGCAGCATTGTACAGGGTTA	miRNA	miR-103a
t0000932	23	406	TGAGGTGCAGAGAGCGAGACTTT	miRNA	miR-423-5p
t0000934	22	405	CTAGACTGAAGCTCCTTGAGGA	miRNA	miR-151-3p
t0000938	23	403	TCTTGTACTGAGCTGCCCCGAGA	miRNA	miR-486-5p
t0000940	20	403	TGTAGAGCAGGGAGCAGGAA	miRNA	miR-4732-5p
t0000947	20	403	TTTGGCAATGGTAGAACTCA	miRNA	miR-182
t0000944	22	402	ACTGGGAAGAGGAGCTGAGGGA	miRNA	miR-4646-5p
t0000948	22	402	GAAAAGCTGGGTTGAGAGGGCG	miRNA	miR-320a
t0000943	22	401	TAGCAGCACATAAATATTGGCG	miRNA	miR-16
t0000946	22	400	TGGGGAGAAAGGCAGTTCCTGA	miRNA	miR-185
t0000950	22	400	TAGCAGCACGTAAATATTCGCG	miRNA	miR-16
t0000951	23	399	TAAAGTGCTTATAGTGCAGGTAG	miRNA	miR-20
t0000953	22	399	TGAGGTAGTTGATTGTATAGTT	miRNA	let-7f
t0000952	22	399	TGAGGTAGTAGGTTGTGTGGGT	miRNA	let-7b
t0000954	21	398	TGGAGAGAAAGGCAGTTCCTA	miRNA	miR-185
t0000957	22	398	TATCAGCACGTAAATATTGGCG	miRNA	miR-16
t0000964	21	397	TCCTGTACTGAGCTGCCCTGA	miRNA	miR-486-5p
t0000960	26	397	TGGAGTGTGACAATGGTGTTTGTATC	miRNA	miR-122
t0000961	22	396	TATTGCACTCGTCCCGGCCTAA	miRNA	miR-92
t0000967	20	396	AAGGTAGATAGAACAGGTCT	miRNA	miR-1839
t0000965	23	396	ACCACAGGGTAGAACCACGGAAT	miRNA	miR-140
t0000968	26	395	TCCTGTACTGAGCTGCCCCGAGAAGA	miRNA	miR-486-5p
t0000969	22	394	TACCACAGGGGAGAACCACGGA	miRNA	miR-140
t0000970	26	392	TCCTGTACTGAGCTGCCCCGAGGTCC	miRNA	miR-486-5p
t0000973	24	392	TGAGGGGCAGAGAGCGAGACTTAT	miRNA	miR-423-5p

t0000976	20	392	ATCACATTGCCAGGGATTTT	miRNA	miR-23a
t0000975	23	391	ACTGGACTTGGAGTCAGAAGGCG	miRNA	miR-378
t0000977	23	390	ACTGGCTAGGGAAAATGATTGGA	miRNA	miR-664*
t0000978	21	390	ACCATAGGGTAGAACCACGGA	miRNA	miR-140
t0000979	22	390	TGATGTAGTAGGTTGTGTGGTT	miRNA	let-7b
t0000980	21	386	TGAGGTAGTAGATTGAATAGT	miRNA	let-7k
t0000982	22	385	ACTGGACTTGGAGTCAGAAGGA	miRNA	miR-378c
t0000981	20	385	TTCAAGTAATTCAGGATAGG	miRNA	miR-26b
t0000983	21	384	AAACCGTTACCATTACTGAGA	miRNA	miR-451
t0000985	23	384	TGTAACAGCAACTCCATGTGGAA	miRNA	miR-194
t0000987	24	382	TGAGGGGCAGAGAGCGAGACTTAG	miRNA	miR-423-5p
t0000991	22	381	GTAGAGGAGATGGCGCAGGGGA	miRNA	miR-877
t0000989	25	381	TCTCACACAGAAATCGCACCCGTCA	miRNA	miR-342-3p
t0000990	22	381	AGCTACATTGTCTGCTGGGTTT	miRNA	miR-221
t0000993	21	381	TGAGGTAGTAGGGTGTGTGGT	miRNA	let-7b
t0000994	22	380	CACCCGGCTGTGTGCACATGTG	miRNA	miR-941
t0000996	21	380	GGAGAGAAAGGCAGTTCCTGA	miRNA	miR-185
t0000995	21	380	TGAGGTAGTAGATTGTATCGT	miRNA	let-7f
t0000999	19	380	TGAGGTAGTAGGTTGTGGT	miRNA	let-7b
t0001003	22	379	TGGAGAGAAAGGCAGTTTTTGA	miRNA	miR-185
t0001001	20	378	TGAGGTGGTAGATTGTATAG	miRNA	let-7f
t0001004	22	377	CCACAGGGTAGAACCACGGACA	miRNA	miR-140
t0001006	22	376	TGGAGAGAAAGGCAGTTCCCGA	miRNA	miR-185
t0001008	24	375	GAAGACTGAAGTGGAGAAGGGTTT	miRNA	miR-739
t0001012	22	374	TGGAGAGAAAGGCAGTCCCTGA	miRNA	miR-185
t0001011	24	373	TGTAAACATCCTACACTCTCAGCT	miRNA	miR-30c
t0001013	21	373	ATCACAGGGTAGAACCACGGA	miRNA	miR-140
t0001010	22	372	TGTCAGTTTGTCAAATACCCCA	miRNA	miR-223
t0001017	24	372	ACCACAGGGTAGAACCACGGAAGA	miRNA	miR-140
t0001016	23	371	TGAGGGGAAGAGAGCGAGACTTT	miRNA	miR-423-5p
t0001014	21	371	TGAGGATGGATAGCAAGGAAG	miRNA	miR-3605-5p
t0001015	22	371	AAAAGCTGGGTTGAGAGGGAGA	miRNA	miR-320d
t0001019	25	371	TACCCTGTAGATCCGAATTTGTATC	miRNA	miR-10a
t0001020	26	369	TCTTTGGTTATCTAGCTGTATGAATC	miRNA	miR-9a
t0001021	28	369	TCCTGTACTGAGCTGCCCCGAGACGGCA	miRNA	miR-486-5p
t0001023	22	369	TGAGGTTGTAGATTGTATAGTT	miRNA	let-7f
t0001024	24	368	AAAAGCTGGGTTGAGAGGGCGAAG	miRNA	miR-320a
t0001025	21	367	AAGACTGAAGTGGAGAAGGGT	miRNA	miR-739
t0001028	21	366	TCAAGTAATCCAGGATAGGCT	miRNA	miR-26a
t0001029	26	366	TGAGGTAGTAGGTTGTATGGTTTATC	miRNA	let-7b
t0001032	22	365	AAAGCTGGGTTGAGAGGGCGTT	miRNA	miR-320d
t0001031	21	365	TGAGGTACTAGGTTGTGTGGT	miRNA	let-7b
t0001037	21	365	TGAGGTAGTAGGGTGTATAGT	miRNA	let-7
t0001036	26	364	AGGGCTGGGTCGGTCGGGCTGGGGCG	miRNA	miR-4651
t0001035	21	364	TGAGGTAGTAGACTGTATAGT	miRNA	let-7f
t0001034	22	363	AAACTGTTACCATTACTGAGTT	miRNA	miR-451
t0001040	21	362	TCCTGTACTGAGCTGTCCCGA	miRNA	miR-486-5p
t0001041	22	362	CGGAGAGAAAGGCAGTTCCTGA	miRNA	miR-185
t0001039	20	362	CTAGACTGAAGCTCCTTGAG	miRNA	miR-151-3p
t0001042	21	362	TGAGGTAGTAGGTTGTGCGGT	miRNA	let-7b
t0001043	21	361	TGAGGGGCAGAGAGCGGGACT	miRNA	miR-423-5p
t0001045	21	361	TGAGAGAAAGGCAGTTCCTGA	miRNA	miR-185
t0001047	22	361	TGAGGTAGTAAATTGTATAGTT	miRNA	let-7f
t0001051	22	360	AAAAGCTGGGTTGAGATGGCGA	miRNA	miR-320a
t0001049	22	360	TGGGGTAGTAGGTTGTGTGGTT	miRNA	let-7b
t0001048	24	359	TAGCAGCACGTAAATATTGGCGTT	miRNA	miR-16
t0001053	22	359	TGAGGTAGTAGGTTGTACAGTT	miRNA	let-7
t0001054	24	358	GGATATCATCATATACTGTAAGTT	miRNA	miR-144*

t0001055	24	358	TACCACAGGGTAGAACCACGGCCA	miRNA	miR-140
t0001060	22	355	AACTTTGAAGACTGAAGTGGAG	miRNA	miR-3526
t0001057	22	355	TACCACAGGGTAGAACCAAGGA	miRNA	miR-140
t0001059	25	354	TCGTACCGTGAGTAATAATGCGATC	miRNA	miR-126
t0001058	22	354	TACAGTACTGTGATAACTGATT	miRNA	miR-101c
t0001062	18	352	TCCTGTACTGAGCTGCCC	miRNA	miR-486-5p
t0001063	21	352	TGAGGTAGTAGTTTGTGTGTT	miRNA	let-7b
t0001064	24	351	TGAGGTAGTAGGTTGTGTGGTTTT	miRNA	let-7b
t0001066	24	350	TGGAGAGAAAGGCAGTTCCTGAAG	miRNA	miR-185
t0001067	19	349	CTCGGCGTGGCGTCGGTCG	miRNA	miR-1307
t0001069	20	348	CTGTACTGAGCTGCCCCGAG	miRNA	miR-486-5p
t0001068	22	347	AATCTGAGAAGGCGCACAAGGT	miRNA	miR-3200-5p
t0001072	22	347	TGGAGAAAAAGGCAGTTCCTGA	miRNA	miR-185
t0001070	25	347	TCAGTGCACTACAGAACTTTGTATC	miRNA	miR-148a
t0001074	21	347	TGAGGAAGTAGGTTGTGTGGT	miRNA	let-7b
t0001073	20	345	TAGCAGCACATTATGGTTTA	miRNA	miR-15b
t0001076	19	343	GAGGTAGTAGATTGTATAG	miRNA	let-7f
t0001077	21	342	TGAGGTAGTAGGTTGTGTTGT	miRNA	let-7b
t0001079	19	341	TGTAGAGCAGGGAGCAGGA	miRNA	miR-4732-5p
t0001080	22	341	TGGAGAGAAGGGCAGTTCCTGA	miRNA	miR-185
t0001083	21	341	TGAGGTGGTAGGTTGTATAGT	miRNA	let-7
t0001084	23	340	CAAAGTGCTGTTCGTGCAGGTAT	miRNA	miR-93
t0001085	19	340	TGAGGTAGTAGTTTGTACA	miRNA	let-7g
t0001087	25	340	TGAGGTAGTAGATTGTATAGTTATC	miRNA	let-7f
t0001093	21	340	TGAGGCAGTAGGTTGTGTGGT	miRNA	let-7b
t0001096	19	338	CTGTACTGAGCTGCCCCGA	miRNA	miR-486-5p
t0001092	23	338	AGCAGCATTGTACAGGGCTATAA	miRNA	miR-107
t0001095	21	337	GTGAGGACTCGGGAGGTGGAG	miRNA	miR-1224
t0001098	21	337	TAAGGTAGTAGATTGTATAGT	miRNA	let-7f
t0001102	24	337	TGAGGTAGTAGGTTGTATAGTATC	miRNA	let-7-5p
t0001103	22	336	TGGGTCTTTGCGGGCGAGATGA	miRNA	miR-193a-5p
t0001104	19	335	TACAGTATAGATGATGTAC	miRNA	miR-144
t0001105	23	334	AAAAGCTGGGTTGAGAGGGCAAA	miRNA	miR-320b
t0001106	22	334	TTCAAGTAATTCAGGATAGGTA	miRNA	miR-26b
t0001109	21	333	TATTGCACTTGTCCCGGCCTA	miRNA	miR-92a
t0001110	22	333	AAACCTTACCATTACTGAGTTT	miRNA	miR-451
t0001108	21	333	TTCGGGGTCTGGGCGCGGCGA	miRNA	miR-4508
t0001107	22	333	TGAGGTATTAGGTTGTGTGGTT	miRNA	let-7b
t0001116	23	331	TGAGGTAGTAGTTTGTGCTGTAA	miRNA	let-7i
t0001115	21	331	TGAGGTAGTAGTTTGTGTGGT	miRNA	let-7b
t0001120	22	330	TGGAGAGAAAAGCAGTTCCTGA	miRNA	miR-185
t0001117	20	329	AAACCTTACCATTACTGAGT	miRNA	miR-451
t0001119	22	329	TGTAAACATCCCCGACTGGAAG	miRNA	miR-30d
t0001124	23	329	TACCACAGGGTAGAACCACGGCA	miRNA	miR-140
t0001125	20	329	TGAGGTAGTAGGTTGTATGG	miRNA	let-7c
t0001126	22	328	TCAGTGCACTACAGAACTTTGA	miRNA	miR-148a
t0001127	21	327	ACCACAGGGTAGAAACACGGA	miRNA	miR-140
t0001128	22	326	ATGAGGTAGTAGATTGTATAGT	miRNA	let-7f
t0001129	19	325	AAACCTTACCATTACTGAG	miRNA	miR-451
t0001133	21	324	TGAGGGGCAGAGAGCGAGACG	miRNA	miR-423-5p
t0001130	21	324	ATCACATTGCCAGGGATTTCC	miRNA	miR-23a
t0001136	20	324	TGAGGTAGTAGTTTGTGCTT	miRNA	let-7i
t0001137	18	323	TGGGTTTACGTTGGGAGA	miRNA	miR-629
t0001138	21	323	AAACCCTTACCATTACTGAGT	miRNA	miR-451
t0001140	20	323	AACTTTGAAGACTGAAGTGG	miRNA	miR-3526
t0001141	19	323	TGTAGTAGATTGTATAGTT	miRNA	let-7f
t0001139	22	322	TGAGGTGGTAGTTTGTGCTGTT	miRNA	let-7i
t0001142	21	322	TGAGGTAGTAGATTGGATAGT	miRNA	let-7f

t0001145	22	322	TGAGGTAGTAGAATGTATAGTT	miRNA	let-7f
t0001146	21	322	TGAGGTAGTAGATTATATAGT	miRNA	let-7f
t0001148	22	321	TGAGGGGCAGAGAGAGAGACTT	miRNA	miR-423-5p
t0001151	22	321	TAGCAGCTCGTAAATATTGGCG	miRNA	miR-16
t0001155	26	321	TGAGGTAGTAGGTTGTGTGGTTTATC	miRNA	let-7b
t0001157	21	320	TGAGGGGAAGAGAGCGAGACT	miRNA	miR-423-5p
t0001154	22	320	AAAAGCTGGTTTGAGAGGGCGA	miRNA	miR-320a
t0001159	22	319	TGAGGTAGTAGGTTGTATATTT	miRNA	let-7
t0001162	21	319	GAGGTAGTAGGTTGTATAGTT	miRNA	let-7
t0001160	22	318	TCCTGTACTGAGTTGCCCCGAG	miRNA	miR-486-5p
t0001161	21	318	TCCTGTACTGAGCTCCCCCGA	miRNA	miR-486-5p
t0001165	20	318	TGTGAGGTTGGCATTGTTGT	miRNA	miR-1294
t0001167	22	316	TGTGGTAGTAGATTGTATAGTT	miRNA	let-7f
t0001168	20	315	TCAGTGCACTACAGAACTTT	miRNA	miR-148a
t0001169	22	315	TCAGGTAGTAGATTGTATAGTT	miRNA	let-7f
t0001173	23	315	TGAGGTAGTAGGTTGTGTGGTGA	miRNA	let-7b
t0001171	22	314	TGGAGAGGAAGGCAGTTCCTGA	miRNA	miR-185
t0001170	23	314	ACCACAGGGTAGAATCACGGACA	miRNA	miR-140
t0001175	22	313	TTATAATACAACCTGATAAGTG	miRNA	miR-374a
t0001177	22	313	CAGCAGCACGTAAATATTGGCG	miRNA	miR-16
t0001178	24	313	TCTGGGCAACAAAGTGAGACCTGA	miRNA	miR-1285
t0001180	22	313	TGAGGTAGTAGGTTGTGTGGAT	miRNA	let-7b
t0001181	21	312	AGCTACATTGTCTGCTGGGTT	miRNA	miR-221
t0001182	23	311	TGAGGGGCAGAGACCGAGACTTT	miRNA	miR-423-5p
t0001184	22	311	CATTGCACTTGTCTCGGGCTGA	miRNA	miR-25
t0001186	22	311	TGAGGTGGTAGGTTGTATAGTT	miRNA	let-7
t0001193	20	310	ACTGGACTTGGAGTCAGAAG	miRNA	miR-378
t0001192	21	309	TGAGGGGCAGAGAGCGAGGCT	miRNA	miR-423-5p
t0001191	23	309	TGGAGAGAAAGGCAGTTCCTGTA	miRNA	miR-185
t0001198	24	309	TACCACAGGGTAGAACCACGGAAG	miRNA	miR-140
t0001196	20	308	GAGGTAGTAGGTTGTATAGT	miRNA	let-7
t0001199	22	307	TGGAGAGAAAGCCAGTTCCTGA	miRNA	miR-185
t0001200	21	306	TGGAAGGTAGACGGCCAGAGA	miRNA	miR-3190
t0001201	24	305	ACTGGACTTGGAGTCAGAAGGATC	miRNA	miR-378f
t0001202	20	305	TAGCAGCATATAATGGTTTG	miRNA	miR-15a
t0001204	22	305	ACCACAGGGTAGAACCACGGCC	miRNA	miR-140
t0001206	22	304	TGACGTAGTAGATTGTATAGTT	miRNA	let-7f
t0001208	23	303	TACCACAGGGTAGAACAACGGAC	miRNA	miR-140
t0001205	22	303	TGAGGTAGTAGGCTGTGTGGTT	miRNA	let-7b
t0001210	21	302	TGAGGTAGTAGATTGTATAGC	miRNA	let-7f
t0001213	21	302	AGAGGTAGTAGGTTGCATAGA	miRNA	let-7d
t0001214	21	301	TACGTCATCGTTGTCATCGTC	miRNA	miR-598
t0001215	21	301	CGGGGCAGCTCAGTACAGGAT	miRNA	miR-486-3p
t0001217	20	300	TGAGGGAGTAGGTTGTATAG	miRNA	miR-1827
t0001218	19	300	TAGCAGCAAATCATGGTTT	miRNA	miR-15b
t0001220	22	300	TGAGGTAGTAGATTGTATAATT	miRNA	let-7f
t0001225	20	299	TGAGGTAGTAAGTTGTATTG	miRNA	miR-98
t0001223	21	299	AAACTGTTACCATTACTGAGT	miRNA	miR-451
t0001224	20	299	TTAGGTAGTAGATTGTATAG	miRNA	let-7f
t0001226	20	299	TGAGGTAGTAGATTGCATAG	miRNA	let-7f
t0001231	21	298	CCGCACTGTGGGTACTTGCTG	miRNA	miR-106b*
t0001232	22	297	AAACCGTTACTATTACTGAGTT	miRNA	miR-451
t0001234	22	297	TGGAGAGAAATGCAGTTCCTGA	miRNA	miR-185
t0001235	23	297	AGGTAGATAGAACAGGTCTTGTT	miRNA	miR-1839
t0001237	21	297	TCACAGTGAACCGGTCTCTTT	miRNA	miR-128
t0001238	21	297	CAGCATTGTACAGGGCTATGA	miRNA	miR-103a
t0001240	22	296	ACCACAGGGTAGAACTACGGAT	miRNA	miR-140
t0001241	25	295	CACCCGTAGAACCGACCTTGCGATC	miRNA	miR-99b

t0001239	21	295 TATTGCACTTGTCCCGGCCTT	miRNA	miR-92b
t0001247	19	295 TAGCAGAACATCATGGTTT	miRNA	miR-15b
t0001245	23	295 TAGCAGCACATAATGGTTTGTGA	miRNA	miR-15a
t0001248	23	295 TCTCCCAACCCTTGTACCAGTGA	miRNA	miR-150
t0001249	21	295 TGAGGTAGTAGATTCTATAGT	miRNA	let-7f
t0001251	22	294 TGTAGAGCAGGGAGCAGGAAGA	miRNA	miR-4732-5p
t0001256	23	294 TAAAGTGCTTATAGTGCAGGTAA	miRNA	miR-20
t0001257	22	294 TGGAGAGAAAGACAGTTCCTGA	miRNA	miR-185
t0001252	18	294 TAGCAGCACGTAAATATT	miRNA	miR-16
t0001253	21	294 ACCACAGGGTAGAACCCCGGA	miRNA	miR-140
t0001255	19	294 TGAGGTAGTAGGTGTGGTT	miRNA	let-7a
t0001258	24	293 AAAAGCTGGGTTGAGAGGGCGTAA	miRNA	miR-320d
t0001263	22	293 TGGAGAGAAAGGCAGTTCCTAA	miRNA	miR-185
t0001264	22	293 TGAGGTAGTAGGTTGCATAGTT	miRNA	let-7
t0001261	22	292 CATGCCTTGAGTGTAGGACCGT	miRNA	miR-532-5p
t0001266	23	292 AAACCGTTACCATTATTGAGTTT	miRNA	miR-451
t0001259	20	292 AGCTACATTGTCTGCTGGGT	miRNA	miR-221
t0001260	23	292 TGGAGAGAAAGGAAGTTCCTGAA	miRNA	miR-185
t0001271	19	292 ACCACTGACCGTTGACTGT	miRNA	miR-181a-2*
t0001267	21	291 TGAGGTAGTAGGTTGTATAGA	miRNA	let-7
t0001268	22	290 GAGGGGCAGAGAGCGAGACTTT	miRNA	miR-423-5p
t0001270	26	290 TGTAAACATCCTTGACTGGAAGCATC	miRNA	miR-30e-5p
t0001273	19	290 TAGCAGCATATCATGGTTT	miRNA	miR-15b
t0001269	26	289 CTTTGAAGACTGAAGTGGAGAAGGGT	miRNA	miR-3526
t0001276	22	289 TGAGGTAGTAGGTTGTATAGTG	miRNA	let-7-5p
t0001279	22	288 AAACCGTTACCATTACTGAGTA	miRNA	miR-451
t0001274	22	288 TGGAGTGAAAGGCAGTTCCTGA	miRNA	miR-185
t0001287	22	288 TAGCAGCACATCATGGTTTTCA	miRNA	miR-15c
t0001281	22	287 AAACAGTTACCATTACTGAGTT	miRNA	miR-451
t0001286	23	287 TGATGGGCAGAGAGCGAGACTTT	miRNA	miR-423-5p
t0001285	22	287 AAGCTGCCAGTTGAAGAACTGT	miRNA	miR-22-3p
t0001282	24	287 ACCACAGGGTAGAACCACGGACAA	miRNA	miR-140
t0001288	24	286 TCTGGGCACAGGCGGATGGACAGA	miRNA	miR-5107
t0001292	18	285 TGGCTCAGTTCAGCAGGA	miRNA	miR-24
t0001293	22	285 TAGCAGCACGTAAATATTGGGG	miRNA	miR-16
t0001290	23	284 AAAGGTAGATAGAACAGGTCTTG	miRNA	miR-1839
t0001294	26	284 TGAGATGAAGCACTGTAGCTCTTATC	miRNA	miR-143
t0001295	22	283 CATTGCACTTGTCTCGGTCTGC	miRNA	miR-25
t0001298	21	282 TGAGGGGCAGAGACCGAGACT	miRNA	miR-423-5p
t0001297	23	282 TGAGGGGCAGAGAGCGAGGCTTT	miRNA	miR-423-5p
t0001299	21	282 TTGGGGAAACGGCCGCTGAGT	miRNA	miR-2110
t0001302	22	282 CGAGGTAGTAGGTTGTGTGGTT	miRNA	let-7b
t0001303	20	281 AAACCGTTACCATTACTTAG	miRNA	miR-451
t0001301	23	281 TGAGGGGCAGAGAGCGATACTTT	miRNA	miR-423-5p
t0001300	23	280 TTGAAGACTGAAGTGGAGAAGGG	miRNA	miR-3526
t0001305	22	280 TGGAGAGAAAGGCGGTTCCTGA	miRNA	miR-185
t0001306	20	280 TATGGCACTGGTAGAATTCA	miRNA	miR-183
t0001307	23	279 AAACCCTTACCATTACTGAGTTT	miRNA	miR-451
t0001310	22	279 TGAGGGGCAGAGAGCGAGAATT	miRNA	miR-423-5p
t0001308	23	279 TGGAGAGAAAGGCAGTTCCTGCA	miRNA	miR-185
t0001312	21	278 GTGGAAGGTAGACGGCCAGAG	miRNA	miR-3190
t0001316	22	278 TAGCAGCACATTATGGTTTACA	miRNA	miR-15b
t0001317	25	278 TGAGGTAGTAGGTTGTGTGGTTAAA	miRNA	let-7b
t0001315	20	277 TCTGGGCACAGGCGGATGGA	miRNA	miR-5107
t0001314	21	277 AAACCGTTACCATTACCGAGT	miRNA	miR-451
t0001319	20	277 AACGGAATCCCAAAAGCAGC	miRNA	miR-191
t0001321	21	277 TAGCAGCACGTAAATATTGGG	miRNA	miR-16
t0001322	21	277 TGAGGTAGTAGATTGTATACT	miRNA	let-7f

t0001324	21	276	TGAGGTAGAAGATTGTATAGT	miRNA	let-7f
t0001326	26	275	TTGAAGACTGAAGTGGAGAAGGGTTT	miRNA	miR-3526
t0001332	20	274	TAGCAGAACATCATGGTTTA	miRNA	miR-15b
t0001330	21	273	TCCTGTACTGAGCTGCCCCGT	miRNA	miR-486-5p
t0001328	22	273	AGAGGTAGTAGATTGTATAGTT	miRNA	let-7f
t0001334	22	272	AAACCGTCACCATTACTGAGTT	miRNA	miR-451
t0001333	24	271	TCCTGTACTGAGCTGCCCCGAGTT	miRNA	miR-486-5p
t0001335	24	271	AAACCTTTACCATTACTGAGTTTA	miRNA	miR-451
t0001341	22	270	TCCTGTACTGAGCTGCCTTGAG	miRNA	miR-486-5p
t0001342	21	270	AAACCGTTACCATTACTAAGT	miRNA	miR-451
t0001339	21	270	AAACCGTTAACATTACTGAGT	miRNA	miR-451
t0001337	24	270	AAAAGCTGGGTTGAGAGGGCGAGA	miRNA	miR-320a
t0001344	22	270	TAGCAGCACGGAAATATTGGCG	miRNA	miR-16
t0001345	23	270	ACCACAGGGTAGAACCACGGATT	miRNA	miR-140
t0001348	21	270	TGAGTTAGTAGGTTGTGTGGT	miRNA	let-7b
t0001351	20	269	TCTCCCTTCCTGCCCTGGCT	miRNA	miR-4685-3p
t0001354	23	269	TGAGGGGCAGAGCGCGAGACTTT	miRNA	miR-423-5p
t0001353	22	269	TAGCAGCGCGTAAATATTGGCG	miRNA	miR-16
t0001358	21	268	TCTGGGCACAGGCGGATGGAC	miRNA	miR-5107
t0001361	21	268	TGAGGTATTAGGTTGTGTGGT	miRNA	let-7b
t0001362	23	268	TGAGGTAGTAGGTTGTATAGTAA	miRNA	let-7-5p
t0001366	20	267	TTGTACTGAGCTGCCCCGAG	miRNA	miR-486-5p
t0001364	23	267	TACCACAGGGTAGAACCCCGGAT	miRNA	miR-140
t0001372	23	266	TGAGGGGCAGAGATCGAGACTTT	miRNA	miR-423-5p
t0001365	23	266	TGGAGAGAAAGGCAGTTCCTGGA	miRNA	miR-185
t0001371	23	265	TTGGGGAAACGGCCGCTGAGTGA	miRNA	miR-2110
t0001363	20	265	TGAGGCAGTAGATTGTATAG	miRNA	let-7f
t0001367	21	265	TGAGGTAGTAGATTGTGTGGT	miRNA	let-7b
t0001376	20	264	TCTCCCAACCCTTGTACCAG	miRNA	miR-150
t0001377	20	264	TACCGCACTGTGGGTACTTG	miRNA	miR-106b*
t0001374	20	264	TGAGGTAGTAGATCGTATAG	miRNA	let-7f
t0001378	24	263	AAAAGCTGGGTTGAGAGGGCGTTT	miRNA	miR-320d
t0001381	19	263	TGGCTCAGTTCAGCAGGAA	miRNA	miR-24
t0001379	24	263	GACTGGGGCGGTACATCTGTTAAA	miRNA	miR-219-2-3p
t0001383	22	263	TAGCAGCACGTAAATATCGGCG	miRNA	miR-16
t0001382	21	263	ACCACAGGGAAGAACCACGGA	miRNA	miR-140
t0001387	22	263	TGAGGAAGTAGGTTGTATAGTT	miRNA	let-7
t0001385	22	261	TGAGGGGCAGAGAGTGAGACTT	miRNA	miR-423-5p
t0001388	24	261	TTGGGGAAACGGCCGCTGAGTGAG	miRNA	miR-2110
t0001390	21	261	TGGAGAGAAAGGCAGTTACTG	miRNA	miR-185
t0001391	26	261	TACCCTGTAGATCCGAATTTGTGATC	miRNA	miR-10
t0001394	22	261	TGAGGTAGTAGGTTGTCTGGTT	miRNA	let-7b
t0001393	22	260	TAGCAGCACATCATGGTTTACC	miRNA	miR-15b
t0001397	21	260	TAGCAGCACATAATGGGTTGT	miRNA	miR-15a
t0001405	24	260	AAGCAGCATTGTACAGGGCTATGA	miRNA	miR-103a
t0001406	22	260	AGCAGCATTGTACATGGCTATG	miRNA	miR-103a
t0001404	21	260	TGATGTAGTAGGTTGTGTGGT	miRNA	let-7b
t0001402	22	259	TGAGGTAGCAGGTTGTGTGGTT	miRNA	let-7b
t0001400	21	259	TGAGGTGGTAGGTTGTGTGGT	miRNA	let-7b
t0001401	24	258	TTTGGCAATGGTAGAACTCACACT	miRNA	miR-182
t0001410	21	258	TGATGTAGTAGATTGTATAGT	miRNA	let-7f
t0001408	23	256	TCCTGTACTGAGCTGCCCCGAGG	miRNA	miR-486-5p
t0001407	23	256	TCCTGTACTGAGCTGTCCCGAGA	miRNA	miR-486-5p
t0001411	22	256	AGGGGCAGAGAGCGAGACTTTT	miRNA	miR-423-5p
t0001409	25	255	TAGCTTATCAGACTGATGTTGAATC	miRNA	miR-21
t0001419	22	255	TACCAGCACGTAAATATTGGCG	miRNA	miR-16
t0001413	22	255	GGAGGTAGTAGATTGTATAGTT	miRNA	let-7f
t0001412	23	254	AAAAGCTGGGTTGAGAGGGCATA	miRNA	miR-320d

t0001414	22	253 TCCTGTATTGAGCTGCCCCGAG	miRNA	miR-486-5p
t0001415	19	253 AACATTCATTGCTGTCGGT	miRNA	miR-181b
t0001422	22	252 TCCTGTACTGAGCTCCCCCGAG	miRNA	miR-486-5p
t0001423	21	251 GAGGTAGTAGGTTGTGTGGTT	miRNA	let-7b
t0001424	21	250 ATTGCACTTGTCCCGGCCTGT	miRNA	miR-92c
t0001425	21	250 TGGAGAGAAAGGCAGTTTTTG	miRNA	miR-185
t0001427	21	250 TGAGATGAAGCACTGTAGATC	miRNA	miR-143
t0001428	21	250 TGAGGTAGTAGGTTCTGTGGT	miRNA	let-7b
t0001429	22	249 TGAGGGGCAGAGAGCTAGACTT	miRNA	miR-423-5p
t0001430	22	249 TGGATAGAAAGGCAGTTCCTGA	miRNA	miR-185
t0001432	23	249 TACCACAGGGTAGAACCAAGGAC	miRNA	miR-140
t0001431	23	249 TGAGGTAGTAGGTTGTGTGGATC	miRNA	let-7b
t0001436	19	248 TAGCAGCGGGAACAGTTCT	miRNA	miR-503
t0001438	23	248 AAAAGCTGGGTTGAGAGGGCGGT	miRNA	miR-320d
t0001440	22	248 AAAGTGCTTACAGTGCAGGTAG	miRNA	miR-17
t0001441	24	248 TACCACAGGGTAGAATCACGGACA	miRNA	miR-140
t0001443	21	248 TGAGGTAGTAGATTGTAAAGT	miRNA	let-7f
t0001447	22	247 TGAGGGGCAGAGAGCGAGCCTT	miRNA	miR-423-5p
t0001448	22	247 AGCAGTATTGTACAGGGCTATG	miRNA	miR-103a
t0001446	21	246 AAACCGTTATCATTACTGAGT	miRNA	miR-451
t0001445	26	246 AAACCGTTACCATTACTGAGTTTAGA	miRNA	miR-451
t0001455	20	246 CGAGGTAGTAGATTGTATAG	miRNA	let-7f
t0001453	22	245 TGAGGGGCAGATAGCGAGACTT	miRNA	miR-423-5p
t0001450	22	245 TGGAGAGCAAGGCAGTTCCTGA	miRNA	miR-185
t0001452	25	245 TACCACAGGGTAGAACCACGGACAA	miRNA	miR-140
t0001461	21	245 TGCGGTAGTAGATTGTATAGT	miRNA	let-7f
t0001457	21	244 CCACAGGGTAGAACCACGGAT	miRNA	miR-140
t0001460	23	244 TGAGGTAGTAGATTGTATAGTGA	miRNA	let-7f
t0001459	23	243 GACTGGGGCGGTACATCTGTTAA	miRNA	miR-219-2-3p
t0001458	21	243 AGACCTACTTATCTACCAACA	miRNA	miR-1839-3p
t0001463	24	243 TCAGTGCATGACAGAACTTGGATC	miRNA	miR-152
t0001464	23	242 CAAAGTGCTGTTCGTGCAGGTAA	miRNA	miR-93
t0001467	22	242 TAGCAGCACATAATGGTTTGTA	miRNA	miR-15a
t0001468	23	242 TACCACAGGGTAGAAACACGGAC	miRNA	miR-140
t0001469	20	242 CTATACGACCTGCTGCCTTT	miRNA	let-7d*
t0001476	21	238 TCCTGTACTGAGCTGCTGCGA	miRNA	miR-486-5p
t0001472	26	238 TGAGGTAGTAGGTTGTATGGTTAATC	miRNA	let-7b
t0001473	20	237 TCCTGTACTGAGCTGCCCTG	miRNA	miR-486-5p
t0001470	24	237 GAAAAGCTGGGTTGAGAGGGCGTA	miRNA	miR-320d
t0001479	24	237 AAAAGCTGGGTTGAGAGGGCGGTA	miRNA	miR-320d
t0001480	20	237 TGAGGTAGTAGATTGTATAT	miRNA	let-7f
t0001483	22	237 TGAGGCAGTAGGTTGTATAGTT	miRNA	let-7
t0001481	23	236 AAACCGTTATCATTACTGAGTTT	miRNA	miR-451
t0001484	22	234 TGAGGTAGTATGTTGTGTGGTT	miRNA	let-7b
t0001490	27	233 TGAAGACTGAAGTGGAGAAGGGTTTCT	miRNA	miR-3526
t0001491	23	233 AAAAGCTGGGTTGAGAGGGCGCT	miRNA	miR-320d
t0001486	23	233 TGTAAACATCCCCGACTGGAAGA	miRNA	miR-30d
t0001487	22	232 TAGCAACACGTAAATATTGGCG	miRNA	miR-16
t0001498	22	232 TGAGGTAGTAGGTTGCGTGGTT	miRNA	let-7b
t0001499	22	231 AAACCGTTAACATTACTGAGTT	miRNA	miR-451
t0001500	20	230 TGAGGAAGTAGATTGTATAG	miRNA	let-7f
t0001503	21	230 TGAGGTAGTAGGTTGTATTGT	miRNA	let-7
t0001505	21	229 TGAGAACCACGTCTGCTCTGA	miRNA	miR-589
t0001502	22	229 TAGCAGCAAGTAAATATTGGCG	miRNA	miR-16
t0001504	21	229 TAGCAGCACGTAAATATTGTG	miRNA	miR-16
t0001507	23	229 ACCACAGGGTAGAACCACGGAGA	miRNA	miR-140
t0001512	22	228 ATGACACGATCACTCCCGTTGA	miRNA	miR-425-5p
t0001515	23	228 TGAGGGGCAGAGAGCCAGACTTT	miRNA	miR-423-5p

t0001513	21	228	TTATAATACAACCTGATAAGT	miRNA	miR-374a
t0001514	25	227	TGGAGAGAAAGGCAGTTCCTGAAAA	miRNA	miR-185
t0001521	22	227	TACTACAGGGTAGAACCACGGA	miRNA	miR-140
t0001518	22	227	AGAGGTAGTAGGGTGCATAGTT	miRNA	let-7d
t0001523	20	226	ATATAATACAACCTGCTAAG	miRNA	miR-374
t0001527	23	226	TAGCAGCACATCATGGTTTACAA	miRNA	miR-15b
t0001528	23	226	ACCACAGGGTAGAACCACGGATG	miRNA	miR-140
t0001529	21	225	TGAGGGGCAGAGATCGAGACT	miRNA	miR-423-5p
t0001531	21	225	TGGAGAGAAAGGCAGTTTCTG	miRNA	miR-185
t0001533	22	225	TAGAGAGAAAGGCAGTTCCTGA	miRNA	miR-185
t0001530	23	224	TCCTGTACTGAGCTGCTCCGAGA	miRNA	miR-486-5p
t0001541	21	224	TCCTGTATTGAGCTGCCCCGA	miRNA	miR-486-5p
t0001536	20	224	TAGGTAGTAGATTGTATAGT	miRNA	let-7f
t0001543	20	224	TGAGGTAGTAGTTTGTATAG	miRNA	let-7f
t0001548	21	222	TGAGGGGTAGAGAGCGAGACT	miRNA	miR-423-5p
t0001547	23	222	AAAAGCTGGGTTGAGAGGGCGAC	miRNA	miR-320a
t0001545	20	222	TGTAAACATCCCCGACTGGA	miRNA	miR-30d
t0001549	22	222	TCAGTGCATGACAGAACTTGGT	miRNA	miR-152
t0001555	21	221	ACCACAGGGTAGAACCAAGGA	miRNA	miR-140
t0001552	22	221	CCACAGGGTAGAACCACGGACT	miRNA	miR-140
t0001558	21	221	TGAGGTAGTAGTTTGTGCTTT	miRNA	let-7i
t0001556	22	221	TGAGGTAGGAGGTTGTATAGTT	miRNA	let-7
t0001560	25	220	TGGCTCAGTTCAGCAGGAACAGATC	miRNA	miR-24b
t0001563	22	218	AAACCGTTATCATTACTGAGTT	miRNA	miR-451
t0001564	23	218	CGAGGGGCAGAGAGCGAGACTTT	miRNA	miR-423-5p
t0001562	20	218	TAGCAGCACATCATGGTTTT	miRNA	miR-15c
t0001565	23	218	AGCAGCATTGTACAGGGCTATTT	miRNA	miR-107
t0001570	21	217	AGCAGCATTGTACAGGGCTAG	miRNA	miR-103a
t0001571	22	217	TGAGGTAGTAGGTTGTGTGGTC	miRNA	let-7b
t0001569	22	215	AAACCGTTACCATTACTGAGGT	miRNA	miR-451
t0001568	24	215	TAGCAGCACGTAAATATTGGCGAG	miRNA	miR-16
t0001574	23	215	TCAGTGCACTACAGAACTTTGAA	miRNA	miR-148a
t0001575	21	215	AGCAGCATTGTACAGGGTTAT	miRNA	miR-103a
t0001572	21	215	TGAGGTAATAGATTGTATAGT	miRNA	let-7f
t0001576	21	215	TGAGGTAGCAGGTTGTGTGGT	miRNA	let-7b
t0001582	22	214	TGGAGAGAAAGGCACTTCCTGA	miRNA	miR-185
t0001589	21	214	AGCAGCATTGTACAGGGCTTT	miRNA	miR-107b
t0001586	22	213	TAAAGTGCTGACAGTGCAGATT	miRNA	miR-106b
t0001584	21	212	GCCCCTGGGCCTATCCTAGAA	miRNA	miR-331
t0001585	24	212	AATCTGAGAAGGCGCACAAGGTTT	miRNA	miR-3200-5p
t0001593	22	212	TAACAGCACGTAAATATTGGCG	miRNA	miR-16
t0001590	24	211	TCCTGTACTGAGCTGCCCCGAGGA	miRNA	miR-486-5p
t0001606	23	211	TGGAATGTAAAGAAGTATGTATC	miRNA	miR-1a
t0001601	22	211	TAGCAGCACATCATGGTTTATA	miRNA	miR-15b
t0001598	22	211	CACCACAGGGTAGAACCACGGA	miRNA	miR-140
t0001604	22	210	TATTGCATTTGTCCCGGCCTGT	miRNA	miR-92a
t0001599	23	210	TGAGGGGCAGAGAGCGGGACTTT	miRNA	miR-423-5p
t0001600	20	210	TGAGGTAGGAGATTGTATAG	miRNA	let-7e
t0001612	23	209	AAAAGCTGGGTTGAGAGGGCAAT	miRNA	miR-320b
t0001613	24	209	CTGACCTATGAATTGACAGCCAGT	miRNA	miR-192
t0001616	23	209	TAGCAGCACATAATGGTTTGTGG	miRNA	miR-15a
t0001623	22	209	AGAGGTAGTAGGTTGTATAGTT	miRNA	let-7d
t0001619	22	209	TAAGGTAGTAGGTTGTGTGGTT	miRNA	let-7b
t0001617	22	209	TGAGGTAGTGGGTTGTGTGGTT	miRNA	let-7b
t0001624	19	208	AGGGACGGGACGCGGTGCA	miRNA	miR-92b*
t0001628	20	208	TACGTCATCGTTGTCATCGT	miRNA	miR-598
t0001625	21	208	TGAGGGGCAGAGGGCGAGACT	miRNA	miR-423-5p
t0001627	22	208	AAAAGTCGGGTTGAGAGGGCGA	miRNA	miR-320c

t0001631	22	208	TGGAGAGAAAGGCAGCTCCTGA	miRNA	miR-185
t0001632	22	208	TAGCAGCACATCATGGGTTACA	miRNA	miR-15b
t0001630	22	208	TACCACAGGGTAGAATTACGGA	miRNA	miR-140
t0001635	20	207	TCTGTACTGAGCTGCCCCGA	miRNA	miR-486-5p
t0001634	23	207	AAACCGTTACCATTACTGAGTTG	miRNA	miR-451
t0001637	22	207	TGTGCAAATCCATGCAAAACTG	miRNA	miR-19b
t0001639	22	207	TCTGACCTATGAATTGACAGCC	miRNA	miR-192
t0001636	22	207	TAGCAGCACCTAAATATTGGCG	miRNA	miR-16
t0001638	21	207	CATAAAGTAGAAAGCACCACT	miRNA	miR-142-5p
t0001646	23	207	TACCACAGGGTAGAATCACGGAC	miRNA	miR-140
t0001644	22	207	AGCAGCATTGTATAGGGCTATG	miRNA	miR-103a
t0001649	20	206	AACCGTTACCATTACTGAGT	miRNA	miR-451
t0001642	21	206	TGACCTATGAATTGACAGCTA	miRNA	miR-192
t0001641	21	206	TCAGTGCATGACAGAACTTGG	miRNA	miR-152
t0001647	20	205	TGGGGTAGTAGATTGTATAG	miRNA	let-7f
t0001645	21	204	TAGCAGCGGGAACAGTTCTGA	miRNA	miR-503
t0001650	21	204	AAACCGTTACCATTACTGCGT	miRNA	miR-451
t0001652	20	204	AAACCGTTACCATTACTAAG	miRNA	miR-451
t0001660	22	204	TGAGGTAGTACATTGTATAGTT	miRNA	let-7f
t0001653	24	204	TGAGGTAGTAGGTTGTGTGGTAGA	miRNA	let-7b
t0001654	23	203	TCCTGTACTGAGCTGCCCCGAAA	miRNA	miR-486-5p
t0001658	21	203	AACCGTTACCATTACTGAGTT	miRNA	miR-451
t0001656	24	203	TGAGGGGCAGAGAGCGAGACTTTG	miRNA	miR-423-5p
t0001651	21	203	AACATTCAACGCTGTCGGTGA	miRNA	miR-181a
t0001663	21	203	TGGGGTAGTAGGTTGTGTGGT	miRNA	let-7b
t0001664	21	203	CGAGGTAGTAGGTTGTGTGGT	miRNA	let-7b
t0001666	21	203	TGATGTAGTAGGTTGTATAGT	miRNA	let-7
t0001668	24	202	TCCTGTACTGAGCTGCCCCGACGG	miRNA	miR-486-5p
t0001676	22	202	AAACCGTTACCATTACTGGGTT	miRNA	miR-451
t0001670	22	202	TGAGGGGCAGACAGCGAGACTT	miRNA	miR-423-5p
t0001673	20	202	AAAGCTGGGTTGAGAGGGCA	miRNA	miR-320b
t0001671	23	202	CATTGCACTTGTCTCGGTCTGAT	miRNA	miR-25
t0001675	26	202	TGGCTCAGTTCAGCAGGAACAGTATC	miRNA	miR-24
t0001685	20	202	GCAGCACGTAAATATTGGCG	miRNA	miR-16
t0001686	21	202	TACAGTACTGTGATAACTGAC	miRNA	miR-101c
t0001678	22	201	TAGCGGCACGTAAATATTGGCG	miRNA	miR-16
t0001680	21	200	TGGGTTTACGTTGGGAGAACT	miRNA	miR-629
t0001687	21	200	AAACAGTTACCATTACTGAGT	miRNA	miR-451
t0001688	20	200	GAGGGGCAGAGAGCGAGACT	miRNA	miR-423-5p
t0001681	21	200	TGAGATAGTAGATTGTATAGT	miRNA	let-7f
t0001692	23	199	TGAGGGGCAGAGAGCAAGACTTT	miRNA	miR-423-5p
t0001694	23	199	TGAGGGGCGGAGAGCGAGACTTT	miRNA	miR-423-5p
t0001689	20	199	CTGTGCGTGTGACAGCGGCT	miRNA	miR-210
t0001693	22	199	TCAGTGCATCACAGAACTTTGT	miRNA	miR-148b-3p
t0001691	18	199	TCAGTGCATCACAGAACT	miRNA	miR-148b-3p
t0001698	18	199	TGAGGTAGTAGATTATAG	miRNA	let-7f
t0001695	22	198	ATTCTAATTTCTCCACGTCTTT	miRNA	miR-576-5p
t0001697	23	198	AAAAGCTGGGTTGAGAGGGCATT	miRNA	miR-320d
t0001704	26	198	TAGCAGCACGTAAATATTGGCGAAAA	miRNA	miR-16
t0001708	18	198	CATAAAGTAGAAAGCACT	miRNA	miR-142-5p
t0001714	22	198	AGCAGCATTGTACAGGGCTAAT	miRNA	miR-107
t0001707	22	198	TAAAGTGCTGACAGTGCAGAAA	miRNA	miR-106b
t0001710	22	197	TATTGCACTTGTCTCGGTCTGA	miRNA	miR-25
t0001715	21	197	TGAGGAAGTAGGTTGTATAGT	miRNA	let-7
t0001720	19	196	TACGTCATCGTTGTCATCG	miRNA	miR-598
t0001719	22	196	TGGAGAGAAAGGCAATTCCTGA	miRNA	miR-185
t0001721	21	196	TGAGGTAGTAGATTGTCTAGT	miRNA	let-7f
t0001724	22	195	TACCCATTGCATATCGGAGTTG	miRNA	miR-660

t0001716	21	195	TCAGGTAGTAGATTGTATAGT	miRNA	let-7f
t0001734	19	194	AAGGGCTGGGTCGGTCGGG	miRNA	miR-4651
t0001732	22	194	TGGAGAGAAAGGCAGTAGTTGA	miRNA	miR-4306
t0001733	21	194	TGGAGAGAAAGGCAGTTCCTT	miRNA	miR-185
t0001731	22	194	TAGCAGCACGTAAATCTTGGCG	miRNA	miR-16
t0001729	22	194	TAGCAGCCCGTAAATATTGGCG	miRNA	miR-16
t0001727	20	194	CAGCATTGTACAGGGCTATG	miRNA	miR-103a
t0001736	21	193	TATTGCACTCGTCCCGGCCTT	miRNA	miR-92
t0001735	20	193	AAACCCTTACCATTACTGAG	miRNA	miR-451
t0001741	24	193	AAACCGTTACCATTACTGAGTTTG	miRNA	miR-451
t0001738	23	193	AAACCGTCACCATTACTGAGTTT	miRNA	miR-451
t0001742	24	193	TGACCTATGAATTGACAGCCAGAA	miRNA	miR-192
t0001744	23	193	CTGACCTATGAATTGACAGCATC	miRNA	miR-192
t0001747	22	193	CCCATAAAGTAGAAAGCACTAA	miRNA	miR-142
t0001753	26	193	TGGAATGTAAAGAAGTATGTACCATC	miRNA	miR-1-3p
t0001748	24	193	TGAGGTAGTAGGTTGTATAGTTAA	miRNA	let-7
t0001751	23	192	AGGTGGAAGGTTGTAGGCATGTA	miRNA	miR-965-5p
t0001749	21	192	TGAGGGGCAGAGAGCGCGACT	miRNA	miR-423-5p
t0001756	20	192	TAGCTTATCAGACTGATGTT	miRNA	miR-21
t0001754	22	192	TAAAGTGCTGACAGTGCAGATG	miRNA	miR-106b
t0001760	21	192	AGCAGTATTGTACAGGGCTAT	miRNA	miR-103a
t0001758	22	191	TGAGGTAGTAAGTTGTATTGTT	miRNA	miR-98
t0001762	21	191	CATGCCTTGAGTGTAGGACCG	miRNA	miR-532-5p
t0001766	24	191	TCCTGTACTGAGCTGCCCCGAAGA	miRNA	miR-486-5p
t0001761	22	191	TACCACAGCGTAGAACCACGGA	miRNA	miR-140
t0001765	23	190	TAAAGTGCTGACAGTGCAGATAG	miRNA	miR-106b
t0001759	22	190	AGAGGTGGTAGGTTGCATAGTT	miRNA	let-7d
t0001767	22	189	TGGAGAGAAAGTCAGTTCCTGA	miRNA	miR-185
t0001768	25	189	TACAGTACTGTGATAGCTGAAGATC	miRNA	miR-101
t0001770	20	189	TGAGGTAGTAGTTTGTGTTG	miRNA	let-7i
t0001776	20	189	TGAGGTAGTAGATTGTATTG	miRNA	let-7f
t0001778	20	188	AAACTGTTACCATTACTGAG	miRNA	miR-451
t0001771	22	188	TAGCAGGACGTAAATATTGGCG	miRNA	miR-16
t0001772	22	188	TACTAGACTGAAGCTCCTTGAG	miRNA	miR-151-3p
t0001785	18	187	TGTACTGAGCTGCCCCGA	miRNA	miR-486-5p
t0001782	23	187	TGAGGAGCAGAGAGCGAGACTTT	miRNA	miR-423-5p
t0001783	23	187	TCACACAGAAATCGCACCCGTCA	miRNA	miR-342-3p
t0001781	20	186	TCTCTCGGCTCCTCGCGGCT	miRNA	miR-3615
t0001789	22	186	TTGAAGACTGAAGTGGAGAAGG	miRNA	miR-3526
t0001786	19	186	AACGGAATCCCAAAAGCAG	miRNA	miR-191
t0001792	22	186	TAGCAGCACGTGAATATTGGCG	miRNA	miR-16
t0001794	20	186	TACCACAGGGTAGAACCACG	miRNA	miR-140
t0001796	24	185	TTCACAGTGGCTAAGTTCTGCATC	miRNA	miR-27b
t0001795	23	185	TTCAAGTAATCCAGGATAGGATC	miRNA	miR-26a
t0001809	22	185	CATTGTACTTGTCTCGGTCTGA	miRNA	miR-25
t0001804	22	185	TGAGGTAGTAGGTTGTATTGTT	miRNA	let-7
t0001798	19	184	TGTACTGAGCTGCCCCGAG	miRNA	miR-486-5p
t0001812	21	184	AAACCGTCACCATTACTGAGT	miRNA	miR-451
t0001810	24	184	CTGGACTTGGAGTCAGAAGGCATC	miRNA	miR-378
t0001811	23	184	TAAAGTGCTGACAGTGCAGATAA	miRNA	miR-106b
t0001815	22	184	TACAGTACTGTGATAACTGACA	miRNA	miR-101c
t0001817	21	184	TGAGGTCGTAGATTGTATAGT	miRNA	let-7f
t0001821	21	184	TGAGGTAGTAGGCTGTGTGGT	miRNA	let-7b
t0001814	20	183	TAGCAGCGGGAACAGTTCTG	miRNA	miR-503
t0001816	22	183	ATCCTGTACTGAGCTGCCCCGA	miRNA	miR-486-5p
t0001823	23	183	TGAGGGGCAGAGAGCGAGACTCT	miRNA	miR-423-5p
t0001824	22	183	AAAAGCTGGGTTGAGAGGGTGA	miRNA	miR-320d
t0001828	25	183	TGGACGGAGAACTGATAAGGGAATC	miRNA	miR-184-3p

t0001827	24	183	TACCACAGGGTAGAACTACGGACA	miRNA	miR-140
t0001837	24	183	TACCATAGGGTAGAACCACGGACA	miRNA	miR-140
t0001830	19	182	AGGGGCAGAGAGCGAGACT	miRNA	miR-423-5p
t0001838	21	182	TGAGTGGCAGAGAGCGAGACT	miRNA	miR-423-5p
t0001835	21	182	TTTGAGGCTACAGTGAGATGT	miRNA	miR-1304
t0001834	19	182	GTACAGTACTGTGATAACT	miRNA	miR-101
t0001833	22	182	TGAGGTACTAGGTTGTGTGGTT	miRNA	let-7b
t0001832	21	182	TGAGGCAGTAGGTTGTATAGT	miRNA	let-7
t0001845	21	181	TGAGGGGCAGAGAGCCAGACT	miRNA	miR-423-5p
t0001843	24	181	CTTTGAAGACTGAAGTGGAGAAGG	miRNA	miR-3526
t0001839	21	181	CATTGTACTTGTCTCGGTCTG	miRNA	miR-25
t0001848	22	181	CAACGGAATCTCAAAAGCAGCT	miRNA	miR-191
t0001846	22	181	TCTCCCAACCCTTGTACCAGTA	miRNA	miR-150
t0001844	20	181	TCAGTGCATCACAGAACTTT	miRNA	miR-148b-3p
t0001847	21	181	TGAGGTAGTATATTGTATAGT	miRNA	let-7f
t0001863	21	180	TAGCACCATCTGAAATCGGTT	miRNA	miR-29c
t0001865	24	180	TGGAGAGAAAGGCAGTTCCTGAAT	miRNA	miR-185
t0001852	22	180	TGGAGAGAGAGGCAGTTCCTGA	miRNA	miR-185
t0001849	20	180	TAGCAGTACATCATGGTTTA	miRNA	miR-15b
t0001857	23	180	TACCACATGGTAGAACCACGGAC	miRNA	miR-140
t0001862	22	180	TGGAATGTAAAGAAGTATGGAG	miRNA	miR-1-3p
t0001872	22	180	CTTCCCCACCCTCTCCTGCAGA	miRNA	miR-1224-3p
t0001870	19	179	TATTGCACTCGTCCCGGCC	miRNA	miR-92
t0001868	21	179	AAACCGCTACCATTACTGAGT	miRNA	miR-451
t0001869	24	179	TCTCACACAGAAATCGCACCCGTT	miRNA	miR-342-3p
t0001874	22	179	TGAGGTAGTAGTTTTTACAGTT	miRNA	let-7g
t0001877	20	179	TGAGGTAGTAGGTTGTATTT	miRNA	let-7
t0001873	21	178	CACCTTGCGCTACTCAGGTCT	miRNA	miR-3200-3p
t0001888	22	178	TGAGGTAGTAGGTTGTGTGCTT	miRNA	let-7b
t0001883	21	177	TCCTGTACTGAGCTGCTTCGA	miRNA	miR-486-5p
t0001884	24	177	TGTAAACATCCTTGACTGGAAGCT	miRNA	miR-30e
t0001887	19	177	AAGCTGCCAGTTGAAGAAC	miRNA	miR-22-3p
t0001885	22	177	TGAGGTAGTAGTTTGTGTTGTT	miRNA	let-7i
t0001881	22	177	TGAGGTAGTAGGTTGTTTAGTT	miRNA	let-7
t0001882	24	176	AAAGATGCCACGCTATGTAGAATC	miRNA	miR-741-3p
t0001894	23	176	GCAAAGCACACGGCCTGCAGAGA	miRNA	miR-330-3p
t0001896	21	176	TGTGCAAATCCATGCAAAACT	miRNA	miR-19b
t0001889	23	176	AGCAGCATTGTACATGGCTATGA	miRNA	miR-103a
t0001892	21	176	TACAGTATTGTGATAACTGAA	miRNA	miR-101
t0001895	20	176	TGAGGTAGTAGGTTGTGTAG	miRNA	let-7b
t0001898	21	175	AAACCGTTACCATTACTGGGT	miRNA	miR-451
t0001901	22	175	AACCGTTACCATTACTGAGTTT	miRNA	miR-451
t0001906	22	175	TGGCAGCACGTAAATATTGGCG	miRNA	miR-16
t0001908	22	175	TGAGGTAGTAGGTTCTATAGTT	miRNA	let-7
t0001907	26	174	AAACCTTTACCATTACTGAGTTTAGT	miRNA	miR-451
t0001910	21	174	AAACCGTTACCATTAGTGAGT	miRNA	miR-451
t0001909	23	174	TGAGCGGCAGAGAGCGAGACTTT	miRNA	miR-423-5p
t0001915	22	174	AAAAGCTGTGTTGAGAGGGCGA	miRNA	miR-320a
t0001914	22	174	TAGCAGCACGTAAACATTGGCG	miRNA	miR-16
t0001918	22	174	TACAGTATAGATGATGTACTTT	miRNA	miR-144
t0001912	20	174	TGAGGTAGCAGATTGTATAG	miRNA	let-7f
t0001916	22	173	TCCTGTACTGAGCTGACCCGAG	miRNA	miR-486-5p
t0001917	22	173	CTGACCTATGAATTGACAGCCG	miRNA	miR-192
t0001926	21	173	ACCACAAGGTAGAACCACGGA	miRNA	miR-140
t0001928	20	173	TGAGGTAGTGGATTGTATAG	miRNA	let-7f-5p
t0001922	21	173	TGAGGTAGTAGATGGTATAGT	miRNA	let-7f
t0001923	22	173	TGCGGTAGTAGGTTGTGTGGTT	miRNA	let-7b
t0001930	23	172	TCCTGTACTGAGCTGCTTCGAGA	miRNA	miR-486-5p

t0001934	21	172	AGGGGCTGGCTTTCCTCTGGT	miRNA	miR-185*
t0001933	20	171	TTCGGGGTCTGGGCGCGGCG	miRNA	miR-4508
t0001937	20	171	AACATTCATTGCTGTCGGTG	miRNA	miR-181b
t0001935	22	171	TAGCAGCACGTAAGTATTGGCG	miRNA	miR-16
t0001931	21	171	TCGAGGAGCTCACAGTCTAGT	miRNA	miR-151-5p
t0001932	23	171	AGCAGTATTGTACAGGGCTATGA	miRNA	miR-103a
t0001948	19	170	AAACCGTTACCATTACAGT	miRNA	miR-451
t0001945	20	170	ATCACATTGCCAGGGATTAC	miRNA	miR-23b
t0001942	23	170	TGGAGAGAAAGGCAGTTTCTGAT	miRNA	miR-185
t0001946	21	170	TGAGTTAGTAGATTGTATAGT	miRNA	let-7f
t0001941	24	170	TGAGGTAGTAGGTTGTGTGGTAAA	miRNA	let-7b
t0001944	21	169	AAACCGTTACTATTACTGAGT	miRNA	miR-451
t0001953	22	169	TAGCAGCACGTAACTATTGGCG	miRNA	miR-16
t0001950	22	169	TAGCAGCACGTAGATATTGGCG	miRNA	miR-16
t0001955	19	169	AGGTAGTAGATTGTATAGT	miRNA	let-7f
t0001961	22	169	TGAGGTAGGAGGTTGTGTGGTT	miRNA	let-7b
t0001960	21	169	TGAGGTAGTAGGTTGTGTCGT	miRNA	let-7b
t0001959	22	169	TGAGGTAGTGGGTTGTATAGTT	miRNA	let-7
t0001956	21	168	TACCCATTGCATATCGGAGTT	miRNA	miR-660
t0001967	21	168	TGAGGGGCAGAGCGCGAGACT	miRNA	miR-423-5p
t0001973	23	168	GGCTGGTCCGAGTGCAGTGGTGT	miRNA	miR-3135b
t0001970	21	168	CTGACCTATGAATTGACAGCT	miRNA	miR-192
t0001972	23	168	TGGAGAGAAAGGCATTTCCTGAA	miRNA	miR-185
t0001975	20	168	AGGTAGTAGATTGTATAGTT	miRNA	let-7f
t0001976	23	167	AGGGACGGGACGCGGTGCAGTGT	miRNA	miR-92b*
t0001978	19	167	AAACCGTTACCATTACTAG	miRNA	miR-451
t0001979	23	167	AATTGCACGGTATCCATCTGTAA	miRNA	miR-363
t0001981	24	167	TTTGAAGACTGAAGTGGAGAAGGG	miRNA	miR-3526
t0001983	26	167	TGAGATGAAGCACTGTAGCTCTAATC	miRNA	miR-143
t0001987	19	167	TAAAGTGCTGACAGTGCAG	miRNA	miR-106b
t0001985	21	166	TGTAAACATCCCCGACTGGAA	miRNA	miR-30d
t0001995	22	166	CAACGGAATCCTAAAAGCAGCT	miRNA	miR-191
t0001988	22	166	TGGAGAGAAAGGCAGGTCCTGA	miRNA	miR-185
t0001989	21	166	TAGCAGCACATCATGGTTTAT	miRNA	miR-15b
t0001993	21	166	TGAGGTGGTAGTTTGTGCTGT	miRNA	let-7i
t0001991	20	165	TAAGGGCTGGGTCGGTCGGG	miRNA	miR-4651
t0001990	23	165	TGAGAGGCAGAGAGCGAGACTTT	miRNA	miR-423-5p
t0002005	23	165	TACCACAGGGTAGAATTACGGAC	miRNA	miR-140
t0001998	20	165	TGAGGTAGTAGATTGAATAG	miRNA	let-7k
t0002001	24	164	AATGACACGATCACTCCCGTTGAG	miRNA	miR-425
t0002000	21	164	TTCCTATGCATATACTTCTTT	miRNA	miR-202-5p
t0002004	25	164	CAACGGAATCCCAAAAGCAGCTGTT	miRNA	miR-191
t0002003	24	164	AACATTCAACGCTGTCGGTGAATC	miRNA	miR-181a
t0002012	22	164	TGGAGTGTGACAATGGTGTTTG	miRNA	miR-122
t0002013	22	163	ATTGCACTTGTCCCGGCCTGTA	miRNA	miR-92b-3p
t0002011	22	163	AAACCGTTACCATTACCGAGTT	miRNA	miR-451
t0002015	22	163	TGAGGGGCAGAGACCGAGACTT	miRNA	miR-423-5p
t0002009	21	163	CGAGGGGCAGAGAGCGAGACT	miRNA	miR-423-5p
t0002023	23	163	AAAAGCTGGGTTGAGAGGGAGAA	miRNA	miR-320d
t0002025	22	163	TGAAGAGAAAGGCAGTTCCTGA	miRNA	miR-185
t0002019	23	163	AACATTCAACGCTGTCGGTGAGT	miRNA	miR-181a
t0002024	23	163	TGAGAACTGAATTCCATAGGCTG	miRNA	miR-146b
t0002032	22	163	TATCACAGGGTAGAACCACGGA	miRNA	miR-140
t0002027	21	163	GTGAGGACTCGGGAGGTGGAA	miRNA	miR-1224
t0002030	22	162	AAACCGCTACCATTACTGAGTT	miRNA	miR-451
t0002029	22	162	TGGAGAGAAACGCAGTTCCTGA	miRNA	miR-185
t0002031	22	162	TATGGCACTGGTAGAATTCACT	miRNA	miR-183
t0002041	20	162	AGCAGCATTGTATAGGGCTA	miRNA	miR-103a

t0002048	21	162	TGAGGTAGTCGATTGTATAGT	miRNA	let-7f
t0002042	22	161	AACGGAATCTCAAAAGCAGCTG	miRNA	miR-191
t0002045	23	161	TGGAGAGAAAGGCAGTTCCTGTT	miRNA	miR-185
t0002044	22	161	TAGCAGCACGTAAATATTTTCG	miRNA	miR-16
t0002047	22	161	ACCACAGGGTATAACCACGGAC	miRNA	miR-140
t0002049	23	160	TGAGGGGCAGTGAGCGAGACTTT	miRNA	miR-423-5p
t0002038	22	160	TGGAGACAAAGGCAGTTCCTGA	miRNA	miR-185
t0002046	20	160	TAGCAGCACACAATGGTTTG	miRNA	miR-15a
t0002039	22	160	TACCACAGGGTAGAACCACGGG	miRNA	miR-140
t0002053	21	160	TGAGCTAGTAGATTGTATAGT	miRNA	let-7f
t0002060	21	160	TGAGGTAGTAGGCTGTATAGT	miRNA	let-7
t0002055	23	159	AAACTGTTACCATTACTGAGTTT	miRNA	miR-451
t0002054	20	159	TAGCAGCACATAATGGTTTT	miRNA	miR-15a
t0002059	25	159	CTAGACTGAAGCTCCTTGAGGAAGA	miRNA	miR-151-3p
t0002066	22	159	AGCAGCATTGTACAGCGCTATG	miRNA	miR-103a
t0002067	23	159	TACAGTACTGTGATAACTGAAAA	miRNA	miR-101
t0002065	21	159	ATGAGGTAGTAGATTGTATAG	miRNA	let-7f
t0002062	22	158	TAGAGGAGATGGCGCAGGGGAA	miRNA	miR-877
t0002074	21	158	CATTATTACTTTTGGTACGCG	miRNA	miR-126-5p
t0002075	24	158	TGAGGTAGTAGGTTGTGTGGTTGT	miRNA	let-7b
t0002072	20	157	AAACCGTCACCATTACTGAG	miRNA	miR-451
t0002070	20	157	AAAAGCTGGGTTGAGAGGGC	miRNA	miR-320a
t0002068	22	157	TTCAAGTAATCCAGGATAGGCG	miRNA	miR-26a
t0002069	22	157	TGGAGAGAAAGGCCGTTCCTGA	miRNA	miR-185
t0002086	21	157	AAGGTAGATAGAACAGGTCTT	miRNA	miR-1839
t0002083	21	156	AATTGCACGGTATCCATCTGT	miRNA	miR-363
t0002077	24	156	AAAAGCTGGGTTGAGAGGGCGTTG	miRNA	miR-320d
t0002085	25	156	TTGGGGAAACGGCCGCTGAGTGAGA	miRNA	miR-2110
t0002080	24	156	TGGAGAGAAAGGCAGTTCCTGATA	miRNA	miR-185
t0002084	25	156	AGCAGCATTGTACAGGGCTATGAAA	miRNA	miR-103a
t0002087	22	156	TGAGGTAGTAGGTTGTGTGGCT	miRNA	let-7b
t0002090	23	155	TCCTGTACTGAGCTCCCCCGAGT	miRNA	miR-486-5p
t0002092	23	155	TGAGGGGCAGGGAGCGAGACTTT	miRNA	miR-423-5p
t0002096	24	155	AAAAGCTGGGTTGAGAGGGCGTTA	miRNA	miR-320d
t0002099	22	155	TAACGGAATCCCAAAAGCAGCT	miRNA	miR-191
t0002093	21	155	ACCACAGGTTAGAACCACGGA	miRNA	miR-140
t0002095	20	155	TGAGGTAGTACATTGTATAG	miRNA	let-7f
t0002094	22	155	TGAGGTAGTAGATGGTATAGTT	miRNA	let-7f
t0002105	20	155	TGAGGTAGTAGATTGTACAG	miRNA	let-7f
t0002113	22	154	AAACCGTTACCTTTACTGAGTT	miRNA	miR-451
t0002107	22	154	TGAGGGGAAGAGAGCGAGACTT	miRNA	miR-423-5p
t0002111	20	154	ATCACATTGCCAGGGATTAA	miRNA	miR-23b
t0002103	22	154	TGGAGAGTAAGGCAGTTCCTGA	miRNA	miR-185
t0002108	21	154	ACTGCAGTGAAGGCACTTGTA	miRNA	miR-17*
t0002126	19	154	TGAGGTAGTAGGTTGTGTT	miRNA	let-7b
t0002121	21	154	TGAGGTAGTAGGTTGCGTGGT	miRNA	let-7b
t0002124	22	153	TCCTGTACTGAGCTGCACCGAG	miRNA	miR-486-5p
t0002114	19	152	AAACCGTTACCATTACTGT	miRNA	miR-451
t0002116	23	152	TGAGGGCCAGAGAGCGAGACTTT	miRNA	miR-423-5p
t0002119	27	152	ACTTTGAAGACTGAAGTGGAGAAGGGT	miRNA	miR-3526
t0002123	23	152	AAAAGCTGGGTTGAGATGGCGAA	miRNA	miR-320a
t0002125	20	152	TGGAGAGAAAGGCAGTTGAT	miRNA	miR-185
t0002127	20	152	TCACAGTGAACCGGTCTCTT	miRNA	miR-128
t0002128	22	152	TGAGGTGGTAGTTTGTACAGTT	miRNA	let-7g
t0002133	20	152	TGGTAGTAGATTGTATAGTT	miRNA	let-7f
t0002134	22	152	TGAGGTAGTAGGTTGTGTGGAG	miRNA	let-7b
t0002141	22	152	TGGGGTAGTAGGTTGTATAGTT	miRNA	let-7
t0002131	21	152	TGAGGTAGTAGGTTGTACAGT	miRNA	let-7

t0002137	23	151	TCCTGTACTGAGCTGCCCCGCGA	miRNA	miR-486-5p
t0002146	24	151	TGGTAGACTATGGAACGTAGGATC	miRNA	miR-379-5p
t0002154	25	151	AAATAGCAGCACGTAAATATTGGCG	miRNA	miR-16
t0002161	22	151	ACCACAGGGTAGAACTACGGAC	miRNA	miR-140
t0002162	21	151	TGAGGTAGTAGATTTTATAGT	miRNA	let-7f
t0002150	25	151	TGAGGTAGTAGGTTGTGTGGTTAAG	miRNA	let-7b
t0002158	22	151	CGAGGTAGTAGGTTGTATAGTT	miRNA	let-7
t0002151	23	150	TGAGGGGCAGAGAGCGAGACTAG	miRNA	miR-423-5p
t0002163	23	150	TGAGGGGCAGAGAGCGAGACTAT	miRNA	miR-423-5p
t0002152	27	150	TGGTAACTCCGAACCACCGTTGGCATC	miRNA	miR-2765
t0002165	23	150	CTAGCAGCACGTAAATATTGGCG	miRNA	miR-16
t0002155	22	150	TAGCAGCACGTAATTATTGGCG	miRNA	miR-16
t0002166	20	150	TAGCAGCACATAATGGTTCG	miRNA	miR-15a
t0002172	25	150	TGAGGTAGTAGGTTGTGTGGTTAGT	miRNA	let-7b
t0002180	21	150	CGAGGTAGTAGGTTGTATAGT	miRNA	let-7
t0002173	21	149	TGAGGGGCAGACAGCGAGACT	miRNA	miR-423-5p
t0002184	23	149	TGAGGGACAGAGAGCGAGACTTT	miRNA	miR-423-5p
t0002178	21	149	ACCTGGCATACAATGTAGATT	miRNA	miR-221*
t0002179	22	149	TGGAGAGAAAGGCAGATCCTGA	miRNA	miR-185
t0002169	22	149	AGAAGCATTGTACAGGGCTATG	miRNA	miR-103a
t0002191	23	149	TGAGGTAGTAGGTTGTATGGTTA	miRNA	let-7c
t0002192	21	148	TGAGGTGCAGAGAGCGAGACT	miRNA	miR-423-5p
t0002200	23	148	CAACGGAATCCCAAAAGCAGCTA	miRNA	miR-191
t0002187	23	148	CAGTGCAATGTTAAAAGGGCATA	miRNA	miR-130a
t0002189	20	148	GTACAGTACTGTGATAACTG	miRNA	miR-101
t0002196	23	148	CTGAGGTAGTAGTTTGTACAGTT	miRNA	let-7g
t0002194	23	147	TGAGGGGCATAGAGCGAGACTTT	miRNA	miR-423-5p
t0002198	19	147	AACTTTGAAGACTGAAGTG	miRNA	miR-3526
t0002205	20	147	CCCATAAAGTAGAAAGCATT	miRNA	miR-142
t0002209	20	146	CCTCCCACACCCAAGGCTTG	miRNA	miR-532-3p
t0002211	22	146	TGGAGAGAAAGGCAGTGTTTGA	miRNA	miR-4306
t0002201	23	146	TGAGGGGTAGAGAGCGAGACTTT	miRNA	miR-423-5p
t0002204	21	146	TGAAGGGCAGAGAGCGAGACT	miRNA	miR-423-5p
t0002202	24	146	AACGGAATCCCAAAAGCAGCTGAA	miRNA	miR-191
t0002218	22	146	CTGGAGATATGGAAGAGCTGTG	miRNA	miR-1270
t0002229	22	146	TGAGGTAGTAGGTTTTGTGGTT	miRNA	let-7b
t0002223	22	145	TGAGTGGCAGAGAGCGAGACTT	miRNA	miR-423-5p
t0002224	23	145	TTCAAGTAATCCAGGATAGGCAA	miRNA	miR-26a
t0002219	22	145	AGCTACATCTGGCTACTGGGTC	miRNA	miR-222
t0002238	23	145	TACCACAGGGTAGAACCACGGCT	miRNA	miR-140
t0002235	22	145	AGCAGCATTGTACAGTGCTATG	miRNA	miR-103a
t0002236	23	144	TGAGGGGCAGAGAGCGACACTTT	miRNA	miR-423-5p
t0002244	23	144	TGGAGAGAAAGGCAGTTCCTGCT	miRNA	miR-185
t0002243	21	144	TAGCAGCACATAATGGTTTGA	miRNA	miR-15a
t0002239	22	144	TGAGGTAGTAGATTGTATAGAA	miRNA	let-7f-5p
t0002246	21	143	TTAAGACTTGCAGTGATGTTT	miRNA	miR-499-5p
t0002245	22	143	AAACCGTTACCATTACTGCGTT	miRNA	miR-451
t0002248	24	143	AAAAGCTGGGTTGAGAGGGCGTAG	miRNA	miR-320d
t0002249	22	143	TACCACAGGGTAGAACCATGGA	miRNA	miR-140
t0002257	22	143	ACTCGGCGTGGCGTCGGTCGTG	miRNA	miR-1307
t0002259	22	143	TTTGAGGCTACAGTGAGATGTG	miRNA	miR-1304
t0002254	22	143	TGAGGTAGTAGTTTGTGCAGTT	miRNA	let-7g
t0002263	21	143	GTTGAGGTAGTAGATTGTATA	miRNA	let-7f
t0002255	21	143	TGAGGTAGTGGGTTGTGTGGT	miRNA	let-7b
t0002269	21	142	TATTGCACTTGTCCCGGCCAA	miRNA	miR-92a
t0002274	22	142	TCCTGTACTGAGCTGCTTCGAG	miRNA	miR-486-5p
t0002266	23	142	TGAAGGGCAGAGAGCGAGACTTT	miRNA	miR-423-5p
t0002273	22	142	TGTGACTTTAAGGGAAATGGCG	miRNA	miR-3164

t0002285	22	142	TGGAGCGAAAGGCAGTTCCTGA	miRNA	miR-185
t0002289	21	142	TGGAGAGAAAGGCAGTTCATG	miRNA	miR-185
t0002281	22	142	TGAGGTAGCAGGTTGTATAGTT	miRNA	let-7
t0002284	20	141	GAAGACTGAAGTGGAGAAGG	miRNA	miR-739
t0002278	21	141	TAAGGTGCATCTAGTGCAGAT	miRNA	miR-18a
t0002275	22	141	TGTAGAGAAAGGCAGTTCCTGA	miRNA	miR-185
t0002280	26	141	TGAGGTAGTAGTTTGTGCTGTTAATC	miRNA	let-7i
t0002292	20	141	TGAGGTAGTAGATTGTATCG	miRNA	let-7f
t0002286	22	141	TGTGGTAGTAGGTTGTGTGGTT	miRNA	let-7b
t0002307	21	141	TGGGGTAGTAGGTTGTATAGT	miRNA	let-7
t0002297	19	140	CTCTTTTCTTTGAGACTCA	miRNA	miR-627
t0002299	22	140	ATCTGTACTGAGCTGCCCCGAG	miRNA	miR-486-5p
t0002295	22	140	TAGCAGTACATCATGGTTTACA	miRNA	miR-15b
t0002296	22	140	AGGCATTGACTTCTCACTAGCT	miRNA	miR-1256
t0002303	22	140	TACAGTACTGTGATAACTGATA	miRNA	miR-101c
t0002300	21	140	TGAGGTAGTAAGTTGTGTGGT	miRNA	let-7b
t0002312	25	139	TCTTTGGTTATCTAGCTGTATGATC	miRNA	miR-9a
t0002325	21	139	AATGACACGATCACTCCCGTT	miRNA	miR-425
t0002323	23	139	AGCTACATCTGGCTACTGGGTCT	miRNA	miR-222
t0002313	22	139	TAGGAGCACGTAAATATTGGCG	miRNA	miR-16
t0002320	23	139	TACCACAGGGTAGAACTACGGAT	miRNA	miR-140
t0002314	24	139	TACCACAGGGTAGAACAACGGACA	miRNA	miR-140
t0002315	23	139	TACCACAGGGTAGAACAACGGAA	miRNA	miR-140
t0002338	18	139	TGAGGTAGTAGTTTGTGC	miRNA	let-7i
t0002327	22	139	TGAGGGAGTAGTTTGTGCTGTT	miRNA	let-7i
t0002331	21	139	TGTGGTAGTAGATTGTATAGT	miRNA	let-7f
t0002333	22	138	CATTTCACTTGTCTCGGTCTGA	miRNA	miR-25
t0002341	21	138	TGAGGTAGTAGGTTGTTTAGT	miRNA	let-7
t0002330	21	137	TTTGGCACTAGCACATTTTTG	miRNA	miR-96
t0002360	23	137	TCCTGTACTGAGCTGCCTCGAGA	miRNA	miR-486-5p
t0002343	24	137	GAAAAGCTGGGTTGAGAGGGCGAT	miRNA	miR-320a
t0002351	24	137	AACATTCAACGCTGTCGGTGAGTT	miRNA	miR-181a
t0002366	23	137	TACCACAGGGTAGAAACACGGAA	miRNA	miR-140
t0002348	21	136	AAACCGTTACCATTACTGAGG	miRNA	miR-451
t0002362	20	136	CAAAACGTGAGGCGCTGCTA	miRNA	miR-424*
t0002369	23	136	TGGGGGGCAGAGAGCGAGACTTT	miRNA	miR-423-5p
t0002355	21	136	CCAAAGAATTCTCCTTTTGGG	miRNA	miR-186
t0002357	21	136	TGGAGAGAAAGGCAGTTCCGA	miRNA	miR-185
t0002364	22	136	ACCACAGGGTAGAACCACGGCA	miRNA	miR-140
t0002372	21	135	GGGAGCCAGGAAGTATTGATG	miRNA	miR-505*
t0002371	22	135	TCTCACACAGAAATCGCATCCG	miRNA	miR-342-3p
t0002381	20	135	CACCTTGCGCTACTCAGGTC	miRNA	miR-3200-3p
t0002389	19	135	TGACCTATGAATTGACAGC	miRNA	miR-192
t0002387	20	135	TATCAGCACATCATGGTTTA	miRNA	miR-15b
t0002383	21	135	AGCAGCATTGTACAGTGCTAT	miRNA	miR-103a
t0002377	21	135	TGAGGTAGTAGATTGTATAAT	miRNA	let-7f
t0002403	20	135	TGAGGTAGTAGCTTGTATAG	miRNA	let-7f
t0002392	22	134	GGCTGGTCCGAGTGCAGTGGTG	miRNA	miR-3135b
t0002404	22	134	AGACCTACTTATCTACCAACAG	miRNA	miR-1839-3p
t0002399	21	134	TGGAATGTAAAGAAGTATGGA	miRNA	miR-1-3p
t0002400	21	134	TGAGGTAGTAGTTTGTGCTTA	miRNA	let-7i
t0002405	21	134	GAGGTAGTAGGTTGCATAGTT	miRNA	let-7d
t0002413	18	133	TGAGGTAGATTGTATAGT	miRNA	miR-1961
t0002425	24	133	AACATTCATTGTTGTCGGTGGATC	miRNA	miR-181d-5p
t0002424	27	133	TGAGAACTGAATTCCATAGGCTGTATC	miRNA	miR-146b
t0002417	24	133	CTTTTTGCGGTCTGGGCTTGCATC	miRNA	miR-129
t0002415	21	133	TGAAGTAGTAGATTGTATAGT	miRNA	let-7f
t0002416	20	133	GAGGTAGTAGGTTGTGTGGT	miRNA	let-7b

t0002412	21	133	TGAGGTAGTAGGTTGTCTGGT	miRNA	let-7b
t0002420	22	133	TGAGCTAGTAGGTTGTGTGGTT	miRNA	let-7b
t0002435	20	133	TGAGGTAGTAGGTTGTATAA	miRNA	let-7
t0002440	22	132	TATTGCACTCGTCCCGGCCTCA	miRNA	miR-92
t0002437	22	132	CATTGCACTTGTCTCGGTTTGA	miRNA	miR-25
t0002441	21	132	ACCACAGGGCAGAACCACGGA	miRNA	miR-140
t0002434	20	132	TGAGGTATTAGATTGTATAG	miRNA	let-7f
t0002458	24	131	TCTGGGCACAGGCGGATGGACAGT	miRNA	miR-5107
t0002453	25	131	CTTTGAAGACTGAAGTGGAGAAGGG	miRNA	miR-3526
t0002456	21	131	TGGGTTCCTGGCATGCTGATT	miRNA	miR-23b*
t0002448	27	131	GACTGGGGCGGTACATCTGTTAAATAT	miRNA	miR-219-2-3p
t0002446	24	131	TACCACAGGGTAGAACCAAGGACA	miRNA	miR-140
t0002451	21	131	TGAGGTAGTAGATTGTAGAGT	miRNA	let-7f
t0002457	22	131	AGAGGGAGTAGGTTGCATAGTT	miRNA	let-7d
t0002460	22	131	TGAGGTAGTAGGTTGTGTTGTT	miRNA	let-7b
t0002459	22	130	TGTAGAGCAGGGAGCAGGAAGT	miRNA	miR-4732-5p
t0002465	23	130	CATTGCACTTGTCTCGGTCTGTA	miRNA	miR-25
t0002466	20	130	ATTGCACTTGTCTCGGTCTG	miRNA	miR-25
t0002469	22	130	TGGCGAGAAAGGCAGTTCCTGA	miRNA	miR-185
t0002468	23	130	AGAAGCATTGTACAGGGCTATGA	miRNA	miR-103a
t0002476	22	129	TACGTCATCGTTGTTATCGTCA	miRNA	miR-598
t0002485	22	129	TCCTGTACTGAGCTGCCCAGAG	miRNA	miR-486-5p
t0002480	23	129	AAACCGTTACCATTATCGAGTTT	miRNA	miR-451
t0002482	24	129	CTGAGGGGCAGAGAGCGAGACTTT	miRNA	miR-423-5p
t0002483	23	129	TGAGGGGCAGAGAGCGAGACTTC	miRNA	miR-423-5p
t0002477	23	129	CTAGACTGAAGCTCCTTGAGGAA	miRNA	miR-151-3p
t0002473	22	129	ACCACAGGGTAGAACAACGGAC	miRNA	miR-140
t0002479	22	129	TGAGGTAGTAGGCTGTATAGTT	miRNA	let-7
t0002497	20	129	TGAGGTAGTAGGGTGTATAG	miRNA	let-7
t0002495	21	128	CCTCCCACACCCAAGGCTTGC	miRNA	miR-532-3p
t0002498	24	128	AAAACCGTTACCATTACTGAGTTA	miRNA	miR-451
t0002488	25	128	TGTAAACATCCCCGACTGGAAGATC	miRNA	miR-30d
t0002492	22	128	TAGCAGCACGTTAATATTGGCG	miRNA	miR-16
t0002507	20	128	CTATACAACCTACTGCCTTC	miRNA	let-7b*
t0002515	21	128	TGAGGTAGGAGGTTGTATAGT	miRNA	let-7
t0002522	22	127	AAACCGTTACCATTATCGAGTT	miRNA	miR-451
t0002508	22	127	AAACTTTACCATTACTGAGTTT	miRNA	miR-451
t0002518	22	127	TGAGGGGCAGAGAGCGGGACTT	miRNA	miR-423-5p
t0002510	22	127	AAGCGGCGATGGCGGAGCTGAA	miRNA	miR-1636
t0002519	21	127	ACCACAGGCTAGAACCACGGA	miRNA	miR-140
t0002513	23	127	AGCAGCATTGTACAGGGTTATGA	miRNA	miR-103a
t0002517	21	127	CTATACAACCTACTGCCTTCT	miRNA	let-7b*
t0002536	20	126	TATTGCACTTGTCCCGGCCA	miRNA	miR-92a
t0002532	25	126	TGAGGGGCAGAGAGCGAGACTTTTT	miRNA	miR-423-5p
t0002526	22	126	CATTGCACTTGTTTCGGTCTGA	miRNA	miR-25
t0002523	20	126	TAGCAGCACATAATGGTTTA	miRNA	miR-15b
t0002528	22	126	TACCACAGGGTAGAACCACAGA	miRNA	miR-140
t0002527	21	126	GAGGTAGTAGTTTGTACAGTT	miRNA	let-7g
t0002541	19	126	TGTAGTAGGTTGTGTGGTT	miRNA	let-7b
t0002543	21	126	TGAGGTAGTGGGTTGTATAGT	miRNA	let-7
t0002545	22	125	TATTGCACTCGTCCCGGCCTCT	miRNA	miR-92
t0002552	20	125	TGAGGGGCAGAGAGCGAGAT	miRNA	miR-423-5p
t0002550	23	125	TGAGGGGCAGAGTGCGAGACTTT	miRNA	miR-423-5p
t0002551	22	125	ACATTCATTGCTGTCGGTGGGT	miRNA	miR-181b
t0002549	21	125	TAGCAGCACGTAAATATTGCC	miRNA	miR-16
t0002570	23	125	TACCACAGTGTAGAACCACGGAA	miRNA	miR-140
t0002558	22	125	TGAGGTAGTAGATAGTATAGTT	miRNA	let-7f
t0002566	22	125	TGAGGTAGTAGGTTATGTGGTT	miRNA	let-7b

t0002567	21	125	TGACGTAGTAGGTTGTGTGGT	miRNA	let-7b
t0002561	21	125	TGAGGTAGTAGGTTGCATAGT	miRNA	let-7
t0002563	22	124	TTCTCTGTTTTGGCCATGTGTG	miRNA	miR-942
t0002572	21	124	GAGGGGCAGAGAGCGAGACTT	miRNA	miR-423-5p
t0002559	24	124	ACTGGACTTGGAGTCAGAAGGCAA	miRNA	miR-378
t0002594	23	124	AAAAGCTGGGTTGAGAGGGCAAG	miRNA	miR-320b
t0002574	23	124	ATCACATTGCCAGGGATTTCCAA	miRNA	miR-23a
t0002588	23	124	TACCACAGGGGAGAACCACGGAC	miRNA	miR-140
t0002582	22	124	TGAGGTAGTTGGTTGTGTGGTT	miRNA	let-7b
t0002590	23	123	AAAACCGTTACCATTACTGAGTT	miRNA	miR-451
t0002587	21	123	AGAAGCATTGTACAGGGCTAT	miRNA	miR-4289
t0002583	22	123	TTCAAGTAATTCAGGATAGGCT	miRNA	miR-26a
t0002592	24	123	TGGAGAGAAAGGCAGTTCCTGAGT	miRNA	miR-185
t0002593	18	123	CTACAGTATAGATGATGT	miRNA	miR-144
t0002591	22	123	TACCACAAGGTAGAACCACGGA	miRNA	miR-140
t0002600	22	123	TACCACAGGGTAGAACCTCGGA	miRNA	miR-140
t0002609	22	123	TGAGGTAGTAGGATGTGTGGTT	miRNA	let-7b
t0002602	21	123	TAAGGTAGTAGGTTGTATAGT	miRNA	let-7
t0002610	24	122	AAAAGCTGGGTTGAGAGGGCGAGT	miRNA	miR-320a
t0002597	22	122	TACCACAGGGCAGAACCACGGA	miRNA	miR-140
t0002611	25	122	ACCACAGGGTAGAACCACGGACAGA	miRNA	miR-140
t0002607	19	122	GTGAGGACTCGGGAGGTGG	miRNA	miR-1224
t0002634	21	122	AAGCAGCATTGTACAGGGCTA	miRNA	miR-103a
t0002623	21	122	TGAGGTAGTTGATTGTATAGT	miRNA	let-7f
t0002636	23	121	TATTGCACTTGTCCCGGCCTGAA	miRNA	miR-92b-3p
t0002620	22	121	TCACACAGAAATCGCACCCGTC	miRNA	miR-342-3p
t0002628	24	121	GAAAAGCTGGGTTGAGAGGGCGTT	miRNA	miR-320d
t0002612	25	121	AAAAGCTGGGTTGAGAGGGCGAAGT	miRNA	miR-320a
t0002629	21	121	CATTGCATTTGTCTCGGTCTG	miRNA	miR-25
t0002626	22	121	TAGCAGCACGTAAATACTGGCG	miRNA	miR-16
t0002613	22	121	TAGCACCACGTAAATATTGGCG	miRNA	miR-16
t0002648	23	121	TCTCCCAACCCTTGTACCAGTGT	miRNA	miR-150
t0002641	22	121	TGAGGTAGTAGTTTGTAAAGTT	miRNA	let-7g
t0002642	20	121	TGAGGTAGTAGACTGTATAG	miRNA	let-7f
t0002646	22	121	CTGAGGTAGTAGGTTGTATAGT	miRNA	let-7
t0002650	22	120	TATTGCACTTGTCCCGGCTTGT	miRNA	miR-92a
t0002651	21	120	TAGAGGAGATGGCGCAGGGGA	miRNA	miR-877
t0002668	23	120	TAAGGGGCAGAGAGCGAGACTTT	miRNA	miR-423-5p
t0002664	22	120	TGAGGGGCAGAGGGCGAGACTT	miRNA	miR-423-5p
t0002665	21	120	TGAGGGGCAGAGAGCGAGTCT	miRNA	miR-423-5p
t0002657	22	120	TGAGGGGCAGAGAGCCAGACTT	miRNA	miR-423-5p
t0002662	25	120	ACTGGACTTGGAGTCAGAAGGAATC	miRNA	miR-378c
t0002654	23	120	TGGAGAGAAAGGTAGTTCCTGAA	miRNA	miR-185
t0002658	22	120	TAGCAGCACGTACATATTGGCG	miRNA	miR-16
t0002670	24	120	ATAGCAGCACGTAAATATTGGCGA	miRNA	miR-16
t0002656	22	120	ACCACAGGGTAGAACCACGGTA	miRNA	miR-140
t0002672	22	120	TACAACAGGGTAGAACCACGGA	miRNA	miR-140
t0002675	21	120	TTAGGTAGTAGGTTGTGTGGT	miRNA	let-7b
t0002677	21	120	TGAGGTAGTATGTTGTGTGGT	miRNA	let-7b
t0002691	21	120	TGAGCTAGTAGGTTGTGTGGT	miRNA	let-7b
t0002682	21	120	TGAGGTAGTAGGTTGTATATT	miRNA	let-7
t0002685	22	119	CGGGGCAGCTCAGTACAGGATA	miRNA	miR-486-3p
t0002695	25	119	TGTAGAGCAGGGAGCAGGAAGCTGA	miRNA	miR-4732-5p
t0002700	21	119	AAAAGCTGGGTTGAGAGGCGA	miRNA	miR-320d
t0002693	21	119	ACAGTAGTCTGCACATTGGTT	miRNA	miR-199
t0002690	22	119	TGGAGAGAAAGGCAGTTCCTCA	miRNA	miR-185
t0002689	19	119	TAGCAGTACATCATGGTTT	miRNA	miR-15b
t0002699	22	119	TCAGTGCACTACAGAACTTTGT	miRNA	miR-148a

t0002680	20	119	AGAGGTAGTAGGTTGCATTT	miRNA	let-7d
t0002694	21	118	TGAGGGACAGAGAGCGAGACT	miRNA	miR-423-5p
t0002678	22	118	AAAAGCTGGGGTGAGAGGGCGA	miRNA	miR-320a
t0002686	22	118	TAGCAGCACGTCAATATTGGCG	miRNA	miR-16
t0002688	25	118	TGAGAACTGAATTCCATGGGTTATC	miRNA	miR-146a
t0002684	24	118	TACCACAGGGTAGAACCACGGAGA	miRNA	miR-140
t0002713	22	118	TGAGGTAGTAGTTTGCACAGTT	miRNA	let-7g
t0002720	21	118	TGGGTAGTAGATTGTATAGTT	miRNA	let-7f
t0002723	21	118	TAGAGGTAGTAGGTTGCATAG	miRNA	let-7d
t0002706	21	118	TGAGGTAGTAGGTTGTGTGGG	miRNA	let-7b
t0002712	22	117	AAATCGTTACCATTACTGAGTT	miRNA	miR-451
t0002717	23	117	TGAGGGGCAGAGAGGGAGACTTT	miRNA	miR-423-5p
t0002719	22	117	TGATGGGCAGAGAGCGAGACTT	miRNA	miR-423-5p
t0002707	22	117	TAGCACCATCTGAAATCGGTAA	miRNA	miR-29d
t0002725	23	117	ATAAAGTGCTTATAGTGCAGGTA	miRNA	miR-20
t0002740	20	117	CAAAGAATTCTCCTTTTGGG	miRNA	miR-186
t0002724	22	117	TGGAGAGAATGGCAGTTCCTGA	miRNA	miR-185
t0002726	21	117	TCAAAGTGCTTACAGTGCAGG	miRNA	miR-17
t0002730	19	117	TAGCAGCCCATCATGGTTT	miRNA	miR-15b
t0002729	22	117	CCCATAAAGTAGAAAGCACTAT	miRNA	miR-142
t0002735	25	117	TACCACAGGGTAGAACCACGGAAGA	miRNA	miR-140
t0002742	21	117	ACAACAGGGTAGAACCACGGA	miRNA	miR-140
t0002741	21	117	TGAGGTAGTAGGTTTTGTGGT	miRNA	let-7b
t0002758	23	116	TAGAGGAGATGGCGCAGGGGACT	miRNA	miR-877
t0002751	23	116	TCTGGGCACAGGCGGATGGACAG	miRNA	miR-5107
t0002757	21	116	TGAGGGGCGGAGAGCGAGACT	miRNA	miR-423-5p
t0002770	21	116	CTGGACTTGGAGTCAGAAGGC	miRNA	miR-378
t0002766	25	116	TTTGTTCGTTCGGCTCGCGTGAATC	miRNA	miR-375
t0002748	22	116	AAAAGCTGGGTTGAGAGGCGAA	miRNA	miR-320d
t0002769	22	116	AAAAGCTGGGTTGAGTGGGCGA	miRNA	miR-320a
t0002750	20	116	TGGAGAGAAAGGCAGTTTCT	miRNA	miR-185
t0002760	22	116	TGGAGAGAACGGCAGTTCCTGA	miRNA	miR-185
t0002747	22	116	TCGAGGAGCTCACAGTCTAGTA	miRNA	miR-151-5p
t0002746	20	116	TAGTACTGTGCATATCATCT	miRNA	miR-1278
t0002754	25	116	TGAGGTAGTAGATTGTATAGTTGTG	miRNA	let-7f
t0002744	21	116	TGACGTAGTAGATTGTATAGT	miRNA	let-7f
t0002785	18	116	TGTAGTAGGTTGTGTGGT	miRNA	let-7b
t0002792	21	115	TGAGGGGCAGAGAGCAAGACT	miRNA	miR-423-5p
t0002789	21	115	TAAGGGGCAGAGAGCGAGACT	miRNA	miR-423-5p
t0002774	22	115	CATTGCACTTGTCTCGGCCTGA	miRNA	miR-25
t0002773	24	115	TGACTGGGGCGGTACATCTGTTAA	miRNA	miR-219-2-3p
t0002777	22	115	TAGGTAGTTTCCTGTTGTTGGA	miRNA	miR-196b
t0002791	22	115	ACTCGGCGTGGCGTCGGTCGTT	miRNA	miR-1307
t0002780	21	115	TGAGGTAGTTGGTTGTGTGGT	miRNA	let-7b
t0002793	22	115	TGAGGTAGTAGGTCGTATAGTT	miRNA	let-7
t0002772	21	115	TGAGGTAGCAGGTTGTATAGT	miRNA	let-7
t0002778	21	114	AAACCGTTACCATTAATGAGT	miRNA	miR-451
t0002771	28	114	AACTTTGAAGGCCGAAGTGGAGAAGGGT	miRNA	miR-3526
t0002775	19	114	TACAGTATAGATGATGTAT	miRNA	miR-144
t0002787	22	114	TACCACAGGGTATAACCACGGA	miRNA	miR-140
t0002801	20	114	TGAGGTAGTAGATTGTTTAG	miRNA	let-7f
t0002820	21	114	AGAGGTAGTAGGTTGTATAGT	miRNA	let-7d
t0002817	22	114	TGAGGTAGTAGGTTGTATACTT	miRNA	let-7
t0002812	21	114	TGAGGTAGTAGGATGTATAGT	miRNA	let-7
t0002803	22	113	TATTGCACTTGTCCTGGCCTGT	miRNA	miR-92a
t0002813	23	113	TAGTGCAATATTGCTTATAGGGT	miRNA	miR-454
t0002818	23	113	AAACCGTTACCATTACTCAGTTT	miRNA	miR-451
t0002811	23	113	TGAGGGGCAGAGAGCGAGAGTTT	miRNA	miR-423-5p

t0002821	23	113	TGAGGGGCAGAGAGCGAGACCTT	miRNA	miR-423-5p
t0002833	21	113	CGCATCCCCTAGGGCATTGGT	miRNA	miR-324-5p
t0002835	24	113	AAAAGCTGGGTTGAGAGGGCGTAT	miRNA	miR-320d
t0002836	22	113	CATTGCACTTGTCTCGGTCTTA	miRNA	miR-25
t0002832	24	113	CTAGACTGAAGCTCCTTGAGGAAG	miRNA	miR-151-3p
t0002830	22	113	TACCACGGGGTAGAACCACGGA	miRNA	miR-140
t0002827	21	113	TGAGGGAGTAGTTTGTACAGT	miRNA	let-7g
t0002828	22	113	TGAGGTAGTAGTTTGTACATTT	miRNA	let-7g
t0002837	21	113	TGAGGTACTAGGTTGTATAGT	miRNA	let-7
t0002850	22	112	TCCTGTACTGAGCTGCCCCGAC	miRNA	miR-486-5p
t0002846	23	112	AAATTGTTACCATTACTGAGTTT	miRNA	miR-451
t0002856	22	112	TAGGGGCAGAGAGCGAGACTTT	miRNA	miR-423-5p
t0002844	23	112	TGAGGGGCAGAGAGCGTGACTTT	miRNA	miR-423-5p
t0002855	23	112	TCTCACACAGAAATCGCACCCGA	miRNA	miR-342-3p
t0002864	25	112	TTCAAGTAATCCAGGATAGGCTATC	miRNA	miR-26a
t0002842	23	112	TGGAGAGAAAGGCAGTTACTGAA	miRNA	miR-185
t0002841	19	112	TAGCAGCAGATCATGGTTT	miRNA	miR-15b
t0002862	23	112	TACCACAGGGTAGAACCATGGAC	miRNA	miR-140
t0002843	25	112	TGGAATGTAAAGAAGTATGGAGATC	miRNA	miR-1-3p
t0002840	23	112	AGCAGCATTGTACAGGGCTATGG	miRNA	miR-103a
t0002851	21	112	ACAGTACTGTGATAACTGAAG	miRNA	miR-101c
t0002849	23	111	TCCTGTACTGAGCTGCCCCGACA	miRNA	miR-486-5p
t0002866	22	111	AAGCAGCACGTAAATATTGGCG	miRNA	miR-457b
t0002891	20	111	TGAGGGAGTAGGTTGTGTGG	miRNA	miR-4510
t0002869	22	111	CACCTTGCGCTACTCAGGTCTG	miRNA	miR-3200-3p
t0002878	22	111	TGTGGACAGTGAGGTAGAGGGA	miRNA	miR-3138
t0002874	23	111	TAGCTTATCAGACTGATGTTGAC	miRNA	miR-21
t0002890	20	111	TGACCTATGAATTGACAGCA	miRNA	miR-192
t0002894	21	111	TGGAGAAAAAGGCAGTTCCTG	miRNA	miR-185
t0002886	22	111	TACCACAGGATAGAACCACGGA	miRNA	miR-140
t0002868	18	111	TGAGGTAGTAGGTGTGGT	miRNA	let-7a
t0002867	22	110	TATTGCACTTGTCTTGGCCTGT	miRNA	miR-92a
t0002881	23	110	TGAAAGACGATGGTAGTGAGATG	miRNA	miR-71
t0002892	23	110	CATGCCTTGAGTGTAGGACCGTA	miRNA	miR-532-5p
t0002913	21	110	TCCTGTACTGAGCTGACCCGA	miRNA	miR-486-5p
t0002919	28	110	AGGGCTGGGTCGGTCGGGCTGGGGCGC[illegible]	miRNA	miR-4651
t0002901	22	110	AAACCGTTACCATCACTGAGTT	miRNA	miR-451
t0002910	25	110	AAACCTTTACCATTACTGAGTTTAG	miRNA	miR-451
t0002914	21	110	TGGAGAGAAAGGCAGTAGTTG	miRNA	miR-4306
t0002905	24	110	TGTAAACATCCTACACTCTCAGCA	miRNA	miR-30c
t0002897	20	110	TAAGGTGCATCTAGTGCAGA	miRNA	miR-18a
t0002915	20	110	TGGAGAGAAAGGCAGTTCCC	miRNA	miR-185
t0002912	23	110	TACCATAGGGTAGAACCACGGAA	miRNA	miR-140
t0002916	24	110	ACCACAGGGTAGAACCACGGATAA	miRNA	miR-140
t0002949	20	110	TTTAGAGACGGGGTCTTGCT	miRNA	miR-1303
t0002942	19	110	TCACAGTGAACCGGTCTCT	miRNA	miR-128
t0002923	21	110	GTGGGCGGGGGCAGGTGTGTG	miRNA	miR-1228*
t0002938	23	110	AAGCAGCATTGTACAGGGCTATG	miRNA	miR-103a
t0002940	20	110	TGAGGTAGTAGATTGTATGG	miRNA	let-7f
t0002920	22	110	TTAGGTAGTAGGTTGTGTGGTT	miRNA	let-7b
t0002943	22	110	TTAGGTAGTAGGTTGTGTGGTA	miRNA	let-7b
t0002927	24	109	AAACCGTTACCATTACTGAGTTTT	miRNA	miR-451
t0002952	30	109	AACTTTGAAGACTGAAGTGGAGAAGGGT[illegible]	miRNA	miR-3526
t0002932	22	109	TGGAGAGAAAGGCAGTTGCTGA	miRNA	miR-185
t0002926	22	109	ACCACAGGGTAGAACCACGGCT	miRNA	miR-140
t0002935	23	109	TACCACAGTGTAGAACCACGGAC	miRNA	miR-140
t0002945	23	109	TACCACAGGGTAGAACCACGGTA	miRNA	miR-140
t0002933	23	109	AGCAGCATTGTACAGGGCTATAT	miRNA	miR-107

t0002936	22	109	GAGGTAGTAGATTGTATAGTTA	miRNA	let-7f
t0002941	22	109	TGAGGTAGTAGGTTGTATAGGT	miRNA	let-7
t0002946	21	108	TCCTGTACTGAGCTGCCCAGA	miRNA	miR-486-5p
t0002922	21	108	TCAGGCTCAGTCCCCTCCCGA	miRNA	miR-484
t0002944	21	108	TGAGAGGCAGAGAGCGAGACT	miRNA	miR-423-5p
t0002947	21	108	TGAGCGGCAGAGAGCGAGACT	miRNA	miR-423-5p
t0002968	22	108	TGAGGTAGTAGGTCGTGTGGTT	miRNA	let-7b
t0002963	22	107	TGAGGGGCAGAGAGCGAGGCTT	miRNA	miR-423-5p
t0002957	23	107	AAAAGTTGGGTTGAGAGGGCGTT	miRNA	miR-320d
t0002954	26	107	TGGAATGTAAGGAAGTGTGTGGAATC	miRNA	miR-206
t0002966	23	107	CAACGGAATCCCAAAAGCAGCTT	miRNA	miR-191
t0002969	20	107	TAGCATCACGTAAATATTGG	miRNA	miR-16
t0002962	22	107	TAGCCGCACGTAAATATTGGCG	miRNA	miR-16
t0002977	20	107	TACAGTATAGATGATGTATT	miRNA	miR-144
t0002960	24	107	TACCACAGTGTAGAACCACGGACA	miRNA	miR-140
t0002985	22	107	ACCACAGGGTAGAATCACGGAA	miRNA	miR-140
t0002993	21	107	TAAGGTAGTAGGTTGTGTGGT	miRNA	let-7b
t0002983	23	107	TGAGGTAGTATGTTGTGTGGTTA	miRNA	let-7b
t0002999	19	107	TGAGGTAGTAGGTTGTAGT	miRNA	let-7
t0002997	21	107	TGAGGTAGTAGGTTCTATAGT	miRNA	let-7
t0003014	24	106	TGGAAGACTAGTGATTTTGTTGTT	miRNA	miR-7
t0003017	20	106	TCTTGTACTGAGCTGCCCCG	miRNA	miR-486-5p
t0003010	22	106	TCCCACTGCTTCACTTGACTAG	miRNA	miR-4301
t0003008	22	106	CATTGCACTCGTCTCGGTTTGA	miRNA	miR-25
t0003007	21	106	TGGAGAGAAAGGCAGTCCCTG	miRNA	miR-185
t0003025	22	106	TGGAGAGAAAGGCAGTACCTGA	miRNA	miR-185
t0003026	24	106	TAGCAGCACGTAAATATTGGCGAT	miRNA	miR-16
t0003009	22	106	TAGCAGAACATCATGGTTTACA	miRNA	miR-15b
t0003015	21	106	ACCACATGGTAGAACCACGGA	miRNA	miR-140
t0003005	22	106	ACCATAGGGTAGAACCACGGAC	miRNA	miR-140
t0003018	20	106	ACCACAGGGTAGAACTACGG	miRNA	miR-140
t0003004	21	106	TGAGGTAGTAGGTTGGGTGGT	miRNA	let-7b
t0003012	22	105	ACTGGCTAGGGAAAATGATTGG	miRNA	miR-664*
t0003044	22	105	GAGCTTATTCATAAAAGTGCAG	miRNA	miR-590-5p
t0003045	21	105	TCACACAGAAATCGCACCCGT	miRNA	miR-342-3p
t0003042	21	105	CGGGGAGAGAACGCAGTGACG	miRNA	miR-3175
t0003039	23	105	TGGAGAGAAAGGCAGTTCCTGAC	miRNA	miR-185
t0003038	20	105	TAGAGAAAGGCAGTTCCTGA	miRNA	miR-185
t0003043	20	105	TAGCAGCAGATCATGGTTTA	miRNA	miR-15b
t0003046	21	105	ATACAGTATAGATGATGTACT	miRNA	miR-144
t0003047	25	105	AGCAGCATTGTACAGGGCTATGAGC	miRNA	miR-103a
t0003029	22	105	TGAGGTAGTAGAGTGTATAGTT	miRNA	let-7f
t0003037	21	105	TGAGGTAGTAGGTTATGTGGT	miRNA	let-7b
t0003053	20	104	CAAAGTGCTGTTCGTGCAGG	miRNA	miR-93
t0003041	23	104	AGGGACGGGACGCGGTGCAGTGA	miRNA	miR-92b*
t0003075	23	104	TGTAGAGCAGGGAGCAGGAAGTT	miRNA	miR-4732-5p
t0003073	20	104	AAACCATTACCATTACTGAG	miRNA	miR-451
t0003057	23	104	ATATAATACAACCTGCTAAGTGA	miRNA	miR-374
t0003078	26	104	AGGCAGTGTAGTTAGCTGATTGCATC	miRNA	miR-34c
t0003058	18	104	AAGCTGCCAGTTGAAGAA	miRNA	miR-22-3p
t0003071	22	104	CAACGGAATCCCAAAAGAAGCT	miRNA	miR-191
t0003056	20	104	TGGAGAGAAAGGCAGTTCCG	miRNA	miR-185
t0003065	20	104	TGGAGAGAAAGGCAGTTCGA	miRNA	miR-185
t0003064	23	104	ATAGCAGCACATAATGGTTTGTG	miRNA	miR-15a
t0003077	22	104	TGAGGTAGTATTTTGTACAGTT	miRNA	let-7g
t0003068	21	104	TGAGGTTGTAGATTGTATAGT	miRNA	let-7f
t0003108	20	104	GGTAGTAGATTGTATAGTGA	miRNA	let-7f
t0003094	22	103	TCCTGTACTTAGCTGCCCCGAG	miRNA	miR-486-5p

t0003102	22	103	ATTGCACGGTATCCATCTGTAA	miRNA	miR-363
t0003093	20	103	TAGAAGCACATCATGGTTTA	miRNA	miR-15b
t0003098	20	103	CTACAGTATAGATGATGTAC	miRNA	miR-144
t0003105	26	103	ACCACAGGGTAGAACCACGGACAAGA	miRNA	miR-140
t0003099	22	103	GTGAGGACTCGGGAGGTGGAGA	miRNA	miR-1224
t0003106	23	103	ATCAGCATTGTACAGGGCTATGA	miRNA	miR-103a
t0003100	24	102	AGGTGGAAGGTTGTAGGCATGTAT	miRNA	miR-965-5p
t0003107	19	102	TGTACTGAGCTGCCCCGAA	miRNA	miR-486-5p
t0003091	22	102	AGGAGAGAAAGGCAGTTCCTGA	miRNA	miR-185
t0003085	22	102	TAGCAGCACGTAAATATAGGCG	miRNA	miR-16
t0003087	20	102	TAGCAGAACATAATGGTTTG	miRNA	miR-15a
t0003092	22	102	CCCATAAAGTAGAAAGCACTAG	miRNA	miR-142
t0003129	23	102	CAGTGCAATGTTAAAAGGGCATT	miRNA	miR-130a
t0003118	21	102	AGCAGCATTGTACAGGGTCAT	miRNA	miR-103a
t0003119	20	102	TGAGGTACTAGATTGTATAG	miRNA	let-7f
t0003121	26	101	GAAGACTGAAGTGGAGAAGGGTTTCT	miRNA	miR-739
t0003111	20	101	GGAGAGAAAGGCAGTTCCTG	miRNA	miR-185
t0003124	20	101	TAGCAGCACGTAAATATTGT	miRNA	miR-16
t0003120	23	101	TGAGGTAGTAGATTGTATATGTT	miRNA	let-7f-5p
t0003144	21	101	TGAGGTAGTACATTGTATAGT	miRNA	let-7f
t0003155	22	101	TGATGTAGTAGGTTGTATAGTT	miRNA	let-7
t0003145	23	100	AAATCGTTACCATTACTGAGTTT	miRNA	miR-451
t0003162	20	100	AAACCGTTACCATTACCGAG	miRNA	miR-451
t0003142	23	100	TGAGGGGCAGAGAACGAGACTTT	miRNA	miR-423-5p
t0003135	21	100	CATTGCACTTGTCTCGGGCTG	miRNA	miR-25
t0003158	22	100	TAGCAGCACGTAAATGTTGGCG	miRNA	miR-16
t0003149	24	100	TACCACATGGTAGAACCACGGACA	miRNA	miR-140
t0003156	24	100	TACCAGAGGGTAGAACCACGGACA	miRNA	miR-140
t0003171	24	100	TACCACAGGGTAGAACCACGGATC	miRNA	miR-140
t0003179	22	100	CTGAGGTAGTAGTTTGTACAGT	miRNA	let-7g
t0003177	24	100	TGAGGTAGTAGTTTGTACAGTTAA	miRNA	let-7g
t0003181	23	100	TGAGGTAGTAGTTTGTACAGTAA	miRNA	let-7g
t0003172	20	100	TAAGGTAGTAGATTGTATAG	miRNA	let-7f
t0003170	20	100	TGAGTTAGTAGATTGTATAG	miRNA	let-7f
t0003190	22	100	TGAGGTCGTAGGTTGTGTGGTT	miRNA	let-7b
t0003205	22	100	TGAGGTAGAAGGTTGTGTGGTT	miRNA	let-7b
t0003218	22	99	CTGGCTAGGGAAAATGATTGGA	miRNA	miR-664*
t0003207	26	99	TCCTGTACTGAGCTGCCCCGAGATGG	miRNA	miR-486-5p
t0003197	20	99	AAACCGTTAACATTACTGAG	miRNA	miR-451
t0003192	23	99	AAACCGTTACCATTACTGAGGTT	miRNA	miR-451
t0003198	21	99	AAAGCTGGGTTGAGAGGGCAT	miRNA	miR-320d
t0003210	22	99	AAAAGTCGGGTTGAGAGGGCGT	miRNA	miR-320c
t0003195	23	99	CTTTCAGTCGGATGTTTACAGCA	miRNA	miR-30e*
t0003204	22	99	TAGCAGCACGTAAATATTGGTA	miRNA	miR-16b
t0003219	23	99	TACCACAGGGTAGAACCAAGGAA	miRNA	miR-140
t0003193	22	99	TGAAGTAGTAGTTTGTACAGTT	miRNA	let-7g
t0003214	18	99	AGAGGTAGTAGGTTGCAT	miRNA	let-7d
t0003209	25	98	TCCTGTACTGAGCTGCCCCGACGGT	miRNA	miR-486-5p
t0003211	21	98	TCCTGTACTGAGCTGCCCAAA	miRNA	miR-486-5p
t0003237	20	98	AAATTGTTACCATTACTGAG	miRNA	miR-451
t0003231	22	98	AAACCGTTACCATTAATGAGTT	miRNA	miR-451
t0003236	23	98	ATGACACGATCACTCCCGTTGAG	miRNA	miR-425-5p
t0003247	27	98	ACTGGACTTGGAGTCAGAAGGCATATC	miRNA	miR-378
t0003222	25	98	TTTGAAGACTGAAGTGGAGAAGGGT	miRNA	miR-3526
t0003225	23	98	TTCAAGTAATCCAGGATAGGCTT	miRNA	miR-26a
t0003220	23	98	CAACGGAATCTCAAAAGCAGCTG	miRNA	miR-191
t0003221	23	98	TGGAGAGAAAGGCAGTTTCTGAA	miRNA	miR-185
t0003242	23	98	TACCACAGGGTAGAATCACGGAA	miRNA	miR-140

t0003241	22	97	TACGTCATTGTTGTCATCGTCA	miRNA	miR-598
t0003240	22	97	TCCTGTACTGAGATGCCCCGAG	miRNA	miR-486-5p
t0003271	20	97	TCCTGTACTGAGCTGCCGAG	miRNA	miR-486-5p
t0003278	21	97	TCTCCCTTCCTGCCCTGGCTA	miRNA	miR-4685-3p
t0003268	24	97	AAACCGTTACCATTACTGAGTAAA	miRNA	miR-451
t0003282	22	97	CATTGCAATTGTCTCGGTCTGA	miRNA	miR-25
t0003280	23	97	TGTCAGTTTGTCAAATACCCCAA	miRNA	miR-223
t0003269	20	97	TAGCAGCACGTAAAAATTGG	miRNA	miR-16
t0003263	24	97	ACCACAGGGTAGAACCACGGAATC	miRNA	miR-140
t0003260	22	97	TCTGGGCAACAAAGTGAGACCT	miRNA	miR-1285
t0003283	22	97	GTACAGTACTGTGATAACTGAC	miRNA	miR-101c
t0003248	18	97	TACAGTACTGTGATAACT	miRNA	miR-101
t0003253	20	97	TGAGGTCGTAGATTGTATAG	miRNA	let-7f
t0003305	21	96	TATTGCACTCGTCCCGGCCAT	miRNA	miR-92
t0003286	22	96	AAACCGTTACCATTACTGACTT	miRNA	miR-451
t0003295	26	96	ACTTTGAAGACTGAAGTGGAGAAGGG	miRNA	miR-3526
t0003306	22	96	AAAAGCTGGGTTGAGAGGGGGA	miRNA	miR-320d
t0003288	23	96	AAAAGCTGGTTTGAGAGGGCGAA	miRNA	miR-320a
t0003297	20	96	ACTGCAGTGAAGGCACTTGT	miRNA	miR-17*
t0003312	21	96	ACCACAGGGTAGAACCACAGA	miRNA	miR-140
t0003303	22	96	TACAGTACTGTGATAACTGAAC	miRNA	miR-101
t0003299	21	96	TGAGGTAGTAGAATGTATAGT	miRNA	let-7f
t0003317	21	96	TGCGGTAGTAGGTTGTGTGGT	miRNA	let-7b
t0003300	23	95	TGGAAGACTGGTGATATGTTGTT	miRNA	miR-7-5p
t0003293	21	95	TGCAGCACGTAAATATTGGCG	miRNA	miR-16
t0003294	22	95	TAGCAGCATATCATGGTTTACA	miRNA	miR-15b
t0003340	19	95	TAGCAGCACATCATGGGTT	miRNA	miR-15b
t0003334	18	95	GGATATCATCATATACTG	miRNA	miR-144*
t0003336	23	95	TGAGATGAAGCACTGTAGCTCTC	miRNA	miR-143
t0003321	24	95	TACCACAGGGTAGAATTACGGACA	miRNA	miR-140
t0003326	20	95	TGCGGTAGTAGATTGTATAG	miRNA	let-7f
t0003330	20	95	TGAAGTAGTAGATTGTATAG	miRNA	let-7f
t0003327	22	95	TGAGGTAGTCGGTTGTGTGGTT	miRNA	let-7b
t0003328	20	94	GTAGAGGAGATGGCGCAGGA	miRNA	miR-877
t0003329	22	94	TCTGGGCACAGGCGGATGGACT	miRNA	miR-5107
t0003318	26	94	CTTGCTATCTGGGCATGCTACTATGA	miRNA	miR-500b
t0003349	21	94	ATCTGTACTGAGCTGCCCCGA	miRNA	miR-486-5p
t0003363	21	94	ACTCCCACTGCTTCACTTGAC	miRNA	miR-4301
t0003342	21	94	TGAGGGGCAGAGAGCGATACT	miRNA	miR-423-5p
t0003344	22	94	TGGAAGGTAGACGGCCAGAGTT	miRNA	miR-3190
t0003347	20	94	TGGAAGGTAGACGGCCAGAG	miRNA	miR-3190
t0003345	23	94	GAGGGTTGGGTGGAGGCTCTATC	miRNA	miR-296-3p
t0003356	24	94	TTCAAGTAATCCAGGATAGGCTAG	miRNA	miR-26a
t0003352	22	94	TGGAAAGAAAGGCAGTTCCTGA	miRNA	miR-185
t0003355	24	94	TAGCAGCACATCATGGTTTACAAA	miRNA	miR-15b
t0003354	21	94	ACCACAGGGTAGAACCATGGA	miRNA	miR-140
t0003384	22	94	TGCCACAGGGTAGAACCACGGA	miRNA	miR-140
t0003389	21	94	TCCCTGAGACCCTAACTTGTG	miRNA	miR-125b-5p
t0003413	20	94	TGAGGTAGTAGATTTTATAG	miRNA	let-7f
t0003375	24	94	TGAGGTAGTAGATTGTATAGTTTG	miRNA	let-7f
t0003371	22	94	TGAGGTAATAGGTTGTGTGGTT	miRNA	let-7b
t0003387	21	94	TGAGGTAGTACGTTGTATAGT	miRNA	let-7
t0003382	22	94	TTAGGTAGTAGGTTGTATAGTT	miRNA	let-7
t0003401	22	93	TATTGCACTCGTCCCGGCCTAT	miRNA	miR-92
t0003386	21	93	CAAAAGTGATCGTGGTTTTTG	miRNA	miR-548t
t0003395	20	93	TCCTGTACTGAGCTGCCCGA	miRNA	miR-486-5p
t0003365	22	93	TCCTGTACTGAGCTGCCCCTAG	miRNA	miR-486-5p
t0003398	22	93	AAACCGTTACCATTACTAAGTT	miRNA	miR-451

t0003377	20	93	AAACAGTTACCATTACTGAG	miRNA	miR-451
t0003393	20	93	AAACCGTTATCATTACTGAG	miRNA	miR-451
t0003390	23	93	GATGAGGATGGATAGCAAGGAAG	miRNA	miR-3605-5p
t0003396	25	93	CTTTCAGTCGGATGTTTGCAGCATC	miRNA	miR-30a*
t0003411	25	93	TGAAATGTTTAGGACCACTAGTATC	miRNA	miR-203
t0003408	23	93	TAAAGTGCTTATAGTGCAGGTAT	miRNA	miR-20
t0003368	22	93	CAACGGAATCCCAAAAGCAGTT	miRNA	miR-191
t0003379	22	93	TGGACAGAAAGGCAGTTCCTGA	miRNA	miR-185
t0003436	24	93	AATAGCAGCACGTAAATATTGGCG	miRNA	miR-16
t0003446	21	93	CCACAGGGTAGAACCACGGTA	miRNA	miR-140
t0003420	24	93	TCGGATCCGTCTGAGCTTGGTATC	miRNA	miR-127
t0003423	22	93	ACCGCACTGTGGGTACTTGCTG	miRNA	miR-106b*
t0003421	21	93	TACAGTACTGTGATAATTGAA	miRNA	miR-101
t0003431	24	93	TGAGGTAGTAGTTTGTACAGTTGA	miRNA	let-7g
t0003437	23	92	TAAGGGCTGGGTCGGTCGGGCTG	miRNA	miR-4651
t0003434	25	92	AATCTGAGAAGGCGCACAAGGTTTG	miRNA	miR-3200-5p
t0003425	23	92	ATGGAGAGAAAGGCAGTTCCTGA	miRNA	miR-185
t0003441	26	92	ACCACAGGGTAGAACCACGGAAGATC	miRNA	miR-140
t0003422	23	92	TACAGTACTGTGATAACTGAAGT	miRNA	miR-101
t0003442	21	92	CTGAGGTAGTAGATTGTATAG	miRNA	let-7f
t0003415	20	92	TGAGGTAGTAGATTATATAG	miRNA	let-7f
t0003443	24	92	TGAGGTAGTAGATTGTATAGTTAG	miRNA	let-7f
t0003430	21	92	TGAGGTAGTAAATTGTATAGT	miRNA	let-7f
t0003438	22	92	TGAGGTAGTAAGTTGTGTGGTT	miRNA	let-7b
t0003447	22	92	TGAGATAGTAGGTTGTGTGGTT	miRNA	let-7b
t0003435	21	92	TGAGTTAGTAGGTTGTGTGGA	miRNA	let-7b
t0003444	22	92	TGAGGTAGTAGGTTGAGTGGTT	miRNA	let-7b
t0003473	23	91	TGGAAGACTAGTGATTTTGTTGT	miRNA	miR-7
t0003474	21	91	AAACCGTTACAATTACTGAGT	miRNA	miR-451
t0003472	21	91	ATCAGCATTGTACAGGGCTAT	miRNA	miR-4289
t0003475	23	91	TGCGGGGCAGAGAGCGAGACTTT	miRNA	miR-423-5p
t0003482	23	91	TGAGGGGCAGAGAGCGAGACTGA	miRNA	miR-423-5p
t0003449	23	91	TGAGGGGCAGAGAGCGCGACTTT	miRNA	miR-423-5p
t0003456	23	91	TGAGGGGCAGAAAGCGAGACTTT	miRNA	miR-423-5p
t0003469	21	91	ATCACATTGCCAGGGATTTAA	miRNA	miR-23a
t0003484	23	91	TAGCAGCACGTAAATATTGGCAA	miRNA	miR-16b
t0003448	22	91	AGCAGCACGTAAATATTGGCGT	miRNA	miR-16
t0003451	23	91	GTAGTGTTTCCTACTTTATGGAA	miRNA	miR-142-3p
t0003452	25	91	TCCCTGAGACCCTAACTTGTGAATC	miRNA	miR-125b-5p
t0003460	22	91	TGAGGAAGTAGTTTGTACAGTT	miRNA	let-7g
t0003501	22	91	TGAGGTAGTAGTATGTACAGTT	miRNA	let-7g
t0003493	20	91	TGAGGTAGTAGATTGTCTAG	miRNA	let-7f
t0003498	20	91	TGGGTAGTAGATTGTATAGT	miRNA	let-7f
t0003505	21	91	TGAGGTAGGAGGTTGTGTGGT	miRNA	let-7b
t0003512	21	90	TGAGGTAGTAAGTTGTATTGT	miRNA	miR-98
t0003517	23	90	TATTGCATTTGTCCCGGCCTGTA	miRNA	miR-92a
t0003523	22	90	TCCTGTACTGAGCTACCCCGAG	miRNA	miR-486-5p
t0003494	25	90	TGTAGAGCAGGGAGCAGGAAGCTGT	miRNA	miR-4732-5p
t0003527	22	90	AAACCGTTACCATTACTGATTT	miRNA	miR-451
t0003525	21	90	AGTAGCATTGTACAGGGCTAT	miRNA	miR-4289
t0003510	21	90	CATCGGGAATGTCGTGTCCGT	miRNA	miR-425*
t0003518	23	90	TGAGGGTCAGAGAGCGAGACTTT	miRNA	miR-423-5p
t0003508	24	90	AAAAGCTGGGTTGAGAGGGCGGAT	miRNA	miR-320d
t0003529	24	90	AAAGGTAGATAGAACAGGTCTTGA	miRNA	miR-1839
t0003514	23	90	TGAGGGAGTAGATTGTATAGTTG	miRNA	miR-1827
t0003528	23	90	TACCACAGGCTAGAACCACGGAC	miRNA	miR-140
t0003500	22	90	ACCACAGGGTAGAACAACGGAA	miRNA	miR-140
t0003572	19	90	AGCAGCATTGTACAGGGTT	miRNA	miR-103a

t0003557	21	90	AGCAGCATTGTACATGGCTAT	miRNA	miR-103a
t0003565	24	90	GTACAGTACTGTGATAGCTGAATC	miRNA	miR-101
t0003554	23	90	TGAGGTAGTAGATTGTATAGGGA	miRNA	let-7f-5p
t0003576	22	90	TGAGGTAGTAGATTGTGTAGTA	miRNA	let-7f
t0003546	22	90	TGAGGTAGTAGGTTGTGTCGTT	miRNA	let-7b
t0003537	24	90	TGAGGTAGTAGGTTGTGTGGTTTA	miRNA	let-7b
t0003540	20	89	AGGTGGAAGGTTGTAGGCAT	miRNA	miR-965-5p
t0003574	22	89	ATTGCACTTGTCCCGGCCTGTT	miRNA	miR-92c
t0003556	20	89	AAAAGTACTTGCGGATTTTG	miRNA	miR-548k
t0003575	21	89	TCCTGTACTGAGATGCCCCGA	miRNA	miR-486-5p
t0003562	21	89	TGGGGCTAGTGATGCAGGACG	miRNA	miR-4489
t0003564	22	89	TGAGGGGCAGAGCGCGAGACTT	miRNA	miR-423-5p
t0003551	21	89	TGAGGAGCAGAGAGCGAGACT	miRNA	miR-423-5p
t0003578	22	89	AAAAGCTGGGTTGAGAGGGAGT	miRNA	miR-320d
t0003568	22	89	AAAAGCTGGGTTGAGGGGGCGA	miRNA	miR-320a
t0003533	24	89	ATGACCTATGATTTGACAGACATC	miRNA	miR-215
t0003567	23	89	TGGAGAGAAAGGCAGTTCTTGAA	miRNA	miR-185
t0003555	21	89	TGGAGGGAAAGGCAGTTCCTG	miRNA	miR-185
t0003558	21	89	TAGCAGCACGTAAATTTTGGC	miRNA	miR-16
t0003588	22	89	ATAGCAGCACATCATGGTTTAC	miRNA	miR-15b
t0003605	23	89	TCAGTGCATCACAGAACTTTGAA	miRNA	miR-148b-3p
t0003601	23	89	TGAGATTAAGCACTGTAGCTATC	miRNA	miR-143
t0003592	21	89	ACCACGGGGTAGAACCACGGA	miRNA	miR-140
t0003620	22	89	TACCACAGGGTAGAACCACCGA	miRNA	miR-140
t0003602	22	89	GGAGGTAGTAGGTTGTGTGGTT	miRNA	let-7b
t0003616	22	89	TGAGGTAGTAGGTTGTGTGTTA	miRNA	let-7b
t0003599	23	89	CTGAGGTAGTAGGTTGTATAGTT	miRNA	let-7
t0003609	24	88	CAAAGTGCTGTTCGTGCAGGTAGA	miRNA	miR-93
t0003621	22	88	AAGACTGAAGTGGAGAAGGGTT	miRNA	miR-739
t0003608	20	88	GACTATAGAACTTTCCCCCT	miRNA	miR-625*
t0003612	24	88	TCCTGTACTGAGCTGCCCCGAGAG	miRNA	miR-486-5p
t0003622	23	88	TGAGGGGCAGCGAGCGAGACTTT	miRNA	miR-423-5p
t0003595	24	88	GGCTGGTCCGAGTGCAGTGGTGTT	miRNA	miR-3135b
t0003619	20	88	TGACCTATGAATTGACAGCT	miRNA	miR-192
t0003583	19	88	AGGTAGATAGAACAGGTCT	miRNA	miR-1839
t0003586	29	88	TTTGGCAATGGTAGAACTCACACTGGATC	miRNA	miR-182
t0003614	23	88	AACATTCATTGCTGTCGGTGGGA	miRNA	miR-181b
t0003615	22	88	TAGCAGCACATCATGGTTCACA	miRNA	miR-15b
t0003627	23	88	TAAAGTGCTGACAGTGCAGATGA	miRNA	miR-106b
t0003629	22	88	AACAGCATTGTACAGGGCTATG	miRNA	miR-103a
t0003671	22	88	TGAGGTAGTAGTTTGTACAGTC	miRNA	let-7g
t0003673	22	88	TGAGGTAGTAGCTTGTGTGGTT	miRNA	let-7b
t0003656	22	88	TGAGGTAGTAGGTTGTGAGGTT	miRNA	let-7b
t0003654	18	88	TGAGGTAGTAGGTTGTAG	miRNA	let-7
t0003639	21	87	TCCTGTACTGAGCTGCACCGA	miRNA	miR-486-5p
t0003637	23	87	TGAGGGAGTAGGTTGTGTGGTTA	miRNA	miR-4510
t0003649	20	87	AAACCGTTACCATTAGTGAG	miRNA	miR-451
t0003661	22	87	TGAGGGGCAGAGATCGAGACTT	miRNA	miR-423-5p
t0003658	23	87	TTCAAGTAATCCAGGATAGGCTA	miRNA	miR-26a
t0003665	24	87	TTCAAGTAATCCAGGATAGGCTAA	miRNA	miR-26a
t0003660	23	87	CAACGGAATTCCAAAAGCAGCTG	miRNA	miR-191
t0003640	23	87	CTGGAGAGAAAGGCAGTTCCTGA	miRNA	miR-185
t0003663	25	87	TGGACGGAGAACTGATAAGGGTATC	miRNA	miR-184-3p
t0003638	19	87	TAGCATGTAAATATTGGCG	miRNA	miR-16c
t0003647	20	87	TAGCAGCACATCATGGTCTA	miRNA	miR-15b
t0003662	19	87	TAGCAGCGCATCATGGTTT	miRNA	miR-15b
t0003674	22	87	ACCACAGGGTAGAATTACGGAT	miRNA	miR-140
t0003732	22	87	TACCACAGGGTAGAACCACGTA	miRNA	miR-140

t0003714	21	87	TGAGGTAGTAGTTTGTGTTGT	miRNA	let-7i
t0003728	22	87	GAGGTAGTAGATTGTATAGTTT	miRNA	let-7f
t0003681	25	87	AGAGGTAGTAGGTTGCATAGTTAAA	miRNA	let-7d
t0003706	22	87	TGAGGTAGTAGGTTGTGTGATT	miRNA	let-7b
t0003708	23	87	TGAGGCAGTAGGTTGTGTGGTTA	miRNA	let-7b
t0003713	20	87	TGAGGTAGTAGGTTGTGTTT	miRNA	let-7b
t0003712	19	86	CTATACAACTTACTACTTT	miRNA	miR-98*
t0003717	20	86	ATTGCACTTGTCCCGGCCTG	miRNA	miR-92c
t0003707	23	86	CATGCCTTGAGTGTAGGACCGTT	miRNA	miR-532-5p
t0003709	20	86	TAGCAGCGGGAACAGTTCAA	miRNA	miR-503
t0003727	20	86	TCCTGTACTGAGCTGCCTCG	miRNA	miR-486-5p
t0003683	21	86	TCCTGTACTGAGCTGCCACGA	miRNA	miR-486-5p
t0003701	20	86	TGTACTGAGCTGCCCCGAGA	miRNA	miR-486-5p
t0003687	20	86	AAATCGTTACCATTACTGAG	miRNA	miR-451
t0003693	22	86	AAACCTTTACCACTACTGAGTT	miRNA	miR-451
t0003684	24	86	TCCCACTGCTTCACTTGACTAGCC	miRNA	miR-4301
t0003718	22	86	AACGGAATTCCAAAAGCAGCTG	miRNA	miR-191
t0003725	22	86	TGGAGAGAAAGGCTGTTCCTGA	miRNA	miR-185
t0003733	22	86	TAGCAGCACATCATGTTTTACA	miRNA	miR-15b
t0003769	25	86	CCCATAAAGTAGAAAGCACTACATC	miRNA	miR-142
t0003764	23	86	TACCACAGGGGAGAACCACGGAA	miRNA	miR-140
t0003741	22	86	CTCGGCGTGGCGTCGGTCGTGG	miRNA	miR-1307
t0003734	27	85	AGGGCTGGGTCGGTCGGGCTGGGGCGC	miRNA	miR-4651
t0003753	21	85	AAAAGCTGGGTTCAGAGGGCG	miRNA	miR-320a
t0003754	23	85	TGGCTCAGTTCAGCAGGAACAGA	miRNA	miR-24b
t0003772	23	85	TGGAGAGAAAGGAAGTTCCTGAT	miRNA	miR-185
t0003765	21	85	TTGAGAGAAAGGCAGTTCCTG	miRNA	miR-185
t0003766	20	85	TGGAGAGAAAGGCAGTTCTT	miRNA	miR-185
t0003752	22	85	TGGAGAGAAAGGCAGTTCGTGA	miRNA	miR-185
t0003767	18	85	AAGGTAGATAGAACAGGT	miRNA	miR-1839
t0003761	18	85	TAGCAGCACATCATGTTT	miRNA	miR-15b
t0003758	20	85	CAGTGCAATGTTAAAAGGGC	miRNA	miR-130a
t0003735	21	85	AGAGGTAGTAGATTGTATAGT	miRNA	let-7f
t0003737	24	85	AGAGGTAGTAGGTTGCATAGTTAA	miRNA	let-7d
t0003744	21	85	ATAGGTAGTAGGTTGCATAGT	miRNA	let-7d
t0003784	22	85	TGAGGTAGTAGGTGGTGTGGTT	miRNA	let-7b
t0003797	20	84	AAACCGTTACCATTAATGAG	miRNA	miR-451
t0003800	23	84	TGACGGGCAGAGAGCGAGACTTT	miRNA	miR-423-5p
t0003781	20	84	AGGGGCAGAGAGCGAGACTT	miRNA	miR-423-5p
t0003796	23	84	AATTGCACGGTATCCATCTGTAT	miRNA	miR-363
t0003804	23	84	AGCTACATCTGGCTACTGGGTCA	miRNA	miR-222
t0003815	20	84	CAACGGAATTCCAAAAGCAG	miRNA	miR-191
t0003818	20	84	TGGAGAGAAAGGCAGTTCTG	miRNA	miR-185
t0003790	19	84	CGAATCATTATTTGCTGCT	miRNA	miR-15b*
t0003786	22	84	TAGCAGCACATAATGGTTTTTG	miRNA	miR-15a
t0003814	21	84	TACCACAGGGTAGAACCCCGG	miRNA	miR-140
t0003810	23	84	TACCACAGGGTAGAACCACGGGC	miRNA	miR-140
t0003825	23	84	CCGCACTGTGGGTACTTGCTGAA	miRNA	miR-106b*
t0003827	22	84	AGCAGCATTATACAGGGCTATG	miRNA	miR-103a
t0003830	24	84	TGAGGTAGTAGATTGTATAGTATC	miRNA	let-7f
t0003832	25	84	TGAGGTAGTAGGTTGTGTGGTTAAT	miRNA	let-7b
t0003876	22	84	TGAGGTAGTAGGTTGGGTGGTT	miRNA	let-7b
t0003867	22	83	TATTGCACTCGTCCCGGCCTTA	miRNA	miR-92
t0003866	20	83	ATCAGCATTGTACAGGGCTA	miRNA	miR-4289
t0003846	19	83	CAGCAGCAATTCATGTTTT	miRNA	miR-424
t0003851	21	83	TGAGGGCCAGAGAGCGAGACT	miRNA	miR-423-5p
t0003845	22	83	TTATAAAGCAATGAGACTGATA	miRNA	miR-340-5p
t0003831	22	83	AAAAGCTTGGTTGAGAGGGCGA	miRNA	miR-320a

t0003857	21	83	AGGTAGATAGAACAGGTCTTA	miRNA	miR-1839
t0003868	22	83	TAGCAGCACGTAAATATGGGCG	miRNA	miR-16
t0003864	23	83	CTAGACTGAAGCTCCTTGAGGTA	miRNA	miR-151-3p
t0003839	25	83	AGATGAAGCACTGTAGCTCTTTATC	miRNA	miR-143
t0003849	26	83	AGCAGCATTGTACAGGGCTATGAATC	miRNA	miR-103a
t0003841	20	83	TGAGGTAGAAGATTGTATAG	miRNA	let-7f
t0003854	20	83	TGAGGAAGTAGGTTGTATAG	miRNA	let-7
t0003911	20	82	TGGGTTTACGTTGGGAGAAC	miRNA	miR-629
t0003892	21	82	CTCCCACACCCAAGGCTTGCA	miRNA	miR-532-3p
t0003912	23	82	TCTTGTACTGAGCTGCCCCGAGT	miRNA	miR-486-5p
t0003904	22	82	AAATTGTTACCATTACTGAGTT	miRNA	miR-451
t0003915	24	82	TGAGGGGCAGAGAGCGAGACTAGA	miRNA	miR-423-5p
t0003910	23	82	AATTGCACGGTATCCATCTGTAG	miRNA	miR-363
t0003883	23	82	TGTAAACATCCCCGACTGGAAGC	miRNA	miR-30d
t0003907	22	82	CATTCCACTTGTCTCGGTCTGA	miRNA	miR-25
t0003908	21	82	TGGGAGAAAGGCAGTTCCTGA	miRNA	miR-185
t0003920	22	82	TCGAGAGAAAGGCAGTTCCTGA	miRNA	miR-185
t0003916	21	82	ATGGCACTGGTAGAATTCACT	miRNA	miR-183
t0003902	20	82	CATAAAGTAGAAAGCACTAC	miRNA	miR-142-5p
t0003899	22	82	CCCATAAAGTAGAAAGCACTAC	miRNA	miR-142
t0003962	24	82	TGAGGTAGTAGTTTGTGCTGTTAA	miRNA	let-7i
t0003958	23	82	AGAGGTAGTAGGTTGCATAGTAA	miRNA	let-7d
t0003927	21	82	TGAGGTAGTACGTTGTGTGGT	miRNA	let-7b
t0003952	22	81	TATTGCACTCGTCCCGGCCTTT	miRNA	miR-92
t0003930	19	81	TGCGGGGCTAGGGCTAACA	miRNA	miR-744
t0003965	20	81	AAGACTGAAGTGGAGAAGGG	miRNA	miR-739
t0003948	20	81	AAAAGTAATCGTGGTCTTTG	miRNA	miR-548j
t0003945	22	81	TCCTGTACTGAGCTGCCACGAG	miRNA	miR-486-5p
t0003950	22	81	TGAGGGAGTAGGTTGTGTGGTA	miRNA	miR-4510
t0003960	21	81	AAACCGTTACCGTTACTGAGT	miRNA	miR-451
t0003963	21	81	TGAGGGGCAGAGAGCGAGAGT	miRNA	miR-423-5p
t0003966	27	81	TGTAAACATCCTCGACTGGAAGCTATC	miRNA	miR-30d
t0003957	23	81	TGTGCAAATCCATGCAAAACTGA	miRNA	miR-19b
t0003961	26	81	CAACGGAATCCCAAAAGCAGCTGATC	miRNA	miR-191
t0003935	24	81	CAACGGAATTCCAAAAGCAGCTGT	miRNA	miR-191
t0003931	20	81	TACAGTATAGATGATGTTCT	miRNA	miR-144
t0003989	23	81	TACCACAAGGTAGAACCACGGAC	miRNA	miR-140
t0003980	21	81	TACCCTGTAGATCCGAATTTG	miRNA	miR-10a
t0004007	22	81	AAGAGGTAGTAGGTTGCATAGT	miRNA	let-7d
t0004000	22	81	TGAGGTAGTATGTTGTATAGTT	miRNA	let-7
t0003988	20	80	AGAGGAGATGGCGCAGGGGA	miRNA	miR-877
t0003990	21	80	TCCTGTACTGAGCTACCCCGA	miRNA	miR-486-5p
t0003981	27	80	AAACCGTTACCATTACTGAGTTTAAAA	miRNA	miR-451
t0003972	20	80	AAACCGTTACCATTACTGAA	miRNA	miR-451
t0003967	21	80	TGAGGGGCAGAGAGGGAGACT	miRNA	miR-423-5p
t0003984	23	80	TTTTCAGTCGGATGTTTACAGTA	miRNA	miR-30b-3p
t0003995	21	80	TGGAGAGAAAGGGAGTTCCTG	miRNA	miR-185
t0004006	23	80	TGAGATGAAGCACTGTACCTATC	miRNA	miR-143
t0003968	23	80	TACCACAGGGTAGAACTACGGAC	miRNA	miR-140
t0003975	19	80	ACTCGGCGTGGCGTCGGTC	miRNA	miR-1307
t0004011	22	80	CATTATTACTTTTGGTACGCGA	miRNA	miR-126-5p
t0004005	21	80	CGTACCGTGAGTAATAATGCG	miRNA	miR-126
t0003997	20	80	AGCAGCATTGTACAGGTCTA	miRNA	miR-103a
t0004017	22	80	AGCAGCATTGTACAGGGTTATG	miRNA	miR-103a
t0004033	19	80	TGAGGTAGTAGATTGTTAG	miRNA	let-7j
t0004042	22	80	TGAGGTAGCAGTTTGTACAGTT	miRNA	let-7g
t0004054	22	80	TGAGGTAGTAGTTTGTACAGGT	miRNA	let-7g
t0004065	20	79	AGGGACGGGACGCGGTGCAA	miRNA	miR-92b*

t0004030	25	79	TATTGCACTTGTCCCGGCCTGTAGA	miRNA	miR-92a
t0004048	21	79	TAAGGGCTGGGTCGGTCGGGC	miRNA	miR-4651
t0004025	18	79	AGGGCTGGGTCGGTCGGG	miRNA	miR-4651
t0004057	20	79	AGTAGCATTGTACAGGGCTA	miRNA	miR-4289
t0004050	21	79	TGGGGGGCAGAGAGCGAGACT	miRNA	miR-423-5p
t0004034	19	79	AGAGGTAGGTTGCATAGTT	miRNA	miR-352
t0004064	26	79	TAATACTGCCGGGTAATGATGGAATC	miRNA	miR-200c
t0004044	21	79	TCTGACCTATGAATTGACAGC	miRNA	miR-192
t0004049	20	79	GAGAGAAAGGCAGTTCCTGA	miRNA	miR-185
t0004043	21	79	TGGAGAGATAGGCAGTTCCTG	miRNA	miR-185
t0004061	24	79	ATAGCAGCACGTAAATATTGGCGT	miRNA	miR-16
t0004067	20	79	TAGCAGCACGTAAATATTTG	miRNA	miR-16
t0004094	23	79	TAGCAGCACATCATGGTTTAAAA	miRNA	miR-15b
t0004132	24	79	TACCACAGGGTAGAAACACGGACA	miRNA	miR-140
t0004089	21	79	ACCACAGCGTAGAACCACGGA	miRNA	miR-140
t0004113	19	79	TGAGTTAGTAGTTTGTGCT	miRNA	let-7i
t0004079	20	79	TGAGGTAGTAGATTGGATAG	miRNA	let-7f
t0004127	21	79	TTAGGTAGTAGGTTGTATAGT	miRNA	let-7
t0004118	22	79	TGCGGTAGTAGGTTGTATAGTT	miRNA	let-7
t0004131	21	78	TGTCTTACTCCCTCAGGCACA	miRNA	miR-550a*
t0004123	21	78	TCCTGTACTGAGCTGCCCCAA	miRNA	miR-486-5p
t0004106	28	78	TCCTGTACTGAGCTGCCCCGAGACGGTA	miRNA	miR-486-5p
t0004107	26	78	AAACCGTTACTATTACTGAGTTTAGT	miRNA	miR-451
t0004072	20	78	AAACCGTTATTATTACTGAG	miRNA	miR-451
t0004124	22	78	TGAGGGGCGGAGAGCGAGACTT	miRNA	miR-423-5p
t0004087	23	78	GGAGGAACCTTGGAGCTTCGGCA	miRNA	miR-3928
t0004098	25	78	AAAAGCTGGGTTGAGAGGGCGATAT	miRNA	miR-320a
t0004116	24	78	AAAAGCTGGGTTGAGAGGGCGATG	miRNA	miR-320a
t0004080	22	78	AAAAGCTGCGTTGAGAGGGCGA	miRNA	miR-320a
t0004122	19	78	AAGTAATCCAGGATAGGCT	miRNA	miR-26a
t0004121	25	78	TAGGTAGTTTCATGTTGTTGGGATC	miRNA	miR-196b
t0004128	24	78	TACCACAGGGTAGAACCATGGACA	miRNA	miR-140
t0004158	21	78	ACCACAGGGTAGATCCACGGA	miRNA	miR-140
t0004179	22	78	TACCACAGGGAAGAACCACGGA	miRNA	miR-140
t0004186	23	78	TGAGGTAGTAGATTGTATAGTGT	miRNA	let-7f
t0004164	21	78	TGAGGTAGTAGGATGTGTGGT	miRNA	let-7b
t0004165	26	77	AAACCGTTACCATTACTGAGTTTAAA	miRNA	miR-451
t0004194	22	77	AAACCATTACCATTACTGAGTT	miRNA	miR-451
t0004155	18	77	AACTTTGAAGACTGAAGT	miRNA	miR-3526
t0004145	21	77	CGGAGAGAAAGGCAGTTCCTG	miRNA	miR-185
t0004148	22	77	TGAGGGAGGAGATTGTATAGTT	miRNA	miR-1827
t0004175	20	77	AAGCGGCGATGGCGGAGCTG	miRNA	miR-1636
t0004163	20	77	TAGCAGCACATAATGCTTTG	miRNA	miR-15a
t0004157	22	77	TAACACAGGGTAGAACCACGGA	miRNA	miR-140
t0004139	22	77	TACCGCAGGGTAGAACCACGGA	miRNA	miR-140
t0004144	20	77	TGAGATAGTAGATTGTATAG	miRNA	let-7f
t0004188	22	77	TGAGGTAGTAGGTTATATAGTT	miRNA	let-7
t0004141	20	76	CTATACAACTTACTACTTTC	miRNA	miR-98*
t0004153	21	76	GGTGGAAGGTTGTAGGCATGT	miRNA	miR-965-5p
t0004197	23	76	AAAGCTGGGTTGAGAGGGCGTTT	miRNA	miR-320d
t0004223	24	76	AAAAGCTGGGTTGAGAGGGCGACA	miRNA	miR-320a
t0004233	23	76	AAAAGCTGGGTTTAGAGGGCGAT	miRNA	miR-320a
t0004225	21	76	TGACTGGGGCGGGACATCTGT	miRNA	miR-219-2-3p
t0004206	20	76	TAGCAGCACGTAAATATCGG	miRNA	miR-16
t0004204	21	76	ACCACAGGGTGGAACCACGGA	miRNA	miR-140
t0004219	23	76	TACCACAGGGCAGAACCACGGAC	miRNA	miR-140
t0004241	22	76	AGCAGCATTGTACAGGGCTAAA	miRNA	miR-107
t0004244	20	76	ACAGTACTGTGATAACTGAA	miRNA	miR-101c

t0004201	22	76	TGAGGCAGTAGTTTGTACAGTT	miRNA	let-7g
t0004247	20	76	GAGGTAGTAGGTTGCATAGT	miRNA	let-7d
t0004273	21	76	TGCGGTAGTAGGTTGTATAGT	miRNA	let-7
t0004255	20	76	TGAGGTGGTAGGTTGTATAG	miRNA	let-7
t0004302	22	76	TAAGGTAGTAGGTTGTATAGTT	miRNA	let-7
t0004286	19	75	AATGGCGCCACTAGGGTTG	miRNA	miR-652
t0004293	22	75	TCCTGTACTAAGCTGCCCCGAG	miRNA	miR-486-5p
t0004284	23	75	AAACAGTTACCATTACTGAGTTT	miRNA	miR-451
t0004294	23	75	AAACCGTTACCATTACTGATTTT	miRNA	miR-451
t0004272	21	75	TGAGGGGCAGAGAGCGTGACT	miRNA	miR-423-5p
t0004274	22	75	TGAGGAGCAGAGAGCGAGACTT	miRNA	miR-423-5p
t0004287	24	75	AAAAGCTGGGTTGAGAGGGCATAA	miRNA	miR-320d
t0004278	24	75	AAAAGCTGGGTTGAGAGGGCGATC	miRNA	miR-320a
t0004276	25	75	AAAAGCTGGGTTGAGAGGGCGAAAG	miRNA	miR-320a
t0004283	23	75	TGGAAGGTAGACGGCCAGAGAGA	miRNA	miR-3190
t0004280	21	75	CATTGCACTTGTCTCGGTCTA	miRNA	miR-25
t0004291	24	75	CTGACCTATGAATTGACAGCCATC	miRNA	miR-192
t0004296	22	75	TAGCAGCACGTAAATAATGGCG	miRNA	miR-16
t0004330	23	75	CAGTGCAATGATGAAAGGGCATA	miRNA	miR-130b
t0004309	21	75	AGCAGCATTGTACAGGGCATT	miRNA	miR-107b
t0004329	26	75	ACCCTGTAGATCCGAATTTGTGAATC	miRNA	miR-10-5p
t0004358	22	75	GCAGCATTGTACAGGGCTATGA	miRNA	miR-103a
t0004323	20	75	TGATGTAGTAGATTGTATAG	miRNA	let-7f
t0004359	21	75	AGAGGTAGTAGGGTGCATAGT	miRNA	let-7d
t0004355	21	75	TGAGGTAGTAGGTTGTGAGGT	miRNA	let-7b
t0004334	21	75	TGAGGTAGTAGGTCGTGTGGT	miRNA	let-7b
t0004348	20	74	ATTCTAATTTCTCCACGTCT	miRNA	miR-576-5p
t0004317	22	74	TATCCACACAAACACACAGGCT	miRNA	miR-467f
t0004332	22	74	GAATGTTGCTCGGTGAACCCCT	miRNA	miR-409-3p
t0004360	24	74	TTCAAGTAATCCAGGATAGGCTAT	miRNA	miR-26a
t0004345	20	74	AACTGGCCTACAAAGTCCCA	miRNA	miR-193
t0004336	24	74	CAACGGAATCTCAAAAGCAGCTGA	miRNA	miR-191
t0004343	26	74	CAAAGAATTCTCCTTTTGGGCTTATC	miRNA	miR-186
t0004315	19	74	AAGGTAGATAGAACAGGTC	miRNA	miR-1839
t0004331	21	74	AACCACTGACCGTTGACTGTA	miRNA	miR-181a-2*
t0004353	20	74	ATCCCACCGCTGCCACCAAG	miRNA	miR-1260b
t0004342	22	74	AAGCAGCATTGTACAGGGCTAT	miRNA	miR-103a
t0004313	21	74	TACATTACTGTGATAACTGAA	miRNA	miR-101
t0004340	22	74	TTTGAGGTAGTAGATTGTATAG	miRNA	let-7f
t0004389	22	74	TGATGTAGTAGGTTGTGTGGTA	miRNA	let-7b
t0004375	22	74	TGAGGTAGTTGGTTGTATAGTT	miRNA	let-7
t0004413	23	73	GGGAGCCAGGAAGTATTGATGTT	miRNA	miR-505*
t0004374	28	73	TCCTGTACTGAGCTGCCCCGAGACGGCT	miRNA	miR-486-5p
t0004376	29	73	TCCTGTACTGAGCTGCCCCGAGACGGCA(	miRNA	miR-486-5p
t0004399	25	73	CGGCGGCGGCGACTCTGGACGCGAG	miRNA	miR-3885-5p
t0004406	22	73	ACAAAAAAAAAAGCCCAACCCT	miRNA	miR-3613-3p
t0004383	22	73	ACCTGGCATACAATGTAGATTT	miRNA	miR-221*
t0004377	21	73	TGGGGAGAAAGGCAGTTCCTG	miRNA	miR-185
t0004392	22	73	AACATTCAATGCTGTCGGTGAG	miRNA	miR-181a
t0004396	24	73	TAGCAGCACGTAAATATTGGCAAA	miRNA	miR-16b
t0004371	20	73	TAGTAGCACATCATGGTTTA	miRNA	miR-15b
t0004366	21	73	TAGCAGCACATAATGGTTTTT	miRNA	miR-15a
t0004381	22	73	TACCACAGGGTAGAACGACGGA	miRNA	miR-140
t0004409	23	73	TGAGGTAGTAGATTGTTCAGAGT	miRNA	let-7j
t0004454	20	73	AGAGGGAGTAGGTTGCATAG	miRNA	let-7d
t0004414	21	73	TGAGGTAATAGGTTGTGTGGT	miRNA	let-7b
t0004451	21	73	TGAGGTAGTAGGTGGTGTGGT	miRNA	let-7b
t0004453	22	72	AGGTGGAAGGTTGTAGGCATGT	miRNA	miR-965-5p

t0004460	23	72	GTAGAGGAGATGGCGCAGGGGAT	miRNA	miR-877
t0004448	23	72	AAGGTTACTTGTTAGTTCAGATC	miRNA	miR-872
t0004457	25	72	AGGGCGCGCGGGTCGGGGCGGCGGT	miRNA	miR-638
t0004462	23	72	TGTAGAGCAGGGAGCAGGAAGTA	miRNA	miR-4732-5p
t0004422	20	72	TTTGGAGGAGATGCGGTTAT	miRNA	miR-4443
t0004459	21	72	TGAGGGGCAGAGAGCGACACT	miRNA	miR-423-5p
t0004426	23	72	AAAAGCTGTGTTGAGAGGGCGAT	miRNA	miR-320a
t0004418	21	72	AGCTTTTGGGAATTCAGGTAT	miRNA	miR-3140-3p
t0004425	24	72	TAGCTTATCAGACTGATGTTGACA	miRNA	miR-21
t0004471	25	72	TGGAGAGAAAGGCAGTTCCTGAATC	miRNA	miR-185
t0004444	25	72	TAGCAGCACGTAAATATTGGCGATC	miRNA	miR-16
t0004449	22	72	TAGCAGCACGAAAATATTGGCG	miRNA	miR-16
t0004514	26	72	CTAGACTGAGGCTCCTTGAGGAAATC	miRNA	miR-151-3p
t0004539	22	72	TACCACAGGGTAGAACCGCGGA	miRNA	miR-140
t0004542	22	72	TACCACAGGGTGGAACCACGGA	miRNA	miR-140
t0004502	23	72	AGCAGCATTCTACAGGGCTATGA	miRNA	miR-103a
t0004527	22	72	TGAGGTAGTAGTTTGTAGAGTT	miRNA	let-7g
t0004530	20	72	TGAGGTAGTAGATTGTAAAG	miRNA	let-7f
t0004544	20	72	TGAGGTAGTAGGTTTGTGGT	miRNA	let-7a
t0004493	21	72	TGAGGTAGTTGGTTGTATAGT	miRNA	let-7
t0004518	26	71	TCAATTCCGTAGTGCATTGCAGAATC	miRNA	miR-932-5p
t0004482	22	71	AGAGGAGATGGCGCAGGGGACT	miRNA	miR-877
t0004524	22	71	TCCTGCACTGAGCTGCCCCGAG	miRNA	miR-486-5p
t0004504	22	71	TCCTGTACTGAGCTGCCCCGGG	miRNA	miR-486-5p
t0004509	23	71	TTCTGTACTGAGCTGCCCCGAGA	miRNA	miR-486-5p
t0004503	21	71	ATCCTGTACTGAGCTGCCCCG	miRNA	miR-486-5p
t0004506	21	71	AAACCGTTACCACTACTGAGT	miRNA	miR-451
t0004538	23	71	TGAGGCGCAGAGAGCGAGACTTT	miRNA	miR-423-5p
t0004523	28	71	TTGAAGACTGAAGTGGAGAAGGGTTTCT	miRNA	miR-3526
t0004488	22	71	AAAAGCTGGGTTGAGAGGGCTA	miRNA	miR-320d
t0004511	21	71	AAAAGCTGGGTTGAGATGGCG	miRNA	miR-320a
t0004507	23	71	TGTAAACATCCTACACACTCAGC	miRNA	miR-30c
t0004505	22	71	ATGGAGAGAAAGGCAGTTCCTG	miRNA	miR-185
t0004561	20	71	TAGCAGAACGTAAATATTGG	miRNA	miR-16
t0004565	20	71	CATAAAGTAGAAAGCACTAA	miRNA	miR-142-5p
t0004549	22	71	ATAAAGTAGAAAGCACTACTAA	miRNA	miR-142-5p
t0004590	20	71	GTAGTGTTTCCTACTTTATG	miRNA	miR-142-3p
t0004596	20	71	CCTATAAAGTAGAAAGCACT	miRNA	miR-142
t0004598	23	71	CACCACAGGGTAGAACCACGGAC	miRNA	miR-140
t0004567	23	71	TGAGGTAGTAGTTTGCGCTGTTT	miRNA	let-7i
t0004573	22	71	TGAGCTAGTAGTTTGTACAGTT	miRNA	let-7g
t0004594	21	71	TGAGGTAGTAGAGCGTATAGT	miRNA	let-7f
t0004599	21	71	TAGGTAGTAGGTTGTGTGGTT	miRNA	let-7b
t0004569	22	71	TGAGGTAGTAGGATGTATAGTT	miRNA	let-7
t0004574	23	70	TATTCATTTATCCCCTCCCTACA	miRNA	miR-664
t0004566	20	70	TAGCAGCGGGAACAGTTCTA	miRNA	miR-503
t0004578	24	70	AAACCGTTACCATTACTGAGTTAA	miRNA	miR-451
t0004558	19	70	AAATTGTTACCATTACTGA	miRNA	miR-451
t0004562	25	70	AAACCGTTACCATTACTGAGTTTAA	miRNA	miR-451
t0004597	22	70	GATGAGGATGGATAGCAAGGAA	miRNA	miR-3605-5p
t0004580	22	70	AAAAGCTGGGTTGAGATGGCGT	miRNA	miR-320d
t0004583	22	70	CATTGCACTTGTCTTGGTCTGA	miRNA	miR-25
t0004545	19	70	ACCTGGCATACAATGTAGA	miRNA	miR-221*
t0004577	25	70	CTGACCTATGAATTGACAGCCAGTG	miRNA	miR-192
t0004610	20	70	TGGAGAGAAAGGCAGTTCCA	miRNA	miR-185
t0004581	21	70	TGGAGAGAAAGACAGTTCCTG	miRNA	miR-185
t0004605	23	70	TTTGGCAATGGTAGAACTCACAC	miRNA	miR-182
t0004657	21	70	ATAGCAGCACATCATGGTTTA	miRNA	miR-15b

t0004624	23	70	ACCACAGGGTAGAACCACGGCCA	miRNA	miR-140
t0004621	22	70	ACCACAGGGGAGAACCACGGAA	miRNA	miR-140
t0004635	21	70	ACCACAGAGTAGAACCACGGA	miRNA	miR-140
t0004675	24	70	ACCACAGGGTAGAACCACGGACGA	miRNA	miR-140
t0004612	23	70	TGCTGGATCAGTGGTTCGAGTCA	miRNA	miR-1287
t0004685	20	70	ATCCCACCGCTGCCACCATT	miRNA	miR-1260b
t0004613	21	70	CTTCCCCACCCTCTCCTGCAG	miRNA	miR-1224-3p
t0004627	21	70	TGAGGTAGTAGTTTGTACATT	miRNA	let-7g
t0004680	19	70	TGAGGTAGTAGATTGTATG	miRNA	let-7f
t0004647	21	70	GGAGGTAGTAGATTGTATAGT	miRNA	let-7f
t0004688	22	70	TAGGTAGTAGGTTGTGTGGTTA	miRNA	let-7b
t0004668	22	70	TCAGGTAGTAGGTTGTGTGGTT	miRNA	let-7b
t0004658	22	69	TATTGCACTTGTCCCGGCCTAT	miRNA	miR-92a
t0004619	18	69	TATTGCACTTGTCCCGGC	miRNA	miR-92a
t0004636	21	69	TCCTGTACTGAGCTTCCCCGA	miRNA	miR-486-5p
t0004642	23	69	TCCTGTACTGAGCTGCCCAGAGA	miRNA	miR-486-5p
t0004689	20	69	TGGGAAGAGGAGCTGAGGGA	miRNA	miR-4646-5p
t0004643	23	69	AAACCGTTACCGTTACTGAGTTT	miRNA	miR-451
t0004670	21	69	TGAGGGGCAGGGAGCGAGACT	miRNA	miR-423-5p
t0004676	22	69	TGAGGGGTAGAGAGCGAGACTT	miRNA	miR-423-5p
t0004653	23	69	AACTTTGAAGGCCGAAGTGGAGA	miRNA	miR-3526
t0004666	22	69	TTCAAGTAATCCAGGATAGCCT	miRNA	miR-26a
t0004686	23	69	ACAACGGAATCCCAAAAGCAGCT	miRNA	miR-191
t0004622	23	69	TGGAGAGAAAGGCAGTTCCTGCG	miRNA	miR-185
t0004633	22	69	ACATTCAACGCTGTCGGTGAGT	miRNA	miR-181a
t0004759	20	69	CCAATATTACTGTGCTGCTT	miRNA	miR-16-2*
t0004767	20	69	TAGCAGCACATCATCGTTTA	miRNA	miR-15b
t0004698	23	69	CCCATAAAGTAGAAAGCACTAGA	miRNA	miR-142
t0004701	21	69	ACCACAGGGTAGAACCACTGA	miRNA	miR-140
t0004743	24	69	TACCACAGGGTACAACCACGGACG	miRNA	miR-140
t0004695	22	69	AGCAGCGTTGTACAGGGCTATG	miRNA	miR-103a
t0004717	20	69	TACATTACTGTGATAACTGA	miRNA	miR-101
t0004752	23	69	TGGAATGTAAGGAAGTGTGTATC	miRNA	miR-1
t0004714	23	69	TGAGGTAGTAGATTGCATAGTTA	miRNA	let-7f
t0004713	23	68	CAAAGTGCTTTTCGTGCAGGTAG	miRNA	miR-93
t0004697	22	68	TATTGCATTTGTCCCGGTCTGT	miRNA	miR-92a
t0004721	22	68	TATTGCACTTGTCCCGGGTTGT	miRNA	miR-92a
t0004763	19	68	AAGACTGAAGTGGAGAAGG	miRNA	miR-739
t0004762	21	68	AATGGCGCCACTAGGGTTGTG	miRNA	miR-652
t0004757	23	68	TGTAGAGCAGGGAGCAGGAAGCG	miRNA	miR-4732-5p
t0004772	21	68	AAATCGTTACCATTACTGAGT	miRNA	miR-451
t0004707	23	68	TTAGGGGCAGAGAGCGAGACTTT	miRNA	miR-423-5p
t0004742	21	68	ATTGCACGGTATCCATCTGTA	miRNA	miR-363
t0004709	22	68	TATTGCACATTACTAAGTTGCA	miRNA	miR-32
t0004724	19	68	GTTGGGACAAGAGGACGGT	miRNA	miR-3122
t0004761	23	68	CGGGGCAGCTCAGTACAGGAAGA	miRNA	miR-3107*
t0004719	23	68	TAGCACCATCTGAAATCGGTTAA	miRNA	miR-29c
t0004799	25	68	AATGGCACTGGAAGAATTCACGATC	miRNA	miR-263a-5p
t0004779	21	68	CATTGCACTTGTCTTGGTCTG	miRNA	miR-25
t0004847	21	68	TGGGTCTTTGCGGGCGAGATG	miRNA	miR-193a-5p
t0004838	23	68	TGGACGGAGAACTGATAAGGGCA	miRNA	miR-184-3p
t0004788	22	68	TAGCAGCACATAATGGTTTGGG	miRNA	miR-15a
t0004808	22	68	GATGAAGCACTGTAGCTCTATC	miRNA	miR-143
t0004804	23	68	TACCACAGGGTAGAACCACAGAC	miRNA	miR-140
t0004802	24	68	TGGAATGTAAAGAAGTATGTAATC	miRNA	miR-1-3p
t0004820	22	68	TTACAGTACTGTGATAACTGAA	miRNA	miR-101
t0004826	25	68	ATCCTGTAGATCCGAATTTGTGATC	miRNA	miR-10
t0004781	23	68	TGAGGTGGTAGTTTGTGCTGTTA	miRNA	let-7i

t0004794	22	68	TGAGGTAGTAGTTTGTACCGTT	miRNA	let-7g
t0004809	21	68	TGAGGTAGTAGATTGTATTTT	miRNA	let-7f-5p
t0004786	20	68	TCAGGTAGTAGATTGTATAG	miRNA	let-7f
t0004821	22	68	TGAGGTATTAGGTTGTGTGGTA	miRNA	let-7b
t0004818	21	68	TGAGGTAGTAGGTTTGTGGTT	miRNA	let-7a
t0004848	21	68	TGAGGTAGTAGGTCGTATAGT	miRNA	let-7
t0004813	21	68	TGAGGTAGTAGGTTGTATCGT	miRNA	let-7
t0004834	23	67	TATTGCACTCGTCCCGGCCAAGA	miRNA	miR-92
t0004793	22	67	TCCTGTACTCAGCTGCCCCGAG	miRNA	miR-486-5p
t0004777	21	67	TCCTGTACTGAGCTGTTCCGA	miRNA	miR-486-5p
t0004861	21	67	AAACCGTTACCATTACTGTGT	miRNA	miR-451
t0004792	22	67	AAACCGTTACCGTTACTTAGTT	miRNA	miR-451
t0004822	23	67	AAACCGTTAACATTACTGAGTTT	miRNA	miR-451
t0004796	21	67	AAAACGTTACCATTACTGAGT	miRNA	miR-451
t0004844	22	67	ACTCCCACTGCTTCACTTGACT	miRNA	miR-4301
t0004806	20	67	AATTGCACGGTATCCATCTT	miRNA	miR-363
t0004837	26	67	TGAAGACTGAAGTGGAGAAGGCTCCA	miRNA	miR-3526
t0004833	22	67	AAAAGCTGAGTTGAGAGGGCGA	miRNA	miR-320a
t0004778	21	67	ATATTGCACATTACTAAGTTG	miRNA	miR-32
t0004840	23	67	TTCAAGTAATTCAGGATAGGTTA	miRNA	miR-26b
t0004869	22	67	CATTGCTCTTGTCTCGGTCTGA	miRNA	miR-25
t0004895	24	67	TGACTGGGGCGGTACATCTGTTAT	miRNA	miR-219-2-3p
t0004902	25	67	TAAAGTGCTTATAGTGCAGGTAGAA	miRNA	miR-20
t0004878	22	67	CAAAGTGCTTACAGTGCAGCTA	miRNA	miR-17
t0004894	25	67	ATAGCAGCACGTAAATATTGGCGAA	miRNA	miR-16
t0004899	21	67	TCCAGCACGTAAATATTGGCG	miRNA	miR-16
t0004920	21	67	TAGCAGCACGTAAATATTGTC	miRNA	miR-16
t0004932	22	67	TATAGCAGCACATAATGGTTTG	miRNA	miR-15a
t0004876	22	67	TAGCAGCACATAATGGTTTCTG	miRNA	miR-15a
t0004956	19	67	CACAGGGTAGAACCACGGA	miRNA	miR-140
t0004914	21	67	ACCGCAGGGTAGAACCACGGA	miRNA	miR-140
t0004862	24	67	TAAAGTGCTGACAGTGCAGATAGT	miRNA	miR-106b
t0004943	25	67	AAGCAGCATTGTACAGGGCTATGAA	miRNA	miR-103a
t0004867	22	67	TGAGGTATTAGTTTGTACAGTT	miRNA	let-7g
t0004964	22	67	TGAGGTAGTAGCTTGTACAGTT	miRNA	let-7g
t0004903	23	67	AGAGGTAGTAGGTTGCATAGTCA	miRNA	let-7d
t0004951	24	67	CTGAGGTAGTAGGTTGTGTGGTTA	miRNA	let-7b
t0004874	22	67	TGAGGTATTAGGTTGTATAGTT	miRNA	let-7
t0004879	21	66	TTATGGTTTGCCTGGGACTGT	miRNA	miR-584
t0004906	21	66	TCCTGTACTGAGCTGCCGAGA	miRNA	miR-486-5p
t0004917	23	66	TGAGGGGCTGAGAGCGAGACTTT	miRNA	miR-423-5p
t0004959	23	66	ACTTGACCTGGAGTCAGAAGGCA	miRNA	miR-378
t0004923	21	66	TAGCATTATCTGAAATCGGTT	miRNA	miR-29e
t0004957	22	66	TCAAGTAATTCAGGATAGGTTG	miRNA	miR-26b
t0004868	24	66	CAAAGTGCTCATAGTGCAGGTAGA	miRNA	miR-20b
t0004904	24	66	TGACCTATGAATTGACAGCCAGTT	miRNA	miR-192
t0004939	20	66	TGGAGAAAGGCAGTTCCTGA	miRNA	miR-185
t0004941	22	66	TGGACGGAGAACTGATAAGATC	miRNA	miR-184-3p
t0004924	20	66	AACCACTGACCGTTGACTGT	miRNA	miR-181a-2*
t0004919	22	66	TAGCAGCACGTAAATATTGGAA	miRNA	miR-16b
t0004916	23	66	TAGCAGCACGTAAATATTGGCCA	miRNA	miR-16b
t0005011	20	66	TACCAGCACGTAAATATTGG	miRNA	miR-16
t0005006	22	66	TAGCAGCACATAATGGTTTGTT	miRNA	miR-15a
t0005055	22	66	TACCACAGGCTAGAACCACGGA	miRNA	miR-140
t0005008	22	66	TACCACCGGGTAGAACCACGGA	miRNA	miR-140
t0004974	24	66	AAAAGTGCTTACAGTGCAGGTAGA	miRNA	miR-106
t0004975	23	66	AGCAGCATTGTACAGGCCTATGA	miRNA	miR-103a
t0005040	24	66	AGCAGCATTGTACAGGGCTATGGT	miRNA	miR-103a

t0005057	18	66	TGAGGTAGTAGATTGTTT	miRNA	let-7f
t0004970	20	66	AGAGAGTAGGTTGCATAGTT	miRNA	let-7d
t0005038	28	66	TGAGGTAGTAGGTTGTGTGGTTTTTATC	miRNA	let-7b
t0005007	25	66	TATGAGGTAGTAGGTTGTGTGGTTA	miRNA	let-7b
t0005012	22	66	TGAGGTAGTAGGTTGTATAGAA	miRNA	let-7-5p
t0005052	24	66	TGAGGTAGTAGGTTGTATAGTTGA	miRNA	let-7
t0004982	24	65	TATTGCACTTGTCCCGGCCTGTAG	miRNA	miR-92a
t0004983	21	65	TATTGCACTTGTTCCGGCCTG	miRNA	miR-92a
t0004981	18	65	TTGTACTGAGCTGCCCCG	miRNA	miR-486-5p
t0004997	21	65	AAACCATTACCATTACTGAGT	miRNA	miR-451
t0005002	23	65	AAAGCTGGGTTGAGAGGGCGTTA	miRNA	miR-320d
t0005009	22	65	AAAAGCTGGGTTGAGAGTGCGA	miRNA	miR-320a
t0005010	22	65	TGACTAGATATACACTCATCCA	miRNA	miR-279
t0005046	19	65	TGAGGTAGGTTGTGTGGTA	miRNA	miR-1961
t0004988	23	65	TTGAGAGAAAGGCAGTTCCTGAA	miRNA	miR-185
t0005024	20	65	CAAAGTGCTTACAGTGCAGG	miRNA	miR-17
t0005045	20	65	TAGCAGCACATCATGGTTAC	miRNA	miR-15b
t0005042	18	65	AGCAGCACATCATGGTTT	miRNA	miR-15b
t0005015	24	65	AAAGCAGCATTGTACAGGGCTATT	miRNA	miR-107
t0005072	20	65	AAAAGTGCTTACAGTGCAGG	miRNA	miR-106
t0005108	20	65	AGCAGCATTGTACACGGCTA	miRNA	miR-103a
t0005116	22	65	AGCAGCATTGTACAGGTCTATG	miRNA	miR-103a
t0005070	22	65	TACAGTACTGTTATAACTGAAG	miRNA	miR-101
t0005112	21	65	TGAGGTAGTAGATTGTAGTTT	miRNA	let-7f-5p
t0005081	20	65	TGAGGTAGTAGATTCTATAG	miRNA	let-7f
t0005061	21	65	TGAGGTAGTAGGTTGTATAGG	miRNA	let-7
t0005103	22	65	TGAGGTAGAAGGTTGTATAGTT	miRNA	let-7
t0005121	20	65	TGAGGCAGTAGGTTGTATAG	miRNA	let-7
t0005115	20	64	TATTGCACTCGTCCCGGCAT	miRNA	miR-92
t0005059	23	64	AAGGGATTCTGATGTTGGTCATC	miRNA	miR-541
t0005137	22	64	AAACCGTTACCATTACTGAGAT	miRNA	miR-451
t0005102	22	64	AAACCGTTACAATTACTGAGTT	miRNA	miR-451
t0005114	20	64	AAACCGTTACCGTTACTGAG	miRNA	miR-451
t0005078	25	64	AACCGTTACCATTACTGAGTTTAGT	miRNA	miR-451
t0005101	20	64	AAACCGTTACTATTACTGAG	miRNA	miR-451
t0005141	22	64	CTTATCAGATTGTATTGTAATT	miRNA	miR-374a*
t0005092	23	64	AAAAGCTGTGTTGAGAGGGCGTT	miRNA	miR-320d
t0005099	22	64	AAAAGCTGGGTTGAGCGGGCGA	miRNA	miR-320a
t0005068	22	64	TGTAAACATCTCCGACTGGAAG	miRNA	miR-30d
t0005120	23	64	ATCACATTGCCAGGGATTTCCAT	miRNA	miR-23a
t0005125	20	64	ACCTGGCATACAATGTAGAT	miRNA	miR-221*
t0005182	26	64	TAAAGTGCTTATAGTGCAGGTAGATC	miRNA	miR-20
t0005245	23	64	TGTGCAAATCCATGCAAAACTGC	miRNA	miR-19b
t0005150	23	64	TGGAGAGAAAGGCAGTTCCTCAA	miRNA	miR-185
t0005167	21	64	TGGAGAGAAAGGCTGTTCCTG	miRNA	miR-185
t0005192	21	64	TGGACGGAGAACTGATAAGGG	miRNA	miR-184
t0005221	19	64	AAAGGTAGATAGAACAGGT	miRNA	miR-1839
t0005240	23	64	TGAGGGAGTAGATTGTATAGTTA	miRNA	miR-1827
t0005249	26	64	GAGTTAGCAGCACATAATGGTTTGTG	miRNA	miR-15a
t0005193	19	64	TCAGTGCACTACAGAACTT	miRNA	miR-148a
t0005235	21	64	GCCACAGGGTAGAACCACGGA	miRNA	miR-140
t0005171	21	64	AACCACAGGGTAGAACCACGG	miRNA	miR-140
t0005208	24	64	TACCACAGGGTAGAACCACGGTCA	miRNA	miR-140
t0005190	22	64	TACCTCAGGGTAGAACCACGGA	miRNA	miR-140
t0005212	23	64	TACCACATGGTAGAACCACGGAA	miRNA	miR-140
t0005196	24	64	AAGCAGCATTGTACAGGGCTATAA	miRNA	miR-107
t0005174	21	64	AAAAGTGCTTACATTGCAGGT	miRNA	miR-106
t0005216	22	64	AGCAGCATTGTACAGGGATATG	miRNA	miR-103a

t0005219	22	64	TGAGGTAGTACGTTGTGTGGTA	miRNA	let-7b
t0005243	25	64	TGAGGTAGTATGTTGTTTGGTTATC	miRNA	let-7b
t0005187	21	64	TGAGGTAATAGGTTGTATAGT	miRNA	let-7
t0005179	20	63	AGAAGCATTGTACAGGGCTA	miRNA	miR-4289
t0005201	22	63	AAGCTCGGTCTGAGGCCCCTCA	miRNA	miR-423-3p
t0005146	19	63	AGGTAGTAGTTTGTACAGT	miRNA	miR-3962
t0005156	23	63	AAAGGCTGGGTTGAGAGGGCGGA	miRNA	miR-320d
t0005169	21	63	AAAGCTGGTTTGAGAGGGCGA	miRNA	miR-320a
t0005251	22	63	CTTTCAGTCGGATGTTTACAGT	miRNA	miR-30b-3p
t0005252	24	63	CAGTGCAATAGTATTGTCAAAGCA	miRNA	miR-301b-3p
t0005149	19	63	AAGTAATTCAGGATAGGTT	miRNA	miR-26b
t0005191	25	63	CAACGGAATCCCAAAAGCAGCTGTG	miRNA	miR-191
t0005152	19	63	TGAGGGAGTAGATTGTATA	miRNA	miR-1827
t0005180	22	63	TAGCAGCGCATAAATATTGGCG	miRNA	miR-16
t0005237	23	63	TAGCAGCACATAATGGTTTGAAA	miRNA	miR-15a
t0005233	20	63	TAGCAGCACTTAATGGTTTG	miRNA	miR-15a
t0005303	22	63	TGAGAACTGAATTCCATAGGCT	miRNA	miR-146b
t0005305	24	63	TACCACAGGGTAGAATCACGGACG	miRNA	miR-140
t0005318	21	63	AGCAGCATTCTACAGGGCTAT	miRNA	miR-103a
t0005294	23	63	AGCAGCATTGTATAGGGCTATGA	miRNA	miR-103a
t0005324	22	63	GTACAGTACTGTGATAACTGCA	miRNA	miR-101
t0005259	21	63	TGAGTTAGTAGTTTGTACAGT	miRNA	let-7g
t0005302	20	63	TGAGGTAGTAAATTGTATAG	miRNA	let-7f
t0005287	22	63	AGAGGTAGTAGATTGCATAGTT	miRNA	let-7d
t0005255	21	63	CTATACAACCTACTGCCTTCC	miRNA	let-7b*
t0005282	21	63	TGAGGTAGTATGTTGTATAGT	miRNA	let-7
t0005270	21	63	TGAGGTAGAAGGTTGTATAGT	miRNA	let-7
t0005279	22	62	TCTGGGCACAGGCGGATGGACC	miRNA	miR-5107
t0005300	20	62	GGAGAAGGGTCGGGGCGGCT	miRNA	miR-4516
t0005337	21	62	AAACCGTTACCATTACTGAAT	miRNA	miR-451
t0005262	22	62	CAGCAGCAATTCATGTTTTGAA	miRNA	miR-424
t0005334	21	62	TGAGGCGCAGAGAGCGAGACT	miRNA	miR-423-5p
t0005338	21	62	TTAGGGGCAGAGAGCGAGACT	miRNA	miR-423-5p
t0005265	23	62	GAAAAGCTGGGTTGAGAGGGCGT	miRNA	miR-320d
t0005332	23	62	AAAAGCTGGGTTGAGAGGGTGAA	miRNA	miR-320d
t0005264	23	62	AAAAGCTGGGTTGAGAGGGCGTC	miRNA	miR-320d
t0005309	22	62	AAGCTGGGTTGAGAGGGCGAAA	miRNA	miR-320a
t0005335	22	62	TCTGGGAGTTTGTAGCAGTGGA	miRNA	miR-3192
t0005267	18	62	CTTTCAGTCGGATGTTTA	miRNA	miR-30e*
t0005295	23	62	GTAAACATCCTTGACTGGAAGCT	miRNA	miR-30e
t0005377	22	62	TTCAAGTAATCTAGGATAGGCT	miRNA	miR-26a
t0005344	26	62	TAGCTTACCAGACTGATGTTGACATC	miRNA	miR-21
t0005382	18	62	TGGAGAGAAAGGCAGTTT	miRNA	miR-185
t0005355	24	62	TAGCAGCACATAATGGTTTGTGAA	miRNA	miR-15a
t0005392	21	62	ACCACAGGGTAGAACCGCGGA	miRNA	miR-140
t0005423	21	62	ACCACAGGGTAGAACCACGGG	miRNA	miR-140
t0005353	23	62	TACCACAGGGTAGAACCACGGTT	miRNA	miR-140
t0005449	22	62	CAGTGCAATGTTAAAAGGGCAA	miRNA	miR-130a
t0005451	26	62	CTCGTACCGTGAGTAATAATGCGATC	miRNA	miR-126
t0005436	22	62	AGTAGCATTGTACAGGGCTATG	miRNA	miR-103a
t0005359	28	62	TACAGTACTGTGATAACTGAACCAAGCA	miRNA	miR-101
t0005389	22	62	GTACAGTATTGTGATAACTGAA	miRNA	miR-101
t0005409	23	62	TGAGGTAGTAGTTTGTGCTGTAT	miRNA	let-7i
t0005446	24	62	TGAGGTAGTAGATTGTATAGTTAT	miRNA	let-7f
t0005354	23	62	TGAGGTAGTAGATTGTATAGTAG	miRNA	let-7f
t0005399	21	62	TTAGGTAGTAGATTGTATTGT	miRNA	let-7f
t0005459	20	62	TGAGGTAGTCGATTGTATAG	miRNA	let-7f
t0005360	23	62	TGAGGCAGTAGATTGTATAGTTG	miRNA	let-7f

t0005388	21	62	TGAGGTAGTAGGTTGTGTGGC	miRNA	let-7b
t0005450	23	62	TGAGGTACTAGGTTGTGTGGTTA	miRNA	let-7b
t0005396	23	62	TGAGGTAGTAGGTTGTGTGTTAA	miRNA	let-7b
t0005378	22	62	TGAGGTACTAGGTTGTATAGTT	miRNA	let-7
t0005415	22	61	CAAAGTGCTGTTCGTGCAGGTG	miRNA	miR-93
t0005408	22	61	TATTGCACTTGTCCCGGCCTTT	miRNA	miR-92b
t0005438	22	61	TCTCCCACACCCAAGGCTTGCA	miRNA	miR-532-3p
t0005420	25	61	TCCTGTACTGAGCTGCCCCGAGAGA	miRNA	miR-486-5p
t0005356	21	61	AACCGTTACCATTATTGAGTT	miRNA	miR-451
t0005366	21	61	AAACCGTTACCTTTACTGAGT	miRNA	miR-451
t0005370	22	61	AAAACGTTACCATTACTGAGTT	miRNA	miR-451
t0005373	22	61	AAACCGTTACCCTTACTGAGTT	miRNA	miR-451
t0005462	21	61	AAACCGTTATTATTACTGAGT	miRNA	miR-451
t0005425	27	61	AAACCGTTACCATTACTGAGTCGTCGG	miRNA	miR-451
t0005443	22	61	AAACCGTTACCATTAGTGAGTT	miRNA	miR-451
t0005456	22	61	CGAGGGGCAGAGAGCGAGACTT	miRNA	miR-423-5p
t0005432	19	61	AATTGCACGGTATCCATTG	miRNA	miR-363
t0005575	23	61	AAAAGCTGGTTTGAGAGGGCGTA	miRNA	miR-320d
t0005564	25	61	TCTAAAAGCTGGGTTGAGAGGGCGT	miRNA	miR-320d
t0005517	23	61	AAAAGTTGGGTTGAGAGGGCGTA	miRNA	miR-320d
t0005585	19	61	CACCTTGCGCTACTCAGGT	miRNA	miR-3200-3p
t0005466	18	61	TGAGGTAGGTTGTGTGGT	miRNA	miR-1961
t0005467	25	61	GAGTAGCAGCACGTAAATATTGGCG	miRNA	miR-16
t0005480	25	61	TGCAGTGCTTCCTACTTTATGGATC	miRNA	miR-142-3p
t0005494	25	61	TACCACAGGGTAGAACCACGGAAAA	miRNA	miR-140
t0005526	23	61	TACCATAGGGTAGAATCACGGAC	miRNA	miR-140
t0005532	23	61	ACCACAGGGTAGAACCACGGAGT	miRNA	miR-140
t0005555	21	61	TGGAGTGTGACAATGGTGTTT	miRNA	miR-122
t0005495	23	61	AAAAGTGCTTACAGTGCAGGTAT	miRNA	miR-106
t0005496	23	61	TGAGGTAGTAGTTTGTGCTGCTT	miRNA	let-7i
t0005478	22	61	TGAGGTAGTAGTTTGTGCTGTG	miRNA	let-7i
t0005481	21	60	GTAGAGGAGATGGCGCAGGGG	miRNA	miR-877
t0005483	21	60	TGCGGGGCTAGGGCTAACAGC	miRNA	miR-744
t0005513	29	60	CTGGTGCGGACCAGGGGAATCCGACTGT	miRNA	miR-5109
t0005563	22	60	TCCTGTACTGAGCTGCTGCGAG	miRNA	miR-486-5p
t0005541	21	60	CCTGTACTGAGCTGCCCCGAG	miRNA	miR-486-5p
t0005551	22	60	TCCTGTACTGAGCAGCCCCGAG	miRNA	miR-486-5p
t0005497	23	60	TGAGGGGCAGAGAGCGAAACTTT	miRNA	miR-423-5p
t0005546	22	60	TAAGGGGCAGAGAGCGAGACTT	miRNA	miR-423-5p
t0005580	21	60	TGAGGGGCAGCGAGCGAGACT	miRNA	miR-423-5p
t0005535	23	60	AAAAGCTAGGTTGAGAGGGCGTA	miRNA	miR-320d
t0005465	21	60	AAAAGCTGGGTTGAGAGGGAG	miRNA	miR-320d
t0005473	23	60	CACCTTGCGCTACTCAGGTTTGA	miRNA	miR-3200-3p
t0005550	24	60	TAGCTTATCAGACTGATGTTGATT	miRNA	miR-21
t0005608	21	60	TGGAGAGAAAGGCACTTCCTG	miRNA	miR-185
t0005611	24	60	CAAAGTGCTTACAGTGCAGGTAGT	miRNA	miR-17
t0005586	23	60	TACAACAGGGTAGAACCACGGAC	miRNA	miR-140
t0005613	23	60	ACCACAGGGTAGAACTACGGACA	miRNA	miR-140
t0005647	22	60	ACCACAGGGTAGAAACACGGAC	miRNA	miR-140
t0005699	21	60	ACCACACGGTAGAACCACGGA	miRNA	miR-140
t0005638	19	60	ACCAGGGTAGAACCACGGA	miRNA	miR-140
t0005643	24	60	TAAAGTGCTGACAGTGCAGATAGA	miRNA	miR-106b
t0005696	19	60	AGCAGCATTGTACAGGGCA	miRNA	miR-103a
t0005595	20	60	AGCAGCATTGTTCAGGGCTA	miRNA	miR-103a
t0005666	23	60	TGAGGTAGTAGGTTGTGTGGACA	miRNA	let-7b
t0005686	21	60	TGAGGTAGTAGGTTGTGTGAT	miRNA	let-7b
t0005668	21	60	TGAGCTAGTAGGTTGTATAGT	miRNA	let-7
t0005627	20	60	TGAGGTAGTAGGTTGCATAG	miRNA	let-7

t0005654	22	60	TGTGGTAGTAGGTTGTATAGTT	miRNA	let-7
t0005588	21	59	AAAGTGCTGTTCGTGCAGGTA	miRNA	miR-93
t0005604	24	59	GTAGAGGAGATGGCGCAGGGGACT	miRNA	miR-877
t0005612	21	59	TCCTGTACTAAGCTGCCCCGA	miRNA	miR-486-5p
t0005621	18	59	CTGTACTGAGCTGCCCCG	miRNA	miR-486-5p
t0005692	22	59	TCTTGTACTGAGCTCCCCCGAG	miRNA	miR-486-5p
t0005597	23	59	TCCTGTACTGAGCTAGCCCGAGA	miRNA	miR-486-5p
t0005646	23	59	AAACCGTTACCATTCCTGAGTTT	miRNA	miR-451
t0005662	22	59	AACCTTTACCATTACTGAGTTT	miRNA	miR-451
t0005632	23	59	AAACCGTTACCATTACTGACTTT	miRNA	miR-451
t0005671	22	59	AAACCGTTACCATTCCTGAGTT	miRNA	miR-451
t0005598	22	59	AATTTTGGAGGAGATGCGGTTA	miRNA	miR-4443
t0005619	21	59	AGTACAGGGCTATAGCAGCAT	miRNA	miR-4289
t0005640	22	59	CGAGGTAGTAGTTTGTACAGTT	miRNA	miR-3962
t0005782	21	59	ACTGGACTTGGAGTTAGAAGG	miRNA	miR-378
t0005752	22	59	AAAAACTGGGTTGAGAGGGCGA	miRNA	miR-320a
t0005735	22	59	CATTGCACTTGTCTCGGTATGA	miRNA	miR-25
t0005778	22	59	CATTGCTTTTGTCTCGGTCTGA	miRNA	miR-25
t0005805	22	59	CATTGCACTTCTCTCGGTCTGA	miRNA	miR-25
t0005832	21	59	TTAGAGAAAGGCAGTTCCTGA	miRNA	miR-185
t0005759	23	59	AACATTCATTGCTGTCGGTGGGT	miRNA	miR-181b
t0005762	19	59	CAGCATGTAAATATTGGCG	miRNA	miR-16c
t0005763	26	59	GCTGGGGGCGGGGAGCGGTCGGGCGG	miRNA	miR-1607
t0005796	25	59	TAGCAGCACGTAAATATTGGCGGAA	miRNA	miR-16
t0005830	22	59	TAGCAGCACATCATGGTTTAAA	miRNA	miR-15b
t0005788	20	59	TAGCAGCAAATCATGGTTTA	miRNA	miR-15b
t0005705	28	59	TAGCAGCACATCATGGTTTACACCCCAT	miRNA	miR-15b
t0005727	23	59	TACCACAAGGTAGAACCACGGAA	miRNA	miR-140
t0005797	23	59	TACCACAGGGTAGAACCATGGAA	miRNA	miR-140
t0005754	24	59	ACCACAGGGTAGAACCACGGACGG	miRNA	miR-140
t0005783	25	59	TACACTGTAGATCCGAATTTGTATC	miRNA	miR-10a
t0005790	20	59	AGCAGTATTGTACAGGGCTA	miRNA	miR-103a
t0005739	26	59	GTACAGTACTGTGATAACTGACGGGT	miRNA	miR-101c
t0005828	21	59	TACAGTACTGTGATAACTGAG	miRNA	miR-101c
t0005824	21	59	TACAGTACTGTGATGACTGAA	miRNA	miR-101
t0005801	27	59	TACAGTACTGTGATAACTGAAGAGGGA	miRNA	miR-101
t0005829	21	59	GGTAGTAGATTGTATAGTTGA	miRNA	let-7f
t0005731	22	59	TGAGGAGGTAGATTGTATAGTT	miRNA	let-7f
t0005766	22	59	TGAGGTAGTAGATTGTAGAGTT	miRNA	let-7f
t0005827	21	59	TGAGGTAGTAGATCGTATAGA	miRNA	let-7f
t0005702	23	59	TGAGGTAGTAGATTGTATAGATC	miRNA	let-7f
t0005722	20	59	ATAGGTAGTAGGTTGCATAG	miRNA	let-7d
t0005818	26	59	AGAGGTAGTAGGTTGCATAGTTACCG	miRNA	let-7d
t0005808	22	59	TGAGGTAGTACGTTGTATAGTT	miRNA	let-7
t0005770	21	59	TGAGGTAGTAGGTTATATAGT	miRNA	let-7
t0005726	20	58	TATACAACTTACTACTTTCT	miRNA	miR-98*
t0005789	22	58	TATTGCACTTGTCCCGGCCTAA	miRNA	miR-92
t0005807	21	58	GTAGAGGAGATGGCGCAGGAA	miRNA	miR-877
t0005809	24	58	GATCTGTACTGAGCTGCCCCGAGT	miRNA	miR-486-5p
t0005707	22	58	TCTTGTACTGATCTGCCCCGAG	miRNA	miR-486-5p
t0005768	19	58	AAACCGTTACCGTTACTGA	miRNA	miR-451
t0005775	21	58	TGAGGGTCAGAGAGCGAGACT	miRNA	miR-423-5p
t0005815	22	58	TGAGGGGCAGAGAACGAGACTT	miRNA	miR-423-5p
t0005817	24	58	CTGGACTTGGAGTCAGAAGGCTAG	miRNA	miR-378
t0005701	19	58	CCGGGGGTGGGGTCGGCGG	miRNA	miR-3621
t0005795	24	58	CTTGAAGACTGAAGTGGAGAAGGG	miRNA	miR-3526
t0005704	22	58	AAAAGCTTGGTTGAGAGGGCGT	miRNA	miR-320d
t0005730	22	58	AAAAGTTGGGTTGAGAGGGCGT	miRNA	miR-320d

t0005965	20	58	AAACCTGGGTTGAGAGGGCG	miRNA	miR-320a
t0005941	21	58	AGCTTTTGGGAATTCAGGTAG	miRNA	miR-3140-3p
t0005849	22	58	CTGGCTCAGTTCAGCAGGAACA	miRNA	miR-24
t0005862	23	58	AAGCTGCCAGTTGAAGAACTGTA	miRNA	miR-22-3p
t0005898	23	58	AGCTACATTGTCTGCTGGGTTAA	miRNA	miR-221
t0005853	23	58	TAGCAGCACGTAAATATTGCCGT	miRNA	miR-16
t0005935	18	58	TAGCAGCATATCATGGTT	miRNA	miR-15b
t0005959	21	58	TATCAGCACATAATGGTTTGT	miRNA	miR-15a
t0005958	22	58	GTAGTGTTTCCTACTTTATGGT	miRNA	miR-142-3p
t0005906	21	58	ACCACAGGATAGAACCACGGA	miRNA	miR-140
t0005886	23	58	TACCACAAGGTAGAACCACGGAT	miRNA	miR-140
t0005882	18	58	ACAGGGTAGAACCACGGA	miRNA	miR-140
t0005909	24	58	TCGGATCCGTCTGAGCTTGGCATC	miRNA	miR-127
t0005857	18	58	TGGAGTGTGACAATGGTG	miRNA	miR-122
t0005917	20	58	TGAGGTAGTTGATTGTATAG	miRNA	let-7f
t0005842	23	58	TGAGGTAGTAGATTGTATAGTTC	miRNA	let-7f
t0005855	22	58	AGAGGTAGTAGGCTGCATAGTT	miRNA	let-7d
t0005945	22	58	TGATGTAGTAGGTTGTATGGTT	miRNA	let-7c
t0005940	24	58	TGAGGTAGTAGGTTGTGTGGTTTG	miRNA	let-7b
t0005843	21	58	TGAGGTAGAAGGTTGTGTGGT	miRNA	let-7b
t0005921	21	58	TGAGGTATTAGGTTGTATAGT	miRNA	let-7
t0005904	23	57	AGGTTGGGATCGGTTGCAATGCT	miRNA	miR-92a-1*
t0005863	28	57	TCCTGTACTGAGCTGCCCCGAGACGGCG	miRNA	miR-486-5p
t0005880	23	57	ATCCTGTACTGAGCTGCCCCGAG	miRNA	miR-486-5p
t0005949	21	57	AAACCGTTACCATTACTCAGC	miRNA	miR-451
t0005895	22	57	TGAGGGGCAGAGAGCGACACTT	miRNA	miR-423-5p
t0005971	22	57	TGAGGGCCAGAGAGCGAGACTT	miRNA	miR-423-5p
t0005883	23	57	TGAGGGGCAAAGAGCGAGACTTT	miRNA	miR-423-5p
t0005913	24	57	TGAGGGGCAGAGAGCGAGACCTTT	miRNA	miR-423-5p
t0005970	25	57	TGAGGGGCAGAGAGCGAGACTTAGA	miRNA	miR-423-5p
t0005947	20	57	CTGGACTTGGAGTCAGAAGG	miRNA	miR-378
t0005876	27	57	ACTGGACTTGGAGTCAGAAGGCTTATC	miRNA	miR-378
t0005939	21	57	TCAGAATCGTTTGCAATGGCT	miRNA	miR-3495
t0005901	22	57	TTCCACACAGAAATCGCACCCG	miRNA	miR-342-3p
t0006124	25	57	AAAAAAGCTGGGTTGAGAGGGCGTA	miRNA	miR-320d
t0005998	22	57	AAAAGCTGTGTTGAGAGGGCGC	miRNA	miR-320d
t0006088	25	57	AAAAGCTGGGTTGAGAGGGCGATTT	miRNA	miR-320a
t0006073	23	57	AAAACCTGGGTTGAGAGGGCGAA	miRNA	miR-320a
t0005988	23	57	AAAAGTTGGGTTGAGAGGGCGAA	miRNA	miR-320a
t0005992	20	57	CTTTCAGTCGGATGTTTACA	miRNA	miR-30e*
t0006023	25	57	TGACTGGGGCGGTACATCTGTTAAA	miRNA	miR-219-2-3p
t0006074	19	57	ACTGCATTATGAGCACTTA	miRNA	miR-20a*
t0006114	21	57	TGGACAGAAAGGCAGTTCCTG	miRNA	miR-185
t0006083	22	57	ATAGCAGCACATAATGGTTTGT	miRNA	miR-15a
t0006087	20	57	TACAGTATAGATGATGTACA	miRNA	miR-144
t0006103	23	57	CTACAGTATAGATGATGTACTTG	miRNA	miR-144
t0006068	24	57	TACCACAGGGGAGAACCACGGACA	miRNA	miR-140
t0006070	25	57	TACCACAGGGTAGAACCACGGATAT	miRNA	miR-140
t0006115	22	57	ACCACAGGGTAGAACCAAGGAC	miRNA	miR-140
t0006062	22	57	TACCACACGGTAGAACCACGGA	miRNA	miR-140
t0006109	24	57	TCCCTGAGACCCTTTAACCTGATC	miRNA	miR-125a
t0006120	22	57	AGCAGCATTGTACAGGGCTAGA	miRNA	miR-107
t0006130	23	57	AGCAGCATTGTACAGGGCCATGA	miRNA	miR-103a
t0006003	28	57	AGCAGCATTGTACAGGGCTATGACGTCG	miRNA	miR-103a
t0005986	23	57	TGAGGTAGTAGGTTGTGTGGGTT	miRNA	let-7b
t0006131	21	57	TGAGGTCGTAGGTTGTGTGGT	miRNA	let-7b
t0006108	24	57	GTTGAGGTAGTAGGTTGTGTGGTT	miRNA	let-7b
t0005982	23	57	TGAGGTAGTAGGTTGTGTAGTTG	miRNA	let-7b

t0006119	26	57	TGAGGTAGTAGGTTGTGTGGTAAGAA	miRNA	let-7b
t0005999	20	57	TGAGGTAGTAGGTCGTATAG	miRNA	let-7
t0006008	24	56	TATTGCACTCGTCCCGGCCTAAGA	miRNA	miR-92
t0006026	23	56	GTAGAGGAGATGGCGCAGGGGAC	miRNA	miR-877
t0006049	20	56	TAGTACCAGTACCTTGTGTT	miRNA	miR-624*
t0006128	22	56	TAGCAGCGGGAACAGTTCTGAA	miRNA	miR-503
t0006123	23	56	TCCTGTACTGAGCTGCCCCGACG	miRNA	miR-486-5p
t0006028	23	56	TCCTGTACTGAGCTCCCCCGAGA	miRNA	miR-486-5p
t0006091	20	56	AAACCGTTACCATTACTGGG	miRNA	miR-451
t0006014	25	56	AATGACACGATCACTCCCGTTGAGA	miRNA	miR-425
t0006029	22	56	TGAGGGGCAGAGAGGGAGACTT	miRNA	miR-423-5p
t0006039	22	56	AGCACTTTGGGAGGTCAGGTGA	miRNA	miR-3622b-5p
t0006090	28	56	ACTTTGAAGACTGAAGTGGAGAAGGGTT	miRNA	miR-3526
t0006110	21	56	AAAGCTGGGTTTAGAGGGCGT	miRNA	miR-320d
t0005991	26	56	CGCAAAAGCTGGGTTGAGAGGGCGTA	miRNA	miR-320d
t0006259	22	56	AAAAGCTGCGTTGAGAGGGCGT	miRNA	miR-320d
t0006296	24	56	AAAAGCTGGGTTGAGAGGGCAAAA	miRNA	miR-320b
t0006270	23	56	AAAAGCTGGGGTGAGAGGGCGAA	miRNA	miR-320a
t0006183	22	56	AGAGCTTTTGGGAATTCAGGTA	miRNA	miR-3140-3p
t0006218	25	56	TTCAAGTAATCCAGGATAGGCTAGA	miRNA	miR-26a
t0006268	21	56	CATTGCACTTCTCTCGGTCTG	miRNA	miR-25
t0006156	21	56	CATGCACTTGTCTCGGTCTGA	miRNA	miR-25
t0006150	21	56	AGTGAGATTGTTGCATATTTA	miRNA	miR-2162-5p
t0006223	21	56	TTCTTTTTGTCATCCTTCGCC	miRNA	miR-211
t0006222	21	56	TAAGGTGCATCTAGTGCAGTT	miRNA	miR-18b
t0006144	21	56	CAAAGAATTCTCCTTTTGGGA	miRNA	miR-186
t0006175	23	56	TGGAGAGAAAGGCAGTTCCTTAA	miRNA	miR-185
t0006232	22	56	AAGGTAGATAGAACAGGTTTTG	miRNA	miR-1839
t0006271	20	56	AGCAGCACGTAAATATTGGT	miRNA	miR-16
t0006182	22	56	TCGCAGCACGTAAATATTGGCG	miRNA	miR-16
t0006286	19	56	TAGCAGCACGTAAATATGG	miRNA	miR-16
t0006300	28	56	TAGCAGCACATCATGGTTTACATGCCAC	miRNA	miR-15b
t0006267	22	56	TGTCCCAACCCTTGTACCAGTG	miRNA	miR-150
t0006206	24	56	TACCACAGGGTAGAACCACGGTAA	miRNA	miR-140
t0006299	21	56	TGGGAGGCGGAGGTTGCAGTG	miRNA	miR-1273f
t0006251	22	56	AGGCATTGACTTCTCTCTAGCT	miRNA	miR-1256
t0006210	20	56	TAAAGTGCTGACAGTGCATA	miRNA	miR-106b
t0006147	21	56	AGCAGCATTGTATAGGGCTAT	miRNA	miR-103a
t0006266	18	56	GTACAGTACTGTGATAAC	miRNA	miR-101
t0006236	25	56	TGAGGTAGTAGTTTGTGCTGTTAGA	miRNA	let-7i
t0006200	21	56	TGAGGTAGTAGTTTGTGCTGA	miRNA	let-7i
t0006191	22	56	TGGGGTAGTAGTTTGTACAGTT	miRNA	let-7g
t0006214	21	56	TGAGGTAGTAGTTTTTACAGT	miRNA	let-7g
t0006301	25	56	TGAGGTAGTAGATTGTATAGTACGG	miRNA	let-7f
t0006246	25	56	TGAGGTAGTAGGTTGTGTGGTGAGT	miRNA	let-7b
t0006253	20	55	GTGGAAGGTTGTAGGCATGT	miRNA	miR-965-5p
t0006199	22	55	TATTGCACTTGTCCCGGCCTGG	miRNA	miR-92b-3p
t0006167	20	55	AGGGACGGGACGCGGTGTAG	miRNA	miR-92b*
t0006174	23	55	TCCTGTACTGAGCTGCTGCGAGA	miRNA	miR-486-5p
t0006250	21	55	TCCTGTACTGAGCAGCCCCGA	miRNA	miR-486-5p
t0006273	21	55	AAACCGTTACCATTACTGAGC	miRNA	miR-451
t0006240	19	55	AGCACATTGTACAGGGCTA	miRNA	miR-4289
t0006138	22	55	TGAGGGGCAGAGAGCAAGACTT	miRNA	miR-423-5p
t0006185	21	55	AATCCTTGGAACCTAGGTGTG	miRNA	miR-362-5p
t0006148	22	55	AAAAGCTGGGTTGAGAGGGTGT	miRNA	miR-320d
t0006234	23	55	AAAAGCTGGGTTGAGAGGGTATT	miRNA	miR-320d
t0006293	22	55	AAAGCTGGGTTGAGAGGGCAAA	miRNA	miR-320b
t0006302	25	55	CCAAAAAGCTGGGTTGAGAGGGCGA	miRNA	miR-320a

t0006445	22	55	AAAAGCTGGGTTCAGAGGGCGA	miRNA	miR-320a
t0006386	23	55	CATGCTAGGATAGAAAGAATGGG	miRNA	miR-3146
t0006325	23	55	CATTGCACTTGTCTCGGTCTGAG	miRNA	miR-25
t0006391	21	55	CATTGCACTTGTCTCGGTCTT	miRNA	miR-25
t0006419	21	55	AATCACATTGCCAGGGATTTA	miRNA	miR-23a
t0006381	20	55	TTGGGGAAACGGCCGCTGAG	miRNA	miR-2110
t0006306	23	55	CTGACCTATGAATTGACAGCCAT	miRNA	miR-192
t0006362	22	55	CAACGGAATTCCAAAAGCAGCT	miRNA	miR-191
t0006356	24	55	CAACGGAATCCCAAAAGCATCTGA	miRNA	miR-191
t0006341	27	55	TTTGGCAATGGTAGAACTCACACTGGA	miRNA	miR-182
t0006407	20	55	TAGTAGCACATAATGGTTTG	miRNA	miR-15a
t0006346	22	55	TGTAGTGTTTCCTACTTTATGG	miRNA	miR-142-3p
t0006319	22	55	TACCACAGGGTAGAACTATGGA	miRNA	miR-140
t0006394	23	55	AACCACAGGGTAGAACCACGGAC	miRNA	miR-140
t0006444	19	55	CCACAGGGTAGAACCACGG	miRNA	miR-140
t0006309	22	55	ACCACAGGGTAGAACCCCGGAC	miRNA	miR-140
t0006458	24	55	ACCACAGGGTAGAACCACGGATGA	miRNA	miR-140
t0006395	24	55	TACAGTACTGTGATAACTGAAAAA	miRNA	miR-101
t0006401	21	55	TACAGCACTGTGATAACTGAA	miRNA	miR-101
t0006315	22	55	TGAGGTAGTAGTTTCTACAGTT	miRNA	let-7g
t0006404	23	55	TGAGTTAGTAGTTTGTACAGTTA	miRNA	let-7g
t0006452	22	55	AGAGGTAGTAGGTTCCATAGTT	miRNA	let-7d
t0006371	20	55	TGAGGTAGTAGGTTGTGGGG	miRNA	let-7b
t0006331	22	55	TGAGGTAGTAGGTTGTATCGTT	miRNA	let-7
t0006392	22	55	TGAGGTCGTAGGTTGTATAGTT	miRNA	let-7
t0006321	22	55	TGAGGTAGTAAGTTGTATAGTT	miRNA	let-7
t0006383	21	54	CAATCATGTGCAGTGCCAATA	miRNA	miR-96*
t0006408	22	54	TAGAGGAGATGGCGCAGGGGAC	miRNA	miR-877
t0006427	21	54	TCCTGTAATGAGCTGCCCCGA	miRNA	miR-486-5p
t0006437	28	54	TCCTGTACTGAGCTGCCCCGAGACTGCA	miRNA	miR-486-5p
t0006375	21	54	AAGGTATTGTTCAGACTTATG	miRNA	miR-4678
t0006403	27	54	AAACCGTTACCATTACTGAGTACGGGG	miRNA	miR-451
t0006374	20	54	AAACCGTTACCATTACTGCG	miRNA	miR-451
t0006418	22	54	AAACCGTTACCATTACTGAGCT	miRNA	miR-451
t0006436	22	54	AAACCCTTACCATTAGTGAGTT	miRNA	miR-451
t0006422	22	54	AAACCGTTACCATTTCTGAGTT	miRNA	miR-451
t0006380	23	54	AATGATACGATCACTCCCGTTGA	miRNA	miR-425
t0006420	22	54	TGAGGTGCAGAGAGCGAGACTT	miRNA	miR-423-5p
t0006454	22	54	TGGACTCGCAGGAACAGGCCCT	miRNA	miR-4001b-5p
t0006648	22	54	TCGAATCCCACTCCTGACACCA	miRNA	miR-3968
t0006573	23	54	TGAAGACTGAAGTGGAGAAGGGG	miRNA	miR-3526
t0006543	23	54	AAAAGCTGGGTTGAGCGGGCGTT	miRNA	miR-320d
t0006564	23	54	AAAAGCTTGGTTGAGAGGGCGAA	miRNA	miR-320a
t0006561	25	54	AAAAGCTGGGTTGAGAGGGCGAAAT	miRNA	miR-320a
t0006560	23	54	GAATCTGAGAAGGCGCACAAGGT	miRNA	miR-3200-5p
t0006575	20	54	TATTGCACATTACTAAGTTG	miRNA	miR-32
t0006582	22	54	TGTAAACATCCTCGACTGGATC	miRNA	miR-30a
t0006615	19	54	AAGTAATCCAGGATAGGCA	miRNA	miR-26a
t0006640	25	54	TAATCTCAGCTGGAAACTGTGAATC	miRNA	miR-216
t0006670	24	54	CAAAGTGCTCATAGTGCAGGTAGT	miRNA	miR-20b
t0006632	22	54	TGGAGAGATAGGCAGTTCCTGA	miRNA	miR-185
t0006642	23	54	CGAATCATTATTTGCTGCTCTAG	miRNA	miR-15b*
t0006483	20	54	TATAGCAGCACATCATGGTT	miRNA	miR-15b
t0006577	22	54	TATCAGCACATCATGGTTTACA	miRNA	miR-15b
t0006529	21	54	TAGCAGCACATAATGGTTTGG	miRNA	miR-15a
t0006462	20	54	TACAGTATACATGATGTACT	miRNA	miR-144
t0006655	20	54	CTACAGTATAGATGATGTAA	miRNA	miR-144
t0006639	22	54	TACCACAGGGTAGAACTACGGT	miRNA	miR-140

t0006659	22	54	ACCACAGGGTAGAACTACGGAG	miRNA	miR-140
t0006511	23	54	TACCACATGGTAGAACCACGGAT	miRNA	miR-140
t0006550	21	54	ACCACAGGGTAGAAGTACGGA	miRNA	miR-140
t0006486	22	54	CCACAGGGTAGAACCACGGATG	miRNA	miR-140
t0006512	20	54	CTCGGCGTGGCGTCGGTCGT	miRNA	miR-1307
t0006667	23	54	TGCAGCATTGTACAGGGCTATGA	miRNA	miR-103a
t0006624	24	54	AAAGCAGCATTGTACAGGGCTATG	miRNA	miR-103a
t0006464	20	54	AAGCAGCATTGTACAGGGCT	miRNA	miR-103a
t0006516	23	54	GTACAGTACTGTGATAACTGAAG	miRNA	miR-101
t0006471	24	54	AACCCGTAGATCCGAACTTGTATC	miRNA	miR-100
t0006614	22	54	TGAGGTAGTAGTTTGTACGGTT	miRNA	let-7g
t0006638	22	54	TGAGGTAGTGTATTGTATAGTT	miRNA	let-7f-5p
t0006469	20	54	TGAGGTAATAGATTGTATAG	miRNA	let-7f
t0006510	24	54	TGAGGTAGTAGGTTGTGTGGAGAT	miRNA	let-7b
t0006545	22	54	TGAGGTAGTACGTTGTGTGGTT	miRNA	let-7b
t0006567	21	53	ATTGCACTTGTCCCGGCTTGT	miRNA	miR-92c
t0006620	20	53	TCCTGTACTGAGCTGTCCCG	miRNA	miR-486-5p
t0006530	22	53	TCCTGTTCTGAGCTGCCCCGAG	miRNA	miR-486-5p
t0006522	22	53	TCCTGTACTGAGCCGCCCCGAG	miRNA	miR-486-5p
t0006602	23	53	AAACCGTTACCATTACTTAGTTT	miRNA	miR-451
t0006629	23	53	AAACCTTTACCATTACTTAGTTT	miRNA	miR-451
t0006503	19	53	AAACTGTTACCATTACTGA	miRNA	miR-451
t0006554	22	53	TTGGACTTGGAGTCAGAAGGCA	miRNA	miR-378b
t0006463	23	53	TTATAATACAACCTGATAAGTAA	miRNA	miR-374a
t0006651	25	53	AACTTTGAAGACTGAAGTGGAGAAG	miRNA	miR-3526
t0006584	23	53	AAAAGCTGGGTTGAGAGGGAGTT	miRNA	miR-320d
t0006574	23	53	AAAAGCTGGGTTGAGAGGGAGAT	miRNA	miR-320d
t0006590	22	53	AAAGCTGGGTTGAGAGGGCGAG	miRNA	miR-320a
t0006767	23	53	GAAAGCTGGGTTGAGAGGGCGAA	miRNA	miR-320a
t0006700	22	53	CTAGCACCATCTGAAATCGGTT	miRNA	miR-29c
t0006744	23	53	TGGACAGAAAGGCAGTTCCTGAA	miRNA	miR-185
t0006786	21	53	TACAGCACGTAAATATTGGCG	miRNA	miR-16
t0006712	20	53	TAGCAGCACGTAAATATTGA	miRNA	miR-16
t0006722	20	53	TAGCATCACATAATGGTTTG	miRNA	miR-15a
t0006822	21	53	TAGCAGCACACAATGGTTTGT	miRNA	miR-15a
t0006850	20	53	TACAGTGTAGATGATGTACT	miRNA	miR-144
t0006826	22	53	TACAGTATAGATGATGTACTAG	miRNA	miR-144
t0006706	24	53	TCTGGGCAACAAAGTGAGACCTAA	miRNA	miR-1285
t0006788	21	53	AGCAGCATTGTACAGGGATAT	miRNA	miR-103a
t0006861	21	53	TACAGTACTGTGATAAATGAA	miRNA	miR-101
t0006818	22	53	TGACGTAGTAGTTTGTACAGTT	miRNA	let-7g
t0006823	28	53	TGAGGTAGTAGATTGTATAGTCGACCGG	miRNA	let-7f
t0006842	19	53	TGAGGTGGTAGATTGTATA	miRNA	let-7f
t0006736	24	53	AGAGGTAGTAGGTTGCATAGTTAT	miRNA	let-7d
t0006707	21	53	TGAGGTAGTAGGTTGTATACT	miRNA	let-7
t0006833	19	53	TGAGGTAGTAGGTTGTATT	miRNA	let-7
t0006689	26	52	AAACTTGTCCCGGCCTGTTATTGCAA	miRNA	miR-92c
t0006726	22	52	TATTGCACTTGTCTCGGCCTGT	miRNA	miR-92a
t0006745	18	52	TATTGCACTTGTCCCGGT	miRNA	miR-92a
t0006876	24	52	GTAGAGGAGATGGCGCAGGGGACA	miRNA	miR-877
t0006872	23	52	TTCTGTACTGAGCTGCCCCGAGT	miRNA	miR-486-5p
t0006847	22	52	TTTGTACTGAGCTGCCCCGAGA	miRNA	miR-486-5p
t0006871	24	52	TCCTGTACTGAGCTGCCCCGAGTG	miRNA	miR-486-5p
t0006820	22	52	TTTTGTACTGAGCTGCCCCGAG	miRNA	miR-486-5p
t0006755	21	52	ATAGCAAGAGAACCATTACCA	miRNA	miR-451b
t0006797	21	52	ATCGTTACCATTACTGAGTTT	miRNA	miR-451
t0006691	23	52	AAAAGCTGGGCTGAGAGGGCGAA	miRNA	miR-4429
t0006742	21	52	TGAGGGGCATAGAGCGAGACT	miRNA	miR-423-5p

t0006830	21	52	ACTGGACTTGGAGTCAGAAGA	miRNA	miR-378
t0006961	23	52	ACTGGACTTGGAGTTAGAAGGCA	miRNA	miR-378
t0007058	23	52	TGAAGACTGAAGTGGAGAAGGTT	miRNA	miR-3526
t0007068	22	52	GATTATAAAGCAATGAGACTGA	miRNA	miR-340-5p
t0006982	26	52	TGCAAACATCCTTCACTGGAAGCATC	miRNA	miR-30e-5p
t0006911	22	52	TTCAAGTAATTTAGGATAGGCT	miRNA	miR-26a
t0006987	21	52	TCGAGAGAAAGGCAGTTCCTG	miRNA	miR-185
t0007007	23	52	TGGAGAGAAATGCAGTTCCTGAA	miRNA	miR-185
t0007010	20	52	TGGAGAGAAAGGAAGTTCCT	miRNA	miR-185
t0006913	22	52	AAGGGAGATAGAACAGGTCTTG	miRNA	miR-1839
t0007030	21	52	AAGCGGCGATGGCGGAGCTGA	miRNA	miR-1636
t0006934	23	52	TTAGCAGCACGTAAATATTGGCG	miRNA	miR-16
t0006972	22	52	AGCAGCACGTAAATATTGGCGA	miRNA	miR-16
t0006889	18	52	TAGCAGCACATTATGGTT	miRNA	miR-15b
t0007025	20	52	TACCAGCACATAATGGTTTG	miRNA	miR-15a
t0006912	25	52	TTAATGCTAATTGTGATAGGGAATC	miRNA	miR-155
t0006890	19	52	TTACAGTATAGATGATGTA	miRNA	miR-144
t0006975	23	52	ACCACAGGGTAGAACCACGCACA	miRNA	miR-140
t0006996	22	52	TACCACAGGGTAAAACCACGGA	miRNA	miR-140
t0006905	22	52	ACCACAGGTTAGAACCACGGAA	miRNA	miR-140
t0006947	22	52	ATCACAGGGTAGAACCACGGAA	miRNA	miR-140
t0006970	20	52	AGCAGCATTGTACAGGGATA	miRNA	miR-103a
t0006936	24	52	TACAGTACTGTGATAACTGAAGAA	miRNA	miR-101
t0006910	20	52	TGAGGTGGTAGTTTGTGCTG	miRNA	let-7i
t0006956	22	52	TGAAGTAGTAGTTTGTGCTGTT	miRNA	let-7i
t0006983	22	52	TGAGGTAGTAGTTTGTGCTCTT	miRNA	let-7i
t0007020	22	52	TGAGGTAGTAGATTGTATACTA	miRNA	let-7f-5p
t0006942	21	52	AGAGGGAGTAGGTTGCATAGT	miRNA	let-7d
t0006944	22	52	AGAGGTAGTAGGTTGAATAGTT	miRNA	let-7d
t0006919	21	52	TGTGGTAGTAGGTTGTGTGGT	miRNA	let-7b
t0006933	20	52	TGAGGTAGTAGTTTGTGTGT	miRNA	let-7b
t0006943	20	52	GAGGTAGTATGTTGTATAGT	miRNA	let-7
t0006965	22	51	TATACAACTTACTACTTTCCTT	miRNA	miR-98*
t0007000	24	51	TATTGCATTTGTCCCGGCCTGTAA	miRNA	miR-92a
t0007061	22	51	AAAAGTACTTGCGGATTTTGCT	miRNA	miR-548k
t0007064	20	51	AAAAACTGAGACTACTTTTG	miRNA	miR-548e
t0006903	20	51	TGGGCACAGGCGGATGGACA	miRNA	miR-5107
t0007059	22	51	TCCTCTACTGAGCTGCCCCGAG	miRNA	miR-486-5p
t0007060	27	51	TCCTGTACTGAGCTGCCCCGAGACGGT	miRNA	miR-486-5p
t0006902	21	51	TCCTGTACCGAGCTGCCCCGA	miRNA	miR-486-5p
t0007005	21	51	CCTGTACTGAGCTGCCCCGAT	miRNA	miR-486-5p
t0007054	21	51	TCGTACTGAGCTGCCCCGAGA	miRNA	miR-486-5p
t0006986	22	51	TCCTGTAATGAGCTGCCCCGAG	miRNA	miR-486-5p
t0006992	24	51	AAACCCTTACCATTACTGAGTTTA	miRNA	miR-451
t0006974	24	51	AAACCGTTACCATTACTGAGTTAG	miRNA	miR-451
t0007168	25	51	TGAGGGGCAGAGAGCGAGACTTTAA	miRNA	miR-423-5p
t0007169	23	51	TTTGAAGACTGAAGTGGAGAAGG	miRNA	miR-3526
t0007209	24	51	TGAAGACTGAAGTGGAGAAGGGGT	miRNA	miR-3526
t0007161	22	51	TGACTGTCTGGACACAGTAGTA	miRNA	miR-3505
t0007116	22	51	AAAAGCTGGGTTGAGAGGGCTT	miRNA	miR-320d
t0007077	24	51	AAAAAAGCTGGGTTGAGAGGGCGA	miRNA	miR-320a
t0007215	22	51	AAAAGCTGGGTTGAGACGGCGA	miRNA	miR-320a
t0007228	21	51	CACTTGCGCTACTCAGGTCTG	miRNA	miR-3200-3p
t0007245	24	51	TGTGGACAGTGAGGTAGAGGGAGA	miRNA	miR-3138
t0007254	24	51	TAGGAACTTCATACCGTGCTCATC	miRNA	miR-276a-3p
t0007262	20	51	AGCTACATCTGGCTACTGGG	miRNA	miR-222
t0007091	24	51	TAAAGTGCTTATAGTGCAGGTAGT	miRNA	miR-20
t0007133	19	51	TGAGGTAGGTTGTATAGTT	miRNA	miR-1961

t0007085	23	51	CTGACCTATGAGTTGACAGCATC	miRNA	miR-192
t0007088	21	51	TGGAGAGAAAAGCAGTTCCTG	miRNA	miR-185
t0007230	21	51	TGGAGAGAAAGGCAATTCCTG	miRNA	miR-185
t0007165	21	51	TGTAGAGAAAGGCAGTTCCTG	miRNA	miR-185
t0007079	23	51	GTGGAGAGAAAGGCAGTTCCTGA	miRNA	miR-185
t0007090	22	51	TGGAGAGACAGGCAGTTCCTGA	miRNA	miR-185
t0007108	23	51	TGGAGAGAAAGGCAGTTCTCGAT	miRNA	miR-185
t0007115	23	51	AAGGTAGATAGAACAGGTCTTGT	miRNA	miR-1839
t0007137	21	51	TAGCAGCACGTAAATATTTGC	miRNA	miR-16
t0007129	22	51	TAGCAGCACATCTTGGTTTACA	miRNA	miR-15b
t0007196	23	51	TACTAGACTGAAGCTCCTTGAGG	miRNA	miR-151-3p
t0007181	22	51	TCTCCCAACCCTTGTACCAGAA	miRNA	miR-150
t0007072	24	51	TCTCCCAACCCTTGTACCAGTGAA	miRNA	miR-150
t0007202	20	51	CATAAAGTAGAAAGCACTAG	miRNA	miR-142-5p
t0007100	21	51	ACCACAGGGTAGAACCACGTA	miRNA	miR-140
t0007130	23	51	ACCACAGGGTAGAACCATGGACA	miRNA	miR-140
t0007182	23	51	ACCACAGTGTAGAACCACGGAAA	miRNA	miR-140
t0007216	23	51	ACCACAGGGTAGAACAACGGACA	miRNA	miR-140
t0007246	22	51	CTCGGCGTGGCGTCGGTCGTGT	miRNA	miR-1307
t0007253	22	51	AGCAGCATTGGACAGGGCTATG	miRNA	miR-103a
t0007117	21	51	AGCAGCATCGTACAGGGCTAT	miRNA	miR-103a
t0007076	20	51	AGCAGCATTGTACATGGCTA	miRNA	miR-103a
t0007242	22	51	TACAGTATTGTGATAACTGAAA	miRNA	miR-101
t0007103	23	51	TGAGGTAGTAGTTTGTGTTGTTT	miRNA	let-7i
t0007243	23	51	TGAGGTATTAGTTTGTACAGTTA	miRNA	let-7g
t0007248	24	51	TCTGAGGTAGTAGATTGTATAGTT	miRNA	let-7f
t0007235	23	51	TGAGGTAGGAGGTTGTATAGTTG	miRNA	let-7
t0007125	21	50	ATTGCACTTGTCTCGGCCTGT	miRNA	miR-92c
t0007145	22	50	TATTGCACTTGTCCCGGCCTTA	miRNA	miR-92b
t0007206	22	50	TATTGCACTCGTCCCGGCCAAA	miRNA	miR-92
t0007217	24	50	TGCGGGGCTAGGGCTAACAGCATC	miRNA	miR-744
t0007150	21	50	CTTGTACTGAGCTGCCCCGAG	miRNA	miR-486-5p
t0007107	23	50	TCCTGTACTGAGCTGCCCCGTAA	miRNA	miR-486-5p
t0007203	21	50	TTGTACTGAGCTGCCCCGAGA	miRNA	miR-486-5p
t0007205	23	50	TTCGGGGTCTGGGCGCGGCGATT	miRNA	miR-4508
t0007259	22	50	TCGAAATCGAGTCACGGCACCA	miRNA	miR-4454
t0007229	23	50	ACTGGACTTGGAGTCAGAAGGCC	miRNA	miR-378
t0007143	23	50	AATCCTTGGAACCTAGGTGTGAG	miRNA	miR-362-5p
t0007192	24	50	TTGAAGACTGAAGTTGAGAACGGT	miRNA	miR-3526
t0007238	24	50	GAAAAGCTGGGTTGAGAGGGCGAA	miRNA	miR-320a
t0007268	23	50	AAAAGCTGGCTTGAGAGGGCGAA	miRNA	miR-320a
t0007379	20	50	CTGGGAGGTGGATGTTTACT	miRNA	miR-30b*
t0007464	26	50	GTAGGCCGGCGGAAACTACTTGCATC	miRNA	miR-2796-3p
t0007323	22	50	AGGCGGAGACTTGGGCAATTGA	miRNA	miR-25*
t0007263	22	50	CATTGCACTTGTCTTGGTCTGT	miRNA	miR-25
t0007409	22	50	CATTGCACTTGTCTCGCTCTGT	miRNA	miR-25
t0007418	22	50	ATCACATTGTCAGGGATTTCCA	miRNA	miR-23a
t0007476	25	50	CTGACCTATGAATTGACAGCCAATC	miRNA	miR-192
t0007510	25	50	CTGACCTATGAATTGACAGCCAGTA	miRNA	miR-192
t0007431	23	50	TGGAGGGAAAGGCAGTTCCTGAA	miRNA	miR-185
t0007339	23	50	CAAAGTGCTTACAGTGCAGGTAT	miRNA	miR-17a
t0007478	22	50	TAGCAGCACGTAAACATTGGTG	miRNA	miR-16
t0007275	22	50	TTGCAGCACGTAAATATTGGCG	miRNA	miR-16
t0007381	24	50	TAGCAGTACGTAAATATTGGCGAT	miRNA	miR-16
t0007486	22	50	ACCCATAAAGTAGAAAGCACTA	miRNA	miR-142
t0007504	25	50	ACCACAGGGTAGAACCACGGACATC	miRNA	miR-140
t0007479	23	50	CCACAGGGTAGAACCACGGAAGA	miRNA	miR-140
t0007453	22	50	TACCACAGGGTAGGACCACGGA	miRNA	miR-140

t0007484	20	50	ACCATAGGGTAGAACCACGG	miRNA	miR-140
t0007271	21	50	TGCTGGATCAGTGGTTCGAGT	miRNA	miR-1287
t0007343	22	50	TGGGAGGCGGAGGTTGCAGTGA	miRNA	miR-1273f
t0007406	20	50	AGCAGCATTGTACGGGGCTA	miRNA	miR-103a
t0007393	21	50	AGCAGCATTGTATTGGGCTAT	miRNA	miR-103a
t0007477	22	50	AGCAGCATTGTAGAGGGCTATG	miRNA	miR-103a
t0007299	21	50	AGCAGCATTGTACAGGGCTCT	miRNA	miR-103a
t0007313	22	50	GTACAGTACTGTGATAATCGAC	miRNA	miR-101c
t0007353	22	50	TGAGGTAGTAGATTGTAATGTT	miRNA	let-7f
t0007272	21	50	TGAGGTAGTAGGTTGTGTGCT	miRNA	let-7b
t0007334	21	50	TGAGGTTGTAGGTTGTGTGGT	miRNA	let-7b
t0007370	22	50	TGACGTAGTAGGTTGTGTGGTT	miRNA	let-7b
t0007401	19	50	TGAGGTAGTAGGTATAGTT	miRNA	let-7a
t0007460	21	50	TGAGGTCGTAGGTTGTATAGT	miRNA	let-7
t0007335	22	49	CAAAGTGCTGTTCGTGCAGTTA	miRNA	miR-93
t0007427	23	49	TATTGCACTTGTCTTGGCCTGTA	miRNA	miR-92a
t0007286	23	49	GTAGAGGAGATGGCGCAGGGGAA	miRNA	miR-877
t0007338	23	49	GACTATAGAACTTTCCCCCTGAA	miRNA	miR-625*
t0007366	21	49	GAGCTTATTCATAAAAGTGCA	miRNA	miR-590-5p
t0007482	22	49	CCTCCCACACCTAAGGCTTGCA	miRNA	miR-532-3p
t0007459	22	49	TCCTGTACTGGGCTGCCCCGAG	miRNA	miR-486-5p
t0007320	22	49	AGCTGTAGAGCAGGGAGCAGGA	miRNA	miR-4732-5p
t0007372	21	49	CTGGGAAGAGGAGCTGAGGGA	miRNA	miR-4646-5p
t0007450	27	49	AAACCGTTACCATTACTGAGTTTAGTA	miRNA	miR-451
t0007452	23	49	AAACCGTTACTATTACTGAGTTT	miRNA	miR-451
t0007454	19	49	TGAGGGGCAGAGAGCGATA	miRNA	miR-423-5p
t0007493	21	49	TGATGGGCAGAGAGCGAGACT	miRNA	miR-423-5p
t0007585	22	49	TGGGGGGCAGAGAGCGAGACTT	miRNA	miR-423-5p
t0007588	29	49	GACTTTGAAGACTGAAGTGGAGAAGGGT	miRNA	miR-3526
t0007522	21	49	AACCTTGAAGACTGAAGTGGA	miRNA	miR-3526
t0007565	22	49	TGAAGACTGAAGTGGAGAAGTG	miRNA	miR-3526
t0007531	25	49	TCTCACACAGAAATCGCATCCGTCA	miRNA	miR-342-3p
t0007595	21	49	TTATAAAGCAATGAGACTGAA	miRNA	miR-340-5p
t0007557	22	49	AAAAGCTGGGTTGAGAGGCGTT	miRNA	miR-320d
t0007594	21	49	AAAAGCTGGGTTGAGAGGGAA	miRNA	miR-320d
t0007726	21	49	AAAAGTTGGGTTGAGAGGGCG	miRNA	miR-320a
t0007608	22	49	AAAATCTGGGTTGAGAGGGCGA	miRNA	miR-320a
t0007694	21	49	AAGCTGGGTTGAGAGGGCGAA	miRNA	miR-320a
t0007530	22	49	CATTGCACTTGTCTCTGTCTGA	miRNA	miR-25
t0007634	22	49	TAGGTAGTTTCCTGTTGTTGGG	miRNA	miR-196b
t0007690	24	49	TAGGTAGTTTCATGTTGTTGGATC	miRNA	miR-196a
t0007574	24	49	CTGACCTATGAATTGACAGCCATT	miRNA	miR-192
t0007683	21	49	CTGATCTATGAATTGACAGCC	miRNA	miR-192
t0007739	22	49	CAAAGAATTCTCCTTTTGGGCT	miRNA	miR-186
t0007600	21	49	TAGAGAGAAAGGCAGTTCCTG	miRNA	miR-185
t0007547	23	49	TGGAGAGAAAGGCAGTTCATGAA	miRNA	miR-185
t0007635	22	49	TGCAGAGAAAGGCAGTTCCTGA	miRNA	miR-185
t0007741	22	49	TGGAGAGAAAGGCATTTCCTGT	miRNA	miR-185
t0007616	21	49	AGACCTACTTATCTACCAACT	miRNA	miR-1839-3p
t0007655	26	49	TGGGGGCGGGGAGCGGTCGGGCGGTT	miRNA	miR-1607
t0007712	22	49	TAGCAGCACGTAAATAGTGGCG	miRNA	miR-16
t0007584	26	49	ATTTTAGCAGCACGTAAATATTGGCG	miRNA	miR-16
t0007693	23	49	TAGCAGCACGTTAATATTGGCGA	miRNA	miR-16
t0007680	22	49	TTAATGCTAATCGTGATAGGGG	miRNA	miR-155
t0007546	21	49	TGAGATGAAGCACTGTAGCTC	miRNA	miR-143
t0007576	21	49	ACCACAGGGTAGAACCACCGA	miRNA	miR-140
t0007572	22	49	TACCACAGAGTAGAACCACGGA	miRNA	miR-140
t0007613	23	49	TACCACAGGGTGGAACCACGGAC	miRNA	miR-140

t0007622	23	49	TACCACAGGGTAGAACAACGGAT	miRNA	miR-140
t0007724	26	49	TTATTGCTTAAGAATACGCGTAGATC	miRNA	miR-137
t0007729	22	49	TACCGCACTGTGGGTACTTGCA	miRNA	miR-106b*
t0007723	21	49	AGCAGCATTGGACAGGGCTAT	miRNA	miR-103a
t0007519	20	49	TACAGTACTTTGATAACTGA	miRNA	miR-101
t0007520	21	49	GTACAGTACTGTGATAACTGC	miRNA	miR-101
t0007631	22	49	TGAGGTAGTAGATTGTGCTGTT	miRNA	let-7i
t0007669	23	49	TGAGGTAGTACATTGTATAGTTG	miRNA	let-7f
t0007528	22	49	TGAGGTAGTAGGTAGTGTGGTT	miRNA	let-7b
t0007681	25	49	TGAGGTAGTAGGTTGTGTGGTTTAA	miRNA	let-7b
t0007525	25	49	TGAGGTAGTAGGTTGTGTGGTAGTC	miRNA	let-7b
t0007575	21	49	TGAGATAGTAGGTTGTGTGGT	miRNA	let-7b
t0007656	21	49	TGAGTTAGTAGGTTGTATAGT	miRNA	let-7
t0007658	23	48	CACCCGGCTGTGTGCACATGTGA	miRNA	miR-941
t0007636	22	48	ATTGCATTTGTCCCGGCCTGTT	miRNA	miR-92c
t0007700	24	48	TACGTCATCGTTGTCATCGTCATT	miRNA	miR-598
t0007597	20	48	TTCTGTACTGAGCTGCCCCG	miRNA	miR-486-5p
t0007692	22	48	TCCTTTACTGAGCTGCCCCGAG	miRNA	miR-486-5p
t0007736	20	48	TCTTGTATTGAGCTGCCCCG	miRNA	miR-486-5p
t0007623	24	48	TCCTGTACTGAGCTGCTTCGAGGA	miRNA	miR-486-5p
t0007556	24	48	TGTAGAGCAGGGAGCAGGAAGTTG	miRNA	miR-4732-5p
t0007612	22	48	AAGGGCTGGGTCGGTCGGGCTG	miRNA	miR-4651
t0007643	23	48	AAACCGTTACCATTACTGGGTTT	miRNA	miR-451
t0007708	23	48	AATGACACGATCACTCTCGTTGA	miRNA	miR-425
t0007727	22	48	TGAAGGGCAGAGAGCGAGACTT	miRNA	miR-423-5p
t0007535	22	48	TGAGGGGCAGAGAGCGCGACTT	miRNA	miR-423-5p
t0008008	21	48	TGAGGGGCAGAAAGCGAGACT	miRNA	miR-423-5p
t0007873	23	48	AAAAGCTGGGTTGATAGGGCGTA	miRNA	miR-320d
t0007745	22	48	AAAATCTGGGTTGAGAGGGCGT	miRNA	miR-320d
t0007760	22	48	TTCAAGTAATCCAGGATAGGAA	miRNA	miR-26a
t0007822	22	48	CATTGCACTTGTCTCGGTTTTA	miRNA	miR-25
t0007843	25	48	TTGGGGAAACGGCCGCTGAGTGAGT	miRNA	miR-2110
t0007959	27	48	TAGCTTATCAGACTGATGTTGACTATC	miRNA	miR-21
t0007886	23	48	TAGGTAGTTTCCTGTTGTTGGGT	miRNA	miR-196b
t0007990	21	48	TGGAGTGAAAGGCAGTTCCTG	miRNA	miR-185
t0007958	19	48	TACAGCACGTAAATATTGG	miRNA	miR-16
t0007903	21	48	CGAATCATTATTTGCTGCTCT	miRNA	miR-15b*
t0007761	20	48	TAGCGGCACATCATGGTTTA	miRNA	miR-15b
t0007853	23	48	ACCACAGGGTAGAACTACGGATA	miRNA	miR-140
t0007915	23	48	TACCACAGTGTAGAACCACGGAT	miRNA	miR-140
t0007747	21	48	TCTGGGCAACAAAGTGAGACA	miRNA	miR-1285
t0007872	23	48	CTGGAGATATGGAAGAGCTGTGT	miRNA	miR-1270
t0008010	22	48	AGCAGCATTTTACAGGGCTATG	miRNA	miR-103a
t0007815	20	48	TACAGTATTGTGATAACTGA	miRNA	miR-101
t0007864	22	48	TACAGTATTGTGATAACTGAAG	miRNA	miR-101
t0007993	20	48	TGAGGTAGTAGATTGTATAC	miRNA	let-7f
t0007792	18	48	TGTAGTAGATTGTATAGT	miRNA	let-7f
t0007905	25	48	TGAGGTAGTAGATTGTATAGTTCGT	miRNA	let-7f
t0007871	22	48	AGAGGTAGTAGGTTGCGTAGTT	miRNA	let-7d
t0007874	22	48	TGAGGTAGTGGGTTGTGTGGTA	miRNA	let-7b
t0007762	20	48	TGAGGTAGTAGGGTGTGTGG	miRNA	let-7b
t0007787	20	48	TGAGGTAGTAGGCTGTATAG	miRNA	let-7
t0007790	26	48	TGAGGTAGTAGGTTGTATAGTTTATC	miRNA	let-7
t0007943	20	48	CGAGGTAGTAGGTTGTATAG	miRNA	let-7
t0007858	19	47	ATTGCACTTGTCCCGGCCT	miRNA	miR-92c
t0007979	22	47	AGGGACGGGACGCGGTGCAGTA	miRNA	miR-92b*
t0007888	23	47	TATTTCACTTGTCCCGGCCTGTA	miRNA	miR-92a
t0007996	22	47	GTAGAGGAGATGGCGTAGGGGA	miRNA	miR-877

t0007929	24	47	TGAAGATTGAAGTGGAGAAGGGTT	miRNA	miR-739
t0007975	19	47	AAAGTAATCGTGGTCTTTG	miRNA	miR-548j
t0008000	26	47	TCCTGTACTGAGCTGCCCCGAGACGG	miRNA	miR-486-5p
t0007866	21	47	TCCTGTACTGAACTGCCCCGA	miRNA	miR-486-5p
t0007896	25	47	TCCTGTACTGAGCTGCCCCGAGTGA	miRNA	miR-486-5p
t0007977	22	47	TCAGGCTCAGTCCCCTCCCGAT	miRNA	miR-484
t0007828	22	47	AAACCGTTACCACTACTGAGTT	miRNA	miR-451
t0007904	23	47	TGAGGGGCAGAGAGAGAGACTTA	miRNA	miR-423-5p
t0007985	21	47	TGAGGGGCACAGAGCGAGACT	miRNA	miR-423-5p
t0008092	21	47	TGAGGGGCAGAGAGCGAGACC	miRNA	miR-423-5p
t0008088	21	47	GAGGGACTTTCAGGGGCAGCT	miRNA	miR-365-5p
t0008156	22	47	AATTGCACGGTATCCATCTCTA	miRNA	miR-363
t0008118	21	47	TTGAGGACTGAAGTGGGGAAA	miRNA	miR-3526
t0008246	20	47	TCTCACACAGAAATCGCACC	miRNA	miR-342-3p
t0008059	23	47	GCAAAGCACACGGCCTGCAGAGT	miRNA	miR-330-3p
t0008052	21	47	AAAAGCTGGGTTGAGAGGGTA	miRNA	miR-320d
t0008089	23	47	AAAAGCTGGGTTGAGAGGGCAGA	miRNA	miR-320d
t0008284	21	47	AAAGCTGGGTTGAGAGGGCAA	miRNA	miR-320b
t0008172	22	47	AAAAGATGGGTTGAGAGGGCGA	miRNA	miR-320a
t0008220	22	47	AAAAGCTGGGTTGAAAGGGCGA	miRNA	miR-320a
t0008033	23	47	AAAAGCTGGGTAGAGAGGGCGAT	miRNA	miR-320a
t0008206	19	47	TGGGACAAGAGGACGGTCT	miRNA	miR-3122
t0008255	22	47	CATTGCGCTTGTCTCGGTCTGA	miRNA	miR-25
t0008276	21	47	CATTGCACTTGTCTCGGTTTG	miRNA	miR-25
t0008055	21	47	AGAGTTGAGTCTGGACGTCTT	miRNA	miR-219-1-3p
t0008084	20	47	TAAAGTGCTTATAGTGCAGG	miRNA	miR-20
t0008098	23	47	CAACGGAATCCCAAAAGAAGCTG	miRNA	miR-191
t0008203	21	47	AACGGAATTCCAAAAGCAGCT	miRNA	miR-191
t0008181	21	47	TGGAGAGAAAGGCGGTTCCTG	miRNA	miR-185
t0008186	22	47	GGGAGAGAAAGGCAGTTCCTGA	miRNA	miR-185
t0008097	24	47	TGGAGAGAAAGGCAGTTCCTGACA	miRNA	miR-185
t0008091	23	47	TAGCAGCACGTAAATATTGGCTT	miRNA	miR-16b
t0008224	23	47	TAGCAGCATGTAAATATTGGCGA	miRNA	miR-16
t0008254	20	47	AGCAGCACGTAAATATTGCG	miRNA	miR-16
t0008211	22	47	AGCAGCACATCATGGTTTACAT	miRNA	miR-15b
t0008069	20	47	TAGCAGCACATCATGGATTA	miRNA	miR-15b
t0008288	30	47	TAGCAGCACATCATGGTTTACAAATCATG	miRNA	miR-15b
t0008094	19	47	AGCAGCACATAATGGTTTG	miRNA	miR-15a
t0008235	24	47	TATCAGCACATAATGGTTTGTGGA	miRNA	miR-15a
t0008034	22	47	ACCACAGGGTAGAATCACGGAT	miRNA	miR-140
t0008099	22	47	CCACAGGGTAGAACCACGGAAA	miRNA	miR-140
t0008124	22	47	ACCACAGGGGAGAACCACGGAC	miRNA	miR-140
t0008126	23	47	ACTGGAGATATGGAAGAGCTGTG	miRNA	miR-1270
t0008134	22	47	TAGCGCACTGTGGGTACTTGCT	miRNA	miR-106b*
t0008120	21	47	AGCATCATTGTACAGGGCTAT	miRNA	miR-103a
t0008030	21	47	ATACAGTACTGTGATAACTGA	miRNA	miR-101
t0008044	25	47	TGAGGTAGTAGTTTGTACAGTTATC	miRNA	let-7g
t0008063	25	47	TGAGGTAGTAGTTTGTACAGTTAGA	miRNA	let-7g
t0008116	23	47	TGAGGTAGTAGATTGTATAGTAT	miRNA	let-7f
t0008230	24	47	TAGGTAGTAGGTTGTATGGTTATC	miRNA	let-7c
t0008072	23	47	TGAGGTAGTAGGTTGTGTGGAGA	miRNA	let-7b
t0008146	21	47	TGAGGTAGTCGGTTGTGTGGT	miRNA	let-7b
t0008047	21	47	TGAGGTGGTAGGTTGTGTGGA	miRNA	let-7b
t0008154	24	47	TGAGGTAGTAGGTTGTGTGGTAAT	miRNA	let-7b
t0008279	24	47	TGAGGTAGTAGGTTGTGTGGGTTG	miRNA	let-7b
t0008213	22	47	TGAGGTAGTAGGTTGTATAGCT	miRNA	let-7
t0008270	22	47	TGAGGTAGTCGGTTGTATAGTT	miRNA	let-7
t0008078	22	46	CAGGTGGAAGGTTGTAGGCATG	miRNA	miR-965-5p

t0008157	22	46	TATTGCATCTGTCCCGGCCTGT	miRNA	miR-92a
t0008024	23	46	AAAGACTTGAGTAGTGAGACGTT	miRNA	miR-71b-5p
t0008061	24	46	AACTGGCTAGGGAAAATGATTGGA	miRNA	miR-664*
t0008129	20	46	TCCTGTACTGAGCTGCTTCG	miRNA	miR-486-5p
t0008027	23	46	TCTCGTACTGAGCTGCCCCGAGA	miRNA	miR-486-5p
t0008046	22	46	TCCTGTACTGAGCTGATCCGAG	miRNA	miR-486-5p
t0008159	21	46	TCAGGCTCAGTCTTCTCCCGA	miRNA	miR-484
t0008161	22	46	TACACACACACACATACACAGA	miRNA	miR-466i-3p
t0008187	25	46	AGAAACCGTTACCATTACTGAGTTT	miRNA	miR-451
t0008232	23	46	AAACCGTTACCATTAATGAGTTT	miRNA	miR-451
t0008048	20	46	AAACCGTTACCATCACTGAG	miRNA	miR-451
t0008207	27	46	AAACCGTTACCATTACTGAGTTGGATA	miRNA	miR-451
t0008559	20	46	ACTCCCACTGCTTCACTTGA	miRNA	miR-4301
t0008565	22	46	TGAGGGGCAGAGAGCGAGATTA	miRNA	miR-423-5p
t0008448	20	46	AATTGCACGGTATCCATCTA	miRNA	miR-363
t0008406	20	46	TCCGTGCTGAGATTTCGTCA	miRNA	miR-3492
t0008378	24	46	CAAAGCACACGGCCTGCAGAGAGG	miRNA	miR-330-3p
t0008488	22	46	CGCATCCCCTAGGGCATTGGTG	miRNA	miR-324-5p
t0008555	21	46	AAAAGCCGGGTTGAGAGGGCG	miRNA	miR-320c
t0008524	20	46	TGTAAACATCCTACACTCTC	miRNA	miR-30c
t0008535	21	46	TTTCAGTCGGATGTTTACAGT	miRNA	miR-30b-3p
t0008365	22	46	TTCAAGTAATCCAGGATAGGTT	miRNA	miR-26a
t0008471	23	46	AGGCGGAGACTTGGGCAATTGCA	miRNA	miR-25*
t0008495	21	46	CATTGCACTTGTCTCGGCCTG	miRNA	miR-25
t0008527	20	46	CATTGCACTTGTCTCGGTCA	miRNA	miR-25
t0008403	21	46	CATTGCACTTGTCTCTGTCTG	miRNA	miR-25
t0008532	22	46	ATCACATTGCCAGGGATTACCA	miRNA	miR-23b
t0008561	22	46	TAGGATGGGGTGTGATAGGATC	miRNA	miR-2392
t0008446	22	46	TACAGTAGTCTGCACATTGGTT	miRNA	miR-199*
t0008511	18	46	TGAGGTAGGTTGTATAGT	miRNA	miR-1961
t0008453	22	46	CAACGGGATCCCAAAAGCAGCT	miRNA	miR-191
t0008292	21	46	TGGAGAGCAAGGCAGTTCCTG	miRNA	miR-185
t0008539	26	46	TGGAGAGAAAGGCAGTTCCTGAAGGT	miRNA	miR-185
t0008362	27	46	TTTGGCAATGGTAGAACTCACACTGAT	miRNA	miR-182
t0008363	25	46	GGAAGCGGCGATGGCGGAGCTGAAA	miRNA	miR-1636
t0008457	22	46	CGAATCATTATTTGCTGCTCTA	miRNA	miR-15b*
t0008438	19	46	AGCAGCACATCATGGTTTA	miRNA	miR-15b
t0008463	20	46	TAGCAGCACATCATGGTTTC	miRNA	miR-15b
t0008477	20	46	TAGCAACACATAATGGTTTG	miRNA	miR-15a
t0008548	20	46	TCGAGGAGCTCACAGTCTAG	miRNA	miR-151-5p
t0008307	19	46	TACAGTATAGATGATGTTC	miRNA	miR-144
t0008501	20	46	CTACAGTATAGATGATGTAT	miRNA	miR-144
t0008421	24	46	TACCACAGGGTAGAACCACGGCCG	miRNA	miR-140
t0008523	22	46	ATAAAGTGCTGACAGTGCAGAA	miRNA	miR-106b
t0008340	23	46	AGCAGCATTGTACAGGGATATGA	miRNA	miR-103a
t0008505	20	46	TGAGGGAGTAGTTTGTGCTG	miRNA	let-7i
t0008350	23	46	TTAGGTAGTAGATTGTATAGTTT	miRNA	let-7f
t0008445	20	46	TGAGCTAGTAGATTGTATAG	miRNA	let-7f
t0008371	24	46	TGAGGTAGTAGATTGTATAGTTGT	miRNA	let-7f
t0008347	22	46	AGAGGCAGTAGGTTGCATAGTT	miRNA	let-7d
t0008493	23	46	TGAGGTAGTGGGTTGTGTGGTTT	miRNA	let-7b
t0008345	22	46	TGAGGTAGTAGGTGTTGTGGTT	miRNA	let-7b
t0008431	22	46	TGAGGTAGTAGGTTGTGTGTAA	miRNA	let-7a
t0008318	24	46	TGAGGTAGTAGGTTGTATAGAATC	miRNA	let-7-5p
t0008430	22	46	TGAGTTAGTAGGTTGTATAGTT	miRNA	let-7
t0008486	22	46	GTTTGAGGTAGTAGGTTGTATA	miRNA	let-7
t0008329	22	45	TATTGCACTTGTCCCGGCTGTA	miRNA	miR-92b
t0008472	22	45	GGTGGGAGACTGTGAAGCGGTG	miRNA	miR-920

t0008474	20	45	TATTGCACTCGTCCCGGCAA	miRNA	miR-92
t0008353	23	45	TGAAGGCCGAAGTGGAGAAGGGT	miRNA	miR-739
t0008429	22	45	CACGCTCATGCACACACCCACA	miRNA	miR-574-3p
t0008502	26	45	TCCTGTACTGAGCTGCCCCGAGCGCG	miRNA	miR-486-5p
t0008496	22	45	TCCTGTACTGAGTCGCCCCGAG	miRNA	miR-486-5p
t0008344	22	45	TCCTGTACTGAGTTGCCCCGAT	miRNA	miR-486-5p
t0008469	22	45	TCCTGTACTGAGCTGCCCCCAG	miRNA	miR-486-5p
t0008551	22	45	TACTAACTGCAGATTCAAGTGA	miRNA	miR-4637
t0008549	19	45	GAGAGAAGGGTCGGGGCGG	miRNA	miR-4516
t0008497	22	45	TGAGGGTGTAGGTTGTGTGGTT	miRNA	miR-4510
t0008529	19	45	AAACCTTTACCATTACTGA	miRNA	miR-451
t0008629	28	45	AAACCGTTACCATTACTGAGTTGCCCGG	miRNA	miR-451
t0008846	21	45	ACCGTTACCATTACTGAGTAA	miRNA	miR-451
t0008786	22	45	ACAGGAGTGGGGGGTGGGACGT	miRNA	miR-4433
t0008866	23	45	ACTCCCACTGCTTCACTTGACTA	miRNA	miR-4301
t0008586	19	45	AAGCATTGTACAGGGCTAT	miRNA	miR-4289
t0008593	24	45	TGAGGGGCAGAGAGCGAGACCTTG	miRNA	miR-423-5p
t0008594	21	45	TAGGGGCAGAGAGCGAGACTT	miRNA	miR-423-5p
t0008733	21	45	TGAGGGGCTGAGAGCGAGACT	miRNA	miR-423-5p
t0008793	23	45	GCTGGACTTGGAGTCAGAAGGCA	miRNA	miR-378
t0008835	22	45	GAGGACTGAAGTGGGGAAAGGT	miRNA	miR-3526
t0008854	27	45	AACTTTGAAGGCCGAAGTGGAGAAGGG	miRNA	miR-3526
t0008644	21	45	TCTCACACAGAAATCGCACCC	miRNA	miR-342-3p
t0008691	24	45	CAAAAGCTGGGTTGAGAGGGCGTA	miRNA	miR-320d
t0008820	24	45	AAAAGCTGGGTTGAGAGGGCGCAT	miRNA	miR-320d
t0008848	22	45	AAAAGCTAGGTTGAGAGGGCGA	miRNA	miR-320a
t0008712	24	45	GTGGAAGGTAGACGGCCAGAGAGA	miRNA	miR-3190
t0008682	21	45	TAGCAGCATCTGAAATCGGTT	miRNA	miR-29d
t0008706	22	45	AGGCGGAGACTTGGGCAATTGC	miRNA	miR-25*
t0008603	22	45	TTTCCTATGCATATACTTCTTT	miRNA	miR-202*
t0008625	25	45	CAACGGAATCCCAAAAGCAGCTGAT	miRNA	miR-191
t0008853	21	45	TGGAGAGGAAGGCAGTTCCTG	miRNA	miR-185
t0008842	21	45	TGGTTCTGTAGAGGTCGGCCA	miRNA	miR-1842
t0008707	23	45	TAGCAGCACGTAAATATTGGTGA	miRNA	miR-16
t0008741	24	45	TTTTTAGCAGCACGTAAATATTGG	miRNA	miR-16
t0008575	25	45	CATTAGCAGCACGTAAATATTGGCG	miRNA	miR-16
t0008628	19	45	TCTCCCAACCCTTGTACCA	miRNA	miR-150
t0008845	21	45	ACCAAAGGGTAGAACCACGGA	miRNA	miR-140
t0008747	21	45	ACCACAGGGTTGAACCACGGA	miRNA	miR-140
t0008577	22	45	ACCACAGGGTAGAACAACGGAT	miRNA	miR-140
t0008718	20	45	CTGTGAGGTTGGCATTGTTG	miRNA	miR-1294
t0008704	23	45	TGTATGTGTGTATATATGTGTGA	miRNA	miR-1187
t0008830	23	45	AGCAGCATTGTACAGTGCTATGA	miRNA	miR-103a
t0008814	21	45	AGCAGCATTGTACAGGTCTAT	miRNA	miR-103a
t0008572	22	45	AGCAGAATTGTACAGGGCTATG	miRNA	miR-103a
t0008849	21	45	GCAGCATTGTACAGGGCTATG	miRNA	miR-103a
t0008583	22	45	GTACAGTACTGTGATAACTGAG	miRNA	miR-101c
t0008726	22	45	TACAGTACTGTGATAACTGTAG	miRNA	miR-101
t0008797	22	45	GTACATTACTGTGATAACTGAA	miRNA	miR-101
t0008836	21	45	AGTTGAGGTAGTAGATTGTAT	miRNA	let-7f
t0008571	23	45	AAGAGGTAGTAGGTTGCATAGTT	miRNA	let-7d
t0008652	21	45	TGAGGTAGTAGGTTGAGTGGT	miRNA	let-7b
t0008729	24	45	TGAGGTAGTAGGTTGTGTGGTATT	miRNA	let-7b
t0008642	23	45	TGAGGTAGAAGGTTGTGTGGTTA	miRNA	let-7b
t0008609	18	45	TGAGGTAGTAGGTATAGT	miRNA	let-7a
t0008614	18	45	TGAGGTAGTAGGTTATAG	miRNA	let-7
t0008627	19	44	ATTGCACTTGTTCCGGCCT	miRNA	miR-92c
t0008723	31	44	GAAGACTGAAGTGGAGAAGGGTTTCTTG	miRNA	miR-739

t0008805	22	44	TCGAATCCCAGCGGTGCCTCCA	miRNA	miR-5100
t0008746	21	44	TCCTGTACTGAGCTGCCATGA	miRNA	miR-486-5p
t0008592	25	44	TCCTGTACTGAGCTGCCCCGAGAAG	miRNA	miR-486-5p
t0008803	22	44	ATGGCCAGAGCTCACACAGAGA	miRNA	miR-4435
t0008626	20	44	TGGAGAGAAAGGCAGTTGTT	miRNA	miR-4306
t0008826	21	44	TGAGGGGCAGAGAGAGAGACA	miRNA	miR-423-5p
t0008610	21	44	TGAGGGGCAGAGAACGAGACT	miRNA	miR-423-5p
t0008653	24	44	TGAGGGGCAGAGAGCGAGAATTTT	miRNA	miR-423-5p
t0008816	22	44	TGAGAGGCAGAGAGCGAGACTT	miRNA	miR-423-5p
t0008674	23	44	TGAGGGGCACAGAGCGAGACTTT	miRNA	miR-423-5p
t0008584	23	44	CTGGACTTGGAGTCAGAAGGCAA	miRNA	miR-378
t0009138	25	44	TGAAGACTGAAGTGGAGAAGGGGTT	miRNA	miR-3526
t0008905	23	44	AAAAGCTGGGTTGAGACGGCGAA	miRNA	miR-320a
t0009129	23	44	TAAAGCTGGGTTGAGAGGGCGAT	miRNA	miR-320a
t0009036	23	44	TGTAAACATCCTCGACTGGAAGC	miRNA	miR-30a-5p
t0009158	24	44	TGTAAACATCCCCGACTGGAAGCA	miRNA	miR-30a-5p
t0009085	22	44	CATTGAACTTGTCTCGGTCTGA	miRNA	miR-25
t0009098	21	44	AGTTCTTCAGTGGCAAGCTTT	miRNA	miR-22*
t0008942	28	44	TGACTGGGGCGGTACATCTGTTAAATAT	miRNA	miR-219-2-3p
t0008954	26	44	CAACGGAATCCCAAAAGCAGCATAAA	miRNA	miR-191
t0008980	22	44	TGGTGAGAAAGGCAGTTCCTGA	miRNA	miR-185
t0009006	22	44	TAGCAGCACGTAAATATTTGCA	miRNA	miR-16b
t0009133	23	44	TAGCAGCACGTAAATATTGGAGA	miRNA	miR-16
t0009128	21	44	CTAGCAGCACGTAAATATTGG	miRNA	miR-16
t0009012	25	44	TAGCAGCACGTAAATATTGGCGTAA	miRNA	miR-16
t0008908	20	44	GGATATTATCATATACTGTA	miRNA	miR-144*
t0009068	21	44	CTACAGTATAGATGATGCACT	miRNA	miR-144
t0008896	27	44	TGAGATGAAGCACTGTAGCTCTTAATC	miRNA	miR-143
t0009057	23	44	TGAGATGAAGCACTGTAGCAATC	miRNA	miR-143
t0009050	27	44	ACCACAGGGTAGAACCACGGACACCGG	miRNA	miR-140
t0008976	23	44	TACCACAGGGTATAACCACGGAA	miRNA	miR-140
t0009048	23	44	ACCACAGGGTATAACCACGGACA	miRNA	miR-140
t0009149	24	44	TACCACAGGGTAGAACCACGGACC	miRNA	miR-140
t0009077	21	44	GTACAGTACTCTGATAACTGA	miRNA	miR-101
t0009045	23	44	TCTGAGGTAGTAGTTTGTACAGT	miRNA	let-7g
t0009027	25	44	TGAGGTAGTAGATTGTATAGTCGGT	miRNA	let-7f
t0009037	20	44	AGAGGTACTAGGTTGCATAG	miRNA	let-7d
t0009071	22	44	AGAGGTAGTAGGATGCATAGTT	miRNA	let-7d
t0008990	20	44	TGAGGTAGTAGGTTGTATGT	miRNA	let-7c
t0008912	21	44	TGAGGTAGTAGGTTGTATGTT	miRNA	let-7c
t0008950	23	44	TGAGGTAGTAGGTTGTGTAGTTA	miRNA	let-7b
t0008952	22	44	TGAGGTAGTAGGTTGGTTGGTT	miRNA	let-7b
t0008881	20	44	TGAGGTACTAGGTTGTGTGG	miRNA	let-7b
t0008931	26	44	TGAGGTAGTAGGTTGTATGGTAAATC	miRNA	let-7-5p
t0008953	21	44	TGTGGTAGTAGGTTGTATAGT	miRNA	let-7
t0009030	26	43	AACCCGTAGATCCGAACTTGTGTATC	miRNA	miR-99a
t0008961	21	43	TGAACTGATAGATACTCGGCT	miRNA	miR-996
t0008987	22	43	TATTGCACTCGTCCCGGCCATA	miRNA	miR-92
t0008887	22	43	TATTGCACTCGTCCCGGCCAAT	miRNA	miR-92
t0009019	22	43	TGGAAGACTGGTGATATGTTGT	miRNA	miR-7-5p
t0009151	27	43	AAGTTGGATTGTGACTGGCAAGTGGAA	miRNA	miR-753c
t0008909	22	43	TTCTAGTAAGAGTGGCAGTCGA	miRNA	miR-628-3p
t0008979	21	43	ATTCTAATTTCTCCACGTCTT	miRNA	miR-576-5p
t0008882	19	43	AACCACAATTACTTTTGCA	miRNA	miR-548aa
t0008956	22	43	CCTCCCACACTTAAGGCTTGCA	miRNA	miR-532-3p
t0009020	21	43	TCATGTACTGAGCTGCCCCGA	miRNA	miR-486-5p
t0009118	22	43	CCCTGTACTGAGCTGCCCCGAG	miRNA	miR-486-5p
t0009101	27	43	TCCTGTACTGAGCTGCCCCGAGTAGGA	miRNA	miR-486-5p

t0009170	22	43	TCCTGTACTGAGCTGCCCCGGA	miRNA	miR-486-5p
t0009356	21	43	TAGATGTAGGATTGTGTCGGT	miRNA	miR-4860
t0009279	23	43	TGTAGAGCAGGGAGTAGGAAGCT	miRNA	miR-4732-5p
t0009399	25	43	AGGCTGGGTCGGTCGGGCTGGGGCG	miRNA	miR-4651
t0009330	22	43	TAGTGCAATATTGCTTATAGGG	miRNA	miR-454
t0009162	25	43	AAACCGTTACCATTACTGAGTTTGA	miRNA	miR-451
t0009228	21	43	AAACCGTTACCCTTACTGAGT	miRNA	miR-451
t0009205	21	43	TCCCACTGCTTCACTTGACTA	miRNA	miR-4301
t0009409	22	43	TGAGGGGCAGAGAGCGAGACCT	miRNA	miR-423-5p
t0009281	22	43	TGAGGGGCAGAGAGCGAGACTG	miRNA	miR-423-5p
t0009172	22	43	TGAGGGGCAGAGAGCGAGAGTT	miRNA	miR-423-5p
t0009288	20	43	TGAGGGGCAGAGAGCGAGTT	miRNA	miR-423-5p
t0009379	22	43	TGAGGGGCAGAAAGCGAGACTT	miRNA	miR-423-5p
t0009407	27	43	ACTGGACTTGGGGTCAGAAGGCATATC	miRNA	miR-378
t0009262	20	43	CCGGGGGTGGGGTCGGCGGG	miRNA	miR-3621
t0009372	23	43	AAAAGCTGCGTTGAGAGGGCGTT	miRNA	miR-320d
t0009422	22	43	AAAAGCTGGGGTGAGAGGGCGT	miRNA	miR-320d
t0009333	21	43	AAAAGCTGGGTTGAGAGGGCT	miRNA	miR-320d
t0009174	22	43	GAAAGCTGGGTTGAGAGGGCGA	miRNA	miR-320a
t0009342	22	43	AAAAGCTCGGTTGAGAGGGCGA	miRNA	miR-320a
t0009483	23	43	AAAAGCTGCGTTGAGAGGGCGAT	miRNA	miR-320a
t0009185	26	43	CTGGAAAAGCTGGGTTGAGAGGGCGA	miRNA	miR-320a
t0009192	22	43	AAAAGCTGGGTTTAGAGGGCGA	miRNA	miR-320a
t0009210	22	43	AAAAGGTGGGTTGAGAGGGCGA	miRNA	miR-320a
t0009289	23	43	AAAGCTGGGTTGAGAGGGCGATG	miRNA	miR-320a
t0009428	22	43	AAAAGCTGGGTTGGGAGGGCGA	miRNA	miR-320a
t0009360	24	43	TTCAAGTAATCCAGGATAGGCAGA	miRNA	miR-26a
t0009294	22	43	TTCCTATGCATATACTTCTTTG	miRNA	miR-202-5p
t0009335	23	43	TGTGCAAATCTATGCAAAACTGA	miRNA	miR-19a
t0009373	23	43	TAACGGAATCCCAAAAGCAGCTG	miRNA	miR-191
t0009501	18	43	TGGAGAGAAAGGCAGTGA	miRNA	miR-185
t0009171	22	43	AGGTAGATAGAACAGGTTTTGT	miRNA	miR-1839
t0009195	25	43	ATGGCACTGGTAGAATTCACTGTAC	miRNA	miR-183
t0009208	23	43	TAGGGACGGGAGCGCGCGCGGGA	miRNA	miR-1763
t0009332	26	43	AAAATAGCAGCATGTAAATATTGGCG	miRNA	miR-16
t0009426	21	43	CTAGCAGCACATCATGGTTTA	miRNA	miR-15b
t0009447	20	43	CAGCAGCACATCATGGTTTA	miRNA	miR-15b
t0009466	18	43	GCAGCACATCATGGTTTA	miRNA	miR-15b
t0009485	21	43	CCCATAAAGTAGAAAGCATTA	miRNA	miR-142
t0009175	23	43	ACCACAGGGTAGAATTACGGACA	miRNA	miR-140
t0009402	20	43	TCACAGGGTAGAACCACGGA	miRNA	miR-140
t0009223	20	43	ACACAGGGTAGAACCACGGA	miRNA	miR-140
t0009217	23	43	TAACACAGGGTAGAACCACGGAC	miRNA	miR-140
t0009190	21	43	ATCCCACCGCTGCCACCAAGA	miRNA	miR-1260b
t0009234	20	43	TAAAGTGCTGACAGTGCCGA	miRNA	miR-106b
t0009182	27	43	AGCAGCATTGTACAGGGCTATGAACTC	miRNA	miR-103a
t0009448	24	43	AAAAAGCAGCATTGTACAGGGCTA	miRNA	miR-103a
t0009218	21	43	AGCAGCATTGTAAAGGGCTAT	miRNA	miR-103a
t0009191	21	43	AGCAGCATTGTACACGGCTAT	miRNA	miR-103a
t0009397	23	43	TGGAATGTAAAGAAGTGTGTATC	miRNA	miR-1
t0009424	20	43	TTAGGTAGTAGTTTGTGCTT	miRNA	let-7i
t0009361	22	43	CGAGGTAGTAGATTGTATTGTT	miRNA	let-7f
t0009227	19	43	TGAGGTACTAGATTGTATA	miRNA	let-7f
t0009337	22	43	AGAGGTAGTAGGTTGCATAGAA	miRNA	let-7d
t0009417	24	43	TGAGGTAGTAGGTTGTGTGTGTTA	miRNA	let-7b
t0009502	22	43	TGAGGTAGTAGGTTGTGTGGGA	miRNA	let-7b
t0009229	22	43	TGAGGTTGTAGGTTGTGTGGTT	miRNA	let-7b
t0009198	24	43	TGAGGTAGTAGGTTGTATGGTTAG	miRNA	let-7b

t0009194	20	43	TGAGGTAGTAGGTTGTATAT	miRNA	let-7
t0009302	22	43	TGAAGTAGTAGGTTGTATAGTT	miRNA	let-7
t0009452	21	43	TGAGGTAGTAGGTTGTATAGC	miRNA	let-7
t0009458	21	43	TGAGATAGTAGGTTGTATAGT	miRNA	let-7
t0009353	22	43	TGAGGTTGTAGGTTGTATAGTT	miRNA	let-7
t0009470	19	42	CAAAGTGCTGTTCGTGCAG	miRNA	miR-93
t0009273	25	42	TGAAGATTGAAGTGGAGAAGGGTTT	miRNA	miR-739
t0009491	22	42	TCTGGAGACGGAACTCCACAGA	miRNA	miR-5114
t0009410	21	42	TCCTGTACTGATCTGCCCCGA	miRNA	miR-486-5p
t0009271	22	42	TCCCGTACTGAGCTGCCCCGAG	miRNA	miR-486-5p
t0009280	20	42	TCCTGTACTTAGCTGCCCCG	miRNA	miR-486-5p
t0009303	28	42	TCCTGTACTGAGCTGCCCCGAAGCTCGG	miRNA	miR-486-5p
t0009377	25	42	TCCTGTACTGAGCTGCCCCGAGTCC	miRNA	miR-486-5p
t0009253	27	42	TCCTGTACTGAGCTGCCCCGAGCTGCT	miRNA	miR-486-5p
t0009306	23	42	TCCTGTACTGAGCTGCCCCGAAG	miRNA	miR-486-5p
t0009418	26	42	TGTAGAGCAGGGAGCAGGAAGCTGAA	miRNA	miR-4732-5p
t0009435	23	42	ATTCGACTCTGGATAGCTGGTAT	miRNA	miR-4538
t0009460	21	42	AAACTTTACCATTACTGAGTT	miRNA	miR-451
t0009802	21	42	AAACCTTTACTATTACTGAGT	miRNA	miR-451
t0009619	23	42	TGGAGAGAAAGGCAGTAGTTGAT	miRNA	miR-4306
t0009522	22	42	TTGAGGGGCAGAGAGCGAGACT	miRNA	miR-423-5p
t0009532	24	42	TGACTGAGCGATTACTGGAGTTTT	miRNA	miR-3491
t0009564	22	42	AAAAGCTGGTTTGAGAGGGCGT	miRNA	miR-320d
t0009618	25	42	GAAAAGCTGGGTTGAGAGGGCGTAT	miRNA	miR-320d
t0009653	21	42	AAAAGCTGGGTTGAGAGGGTT	miRNA	miR-320d
t0009659	23	42	AAAAGCTGGGTTGAGAGGGAGTA	miRNA	miR-320d
t0009699	24	42	AAAGCTGGGTTGAGAGGGCGTATT	miRNA	miR-320d
t0009792	22	42	AAAAGCCGGGTTGAGAGGGCGA	miRNA	miR-320a
t0009524	23	42	TTCTGGGAGGTTGTAGCAGTGGA	miRNA	miR-3192
t0009683	22	42	TGTAAACATCCTACACTCAGCG	miRNA	miR-30b
t0009806	21	42	TAGCACCATTTGAAATCAGTG	miRNA	miR-29b
t0009837	24	42	TTCAAGTAATTCAGGATAGGTAGA	miRNA	miR-26b
t0009775	24	42	AATTCAAGTAATCCAGGATAGGCT	miRNA	miR-26a
t0009692	19	42	AGGCGGAGACTTGGGCAAT	miRNA	miR-25*
t0009673	22	42	CATTGCACTTGTCTCGGTCTCA	miRNA	miR-25
t0009517	20	42	TGGCTCAGTTCAGCAGGAAC	miRNA	miR-24
t0009542	24	42	TGGCTCAGTTCAGCAGGAACAGTG	miRNA	miR-24
t0009544	23	42	AAGCTGCCAGTTGAAGAACTGTT	miRNA	miR-22-3p
t0009549	25	42	TAGGTAGTTTCCTGTTGTTGGGATC	miRNA	miR-196b
t0009708	23	42	AACGGAATTCTAAAAGCAGCTGA	miRNA	miR-191
t0009742	20	42	CAAAGAATTCTCCTTTTGGA	miRNA	miR-186
t0009737	23	42	TGGAGAAAAAGGCAGTTCCTGAA	miRNA	miR-185
t0009644	21	42	TGGAGAGAAAGGCAGGTCCTG	miRNA	miR-185
t0009685	21	42	TGGAGAGAAAGGCAGTTCCCG	miRNA	miR-185
t0009694	20	42	TGGACGGAGAACTGATAGGG	miRNA	miR-184
t0009565	20	42	AACATTCAATGCTGTCGGTG	miRNA	miR-181a
t0009666	23	42	TAGCAGCACGTAAATATTTGGTT	miRNA	miR-16b
t0009815	20	42	GCAGCACGTAAATATTGGCA	miRNA	miR-16b
t0009671	20	42	TAGCAGCACTTCATGGTTTA	miRNA	miR-15b
t0009814	20	42	TAGCAGCACATCATGGTTAA	miRNA	miR-15b
t0009768	20	42	TAGCAGCACATCATGTTTTA	miRNA	miR-15b
t0009665	20	42	TAGCAGCACATCGTGGTTTA	miRNA	miR-15b
t0009612	20	42	TAGCAGCGCATAATGGTTTG	miRNA	miR-15a
t0009749	18	42	TCAGTGCACTACAGAACT	miRNA	miR-148a
t0009622	21	42	TACAGTATAGATGATGTTCTA	miRNA	miR-144
t0009660	18	42	TACAGTATAGATGATGTG	miRNA	miR-144
t0009767	21	42	TGTAGTGTTTCCTACTTTATG	miRNA	miR-142-3p
t0009552	23	42	TACCACAGGGTAGAATCACGGAT	miRNA	miR-140

t0009574	22	42	ACCACAGTGTAGAACCACGGAA	miRNA	miR-140
t0009623	22	42	CCACAGGGTAGAACCACGGAAG	miRNA	miR-140
t0009624	21	42	ACGACAGGGTAGAACCACGGA	miRNA	miR-140
t0009534	23	42	TACTACAGGGTAGAACCACGGAC	miRNA	miR-140
t0009558	20	42	ATTTTAGAGACGGGGTTTTG	miRNA	miR-1303
t0009787	22	42	TCCCTGAGACCCTTTAACCTGT	miRNA	miR-125a
t0009839	21	42	CGGATGAGCAAAGAAAGTGGT	miRNA	miR-1255b
t0009641	18	42	CGGGTTAGGTTCTTGTAG	miRNA	miR-125*
t0009834	22	42	AGCAGCATTGTACAAGGCTATG	miRNA	miR-103a
t0009604	22	42	AGCAGCATTGCACAGGGCTATG	miRNA	miR-103a
t0009629	21	42	AGCAGCATTGTACTGGGCTAT	miRNA	miR-103a
t0009700	24	42	TACAGTACTGTGATAACTGATGGC	miRNA	miR-101c
t0009563	21	42	TACAGTACTGCGATAACTGAA	miRNA	miR-101
t0009567	21	42	GTACAGTGTTGTGATAACTGA	miRNA	miR-101
t0009667	23	42	TGAGGTAGTAGTTTGTGCTGTAG	miRNA	let-7i
t0009821	22	42	TGAGGTAGTAGTTTGTGCTTTT	miRNA	let-7i
t0009832	26	42	CGAGGTAGTAGTTTGTACAGTTTATC	miRNA	let-7g
t0009621	22	42	TGAGGTAGTAGTTTATACAGTT	miRNA	let-7g
t0009723	25	42	TGAGGTAGTAGATTGTATAGTTGTC	miRNA	let-7f
t0009703	20	42	TGAGGTAGTAGAATGTATAG	miRNA	let-7f
t0009551	21	42	TGAGGTAGTAGAGTGTATAGT	miRNA	let-7f
t0009709	18	42	TGGTAGTAGATTGTATAG	miRNA	let-7f
t0009745	25	42	AGAGGTAGTAGGTTGCATAGTTATC	miRNA	let-7d
t0009805	22	42	AGAGGAAGTAGGTTGCATAGTT	miRNA	let-7d
t0009602	24	42	TGAGGTAGTAGGTTCTGTGGTTAA	miRNA	let-7b
t0009679	22	42	CTGAGGTAGTAGGTTGTGTGGT	miRNA	let-7b
t0009764	25	42	TGAGGTAGTAGGTTGTGTGGTAATC	miRNA	let-7b
t0009662	22	42	TGAGGTAATAGGTTGTATAGTT	miRNA	let-7
t0009830	22	41	AGGGACGGGACGCGGTGCAGTT	miRNA	miR-92b*
t0009507	19	41	TATTGCACTCGTCCCGGCA	miRNA	miR-92
t0009598	22	41	TGGAAGACTAGTGATTTTGTTG	miRNA	miR-7
t0009506	20	41	TACGTCATCATTGTCATCGT	miRNA	miR-598
t0009508	21	41	AAAAGTAATTGCGGTTTTTGA	miRNA	miR-548ak
t0009529	23	41	TCTGGGCGCAGGCGGATGGACAG	miRNA	miR-5107
t0009595	22	41	TCCTGTACTGAGCTGCTCCGAA	miRNA	miR-486-5p
t0009540	22	41	TCCTGTACTGAGCTGCCCCGGC	miRNA	miR-486-5p
t0009758	22	41	TCCTGTACTGAGCTGCCCCAAG	miRNA	miR-486-5p
t0009512	22	41	TCATGTACTGAGCTGCCCCGAG	miRNA	miR-486-5p
t0009702	23	41	TCCTGTACTGAGCTGCCCTGAGG	miRNA	miR-486-5p
t0009829	29	41	TCCTGTACTGAGCTGCCCCGAGACGACCT	miRNA	miR-486-5p
t0009800	20	41	GAGACGGGTCGGGGCGTGCG	miRNA	miR-4516
t0010144	23	41	TGAGGGAGTAGGTTGTGTGGTTT	miRNA	miR-4510
t0010072	22	41	AAACCGTTACCGTTACTGAGTT	miRNA	miR-451
t0009871	26	41	ACTCCCACTGCTTCACTTGACTAGCC	miRNA	miR-4301
t0010174	19	41	CAAAACGTGAGGCGCTGCT	miRNA	miR-424*
t0009952	22	41	TGAGGGACAGAGAGCGAGACTT	miRNA	miR-423-5p
t0010097	27	41	TGAGGGGCAGAGAGCGAGACTTTAGGT	miRNA	miR-423-5p
t0009858	23	41	TGAGGGGCCGAGAGCGAGACTTT	miRNA	miR-423-5p
t0009900	26	41	ACTGGACTTGGAGTCAGAAGGTAATC	miRNA	miR-378
t0010051	25	41	ACTGGACCTGGACTCAGAAGGCATC	miRNA	miR-378
t0010083	21	41	AATTGCACGGTATCCATCTTT	miRNA	miR-363
t0010093	21	41	TGAAGACTGAAGTGGAGAAGA	miRNA	miR-3526
t0010099	23	41	AAAAGCTGGGTTGAGAGGGGGAA	miRNA	miR-320d
t0009938	22	41	AAAAGCTGGGTTGAGAAGGCGA	miRNA	miR-320a
t0010175	21	41	AAAAGCTGGTTTGAGAGGGCG	miRNA	miR-320a
t0009991	24	41	AAAGCTGGGTTGAGAGGGCGAAAA	miRNA	miR-320a
t0009930	20	41	AGCTTTTGGGAATTCAGGTA	miRNA	miR-3140-3p
t0010025	26	41	TTCAAGTAATCCAGGATAGGCAGATC	miRNA	miR-26a

t0010029	20	41	TGGGTTCCTGGCATGCTGAT	miRNA	miR-23b*
t0010125	26	41	TTGGGGAAACGGCCGCTGAGTGAGAA	miRNA	miR-2110
t0010163	23	41	CTGTGCGTGTGACAGCGGCTGAA	miRNA	miR-210
t0010031	22	41	TACTGTAGTATGGGCACTTTTT	miRNA	miR-20b*
t0010078	23	41	TTCACCACCTTCTCCACCCAGCA	miRNA	miR-197
t0009911	23	41	AACGGAATCTCAAAAGCAGCTGA	miRNA	miR-191
t0009889	22	41	TGGAGAGAAAAGGAGTTCCTGA	miRNA	miR-185
t0009925	23	41	TGGAGAGAAAGGAAGTTCCTGAG	miRNA	miR-185
t0009874	21	41	TGGAGAGAAAGGTAGTTCCTG	miRNA	miR-185
t0010037	20	41	AGCAGCACATAATGGTTTGT	miRNA	miR-15a
t0010090	24	41	AAATAGCAGCACATAATGGTTTGT	miRNA	miR-15a
t0010095	20	41	TAGCAGTACATAATGGTTTG	miRNA	miR-15a
t0010080	22	41	TCAGTGCATGACAGAACTTGGA	miRNA	miR-152
t0009855	20	41	TCTCAACCCTTGTACCAGTG	miRNA	miR-150
t0010055	20	41	CCCATAAAGTAGAAAGCCCT	miRNA	miR-142
t0010004	21	41	TACCACAGGGTAGAACCAGGA	miRNA	miR-140
t0009861	23	41	TACCAGAGGGTAGAACCACGGAC	miRNA	miR-140
t0009881	25	41	ACCACAGGGTAGAACCACGGACGGC	miRNA	miR-140
t0010180	21	41	TACCACAGCGTAGAACCACGG	miRNA	miR-140
t0009863	22	41	ACCACAGGGTAGAACCAGGGAA	miRNA	miR-140
t0009950	21	41	ACCACAGGGTAGAGCCACGGA	miRNA	miR-140
t0009989	22	41	TACCACAGGGTAGAAATACGGA	miRNA	miR-140
t0009877	24	41	ACCACAGGGTAGAACCACGGAGAA	miRNA	miR-140
t0010014	23	41	TCCCTGAGACCCTAACTTGTGAA	miRNA	miR-125b-5p
t0010028	23	41	CCGCACTGTGGGTACTTGCTGCA	miRNA	miR-106b*
t0010011	21	41	ACCCTGTAGATCCGAATTTGT	miRNA	miR-10-5p
t0009957	22	41	AGCAGCATTGTAAAGGGCTATG	miRNA	miR-103a
t0010102	22	41	AGCAGCATTGTACAGGGCTCTG	miRNA	miR-103a
t0009867	22	41	ACAGTACTGTGATAACTGAAGA	miRNA	miR-101c
t0009878	21	41	ACAGTACTGTGATAACTGAAA	miRNA	miR-101c
t0009993	21	41	GTACAGTACTTTGATAACTGA	miRNA	miR-101
t0010036	29	41	GTACAGTACTGTGATAACTGAACTCTGAA	miRNA	miR-101
t0010128	22	41	GTATAGTACTGTGATAACTGAA	miRNA	miR-101
t0010130	22	41	TGCGGTAGTAGTTTGTACAGTT	miRNA	let-7g
t0010145	20	41	TGAGGTAGTAGATTGTATCT	miRNA	let-7f-5p
t0010146	18	41	AGGTAGTAGATTGTATAG	miRNA	let-7f
t0009951	20	41	AGGTAGTAGATTGTATAGTG	miRNA	let-7f
t0010166	21	41	TGAGGTAGTAGATTGTACGGT	miRNA	let-7f
t0010139	18	41	GAGGTAGTAGGTTGCATA	miRNA	let-7d
t0009954	27	41	TGAGGTAGTAGGTTGTGTGGTTTTATC	miRNA	let-7b
t0010016	21	41	TTGAGGTAGTAGGTTGTGTGG	miRNA	let-7b
t0009849	22	41	TGAGGTAGTAGGTTGTGGGGTA	miRNA	let-7b
t0010106	24	40	AACCCGTAGATCCGATCTTGTATC	miRNA	miR-99a
t0009984	21	40	TATACAACTTACTACTTTCCG	miRNA	miR-98*
t0010069	22	40	TATTGCACTTGTCCCGGCCAAA	miRNA	miR-92
t0010152	22	40	TCCATTACACTACCCTGCCTCT	miRNA	miR-885-5p
t0009979	20	40	TGCGGGGCTAGGGCTAACAG	miRNA	miR-744
t0009972	22	40	GAAGACTGAAGTGGATAAGGGT	miRNA	miR-739
t0009915	22	40	TGAAAGACGATGGTAGTGAGAT	miRNA	miR-71
t0009919	22	40	ATTATGGTTTGCCTGGGACTGA	miRNA	miR-584
t0010027	20	40	CAAAAGTGATCGTGGTTTTT	miRNA	miR-548t
t0010109	20	40	CTCCCCACACCCAAGGCTTG	miRNA	miR-532-3p
t0009880	22	40	TCCTGTACTGAACTGCCCCGAG	miRNA	miR-486-5p
t0010012	23	40	TCCTGTACTGAGCTGACCCGAGA	miRNA	miR-486-5p
t0010183	23	40	TCCTGTACTGAGCTGTTCCGAGT	miRNA	miR-486-5p
t0010511	26	40	TCCTGTACTGAGCTGCCCCGAGTTAG	miRNA	miR-486-5p
t0010215	21	40	TGACGTCACAGAGGCTTCGCG	miRNA	miR-4757-3p
t0010388	24	40	AAACCGTTACCATTACTGAGTCGG	miRNA	miR-451

t0010516	22	40	AACCGTTACCATTACTGAGTTA	miRNA	miR-451
t0010416	22	40	AAACCGTTACCATTGCTGAGTT	miRNA	miR-451
t0010308	19	40	AAATCGTTACCATTACTGA	miRNA	miR-451
t0010338	19	40	CTCCCACTGCTTCACTTGA	miRNA	miR-4301
t0010393	19	40	TGAGGGGCAGACAGCGAGA	miRNA	miR-423-5p
t0010246	21	40	TGAGGGGCAAAGAGCGAGACT	miRNA	miR-423-5p
t0010351	22	40	TTAGGTAGTAGTTTGTACAGTT	miRNA	miR-3962
t0010207	21	40	AGGGACTTTCAGGGGCAGCTG	miRNA	miR-365*
t0010244	26	40	CTTTGAGGACTGAAGTGGGGAAAGGT	miRNA	miR-3526
t0010405	23	40	TCTCACACAGAAATCGCCCCCGT	miRNA	miR-342-3p
t0010478	22	40	TCTCACACAGAAATCGCCCCCG	miRNA	miR-342-3p
t0010242	24	40	TTCCACACAGAAATCGCACCCGTC	miRNA	miR-342-3p
t0010196	21	40	AAAAGCTGGGTTGAGAGGGAT	miRNA	miR-320d
t0010270	23	40	AAAAGCTGGGTTGAGATGGCGTT	miRNA	miR-320d
t0010331	24	40	AAAAGCTGGGTTGAGATGGCGAAA	miRNA	miR-320a
t0010369	22	40	AAAAGCTGGGTCGAGAGGGCGA	miRNA	miR-320a
t0010394	21	40	TCTGGGAGGTTGTAGCAGTGG	miRNA	miR-3192
t0010190	23	40	TAGCACCATTTGAAATCAGTGTT	miRNA	miR-29b
t0010377	23	40	CATTGCACTTGTCTCGGTCTGGA	miRNA	miR-25
t0010306	27	40	CATTGCACTTGTCTCGGTCTGGCGGGC	miRNA	miR-25
t0010265	23	40	TGGCTCAGTTCAGCAGGAACATC	miRNA	miR-24b
t0010238	22	40	GCTCAGTTCAGCAGGAACAGGA	miRNA	miR-24b
t0010197	20	40	TGAGGTAGGTTGTGTGGTAA	miRNA	miR-1961
t0010381	22	40	CAACGGAATCCAAAAAGCAGCT	miRNA	miR-191
t0010460	23	40	CAACGGATTCCCAAAAGCAGCTG	miRNA	miR-191
t0010230	20	40	TGGATAGAAAGGCAGTTCCT	miRNA	miR-185
t0010358	19	40	TGGAGAGAAAGGCAGTTTG	miRNA	miR-185
t0010352	23	40	TGGAGAGAAAGGCAGTTCCCGAT	miRNA	miR-185
t0010454	24	40	ATAGCAGCACATCATGGTTTACAA	miRNA	miR-15b
t0010259	24	40	TAGCAGCACATCATGGTTTAAAAA	miRNA	miR-15b
t0010314	21	40	TAGCAGCACATAATGGTTGTG	miRNA	miR-15a
t0010293	25	40	TCAGTGCACTACAGAACTTTGGTAG	miRNA	miR-148a
t0010501	21	40	TACAGTATAGATGATGTTTTT	miRNA	miR-144
t0010375	22	40	TACCACAGGGTAGAACCACGCA	miRNA	miR-140
t0010380	23	40	CCACAGGGTAGAACCACGGAGGA	miRNA	miR-140
t0010299	22	40	TACCACAGGGTAGAGCCACGGA	miRNA	miR-140
t0010237	22	40	ACCACAGGGTAGAAACACGGAA	miRNA	miR-140
t0010323	22	40	AGCCTGGAAGCTGGAGCCTGCA	miRNA	miR-1254
t0010210	21	40	CCTTCCCCACCCTCTCCTGCA	miRNA	miR-1224-3p
t0010297	25	40	AGCAGCATTGTACAGGGCTATGTAA	miRNA	miR-103a
t0010428	22	40	AAAGCAGCATTGTACAGGGCTA	miRNA	miR-103a
t0010239	24	40	GTACAGTACTGTGATAACTGAAAA	miRNA	miR-101
t0010400	22	40	TGAGGTAGTGGTTTGTACAGTT	miRNA	let-7g
t0010203	19	40	TGAGGTAGTAGATTGTCTA	miRNA	let-7f
t0010220	20	40	AGAGGTAGTTGGTTGCATAG	miRNA	let-7d
t0010452	20	40	TGAGGTAGCAGGTTGTGTGG	miRNA	let-7b
t0010349	24	40	TGAAGTAGTAGGTTGTGTGGTTGA	miRNA	let-7b
t0010366	21	40	TGAGGTAGTCGGTTGTATAGT	miRNA	let-7
t0010373	20	39	TATACAACTTACTACTTTCC	miRNA	miR-98*
t0010487	23	39	TATTGCACTTGTCCCGGCCTGGA	miRNA	miR-92b-3p
t0010430	22	39	AGAGGAGATGGCGCAGGGGACA	miRNA	miR-877
t0010459	26	39	AAGTTGGATTGTGACTGGCATTTTTA	miRNA	miR-753c
t0010475	22	39	CCTCCCACACTCAAGGCTTGCA	miRNA	miR-532-3p
t0010490	20	39	TAGCAGCGGGAACAGTTTTG	miRNA	miR-503
t0010434	24	39	TCCTGTACTGAGCTGCCCCGAGTA	miRNA	miR-486-5p
t0010324	23	39	TCCTGTACTGAGCTGCCCCGAGC	miRNA	miR-486-5p
t0010426	19	39	TCCTGTACTGAGCTGCCGA	miRNA	miR-486-5p
t0010370	20	39	CGGGGCAGCTCAGTACAGGA	miRNA	miR-486-3p

t0010267	21	39	ATGCGGACCTGGGTTAGCGGA	miRNA	miR-4754
t0010359	22	39	TCTCCCTTCCTGCCCTGGCTAG	miRNA	miR-4685-3p
t0010296	23	39	AAGGGCTGGGTCGGTCGGGCTGG	miRNA	miR-4651
t0010868	20	39	AAAACGTTACCATTACTGAG	miRNA	miR-451
t0010859	22	39	AAACCGATACCATTACTGAGTT	miRNA	miR-451
t0010809	21	39	AAACCGTTGCCATTACTGAGT	miRNA	miR-451
t0010693	20	39	AAACCCTCACCATTACTGAG	miRNA	miR-451
t0010569	22	39	AAAAGCTGGGCTGAGAGGGCGA	miRNA	miR-4429
t0010727	19	39	TGAGGGTCAGAGAGCGAGA	miRNA	miR-423-5p
t0010556	22	39	TTTGAGGGGCAGAGAGCGAGAC	miRNA	miR-423-5p
t0010561	23	39	TGAGGGGCAGAGAGCGAGCCTTA	miRNA	miR-423-5p
t0010713	22	39	TGAGGGTCAGAGAGCGAGACTT	miRNA	miR-423-5p
t0010573	22	39	ATCAACAGACATTAATTGGGCG	miRNA	miR-421
t0010818	28	39	GAAACGGACAAGGGGAATCCGACTGTTT	miRNA	miR-3896-3p
t0010851	24	39	TTGAAGATTGAAGTGGAGAAGGGT	miRNA	miR-3526
t0010734	22	39	GCCCCTGGGCCTATCCTAGAAA	miRNA	miR-331
t0010566	20	39	CCACTGCCCCAGGTGCTGCT	miRNA	miR-324-3p
t0010843	23	39	AAAAGCTGGGTTGAGAGGGCTAA	miRNA	miR-320d
t0010880	21	39	AAGCTGGGTTGAGAGGGCGTA	miRNA	miR-320d
t0010891	24	39	AAAAGCTGGGTTGAGAGGGCAATA	miRNA	miR-320b
t0010752	21	39	AAAAGCTAGGTTGAGAGGGCG	miRNA	miR-320a
t0010562	22	39	AAAAGCTGGGTTGTGAGGGCGA	miRNA	miR-320a
t0010748	24	39	AAAAGCTGGGTTGAGAGGGCGAGC	miRNA	miR-320a
t0010758	23	39	AAAAGCTGTGTTGAGAGGGCGAA	miRNA	miR-320a
t0010819	25	39	AAAAGCTGGGTTGAGAGGGCGAAGA	miRNA	miR-320a
t0010598	23	39	AAAAGCTGGTTTGAGAGGGCGAT	miRNA	miR-320a
t0010779	24	39	AATCTGAGAAGGCGCACAAGGTAA	miRNA	miR-3200-5p
t0010586	22	39	GTGGAGTCCTGGGGAATGGAGA	miRNA	miR-3198
t0010541	22	39	CACAGCAAGTGTAGACAGGCAA	miRNA	miR-3120-3p
t0010564	27	39	TGTAAACATCCCCGACTGGAAGCTATC	miRNA	miR-30d
t0010724	23	39	CGCGGGTCGGGGGGCGGGGCGGA	miRNA	miR-2981
t0010829	21	39	TCCAAGTAATTCAGGATAGGT	miRNA	miR-26b
t0010804	24	39	CTGACCTATGAATTGACAGCCAAA	miRNA	miR-192
t0010587	25	39	CTGACCTATGAATTGACAGCCAGAA	miRNA	miR-192
t0010642	23	39	CAACGGAATCCTAAAAGCAGCTG	miRNA	miR-191
t0010725	20	39	CAAAGAATTTTCCTTTTGGG	miRNA	miR-186
t0010736	21	39	TGGAGAGAAAGGCAGGCCCTG	miRNA	miR-185
t0010768	20	39	TGGAGAGAATGGCAGTTCCT	miRNA	miR-185
t0010793	23	39	TGGAGAGAAAGGCAGTTTTTGAG	miRNA	miR-185
t0010687	24	39	AACATTCATTGCTGTCGGTGGATC	miRNA	miR-181b
t0010532	26	39	TAGCAGCACGTAAATATTGCTAGTGT	miRNA	miR-16b
t0010660	23	39	ATAGCAGCACGTAAATATTGGCA	miRNA	miR-16b
t0010613	21	39	TAGCAGCACATAATGGATTGT	miRNA	miR-15a
t0010692	24	39	TACTAGACTGAAGCTCCTTGAGGA	miRNA	miR-151-3p
t0010790	19	39	TACAGTATAGATGATGTTT	miRNA	miR-144
t0010764	23	39	GTAGTGTTTCCTACTTTATGGCA	miRNA	miR-142-3p
t0010872	21	39	ACCACAGGGTAAAACCACGGA	miRNA	miR-140
t0010666	23	39	TACCACAGGGTAGAACCACCGAC	miRNA	miR-140
t0010803	23	39	ACCACATGGTAGAACCACGGACG	miRNA	miR-140
t0010585	24	39	ACCACAGGGTAGAATTACGGAAGG	miRNA	miR-140
t0010707	23	39	TACCACAGGGTAGATCCACGGAA	miRNA	miR-140
t0010882	22	39	ACCACAAGGTAGAACCACGGAA	miRNA	miR-140
t0010881	22	39	CATTATTACTTTTGGTACGCGT	miRNA	miR-126-5p
t0010807	22	39	TACCCTGTAGATCCGAATTTGT	miRNA	miR-10a
t0010817	20	39	TAAAGTGCTGACAGTGTAGA	miRNA	miR-106b
t0010708	24	39	ATAAAGTGCTGACAGTGCAGATAA	miRNA	miR-106b
t0010695	21	39	AGCAGCATTGTACAAGGCTAT	miRNA	miR-103a
t0010640	23	39	AGCAGAATTGTACAGGGCTATGA	miRNA	miR-103a

t0010593	21	39	AGCAGAATTGTACAGGGCTAT	miRNA	miR-103a
t0010534	23	39	GTACAGTACTGTGATAACTGAAT	miRNA	miR-101
t0010567	26	39	TACAGTACTGTGATAACTGAAGGCGC	miRNA	miR-101
t0010616	21	39	TGAGGGAGTAGTTTGTGCTGT	miRNA	let-7i
t0010699	22	39	TGAGGTAGTAGTTTGTTCTGTT	miRNA	let-7i
t0010852	22	39	GAGGTAGTAGATTGTATAGTTG	miRNA	let-7f
t0010893	24	39	TGAGGTAGTAGATTGTATAGTCAA	miRNA	let-7f
t0010612	20	39	AGAAGTAGTAGGTTGCATAG	miRNA	let-7d
t0010629	21	39	AAGAGGTAGTAGGTTGCATAG	miRNA	let-7d
t0010737	20	39	TAGGTAGTAGGTTGTGTGGT	miRNA	let-7b
t0010772	20	39	TGAGGTAGTAGGTTGCGTGG	miRNA	let-7b
t0010540	26	39	TGAGGTAGTAGGTTGTGTGGTTAGAT	miRNA	let-7b
t0010728	24	39	TGAGGTAGTAGGTTGTATGGAATC	miRNA	let-7-5p
t0010886	19	39	AGGTAGTAGGTTGTATAGT	miRNA	let-7
t0010589	23	38	CAGGTGGAAGGTTGTAGGCATGT	miRNA	miR-965-5p
t0010698	26	38	CAAAGTGCTGTTCGTGCAGGTAGATC	miRNA	miR-93
t0010580	23	38	TATTGCACTCGTCCCGGCCTAAG	miRNA	miR-92
t0010814	21	38	TATTGCACTCGTCCCGGCCAA	miRNA	miR-92
t0010606	19	38	GGGCAGGTGGTGTAGTGGT	miRNA	miR-885
t0010732	22	38	CGGGGCTAGGGCTAACAGCAGA	miRNA	miR-744
t0010658	23	38	TATTCATTGCATATCGGAGTTGT	miRNA	miR-660
t0010822	22	38	CCTCCCACACCCAAGGCTTGCC	miRNA	miR-532-3p
t0010568	27	38	TCCTGTACTGAGCTGCCCCGACGCTAC	miRNA	miR-486-5p
t0010766	21	38	TCCTGCACTGAGCTGCCCCGA	miRNA	miR-486-5p
t0010825	21	38	TCCTGTACTGCGCTGCCCCGA	miRNA	miR-486-5p
t0010604	22	38	CTCTGTACTGAGCTGCCCCGAG	miRNA	miR-486-5p
t0010781	23	38	TCCTGTACTGAGCTGCCCCGCGT	miRNA	miR-486-5p
t0010907	20	38	AAGGTATTGTTCAGACTTAT	miRNA	miR-4678
t0011054	19	38	AACCTTTACCATTACTGAG	miRNA	miR-451
t0010930	19	38	AAACATTACCATTACTGAG	miRNA	miR-451
t0011191	23	38	TTCGGGGTCTGGGCGCGGCGAGA	miRNA	miR-4508
t0010909	22	38	TGAGGGGCAGGGAGCGAGACTT	miRNA	miR-423-5p
t0011164	23	38	CTGAGGGGCAGAGAGCGAGACTT	miRNA	miR-423-5p
t0011043	22	38	ATATAATACAATCTGCTAAGTG	miRNA	miR-374
t0010943	22	38	ATATAATATAACCTGCTAAGTG	miRNA	miR-374
t0010962	21	38	ACTGCCCCAGGTGCTGCTGGT	miRNA	miR-324-3p
t0011031	20	38	AAAAGCTGGGTTGAGAGGCG	miRNA	miR-320d
t0011046	22	38	AAAAGCTGAGTTGAGAGGGCGT	miRNA	miR-320d
t0011113	22	38	AAAAGCTGGGTTGAGAGGGAAT	miRNA	miR-320d
t0011162	23	38	TGTAAACATCCTACACTCTCAGC	miRNA	miR-30c
t0011261	27	38	CGCGGGTCGGGGGGCGGGGCGGACTGT	miRNA	miR-2981
t0011134	22	38	CATTGCACTTGTCTCAGTCTGA	miRNA	miR-25
t0011265	24	38	TGGCTCAGTTCAGCAGGAACAGAA	miRNA	miR-24b
t0011058	22	38	AGCTACATTGTCTGCTGGGTTA	miRNA	miR-221
t0011122	20	38	AGTTCTTCAGTGGCAAGCTT	miRNA	miR-22*
t0011168	20	38	GACTGGGGCGGGACATCTGT	miRNA	miR-219-2-3p
t0010945	25	38	TGGAATGTAATGAAGTGTGTGGATC	miRNA	miR-206
t0011047	22	38	TTGAGAGAAAGGCAGTTCCTGA	miRNA	miR-185
t0011232	23	38	TTGGAGAGAAAGGCAGTTCCTGA	miRNA	miR-185
t0011008	22	38	TGGAGAGAAAGGCAGTTCCTAT	miRNA	miR-185
t0011142	19	38	TAGAGAAAGGCAGTTCCTG	miRNA	miR-185
t0011145	23	38	ATGGCACTGGTAGAATTCACTGA	miRNA	miR-183
t0011044	23	38	TGAGGGAGTAGATTGTATAGTTT	miRNA	miR-1827
t0011343	23	38	AACATTCATTGTTGTCGGTGGGT	miRNA	miR-181d
t0011130	18	38	TCGGGGCGGCGGCGGCGG	miRNA	miR-1777a
t0010905	23	38	TAGCAGCACGTAAATATTGGGGT	miRNA	miR-16
t0011067	22	38	TAGCAGCAGGTAAATATTGGCG	miRNA	miR-16
t0010931	22	38	TAGCAGCACATCATGGTTATCA	miRNA	miR-15c

t0011034	21	38	TAGCAGCACATCATGGGTTAC	miRNA	miR-15b
t0011186	20	38	TAGCAGCACATCATGGTTGA	miRNA	miR-15b
t0011041	20	38	TATCAGCACATAATGGTTTG	miRNA	miR-15a
t0011060	21	38	TAGCAGCACATAATCGTTTGT	miRNA	miR-15a
t0011230	22	38	AATTACAGTATAGATGATGTAC	miRNA	miR-144
t0011236	22	38	TACCACAGGGTAGAACCCCGGC	miRNA	miR-140
t0011099	19	38	ACCACAGGGTAGAACCACG	miRNA	miR-140
t0011163	24	38	TACCACAGGGTAGAACTACGGACG	miRNA	miR-140
t0011004	20	38	ACCACAGGATAGAACCACGG	miRNA	miR-140
t0011308	23	38	TACCACAGGGTAGAACTACGGAG	miRNA	miR-140
t0010954	22	38	CCACAGGGTAGAACCACGGACG	miRNA	miR-140
t0010994	21	38	TCACAGTGAACCGGTCTCTTG	miRNA	miR-128
t0010992	23	38	CATTATTACTTTTGGTACGCGAA	miRNA	miR-126-5p
t0011211	22	38	AGCAGCATTGCACAGGGCTATT	miRNA	miR-107
t0011262	23	38	TACCGCACTGTGGGTACTTGCTA	miRNA	miR-106b*
t0010896	23	38	AGCAGTATTGTACAGGGCTATGT	miRNA	miR-103a
t0011339	25	38	TACAGTACTGTGATAACTGACTATC	miRNA	miR-101c
t0011128	24	38	GTACAGTACTGTGATAACTGACGG	miRNA	miR-101c
t0010939	22	38	TACAGTACTCTGATAACTGAAG	miRNA	miR-101
t0011083	21	38	TGAGGTAGTAGTTTGTGCTTG	miRNA	let-7i
t0011176	20	38	TGAGGTAGTGGTTTGTACAG	miRNA	let-7g
t0011187	23	38	TGAGGTATTAGATTGTATAGTTG	miRNA	let-7f
t0011304	22	38	AGGGGTAGTAGGTTGCATAGTT	miRNA	let-7d
t0011346	22	38	AGAGTTAGTAGGTTGCATAGTT	miRNA	let-7d
t0010990	21	38	AGAGGTAGTAGGCTGCATAGT	miRNA	let-7d
t0011116	20	38	AGGTAGTAGGTTGCATAGTT	miRNA	let-7d
t0011139	22	38	AGAGGTAGTAGGTTGCATAGGT	miRNA	let-7d
t0011326	19	38	AGAGGTAGTAGGTTGCAGT	miRNA	let-7d
t0010985	21	38	AGAGGTAGTAGGTTCCATAGT	miRNA	let-7d
t0010925	22	38	AGAGGTAGTAGGTTGTGTGGTT	miRNA	let-7b
t0011190	23	38	TGAGGTAGTAGGTTGTGGGGTTA	miRNA	let-7b
t0011286	23	38	TGAGGTAGGAGGTTGTATAGTTA	miRNA	let-7
t0011040	22	38	TGAGATAGTAGGTTGTATAGTT	miRNA	let-7
t0010961	22	38	TGAGGTAGTAGGTTGTCTAGTT	miRNA	let-7
t0011220	21	37	AGAGGAGATGGCGCAGGGGAA	miRNA	miR-877
t0010953	21	37	AGAGGAGATGGCGCAGGGGAC	miRNA	miR-877
t0011093	21	37	AAGTTGGATTGTGACTGGCCT	miRNA	miR-753c
t0011108	28	37	TGGTGCGGACCAGGGGAATCCGACTGTT	miRNA	miR-5109
t0011171	21	37	TCCTGTACTGAGTCGCCCCGA	miRNA	miR-486-5p
t0011260	29	37	TCCTGTACTGAGCTGCCCCGAGCTCGGCA	miRNA	miR-486-5p
t0011325	25	37	TCCTGTACTGAGCTGCCCCGAGGAA	miRNA	miR-486-5p
t0011341	21	37	TCCCGTACTGAGCTGCCCCGA	miRNA	miR-486-5p
t0011148	29	37	TCCTGTACTGAGCTGCCCCGAGGCGGCA	miRNA	miR-486-5p
t0011342	24	37	TGTAGAGCAGGGAGCAGGAAGTAA	miRNA	miR-4732-5p
t0011003	28	37	AGGGCTGGGTCGGTCGGGCTGGGGCGAT	miRNA	miR-4651
t0011068	24	37	AAACCGTTATTATTACTGAGTTTA	miRNA	miR-451
t0011124	30	37	AAACCGTTACCATTACTGAGTTCGGCACC	miRNA	miR-451
t0011353	23	37	AAACCGTTACCATTACTGCGTTT	miRNA	miR-451
t0011513	27	37	CCACAAACCGTTACCATTACTGAGTTT	miRNA	miR-451
t0011642	23	37	AAATTGTTACCATTACTGAGTTA	miRNA	miR-451
t0011561	21	37	AAACCGTTACCATTACTGATT	miRNA	miR-451
t0011420	22	37	TGGAGAGAAAGGCAGTAGTTGT	miRNA	miR-4306
t0011579	21	37	AGGGGCAGAGAGCGAGACTTA	miRNA	miR-423-5p
t0011625	21	37	TGGACTCGCAGGAACAGGTCT	miRNA	miR-4001b-5p
t0011710	23	37	CCCTCGAACTGTTGTGGCCATTT	miRNA	miR-3487
t0011711	24	37	TCACACAGAAATCGCACCCGTCAT	miRNA	miR-342-3p
t0011542	22	37	CTTCACACAGAAATCGCACCCG	miRNA	miR-342-3p
t0011470	22	37	AAAAGCTGGATTGAGAGGGCGT	miRNA	miR-320d

t0011371	23	37	AAAAGTTGGGTTGAGAGGGCGAC	miRNA	miR-320a
t0011464	23	37	TTTCAGTCGGATGTTTACAGTTC	miRNA	miR-30b-3p
t0011632	19	37	TTCAAGTAATCCAGGATGC	miRNA	miR-26a
t0011679	24	37	GGCTCTAAGGGCTGGGTCGGTCGG	miRNA	miR-2487
t0011683	20	37	TGACCTATGAATTGACAGTC	miRNA	miR-192
t0011751	22	37	AACGGAATCCCAAAAGCAGCTA	miRNA	miR-191
t0011730	22	37	TAAGGTGCATCTAGTGCAGTTA	miRNA	miR-18b
t0011433	23	37	TGTAGAGAAAGGCAGTTCCTGAA	miRNA	miR-185
t0011479	22	37	TGGAGAGAAAGGAAGTTACTGA	miRNA	miR-185
t0011588	24	37	TTTGGCAATGGTAGAATTCACACT	miRNA	miR-182
t0011418	22	37	TTGGTGTATGTGCTTGGCTATC	miRNA	miR-1755
t0011720	22	37	TCAAAGTGCTTACAGTGCAGGT	miRNA	miR-17
t0011681	22	37	ACAAAGTGCTTACAGTGCAGGT	miRNA	miR-17
t0011680	25	37	TAGCAGCACGTAAATATTGGCAGAT	miRNA	miR-16b
t0011384	23	37	TAGCAGCACGTAAATATTGGCCT	miRNA	miR-16b
t0011523	21	37	TAGCAGCATATCATGGTTTAC	miRNA	miR-15b
t0011709	22	37	TAGAAGCACATCATGGTTTACA	miRNA	miR-15b
t0011417	19	37	TAGCAGCACATCATGTTTA	miRNA	miR-15b
t0011776	19	37	TAGCAGCACATCATGCTTT	miRNA	miR-15b
t0011707	20	37	TACAGTATAGATGATGTAAA	miRNA	miR-144
t0011448	23	37	TGAGATGAAGCACTGTAGATATC	miRNA	miR-143
t0011753	23	37	ACCACAGGGTAGAACCATGGACG	miRNA	miR-140
t0011510	22	37	ACCACATGGTAGAACCACGGAA	miRNA	miR-140
t0011456	21	37	AACACAGGGTAGAACCACGGA	miRNA	miR-140
t0011526	22	37	ACCACAGGGAAGAACCACGGAA	miRNA	miR-140
t0011645	22	37	AGCTTCTTTACAGTGCTGCCTT	miRNA	miR-103a-2*
t0011388	22	37	AGCAGCATTGTACACGGCTATG	miRNA	miR-103a
t0011536	20	37	AGCAGCATTGGACAGGGCTA	miRNA	miR-103a
t0011358	22	37	GGCAGCATTGTACAGGGCTATG	miRNA	miR-103a
t0011501	22	37	AGCAGCATTGTTCAGGGCTATG	miRNA	miR-103a
t0011615	22	37	TACAGTATCGTGATAACTGAAG	miRNA	miR-101
t0011712	21	37	TACAGTACTGTGATTACTGAA	miRNA	miR-101
t0011401	22	37	TGAGGTAGTAGTTCGTGTGGTT	miRNA	let-7h
t0011361	20	37	CAGGTAGTAGATTGTATAGT	miRNA	let-7f
t0011528	20	37	TGTGGTAGTAGATTGTATAG	miRNA	let-7f
t0011540	27	37	TGAGGTAGTAGATTGTATAGTGGCCGA	miRNA	let-7f
t0011670	26	37	TGAGGTAGTAGATTGTATAGTTGAAA	miRNA	let-7f
t0011732	20	37	TAGTAGTAGATTGTATAGTT	miRNA	let-7f
t0011515	27	37	TGAGGTAGTAGATTGTATAGTCCTGTT	miRNA	let-7f
t0011356	22	37	TGAGGTAGTAGGTGGTGTGGTA	miRNA	let-7b
t0011537	19	37	TGAGGTAGTAGGTTGTGTA	miRNA	let-7b
t0011605	22	37	GAGGTAGTAGGTTGCATAGTAA	miRNA	let-7-5p
t0011639	20	37	TGGGGTAGTAGGTTGTATAG	miRNA	let-7
t0011435	20	37	TGAGGTAGTAAGTTGTATAG	miRNA	let-7
t0011749	21	37	TGAGGTAGTAGGTTGTCTAGT	miRNA	let-7
t0011653	20	36	TATACAACTTACTACTTTCA	miRNA	miR-98*
t0011549	24	36	AGGTGGAAGGTTGTAGGCATCTAT	miRNA	miR-965-5p
t0011610	23	36	TATTGCACTCGTCCCGGCCTCCA	miRNA	miR-92
t0011627	23	36	TATTGCACTCGTCCCGGCCAATT	miRNA	miR-92
t0011674	28	36	TCCTGTACTGAGCTGCCCCGAGGCGGCA	miRNA	miR-486-5p
t0011697	19	36	TCCTGTACTGAGCTGCCTC	miRNA	miR-486-5p
t0011739	20	36	ATCTGTACTGAGCTGCCCCG	miRNA	miR-486-5p
t0011430	21	36	TTGTACTGAGCTGCCCCGAGT	miRNA	miR-486-5p
t0011766	23	36	TCCTGTACTGAGCCGCCCCGAGA	miRNA	miR-486-5p
t0011383	21	36	CTGTACTGAGCTGCCCCGAGA	miRNA	miR-486-5p
t0011399	19	36	AACAGGACGTCACAGAGGG	miRNA	miR-4757-3p
t0011551	21	36	TGAGGGAAGGAGGCTTGGTCT	miRNA	miR-4747-5p
t0011760	23	36	TCTCCCTTCCTGCCCTGGCTTAA	miRNA	miR-4685-3p

t0011929	24	36	TAGTGCAATATTGCTTATAGGGTT	miRNA	miR-454
t0012094	23	36	AAACCGTTACCATTACTGAGATT	miRNA	miR-451
t0011922	22	36	AAACGTTACCATTACTGAGTTT	miRNA	miR-451
t0012216	19	36	AAACCGTTACTATTACTGA	miRNA	miR-451
t0011846	22	36	GAACCGTTACCATTACTGAGTT	miRNA	miR-451
t0011911	22	36	AAACCGTTACCATCATTGAGTT	miRNA	miR-451
t0011926	22	36	TTCGGGGTCTGGGCGCGGCGAT	miRNA	miR-4508
t0011950	22	36	TCGGATCCGAGTCACGGCACCA	miRNA	miR-4454
t0011981	20	36	TTGGAGGAGATGCGGTTATT	miRNA	miR-4443
t0012117	24	36	ACTCCCACTGCTTCACTTGACTAG	miRNA	miR-4301
t0012219	23	36	TGAGGGGGAGAGAGCGAGACTTT	miRNA	miR-423-5p
t0012109	22	36	TGAGGGGCAGAGAGCGTGACTT	miRNA	miR-423-5p
t0011810	22	36	TGAGGGGCATAGAGCGAGACTT	miRNA	miR-423-5p
t0012019	23	36	TGAGGGGCAGAGAGTGAGACTTA	miRNA	miR-423-5p
t0012078	23	36	TGGACTCGCAGGAACAGGCCTAT	miRNA	miR-4001b-5p
t0012091	21	36	ACTGGACTGGGAGTCAGAAGG	miRNA	miR-378
t0012121	25	36	ACTGGACTTGGTGTCAGAAGGCATC	miRNA	miR-378
t0012136	20	36	ATTGCACGGTATCCATCTGT	miRNA	miR-363
t0012164	26	36	TGAAGACTGAAGTGGAGAAGGGTTTC	miRNA	miR-3526
t0012139	21	36	CGCCCTCGAACTGTTGTGGCA	miRNA	miR-3487
t0011964	21	36	TCCTCGAACTGTTGTGGCCAT	miRNA	miR-3487
t0012124	22	36	ATGCCTGACCACCGTCTACCTT	miRNA	miR-3483-3p
t0011833	23	36	AAAAGCTGGGTTGAGAGGGCCAT	miRNA	miR-320d
t0011878	21	36	CTTTCAGTCGGATGTTTACAG	miRNA	miR-30e*
t0011915	21	36	GTAAACATCCTTGACTGGAAG	miRNA	miR-30e
t0011928	26	36	TGTAAACATCCTACACTCTCAGCATC	miRNA	miR-30c
t0012088	22	36	TTTCAGTCGGATGTTTTACAGC	miRNA	miR-30b-3p
t0011840	22	36	TTTTCAGTCGGATGTTTACAGT	miRNA	miR-30b-3p
t0011996	24	36	TTCAAGTAATTCAGGATAGGTTGA	miRNA	miR-26b
t0012108	28	36	CATTGCACTTGTCTCGGTCTGACGGGGC	miRNA	miR-25
t0011991	23	36	CATTGCACTTGTCTCGGTCTGTT	miRNA	miR-25
t0011904	21	36	CAGCTACATTGTCTGCTGGGT	miRNA	miR-221
t0011938	22	36	CCTCCTGCCCTCCTTGCTGTAG	miRNA	miR-1976
t0012057	22	36	CAACGGAATCCCAAAAGCAGAT	miRNA	miR-191
t0012140	25	36	CAACGGAATCCCAAAAGCAGCTATC	miRNA	miR-191
t0012144	20	36	ACGGAATCCCAAAAGCAGCA	miRNA	miR-191
t0012226	23	36	ACAACGGAATCCCAAAAGCAGAA	miRNA	miR-191
t0011828	18	36	TGGAGAGAAAGGCAGTTA	miRNA	miR-185
t0012022	24	36	TGGACGGAGAACTGATAAGCCATC	miRNA	miR-184
t0011999	19	36	TATGGCACTGGTAGAATTC	miRNA	miR-183
t0012200	28	36	TTTGGCAATGGTAGAACTCACACCGATC	miRNA	miR-182
t0012212	26	36	CAAGTAGCAGCACGTAAATATTGGCG	miRNA	miR-16
t0011876	22	36	TAGCAGCACATCATCGTTTACA	miRNA	miR-15b
t0011967	24	36	TACCACAGGGTATAACCACGGACA	miRNA	miR-140
t0011994	22	36	TACCACAGGGTAGAACCACGTT	miRNA	miR-140
t0012130	21	36	ACCACAGGGTAGGACCACGGA	miRNA	miR-140
t0011949	23	36	TACCACAGGGTAGAATTACGGAT	miRNA	miR-140
t0011972	19	36	ATTTTAGAGACGGGGTTTG	miRNA	miR-1303
t0012073	21	36	TGTGTGTGTGTATGTGTGAGA	miRNA	miR-1187
t0012152	25	36	TACCCTGTAGAACCGAATTTGTATC	miRNA	miR-10b
t0011971	23	36	AGCAGCATTGTACAAGGCTATGA	miRNA	miR-103a
t0012174	23	36	AGCAGCATTGTAAAGGGCTATGA	miRNA	miR-103a
t0011857	22	36	GTACAGTACTGTGATAACTAAA	miRNA	miR-101
t0012165	25	36	TGGAATGTAAAGAAGTGTGTGTATC	miRNA	miR-1
t0012179	25	36	TGAGGTAGTAGATTGAATAGTTATC	miRNA	let-7k
t0012004	21	36	TGAGGTGGTAGTTTGTACAGT	miRNA	let-7g
t0011793	22	36	TGATGTAGTAGTTTGTACAGTT	miRNA	let-7g
t0011871	24	36	TTTGAGGTAGTAGATTGTATAGTT	miRNA	let-7f

t0012064	19	36	TGGGTAGTAGATTGTATAG	miRNA	let-7f
t0012101	22	36	AGAGGTAGTACGTTGCATAGTT	miRNA	let-7d
t0012228	22	36	TGAGGTATCAGGTTGTGTGGTT	miRNA	let-7b
t0011946	25	36	TGAGGTAGTAGGTCGTGTGGTTATC	miRNA	let-7b
t0012050	20	36	TCAGGTAGTAGGTTGTGTGG	miRNA	let-7b
t0012063	25	36	TGAGGTTGTAGGTTGTGTGGTTATC	miRNA	let-7b
t0012204	20	36	TGAGGTAGCAGGTTGTATAG	miRNA	let-7
t0011802	21	36	TGAGGTAGTAGGTTGGATAGT	miRNA	let-7
t0011819	21	35	TATTGCACTCGTCTCGGCCTC	miRNA	miR-92
t0012132	19	35	TGGAAGACTAGTGATTTTG	miRNA	miR-7
t0011813	22	35	ATGCCTTGAGTGTAGGACCGTA	miRNA	miR-532-5p
t0012098	21	35	TCCTATACTGAGCTGCCCCGA	miRNA	miR-486-5p
t0011925	23	35	TCCTCTACTGAGCTGCCCCGAGT	miRNA	miR-486-5p
t0012156	22	35	TCCTGTACTGTGCTGCCCCGAG	miRNA	miR-486-5p
t0012051	24	35	CTGGGTCGGTCGGGCTGGGGCGCG	miRNA	miR-4651
t0012081	22	35	AAACCGTTAGCATTACTGAGTT	miRNA	miR-451
t0012099	23	35	AAACCGTTACCATTACTGAGCTT	miRNA	miR-451
t0012103	22	35	AGACCGTTACCATTACTGAGTT	miRNA	miR-451
t0012223	20	35	AAACGGTTACCATTACTGAG	miRNA	miR-451
t0011791	21	35	TTCGGGGTCTGGGCGCGGCGT	miRNA	miR-4508
t0012016	21	35	TGAGGGGGAGAGAGCGAGACT	miRNA	miR-423-5p
t0012446	25	35	TGAGGGGCAGAGAGCGAGACTTTAG	miRNA	miR-423-5p
t0012462	20	35	AACTGAAACGGACAAGGGGA	miRNA	miR-3896-3p
t0012350	25	35	ACTGGACTTGGAGTCAGAAGGCATT	miRNA	miR-378
t0012310	25	35	CTGGACTTGGAGTCAGAAGGCTATC	miRNA	miR-378
t0012422	19	35	CTTATCAGATTGTATTGTA	miRNA	miR-374a*
t0012611	21	35	CTTATCAGATTGTATTGTAAT	miRNA	miR-374a*
t0012586	19	35	TTGAAGACTGAAGTGGAGA	miRNA	miR-3526
t0012306	22	35	TCTCACACAGAAATCGCATTCG	miRNA	miR-342-3p
t0012401	23	35	AAAAGCTGGGGTGAGAGGGCGTA	miRNA	miR-320d
t0012486	23	35	AAAGCTGGGTTGAGAGGGCGGTT	miRNA	miR-320d
t0012517	23	35	AAAAGCTGGGTTGAGTGGGCGAA	miRNA	miR-320a
t0012488	23	35	AAAAGCTTGGTTGAGAGGGCGAT	miRNA	miR-320a
t0012372	24	35	GAATCTGAGAAGGCGCACAAGGTT	miRNA	miR-3200-5p
t0012605	24	35	AGGTAGACGGAAAGAGAAATGGAG	miRNA	miR-3190
t0012665	23	35	TGTGGACAGTGAGGTAGAGGGAG	miRNA	miR-3138
t0012295	21	35	GCTGGTTTCACATGGTGGCTT	miRNA	miR-29b-2*
t0012434	21	35	CGCGGGTCGGGGGGCGGGGCG	miRNA	miR-2981
t0012287	24	35	TTAAGTAGTAGTGCCGTAGAGATC	miRNA	miR-252b
t0012302	21	35	CATTGCAATTGTCTCGGTCTG	miRNA	miR-25
t0012345	19	35	TCAGTTCAGCAGGAACAGT	miRNA	miR-24
t0012283	23	35	AAGCAGCTGTAGTGTATGCCTGG	miRNA	miR-2278
t0012547	21	35	CTGTGCGTGTGACAGCGGCTT	miRNA	miR-210
t0012572	21	35	TGGAGAGAAGGGCAGTTCCTG	miRNA	miR-185
t0012528	23	35	CAAAGTGCTTACAGTGCAGAAAA	miRNA	miR-17a
t0012631	22	35	TAGCAGCACGTAAATATTGCCA	miRNA	miR-16b
t0012239	20	35	TAGAAGCACGTAAATATTGG	miRNA	miR-16
t0012664	25	35	TAGCAGCACGTAAATATTGGCGACA	miRNA	miR-16
t0012563	20	35	TAGCAGCACGGAAATATTGG	miRNA	miR-16
t0012550	22	35	TAGCAGCACGTAAATTTTGGCG	miRNA	miR-16
t0012349	19	35	TAGCAGCACGTCATGGTTT	miRNA	miR-15b
t0012502	19	35	TAGCATCACATCATGGTTT	miRNA	miR-15b
t0012505	21	35	TAGCAGCGCATAATGGTTTGT	miRNA	miR-15a
t0012545	21	35	TAGCAGAACATAATGGTTTGT	miRNA	miR-15a
t0012469	21	35	CCAGGAAGATCTTGTAGCATA	miRNA	miR-1503
t0012436	19	35	TCAGTGCATCACAGAACTT	miRNA	miR-148b-3p
t0012583	19	35	TACAGTATAGATGATGTCT	miRNA	miR-144
t0012540	19	35	TCCATAAAGTAGAAAGCAC	miRNA	miR-142-5p

t0012670	23	35	CATAAAGTAGAAAGCACTACTAA	miRNA	miR-142-5p
t0012337	22	35	TAGCACAGGGTAGAACCACGGA	miRNA	miR-140
t0012359	20	35	CCACAGGGTAGAACTACGGA	miRNA	miR-140
t0012444	26	35	ACCACAGGGTAGAACCACGGACGGAT	miRNA	miR-140
t0012566	27	35	ACCACAGGGTAGAACCACGGACCGGCC	miRNA	miR-140
t0012347	20	35	CCACCTCCCCTGCAAACGTC	miRNA	miR-1306
t0012365	20	35	CGGTTTGAGGCTACAGTGAG	miRNA	miR-1304
t0012279	20	35	AGCAGCATTGTACAGGGTAT	miRNA	miR-107b
t0012499	22	35	AGCAGCATTGTACAGGGCTAGT	miRNA	miR-107
t0012633	23	35	CCGCACTGTGGGTACTTGCTGCT	miRNA	miR-106b*
t0012477	21	35	AGCAGCATTATACAGGGCTAT	miRNA	miR-103a
t0012543	19	35	AGCAGTATTGTACAGGGCT	miRNA	miR-103a
t0012260	22	35	TACAGTACTGTGATAACTGACG	miRNA	miR-101c
t0012524	22	35	TACATTACTGTGATAACTGAAG	miRNA	miR-101
t0012237	26	35	GTACAGTACTGTGATAACTGAACCCA	miRNA	miR-101
t0012348	19	35	TGAGGTAGTAGTCTGTGCT	miRNA	let-7i
t0012402	22	35	TGAGGTAGTAGTTTGTACAGCT	miRNA	let-7g
t0012590	22	35	GTTTGAGGTAGTAGATTGTATA	miRNA	let-7f
t0012570	20	35	TGACGTAGTAGATTGTATAG	miRNA	let-7f
t0012538	19	35	TGAGGTAGTAGATTGTACT	miRNA	let-7f
t0012432	20	35	AGAGGTAGTAGGGTGCATAG	miRNA	let-7d
t0012413	22	35	AGAGGTAGTAGGTTACATAGTT	miRNA	let-7d
t0012495	20	35	TGAGGTAGTAGGTTCTGTGG	miRNA	let-7b
t0012312	22	35	TGAAGTAGTAGGTTGTGTGGTT	miRNA	let-7b
t0012405	20	35	TGAGGTAGTAGGTCGTGTGG	miRNA	let-7b
t0012418	23	35	TTAGGTAGTAGGTTGTATAGTTG	miRNA	let-7
t0012440	22	35	TGAGATCATTGTGAAGGCCACT	miRNA	bantam
t0012624	22	34	CACCCGTAGAACCGACCTTGCG	miRNA	miR-99b
t0012252	23	34	TATTGCACTTGTCCTGGCCTGTA	miRNA	miR-92a
t0012585	23	34	TATTGCACTCGTCCCGGCCTAAA	miRNA	miR-92
t0012266	21	34	TAGAGGAGATGGCGCAGGAAA	miRNA	miR-877
t0012530	20	34	GTAGAGGAGATGGCGCAGGG	miRNA	miR-877
t0012536	25	34	TGGAAGACTAGTGATTTTGTTAATC	miRNA	miR-7b
t0012308	22	34	TAGTACCAGTACCTTGTGTTCA	miRNA	miR-624*
t0012433	25	34	AAGGGATTCTGATGTTGGTCACATC	miRNA	miR-541
t0012564	29	34	TCCTGTACTGAGCTGCCCCGAGACGGCA(	miRNA	miR-486-5p
t0012592	30	34	AGGGCTGGGTCGGTCGGGCTGTGGCGCA	miRNA	miR-4651
t0012673	22	34	AGGGCTGGGTCGGTCGGGCTGG	miRNA	miR-4651
t0012601	24	34	GAAAACCGTTACCATTACTGAGTT	miRNA	miR-451
t0012377	21	34	AAACCGTTACGATTACTGAGT	miRNA	miR-451
t0012953	24	34	TGAGGGGCAGAGAGCGAGACTTGT	miRNA	miR-423-5p
t0013050	19	34	ATAAGGAGAAAGCACTGTA	miRNA	miR-3964
t0012764	26	34	ACTGGACTTGGAGTCAGAAGGCTATC	miRNA	miR-378
t0012937	23	34	CTTTGAAGACTGAAGTGGAGAAG	miRNA	miR-3526
t0012956	23	34	TCACACAGAAATCGCACCCGTCG	miRNA	miR-342-3p
t0012678	24	34	TCTCACACAGAAATCGCACCCGTA	miRNA	miR-342-3p
t0012731	24	34	AAAAGCTGGGTTGAGAGGGCATTA	miRNA	miR-320d
t0012762	23	34	AAAGCTGGGTTGAGAGGGCATTA	miRNA	miR-320d
t0012964	23	34	AAAAGCTGCTTTGAGAGGGCGTT	miRNA	miR-320d
t0012794	22	34	AGAAGCTGGGTTGAGAGGGCGA	miRNA	miR-320a
t0012913	22	34	TGGAAGGTAGACGGCCAGAGAG	miRNA	miR-3190
t0012922	19	34	CAGTGCAATAGTATTGTCA	miRNA	miR-301b-3p
t0013080	19	34	GGGGGGCGGGGCGGACTGT	miRNA	miR-2885
t0012755	26	34	TGGCTCAGTTCAGCAGTAACAGAATC	miRNA	miR-24b
t0012802	22	34	AGCTACATCTGGCTACTGGGTA	miRNA	miR-222
t0013027	22	34	TGACTGGGGCGGGACATCTGTT	miRNA	miR-219-2-3p
t0013083	25	34	TGGAATGTATAGAAGTATGTATATC	miRNA	miR-1a
t0013104	21	34	CATGGAATCCCAAAAGCAGCT	miRNA	miR-191

t0013106	25	34	CAACGGAATCCCAAAAGCAGCTGAA	miRNA	miR-191
t0012704	22	34	TGGAGAGAAAAGTAGTTCCTGA	miRNA	miR-185
t0012895	22	34	TGGACGGAGAACTGATAAGGGC	miRNA	miR-184-3p
t0012943	23	34	TGGACGGAGAACTGATAAGGGAA	miRNA	miR-184
t0013103	26	34	TGGGGGCGGGGAGCGGTCGGGCGGCG	miRNA	miR-1607
t0012905	19	34	TAGCAGTACGTAAATATTG	miRNA	miR-16
t0012897	21	34	TAGCAGCACATCATGGTTTTA	miRNA	miR-15c
t0012825	24	34	AAAATAGCAGCACATCATGGTTTA	miRNA	miR-15b
t0012861	20	34	TAGCAACACATCATGGTTTA	miRNA	miR-15b
t0013068	20	34	TAGCAGCACGTCATGGTTTA	miRNA	miR-15b
t0012778	20	34	TAGCAGCACATCCTGGTTTA	miRNA	miR-15b
t0012801	21	34	ATAGCAGCACATAATGGTTTG	miRNA	miR-15a
t0012679	19	34	CTAGACTGAAGCTCCTTGA	miRNA	miR-151-3p
t0013009	25	34	TGAGAACTGAATTCCATAGGCTGTA	miRNA	miR-146b
t0012857	24	34	TACCACAGGGTAGAACCACGTACG	miRNA	miR-140
t0012996	23	34	ACCACAGGGTAGAATCACGGACG	miRNA	miR-140
t0013094	23	34	ACCACAGGGTACAACCACGGACA	miRNA	miR-140
t0012784	22	34	ACCACAGGGTAGAACCAAGGAA	miRNA	miR-140
t0012987	20	34	ACCACAGGGTACAACTACGG	miRNA	miR-140
t0012991	26	34	ACCACAGGGTAGAACCACGGACGGCC	miRNA	miR-140
t0012685	23	34	ACAGTGCAATGTTAAAAGGGCAT	miRNA	miR-130a
t0012775	20	34	TTTGAGGCTACAGTGAGATG	miRNA	miR-1304
t0012903	18	34	TGTGAGGTTGGCATTGTT	miRNA	miR-1294
t0012929	24	34	TGGGAACGGGTTCCGGCAGACGCT	miRNA	miR-1292
t0013015	23	34	CGGATGAGCAAAGAAAGTGGTTA	miRNA	miR-1255b
t0012923	23	34	CTTCCCCACCCTCTCCTGCAGTT	miRNA	miR-1224-3p
t0012893	24	34	CCGCACTGTGGGTACTTGCTGAAA	miRNA	miR-106b*
t0012727	21	34	TAAAGTGCTGACAGTTCAGAT	miRNA	miR-106b
t0012761	23	34	AGCAGCATTATACAGGGCTATGA	miRNA	miR-103a
t0012797	24	34	CAGTACAGTACTGTGATAACTGAA	miRNA	miR-101
t0012865	19	34	TCAGGTAGTAGTTTGTGCT	miRNA	let-7i
t0013000	20	34	TGAGGTAGTAGTTTGTGGTT	miRNA	let-7i
t0013105	22	34	TGAGGAAGTAGTTTGTGCTGTT	miRNA	let-7i
t0012951	18	34	TGAGGTAGTAGATTGCAT	miRNA	let-7f
t0012725	21	34	CTATACAACCTACTGCCTTCA	miRNA	let-7b*
t0012686	25	34	TGAGGTAGTAGGTTGTGTGGTTGAT	miRNA	let-7b
t0012901	21	34	AGAGGTAGTAGGTTGTGTGGT	miRNA	let-7b
t0013045	20	34	TGAGGTAGTAGGTGGTGTGG	miRNA	let-7b
t0012763	22	34	TGAGGGGGTAGGTTGTGTGGTT	miRNA	let-7b
t0012894	23	34	TGAGGTTGTAGGTTGTGTGGTTA	miRNA	let-7b
t0012948	20	34	TGAGGTAGTAGGTTGTCTGG	miRNA	let-7b
t0013117	22	34	TGAGGTAGTAGGTTGTATGGTA	miRNA	let-7-5p
t0012696	22	34	TGAGGTAGTAGGTTGTATAGTC	miRNA	let-7-5p
t0012821	22	34	TGAGGTAGTAGGTTGAATAGTT	miRNA	let-7
t0013063	21	34	TGAGGTAGTAGGTTGTAAAGT	miRNA	let-7
t0013073	20	34	TAGGTAGTAGGTTGTATAGT	miRNA	let-7
t0012975	22	34	TCAGGTAGTAGGTTGTATAGTT	miRNA	let-7
t0012823	21	34	TGAGGTAGTAGGTTGAATAGT	miRNA	let-7
t0012676	18	33	GCACTTGTCCCGGCCTGT	miRNA	miR-92c
t0012682	22	33	ATTGCACCTGTCCCGGCCTGTA	miRNA	miR-92b-3p
t0013022	20	33	TATTGCACTTGTCCCGGCGT	miRNA	miR-92a
t0013066	21	33	AAAAGTAATTGCGGTCTTTGG	miRNA	miR-548j
t0013121	21	33	TTCCCACACCCAAGGCTTGCA	miRNA	miR-532-3p
t0013125	23	33	TCCTGTATTGAGCTGCCCCGAGA	miRNA	miR-486-5p
t0012702	22	33	TCCTGTACTGAGCTGCCCCGTG	miRNA	miR-486-5p
t0012836	23	33	TTTTGTACTGAGCTGCCCCGAGA	miRNA	miR-486-5p
t0013064	21	33	TCCTGTACTGAGCCGCCCCGA	miRNA	miR-486-5p
t0013075	22	33	TCCTATACTGAGCTGCCCCGAG	miRNA	miR-486-5p

t0013076	22	33	TCGGGCGGAGGTGGTTGAGTGT	miRNA	miR-4750
t0013087	21	33	TTACACAGCTGGACAGAGGCA	miRNA	miR-4672
t0013126	21	33	AAACCGATACCATTACTGAGT	miRNA	miR-451
t0013474	19	33	AAGCGTTACCATTACTGAG	miRNA	miR-451
t0013427	22	33	TAAACCGTTACCATTACTGAGT	miRNA	miR-451
t0013392	26	33	AAAAACCGTTACCATTACTGAGTTTA	miRNA	miR-451
t0013169	25	33	AAACCGTTACCATTACTGAGTTACA	miRNA	miR-451
t0013521	22	33	AAACCGTTGCCATTACTGAGTT	miRNA	miR-451
t0013155	20	33	TTCGGGGTCTGGGCGCGGTT	miRNA	miR-4508
t0013402	22	33	TGAGGGGCAGAGAGCGAGACAA	miRNA	miR-423-5p
t0013444	21	33	TGAGGGGCAGAGTGCGAGACT	miRNA	miR-423-5p
t0013457	23	33	TGAGGGGCAGATAGCGAGACTTA	miRNA	miR-423-5p
t0013505	21	33	ACTGAAACGGACAAGGGGAAT	miRNA	miR-3896-3p
t0013611	23	33	ACTGGACTTGGAGTCAGAAGGAT	miRNA	miR-378c
t0013398	22	33	CTGGACTTGGAGTCAGAAGGCC	miRNA	miR-378
t0013176	23	33	TCTCACACAGAAATCGCACCTGT	miRNA	miR-342-3p
t0013312	25	33	AAAAGCTGGGTTGAGAGGGCGTAAA	miRNA	miR-320d
t0013372	23	33	AAAAGTCGGGTTGAGAGGGCGAA	miRNA	miR-320c
t0013418	22	33	AAACGCTGGGTTGAGAGGGCGA	miRNA	miR-320a
t0013422	23	33	AAAAGCTGGGTTCAGAGGGCGAA	miRNA	miR-320a
t0013461	23	33	AAAGCTGGGTTGAGAGGGCGAAT	miRNA	miR-320a
t0013514	27	33	TGTAAACATCCTTGACTGGAAGCTATC	miRNA	miR-30e
t0013221	23	33	CACTAGATTGTGAGCTCCTGGAA	miRNA	miR-28-3p
t0013429	23	33	TTCAAGTAATTCAGGATAGGTAA	miRNA	miR-26b
t0013581	22	33	TTCAAGTAACCCAGGATAGGCT	miRNA	miR-26a
t0013234	22	33	TTCAAGTAATCCAGAATAGGCT	miRNA	miR-26a
t0013197	21	33	AGGCGGAGACTTGGGCAATTT	miRNA	miR-25*
t0013343	22	33	AAGCTGCTAGTTGAAGAACTGT	miRNA	miR-22-3p
t0013447	22	33	CTGTGCGTGTGACAGCGGCTAA	miRNA	miR-210
t0013173	21	33	TCTACTGTAGTATGGGCACTT	miRNA	miR-20b*
t0013302	21	33	CCTGACCTATGAATTGACAGC	miRNA	miR-192
t0013353	25	33	CAACGGAATCCCAAAAGCAGCTGTA	miRNA	miR-191
t0013404	22	33	CAACGGAATACCAAAAGCAGCT	miRNA	miR-191
t0013548	20	33	AACGGAATCCCCAAAGCAGC	miRNA	miR-191
t0013235	23	33	TGGGGAGAAAGGCAGTTCCTGAA	miRNA	miR-185
t0013567	19	33	TAGCCCGTAAATATTGGCG	miRNA	miR-16c
t0013485	26	33	TAGCAGCACGTAAATATTGGCGTTAT	miRNA	miR-16
t0013285	21	33	TAGCAGCACGTAAATATTCGT	miRNA	miR-16
t0013557	22	33	TAGCTGCACGTAAATATTGGCG	miRNA	miR-16
t0013510	20	33	TAGCAGCACATCATGGTTTG	miRNA	miR-15b
t0013430	19	33	TAGCAGCACATCATGGTAC	miRNA	miR-15b
t0013549	20	33	TAGCAGCAGATAATGGTTTG	miRNA	miR-15a
t0013578	22	33	TAGCAGCACATAATGGTTGTGG	miRNA	miR-15a
t0013554	21	33	TCAGTGTACTACAGAACTTTG	miRNA	miR-148a
t0013321	24	33	TGAGAACTGAATTCCATAGGCTAT	miRNA	miR-146b-5p
t0013327	22	33	CATAAAGTAGAAAGCACTACTA	miRNA	miR-142-5p
t0013546	23	33	ACCACAGTGTAGAACCACGGACG	miRNA	miR-140
t0013207	23	33	TACCACAGGGTAGAACCTCGGAT	miRNA	miR-140
t0013210	23	33	TACCACAGGGTAGAACCTCGGAC	miRNA	miR-140
t0013345	23	33	ACCACAGGGTAGAACCACGGAGG	miRNA	miR-140
t0013532	22	33	ACCACAGGGAAGAACCACGGAC	miRNA	miR-140
t0013601	22	33	AACCACAGGGTAGAACCACGGA	miRNA	miR-140
t0013476	22	33	GCAGTGCAATGTTAAAAGGGCA	miRNA	miR-130a
t0013524	22	33	AGCAGCATTGTACAGGGCTTAT	miRNA	miR-107b
t0013367	22	33	AGCAGCATTGTATAGGGCTATT	miRNA	miR-107
t0013439	22	33	TACCGCACTGTGGGTACTTGTA	miRNA	miR-106b*
t0013527	22	33	TCGCACTGTGGGTACTTGCTGA	miRNA	miR-106b*
t0013206	23	33	AGCAGCATTGTACAGGGCTAAGT	miRNA	miR-103a

t0013313	26	33	AGCAGCATTGTACAGGGCTATGATAA	miRNA	miR-103a
t0013149	23	33	AGCAGCATTGGACAGGGCTATGA	miRNA	miR-103a
t0013160	23	33	AGTAGCATTGTACAGGGCTATGA	miRNA	miR-103a
t0013178	21	33	AGCAACATTGTACAGGGCTAT	miRNA	miR-103a
t0013231	21	33	TACAGTACTGTGATAACTCAC	miRNA	miR-101c
t0013572	21	33	TACAGTACTGGGATAACTGAA	miRNA	miR-101
t0013594	19	33	TGAGGTAGTAGTTTTTGCT	miRNA	let-7i
t0013183	22	33	TGAGGTAGGAGTTTGTGCTGTT	miRNA	let-7i
t0013331	22	33	TGAGGTAGGAGTTTGTACAGTT	miRNA	let-7g
t0013560	22	33	TGAGGTAGTAGTTTGTACACTT	miRNA	let-7g
t0013145	21	33	TGAGGAGGTAGATTGTATAGT	miRNA	let-7f
t0013568	23	33	TGAGGTAGTAGATTGTATATTTG	miRNA	let-7f
t0013196	19	33	TGAAGTAGGTTGTGTGGTT	miRNA	let-7b
t0013237	22	33	TGAGGTAGTAGGTTGTATAGAG	miRNA	let-7-5p
t0013525	25	33	TGAGGTAGTAGGTTGTATGGTAATC	miRNA	let-7-5p
t0013577	19	33	TGATGTAGTAGGTTGTATA	miRNA	let-7
t0013609	25	32	CAAAGTGCTGTTCGTGCAGGTAAAA	miRNA	miR-93
t0013198	23	32	TATTGCACTTGTCCCGGCCTGAG	miRNA	miR-92b-3p
t0013303	22	32	TATTGCACTTGTCCCGGCCATA	miRNA	miR-92b
t0013143	20	32	AGGTTGGGATCGGTTGCAAT	miRNA	miR-92a-1*
t0013355	22	32	ATATTGCACTTGTCCCGGCCTG	miRNA	miR-92a
t0013305	20	32	TATTGCACTTGTCCCGGCAT	miRNA	miR-92a
t0013555	20	32	TTCATTGCATATCGGAGTTG	miRNA	miR-660
t0013188	21	32	CCTCCCACACCCAAGGCTTGA	miRNA	miR-532-3p
t0013596	23	32	TGGGCACAGGCGGATGGACAGGT	miRNA	miR-5107
t0013262	26	32	GGGTCTGTAGCTCAGGTGGTTATAGC	miRNA	miR-5106
t0013154	22	32	GGGAGTGCAGGGCAGGGTTTCC	miRNA	miR-504*
t0013488	18	32	TAGCAGCGGGAACAGTTC	miRNA	miR-503
t0013171	25	32	TCCTGTACTGAGCTGCCCCGAGACG	miRNA	miR-486-5p
t0013814	22	32	TCCTGTACTGCGCTGCCCCGAG	miRNA	miR-486-5p
t0013911	28	32	TCCTGTACTGAGCTGCCCCGAGCCCGCA	miRNA	miR-486-5p
t0013827	18	32	TCGTACTGAGCTGCCCCG	miRNA	miR-486-5p
t0013793	25	32	TCCTGTACTGAGCTGCCCCGACGGG	miRNA	miR-486-5p
t0013733	21	32	GGGCTGGGTCGGTCGGGCTGG	miRNA	miR-4651
t0013997	22	32	GGGGGGAGAAGGGTCGGGGCGG	miRNA	miR-4516
t0014055	21	32	AAACCGTTACCATTGCTGAGT	miRNA	miR-451
t0013826	23	32	AAACCGTTACCATTAGTGAGTTT	miRNA	miR-451
t0013957	25	32	AAACAAACCGTTACCATTACTGAGA	miRNA	miR-451
t0013917	25	32	AAACCCTTACCATTACTGAGTTTAG	miRNA	miR-451
t0013705	21	32	GCAGGCAGAAGGAAGCAGTGG	miRNA	miR-4419b
t0014059	19	32	TCCCACTGCTTCACTTGAC	miRNA	miR-4301
t0013643	19	32	AATGACACGATCACTCCCG	miRNA	miR-425
t0013672	19	32	TGAGGGGCAGAGATCGAGA	miRNA	miR-423-5p
t0013838	21	32	TGAGGGGCAGAGAGCGAGTTT	miRNA	miR-423-5p
t0013979	22	32	CTGAGGGGCAGAGAGCGAGACT	miRNA	miR-423-5p
t0014014	23	32	TGGACTTGGAGTCAGAAGGCATC	miRNA	miR-378
t0013765	22	32	ATATAATACAACCTGCTAATTG	miRNA	miR-374
t0013653	19	32	TGATGATACAGGTGGAGGT	miRNA	miR-3682-3p
t0013963	22	32	ATGAGGATGGATAGCAAGGAAG	miRNA	miR-3605-5p
t0014051	20	32	AAGTAGTAGGTTGCATAGTT	miRNA	miR-352
t0014061	23	32	TCACACAGAAATCGCACCCGTAA	miRNA	miR-342-3p
t0014064	25	32	AAAAGCTGGGTTGAGAGGGCAATTT	miRNA	miR-320b
t0014117	20	32	CTTTCAGTCGGATGTTTATA	miRNA	miR-30b-3p
t0013767	26	32	CATTGCACTTGTCTCGGTCTGACAAG	miRNA	miR-25
t0013868	20	32	TGGGGTTCCTGGGGATGGGA	miRNA	miR-23a*
t0013704	25	32	TGTAACAGCAACTCCATGTGGAATC	miRNA	miR-194
t0013773	21	32	CTGGGAATTGTAGTTCGGGTC	miRNA	miR-1938
t0013809	22	32	AACGGAATCCCAAAAGCCGCTG	miRNA	miR-191

t0014066	21	32	AACGGAATCCCAAAAGCACCT	miRNA	miR-191
t0014111	23	32	TGGAGAGAAAGGGAGTTCCTGAA	miRNA	miR-185
t0014104	23	32	TGGAGAGAAAGGCGGTTCCTGAA	miRNA	miR-185
t0014048	21	32	AGACCTACTTACTTACCAACA	miRNA	miR-1839-3p
t0013717	26	32	AACATTCATTGCTGTCGGTGGGTATC	miRNA	miR-181b
t0013961	23	32	ACATTCATTGCTGTCGGTGGGTT	miRNA	miR-181b
t0014110	21	32	CTGCAGTGAAGGCACTTGTAG	miRNA	miR-17*
t0013970	19	32	TAGCAGCACATCTTGGTTT	miRNA	miR-15b
t0013655	20	32	GCAGCACATCATGGTTTACA	miRNA	miR-15b
t0013878	20	32	TAGCAGCGCATCATGGTTTA	miRNA	miR-15b
t0013978	19	32	GGATATCATCATATACTGT	miRNA	miR-144*
t0014007	24	32	GGATATCATCATATACTGTAAGTA	miRNA	miR-144*
t0013871	27	32	TGAGATGAAGCACTGTAGCTCTTTATC	miRNA	miR-143
t0014038	22	32	TACCACAGGGTAGAACCACGAC	miRNA	miR-140
t0013906	23	32	TACCACGGGGTAGAACCACGGAC	miRNA	miR-140
t0013912	23	32	TACCACAGGGTAGAACCACGGGA	miRNA	miR-140
t0013790	23	32	TACCACAGGGTAGAACCACTGAA	miRNA	miR-140
t0013685	25	32	TGGAGACGCGGCCCTGTTGGAGATC	miRNA	miR-139*
t0013930	24	32	CGTACCGTGAGTAATAATGCGATC	miRNA	miR-126
t0013805	20	32	TTTCCGGCTCGCGTGGGTGT	miRNA	miR-1180
t0013641	23	32	AGCAGCATTGTACAGGGCTATTA	miRNA	miR-107
t0013803	25	32	AGCAGCATTGTACAGGGCTATAAAC	miRNA	miR-107
t0013895	21	32	AGCAGCACTGTACAGGGCTAT	miRNA	miR-103a
t0013981	21	32	AGCAGCATTTTACAGGGCTAT	miRNA	miR-103a
t0014008	27	32	TACAGTACTGTGATAACTGAACGCGGC	miRNA	miR-101
t0014006	20	32	TGAGGTAGTAGTTTTTGCTG	miRNA	let-7i
t0013675	19	32	TGAGGTAGTAGGTTGTGCT	miRNA	let-7i
t0013718	26	32	TGAGGTAGTAGATTGTATAGTTCTTG	miRNA	let-7f
t0013785	18	32	TGAGGTAGTAGTTTGTCT	miRNA	let-7f
t0014098	22	32	AGAGGTAGGAGGTTGCATAGTT	miRNA	let-7d
t0013866	22	32	GGAGGTAGTAGGTTGCATAGTT	miRNA	let-7d
t0014072	19	32	TGAGGTAGTAGGTTGTTAG	miRNA	let-7b
t0013903	24	32	TGATGTAGTAGGTTGTGTGGTTAA	miRNA	let-7b
t0013662	21	32	GGAGGTAGTAGGTTGTATAGT	miRNA	let-7
t0014042	21	32	TAGGTAGTAGGTTGTATAGTT	miRNA	let-7
t0013682	22	31	CTATACAACTTACTACTTTCCT	miRNA	miR-98*
t0013745	19	31	TGAGGTAGTAAGTTGTATT	miRNA	miR-98
t0014057	20	31	GGTGGAAGGTTGTAGGCATG	miRNA	miR-965-5p
t0013738	26	31	AGGGCGCGCGGGTCGGGGCGGCGGCC	miRNA	miR-638
t0013857	22	31	TTATGGTTTGCCTGGGACTGAT	miRNA	miR-584
t0013831	19	31	TAGCAGCGGGAACAGTTGA	miRNA	miR-503
t0013966	23	31	ATGCACCTGGGCAAGGATTCAGA	miRNA	miR-500a*
t0013988	20	31	TCCTGTACTGACTTGCCCCG	miRNA	miR-486-5p
t0014054	22	31	TCCTGTCCTGAGCTGCCCCGAG	miRNA	miR-486-5p
t0013737	27	31	TCCTGTACTGAGCTGCCCCGAGACGGC	miRNA	miR-486-5p
t0014091	21	31	TCTTCTTAGACATGGCAACGT	miRNA	miR-4659a-3p
t0013751	26	31	GGGCTGGGTCGGTCGGGCTGGGGCGC	miRNA	miR-4651
t0014100	18	31	AAGGGCTGGGTCGGTCGG	miRNA	miR-4651
t0014446	19	31	GTCGCGTGCTGTTCGTCGG	miRNA	miR-4573
t0014479	23	31	ATTCGACTCTGGATAGCTGGTTT	miRNA	miR-4538
t0014578	21	31	GAACCGTTACCATTACTGAGT	miRNA	miR-451
t0014428	23	31	ATGGCCAGAGCTCACACAGAGGA	miRNA	miR-4435
t0014135	19	31	ATGACACGATCACTCCCGT	miRNA	miR-425-5p
t0014286	21	31	ATCGGGAATGTCGTGTCCGCC	miRNA	miR-425*
t0014172	23	31	AGAGGGGCAGAGAGCGAGACTTT	miRNA	miR-423-5p
t0014144	21	31	CCTCGAACTGTTGTGGCCATT	miRNA	miR-3487
t0014160	24	31	TCCTCGAACTGTTGTGGCCATTTT	miRNA	miR-3487
t0014273	24	31	CACCTTGCGCTACTCAGGTCTGAG	miRNA	miR-3200-3p

t0014290	24	31	CTTTCAGTCGGATGTTTACAGCAG	miRNA	miR-30e*
t0014142	21	31	TTCACAGTGGCTAAGTTCCGA	miRNA	miR-27a
t0014362	22	31	TTCAAGTAATCCAGGATCGGCT	miRNA	miR-26a
t0014270	23	31	CATTGCACTTGTCTCGGTCTGAC	miRNA	miR-25
t0014211	21	31	CAAAGTGCTCATAGTGCAGGT	miRNA	miR-20b
t0014244	26	31	CAACGGAATCCCAAAAGCAGCAAAAG	miRNA	miR-191
t0014552	22	31	TAACGGAATCCGAAAAGCAGCT	miRNA	miR-191
t0014148	22	31	CAACAGAATCCCAAAAGCAGCT	miRNA	miR-191
t0014344	22	31	CAACGGAATCCCCCAAGCAGCT	miRNA	miR-191
t0014439	27	31	TGGAGAGAAAGGCAGTTCCTGAACGTT	miRNA	miR-185
t0014220	21	31	TGGAGCGAAAGGCAGTTCCTG	miRNA	miR-185
t0014243	20	31	AGGTAGATAGAACAGGTTTG	miRNA	miR-1839
t0014324	22	31	TGAGGGAGTAGATTGTATAGTA	miRNA	miR-1827
t0014471	25	31	AAGCGGCGATGGCGGAGCTGAATTA	miRNA	miR-1636
t0014548	24	31	GCGGGGAGCGGTCGGGCGGCGGCG	miRNA	miR-1607
t0014183	19	31	TAGCAGCACGTAATTATTG	miRNA	miR-16
t0014228	21	31	AATAGCAGCACATCATGGTTT	miRNA	miR-15b
t0014266	19	31	TAGCAGGACATCATGGTTT	miRNA	miR-15b
t0014259	20	31	AGCAGCACATCATGGTTTAA	miRNA	miR-15b
t0014359	19	31	TAGCAGCACATCATCGTTT	miRNA	miR-15b
t0014417	23	31	TAGCAGCACATCATGGTTTACAT	miRNA	miR-15b
t0014559	18	31	TAGCAGCACATCATGGGT	miRNA	miR-15b
t0014159	19	31	TAGCAGCACTTAATGGTTT	miRNA	miR-15a
t0014427	26	31	CTAGACTGAGGCTCCTTGAGGATATC	miRNA	miR-151-3p
t0014420	19	31	GTCCAGTTTTCCCAGGAAT	miRNA	miR-145
t0014410	20	31	AATACAGTATAGATGATGTA	miRNA	miR-144
t0014325	21	31	TACAGTATAGATGATGTATTT	miRNA	miR-144
t0014168	20	31	CATAAAGTAGAAAGCACTAT	miRNA	miR-142-5p
t0014573	20	31	CTCATAAAGTAGAAAGCACT	miRNA	miR-142-5p
t0014129	23	31	TACCACAGGTTAGAACCACGGAC	miRNA	miR-140
t0014349	23	31	TATCACAGGGTAGAACCACGGAC	miRNA	miR-140
t0014358	23	31	ACCACAGGGTAGAACCACGGTTT	miRNA	miR-140
t0014360	23	31	TACCACAGGGAAGAACCACGGAC	miRNA	miR-140
t0014454	23	31	TCTGGGCAACAAAGTGAGACCTA	miRNA	miR-1285
t0014509	20	31	AGCAGCATTGTACAAGGCTA	miRNA	miR-103a
t0014425	22	31	AGCAGCATTGTACAGAGCTATG	miRNA	miR-103a
t0014261	22	31	TGAGGTAGTAGGTTGTGCTGTT	miRNA	let-7i
t0014368	22	31	TGAGGTAGTAGTTTGTCCAGTT	miRNA	let-7g
t0014319	22	31	TGAGGTAGTAGATTGTATACCT	miRNA	let-7f-5p
t0014489	20	31	ATGAGGTAGTAGATTGTATA	miRNA	let-7f
t0014413	25	31	TTAGGTAGTAGATTGTATAGTTATC	miRNA	let-7f
t0014537	22	31	TGAGGTAGTAGATATTATAGTT	miRNA	let-7f
t0014237	22	31	TGACGTAGTAGATTGTGTAGTT	miRNA	let-7f
t0014341	21	31	AGGTAGTAGATTGTATAGTTA	miRNA	let-7f
t0014393	21	31	TGAGGTAGTAGGGTTTGTGGT	miRNA	let-7b
t0014469	26	31	TGAGGTAGTAGGTTGTGTGGTTTTAT	miRNA	let-7b
t0014180	21	31	TGAGGTAGTAGGTTGGTTGGT	miRNA	let-7b
t0014415	22	31	TGAGGTAGTAGGTTGTATAGAT	miRNA	let-7
t0014477	26	31	TGAGGTAGTAGGTTGTATAGTTAATC	miRNA	let-7
t0014416	21	30	CTATACAACTTACTACTTTCT	miRNA	miR-98*
t0014585	29	30	AGGTGGAAGGTTGTAGGCATGTATCTTAC	miRNA	miR-965-5p
t0014581	23	30	TTTGGCACTAGCACATTTTTGCT	miRNA	miR-96
t0014343	22	30	GGGCAGGTGCTGTAGTGGTATC	miRNA	miR-885
t0014434	22	30	GTAGAGGAGATGGCGCAGGACA	miRNA	miR-877
t0014468	25	30	AATTTGTATTGTGACTGGCATTTTT	miRNA	miR-753c
t0014480	22	30	TCCTGTACTGACCTGCCCCGAG	miRNA	miR-486-5p
t0014507	28	30	TCCTGTACTGAGCTGCCCTGAGACGGCT	miRNA	miR-486-5p
t0014354	25	30	TCCTGTACTGAGCTGCCCCGAGGAC	miRNA	miR-486-5p

t0014388	24	30	TCTTGTACTGAGCTGCCCCGAGAA	miRNA	miR-486-5p
t0014407	21	30	TCCGTACTGAGCTGCCCCGAG	miRNA	miR-486-5p
t0014422	21	30	CCCTGTACTGAGCTGCCCCGA	miRNA	miR-486-5p
t0014520	24	30	TCCTGTACTGAGCTGCCCCGACAA	miRNA	miR-486-5p
t0014925	18	30	TGAGGGAAGGAGGCTTGG	miRNA	miR-4747-5p
t0014604	25	30	GGCTGGGTCGGTCGGGCTGGGGCGC	miRNA	miR-4651
t0014747	20	30	GGAGAAGGGTCGGGGCGTTT	miRNA	miR-4516
t0014872	21	30	CGTTACCATTACTGAGTAAAC	miRNA	miR-451
t0014979	29	30	AAACCGTTACCATTACTGAGTTGTAGTTG	miRNA	miR-451
t0014953	20	30	AAACCGTTACCATTTCTGAG	miRNA	miR-451
t0014690	23	30	AAACCGTTACCATTACTGAGAAA	miRNA	miR-451
t0014734	21	30	TGGAGAGAAAGGCAGTGTTTG	miRNA	miR-4306
t0014749	21	30	GGCAGCATTGTACAGGGCTAT	miRNA	miR-4289
t0014800	21	30	CAAAACGTGAGGCGCTGCTAT	miRNA	miR-424*
t0014860	19	30	TGAGGGGCAGAGAGCGACT	miRNA	miR-423-5p
t0014959	23	30	TGAGGGGCTGAGAGCGAGACTTA	miRNA	miR-423-5p
t0014992	22	30	TGAGGGGCAGAGAGCGAGACTC	miRNA	miR-423-5p
t0015058	23	30	GGAGGGGCAGAGAGCGAGACTTT	miRNA	miR-423-5p
t0014777	20	30	TGAGGGGCAGATAGCGAGAC	miRNA	miR-423-5p
t0014665	21	30	TTGAGGGGCAGAGAGCGAGAC	miRNA	miR-423-5p
t0014648	23	30	TGGACTCGCAGGAACAGGCCTTT	miRNA	miR-4001b-5p
t0014794	25	30	GGAGGAACCTTGGAGCTTCGGCTAT	miRNA	miR-3928
t0014944	20	30	GAACTGAAGCGGACAAGGGG	miRNA	miR-3896-3p
t0015060	23	30	TCCTCGAACTGTTGTGGCCAATT	miRNA	miR-3487
t0014736	24	30	AAAGCTGGGTTGAGAGGGCGAAAT	miRNA	miR-320a
t0014599	23	30	TAGCACCATTTGAAATCGGTTAA	miRNA	miR-29c
t0014911	19	30	CGGGGGGCGGGGCGGACTG	miRNA	miR-2885
t0014634	18	30	TTCACAGTGGCTAAGTTC	miRNA	miR-27b
t0014780	20	30	CATTGCACTTGTCTCGGTTT	miRNA	miR-25
t0014835	29	30	CATTGCACTTGTCTCGGTCTGAAAATCAT	miRNA	miR-25
t0014914	21	30	ATCACATTGCCAGGGATAAAA	miRNA	miR-23b
t0014983	20	30	ACTGTAGTATGGGCACTTTT	miRNA	miR-20b*
t0015069	19	30	TTATGCATATACTTCTTTG	miRNA	miR-202-5p
t0014755	21	30	TGACCTATGAATTGACAGCCT	miRNA	miR-192
t0015011	20	30	AAGGTAGATAGAACAGGTTG	miRNA	miR-1839
t0015008	24	30	ATGGCACTGGTAGAATTCACTATC	miRNA	miR-183
t0014810	19	30	AACATTCATTGCTGTCGGA	miRNA	miR-181b
t0014926	22	30	AATAGCAGCACATCATGGTTTA	miRNA	miR-15b
t0014980	20	30	TACCAGCACATCATGGTTTA	miRNA	miR-15b
t0014948	22	30	TAGCAGCACATCATGGATTACA	miRNA	miR-15b
t0014658	22	30	TAGCAGCACATAATGGTTTACA	miRNA	miR-15b
t0015002	23	30	AAACTAGCAGCACATAATGGTTT	miRNA	miR-15a
t0015037	21	30	ACTAGACTGAAGCTCCTTGAG	miRNA	miR-151-3p
t0014864	23	30	GGATATCATCATATACTGTAAGA	miRNA	miR-144*
t0014617	21	30	AGTGTTTCCTACTTTATGGAA	miRNA	miR-142*
t0014724	23	30	TACCGCAGGGTAGAACCACGGAC	miRNA	miR-140
t0014727	24	30	CTACCACAGGGTAGAACCACGGAC	miRNA	miR-140
t0014846	22	30	ACCACAGGGTAGAATTACGGAA	miRNA	miR-140
t0015082	26	30	TACCACAGGGTAGAACCACGGAAGAA	miRNA	miR-140
t0014928	22	30	ACCACAGCGTAGAACCACGGAA	miRNA	miR-140
t0014729	21	30	TTCGAGGCTACAGTGAGATGT	miRNA	miR-1304
t0014751	20	30	TCTGGGCAACAAAGTGAGAC	miRNA	miR-1285
t0014905	22	30	AGGATGAGCAAAGAAAGTAGAT	miRNA	miR-1255a
t0014939	22	30	TAAAGTGCTGACAGTGTAGATA	miRNA	miR-106b
t0014745	23	30	AGCAGCATTGCACAGGGCTATGA	miRNA	miR-103a
t0014628	22	30	AGCAGGATTGTACAGGGCTATG	miRNA	miR-103a
t0014731	20	30	AAGCAGCATTGTACAGGGCA	miRNA	miR-103a
t0014708	20	30	GCAGCATTGTACAGGGCTAT	miRNA	miR-103a

t0014710	19	30	TACAGTACTGTGATAACTA	miRNA	miR-101
t0014781	21	30	TACGGTACTGTGATAACTGAA	miRNA	miR-101
t0014785	21	30	TACAGTAATGTGATAACTGAA	miRNA	miR-101
t0014811	26	30	TACAGTACTGTGATAACTGAATCCAC	miRNA	miR-101
t0015049	21	30	TGAGGTAGTAGTTTGTGCTGC	miRNA	let-7i
t0015044	22	30	TGAGGTAGAAGTTTGTACAGTT	miRNA	let-7g
t0014882	21	30	TGAGGTAGTAGATTGTATCTT	miRNA	let-7f-5p
t0014954	19	30	TGAGGTAGCAGATTGTATA	miRNA	let-7f
t0014879	22	30	TGAGGTAGTAGATCGTATAGTA	miRNA	let-7f
t0014635	21	30	AGAGGTAGTAGGTTGAATAGT	miRNA	let-7d
t0014706	22	30	AGAGGTAGTGGGTTGCATAGTT	miRNA	let-7d
t0014935	20	30	AGAGGAAGTAGGTTGCATAG	miRNA	let-7d
t0015022	25	30	TGAGGTAGTAGGTTGTATGGCTATC	miRNA	let-7c
t0014768	22	30	TGAGGTAGTAGGGTGTGTGGGT	miRNA	let-7b
t0014986	20	30	TGAGTTAGTAGGTTGTGTGG	miRNA	let-7b
t0014996	20	30	TGAGGTAGTAGGTTGTGTTG	miRNA	let-7b
t0015014	23	30	TGAGGTAGTAGGTTGTATAGATC	miRNA	let-7
t0014807	23	29	AGGGACGGGACGCGGTGCAGTAG	miRNA	miR-92b*
t0014716	21	29	TATTCCACTTGTCCCGGCCTG	miRNA	miR-92a
t0014804	23	29	TATTGCACTCGTCCCGGCCATTT	miRNA	miR-92
t0014773	21	29	GGGCAGGTGGTGTAGTGGTAT	miRNA	miR-885
t0014827	26	29	GTAGAGGAGATGGCGCAGGGGACTTT	miRNA	miR-877
t0014855	26	29	AAGTTGGATTGTGACTGGCACATTTT	miRNA	miR-753c
t0014867	22	29	TGAAAGACAAGGGTAGTGAGAT	miRNA	miR-71
t0015043	22	29	CCTCCCACACCCAAGGCTTGCT	miRNA	miR-532-3p
t0015039	28	29	TCCTGTACTGAGCTGCCCCGAGCGGCAT	miRNA	miR-486-5p
t0014682	22	29	TTCTGTACTGAGCTGCCTCGAG	miRNA	miR-486-5p
t0014829	26	29	TCCTGTACTGAGCTGCCCCGAGGACG	miRNA	miR-486-5p
t0015065	29	29	AAGGGCTGGGTCGGTCGGGCTGGGGCGC	miRNA	miR-4651
t0014898	21	29	TCGACTCTGGATAGCTGGGCT	miRNA	miR-4538
t0015524	22	29	AGAAGGGTCGGGGCGGCAGGGG	miRNA	miR-4516
t0015419	25	29	AGTTACCATTACTGAGTTTAAACCA	miRNA	miR-451
t0015162	27	29	AAACCGTTACCATTACTGAGTTCGTCG	miRNA	miR-451
t0015310	21	29	AAACCGGTACCATTACTGAGT	miRNA	miR-451
t0015384	23	29	AAACCTTACCATTACTGAGTTTA	miRNA	miR-451
t0015333	20	29	ACCCCACTCCTGGTACCAAA	miRNA	miR-4286
t0015111	24	29	TGAGGGGCAGAGAGCGAGACTAAA	miRNA	miR-423-5p
t0015315	22	29	TGAGCGGCAGAGAGCGAGACTT	miRNA	miR-423-5p
t0015159	25	29	GAAGTTCTTCGTGGTGGATTCTATC	miRNA	miR-382
t0015196	22	29	ACTGGACTGGGAGTCAGAAGGA	miRNA	miR-378c
t0015372	23	29	ACTGGACTTGGAGTCAGAAGGTA	miRNA	miR-378
t0015330	23	29	GAGGACTGAAGTGGGGAAAGGTT	miRNA	miR-3526
t0015405	24	29	TGAAGACTGAAGTGGAGAAGGGTA	miRNA	miR-3526
t0015563	25	29	ACTTTGAAGACTGAAGTGGAGAAGG	miRNA	miR-3526
t0015195	21	29	TGAGTAGTAGGTTGTATAGTT	miRNA	miR-352
t0015597	20	29	CAAGTGGAATCCAGGACGCG	miRNA	miR-3503
t0015607	19	29	TTATAAAGCAATGAGACTG	miRNA	miR-340-5p
t0015614	21	29	AAAGCACACGGCCTGCAGAGT	miRNA	miR-330-3p
t0015151	22	29	AAAAGCTGGGTTGAGAGGGGGT	miRNA	miR-320d
t0015352	22	29	GAAAAGTTGGGTTGAGAGGGCT	miRNA	miR-320d
t0015394	25	29	AAAAGCTGGGTTGAGAGGGCGAAAA	miRNA	miR-320a
t0015576	22	29	AAAGGCTGGGTTGAGAGGGCGA	miRNA	miR-320a
t0015185	22	29	ACAAGCTGGGTTGAGAGGGCGA	miRNA	miR-320a
t0015550	23	29	GGCTGGTCCGAGTGCAGTGGTGA	miRNA	miR-3135b
t0015379	26	29	TGTAAACATTCTTGACTTGAAGCATC	miRNA	miR-30e-5p
t0015444	26	29	CGCGGGTCGGGGGGCGGGGCGGACTG	miRNA	miR-2981
t0015611	21	29	AGGGCCCCCCCTCAATCCTGT	miRNA	miR-296-5p
t0015280	20	29	GAGGGTTGGGTGGAGGCTAA	miRNA	miR-296-3p

t0015620	21	29	TCGGGGGGCGGGGCGGACTGT	miRNA	miR-2885
t0015308	23	29	CTTCAAGTAATCCAGGATAGGCA	miRNA	miR-26a
t0015146	23	29	TTCAAGTAATCCAGGATAGGCAT	miRNA	miR-26a
t0015192	23	29	CTTCAAGTAATCCAGGATAGGCT	miRNA	miR-26a
t0015459	21	29	TTCAAGTAATCCAGGATAGGA	miRNA	miR-26a
t0015140	20	29	CATTGCATTTGTCTCGGTCT	miRNA	miR-25
t0015244	25	29	GACTGGGGCGGTACATCTGTTAAAG	miRNA	miR-219-2-3p
t0015406	21	29	CTGACCTATGAATTGACCGCC	miRNA	miR-192
t0015596	19	29	CTGACCTATGAATTGACAG	miRNA	miR-192
t0015275	23	29	TGACCTATGAATTGACAGCCAAT	miRNA	miR-192
t0015489	23	29	TGGAGAGAAAGGCAGTTCTTGAT	miRNA	miR-185
t0015402	21	29	TGGCGAGAAAGGCAGTTCCTG	miRNA	miR-185
t0015622	19	29	TGGAGAGAAAGGCAGTTCG	miRNA	miR-185
t0015412	20	29	AAGGTAGATAGAACAGGTTT	miRNA	miR-1839
t0015442	19	29	AACATTCATTGTTGTCGGT	miRNA	miR-181d-5p
t0015485	24	29	TAGCAGCACATCATGGTTTAAGTT	miRNA	miR-15b
t0015510	19	29	TATCAGCACATCATGGTTT	miRNA	miR-15b
t0015557	20	29	TAACAGCACATCATGGTTTA	miRNA	miR-15b
t0015449	25	29	TCAGTGCACTACAGAACTTTGAGTG	miRNA	miR-148a
t0015593	21	29	CTACAGTATAGATGATGTATT	miRNA	miR-144
t0015361	23	29	TACAGTATAGATGATGTACTAAA	miRNA	miR-144
t0015447	20	29	TCCATAAAGTAGAAAGCACT	miRNA	miR-142-5p
t0015548	24	29	TACCACAGGGTAGAACCACGGAGT	miRNA	miR-140
t0015095	22	29	ACCACAGGGTAGAACCCCGGAA	miRNA	miR-140
t0015456	23	29	ACCACAGGGTAGAACCACGGCCG	miRNA	miR-140
t0015590	19	29	TACAGGGTAGAACCACGGA	miRNA	miR-140
t0015205	22	29	ACCACAGGGTAGAACCCCGGAT	miRNA	miR-140
t0015091	26	29	ACCACAGGGTAGAACCACGGACGGTG	miRNA	miR-140
t0015337	22	29	ACCACAGGGTAGAAACACGGAT	miRNA	miR-140
t0015425	21	29	ATAAAGTGCTGACAGTGCAGA	miRNA	miR-106b
t0015619	21	29	TAAAGTTCTGACAGTGCAGAT	miRNA	miR-106b
t0015279	22	29	AAAGTGCTTACAGTGCAGGTAA	miRNA	miR-106
t0015475	22	29	ACCAGCATTGTACAGGGCTATG	miRNA	miR-103a
t0015572	19	29	AGCAGCATTGTATAGGGCT	miRNA	miR-103a
t0015303	24	29	TACAGTACTGTGATAACTGACGGT	miRNA	miR-101c
t0015222	22	29	TGAGGTAGTAGATTGAATAGTC	miRNA	let-7k
t0015294	22	29	TGAGTTAGTAGTTTGTGCTGTA	miRNA	let-7i
t0015312	21	29	TGATGTAGTAGTTTGTACAGT	miRNA	let-7g
t0015356	19	29	GAGGTAGTAGATTGTAGTT	miRNA	let-7f-5p
t0015467	21	29	TGAGGTAGTAGATTTCATAGT	miRNA	let-7f
t0015495	28	29	TGAGGTAGTAGATTGTATAGTTGGTAGT	miRNA	let-7f
t0015615	22	29	ACAGGTAGTAGGTTGCATAGTT	miRNA	let-7d
t0015229	22	29	AGAGGTAGCAGGTTGCATAGTT	miRNA	let-7d
t0015520	23	29	TGAGGTAGTAGGTTGTGTAGTTT	miRNA	let-7b
t0015571	23	29	TGAGGTAGTAGGTTGTGTGAATC	miRNA	let-7b
t0015289	23	29	TGAGGTAGTAGGTTGTATAGGGA	miRNA	let-7-5p
t0015525	20	29	TGAGGTAGTAGGTTGTTTAG	miRNA	let-7
t0015254	26	29	TGAGGTAGGAGGTTGTATAGTTGATC	miRNA	let-7
t0015163	21	29	TGAGGTAGTAAGTTGTATAGT	miRNA	let-7
t0015600	24	29	TGAGGTAGTAGGTTGTATAGTTTT	miRNA	let-7
t0015621	23	28	AGGACTGACGTGGAGAAGGGTTC	miRNA	miR-739
t0015598	19	28	GAAGACTGAAGTGGAGAAG	miRNA	miR-739
t0015296	22	28	AAGTCATTGGAGGGTTTGAGCA	miRNA	miR-616
t0015561	23	28	ACCTCCCACACCCAAGGCTTGCA	miRNA	miR-532-3p
t0015131	19	28	TAGCAGCGGGAACAGTTCA	miRNA	miR-503
t0015588	25	28	TCCTGTACTGAGCTGCCCCGAGTAG	miRNA	miR-486-5p
t0015154	24	28	TCCTGTACTGAGTTGCCCCGAGAG	miRNA	miR-486-5p
t0015396	21	28	TACTGTACTGAGCTGCCCCGA	miRNA	miR-486-5p

t0015257	21	28	ATCCTGTACTGAGCTGCCTCG	miRNA	miR-486-5p
t0015256	28	28	TCCTGTACTGAGCTGCCCCGAGAATATC	miRNA	miR-486-5p
t0015335	22	28	TCCTGTACCGAGCTGCCCCGAG	miRNA	miR-486-5p
t0015390	22	28	TCCTGTGCTGAGCTGCCCCGAG	miRNA	miR-486-5p
t0015558	25	28	TCCTGTACTGAGCTGCCCCGAGAGT	miRNA	miR-486-5p
t0015953	23	28	TCCTGTACTGAGCTGCACCGAGA	miRNA	miR-486-5p
t0016134	23	28	CGGGGCAGCTCAGTACAGGATGA	miRNA	miR-486-3p
t0015944	22	28	GTAGAATTGTGTCGGCAGATCT	miRNA	miR-4860
t0015656	22	28	TAGGATTGTGTCGGCAGATCTT	miRNA	miR-4860
t0015850	20	28	TGTAGAGCAGGGAGCAGGAG	miRNA	miR-4732-5p
t0015730	21	28	GGGGGGAGAAGGGTCGGGGCG	miRNA	miR-4516
t0015816	19	28	GGGGAGAGAAGGGTCGGGG	miRNA	miR-4516
t0015902	21	28	AAACCGTTACCATTCCTGAGT	miRNA	miR-451
t0015989	23	28	AAACCGTTACAATTACTGAGTTT	miRNA	miR-451
t0016012	20	28	CAGCAGCAATTCATTTTTTG	miRNA	miR-424
t0016117	19	28	TGAGGCAGAGAGCGAGACT	miRNA	miR-423-5p
t0016118	20	28	TGAGGGGCAGAGAGCGATAC	miRNA	miR-423-5p
t0016139	23	28	TGAGGGGCAGAGAGCGAGTCTTT	miRNA	miR-423-5p
t0016177	23	28	TGAGGGTCAGAGAGCGAGACTTA	miRNA	miR-423-5p
t0015678	21	28	TGGGGCAGAGAGCGAGACTTT	miRNA	miR-423-5p
t0015824	23	28	TGAGGGGCAGAGAGCTAGACTTA	miRNA	miR-423-5p
t0016072	21	28	ATCAACAGACATTAATTGGGC	miRNA	miR-421
t0015710	19	28	TGTAGTAGTTTGTACAGTT	miRNA	miR-3962
t0015897	22	28	AATTGCACGGTATTTATCTGTA	miRNA	miR-363
t0016145	23	28	AGGACTGAAGTGGGGAAAGGTTC	miRNA	miR-3526
t0015775	23	28	TCTCACACAGAAATCGCACTCGT	miRNA	miR-342-3p
t0015855	20	28	GCCCCTGGGCCTATCCTAGA	miRNA	miR-331
t0015653	22	28	AAAGCTGGGTTGAGAGGGCATT	miRNA	miR-320d
t0015625	22	28	AAAAGCTGGGATGAGAGGGCGA	miRNA	miR-320a
t0015756	27	28	AAAAGCTGGGTTGAGAGGGCGAATTTT	miRNA	miR-320a
t0015835	22	28	GTGGAAGGTAGACGGCCAGAGA	miRNA	miR-3190
t0015869	21	28	TATAAAATGAGGGCAGTAAGA	miRNA	miR-3163
t0015965	22	28	TATAAAATGAGGGCAGTAAGAC	miRNA	miR-3163
t0016151	23	28	TATAAAATGGGGGTAGTAAGACG	miRNA	miR-3163
t0015957	23	28	TATAAAATGAGGGCAGTAAGACA	miRNA	miR-3163
t0015901	20	28	TGGGACAAGAGGACGGTCTT	miRNA	miR-3122
t0016121	29	28	CGCGCGGGTCGGGGGGCGGGGCGGACT(	miRNA	miR-2981
t0016169	21	28	ACAAGTAATCCAGGATAGGTT	miRNA	miR-26a
t0015672	21	28	AGGCGGAGACTTGGGCAATAA	miRNA	miR-25*
t0015974	21	28	CATTGCACTTGTCTCGCTCTG	miRNA	miR-25
t0015684	21	28	ATCACATTGCCAGGGATTTCG	miRNA	miR-23a
t0015727	22	28	AAGCTGTCAGTTGAAGAACTGT	miRNA	miR-22-3p
t0016062	23	28	CTGTGCGTGTGACAGCGGCTGAT	miRNA	miR-210
t0015963	24	28	CAACGGAATCCCAAAAGCAGCTAT	miRNA	miR-191
t0015734	28	28	CAACGGAATCCCAAAAGCAGCTGTCGGC	miRNA	miR-191
t0015843	23	28	TGGAGAGAAAGGCAGTTCCCGAA	miRNA	miR-185
t0015911	20	28	TGGAGGGAAAGGCAGTTCCT	miRNA	miR-185
t0016059	23	28	TGGAGAGAAAGGCGGTTCCTGAT	miRNA	miR-185
t0016095	21	28	TGGAGAGAAAGGCAGTTGCTG	miRNA	miR-185
t0015640	22	28	AACATTCAACTCTGTCGGTGAG	miRNA	miR-181a
t0016175	19	28	TAGTACGTAAATATTGGCG	miRNA	miR-16c
t0016098	22	28	GAGCAGCACGTAAATATTGGCG	miRNA	miR-16
t0015788	24	28	GTAGCAGCACGTAAATATTGGCGT	miRNA	miR-16
t0016005	19	28	TAGCAGCATATAATGGTTT	miRNA	miR-15a
t0016164	21	28	CTAGACTGAAGCTCCTTGAGG	miRNA	miR-151-3p
t0016100	20	28	CTAGACTGAAGCTTCTTGAG	miRNA	miR-151-3p
t0016080	22	28	TCAGTGCATTACAGAACTTTGA	miRNA	miR-148
t0016143	19	28	TACAGTATAGATGATGTAG	miRNA	miR-144

t0015882	23	28	TACAGTATAGATGATGTACTATT	miRNA	miR-144
t0016019	20	28	ATACAGTATAGATGATGTAA	miRNA	miR-144
t0016101	21	28	ACCACAGGGTAGCACCACGGA	miRNA	miR-140
t0016132	21	28	ACCACCGGGTAGAACCACGGA	miRNA	miR-140
t0015717	20	28	ACCACAGGGTAGAACGACGG	miRNA	miR-140
t0015747	23	28	TACCACTGGGTAGAACCACGGAT	miRNA	miR-140
t0015701	21	28	ACCACAGGGTAGAACTACGGT	miRNA	miR-140
t0015706	23	28	TACCACAGGGTGGAACCACGGAA	miRNA	miR-140
t0016085	23	28	TACCACAGGGTAGAAATACGGAA	miRNA	miR-140
t0016018	23	28	TACCACAGGGCAGAACCACGGAA	miRNA	miR-140
t0016077	23	28	TACCACAGGGTAGAACCCCGGAG	miRNA	miR-140
t0016188	25	28	CTCGGCGTGGCGTCGGTCGTGTAGA	miRNA	miR-1307
t0015627	22	28	TTCGAGGCTACAGTGAGATGTG	miRNA	miR-1304
t0015904	21	28	CGTGCCACCCTTTTCCCCAGT	miRNA	miR-1227
t0015958	23	28	AAGCAGCATTGTACAGGGCTATA	miRNA	miR-107
t0015691	22	28	ATAAAGTGCTGACAGTGCAGAT	miRNA	miR-106b
t0015721	23	28	TAAAGTGCTGACAGTGCAGATAT	miRNA	miR-106b
t0015754	22	28	AGCAGCATTGTACGGGGCTATG	miRNA	miR-103a
t0015804	20	28	TACAGTACAGTGATAACTGA	miRNA	miR-101
t0015916	22	28	GTACAGTACTGTGACAACTGAA	miRNA	miR-101
t0015962	23	28	TGAGGTAGTAGTTTGTGCTTTTT	miRNA	let-7i
t0016035	21	28	TGAGGAAGTAGTTTGTACAGT	miRNA	let-7g
t0016039	21	28	TGAGGTAGTAGTTTGTAAAGT	miRNA	let-7g
t0016091	21	28	TGAGGTAGTATTTTGTACAGT	miRNA	let-7g
t0015810	24	28	TGAGGTAGTAGATTGTATAGCAGA	miRNA	let-7f-5p
t0015817	20	28	TGAGGTAGTAGATTGTAAGT	miRNA	let-7f-5p
t0015941	22	28	TGAGGTAGTAGATTGTATATTA	miRNA	let-7f-5p
t0015759	24	28	TGAGGTAGTAGATTGTATAGTGTT	miRNA	let-7f
t0015803	24	28	AGTGAGGTAGTAGATTGTATAGTT	miRNA	let-7f
t0016138	21	28	TGAGGTAGTAGATTGTGTACT	miRNA	let-7f
t0016154	19	28	TGGTAGTAGATTGTATAGT	miRNA	let-7f
t0015976	21	28	TGAGGTAGTAGATAGTATAGT	miRNA	let-7f
t0016078	27	28	TGAGGTAGTAGGTTGTGTGGTTAAAAA	miRNA	let-7b
t0015750	23	28	TGAGGTAGTAGGTTGTATAGTAT	miRNA	let-7-5p
t0015793	20	28	GTGAGGTAGTAGGTTGTATA	miRNA	let-7
t0016022	21	28	GGTAGTAGGTTGTATAGTTGA	miRNA	let-7
t0016174	26	28	TGAGGTAGTAGGTTGTATAGTTAAAC	miRNA	let-7
t0015927	20	28	TGAGGTAGGAGGTTGTATAG	miRNA	let-7
t0016008	19	27	GTGGAAGGTTGTAGGCATG	miRNA	miR-965-5p
t0016142	21	27	TCTTCTCTGTTTTGGCCATGT	miRNA	miR-942
t0015626	21	27	TATTGCACTCGTCCCGGCATA	miRNA	miR-92
t0015688	25	27	TACGTCATCGTTGTCATCGTCAATT	miRNA	miR-598
t0015694	22	27	AAAAACTGAGACTACTTTTGCA	miRNA	miR-548e
t0015695	20	27	AAAACCCGCAATTACTTTTG	miRNA	miR-548ac
t0015930	24	27	TCCTGTACTGAGCTGCCCCGACGT	miRNA	miR-486-5p
t0015732	21	27	TCCTGTACTGGGCTGCCCCGA	miRNA	miR-486-5p
t0016000	23	27	TCCTGTACTGAGATGCCCCGAGA	miRNA	miR-486-5p
t0015680	29	27	TCCTGTACTGAGCTGCCCCGAGTCGGCAT	miRNA	miR-486-5p
t0015762	21	27	TCTCGTACTGAGCTGCCCCGA	miRNA	miR-486-5p
t0015830	20	27	TCTCCCTTCCTGTTCTGGCT	miRNA	miR-4685-3p
t0016054	22	27	AAGGGCTGTGTCGGTCGGGCTA	miRNA	miR-4651
t0016235	20	27	GGCTGGGTCGGTCGGGCTGG	miRNA	miR-4651
t0016560	21	27	AGAAGGGTCGGGGCGGCAGGT	miRNA	miR-4516
t0016214	20	27	AAACCGTTACAATTACTGAG	miRNA	miR-451
t0016224	22	27	AAACCGTTACGATTACTGAGTT	miRNA	miR-451
t0016343	22	27	AAGCCGTTACCATTACTGAGTT	miRNA	miR-451
t0016503	29	27	AAACCGTTACCATTACTGAGTTGGGGTAG	miRNA	miR-451
t0016393	21	27	AAACCGTTACCATCACTGAGT	miRNA	miR-451

t0016555	21	27	AATCGTTACCATTACTGAGTT	miRNA	miR-451
t0016643	21	27	AAACCGTTAGCATTACTGAGT	miRNA	miR-451
t0016649	25	27	CGAAACCGTTACCATTACTGAGTTT	miRNA	miR-451
t0016778	22	27	TGAGGGGCAGCGAGCGAGACTT	miRNA	miR-423-5p
t0016776	19	27	TGATGGGCAGAGAGCGAGA	miRNA	miR-423-5p
t0016445	21	27	TGAGGGGCAGAGAGCGAGCTT	miRNA	miR-423-5p
t0016487	23	27	TGAGGTGCAGAGAGCGAGACTTA	miRNA	miR-423-5p
t0016704	24	27	TGAGTGGCAGAGAGCGAGACTTTA	miRNA	miR-423-5p
t0016221	22	27	ACTGGACTTGGAGTTAGAAGGC	miRNA	miR-378
t0016310	22	27	TGAGGATGGATAGCAAGGAAGT	miRNA	miR-3605-5p
t0016480	24	27	TGAAGACTGAAGTGGAGAAGGTTT	miRNA	miR-3526
t0016556	25	27	TCTCACACAGAAATCGCACCCGTAT	miRNA	miR-342-3p
t0016667	25	27	AAAAGCTGGGTTGAGAGGGCGGATT	miRNA	miR-320d
t0016672	23	27	AAAACCTGGGTTGAGAGGGCGTT	miRNA	miR-320d
t0016674	21	27	AAAATTGGGTTGAGAGGGCGT	miRNA	miR-320d
t0016727	22	27	AAAAGCTGGGTTAAGAGGGCGA	miRNA	miR-320a
t0016314	32	27	TGTAAACATCCTACACTCAGCTAGACATG	miRNA	miR-30b
t0016633	19	27	TAGCATTTGAAATCGGTTA	miRNA	miR-29c
t0016650	20	27	GGCGGATGTAGCCAAGGGGA	miRNA	miR-270
t0016760	22	27	TTCAAGTAATTCAGGATAGGTG	miRNA	miR-26b
t0016789	22	27	TTCAAGTAATTCAGGATAGGCA	miRNA	miR-26b
t0016604	22	27	AGGCGGAGACTTGGGCAATTGT	miRNA	miR-25*
t0016219	22	27	CATTGCACTTGTCTAGGTCTGA	miRNA	miR-25
t0016500	24	27	TGGCTCAGTTCAGCAGGAACAGTA	miRNA	miR-24
t0016692	24	27	ATCACATTGCCAGGGATTACCACT	miRNA	miR-23b
t0016318	21	27	ATCACATTGCCAGGGATTACC	miRNA	miR-23b
t0016747	21	27	AAGCTGCCAGTTGAAGAATTG	miRNA	miR-22-3p
t0016793	22	27	AGCTACATCTGGCTACTGGGAA	miRNA	miR-222
t0016458	22	27	AGTTCTTCAGTGGCAAGCTTTA	miRNA	miR-22*
t0016352	20	27	TGAGGTAGGTAGGTTGTATA	miRNA	miR-1961
t0016416	21	27	TTGACCTATGAATTGACAGCC	miRNA	miR-192
t0016631	24	27	CTGACCTATGAATTGACAGCCAGA	miRNA	miR-192
t0016456	19	27	CAACGGAATTCCAAAAGCA	miRNA	miR-191
t0016546	21	27	AACGGAATCCCAAGAGCAGCT	miRNA	miR-191
t0016684	22	27	TGGAGAGAAAGGCAGTTCCTAG	miRNA	miR-185
t0016197	21	27	TGGAGAGAGAGGCAGTTCCTG	miRNA	miR-185
t0016262	22	27	GGAGAGAAAGGCAGTTCCTGAA	miRNA	miR-185
t0016258	22	27	TGGAGAGAAAGGCAGTTCCGGA	miRNA	miR-185
t0016469	25	27	TCGACGGAGAACTTATAAGGGCATC	miRNA	miR-184-3p
t0016428	22	27	TGAGGGAGTAGATTGTGTAGTT	miRNA	miR-1827
t0016266	26	27	TTTGGCAATGGTAGAACTCACACAAG	miRNA	miR-182
t0016659	20	27	TAGCAGCACGTAACTATTGG	miRNA	miR-16
t0016305	19	27	TAGCAGCACGTAAATAGCG	miRNA	miR-16
t0016281	20	27	TAGCAGCACTTAAATATTGG	miRNA	miR-16
t0016712	20	27	TAGCAGCACCTCATGGTTTA	miRNA	miR-15b
t0016225	18	27	TAGCAGCACATCATGTTA	miRNA	miR-15b
t0016272	18	27	TAGCAGCATATCATGGTA	miRNA	miR-15b
t0016436	19	27	TAGCAGCACATCATGTTTT	miRNA	miR-15b
t0016441	22	27	TAGCAGTACATAATGGTTTGTG	miRNA	miR-15a
t0016517	20	27	TAGAAGCACATAATGGTTTG	miRNA	miR-15a
t0016714	24	27	TCAGTGTATGACAGAACCTGGATC	miRNA	miR-152
t0016511	24	27	TACCACATGGTAGAACCACGGAAA	miRNA	miR-140
t0016439	22	27	ACAACAGGGTAGAACCACGGAA	miRNA	miR-140
t0016453	23	27	ACCACAGGGTAGAACCACGGTAA	miRNA	miR-140
t0016202	23	27	TACAACAGGGTAGAACCACGGAA	miRNA	miR-140
t0016223	24	27	TACCACAGGGTAGAACCACAGACA	miRNA	miR-140
t0016543	23	27	ACCATAGGGTAGAACCACGGACA	miRNA	miR-140
t0016434	22	27	ACCACAGGGTACAACCACGGAC	miRNA	miR-140

t0016605	22	27 TACCACAGGGTAGCACCACGGA	miRNA	miR-140
t0016613	24	27 CAGTGCAATGTTAAAAGGGCATTT	miRNA	miR-130a
t0016756	21	27 TTCTGGAATTTGTGTGAGGGA	miRNA	miR-1299
t0016621	20	27 TAAAGTGCTGACAGGGCAGA	miRNA	miR-106b
t0016772	22	27 AGCAGCATTGTGCAGGGCTATG	miRNA	miR-103a
t0016312	19	27 CAGCATTGTACAGGGCTAT	miRNA	miR-103a
t0016347	23	27 TACAGTACTGTGATAACTGAATA	miRNA	miR-101
t0016463	22	27 TGAGGTAGTAGTTCGTACAGTT	miRNA	let-7g
t0016590	31	27 TGAGGTAGTAGATTGTATAGTACCACTCT(	miRNA	let-7f
t0016595	23	27 TGAGGTAGTAGATTGTATAATTG	miRNA	let-7f
t0016624	25	27 TGAGGTAGGAGATTGTATAGTTATC	miRNA	let-7e
t0016742	22	27 CTATACGACCTGCTGCCTTTCT	miRNA	let-7d*
t0016769	25	27 TGAGGTAGTAGGTTGTGTGGTTGAA	miRNA	let-7b
t0016797	27	27 TGAGGTAGTAGGTTGTGTGGTTTGATC	miRNA	let-7b
t0016552	27	27 TGAGGTAGTAGGTTGTATGGTTATATC	miRNA	let-7b
t0016350	22	27 TGAGGTAGTAGGTTGTAAAGTT	miRNA	let-7
t0016346	20	26 AACCCGTAGATCCGATCTTG	miRNA	miR-99a
t0016277	23	26 CACCCGGCTGTGTGCACATGTGT	miRNA	miR-941
t0016617	21	26 TATTGCACTTGTCTCGGCCTG	miRNA	miR-92a
t0016279	21	26 TATTGCACTTGTCCCGGCTTG	miRNA	miR-92a
t0016411	23	26 GGAGACTGTGAAGCGGGCACGCC	miRNA	miR-920
t0016583	20	26 TATTGCACTCGTTCCGGCCT	miRNA	miR-92
t0016588	22	26 TGCGGGGCTAGGGCTAATAGCA	miRNA	miR-744
t0016715	25	26 AGGGCGCGCGGGTCGGGGCGGCGTT	miRNA	miR-638
t0016538	23	26 GACTATAGAACTTTCTCCCTAAA	miRNA	miR-625*
t0016438	22	26 GTAATTTTATGTATAAGCTAGT	miRNA	miR-590-3p
t0016735	19	26 CAAAAGTGATCGTGGTTTT	miRNA	miR-548t
t0016787	19	26 CCCACACCCAAGGCTTGCA	miRNA	miR-532-3p
t0016407	24	26 TAGCAGCGGGAACAGTTCTGAAGA	miRNA	miR-503
t0016866	18	26 AATCCTTGCTATCTGGGT	miRNA	miR-500b
t0017242	29	26 TCCTGTACTGAGCTGCCCCGAGATGAGC(	miRNA	miR-486-5p
t0016850	21	26 TCTGTACTGAGCTGCCCCGAG	miRNA	miR-486-5p
t0016885	21	26 TCCTGTGCTGAGCTGCCCCGA	miRNA	miR-486-5p
t0017214	28	26 TCCTGTACTGAGCTGCCCCGAGATGGCT	miRNA	miR-486-5p
t0017133	23	26 TCCTGTACTGAGCTACCCCGAGA	miRNA	miR-486-5p
t0017347	24	26 TGTAGGATTGTGTCGGCAGATTTT	miRNA	miR-4860
t0017091	22	26 TAAATGTAGAATTGTGTCGGTT	miRNA	miR-4860
t0016953	22	26 CTGTAGAGCAGGGAGCAGGAAG	miRNA	miR-4732-5p
t0016819	20	26 AGCAGGCGAGGCTGGGCTGA	miRNA	miR-4690-5p
t0017208	21	26 AGGGCTGGGTCGGTCGGGCTG	miRNA	miR-4651
t0016925	30	26 AGGGCTGGGTCGGTCGGGCTGGGGCGCA	miRNA	miR-4651
t0016988	24	26 AGAGAAGGGTCGGGGCGGCAGGGG	miRNA	miR-4516
t0017180	23	26 AAACCATTACCATTACTGAGTTT	miRNA	miR-451
t0016803	21	26 AAACCCTTACCATTAGTGAGT	miRNA	miR-451
t0016825	26	26 GATAAACCGTTACCATTACTGAGTTT	miRNA	miR-451
t0016877	21	26 AAACCGTTACCATTTCAGAGT	miRNA	miR-451
t0016945	23	26 TTCGGGGTCTGGGCGCGGCGAGT	miRNA	miR-4508
t0017125	19	26 TTTGGAGGAGATGCGGTTA	miRNA	miR-4443
t0016837	23	26 TCCCACTGCTTCACTTGACTAGC	miRNA	miR-4301
t0017352	24	26 TGAGGGGCAGAGAGTGAGACTTTA	miRNA	miR-423-5p
t0016820	23	26 TGTGGGGCAGAGAGCGAGACTTT	miRNA	miR-423-5p
t0017108	24	26 TGAGGGGCAGAGAGCCAGACTTTT	miRNA	miR-423-5p
t0017043	24	26 CTGAGGGGCAGAGAGCTAGACTTT	miRNA	miR-423-5p
t0017060	19	26 CAAGGAAAGTACTAGGTTG	miRNA	miR-4005a-3p
t0017307	22	26 TGTCCTCTAGGGCCTGCAGTCG	miRNA	miR-3909
t0016989	25	26 ACTGGACTTGGACTCAGAAGGAATC	miRNA	miR-378c
t0017157	23	26 CCGGGGGTGGGGTCGGCGGGGGA	miRNA	miR-3621
t0017253	22	26 AAAAGCTGGGTTGAGAGGGTAA	miRNA	miR-320d

t0017254	23	26	AAAAGCTGGGTTGGGAGGGCGAA	miRNA	miR-320a
t0016893	22	26	AAAAGCTGGGTTGAGAGCGCGA	miRNA	miR-320a
t0016804	26	26	GAATCTGAGAAGGCGCACAAGGTAGA	miRNA	miR-3200-5p
t0016839	21	26	GTGGAAGGTAGACGGCCAGTT	miRNA	miR-3190
t0016903	26	26	TGTAAACATCCCAGACTGGAAGCATC	miRNA	miR-30a-5p
t0016981	22	26	TAGCATTATCTGAAATCGGTTT	miRNA	miR-29e
t0017109	23	26	TTCAAGTAATTCAGGATAGGTTT	miRNA	miR-26b
t0017195	22	26	CATTGCATTTGTCTCGGTCTGT	miRNA	miR-25
t0017044	24	26	TGTCAGTTTGTCAAATACCCCAAA	miRNA	miR-223
t0017281	28	26	CGGACAACGGAATCCCAAAAGCAGCTGT	miRNA	miR-191
t0017237	21	26	GCCCAAAGGTGAATTTTTTGG	miRNA	miR-186*
t0016991	25	26	TGGAGAGAAAGGCAGTTCCTGATGG	miRNA	miR-185
t0016902	23	26	CGGAGAGAAAGGCAGTTCCTGAA	miRNA	miR-185
t0017112	25	26	TGGAGAGAAAGGCAGTTCCTGAAAG	miRNA	miR-185
t0017145	24	26	TGGACGGAGAACTGATAAGGGCTT	miRNA	miR-184-3p
t0017359	21	26	AGACCTACTTATCTACCAATT	miRNA	miR-1839-3p
t0016865	20	26	TGAATTACCGAAGGGCCATA	miRNA	miR-183*
t0017105	21	26	AACATTCATTGCTGTCGGTGG	miRNA	miR-181b
t0017181	20	26	TAGCAGCACGCAAATATTGG	miRNA	miR-16
t0016964	19	26	TAGCAGTACATCATGGTTA	miRNA	miR-15b
t0017213	19	26	AAGCAGCACATCATGGTTA	miRNA	miR-15b
t0017052	25	26	TGAGAACTGAATTCCATAGGCTATC	miRNA	miR-146b-5p
t0017368	24	26	TGAGAACTGAATTCCATAGGCTGT	miRNA	miR-146b
t0017364	21	26	GATATCATCATATACTGTAAG	miRNA	miR-144*
t0016975	18	26	TACAGTGTAGATGATGTA	miRNA	miR-144
t0017046	23	26	TGAGATGAAGCACCGTAGCTATC	miRNA	miR-143
t0017144	22	26	ACCATAGGGTAGAACCACGGAT	miRNA	miR-140
t0017206	25	26	TACCACAGGGTAGAACCACGGATAA	miRNA	miR-140
t0017078	22	26	ACCACAGGGTTGAACCACGGAA	miRNA	miR-140
t0016982	21	26	ACCACAGGGTATAACCACGGA	miRNA	miR-140
t0017365	22	26	ACCACAGGGGAGAACCACGGAT	miRNA	miR-140
t0017027	23	26	ACCACAGGGTAGAACTACGGAAT	miRNA	miR-140
t0017134	21	26	ACTCGGCGTGGCGTCGGTCGT	miRNA	miR-1307
t0017234	20	26	TCGGCGTGGCGTCGGTCGTG	miRNA	miR-1307
t0017166	21	26	TCTGGGCAACAAAGTGAGACT	miRNA	miR-1285
t0016915	22	26	TCACAGTGAACCGGTCTCTTAG	miRNA	miR-128
t0016968	21	26	GTGGGGGAGAGGCTGTAATTT	miRNA	miR-1275
t0016967	20	26	GTGGGGGAGAGGCTGTATTT	miRNA	miR-1275
t0017048	24	26	AAAGCAGCATTGTACAGGGCTATA	miRNA	miR-107
t0016801	23	26	TACCCTGTAGATCCGAATTTGTG	miRNA	miR-10-5p
t0016840	23	26	AGCAGCATTGTACAGGGCTATGC	miRNA	miR-103a
t0016974	21	26	AGCAGCCTTGTACAGGGCTAT	miRNA	miR-103a
t0017156	24	26	AGCAGCATTGTACAGGGCTATGAG	miRNA	miR-103a
t0017219	22	26	AGCAACATTGTACAGGGCTATG	miRNA	miR-103a
t0017332	21	26	AGCAGCATTGTACAGGCCTAT	miRNA	miR-103a
t0016983	19	26	ACAGTACTGTGATAACTGA	miRNA	miR-101c
t0017152	22	26	TACAGTACTGTGACAACTGAAG	miRNA	miR-101
t0017269	22	26	TGAGGCAGTAGTTTGTGCTGTT	miRNA	let-7i
t0017358	26	26	TGAGGTAGTAGTTTGTACAGTTCGGC	miRNA	let-7g
t0017141	22	26	TCAGGTAGTAGATTGTATAGTA	miRNA	let-7f
t0017341	20	26	AGAGGTAGTAGATTGTATAG	miRNA	let-7f
t0017227	24	26	TGAGGTAGTAGATTGTATAGTTTT	miRNA	let-7f
t0017073	25	26	TGAGGTAGTAGATTGTATAGTTACG	miRNA	let-7f
t0016836	25	26	TGAGGTAGTAGATTGTATAGTTAAA	miRNA	let-7f
t0016862	19	26	TGAGGTAGTAGACTGTATA	miRNA	let-7f
t0016934	24	26	AGAGGTAGTAGGTTGCATAGTTTT	miRNA	let-7d
t0017018	22	26	TGAGGTAGTAGGTTGTGCGGTA	miRNA	let-7b
t0017158	23	26	AATGAGGTAGTAGGTTGTGTGGT	miRNA	let-7b

t0017015	26	26	TGAGGTAGTAGGTTGTATAGTGGAAA	miRNA	let-7-5p
t0016888	22	26	TGAGGTAGTAGGTGGTATAGTT	miRNA	let-7
t0017007	20	26	TGGTAGTAGGTTGTATAGTT	miRNA	let-7
t0017055	25	26	TGAGGTAGTATGTTGTATAGTTATC	miRNA	let-7
t0016847	24	25	GTGGGCCTGACGTGGAGCTGGATC	miRNA	miR-770-3p
t0016996	20	25	TGGACGCGAGCCGGGCCCTT	miRNA	miR-5115
t0017040	23	25	TCTGGGCACAGGCGGATGGACAA	miRNA	miR-5107
t0017066	28	25	TCCTGTACTGAGCTGCCCCGAGACGGGA	miRNA	miR-486-5p
t0017190	28	25	TCCTGTACTGAGCTGCCCCGAGCCGGCA	miRNA	miR-486-5p
t0017369	26	25	CGGGGGGAGAAGGGTCGGGGCGGCAG	miRNA	miR-4516
t0017292	24	25	TGAGGGAGTAGGTTGTGTGGTTAA	miRNA	miR-4510
t0017196	21	25	AAACCTTTACCATTACTTAGT	miRNA	miR-451
t0017290	25	25	AAAACCGTTACCATTACTGAGTTTA	miRNA	miR-451
t0016926	22	25	GCTCCCTCTAGGGTCGCTCGGA	miRNA	miR-4469
t0016805	21	25	TGGCGGCGGTAGTTATGGGCT	miRNA	miR-4467
t0017151	29	25	TTGGTAGAGCTGAAGACTGTAGATCTTTA	miRNA	miR-4451
t0017168	19	25	TGGAGAGAAAGGCAGTAGA	miRNA	miR-4306
t0017874	22	25	TTAGGGGCAGAGAGCGAGACTT	miRNA	miR-423-5p
t0017936	20	25	TGAGGTGCAGAGAGCGAGAC	miRNA	miR-423-5p
t0017849	21	25	TGCGGGGCAGAGAGCGAGACT	miRNA	miR-423-5p
t0017597	21	25	TGAGGGGCAGAGAGCGAGAAG	miRNA	miR-423-5p
t0017813	20	25	ATAATACAACCTGCTAAGTG	miRNA	miR-374c
t0017818	23	25	ATTGCACGGTATCCATCTGTATT	miRNA	miR-363
t0017883	24	25	TTGAAGACTGAAGTGGAGAAGGTT	miRNA	miR-3526
t0017525	27	25	TGAAGACTGAAGTTGAGAAGGGTTTCT	miRNA	miR-3526
t0017785	21	25	GTAGTAGGTTGCATAGTAGAG	miRNA	miR-352
t0017652	21	25	GCCCCTGGGCCTATCCTAGTT	miRNA	miR-331
t0017711	24	25	AAAAGCTGGGTTGAGAGGGCGCTT	miRNA	miR-320d
t0017624	23	25	AAAAGCTGGGTTGAGAGTGCGTA	miRNA	miR-320d
t0017753	22	25	AAAAGCTGGGTTGAGAGGGCAG	miRNA	miR-320d
t0017382	23	25	AAAAGTTGGGTTGAGAGGGCGAT	miRNA	miR-320a
t0017492	22	25	AAAAGCTGGGTTGAGAGAGCGA	miRNA	miR-320a
t0017728	20	25	AAAGTTGGGTTGAGAGGGCG	miRNA	miR-320a
t0017921	22	25	AAGAGCTGGGTTGAGAGGGCGA	miRNA	miR-320a
t0017850	23	25	TATAAAATGGGGGTAGTAAGACA	miRNA	miR-3163
t0017865	22	25	GAGGAGATCGTCGAGGTTGGCA	miRNA	miR-3150b-3p
t0017524	20	25	TTCGCGGGCGAAGGCAAAGT	miRNA	miR-3124-5p
t0017911	20	25	CTGGGAGAGGGTTGTTTACT	miRNA	miR-30c-1*
t0017588	22	25	CATTGCACTTATCTCGGTCTGA	miRNA	miR-25
t0017673	22	25	CAAAGTGCTCATAGTGCAGGTA	miRNA	miR-20b
t0017391	27	25	TAATACTGCCTGGTAATGATGACTATC	miRNA	miR-200b
t0017809	22	25	TGGAATGTAAAGAAGTATGTAT	miRNA	miR-1a
t0017870	21	25	CAACGGAATCCCAAAAGCAGA	miRNA	miR-191
t0017583	23	25	TGGAGAGGAAGGCAGTTCCTGAT	miRNA	miR-185
t0017723	24	25	TGGAGAGAAAGGCAGTTTCTGAAA	miRNA	miR-185
t0017854	21	25	TGGAGAGAAAGGCAGCTCCTG	miRNA	miR-185
t0017893	19	25	TGGAGAGAAAGGCAGTTCA	miRNA	miR-185
t0017529	25	25	TTTGGCAATGGTAGAACTCACAAAA	miRNA	miR-182
t0017578	20	25	AACATTCATTGTTGTCGGTG	miRNA	miR-181d-5p
t0017465	18	25	ACCACTGACCGTTGACTG	miRNA	miR-181a-2*
t0017627	26	25	AACATTCAACGCTGTCGGTGAGTATC	miRNA	miR-181a
t0017463	20	25	TTAGCACGTAAATATTGGTG	miRNA	miR-16c
t0017461	21	25	ACCAATATTACTGTGCTGCTT	miRNA	miR-16*
t0017464	23	25	ATAGCAGCACATCATGGTTTACA	miRNA	miR-15b
t0017683	20	25	TGGCAGCACATCATGGTTTA	miRNA	miR-15b
t0017505	20	25	TAGCAGCACATAATGTTTTG	miRNA	miR-15a
t0017616	18	25	TCAGCACATAATGGTTTG	miRNA	miR-15a
t0017825	19	25	TAGCAGCACATAATGGGTT	miRNA	miR-15a

t0017886	20	25	TAGCGGCACATAATGGTTTG	miRNA	miR-15a
t0017907	23	25	GGATATCATTATATACTGTAAGT	miRNA	miR-144*
t0017796	19	25	GGATCATCATATACTGTAA	miRNA	miR-144*
t0017655	22	25	ACCACAGGGTAGAACCATGGAA	miRNA	miR-140
t0017726	23	25	TACCACAGGGTAGAACCGCGGAC	miRNA	miR-140
t0017765	23	25	TACCACAGGGTAGAACCCCGGCC	miRNA	miR-140
t0017828	24	25	TACCACAGGGTAGAACCACGGATT	miRNA	miR-140
t0017472	23	25	CAGTGCAATGATGAAAGGGCATT	miRNA	miR-130b
t0017858	20	25	GTGAGGACTTGGGAGGTGGA	miRNA	miR-1224
t0017881	23	25	AGCAGCATTGTACAGGGCTATCT	miRNA	miR-107
t0017454	23	25	TACCGCACTGTGGGTACTTGCAT	miRNA	miR-106b*
t0017773	20	25	CCGCATTGTGGGTACTTGCT	miRNA	miR-106b*
t0017821	20	25	TAAAGTGCTGATAGTGCAGA	miRNA	miR-106b
t0017823	22	25	AGCAGCATTGTACAGGACTATG	miRNA	miR-103a
t0017788	26	25	AGCAGCATTGTACAGGGCTATGAAAA	miRNA	miR-103a
t0017847	21	25	CACAGTACTGTGATAACTGAA	miRNA	miR-101c
t0017435	21	25	TACAGTACTATGATAACTGAA	miRNA	miR-101b
t0017483	27	25	TACAGTACTGTGATAACTGAACACGGC	miRNA	miR-101
t0017677	24	25	TGGAATGTAAAGAAGTGTGTATAT	miRNA	miR-1
t0017799	24	25	TGAGGTAGTAGTTTGTGCTGTTTT	miRNA	let-7i
t0017512	22	25	TGAGGTAGTAGTTTGAACAGTT	miRNA	let-7g
t0017641	23	25	TGAGGTAGTAGATTGTATACTTG	miRNA	let-7f
t0017562	23	25	AATGAGGTAGTAGATTGTATAGT	miRNA	let-7f
t0017557	19	25	CGAGGTAGTAGATTGTATA	miRNA	let-7f
t0017684	20	25	TGAGGTTGTAGATTGTATAG	miRNA	let-7f
t0017895	18	25	GAGGTAGTAGATTGTATA	miRNA	let-7f
t0017691	22	25	TGAGGTGTTAGATTGTATAGTT	miRNA	let-7f
t0017692	23	25	TGAGGTGGTAGATTGTATAGTTA	miRNA	let-7f
t0017956	22	25	AGAGGTAGTAAGTTGCATAGTT	miRNA	let-7d
t0017636	21	25	AGAGGTAGTAGGTTGCATGTT	miRNA	let-7d
t0017482	19	25	CTATACAACCTACTGCCTT	miRNA	let-7b*
t0017740	21	25	TGAGGTAGTAGGTTGTGGGTT	miRNA	let-7b
t0017558	23	25	TGAGGTAGTAGGTTGTGTGGTAC	miRNA	let-7b
t0017469	20	25	TGAGGAAGTAGGTTGTGTGG	miRNA	let-7b
t0017386	18	25	TGAGGTAGTAGGTTTGGT	miRNA	let-7a
t0017409	22	25	TGAGGTAGTAGGTTGGATAGTT	miRNA	let-7
t0017445	27	25	TGAGGTAGTAGGTTGTATAGTTGATCC	miRNA	let-7
t0017449	21	24	CACCCGGCTGTGTGCACATGT	miRNA	miR-941
t0017571	19	24	TACCCATTGCATATCGGAG	miRNA	miR-660
t0017747	22	24	ATGCTGACATATTTACTAGAGG	miRNA	miR-628-5p
t0017853	22	24	AGAACAAATGCCGGTTCCCAGA	miRNA	miR-589*
t0017653	21	24	AAAAGTATTTGCGGGTTTTGT	miRNA	miR-548l
t0017866	22	24	CTTCCCACACCCAAGGCTTGCA	miRNA	miR-532-3p
t0017697	21	24	AACAAACATGGTGCACTTCTT	miRNA	miR-495
t0017428	28	24	TCCTGTACTGAGCTGCCCCGAGACCGCA	miRNA	miR-486-5p
t0017586	23	24	TAGATGTAGGATTGTGTCGGTTT	miRNA	miR-4860
t0017763	20	24	AGGACAGGAAAGTGAAGGCG	miRNA	miR-483
t0017503	22	24	AGAGTCGGCGACGCCGCCAGTT	miRNA	miR-4785
t0017473	22	24	TCTCCCTTCCTGCCCTGGCAAA	miRNA	miR-4685-3p
t0017591	23	24	AAAACGTTACCATTACTGAGTTT	miRNA	miR-451
t0018504	19	24	AAACCGTTAACATTACTGA	miRNA	miR-451
t0018047	22	24	AAACCGTTACCATTACTGAATT	miRNA	miR-451
t0018069	27	24	AAAACCGTTACCATTACTGAGTTTATT	miRNA	miR-451
t0018364	23	24	AAACCGTTACCATTACATAGTTT	miRNA	miR-451
t0018102	23	24	AAACCGTTGCCATTACTGAGTTT	miRNA	miR-451
t0018122	18	24	AAACCGTTACCATTACGA	miRNA	miR-451
t0018061	21	24	AAACCGTTACCACCACTGAGT	miRNA	miR-451
t0018536	20	24	TGTGGGAGGAGACAGTGGCA	miRNA	miR-4419a

t0018123	21	24	TGAGGGGCAGAGAGCGAGAAA	miRNA	miR-423-5p
t0018513	19	24	TGAGCGGCAGAGAGCGAGA	miRNA	miR-423-5p
t0018263	23	24	TGAGGGGCAGAGACCGAGACTTA	miRNA	miR-423-5p
t0018516	22	24	TGAGGGGCAGAGAGCGATACTT	miRNA	miR-423-5p
t0018031	23	24	CGAGGGGCAGAGAGCGAGACTTA	miRNA	miR-423-5p
t0018033	19	24	AGCTCGGTCTGAGGCCCCT	miRNA	miR-423-3p
t0018099	23	24	AGGCGGCGTCGGAGGCGGAGGCG	miRNA	miR-3960
t0018108	22	24	ACAGCCCGGATCCCAGCCCACT	miRNA	miR-3940-3p
t0018235	18	24	CAGAACTGAAACGGACAA	miRNA	miR-3896-3p
t0018237	22	24	ACCCGGGGGGCCGGCGGCGGCG	miRNA	miR-3885-5p
t0018377	22	24	CTGGACTTGGAGTTAGAAGGCT	miRNA	miR-378
t0018407	18	24	AATTGCACGGTATCCATC	miRNA	miR-363
t0018062	20	24	CGGGGGTGGGGTCGGCGGGG	miRNA	miR-3621
t0018009	22	24	TTATAAAGCAATGAGACTCATT	miRNA	miR-340-5p
t0018291	22	24	AAAAGTTGGGTTGAGAGGGCAT	miRNA	miR-320d
t0018292	24	24	AAAAGCTGGGTTGAGAGGGCGGTT	miRNA	miR-320d
t0018310	23	24	AAAGCTGGGTTGAGAGGGCGTAG	miRNA	miR-320d
t0018535	22	24	AACAGCTGGGTTGAGAGGGCGA	miRNA	miR-320a
t0018499	22	24	AAAAGCTGGGTTGCGAGGGCGA	miRNA	miR-320a
t0018192	22	24	ACCTTGCGCTACTCAGGTCTGT	miRNA	miR-3200-3p
t0018245	19	24	TATAAAATGAGGGCAGTAA	miRNA	miR-3163
t0018191	20	24	TTCAGTCGGATGTTTACAGA	miRNA	miR-30b-3p
t0018464	19	24	TTCCGGCTCGAAGGACCAA	miRNA	miR-2779
t0018452	22	24	CATCGCACTTGTCTCGGTCTGA	miRNA	miR-25
t0018022	21	24	CTGGGGCGGTACATCTGTTAA	miRNA	miR-219-2-3p
t0018520	21	24	TACTGTAGTATGGGCACTTTT	miRNA	miR-20b*
t0017971	22	24	TTCCTATGTATATACTTCTTTG	miRNA	miR-202-5p
t0018346	25	24	TGGAATGTAAAGAAGTATGTTTATC	miRNA	miR-1a
t0018490	23	24	TGACCTATGAATTGACAGCCAAA	miRNA	miR-192
t0018503	23	24	TAGAGAGAAAGGCAGTTCCTGAA	miRNA	miR-185
t0018205	23	24	TGGAGAGAAAGGCAGCTCCTGAA	miRNA	miR-185
t0018345	20	24	TGAGGGAGGAGATTGTATAG	miRNA	miR-1827
t0018498	24	24	TTTGGCAATGGTAGAACTCACAAA	miRNA	miR-182
t0018396	23	24	ACATTCAACGCTGTCGGTGAGTT	miRNA	miR-181a
t0018325	24	24	ACTGCAGTGAAGGCACTTGTAGAA	miRNA	miR-17*
t0018247	22	24	TAGCAGCACGTATATATTGGCG	miRNA	miR-16
t0018355	19	24	TAGCAGCACGTAAATTTTG	miRNA	miR-16
t0018104	22	24	TAGAAGCACGTAAATATTGGAG	miRNA	miR-16
t0018334	19	24	TAGCGGCACATCATGGTTT	miRNA	miR-15b
t0018125	20	24	TAGCAGCCCATCATGGTTTA	miRNA	miR-15b
t0018230	25	24	TAGCAGCACATCATGGTTTACAAAA	miRNA	miR-15b
t0018330	20	24	TAGCAGCTCATCATGGTTTA	miRNA	miR-15b
t0017994	21	24	AGCAGCACATAATGGTTTGTG	miRNA	miR-15a
t0018100	22	24	TAGCAGCACACAATGGTTTGTG	miRNA	miR-15a
t0018441	21	24	GGATATCGTCATATACTGTAA	miRNA	miR-144*
t0018253	23	24	TACAGTATAGATGATGTACATAA	miRNA	miR-144
t0018135	21	24	TACAGTATAGATGATGTAAAA	miRNA	miR-144
t0018051	18	24	TCCATAAAGTAGAAAGCA	miRNA	miR-142-5p
t0018000	23	24	ACCACAGGGTAGAAACACGGACA	miRNA	miR-140
t0018223	22	24	TACCACAGGGTAGAACAAAGGA	miRNA	miR-140
t0018326	21	24	TACCACAGGGTAGAACTACGG	miRNA	miR-140
t0018010	21	24	TACCACAGGGTAGAACCACTG	miRNA	miR-140
t0018132	19	24	TCTGGGCAACAAAGTGAGA	miRNA	miR-1285
t0018351	21	24	CTGGAGATATGGAAGAGCTGT	miRNA	miR-1270
t0018398	20	24	AGGATGAGCAAAGAAAGTAG	miRNA	miR-1255a
t0018013	21	24	TACCGCACTGTGGGTACTTGT	miRNA	miR-106b*
t0018137	26	24	AGCAGCATTGTACAGGGCTATGACGG	miRNA	miR-103a
t0018185	20	24	AGCAGCATTATACAGGGCTA	miRNA	miR-103a

t0018111	25	24	TACAGTACTGTGATAACTGAAGTCC	miRNA	miR-101
t0018218	22	24	ATACAGTACTGTGATAACTGAA	miRNA	miR-101
t0018211	18	24	TGAGGTAGTAGATTGTTG	miRNA	let-7j
t0018011	21	24	TGAGGTAGTAGTTTGTGCGTT	miRNA	let-7i
t0018130	24	24	TGAGGTAGTAGTTTGTGTTGTCGG	miRNA	let-7i
t0018296	20	24	CTGTAGTAGATTGTATAGTT	miRNA	let-7f
t0018395	25	24	TGAGGTAGTAGATTGTATAGTCGTC	miRNA	let-7f
t0018264	19	24	TGTGGTAGATTGTATAGTT	miRNA	let-7f
t0018362	22	24	TGAGGTAGTAGATTGTGCAGTT	miRNA	let-7f
t0018006	19	24	TGAGGTAGGAGATTGTATA	miRNA	let-7e
t0018465	20	24	AGAGGTAGTAGTTTGCATAG	miRNA	let-7d
t0017975	19	24	TGAGGTAGTAGGTTGTATG	miRNA	let-7c
t0018369	25	24	TCAGGTAGTAGGTTGTGTGGTTATC	miRNA	let-7b
t0018117	20	24	TGAGGTAGTAGGTGTGGTTA	miRNA	let-7a
t0018340	20	24	TGAGGTAGTAGGTTCTATAG	miRNA	let-7
t0018319	20	24	TGAGGTAGTGGGTTGTATAG	miRNA	let-7
t0017976	19	23	CACCCGTAGAACCGACCTT	miRNA	miR-99b
t0018019	18	23	AGGTGGAAGGTTGTAGGC	miRNA	miR-965-5p
t0018012	20	23	CATCCGGCTGTGTGCACATT	miRNA	miR-941
t0018252	21	23	GGGAGACTGTGAAACGGTATC	miRNA	miR-920
t0018324	26	23	AGGGCGCGCGGGTCGGGGCGGCGGTT	miRNA	miR-638
t0018489	21	23	TAGTACCAGTACCTTGTGTTC	miRNA	miR-624*
t0018056	18	23	CCTCACACCCAAGGCTTG	miRNA	miR-532-3p
t0018200	26	23	TTCTGTACTGAGCTGCCCCGAGAAGA	miRNA	miR-486-5p
t0018460	21	23	TCCTGTACTGACCTGCCCCGA	miRNA	miR-486-5p
t0018119	23	23	TCCTTTACTGAGCTGCCCCGAGA	miRNA	miR-486-5p
t0018109	26	23	TCCTGTACTGAGCTGCCCCGACGGCC	miRNA	miR-486-5p
t0018261	21	23	TCCTGTACTGAGCTGCCCCGG	miRNA	miR-486-5p
t0018456	20	23	CGGGGCAGCTTAGTACAGGA	miRNA	miR-486-3p
t0018586	22	23	TCTCCCTTTCTGCCCTGGCTAT	miRNA	miR-4685-3p
t0018773	25	23	GCTGGGTCGGTCGGGCTGGGGCGTT	miRNA	miR-4651
t0018720	23	23	GGGCTGGGTCGGTCGGGCTGGGG	miRNA	miR-4651
t0018678	22	23	CGTTACCATTACTGAGTTAAAC	miRNA	miR-451
t0018572	21	23	CGAATCCGAGTCACGGCACCA	miRNA	miR-4454
t0018637	23	23	CAAAACGTGAGGCGCTGCTATAT	miRNA	miR-424*
t0018608	22	23	TGAGGGGCAAAGAGCGAGACTT	miRNA	miR-423-5p
t0018693	25	23	TGAGGGGCAGAGAGCGAGACTTCGT	miRNA	miR-423-5p
t0018767	23	23	TGAGGGGCAGAGAGCGGGACTTA	miRNA	miR-423-5p
t0018803	20	23	AAGCTCGGTCTGAGGCCCCT	miRNA	miR-423-3p
t0018949	23	23	CAGAACTGAAACGGACAAGGGGA	miRNA	miR-3896-3p
t0019033	28	23	AACTTTGAAGACTGAAGGGGAGAAGGGT	miRNA	miR-3526
t0018760	20	23	ATAAAGCAATGAGACTGATT	miRNA	miR-340-5p
t0018587	19	23	TCTCTGGGCCTGTGTCTTA	miRNA	miR-330
t0018651	23	23	AAAAGCTGGGTTGAGAGGGGGTT	miRNA	miR-320d
t0018699	23	23	AAAAGCTGGGGTGAGAGGGCGAT	miRNA	miR-320a
t0018828	22	23	CTCCTGCGTAGGATCTGAGGAA	miRNA	miR-3193
t0018933	20	23	GTGGAAGGTAGACGGCCAGA	miRNA	miR-3190
t0018704	22	23	GGCGGATGTAGCCAAGTGGAAA	miRNA	miR-270
t0018591	24	23	TTCAAGTAATTCAGGATAGGTTAG	miRNA	miR-26b
t0018629	22	23	CATTGCACTTGCCTCGGTCTGA	miRNA	miR-25
t0018869	22	23	CATTGCACTCGTCTCGGTCTGA	miRNA	miR-25
t0018812	22	23	CATTGCACTTTTCTCGGTCTGA	miRNA	miR-25
t0019013	25	23	TGACTGGGGCGGTACATCTGTTAAT	miRNA	miR-219-2-3p
t0018872	25	23	TGGAATGTAAGGAAGTGTGTACATC	miRNA	miR-206
t0018944	22	23	TAGGTAGTTCCCTGTTGTTGGA	miRNA	miR-196b
t0018876	21	23	TGACCTATGAATTGACAGCCC	miRNA	miR-192
t0018573	24	23	CAACGAAATCCCAAAAGCAGCTGA	miRNA	miR-191
t0019019	23	23	CAACGGAATCCCAAAAGCAGCCG	miRNA	miR-191

t0018947	22	23	CAGGGGCTGGCTTTCCTCTGGT	miRNA	miR-185*
t0019006	23	23	TGGAGAGGAAGGCAGTTCCTGAA	miRNA	miR-185
t0019038	23	23	TGGAGAGAAAAGCAGTTCCTGAA	miRNA	miR-185
t0018844	22	23	GGAGAGAAAGGCAGTTCCTGAT	miRNA	miR-185
t0018585	23	23	AACATTCAATGCTGTCGGTGAGT	miRNA	miR-181a
t0018963	23	23	TAGCAGCACGTAAATATTGGCAT	miRNA	miR-16b
t0018966	27	23	TAGCAGCACGTAAATATTGGCAATAAC	miRNA	miR-16b
t0018976	22	23	GGGGGCGGGGAGCGGTCGGGCG	miRNA	miR-1607
t0019070	21	23	TAGCAGCACGTAAATATTAGC	miRNA	miR-16
t0018668	20	23	TAGCAGCACATCATGGCTTA	miRNA	miR-15b
t0018871	21	23	ACTAGCAGCACATCATGGTTT	miRNA	miR-15b
t0018918	18	23	TAGCAGCACATAATGTTG	miRNA	miR-15a
t0018954	19	23	TAGCAGCACATAATGGTGT	miRNA	miR-15a
t0018969	20	23	TAGCAGCACGTAATGGTTTG	miRNA	miR-15a
t0018558	21	23	TAGCAGCACATAATGTTTTGT	miRNA	miR-15a
t0018757	22	23	CGGGCACGGGCTGGGCGAGGCT	miRNA	miR-1587
t0019126	19	23	ATACAGTATAGATGATGTA	miRNA	miR-144
t0019093	22	23	ACCACAGGGTAGAACCATGGAC	miRNA	miR-140
t0018570	24	23	TACCACAGGGTAGAACCACGGGCA	miRNA	miR-140
t0018996	22	23	ACCACATGGTAGAACCACGGAT	miRNA	miR-140
t0019116	23	23	CACCACAGGGTAGAACCACGGAA	miRNA	miR-140
t0018798	22	23	ACCACAGGGAAGAACCACGGAT	miRNA	miR-140
t0018731	23	23	TACCACAGGGTAGAACCAAGGAT	miRNA	miR-140
t0018733	22	23	ACCACAGGGTAGAACCACAGAC	miRNA	miR-140
t0018644	22	23	TACCAAAGGGTAGAACCACGGA	miRNA	miR-140
t0018695	23	23	ACCACAGGGTAGAACCAAGGACA	miRNA	miR-140
t0018817	24	23	TACAACAGGGTAGAACCACGGACA	miRNA	miR-140
t0019099	24	23	CACCACAGGGTAGAACCACGGACA	miRNA	miR-140
t0018815	23	23	CAGTGCAATGATGAAAGGGCAAA	miRNA	miR-130b
t0018771	22	23	CTCGGCGTGGCGTCGGTCGTGA	miRNA	miR-1307
t0018680	23	23	TGAGAGGCAGAGGTTGCAGTGGA	miRNA	miR-1273f
t0018689	22	23	AGCAGCATTCTACAGGGCTATG	miRNA	miR-103a
t0018700	21	23	AGCAGCATTGCACAGGGCTAT	miRNA	miR-103a
t0018772	22	23	AGCAGCATCGTACAGGGCTATG	miRNA	miR-103a
t0018774	23	23	AGCAGCCTTGTACAGGGCTATGA	miRNA	miR-103a
t0018790	18	23	GTTACTGTGATAACTGAA	miRNA	miR-101c
t0018921	22	23	GTACAGTACTGTGATAAATGAA	miRNA	miR-101
t0018960	29	23	TACAGTACTGTGATAACTGAACTCTGAAC	miRNA	miR-101
t0018980	21	23	TACAGTACTGTGATAACCGAA	miRNA	miR-101
t0019035	21	23	TGAGGTAGTTGTTTGTGCTGT	miRNA	let-7i
t0019065	21	23	TGAGGTAGTAGTTTGTGCAGT	miRNA	let-7g
t0018852	22	23	TGAGGTAGTAGATTGTATGCTT	miRNA	let-7f-5p
t0018686	23	23	ATGAGGTAGTAGATTGTATAGTG	miRNA	let-7f
t0019081	20	23	AGAGGTGGTAGGTTGCATAG	miRNA	let-7d
t0018811	26	23	AAGAGGTAGTAGGTTGCATAGTAATT	miRNA	let-7d
t0018897	21	23	GGAGGTAGTAGGTTGTGTGGT	miRNA	let-7b
t0018995	20	23	TGAGGTAGTAGGTTGTGGGT	miRNA	let-7b
t0019118	22	23	TCTGGTAGTAGGTTGTGTGGTT	miRNA	let-7b
t0018694	19	23	TGAGGTAGTAGGTGTGTTT	miRNA	let-7a
t0018685	20	23	TTAGGTAGTAGGTTGTATAG	miRNA	let-7
t0018800	21	23	TGAGGTAGTAGGTTTTATAGT	miRNA	let-7
t0018920	22	22	CAAGGAAATAGTCAGACGGGAT	miRNA	miR-994-5p
t0018965	22	22	CAAATTGCTGTTCGTGCAGGTA	miRNA	miR-93
t0018749	21	22	TATTGCACTTGTCTCGGCCTT	miRNA	miR-92b
t0018781	23	22	TATTGCACTTGTCCCGGCTTGTG	miRNA	miR-92a
t0018984	25	22	TAATACTGTCAGGTAAAGATGTATC	miRNA	miR-8-3p
t0018566	19	22	GACTATAGAACTTTCCCCC	miRNA	miR-625*
t0018632	21	22	TTATGGTTTGCTTGGGACTGA	miRNA	miR-584

t0018643	19	22	AAGATGTGGAAAAATTGGA	miRNA	miR-576-3p
t0019103	23	22	TGGGCACAGGCGGATGGACAGTA	miRNA	miR-5107
t0019107	20	22	TAGCAGCGGGTACAGTTCTG	miRNA	miR-503
t0019040	21	22	TAGCAGCGGGAACAGTTCTAA	miRNA	miR-503
t0018579	20	22	TCCTGTACTGAGCTGCCCCC	miRNA	miR-486-5p
t0018937	24	22	TCGTACTGAGCTGCCCCGAGTAAA	miRNA	miR-486-5p
t0019290	25	22	TCCTGTACTGAGCTGCCCCGAGTAA	miRNA	miR-486-5p
t0019641	22	22	TACTGTACTGAGCTGCCCCGAG	miRNA	miR-486-5p
t0019611	23	22	TCCTGTACTGAGCTGCCCCTAGA	miRNA	miR-486-5p
t0019266	21	22	ACCTGTACTGAGCTGCCCCGA	miRNA	miR-486-5p
t0019480	29	22	TCCTGTACTGAGCTGCCCCGAGTCCGGCA	miRNA	miR-486-5p
t0019538	23	22	ATACACACACACACATACACAGA	miRNA	miR-466b-2*
t0019640	25	22	GCTGGGTCGGTCGGGCTGGGGCGCG	miRNA	miR-4651
t0019690	21	22	TGAGGGAGTAGGGTGTGTGGT	miRNA	miR-4510
t0019763	22	22	AAACCGGTACCATTACTGAGTT	miRNA	miR-451
t0019251	20	22	AAACCGTTACCATTACTGAC	miRNA	miR-451
t0019728	22	22	AAACCGTTACCATTACTGAGTC	miRNA	miR-451
t0019218	23	22	AAACCGCTACCATTACTGAGTTT	miRNA	miR-451
t0019394	21	22	AAACCGTTACCATTACGGAGT	miRNA	miR-451
t0019402	21	22	AACCGTTACCATTACTGAGTA	miRNA	miR-451
t0019444	23	22	AAACCGTTACCATTACAGAGTTT	miRNA	miR-451
t0019559	25	22	AAACCGTTACCATTACTGATTTTAG	miRNA	miR-451
t0019565	20	22	TCCGGGACGGCTGGGAAGGC	miRNA	miR-4497
t0019706	18	22	TTTGGAGGAGATGCGGTT	miRNA	miR-4443
t0019405	23	22	TCAGGGGCAGAGATCGAGACTTT	miRNA	miR-423-5p
t0019528	24	22	TGAGGGGCAGAGAGCGAGACTATA	miRNA	miR-423-5p
t0019441	24	22	TGAGGGGCAGAGAGCGAGACTTAC	miRNA	miR-423-5p
t0019313	23	22	TCAGGGGCAGAGAGCGAGACTTT	miRNA	miR-423-5p
t0019372	20	22	TGAGGGGCAGAGAGCGTGAC	miRNA	miR-423-5p
t0019459	21	22	AGCTCGGTCTGAGGCCCTTCA	miRNA	miR-423-3p
t0019595	24	22	ATGGACTCGCAGGAACAGGCTCTT	miRNA	miR-4001b-5p
t0019629	25	22	ACTGTACTTGGAGTCAGAAGGCATC	miRNA	miR-378
t0019720	21	22	ACTGGACTTGGAGTCAGAAAG	miRNA	miR-378
t0019738	19	22	ATTGCACGGTATCCATCTG	miRNA	miR-363
t0019748	28	22	TGAAGACTGAAGTGGAGAAGGGTTTCTT	miRNA	miR-3526
t0019425	30	22	TGAAGACTGAAGTGGAGAAGGGTTTCTTC	miRNA	miR-3526
t0019461	18	22	TGAAGACTGAAGTGGAGA	miRNA	miR-3526
t0019173	22	22	CCCTCGAACTGTTGTGGCCATT	miRNA	miR-3487
t0019181	21	22	TGAGCGCCTCGACGACAGAGA	miRNA	miR-339-3p
t0019225	23	22	AAAAGCTGGGGTGAGAGGGCGTT	miRNA	miR-320d
t0019285	23	22	AAAAGCTGGGTTGAGAGGGCGCG	miRNA	miR-320d
t0019664	23	22	AATCTGAGAAGGCGCACAAGGTA	miRNA	miR-3200-5p
t0019624	20	22	TCTGGGAGGTTGTAGCAGTG	miRNA	miR-3192
t0019257	22	22	GTGGAAGGTAGACGGCCAGAGT	miRNA	miR-3190
t0019452	21	22	TGGAAGGTAGACGGCCAGACG	miRNA	miR-3190
t0019754	22	22	AGCTTTTGGGAATTCAGGTAGA	miRNA	miR-3140-3p
t0019638	25	22	CTTTCAGTCGGATGTTTACAGCATC	miRNA	miR-30e*
t0019303	24	22	CGCGCGGGTCGGGGGGCGGGGCGG	miRNA	miR-2981
t0019561	21	22	TTCAAGTAATTCAGGACAGGT	miRNA	miR-26b
t0019770	21	22	TTGGGGAAACGGCCGCTGAGA	miRNA	miR-2110
t0019470	23	22	TGTGCAAATCCATGCAAAACTAA	miRNA	miR-19b
t0019195	22	22	TGGGTCTTTGCGGGTGAGATGA	miRNA	miR-193a-5p
t0019370	21	22	AACGGAATCCCAAAAGCAGCC	miRNA	miR-191
t0019630	20	22	CGGCGGGGACGGCGATTGGT	miRNA	miR-1908
t0019230	19	22	AGAGAAAGGCAGTTCCTGA	miRNA	miR-185
t0019244	23	22	TGGAGAGAAAGGCAGTTCCTGTG	miRNA	miR-185
t0019601	21	22	TGGAGAGAAAGCCAGTTCCTG	miRNA	miR-185
t0019634	22	22	TGGAGAGAAAGGCAGTTCCAGA	miRNA	miR-185

t0019133	22	22	TGGACGGAGAACTGATAGGGGC	miRNA	miR-184-3p
t0019454	22	22	TGGACGGAGAACTGATAAGGGT	miRNA	miR-184
t0019340	28	22	TATGGCACTTGTAGAATTCACTAAACAG	miRNA	miR-183
t0019645	21	22	GAGGGAGTAGATTGTATAGTT	miRNA	miR-1827
t0019543	20	22	AACATTCAACGTTGTCGGTG	miRNA	miR-181a
t0019349	24	22	TAGCAGCACGTAAATATTGGCTCC	miRNA	miR-16b
t0019585	24	22	GAAGCGGCGATGGCGGAGCTGAAA	miRNA	miR-1636
t0019137	18	22	AGCACGTAAATATTGGCG	miRNA	miR-16
t0019364	24	22	TAGCAGCACGTAAATATTGGCGGA	miRNA	miR-16
t0019317	20	22	TAGCAGCACATCACGGTTTA	miRNA	miR-15b
t0019487	24	22	AAACTAGCAGCACATCATGGTTTA	miRNA	miR-15b
t0019594	20	22	AGCAGCACATAATGGTTTAA	miRNA	miR-15b
t0019599	23	22	TAGCAGCACATAATGGTTTGTAA	miRNA	miR-15a
t0019328	20	22	CCCATAAAGTAGAAAGCATC	miRNA	miR-142
t0019730	22	22	TACCCCAGGGTAGAACCACGGA	miRNA	miR-140
t0019134	22	22	TACGACAGGGTAGAACCACGGA	miRNA	miR-140
t0019642	21	22	CCACAGGGTAGAACTACGGAT	miRNA	miR-140
t0019544	23	22	TACCACAGGGTAGAAACACGGAT	miRNA	miR-140
t0019140	23	22	TACCACAGGGTAGAACCACAGAA	miRNA	miR-140
t0019672	22	22	ACCACAGGGTAGAACCACGGGC	miRNA	miR-140
t0019521	21	22	TACCACAGGGGAGAACCACGG	miRNA	miR-140
t0019737	22	22	ATCACAGGGTAGAACCACGGAT	miRNA	miR-140
t0019265	24	22	ACCACAGGGTAGAACCACGGAGGA	miRNA	miR-140
t0019583	23	22	TACCACAGGATAGAACCACGGAC	miRNA	miR-140
t0019675	19	22	CTCGGCGTGGCTTCGGTCG	miRNA	miR-1307
t0019526	18	22	ATTTTAGAGACGGGGTTG	miRNA	miR-1303
t0019315	23	22	TAGGGCCCTGGCTCCATCTCCTT	miRNA	miR-1296
t0019152	25	22	TCTGGGCAACAAAGTGAGACCTGAT	miRNA	miR-1285
t0019169	21	22	ATCCCACCGCTGCCACCAAAA	miRNA	miR-1260b
t0019184	19	22	TACCCTGTAGATCCGAATT	miRNA	miR-10a
t0019286	20	22	GCGCACTGTGGGTACTTGCT	miRNA	miR-106b*
t0019448	25	22	TACCGCACTGTGGGTACTTGCTAAA	miRNA	miR-106b*
t0019467	23	22	AGCAGCATTGTACAGGGCTCTGA	miRNA	miR-103a
t0019657	23	22	GGCAGCATTGTACAGGGCTATGA	miRNA	miR-103a
t0019659	21	22	AGCAGCATCGTACACGGCTAT	miRNA	miR-103a
t0019685	21	22	AGCAGCATTGAACAGGGCTAT	miRNA	miR-103a
t0019729	22	22	AGCAGCATTGTACAGGGCTGTG	miRNA	miR-103a
t0019325	23	22	AGCAGCGTTGTACAGGGCTATGA	miRNA	miR-103a
t0019649	21	22	GTACAGTACTGTGATAACTAA	miRNA	miR-101
t0019643	27	22	GTACAGTACTGTGATAACTGAACCGGC	miRNA	miR-101
t0019304	22	22	TGAGGTAATAGTTTGTACAGTT	miRNA	let-7g
t0019164	21	22	TGAGGTAGTAGTTTCTACAGT	miRNA	let-7g
t0019488	23	22	TGAGGTAGTAGATTGTATAGAAA	miRNA	let-7f-5p
t0019236	20	22	GTGAGGTAGTAGATTGTATA	miRNA	let-7f
t0019490	21	22	GTAGTAGATTGTATAGTTGAG	miRNA	let-7f
t0019254	24	22	TGAGGTAGTAGATTGTATAGTTGC	miRNA	let-7f
t0019421	24	22	TTAGAGGTAGTAGGTTGCATAGTT	miRNA	let-7d
t0019471	20	22	TGAGGTAGTAGCTTGTGTGG	miRNA	let-7b
t0019586	20	22	TGAGGCAGTAGGTTGTGTGG	miRNA	let-7b
t0019761	20	22	TGATGTAGTAGGTTGTGTGG	miRNA	let-7b
t0019774	22	22	TGAGGCAGTAGGTTGTGTGGTA	miRNA	let-7b
t0019283	19	22	TGAGGTAGTAGATTTGTTA	miRNA	let-7a
t0019435	24	22	TGAGGTAGTAGGTTGTATAGAAAA	miRNA	let-7-5p
t0019398	20	22	AGGTAGTAGGTTGTATAGTT	miRNA	let-7
t0019557	21	22	TGAAGTAGTAGGTTGTATAGT	miRNA	let-7
t0019183	24	22	TGAGGTAGTAGGTTGTATAGTTAT	miRNA	let-7
t0019326	18	22	TGAGGTAGTAGGTTGTAA	miRNA	let-7
t0019529	20	22	TGAGGTAGTAGGATGTATAG	miRNA	let-7

t0019627	19	21	CAGGTGGAAGGTTGTAGGC	miRNA	miR-965-5p
t0019577	25	21	GGTGGAAGGTTGTAGGCATGTATCT	miRNA	miR-965-5p
t0019155	25	21	CACCCGGCTGTGTGCACATGTGAAA	miRNA	miR-941
t0019261	22	21	AGGTTGGGATCGGTTGCAATGT	miRNA	miR-92a-1*
t0019546	29	21	GAAGACTGAAGTGGAGAAGGGTTTCTTGT	miRNA	miR-739
t0019701	22	21	AAAGTAATTGCGGTTTTTGACT	miRNA	miR-548w
t0019747	23	21	GCAAAAGTAATTGCGGTCTTTGG	miRNA	miR-548j
t0019418	24	21	GGGGCCCGGGGGGCGGGGCGGATC	miRNA	miR-5126
t0019174	26	21	CTGGTGCGGACCAGGGGAATCCGACT	miRNA	miR-5109
t0019302	23	21	TCCTGTACTAAGCTGCCCCGAGA	miRNA	miR-486-5p
t0019363	23	21	TCCTGTACTGAGCTGCCCCGGGA	miRNA	miR-486-5p
t0019702	22	21	TCCTGTACTGAGCTGGCCCGAG	miRNA	miR-486-5p
t0019716	23	21	CGGGGCAGCTCAGTACAGGATAT	miRNA	miR-486-3p
t0020078	21	21	TCGGGCGGAGGTGGTTGAGTG	miRNA	miR-4750
t0020352	23	21	TGTAGAGCAGGGAGCAGGAAGAT	miRNA	miR-4732-5p
t0020150	23	21	TGTAGAGCAGGGCGCAGGAAGCT	miRNA	miR-4732-5p
t0020233	21	21	GAAGGGTCGGGGCGGCAGTTT	miRNA	miR-4516
t0020294	23	21	GAGAGAAGGGTCGGGGCGGCAGG	miRNA	miR-4516
t0020363	22	21	TGAGGGAGTAGGGTGTGTGGTT	miRNA	miR-4510
t0020060	19	21	AACCGTTACCATTACTGAG	miRNA	miR-451
t0020189	21	21	AGACCGTTACCATTACTGAGT	miRNA	miR-451
t0020360	21	21	AAACCGTTACCATTATTCAGT	miRNA	miR-451
t0019831	23	21	AAACCGATACCATTACTGAGTTT	miRNA	miR-451
t0019868	21	21	AAACCGTTATCATTACTGATT	miRNA	miR-451
t0019943	26	21	AGCGAAACCGTTATCATTACTGAGTT	miRNA	miR-451
t0020376	30	21	AAACCGTTACCATTACTGAGTTCGTCGGG	miRNA	miR-451
t0019936	20	21	AAACCGCTACCATTACTGAG	miRNA	miR-451
t0020184	19	21	GGGTGCGGGCCGGCGGGGT	miRNA	miR-4466
t0020408	23	21	TCGAATCCGAGTCACGGCACCAA	miRNA	miR-4454
t0020312	21	21	TGAGGGGCAGTGAGCGAGACT	miRNA	miR-423-5p
t0020318	21	21	TGAGGGGCAGATAGCGAGACA	miRNA	miR-423-5p
t0020115	23	21	TGAGGGGCAGAGGGCGAGACTTA	miRNA	miR-423-5p
t0019856	23	21	TGAGGGGCAGAGAGAGAGAATTT	miRNA	miR-423-5p
t0020018	21	21	TGAGGGGCAGAGAGAGAGAAT	miRNA	miR-423-5p
t0020052	22	21	TGAGGGGCACAGAGCGAGACTT	miRNA	miR-423-5p
t0020146	22	21	TAAGGTAGTAGTTTGTACAGTT	miRNA	miR-3962
t0020249	21	21	TGAGTAGTAGTTTGTACAGTT	miRNA	miR-3962
t0020354	22	21	TAAGGGGTGTATGGCAGATGCG	miRNA	miR-3936
t0020369	21	21	CGGCGAGTCGGGTTGTTTGGG	miRNA	miR-3787
t0020372	22	21	TCAAAATCGTTGGCAATGGCTT	miRNA	miR-3495
t0020244	21	21	TTATAAAGCAATGAGACTATT	miRNA	miR-340-5p
t0020342	25	21	AAAAGCTGGGTTGAGAGGGCGTTAA	miRNA	miR-320d
t0019792	23	21	AAAAGCTGGGTTGAGGGGGCGAA	miRNA	miR-320a
t0020134	22	21	AAAAGCTGGATTGAGAGGGCGA	miRNA	miR-320a
t0020216	21	21	GAAGGGAGAAGAGCTTTAATG	miRNA	miR-3202
t0020222	21	21	TCTGGGAGGTTGTAGCAGTGA	miRNA	miR-3192
t0020410	20	21	AGAGGCTTTGTGCGGATACA	miRNA	miR-3188
t0020417	22	21	TGCACGGCACTGGGGACACGTT	miRNA	miR-3177-3p
t0019793	22	21	TGGGACAAACGGACGGTTTTTA	miRNA	miR-3122
t0020179	19	21	AGCTTTCAGTCGGATGTTT	miRNA	miR-30e*
t0020088	21	21	GCCTGTAAACTGGTATGCTCT	miRNA	miR-3048
t0019805	23	21	AGTGCAATGATATTGTCAAAGCA	miRNA	miR-301b
t0020171	21	21	ACTGATTTCTTTTGGTGTTCA	miRNA	miR-29a*
t0019898	22	21	TAGCACCATTTGAAATCGGTTG	miRNA	miR-29a
t0020137	18	21	TCCGGCTCGAAGGACCAT	miRNA	miR-2779
t0019808	19	21	TCAGTCTTGTCGAATGGTG	miRNA	miR-2766
t0020412	19	21	TTCAAGTAATTCAGGATAG	miRNA	miR-26b
t0020261	18	21	GCACTTGTCTCGGTCTGA	miRNA	miR-25

t0020208	22	21	CATTGCACTTGTCCCGGTCTGA	miRNA	miR-25
t0019924	22	21	CATTGCACTTGTATCGGTCTGA	miRNA	miR-25
t0020127	20	21	TGGCTCAGTTTAGCAGGAAC	miRNA	miR-24
t0020196	22	21	TGGGTTCCTGGCATGCTGATTT	miRNA	miR-23b*
t0019932	18	21	AATCACATTGCCAGGGAT	miRNA	miR-23a
t0020199	20	21	GACTGTGGCGGTACATCTGT	miRNA	miR-219-2-3p
t0020398	18	21	AGAGGTAGGTTGCATAGT	miRNA	miR-1961
t0020158	22	21	CAACGGAATCCCAAAAGCCGCT	miRNA	miR-191
t0020328	21	21	AACGGAATCTCAAAAGCAGCT	miRNA	miR-191
t0020405	21	21	AGGAGAGAAAGGCAGTTCCTG	miRNA	miR-185
t0020200	24	21	TGGAGAGAAAGGCAGTTCCTGAGG	miRNA	miR-185
t0020250	21	21	TGGAGAGAACGGCAGTTCCTG	miRNA	miR-185
t0019951	20	21	TGGAGAGAAAGGCAGTTCAT	miRNA	miR-185
t0020155	23	21	TGGAGAGAAAGACAGTTCCTGAA	miRNA	miR-185
t0020217	21	21	TGAGGGAGGAGATTGTATAGT	miRNA	miR-1827
t0020392	23	21	CAAAGTGCTTACAGTGCAGGTAA	miRNA	miR-17a
t0020231	19	21	CAAAGTGCTTACAGTGCAG	miRNA	miR-17
t0019976	19	21	TAGCAGCTAAATATTGGCG	miRNA	miR-16c
t0020260	21	21	TGGGGGCGGGGAGCGGTCGGG	miRNA	miR-1607
t0020257	27	21	GGGGCGGGGAGCGGTCGGGCGGCGGCG	miRNA	miR-1607
t0020081	26	21	CTGGGGGCGGGGAGCGGTCGGGCGGC	miRNA	miR-1607
t0020112	19	21	TGGGGGCGGGGAGCGGTCG	miRNA	miR-1607
t0019790	22	21	TAGCAGCATGTATATATTGGCG	miRNA	miR-16
t0019799	24	21	GTTAGCAGCACGTAAATATTGGCG	miRNA	miR-16
t0019893	19	21	TAGAAGCACATCATGGTTT	miRNA	miR-15b
t0020027	19	21	TAGCAGCTCATCATGGTTT	miRNA	miR-15b
t0020415	20	21	TAGCAGCACATCATAGTTTA	miRNA	miR-15b
t0020339	20	21	TAGCAGCACATCATGGGCTA	miRNA	miR-15b
t0019794	21	21	TAGCAGCATATAATGGTTTGT	miRNA	miR-15a
t0019894	20	21	CAGCAGCACATAATGGTTTG	miRNA	miR-15a
t0020283	21	21	CTACAGTATAGATGATGTACT	miRNA	miR-144
t0019866	18	21	TACAGTATAGATGATGTC	miRNA	miR-144
t0020175	20	21	CTCATAAAGTAGAAAGCATT	miRNA	miR-142-5p
t0020272	23	21	ACCACAGGGTAGAACCACGGCCT	miRNA	miR-140
t0020335	23	21	TAACACAGGGTAGAACCACGGAA	miRNA	miR-140
t0019885	22	21	TACCACAGGGTAGAACCAGGGA	miRNA	miR-140
t0020203	22	21	ACAACAGGGTAGAACCACGGAC	miRNA	miR-140
t0020020	20	21	ACCACAGGGGAGAACCACGG	miRNA	miR-140
t0020119	23	21	TACCACGGGGTAGAACCACGGAA	miRNA	miR-140
t0020338	21	21	CTCGGCGTGGCGTCGGTCGTT	miRNA	miR-1307
t0020344	25	21	TCGTACCGTGAGTAATAATGCAATC	miRNA	miR-126
t0020104	23	21	AAGCAGCATTGTACAGGGCTATT	miRNA	miR-107
t0020288	23	21	AGCAGCATTGTACAGGGCTATTG	miRNA	miR-107
t0019997	24	21	CCGCACTGTGGGTACTTGCTGCGA	miRNA	miR-106b*
t0020253	21	21	TAAAGTGCTGACAGTGCCGAT	miRNA	miR-106b
t0020425	21	21	AGCAGCGTTGTACAGGGCTAT	miRNA	miR-103a
t0019785	22	21	AGCAGCCTTGTACAGGGCTATG	miRNA	miR-103a
t0019845	20	21	AGCAGAATTGTACAGGGCTA	miRNA	miR-103a
t0019873	23	21	AACAGCATTGTACAGGGCTATGA	miRNA	miR-103a
t0019931	20	21	AGCAGCATTGTACAGGGCTC	miRNA	miR-103a
t0020242	21	21	AGCAGCATTGTACGGGGCTAT	miRNA	miR-103a
t0020302	22	21	TACAGTACTGTGATAACTCAAG	miRNA	miR-101
t0019991	25	21	ATACCCTGTAGATACGAATTTGATC	miRNA	miR-10
t0020157	22	21	TGAGGTAGTAGTTTGTACAGAT	miRNA	let-7g
t0020170	21	21	TGAGGCAGTAGTTTGTACAGT	miRNA	let-7g
t0020418	21	21	TGAGGTAGTGTATTGTATAGT	miRNA	let-7f-5p
t0019815	20	21	TGAGGTAGTAGATTGTAATT	miRNA	let-7f-5p
t0020205	23	21	CGAGGTAGTAGATTGTATAGTTA	miRNA	let-7f

t0019826	19	21	TGAGGTAGTAGATTGTACA	miRNA	let-7f
t0019840	23	21	TGAGGTAGTAGATTGTATAGGTT	miRNA	let-7f
t0019941	19	21	TGAGGTAGTAGAGTGTATA	miRNA	let-7f
t0020279	21	21	TGAGGTAGTAGATTGTACATT	miRNA	let-7f
t0020357	19	21	TAGGTAGTAGATTGTATAG	miRNA	let-7f
t0020382	20	21	AGAGGTAGTAGGTTGTATAG	miRNA	let-7d
t0020032	21	21	AGAGGTAGTAGGTTGCGTAGT	miRNA	let-7d
t0020162	22	21	AGAGGTAGTAGGTTGCATCGTT	miRNA	let-7d
t0019877	23	21	TGAGGTAGTAGGGTGTGTGGTTA	miRNA	let-7b
t0020322	21	21	TGAAGTAGTAGGTTGTGTGGT	miRNA	let-7b
t0020284	28	21	GATCTGAGGTAGTAGGTTGTGTGGTTAA	miRNA	let-7b
t0020374	20	21	TGAGGTAGTAGGTTGTGCGG	miRNA	let-7b
t0019853	22	21	TGAGGTAGTAGGTTGTATAATT	miRNA	let-7
t0019939	24	21	TGAGGTAGGAGGTTGTATAGTTGA	miRNA	let-7
t0020025	21	20	TATACAACTTACTACTTTCCT	miRNA	miR-98*
t0020326	35	20	ATTGCACTTGTCCCGGCCTGTAATACTCTC[illegible]	miRNA	miR-92b-3p
t0020337	19	20	TATTGCACTTGTCCCGGCA	miRNA	miR-92a
t0020393	18	20	GGGCAGGTGGTGTAGTGG	miRNA	miR-885
t0019870	19	20	TAGGAGATGGCGCAGGGGA	miRNA	miR-877
t0019995	23	20	AGGGCGCGCGGGTCGGGGCGGCG	miRNA	miR-638
t0020221	22	20	TCTTTTCTTTGAGACTCACTAT	miRNA	miR-627
t0019983	22	20	TACGTCATCGTTGTCATCATCT	miRNA	miR-598
t0020037	22	20	GAGCTTATTCATAAAAGTGCAA	miRNA	miR-590-5p
t0020113	28	20	TCCTGTACTGAGCTGCCCCGAGACGGCC	miRNA	miR-486-5p
t0020223	27	20	TCCTGTACTGAGCTGCCCCGAGAAGAA	miRNA	miR-486-5p
t0019972	26	20	TCCTGTACTGAGCTGCCCCGAGACCA	miRNA	miR-486-5p
t0020285	21	20	TCCTGTACTGAGCTGCGCCGA	miRNA	miR-486-5p
t0020554	22	20	AGGGAAGGAGGCTTGGTCTTAG	miRNA	miR-4747-5p
t0020563	21	20	TGGGAAGAGGAGCTGAGGGAA	miRNA	miR-4646-5p
t0020891	20	20	GGGGGAGAAGGGTCGGGGCG	miRNA	miR-4516
t0020495	23	20	GAAGGGTCGGGGCGGCAGGGGCT	miRNA	miR-4516
t0020854	22	20	GAAGGGTCGGGGCGGCAGGGGT	miRNA	miR-4516
t0020600	22	20	GAGAGAAGGGTCGGGGCGGCAA	miRNA	miR-4516
t0020602	19	20	AAACCGTTATCATTACTGA	miRNA	miR-451
t0020753	18	20	AAACCGTTACCATTACTT	miRNA	miR-451
t0020585	21	20	AAGCCGTTACCATTACTGAGT	miRNA	miR-451
t0020500	21	20	AAACGGTTACCATTACTGAGT	miRNA	miR-451
t0020535	20	20	TCCCGGGGCTGCGCGAGGCA	miRNA	miR-4449
t0020609	23	20	TGAGTGGCAGAGAGCGAGACTTA	miRNA	miR-423-5p
t0021039	25	20	TGAGGGGCAGAGAGCGAGACTTAAG	miRNA	miR-423-5p
t0020972	24	20	TGAGGGGCAGAGATCGAGACTTTT	miRNA	miR-423-5p
t0020755	24	20	TGAGGGGCAGACAGCGAGACTTTT	miRNA	miR-423-5p
t0020566	21	20	AGAGGGGCAGAGAGCGAGACT	miRNA	miR-423-5p
t0020629	20	20	TAGGGGCAGAGAGCGAGACT	miRNA	miR-423-5p
t0020633	24	20	TGAGGGGCAGAGAGCGAGACATTT	miRNA	miR-423-5p
t0020955	20	20	TAGTGGCAGAGAGCGAGACT	miRNA	miR-423-5p
t0020672	22	20	AGGAACCTTGGAGCTTCGGCAG	miRNA	miR-3928
t0020835	27	20	AACTTTGAAGACTGAAGTGGCGAAGGG	miRNA	miR-3526
t0020907	25	20	AGGAGATTGGTGGTAGATTGTTTTT	miRNA	miR-3500
t0020961	23	20	TCCTCGAACTGTTGTGGCCATTT	miRNA	miR-3487
t0020498	22	20	TCAAGAGCAATAACGAAAAATG	miRNA	miR-335
t0020492	20	20	TGCACGGCACTGGGGACACG	miRNA	miR-3177-3p
t0020761	18	20	TATTGCACTCCCGGCCTG	miRNA	miR-311-3p
t0020766	20	20	TTTTCAGTCGGATGTTTACA	miRNA	miR-30e*
t0020796	23	20	CTAGCACCATCTGAAATCGGTTA	miRNA	miR-29c
t0020895	19	20	GCGCGGGTCGGGGGGCGGG	miRNA	miR-2981
t0020975	21	20	TTCAAGTAATTCAGGATAGGC	miRNA	miR-26a
t0021003	22	20	CATTGCACTTGTCTCGGTCTGG	miRNA	miR-25

t0021031	19	20	TAGCTTATCAGACTGATGT	miRNA	miR-21
t0020702	21	20	TTTACCACCTTCTCCACCCAG	miRNA	miR-197
t0020887	24	20	CAACGGAATCCCAAAAGCAGCTAA	miRNA	miR-191
t0020982	22	20	TGATATGTATGGGTTACTTGGT	miRNA	miR-190-5p
t0020881	23	20	TGGAGAGAAAGGCAGTTCCTTAT	miRNA	miR-185
t0020577	19	20	TGGAGAGAAAGGCAGTTCT	miRNA	miR-185
t0020910	21	20	TATGGAGGTCTCTGTCTGGCT	miRNA	miR-1843-5p
t0020584	19	20	AGGTAGATAGAACAGGTTT	miRNA	miR-1839
t0020989	21	20	TATGGCACTGGTAGAATTCAC	miRNA	miR-183
t0020434	22	20	TGAGGGAGTAGATTGAATAGTT	miRNA	miR-1827
t0020696	28	20	TTTGGCAATGGTAGAACTCACACGGCCG	miRNA	miR-182
t0020833	19	20	GCAGTGAAGGCACTTGTAG	miRNA	miR-17*
t0020689	23	20	CTGCAGTGAAGGCACTTGTAGAA	miRNA	miR-17*
t0020628	26	20	ACGTAGCAGCACGTAAATATTGGAAA	miRNA	miR-16b
t0020869	23	20	TAGCAGCACGTAAATATTGGAAA	miRNA	miR-16b
t0020447	21	20	GCGGGGAGCGGTCGGGCGGCG	miRNA	miR-1607
t0020588	25	20	GGCGGGGAGCGGTCGGGCGGCGGCG	miRNA	miR-1607
t0020620	20	20	TAGCAGCACGTAAATATTAG	miRNA	miR-16
t0020703	25	20	TAGCAGCACGTAAATATTGGCGTAT	miRNA	miR-16
t0020683	22	20	TAGCAGCACGTCATGGTTTACA	miRNA	miR-15b
t0020502	26	20	AAAGCACATCATGGTTTACATAGCAA	miRNA	miR-15b
t0020642	19	20	TCAGCACATAATGGTTTGT	miRNA	miR-15a
t0020960	21	20	TAGAAGCACATAATGGTTTGT	miRNA	miR-15a
t0020956	20	20	TAGCAGCACATAATGGATTG	miRNA	miR-15a
t0020734	20	20	AGTAGCAGCACATAATGGTT	miRNA	miR-15a
t0020949	22	20	TACAGTATAGATGATGTAAAAA	miRNA	miR-144
t0020445	18	20	ACAGTATAGATGATGTAC	miRNA	miR-144
t0021037	23	20	ACAGGGTAGAACCACGGACAGGA	miRNA	miR-140
t0020614	23	20	ACCACAGGGTAGAACTACGGACT	miRNA	miR-140
t0020818	21	20	ACCCCAGGGTAGAACCACGGA	miRNA	miR-140
t0020510	24	20	TACCACAGGGTGGAACCACGGACA	miRNA	miR-140
t0020745	22	20	CGGATGAGCAAATAAAGTGGTT	miRNA	miR-1255b
t0020640	19	20	ACCGCACTGTGGGTACTTG	miRNA	miR-106b*
t0020918	27	20	TAAAGTGCTGACAGTGCAGAAAAAACC	miRNA	miR-106b
t0020446	21	20	TAAAGTGCTGACAGTGCAGAG	miRNA	miR-106b
t0020748	20	20	AGCAGCATTGTAAAGGGCTA	miRNA	miR-103a
t0020874	35	20	AGCAGCATTGTACAGGGCTATGATACTCT	miRNA	miR-103a
t0021010	20	20	AGCATCATTGTACAGGGCTA	miRNA	miR-103a
t0020478	19	20	AGCAGCATTGTACAGGGAT	miRNA	miR-103a
t0020948	23	20	TACAGTACTGTGATAACTGACAT	miRNA	miR-101c
t0020470	20	20	TACAGTACTGTGATAAATGA	miRNA	miR-101
t0020618	22	20	GTACAGTACTGTTATAACTGAA	miRNA	miR-101
t0020656	21	20	TACAGTACTGTGATAACTAAA	miRNA	miR-101
t0020853	20	20	TGAGGTAGTAGATTGTATTA	miRNA	let-7f-5p
t0020572	22	20	TGAGGTAGTAGATTGTATAGGA	miRNA	let-7f-5p
t0020928	24	20	TGAGGTAGTAGATTGTATAGAAGA	miRNA	let-7f-5p
t0020758	20	20	TTGAGGTAGTAGATTGTATA	miRNA	let-7f
t0021023	22	20	TGAGGTAGTAGAACGTATAGTT	miRNA	let-7f
t0020457	21	20	TCTGGTAGTAGATTGTATAGT	miRNA	let-7f
t0020521	20	20	GGAGGTAGTAGATTGTATAG	miRNA	let-7f
t0020783	22	20	TGGGGTGGTAGATTGTATAGTT	miRNA	let-7f
t0020797	23	20	TGAGGTAGGAGATTGTATAGTTG	miRNA	let-7e
t0020670	22	20	AGAGGTAGTAGGTTGCCTAGTT	miRNA	let-7d
t0020646	22	20	AGAGGTAGTAGGTTGCATGGTT	miRNA	let-7d
t0020715	22	20	AGAGGTAGTAGGTTGCATACTT	miRNA	let-7d
t0020880	26	20	TGAGGTAGTAGGTTGTGTGGTTAATC	miRNA	let-7b
t0020816	21	20	TCAGGTAGTAGGTTGTGTGGT	miRNA	let-7b
t0020901	21	20	TGAGGTTGTAGGTTGTATAGT	miRNA	let-7

t0021030	19	20	GAGGTAGTAGGTTGTATAG	miRNA	let-7
t0020747	20	20	TGAGGTAGTAGGTTGTACAG	miRNA	let-7
t0020899	20	20	TGAGGTAGTAGGTTTTATAG	miRNA	let-7
t0020940	20	19	CTATACAACCTACTACTTTC	miRNA	miR-98*
t0020990	22	19	AGGTCGAAGGTTGTAGGCATGT	miRNA	miR-965-5p
t0021021	23	19	GGGAGGGCGCGCGGGTCGGGGCG	miRNA	miR-638
t0020488	21	19	CTCTTTTCTTTGAGACTCACT	miRNA	miR-627
t0020529	21	19	TGTCTTACTCCCTCAGGCATT	miRNA	miR-550a*
t0020739	21	19	CAAAATTGATCGTGGTTTTTG	miRNA	miR-548t
t0020977	19	19	AAACCCGCAATTACTTTTG	miRNA	miR-548ac
t0020522	21	19	ACTGGTGCGGACCAGGGGAAT	miRNA	miR-5109
t0021004	22	19	TCTGGGCACAGGCGGATGGAAA	miRNA	miR-5107
t0020555	22	19	GGGAGCCAGGAAGTATTGATGT	miRNA	miR-505*
t0020674	26	19	TCCTGTACTGAGCTGCCCCGAGCAAG	miRNA	miR-486-5p
t0021027	29	19	TCCTGTACTGAGCTGCCCCGAGACGGCCT	miRNA	miR-486-5p
t0021032	21	19	AGAGTCGGCGACGCCGCCAAG	miRNA	miR-4785
t0021654	21	19	TCAGGCAAAGGGATATTTACA	miRNA	miR-4742-5p
t0021672	24	19	ATGTAGAGCAGGGAGCAGGAAGCA	miRNA	miR-4732-5p
t0021276	19	19	TAAGGGCTGGGTCGGTCGG	miRNA	miR-4651
t0021582	21	19	TGAGGGAGTAGGGTGTATAGT	miRNA	miR-4510
t0021150	23	19	AAACCGTTACCATTACTGAGTTC	miRNA	miR-451
t0021137	18	19	AAACCGTTACCATTACAA	miRNA	miR-451
t0021391	20	19	AAGCCGTTACCATTACTGAG	miRNA	miR-451
t0021134	20	19	AAACCGTTACCTTTACTGAG	miRNA	miR-451
t0021103	22	19	AAAGCGTTACCATTACTGAGTT	miRNA	miR-451
t0021465	22	19	ACGTTACCATTACTGAGTAAAC	miRNA	miR-451
t0021335	20	19	AAACCGTTGCCATTACTGAG	miRNA	miR-451
t0021588	23	19	AAACCGTTACCCTTACTGAGTTT	miRNA	miR-451
t0021109	26	19	AAACCGTTACCATTACTGAGTTTAAT	miRNA	miR-451
t0021171	22	19	TCCGGGACGGCTGGGAAGGCAT	miRNA	miR-4497
t0021106	19	19	CGGCTCCGGGACGGCTGGG	miRNA	miR-4497
t0021489	20	19	CGGGCTGGGCGAGGCTGGCT	miRNA	miR-4486
t0021428	20	19	ACAGGAGTGGGGGGTGGGAT	miRNA	miR-4433
t0021115	21	19	AGCGGCATTGTACAGGGCTAT	miRNA	miR-4289
t0021216	21	19	ATGACACGATCACTCCCGTTG	miRNA	miR-425-5p
t0021222	19	19	TGAGGGGCAGAGAGAGAGA	miRNA	miR-423-5p
t0021241	20	19	TGAGGGGCAGAGAGCGAGAA	miRNA	miR-423-5p
t0021342	23	19	TGAGGGGCAGAGAGCGAGGCTTA	miRNA	miR-423-5p
t0021377	19	19	GAGGGGCAGAGAGCGAGAC	miRNA	miR-423-5p
t0021494	20	19	TGGGCAGAGAGCGAGACTTT	miRNA	miR-423-5p
t0021633	24	19	TGAGGGGCAGAGAGCGAGCCTTTT	miRNA	miR-423-5p
t0021666	19	19	TGAGGGGCAGAGAGCGTGA	miRNA	miR-423-5p
t0021232	24	19	TGAGGGGCAGAGAGCGAGACTATT	miRNA	miR-423-5p
t0021585	21	19	TGGAACTCTGATATTGGCAGA	miRNA	miR-4001g-3p
t0021173	26	19	ACTGGACTTGGAGTCAGAAGGAAATC	miRNA	miR-378
t0021597	21	19	ACTGGACTTGGAGTCAGAAGT	miRNA	miR-378
t0021523	22	19	GTGGATGATGGAGACTCGGTAT	miRNA	miR-3691-5p
t0021168	22	19	GGGTGGATCACGATGCAATTTT	miRNA	miR-363*
t0021066	20	19	TTTGAAGACTGAAGTGGAGA	miRNA	miR-3526
t0021169	20	19	TAGAAGACTGCCGCTCGTTT	miRNA	miR-3494
t0021189	19	19	TCCCTGTCCTCCAGGAGCT	miRNA	miR-339b
t0021271	23	19	AAAAGCTGGGTTGAGAGGGCGCC	miRNA	miR-320d
t0021435	23	19	AAAAGATGGGTTGAGAGGGCGAA	miRNA	miR-320a
t0021480	20	19	AAAATGGGTTGAGAGGGCGA	miRNA	miR-320a
t0021520	22	19	TAGAAGGGGTGAAATTTAAACG	miRNA	miR-3179
t0021656	23	19	GGCTGGTCCGAGTGCAGTGGGGT	miRNA	miR-3135b
t0021069	22	19	GGCTGGTCCGAGTGCAGTGGGG	miRNA	miR-3135b
t0021263	24	19	TGTAAACATCCTTGACTGGAAATC	miRNA	miR-30e

t0021448	22	19	TAGTACCATCTGAAATCGGTTA	miRNA	miR-29c
t0021231	23	19	GGCGGATGTAGCCAAGTGGAGAC	miRNA	miR-270
t0021245	21	19	TTCAAGTAATTCAGGATCGGT	miRNA	miR-26b
t0021161	27	19	CATTGCACTTGTCTCGGTCTGAAGAAC	miRNA	miR-25
t0021519	22	19	AATTGCACTTGTCTCGGTCTGA	miRNA	miR-25
t0021401	19	19	CTCTAAGGGCTGGGTCGGT	miRNA	miR-2487
t0021674	23	19	TTGGGGAAACGGCCGCTGAGTAA	miRNA	miR-2110
t0021300	25	19	TAACACTGTCTGGTAACGATGTATC	miRNA	miR-200a
t0021596	23	19	CAACGGAATCCCAAAAGCAGATG	miRNA	miR-191
t0021696	22	19	TGGAGAAAAAGGCAGTTCCTGT	miRNA	miR-185
t0021373	23	19	TGGAGAGAAAGGCAATTCCTGAA	miRNA	miR-185
t0021440	23	19	TGGAGAGAAGGGCAGTTCCTGAA	miRNA	miR-185
t0021057	19	19	TGGAGAGAAAGGCAGTTTT	miRNA	miR-185
t0021053	19	19	TGGAGAGAAAGGCAGTTTC	miRNA	miR-185
t0021274	21	19	TGGAAAGAAAGGCAGTTCCTG	miRNA	miR-185
t0021338	21	19	AGGTAGATAGAACAGGGTTTG	miRNA	miR-1839
t0021414	20	19	TGAGGGAGTAGATTGTAGTT	miRNA	miR-1827
t0021510	20	19	AAGCAGCACATCATGGTTTA	miRNA	miR-15b
t0021119	21	19	TAGCAGCACATCATGGTTTCA	miRNA	miR-15b
t0021522	21	19	TAGCAGCACATAATGGTTTGC	miRNA	miR-15a
t0021605	20	19	TATAGTATAGATGATGTACT	miRNA	miR-144
t0021354	19	19	CATAAAGTAGAAAGCATTA	miRNA	miR-142-5p
t0021125	21	19	TCCATAAAGTAGAAAGCACTA	miRNA	miR-142-5p
t0021258	23	19	TACCACAGGGTAGAACCGCGGAA	miRNA	miR-140
t0021282	23	19	TATCACAGGGTAGAACCACGGAA	miRNA	miR-140
t0021400	24	19	TACCGCAGGGTAGAACCACGGACA	miRNA	miR-140
t0021422	22	19	ACCACAGGGCAGAACCACGGAC	miRNA	miR-140
t0021628	23	19	TACTACAGGGTAGAACCACGGAA	miRNA	miR-140
t0021184	24	19	TACCACGGGGTAGAACCACGGACA	miRNA	miR-140
t0021083	22	19	ACCACGGGGTAGAACCACGGAA	miRNA	miR-140
t0021657	23	19	TACCGCAGGGTAGAACCACGGAA	miRNA	miR-140
t0021688	24	19	TACCACAGGGTAGAACCCCGGACG	miRNA	miR-140
t0021054	23	19	TACCACAGGGTAGAACCACGGGT	miRNA	miR-140
t0021314	22	19	GACCACAGGGTAGAACCACGGA	miRNA	miR-140
t0021181	21	19	TCCACAGGGTAGAACCACGGA	miRNA	miR-140
t0021542	24	19	ACCACAGGGTAGAACCACGGATTA	miRNA	miR-140
t0021065	24	19	TAACACAGGGTAGAACCACGGACA	miRNA	miR-140
t0021098	19	19	CCGCACTGTGGGTACTTGC	miRNA	miR-106b*
t0021117	21	19	AAAGTGCTGACAGTGCAGATA	miRNA	miR-106b
t0021243	24	19	AGCAGCATTGTACAGGGCTATGTA	miRNA	miR-103a
t0021246	21	19	AGCAGCATTGTACCGGGCTAT	miRNA	miR-103a
t0021280	22	19	TACAGTACTATGATAACTGAAT	miRNA	miR-101b
t0021356	27	19	TACAGTACTGTGATAACTGAACGACGG	miRNA	miR-101
t0021446	19	19	ATACAGTACTGTGATAACT	miRNA	miR-101
t0021513	22	19	GTACAGTACTGGGATAACTGAA	miRNA	miR-101
t0021525	18	19	TACAGTACTTTGATAACT	miRNA	miR-101
t0021560	20	19	CTGCGCAAGCTACTGCCTTG	miRNA	let-7i*
t0021561	21	19	TGAGGTAGTAGATTGTATAAA	miRNA	let-7f-5p
t0021622	24	19	AATGAGGTAGTAGATTGTATAGTT	miRNA	let-7f
t0021658	22	19	TAGGTAGTAGATTGTATAGTTA	miRNA	let-7f
t0021374	22	19	AGTTTGAGGTAGTAGATTGTAT	miRNA	let-7f
t0021154	19	19	TGAGTTAGTAGATTGTATA	miRNA	let-7f
t0021060	22	19	TGAGGTAGTAGATTTCATAGTT	miRNA	let-7f
t0021254	19	19	TGAGGTAGTAGATTGTTTA	miRNA	let-7f
t0021349	22	19	AGCGGTAGTAGGTTGCATAGTT	miRNA	let-7d
t0021331	22	19	AGAGGTAGTAGGTTGCATATTT	miRNA	let-7d
t0021430	26	19	AGAGGTAGTAGGTTGCATAGTTAAAA	miRNA	let-7d
t0021508	22	19	AGAGGTAGTAGGTCGCATAGTT	miRNA	let-7d

t0021612	19	19	TGAGGTAGTAGGTTGTGGA	miRNA	let-7b
t0021205	23	19	TGAGGAAGTAGGTTGTGTGGTTA	miRNA	let-7b
t0021233	25	19	TGAGGTAGTAGGTTGTGTTGTTATC	miRNA	let-7b
t0021199	20	19	TGCGGTAGTAGGTTGTATAG	miRNA	let-7
t0021212	23	19	TGAGGTAGTAGGTTGTATATTTG	miRNA	let-7
t0021351	20	19	TGAGGTAGTAGGTTATATAG	miRNA	let-7
t0021445	27	19	TGAGGTAGTAGGTTGTATAGTTAAAAA	miRNA	let-7
t0021543	19	18	TTTGGCACTAGCACATTTT	miRNA	miR-96
t0021683	22	18	TATTGCACTCGTCCCGGCCTCC	miRNA	miR-92
t0021355	22	18	TTATGGTTTGCCTGGGACTGAG	miRNA	miR-584
t0021407	20	18	CCCCACACCCAAGGCTTGCA	miRNA	miR-532-3p
t0021629	22	18	AACAAACATGGTGCACTTCTTT	miRNA	miR-495
t0021680	22	18	TCCTGTACTGAGCTGCGCCGAG	miRNA	miR-486-5p
t0021358	29	18	TCCTGTACTGAGCTGCCCCGAGACGGCTC	miRNA	miR-486-5p
t0021387	22	18	TCCTGTACTGAGCTTCCCCGAG	miRNA	miR-486-5p
t0021573	21	18	TTGTACTGAGCTGCCTCGAGA	miRNA	miR-486-5p
t0021244	18	18	TCCTGTACTGAGCTGCGA	miRNA	miR-486-5p
t0021339	23	18	TCCTGTACCGAGCTGCCCCGAGT	miRNA	miR-486-5p
t0021411	20	18	GTAGGATTGTGTCGGCAGAT	miRNA	miR-4860
t0021692	21	18	AGAGTCGGCGACGCCGCCAGT	miRNA	miR-4785
t0022103	23	18	GGAGAAGGGTCGGGGCGGCAGGG	miRNA	miR-4516
t0021710	19	18	AGAGAAGGGTCGGGGCGGC	miRNA	miR-4516
t0021744	22	18	ATACCGTTACCATTACTGAGTT	miRNA	miR-451
t0021962	20	18	ACCGTTACCATTACTGAGTT	miRNA	miR-451
t0021826	29	18	AAACCGTTACCATTACTGAGTTGCGTCGG	miRNA	miR-451
t0021815	23	18	AAACCGTTACGATTACTGAGTTT	miRNA	miR-451
t0021844	21	18	AGCGTTACCATTACTGAGTTT	miRNA	miR-451
t0021872	23	18	AAACCGTTACCATTGCTGAGTTT	miRNA	miR-451
t0022061	23	18	AAACCGTTACCATTACCGAGTTT	miRNA	miR-451
t0022090	20	18	AAACCGATACCATTACTGAG	miRNA	miR-451
t0022324	21	18	AAACCGTTACCATTACTGACT	miRNA	miR-451
t0021841	22	18	TTCGGGGTCTGGGCGCGGCGAG	miRNA	miR-4508
t0021966	23	18	TTCGGGGTCTGGGCGCGGCGAAT	miRNA	miR-4508
t0021921	19	18	TCCGGGACGGCTGGGAAGG	miRNA	miR-4497
t0022175	22	18	TCGTATCCGAGTCACGGCACCA	miRNA	miR-4454
t0021801	20	18	GGCAGCATTGTACAGGGCTA	miRNA	miR-4289
t0021870	22	18	TGACGGGCAGAGAGCGAGACTT	miRNA	miR-423-5p
t0021879	21	18	GGAGGGGCAGAGAGCGAGACT	miRNA	miR-423-5p
t0021911	23	18	TGAGGGGCAGAGAGCGAGAATTA	miRNA	miR-423-5p
t0022023	23	18	TGATTGGCAGAGAGCGAGACTTT	miRNA	miR-423-5p
t0022114	25	18	TGAGGGGCAGAGAGCGAGACTTAAT	miRNA	miR-423-5p
t0022124	20	18	TGGGGCAGAGAGCGAGACTT	miRNA	miR-423-5p
t0022150	21	18	TGAGGGGCCGAGAGCGAGACT	miRNA	miR-423-5p
t0022314	22	18	TGAGGGGCAGAGAGCGAGACAT	miRNA	miR-423-5p
t0022315	23	18	TGAGGGGCAGAGAGCGCGACTTA	miRNA	miR-423-5p
t0022319	21	18	TGGAACTCTGATATTGGCAGG	miRNA	miR-4001g-3p
t0021727	22	18	TGTGTGGATCCTGGAGGAGGCA	miRNA	miR-3911
t0021753	25	18	ACTGGACTTGGAGTTAGAAGGAATC	miRNA	miR-378c
t0022208	22	18	CTGGACTTGGAGTCAGAAGGCG	miRNA	miR-378
t0021852	21	18	ACTGGACTTGGATTCAGAAGG	miRNA	miR-378
t0021916	18	18	ATATAATACAACCTGCTA	miRNA	miR-374
t0021927	22	18	GAGGGACTTTCAGGGGCAGCTG	miRNA	miR-365-5p
t0021985	21	18	CCGGGGGTGGGGTCGGCGGAT	miRNA	miR-3621
t0022011	26	18	AAGTGGAATCCAGGACGCGCGTGTTT	miRNA	miR-3503
t0022027	20	18	CAGAAGACTGCCGCTCATTT	miRNA	miR-3494
t0022068	20	18	TCACACAGAAATCGCACCCG	miRNA	miR-342-3p
t0022089	22	18	TTCACACAGAAATCGCACCCGT	miRNA	miR-342-3p
t0022298	25	18	TCTCACACAGAAATCGCACCCGTCG	miRNA	miR-342-3p

t0022328	23	18	AAAAGCTGGGTTAAGAGGGCGTA	miRNA	miR-320d
t0022228	23	18	AAAAGCTGGGTTGAGAGGGCTAT	miRNA	miR-320d
t0022308	22	18	AAAAGCTGGGTTGAGAGGACGA	miRNA	miR-320d
t0022188	22	18	AAAAGCTGGGTTGAGGGGGCGT	miRNA	miR-320d
t0022318	22	18	AAAAGCTGGGTTGAGCGGGCGT	miRNA	miR-320d
t0021957	24	18	GGCTGGTCCGAGTGCAGTGGGGTT	miRNA	miR-3135b
t0022143	23	18	CAGTGCAATAGTATTGTCAAAGC	miRNA	miR-301b-3p
t0022111	27	18	GCGCGGGTCGGGGGCGGGGCGGACTG	miRNA	miR-2981
t0022222	23	18	GCGGGTCGGGGGCGGGGCGGAC	miRNA	miR-2981
t0021834	21	18	CATTGCACTTGTCTCGGTATG	miRNA	miR-25
t0021762	21	18	CATTGCACTTGTTTCGGTCTG	miRNA	miR-25
t0021919	25	18	CATTGCACTTGTCTCGGTCTGACGG	miRNA	miR-25
t0022339	26	18	GGCTCTAAGGGCTGGGTCGGTCGGGC	miRNA	miR-2487
t0021881	24	18	ATCACATTGCCAGGGATTTCCAAT	miRNA	miR-23a
t0021891	24	18	TAGCTTATCAGACTTATGTTGATC	miRNA	miR-21
t0022186	22	18	CAACGGAATCCCATAAGCAGCT	miRNA	miR-191
t0022325	21	18	TGGAGAGAAACGCAGTTCCTG	miRNA	miR-185
t0022327	21	18	TGGAGAGAAAGGCCGTTCCTG	miRNA	miR-185
t0022199	24	18	TGGAGAGAAAGGCAGTTCCTGATT	miRNA	miR-185
t0022297	21	18	TGAAGAGAAAGGCAGTTCCTG	miRNA	miR-185
t0021929	21	18	TGGAGAGAAAGGCAGATCCTG	miRNA	miR-185
t0021991	22	18	AAGGAAGATAGAACAGGTCTTG	miRNA	miR-1839
t0021998	20	18	AGGTAGATAGAACAGGTCTT	miRNA	miR-1839
t0022080	24	18	AACATTCAACCTGTCGGTGAGATC	miRNA	miR-181c
t0022329	24	18	AACATTCATTGCTGTCGGTGGGTT	miRNA	miR-181b
t0022288	23	18	TCAAAGTGCTTACAGTGCAGGTA	miRNA	miR-17
t0021853	18	18	AGCAGCACGTAAATATTG	miRNA	miR-16
t0021858	23	18	ATAGCAGCACGTAAATATTGGTG	miRNA	miR-16
t0021923	19	18	TAGAAGCACGTAAATATTG	miRNA	miR-16
t0021740	18	18	TAGCAGCACATCATGCTA	miRNA	miR-15b
t0021802	19	18	CAGCAGCACATCATGGTTT	miRNA	miR-15b
t0021907	22	18	TAGCAGCACATCATGCTTTACA	miRNA	miR-15b
t0022079	20	18	TAGCAGCACACCATGGTTTA	miRNA	miR-15b
t0022236	24	18	TAGCAGCACATAATGGTTTGAAAA	miRNA	miR-15a
t0021720	23	18	TCTCCCAATTCTTGTACCAGTGA	miRNA	miR-150
t0022240	20	18	TCAGTGCATTACAGAACTTT	miRNA	miR-148
t0021845	21	18	ACAGTATAGATGATGTACTTT	miRNA	miR-144
t0021920	21	18	CATAAAGTAGAAAGCACTATT	miRNA	miR-142-5p
t0021996	20	18	CCCATAAAGTAGAAAGCTCT	miRNA	miR-142
t0021784	19	18	CCATAAAGTAGAAAGCACT	miRNA	miR-142
t0022051	18	18	CCCATAAAGTAGAATGCA	miRNA	miR-142
t0022230	21	18	ACCACTGGGTAGAACCACGGA	miRNA	miR-140
t0021859	24	18	TACCACAAGGTAGAACCACGGACA	miRNA	miR-140
t0022084	23	18	ACCACAGGGTAGAACCACGGACC	miRNA	miR-140
t0022118	23	18	TACCACAGGGTAGAACCACGAAC	miRNA	miR-140
t0022131	24	18	TACCACAGGGTAGAACCGCGGACA	miRNA	miR-140
t0022293	23	18	ACCACAGGGGAGAACCACGGACA	miRNA	miR-140
t0021854	23	18	CACAGGGTAGAACCACGGAAAAC	miRNA	miR-140
t0022005	23	18	TACCACAGGGTCGAACCACGGAC	miRNA	miR-140
t0021955	22	18	TACCACAGGGTCGAACCACGGA	miRNA	miR-140
t0022252	20	18	ACCACAGGGAAGAACCACGG	miRNA	miR-140
t0021771	23	18	TGGAATGTAAAGAAGTATGGAGA	miRNA	miR-1-3p
t0021888	21	18	CAGTGTAATGTTAAAAGGGCA	miRNA	miR-130a
t0022086	20	18	TGCTGGATCAGTGGTTCGAG	miRNA	miR-1287
t0021746	20	18	GTGAGGACTCGGGAGGGGGA	miRNA	miR-1224
t0021772	29	18	TACCGCACTGTGGGTACTTGCTAAAAAGC	miRNA	miR-106b*
t0021774	21	18	TAAAGTGCTGACAGGGCAGAT	miRNA	miR-106b
t0021848	21	18	AAAGTGCTGACAGTGCAGATT	miRNA	miR-106b

t0021898	23	18	AGCAGCATTGTACGGGGCTATGA	miRNA	miR-103a
t0021983	22	18	AGCAGCACTGTACAGGGCTATG	miRNA	miR-103a
t0022173	23	18	AGCAGCATCGTACAGGGCTATGA	miRNA	miR-103a
t0022224	20	18	AGCAGCATTGTACAGCGCTA	miRNA	miR-103a
t0022269	22	18	AGCGGCATTGTACAGGGCTATG	miRNA	miR-103a
t0022321	21	18	AGCAGCATTGTCCAGGGCTAT	miRNA	miR-103a
t0022212	23	18	TACAGTACTGTGATAACTGATTA	miRNA	miR-101c
t0021819	21	18	TGCAGTACTGTGATAACTGAA	miRNA	miR-101
t0022305	23	18	TACAGTACTGTGATAACTGAATC	miRNA	miR-101
t0022071	21	18	TATAGTACTGTGATAACTGAA	miRNA	miR-101
t0021795	19	18	TGAGGTAGTAGTTTGTCCT	miRNA	let-7i
t0022174	24	18	ATGAGGTAGTAGATTGTATAGTTA	miRNA	let-7f
t0022312	19	18	TGAGGCAGTAGATTGTATA	miRNA	let-7f
t0021707	21	18	TGAGGTAGCATATTGTATAGT	miRNA	let-7f
t0021767	23	18	TGAGGCAGTAGATTGTATAGTTA	miRNA	let-7f
t0021889	19	18	TCTGGTAGTAGATTGTATA	miRNA	let-7f
t0022018	21	18	CTATACGACCTGCTGCCTTTC	miRNA	let-7d*
t0022047	21	18	AGAGGTAGTAGGTTGCACAGT	miRNA	let-7d
t0022049	20	18	AGAGGCAGTAGGTTGCATAG	miRNA	let-7d
t0022069	27	18	TGAGGTAGTAGGTTGTGTGGTTGAAAC	miRNA	let-7b
t0021716	22	18	TGAGGAAGTAGGTTGTGTGGTA	miRNA	let-7b
t0022161	27	18	TGAGGTAGTAGGTTGTGTGGTTAAATC	miRNA	let-7b
t0022257	22	18	TGAGGAGGTAGGTTGTATAGTT	miRNA	let-7
t0022221	19	18	TGAGGTAGTACGTTGTATA	miRNA	let-7
t0021760	20	18	TAAGGTAGTAGGTTGTATAG	miRNA	let-7
t0021867	21	17	CTATACAACTTACTACTTTCA	miRNA	miR-98*
t0021894	23	17	CAAAGTGCTGTTCGTGCCGGTAG	miRNA	miR-93
t0022012	22	17	TATTGCACTCGTCCCGGCCATT	miRNA	miR-92
t0022044	19	17	TTGCACTCGTCCCGGCCTC	miRNA	miR-92
t0021839	21	17	TTAATATCGGACAACCATTGT	miRNA	miR-889
t0021909	19	17	AATGGCGCCACTAGGTGTG	miRNA	miR-652
t0022122	25	17	AGGGCGCGCGGGTCGGGGCGCCGGA	miRNA	miR-638
t0021787	20	17	CCAAAACTGCAGTTACTTTT	miRNA	miR-548o
t0022062	18	17	AAAAGTATTTGCGGGTTT	miRNA	miR-548l
t0022276	21	17	TAGCAGCGGGAACAGTTCAAG	miRNA	miR-503
t0021725	20	17	ATGCACCTGGGCAAGGATTC	miRNA	miR-500a*
t0021901	21	17	TCCTGTCCTGAGCTGCCCCGA	miRNA	miR-486-5p
t0022119	24	17	TCTTGTACTGAGCTGCCCCGAGTT	miRNA	miR-486-5p
t0022595	22	17	TCCTGTACTGAGCTCCCCCGAA	miRNA	miR-486-5p
t0022966	21	17	TGTAGAATTGTGTCGGCAGAT	miRNA	miR-4860
t0022789	24	17	TGTAGAATTGTGTCGGCAGATTTT	miRNA	miR-4860
t0022985	20	17	TCAGGCTCAGTCCCCTCCCG	miRNA	miR-484
t0022558	20	17	CTCGGGCGGAGGTGGTTGAG	miRNA	miR-4750
t0022517	27	17	TGTAGAGCAGGGAGCAGGAAGCTGAGA	miRNA	miR-4732-5p
t0022474	24	17	ACTGGGGAGCAGAAGGAGAACAAG	miRNA	miR-4667-5p
t0022722	20	17	GAGAAGGGTCGGGGCGGCAG	miRNA	miR-4516
t0022539	22	17	TGAGGTAGTAGGGTGTGTGGTA	miRNA	miR-4510
t0022362	22	17	TGAGGGAGTAGGGTGTATAGTT	miRNA	miR-4510
t0022645	23	17	GAACCGTTACCATTACTGAGTTT	miRNA	miR-451
t0022523	21	17	AAACCGTTACCATTACTTAGA	miRNA	miR-451
t0022764	20	17	AAACCGTTACCATTACTCAG	miRNA	miR-451
t0022967	21	17	AAACCGTTACCAATACTGAGT	miRNA	miR-451
t0022936	29	17	AAACCGTTACCATTACTGAGTTCGTCGAT	miRNA	miR-451
t0022989	20	17	AGACCGTTACCATTACTGAG	miRNA	miR-451
t0022653	20	17	GAACCGTTACCATTACTGAG	miRNA	miR-451
t0022567	19	17	TTCGGGGTCTGGGCGCGGA	miRNA	miR-4508
t0022457	23	17	CTCGAATCCGAGTCACGGCACCA	miRNA	miR-4454
t0022795	18	17	GCATTGTACAGGGCTATT	miRNA	miR-4289

t0022755	22	17	AATTGCACGGTATCCATCTTTT	miRNA	miR-363
t0022414	21	17	CCGGGGGTGGGGTCGGCGGGG	miRNA	miR-3621
t0022441	23	17	TGAAGACTTAAGTGGAGAAGGGT	miRNA	miR-3526
t0022361	21	17	TTTGAAGACTGAAGTGGAGAA	miRNA	miR-3526
t0022463	28	17	AACTTTGAAGACTGAAGTGGCGAAGGGT	miRNA	miR-3526
t0022611	30	17	ACTTTGAAGACTGAAGTGGAGAAGGGTTT	miRNA	miR-3526
t0022684	18	17	TGAGTAGTAGGTTGTATA	miRNA	miR-352
t0022724	22	17	GCAAAGCACACGGCCTGCAGAG	miRNA	miR-330-3p
t0022757	22	17	AAAAGATGGGTTGAGAGGGCGT	miRNA	miR-320d
t0022799	23	17	GAAAAGCTGGGTTGAGAGGGCAG	miRNA	miR-320d
t0022352	24	17	AAAAGCTGGGTTGAGAGGGTAGAA	miRNA	miR-320d
t0022894	20	17	AAAGCTGCGTTGAGAGGGCA	miRNA	miR-320b
t0022482	23	17	AAAAGCTGGGTTGAGCGGGCGAA	miRNA	miR-320a
t0022716	21	17	CTGGGAGGTGGATGTTTACTT	miRNA	miR-30b*
t0022569	26	17	TGTAAACATCCCCGACTGGTAGCATC	miRNA	miR-30a-5p
t0022430	24	17	GCGCGGGTCGGGGGCGGGGCGGA	miRNA	miR-2981
t0022509	22	17	TTCACAGTGGCTAAGTTCCGCA	miRNA	miR-27a-3p
t0022707	21	17	TTCAAGTAATTCAGTATAGGT	miRNA	miR-26b
t0022970	22	17	AGGCGGAGACTTGGGCAATTTT	miRNA	miR-25*
t0022485	22	17	CATTGCACTTGTCTCGGACTGA	miRNA	miR-25
t0022371	20	17	GACTGGGGCGGAACATCTGT	miRNA	miR-219-2-3p
t0022425	25	17	CGGAATGTAAAGAAGTATGTATATC	miRNA	miR-1a
t0022594	25	17	TGTGCAAATCTATGCAAAACTGAAA	miRNA	miR-19a
t0022712	21	17	TAGGTAGTTTCCTGTTGTTGG	miRNA	miR-196b
t0022964	24	17	TGACCTATGAATTGACAGCCAAAA	miRNA	miR-192
t0022628	21	17	AACGGAATCCCAAAAGCAGCA	miRNA	miR-191
t0022765	21	17	TACTGCCCTAAATGCCCCTTA	miRNA	miR-18b*
t0022918	23	17	TGGAGAGAAAGGCAGGTCCTGAA	miRNA	miR-185
t0022771	22	17	TGGAGAGAAAGGCGGTTCCTGT	miRNA	miR-185
t0022802	19	17	TGGAGAGAAAGGCAGTTAA	miRNA	miR-185
t0022560	23	17	TGGAGAGCAAGGCAGTTCCTGAA	miRNA	miR-185
t0022586	21	17	AGGGAGATAGAACAGGTCTTG	miRNA	miR-1839
t0022429	21	17	AGGTAGATACAACAGGTCTTG	miRNA	miR-1839
t0022453	19	17	AAGCGGCGATGGCGGAGCA	miRNA	miR-1636
t0022627	23	17	TAGAAGCACGTAAATATTGGCGA	miRNA	miR-16
t0022719	20	17	TAACAGCACGTAAATATTGG	miRNA	miR-16
t0022743	18	17	TAGCAGCACGTAAATTTG	miRNA	miR-16
t0022897	22	17	TAGCAGCACATCATTGTTTACA	miRNA	miR-15b
t0022381	20	17	TAGCAGCACATAGTGGTTTG	miRNA	miR-15a
t0022865	20	17	TAGCAGCACATAACGGTTTG	miRNA	miR-15a
t0022436	21	17	CTCCCAACCCTTGTACCAGTG	miRNA	miR-150
t0022636	23	17	TGAGATGAAGCATTGTAGCTATC	miRNA	miR-143
t0022751	19	17	CATAAACTAGAAAGCACTA	miRNA	miR-142-5p
t0022774	23	17	TACCACCGGGTAGAACCACGGAA	miRNA	miR-140
t0022377	24	17	TACCACAGGGTAAAACCACGGACA	miRNA	miR-140
t0022805	23	17	TACCACAGGGTAGGACCACGGAC	miRNA	miR-140
t0022917	24	17	TACCACAGGGCAGAACCACGGACA	miRNA	miR-140
t0022493	23	17	TACCACAGGGTAGTACCACGGAT	miRNA	miR-140
t0022564	24	17	TATCACAGGGTAGAACCACGGAAA	miRNA	miR-140
t0022990	23	17	TACCACAGGGAAGAACCACGGAA	miRNA	miR-140
t0022566	22	17	TACCACAGGGTAGAACCACGAA	miRNA	miR-140
t0022610	21	17	ACCACAGGGTAGAACCTCGGA	miRNA	miR-140
t0022652	26	17	TACCACAGGGTAGAACCACGGACAAT	miRNA	miR-140
t0022957	23	17	TACCACCGGGTAGAACCACGGAC	miRNA	miR-140
t0022959	23	17	GCAGTGCAATGTTAAAAGGGCAT	miRNA	miR-130a
t0022909	21	17	TCTGGGCAACAAAGTGAGACC	miRNA	miR-1285
t0022458	25	17	GGCGACAAAACGAGACCCTGTCAAA	miRNA	miR-1273c
t0022470	22	17	ATCCCACCGCTGCCACCATAAA	miRNA	miR-1260b

t0022518	25	17	TCGTACCGTGAGTACTAATGCGATC	miRNA	miR-126
t0022532	25	17	TACCCTGTATATCCGAATTTGTATC	miRNA	miR-10a
t0022601	23	17	ATAAAGTGCTGACAGTGCAGAAA	miRNA	miR-106b
t0022671	21	17	AGCAGCATTGTGCAGGGCTAT	miRNA	miR-103a
t0022768	18	17	AGCAGCATTGTACAGGGT	miRNA	miR-103a
t0022835	22	17	AGGAGCATTGTACAGGGCTATG	miRNA	miR-103a
t0022847	21	17	AGCAGCATTGTACAGAGCTAT	miRNA	miR-103a
t0022849	23	17	AGCGGCATTGTACAGGGCTATGA	miRNA	miR-103a
t0022850	26	17	GTACAGTACTGTGATAACTGAGGCAT	miRNA	miR-101c
t0022999	22	17	GTACAGCACTGTGATAACTGAA	miRNA	miR-101
t0022906	21	17	TACAGTACCGTGATAACTGAA	miRNA	miR-101
t0022396	19	17	TGTAGTAGTTTGTGCTGTT	miRNA	let-7i
t0022478	22	17	TGGGGTAGTAGTTTGTGCTGTT	miRNA	let-7i
t0022409	22	17	TGAGGTAGTAGTCTGTACAGTT	miRNA	let-7g
t0022630	22	17	TGAGGTAGTAGTTTGGACAGTT	miRNA	let-7g
t0022700	22	17	TGAGATAGTAGTTTGTACAGTT	miRNA	let-7g
t0022496	25	17	TGAGGTAGTAGATTGTATAGAGATT	miRNA	let-7f-5p
t0022622	24	17	TTGAGGTAGTAGATTGTATAGTTA	miRNA	let-7f
t0022677	20	17	TGAGGTAGTAGATTGTAGAT	miRNA	let-7f
t0022883	20	17	AGAGGTAGTAGGTTGAATAG	miRNA	let-7d
t0022904	22	17	AGAGGTAGTAGGTTGCATAGAT	miRNA	let-7d
t0022434	22	17	AGAGGTAGTAGTTTGCATAGTT	miRNA	let-7d
t0022624	25	17	AGAGGTAGTAGGTTGCATAGTTATG	miRNA	let-7d
t0022925	26	17	TGAGGTAGTAGGTTGTATGGTTATCT	miRNA	let-7c
t0022418	20	17	TGGGGTAGTAGGTTGTGTGG	miRNA	let-7b
t0022698	24	17	TGAGGTAGTAGGTTGTGTAGTTTT	miRNA	let-7b
t0022620	18	17	TGAGGTACTAGGTTGTGT	miRNA	let-7b
t0022866	23	17	TGAGGTAGTAGGTTGTGGGGTTT	miRNA	let-7b
t0022869	20	17	CGAGGTAGTAGGTTGTGTGG	miRNA	let-7b
t0022579	22	17	TGAGGTAGTAGGTTGTGCAGTT	miRNA	let-7b
t0022562	24	17	TGAGGTAGTAGGTTGTGGGGTTAA	miRNA	let-7b
t0022984	24	17	TGAGGTAGTAGGTTGTATAGTAAT	miRNA	let-7-5p
t0022634	19	17	TGAGGTAGTAGGGTGTATA	miRNA	let-7
t0022738	21	17	GTGAGGTAGTAGGTTGTATAG	miRNA	let-7
t0022873	20	17	TGAGGTAGTCGGTTGTATAG	miRNA	let-7
t0022902	21	17	CTGAGGTAGTAGGTTGTATAG	miRNA	let-7
t0022411	20	17	TGAGGTAGTAGGTTGTATAC	miRNA	let-7
t0022370	22	16	TATTGTACTTGTTCCGGCCTGT	miRNA	miR-92d
t0022455	22	16	TATTGCACTTGTCCCGGCTCGT	miRNA	miR-92a
t0022520	23	16	TATTGCACTTGTCCCGGTCTGTA	miRNA	miR-92a
t0022568	21	16	TGAGTGTGTGTGTGTGAGTGT	miRNA	miR-574-5p
t0022872	22	16	CAAAAGTAATTGCGGTCTTTGG	miRNA	miR-548j
t0022882	22	16	AAAGTGATTGTGGTTTTTGTCA	miRNA	miR-548d-5p
t0022891	25	16	TCTGGGCACAGGCGGATGGACAGGA	miRNA	miR-5107
t0022621	20	16	GGGAGCCAGGAAGTATTGAT	miRNA	miR-505*
t0022582	23	16	TCCTGTACTGAGCTGCCACGAGA	miRNA	miR-486-5p
t0022642	21	16	TCCTGTACTGAGCTGCCCCTA	miRNA	miR-486-5p
t0022715	21	16	TCCTGTACTGAGCTGGCCCGA	miRNA	miR-486-5p
t0022787	21	16	TCCTGTACTTAGCTGCCCCGA	miRNA	miR-486-5p
t0022794	23	16	GCTGGGTCGGTCGGGCTGGGGCG	miRNA	miR-4651
t0023019	20	16	AAACCGTTAGCATTACTGAG	miRNA	miR-451
t0023298	22	16	AAACCTTTACCATTACTTAGTT	miRNA	miR-451
t0023399	25	16	AAACCGTTACCATTACTGGGTTTAG	miRNA	miR-451
t0023502	24	16	CAGAAACCGTTACCATTACTGAGT	miRNA	miR-451
t0023035	23	16	CGTTACCATTACTGAGTAAACAA	miRNA	miR-451
t0023303	22	16	AAACCTTACCATTACTGAGTTA	miRNA	miR-451
t0023055	23	16	CAGCAGCAATTCATGTTTTGAAA	miRNA	miR-424
t0023170	25	16	TGAGGGGCAGAGAGCGAGACTTTTA	miRNA	miR-423-5p

t0023063	22	16	TGCGGGGCAGAGAGCGAGACTT	miRNA	miR-423-5p
t0023205	24	16	TGAGGGGCAGAGAGCGAGATTTTT	miRNA	miR-423-5p
t0023386	22	16	TAGGGGCAGAGAGCGAGACTTA	miRNA	miR-423-5p
t0023649	22	16	GAGGGGCAGAGAGCGAGACTTA	miRNA	miR-423-5p
t0023333	23	16	TGAGGGGAAGAGAGCGAGACTTA	miRNA	miR-423-5p
t0023142	24	16	TGAGCGGCAGAGAGCGAGACTTTT	miRNA	miR-423-5p
t0023150	19	16	CGGCGGCGGCGGGGGTGTG	miRNA	miR-3885-5p
t0023323	19	16	GGCGGCGGCGGCGGGGGCG	miRNA	miR-3885-5p
t0023443	25	16	GAGACTTACGTTGCGTCAAGAGTTA	miRNA	miR-3850-3p
t0023498	24	16	ACTGGACTTGGAGTCAGAAGGCAT	miRNA	miR-378
t0023655	25	16	TTGAAGACTGAAGTGGAGAAGGTTT	miRNA	miR-3526
t0023565	23	16	TGAGGACTGAAGTGGGGAAAGGT	miRNA	miR-3526
t0023050	21	16	TCTGTATCCGTGCTGAGATTT	miRNA	miR-3499
t0023198	23	16	TCTCACACAGAAATAGCACCCGT	miRNA	miR-342-3p
t0023338	18	16	TCTCACACAGAAATCGCG	miRNA	miR-342-3p
t0023416	22	16	AAAAGCTGGGTTGAAAGGGCGT	miRNA	miR-320d
t0023473	20	16	AAAGCTGGGTTGAGAGGGAG	miRNA	miR-320d
t0023537	22	16	GAAAAGCTGGGTTGAGAGGGCA	miRNA	miR-320b
t0023630	23	16	AAAGCTGGGTTGAGAGGGCGAAA	miRNA	miR-320a
t0023083	22	16	TTCAAGTAATTCAGGATAGGGT	miRNA	miR-26b
t0023596	24	16	TTCAAGTAATTCAGGATAGGTTAA	miRNA	miR-26b
t0023185	18	16	TTCAAGTAATCCAGGATA	miRNA	miR-26a
t0023540	35	16	CATTGCACTTGTCTCGGTCTGCAGGAATT	miRNA	miR-25
t0023251	22	16	CATTGCCCTTGTCTCGGTCTGA	miRNA	miR-25
t0023334	23	16	TGGCTCAGTTCAGCAGGAACCGT	miRNA	miR-24b
t0023301	19	16	ATCATATTGCCAGGGATTT	miRNA	miR-23a
t0023023	23	16	GTCAGTTTGTCAAATACCCCAAT	miRNA	miR-223
t0023066	22	16	CTGACCTATGAATTGACAGCAA	miRNA	miR-192
t0023207	24	16	TGACCTATGAATTGACAGCCAGAT	miRNA	miR-192
t0023420	23	16	TCTGACCTATGAATTGACAGCCT	miRNA	miR-192
t0023054	20	16	TAACGGAATCCCAAAAGCAG	miRNA	miR-191
t0023192	22	16	AAAGGAATCCCAAAAGCAGCTG	miRNA	miR-191
t0023106	23	16	CAACGGAATCCCAAAAGCCGCTG	miRNA	miR-191
t0023391	22	16	TGGAGAGAAAGGCAGTGCCTGA	miRNA	miR-185
t0023350	23	16	TGGAGAGAAAGGCAGTTCCTGGT	miRNA	miR-185
t0023256	20	16	TGGAGAGAAAGGCAGTTACT	miRNA	miR-185
t0023318	20	16	TGAGGGAGTAGATTGTATTT	miRNA	miR-1827
t0023267	18	16	ACATTCATTGCTGTCGGT	miRNA	miR-181b
t0023233	26	16	TAGCAGCACGTAAATATTGGCCATCA	miRNA	miR-16b
t0023487	20	16	GCGGGGAGCGGTCGGGCGGT	miRNA	miR-1607
t0023094	26	16	GGGGCGGGGAGCGGTCGGGCGGCGGC	miRNA	miR-1607
t0023223	21	16	TAGCAGCACGTAAATATTCGC	miRNA	miR-16
t0023248	20	16	TAGCAGTACGTAAATATTGG	miRNA	miR-16
t0023032	22	16	TAGCAGAACGTAAATATTGGAG	miRNA	miR-16
t0023526	19	16	TAGCAGCACATCATGGCTA	miRNA	miR-15b
t0023615	21	16	CAGCAGCACATCATGGTTTAC	miRNA	miR-15b
t0023263	22	16	TAGCATCACATCATGGTTTACA	miRNA	miR-15b
t0023280	22	16	TAGCAGCACATCATGGTTTACG	miRNA	miR-15b
t0023467	20	16	TAGGAGCACATCATGGTTTA	miRNA	miR-15b
t0023090	22	16	TAGTAGCAGCACATCATGGTTT	miRNA	miR-15b
t0023097	22	16	TAGCAGCCCATCATGGTTTACA	miRNA	miR-15b
t0023362	20	16	TAGCAGCACATAATGGCTTG	miRNA	miR-15a
t0023189	22	16	CTCCCAACCCTTGTACCAGTGA	miRNA	miR-150
t0023595	26	16	ACAGTGCACTACAGAACTTTGTCATC	miRNA	miR-148a
t0023136	19	16	CTACAGTATAGATGATGTT	miRNA	miR-144
t0023290	21	16	CATAAAGTAGAAAGCACTTCT	miRNA	miR-142
t0023518	22	16	TACCACAGGGTAGAACTACGGC	miRNA	miR-140
t0023056	22	16	ACTACAGGGTAGAACCACGGAC	miRNA	miR-140

t0023117	23	16	TACCACAGGGGAGAACCACGGAT	miRNA	miR-140
t0023212	23	16	TACCACAGGGTAAAACCACGGAC	miRNA	miR-140
t0023328	23	16	ACCACAGGGTAGAACCCCGGACA	miRNA	miR-140
t0023428	23	16	TGCCACAGGGTAGAACCACGGAC	miRNA	miR-140
t0023523	25	16	TACCACAGGGTAGAACCACGGAATC	miRNA	miR-140
t0023561	23	16	TACCACAGGGTAGAACAAAGGAC	miRNA	miR-140
t0023209	22	16	TACCACAGGGTAGAACCACTGA	miRNA	miR-140
t0023651	23	16	TACCACAGAGTAGAACCACGGAC	miRNA	miR-140
t0023425	21	16	TACCACAGGGTAGAACAACGG	miRNA	miR-140
t0023197	22	16	ACCACAGGGTAGAACCGCGGAC	miRNA	miR-140
t0023369	23	16	TACCACAGGGTAGAGCCACGGAC	miRNA	miR-140
t0023025	23	16	CTACCACAGGGTAGAACCACGGA	miRNA	miR-140
t0023074	21	16	CCACCTCCCCTGCAAACGTCC	miRNA	miR-1306
t0023133	23	16	TCTGGGCAACAAAGTGAGACCTG	miRNA	miR-1285
t0023163	21	16	TAGTACTGTGCATATCATCTA	miRNA	miR-1278
t0023289	20	16	AGGCATTGACTTCTCACTAG	miRNA	miR-1256
t0023361	25	16	TATCCTGTAGATCCGAATTTGTATC	miRNA	miR-10a
t0023363	23	16	AGCAGCATTGTACCGGGCTATGA	miRNA	miR-103a
t0023411	22	16	AGCAGCATTGAACAGGGCTATG	miRNA	miR-103a
t0023462	24	16	AGCAGCATTTTACAGGGCTATGAT	miRNA	miR-103a
t0023474	22	16	TACAGTACTGTGATAACTTATA	miRNA	miR-101c
t0023494	20	16	TACAGTAATGTGATAACTGA	miRNA	miR-101
t0023495	25	16	GTACAGTACTGTGATAACTGAAAAA	miRNA	miR-101
t0023575	22	16	TACAGTACTGTGATAACTTAAG	miRNA	miR-101
t0023578	20	16	GTACAGTACTGTGATAACTA	miRNA	miR-101
t0023336	19	16	TGAGGTAGTGTATTGTATA	miRNA	let-7f-5p
t0023531	22	16	TGAGGTGCTAGATTGTATAGTT	miRNA	let-7f
t0023442	19	16	TGAGGAAGTAGATTGTATA	miRNA	let-7f
t0023283	23	16	TGAGGAAGTAGATTGTATAGTTA	miRNA	let-7f
t0023051	25	16	TGAGGTAGTAGATTGTATAGTTACA	miRNA	let-7f
t0023068	21	16	AGAGGCAGTAGGTTGCATAGT	miRNA	let-7d
t0023081	22	16	AGAGGTAGTAGGTTGGATAGTT	miRNA	let-7d
t0023295	21	16	AGAGGAAGTAGGTTGCATAGT	miRNA	let-7d
t0023291	22	16	AGAGGTAGTAGGTTGCACAGTT	miRNA	let-7d
t0023112	21	16	AGAGGTAGTAGATTGCATAGT	miRNA	let-7d
t0023314	23	16	TGAGGTAGTAGGTTGTGCGGTTA	miRNA	let-7b
t0023482	23	16	CTGAGGTAGTAGGTTGTGTGGTT	miRNA	let-7b
t0023632	27	16	TGAGGTAGTAGGTTGTGTGGTAATATC	miRNA	let-7b
t0023553	34	16	TGAGGTAGTAGGTTGTGTGGTTGCTTACT(	miRNA	let-7b
t0023101	20	16	TGAGGTAGTAGTTTGTGTGG	miRNA	let-7b
t0023194	22	16	TGAGGTAGTAGGTTGTGTTTTT	miRNA	let-7b
t0023253	26	16	TGAGGTAGTAGGTTGTGTGGTTAGAA	miRNA	let-7b
t0023441	22	16	TGAGGTAGTAGGTTGTATATTC	miRNA	let-7-5p
t0023088	21	16	TCAGGTAGTAGGTTGTATAGT	miRNA	let-7
t0023100	20	16	TGAGGTAGTAGGTTGGATAG	miRNA	let-7
t0023341	20	16	TGAGGTAGTAGGTTGAATAG	miRNA	let-7
t0023409	24	16	TGAGGTAGTAGGTTGTATAGTTAG	miRNA	let-7
t0023601	20	16	TGAGTTAGTAGGTTGTATAG	miRNA	let-7
t0023127	21	16	TGAGGTAGTAGGTGGTATAGT	miRNA	let-7
t0023164	21	15	TATACAACTTACTACTTTCAT	miRNA	miR-98*
t0023324	19	15	GGTGGAAGGTTGTAGGCAT	miRNA	miR-965-5p
t0023340	23	15	GGTGGAAGGTTGTAGGCATGTAT	miRNA	miR-965-5p
t0023452	22	15	CAAAGTGCTGTTCGTGCAGGAA	miRNA	miR-93
t0023514	22	15	CAAAGTGCTGTTCGTGCAGGGA	miRNA	miR-93
t0023585	20	15	GGGCAGGTGGTGTAGTGGTA	miRNA	miR-885
t0023648	24	15	AGAGGAGATGGCGCAGGGGACACC	miRNA	miR-877
t0023155	24	15	TGCGGGGCTAGGGCTAACAGCAGA	miRNA	miR-744
t0023165	21	15	TGGAAGACTAGTGATTTTGTT	miRNA	miR-7

t0023310	19	15	CTTTTTTCTTTGAGACTCA	miRNA	miR-627
t0023461	23	15	TGTGACAGATTGATAACTGAATC	miRNA	miR-542-3p
t0023547	20	15	CCGCGAGGGGCCCGGGGCGG	miRNA	miR-5126
t0023548	20	15	TCCTGTACTGAGCTGACCCG	miRNA	miR-486-5p
t0023807	22	15	TCCTGTACTGAGCTGCCGCGAG	miRNA	miR-486-5p
t0023852	20	15	TCCTGTACTGAGCTGCCCAG	miRNA	miR-486-5p
t0023882	27	15	TCCTGTACTGAGCTGCCCCGAGCGGCA	miRNA	miR-486-5p
t0023782	21	15	TCCTGTACTGAGCTGCCCCCA	miRNA	miR-486-5p
t0024303	23	15	TGTAGAGCAGGGAGCTGGAAGCT	miRNA	miR-4732-5p
t0024140	20	15	AGAAGGGTCGGGGCGGTTTT	miRNA	miR-4516
t0024347	20	15	GAGACGGGTCGGGGCGTGCT	miRNA	miR-4516
t0023778	22	15	TGAGGGAGTAGGTTGTGGGGTT	miRNA	miR-4510
t0023773	22	15	AAACCGTTACCAATACTGAGTT	miRNA	miR-451
t0023886	26	15	AAACCGTTACCATTACTGAGAGTAAC	miRNA	miR-451
t0024143	24	15	AAAACCGTTACCATTACTGAGTTT	miRNA	miR-451
t0024041	19	15	ACCGTTACCATCACTGATT	miRNA	miR-451
t0023671	26	15	AAACCGTTACCATTACTGAGTAAAAA	miRNA	miR-451
t0023779	18	15	AAACTTTACCATTACTGA	miRNA	miR-451
t0023991	23	15	CAAACCGTTACCATTACTGAGTT	miRNA	miR-451
t0024072	28	15	AAACCGTTACCATTACTGAACTCTGAAC	miRNA	miR-451
t0024219	20	15	TTCGGGGTCTGGGCGCGGCT	miRNA	miR-4508
t0024335	19	15	GCACGGGCTGGGCGAGGCT	miRNA	miR-4486
t0023946	26	15	GTAGAGCTGAAGACTGTAGATCTTTA	miRNA	miR-4451
t0024299	22	15	AAAAGCTGGGCTGAGAGGGCGT	miRNA	miR-4429
t0024158	20	15	AGCCGCATTGTACAGGGCTA	miRNA	miR-4289
t0023704	21	15	TGACGGGCAGAGAGCGAGACT	miRNA	miR-423-5p
t0023810	24	15	TGAGGGGCAGAGAGCGAGGCTTTT	miRNA	miR-423-5p
t0023905	22	15	TGAGGGGCAGAGAGAGAGAATT	miRNA	miR-423-5p
t0023977	21	15	TGTGGGGCAGAGAGCGAGACT	miRNA	miR-423-5p
t0023993	23	15	TGAGGGGCAGAGAGCGAGACTGT	miRNA	miR-423-5p
t0024180	22	15	TGGGGGCAGAGAGCGAGACTTT	miRNA	miR-423-5p
t0024239	25	15	TGAGGGGCAGAGAGCGAGACTTTTG	miRNA	miR-423-5p
t0024290	22	15	AGGGGCAGAGAGCGAGACTTTA	miRNA	miR-423-5p
t0024077	24	15	TGAGGGGCAGATAGCGAGACTTTT	miRNA	miR-423-5p
t0024354	23	15	TGAGGGGCAGAGCGCGAGACTTA	miRNA	miR-423-5p
t0024160	22	15	TGAGGGGGAGAGAGCGAGACTT	miRNA	miR-423-5p
t0023749	22	15	ATGAGGATGGATAGCAAGGAGA	miRNA	miR-3605-5p
t0024192	30	15	TTTGAAGACTGAAGTGGAGAAGGGTTTCT	miRNA	miR-3526
t0023685	19	15	TGAAGACTGAAGTGGAGAA	miRNA	miR-3526
t0023885	22	15	GCCCCTGGGCCTATCCTAGACA	miRNA	miR-331
t0023900	22	15	AAAAGCTGGGTTGGGAGGGCGT	miRNA	miR-320d
t0023943	23	15	AAAAGCTAGGTTGAGAGGGCGAA	miRNA	miR-320a
t0024067	22	15	AATAGAGTAGGCAAAGGACAGA	miRNA	miR-3121-3p
t0024069	24	15	TGTAAACATCCTTGACTGGAAGAA	miRNA	miR-30e
t0024070	26	15	GCGCGCGGGTCGGGGGGCGGGGCGGA	miRNA	miR-2981
t0024172	22	15	CGGGTCGGGGGGCGGGGCGGAC	miRNA	miR-2981
t0024183	22	15	TTCAAGTAATCCAGGATAGGAT	miRNA	miR-26a
t0024304	35	15	CATTGCACTTGTCTCGGTCTGAATACTCT	miRNA	miR-25
t0024317	22	15	CGTTGCACTTGTCTCGGTCTGA	miRNA	miR-25
t0023937	36	15	CATTGCACTTGTCTCGGTCTGCTTGAACT	miRNA	miR-25
t0024006	22	15	TGGCTCAGTTCAGCAGGAACCG	miRNA	miR-24b
t0024231	24	15	ATAAAGTGCTTATAGTGCAGGTAG	miRNA	miR-20
t0024184	20	15	TAGCAGCACATAAATATTGG	miRNA	miR-195
t0024319	22	15	CAACGGAATCCCAAAAGCAGAA	miRNA	miR-191
t0023920	22	15	AAACGGAATCCCAAAAGCAGCT	miRNA	miR-191
t0023913	20	15	CAACGGAATCCCAAAAGAAG	miRNA	miR-191
t0023973	22	15	TGATATGTTTGATATATTAGGT	miRNA	miR-190
t0023752	24	15	TGGAGAGAAAGGCAGTTCCTGGGG	miRNA	miR-185

t0024325	25	15	TGAGGGAGTAGGTTGTATGGTTATC	miRNA	miR-1827
t0024134	19	15	TGAGGGAGTAGGTTGTATA	miRNA	miR-1827
t0023690	24	15	TTTGGCAATGGTAGAACTTACACT	miRNA	miR-182
t0023965	24	15	AACATTCATTGTTGTCGGTGGGAT	miRNA	miR-181b
t0024127	21	15	AACATTCAACGCTGTCGGTTA	miRNA	miR-181a
t0024170	24	15	AAGCGGCGATGGCGGAGCTGAATT	miRNA	miR-1636
t0024294	19	15	CCAATATTACTGTGCTGCT	miRNA	miR-16-2*
t0024248	24	15	GGGGCGGGGAGCGGTCGGGCGGCG	miRNA	miR-1607
t0023777	22	15	CTGGGGGCGGGGAGCGGTCGGG	miRNA	miR-1607
t0023962	19	15	TAGCAGCACGTAAATATTC	miRNA	miR-16
t0024238	20	15	CAGCAGCACGTAAATATTGG	miRNA	miR-16
t0024173	20	15	TAGTAGCACGTAAATATTGG	miRNA	miR-16
t0023745	22	15	ATAGCAGCACGTAAATATTGGA	miRNA	miR-16
t0023941	18	15	GCAGCACGTAAATATTGG	miRNA	miR-16
t0024194	22	15	TAGCAGCACATCATGGTTTACT	miRNA	miR-15b
t0024345	22	15	TAGCAGCGCATCATGGTTTACA	miRNA	miR-15b
t0023841	19	15	TAGCAGCACATCATTGTTT	miRNA	miR-15b
t0024275	25	15	TAGCAGCACATCATGGTTTACAATA	miRNA	miR-15b
t0023684	19	15	TAGCAGCACATCGTGGTTT	miRNA	miR-15b
t0023696	21	15	TAGCGGCACATAATGGTTTGT	miRNA	miR-15a
t0023906	20	15	GCAGCACATAATGGTTTGTG	miRNA	miR-15a
t0024209	21	15	TAGCAGCACATAATGCTTTGT	miRNA	miR-15a
t0023726	20	15	TAGCACCACATAATGGTTTG	miRNA	miR-15a
t0023808	22	15	TCAGTGCATCACAGAACTTTGA	miRNA	miR-148b-3p
t0023857	21	15	GGATATCATCATATATTGTAA	miRNA	miR-144*
t0024012	23	15	CTACAGTATAGATGATGTACTTT	miRNA	miR-144
t0024097	20	15	CCCATAAAGTAGAAAGAACT	miRNA	miR-142
t0024014	18	15	CCATAAAGTAGAAAGCTA	miRNA	miR-142
t0024056	25	15	TACCACAGGGTAGAACTACGGAAAA	miRNA	miR-140
t0024087	23	15	TACCACAGGGTAGGACCACGGAA	miRNA	miR-140
t0024222	24	15	TACCACAGGGTAGAACCCCGGAAA	miRNA	miR-140
t0024259	21	15	ACCACAGGGTACAACCACGGA	miRNA	miR-140
t0024063	23	15	TACCACAGGGTAGAACCACGGCG	miRNA	miR-140
t0024188	20	15	ACTCGGCGTGGCGTCGGGCG	miRNA	miR-1307
t0024258	25	15	TCTGGGCAACAAAGTGAGACCTTAA	miRNA	miR-1285
t0024342	21	15	AGGATGAGCAAAGAAAGTAGA	miRNA	miR-1255a
t0023781	23	15	CTTCCCCACCCTCTCCTGCAGAA	miRNA	miR-1224-3p
t0023763	24	15	TAAAGTGCTGACAGTGCAGAAAAA	miRNA	miR-106b
t0023939	20	15	AGCAGCCTTGTACAGGGCTA	miRNA	miR-103a
t0024249	23	15	AGCAGCATTGTGCAGGGCTATGA	miRNA	miR-103a
t0024333	22	15	AGCAGCATTGTACAGGGCTAAG	miRNA	miR-103a
t0023681	23	15	GTACAGTACTGTGATAACTAAGA	miRNA	miR-101c
t0023923	21	15	GGACAGTACTGTGATAACTGA	miRNA	miR-101c
t0023926	22	15	GTACAGTACTATGATAACTGAA	miRNA	miR-101b
t0023928	21	15	GTACAGTACTGTGATAAATGA	miRNA	miR-101
t0024163	22	15	TACAGTATTGTGATAACTGAAT	miRNA	miR-101
t0024215	21	15	TACAGTACTGTAATAACTGAA	miRNA	miR-101
t0023785	20	15	TACAGTACTGGGATAACTGA	miRNA	miR-101
t0024300	18	15	TGAGGTAGTAGATTGTTA	miRNA	let-7j
t0023869	23	15	TTGAGGTAGTAGATTGTATAGTT	miRNA	let-7f
t0023872	22	15	GGTAGTAGATTGTATAGTTATC	miRNA	let-7f
t0023815	23	15	TGAGTTAGTAGATTGTATAGTTA	miRNA	let-7f
t0024203	20	15	TGAGGTAGTAGATTGTAGAG	miRNA	let-7f
t0024274	23	15	TGAGGAAGTAGATTGTATAGTTG	miRNA	let-7f
t0024086	23	15	TGAGGTAGTAGATTGTATATTTA	miRNA	let-7f
t0024109	22	15	AGAGGTAGTAGGTTGCATAGCT	miRNA	let-7d
t0023990	20	15	AGAGGTAGTAGGTTGCATAA	miRNA	let-7d
t0023819	25	15	TGAGGTACTAGGTTGTATGGTTATC	miRNA	let-7c

t0023910	19	15	TGAGGTAGTAGGTTTGGTT	miRNA	let-7a
t0023969	21	15	TGAGGTAGTAGGTTGTATAAT	miRNA	let-7
t0024181	22	15	TGAGCTAGTAGGTTGTATAGTT	miRNA	let-7
t0024051	20	14	TTGCACTTGTCCCGGCCTGT	miRNA	miR-92c
t0023701	22	14	TATTGCACTTGTCCCGGCCATT	miRNA	miR-92b
t0023730	24	14	ATTCTAATTTCTCCACGTCTTTGT	miRNA	miR-576-5p
t0023877	19	14	GCGAGGGGCCCGGGGCGGG	miRNA	miR-5126
t0023889	23	14	AAACAAACATGGTGCACTTCTTT	miRNA	miR-495
t0024068	23	14	TCCTGTACTGAGCTGCCCCAAGA	miRNA	miR-486-5p
t0024220	26	14	TCCTGTACTGAGTTGCCCCGAGAAGA	miRNA	miR-486-5p
t0023932	21	14	TCCTGGACTGAGCTGCCCCGA	miRNA	miR-486-5p
t0024114	22	14	TCCTGTACTGAGGTGCCCCGAG	miRNA	miR-486-5p
t0023790	23	14	TCCTGTACTGGGCTGCCCCGAGA	miRNA	miR-486-5p
t0024135	19	14	AGAAGGGTCGGGGCGGATC	miRNA	miR-4516
t0023836	19	14	CGGGGGGAGAAGGGTCGGG	miRNA	miR-4516
t0024196	19	14	TGAGGGAGTAGGTTGTGTG	miRNA	miR-4510
t0024979	23	14	AAACCGTTACCATCACTGAGTTT	miRNA	miR-451
t0024472	21	14	ACACCGTTACCATTACTGAGT	miRNA	miR-451
t0024948	22	14	AAACCGTTACCATTACTGTGTT	miRNA	miR-451
t0024373	24	14	CGTTACCATTACTGAGTAAACAAA	miRNA	miR-451
t0024429	34	14	AAACCGTTACCATTACTGAGAACTTTGAG	miRNA	miR-451
t0024578	20	14	AAACCGGTACCATTACTGAG	miRNA	miR-451
t0024618	25	14	AAACCGTTACCATTACTGAGTTAAA	miRNA	miR-451
t0024793	23	14	AAACCGTTACCACTACTGAGTTT	miRNA	miR-451
t0024836	21	14	TTCGGGGTCTGGGCGCGGTTA	miRNA	miR-4508
t0024923	20	14	AACAGCATTGTACAGGGCTA	miRNA	miR-4289
t0024573	24	14	AATGACACGATCACTCCCGTTGAT	miRNA	miR-425
t0024742	23	14	TGAGGGGAAGAGAGCGAGAATTT	miRNA	miR-423-5p
t0024376	21	14	TGAGGCAGAGAGCGAGACTTT	miRNA	miR-423-5p
t0024478	23	14	TGAGGGGCAGGCAGCGAGACTTT	miRNA	miR-423-5p
t0024497	20	14	TGAGGGGCAGAGAGCCAGAC	miRNA	miR-423-5p
t0024549	24	14	TGAGGGGAAGAGAGCGAGACTTTT	miRNA	miR-423-5p
t0024665	24	14	TTTGTTCGTTCGGCTCGCGTGATC	miRNA	miR-375
t0024784	22	14	TTTCCTACCCTACCTGAAGACT	miRNA	miR-3685
t0024826	28	14	AACTTTGAAGACTGAAGTGGAGAAGGGG	miRNA	miR-3526
t0024852	23	14	GGCTCTGTAGCTCAGTAGGTATC	miRNA	miR-3488
t0024888	23	14	AAAAGCTGGGTTGAGCGGGCGTA	miRNA	miR-320d
t0024452	22	14	AAAAGCTGGGTTGAGAGGCCGA	miRNA	miR-320d
t0024535	21	14	AAAAGCTGGGTTGAGAGGGGG	miRNA	miR-320d
t0024550	23	14	AAAAGCTGGGTTGAGAGGACGAA	miRNA	miR-320d
t0024483	22	14	ATAAGCTGGGTTGAGAGGGCGG	miRNA	miR-320d
t0024837	22	14	CAAAGCTGGGTTGAGAGGGCGA	miRNA	miR-320a
t0024876	23	14	AAAAGCTGGGTTGAGACGGCGAT	miRNA	miR-320a
t0024902	22	14	AAAACCTGGGTTGAGAGGGCGA	miRNA	miR-320a
t0024954	20	14	ATATTGCACATTACTAAGTT	miRNA	miR-32
t0024704	23	14	TTTTCAGTCGGATGTTTACAGCA	miRNA	miR-30e*
t0024958	26	14	TTCAAGTAATCCAGGATAGGCTTATC	miRNA	miR-26a
t0024658	21	14	CATTGCACTTGTCTAGGTCTG	miRNA	miR-25
t0024759	22	14	CATTACACTTGTCTCGGTCTGA	miRNA	miR-25
t0024450	21	14	TGACTGGGGCGGAACATCTGT	miRNA	miR-219-2-3p
t0024667	25	14	TAGCTTATCAGACTGGTGTTGGATC	miRNA	miR-21
t0024709	25	14	TGGAATGTAAAGAAGTGTGTGGATC	miRNA	miR-206
t0024725	22	14	TGTGCAAATCCATGCAAAACTA	miRNA	miR-19b
t0024924	23	14	CAACGGAATACCAAAAGCAGCTG	miRNA	miR-191
t0024574	21	14	CGGCGGGGACGGCGATTGGAA	miRNA	miR-1908
t0024904	22	14	TGGAGAGAAAGGAAGTTCCTGT	miRNA	miR-185
t0024992	21	14	TGGAGAGAAAGGCAGTTCGTG	miRNA	miR-185
t0024803	18	14	ATGGAGAGAAAGGCAGTT	miRNA	miR-185

t0024975	23	14	TCGAGAGAAAGGCAGTTCCTGAA	miRNA	miR-185
t0024617	23	14	TGGAGAGAAAGGCAGTTACTGAT	miRNA	miR-185
t0024541	27	14	ATGGCACTGGTAGAATTCACTTCGTAT	miRNA	miR-183
t0024679	24	14	TATGGCACTGGTAGAATTCACATC	miRNA	miR-183
t0024960	24	14	AACATTCATTGTTGTCGGTGGGTA	miRNA	miR-181d
t0024671	25	14	AACATTCAACGCTGTCGGTGAGTTT	miRNA	miR-181a
t0024942	22	14	AACATTCAACGCTGTCGGGGAG	miRNA	miR-181a
t0024806	26	14	ATAGCAGCACGTAAATATTGGCGAAA	miRNA	miR-16
t0024827	22	14	TAGCAGCAAATCATGGTTTACA	miRNA	miR-15b
t0024833	22	14	TAGCAGCACATCGTGGTTTACA	miRNA	miR-15b
t0024486	22	14	TAGCAGCACCTCATGGTTTACA	miRNA	miR-15b
t0024597	22	14	TAGCGGCACATCATGGTTTACA	miRNA	miR-15b
t0024712	22	14	TAGCAGCACATAATGGTTTGTC	miRNA	miR-15a
t0024730	19	14	TAGCAGCACACAATGGTTG	miRNA	miR-15a
t0024769	20	14	TAGCAGCACCTAATGGTTTG	miRNA	miR-15a
t0024533	23	14	TGAGAACTGAATTCCATAGGCAA	miRNA	miR-146b-5p
t0024814	21	14	GTATTGTTTCCTACTTTATGG	miRNA	miR-142-3p
t0024656	19	14	AGTGTTTCCTACTTTATGG	miRNA	miR-142*
t0024788	20	14	CCCATAAAGAAGAAAGCACT	miRNA	miR-142
t0024790	24	14	TACTACAGGGTAGAACCACGGACA	miRNA	miR-140
t0024818	24	14	TACCACAGCGTAGAACCACGGAAT	miRNA	miR-140
t0024538	25	14	TACCACAGGGTAGAACCACGGAATT	miRNA	miR-140
t0024566	22	14	TACCACTGGGTAGAACCACGGA	miRNA	miR-140
t0024642	25	14	TACCACAGGGTAGAACCACGGAAGT	miRNA	miR-140
t0024821	23	14	TACCACAGGGTAGAACCACGTAC	miRNA	miR-140
t0024757	21	14	ACCACAGGGTCGAACCACGGA	miRNA	miR-140
t0024914	28	14	ACCACAGGGTAGAACCACGGACAAAAAA	miRNA	miR-140
t0024956	22	14	ACCACAGGGCAGAACCACGGAA	miRNA	miR-140
t0024594	23	14	TGCCACAGGGTAGAACCACGGAA	miRNA	miR-140
t0024423	22	14	CAGTGCAATGTTTAAAGGGCAT	miRNA	miR-130a
t0024404	21	14	TCTGGGCAACAAAGTGAGACG	miRNA	miR-1285
t0024877	24	14	TCCCTGAGCCCTTTAACCTGTATC	miRNA	miR-125a-5p
t0024394	23	14	AGGATGAGCAAAGAAAGTAGATT	miRNA	miR-1255a
t0024463	21	14	GTGAGGACTCGGGAGGGGGAA	miRNA	miR-1224
t0024514	20	14	AAAGTGCTGACAGTGCAGAT	miRNA	miR-106b
t0024521	23	14	AGCAGCATTGTCCAGGGCTATGA	miRNA	miR-103a
t0024559	23	14	AGCAGCATTGTACAGGGCTAAGA	miRNA	miR-103a
t0024696	21	14	AGCAGCATTGTACAGCGCTAT	miRNA	miR-103a
t0024792	23	14	AGCAGCATTGTACAGAGCTATGA	miRNA	miR-103a
t0024797	22	14	AGCAGCATTGTACCGGGCTATG	miRNA	miR-103a
t0024990	21	14	AGCAGCATTGTACAGGGCTGT	miRNA	miR-103a
t0025003	21	14	AGCAGCATTGTACAGGGGTAT	miRNA	miR-103a
t0024607	20	14	AGCAACATTGTACAGGGCTA	miRNA	miR-103a
t0024507	23	14	AGCAGCACTGTACAGGGCTATGA	miRNA	miR-103a
t0024359	22	14	GGACAGTACTGTGATAACTGAA	miRNA	miR-101c
t0024898	23	14	GTACAGTACTGTGATAACTGAAC	miRNA	miR-101
t0024493	22	14	TACAGTACTTTGATAACTGAAG	miRNA	miR-101
t0024687	22	14	TACAGTACTGTGATAACTGTAA	miRNA	miR-101
t0024590	22	14	TGAGGTAGTAGTTTGTGGTGTT	miRNA	let-7i
t0024601	19	14	TGAGGTAGTAGTTTGCGCT	miRNA	let-7i
t0024689	22	14	TGAGGTAGTAGTTTGTGATGTT	miRNA	let-7i
t0024702	26	14	TGAGGTAGTAGTTTGTACAGTTTATC	miRNA	let-7g
t0024841	22	14	TGAGGTAGTAGTTTGTACTGTT	miRNA	let-7g
t0024919	21	14	TGAGGTAGTAGTTTGTACCGT	miRNA	let-7g
t0024946	21	14	TGAGGTAGTAGATTGTAGTTG	miRNA	let-7f-5p
t0024962	19	14	TGAGGTAGTAGATTGTGTA	miRNA	let-7f
t0024987	23	14	TGAGGTAGTAGATTGTGTAGTTA	miRNA	let-7f
t0024413	19	14	TGAGGTAGTAGCTTGTATA	miRNA	let-7f

t0024635	26	14	TGAGGTAGTAGATTGTATAGTTACGT	miRNA	let-7f
t0024932	21	14	TGAGGTGTTAGATTGTATAGT	miRNA	let-7f
t0024967	22	14	TGAGGTAGTAGATTGTGTATTT	miRNA	let-7f
t0024624	19	14	TGGGGTAGTAGATTGTATA	miRNA	let-7f
t0024690	21	14	GAGGTAGTAGGTTGTGTGGTA	miRNA	let-7b
t0024955	22	14	TGAGGTAGTAGATTGTGTGGTA	miRNA	let-7b
t0024763	24	14	TGAGGTAGTAGGTTGTATAAAAGT	miRNA	let-7-5p
t0024785	20	14	TGAGGTAGTAGGTTGTATCG	miRNA	let-7
t0024815	18	14	TGTAGTAGGTTGTATAGT	miRNA	let-7
t0024660	24	14	TGAGGTAGTAGGTTGTATAGTTGC	miRNA	let-7
t0024416	19	14	TGGGGTAGTAGGTTGTATA	miRNA	let-7
t0024611	18	14	GAGGTAGTAGGTTGTATA	miRNA	let-7
t0024634	21	13	TTCTAGTAAGAGTGGCAGTCG	miRNA	miR-628-3p
t0024649	20	13	CCTCTTTTCTTTGAGACTCA	miRNA	miR-627
t0024807	19	13	CGGGAAGGCATTCTGATAT	miRNA	miR-541
t0024842	23	13	TGTACTGAGTTGCCCCGAAGGCG	miRNA	miR-486-5p
t0024966	29	13	TCCTGTACTGAGCTGCCCCGAGACGGCCC	miRNA	miR-486-5p
t0024777	22	13	ACCTGTACTGAGCTGCCCCGAG	miRNA	miR-486-5p
t0024848	23	13	TCCTATACTGAGCTGCCCCGAGA	miRNA	miR-486-5p
t0024558	22	13	TCCTGTACTTAGCTGCCCCGAA	miRNA	miR-486-5p
t0024529	21	13	TAGAGCAGGGAGCAGGAAGCT	miRNA	miR-4732-5p
t0024542	26	13	GAGAGAAGGGTCGGGGCGGCAGGGGC	miRNA	miR-4516
t0024636	22	13	AGAAGGGTCGGGGCGGCAGGTT	miRNA	miR-4516
t0024738	24	13	TGAGGGAGTAGGTTGTGTGGTTAT	miRNA	miR-4510
t0024978	20	13	AAACTTTACCATTACTGAGT	miRNA	miR-451
t0025231	22	13	TAACCGTTACCATTACTGAGTT	miRNA	miR-451
t0025194	23	13	AAACCGTTAGCATTACTGAGTTT	miRNA	miR-451
t0025627	20	13	GAGAAACCGTTACCATTACT	miRNA	miR-451
t0025228	24	13	AAACCGTTAACATTACTGAGTTTA	miRNA	miR-451
t0025370	24	13	AAACCGTTACCATTATTGAGTTTA	miRNA	miR-451
t0025495	20	13	AAACCGTTACCACTACTGAG	miRNA	miR-451
t0025520	24	13	AGTTACCATTACTGAGTTAAACCA	miRNA	miR-451
t0025616	22	13	TCGCATCCGAGTCACGGCACCA	miRNA	miR-4454
t0025585	22	13	TGAGGGGCAGAGAGCGAAACTT	miRNA	miR-423-5p
t0025226	23	13	GAGGGGCAGAGAGCGAGACTTTT	miRNA	miR-423-5p
t0025281	22	13	TGAGGGGCCGAGAGCGAGACTT	miRNA	miR-423-5p
t0025499	19	13	TGAGGGGCAGATAGCGAGA	miRNA	miR-423-5p
t0025041	21	13	TGAGGGGCAGAAAGCGAGATT	miRNA	miR-423-5p
t0025070	24	13	TGAGGGGCAGAGAGCGAGACTCGC	miRNA	miR-423-5p
t0025078	23	13	TGGACTCGCAGGAACAGGCCTTA	miRNA	miR-4001b-5p
t0025131	19	13	ATCGTAGAGGAAAATCCAC	miRNA	miR-376a
t0025233	20	13	GGGTGGATCACGATGCAATT	miRNA	miR-363*
t0025339	18	13	GGGGTGGGGTCGGCGGGG	miRNA	miR-3621
t0025387	27	13	CCTTGAGGACTGATGTGGAGAAGGGTT	miRNA	miR-3526
t0025620	22	13	GTGGAATCCAGGACGCGCGTTT	miRNA	miR-3503
t0025436	22	13	TCTCACACAGAAATAGCACCCG	miRNA	miR-342-3p
t0025182	22	13	TCTCACACAGAAATCGAACCCG	miRNA	miR-342-3p
t0025340	22	13	AAAAGCTGGGTTAAGAGGGCGT	miRNA	miR-320d
t0025412	22	13	AAAAGCTGGGTTGAGAGGCGAT	miRNA	miR-320d
t0025446	25	13	AAAAGCTGGGTTGAGAGGGCGTTTT	miRNA	miR-320d
t0025461	22	13	AAAAGCCGGGTTGAGAGGGCGT	miRNA	miR-320c
t0025536	23	13	AGAAGCTGGGTTGAGAGGGCGAA	miRNA	miR-320a
t0025459	23	13	AAAAGCTGGGTTGAGAGAGCGAA	miRNA	miR-320a
t0025090	25	13	CGAAAAGCTGGGTTGAGAGGGCGAA	miRNA	miR-320a
t0025330	23	13	AAAAGCTGGGTTAAGAGGGCGAA	miRNA	miR-320a
t0025264	21	13	TGGAAGGTAGACGGCCAGAGT	miRNA	miR-3190
t0025466	19	13	CGCGGGTCGGGGGCGGGG	miRNA	miR-2981
t0025580	22	13	CATTGCACTTGTCTCGGTCTAA	miRNA	miR-25

t0025127	21	13	ATTGCACTTGTCTCGGTCTGT	miRNA	miR-25
t0025565	22	13	GGCTCAGTTCAGCAGGAACAGA	miRNA	miR-24b
t0025155	21	13	CTAAGGGCTGGGTCGGTCGGG	miRNA	miR-2487
t0025223	23	13	TGTCAGTTTGTCAAATACCCCAT	miRNA	miR-223
t0025460	22	13	AGTGAGATTGTTGCATATTTAT	miRNA	miR-2162-5p
t0025624	24	13	TGAATGTAAAGAAGTATGTATATC	miRNA	miR-1a
t0025040	24	13	ATGACCTATGAATTGACAGCCATC	miRNA	miR-192
t0025214	23	13	CAACGGAATCCCAAAAGCAGCGG	miRNA	miR-191
t0025440	22	13	CAACGGCATCCCAAAAGCAGCT	miRNA	miR-191
t0025594	23	13	AACGGAATCCCAAAAGCCGCTGA	miRNA	miR-191
t0025365	23	13	TGGAGGGAAAGGCAGTTCCTGAT	miRNA	miR-185
t0025555	21	13	TGGAGAGAAAGGCAGTACCTG	miRNA	miR-185
t0025187	23	13	AGACCTACTTATCTACCAACAGC	miRNA	miR-1839-3p
t0025326	22	13	AAGGTAGATAGAACAGGGCTTG	miRNA	miR-1839
t0025081	21	13	TGAATTACCGAAGGGCCATAA	miRNA	miR-183*
t0025442	32	13	TATGGCACTGGTAGAATTCACTAATCCCA	miRNA	miR-183
t0025622	22	13	TGAGGGAGTAGATTGTATACTT	miRNA	miR-1827
t0025498	23	13	AACATTCATTGTTGTCGGTGGGA	miRNA	miR-181b
t0025193	23	13	GCTGGGGGCGGGGAGCGGTCGGG	miRNA	miR-1607
t0025046	21	13	AGCAGCACGTAAATATTGTCG	miRNA	miR-16
t0025096	26	13	AATCTAGCAGCACGTAAATATTGGCG	miRNA	miR-16
t0025417	19	13	TAGTAGCACATCATGGTTT	miRNA	miR-15b
t0025546	19	13	TGGCAGCACATCATGGTTT	miRNA	miR-15b
t0025093	22	13	TAGCAACACATCATGGTTTACA	miRNA	miR-15b
t0025375	22	13	TAGCAGCACATAATGGTTTGAA	miRNA	miR-15a
t0025252	21	13	TGGCAGCACATAATGGTTTGT	miRNA	miR-15a
t0025050	18	13	TAGCAGCACATAATGGTG	miRNA	miR-15a
t0025064	21	13	TAGCAACACATAATGGTTTGT	miRNA	miR-15a
t0025389	20	13	TGGCAGCACATAATGGTTTG	miRNA	miR-15a
t0025065	18	13	TCTCCCAACCCTTGTACC	miRNA	miR-150
t0025366	18	13	ACAGTATAGATGATGTAT	miRNA	miR-144
t0025348	22	13	TACAGTATAGATGATGTACATT	miRNA	miR-144
t0025301	26	13	TACAGTATAGATGATGTACTAAAAAA	miRNA	miR-144
t0025316	21	13	ACAGTATAGATGATGTACTAA	miRNA	miR-144
t0025008	23	13	TGAGATGAAGCACTGTAGAGATC	miRNA	miR-143
t0025016	21	13	CATAAAGTAGAAAGCACTACA	miRNA	miR-142-5p
t0025120	22	13	GCCACAGGGTAGAACCACGGAA	miRNA	miR-140
t0025057	22	13	ACCGCAGGGTAGAACCACGGAC	miRNA	miR-140
t0025119	21	13	TACCACAGGGTAGAACCAAGG	miRNA	miR-140
t0025144	24	13	TACCACAGGATAGAACCACGGACA	miRNA	miR-140
t0025229	22	13	ACCACAGGGTAGAACCACGGCG	miRNA	miR-140
t0025434	21	13	ACCTCAGGGTAGAACCACGGA	miRNA	miR-140
t0025066	22	13	TACCACAGGGTAGACCCACGGA	miRNA	miR-140
t0025068	23	13	TACCACAGGGTAGAAACAAGGAC	miRNA	miR-140
t0025312	22	13	ACCACGGGGTAGAACCACGGAC	miRNA	miR-140
t0025323	22	13	ACCATAGGGTAGAACCACGGAA	miRNA	miR-140
t0025369	21	13	CCCACAGGGTAGAACCACGGA	miRNA	miR-140
t0025587	20	13	TTTCCGGCTCGCGTGGGGGT	miRNA	miR-1180
t0025077	20	13	CAGCATTGTACAGGGCTAGA	miRNA	miR-107
t0025029	20	13	CCGCACTGTGGGGACTTGCT	miRNA	miR-106b*
t0025105	22	13	AGCCGCATTGTACAGGGCTATG	miRNA	miR-103a
t0025121	23	13	AGCAGCATTGTACAGGGCTACGA	miRNA	miR-103a
t0025163	22	13	AGCAGCATTGTCCAGGGCTATG	miRNA	miR-103a
t0025405	23	13	AGCAGCATTGTACAGGGCTGTGA	miRNA	miR-103a
t0025420	22	13	AGCAGCATTGTACAGGGCTACG	miRNA	miR-103a
t0025482	22	13	AGCACCATTGTACAGGGCTATG	miRNA	miR-103a
t0025494	23	13	AGCAACATTGTACAGGGCTATGA	miRNA	miR-103a
t0025511	20	13	AGCAGCGTTGTACAGGGCTA	miRNA	miR-103a

t0025514	20	13 AGCAGCATTGCACAGGGCTA	miRNA	miR-103a
t0025605	20	13 AGCAGCATTGTACAGAGCTA	miRNA	miR-103a
t0025266	18	13 CAGTACTGTGATAACTGA	miRNA	miR-101c
t0025322	22	13 GCACAGTACTGTGATAACTGAA	miRNA	miR-101c
t0025506	21	13 TACAGTACTGTGATAACTGGA	miRNA	miR-101
t0025019	22	13 GTACAGGACTGTGATAACTGAA	miRNA	miR-101
t0025164	22	13 GTACAGTACCGTGATAACTGAA	miRNA	miR-101
t0025224	21	13 TACAGTACTGTGGTAACTGAA	miRNA	miR-101
t0025250	21	13 GTACAGTACTGTGATAACTTA	miRNA	miR-101
t0025374	25	13 ACCCTGTAGATCCGAATTTGTGATC	miRNA	miR-10
t0025458	23	13 TGAGGGAGTAGTTTGTGCTGTTA	miRNA	let-7i
t0025474	22	13 TGAGGTAGTCGTTTGTACAGTT	miRNA	let-7g
t0025478	22	13 TGAGGTACTAGTTTGTACAGTT	miRNA	let-7g
t0025554	21	13 TGGGGTAGTAGTTTGTACAGT	miRNA	let-7g
t0025619	22	13 TGTGGTAGTAGTTTGTACAGTT	miRNA	let-7g
t0025122	21	13 TGAGGTAGTAGACTTTATAGT	miRNA	let-7f
t0025177	26	13 GTTCTGAGGTAGTAGATTGTATAGTT	miRNA	let-7f
t0025091	23	13 TGAGGTAGTAGATTGCATAGTTG	miRNA	let-7f
t0025167	18	13 TGAGGTAGTAGATTGTCG	miRNA	let-7f
t0025171	24	13 ATGAGGTAGTAGATTGTATAGTTT	miRNA	let-7f
t0025328	19	13 TGAGGTAGTAGTTTGTATA	miRNA	let-7f
t0025439	23	13 TGGGGTAGTAGATTGTATAGTTG	miRNA	let-7f
t0025085	23	13 TGAGGTAGTAGATTGTATAGGTG	miRNA	let-7f
t0025075	21	13 AGAGGTAGTAGGTTGCATAAT	miRNA	let-7d
t0025060	22	13 AGAGGTATTAGGTTGCATAGTT	miRNA	let-7d
t0025165	21	13 AGAGGTAGTAGGTTACATAGT	miRNA	let-7d
t0025261	18	13 TGAGGTAGTAGGTTGTGG	miRNA	let-7b
t0025489	20	13 TGAGGTAGTGGGTTGTGTGG	miRNA	let-7b
t0025143	20	13 TGAGGTGGTAGGTTGTGTGG	miRNA	let-7b
t0025179	20	13 TGAGGTAGTAGGCTGTGTGG	miRNA	let-7b
t0025237	25	13 TGAGGTAGTAGGTTGTATAGTAATC	miRNA	let-7-5p
t0025253	25	13 TGAGGTATTAGGTTGTATAGTTATC	miRNA	let-7
t0025307	22	13 GGAGGTAGTAGGTTGTATAGTT	miRNA	let-7
t0025481	20	13 TGAGGTAGTAGGTTGTCTAG	miRNA	let-7
t0025548	22	12 CAAAGTGCTGTTCGTGCCGGTA	miRNA	miR-93
t0025614	22	12 CAAAGTGCTGTTCGTGCAGGCA	miRNA	miR-93
t0025017	20	12 AGGGACGGGACGCGGTGCAT	miRNA	miR-92b*
t0025356	22	12 TATTGCACTTGTCACGGCCTGT	miRNA	miR-92a
t0025609	22	12 TGGAGAGAAAGGAAGTTCATGA	miRNA	miR-765
t0025054	25	12 AGGGCGCGCGGGTCGGGGCGGCGGA	miRNA	miR-638
t0025218	24	12 GAGGGCGCGCGGGTCGGGGCGGCG	miRNA	miR-638
t0025379	21	12 AGGGCGCGCGGGTCGGGGCGG	miRNA	miR-638
t0025551	26	12 AGGGCGCGCGGGTCGGGGCGGCGGCG	miRNA	miR-638
t0025094	20	12 TCTAGTAAGAGTGGCAGGCG	miRNA	miR-628-3p
t0025290	21	12 GACTATAGAACTTTTCCCCTT	miRNA	miR-625*
t0025547	18	12 CAAAAGTGATCGTGGTTG	miRNA	miR-548t
t0025595	23	12 TCCTGTGCTGAGCTGCCCCGAGA	miRNA	miR-486-5p
t0026006	22	12 TCCTGTACTGAGCTGCCCCGCA	miRNA	miR-486-5p
t0026290	22	12 CCTGTACTGAGCTGCCCCGAGT	miRNA	miR-486-5p
t0025977	21	12 TCCTGTACTGAGCGGCCCCGA	miRNA	miR-486-5p
t0025756	23	12 TCCTGCACTGAGCTGCCCCGAGA	miRNA	miR-486-5p
t0025844	22	12 TCCTGTATTGAGCTGCCCCTAG	miRNA	miR-486-5p
t0025636	20	12 GATGTAGGATTGTGTCGGCA	miRNA	miR-4860
t0025984	23	12 TGTAGAGCAGGGGGCAGGAAGCT	miRNA	miR-4732-5p
t0026143	20	12 AGAGAAGGGTCGGGGCGGTT	miRNA	miR-4516
t0026213	25	12 AATTCGTTACCATTACTGAGTTTAG	miRNA	miR-451
t0025885	20	12 AAACCGTTACCCTTACTGAG	miRNA	miR-451
t0025905	20	12 AAACCGTTACCATTGCTGAG	miRNA	miR-451

t0025896	21	12	AAACGTTACCATTACTGAGTT	miRNA	miR-451
t0025728	21	12	AACCCGTTACCATTACTGAGT	miRNA	miR-451
t0025944	20	12	GGGTGCGGGCCGGCGGGGTC	miRNA	miR-4466
t0025955	21	12	TCGTATCCGAGTCACGGCACC	miRNA	miR-4454
t0026007	21	12	GCAGGACAGGCAGAAGTGGAT	miRNA	miR-4436a
t0026009	21	12	AACAGCATTGTACAGGGCTAT	miRNA	miR-4289
t0026122	20	12	CAGCAGCAATTCATGTTTGA	miRNA	miR-424
t0025725	22	12	TGAGGCGCAGAGAGCGAGACTT	miRNA	miR-423-5p
t0025748	22	12	TGAGGGGCAGAGAGCGAGTCTT	miRNA	miR-423-5p
t0026114	23	12	TGAGGGGCAGAGAGCGAGACATT	miRNA	miR-423-5p
t0025641	26	12	TGAGGGGCAGAGAGCGAGACTTTTCT	miRNA	miR-423-5p
t0025811	21	12	TGAGGGGCAGAGAGCGAAACT	miRNA	miR-423-5p
t0025914	20	12	TGAGTGGCAGAGAGCGAGAC	miRNA	miR-423-5p
t0026044	26	12	ACTGGACTTGGAGTCAGAAGGCAATC	miRNA	miR-378
t0026188	22	12	TGAGGATGGATAGCAAGGAAGA	miRNA	miR-3605-5p
t0026246	27	12	AACTTTGAAGACTGAAGGGGAGAAGGG	miRNA	miR-3526
t0025664	23	12	TGAAGACTGAAGTGGAGCAGGGT	miRNA	miR-3526
t0025642	23	12	GAAGACTGAAGTGGAGAAGGGGT	miRNA	miR-3526
t0025730	22	12	AAAAACTGGGTTGAGAGGGCGT	miRNA	miR-320d
t0025881	22	12	AAAAGCTGGGATGAGAGGGCGT	miRNA	miR-320d
t0025686	22	12	AAAAGCTGGGTTGCGAGGGCGT	miRNA	miR-320d
t0025692	22	12	AAAAGCTGGGTTGAGAGGACGT	miRNA	miR-320d
t0025856	23	12	TGTGGAAGGTAGACGGCCAGAGA	miRNA	miR-3190
t0025998	22	12	TAGCACCATCTGAAATCGGTTT	miRNA	miR-29a
t0026156	18	12	TTCAAGTAATTCAGGATA	miRNA	miR-26b
t0026261	22	12	TGAGGGAGGAGGTTGTGTGGTT	miRNA	miR-265
t0026233	21	12	CATCGCACTTGTCTCGGTCTG	miRNA	miR-25
t0025789	21	12	CATTGCACTTATCTCGGTCTG	miRNA	miR-25
t0025976	23	12	TTGGGTTCCTGGCATGCTGATTT	miRNA	miR-23b*
t0025991	18	12	ATCACATTGCCAGGATTT	miRNA	miR-23a
t0026203	20	12	AAGCTGCCAGTTGAAGAATT	miRNA	miR-22-3p
t0025676	23	12	AACCTGGCATACAATGTAGATTT	miRNA	miR-221*
t0025778	21	12	TGACTGGGGCGGCACATCTGT	miRNA	miR-219-2-3p
t0025975	18	12	TGAGGTAGTATGTATAGT	miRNA	miR-1961
t0026035	22	12	AACGGAATCCCAAAAGAAGCTG	miRNA	miR-191
t0026172	22	12	CAAAGGAATCCCAAAAGCAGCT	miRNA	miR-191
t0025747	24	12	CAACGGAATCCCAAAAGAAGCTGA	miRNA	miR-191
t0026019	21	12	AACGGAATCCCAAAAGCCGCT	miRNA	miR-191
t0025719	21	12	CGGCGGGGACGGCGATTGGTC	miRNA	miR-1908
t0025947	21	12	TGATATGTTTGATATATTAGG	miRNA	miR-190
t0025956	23	12	TGGAGAGAAAGGCAGTTCCTAAA	miRNA	miR-185
t0026164	23	12	TGGGGAGAAAGGCAGTTCCTGAT	miRNA	miR-185
t0025957	23	12	TGGAGAGAAAGGGAGTTCCTGAT	miRNA	miR-185
t0025776	23	12	TGGAGAGAGAGGCAGTTCCTGAA	miRNA	miR-185
t0026077	23	12	TGGAGCGAAAGGCAGTTCCTGAA	miRNA	miR-185
t0026103	23	12	AAAGGTAGATAGAACAGGTTTTG	miRNA	miR-1839
t0025784	20	12	AACATTCATTGCTGTCGGTT	miRNA	miR-181b
t0025665	23	12	TCGGGGCGGCGGCGGCGGCGGGG	miRNA	miR-1777a
t0026046	18	12	ACTGCAGTGAAGGCACTT	miRNA	miR-17*
t0026090	18	12	AAGCGGCGATGGCGGAGC	miRNA	miR-1636
t0026228	27	12	TAGCAGCACGTAAATATTGGCGAAAAA	miRNA	miR-16
t0026068	20	12	TAGCAGCAAGTAAATATTGG	miRNA	miR-16
t0026130	20	12	TAGCAGCATGTAAATATTGG	miRNA	miR-16
t0025882	20	12	TAGCAGCGCGTAAATATTGG	miRNA	miR-16
t0025963	26	12	TAGCAGCACGTAAATATTGGCGGAAA	miRNA	miR-16
t0026265	23	12	TAGCAGCACGTAAATATTGGCGG	miRNA	miR-16
t0026267	21	12	ATAGCAGCACGTAAATATTGG	miRNA	miR-16
t0026286	24	12	TAGCAGCACGTAAATATTGGTGTA	miRNA	miR-16

t0026198	22	12 TAGCAGCACATCATGGCTTACA	miRNA	miR-15b
t0025860	20	12 TAGCCGCACATCATGGTTTA	miRNA	miR-15b
t0026273	20	12 TAGCACCACATCATGGTTTA	miRNA	miR-15b
t0025899	19	12 TAGCCGCACATCATGGTTT	miRNA	miR-15b
t0026291	19	12 TAGCAGCACCTCATGGTTT	miRNA	miR-15b
t0025673	21	12 TAGCAGCACATCATGGTTACC	miRNA	miR-15b
t0025741	19	12 TAGCAGCACATCATGGATT	miRNA	miR-15b
t0025775	19	12 TAGCAGCACATCACGGTTT	miRNA	miR-15b
t0025816	20	12 TAGCATCACATCATGGTTTA	miRNA	miR-15b
t0025827	18	12 TAGCAGCACAATATGGTT	miRNA	miR-15b
t0025961	22	12 TAGCAGCACACCATGGTTTACA	miRNA	miR-15b
t0026111	18	12 GCAGCACATAATGGTTTG	miRNA	miR-15a
t0026146	20	12 TAGCAGCACATAATGGTTTC	miRNA	miR-15a
t0025707	21	12 CAGCAGCACATAATGGTTTGT	miRNA	miR-15a
t0025713	20	12 TAGCAGCACATAATGATTTG	miRNA	miR-15a
t0025734	22	12 TAGCAGCACATAATGTTTTGTG	miRNA	miR-15a
t0025796	20	12 TAGCAGCACATAATAGTTTG	miRNA	miR-15a
t0025824	23	12 TCAGTGCATCACAGAACTTTGTT	miRNA	miR-148b-3p
t0025840	20	12 TACAGTATAGATGATGTCCT	miRNA	miR-144
t0025931	23	12 TTAGATGAAGCACTGTAGCTATC	miRNA	miR-143
t0025939	20	12 CATAAAGTAGAAAGCATTAC	miRNA	miR-142-5p
t0025965	20	12 CCCATAAAGTATAAAGCACT	miRNA	miR-142
t0026214	22	12 ACCACGGGGTAGAACCACGGAT	miRNA	miR-140
t0026281	23	12 TACCACAGGGTAAAACCACGGAA	miRNA	miR-140
t0025651	23	12 ACCACAGGGAAGAACCACGGACA	miRNA	miR-140
t0025685	22	12 ACCACAGGGTAGAACCACAGAT	miRNA	miR-140
t0025799	26	12 TACCACAGGGTAGAACCACGGAATTT	miRNA	miR-140
t0026195	22	12 ACCACAGGGTAGAACCAAGGAT	miRNA	miR-140
t0026264	24	12 TACCACAGGGTAGAGCCACGGACA	miRNA	miR-140
t0026275	21	12 AGCACAGGGTAGAACCACGGA	miRNA	miR-140
t0025722	23	12 TACCACAGAGTAGAACCACGGAA	miRNA	miR-140
t0026197	23	12 TACCAAAGGGTAGAACCACGGAC	miRNA	miR-140
t0026012	23	12 TACAACAGGGTAGAACCACGGAT	miRNA	miR-140
t0026082	23	12 TACCACAGGATAGAACCACGGAA	miRNA	miR-140
t0025731	23	12 TACCATAGGGTAGAACCACGGAT	miRNA	miR-140
t0025643	24	12 TACCACAGGGTAGAACCAAGGACG	miRNA	miR-140
t0025693	22	12 CAGTGCAATGTTAAAAGGGCCT	miRNA	miR-130a
t0025777	20	12 AGAGGCAGAGGTTGCAGTGG	miRNA	miR-1273f
t0025916	22	12 AGCAGCATTGTACAGGGCTATC	miRNA	miR-107
t0025986	23	12 AGCAGCATTGTACAGGACTATGA	miRNA	miR-103a
t0026001	21	12 TACAGTACTGTGAAAACTGAA	miRNA	miR-101
t0026137	28	12 GTACAGTACTGTGATAACTGAACGGGCT	miRNA	miR-101
t0026148	28	12 TACAGTACTGTGATAACTGAAGCGGTCG	miRNA	miR-101
t0026150	22	12 GTACAGTACTGTGATAACTGGA	miRNA	miR-101
t0026155	23	12 TTAGGTAGTAGTTTGTGCTGTTT	miRNA	let-7i
t0026170	23	12 TGAGGGAGTAGTTTGTGCTGTTT	miRNA	let-7i
t0026176	22	12 TGAGGTAGTAGTTTGTTCAGTT	miRNA	let-7g
t0026219	19	12 TGAGGTAGTAGATTTTATA	miRNA	let-7f
t0026225	18	12 TGAGGTAGTAGATTTTAG	miRNA	let-7f
t0026266	23	12 TAAGGTAGTAGATTGTATAGTTA	miRNA	let-7f
t0026238	26	12 TGAGGTAGTAGATTGTATAGTTGATC	miRNA	let-7f
t0026106	20	12 TGAGGTAGTATATTGTATAG	miRNA	let-7f
t0025663	24	12 TGAGGTAGTAGATTGTATAGTTTA	miRNA	let-7f
t0025805	19	12 TGAGGTAGTAGATCGTATA	miRNA	let-7f
t0025945	19	12 TGAGGTAGTAGATTGCATA	miRNA	let-7f
t0025972	23	12 CGAGGTAGTAGATTGTATAGTTG	miRNA	let-7f
t0026161	22	12 AGAGGTAGTATGTTGCATAGTT	miRNA	let-7d
t0025830	21	12 AGAGGTAGTAGGTTGCCTAGT	miRNA	let-7d

t0026282	20	12	AGGGGTAGTAGGTTGCATAG	miRNA	let-7d
t0025880	20	12	AGAGGTAGTACGTTGCATAG	miRNA	let-7d
t0025680	20	12	AGAGGTAGTAGGTTGCCTAG	miRNA	let-7d
t0025704	22	12	AGAGGTAGTAGGTTGCATAGTC	miRNA	let-7d
t0025868	22	12	AAAGGTAGTAGGTTGCATAGTT	miRNA	let-7d
t0025979	22	12	AGAGGTAGAAGGTTGCATAGTT	miRNA	let-7d
t0026027	22	12	TGAGGTAGTAGGTTGTACGGTT	miRNA	let-7c
t0026049	21	12	TGAGGTAGTAGCTTGTGTGGT	miRNA	let-7b
t0026064	22	12	TGAGGTAGTAGGTTGTGTGGCA	miRNA	let-7b
t0026076	20	12	TGAGGTAGTAGGTTATGTGG	miRNA	let-7b
t0026280	20	12	TGAGGTAGTAGATTGTGTGG	miRNA	let-7b
t0025656	25	12	TGAGGTAGTGGGTTGTGTGGTTATC	miRNA	let-7b
t0025708	22	12	GAGGTAGTAGGTTGTATAGTTG	miRNA	let-7
t0025813	19	12	TGAGGTAGTAGGTTGGTTA	miRNA	let-7
t0025845	18	12	TGAGGTAGTAGGTGGTAT	miRNA	let-7
t0025867	20	12	TGAGGTAGAAGGTTGTATAG	miRNA	let-7
t0025934	22	11	TATACAACTTACTACTTTCTTT	miRNA	miR-98*
t0026097	23	11	CAAAGTGCTGTTCGTGCAGGTCG	miRNA	miR-93
t0026136	26	11	AGGGCGCGTGGGTCGGGGCGGCGGCC	miRNA	miR-638
t0025780	21	11	TTCTAGTAAGAGTGGCAGGCG	miRNA	miR-628-3p
t0025791	22	11	TCTGGGCACAGGCGGATGTACA	miRNA	miR-5107
t0025836	22	11	TCCTGGACTGAGCTGCCCCGAG	miRNA	miR-486-5p
t0025887	22	11	TCCTGAACTGAGCTGCCCCGAG	miRNA	miR-486-5p
t0025941	21	11	TCCTGTACTGAGCTGCCGCGA	miRNA	miR-486-5p
t0025994	25	11	TCCTGTACTGAGCTGCCCCGAGATA	miRNA	miR-486-5p
t0025724	21	11	TCCTGTACTGAGGTGCCCCGA	miRNA	miR-486-5p
t0025823	20	11	TCCTGTACTGAGCAGCCCCG	miRNA	miR-486-5p
t0025915	22	11	GCCTGTACTGAGCTGCCCCGAG	miRNA	miR-486-5p
t0026032	28	11	TCCTGTACTGAGCTGCCCCGAGCAGATT	miRNA	miR-486-5p
t0026689	22	11	CGGGGCAGCTCAGTACAGGATT	miRNA	miR-486-3p
t0026620	21	11	TGTAGAGCAGGGAGAAGGAAG	miRNA	miR-4732-5p
t0026691	20	11	AGGGCTGGGTCGGTCGGGCT	miRNA	miR-4651
t0026602	28	11	AAGGGCTGGGTCGGTCGGGCTGGGGCGC	miRNA	miR-4651
t0026833	23	11	GAAGGGTCGGGGCGGCAGGGGCA	miRNA	miR-4516
t0026348	26	11	GGGAGAAGGGTCGGGGCGGCAGGGGC	miRNA	miR-4516
t0026438	22	11	AAACCGTAACCATTACTGAGTT	miRNA	miR-451
t0026478	22	11	AAACGGTTACCATTACTGAGTT	miRNA	miR-451
t0026587	23	11	AAACCGTTACCATTACTAAGTTT	miRNA	miR-451
t0026635	22	11	AACCGTTACCATTACTGAGTTC	miRNA	miR-451
t0026644	23	11	AAACCGGTACCATTACTGAGTTT	miRNA	miR-451
t0026683	25	11	AAACCGTTACCATTACCGAGTTTAG	miRNA	miR-451
t0026729	30	11	AAACCGTTACCATTACTGAGTTATACTTAC	miRNA	miR-451
t0026402	21	11	TAACCGTTACCATTACTGAGT	miRNA	miR-451
t0026422	21	11	AAAGCGTTACCATTACTGAGT	miRNA	miR-451
t0026796	21	11	GGGTGCGGGCCGGCGGGGTAA	miRNA	miR-4466
t0026906	23	11	AAAAGCTGGGCTGAGAGGGCGTA	miRNA	miR-4429
t0026429	22	11	AGAAGCATTGTACAGGGCTATT	miRNA	miR-4289
t0026830	23	11	TGAGGGGAAGAGAGAGAGACTTT	miRNA	miR-423-5p
t0026363	23	11	TGAGGGGCAGATAGCGATACTTT	miRNA	miR-423-5p
t0026446	21	11	TCAGGGGCAGAGAGCGAGACT	miRNA	miR-423-5p
t0026543	24	11	TGAGGGGCAGAGGGCGAGACTTTT	miRNA	miR-423-5p
t0026610	22	11	GGAGGGGCAGAGAGCGAGACTT	miRNA	miR-423-5p
t0026760	24	11	TGAGTGGCAGAGAGCGAGACTTTT	miRNA	miR-423-5p
t0026846	22	11	TCAGGTAGTAGTTTGTACAGTT	miRNA	miR-3962
t0026874	21	11	CGAGGTAGTAGTTTGTACAGT	miRNA	miR-3962
t0026897	23	11	ATTGGACTTGGAGTCAGAAGGCA	miRNA	miR-378
t0026911	22	11	ATATAATACAACCTGCTAAGGG	miRNA	miR-374
t0026915	18	11	ATTGCACGGTATCCATCT	miRNA	miR-363

t0026510	20	11	TTTCTCGGCTCCTCGCGGCT	miRNA	miR-3615
t0026696	20	11	TGTTGTACTTTTTTTTTTGT	miRNA	miR-3613-5p
t0026317	22	11	TCCTCGAACTGTTGTGGCCATT	miRNA	miR-3487
t0026327	23	11	TCTCACACAGAAATCGCACCCGG	miRNA	miR-342-3p
t0026377	22	11	AAAAGCTGGGTTGAGAAGGCGT	miRNA	miR-320d
t0026604	23	11	AAAATCTGGGTTGAGAGGGCGTT	miRNA	miR-320d
t0026754	23	11	GAAAGCTGGGTTGAGAGGGCGTA	miRNA	miR-320d
t0026817	23	11	AAAAGCTGGGTTGAGAGGGTGAT	miRNA	miR-320d
t0026321	22	11	AAAAGCTGGGTCGAGAGGGCGT	miRNA	miR-320d
t0026492	22	11	AAAAGCTAGGTTGAGAGGGCGT	miRNA	miR-320d
t0026798	23	11	AAACGCTGGGTTGAGAGGGCGAA	miRNA	miR-320a
t0026418	23	11	AAAAGCCGGGTTGAGAGGGCGAA	miRNA	miR-320a
t0026896	23	11	AAAAGCTGGGTTGCGAGGGCGAA	miRNA	miR-320a
t0026415	21	11	TTCAAGTAATTCAGGATAGGG	miRNA	miR-26b
t0026297	20	11	TCAAGTAATCCAGGATAGGT	miRNA	miR-26a
t0026795	21	11	CATTGCCCTTGTCTCGGTCTG	miRNA	miR-25
t0026747	24	11	TGTCAGTTTGTCAAATACCCCTAT	miRNA	miR-223
t0026385	23	11	GGGGAAACGGCCGCTGAGTGAGA	miRNA	miR-2110
t0026442	20	11	TAGCTTATCAGACTGATTTG	miRNA	miR-21
t0026588	22	11	TCTACTGTAGTATGGGCACTTT	miRNA	miR-20b*
t0026662	23	11	CTGACCTATGAATTGACAGCCAG	miRNA	miR-192
t0026758	23	11	TGACCTATGAATTGACAGCCCGA	miRNA	miR-192
t0026811	22	11	CAACGGAATCCCAAAAGCACCT	miRNA	miR-191
t0026355	21	11	TGGAGAGAAAGGCAGTTCCGG	miRNA	miR-185
t0026467	21	11	TGGAGAGTAAGGCAGTTCCTG	miRNA	miR-185
t0026522	21	11	TGGAGAGACAGGCAGTTCCTG	miRNA	miR-185
t0026732	21	11	TGGAGAGAAATGCAGTTCCTG	miRNA	miR-185
t0026512	23	11	TGGAGAGAAAGGTAGTTCCTGAT	miRNA	miR-185
t0026715	20	11	TGTAGAGAAAGGCAGTTCCT	miRNA	miR-185
t0026548	20	11	TGGAGAGAAAGGTAGTTCCT	miRNA	miR-185
t0026668	23	11	AGGAGAGAAAGGCAGTTCCTGAA	miRNA	miR-185
t0026601	23	11	TGGAGAGAAACGCAGTTCCTGAA	miRNA	miR-185
t0026441	23	11	TGGAGAGAAAGGCAGTCCCTGAA	miRNA	miR-185
t0026437	21	11	AAAGGTAGATAGAACAGGTCT	miRNA	miR-1839
t0026806	22	11	TTTGGCAATGGTAGAACTCACC	miRNA	miR-182
t0026303	24	11	TAGCATGTAAATATTGGCGAAAAA	miRNA	miR-16c
t0026301	20	11	AAGCGGCGATGGCGGAGCTA	miRNA	miR-1636
t0026305	21	11	GAAGCGGCGATGGCGGAGCTG	miRNA	miR-1636
t0026494	23	11	AAGCGGCGATGGCGGAGCTGAAA	miRNA	miR-1636
t0026502	26	11	TAGCAGCACGTAAATATTGGCGTAGC	miRNA	miR-16
t0026545	22	11	TAGCATTACGTAAATATTGGCG	miRNA	miR-16
t0026627	22	11	TAGCAGCAAGTAAATATTGGAG	miRNA	miR-16
t0026705	20	11	TAGCAGCACGTAAACATTGG	miRNA	miR-16
t0026708	22	11	TGGCAGCACATCATGGTTTACA	miRNA	miR-15b
t0026727	22	11	CAGCAGCACATCATGGTTTACA	miRNA	miR-15b
t0026740	19	11	TAGCAGCACATCATGGCTT	miRNA	miR-15b
t0026607	21	11	TAGTAGCACATAATGGTTTGT	miRNA	miR-15a
t0026430	21	11	TAGCAGCACATAACGGTTTGT	miRNA	miR-15a
t0026803	21	11	TAGCAGCACGTAATGGTTTGT	miRNA	miR-15a
t0026485	21	11	TAGCAGCACATAATAGTTTGT	miRNA	miR-15a
t0026781	22	11	TCAGTGCATCATAGAACTTTGT	miRNA	miR-148b-3p
t0026860	21	11	CTACAGTATAGATGATGTAAA	miRNA	miR-144
t0026533	19	11	ATAAAGTAGAAAGCACTAA	miRNA	miR-142-5p
t0026544	21	11	CCCATAAAGTAGAAAGCCCTA	miRNA	miR-142
t0026550	22	11	TACCACAGGGTAGAAGCACGGA	miRNA	miR-140
t0026839	22	11	ACCACAGGGTAGAACCGCGGAA	miRNA	miR-140
t0026399	24	11	TACCACAGAGTAGAACCACGGACA	miRNA	miR-140
t0026406	24	11	TACCACAGGGTAGAACCACCGACA	miRNA	miR-140

t0026651	20	11	ACCACAGGGTAGAACAACGG	miRNA	miR-140
t0026612	22	11	ACCACAGTGTAGAACCACGGAT	miRNA	miR-140
t0026647	20	11	CCACAGGGTAGAACCACGGT	miRNA	miR-140
t0026768	24	11	TACCACAGGGTAGAAACAAGGACA	miRNA	miR-140
t0026920	20	11	ACCACAGGGTAGAAACACGG	miRNA	miR-140
t0026404	22	11	ACCACAGGGTGGAACCACGGAC	miRNA	miR-140
t0026589	21	11	TACCACAGGGTAGAAACACGG	miRNA	miR-140
t0026360	22	11	TACCACAGGGTAGAACCCCGGT	miRNA	miR-140
t0026787	24	11	TACCACAGGGTAGAACCCCGGACT	miRNA	miR-140
t0026351	23	11	TACCCCAGGGTAGAACCACGGAC	miRNA	miR-140
t0026373	24	11	TACCACAGGGTAGAACCACGGCCT	miRNA	miR-140
t0026392	23	11	TAGCACAGGGTAGAACCACGGAC	miRNA	miR-140
t0026398	21	11	TTCGTGGCTACAGTGAGATGT	miRNA	miR-1304
t0026409	21	11	CCCCCCCACCCTCTCCTGCAG	miRNA	miR-1224-3p
t0026444	24	11	AGCAGCATTGTACAGGGCTATTAT	miRNA	miR-107
t0026454	26	11	TAAAGTGCTGACAGTGCAGATAAAAA	miRNA	miR-106b
t0026525	22	11	TAAAGTGCTGACAGTGCCGATA	miRNA	miR-106b
t0026592	21	11	AGCAGCATTGTACAGGACTAT	miRNA	miR-103a
t0026597	20	11	AGCAGCATTGTACCGGGCTA	miRNA	miR-103a
t0026774	23	11	AGCACCATTGTACAGGGCTATGA	miRNA	miR-103a
t0026845	23	11	AGGAGCATTGTACAGGGCTATGA	miRNA	miR-103a
t0026848	21	11	GCACAGTACTGTGATAACTGA	miRNA	miR-101c
t0026850	21	11	TACAGTACTGAGATAACTGAA	miRNA	miR-101
t0026882	22	11	GTACAGAACTGTGATAACTGAA	miRNA	miR-101
t0026910	21	11	TCCAGTACTGTGATAACTGAA	miRNA	miR-101
t0026713	21	11	TAAAGTACTGTGATAACTGAA	miRNA	miR-101
t0026483	20	11	TGAGGTAGTAGATTGTTCTT	miRNA	let-7j
t0026762	22	11	TGAGGTAGTAGTTTGGGCTGTT	miRNA	let-7i
t0026335	19	11	TGAGGTAGTAGTTTCTGCT	miRNA	let-7i
t0026609	19	11	TGAGGGAGTAGTTTGTGCT	miRNA	let-7i
t0026339	22	11	TGAGGTACTAGTTTGTGCTGTT	miRNA	let-7i
t0026370	22	11	TGAGGTAGTAATTTGTACAGTT	miRNA	let-7g
t0026655	21	11	TGAGGTAGTAGATTGTATATA	miRNA	let-7f-5p
t0026869	22	11	TGAGGTAGTAGATTGTATATCT	miRNA	let-7f-5p
t0026908	19	11	TGAGGTAGTGGATTGTATA	miRNA	let-7f-5p
t0026581	19	11	GGTAGTAGATTGTATAGTT	miRNA	let-7f
t0026690	23	11	TGAGGTGGTAGATTGTATAGTTG	miRNA	let-7f
t0026837	19	11	TGCGGTAGTAGATTGTATA	miRNA	let-7f
t0026913	21	11	CGAGGTAGTAGATTGGATAGT	miRNA	let-7f
t0026735	23	11	TGCGGTAGTAGATTGTATAGTTA	miRNA	let-7f
t0026353	23	11	TGAGGTAGGAGATTGTATAGTTA	miRNA	let-7e
t0026618	25	11	CTATACGACCTGCTGCCTTTCTATC	miRNA	let-7d*
t0026757	22	11	AGAGGTAATAGGTTGCATAGTT	miRNA	let-7d
t0026829	20	11	TATACAACCTACTGCCTTTT	miRNA	let-7b*
t0026856	27	11	TGAGGTAGTAGGTTGTGTGGTTATATC	miRNA	let-7b
t0026434	22	11	TGAGGTAGTAGGCTGTGTGGTA	miRNA	let-7b
t0026912	24	11	TGAGGTAGTAGGTTGTGCGGTTAA	miRNA	let-7b
t0026918	23	11	TGAGGTAGTAGGTTGTGTGGTCT	miRNA	let-7b
t0026390	22	11	TGAGGTGGTAGGTTGTGTGGTA	miRNA	let-7b
t0026530	22	11	TGGGGTAGTAGGTTGTGTGGTA	miRNA	let-7b
t0026672	23	11	TGAGGTAGTAGGCTGTGTGGTTA	miRNA	let-7b
t0026738	20	11	CTATACAATCTACTGTCTTT	miRNA	let-7a*
t0026884	20	11	TGAGGTCGTAGGTTGTATAG	miRNA	let-7
t0026488	20	11	TGAGGTAATAGGTTGTATAG	miRNA	let-7
t0026310	23	11	TGAGGTAGTAGGTTGTATATGTT	miRNA	let-7
t0026643	20	11	TGAGGTAGTAGGTTGTAAAG	miRNA	let-7
t0026381	21	10	CTATACAACTTACTACTTTTT	miRNA	miR-98*
t0026304	22	10	CAAAGTGCTGTTCGTGCAGGTC	miRNA	miR-93

t0026346	23	10	CAAAGTGCTGTTCGTGCAGGGAG	miRNA	miR-93
t0026523	22	10	TATTGCACTTGTACCGGCCTGT	miRNA	miR-92a
t0026541	18	10	GGGAGACTGTGAAGCGGA	miRNA	miR-920
t0026573	20	10	GGGAGACTTTGAAGCGGCGT	miRNA	miR-920
t0026631	23	10	TATTGCACTCGTCCCGGCCTATT	miRNA	miR-92
t0026919	20	10	GAAGGCCGAAGTGGAGAAGG	miRNA	miR-739
t0026843	26	10	AGGGCGCGCGGGTCGGGGCGGCGGCT	miRNA	miR-638
t0026329	22	10	TGGTGCAAAAGTAATGGCGGGT	miRNA	miR-548q
t0026400	22	10	TGGGCACAGGCGGATGGACAGG	miRNA	miR-5107
t0026863	22	10	TCCTGTAGTGAGCTGCCCCGAG	miRNA	miR-486-5p
t0026864	20	10	TCCTGTACTGAGCTGCACCG	miRNA	miR-486-5p
t0027566	23	10	TCCTGTACCGAGCTGCCCCGAGA	miRNA	miR-486-5p
t0026922	20	10	TCCTGTACTGAGCTGCCACG	miRNA	miR-486-5p
t0027369	28	10	TCCTGTACTGAGCTGCCCCGAGTATAGT	miRNA	miR-486-5p
t0027135	20	10	CCTGTACTGAGCTGCCCCGA	miRNA	miR-486-5p
t0026921	29	10	TCCTGTACTGAGCTGCCCCGAGACGGCAT	miRNA	miR-486-5p
t0026967	24	10	GTAGGATTGTGTCGGCAGATTTTT	miRNA	miR-4860
t0027290	20	10	TTAATTTTTTGTTTCGGTCA	miRNA	miR-4775
t0027405	20	10	TGAGGGAAGGAGGCTTGGTA	miRNA	miR-4747-5p
t0027242	18	10	GGGCTGGGTCGGTCGGGC	miRNA	miR-4651
t0027282	20	10	AAGCAGCACGTAAATATTGG	miRNA	miR-457b
t0027474	22	10	AGAGAAGGGTCGGGGCGGTTTT	miRNA	miR-4516
t0026970	23	10	CGGGGGGAGAAGGGTCGGGGCGG	miRNA	miR-4516
t0027040	23	10	TGAGGGAGTAGGTTGTGTGGTAA	miRNA	miR-4510
t0027260	22	10	TGAGGGAGTAGGTTGTGCGGTT	miRNA	miR-4510
t0027274	23	10	AAGCCGTTACCATTACTGAGTTT	miRNA	miR-451
t0027397	25	10	AAACAGTTACCATTACTGAGTTTAG	miRNA	miR-451
t0027477	24	10	AACCGTTACCATTACTGAGTTTAG	miRNA	miR-451
t0027577	22	10	AACCCGTTACCATTACTGAGTT	miRNA	miR-451
t0027499	19	10	AAACAGTTACCATTACTGA	miRNA	miR-451
t0027038	22	10	CCGTTACCATTACTGAGTTAAA	miRNA	miR-451
t0027063	22	10	AAACCGTTACCATTACGGAGTT	miRNA	miR-451
t0027372	21	10	TAAACCGTTACCATTACTGAG	miRNA	miR-451
t0027513	25	10	AAACCGTTACCATTACTGAGGTTAG	miRNA	miR-451
t0026977	21	10	AAACCGTAACCATTACTGAGT	miRNA	miR-451
t0027031	22	10	AGCGGGGCGGCGGGGTCGGGGT	miRNA	miR-4466
t0026985	22	10	GGGTGCGGGCCGGCGGGGTCCT	miRNA	miR-4466
t0027331	22	10	AGAGGGGCAGAGAGCGAGACTT	miRNA	miR-423-5p
t0027232	18	10	TAGGGGCAGAGAGCGAGA	miRNA	miR-423-5p
t0027305	22	10	TGTGGGGCAGAGAGCGAGACTT	miRNA	miR-423-5p
t0027390	21	10	TGAGGGGAAGAGAGAGAGACT	miRNA	miR-423-5p
t0027399	21	10	TGAGGGGCAGAGATCCAGACT	miRNA	miR-423-5p
t0027432	25	10	TGAGGGGCAGAGAGCGAGACTTAAA	miRNA	miR-423-5p
t0027461	22	10	AGAGGTAGTAGTTTGTACAGTT	miRNA	miR-3962
t0027507	20	10	CGGGGTCGGCGGCGACGTTT	miRNA	miR-3885-5p
t0027536	25	10	CCCCGGCGGCGGCGCCTCACTAATC	miRNA	miR-3885-5p
t0027587	20	10	TGGCGGCGGCGGCGGGGGCG	miRNA	miR-3885-5p
t0027594	28	10	CGGCGGCGGCGGCGACTCTGGACGCGAC	miRNA	miR-3885-5p
t0027093	23	10	ACTGGACTTGGAGGCAGAAGGCA	miRNA	miR-378f
t0027570	23	10	ACTGGACTTGGAGTCAGAAGGAA	miRNA	miR-378c
t0027129	22	10	TCTCACACAGAAATCGCAACCG	miRNA	miR-342-3p
t0027283	18	10	TTATAAAGCAATGAGACT	miRNA	miR-340-5p
t0027363	22	10	GAAAGCTGGGTTGAGAGGGCGT	miRNA	miR-320d
t0027381	22	10	AAAAGCTGGGTTGAGAGGGTAT	miRNA	miR-320d
t0027571	22	10	AAAAGCTGGGTTGAGAGGGCCT	miRNA	miR-320d
t0027600	22	10	ACAAGCTGGGTTGAGAGGGCGT	miRNA	miR-320d
t0027238	22	10	AAAAGCTGGGTTGAGTGGGCGT	miRNA	miR-320d
t0027022	22	10	AAAAGCTGGGTTGAGAGGGCAC	miRNA	miR-320d

t0027463	23	10	GAAAGCTGGGTTGAGAGGGCGAT	miRNA	miR-320a
t0027563	23	10	AAAAGCTGGGATGAGAGGGCGAA	miRNA	miR-320a
t0027602	23	10	AAAAACTGGGTTGAGAGGGCGAA	miRNA	miR-320a
t0026987	23	10	AAAAGCTGGGTTGAGCGGGCGAT	miRNA	miR-320a
t0027598	23	10	AAAGGCTGGGTTGAGAGGGCGAA	miRNA	miR-320a
t0027311	24	10	AGAGGCTTTGTGCGGATACGGGTT	miRNA	miR-3188
t0027054	21	10	GGCTGGTCCGAGTGCAGTGGT	miRNA	miR-3135b
t0027070	20	10	GGCTGGTCCGAGTGCAGTGG	miRNA	miR-3135b
t0027133	22	10	TTCAAGTAATCCAGGATAGGCC	miRNA	miR-26a
t0027280	21	10	CAAGTAATCCAGGATAGGCTT	miRNA	miR-26a
t0027438	22	10	CATTGCACTTGTCTCGGGCTGT	miRNA	miR-25
t0027578	21	10	CATTGCACTTGTCTCAGTCTG	miRNA	miR-25
t0027597	22	10	CATTGCACCTGTCTCGGTCTGA	miRNA	miR-25
t0026930	21	10	TATTGCACTTGTCTCGGTCTG	miRNA	miR-25
t0026955	21	10	CATTGCACTTGTATCGGTCTG	miRNA	miR-25
t0027086	22	10	CACTGCACTTGTCTCGGTCTGA	miRNA	miR-25
t0027095	22	10	CATTGCACTTGTCTGGGTCTGA	miRNA	miR-25
t0027148	21	10	CATTGAACTTGTCTCGGTCTG	miRNA	miR-25
t0027272	22	10	CATTGCACTTGGCTCGGTCTGA	miRNA	miR-25
t0027024	23	10	TGGCTCAGTTCAGCAGGAACAGG	miRNA	miR-24b
t0027198	22	10	TCTAAGGGCTGGGTCGGTCGGG	miRNA	miR-2487
t0027156	21	10	ATCACATTGCCAGGGATTAGA	miRNA	miR-23b
t0027567	24	10	ATCACATTGCCAGGGATTTCCTTT	miRNA	miR-23a
t0026961	23	10	AGCTACATTGTCTGCTGGGTTTT	miRNA	miR-221
t0027071	19	10	GGGGCGGTACATCTGTTAA	miRNA	miR-219-2-3p
t0027138	25	10	TTGACTGGGGCGGTACATCTGTTAA	miRNA	miR-219-2-3p
t0027203	21	10	ACTGTAGTATGGGCACTTTTT	miRNA	miR-20b*
t0027353	25	10	TTGAATGTAAAGAAGTATGTATATC	miRNA	miR-1a
t0027378	20	10	TGAGGTAGGTTGTATAGTAA	miRNA	miR-1961
t0027392	22	10	CTGACCTATGAATTGACAGCCC	miRNA	miR-192
t0027503	23	10	CAACGGAATCCAAAAAGCAGCTG	miRNA	miR-191
t0027526	22	10	CAACGGAATCCCGAAAGCAGCT	miRNA	miR-191
t0027266	20	10	AGGGGCTGGCTTTTCTCTGG	miRNA	miR-185*
t0027337	23	10	TGGAGAGAAAGGCAGTTACTGAG	miRNA	miR-185
t0027229	23	10	TGGAGAGAAAGGCAGATCCTGAA	miRNA	miR-185
t0027288	23	10	TGGCGAGAAAGGCAGTTCCTGAA	miRNA	miR-185
t0027506	20	10	CGGAGAGAAAGGCAGTTCCT	miRNA	miR-185
t0027246	22	10	TGGAGAGAAAGGCAGTTCTTGT	miRNA	miR-185
t0027490	23	10	CGGAGAGAAAGGCAGTTCCTGAT	miRNA	miR-185
t0027014	23	10	TGGAGAGAACGGCAGTTCCTGAA	miRNA	miR-185
t0027537	23	10	TGGAGAGAAAGGCAGTTCCTTAG	miRNA	miR-185
t0027366	23	10	TGGAGAGAAAGGCAGTTCCGGAA	miRNA	miR-185
t0027008	23	10	TGGAGAGAAAGGCAGTTCATGAT	miRNA	miR-185
t0027428	22	10	AAGGCAGATAGAACAGGTCTTG	miRNA	miR-1839
t0027462	21	10	AGGAAGATAGAACAGGTCTTG	miRNA	miR-1839
t0027373	22	10	TGAGGGAGTAGATTGGATAGTT	miRNA	miR-1827
t0026938	22	10	TGAGGAAGTAGATTGTATAGTA	miRNA	miR-1827
t0026939	20	10	ACATTCATTGCTGTCGGTGG	miRNA	miR-181b
t0027003	20	10	TCGGGGCGGCGGCGGCGGTG	miRNA	miR-1777a
t0027047	23	10	TAGCAGCACGTAAATATTGGCTC	miRNA	miR-16b
t0027141	18	10	GGGGGCGGGGAGCGGTCG	miRNA	miR-1607
t0027190	29	10	GGGGGCGGGGAGCGGTCGGGCGGCGGC	miRNA	miR-1607
t0027215	21	10	TAGCAGCATGTAAATATTGGC	miRNA	miR-16
t0027262	20	10	TAGCGGCACGTAAATATTGG	miRNA	miR-16
t0027264	22	10	TAGAAGCAAGTAAATATTGGCG	miRNA	miR-16
t0027320	23	10	TAGCAGCACGTAAATATTGGCGC	miRNA	miR-16
t0026937	22	10	TAGCAGCACATCCTGGTTTACA	miRNA	miR-15b
t0027423	24	10	AATAGCAGCACATCATGGTTTACA	miRNA	miR-15b

t0027475	22	10	TAGCAGCACATCATAGTTTACA	miRNA	miR-15b
t0027550	22	10	TAGCAGCACATCATGGTTTCCA	miRNA	miR-15b
t0027226	21	10	TAGCAGAACATCATGGTTTAC	miRNA	miR-15b
t0026951	19	10	TAGCAGAACATAATGGTTT	miRNA	miR-15a
t0027187	20	10	TAGCAGCACATAATGGTCTG	miRNA	miR-15a
t0027101	20	10	TAGGAGCACATAATGGTTTG	miRNA	miR-15a
t0027299	20	10	CGGCCCGGGCTGCTGCTGTT	miRNA	miR-1538
t0026925	20	10	GGATATCATCATATACTCTA	miRNA	miR-144*
t0027254	18	10	TACAGTATAGATGATGGA	miRNA	miR-144
t0027316	20	10	ATAAAGTAGAAAGCACTACT	miRNA	miR-142-5p
t0027418	18	10	AGTGTTTCCTACTTTATG	miRNA	miR-142*
t0026972	20	10	ACCACAGGGTAGAACCAAGG	miRNA	miR-140
t0027306	23	10	TACCAAAGGGTAGAACCACGGAA	miRNA	miR-140
t0027345	24	10	AACCACAGGGTAGAACCACGGACA	miRNA	miR-140
t0027511	21	10	ACCACAGGGTAGAAAAACGGA	miRNA	miR-140
t0027585	22	10	ACCACAGGGTAGAGCCACGGAC	miRNA	miR-140
t0027064	23	10	TACCACAGGGTAGAACCATGGAT	miRNA	miR-140
t0027310	23	10	ACCATAGGGTAGAACCACGGATT	miRNA	miR-140
t0027387	22	10	ACAACAGGGTAGAACCACGGAT	miRNA	miR-140
t0027053	24	10	TACCACAGGGTAGGACCACGGACA	miRNA	miR-140
t0026964	24	10	ACCACAGGGTAGAACCACGGATAT	miRNA	miR-140
t0027117	23	10	TAACACAGGGTAGAACCACGGAT	miRNA	miR-140
t0027243	21	10	TACACAGGGTAGAACCACGGA	miRNA	miR-140
t0027558	20	10	ATCCCACCGCTGCCACCAAA	miRNA	miR-1260b
t0027046	20	10	GTGGGCGGGGGCAGGTGTGT	miRNA	miR-1228*
t0027062	37	10	AGCAGCATTGTACAGGGCTATGACATACT	miRNA	miR-103a
t0027114	18	10	GCAGCATTGTACAGGGCT	miRNA	miR-103a
t0027126	20	10	AGCAGCATTGAACAGGGCTA	miRNA	miR-103a
t0027171	20	10	AGCAGCATTGTGCAGGGCTA	miRNA	miR-103a
t0027217	19	10	AGCATTGTACAGGGCTATG	miRNA	miR-103a
t0027309	23	10	AGCCGCATTGTACAGGGCTATGA	miRNA	miR-103a
t0027411	20	10	AGCAGCACTGTACAGGGCTA	miRNA	miR-103a
t0027429	21	10	TACAGTACTGTGACAACTGAA	miRNA	miR-101
t0027433	20	10	TATAGTACTGTGATAACTGA	miRNA	miR-101
t0027460	21	10	GTACAGTACTGCGATAACTGA	miRNA	miR-101
t0027518	21	10	GTACGGTACTGTGATAACTGA	miRNA	miR-101
t0027075	20	10	TACAGTACTGTGATAACCGA	miRNA	miR-101
t0027130	21	10	TACAGTACTGTGATAACTGCA	miRNA	miR-101
t0026973	20	10	TAAAGTACTGTGATAACTGA	miRNA	miR-101
t0027000	21	10	TGAGGTAGTAGATTGTGCTGT	miRNA	let-7i
t0027034	22	10	TGCGGTAGTAGTTTGTGCTGTT	miRNA	let-7i
t0027267	20	10	TGAGGTAGGAGTTTGTGCTG	miRNA	let-7i
t0027383	22	10	TAAGGTAGTAGTTTGTGCTGTT	miRNA	let-7i
t0027492	22	10	TGAGGTAGCAGTTTGTGCTGTT	miRNA	let-7i
t0027508	21	10	TGAGGTAGTGGTTTGTACAGT	miRNA	let-7g
t0027025	22	10	TGAGGTCGTAGTTTGTACAGTT	miRNA	let-7g
t0027180	23	10	TGAGGTAGTGGATTGTATAGTTG	miRNA	let-7f-5p
t0027258	23	10	TGAGGTAGTGGATTGTATAGTTA	miRNA	let-7f-5p
t0027389	21	10	GAGGAAGTAGATTGTATAGTT	miRNA	let-7f
t0027401	23	10	ATTGAGGTAGTAGATTGTATAGT	miRNA	let-7f
t0027471	22	10	TGAGGTACTAGATTGTATATTT	miRNA	let-7f
t0027519	23	10	TGAGGTAGTAGATTGTATAGCTG	miRNA	let-7f
t0027021	23	10	TGAGGTAGTAGACTGTATAGTTG	miRNA	let-7f
t0027172	23	10	TGAGGTAGTAGATTGTGTAGTTG	miRNA	let-7f
t0027223	23	10	TGAGGTACTAGATTGTATAGTTA	miRNA	let-7f
t0027249	25	10	TGAGGTAGTAGATTGTATATTTATC	miRNA	let-7f
t0027545	26	10	TGAGGTAGTAGATTGTATAGTTAGTT	miRNA	let-7f
t0027374	19	10	TGAGGTAGAAGATTGTATA	miRNA	let-7f

t0027391	22	10	AGAGGTACTAGGTTGCATAGTT	miRNA	let-7d
t0026993	20	10	AGAGGTAGTAGGCTGCATAG	miRNA	let-7d
t0027009	21	10	AGAGGTGGTAGGTTGCATAGT	miRNA	let-7d
t0027068	21	10	AGAGGTAGTAGGATGCATAGT	miRNA	let-7d
t0027082	21	10	AGAGGTAGTAGGTTGCATCGT	miRNA	let-7d
t0027116	23	10	TGAGGAAGTAGGTTGTGTGGTTT	miRNA	let-7b
t0027127	26	10	TGAGGTAGTAGGTTGTGTGGTTAAAA	miRNA	let-7b
t0027179	22	10	CGAGGTAGTAGGTTGTGTGGTA	miRNA	let-7b
t0027182	23	10	TGAGGTAGTAGGTTGTGGGGTTG	miRNA	let-7b
t0027416	23	10	TGGGGTAGTAGGTTGTGTGGTTA	miRNA	let-7b
t0027420	19	10	TGAGGCAGTAGGTTGTGTG	miRNA	let-7b
t0027489	20	10	TGAGGTAGTAGTTTTGTTGT	miRNA	let-7a
t0027582	22	9	TATTGCACTTGTCCAGGCCTGT	miRNA	miR-92a
t0026942	23	9	TATTGCACTCGTCCCGGCCTCAT	miRNA	miR-92
t0027270	22	9	TACGTCATCGTTGTCATCGTCG	miRNA	miR-598
t0027271	21	9	AAAACCGCCATTACTTTTGCA	miRNA	miR-548z
t0027278	21	9	AAGACTGTAGTTGATGTTAAT	miRNA	miR-499
t0027527	20	9	TCCTGTACTGAGATGCCCCG	miRNA	miR-486-5p
t0027228	23	9	TACTGTACTGAGCTGCCCCGAGA	miRNA	miR-486-5p
t0027279	21	9	CGGGCTGTCCGGAGGGGTCGG	miRNA	miR-4741
t0027382	23	9	TGTAGAGAAGGGAGCAGGAAGCT	miRNA	miR-4732-5p
t0027483	20	9	GGAGAAGGGTCGGGGCGGCA	miRNA	miR-4516
t0027525	24	9	TGAGGGAGTAGGTTGTGTGGTTAG	miRNA	miR-4510
t0027603	20	9	TGAGGGAGTAGGGTGTATAG	miRNA	miR-4510
t0027212	22	9	AAACCGTTTCCATTACTGAGTT	miRNA	miR-451
t0028063	25	9	AAACCGTTAACATTACTGAGTTTAG	miRNA	miR-451
t0028293	23	9	AAACCGTTACCATTACTGAGTCT	miRNA	miR-451
t0027753	27	9	AAACCGTTACCATTACTGAGTTAAAAA	miRNA	miR-451
t0028300	22	9	AAACCGTTACCATTACTGAGTG	miRNA	miR-451
t0027701	24	9	AAACCGTTACCATTACTGGGTTTA	miRNA	miR-451
t0027723	21	9	AGCCGCATTGTACAGGGCTAT	miRNA	miR-4289
t0028046	23	9	TGAGGTGCAGAGAGCGAGATTTT	miRNA	miR-423-5p
t0028029	19	9	TGAGGGGCAGAGCGCGAGA	miRNA	miR-423-5p
t0028211	20	9	TGAGGGGCAGAGAGAGAGAC	miRNA	miR-423-5p
t0027674	23	9	TGAGGGGCAGAGAGCGAGACGTT	miRNA	miR-423-5p
t0027670	24	9	TGAGGGGCGGAGAGCGAGACTTTT	miRNA	miR-423-5p
t0028055	24	9	TGAGGGGCAGAGAGAGAGACTTTT	miRNA	miR-423-5p
t0027809	24	9	AAGCTCGGTCTGAGGCCCCTCAGT	miRNA	miR-423-3p
t0027888	22	9	GGAGGTAGTAGTTTGTACAGTT	miRNA	miR-3962
t0027936	22	9	ACTGGACTTGGAGTCAGAAGAA	miRNA	miR-378c
t0028023	22	9	ACTGGACTTGGAGTCAGAAGGT	miRNA	miR-378
t0028069	24	9	CTGGACTTGGAGTCAGAAGGCTTT	miRNA	miR-378
t0028073	28	9	AACTTTGAAGACTGAAGTGGGGAAGGGT	miRNA	miR-3526
t0027883	23	9	TGAAGACTGAAGGGGAGAAGGGT	miRNA	miR-3526
t0027664	29	9	GGCCCGGTAGCTCAGTCGGTAGAGCAGA	miRNA	miR-3488
t0027777	23	9	TCTCACACAGAAATCGAACCCGT	miRNA	miR-342-3p
t0027862	23	9	AAAAGCTGGGTTGAGGGGGCGTA	miRNA	miR-320d
t0027884	22	9	AAAAGCTGGGTTGAGAGGGCCA	miRNA	miR-320d
t0027958	23	9	AAAAGCTGGGTTGAGAGGGGGAT	miRNA	miR-320d
t0028303	22	9	AAAAGCTGGGTTGAGAGGGATT	miRNA	miR-320d
t0028085	21	9	AAAGTCGGGTTGAGAGGGCGA	miRNA	miR-320c
t0027778	21	9	AAAAGCTGGGTTGAGCGGGCG	miRNA	miR-320a
t0028043	22	9	AAAAGCTGGGTAGAGAGGGCGA	miRNA	miR-320a
t0027897	23	9	AACAGCTGGGTTGAGAGGGCGAT	miRNA	miR-320a
t0028093	23	9	AAAAGCTGGGTTGAAAGGGCGAA	miRNA	miR-320a
t0027677	21	9	AATCTGGGTTGAGAGGGCGAT	miRNA	miR-320a
t0027808	23	9	AAAAGATGGGTTGAGAGGGCGAT	miRNA	miR-320a
t0028289	22	9	GGCTGGTCTGAGTGCAGTGGTG	miRNA	miR-3135b

t0027881	27	9	GGCTGGTCCGAGTGCAGTGGTGTTCCT	miRNA	miR-3135b
t0027716	20	9	TAGCACCATTTGAAATCGGT	miRNA	miR-29c
t0027964	26	9	CGCGCGCGGGTCGGGGGCGGGGCGG	miRNA	miR-2981
t0028086	31	9	CGCGCGCGGGTCGGGGGCGGGGCGGA	miRNA	miR-2981
t0028094	31	9	CCGCGCGCGGGTCGGGGGCGGGGCGG.	miRNA	miR-2981
t0027666	18	9	TCGTTGTCGGGCGGCGGG	miRNA	miR-2885
t0027654	22	9	TTCAAGTAATCCAGGATAAGCT	miRNA	miR-26a
t0027779	22	9	TTCAAGTAATACAGGATAGGCT	miRNA	miR-26a
t0027824	21	9	CATTGGACTTGTCTCGGTCTG	miRNA	miR-25
t0027839	21	9	CATTGCGCTTGTCTCGGTCTG	miRNA	miR-25
t0028252	18	9	AAGCTGCCATTTGAAGAA	miRNA	miR-22-3p
t0028279	22	9	AGCTACATTGTCTGCTGGGTAT	miRNA	miR-221
t0027635	22	9	AGCTACATTGTCTGCTGGGGTT	miRNA	miR-221
t0027675	20	9	GACTGGGGCGGCACATCTGT	miRNA	miR-219-2-3p
t0028291	21	9	GACTGGGGCGGGACATCTGTT	miRNA	miR-219-2-3p
t0027684	22	9	TTGGGGAAACGGCCGCTGAGGG	miRNA	miR-2110
t0027830	21	9	CTGTGCGTGTGACAGCGGCTG	miRNA	miR-210
t0028274	18	9	TAGCTTATCAGACTGATG	miRNA	miR-21
t0028264	23	9	TGACCTATGAATTGACAGCCCGT	miRNA	miR-192
t0027631	20	9	CTGACCTATGAATTGACCGC	miRNA	miR-192
t0028244	23	9	CAACGGAATCACAAAAGCAGCTG	miRNA	miR-191
t0028048	18	9	CAACGGAATCCCAAAAGC	miRNA	miR-191
t0028299	24	9	CAACAGAATCCCAAAAGCAGCTGA	miRNA	miR-191
t0027947	22	9	TGGAGAGAAAGGCAGTTAATGA	miRNA	miR-185
t0027619	21	9	TGGAGAGTAAGGCACTTCCTG	miRNA	miR-185
t0028031	23	9	TGGAGTGAAAGGCAGTTCCTGAA	miRNA	miR-185
t0027923	21	9	TGGAGACAAAGGCAGTTCCTG	miRNA	miR-185
t0028056	23	9	TGGAGAGAAAGGCAGTTCCTGGG	miRNA	miR-185
t0027954	25	9	TGGAGAGAAAGGCAGTTCCTGAAAC	miRNA	miR-185
t0027703	23	9	TGGAGAGAAAGGCAGATCCTGAT	miRNA	miR-185
t0028221	23	9	TGGAGAGAAAAGCAGTTCCTGAT	miRNA	miR-185
t0027823	22	9	AAGGTAGATAGAACAGGTCTTT	miRNA	miR-1839
t0028114	22	9	TGAGTTAGTAGATTGTATACTA	miRNA	miR-1827
t0027784	22	9	TGAGGCAGTAGATTGTATAGTA	miRNA	miR-1827
t0027861	20	9	GAGGGAGTAGATTGTATAGT	miRNA	miR-1827
t0028175	22	9	TGAGGGAGTAGATTGTACAGTT	miRNA	miR-1827
t0027689	22	9	TGAGGGGGTAGATTGTATAGTT	miRNA	miR-1827
t0027806	21	9	TGAGGGAGTAGATTGTGTAGT	miRNA	miR-1827
t0027885	22	9	AACATTCAACGCTGTCGGTGCG	miRNA	miR-181a
t0027903	18	9	AACATTCAACGCTGTCGG	miRNA	miR-181a
t0028030	20	9	TCGGGGCGGCGGCGGCGTTG	miRNA	miR-1777a
t0028038	21	9	AAGCGGCGATGGCGGAGCTCA	miRNA	miR-1636
t0028131	26	9	GGGGGCGGGGAGCGGTCGGGCGGCGG	miRNA	miR-1607
t0028267	23	9	GGGGGCGGGGAGCGGTCGGGCGG	miRNA	miR-1607
t0027997	18	9	TGGGGGCGGGGAGCGGTC	miRNA	miR-1607
t0027609	23	9	TAGCAGAACGTAAATATTGGCGT	miRNA	miR-16
t0027798	23	9	TAGCAGCACGTAAATATTGGAGT	miRNA	miR-16
t0027814	22	9	GCGTAGCAGCACGTAAATATTG	miRNA	miR-16
t0027821	22	9	TAGCAGCACGTAAATATTGTAG	miRNA	miR-16
t0027863	23	9	TAGCAGCACGCAAATATTGGCGA	miRNA	miR-16
t0028298	20	9	TAGCAGCACGTACATATTGG	miRNA	miR-16
t0027908	20	9	TAGCAGCACGTAGATATTGG	miRNA	miR-16
t0027919	20	9	TAGCAGCACATCATGGTTAT	miRNA	miR-15c
t0027967	21	9	TAGCAGCACATCATGGTTCAC	miRNA	miR-15b
t0027921	23	9	TAGCAGCACATCATGGTTTAATT	miRNA	miR-15b
t0027615	19	9	TAGCAGCACATCATAGTTT	miRNA	miR-15b
t0027775	22	9	TAGCAGCACATCATGGTTTGCA	miRNA	miR-15b
t0028005	19	9	TAGCAGCACATCCTGGTTT	miRNA	miR-15b

t0027818	22	9	TAACAGCACATCATGGTTTACA	miRNA	miR-15b
t0027846	20	9	GAGCAGCACATCATGGTTTA	miRNA	miR-15b
t0028000	22	9	AAGCAGCACATCATGGTTTACA	miRNA	miR-15b
t0028010	20	9	TAGCAGCACATCTTGGTTTA	miRNA	miR-15b
t0028109	22	9	TAGCAGCACAACATGGTTTACA	miRNA	miR-15b
t0028183	21	9	TAGGAGCACATAATGGTTTGT	miRNA	miR-15a
t0028292	20	9	TAGCAGCACATGATGGTTTG	miRNA	miR-15a
t0027999	20	9	TAACAGCACATAATGGTTTG	miRNA	miR-15a
t0027637	21	9	TAGCAGCACATAATGGTTTCT	miRNA	miR-15a
t0027933	21	9	TAGCAGCCCATAATGGTTTGT	miRNA	miR-15a
t0027951	24	9	GGATATCATCATATATTGTAAGTT	miRNA	miR-144*
t0027998	21	9	ATAAAGTAGAAAGCACTACTA	miRNA	miR-142-5p
t0028116	20	9	CCCATAAAGGAGAAAGCACT	miRNA	miR-142
t0028133	19	9	CCCATAAAGTAGAAAGCAA	miRNA	miR-142
t0028206	22	9	ACCACAGGGTAGAACCACGGTC	miRNA	miR-140
t0028220	21	9	ACCACAGGGTAGACCCACGGA	miRNA	miR-140
t0028232	23	9	TAGCACAGGGTAGAACCACGGAA	miRNA	miR-140
t0028276	22	9	ACTACAGGGTAGAACCACGGAA	miRNA	miR-140
t0027693	23	9	TACCACAGGGTAGCACCACGGAA	miRNA	miR-140
t0027721	24	9	TACCACCGGGTAGAACCACGGACA	miRNA	miR-140
t0027794	23	9	TACCACAGGCTAGAACCACGGAA	miRNA	miR-140
t0027870	21	9	ACCACAGGGTAGAACCAGGGA	miRNA	miR-140
t0028258	22	9	TACCACAGGGTTGAACCACGGA	miRNA	miR-140
t0027944	22	9	TACCACAGGGTAGTACCACGGA	miRNA	miR-140
t0027735	22	9	TACACAGGGTAGAACCACGGAC	miRNA	miR-140
t0028022	23	9	TACCACAGGGTAGAACCACCGAA	miRNA	miR-140
t0028039	26	9	TACCACAGGGTAGAACCACGGACAGA	miRNA	miR-140
t0027708	24	9	ACCACAGGGTAGAACCACGGAAAA	miRNA	miR-140
t0027715	21	9	ACCACAGGGTAGAACGACGGA	miRNA	miR-140
t0027733	23	9	TACCCCAGGGTAGAACCACGGAA	miRNA	miR-140
t0027740	19	9	ACAGGGTAGAACCACGGAC	miRNA	miR-140
t0027756	24	9	TACCACAGGGAAGAACCACGGACA	miRNA	miR-140
t0027786	24	9	TACCTCAGGGTAGAACCACGGACA	miRNA	miR-140
t0027807	23	9	TACCACAGGGTAGAACGACGGAA	miRNA	miR-140
t0027891	22	9	ACCACAGGGTAGAACCACGGTT	miRNA	miR-140
t0027909	23	9	AGCAGCATTGAACAGGGCTATGA	miRNA	miR-103a
t0027941	22	9	AGCAGCATTGTACTGGGCTATG	miRNA	miR-103a
t0027978	20	9	AGCAGCATTGTCCAGGGCTA	miRNA	miR-103a
t0028006	31	9	TACAGTACTGTGATAACTGAAACATAGAC	miRNA	miR-101
t0028047	22	9	GTACAGTACTGTGATAACTCAA	miRNA	miR-101
t0028076	22	9	GTACAGTGCTGTGATAACTGAA	miRNA	miR-101
t0028110	22	9	TACAGTTCTGTGATAACTGAAG	miRNA	miR-101
t0028158	21	9	TACAGTGCTGTGATAACTGAA	miRNA	miR-101
t0028181	22	9	GTAAAGTACTGTGATAACTGAA	miRNA	miR-101
t0028203	21	9	TACAGGACTGTGATAACTGAA	miRNA	miR-101
t0028245	22	9	TACAGTACTGCGATAACTGAAG	miRNA	miR-101
t0028254	21	9	TACAGAACTGTGATAACTGAA	miRNA	miR-101
t0028273	20	9	TACAGTACTGTGATAACTGC	miRNA	miR-101
t0027627	22	9	TACAGTACTGTGATAACCGAAG	miRNA	miR-101
t0027956	22	9	TGAGGTAGTAGTTTGCGCTGTT	miRNA	let-7i
t0028208	22	9	TGAGGTAGTAGTTTGTGCGGTT	miRNA	let-7i
t0027648	22	9	CGAGGTAGTAGTTTGTGCTGTT	miRNA	let-7i
t0027697	22	9	TGAGGTAGAAGTTTGTGCTGTT	miRNA	let-7i
t0027698	22	9	TGAGGTAGTAGTTTATGCTGTT	miRNA	let-7i
t0027736	21	9	TGAGGTAGTAGTTTGCACAGT	miRNA	let-7g
t0027774	21	9	TGAGGTAGTAGCTTGTACAGT	miRNA	let-7g
t0027875	21	9	TGAGGTAGTAGTTTGTACAGG	miRNA	let-7g
t0027989	22	9	TGAGGTAGTAGATTGTATAGGG	miRNA	let-7f-5p

t0028013	22	9	TGAGGTAGTAGATTGTATAACT	miRNA	let-7f-5p
t0028200	21	9	GAGGTAGTAGATTGTATCGTT	miRNA	let-7f
t0028224	23	9	TGAGGTGGTAGATTGTATAGTTT	miRNA	let-7f
t0028249	23	9	TGCGGTAGTAGATTGTATAGTTG	miRNA	let-7f
t0027633	19	9	TGAGGTAGTATATTGTATA	miRNA	let-7f
t0027959	22	9	TGAGGTAGTAGATTGTGTTGTT	miRNA	let-7f
t0028207	23	9	TGAGGTAGCAGATTGTATAGTTG	miRNA	let-7f
t0028229	18	9	TGAGGTAGTAGATTGTAA	miRNA	let-7f
t0028297	22	9	TGAGGTAGTAGATTTGATAGTT	miRNA	let-7f
t0027799	27	9	TGAGGTAGTAGATTGTATAGTTGGCAA	miRNA	let-7f
t0027813	19	9	TGAGGTAGTAGATTATATA	miRNA	let-7f
t0028286	23	9	TGAGGTAGTAGATTTTATAGTTT	miRNA	let-7f
t0027981	23	9	TGAGGTAGTAGATTGTACAGTTG	miRNA	let-7f
t0028155	20	9	AGAGGTAGCAGGTTGCATAG	miRNA	let-7d
t0027620	22	9	AGAGGTAGTAGGTTGCAAAGTT	miRNA	let-7d
t0027653	22	9	AGAAGTAGTAGGTTGCATAGTT	miRNA	let-7d
t0027705	20	9	AGAGGTAGGAGGTTGCATAG	miRNA	let-7d
t0027722	22	9	AGAGGTAGTCGGTTGCATAGTT	miRNA	let-7d
t0027792	21	9	AGAGGTAGTAGGTTGCATGGT	miRNA	let-7d
t0027925	22	9	AGAGGTCGTAGGTTGCATAGTT	miRNA	let-7d
t0028027	22	9	AGAGATAGTAGGTTGCATAGTT	miRNA	let-7d
t0028127	23	9	TGAGGTAGGAGGTTGTGTGGTTA	miRNA	let-7b
t0028193	20	9	TAAGGTAGTAGGTTGTGTGG	miRNA	let-7b
t0028201	24	9	TGAGGTAGTAGGTTGTGTTGTTAA	miRNA	let-7b
t0028250	20	9	TGAGGTAGTAGGATGTGTGG	miRNA	let-7b
t0028255	23	9	TGAGGTAGTAGGTTGTGTGGTCA	miRNA	let-7b
t0027625	23	9	TGAGGTAGTGGGTTGTGTGGTTA	miRNA	let-7b
t0027731	20	9	TGAGGTAGGAGGTTGTGTGG	miRNA	let-7b
t0027910	22	9	TAAGGTAGTAGGTTGTGTGGTA	miRNA	let-7b
t0027965	22	9	GAGGTAGTAGGTTGTATAGTTT	miRNA	let-7
t0028165	20	9	TGAAGTAGTAGGTTGTATAG	miRNA	let-7
t0028269	24	8	TCCAGGTGGAAGGTTGTAGGCATG	miRNA	miR-965-5p
t0028281	22	8	TATTGTACTTGTCCCGGCCTGT	miRNA	miR-92d
t0028296	22	8	TATTGCACTTGTCCCGGCCTGC	miRNA	miR-92b-3p
t0027710	22	8	TATTGCACTTGTCCCAGCCTGT	miRNA	miR-92a
t0027766	21	8	TATTGCACTTGTCCCGGACTG	miRNA	miR-92a
t0027773	21	8	TATTGCATTTGTCCCGGCCTG	miRNA	miR-92a
t0027847	19	8	TATTGCACTCGTCCCGGCT	miRNA	miR-92
t0028024	22	8	AATGGCGCCACTAGGGTTGTGT	miRNA	miR-652
t0028066	21	8	TCCTGAACTGAGCTGCCCCGA	miRNA	miR-486-5p
t0028121	22	8	TCCTGTACTGAGCTGCACAGAG	miRNA	miR-486-5p
t0028242	29	8	TCCTGTACTGAGCTGCCCCGAGACCGGC	miRNA	miR-486-5p
t0027952	24	8	TTCCTGTACTGAGCTGCCCCGAGT	miRNA	miR-486-5p
t0028209	21	8	TCCTGTACTGAGCTGCCCGGA	miRNA	miR-486-5p
t0028635	22	8	TCGTGTACTGAGCTGCCCCGAG	miRNA	miR-486-5p
t0028323	23	8	TCCTGTCCTGAGCTGCCCCGAGA	miRNA	miR-486-5p
t0028368	22	8	TCCTGTACTGAGCGGCCCCGAG	miRNA	miR-486-5p
t0028476	23	8	TCCTGTACTGAACTGCCCCGAGA	miRNA	miR-486-5p
t0028621	22	8	TCCTGTACTGAGCTGACCAGAG	miRNA	miR-486-5p
t0028804	27	8	TTCTGTACTGAGCTGCCCCGAGACGGC	miRNA	miR-486-5p
t0028458	23	8	TGTAGAGCAGGGAGAAGGAAGCT	miRNA	miR-4732-5p
t0028388	21	8	TGTAGAGCAGGGCGCAGGAAG	miRNA	miR-4732-5p
t0028355	21	8	TGTAGAGCAGGGGGCAGGAAG	miRNA	miR-4732-5p
t0028455	21	8	AAGGGCTGGGTCGGTCGGGCT	miRNA	miR-4651
t0028490	22	8	TAGCAAGAGAACCATTACCATT	miRNA	miR-451b
t0028554	21	8	GAGAAGGGTCGGGGCGGCAGG	miRNA	miR-4516
t0028573	19	8	GAGAAGGGTCGGGGCGGCA	miRNA	miR-4516
t0028603	24	8	AGAAGGGTCGGGGCGGCAGGGGCT	miRNA	miR-4516

t0028824	18	8	TGAGGGAGTAGGTTGTGT	miRNA	miR-4510
t0028853	20	8	AAACCGTTACCATTACTGGT	miRNA	miR-451
t0028880	19	8	AAACCGTTACCATTACTTT	miRNA	miR-451
t0028921	29	8	AAACCGTTACCATTACTGAGTTTCGTCGG	miRNA	miR-451
t0029030	19	8	AAACCGTTACAATTACTGA	miRNA	miR-451
t0028656	23	8	AAACCGCTACCATTACTTAGTTT	miRNA	miR-451
t0028844	23	8	AGACCGTTACCATTACTGAGTTT	miRNA	miR-451
t0028961	20	8	AAACCGTTACCATTACTGAT	miRNA	miR-451
t0028683	18	8	AATGTTACCATTACTGAG	miRNA	miR-451
t0028604	21	8	AAACCGTTACCATTACAGAGT	miRNA	miR-451
t0028574	20	8	AAACCGTTACCATTACTGTG	miRNA	miR-451
t0028867	21	8	CGACCGGCTCCGGGACGGCTG	miRNA	miR-4497
t0028949	23	8	CTCCCACTGCTTCACTTGACTAG	miRNA	miR-4301
t0028443	20	8	AGCGGCATTGTACAGGGCTA	miRNA	miR-4289
t0028312	21	8	AGGAGCATTGTACAGGGCTAT	miRNA	miR-4289
t0028329	27	8	TGAGGGGCAGAGAGCGAGACTTTTATT	miRNA	miR-423-5p
t0028530	23	8	TGGGGGGCAGAGAGCGAGACTTA	miRNA	miR-423-5p
t0028662	23	8	TGAGAGGCAGAGAGCGAGACTTA	miRNA	miR-423-5p
t0028712	23	8	TGAGGGACAGAGAGCGAGACTTA	miRNA	miR-423-5p
t0028776	24	8	TGAGGCGCAGAGAGCGAGACTTTT	miRNA	miR-423-5p
t0028816	21	8	TGAGAGGCAGAGAGCTAGACT	miRNA	miR-423-5p
t0028971	23	8	TGAGGAGCAGAGAGCGAGACTTA	miRNA	miR-423-5p
t0029018	24	8	CGAGGGGCAGAGAGCGAGACTTTT	miRNA	miR-423-5p
t0029021	24	8	TGAGGGGCAGAGAGCGGGACTTTT	miRNA	miR-423-5p
t0029034	23	8	TGAGGGGCAGGGAGCGAGACTTA	miRNA	miR-423-5p
t0028849	20	8	TGAGGGGCAGAGAGCGCGAC	miRNA	miR-423-5p
t0028658	19	8	TGAGTGGCAGAGAGCGAGA	miRNA	miR-423-5p
t0028891	23	8	AGCTCGGTCTGAGGCCCCTCATT	miRNA	miR-423-3p
t0028337	21	8	TAAGGTAGTAGTTTGTACAGT	miRNA	miR-3962
t0028353	23	8	AGAACTGAAACGGACAAGGTGAA	miRNA	miR-3896-3p
t0028451	29	8	GCCGGCGGCGGCGGCGACTCTGGACGC[illegible]	miRNA	miR-3885-5p
t0028536	22	8	TCGGGGCGGCGGCGGCGGCGGT	miRNA	miR-3885-5p
t0028666	30	8	GGCCGGCGGCGGCGGCGACTCTGGACG[illegible]	miRNA	miR-3885-5p
t0028669	24	8	CGAGACTTACGTTGCGTCAATTCT	miRNA	miR-3850-3p
t0028706	22	8	ATATAATACAACCTGCTCAGTG	miRNA	miR-374
t0028756	18	8	CGGGGGTGGGGTCGGCGT	miRNA	miR-3621
t0028795	27	8	TGAAGACTGAAGTGGAGAAGGGGTTCT	miRNA	miR-3526
t0028838	23	8	TCTCACAAAGAAATCGCACCCGT	miRNA	miR-342-3p
t0028881	23	8	TCAAGAGCAATAACGAAAAATGA	miRNA	miR-335
t0028907	22	8	AAAACCTGGGTTGAGAGGGCGT	miRNA	miR-320d
t0028358	23	8	AAAAGCTGGGTTGAGAGGGTGTA	miRNA	miR-320d
t0028625	23	8	AAAAGATGGGTTGAGAGGGCGTA	miRNA	miR-320d
t0028928	23	8	AAAAGCTGGGTTGGGAGGGCGTT	miRNA	miR-320d
t0028417	23	8	AAAAGCTGGGTTGAGAGGGCGGG	miRNA	miR-320d
t0028647	23	8	AAAAGCTGGGTTGAGAGGGAGAG	miRNA	miR-320d
t0028873	23	8	AAAAGCTGGGATGAGAGGGCGTA	miRNA	miR-320d
t0029024	22	8	AGAAGCTGGGTTGAGAGGGCGT	miRNA	miR-320d
t0028508	23	8	AGAAGCTGGGTTGAGAGGGCGTA	miRNA	miR-320d
t0028470	23	8	AAAAGCTGGGTTGAGAAGGCGAA	miRNA	miR-320a
t0028350	23	8	AAGAGCTGGGTTGAGAGGGCGAT	miRNA	miR-320a
t0028356	24	8	AATCTGAGAAGGCGCACAAGGTTA	miRNA	miR-3200-5p
t0028474	23	8	CACCTTGCGCTACTCAGGTCTGA	miRNA	miR-3200-3p
t0028399	18	8	TCAGTCGGATGTTTACAG	miRNA	miR-30e*
t0028484	26	8	TGTAAACATCCTACACTCTCAAAAAA	miRNA	miR-30c
t0028514	23	8	TGTAAACATCCTACACTCAGCTA	miRNA	miR-30b
t0028637	19	8	GCGGGTCGGGGGGCGGGGC	miRNA	miR-2981
t0028738	18	8	CGCGGGTCGGGGGGCGGG	miRNA	miR-2981
t0028774	22	8	TTCAAGAAATTCAGGATAGGTT	miRNA	miR-26b

t0028826	22	8	TTCAAGTAATTCAGGATCGGTT	miRNA	miR-26b
t0028903	20	8	TCAAGTAATCCAGGATAGGC	miRNA	miR-26a
t0028980	22	8	TTCGAGTAATCCAGGATAGGCT	miRNA	miR-26a
t0028585	21	8	AATTGCACTTGTCTCGGTCTG	miRNA	miR-25
t0028742	22	8	CATTGCACTTGACTCGGTCTGA	miRNA	miR-25
t0028442	27	8	CATTGCACTTGTCTCGGTCTGAAGTAG	miRNA	miR-25
t0028785	23	8	TGGCTCAGTTCAGCAGGCACAGT	miRNA	miR-24
t0028664	19	8	TCACATTGCCAGGGATTTT	miRNA	miR-23a
t0028381	20	8	AAGCTTCCAGTTGAAGAACT	miRNA	miR-22-3p
t0028650	19	8	AGAGTTGAGTCTGGACGTC	miRNA	miR-219-1-3p
t0028940	24	8	AGTGAGATTGTTGCATATTTACTT	miRNA	miR-2162-5p
t0028964	23	8	CAACGGCATCCCAAAAGCAGCTG	miRNA	miR-191
t0028600	22	8	CAACGCAATCCCAAAAGCAGCT	miRNA	miR-191
t0028822	22	8	CAACGGAGTCCCAAAAGCAGCT	miRNA	miR-191
t0028719	22	8	CAACGGAACCCCAAAAGCAGCT	miRNA	miR-191
t0028914	22	8	CAACGGAATCCCAGAAGCAGCT	miRNA	miR-191
t0028779	22	8	CAACGGAATCCCAAAAGTAGCT	miRNA	miR-191
t0028845	22	8	CAACGGAATCACAAAAGCAGCT	miRNA	miR-191
t0028857	23	8	AACGGAATCCCAAAAGAAGCTGA	miRNA	miR-191
t0028595	27	8	TGGAGAGAAAGGCAGTTCCTGAAAAAA	miRNA	miR-185
t0028398	23	8	TGGAGAGAAAGGCAGTTGCTGAA	miRNA	miR-185
t0028764	21	8	TGCAGAGAAAGGCAGTTCCTG	miRNA	miR-185
t0028477	19	8	GGAGAGAAAGGCAGTTCCT	miRNA	miR-185
t0028744	23	8	TGGAGAGAAAGGCAGTACCTGAA	miRNA	miR-185
t0028326	21	8	TGGAGAGAAAGGCAGTTCCTC	miRNA	miR-185
t0028354	23	8	TGGAGAGAAAGGCAGGTCCTGAT	miRNA	miR-185
t0028387	23	8	TGGAGAGAAAGGCCGTTCCTGAA	miRNA	miR-185
t0028542	20	8	TGGAGAGAAAGGCGGTTCCT	miRNA	miR-185
t0028555	20	8	TGGGGAGAAAGGCAGTTCCT	miRNA	miR-185
t0028597	21	8	AGACCTACTTATTTACCAACA	miRNA	miR-1839-3p
t0028722	25	8	TATGGCACTGGTAGAATTCACTTTT	miRNA	miR-183
t0028966	22	8	TGAGGGAGTAGGTTGTATAGTA	miRNA	miR-1827
t0028325	20	8	TGAGGGAGTAGATTGTGTAG	miRNA	miR-1827
t0028472	21	8	TGAGGGAGTAGATTGTATTGT	miRNA	miR-1827
t0028582	22	8	TGAGGGAGTAGATTGTATAGTG	miRNA	miR-1827
t0028584	24	8	TAGCAGCACGTAAATATTGGCTCG	miRNA	miR-16b
t0028593	20	8	GAAGCGGCGATGGCGGAGCT	miRNA	miR-1636
t0028682	29	8	GGGGGCGGGGAGCGGTCGGGCGGCGGC	miRNA	miR-1607
t0028694	26	8	GGCGGGGAGCGGTCGGGCGGCGGCGG	miRNA	miR-1607
t0028726	22	8	TAGAAGCACGTAAATATTGTCG	miRNA	miR-16
t0028852	22	8	TAGAAGAACGTAAATATTGGCG	miRNA	miR-16
t0028911	23	8	ATAGAAGCACGTAAATATTGGCG	miRNA	miR-16
t0028550	20	8	TAGCAGCACGTGAATATTGG	miRNA	miR-16
t0028930	20	8	TAGCAGCACGAAAATATTGG	miRNA	miR-16
t0028435	19	8	TAGCAGAACGTAAATATTG	miRNA	miR-16
t0028832	25	8	CCTGTAGCAGCACGTAAATATTGGC	miRNA	miR-16
t0028913	23	8	AGGTAGCAGCACGTAAATATTAA	miRNA	miR-16
t0029041	20	8	TAGCAGCACAACATGGTTTA	miRNA	miR-15b
t0028948	18	8	TAGAAGCACATCATGGTT	miRNA	miR-15b
t0028535	18	8	TAGCAGAACATCATGGTT	miRNA	miR-15b
t0028760	22	8	TAGTAGCACATCATGGTTTACA	miRNA	miR-15b
t0028993	19	8	TAGCAGCATATCATGTTTT	miRNA	miR-15b
t0028400	20	8	TAGCAGGACATCATGGTTTA	miRNA	miR-15b
t0028430	19	8	TAGCAGCACACCATGGTTT	miRNA	miR-15b
t0028538	20	8	TAGCAGCACATACTGGTTTG	miRNA	miR-15a
t0028754	22	8	TAGCAGCACATAATGGGTTGTG	miRNA	miR-15a
t0028772	23	8	TGAGAACTGAATTCTATAGGCTG	miRNA	miR-146b
t0028786	24	8	GGATATCATCATATACTGTAAGAA	miRNA	miR-144*

t0028894	23	8	AAGTGGATATCATCATATACTGT	miRNA	miR-144*
t0028897	21	8	ATACAGTATAGATGATGTAAA	miRNA	miR-144
t0028324	18	8	TACAGTATGGATGATGTA	miRNA	miR-144
t0028419	21	8	CCCATAAAGGAGAAAGCACTA	miRNA	miR-142
t0028612	23	8	CCCATAAAGTAGAAAGCACTATT	miRNA	miR-142
t0028752	20	8	CCCAAAAGTAGAAAGCACTA	miRNA	miR-142
t0028843	23	8	TACCACAGGGTAGAACGACGGAC	miRNA	miR-140
t0028893	24	8	TACCCCAGGGTAGAACCACGGACA	miRNA	miR-140
t0028978	22	8	TACCACAGGTTAGAACCACGGA	miRNA	miR-140
t0028541	22	8	ACCACAGGGTAGAACCATGGAT	miRNA	miR-140
t0028626	23	8	TTCCACAGGGTAGAACCACGGAT	miRNA	miR-140
t0028629	22	8	TACCACAGGGTAGAACTCCGGA	miRNA	miR-140
t0028681	22	8	ATCACAGGGTAGAACCACGGAC	miRNA	miR-140
t0028739	22	8	ACCACAGAGTAGAACCACGGAC	miRNA	miR-140
t0028677	22	8	ACCACAGGGGAGAACCACGGAG	miRNA	miR-140
t0028687	22	8	ACCACAGGGTGGAACCACGGAA	miRNA	miR-140
t0028401	22	8	ACCACAGGGAAGAACCACGGAG	miRNA	miR-140
t0028522	23	8	TACCACACGGTAGAACCACGGAC	miRNA	miR-140
t0028850	23	8	TACCACAGGGCAGAACCACGGAT	miRNA	miR-140
t0028334	23	8	ACCACAGGGTGGAACCACGGACA	miRNA	miR-140
t0028378	20	8	CACAGGGTAGAACCACGGAC	miRNA	miR-140
t0028390	21	8	ACCACAGGGTAGAACCACGAA	miRNA	miR-140
t0028391	23	8	TACCACAGGGTAGAACAAAGGAA	miRNA	miR-140
t0028393	21	8	ACCACAGGGTAGAAGCACGGA	miRNA	miR-140
t0028439	24	8	TAGCACAGGGTAGAACCACGGACA	miRNA	miR-140
t0028449	23	8	TACCACAGGGTAGCACCACGGAC	miRNA	miR-140
t0028524	22	8	TACCAGAGGGTAGAACCACGGA	miRNA	miR-140
t0028529	22	8	ACCACAGGGTAGCACCACGGAC	miRNA	miR-140
t0028552	22	8	ACCACAGGGTAGAACCACGGGT	miRNA	miR-140
t0028606	24	8	TACCACAGGGTAGAACCACGGCAA	miRNA	miR-140
t0028607	23	8	TACCACAGGGTAGAACCACGGTC	miRNA	miR-140
t0028634	24	8	TATCACAGGGTAGAACCACGGACA	miRNA	miR-140
t0028665	23	8	ACCACAGGGCAGAACCACGGACA	miRNA	miR-140
t0028679	23	8	GTGGGGGAGAGGCTGTTTTTTTT	miRNA	miR-1275
t0028691	21	8	TAAAGGGCTGACAGTGCAGAT	miRNA	miR-106b
t0028702	23	8	ACCAGCATTGTACAGGGCTATGA	miRNA	miR-103a
t0028711	23	8	AGCAGCAATGTACAGGGCTATGA	miRNA	miR-103a
t0028725	20	8	AGCAGCATCGTACAGGGCTA	miRNA	miR-103a
t0028731	21	8	GTACAGCACTGTGATAACTGA	miRNA	miR-101
t0028765	22	8	GTACAGTACTGTGGTAACTGAA	miRNA	miR-101
t0028787	22	8	GTGCAGTACTGTGATAACTGAA	miRNA	miR-101
t0028792	21	8	GTACAGAACTGTGATAACTGA	miRNA	miR-101
t0028865	21	8	GTATAGTACTGTGATAACTGA	miRNA	miR-101
t0028890	20	8	TACAGAACTGTGATAACTGA	miRNA	miR-101
t0028945	22	8	GTACAGTACTGCGATAACTGAA	miRNA	miR-101
t0028959	22	8	GTACAGTACTGTGATAGCTGAA	miRNA	miR-101
t0028318	22	8	GTACAGTACTGTGCTAACTGAA	miRNA	miR-101
t0028708	22	8	GTACGGTACTGTGATAACTGAA	miRNA	miR-101
t0028316	19	8	GAGGTAGTAGTTTGTGCTG	miRNA	let-7i
t0028769	22	8	TGAGGTAGTAGTCTGTGCTGTT	miRNA	let-7i
t0028793	21	8	GAGGTAGTAGTTTGTGCTGTT	miRNA	let-7i
t0028319	23	8	TGAGGGAGTAGTTTGTACAGTTA	miRNA	let-7g
t0028348	21	8	TGAGATAGTAGTTTGTACAGT	miRNA	let-7g
t0028379	21	8	TGAGGTAGTAGTTTGTACAGC	miRNA	let-7g
t0028389	21	8	TGAGGTAGTAGTTTGTACGGT	miRNA	let-7g
t0028429	20	8	TGAGGTAGTAGATTATATTT	miRNA	let-7f-5p
t0028496	24	8	TGAGGTAGTAGATTGTATATGTTT	miRNA	let-7f-5p
t0028558	28	8	TGAGGTAGTAGATTGTATAGTTCCGAAG	miRNA	let-7f

t0028659	21	8	TGAGGTAGTAGATATTATAGT	miRNA	let-7f
t0028967	22	8	AGGTAGTAGATTGTATAGTTGT	miRNA	let-7f
t0029009	23	8	TGAGGCAGTAGATTGTATAGTTT	miRNA	let-7f
t0028846	22	8	GGTAGTAGATTGTATAGTTTGA	miRNA	let-7f
t0028869	22	8	TGAGGTAGTAGTTTGTGTAGTT	miRNA	let-7f
t0029022	23	8	TGAGGTAGTAGATTGTACAGTTA	miRNA	let-7f
t0028566	23	8	TGAGGTAGTAGATTGTATAGGTA	miRNA	let-7f
t0028799	19	8	TGAGCTAGTAGATTGTATA	miRNA	let-7f
t0028817	23	8	TGAGGTAGTAGATTATATAGTTG	miRNA	let-7f
t0029015	23	8	TGGGGTAGTAGATTGTATAGTTA	miRNA	let-7f
t0028583	25	8	TGAGGTAGTAGATTGTATAGTTGAA	miRNA	let-7f
t0028590	21	8	AGAGGTAGAAGGTTGCATAGT	miRNA	let-7d
t0028364	22	8	AGAGGTAGTAGGTTGCTTAGTT	miRNA	let-7d
t0028375	20	8	AGAGGTAGTAGGTTGCATCG	miRNA	let-7d
t0028436	21	8	AGAGGTAGTAGGTTGCATAGG	miRNA	let-7d
t0028438	21	8	AGAGGTAGCAGGTTGCATAGT	miRNA	let-7d
t0028511	21	8	AGAGGTAGGAGGTTGCATAGT	miRNA	let-7d
t0028512	22	8	AGAGCTAGTAGGTTGCATAGTT	miRNA	let-7d
t0028559	25	8	TGAGGTAGTAGGGTGTATGGTTATC	miRNA	let-7c
t0028567	23	8	TGAGGTAGCAGGTTGTGTGGTTA	miRNA	let-7b
t0028649	20	8	TGCGGTAGTAGGTTGTGTGG	miRNA	let-7b
t0028854	20	8	TGAGGTAGTAGGTTGGGTGG	miRNA	let-7b
t0028963	22	8	GAGGTAGTAGGTTGTGTGGTTT	miRNA	let-7b
t0029040	19	8	TGAGGTAGTAGGTTGTGGG	miRNA	let-7b
t0028560	19	8	TGGGGTAGTAGGTTGTGTG	miRNA	let-7b
t0028570	19	8	TGAGGTAGTGGGTTGTATA	miRNA	let-7
t0028578	19	8	TGAGGAAGTAGGTTGTATA	miRNA	let-7
t0028690	21	8	TGACGTAGTAGGTTGTATAGT	miRNA	let-7
t0028750	20	8	TGAGATAGTAGGTTGTATAG	miRNA	let-7
t0028768	19	7	AGGTGGAAGGTTGTAGGCA	miRNA	miR-965-5p
t0028848	22	7	CAAAGTGCTGTTCGGGCAGGTA	miRNA	miR-93
t0028605	22	7	CAAAGGGCTGTTCGTGCAGGTA	miRNA	miR-93
t0028346	23	7	CAAAGTGCTGTTCGTGCAGGAAG	miRNA	miR-93
t0028411	21	7	GGGACGGGACGCGGTGCAGTA	miRNA	miR-92b*
t0028505	23	7	TATTGCACTTGCCCCGGCCTGTA	miRNA	miR-92a
t0028671	21	7	TATTGCACTTGTCACGGCCTG	miRNA	miR-92a
t0028831	22	7	TATTGCACTTGTCCCGGCATGT	miRNA	miR-92a
t0028875	22	7	TATTGCACTTGTCCCGACCTGT	miRNA	miR-92a
t0028462	20	7	TATTGCACTCGTCCCGGCAG	miRNA	miR-92
t0028500	23	7	CCTGGAGGACTGACGTGGAGAAG	miRNA	miR-739
t0028564	21	7	GAGGGCGCGCGGGTCGGGGCG	miRNA	miR-638
t0028701	19	7	TGTCTTACTCCCTCAGGCA	miRNA	miR-550a*
t0029637	19	7	AAAAGCAATCGCGGTTTTT	miRNA	miR-548h
t0029297	20	7	GCGAGGGTCCCGGGGCGGGG	miRNA	miR-5126
t0029390	21	7	CTTGCCATCTGGGCCATCATA	miRNA	miR-500b
t0029423	23	7	ACCTGTACTGAGCTGCCCCGAGA	miRNA	miR-486-5p
t0029384	28	7	TCCTGTACTGAGCTGCCCCGAGATGGCA	miRNA	miR-486-5p
t0029156	20	7	TCCTGTACTGGGCTGCCCCG	miRNA	miR-486-5p
t0029181	22	7	TCCTGTACTGAGCTGCCCCGCT	miRNA	miR-486-5p
t0029365	21	7	GCCTGTACTGAGCTGCCCCGA	miRNA	miR-486-5p
t0029809	21	7	TCCTGTACTGTGCTGCCCCGA	miRNA	miR-486-5p
t0029315	21	7	TCCTGTAGTGAGCTGCCCCGA	miRNA	miR-486-5p
t0029479	22	7	TCCTGTACTGATCTGCCCCGAG	miRNA	miR-486-5p
t0029067	22	7	TCCTGTACTGAGCTGCCCGGAG	miRNA	miR-486-5p
t0029229	26	7	TCCTGTACTGAGCTGCCCCGACGGCT	miRNA	miR-486-5p
t0029347	23	7	TCCTGTAATGAGCTGCCCCGAGA	miRNA	miR-486-5p
t0029716	20	7	TCCTGTAATGAGCTGCCCCG	miRNA	miR-486-5p
t0029081	23	7	CGTAGAGCAGGGAGCAGGAAGCT	miRNA	miR-4732-5p

t0029079	20	7	GAGAAGGGTCGGGGCGGTTA	miRNA	miR-4516
t0029098	25	7	CGGGGGGAGAAGGGTCGGGGCGGCA	miRNA	miR-4516
t0029353	21	7	TGAGGGAGTAGGTTGTGGGGT	miRNA	miR-4510
t0029370	21	7	TGAGGGAGTAGGTTGTGTGGA	miRNA	miR-4510
t0029523	25	7	AAACCGTTACCATTACTGAGTTCGT	miRNA	miR-451
t0029566	24	7	AAACCGTTACCATTACTGCGTTTA	miRNA	miR-451
t0029581	25	7	AAACCATTACCATTACTGAGTTTAG	miRNA	miR-451
t0029684	22	7	TCGAATCCGAGTCACGGCACCC	miRNA	miR-4454
t0029711	20	7	AGGAGCATTGTACAGGGCTA	miRNA	miR-4289
t0029732	22	7	TGAGGGGCAGAGTGCGAGACTT	miRNA	miR-423-5p
t0029747	21	7	TGAGGGGAAGAGAGCGAGAAT	miRNA	miR-423-5p
t0029780	20	7	TGGCAGAGAGCGAGACTTTT	miRNA	miR-423-5p
t0029298	19	7	TGAGGGGCAGAGAGCGGGA	miRNA	miR-423-5p
t0029493	23	7	TGAGGGGCAGAGAGGGAGACTTA	miRNA	miR-423-5p
t0029800	19	7	TGGGGGGCAGAGAGCGAGA	miRNA	miR-423-5p
t0029612	24	7	TGAGGGGCAGAAAGCGAGACTTTT	miRNA	miR-423-5p
t0029759	23	7	ACCCGGGGGGCCGGCGGCGGCGG	miRNA	miR-3885-5p
t0029302	20	7	CGGCGGCGTCGGCGGCGGCG	miRNA	miR-3885-5p
t0029488	22	7	CGGCGGCGGCGGGGGTGTGGGG	miRNA	miR-3885-5p
t0029802	23	7	ACTGGACTTGGAGCCAGAAGGCA	miRNA	miR-378
t0029248	23	7	ACTGGACTTGGAGACAGAAGGCA	miRNA	miR-378
t0029730	25	7	CTTATCAGATTGTATTGTAATTATT	miRNA	miR-374a*
t0029159	18	7	CGGGGGTGGGGTCGGCGG	miRNA	miR-3621
t0029255	22	7	TGAGGATGGATAGCAAGGAAGC	miRNA	miR-3605-5p
t0029268	23	7	TGAAGACTGAAGTGGAGAAGGGA	miRNA	miR-3526
t0029362	22	7	GAAGACTGAAGTGGAGAAGGGG	miRNA	miR-3526
t0029419	25	7	TTGAAGACTGAAGTGGAGAAGGGGT	miRNA	miR-3526
t0029462	21	7	AAAGCTGGGTTGAGAGGGCGC	miRNA	miR-320d
t0029767	23	7	AAAAGCTGGGTTGGGAGGGCGTA	miRNA	miR-320d
t0029131	21	7	AAAAGCTGGGTTGAGAGGGCC	miRNA	miR-320d
t0029150	22	7	AAACGCTGGGTTGAGAGGGCGT	miRNA	miR-320d
t0029288	23	7	AAAACCTGGGTTGAGAGGGCGTA	miRNA	miR-320d
t0029503	24	7	AAAAGCTGGGTTGAGAGGGCGGGA	miRNA	miR-320d
t0029757	23	7	AAAAACTGGGTTGAGAGGGCGTT	miRNA	miR-320d
t0029271	23	7	AAAAGCTGGGTTGAGAGGGCTTA	miRNA	miR-320d
t0029392	22	7	AAAAGCTGGGTTGAGAGGGAAA	miRNA	miR-320d
t0029115	23	7	AAAAGCCGGGTTGAGAGGGCGTT	miRNA	miR-320c
t0029307	21	7	AAAAGCTGGGTCGAGAGGGCG	miRNA	miR-320a
t0029463	23	7	AAAAGCTGGATTGAGAGGGCGAA	miRNA	miR-320a
t0029549	23	7	AAAGCTGGGTTGAGAGGGCGATT	miRNA	miR-320a
t0029265	23	7	AAGAGCTGGGTTGAGAGGGCGAA	miRNA	miR-320a
t0029358	23	7	AAAAGCTGGGTTGAGGGGGCGAT	miRNA	miR-320a
t0029425	23	7	AAAAGCTGGGTCGAGAGGGCGAA	miRNA	miR-320a
t0029429	22	7	AAAAGCTGGGTTGACAGGGCGA	miRNA	miR-320a
t0029450	25	7	AAAAGCTGGGTTGAGAGGGCGAATA	miRNA	miR-320a
t0029536	23	7	AATCTGAGAAGGCGCACCAGGTT	miRNA	miR-3200-5p
t0029658	23	7	AATCTGAGAAGGCGCACAAGGAT	miRNA	miR-3200-5p
t0029659	23	7	AATCTGAGAAGGAGCACAAGGTT	miRNA	miR-3200-5p
t0029756	26	7	GGCTGGTCCGAGTGCAGTGGTGTTTA	miRNA	miR-3135b
t0029444	24	7	TGTAAACATCCTCGACTGGAAGCT	miRNA	miR-30a-5p
t0029182	22	7	TTCAAGTAATCCAGGAAAGGCT	miRNA	miR-26a
t0029357	22	7	TTCAGGTAATCCAGGATAGGCT	miRNA	miR-26a
t0029434	22	7	TTAAAGTAATCCAGGATAGGCT	miRNA	miR-26a
t0029446	22	7	TTTAAGTAATCCAGGATAGGCT	miRNA	miR-26a
t0029517	20	7	AGGCGGAGACTTGGGCACTT	miRNA	miR-25*
t0029578	22	7	CATTGGACTTGTCTCGGTCTGA	miRNA	miR-25
t0029616	21	7	CATTACACTTGTCTCGGTCTG	miRNA	miR-25
t0029804	21	7	CATTGCACTTGTCCCGGTCTG	miRNA	miR-25

t0029262	22	7	CATTGCACTTGTCTCGGTCCGA	miRNA	miR-25
t0029484	21	7	CATTGCACTCGTCTCGGTCTG	miRNA	miR-25
t0029640	21	7	CGTTGCACTTGTCTCGGTCTG	miRNA	miR-25
t0029555	21	7	CATTGCACTTGCCTCGGTCTG	miRNA	miR-25
t0029675	22	7	CATTGCACTTGTCACGGTCTGA	miRNA	miR-25
t0029148	22	7	CATAGCACTTGTCTCGGTCTGA	miRNA	miR-25
t0029316	21	7	AAGCTGCCAGTTGAAGAACTG	miRNA	miR-22-3p
t0029412	25	7	TGGAATGTAAGGAAGTGGGTGGATC	miRNA	miR-206
t0029742	19	7	TTCCTATGCATATACTTCT	miRNA	miR-202-5p
t0029695	18	7	TGAGCTAGATTGTATAGT	miRNA	miR-1961
t0029143	23	7	TGACCTATGAATTGACAGCCATT	miRNA	miR-192
t0029222	21	7	CTGACCTATGAATTGACCGCA	miRNA	miR-192
t0029360	22	7	CAACGAAATCCCAAAAGCAGCT	miRNA	miR-191
t0029525	22	7	CAGCGGAATCCCAAAAGCAGCT	miRNA	miR-191
t0029560	22	7	CAACGGAATCCCAAAAGCAGCG	miRNA	miR-191
t0029567	22	7	AACGGAATCCCAAAAGCAGATG	miRNA	miR-191
t0029583	23	7	CAACGGAATCCCAAAAGCGGCTG	miRNA	miR-191
t0029729	21	7	CAACGGAATCCCAAAAGAAGC	miRNA	miR-191
t0029791	23	7	CAACAGAATCCCAAAAGCAGCTG	miRNA	miR-191
t0029458	22	7	CAACGGAATCCCCAAAGCAGCT	miRNA	miR-191
t0029180	23	7	TGGAGAGAAAGGCAGTTCCAGAA	miRNA	miR-185
t0029065	20	7	TGGAGAGAAAGGCCGTTCCT	miRNA	miR-185
t0029676	21	7	TGGAGAGAAAGGCGTTTCCTG	miRNA	miR-185
t0029361	23	7	TGAAGAGAAAGGCAGTTCCTGAA	miRNA	miR-185
t0029706	22	7	TGGAGAGAAAAGAAGTTCCTGA	miRNA	miR-185
t0029114	23	7	TGGAGAGCAAGGCAGTTCCTGAT	miRNA	miR-185
t0029250	20	7	TGGAGTGAAAGGCAGTTCCT	miRNA	miR-185
t0029264	21	7	AGGCAGATAGAACAGGTCTTG	miRNA	miR-1839
t0029295	18	7	TGAGGGAGTAGATTGTAT	miRNA	miR-1827
t0029389	22	7	TGAGGGAGTAGATTGTATATTT	miRNA	miR-1827
t0029587	22	7	TGAGGGAGTAGGTTGTATGGTT	miRNA	miR-1827
t0029655	23	7	TGAGGGAGTAGGTTGTATAGTTG	miRNA	miR-1827
t0029796	18	7	TTTGGCAATGGTAGAACT	miRNA	miR-182
t0029352	20	7	TTTGGCAATGGTAGAATTCA	miRNA	miR-182
t0029396	20	7	AACATTCAACGCTGTCGGGG	miRNA	miR-181a
t0029509	20	7	TCGGGGCGGCGGCGGCGGGG	miRNA	miR-1777a
t0029522	21	7	AGCAGCACGTAAATATTGGCA	miRNA	miR-16b
t0029653	25	7	GCGGGGAGCGGTCGGGCGGCGGCGG	miRNA	miR-1607
t0029693	27	7	GGCGGGGAGCGGTCGGGCGGCGGCGGT	miRNA	miR-1607
t0029818	19	7	GGGGGCGGGGAGCGGTCGG	miRNA	miR-1607
t0029226	19	7	GGCGGGGAGCGGTCGGGCG	miRNA	miR-1607
t0029212	21	7	TAGCAGCACGTAAATATTGAC	miRNA	miR-16
t0029393	20	7	TAGCAGCACGTCAATATTGG	miRNA	miR-16
t0029542	20	7	TAGGAGCACGTAAATATTGG	miRNA	miR-16
t0029682	20	7	TAGCACCACGTAAATATTGG	miRNA	miR-16
t0029601	24	7	TAGCAGCACGTAAATATTGGAGAA	miRNA	miR-16
t0029677	20	7	TAGCAGCACGTAAATATTTC	miRNA	miR-16
t0029092	20	7	TAGCAGCCCGTAAATATTGG	miRNA	miR-16
t0029697	23	7	TAGCAGAACGTAAATATTGGCGA	miRNA	miR-16
t0029698	19	7	TAACAGCACATCATGGTTT	miRNA	miR-15b
t0029310	22	7	TAGCAGCACATCACGGTTTACA	miRNA	miR-15b
t0029322	18	7	TAGCAGCAAATCATGGTT	miRNA	miR-15b
t0029600	19	7	TAGCAACACATCATGGTTT	miRNA	miR-15b
t0029753	22	7	TAGCAGGACATCATGGTTTACA	miRNA	miR-15b
t0029178	22	7	TAGCAGCACATCATGATTTACA	miRNA	miR-15b
t0029417	19	7	TAGCAGCACATCATGGTCT	miRNA	miR-15b
t0029445	22	7	TAGCACCACATCATGGTTTACA	miRNA	miR-15b
t0029532	21	7	TAGCAGCACATAGTGGTTTGT	miRNA	miR-15a

t0029544	20	7	TAGCAGCAAATAATGGTTTG	miRNA	miR-15a
t0029690	21	7	TAGCAGCACATAATGGTCTGT	miRNA	miR-15a
t0029795	21	7	TAGCAGCAAATAATGGTTTGT	miRNA	miR-15a
t0029823	22	7	TAGCAGCACATAATGGTTTGCG	miRNA	miR-15a
t0029069	21	7	TAGCAGCACCTAATGGTTTGT	miRNA	miR-15a
t0029072	21	7	TAGCAGCACATAATGGCTTGT	miRNA	miR-15a
t0029203	20	7	TAGCAGCACATAAAGGTTTG	miRNA	miR-15a
t0029284	20	7	TAGCAGAACATAATGGTTTA	miRNA	miR-15a
t0029332	21	7	TAGCAGTACATAATGGTTTGT	miRNA	miR-15a
t0029480	21	7	TAACAGCACATAATGGTTTGT	miRNA	miR-15a
t0029541	26	7	TCTCCCAACCCTTGTACCAGTGAATC	miRNA	miR-150
t0029758	23	7	GGATATCATCATATACTGTAAGG	miRNA	miR-144*
t0029059	21	7	TACAGTATAGATGATGTACAT	miRNA	miR-144
t0029141	20	7	CCCATAAAGTAGAAAGCAAT	miRNA	miR-142
t0029247	23	7	TACCACAGGGTGGAACCACGGAT	miRNA	miR-140
t0029375	23	7	ACCATAGGGTAGAACCACGGACG	miRNA	miR-140
t0029395	23	7	TACCACAGGGTAGAACCACAGAT	miRNA	miR-140
t0029569	23	7	TACCACAGGGTAGAACCACTGAC	miRNA	miR-140
t0029630	24	7	TGCCACAGGGTAGAACCACGGACA	miRNA	miR-140
t0029643	24	7	TACCACAGGGTAGAACAAAGGACA	miRNA	miR-140
t0029656	21	7	ACCAGAGGGTAGAACCACGGA	miRNA	miR-140
t0029740	23	7	TACCTCAGGGTAGAACCACGGAC	miRNA	miR-140
t0029807	22	7	ACCACAAGGTAGAACCACGGAC	miRNA	miR-140
t0029327	24	7	TACCACAGGGTAGAACCAGGGACA	miRNA	miR-140
t0029300	22	7	ACCAAAGGGTAGAACCACGGAA	miRNA	miR-140
t0029570	23	7	TACGACAGGGTAGAACCACGGAC	miRNA	miR-140
t0029651	22	7	TACCACAGGGTAGAAACAAGGA	miRNA	miR-140
t0029048	23	7	TACCACAGGGTAGAACCCCGGCA	miRNA	miR-140
t0029062	22	7	ACCACAGGGTGGAACCACGGAT	miRNA	miR-140
t0029075	24	7	TACCACAGGGTAGAACCCCGGCCA	miRNA	miR-140
t0029107	23	7	TACCACAGGGTAGAGCCACGGAA	miRNA	miR-140
t0029168	22	7	AACACAGGGTAGAACCACGGAC	miRNA	miR-140
t0029188	22	7	ACCACAGGGTAGAACCACAGAA	miRNA	miR-140
t0029201	21	7	ACCACAGGGTAGAACCACGCA	miRNA	miR-140
t0029225	22	7	ACCACAGGATAGAACCACGGAC	miRNA	miR-140
t0029336	22	7	ACCACAGGGTAGAACCACCGAC	miRNA	miR-140
t0029341	22	7	ACCGCAGGGTAGAACCACGGAA	miRNA	miR-140
t0029342	24	7	TCCCACAGGGTAGAACCACGGACA	miRNA	miR-140
t0029349	23	7	ACCACAGGGTAGAACCACGGCAA	miRNA	miR-140
t0029372	22	7	GCCACAGGGTAGAACCACGGAT	miRNA	miR-140
t0029415	23	7	TACCACAGGGTAGAACCGCGGAT	miRNA	miR-140
t0029440	19	7	CAGTGCAATGTTAAAAGGC	miRNA	miR-130a
t0029494	19	7	TACGTAGATATATATGTAT	miRNA	miR-1277
t0029497	22	7	CGGATGAGCAAAGAAAGGGGTT	miRNA	miR-1255b
t0029514	22	7	AGAAGCATTGTACAGGGCTATA	miRNA	miR-107
t0029568	20	7	CCGCACTGTGGGTATTTGCT	miRNA	miR-106b*
t0029577	20	7	TAAAGGGCTGACAGTGCAGA	miRNA	miR-106b
t0029586	20	7	TAAAGTGCTGACAGTGAAGA	miRNA	miR-106b
t0029632	20	7	TAAAGTGCTGACAGAGCAGA	miRNA	miR-106b
t0029661	23	7	AGCAGCATTGTACTGGGCTATGA	miRNA	miR-103a
t0029670	21	7	AGCAGGATTGTACAGGGCTAT	miRNA	miR-103a
t0029731	21	7	AGCACCATTGTACAGGGCTAT	miRNA	miR-103a
t0029739	22	7	AGCAGCATTGTACAGGGCTAGG	miRNA	miR-103a
t0029805	23	7	CGCAGCATTGTACAGGGCTATGA	miRNA	miR-103a
t0029195	20	7	AGCAGGATTGTACAGGGCTA	miRNA	miR-103a
t0029381	23	7	AGAAGCATTGTACAGGGCTATGT	miRNA	miR-103a
t0029101	20	7	CACAGTACTGTGATAACTGA	miRNA	miR-101c
t0029565	20	7	TACAGTACTATGATAACTGA	miRNA	miR-101b

t0029692	22	7 GTACAGTACTGTGATACCTGAA	miRNA	miR-101
t0029089	21	7 TACAGTACTGTGTTAACTGAA	miRNA	miR-101
t0029371	22	7 TACAGTACTGTGATAAATGAAG	miRNA	miR-101
t0029551	21	7 TACAGTACTGTGCTAACTGAA	miRNA	miR-101
t0029317	21	7 TACAGTACTGTGATAACTTAA	miRNA	miR-101
t0029326	21	7 TACAATACTGTGATAACTGAA	miRNA	miR-101
t0029387	21	7 GTACAGTACTGGGATAACTGA	miRNA	miR-101
t0029482	20	7 TACGGTACTGTGATAACTGA	miRNA	miR-101
t0029527	22	7 GTACAGTACTGAGATAACTGAA	miRNA	miR-101
t0029792	21	7 TACAGTACTTTGATAACTGAA	miRNA	miR-101
t0029803	19	7 TGAGGTAGTAGATTGAATA	miRNA	let-7k
t0029368	22	7 AGAGGTAGTAGTTTGTGCTGTT	miRNA	let-7i
t0029518	22	7 TGAGGTAGTAATTTGTGCTGTT	miRNA	let-7i
t0029074	27	7 TGAGGTAGTAGTTTGTGCTGTTCGGCA	miRNA	let-7i
t0029220	22	7 TGAGGTAGTAGTTTGTGCTGGT	miRNA	let-7i
t0029228	21	7 TGAGGAAGTAGTTTGTGCTGT	miRNA	let-7i
t0029239	22	7 TGAGGTAGTAGTTTGAGCTGTT	miRNA	let-7i
t0029293	21	7 TGAGGTAGTGGTTTGTGCTGT	miRNA	let-7i
t0029402	21	7 TGAGGTAGTAGGTTGTGCTGT	miRNA	let-7i
t0029545	21	7 TGCGGTAGTAGTTTGTACAGT	miRNA	let-7g
t0029627	21	7 TGAGGTAGGAGTTTGTACAGT	miRNA	let-7g
t0029654	21	7 TGAGGTAGTAGTTTATACAGT	miRNA	let-7g
t0029734	20	7 TGAGGGAGTAGTTTGTACAG	miRNA	let-7g
t0029146	21	7 TGAGGTAGTAGTTTGTCCAGT	miRNA	let-7g
t0029090	22	7 TGAGGTAGTAGTTTGTACAATT	miRNA	let-7g
t0029193	22	7 TGAGGTAGTAGATTGTATTCTT	miRNA	let-7f-5p
t0029253	22	7 TGAGGTAGTGGATTGTATAGGT	miRNA	let-7f-5p
t0029436	21	7 TGAGGTAGTAGATTGTAGTTA	miRNA	let-7f-5p
t0029703	22	7 TGAGGTAGTAGATTGTACAGTA	miRNA	let-7f
t0029722	23	7 TGAGGTAGCAGATTGTATAGTTT	miRNA	let-7f
t0029723	25	7 TGAGGTAGTAGATTGTATAGTAAAA	miRNA	let-7f
t0029778	23	7 TGAGGTAGTAGCTTGTATAGTTA	miRNA	let-7f
t0029348	25	7 TGAGGTAGTATATTGTATAGTTATC	miRNA	let-7f
t0029376	23	7 TGAGGTAGTAGATTGTATATTTT	miRNA	let-7f
t0029408	19	7 TGAGATAGTAGATTGTATA	miRNA	let-7f
t0029474	23	7 TGAGGTAGTAGACTGTATAGTTT	miRNA	let-7f
t0029546	23	7 TGAGGTAGTAGCTTGTATAGTTG	miRNA	let-7f
t0029552	23	7 TGAGGTAGTAGATTGTATAGTCA	miRNA	let-7f
t0029060	19	7 TAAGGTAGTAGATTGTATA	miRNA	let-7f
t0029122	22	7 CGAGGTAGTAGATTGTATAGTA	miRNA	let-7f
t0029460	23	7 TGAGGTAGAAGATTGTATAGTTG	miRNA	let-7f
t0029103	23	7 TGAGGTAGTAGATTATATAGTTA	miRNA	let-7f
t0029117	20	7 TGAGGTAGTAGATAGTATAG	miRNA	let-7f
t0029124	24	7 CTGAGGTAGTAGATTGTATAGTTA	miRNA	let-7f
t0029206	20	7 TGAGGTAGTAGATATTATAG	miRNA	let-7f
t0029299	23	7 TGAGGTAGTAGATTGTATGGTTA	miRNA	let-7f
t0029319	23	7 TGAGGTAGTAGATTGTATGGTTG	miRNA	let-7f
t0029320	20	7 AGCGGTAGTAGGTTGCATAG	miRNA	let-7d
t0029339	19	7 AGAGGGAGTAGGTTGCATA	miRNA	let-7d
t0029374	20	7 AGAGGTAGTAGGTTGCACAG	miRNA	let-7d
t0029379	22	7 AGAGGTAGTAGGTTGCATAGTG	miRNA	let-7d
t0029399	20	7 AGAGGTAGTAGGATGCATAG	miRNA	let-7d
t0029426	21	7 AGAGGTAGTAGTTTGCATAGT	miRNA	let-7d
t0029465	21	7 AGGGGTAGTAGGTTGCATAGT	miRNA	let-7d
t0029499	20	7 GGAGGTAGTAGGTTGCATAG	miRNA	let-7d
t0029622	22	7 TGAGGTAGGAGGTTGTGTGGTA	miRNA	let-7b
t0029667	22	7 TGCGGTAGTAGGTTGTGTGGTA	miRNA	let-7b
t0029713	22	7 TGAGGTAGCAGGTTGTGTGGTA	miRNA	let-7b

t0029724	23	7	TGAGGTAGCAGGTTGTGTGGTTT	miRNA	let-7b
t0029735	23	7	TGAGGTAGTAGGTTGTGCGGTTT	miRNA	let-7b
t0029140	22	7	TGAGGTAGTAGGTTGCGTGGTA	miRNA	let-7b
t0029161	26	7	TGAGGTAGTAGGTTGTATATTTAATC	miRNA	let-7
t0029190	19	7	CGAGGTAGTAGGTTGTATA	miRNA	let-7
t0029230	20	7	GGAGGTAGTAGGTTGTATAG	miRNA	let-7
t0029386	19	7	TGAGGCAGTAGGTTGTATA	miRNA	let-7
t0029432	20	7	TGAGGTACTAGGTTGTATAG	miRNA	let-7
t0029644	21	6	CAAAGTGCTGTTCGTGCCGGT	miRNA	miR-93
t0029801	23	6	TATTGCACTTGTCCCGGCCTTTA	miRNA	miR-92b
t0029304	22	6	TGGAGAGAAAGGAAGTTTCTGA	miRNA	miR-765
t0029331	23	6	TGCGGGGCTAGGGCTAACAGCAT	miRNA	miR-744
t0029391	22	6	GACTATAGAACTTTCCCCCTCA	miRNA	miR-625*
t0029491	21	6	TTATGGTTTGCCTGGGGCTGA	miRNA	miR-584
t0029507	19	6	TGCAAAAGTGATCGTGGTT	miRNA	miR-548t
t0029705	20	6	GCGAGGGGCCCGGGGCGGGG	miRNA	miR-5126
t0029191	24	6	TCTGGGCACAGGCGGATGGACCGT	miRNA	miR-5107
t0029599	22	6	TCTGGGCACAGGCGGAAGGACA	miRNA	miR-5107
t0029786	22	6	TCTGGGCACAGGCGGATGGGCA	miRNA	miR-5107
t0029810	20	6	TAATCCTTGCTACCTGGGTG	miRNA	miR-500a
t0029816	22	6	TGCTGTACTGAGCTGCCCCGAG	miRNA	miR-486-5p
t0030066	23	6	TCCTGTACTGAGCTGCCACGAGT	miRNA	miR-486-5p
t0030651	23	6	TCCTGTACTGCGCTGCCCCGAGA	miRNA	miR-486-5p
t0029831	22	6	TCTTGTACTGAGCTGCCCCGCG	miRNA	miR-486-5p
t0030007	21	6	TCGTGTACTGAGCTGCCCCGA	miRNA	miR-486-5p
t0030617	32	6	TCCTGTACTGAGCTGCCCCGAGGTAGAAT	miRNA	miR-486-5p
t0030376	23	6	TCCTGGACTGAGCTGCCCCGAGA	miRNA	miR-486-5p
t0030639	22	6	TCCTGTACAGAGCTGCCCCGAG	miRNA	miR-486-5p
t0030477	23	6	TCATGTACTGAGCTGCCCCGAGA	miRNA	miR-486-5p
t0030029	21	6	TCCTCTACTGAGCTGCCCCGA	miRNA	miR-486-5p
t0030225	22	6	TCCTGTACTGAGCTGCCCAGAA	miRNA	miR-486-5p
t0030609	23	6	TCCTGTACTGAGCTGCGCCGAGA	miRNA	miR-486-5p
t0030551	23	6	ACTGGGAAGAGGAGCTGAGGGAT	miRNA	miR-4646-5p
t0029825	23	6	TGAGGTAGTAGGGTGTGTGGTAA	miRNA	miR-4510
t0029838	22	6	TGAGGGAGTAGGTTGTGTAGTT	miRNA	miR-4510
t0029990	19	6	CGTTACCATTACTGAGTTT	miRNA	miR-451
t0029993	22	6	ACCGTTACCATTACTGAGTTAA	miRNA	miR-451
t0030164	21	6	AAACCTTTAACATTACTGAGT	miRNA	miR-451
t0030240	26	6	AAACAGTTACCATTACTGAGTTTAGT	miRNA	miR-451
t0030289	27	6	AAATACGTTACCATTACTGAGTTAAAC	miRNA	miR-451
t0030292	20	6	TAACCGTTACCATTACTGAG	miRNA	miR-451
t0030304	25	6	AAACCGTTACCATTACTGCGTTTAG	miRNA	miR-451
t0030442	23	6	AAACCGTTACCTTTACTGAGTTT	miRNA	miR-451
t0030514	20	6	AAACCGTTACCATTACTGTT	miRNA	miR-451
t0030562	20	6	AAACCGTTACCATTACGGAG	miRNA	miR-451
t0029906	21	6	AAACCGTTACCATTTCTGAGT	miRNA	miR-451
t0030338	19	6	AAACCGTTACCATTAATGA	miRNA	miR-451
t0030418	24	6	AAACCGTTACCATTACTGAGGTTA	miRNA	miR-451
t0029879	23	6	AAAAGCTGGGCTGAGAGGGCGAT	miRNA	miR-4429
t0030009	23	6	AAAAGCTGGGCTGAGAGGGCGTT	miRNA	miR-4429
t0030056	19	6	CATCGGGAATGTCGTGTCC	miRNA	miR-425*
t0030105	20	6	TGAGGGGCAGAGAGCGAGCC	miRNA	miR-423-5p
t0030174	23	6	TGAGGGGCAGAGATCGAGACTTA	miRNA	miR-423-5p
t0030196	23	6	TGAGGGTCACAGAGCGAGACTTT	miRNA	miR-423-5p
t0030341	23	6	TGAGTGGCAGAGAGCGAGAATTT	miRNA	miR-423-5p
t0030343	21	6	TGAGGGGCAGAGAGCGAGATA	miRNA	miR-423-5p
t0030345	24	6	TGAGGGGTTGAGAGCGAGACTTTT	miRNA	miR-423-5p
t0030508	23	6	TGAGGGGCAGAGAGCAAGACTTA	miRNA	miR-423-5p

t0030579	24	6	TGAGGGGCAGAGAGCGCGACTTTA	miRNA	miR-423-5p
t0030645	19	6	TGAGGGGCAGAGGGCGAGA	miRNA	miR-423-5p
t0030657	22	6	TCAGGGGCAGAGAGCGAGACTT	miRNA	miR-423-5p
t0030502	19	6	TGAGGGGCAGAGAGCGAGC	miRNA	miR-423-5p
t0030641	23	6	TGAGGGGCAGAGAGCGAGATTTA	miRNA	miR-423-5p
t0030117	19	6	TGAGGGGAAGAGAGCGAGA	miRNA	miR-423-5p
t0029923	24	6	TGAGGGGCAGAGCGCGAGACTTTT	miRNA	miR-423-5p
t0029929	23	6	TGAGGGGTAGAGAGCGAGACTTA	miRNA	miR-423-5p
t0029949	20	6	TGAGGGGAAGAGAGCGAGAC	miRNA	miR-423-5p
t0030051	21	6	GGAGGTAGTAGTTTGTACAGT	miRNA	miR-3962
t0030127	26	6	GAAACGGACAAGGGGAATCCGACTGT	miRNA	miR-3896-3p
t0030154	22	6	GGGGGGCCGGCGGCGGCGGCGA	miRNA	miR-3885-5p
t0030288	20	6	TCGGAGGGCGGCGGCGGCGG	miRNA	miR-3885-5p
t0030314	21	6	GGGCGGCGGCGGGGGGAGAAG	miRNA	miR-3885-5p
t0030317	18	6	GGGGGTGGGGTCGGCGGG	miRNA	miR-3621
t0030347	24	6	TGAAGACTGAAGGGGAGAAGGGTT	miRNA	miR-3526
t0030476	29	6	AACTTTGAAGACTGAAGTGGAGAAGGGG	miRNA	miR-3526
t0030479	24	6	GAAGACTGAAGTGGAGAAGGGGTT	miRNA	miR-3526
t0030499	21	6	AACTTTGAAGACTGAAGGGGA	miRNA	miR-3526
t0030567	20	6	TGAGTAGTAGGTTGTATAGT	miRNA	miR-352
t0030632	20	6	CACACAGAAATCGCACCCGT	miRNA	miR-342-3p
t0030660	24	6	TCTCACACAGAAATCGCACCCGCC	miRNA	miR-342-3p
t0030266	22	6	TCTCACACAGAAATCACACCCG	miRNA	miR-342-3p
t0030137	22	6	TCTCACACAGAAATCGCACCAG	miRNA	miR-342-3p
t0029878	23	6	AAAAGCTGGGTTGAGGGGGCGTT	miRNA	miR-320d
t0029916	22	6	AAGAGCTGGGTTGAGAGGGCGT	miRNA	miR-320d
t0030297	23	6	ACAAGCTGGGTTGAGAGGGCGTA	miRNA	miR-320d
t0030026	23	6	AAAAGCTGGGTTGAGAGGGGGTA	miRNA	miR-320d
t0030078	22	6	AAAAGCTGGGTTGAGAGAGCGT	miRNA	miR-320d
t0030140	24	6	AAAAGCTGGGTTGAGAGGGCGCAA	miRNA	miR-320d
t0030346	23	6	AAAAGCTGGGTTGACAGGGCGTA	miRNA	miR-320d
t0030603	23	6	AAAAGCTGGGTCGAGAGGGCGTA	miRNA	miR-320d
t0030185	23	6	AGAAGCTGGGTTGAGAGGGCGTT	miRNA	miR-320d
t0029996	23	6	AAAAGCCGGGTTGAGAGGGCGTA	miRNA	miR-320c
t0030254	21	6	AAAAGCTGGGTTGGGAGGGCG	miRNA	miR-320a
t0030331	25	6	AAAAGCTGGGTTGAGAGGGCGATAG	miRNA	miR-320a
t0030558	23	6	AAAAGCTGAGTTGAGAGGGCGAA	miRNA	miR-320a
t0029836	23	6	AAAAGCTGGGATGAGAGGGCGAT	miRNA	miR-320a
t0030123	21	6	AAAAGCTGGGTTAAGAGGGCG	miRNA	miR-320a
t0030180	23	6	AAAAGCTGGGTTAAGAGGGCGAT	miRNA	miR-320a
t0030214	23	6	AATCTGAGAAGGCGCACAAGGGT	miRNA	miR-3200-5p
t0030306	25	6	GGCTGGTCCGAGTGCAGTGGTGTTT	miRNA	miR-3135b
t0030356	20	6	CGCGGGTCGGGGGGCGGGGC	miRNA	miR-2981
t0030451	22	6	CTTCAAGTAATCCAGGATAGGC	miRNA	miR-26a
t0030522	22	6	TTCAAGTAATCCAGGGTAGGCT	miRNA	miR-26a
t0030640	22	6	CAATGCACTTGTCTCGGTCTGA	miRNA	miR-25
t0029904	22	6	GATTGCACTTGTCTCGGTCTGA	miRNA	miR-25
t0030041	21	6	CATTGCACTTGTCTCGGTCCG	miRNA	miR-25
t0030119	19	6	ATCACATTGCCAGGGATTC	miRNA	miR-23a
t0030230	22	6	AAGCTGCCAGTTGAAGAACTTA	miRNA	miR-22-3p
t0030295	21	6	GACTGGGGCGGAACATCTGTT	miRNA	miR-219-2-3p
t0030398	23	6	CAAAGTGCTCATAGTGCCGGTAG	miRNA	miR-20b
t0030494	25	6	TGGAATGTAAGGAAGTGTGTGAATC	miRNA	miR-206
t0030521	24	6	TAAAGTGCTTATAGTGCAGGTAAA	miRNA	miR-20
t0030089	25	6	TGGAATCTAAAGAAGTATGTATATC	miRNA	miR-1a
t0030582	20	6	TGTGCAAATCCATGCAAAAC	miRNA	miR-19b
t0030097	23	6	TCCTGCCCTCCTTGCTGTAGTTT	miRNA	miR-1976
t0030253	22	6	CTGACCTATGAATTGACCGCCA	miRNA	miR-192

t0029992	21	6	CTGACCTATGAATTGAAAGCC	miRNA	miR-192
t0030241	23	6	CAAAGGAATCCCAAAAGCAGCTG	miRNA	miR-191
t0030425	23	6	CAACGGAACCCCAAAAGCAGCTG	miRNA	miR-191
t0030495	22	6	CAATGGAATCCCAAAAGCAGCT	miRNA	miR-191
t0029922	22	6	CAACGGAATCCCAAAAGCAGCC	miRNA	miR-191
t0030409	23	6	CAACGGGATCCCAAAAGCAGCTG	miRNA	miR-191
t0030545	27	6	CAACGGAATCCCAAAAGCAGCTGAATC	miRNA	miR-191
t0030437	22	6	TGGAGAGAAAGGCAGTTCCTTC	miRNA	miR-185
t0029887	23	6	TGGAGAGAAAGCCAGTTCCTGAA	miRNA	miR-185
t0030524	21	6	TGGAGAGAAAGTCAGTTCCTG	miRNA	miR-185
t0029894	23	6	TGGAGAGAAAGGCAATTCCTGAT	miRNA	miR-185
t0029978	23	6	TGGTGAGAAAGGCAGTTCCTGAA	miRNA	miR-185
t0030377	23	6	TGGAGAGAATGGCAGTTCCTGAA	miRNA	miR-185
t0030661	23	6	TGGAAAGAAAGGCAGTTCCTGAA	miRNA	miR-185
t0029833	22	6	TGGAGAGAAAGGAAGTTCCTGC	miRNA	miR-185
t0029934	23	6	TGGAGAGAAAGGCAGCTCCTGAT	miRNA	miR-185
t0029946	21	6	TGGAGAGAAAGGAAGTTACTG	miRNA	miR-185
t0030125	21	6	TGGTGAGAAAGGCAGTTCCTG	miRNA	miR-185
t0030324	23	6	TGGAGGGAAAGGCAGTTCCTGAG	miRNA	miR-185
t0030382	21	6	TGGAGAGAATGGCAGTTCCTG	miRNA	miR-185
t0029902	24	6	TGGAGAGAAAGGAAGTTCCTGAAA	miRNA	miR-185
t0029998	21	6	TGGAGAGAAAGGCAGTTCCAG	miRNA	miR-185
t0030068	22	6	AAGGTAGATAGAACAGGTATTG	miRNA	miR-1839
t0030076	21	6	TGAGGGAGTAGGTTGTATGGT	miRNA	miR-1827
t0030175	22	6	TGAGGGATTAGATTGTATAGTT	miRNA	miR-1827
t0030191	21	6	TGAGGGAGTAGATTGAATAGT	miRNA	miR-1827
t0030283	23	6	TGAGGGAGTAGGTTGTATAGTTA	miRNA	miR-1827
t0030350	22	6	AACATTCATTGCTGTCGGTGGG	miRNA	miR-181b
t0030464	18	6	CGGGTCGGGGCGGCGGCG	miRNA	miR-1777a
t0030485	28	6	TAGCAGCACGTAAATATTGGCAGAAAAA	miRNA	miR-16b
t0030526	28	6	GGGGCGGGGAGCGGTCGGGCGGCGGCG	miRNA	miR-1607
t0030535	23	6	TAGCAGCACGTAAATATTAGCGA	miRNA	miR-16
t0030593	23	6	TAGCAGCACGTAAATATCGGCGT	miRNA	miR-16
t0030608	20	6	TAGCAACACGTAAATATTGG	miRNA	miR-16
t0030647	23	6	TAGCAGCACATAAATATTGGCGA	miRNA	miR-16
t0030427	20	6	TGGCAGCACGTAAATATTGG	miRNA	miR-16
t0029912	21	6	TAGCAGCACGTAAATGTTGGC	miRNA	miR-16
t0029952	19	6	TAGCAGCACGTAAATATAA	miRNA	miR-16
t0029956	23	6	TAGCAGCACATAAATATTGGCGT	miRNA	miR-16
t0030560	23	6	TAGCCGCACGTAAATATTGGCGA	miRNA	miR-16
t0030038	22	6	TAGCAGAACGTAAATATTGTCG	miRNA	miR-16
t0029935	20	6	TAGCAGCTCGTAAATATTGG	miRNA	miR-16
t0029997	23	6	TAGCGGCACGTAAATATTGGCGA	miRNA	miR-16
t0030540	20	6	TAGCAGCACGTAAATCTTGG	miRNA	miR-16
t0030016	20	6	TAGCCGCACGTAAATATTGG	miRNA	miR-16
t0030057	18	6	CAGCAGCACATCATGGTT	miRNA	miR-15b
t0030060	19	6	TAGCAGAACATCATGGTTA	miRNA	miR-15b
t0030064	18	6	TAGCAGCACATCATGGTA	miRNA	miR-15b
t0030103	22	6	TAGCAGCACATCATGGTCTACA	miRNA	miR-15b
t0030162	22	6	GAGCAGCACATCATGGTTTACA	miRNA	miR-15b
t0030218	19	6	AAGCAGCACATCATGGTTT	miRNA	miR-15b
t0030227	20	6	TAGCAGCACATAATTGTTTG	miRNA	miR-15a
t0030291	20	6	AAGCAGCACATAATGGTTTG	miRNA	miR-15a
t0030333	21	6	TAGCAGCACATACTGGTTTGT	miRNA	miR-15a
t0030407	21	6	TAGCAGCTCATAATGGTTTGT	miRNA	miR-15a
t0030448	30	6	AATAGCAGCACATAATGGTTTGAAAAAA/	miRNA	miR-15a
t0030516	21	6	AAGCAGCACATAATGGTTTGT	miRNA	miR-15a
t0030662	22	6	TAGCAGAACATAATGGTTTGTG	miRNA	miR-15a

t0029910	18	6	CCAACCCTTGTACCAGTG	miRNA	miR-150
t0029975	23	6	TGAGAACTGAATTCCATAGGCTA	miRNA	miR-146b-5p
t0030092	25	6	GGATATCATCATATACTGTAAGTTA	miRNA	miR-144*
t0030093	23	6	GGATATCATCATATACTGTAAAA	miRNA	miR-144*
t0030205	20	6	CTACAGTATAGATGATTTAC	miRNA	miR-144
t0030581	20	6	TACAGTATAGATGATGTAAT	miRNA	miR-144
t0030027	21	6	CCCATAAAGTAGAAAGAACTA	miRNA	miR-142
t0030192	21	6	CCCATAAAGAAGAAAGCACTA	miRNA	miR-142
t0030231	19	6	CCCATAAAGTAGAAAGCAT	miRNA	miR-142
t0030252	23	6	TACCACAGGGTATAACCACGGAC	miRNA	miR-140
t0030391	32	6	TACCACAGGGTAGAACCACGGATGTGGG	miRNA	miR-140
t0030486	23	6	TACCTCAGGGTAGAACCACGGAA	miRNA	miR-140
t0030566	23	6	GACCACAGGGTAGAACCACGGAC	miRNA	miR-140
t0029884	22	6	ACCACAGGGTAGAACCACGGGA	miRNA	miR-140
t0030618	23	6	TACCACAGGGTAGAACCAGGGAC	miRNA	miR-140
t0030272	22	6	ACCGCAGGGTAGAACCACGGAT	miRNA	miR-140
t0030611	23	6	ACCACAGGGTAGAACCACACACT	miRNA	miR-140
t0030050	22	6	ACCACAGGGCAGAACCACGGAT	miRNA	miR-140
t0030378	20	6	CCACAGGGGAGAACCACGGA	miRNA	miR-140
t0029893	22	6	TCCCACAGGGTAGAACCACGGA	miRNA	miR-140
t0029930	24	6	GACCACAGGGTAGAACCACGGACA	miRNA	miR-140
t0030367	23	6	TACCACAGGGTAGAACCACGAAA	miRNA	miR-140
t0029837	24	6	TACCACAGGGTAGAACCACAGACG	miRNA	miR-140
t0029840	23	6	TACTACAGGGTAGAACCACGGAT	miRNA	miR-140
t0029846	24	6	TACCACAGGGTAGAACCCCGGATA	miRNA	miR-140
t0029848	22	6	ACCACAGGGTAAAACCACGGAC	miRNA	miR-140
t0029932	22	6	ACCACAGGGTAGAACCACGAAC	miRNA	miR-140
t0029948	22	6	TACCACAGGGTACAACCACGGA	miRNA	miR-140
t0029981	21	6	ACCACAGGGTAGAACTACGGC	miRNA	miR-140
t0030015	23	6	ACCACAGGGTAGAACCACGGGCA	miRNA	miR-140
t0030034	23	6	TATCACAGGGTAGAACCACGGAT	miRNA	miR-140
t0030079	22	6	AACACAGGGTAGAACCACGGAA	miRNA	miR-140
t0030104	23	6	TACCACAGGGTAGACCCACGGAC	miRNA	miR-140
t0030124	23	6	ACAACAGGGTAGAACCACGGACA	miRNA	miR-140
t0030134	23	6	TACCACAGGGTAGAACCAAGGAG	miRNA	miR-140
t0030141	23	6	ACCACAGGGTAGAACCACAGACA	miRNA	miR-140
t0030161	23	6	ACCACAGAGTAGAACCACGGACA	miRNA	miR-140
t0030187	22	6	TACCACATGGTAGAACCCCGGA	miRNA	miR-140
t0030200	22	6	TACCACAGGGTAGAATCCCGGA	miRNA	miR-140
t0030209	23	6	ACCACAGGGTAGAACCCCGGACT	miRNA	miR-140
t0030212	23	6	TACCACAGAGTAGAACCACGGAT	miRNA	miR-140
t0030216	23	6	TACCACAGGGTAGACCCACGGAA	miRNA	miR-140
t0030233	23	6	ACCACAGGGTAGAACAACGGACT	miRNA	miR-140
t0030238	22	6	ACCACAGGGTAGAAACACGGAG	miRNA	miR-140
t0030244	23	6	TACCGCAGGGTAGAACCACGGAT	miRNA	miR-140
t0030249	22	6	ACCACAGGGTAAAACCACGGAA	miRNA	miR-140
t0030316	22	6	TACCACAGGGTAGAACCCAGGA	miRNA	miR-140
t0030322	22	6	GCCACAGGGTAGAACCACGGAC	miRNA	miR-140
t0030325	24	6	TACAACAGGGTAGAACCACGGACG	miRNA	miR-140
t0030334	22	6	ACCACAGAGTAGAACCACGGAA	miRNA	miR-140
t0030410	20	6	ACCACAGGGTAGAACCCCGG	miRNA	miR-140
t0030414	22	6	ACCAAAGGGTAGAACCACGGAC	miRNA	miR-140
t0030432	23	6	TACGACAGGGTAGAACCACGGAA	miRNA	miR-140
t0030449	22	6	CAGTGCAATGTTAAAAGGGAAT	miRNA	miR-130a
t0030458	24	6	TGGAGTGTGACAATGGGGTTTATC	miRNA	miR-122
t0030493	22	6	AGCAGCATTGTACAGGGATATT	miRNA	miR-107
t0030500	22	6	TACCGCACTGTGGGGACTTGCT	miRNA	miR-106b*
t0030536	19	6	AAAGTGCTGACAGTGCAGA	miRNA	miR-106b

t0030541	21	6 TAAAGTGCTGACAGCGCAGAT	miRNA	miR-106b
t0030544	20	6 TAAAGTGCTGACGGTGCAGA	miRNA	miR-106b
t0030570	21	6 CAAAGTGCTGACAGTGCAGAT	miRNA	miR-106b
t0030574	22	6 AAAAGTGCTTACAGTGCAGGAA	miRNA	miR-106
t0030577	20	6 AGCAGCATTGTACAGGGGTA	miRNA	miR-103a
t0030578	22	6 AGCAGCATTGTACAGGGGTATG	miRNA	miR-103a
t0030643	26	6 AGCAGCATCGTACAGGGCTATGAATC	miRNA	miR-103a
t0030648	21	6 CAGCAGCATTGTACAGGGCTA	miRNA	miR-103a
t0030014	21	6 AGCAGCATTGTACAGGGCCAT	miRNA	miR-103a
t0030257	23	6 AGCAGCATTGTACAGGGGTATGA	miRNA	miR-103a
t0030511	20	6 AGCAGCATTGTACAGGACTA	miRNA	miR-103a
t0030417	23	6 AGCAGGATTGTACAGGGCTATGA	miRNA	miR-103a
t0030189	22	6 AGCAGCATTGTACAGGGCCATG	miRNA	miR-103a
t0030300	22	6 GAACAGTACTGTGATAACTGAA	miRNA	miR-101c
t0030340	20	6 ACAGTACTGTGATAACTGAT	miRNA	miR-101c
t0030397	22	6 GTACAGTAATGTGATAACTGAA	miRNA	miR-101
t0030474	22	6 GTACAGTACTGTGATAATTGAA	miRNA	miR-101
t0029921	22	6 GTACAGTACTGTGATATCTGAA	miRNA	miR-101
t0030118	20	6 TACAATACTGTGATAACTGA	miRNA	miR-101
t0030359	21	6 GTACAGTAATGTGATAACTGA	miRNA	miR-101
t0030370	21	6 TACAGTACTGTGATAGCTGAA	miRNA	miR-101
t0030389	22	6 TACAGTACTGTGATAACTGCAG	miRNA	miR-101
t0030434	26	6 GTACAGTACTGTGATAACTGAACTGT	miRNA	miR-101
t0030460	22	6 GTACAGTACTGTGATAACCGAA	miRNA	miR-101
t0030519	24	6 TACAGTACTGTGATAACTGAAGAT	miRNA	miR-101
t0030561	20	6 TACAGCACTGTGATAACTGA	miRNA	miR-101
t0030620	21	6 TAGAGTACTGTGATAACTGAA	miRNA	miR-101
t0030659	23	6 TATAGTACTGTGATAACTGAAGA	miRNA	miR-101
t0029841	20	6 TACAGTACTGTGATCACTGA	miRNA	miR-101
t0029849	22	6 CTACAGTACTGTGATAACTGAA	miRNA	miR-101
t0029940	25	6 TGGAATGTAAAGAAGTGTGTCCATC	miRNA	miR-1
t0029943	23	6 TGAGGTAGTAGATTGAATAGTTG	miRNA	let-7k
t0029953	20	6 TGAGGTAGTAGATTGTGCTG	miRNA	let-7i
t0029964	19	6 TGAGGCAGTAGTTTGTGCT	miRNA	let-7i
t0029970	21	6 TGAGGTAGGAGTTTGTGCTGT	miRNA	let-7i
t0030044	21	6 TGAGGCAGTAGTTTGTGCTGT	miRNA	let-7i
t0030167	20	6 TGAGGTAGTAGGTTGTGCTG	miRNA	let-7i
t0030201	21	6 TGAGGTAGTAGTTTGTAGAGT	miRNA	let-7g
t0030265	21	6 TGAGGTAGTAGTTTGTACTGT	miRNA	let-7g
t0030329	21	6 TGAGGTAGTAGTTTGTACAGA	miRNA	let-7g
t0030436	22	6 TGAGGTAGTAGATTGTATAGCG	miRNA	let-7f-5p
t0030463	22	6 TGAGGTAGTAGATTGTATTGTC	miRNA	let-7f-5p
t0030478	22	6 TGAGGTAGTAGTATGTATAGTT	miRNA	let-7f
t0030045	22	6 TGAGGTAGTAGATTGGATAGTA	miRNA	let-7f
t0030564	23	6 TGAGGAAGTAGATTGTATAGTTT	miRNA	let-7f
t0029861	25	6 ATGAGGTAGTAGATTGTATAGTAAA	miRNA	let-7f
t0030018	19	6 AGAGGTAGTAGATTGTATA	miRNA	let-7f
t0030019	20	6 CTGAGGTAGTAGATTGTATA	miRNA	let-7f
t0030226	23	6 TGAGGTAGTAGATTGTATAGCTA	miRNA	let-7f
t0030280	22	6 TGAGGTAGTAGACTGTATAGTA	miRNA	let-7f
t0029888	23	6 TGAGGTAGTAGATTGTATCGTTA	miRNA	let-7f
t0030586	22	6 CGAGGTAGTAGGTTGCATAGTT	miRNA	let-7d
t0030650	22	6 AGAGGTAGTTGGTTGCATAGTT	miRNA	let-7d
t0030023	20	6 AGAGGTAGTGGGTTGCATAG	miRNA	let-7d
t0030271	20	6 AGAGGTAGTAGATTGCATAG	miRNA	let-7d
t0029927	22	6 AGTGGTAGTAGGTTGCATAGTT	miRNA	let-7d
t0030194	20	6 AGAGGTAGTAGGTTGCGTAG	miRNA	let-7d
t0030213	21	6 GGAGGTAGTAGGTTGCATAGT	miRNA	let-7d

t0030277	21	6 AGAGGTCGTAGGTTGCATAGT	miRNA	let-7d
t0030431	21	6 AGAGGTAGTGGGTTGCATAGT	miRNA	let-7d
t0030515	25	6 TGAGGAAGTAGGTTGTATGGTTATC	miRNA	let-7c
t0030576	22	6 TGAGGTAGTAGCTTGTATGGTT	miRNA	let-7c
t0030631	19	6 TGAGGTAGTAGGGTGTGTG	miRNA	let-7b
t0030634	23	6 TGAGGTGGTAGGTTGTGTGGTTT	miRNA	let-7b
t0029857	23	6 TGAGGTGGTAGGTTGTGTGGTTA	miRNA	let-7b
t0029988	25	6 TGAGGTAGTAGGTTGTGTGGTCAAA	miRNA	let-7b
t0030037	24	6 TGAGGAAGTAGGTTGTGTGGTTAA	miRNA	let-7b
t0030135	23	6 TGAGGCAGTAGGTTGTGTGGTTT	miRNA	let-7b
t0030170	20	6 TGAGGTAGAAGGTTGTGTGG	miRNA	let-7b
t0030261	22	6 TGAGGTAGTAGGATGTGTGGTA	miRNA	let-7b
t0030362	23	6 TGAGGTAGTAGGTTGCGTGGTTG	miRNA	let-7b
t0030380	23	6 TGAGGTAGTAGGCTGTGTGGTTT	miRNA	let-7b
t0030393	19	6 TGAGGTGGTAGGTTGTGTG	miRNA	let-7b
t0029901	23	6 TGAGGTAGTAGGATGTGTGGTTA	miRNA	let-7b
t0029950	23	6 CGAGGTAGTAGGTTGTGTGGTTA	miRNA	let-7b
t0029832	23	6 TGAGGTAGTAGGGTGTGTGGTTT	miRNA	let-7b
t0029835	20	6 TGAGGTAGTCGGTTGTGTGG	miRNA	let-7b
t0029858	23	6 TGAGGTAGTAGGTTGTGTGGTTC	miRNA	let-7b
t0029896	23	6 TGAGGTAGTAGATTGTGTGGTTA	miRNA	let-7b
t0029911	22	6 TGAGGTAGTAGGTTGTGTAGTA	miRNA	let-7-5p
t0029918	20	6 TGTGGTAGTAGGTTGTATAG	miRNA	let-7
t0030054	19	6 TGAGGTAGTAGGCTGTATA	miRNA	let-7
t0030063	26	6 TGAGGTATGAGGTTGTATAGTTGATC	miRNA	let-7
t0030279	21	6 TGAGGTAGGAGGGTGTATAGT	miRNA	let-7
t0030348	23	5 CAAAGTGCTGTTCGGGCAGGTAG	miRNA	miR-93
t0030352	20	5 AGGGACGGGACGCGGTGAAG	miRNA	miR-92b*
t0030373	22	5 CATTGCACTTGTCTCGGCCTGT	miRNA	miR-92a-3p
t0030406	22	5 TATTGCAATTGTCCCGGCCTGT	miRNA	miR-92a
t0030420	21	5 TATTGCAATTGTCCCGGCCTG	miRNA	miR-92a
t0030424	22	5 TATTGCACTTGTCCCGGACTGT	miRNA	miR-92a
t0030465	21	5 TATTGCACTTGTACCGGCCTG	miRNA	miR-92a
t0030482	22	5 TATTGCCCTTGTCCCGGCCTGT	miRNA	miR-92a
t0030204	21	5 TGTTGCACTTGTCCCGGCCTG	miRNA	miR-92a
t0030168	22	5 TGGAGAGAAAGGCAGTTTATGA	miRNA	miR-765
t0030276	21	5 TGAAAGACTTGAGTAGTGAGA	miRNA	miR-71b-5p
t0030626	19	5 TGGGTTTACGTTGGGAGCA	miRNA	miR-629
t0030630	19	5 AAAAACTGAGACTACTTTT	miRNA	miR-548e
t0031442	22	5 CCTCCCACACCCAAGGCTTGAA	miRNA	miR-532-3p
t0030686	22	5 TCTGGGCACAGGCGGAGGGACA	miRNA	miR-5107
t0031229	22	5 AAACAAACATGGTGCACTTCTT	miRNA	miR-495
t0030708	22	5 TCCAGTACTGAGCTGCCCCGAG	miRNA	miR-486-5p
t0030829	23	5 TCCTGTACTGAGCTGCCCCGCGG	miRNA	miR-486-5p
t0030850	23	5 TCCTGTACTGAGCTGGCCCGAGA	miRNA	miR-486-5p
t0031276	23	5 TCCTGTACTGAGCTGCCCCCAGA	miRNA	miR-486-5p
t0031302	20	5 TCCTGTACTGCGCTGCCCCG	miRNA	miR-486-5p
t0031491	21	5 TCCGGTACTGAGCTGCCCCGA	miRNA	miR-486-5p
t0030918	21	5 TCCTGTACTCAGCTGCCCCGA	miRNA	miR-486-5p
t0031078	20	5 TCCTGGACTGAGCTGCCCCG	miRNA	miR-486-5p
t0031665	22	5 TCCGGTACTGAGCTGCCCCGAG	miRNA	miR-486-5p
t0031518	22	5 TTCTGTACTGAGCTGCCCCGCG	miRNA	miR-486-5p
t0030827	21	5 TCCTGTACTGAGATGCCACGA	miRNA	miR-486-5p
t0030911	22	5 TCCTGTACTGAGATGCCCCGAA	miRNA	miR-486-5p
t0030697	23	5 TCCTGTACTGAGCTGACCCGAGT	miRNA	miR-486-5p
t0030685	28	5 TCCTGTACTGAGCTGCCCCGCGACGGCA	miRNA	miR-486-5p
t0030712	23	5 TCCTGTACTGAGTTGCCCCGAGA	miRNA	miR-486-5p
t0030736	26	5 GGATTGTGTCGGCAGATCAGTATTTT	miRNA	miR-4860

t0030739	23	5 TGTAGAGCAGGGAGCAGGGAGCT	miRNA	miR-4732-5p
t0030762	23	5 TATAGAGCAGGGAGCAGGAAGCT	miRNA	miR-4732-5p
t0030777	23	5 TGTAGAGCAGGGAGCAGGAAGGT	miRNA	miR-4732-5p
t0030787	18	5 AGGCTGGGTCGGTCGGGC	miRNA	miR-4651
t0031014	29	5 TGCGTCGGTCGGGCTGGGGCGCGAAGCG	miRNA	miR-4651
t0031035	21	5 AGAGAAGGGTCGGGGCGGCAG	miRNA	miR-4516
t0031197	20	5 TGAGGGAGTAGGGTGTGTGG	miRNA	miR-4510
t0031213	23	5 TGAGGGAGTAGGTTGTGTGGTTG	miRNA	miR-4510
t0031226	23	5 TGAGGGAGTAGGTTGTGTGGTAT	miRNA	miR-4510
t0031257	19	5 AAACCGCTACCATTACTGA	miRNA	miR-451
t0031482	20	5 AAACCGTTACCATTCCTGAG	miRNA	miR-451
t0031590	20	5 AAACCGTTACCAATACTGAG	miRNA	miR-451
t0031654	22	5 AAACCGTGACCATTACTGAGTT	miRNA	miR-451
t0030941	25	5 AAACCGTTACCATTACTGAGTTTCG	miRNA	miR-451
t0031529	22	5 AAACAGTTAACATTACTGAGTT	miRNA	miR-451
t0031593	25	5 AAACCGTTACCATTACTGAGATTAG	miRNA	miR-451
t0031123	20	5 AAACCGTTACCATTACAGAG	miRNA	miR-451
t0031476	22	5 AAACCGTTCCCATTACTGAGTT	miRNA	miR-451
t0031201	21	5 AAACATTTACCATTACTGAGT	miRNA	miR-451
t0030969	20	5 AACCCGTTACCATTACTGAG	miRNA	miR-451
t0031032	25	5 AAACCGTTACTATTACTGAGTTTAG	miRNA	miR-451
t0031394	22	5 AAACCGTTACCATTACAGAGTT	miRNA	miR-451
t0030700	24	5 AAACCGTTAGCATTACTGAGTTTA	miRNA	miR-451
t0030715	24	5 AAACAGTTACCATTACTGAGTTTA	miRNA	miR-451
t0030814	22	5 AAACCTTTACCATTATTGAGTT	miRNA	miR-451
t0030821	24	5 AAAACGTTACCATTACTGAGTTTA	miRNA	miR-451
t0030843	26	5 AAACCGTTACCATTACTGGGTTTAGT	miRNA	miR-451
t0030908	24	5 AAACCGTTACCATTACTTAGTTTA	miRNA	miR-451
t0030915	23	5 AAAGCGTTACCATTACTGAGTTT	miRNA	miR-451
t0030933	23	5 CAACCGTTACCATTACTGAGTTT	miRNA	miR-451
t0030944	22	5 TGTGACAATAGAGATGAACATG	miRNA	miR-4504
t0030949	21	5 CGCAGCATTGTACAGGGCTAT	miRNA	miR-4289
t0031026	19	5 AGAAGCATTGTACAGGGCT	miRNA	miR-4289
t0031112	21	5 TGCAGCATTGTACAGGGCTAT	miRNA	miR-4289
t0031171	21	5 CAGCAGCAGTTCATGTTTTGA	miRNA	miR-424
t0031173	21	5 CAGCAGCACTTCATGTTTTGA	miRNA	miR-424
t0031184	19	5 TGAGAGGCAGAGAGCGAGA	miRNA	miR-423-5p
t0031207	24	5 TGAGGGGCAGAGAGCGAGAGTTTT	miRNA	miR-423-5p
t0031209	24	5 TGAGGGGCAGGGAGCGAGACTTTT	miRNA	miR-423-5p
t0031306	23	5 TGAGGGGCAGAGAGCGAGCATTT	miRNA	miR-423-5p
t0031431	20	5 TGAGGGGCAGAGCGCGAGAC	miRNA	miR-423-5p
t0031539	23	5 TGAGGGGCAGAGAACGAGACTTA	miRNA	miR-423-5p
t0031640	25	5 TGAGGGGCAGAGAGCGAGACTTTCA	miRNA	miR-423-5p
t0031219	24	5 TGAAGGGCAGAGAGCGAGACTTTT	miRNA	miR-423-5p
t0031093	24	5 TGAGGGGCAGAGAGTGAGACTTTT	miRNA	miR-423-5p
t0031441	20	5 TGAGGGGCAGAGAGCGGGAC	miRNA	miR-423-5p
t0031533	19	5 TGAGGTGCAGAGAGCGAGA	miRNA	miR-423-5p
t0031011	19	5 TGAGGGGCCGAGAGCGAGA	miRNA	miR-423-5p
t0031258	23	5 TGAGGGGCGGAGAGCGAGACTTA	miRNA	miR-423-5p
t0030851	24	5 TGAGGGGCAGAGAGCCAGACTTTA	miRNA	miR-423-5p
t0030925	24	5 TGAGGGGCAGAGAGCAAGACTTTT	miRNA	miR-423-5p
t0030965	23	5 TGAGGGGCAGAGCGCGAGCCTTT	miRNA	miR-423-5p
t0030999	22	5 TGAGGGGCAGTGAGCGAGACTT	miRNA	miR-423-5p
t0031050	24	5 TGAGGGGCAGAGAGGGAGACTTTT	miRNA	miR-423-5p
t0031063	22	5 TCGGAGGGCGGCGGCGGCGGCG	miRNA	miR-3885-5p
t0031149	23	5 TCGGGGCGGCGGCGGCGGCGGCG	miRNA	miR-3885-5p
t0031162	25	5 ACTGGACTTGGAGGCAGAAGGCATC	miRNA	miR-378f
t0031189	21	5 GCTGGACTTGGAGTCAGAAGG	miRNA	miR-378

t0031293	25	5 ACTGGACTTGGAGACAGAAGGCATC	miRNA	miR-378
t0031298	25	5 ACTGGACTTGGAGTCAGCAGGCATC	miRNA	miR-378
t0031355	23	5 ACTGGACTTGGAGTCAGCAGGCA	miRNA	miR-378
t0031378	23	5 ACCGGACTTGGAGTCAGAAGGCA	miRNA	miR-378
t0031494	25	5 TTTGTTCGTTCGGCTCGCGGGAATC	miRNA	miR-375
t0031497	27	5 ATATAATACAACCTGCTAAGTGAAAAA	miRNA	miR-374
t0031514	26	5 AACTTTGAAGACTGAAGGGGAGAAGG	miRNA	miR-3526
t0031632	21	5 AACTTTGAAGACTGAAGTGGC	miRNA	miR-3526
t0031643	24	5 TTGAAGACTGAAGTGGAGAAGGGG	miRNA	miR-3526
t0031314	26	5 AACTTTGAAGACTGAAGTGGCGAAGG	miRNA	miR-3526
t0031403	24	5 TGAAGACTGAAGTGGAGAAGGGCT	miRNA	miR-3526
t0030894	24	5 TGAAGACTGAAGTGGAGAAGGGAT	miRNA	miR-3526
t0030877	21	5 TCAGAATCGTTCGCAATGGCT	miRNA	miR-3495
t0031433	21	5 CCCTCGAACTGTTGTGGCCAT	miRNA	miR-3487
t0031489	22	5 TCTCATACAGAAATCGCACCCG	miRNA	miR-342-3p
t0031520	21	5 AAAAGCTGGGTTGAGAGGGTG	miRNA	miR-320d
t0031543	20	5 AAAGCTGGGTTGAGAGGGGG	miRNA	miR-320d
t0031406	24	5 AAAAGCTGGGTTGAGAGGGAGAAT	miRNA	miR-320d
t0031127	23	5 AAAAGCTGGGTTGAAAGGGCGTA	miRNA	miR-320d
t0030675	22	5 AAAAGATGGGTTGAGAGGGAGA	miRNA	miR-320d
t0030719	23	5 AAAAACTGGGTTGAGAGGGCGTA	miRNA	miR-320d
t0031003	23	5 AAAAGCTGGGTTGAGAGAGCGTA	miRNA	miR-320d
t0031091	23	5 AAAAGCTGAGTTGAGAGGGCGTT	miRNA	miR-320d
t0031114	21	5 AAAGCTGGGTTGAGAGGGAGA	miRNA	miR-320d
t0031432	22	5 AAAAGCTGGGTTCAGAGGGCGT	miRNA	miR-320d
t0031337	21	5 AAAGCTGGGGTGAGAGGGCGT	miRNA	miR-320d
t0031445	23	5 AAAAGCTGAGTTGAGAGGGCGTA	miRNA	miR-320d
t0030907	22	5 AAAAGCTGGGTTGTGAGGGCGT	miRNA	miR-320d
t0030668	23	5 AAAAGCTAGGTTGAGAGGGCGTT	miRNA	miR-320d
t0030871	22	5 AAAAGCTGGGGTGAGAGGGCAA	miRNA	miR-320b
t0030970	22	5 AAAAGCTGGGTTGGGAGGGCAA	miRNA	miR-320b
t0031068	22	5 AAAAGCTGGGTGGAGAGGGCGA	miRNA	miR-320a
t0031115	21	5 AAAGGCTGGGTTGAGAGGGCG	miRNA	miR-320a
t0031120	23	5 AAAAGCTGGGTTGAAAGGGCGAT	miRNA	miR-320a
t0031180	22	5 ATAAGCTGGGTTGAGAGGGCGA	miRNA	miR-320a
t0031283	18	5 AAAAGCTGGGTTGAGAGG	miRNA	miR-320a
t0031349	23	5 AAAAGCTAGGTTGAGAGGGCGAT	miRNA	miR-320a
t0031454	23	5 AAAGGCTGGGTTGAGAGGGCGAT	miRNA	miR-320a
t0031462	23	5 AAAAGCTGGGTTGAGAGCGCGAT	miRNA	miR-320a
t0031486	23	5 AAAAACTGGGTTGAGAGGGCGAT	miRNA	miR-320a
t0031569	23	5 AAAAGCTGGGTTGAGAGCGCGAA	miRNA	miR-320a
t0031584	22	5 TAAAGCTGGGTTGAGAGGGCGA	miRNA	miR-320a
t0030703	23	5 AAAAGCCGGGTTGAGAGGGCGAT	miRNA	miR-320a
t0031044	22	5 AAAAGCTGGCTTGAGAGGGCGA	miRNA	miR-320a
t0030692	23	5 AAAAGCTGGGGTGAGAGGGCGAG	miRNA	miR-320a
t0030745	21	5 AAAAGCTGGGTTGAAAGGGCG	miRNA	miR-320a
t0030790	23	5 AAAAGCTGGATTGAGAGGGCGAT	miRNA	miR-320a
t0030865	23	5 AAAAGCTGGGTTGCGAGGGCGAT	miRNA	miR-320a
t0030906	21	5 TATTGCACATTACTAAGTTGC	miRNA	miR-32
t0030928	18	5 TGGGACAAGAGGACGGTT	miRNA	miR-3122
t0030997	26	5 TGTAAACATCCCCGACTGGAAGCCTC	miRNA	miR-30d
t0031016	19	5 TGTAAACATCCTACACTCT	miRNA	miR-30c
t0031031	20	5 GCGCGCGGGTCGGGGGGCGG	miRNA	miR-2981
t0031191	23	5 CGGCGGCGGGCGGCGGAGGGGCC	miRNA	miR-2885
t0031221	22	5 TTCAAGCAATTCAGGATAGGTT	miRNA	miR-26b
t0031353	22	5 TTCAAGGAATCCAGGATAGGCT	miRNA	miR-26a
t0031418	22	5 CTCAAGTAATCCAGGATAGGCT	miRNA	miR-26a
t0031512	22	5 TTCAAGTAATCCGGGATAGGCT	miRNA	miR-26a

t0031577	19	5 TTCAAGTAATCCAGGATAG	miRNA	miR-26a
t0031610	22	5 TTCAAGTAATCCAGGATAGGGT	miRNA	miR-26a
t0031644	21	5 TGAGGGAGGAGGTTGTGTGGT	miRNA	miR-265
t0031634	23	5 CATTGCACTTGTCTCGGGCTGAA	miRNA	miR-25
t0031317	22	5 CCTTGCACTTGTCTCGGTCTGA	miRNA	miR-25
t0031088	22	5 CATTGCACTTGTCTCGGTCTTT	miRNA	miR-25
t0030818	21	5 CATTGCACTTGTCTCGGACTG	miRNA	miR-25
t0030725	22	5 CATTGCACTTGTGTCGGTCTGA	miRNA	miR-25
t0031065	21	5 CATTGCACTTGTCTCGATCTG	miRNA	miR-25
t0031554	26	5 CATTGCACTTGTCTCGGTCTGAGTCG	miRNA	miR-25
t0030974	23	5 CATTGCACTTGTCTCGGCCTGAA	miRNA	miR-25
t0031275	22	5 TGGCTCAGTTCAGCAGGAAAAG	miRNA	miR-24b
t0031346	21	5 TGGCTCAGTTCAGCAGGCACA	miRNA	miR-24
t0031390	21	5 AGCTACATCTGGCTACTGGGG	miRNA	miR-222
t0031517	21	5 AGCTACATTGTCTGCTGGGGT	miRNA	miR-221
t0030887	26	5 GACTGGGGCGGTACATCTGTTAAACA	miRNA	miR-219-2-3p
t0030891	23	5 TTGGGGAAACGGCCGCTGAGGGA	miRNA	miR-2110
t0031021	22	5 TAGCTTATCAGACTGATGTTGC	miRNA	miR-21
t0031033	25	5 TGTAATGTAAGGAAGTGTGTGGATC	miRNA	miR-206
t0031069	21	5 CTGACTTATGAATTGACAGCC	miRNA	miR-192
t0031137	22	5 TGACCTATGAATTGACAGCCAA	miRNA	miR-192
t0031167	21	5 CTGACCTATGAATTGACAGAC	miRNA	miR-192
t0031364	20	5 CAACGGAATCCCAAAAGCAC	miRNA	miR-191
t0031652	22	5 CGACGGAATCCCAAAAGCAGCT	miRNA	miR-191
t0031022	22	5 AACAGAATCCCAAAAGCAGCTG	miRNA	miR-191
t0031307	21	5 AACGGAATCCCAAAAGAAGCT	miRNA	miR-191
t0031034	22	5 CACCGGAATCCCAAAAGCAGCT	miRNA	miR-191
t0031463	22	5 CAACGTAATCCCAAAAGCAGCT	miRNA	miR-191
t0031405	22	5 CAACGGAATCCCAAGAGCAGCT	miRNA	miR-191
t0031048	20	5 CAACGGAATCCAAAAAGCAG	miRNA	miR-191
t0030768	21	5 CAACGGAATACCAAAAGCAGC	miRNA	miR-191
t0031185	23	5 CAACGGAATCCCAAAGGCAGCTG	miRNA	miR-191
t0030783	23	5 CAACGGAATCCCAAAAGCAGCAG	miRNA	miR-191
t0030844	22	5 CAACGGAATCCCAAAAACAGCT	miRNA	miR-191
t0031236	23	5 GGGAGAGAAAGGCAGTTCCTGAA	miRNA	miR-185
t0030699	23	5 TGGAGAGAGAGGCAGTTCCTGAT	miRNA	miR-185
t0030808	20	5 TGGAGCGAAAGGCAGTTCCT	miRNA	miR-185
t0030885	23	5 TAGAGAGAAAGGCAGTTCCTGAT	miRNA	miR-185
t0030903	23	5 TGGAGAGACAGGCAGTTCCTGAA	miRNA	miR-185
t0031130	21	5 TGAGAGAAAGGAAGTTCCTGA	miRNA	miR-185
t0031139	23	5 TGGAGAGAAGGGCAGTTCCTGAT	miRNA	miR-185
t0031143	20	5 TGGAGAGAAAGACAGTTCCT	miRNA	miR-185
t0031182	23	5 TGGAGAGAAAGGCCGTTCCTGAT	miRNA	miR-185
t0031366	23	5 TGGAGAGAAAGGGAGTTCCTGAG	miRNA	miR-185
t0031508	20	5 TGGAGAGAAAAGCAGTTCCT	miRNA	miR-185
t0031547	23	5 TGGAGACAAAGGCAGTTCCTGAA	miRNA	miR-185
t0031587	21	5 AGGTAGATAGAACAGGGCTTG	miRNA	miR-1839
t0030696	23	5 AAGGGAGATAGAACAGGTCTTGA	miRNA	miR-1839
t0030701	22	5 AAAGGTAGATAGAACAGGTCTT	miRNA	miR-1839
t0030939	18	5 TATGGCACTGGTAGAATT	miRNA	miR-183
t0030993	21	5 TTGAGGGAGTAGATTGTATAG	miRNA	miR-1827
t0031313	22	5 TGAGGCAGTAGATTGTATAGGT	miRNA	miR-1827
t0031361	21	5 TGAGGGAGTAGATTGTATATT	miRNA	miR-1827
t0031391	22	5 TGAGGGAGTAGAGTGTATAGTT	miRNA	miR-1827
t0031424	25	5 TGAGGGAGTAGGTTGTATAGTTATC	miRNA	miR-1827
t0031451	21	5 TGAGGGAATAGATTGTATAGT	miRNA	miR-1827
t0031499	22	5 TGAGGGAGTAGCTTGTATAGTT	miRNA	miR-1827
t0031598	21	5 TGAGGGGGTAGATTGTATAGT	miRNA	miR-1827

t0031611	20	5 AACATTTATTGCTGTCGGTG	miRNA	miR-181b
t0030938	21	5 CAAAGTGCTTACAGTGCCGGT	miRNA	miR-17
t0031240	30	5 GGGGGCGGGGAGCGGTCGGGCGGCGGC	miRNA	miR-1607
t0031372	25	5 GGACGGGGAGCGGTCGGGCGGCGGC	miRNA	miR-1607
t0031039	20	5 TAGCAGCACGTAAGTATTGG	miRNA	miR-16
t0030897	20	5 TAGCAGCACGTAAATATTGC	miRNA	miR-16
t0031029	23	5 TAGAAGCACGTAAATATTGGCGT	miRNA	miR-16
t0031181	22	5 TAGCAGTACGTAAATATTGGAG	miRNA	miR-16
t0031210	23	5 TAGCAGCAAGTAAATATTGGCGT	miRNA	miR-16
t0031268	23	5 TAGCAGCACGTAAATATTGCCGA	miRNA	miR-16
t0031393	23	5 TAGCAGCACGGAAATATTGGCGT	miRNA	miR-16
t0031487	22	5 TAGCAGCACGTAAAGATTGGCG	miRNA	miR-16
t0031506	19	5 TAGGAGCACATCATGGTTT	miRNA	miR-15b
t0031392	19	5 TAGCAGCACATGATGGTTT	miRNA	miR-15b
t0031575	22	5 TAGCAGCAGATCATGGTTTACA	miRNA	miR-15b
t0030711	21	5 TAGCAGCACATCATGGTTTGT	miRNA	miR-15b
t0031623	20	5 TAGCTGCACATCATGGTTTA	miRNA	miR-15b
t0030785	20	5 TAGCAGCACATCAAGGTTTA	miRNA	miR-15b
t0030868	20	5 TAGCAGCACATCATGGTTCA	miRNA	miR-15b
t0030919	20	5 TAGCCGCACATAATGGTTTG	miRNA	miR-15a
t0030927	22	5 TAGCAGCACGTAATGGTTTGTG	miRNA	miR-15a
t0031101	21	5 TAGCACCACATAATGGTTTGT	miRNA	miR-15a
t0031142	19	5 TAGCAGCACATAATGGATT	miRNA	miR-15a
t0031354	20	5 TAGCAGCTCATAATGGTTTG	miRNA	miR-15a
t0031642	18	5 TAGCAGCACATAATGGGT	miRNA	miR-15a
t0030705	20	5 TAGCAGCCCATAATGGTTTG	miRNA	miR-15a
t0030782	21	5 TAGCAGCACAAAATGGTTTGT	miRNA	miR-15a
t0030833	22	5 GGATATCATCATACACTGTAAG	miRNA	miR-144*
t0030952	19	5 CTACAGTATAGATGATGGA	miRNA	miR-144
t0031077	25	5 TACAGTATAGATGATGTACTAAAAA	miRNA	miR-144
t0031513	23	5 TGAGATGAGGCACTGTAGCTATC	miRNA	miR-143
t0031603	23	5 TGAGATGAAGCACTGTAGCTTTC	miRNA	miR-143
t0030676	20	5 CCAATAAAGTAGAAAGCACT	miRNA	miR-142
t0030744	18	5 CCTATAAAGTAGAAAGCA	miRNA	miR-142
t0030831	24	5 TACCACAGGGTAGAACCTCGGACA	miRNA	miR-140
t0030861	23	5 TACCACAGCGTAGAACCACGGAC	miRNA	miR-140
t0030882	22	5 TACCACAGGGGAGAACCAAGGA	miRNA	miR-140
t0030888	23	5 TACCACAGGGTAGAACTCCGGAA	miRNA	miR-140
t0031303	22	5 TACCACAGGGTAGAAACCCGGA	miRNA	miR-140
t0031329	23	5 TACCATAGGGTAGAACCCCGGAC	miRNA	miR-140
t0031287	23	5 TACCACAGGCTAGAACCCCGGAC	miRNA	miR-140
t0030743	23	5 TACCACAGGGTAGAAGCACGGAA	miRNA	miR-140
t0031215	22	5 ACCACAGGGTAGAGCCACGGAT	miRNA	miR-140
t0031262	22	5 CCACAGGGTAGAATCACGGACA	miRNA	miR-140
t0031567	24	5 TACCACAGGGGAGAACCACGGACG	miRNA	miR-140
t0030807	24	5 TACCACAGGGTAGAACCCCGGAAT	miRNA	miR-140
t0031573	24	5 TACCACAGGGTAGAACCACGAACA	miRNA	miR-140
t0030693	22	5 ACCACAGGGTAGGACCACGGAA	miRNA	miR-140
t0030698	24	5 TACCACACGGTAGAACCACGGACA	miRNA	miR-140
t0030714	23	5 ACCACGGGGTAGAACCACGGACA	miRNA	miR-140
t0030728	23	5 AACCACAGGGTAGAACCACGGAA	miRNA	miR-140
t0030751	23	5 TACCACAGGGTAGAAGCACGGAC	miRNA	miR-140
t0030789	23	5 TCCCACAGGGTAGAACCACGGAA	miRNA	miR-140
t0030832	23	5 TACCACAGGGTAGAACCACGTAA	miRNA	miR-140
t0030858	23	5 TACCACACGGTAGAACCACGGAA	miRNA	miR-140
t0030862	22	5 TTCCACAGGGTAGAACCACGGA	miRNA	miR-140
t0030864	20	5 ACCACAGGGCAGAACCACGG	miRNA	miR-140
t0030942	22	5 ACCACAGGGTAGCACCACGGAA	miRNA	miR-140

t0030946	21	5 TACCACAGGGCAGAACCACGG	miRNA	miR-140
t0030967	23	5 TACCACAGGGTAGAACCAGGGAA	miRNA	miR-140
t0030976	24	5 TACCACAGGGTAGAAACACGGACG	miRNA	miR-140
t0031005	22	5 ACCACAGGGTAGAACCGCGGAT	miRNA	miR-140
t0031045	23	5 CACCACAGGGTAGAACCACGGAT	miRNA	miR-140
t0031095	21	5 TACCACAGGGTAGAACCGCGG	miRNA	miR-140
t0031104	22	5 TACAACAGGGTAGAAACACGGA	miRNA	miR-140
t0031108	24	5 TACCACAGCGTAGAACCACGGACA	miRNA	miR-140
t0031125	23	5 TACCACAGGGTAGAACAACGGAG	miRNA	miR-140
t0031128	22	5 AGCACAGGGTAGAACCACGGAC	miRNA	miR-140
t0031135	23	5 GCCACAGGGTAGAACCACGGACA	miRNA	miR-140
t0031138	21	5 TACCACAGGGTAGAATCACGG	miRNA	miR-140
t0031188	24	5 TACCACAGGGTAGCACCACGGACA	miRNA	miR-140
t0031196	23	5 ACCACAGGGAAGAACCACGGACT	miRNA	miR-140
t0031238	23	5 GCCACAGGGTAGAACCACGGACG	miRNA	miR-140
t0031270	23	5 TACCACAGGGTAAAACCACGGAT	miRNA	miR-140
t0031316	22	5 CAGGGCAATGTTAAAAGGGCAT	miRNA	miR-130a
t0031341	24	5 TCTGGGCAACAAAGGGAGACCTGA	miRNA	miR-1285
t0031377	26	5 TCGTACCGTGAGTAATAATGCGAATC	miRNA	miR-126
t0031385	19	5 AGGATGAGCAAAGAAAGTA	miRNA	miR-1255a
t0031396	19	5 GTGAGGACTCGGGAGGGGG	miRNA	miR-1224
t0031414	21	5 GTGAGGACTCGGGAGGGGGAG	miRNA	miR-1224
t0031519	22	5 AGCAGCATTGTACAGGGCTTTG	miRNA	miR-107b
t0031523	22	5 AGCAGCATTGTACAGGGCTCTT	miRNA	miR-107
t0031531	20	5 TAAAGCGCTGACAGTGCAGA	miRNA	miR-106b
t0031550	21	5 TAAAGTGCTGACAGTGCAGCT	miRNA	miR-106b
t0031562	21	5 TAAAGTGCTGACAGTGCAGAC	miRNA	miR-106b
t0031638	22	5 CGCAGCATTGTACAGGGCTATG	miRNA	miR-103a
t0031656	23	5 AGCAGCATTGTACACGGCTATGA	miRNA	miR-103a
t0030991	21	5 AGCAGCATTGTAGAGGGCTAT	miRNA	miR-103a
t0031325	20	5 AGCAGCATTGTACAGGGCTG	miRNA	miR-103a
t0031299	23	5 AGCAGCATTGTAGAGGGCTATGA	miRNA	miR-103a
t0030673	23	5 AGCAGCATTGTACAGGGATATGT	miRNA	miR-103a
t0030694	21	5 AACAGTACTGTGATAACTGAA	miRNA	miR-101c
t0031010	21	5 GTACAGTACTATGATAACTGA	miRNA	miR-101b
t0031289	21	5 TACCGTACTGTGATAACTGAA	miRNA	miR-101
t0030860	21	5 TACAGTACTGTGATCACTGAA	miRNA	miR-101
t0030707	21	5 GTACAGTACTGAGATAACTGA	miRNA	miR-101
t0030947	22	5 TAAAGTACTGTGATAACTGAAG	miRNA	miR-101
t0031370	22	5 GTACAGTACTGTGATGACTGAA	miRNA	miR-101
t0030748	20	5 TACAGTACTGTGATAATTGA	miRNA	miR-101
t0030763	21	5 TACAGTACTGTGATAAGTGAA	miRNA	miR-101
t0030879	21	5 GTACAGGACTGTGATAACTGA	miRNA	miR-101
t0030920	21	5 TACAGTCCTGTGATAACTGAA	miRNA	miR-101
t0031064	22	5 GTACAGTACTGTAATAACTGAA	miRNA	miR-101
t0031630	20	5 TACAGTACTGCGATAACTGA	miRNA	miR-101
t0030817	22	5 TACAGTACTGGGATAACTGAAA	miRNA	miR-101
t0030909	22	5 GTACAGTACTGTGTTAACTGAA	miRNA	miR-101
t0030702	21	5 GTACAGTACCGTGATAACTGA	miRNA	miR-101
t0030729	22	5 TACAGTACCGTGATAACTGAAG	miRNA	miR-101
t0030756	22	5 GTCCAGTACTGTGATAACTGAA	miRNA	miR-101
t0031001	23	5 TGAGGTAGTAGATTGAATAGTTA	miRNA	let-7k
t0031082	20	5 TGAGGTAGTAGATTGTGCAG	miRNA	let-7j
t0031099	22	5 TGAGGTAGTAGTTCGTGCTGTT	miRNA	let-7i
t0031205	22	5 TGAGGTAGTAGTTTGTGCTGTC	miRNA	let-7i
t0031312	22	5 TGAGGTCGTAGTTTGTGCTGTT	miRNA	let-7i
t0031398	21	5 TGAGGTAGTAGTTTGTGCGGT	miRNA	let-7i
t0031416	22	5 TGAGATAGTAGTTTGTGCTGTT	miRNA	let-7i

t0031443	22	5 TGAGGTAGTCGTTTGTGCTGTT	miRNA	let-7i
t0031450	22	5 TGAGGTAGTAGCTTGTGCTGTT	miRNA	let-7i
t0031452	20	5 TGAGGAAGTAGTTTGTGCTG	miRNA	let-7i
t0031478	20	5 TGAGGTAGTAGTTTATGCTG	miRNA	let-7i
t0031493	21	5 TGGGGTAGTAGTTTGTGCTGT	miRNA	let-7i
t0031646	21	5 TGAGGTAGTCGTTTGTACAGT	miRNA	let-7g
t0031589	22	5 TGAGGGAGGAGTTTGTACAGTT	miRNA	let-7g
t0031239	20	5 TGAGGTAGTAGTTTGTACTG	miRNA	let-7g
t0030774	26	5 TGAGGTAGTAGTTTGTACAGTCGGTT	miRNA	let-7g
t0030869	22	5 TGAGGTTGTAGTTTGTACAGTT	miRNA	let-7g
t0031080	21	5 TGAGGTAGTAGTTTGGACAGT	miRNA	let-7g
t0031118	22	5 TGAGGTAGTAGATTGTATAGCA	miRNA	let-7f-5p
t0031157	21	5 TGAGGTAGTAGATTGTATAAC	miRNA	let-7f-5p
t0031160	20	5 TGAGGTAGTAGATTGTACTT	miRNA	let-7f-5p
t0031230	21	5 GAGGTAGTGGATTGTATAGTT	miRNA	let-7f-5p
t0031259	22	5 TGAGGTAGTGGATTGTGTAGTT	miRNA	let-7f-5p
t0031459	23	5 TGCGGTAGTAGATTGTATAGTTT	miRNA	let-7f
t0031488	23	5 TGAGGTAGTAGATTGTATCGTTG	miRNA	let-7f
t0030945	19	5 TGAGGTAGTAGATTGTAAA	miRNA	let-7f
t0030948	24	5 TGAGGTAGTAGATTGTATAGTAAT	miRNA	let-7f
t0031199	23	5 TGAGGTAGTAGATTGTTTAGTTA	miRNA	let-7f
t0031286	23	5 TGAGGTAGTAGTTTGTATAGTTG	miRNA	let-7f
t0031383	23	5 CGAGGTAGTAGATTGTATAGTTT	miRNA	let-7f
t0031474	20	5 GAGGAAGTAGATTGTATAGT	miRNA	let-7f
t0030770	23	5 TGAGGTAGTAGATTGTATAGCTT	miRNA	let-7f
t0031098	23	5 TGAGATAGTAGATTGTATAGTTA	miRNA	let-7f
t0031285	21	5 GGGGTAGTAGATTGTATAGTT	miRNA	let-7f
t0031415	22	5 TGAGGTAGAAGATTGTATAGTA	miRNA	let-7f
t0031648	19	5 TGAGGTAGTAGATTGTATC	miRNA	let-7f
t0030669	23	5 TGAGGTAGAAGATTGTATAGTTA	miRNA	let-7f
t0030730	22	5 TAAGGTAGTAGATTGTATAGTA	miRNA	let-7f
t0030737	19	5 TGAGGTAATAGATTGTATA	miRNA	let-7f
t0030788	23	5 TGAGGTAGTAGATTGTCTAGTTG	miRNA	let-7f
t0030823	23	5 CTGAGGTGGTAGATTGTATAGTT	miRNA	let-7f
t0030835	23	5 TGAGGTAGCAGATTGTATAGTTA	miRNA	let-7f
t0030849	22	5 TGAGGTAGTAGATTGTGGAGTT	miRNA	let-7f
t0030855	22	5 TGAGGTAGTAGCTTGTATAGGT	miRNA	let-7f
t0030922	23	5 TGAGGTAGTAGATTGTAAAGTTT	miRNA	let-7f
t0031103	22	5 TGAGGTGGTAGATTGTATAGTA	miRNA	let-7f
t0031145	21	5 AGAGGTAGTAGGTTGCAAAGT	miRNA	let-7d
t0031159	20	5 AGAGGTAGTAGGTCGCATAG	miRNA	let-7d
t0031169	21	5 AGAGGTAGTAGGTTGGATAGT	miRNA	let-7d
t0031244	22	5 AGAGGGAGTAGGGTGCATAGTT	miRNA	let-7d
t0031264	22	5 AGAGGTAGTAGGGTGAATAGTT	miRNA	let-7d
t0031294	21	5 AGAGGTAATAGGTTGCATAGT	miRNA	let-7d
t0031300	24	5 TGAGGTAGTAGGTTGTGTGGTTCA	miRNA	let-7b
t0031315	24	5 CGAGGTAGTAGGTTGTGTGGTTAA	miRNA	let-7b
t0031327	23	5 TGAGGTAGTAGGTCGTGTGGTTA	miRNA	let-7b
t0031339	19	5 TGAGGAAGTAGGTTGTGTG	miRNA	let-7b
t0031444	24	5 TGAGGTAGTAGGTTGTGGGGTTAT	miRNA	let-7b
t0031561	19	5 TGAGGTAGTAGGCTGTGTG	miRNA	let-7b
t0031614	23	5 TGAGGCAGTAGGTTGTGTGGTAA	miRNA	let-7b
t0030742	23	5 TGCGGTAGTAGGTTGTGTGGTTT	miRNA	let-7b
t0030781	22	5 TGAGGTAATAGGTTGTGTGGTA	miRNA	let-7b
t0030916	21	5 TGAGGTAGTAGGTTGTGGGGA	miRNA	let-7b
t0031151	20	5 TGAGGTAGTAAGTTGTGTGG	miRNA	let-7b
t0031446	23	5 TGAGGTAGTAGGTTGTATGGTTT	miRNA	let-7b
t0031483	19	5 TGAGGTAGTAGGTTATGTG	miRNA	let-7b

t0030671	19	5	TGAGGTAGCAGGTTGTGTG	miRNA	let-7b
t0030733	23	5	TGAGATAGTAGGTTGTGTGGTTA	miRNA	let-7b
t0030753	20	5	TAGTAGTAGGTTGTGTGGTT	miRNA	let-7b
t0030847	23	5	TGAGGTAGTAGATTGTGTGGTTT	miRNA	let-7b
t0030876	23	5	TGAGGTAGTAGGTTGCGTGGTTA	miRNA	let-7b
t0030924	22	5	TGAGGTAGTAGGTTATGTGGTA	miRNA	let-7b
t0030951	20	5	TGAGGTAGTAGGTTGTATTG	miRNA	let-7
t0031015	20	5	TGAGCTAGTAGGTTGTATAG	miRNA	let-7
t0031132	20	5	TGAGGTAGTTGGTTGTATAG	miRNA	let-7
t0031225	22	5	TGAGGTGGTGGGTTGTATAGTT	miRNA	let-7
t0031235	21	4	CAAAGTGCTGTTCGTGCAGGG	miRNA	miR-93
t0031308	23	4	CAAAGTGCTGTTCATGCAGGTAG	miRNA	miR-93
t0031336	21	4	CAAAGGGCTGTTCGTGCAGGT	miRNA	miR-93
t0031386	23	4	CAAAGAGCTGTTCGTGCAGGTAG	miRNA	miR-93
t0031410	22	4	CAAAGTGCTATTCGTGCAGGTA	miRNA	miR-93
t0031530	23	4	TATTGCACTTGTCCCGGCCTGGT	miRNA	miR-92b-3p
t0031548	22	4	AGGGACGGGACGCGGGGCAGTG	miRNA	miR-92b*
t0031571	23	4	TATTGCACTTGTCCCGGCTTTTG	miRNA	miR-92b
t0031572	22	4	TATTGCACTTGTCTCGGTCTGT	miRNA	miR-92a
t0030738	23	4	TATTGCAATTGTCCCGGCCTGTA	miRNA	miR-92a
t0030883	22	4	TATTGCACTTTTCCCGGCCTGT	miRNA	miR-92a
t0031320	21	4	TATTGCACTTGTCCCGGCATG	miRNA	miR-92a
t0031473	21	4	TATTGCACTTGTCCAGGCCTG	miRNA	miR-92a
t0032294	19	4	TGGAGAGAAAGGAAGTTGA	miRNA	miR-765
t0032355	21	4	TGGAGAGAAAGGAAGTTCATG	miRNA	miR-765
t0032578	26	4	AGGGCGCGCGGGTCGGGGCGGCGGCA	miRNA	miR-638
t0032759	22	4	TACGTCATCGTTGTCATCGTCC	miRNA	miR-598
t0032851	22	4	TGGTGCAAAAGTAATGGCGGTT	miRNA	miR-548q
t0032357	22	4	AAAAACCACAATTACTTTTGCA	miRNA	miR-548aa
t0031880	24	4	TCTGGGCACAGGCGGATGGACCGA	miRNA	miR-5107
t0031746	24	4	TCTGGGCACAGGCGGATGGAAAGA	miRNA	miR-5107
t0031755	23	4	TCCTGTACTAAGCTGCCCCGAGT	miRNA	miR-486-5p
t0031852	20	4	TCCCGTACTGAGCTGCCCCG	miRNA	miR-486-5p
t0031862	20	4	TCCTATACTGAGCTGCCCCG	miRNA	miR-486-5p
t0032661	26	4	TCCTGTACTGAGCTGCCCCGCGGTCC	miRNA	miR-486-5p
t0032950	23	4	TCCCGTACTGAGCTGCCCCGAGA	miRNA	miR-486-5p
t0031811	22	4	TCCTGTACGGAGCTGCCCCGAG	miRNA	miR-486-5p
t0032522	20	4	CCCTGTACTGAGCTGCCCCG	miRNA	miR-486-5p
t0032431	21	4	TCCTGTTCTGAGCTGCCCCGA	miRNA	miR-486-5p
t0032301	23	4	TCCTGTAATGAGCTGCCCCGAGT	miRNA	miR-486-5p
t0032045	20	4	TCCTGCACTGAGCTGCCCCG	miRNA	miR-486-5p
t0032586	30	4	TCCTGTACTGAGCTGCCCCGAGCGAAGC(	miRNA	miR-486-5p
t0032071	22	4	TCTTGTACTGAGCTGCACCGAG	miRNA	miR-486-5p
t0032393	21	4	TCCAGTACTGAGCTGCCCCGA	miRNA	miR-486-5p
t0031693	20	4	TCCTGTATTGAGCTGCCCCG	miRNA	miR-486-5p
t0031884	26	4	TCCTGTACTGAGCTGCCCCGAGAAGT	miRNA	miR-486-5p
t0031910	22	4	TCCTGTACTGAGCTGTCCCGCG	miRNA	miR-486-5p
t0031947	20	4	TCCTGTACTGAGCTACCCCG	miRNA	miR-486-5p
t0032035	21	4	TCCTGTACGGAGCTGCCCCGA	miRNA	miR-486-5p
t0032128	26	4	TCCTGTACTGAGCTGCCCCGCGAAGA	miRNA	miR-486-5p
t0032156	21	4	TCCTGTACTGAGCTCCCCCGC	miRNA	miR-486-5p
t0032227	23	4	TCCTGTACTGAGGTGCCCCGAGA	miRNA	miR-486-5p
t0032259	21	4	TCTTGTACTGAGCTGCCCCGC	miRNA	miR-486-5p
t0032315	25	4	TCCTGTACTGAGCTGCCCCGAGGGA	miRNA	miR-486-5p
t0032385	23	4	TCCTGTACTGAGCTGCCCTGAGA	miRNA	miR-486-5p
t0032437	22	4	TCAGGCAAAGGGATATTTACAG	miRNA	miR-4742-5p
t0032461	23	4	TGTAGAGCAAGGAGCAGGAAGCT	miRNA	miR-4732-5p
t0032489	23	4	TGTAGAGCAGGGAGCAGGAAACT	miRNA	miR-4732-5p

t0032517	23	4	TGTAGAGCAGGGAGCAGGAAGCC	miRNA	miR-4732-5p
t0032561	23	4	TGTAAAGCAGGGAGCAGGAAGCT	miRNA	miR-4732-5p
t0032731	23	4	TGAAGAGCAGGGAGCAGGAAGCT	miRNA	miR-4732-5p
t0032735	18	4	TATATACACACACACACA	miRNA	miR-467f
t0032748	31	4	AAGGGCTGGGTCGGTCGGGCTGGGGCGC	miRNA	miR-4651
t0032758	26	4	AGGGCTGGGTCGGTCGGGCTGGGGAG	miRNA	miR-4651
t0032764	22	4	GAAGGGTCGGGGCGGCAGGGGC	miRNA	miR-4516
t0032781	27	4	GGGGAGAAGGGTCGGGGCGGCAGGGGC	miRNA	miR-4516
t0032784	21	4	TGAGGGAGTAGGTTGTGTGTT	miRNA	miR-4510
t0032847	22	4	TGAGGGAGTAGGGTGTGTGGTA	miRNA	miR-4510
t0032228	21	4	TGAGGGAGTAGGTTGTTTGGT	miRNA	miR-4510
t0031692	23	4	AAACCGTTACCATTACTGTGTTT	miRNA	miR-451
t0031944	19	4	AAGCCGTTACCATTACTGA	miRNA	miR-451
t0032080	20	4	AAACCGTTACGATTACTGAG	miRNA	miR-451
t0032713	21	4	AAACAGTTACCATTAATGAGT	miRNA	miR-451
t0032949	21	4	CAACCGTTACCATTACTGAGT	miRNA	miR-451
t0031791	25	4	AAACCGTTATCATTACTGAGTTTAG	miRNA	miR-451
t0031978	21	4	AAACCTTTACCATTGCTGAGT	miRNA	miR-451
t0032016	23	4	AAACCGTTACCAATACTGAGTTT	miRNA	miR-451
t0032135	19	4	AAACCGTCACCATTACTGA	miRNA	miR-451
t0032838	20	4	AAAGCGTTACCATTACTGAG	miRNA	miR-451
t0031922	22	4	ACACCGTTACCATTACTGAGTT	miRNA	miR-451
t0032181	24	4	AAACTGTTACCATTACTGAGTTTA	miRNA	miR-451
t0032511	21	4	AAACCTTTACCATTACCGAGT	miRNA	miR-451
t0031814	25	4	AAACCGTTACCATTACTGAGCTTAG	miRNA	miR-451
t0031818	22	4	AAACCTTTAACATTACTGAGTT	miRNA	miR-451
t0031875	22	4	AAACATTTACCATTACTGAGTT	miRNA	miR-451
t0031879	22	4	AAACCGTTACCATAACTGAGTT	miRNA	miR-451
t0031908	23	4	AAACGGTTACCATTACTGAGTTT	miRNA	miR-451
t0031925	25	4	AAACCGTTACCATTACTGAGTTTTT	miRNA	miR-451
t0031926	20	4	ACACCGTTACCATTACTGAG	miRNA	miR-451
t0031937	19	4	AAACCGTTACCATTACTGC	miRNA	miR-451
t0031948	20	4	CAACCGTTACCATTACTGAG	miRNA	miR-451
t0031954	22	4	CAACCGTTACCATTACTGAGTT	miRNA	miR-451
t0031957	18	4	AACCGTTACCATTACTGA	miRNA	miR-451
t0032013	25	4	AAACCGTTGCCATTACTGAGTTTAG	miRNA	miR-451
t0032020	24	4	AAACCTTACCATTACTGAGTTTAG	miRNA	miR-451
t0032096	23	4	AAACCGTTACCATTTCTGAGTTT	miRNA	miR-451
t0032099	24	4	AAACCGTTACCCTTACTGAGTTTA	miRNA	miR-451
t0032140	23	4	AAACCGTTACCATTACTGAGGAA	miRNA	miR-451
t0032178	20	4	GGGTGCGGGCCGGCGGGGGC	miRNA	miR-4466
t0032271	20	4	AATCCGAGTCACGGCACCAA	miRNA	miR-4454
t0032380	21	4	AAAAGCTGGGCTGAGAGGGCG	miRNA	miR-4429
t0032487	21	4	ACCAGCATTGTACAGGGCTAT	miRNA	miR-4289
t0032584	21	4	CAGCAGCAATTCATGTTTTGC	miRNA	miR-424
t0032587	19	4	CAGCAGCAGTTCATGTTTT	miRNA	miR-424
t0032603	22	4	TGAGGGGCTGAGAGCGAGACTT	miRNA	miR-423-5p
t0032663	21	4	TGAGGGGCAGAGGGCGGGACT	miRNA	miR-423-5p
t0032719	24	4	TGGGGGGCAGAGAGCGAGACTTTT	miRNA	miR-423-5p
t0032825	24	4	TGAGGGACAGAGAGCGAGACTTTT	miRNA	miR-423-5p
t0032844	23	4	TGAGGGGCAGAGAGAGAGACTTG	miRNA	miR-423-5p
t0032909	24	4	TGAGGGGCAGAGAGAGAGACTTAA	miRNA	miR-423-5p
t0032919	22	4	TGAGGGGAAGAGAGCGAGAATT	miRNA	miR-423-5p
t0032959	23	4	TAAGGGGCAGAGAGCGAGACTTA	miRNA	miR-423-5p
t0032162	23	4	TGAGGGGCGGAGGGCGAGACTTT	miRNA	miR-423-5p
t0032669	24	4	TGAGGAGCAGAGAGCGAGACTTTT	miRNA	miR-423-5p
t0032242	24	4	TGAGGGGCAGAGAGCGAGCCTTTA	miRNA	miR-423-5p
t0032031	19	4	TGAGGGGCAGAGAGCAAGA	miRNA	miR-423-5p

t0032563	22	4	TGAGGGGAAGAGAGAGAGACTT	miRNA	miR-423-5p
t0031681	20	4	TGAGGGGCAGAGAACGAGAC	miRNA	miR-423-5p
t0031695	23	4	TGCGGGGCAGAGAGCGAGACTTA	miRNA	miR-423-5p
t0031714	23	4	TGAGGGGCAGATAGCGAGAATTT	miRNA	miR-423-5p
t0031828	23	4	TGAGGGGCAGAGAGCGAGACTCA	miRNA	miR-423-5p
t0032011	24	4	TGAGGGGCAGAGAGCTAGACTTTT	miRNA	miR-423-5p
t0032297	20	4	TGAGGGGCAGAGAGCGAGGC	miRNA	miR-423-5p
t0032335	27	4	ACCCGGGGGGCCGGCGGCGGCGGCGAC	miRNA	miR-3885-5p
t0032347	25	4	GGGCCGGCGGCGGCGGCGACTCTGG	miRNA	miR-3885-5p
t0032397	20	4	CGGCGGCGGGGGTGTGGGGT	miRNA	miR-3885-5p
t0032470	19	4	TGGGGGCGGCGGCGGGGGG	miRNA	miR-3885-5p
t0032539	26	4	GTCGGGGCGGCGGCGGCGGTGGCATC	miRNA	miR-3885-5p
t0032647	26	4	GGGGCCGGCGGCGGCGGCGACTCTGG	miRNA	miR-3885-5p
t0032677	20	4	GCGGCGGCGGCGGGGGTGTA	miRNA	miR-3885-5p
t0032801	26	4	GGCGGCGGCGGCGACTCTGGACGCGA	miRNA	miR-3885-5p
t0032826	27	4	GGCGGCGGCGGCGACTCTGGACGCGAG	miRNA	miR-3885-5p
t0032890	21	4	GGCGGCGTCGGCGGCGGCGGG	miRNA	miR-3885-5p
t0032913	22	4	CTGGACTTGGAGGCAGAAGGCA	miRNA	miR-378f
t0032934	25	4	ACTGGACTTGGAGTAAGAAGGCATC	miRNA	miR-378
t0032968	23	4	ACTGGACTTGGAGTAAGAAGGCA	miRNA	miR-378
t0031840	24	4	ACTGGACTTGGAGTCAGAAGGCGT	miRNA	miR-378
t0031956	21	4	ACTGGACTTGGAGTCAGCAGG	miRNA	miR-378
t0032070	22	4	ATATAATACAACCCGCTAAGTG	miRNA	miR-374
t0032303	22	4	AATTGCACGGATCCATCTGTA	miRNA	miR-363
t0032382	21	4	ACAAAAAAAAAGCCCAACCCT	miRNA	miR-3613-3p
t0032404	23	4	TGAAGACTGAAGCGGAGAAGGGT	miRNA	miR-3526
t0032632	29	4	AACTTTGAAGACTGAAGTGGCGAAGGGT	miRNA	miR-3526
t0032769	23	4	TGAAGACTGAAGGGGAGAAGGGG	miRNA	miR-3526
t0032967	28	4	AACTTTGAAGACTGAAGTGGAGGAGGGT	miRNA	miR-3526
t0032985	28	4	AACTTTGAAGACTGAAGAGGAGAAGGGT	miRNA	miR-3526
t0032943	28	4	AACTTTGAAGACTGAAGCGGAGAAGGGT	miRNA	miR-3526
t0032023	23	4	TGAAGACTGAAGTGGAGAAGGGC	miRNA	miR-3526
t0032398	22	4	GAAGACTGAAGTGGAGAAGGGA	miRNA	miR-3526
t0032412	27	4	AACTTTGAAGACTGAAGCGGAGAAGGG	miRNA	miR-3526
t0032886	28	4	AACTTTGAAGACTGAGGTGGAGAAGGGT	miRNA	miR-3526
t0032529	20	4	AGAAGATTGCCGCTCGTTCT	miRNA	miR-3494
t0032087	20	4	CCCTCGAACTGTTGTGGCCA	miRNA	miR-3487
t0032734	20	4	TCCTCGAACTGTTGTGGCCA	miRNA	miR-3487
t0031696	23	4	CCTCACACAGAAATCGCACCCGT	miRNA	miR-342-3p
t0031701	25	4	TCTCACACAGAAATCGCACCCGGCT	miRNA	miR-342-3p
t0031715	23	4	TCTCACGCAGAAATCGCACCCGT	miRNA	miR-342-3p
t0031769	22	4	TCTCACCCAGAAATCGCACCCG	miRNA	miR-342-3p
t0032367	23	4	TCTCACCCAGAAATCGCACCCGT	miRNA	miR-342-3p
t0032448	22	4	TCTCACACAGAAATCGCACACG	miRNA	miR-342-3p
t0032727	23	4	AAAAGCTGGGATGAGAGGGCGTT	miRNA	miR-320d
t0032828	23	4	AAAAGCTGGGTTGAAAGGGCGTT	miRNA	miR-320d
t0032862	23	4	AAAAGCTGGGTTGAGAGGGTGAG	miRNA	miR-320d
t0032961	22	4	AAAAGCTGGGTTGAGAGGTCGA	miRNA	miR-320d
t0032435	23	4	AAAAGCTGGGTTGCGAGGGCGTA	miRNA	miR-320d
t0031903	23	4	AAAAGCTCGGTTGAGAGGGCGTA	miRNA	miR-320d
t0032400	23	4	AAAGGCTGGGTTGAGAGGGCGTT	miRNA	miR-320d
t0031759	22	4	AACAGCTGGGTTGAGAGGGCGT	miRNA	miR-320d
t0031999	23	4	AAAAGCTGGGTTGAGAGGCCGAA	miRNA	miR-320d
t0032026	22	4	AAAAGCTGGGTTGACAGGGCGT	miRNA	miR-320d
t0032515	23	4	AAACGCTGGGTTGAGAGGGCGTA	miRNA	miR-320d
t0032599	23	4	GAAAGCTGGGTTGAGAGGGCGTT	miRNA	miR-320d
t0032633	23	4	AACAGCTGGGTTGAGAGGGCGTA	miRNA	miR-320d
t0031705	22	4	AAAAGCTGGGATGAGAGGGCAA	miRNA	miR-320b

t0031781	23	4	ACAAGCTGGGTTGAGAGGGCGAA	miRNA	miR-320a
t0031807	21	4	AAAAGCTGGGGTGAGAGGGCG	miRNA	miR-320a
t0031904	22	4	AAAAGCGGGGTTGAGAGGGCGA	miRNA	miR-320a
t0031968	23	4	AAAAGCTGAGTTGAGAGGGCGAT	miRNA	miR-320a
t0032014	23	4	AAAAGCTGGGTTGGGAGGGCGAT	miRNA	miR-320a
t0032154	21	4	AAAAGCTGGATTGAGAGGGCG	miRNA	miR-320a
t0032204	23	4	ACAAGCTGGGTTGAGAGGGCGAT	miRNA	miR-320a
t0032214	22	4	AAAAGCAGGGTTGAGAGGGCGA	miRNA	miR-320a
t0032283	21	4	AAAAGATGGGTTGAGAGGGCG	miRNA	miR-320a
t0032494	21	4	GAAAGCTGGGTTGAGAGGGCG	miRNA	miR-320a
t0032842	23	4	AACAGCTGGGTTGAGAGGGCGAA	miRNA	miR-320a
t0032920	23	4	AATCTGAGAAGGCGCACAAGGCT	miRNA	miR-3200-5p
t0031960	22	4	TCTGGGAGGTTGTAGCAGGGGA	miRNA	miR-3192
t0031699	21	4	GTGGAAGGTAGACGGCCCGAG	miRNA	miR-3190
t0031911	24	4	GGCTGGTCTGAGTGCAGTGGTGTT	miRNA	miR-3135b
t0031918	21	4	TATTGCACTTATCCCGGCCTG	miRNA	miR-311a
t0031979	24	4	TGTAAACATCCCCGACTGGAAGGT	miRNA	miR-30d
t0032000	21	4	TGTAAACATCCTACAATCTCA	miRNA	miR-30c
t0032176	24	4	TTCACAGTGGCTAAGTTCTGCCTC	miRNA	miR-27a-3p
t0032282	20	4	GGCGGATGTAGACAAGTGGA	miRNA	miR-270
t0032289	21	4	TTCAAGTAATTCAGGATAGGA	miRNA	miR-26b
t0032536	21	4	TTCAAGTAATTCGGGATAGGT	miRNA	miR-26b
t0032751	22	4	TTCGAGTAATTCAGGATAGGTT	miRNA	miR-26b
t0033001	22	4	TTCAAGTAATTCAGGATAGGAT	miRNA	miR-26b
t0032543	22	4	TTCAAGTAATTCAGGTTAGGTT	miRNA	miR-26b
t0032499	22	4	TCCAAGTAATCCAGGATAGGCT	miRNA	miR-26a
t0032730	22	4	TTCAAGTGATCCAGGATAGGCT	miRNA	miR-26a
t0032766	24	4	TTCAAGTAATCCAGGATAGGCTGA	miRNA	miR-26a
t0032768	22	4	TTCAAGTAATCCAGGACAGGCT	miRNA	miR-26a
t0032670	22	4	TTCAAGCAATCCAGGATAGGCT	miRNA	miR-26a
t0031703	21	4	AGGCGGAGACTTGGGCACTTG	miRNA	miR-25*
t0031698	22	4	CTTTGCACTTGTCTCGGTCTGA	miRNA	miR-25
t0032137	21	4	CATTGCACTTGGCTCGGTCTG	miRNA	miR-25
t0031736	21	4	CATTGCACTTGACTCGGTCTG	miRNA	miR-25
t0032509	22	4	CATTGCACTTGTCTCGGTGTGA	miRNA	miR-25
t0032777	21	4	CATTGCACTTGTGTCGGTCTG	miRNA	miR-25
t0032879	26	4	TGGCTCAGTTCAGCAGGAACAGGATC	miRNA	miR-24b
t0032923	21	4	TGGCTCAGTTCAGCAGGAACC	miRNA	miR-24b
t0032139	23	4	TGACTCAGTTCAGCAGGAACAGT	miRNA	miR-24
t0032229	24	4	TGGCTCAGTTCAGCAGGCACAGTT	miRNA	miR-24
t0032253	19	4	ATCAAATTGCCAGGGATTT	miRNA	miR-23a
t0032795	22	4	TCACATTGCCAGGGATTTCCAA	miRNA	miR-23a
t0032891	19	4	ATCACATTGCCAGGGGTTT	miRNA	miR-23a
t0031946	20	4	ATCACATTGCCAGGGCTTTC	miRNA	miR-23a
t0031817	21	4	AAGCTGCCAGTTGAAGAACTT	miRNA	miR-22-3p
t0032293	22	4	TGACTGGGGCGGAACATCTGTT	miRNA	miR-219-2-3p
t0032518	22	4	AGTGAGATTGTTGCATATTTTT	miRNA	miR-2162-5p
t0032628	22	4	TTGGGGAAACGGCCGCTGGGTG	miRNA	miR-2110
t0032658	21	4	TAGCTTATCAGACTGATGTTT	miRNA	miR-21
t0032210	21	4	TAGATTATCAGACTGATGTTG	miRNA	miR-21
t0032313	22	4	TAGCAGCACATAAATATTGGCA	miRNA	miR-195
t0032124	19	4	TAGCAGCACATAAATATTG	miRNA	miR-195
t0032712	23	4	TGACCTATGAATTGAAAGCCAGA	miRNA	miR-192
t0032169	21	4	CTGACCTATAAATTGACAGCC	miRNA	miR-192
t0032473	20	4	TGACCTATGAATTGACAGAC	miRNA	miR-192
t0032594	21	4	CTGACCTATGAATTAACAGCC	miRNA	miR-192
t0032624	21	4	CCGACCTATGAATTGACAGCC	miRNA	miR-192
t0032262	22	4	AACGGAATCCCAAAAGCAACTG	miRNA	miR-191

t0031802	22	4	AACGGAATACCAAAAGCAGCTG	miRNA	miR-191
t0032078	22	4	CAACGGAATCCCAAAAGGAGCT	miRNA	miR-191
t0032196	19	4	AACGGAATCCCAAAAGCCG	miRNA	miR-191
t0032445	21	4	CAACGGAATCCCAGAAGCAGC	miRNA	miR-191
t0032582	21	4	CAACGGAATCCAAAAAGCAGC	miRNA	miR-191
t0032792	22	4	CAACGGAATCCCACAAGCAGCT	miRNA	miR-191
t0032820	23	4	CAACGGAATCCCAAAAGCACCTG	miRNA	miR-191
t0031680	23	4	CAACGGACTCCCAAAAGCAGCTG	miRNA	miR-191
t0032247	20	4	CAACGGAATACCAAAAGCAG	miRNA	miR-191
t0032486	24	4	CAACGGAATCCCAAAAGCACCTGA	miRNA	miR-191
t0032595	24	4	CAACGGAATACCAAAAGCAGCTGA	miRNA	miR-191
t0032753	22	4	AACGGGATCCCAAAAGCAGCTG	miRNA	miR-191
t0031672	23	4	CAACGGAATCCCACAAGCAGCTG	miRNA	miR-191
t0031876	23	4	AAAGGAATCCCAAAAGCAGCTGA	miRNA	miR-191
t0031933	22	4	CAACGGACTCCCAAAAGCAGCT	miRNA	miR-191
t0031959	24	4	TGGAGAGAAAGGCAGTTCCTGACG	miRNA	miR-185
t0032056	22	4	TGAGAGAAAGGCAGTTCCTGAA	miRNA	miR-185
t0032180	23	4	TGGAGAGAAAGGCTGTTCCTGAT	miRNA	miR-185
t0032207	23	4	TGGGGAGAAAGGCAGTTCCTGAG	miRNA	miR-185
t0032266	23	4	TGGAGAGATAGGCAGTTCCTGAA	miRNA	miR-185
t0032286	23	4	AGGAGAGAAAGGCAGTTCCTGAT	miRNA	miR-185
t0032334	23	4	TGGAGAGAAAGGCAGATCCTGAG	miRNA	miR-185
t0032374	20	4	TGGAGAGAAAGGGAGTTCCT	miRNA	miR-185
t0032379	23	4	TGGAGAGAAAGGCAGTTCCCGAG	miRNA	miR-185
t0032477	22	4	TGGAGGGAAAGGAAGTTCCTGA	miRNA	miR-185
t0032565	23	4	TGGAGAGAAAGGCAGTTCCTAAG	miRNA	miR-185
t0032580	23	4	TGGAGAGAAAGGCAGTCCCTGAG	miRNA	miR-185
t0032689	23	4	TGGAGAGAAAGACAGTTCCTGAG	miRNA	miR-185
t0031685	20	4	TGGAGAGAAAGGCAGTTGCT	miRNA	miR-185
t0031730	20	4	TGAGAGAAAGGCAGTTCCTG	miRNA	miR-185
t0031994	28	4	TGGAGAGAAAGGCAGTTCCTGAAAAAAA	miRNA	miR-185
t0032034	23	4	TGGAAAGAAAGGCAGTTCCTGAT	miRNA	miR-185
t0032063	23	4	TGGAGAGAATGGCAGTTCCTGAT	miRNA	miR-185
t0032125	23	4	TGGAGAGAAAGGCAGTTCATGAG	miRNA	miR-185
t0032292	22	4	TGGAGAGAAAGGCAGTTCCTTT	miRNA	miR-185
t0032390	23	4	AGACCTACTTATCTACCAACAGA	miRNA	miR-1839-3p
t0032410	22	4	AGGGAGATAGAACAGGTCTTGA	miRNA	miR-1839
t0032438	20	4	AAGGGAGATAGAACAGGTCT	miRNA	miR-1839
t0032450	22	4	AAGGTAGATAGAACAGGCCTTG	miRNA	miR-1839
t0032454	21	4	AGGTAGATGGAACAGGTCTTG	miRNA	miR-1839
t0032462	22	4	TGAGGGAGCAGATTGTATAGTT	miRNA	miR-1827
t0032607	22	4	TGAGGGAGTAGATTGTTTAGTT	miRNA	miR-1827
t0032615	20	4	TGAGGGAGTAGATTATAGTT	miRNA	miR-1827
t0032660	22	4	TGAGGGAATAGATTGTATAGTT	miRNA	miR-1827
t0032721	21	4	TGAGGGAGTAGATTGTATAGA	miRNA	miR-1827
t0032785	22	4	TGAGGAAGTAGATTGTATAGGT	miRNA	miR-1827
t0032894	20	4	TGAGGGAGTAGATTGAATAG	miRNA	miR-1827
t0032928	22	4	TGAGGGAGTAGATTGTATAGGT	miRNA	miR-1827
t0032046	20	4	TGAGGGAGTAGATTGTTTAG	miRNA	miR-1827
t0032197	22	4	TGAGGGAGGAGGTTGTATAGTT	miRNA	miR-1827
t0032321	20	4	GAGGGAGTAGGTTGTATAGT	miRNA	miR-1827
t0032350	23	4	CAAAGTGCTTACAGTGCAGGTCG	miRNA	miR-17a
t0032501	20	4	TCGGGGCGTCGGCGGCGGCG	miRNA	miR-1777a
t0031734	19	4	TCGGGGCGGCGGCGGCGGT	miRNA	miR-1777a
t0031888	22	4	CAAAGTGCTTACAGTGCCGGTA	miRNA	miR-17
t0031955	23	4	CAAAGTGCTTACAGTGCCGGTAG	miRNA	miR-17
t0032018	22	4	AGCACGTAAATATTGGCTAGCA	miRNA	miR-16b
t0032199	25	4	GGAAGCGGCGATGGCGGGGCTGAAA	miRNA	miR-1636

t0032326	22	4	TAGTAGCACGTAAATATTGGAG	miRNA	miR-16
t0032610	23	4	TAGCAGCACGTAAATATCGGCGA	miRNA	miR-16
t0032671	22	4	TAGCAGAATGTAAATATTGGCG	miRNA	miR-16
t0032704	19	4	TAGCAGCAAGTAAATATTG	miRNA	miR-16
t0032715	23	4	TAGCAGCACGTAAATATTAGCGT	miRNA	miR-16
t0032864	20	4	TAGCAGCACGTAAATATGGG	miRNA	miR-16
t0032597	23	4	TAACAGCACGTAAATATTGGCGA	miRNA	miR-16
t0032198	22	4	TAGAAGCACGTAAATATTGCCG	miRNA	miR-16
t0032343	23	4	TAGCAGCACGGAAATATTGGCGA	miRNA	miR-16
t0031787	23	4	TAGCAGCGCGTAAATATTGGCGA	miRNA	miR-16
t0032375	23	4	CAGCAGCACGTAAATATTGGCGT	miRNA	miR-16
t0032040	20	4	TAGCAGCACGTAAATAATGG	miRNA	miR-16
t0032814	21	4	AGCAGCACGTAAATATTGGAG	miRNA	miR-16
t0031707	21	4	AGAAGCACGTAAATATTGGCG	miRNA	miR-16
t0031725	20	4	AGCAGCACGTAAATATTGGC	miRNA	miR-16
t0031741	19	4	TAGCGGCACGTAAATATTG	miRNA	miR-16
t0031804	23	4	CAGCAGCACGTAAATATTGGCGA	miRNA	miR-16
t0031893	22	4	TAGCAGAACGTAAATATTAGCG	miRNA	miR-16
t0031967	20	4	TAGCAGCACGTAAATGTTGG	miRNA	miR-16
t0032072	22	4	TAGTAGTACGTAAATATTGGCG	miRNA	miR-16
t0032182	20	4	TAGCAGCACGTAAATATAGG	miRNA	miR-16
t0032290	20	4	TAGCAGCAAATCATGGTTTT	miRNA	miR-15c
t0032291	19	4	TAGCAGCACTTCATGGTTT	miRNA	miR-15b
t0032336	19	4	TAGCAGAAGATCATGGTTT	miRNA	miR-15b
t0032346	20	4	TCGCAGCACATCATGGTTTA	miRNA	miR-15b
t0032411	19	4	TAGCAGAAAATCATGGTTT	miRNA	miR-15b
t0032440	22	4	TAGGAGCACATCATGGTTTACA	miRNA	miR-15b
t0032496	18	4	TAGCAGCACATCCTGGTT	miRNA	miR-15b
t0032508	20	4	GCAGCACATCATGGTTTATA	miRNA	miR-15b
t0032570	19	4	TAGCAGCACAACATGGTTT	miRNA	miR-15b
t0032691	22	4	TCGCAGCACATCATGGTTTACA	miRNA	miR-15b
t0032714	22	4	TAGCCGCACATCATGGTTTACA	miRNA	miR-15b
t0032833	20	4	TAGCAGCACAGCATGGTTTA	miRNA	miR-15b
t0032892	25	4	TAGCAGCACATCATGGTTTAAAAAA	miRNA	miR-15b
t0032062	21	4	TAGCAGCACGTCATGGTTTAC	miRNA	miR-15b
t0031810	22	4	TAGCAGCACATCAAGGTTTACA	miRNA	miR-15b
t0031907	19	4	TAGCAGCACATCATGGTTC	miRNA	miR-15b
t0031919	19	4	TAGCTGCACATCATGGTTT	miRNA	miR-15b
t0031950	21	4	TAGCAGCACTTAATGGTTTGT	miRNA	miR-15a
t0032029	20	4	TAGCAGCACATTATGGTTTG	miRNA	miR-15a
t0032126	21	4	TAGCAGCACATAAAGGTTTGT	miRNA	miR-15a
t0032328	21	4	TAGCATCACATAATGGTTTGT	miRNA	miR-15a
t0032363	19	4	TAGCGGCACATAATGGTTT	miRNA	miR-15a
t0032433	35	4	TCTCCCAACCCTTGTACCAGTGTTTTTGTT.	miRNA	miR-150
t0032436	25	4	TCAGTGCATGACAGAACTTGGTATC	miRNA	miR-148b-3p
t0032528	19	4	TCAGTGCACTACAGAACTG	miRNA	miR-148a
t0032530	23	4	GGATATCATCATATACTGTACGT	miRNA	miR-144*
t0032641	22	4	GGATATCATCATATACTGTACG	miRNA	miR-144*
t0032683	21	4	TACAGTATAGATGATGTAATT	miRNA	miR-144
t0032722	20	4	TACAGTATAGATGATGGACT	miRNA	miR-144
t0032867	23	4	TGAGATGAAGCACTGCAGCTATC	miRNA	miR-143
t0031762	20	4	CCCATAAAATAGAAAGCACT	miRNA	miR-142
t0032164	20	4	CCCATAAAGCAGAAAGCACT	miRNA	miR-142
t0032222	24	4	TACCACAGGGTAGAACCACGGGCG	miRNA	miR-140
t0032513	20	4	CACAGGGTAGAACCACGGAT	miRNA	miR-140
t0032591	23	4	TACCACAGGGTACAACCACGGAA	miRNA	miR-140
t0032048	23	4	TACCACAGCGTAGAACCACGGAA	miRNA	miR-140
t0032333	25	4	TACCACAGGGTAGAACCCCGGACAA	miRNA	miR-140

t0032969	23	4	TACCACAGGATAGAACCACGGAT	miRNA	miR-140
t0032134	23	4	TACCACCGGGTAGAACCACGGAT	miRNA	miR-140
t0032274	24	4	ACCACAGGGTAGAACCACGGCCAA	miRNA	miR-140
t0031775	23	4	TACCACAGGGTAGAACGACGGAT	miRNA	miR-140
t0031887	23	4	ACCACAGGGTAGAACCAAGGAAA	miRNA	miR-140
t0032889	24	4	TACCACAGGGTAGAACAACGGAAA	miRNA	miR-140
t0032702	23	4	ACCACAGGGAAGAACCACGGAAG	miRNA	miR-140
t0032873	24	4	TACCACAGGGTAGAACGACGGACA	miRNA	miR-140
t0031675	21	4	ACAACAGGGTAGAACAACGGA	miRNA	miR-140
t0031689	23	4	ACCACAGGGGAGAACCACGGACG	miRNA	miR-140
t0031723	23	4	TACCACGGGGTAGAACCACGGAT	miRNA	miR-140
t0031752	22	4	ACCACAGGATAGAACCACGGAA	miRNA	miR-140
t0031780	24	4	TACCACAAGGTAGAACCACGGACG	miRNA	miR-140
t0031794	23	4	TACCACAGGGTCGAACCACGGAA	miRNA	miR-140
t0031822	23	4	TACCACAGGGTAGAACCACGCAC	miRNA	miR-140
t0031877	22	4	ACGACAGGGTAGAACCACGGAC	miRNA	miR-140
t0031890	24	4	TACCACAGGGTAGAACCACGTACA	miRNA	miR-140
t0031931	21	4	ACCACAGGGTAGAACAAAGGA	miRNA	miR-140
t0031958	23	4	ACCACAGGGTAGAACCACGGGCG	miRNA	miR-140
t0031984	21	4	ACCACAGGGTAGAACCCCGGC	miRNA	miR-140
t0032032	22	4	TACACAGGGTAGAACCACGGAT	miRNA	miR-140
t0032036	23	4	ACCACAGGGTAAAACCACGGACA	miRNA	miR-140
t0032149	23	4	ACCACAGGGTAGAACCGCGGACA	miRNA	miR-140
t0032175	21	4	ACCACAGTGTAGAACAACGGA	miRNA	miR-140
t0032177	23	4	TACAACAGGGTAGAACCAAGGAA	miRNA	miR-140
t0032188	23	4	TACCACAGGGTAGAACCTCGGAA	miRNA	miR-140
t0032189	21	4	TACCACGGGGTAGAACCACGG	miRNA	miR-140
t0032203	22	4	ACCAAAGGGTAGAACCACGGAT	miRNA	miR-140
t0032245	22	4	ACCACAGGGTAGAATCACGGTA	miRNA	miR-140
t0032254	23	4	TACCACAGGGTAGAACCAGGGAT	miRNA	miR-140
t0032358	22	4	ACCACCGGGTAGAACCACGGAC	miRNA	miR-140
t0032401	23	4	TACCACAGGCTAGAACCACGGAT	miRNA	miR-140
t0032458	23	4	TACCACAGGGTAGTACCACGGAA	miRNA	miR-140
t0032503	22	4	CCACAGGGTAGAACCACGGACC	miRNA	miR-140
t0032546	23	4	ACCACAGGGTAGAACCACGGCTA	miRNA	miR-140
t0032602	22	4	ACCACAGGGTAGAATCCCGGAC	miRNA	miR-140
t0032644	23	4	ACCACAAGGTAGAACCACGGACA	miRNA	miR-140
t0032648	22	4	ACCACAGGGTAGAACCAGGGAC	miRNA	miR-140
t0032653	20	4	CCACAGGGTAGAACAACGGA	miRNA	miR-140
t0032716	23	4	GACCACAGGGTAGAACCACGGAA	miRNA	miR-140
t0032733	26	4	TGGAATGTAAAGAAGTATGTATTATC	miRNA	miR-1-3p
t0032762	22	4	CAGTGCAATGATGAAAGGGAAT	miRNA	miR-130b
t0032811	21	4	CAGTGCAATGTTAAAAGGGCC	miRNA	miR-130a
t0032815	21	4	GTGAGGACTCGGGAGGTGGAT	miRNA	miR-1224
t0032832	23	4	AGCAGCATTGTACAGGGCTTTGA	miRNA	miR-107b
t0032869	23	4	AGCAGCATTGTACAGGGCTATCA	miRNA	miR-107
t0032882	22	4	AGCAGCATTGGACAGGGCTATT	miRNA	miR-107
t0032901	20	4	TAAAGTGCTGACAGCGCAGA	miRNA	miR-106b
t0032904	21	4	TAAAGTGCTAACAGTGCAGAT	miRNA	miR-106b
t0032905	20	4	CAAAGTGCTGACAGTGCAGA	miRNA	miR-106b
t0032946	21	4	TAAAGTGCTGACAGTGAAGAT	miRNA	miR-106b
t0032971	20	4	TAAAGTGCTAACAGTGCAGA	miRNA	miR-106b
t0032986	21	4	TAAAGTGCTGACAGTGCCGAA	miRNA	miR-106b
t0032541	21	4	TAAAGTCCTGACAGTGCAGAT	miRNA	miR-106b
t0031713	22	4	TAAAGTGCTGACAGGGCAGATA	miRNA	miR-106b
t0032492	21	4	TAAAGTGCTGACAGTGCGGAT	miRNA	miR-106b
t0032491	21	4	AAAAGTGCTTACAGGGCAGGT	miRNA	miR-106
t0032146	23	4	AAAAGTGCTTACAGTGCAGGGAG	miRNA	miR-106

t0031800	23	4	AAAAGTGCTTACAGTGCAGGAAG	miRNA	miR-106
t0032574	22	4	AGCAGCATTGTACAGGGCGATG	miRNA	miR-103a
t0031743	21	4	AGCAGCTTTGTACAGGGCTAT	miRNA	miR-103a
t0032019	22	4	TGCAGCATTGTACAGGGCTATG	miRNA	miR-103a
t0032065	20	4	AGCACCATTGTACAGGGCTA	miRNA	miR-103a
t0032073	22	4	ATCAGCATTGTACAGGGCTATG	miRNA	miR-103a
t0032206	23	4	AGTAGCATTGTACAGGGCTATGT	miRNA	miR-103a
t0032723	22	4	AGCAGCATTGTACAGGCCTATG	miRNA	miR-103a
t0032724	22	4	AGCAGCAATGTACAGGGCTATG	miRNA	miR-103a
t0032798	22	4	AGCATCATTGTACAGGGCTATG	miRNA	miR-103a
t0032870	24	4	AGAAGCATTGTACAGGGCTATGAT	miRNA	miR-103a
t0032395	23	4	GTACAGTACTGTGATAACTGACA	miRNA	miR-101c
t0032657	20	4	AACAGTACTGTGATAACTGA	miRNA	miR-101c
t0032665	22	4	TACAGTACTATGATAACTGAAG	miRNA	miR-101b
t0032003	22	4	TGCAGTACTGTGATAACTGAAG	miRNA	miR-101
t0032144	22	4	GTACAGTACAGTGATAACTGAA	miRNA	miR-101
t0032276	21	4	GTACAGTACTGTGATCACTGA	miRNA	miR-101
t0032279	21	4	GTACAGTGCTGTGATAACTGA	miRNA	miR-101
t0032466	20	4	TACAGTCCTGTGATAACTGA	miRNA	miR-101
t0032484	22	4	TACAGTACTGTGATAAATGAAA	miRNA	miR-101
t0032485	22	4	TAGAGTACTGTGATAACTGAAG	miRNA	miR-101
t0032523	22	4	TACAGTACTGGGATAACTGAAG	miRNA	miR-101
t0032524	29	4	TACAGTACTGTGATAACTGAAGCGTGCGT	miRNA	miR-101
t0032533	21	4	TACAGTACTGTTATAACTGAA	miRNA	miR-101
t0032195	22	4	TACAGCACTGTGATAACTGAAG	miRNA	miR-101
t0032310	22	4	GTACAGTAGTGTGATAACTGAA	miRNA	miR-101
t0032184	21	4	TACAGTACTGTGATAACGGAA	miRNA	miR-101
t0031702	20	4	TACAGTACTGTGGTAACTGA	miRNA	miR-101
t0031720	20	4	TACAGTACCGTGATAACTGA	miRNA	miR-101
t0031732	20	4	TGCAGTACTGTGATAACTGA	miRNA	miR-101
t0031785	21	4	TTCAGTACTGTGATAACTGAA	miRNA	miR-101
t0031971	22	4	GTACAGTACTGTGATAACTTAA	miRNA	miR-101
t0032059	20	4	TACAGTACTGTGACAACTGA	miRNA	miR-101
t0032085	21	4	TACAGTACTGTCATAACTGAA	miRNA	miR-101
t0032086	21	4	GTACAGTACTGTGATAATTGA	miRNA	miR-101
t0032122	22	4	TACAGTAGTGTGATAACTGAAG	miRNA	miR-101
t0032167	21	4	GTACAGTACTGTGATAACTGG	miRNA	miR-101
t0032168	21	4	TACAGTATCGTGATAACTGAA	miRNA	miR-101
t0032221	22	4	GTACAATACTGTGATAACTGAA	miRNA	miR-101
t0032278	21	4	GTGCAGTACTGTGATAACTGA	miRNA	miR-101
t0032287	20	4	TACAGTACTGTAATAACTGA	miRNA	miR-101
t0032341	20	4	TACAGTACTGTGATAACTGG	miRNA	miR-101
t0032361	21	4	TACACTACTGTGATAACTGAA	miRNA	miR-101
t0032540	24	4	ACCCTGTAGATCCGAATTTGTATC	miRNA	miR-10
t0032550	24	4	TACCCTGTAGATCCGAATTTGATC	miRNA	miR-10
t0032631	20	4	TGAGGTAGTAGTTTGTGATG	miRNA	let-7i
t0032651	21	4	TGAGGTAGAAGTTTGTGCTGT	miRNA	let-7i
t0032662	20	4	TAAGGTAGTAGTTTGTGCTG	miRNA	let-7i
t0032709	20	4	TGAGGTAGAAGTTTGTGCTG	miRNA	let-7i
t0032729	21	4	TGAGGTAGTAGTTTGTGGTGT	miRNA	let-7i
t0032755	20	4	TGAGGTAGTAGTTCGTGCTG	miRNA	let-7i
t0032804	23	4	TGAGGAAGTAGTTTGTGCTGTTT	miRNA	let-7i
t0032845	20	4	TGAGGCAGTAGTTTGTGCTG	miRNA	let-7i
t0032875	22	4	TGAGGTTGTAGTTTGTGCTGTT	miRNA	let-7i
t0032877	20	4	TGAGGTAGCAGTTTGTGCTG	miRNA	let-7i
t0032899	21	4	TGAGGTAGTAGTTTGCGCTGT	miRNA	let-7i
t0032202	22	4	TGAGCTAGTAGTTTGTGCTGTT	miRNA	let-7i
t0032596	19	4	TGAGGTAGTAGTTTGTGAT	miRNA	let-7i

t0031739	20	4 TGAGGTAGTAGTTTGTGCAG	miRNA	let-7g
t0031754	21	4 TGAGGTAGTAGTCTGTACAGT	miRNA	let-7g
t0031795	23	4 TGAGGTAGTAGTTTGTGCAGTTA	miRNA	let-7g
t0031808	21	4 TGAGGTAGCAGTTTGTACAGT	miRNA	let-7g
t0032174	22	4 TGAGGTAGTAGATTGTATATTG	miRNA	let-7f-5p
t0032219	23	4 TGAGGTAGTGGATTGTATAGTTT	miRNA	let-7f-5p
t0032317	22	4 TGAGGTAGTAGTTTGTATTGTT	miRNA	let-7f
t0032394	23	4 TGAGGTACTAGATTGTATAGTTG	miRNA	let-7f
t0032396	20	4 TGAGGTAGTAGATCGTATAT	miRNA	let-7f
t0032553	23	4 TGAGGTAGTAGATTGGATAGTTA	miRNA	let-7f
t0032645	22	4 TGAGGTAGTAGATTTTATATTT	miRNA	let-7f
t0032693	22	4 TGAGGTAGTAGATTGTTTGGTT	miRNA	let-7f
t0032998	23	4 TGAGGTAGTAAATTGTATAGTTA	miRNA	let-7f
t0031849	23	4 TGAGGTTGTAGATTGTATAGTTA	miRNA	let-7f
t0031991	22	4 TGAGGTAGTAGCTTGTATCGTT	miRNA	let-7f
t0032464	23	4 TGAGGTAGTAGATTGCATAGTTT	miRNA	let-7f
t0032667	20	4 TGAGGTAGTAGATGGTATAG	miRNA	let-7f
t0031823	23	4 TGAGGTAGTAGATTGTGTAGTTT	miRNA	let-7f
t0031838	21	4 GAGGCAGTAGATTGTATAGTT	miRNA	let-7f
t0031719	23	4 TGGGGTAGTAGATTGTATAGTTT	miRNA	let-7f
t0031799	26	4 TGAGGTAGTAGATTGTATAGTTAAAA	miRNA	let-7f
t0031848	22	4 TGCGGTAGTAGCTTGTATAGTT	miRNA	let-7f
t0031916	23	4 TGAGGTAGTAGTTTGTATAGTTA	miRNA	let-7f
t0031935	22	4 TGAGATAGTAGATTGTATAGTA	miRNA	let-7f
t0031952	25	4 TGAGGTAGTAGATTGTATAGTTAAT	miRNA	let-7f
t0031953	23	4 TGAAGTAGTAGATTGTATAGTTA	miRNA	let-7f
t0031974	23	4 TGAGGTCGTAGATTGTATAGTTG	miRNA	let-7f
t0032095	23	4 TGAGGTAGTAGATTGTAAAGTTG	miRNA	let-7f
t0032120	23	4 TGAGGTAATAGATTGTATAGTTA	miRNA	let-7f
t0032129	23	4 TGAGGTAGTAGATTGTATAGTCT	miRNA	let-7f
t0032192	22	4 TGAGGTAGTAGATTGCATAGTA	miRNA	let-7f
t0032215	23	4 TGAGGTAGTAGATTGTATAGTCG	miRNA	let-7f
t0032226	20	4 AGGTAGTAGATTGTATATTT	miRNA	let-7f
t0032250	19	4 TGAGGTAGTAAATTGTATA	miRNA	let-7f
t0032300	23	4 TGAGGTAGTTGATTGTATAGTTA	miRNA	let-7f
t0032338	21	4 GAGGTAGCAGATTGTATAGTT	miRNA	let-7f
t0032339	19	4 TGAGGTAGTAGATTGGATA	miRNA	let-7f
t0032360	21	4 GAGGTAGTAGATTGTATAGGT	miRNA	let-7f
t0032399	22	4 TGGGGAAGTAGATTGTATAGTT	miRNA	let-7f
t0032408	23	4 TGAGGTAGAAGATTGTATAGTTT	miRNA	let-7f
t0032418	22	4 TGAGGTAGGAGATTGTGTAGTT	miRNA	let-7e
t0032422	22	4 TGAGGTAGGAGATTGTATAGGT	miRNA	let-7e
t0032613	23	4 TGAGGTAGGAGATTGTATAGTTT	miRNA	let-7e
t0032616	21	4 AGAGGTAGTCGGTTGCATAGT	miRNA	let-7d
t0032688	21	4 AGAGATAGTAGGTTGCATAGT	miRNA	let-7d
t0032750	20	4 AGAGGTAATAGGTTGCATAG	miRNA	let-7d
t0032827	22	4 AGAGGTAGTAGGTTGCATTGTT	miRNA	let-7d
t0032831	20	4 AGAGGTAGAAGGTTGCATAG	miRNA	let-7d
t0032878	20	4 AGAGTTAGTAGGTTGCATAG	miRNA	let-7d
t0032902	22	4 AGAGGTAGTAGGTAGCATAGTT	miRNA	let-7d
t0032930	21	4 AAAGGTAGTAGGTTGCATAGT	miRNA	let-7d
t0032953	21	4 AGCGGTAGTAGGTTGCATAGT	miRNA	let-7d
t0033007	20	4 AGAGGTAGTAGGTTCCATAG	miRNA	let-7d
t0031843	22	4 TGAGGTAGTAGTTTGTATGGTT	miRNA	let-7c
t0032109	20	4 TGAGGTCGTAGGTTGTGTGG	miRNA	let-7b
t0032429	23	4 TGAGGTAGGAGGTTGTGTGGTAA	miRNA	let-7b
t0031724	21	4 TGAGGTAGTAGGTAGTGTGGT	miRNA	let-7b
t0031771	23	4 TGAGGTAGTAGGTTGTGTGGCTA	miRNA	let-7b

t0031975	23	4	TGAGGTAGTAGGATGTGTGGTTT	miRNA	let-7b
t0032058	24	4	TGAGGTAGCAGGTTGTGTGGTTAA	miRNA	let-7b
t0032107	23	4	TGAGGTAGTAGGTTATGTGGTTA	miRNA	let-7b
t0032152	19	4	TGAGGTAGTAGGTCGTGTG	miRNA	let-7b
t0032465	23	4	TGAGGTAGTAGGCTGTGTGGTAA	miRNA	let-7b
t0032564	23	4	TGAGGTAGTAGGTTGTGTGGATT	miRNA	let-7b
t0032760	23	4	TGCGGTAGTAGGTTGTGTGGTTG	miRNA	let-7b
t0033002	23	4	TGAGGTAGTAGGTTGTGTGGTCG	miRNA	let-7b
t0032113	22	4	TGAGGTAGAAGGTTGTGTGGTA	miRNA	let-7b
t0031709	22	4	TGAGGTAGTAGGTTGAGTGGTA	miRNA	let-7b
t0031789	24	4	TGAGGTGGTAGGTTGTGTGGTTAA	miRNA	let-7b
t0031841	20	4	TGAGGTAGTAGGTTGAGTGG	miRNA	let-7b
t0031867	20	4	TGAAGTAGTAGGTTGTGTGG	miRNA	let-7b
t0031895	20	4	TGAGGTAATAGGTTGTGTGG	miRNA	let-7b
t0031906	22	4	TGAGGAAGTAGGTTGTGGGGTT	miRNA	let-7b
t0031941	23	4	TAAGGTAGTAGGTTGTGTGGTTA	miRNA	let-7b
t0031981	23	4	TGAGGTAGTAGGTTGCGTGGTTT	miRNA	let-7b
t0032076	24	4	TGAGGTAGTGGGTTGTGTGGTTAA	miRNA	let-7b
t0032079	23	4	TGAGGTAGTTGGTTGTGTGGTTT	miRNA	let-7b
t0032108	20	4	TGAGATAGTAGGTTGTGTGG	miRNA	let-7b
t0032112	19	4	CGAGGTAGTAGGTTGTGTG	miRNA	let-7b
t0032158	22	4	AGAGGTAGTAGGTTGTGTGGTA	miRNA	let-7b
t0032163	24	4	TGAGGTCGTAGGTTGTGTGGTTAA	miRNA	let-7b
t0032191	23	4	TGAGGTAGTTGGTTGTGTGGTTA	miRNA	let-7b
t0032201	24	4	TGAGGCAGTAGGTTGTGTGGTTAA	miRNA	let-7b
t0032234	23	4	TGGGGTAGTAGGTTGTGTGGTTT	miRNA	let-7b
t0032238	20	4	TGAGGTAGTAGGTTGTGAGG	miRNA	let-7b
t0032256	23	4	TGAGGTAGTAGGTTGTGTGGCAA	miRNA	let-7b
t0032258	24	4	TGAGGCAGTAGGTTGTGTGGTTAT	miRNA	let-7b
t0032280	22	4	TGAGGTAGTAGGTTGGGTGGTA	miRNA	let-7a
t0032368	24	4	TGGGGTAGTAGGTTGTATGGTATC	miRNA	let-7-5p
t0032423	20	4	TGAGGTAGTATGTTGTATAG	miRNA	let-7
t0032534	21	4	TGAGGTAGTAGGTAGTATAGT	miRNA	let-7
t0032535	23	4	TGAGGTAGTAGGGTGTATAGTTG	miRNA	let-7
t0032555	19	4	TGAGGTGGTAGGTTGTATA	miRNA	let-7
t0032568	23	4	TGAGGTAGTAGGGTGTATAGTTA	miRNA	let-7
t0032581	20	4	TGACGTAGTAGGTTGTATAG	miRNA	let-7
t0032583	19	4	TGAGGTAGCAGGTTGTATA	miRNA	let-7
t0032699	20	4	TCAGGTAGTAGGTTGTATAG	miRNA	let-7
t0032737	22	3	CACCCGGCTGTGTGCACCTGTG	miRNA	miR-941
t0032829	21	3	CAAAGTGCTGTTCATGCAGGT	miRNA	miR-93
t0032944	22	3	CAAAGTGCTGTTCGCGCAGGTA	miRNA	miR-93
t0032457	23	3	CAAAGTGCTGTTCGTGAAGGTAG	miRNA	miR-93
t0032145	22	3	AGGGACGGGACGCGGTGCAGGG	miRNA	miR-92b*
t0031697	22	3	AGGGACGGGACGCGGTGAAGTG	miRNA	miR-92b*
t0031821	22	3	CATTGCACTTGTCCCGGCCTGT	miRNA	miR-92a-3p
t0031951	21	3	TATTGCACTTGCCCCGGCCTG	miRNA	miR-92a
t0031969	21	3	TATTGCACTTGTCCCGGTCTG	miRNA	miR-92a
t0032223	22	3	TATTGCACTTGTCCCGGTCTGT	miRNA	miR-92a
t0032474	23	3	TATTGCACTTGTCCCGGACTGTT	miRNA	miR-92a
t0032544	21	3	TATTGCACTTGTCCCGGGCTG	miRNA	miR-92a
t0032865	20	3	TATTGCACTTGTCACGGCCT	miRNA	miR-92a
t0034947	20	3	TATTGCACTTGTACCGGCCT	miRNA	miR-92a
t0034006	22	3	TATTACACTTGTCCCGGCCTGT	miRNA	miR-92a
t0034017	22	3	TATTGCACTTATCCCGGCCTGT	miRNA	miR-92a
t0034747	22	3	TATTGCACTTGTTCCGGCCTGT	miRNA	miR-92a
t0033055	22	3	TGTTGCACTTGTCCCGGCCTGT	miRNA	miR-92a
t0034049	21	3	TATTACACTTGTCCCGGCCTG	miRNA	miR-92a

t0035022	20	3	TATTGCAATTGTCCCGGCCT	miRNA	miR-92a
t0033032	22	3	TATTGCACTTGTCCCGGGCTGT	miRNA	miR-92a
t0033226	21	3	TATTGCACTTGTCCCGACCTG	miRNA	miR-92a
t0033246	18	3	TATTGCACTCGTCCCGGC	miRNA	miR-92
t0033405	21	3	TGGAAGACTAGTGATTTTTTT	miRNA	miR-7b
t0033588	23	3	CGAAGACTGAAGTGGAGAAGGGT	miRNA	miR-739
t0034073	23	3	TGAAGAATGAAGTGGAGAAGGGT	miRNA	miR-739
t0034131	23	3	TGAAAGACTTGAGTAGTGAGACG	miRNA	miR-71b-5p
t0034166	24	3	AAAGACTTGAGTAGTGAGACGTTT	miRNA	miR-71b-5p
t0034235	22	3	TACCCATTGCATATCGGCGTTG	miRNA	miR-660
t0034245	18	3	TGAGAACCACGTCTGCTC	miRNA	miR-589
t0034324	22	3	AAACGGCAATGACTTTTGTACA	miRNA	miR-548al
t0034603	23	3	CATGCCTTGAGTGTAGGCCCGTT	miRNA	miR-532-5p
t0034808	22	3	CTTCCCACACCCAAGGCTTGCC	miRNA	miR-532-3p
t0034845	22	3	CCTCCCACACCCAAGGCTTGTA	miRNA	miR-532-3p
t0035167	22	3	CCTCCCACAACCAAGGCTTGCA	miRNA	miR-532-3p
t0033390	22	3	CCTCCTACACCCAAGGCTTGCA	miRNA	miR-532-3p
t0034605	23	3	TCTGGGCACAGGCGGATGGACCG	miRNA	miR-5107
t0034353	22	3	TCTGGGAACAGGCGGATGGACA	miRNA	miR-5107
t0035014	22	3	TCTGGGCACAGGCGGATGGCCA	miRNA	miR-5107
t0034339	20	3	TAGCAGCGGGGACAGTTCTG	miRNA	miR-503
t0034848	21	3	TTAAGACTTGCAGGGATGTTT	miRNA	miR-499-5p
t0034283	28	3	TCCTGTACTGAGCTGCCCCGAGATGGCG	miRNA	miR-486-5p
t0034811	21	3	CTGTACTGAGCTGCCCCGAGT	miRNA	miR-486-5p
t0033607	23	3	TCCTGTACTGAGCTGCCCCGGGT	miRNA	miR-486-5p
t0034470	22	3	TACTGTACTGAGATGCCCCGAG	miRNA	miR-486-5p
t0033739	23	3	TCCTGTACTGAGCTGCACCGAGT	miRNA	miR-486-5p
t0033902	21	3	TGCTGTACTGAGCTGCCCCGA	miRNA	miR-486-5p
t0034726	20	3	TCATGTACTGAGCTGCCCCG	miRNA	miR-486-5p
t0034846	23	3	TCCTGAACTGAGCTGCCCCGAGA	miRNA	miR-486-5p
t0034763	22	3	TCCTGTACTGAGCTGCCAAGAG	miRNA	miR-486-5p
t0035012	20	3	TCCTGTACTGAGCTGCCCCA	miRNA	miR-486-5p
t0035057	20	3	TCCTGTACTGAACTGCCCCG	miRNA	miR-486-5p
t0034012	21	3	TCCTGTATCGAGCTGCCCCGA	miRNA	miR-486-5p
t0034078	23	3	TCATGTACTGAGCTGCCCCGAGT	miRNA	miR-486-5p
t0033023	21	3	TCCTGTACTGAGCTGATCCGA	miRNA	miR-486-5p
t0033372	25	3	TCCTGTACTGAGCTGCCCCGCCGGT	miRNA	miR-486-5p
t0033375	24	3	TCCTGTACTGAGCTGCCCCGCGTT	miRNA	miR-486-5p
t0033432	22	3	TCCTGTACTGAGCTGACCCGAA	miRNA	miR-486-5p
t0033651	23	3	TCCTGCACTGAGCTGCCCCGAGT	miRNA	miR-486-5p
t0033689	22	3	TCCTGTACTGAGATGCACCGAG	miRNA	miR-486-5p
t0033753	22	3	TCATGTACTGAGCTGCACCGAG	miRNA	miR-486-5p
t0033792	28	3	TCCTGTACTGAGCTCCCCCGAGACGGCA	miRNA	miR-486-5p
t0034081	23	3	TCCTATACTGAGCTGCCCCGAGT	miRNA	miR-486-5p
t0034158	23	3	TACTGTACTGAGCTGCCCCGAGT	miRNA	miR-486-5p
t0034196	24	3	TCCTGTACTGAGCTGCCCCGCGAA	miRNA	miR-486-5p
t0034209	22	3	TCCTGTACTGAGCTACCCCGAA	miRNA	miR-486-5p
t0034244	24	3	TCCTGTACTGAGCTGCCCCGCCGG	miRNA	miR-486-5p
t0034255	23	3	TGGAGAGCAGGGAGCAGGAAGCT	miRNA	miR-4732-5p
t0034278	23	3	TCTAGAGCAGGGAGCAGGAAGCT	miRNA	miR-4732-5p
t0034295	23	3	TGTAGAGCAGGGAGCAGGCAGCT	miRNA	miR-4732-5p
t0034317	20	3	TGTAGAGCAGGGGGCAGGAA	miRNA	miR-4732-5p
t0034398	23	3	TGTAGAGCGGGGAGCAGGAAGCT	miRNA	miR-4732-5p
t0034503	23	3	TGTAGAGCAGGGAGCAGGACGCT	miRNA	miR-4732-5p
t0034651	23	3	TGTGGAGCAGGGAGCAGGAAGCT	miRNA	miR-4732-5p
t0034692	21	3	TGTAGAGAAGGGAGCAGGAAG	miRNA	miR-4732-5p
t0034750	23	3	TGCAGAGCAGGGAGCAGGAAGCT	miRNA	miR-4732-5p
t0034891	22	3	TGTGTCCGGGAAGTGGAGGAGG	miRNA	miR-4669

t0034893	22	3	ACTGGGAAGAGGAGCTGAGGGC	miRNA	miR-4646-5p
t0035013	21	3	GAAGGGTCGGGGCGGCAGGGG	miRNA	miR-4516
t0035042	22	3	TGAGGGAGTAGGTTGTTTGGTT	miRNA	miR-4510
t0033152	22	3	TGAGGCAGTAGGGTGTGTGGTT	miRNA	miR-4510
t0033559	22	3	TGAGGGATTAGGTTGTGTGGTT	miRNA	miR-4510
t0034027	24	3	TGAGGGAGTAGGTTGTGTGGTTGA	miRNA	miR-4510
t0034040	21	3	TGAGGGAGTAGGATGTGTGGT	miRNA	miR-4510
t0034144	26	3	AAACCGTTACCATTCCTGAGTTTAGT	miRNA	miR-451
t0034430	20	3	AAACCGTAACCATTACTGAG	miRNA	miR-451
t0034616	23	3	AAACCGTTACCATTACTGAATTT	miRNA	miR-451
t0034966	24	3	AAACCGTTACCATTACTGAGATTA	miRNA	miR-451
t0034968	24	3	AAACCGTTACCATTAATGAGTTTA	miRNA	miR-451
t0034124	25	3	AAACCGTTACCATTACTCAGTTTAG	miRNA	miR-451
t0034741	19	3	AAACCGTTACCATTACTGG	miRNA	miR-451
t0034565	22	3	AAACCTTTACCGTTACTGAGTT	miRNA	miR-451
t0034240	21	3	AATCCGTTACCATTACTGAGT	miRNA	miR-451
t0034258	19	3	AAACCATTACCATTACTGA	miRNA	miR-451
t0034714	18	3	AAACCGTTACAATTACTG	miRNA	miR-451
t0034804	26	3	AAACCGTTAACATTACTGAGTTTAGT	miRNA	miR-451
t0034911	26	3	AAACCGTTACCATTACTGAGGTTAGT	miRNA	miR-451
t0033048	19	3	GAACCGTTACCATTACTGA	miRNA	miR-451
t0033163	25	3	AAATCGTTACCATTACTGAGTTTAG	miRNA	miR-451
t0033184	23	3	AAACAGTTAACATTACTGAGTTT	miRNA	miR-451
t0033322	24	3	AAACCATTACCATTACTGAGTTTA	miRNA	miR-451
t0033334	22	3	AAACCTTTACCATTACTGAGCT	miRNA	miR-451
t0033346	21	3	AAACCTTTACCGTTACTGAGT	miRNA	miR-451
t0033361	19	3	AAACCGTTACCATTCCTGA	miRNA	miR-451
t0033389	19	3	AAACCGTTACCATCACTGA	miRNA	miR-451
t0033472	18	3	AAACCGTTAACATTACTG	miRNA	miR-451
t0033504	20	3	AAACCGTTACCATAACTGAG	miRNA	miR-451
t0033720	24	3	AAACCGTTACCGTTACTGAGTTTA	miRNA	miR-451
t0033754	24	3	AAACCGTAACCATTACTGAGTTTA	miRNA	miR-451
t0033788	24	3	AAACCGTTACTATTACTGAGTTTA	miRNA	miR-451
t0033826	24	3	AAACCGGTACCATTACTGAGTTTA	miRNA	miR-451
t0033974	20	3	AAACAGTTACCATTAATGAG	miRNA	miR-451
t0034021	23	3	AAACCGTAACCATTACTGAGTTT	miRNA	miR-451
t0034135	20	3	AAACCGTTTCCATTACTGAG	miRNA	miR-451
t0034179	23	3	AAACCGTTTCCATTACTGAGTTT	miRNA	miR-451
t0034230	22	3	AAACCTTTACCATTGCTGAGTT	miRNA	miR-451
t0034354	21	3	TTCGGGGTCTGGGCGCGGCGC	miRNA	miR-4508
t0034378	22	3	TGGAGAGAAAGGCAGTTTTTGC	miRNA	miR-4306
t0034541	20	3	TGCAGCATTGTACAGGGCTA	miRNA	miR-4289
t0034656	21	3	AGAAGCATTGTACAGGGCTAA	miRNA	miR-4289
t0034658	20	3	ACCAGCATTGTACAGGGCTA	miRNA	miR-4289
t0034679	19	3	CAGCAGCATTTCATGTTTT	miRNA	miR-424
t0034685	21	3	TGAGGGGCAGAGAGAGGGACT	miRNA	miR-423-5p
t0034729	24	3	TAAGGGGCAGAGAGCGAGACTTTT	miRNA	miR-423-5p
t0034740	21	3	TGAGGGGCAGAGAGCGCGCCT	miRNA	miR-423-5p
t0034749	22	3	TGAGGGGCAGAGAGAGAGACTA	miRNA	miR-423-5p
t0034912	24	3	TGAGGTGCAGAGAGCGAGACTTTT	miRNA	miR-423-5p
t0035116	22	3	TGATGGGCAGAGAGCGAGATTT	miRNA	miR-423-5p
t0035183	24	3	TGAGGGGCAGCGAGCGAGACTTTT	miRNA	miR-423-5p
t0034187	19	3	TGAGGGGTAGAGAGCGAGA	miRNA	miR-423-5p
t0034272	23	3	TGAGGGGCAGAGAGTGAGAATTT	miRNA	miR-423-5p
t0033151	19	3	TGAGGGGCGGAGAGCGAGA	miRNA	miR-423-5p
t0033800	21	3	TGAGGGGCAGAGAGCGAGCCC	miRNA	miR-423-5p
t0033898	23	3	TGAAGGGCAGAGAGCGAGACTTA	miRNA	miR-423-5p
t0035063	22	3	TGAGGGGCAGAGAGCGAGACGT	miRNA	miR-423-5p

t0033065	23	3	AGAGGGGCAGAGAGCGAGACTTA	miRNA	miR-423-5p
t0033182	22	3	TGAGGGGCAGAGAGCGAGCCTA	miRNA	miR-423-5p
t0033192	24	3	TGAGGGGCAGAGAGAGAGACTTTA	miRNA	miR-423-5p
t0033255	23	3	TGAGGGGCAGAGAGCGAGCCTTG	miRNA	miR-423-5p
t0033316	19	3	TGAGGGGCAGAGAGCGAGG	miRNA	miR-423-5p
t0033327	24	3	TGAGAGGCAGAGAGCGAGACTTTT	miRNA	miR-423-5p
t0033424	20	3	TGAGGGGCAGAGAGCGAGTC	miRNA	miR-423-5p
t0033586	19	3	TAAGGGGCAGAGAGCGAGA	miRNA	miR-423-5p
t0033589	20	3	CGAGGGGCAGAGAGCGAGAC	miRNA	miR-423-5p
t0033614	22	3	TGAGGGGCAGAGAGTGAGAATT	miRNA	miR-423-5p
t0033648	19	3	TGAGGGGCAGAGAGCGCGA	miRNA	miR-423-5p
t0033659	23	3	TGAGGGGCAAAGAGCGAGACTTA	miRNA	miR-423-5p
t0033660	24	3	TGAGGGGCAGAGCGCGAGACTTTA	miRNA	miR-423-5p
t0033785	24	3	TGAGGGGCACAGAGCGAGACTTTT	miRNA	miR-423-5p
t0033856	22	3	TGAGGGGCAGAGAGCGCGCCTT	miRNA	miR-423-5p
t0034120	20	3	TGAGGGGCGGAGAGCGAGAC	miRNA	miR-423-5p
t0034220	21	3	TGAGGGGCAGAGCGCGAGCCT	miRNA	miR-423-5p
t0034222	20	3	TGAGAGGCAGAGAGCGAGAC	miRNA	miR-423-5p
t0034242	23	3	TGAGGGGCAGAAAGCGAGACTTA	miRNA	miR-423-5p
t0034282	23	3	TGAGGGGCAGAGAGCGGGACTTG	miRNA	miR-423-5p
t0034355	20	3	TGAGGAGCAGAGAGCGAGAC	miRNA	miR-423-5p
t0034411	19	3	TGAGGGACAGAGAGCGAGA	miRNA	miR-423-5p
t0034418	21	3	TCAGGTAGTAGTTTGTACAGT	miRNA	miR-3962
t0034428	20	3	GGCGTCGGCGGCGGCGGGAC	miRNA	miR-3885-5p
t0034434	19	3	TGGCGGCGGCGGCGGGGGC	miRNA	miR-3885-5p
t0034563	21	3	GGGGCCGGCGGCGGCGGCGAC	miRNA	miR-3885-5p
t0034604	21	3	GCGGCGGCGGCGGGGGTGTGG	miRNA	miR-3885-5p
t0034735	28	3	CCGGCGGCGGCGGCGACTCTGGACGCGA	miRNA	miR-3885-5p
t0034757	22	3	TCGGGGCGGCGGCGGCGGTAGA	miRNA	miR-3885-5p
t0034849	23	3	TCGGGGCGGCGGCGGCGGCGGTG	miRNA	miR-3885-5p
t0034871	18	3	CGGCGGCGGCGGCGGGAC	miRNA	miR-3885-5p
t0034936	20	3	GGCGGCGGCGGGGGTGTGGG	miRNA	miR-3885-5p
t0034939	20	3	CGGCGTCGGCGGCGGCGGGA	miRNA	miR-3885-5p
t0035052	19	3	GGGCGGCGGCGGGGGGAGA	miRNA	miR-3885-5p
t0035085	23	3	ACTGGACTTGGAGTCAGAAGGTG	miRNA	miR-378f
t0034754	21	3	ACTGGAATTGGAGTCAGAAGG	miRNA	miR-378
t0033251	23	3	ACTGGACTTGGAGTCAGACGGCA	miRNA	miR-378
t0033336	23	3	ACTGGAATTGGAGTCAGAAGGCA	miRNA	miR-378
t0033443	23	3	ACTGGGCTTGGAGTCAGAAGGCA	miRNA	miR-378
t0033615	22	3	ACTGGACTTGGAGACAGAAGGC	miRNA	miR-378
t0033657	24	3	ACTGGACTTGGAGTCAGAAGGCTC	miRNA	miR-378
t0033916	23	3	ACTGGACTTTGAGTCAGAAGGCA	miRNA	miR-378
t0034146	22	3	CTGGACTTGGAGTCAGACGGCA	miRNA	miR-378
t0034375	22	3	ATATAATACAACCTGTTAAGTG	miRNA	miR-374
t0034460	22	3	ATATAATACAACCTGATAAGTG	miRNA	miR-374
t0035072	22	3	ATGTAATACAACCTGCTAAGTG	miRNA	miR-374
t0035086	22	3	ATTGCACGGTATCCATCTGTAC	miRNA	miR-363
t0035189	22	3	AATTGCACGGTATCCATCTGTC	miRNA	miR-363
t0033420	19	3	CGGGGGTGGGGTCGGCGGG	miRNA	miR-3621
t0033460	27	3	AACTTTGAAGAATGAAGTGGAGAAGGG	miRNA	miR-3526
t0033522	23	3	TGAAGACTGAAGTGAAGAAGGGT	miRNA	miR-3526
t0033969	28	3	AACTTTGAAGACTGAAGTGGAGAAGGGA	miRNA	miR-3526
t0034181	25	3	TGAAGACTGAAGTGGAGAAGGGCTT	miRNA	miR-3526
t0035009	24	3	TTGAAGACTGAAGTGGAGAAGGGC	miRNA	miR-3526
t0035040	28	3	AACTCTGAAGACTGAAGTGGAGAAGGGT	miRNA	miR-3526
t0035058	27	3	ACTTTGAAGACTGAAGGGGAGAAGGGT	miRNA	miR-3526
t0034100	25	3	TGAAGACCGAAGTGGAGAAGGGTTT	miRNA	miR-3526
t0034473	23	3	TGAAGACTGAAGTAGAGAAGGGT	miRNA	miR-3526

t0034695	23	3	TGAAGACTGAATTGGAGAAGGGT	miRNA	miR-3526
t0034111	23	3	TGAAGACTAAAGTGGAGAAGGGT	miRNA	miR-3526
t0034443	28	3	AACTTTGAAGACCGAAGTGGAGAAGGGT	miRNA	miR-3526
t0034319	25	3	TGAAGACTGAAGTGGAGCAGGGTTT	miRNA	miR-3526
t0033074	23	3	TGAAGACTGAGGTGGAGAAGGGT	miRNA	miR-3526
t0033108	27	3	AACTTTGAAGACTGAAGAGGAGAAGGG	miRNA	miR-3526
t0033117	22	3	CGCCCGGCGTCTGGACGTTTTT	miRNA	miR-3496
t0033259	21	3	AGAAGACTGCCGCTCGTTTTT	miRNA	miR-3494
t0033267	21	3	TCCGTGCTGAGATTTCGTCAT	miRNA	miR-3492
t0033363	22	3	TCCTCGAACTGTTGTGGCCATA	miRNA	miR-3487
t0033558	22	3	TCTCACACAGAAATCGCACCTG	miRNA	miR-342-3p
t0033932	22	3	TCTCACAAAGAAATCGCACCCG	miRNA	miR-342-3p
t0034033	22	3	TCTCACACAGAGATCGCACCCG	miRNA	miR-342-3p
t0034044	22	3	TCTCACGCAGAAATCGCACCCG	miRNA	miR-342-3p
t0034113	22	3	TCTCACACAGAAATCCCACCCG	miRNA	miR-342-3p
t0034356	22	3	TATCACACAGAAATCGCACCCG	miRNA	miR-342-3p
t0034433	24	3	TCTCACACAGAAATCGAACCCGTC	miRNA	miR-342-3p
t0034923	23	3	TCTCACACAGAAATTGCACCCGT	miRNA	miR-342-3p
t0035126	21	3	CTCACACAGAAATCGCACCCG	miRNA	miR-342-3p
t0034024	23	3	TCCCACACAGAAATCGCACCCGT	miRNA	miR-342-3p
t0033644	25	3	TCTCACACAGAAATCGCCCCCGTCT	miRNA	miR-342-3p
t0034261	22	3	TCTCACACAGAAATTGCACCCG	miRNA	miR-342-3p
t0034463	24	3	TCTCACACAGAAATAGCACCCGTC	miRNA	miR-342-3p
t0033525	24	3	TCTCACACAGAAATCGCACCCGGC	miRNA	miR-342-3p
t0033635	23	3	TCTCACACAGAAATCACACCCGT	miRNA	miR-342-3p
t0033696	21	3	TGGCCCTCTCTGCCCTTCCGA	miRNA	miR-328a
t0034001	23	3	AAAAGATGGGTTGAGAGGGCGTT	miRNA	miR-320d
t0034045	22	3	AAAAGCTGGGTTGAGAGGGAGG	miRNA	miR-320d
t0034129	23	3	AAAAGCTGGATTGAGAGGGCGTA	miRNA	miR-320d
t0034269	23	3	AAAAGCTGGGTTGAGAGCGCGTA	miRNA	miR-320d
t0034284	23	3	AAAAGCTGGGTTCAGAGGGCGTT	miRNA	miR-320d
t0034298	22	3	AAAAGCTGGGTTGAGAGTGCGT	miRNA	miR-320d
t0034329	22	3	CAAAGCTGGGTTGAGAGGGCGT	miRNA	miR-320d
t0034807	22	3	AAAAGCTGGGTGGAGAGGGCGT	miRNA	miR-320d
t0034962	22	3	AAAAGCTGGGTTGAGAGGGGAA	miRNA	miR-320d
t0035003	23	3	AAAAGCTGGGTTGAGAGAGCGTT	miRNA	miR-320d
t0035104	21	3	AAAGCTGGGTTGAGAGGGAGT	miRNA	miR-320d
t0035190	23	3	AAGAGCTGGGTTGAGAGGGCGTA	miRNA	miR-320d
t0033409	24	3	AAAAGCTGGGTTGAGAGGGCGCTA	miRNA	miR-320d
t0033030	23	3	AAAAGCTGGGTTGAGAGCGCGTT	miRNA	miR-320d
t0033060	23	3	AAAAGCTGGGTTGAGAGGGTGTT	miRNA	miR-320d
t0033066	20	3	AAAGCTGGGTTGAGAGGGTG	miRNA	miR-320d
t0033081	22	3	AAAGGCTGGGTTGAGAGGGCGT	miRNA	miR-320d
t0033107	23	3	AAAAGCTGGGTTGTGAGGGCGTA	miRNA	miR-320d
t0033110	23	3	AAAAGCTGGGTTGCGAGGGCGTT	miRNA	miR-320d
t0033603	24	3	AAAAGCTGGGTTGAGAGGGAGAAA	miRNA	miR-320d
t0033647	23	3	ACAAGCTGGGTTGAGAGGGCGTT	miRNA	miR-320d
t0033741	23	3	AAACGCTGGGTTGAGAGGGCGTT	miRNA	miR-320d
t0033809	22	3	AAATGCTGGGTTGAGAGGGCGT	miRNA	miR-320d
t0033885	22	3	AAAAGCTGGGTTGAGAGGCCGT	miRNA	miR-320d
t0033939	22	3	GAAAAGCTGGGTTGAGAGGGAG	miRNA	miR-320d
t0034174	23	3	AAAAGCTGGGTTGAGACGGCGTA	miRNA	miR-320d
t0034180	23	3	AAAAGCTGGGTTGAGAGGACGTT	miRNA	miR-320d
t0034340	24	3	AAAAGCTGGGTTGAGAGGGAGATA	miRNA	miR-320d
t0034366	23	3	AAAAGCTGGGTTGAGAGGGCCTT	miRNA	miR-320d
t0034390	23	3	AAAAGCTGGGTTGAGAGGGCCTA	miRNA	miR-320d
t0034467	23	3	AACAGCTGGGTTGAGAGGGCGTT	miRNA	miR-320d
t0034522	22	3	AAAAGCTGGGTTGAGCGGGCAA	miRNA	miR-320b

t0034557	21	3	AAAAGCTGGGTTGAGAAGGCA	miRNA	miR-320b
t0034587	22	3	AAATGCTGGGTTGAGAGGGCGA	miRNA	miR-320a
t0034589	23	3	AAAAGGTGGGTTGAGAGGGCGAA	miRNA	miR-320a
t0034661	20	3	AAAGCTGGGTCGAGAGGGCG	miRNA	miR-320a
t0034702	23	3	AGAAGCTGGGTTGAGAGGGCGAT	miRNA	miR-320a
t0034931	23	3	AAAAGCTGGGTTGTGAGGGCGAA	miRNA	miR-320a
t0034937	23	3	ATAAGCTGGGTTGAGAGGGCGAA	miRNA	miR-320a
t0034948	23	3	AAAAGCTGGGTTGTGAGGGCGAT	miRNA	miR-320a
t0035070	23	3	AAAAGCTGGGTTGAGTGGGCGAT	miRNA	miR-320a
t0035111	21	3	AAAAACTGGGTTGAGAGGGCG	miRNA	miR-320a
t0035117	23	3	CAAAGCTGGGTTGAGAGGGCGAA	miRNA	miR-320a
t0035148	23	3	AAAAGCTGGGTTGAGAGAGCGAT	miRNA	miR-320a
t0033249	24	3	AAAAGCTGGGGTGAGAGGGCGATT	miRNA	miR-320a
t0035028	22	3	AATAGCTGGGTTGAGAGGGCGA	miRNA	miR-320a
t0033036	21	3	AACAGCTGGGTTGAGAGGGCG	miRNA	miR-320a
t0033161	23	3	AAAAGCTGGGTTGACAGGGCGAT	miRNA	miR-320a
t0033254	20	3	AAAGCTGGGTTGAGGGGGCG	miRNA	miR-320a
t0033356	23	3	CAAAGCTGGGTTGAGAGGGCGAT	miRNA	miR-320a
t0033410	22	3	AAAGCTGGGATGAGAGGGCGAA	miRNA	miR-320a
t0033449	21	3	AAAAGCTGGGTTGAGGGGGCG	miRNA	miR-320a
t0033477	23	3	AAAAGCTGGGTCGAGAGGGCGAT	miRNA	miR-320a
t0033505	23	3	AGTCTGAGAAGGCGCACAAGGTT	miRNA	miR-3200-5p
t0033774	23	3	TCTGGGAGGTTGTAGCAGTGGAA	miRNA	miR-3192
t0033849	21	3	GTGGAAGGGAGACGGCCAGAG	miRNA	miR-3190
t0034072	22	3	TGGGACAAACGGACGGTTTTTT	miRNA	miR-3122
t0034205	21	3	TGTAAACATCCTAAACTCTCA	miRNA	miR-30c
t0034273	24	3	TGTAAACATCCTACACTCTCCGCT	miRNA	miR-30c
t0034382	22	3	TGTAAAAATCCTACACTCAGCT	miRNA	miR-30b
t0034481	25	3	CTTTCAGTCGGATGTTTGAAGCATC	miRNA	miR-30a*
t0034736	24	3	TGTAAACATCCTCGACTGGAAATC	miRNA	miR-30a
t0034790	23	3	TAGCACCATCTGAAATCGGTTAT	miRNA	miR-29c
t0034873	22	3	TAGCACCATTTGAAATCGGTTC	miRNA	miR-29a
t0034958	20	3	GCGCGGGTCGGGGGGCGGGG	miRNA	miR-2981
t0035030	24	3	CGGGTCGGGGGGCGGGGCGGACTG	miRNA	miR-2981
t0033482	19	3	GCGGGCGGCGGGGCGGGGC	miRNA	miR-2885
t0033421	20	3	GGCGGATGTAGCCAAGAGGA	miRNA	miR-270
t0033949	22	3	CTCAAGTAATTCAGGATAGGTT	miRNA	miR-26b
t0033292	22	3	TTCAAGTAATTAAGGATAGGTT	miRNA	miR-26b
t0033462	18	3	AAGTAATTCAGGATAGGT	miRNA	miR-26b
t0033663	21	3	TTCAAGAAATTCAGGATAGGT	miRNA	miR-26b
t0034048	21	3	TTCAAGTAATTCCGGATAGGT	miRNA	miR-26b
t0034568	21	3	TTCAAGTGATTCAGGATAGGT	miRNA	miR-26b
t0033691	22	3	TTCAAGAAATCCAGGATAGGCT	miRNA	miR-26a
t0034926	22	3	TTCAAGTAATACAGGATAGGCA	miRNA	miR-26a
t0034199	22	3	TTCAAGTAATCCCGGATAGGCT	miRNA	miR-26a
t0033429	22	3	TTCAAGTAATCCAGGATCGGCA	miRNA	miR-26a
t0034401	22	3	TTCAAGTAATCCAGGATGGGCT	miRNA	miR-26a
t0034954	22	3	TTCAAGTAATCAAGGATAGGCT	miRNA	miR-26a
t0033411	22	3	TGAGGTAGGAGGGTGTGTGGTT	miRNA	miR-265
t0033026	21	3	AGGCGGAGACTTGGGCGATTG	miRNA	miR-25*
t0033486	21	3	CTTTGCACTTGTCTCGGTCTG	miRNA	miR-25
t0033513	21	3	CATTGCACTTTTCTCGGTCTG	miRNA	miR-25
t0033585	21	3	CATTGCAGTTGTCTCGGTCTG	miRNA	miR-25
t0033894	22	3	CATTGCACTTGTCTCGTTCTGA	miRNA	miR-25
t0034733	22	3	CATGGCACTTGTCTCGGTCTGA	miRNA	miR-25
t0033499	22	3	CATTGCACTTGTCTCGATCTGA	miRNA	miR-25
t0034362	21	3	CATAGCACTTGTCTCGGTCTG	miRNA	miR-25
t0034397	21	3	GATTGCACTTGTCTCGGTCTG	miRNA	miR-25

t0034832	20	3	CATTGCACTTGTCTCGGGCT	miRNA	miR-25
t0034869	21	3	CACTGCACTTGTCTCGGTCTG	miRNA	miR-25
t0035098	22	3	CATTGCACATGTCTCGGTCTGA	miRNA	miR-25
t0034964	21	3	CATTGCACTTGTCTCGGTCGG	miRNA	miR-25
t0034212	20	3	CATTGCACTTGTCTCGGCCT	miRNA	miR-25
t0033351	22	3	CATTGAAATTGTCTCGGTCTGA	miRNA	miR-25
t0033366	24	3	TGGCTCAGTTCAGCAGGAACCGTT	miRNA	miR-24b
t0033376	22	3	TGGCTCAGTTCAGCAGGCACAG	miRNA	miR-24
t0033441	19	3	ATCACATTGCCAGGGCTTT	miRNA	miR-23a
t0033454	19	3	ATCACACTGCCAGGGATTT	miRNA	miR-23a
t0033467	19	3	ATCACATCGCCAGGGATTT	miRNA	miR-23a
t0033509	19	3	ATCACATTGACAGGGATTT	miRNA	miR-23a
t0033576	19	3	ATCACATTGCCAGGGAGTT	miRNA	miR-23a
t0033801	20	3	AAGCTGCCAGTTGAAGACCT	miRNA	miR-22-3p
t0034087	22	3	TGTCAGTTTGTCAAATACCCCC	miRNA	miR-223
t0034690	23	3	AGCTACATCTGGCTACTGGGGCT	miRNA	miR-222
t0034721	21	3	AGCCACATCTGGCTACTGGGT	miRNA	miR-222
t0034886	20	3	TGACTGGGGCGGGACATCTG	miRNA	miR-219-2-3p
t0035135	24	3	GACTGGGGCGGGACATCTGTTAAA	miRNA	miR-219-2-3p
t0034370	26	3	TAGCTTGTCAGACTGATGTTGACATC	miRNA	miR-21
t0033896	26	3	TAGCTTATAAGACTGATGTTGACATC	miRNA	miR-21
t0033746	21	3	TAGCTTATCAGGCTGATGTTG	miRNA	miR-21
t0033855	23	3	CAAAGTGCTCATAGTGCAGGGAG	miRNA	miR-20b
t0034117	22	3	TAAAGTGCTTATAGTGCAGGGA	miRNA	miR-20
t0034780	21	3	TAAAGTGCTTACAGTGCAGGT	miRNA	miR-20
t0035094	24	3	TGGAATGTAAAGAAGTATGTATTC	miRNA	miR-1a
t0033566	21	3	TGTGCAAATACATGCAAAACT	miRNA	miR-19b
t0033907	21	3	TGTGCAAATCCATGCAAAATT	miRNA	miR-19b
t0033256	18	3	TGAGGTAGTTTGTATAGT	miRNA	miR-1961
t0034682	20	3	TGACCTATGAATTGAAAGCC	miRNA	miR-192
t0033909	21	3	CTGACCTACGAATTGACAGCC	miRNA	miR-192
t0034151	21	3	CTGACCTATGAATTGGCAGCC	miRNA	miR-192
t0034056	22	3	TGACCTATGAATTGACAGCCCG	miRNA	miR-192
t0034415	23	3	TGACCTATGAATTGACAGACAGA	miRNA	miR-192
t0034827	24	3	TGACCTATGAATTGACAGCCCGAA	miRNA	miR-192
t0034758	21	3	CTGACCTATGAATTGACAGAA	miRNA	miR-192
t0034976	24	3	TGACCTATGAATTGACAGACAGTA	miRNA	miR-192
t0034999	20	3	TGACCTATGAATTGACGGCC	miRNA	miR-192
t0033308	21	3	TGACCTATGAATTGACAGCAA	miRNA	miR-192
t0033206	23	3	AACGGAATCCTAAAAGCAGCTGA	miRNA	miR-191
t0033713	23	3	CAACGGAATCCCGAAAGCAGCTG	miRNA	miR-191
t0033101	22	3	CAACGGAATCCCAAAAGCAACT	miRNA	miR-191
t0033465	23	3	CAACGCAATCCCAAAAGCAGCTG	miRNA	miR-191
t0033474	20	3	CAACGGAATCCCAAAAGCCG	miRNA	miR-191
t0033567	21	3	AACGGAATCCAAAAAGCAGCT	miRNA	miR-191
t0033671	23	3	CAACGGAGTCCCAAAAGCAGCTG	miRNA	miR-191
t0033718	23	3	CAACGGAATCCCCAAAGCAGCTG	miRNA	miR-191
t0033808	23	3	CAACGGAATCCCAAAAGCAGTTG	miRNA	miR-191
t0033929	22	3	CATCGGAATCCCAAAAGCAGCT	miRNA	miR-191
t0034060	22	3	CAACGGAATCCCAAACGCAGCT	miRNA	miR-191
t0034617	22	3	CAACGGAAACCCAAAAGCAGCT	miRNA	miR-191
t0034696	21	3	CAACGGAATCCCAAAAGCCGC	miRNA	miR-191
t0033237	22	3	CCACGGAATCCCAAAAGCAGCT	miRNA	miR-191
t0033250	22	3	CAACGGAATCCCAACAGCAGCT	miRNA	miR-191
t0033271	22	3	CAACGGTATCCCAAAAGCAGCT	miRNA	miR-191
t0033407	23	3	CAACGGAATCCCAAAAACAGCTG	miRNA	miR-191
t0033463	21	3	CAACGAAATCCCAAAAGCAGC	miRNA	miR-191
t0033494	24	3	CAACGGAATCCCACAAGCAGCTGA	miRNA	miR-191

t0033536	24	3 CAACGAAATCCCAAAAGCAGCTGT	miRNA	miR-191
t0033542	23	3 AACGGAATCACAAAAGCAGCTGA	miRNA	miR-191
t0033620	24	3 CAACGGAATCCCAAAAGCAGATGA	miRNA	miR-191
t0033683	22	3 AACGGAATCACAAAAGCAGCTG	miRNA	miR-191
t0033738	20	3 CAACAGAATCCCAAAAGCAG	miRNA	miR-191
t0033965	23	3 TGCAGAGAAAGGCAGTTCCTGAA	miRNA	miR-185
t0033973	23	3 TGGAGAGAAAGGAAGTTACTGAA	miRNA	miR-185
t0034200	22	3 TGGAGAGAAAGGAAGATCCTGA	miRNA	miR-185
t0034211	21	3 TGGATAGAAAGGCAGTTCCTG	miRNA	miR-185
t0034267	20	3 TGGAGAGAAGGGCAGTTCCT	miRNA	miR-185
t0034277	23	3 TGGAGAGAAAGGCAGGTCCTGAG	miRNA	miR-185
t0034424	20	3 TGGAGAGAGAGGCAGTTCCT	miRNA	miR-185
t0034465	20	3 TGGCGAGAAAGGCAGTTCCT	miRNA	miR-185
t0034537	23	3 TGGAGAGTAAGGCAGTTCCTGAA	miRNA	miR-185
t0034540	22	3 TGGAGAGCAAGGAAGTTCCTGA	miRNA	miR-185
t0034698	20	3 TGGAGAGTAAGGCAGTTCCT	miRNA	miR-185
t0034805	22	3 TGGAGAGAAAGGAAGTTCCTTA	miRNA	miR-185
t0034835	23	3 TGGATAGAAAGGCAGTTCCTGAA	miRNA	miR-185
t0033042	22	3 TGGAGAGAAAGGCAGTCCCTGC	miRNA	miR-185
t0033047	20	3 TAGAGAGAAAGGCAGTTCCT	miRNA	miR-185
t0033120	23	3 TGGAGAGAAAGGCAGTCCCTGAT	miRNA	miR-185
t0033126	22	3 TGGAGAGAAAGGCAGTTTCTGC	miRNA	miR-185
t0033201	23	3 CGGAGAGAAAGGCAGTTCCTGAG	miRNA	miR-185
t0033203	22	3 TGGAGAGAAAGGAAGGTCCTGA	miRNA	miR-185
t0033350	23	3 TGGAGTGAAAGGCAGTTCCTGAT	miRNA	miR-185
t0033466	23	3 TGGAGAGAAAGGCAGTTCTTGAG	miRNA	miR-185
t0033471	22	3 TGGCGAGAAAGGAAGTTCCTGA	miRNA	miR-185
t0033483	23	3 TGGAGAGAAAGGCACTTCCTGAA	miRNA	miR-185
t0033532	23	3 TGGAGAGAAAGGCGGTTCCTGAG	miRNA	miR-185
t0033543	20	3 TGGAGAGGAAGGCAGTTCCT	miRNA	miR-185
t0033562	23	3 TGGAGCGAAAGGCAGTTCCTGAG	miRNA	miR-185
t0033609	22	3 TGGAGAGAAAGGCAGTTACTGT	miRNA	miR-185
t0033617	23	3 TGGAGAGCAAGGCAGTTCCTGAG	miRNA	miR-185
t0033722	22	3 TGGAGAGGAAGGAAGTTCCTGA	miRNA	miR-185
t0033758	20	3 AGGAGAGAAAGGCAGTTCCT	miRNA	miR-185
t0033837	23	3 TGGCGAGAAAGGCAGTTCCTGAT	miRNA	miR-185
t0033848	22	3 TGGAGAGAAAGGGAGTTCCTGT	miRNA	miR-185
t0033903	23	3 TGGAGAGAACGGCAGTTCCTGAG	miRNA	miR-185
t0033920	23	3 TGGAGAGAGAGGCAGTTCCTGAG	miRNA	miR-185
t0033994	19	3 TGGAGAAAGGCAGTTCCTG	miRNA	miR-185
t0034016	21	3 TGGAGAGAAAGGAAGTTCCTT	miRNA	miR-185
t0034052	23	3 TGGAGAGAAGGGCAGTTCCTGAG	miRNA	miR-185
t0034070	21	3 GGGAGAGAAAGGCAGTTCCTG	miRNA	miR-185
t0034088	23	3 TGGAGCGAAAGGCAGTTCCTGAT	miRNA	miR-185
t0034105	24	3 TGGAGAGAAAGGCAGTTCCTGCAA	miRNA	miR-185
t0034188	23	3 TCGAGAGAAAGGCAGTTCCTGAT	miRNA	miR-185
t0034268	23	3 TGGAGAGAAAGGCAGTTCGTGAA	miRNA	miR-185
t0034350	24	3 TGGAGAGAAAGGCAGTTCCTTAAA	miRNA	miR-185
t0034367	23	3 TGGAGAGAAACGCAGTTCCTGAT	miRNA	miR-185
t0034372	23	3 TGGAGAGAAAGACAGTTCCTGAT	miRNA	miR-185
t0034380	20	3 TGGAGAGAAAGGCAGGTCCT	miRNA	miR-185
t0034395	23	3 TAGAGAGAAAGGCAGTTCCTGAG	miRNA	miR-185
t0034399	22	3 CGGAGAGAAAGGCAGTTCCTGT	miRNA	miR-185
t0034455	23	3 TGGAGAGAACGGCAGTTCCTGAT	miRNA	miR-185
t0034514	22	3 TGGAGAGAAAGGCAGTTCCCGC	miRNA	miR-185
t0034534	23	3 TGGAGTGAAAGGCAGTTCCTGAG	miRNA	miR-185
t0034584	20	3 TGGAGAGAAAGGCAGATCCT	miRNA	miR-185
t0034588	23	3 TGGAGAGAAAGCCAGTTCCTGAT	miRNA	miR-185

t0034703	22	3 TGGAGAGAAAGGCAGTTCATTA	miRNA	miR-185
t0034732	24	3 TGGACGGAGAACTGATAAGGGCTC	miRNA	miR-184
t0034738	22	3 AAGGTAGATAGAATAGGTCTTG	miRNA	miR-1839
t0034746	21	3 AGGTAGATAGAACAGGTATTG	miRNA	miR-1839
t0034844	21	3 AGGTAGATAGAAAAGGTCTTG	miRNA	miR-1839
t0034866	22	3 AGGTAGATAGAACAGGGCTTGT	miRNA	miR-1839
t0034978	21	3 AGGTGGATAGAACAGGTCTTG	miRNA	miR-1839
t0035016	22	3 AGGTAGATAGAACAGGGCTTGA	miRNA	miR-1839
t0035054	20	3 TGAGGCAGTAGATTGTAGTT	miRNA	miR-1827
t0035066	21	3 GAGGGAGTAGGTTGTATAGTT	miRNA	miR-1827
t0035074	22	3 TGAGGGAGTAGATTGTATCGTT	miRNA	miR-1827
t0035087	22	3 TGAGGCAGTAGATTGTATAGTG	miRNA	miR-1827
t0035156	22	3 CGAGGGAGTAGATTGTATAGTT	miRNA	miR-1827
t0035181	22	3 TGGGGGAGTAGATTGTATAGTT	miRNA	miR-1827
t0035101	21	3 TGAGGGAGGAGGTTGTATAGT	miRNA	miR-1827
t0033012	20	3 TGAGGCAGTAGATTGTATTT	miRNA	miR-1827
t0033264	22	3 TGAGGGCGTAGATTGTATAGTT	miRNA	miR-1827
t0033324	22	3 TGAGGGAGTGGATTGTATAGTT	miRNA	miR-1827
t0033560	20	3 TGAGGGAGTAGATTGTATAC	miRNA	miR-1827
t0034090	21	3 TGAGGGATTAGATTGTATAGT	miRNA	miR-1827
t0034103	21	3 TGAGGCAGTAGATTGTATAGG	miRNA	miR-1827
t0033168	22	3 TTAGGGAGTAGATTGTATAGTT	miRNA	miR-1827
t0033458	22	3 TGAGGGAGTAGATTGTATGGTT	miRNA	miR-1827
t0033674	23	3 TGAGGAAGTAGATTGTATAGTAA	miRNA	miR-1827
t0033891	22	3 TGCGGGAGTAGATTGTATAGTT	miRNA	miR-1827
t0033906	22	3 TGAGGGAGTAGATTGTGTGGTT	miRNA	miR-1827
t0034061	22	3 TGAGGGAGAAGATTGTATAGTT	miRNA	miR-1827
t0034086	20	3 TGAGGAAGTAGATTGTAGTT	miRNA	miR-1827
t0034225	20	3 TGAGGGAGTAGATTGTAGAG	miRNA	miR-1827
t0034315	41	3 TTTGGCAATGGTAGAACTCACAAACAATG	miRNA	miR-182
t0034322	22	3 TTTGGCAATGGTAGAACTCAAA	miRNA	miR-182
t0034386	24	3 AACATTCATTGCTGTCGGTGGGGT	miRNA	miR-181b
t0034404	26	3 AACATTCATTGCTGTCGGTGGGGATC	miRNA	miR-181b
t0034410	20	3 TCGGGGCGGCGGCGGCGGTC	miRNA	miR-1777a
t0034559	22	3 ACTGCAGTGAAGGCACTTGTCG	miRNA	miR-17-3p
t0034567	24	3 CAAAGTGCTTACAGTGCCGGTAGA	miRNA	miR-17
t0034569	21	3 CAAAGTGCTTACACTGCAGGT	miRNA	miR-17
t0034772	22	3 CAAAGTGCTTACAGTGCAGGGA	miRNA	miR-17
t0035000	22	3 AAAGTGCTTACAGTGCAGGAAG	miRNA	miR-17
t0035064	21	3 CAAAGTGCTTACAGTGCAGGG	miRNA	miR-17
t0035107	22	3 CAAAGTGCTTACAGTGCAGGAA	miRNA	miR-17
t0035162	24	3 CAAAGTGCTTACAGTGCAGGGAGA	miRNA	miR-17
t0033294	21	3 TAGAGCACGTAAATATTGGCG	miRNA	miR-16c
t0034711	21	3 AGCAGCACGTAAATATTGGCT	miRNA	miR-16b
t0033398	22	3 TAGCAGCACGTAAATATAGGCA	miRNA	miR-16b
t0033987	22	3 TAGCAGCGCGTAAATATTGGCA	miRNA	miR-16b
t0033760	22	3 TAGAAGCACGTAAATATTGGCA	miRNA	miR-16b
t0034427	22	3 TAGCAGAACGTAAATATTGGCA	miRNA	miR-16b
t0033053	22	3 TAGCAGCACGTAAATATTGTCA	miRNA	miR-16b
t0033157	27	3 GGCGGGGAGCGGTCGGGCGGCGGCGGG	miRNA	miR-1607
t0033637	21	3 AGCAGCACGTAAATATTGGTG	miRNA	miR-16
t0033782	24	3 TAGAAGCACGTAAATATTGGCGAA	miRNA	miR-16
t0033886	23	3 TAGCAACACGTAAATATTGGCGA	miRNA	miR-16
t0034859	22	3 TAGCAGCATGTAAATATTGGAG	miRNA	miR-16
t0034987	22	3 TAGCAGCACGTAAATGTTGTCG	miRNA	miR-16
t0034293	21	3 TAGCAGCACGTAAATATTGAG	miRNA	miR-16
t0033061	23	3 TGGCAGCACGTAAATATTGGCGA	miRNA	miR-16
t0033632	22	3 TAGCAGAAAGTAAATATTGGCG	miRNA	miR-16

t0034323	22	3 TAGCAGCACGGAAATATTGGTG	miRNA	miR-16
t0034328	22	3 TAGCAGCGTGTAAATATTGGCG	miRNA	miR-16
t0034684	22	3 TGGCAGCACGTAAATATTGCCG	miRNA	miR-16
t0034894	21	3 TAGCAGCACTTAAATATTGGC	miRNA	miR-16
t0033105	23	3 TAGCAGCACGTAAATAATGGCGA	miRNA	miR-16
t0033114	22	3 TAGCAGCATGCAAATATTGGCG	miRNA	miR-16
t0033257	23	3 ATAGCAGCACGTAAATATTGGGG	miRNA	miR-16
t0033309	20	3 TAGCAGCACGTAATTATTGG	miRNA	miR-16
t0033335	22	3 TAGCAGCATGTAAATATTGGTG	miRNA	miR-16
t0033393	20	3 TAGCAGCAGGTAAATATTGG	miRNA	miR-16
t0033447	23	3 TAGCAGTACGTAAATATTGGCGA	miRNA	miR-16
t0033480	22	3 TAGCAGGACGTAAATATTGGAG	miRNA	miR-16
t0033508	23	3 TAGCAGGACGTAAATATTGGCGA	miRNA	miR-16
t0033539	23	3 TAGCAGCACGCAAATATTGGCGT	miRNA	miR-16
t0033595	23	3 TAGTAGCACGTAAATATTGGCGT	miRNA	miR-16
t0033802	22	3 TAGCAGCACGTAAAAATTGGAG	miRNA	miR-16
t0033890	22	3 TAGCAGCACGCAAATATTGTCG	miRNA	miR-16
t0033950	22	3 TAGCAGCACGTAAATATTGCAG	miRNA	miR-16
t0033996	22	3 AAGAAGCACGTAAATATTGGCG	miRNA	miR-16
t0034013	22	3 TAGCAGCACGCAAATATTGCCG	miRNA	miR-16
t0034019	21	3 TAGAAGCACGTAAATATTGGC	miRNA	miR-16
t0034032	20	3 TAGCAGCACGTAAATTTTGG	miRNA	miR-16
t0034063	20	3 TAGCAGAACATCATGGTTTT	miRNA	miR-15c
t0034074	19	3 TAGCAGAACATCATGGGTT	miRNA	miR-15b
t0034153	20	3 TAGCAGAACATCATGGGTTA	miRNA	miR-15b
t0034229	20	3 TAGAAGAACATCATGGTTTA	miRNA	miR-15b
t0034260	20	3 TAGCAGCACATCATTGTTTA	miRNA	miR-15b
t0034391	19	3 TAGCAGAATATCATGGTTT	miRNA	miR-15b
t0034447	19	3 TAGCAGCACATCATGGGTA	miRNA	miR-15b
t0034671	19	3 TAGCACCACATCATGGTTT	miRNA	miR-15b
t0034728	19	3 TACCAGCACATCATGGTTT	miRNA	miR-15b
t0034734	21	3 TAGCCGCACATCATGGTTTAC	miRNA	miR-15b
t0034755	20	3 TAGCAGCACATCATGATTTA	miRNA	miR-15b
t0034989	19	3 TAGAAGAACATCATGGTTT	miRNA	miR-15b
t0035047	21	3 TAGCAGCACATAATGGTTTAT	miRNA	miR-15b
t0033175	21	3 TAGCAGCACATCATGATTTAC	miRNA	miR-15b
t0033068	19	3 TCGCAGCACATCATGGTTT	miRNA	miR-15b
t0033187	21	3 TAGCAGCACATCACGGTTTAC	miRNA	miR-15b
t0033298	19	3 TAGAAGCACATAATGGTTT	miRNA	miR-15a
t0033349	18	3 TAGCAGAACATAATGGTT	miRNA	miR-15a
t0033491	19	3 TAGTAGCACATAATGGTTT	miRNA	miR-15a
t0033650	21	3 TACCAGCACATAATGGTTTGT	miRNA	miR-15a
t0033661	20	3 TAGCAGCACATAATGGGTTC	miRNA	miR-15a
t0033810	20	3 TAGCAGCACATAATCGTTTG	miRNA	miR-15a
t0033913	21	3 TAGCAGCACATGATGGTTTGT	miRNA	miR-15a
t0034294	20	3 TAGCAGCACAAAATGGTTTG	miRNA	miR-15a
t0034489	19	3 TAGCAGCACATAATGGTCT	miRNA	miR-15a
t0034812	21	3 TAGCAGCACAGAATGGTTTGT	miRNA	miR-15a
t0034819	21	3 TAGCAGCACATAATGATTTGT	miRNA	miR-15a
t0035062	22	3 TAGCAGCACATAATAGTTTGTG	miRNA	miR-15a
t0035178	19	3 TAGCAGCAAATAATGGTTT	miRNA	miR-15a
t0033202	24	3 TAGCAGCACATAATGGTTTGGGGA	miRNA	miR-15a
t0033220	21	3 TAGCAGCACATTATGGTTTGT	miRNA	miR-15a
t0033330	22	3 AAGCAGCACATAATGGTTTGTG	miRNA	miR-15a
t0033445	22	3 TAGCAGCACATAACGGTTTGTG	miRNA	miR-15a
t0033545	20	3 GGGCTCGGGCTGGGCGAGGC	miRNA	miR-1587
t0033737	21	3 TCAGTGCAATACAGAACTTTG	miRNA	miR-152b
t0033769	21	3 TCAGTGCATAACAGAACTTTG	miRNA	miR-148b-3p

t0033948	21	3 TCAGTGCATCACAGAACCTTG	miRNA	miR-148b-3p
t0033963	21	3 TCAGGGCACTACAGAACTTTG	miRNA	miR-148a
t0033997	21	3 TCAGTGCACTAAAGAACTTTG	miRNA	miR-148a
t0034247	23	3 AGATATCATCATATACTGTAAGT	miRNA	miR-144*
t0034429	21	3 GGATATCATCATATACTGTAC	miRNA	miR-144*
t0034511	21	3 GGATATAATCATATACTGTAA	miRNA	miR-144*
t0034655	19	3 CTACAGTATAGATGATGCA	miRNA	miR-144
t0034868	18	3 TACAGTATAGCTGATGTA	miRNA	miR-144
t0034902	20	3 TACAGTATAGGTGATGTACT	miRNA	miR-144
t0035008	19	3 CTACAGTATGGATGATGTA	miRNA	miR-144
t0033069	21	3 TACAGTATAGATGATGTCCTT	miRNA	miR-144
t0034336	23	3 AGAGATGAAGCACTGTAGCTATC	miRNA	miR-143
t0033268	23	3 TGAGATGAAGCACTGAAGCTATC	miRNA	miR-143
t0033565	21	3 CTCATAAAGTAGAAAGCACTA	miRNA	miR-142-5p
t0034359	20	3 CACATAAAGTAGAAAGCACT	miRNA	miR-142-5p
t0034487	20	3 CCCATACAGTAGAAAGCACT	miRNA	miR-142
t0033834	20	3 CCCATAAAGTAGAGAGCACT	miRNA	miR-142
t0034577	20	3 CCCATAAAGTGGAAAGCACT	miRNA	miR-142
t0035007	20	3 CCCATAAAGTAGAAAGTACT	miRNA	miR-142
t0033086	21	3 CCCATAAAGTAGAAAGCACTC	miRNA	miR-142
t0033731	20	3 CCCGTAAAGTAGAAAGCACT	miRNA	miR-142
t0034743	22	3 TACCACAGGGGAGAACAACGGA	miRNA	miR-140
t0034761	22	3 TACAACAGGGTAGAACAACGGA	miRNA	miR-140
t0034852	22	3 TACCACAGGGTAGAACTACAGA	miRNA	miR-140
t0033575	20	3 CCACAGGGTAGAACCAAGGA	miRNA	miR-140
t0034942	23	3 TACCACAGGGTAGAACCACCGAT	miRNA	miR-140
t0033516	24	3 TACCACAGGGTAGAGCCACGGACG	miRNA	miR-140
t0034218	24	3 TACCACTGGGTAGAACCACGGACA	miRNA	miR-140
t0033059	24	3 TACCACGGGGTAGAACCACGGACG	miRNA	miR-140
t0033485	22	3 CCCACAGGGTAGAACCACGGAC	miRNA	miR-140
t0034059	24	3 TACCACAGGGTTGAACCACGGACA	miRNA	miR-140
t0034321	23	3 AACCACAGGGTAGAACCACGGAT	miRNA	miR-140
t0034495	24	3 TACCACAGGGGAGAACCACGGACT	miRNA	miR-140
t0034601	22	3 ACCACAGGGTAGAACCACGAAA	miRNA	miR-140
t0033025	24	3 TACCAAAGGGTAGAACCACGGACA	miRNA	miR-140
t0033049	22	3 ACCACAGGGTCGAACCACGGAA	miRNA	miR-140
t0033064	23	3 TACCACAGGGTAGATCCACGGAC	miRNA	miR-140
t0033079	23	3 TACCACTGGGTAGAACCACGGAC	miRNA	miR-140
t0033091	23	3 TACCTCAGGGTAGAACCACGGAT	miRNA	miR-140
t0033097	23	3 ACCACAGGATAGAACCACGGACA	miRNA	miR-140
t0033104	23	3 ACAACAGGGTAGAACCACGGACT	miRNA	miR-140
t0033127	22	3 ACTACAGGGTAGAACCACGGAT	miRNA	miR-140
t0033129	23	3 TACCACAGGGTACAACCACGGAC	miRNA	miR-140
t0033131	23	3 ACCACAGGGTAGAACCACGGGCT	miRNA	miR-140
t0033185	22	3 ACCACAGGGTAGGACCACGGAC	miRNA	miR-140
t0033194	23	3 ACCACAGGGTAGAACCACGGCTT	miRNA	miR-140
t0033214	22	3 ACCACACGGTAGAACCACGGAC	miRNA	miR-140
t0033238	23	3 TACCACAGGGTTGAACCACGGAC	miRNA	miR-140
t0033284	21	3 ACCACAGGGTAGAAATACGGA	miRNA	miR-140
t0033293	23	3 AACACAGGGTAGAACCACGGACA	miRNA	miR-140
t0033325	20	3 CCACAGGGTAGAAACACGGA	miRNA	miR-140
t0033328	20	3 ACCGCAGGGTAGAACCACGG	miRNA	miR-140
t0033331	23	3 TACCACAGGGTAGAAACACGGAG	miRNA	miR-140
t0033342	23	3 TACCACAGGGCAGAACCACGGAG	miRNA	miR-140
t0033347	22	3 TACCACAGGGTAGATCCACGGA	miRNA	miR-140
t0033358	23	3 TACCACAGGGTAGAACCCCGGCT	miRNA	miR-140
t0033367	22	3 ACCCCAGGGTAGAACCACGGAA	miRNA	miR-140
t0033378	21	3 ACCACAGGGTAGAACTCCGGA	miRNA	miR-140

t0033380	23	3	ACAACAGGGTAGAACCACGGACG	miRNA	miR-140
t0033384	22	3	TACCACAGGGTAGAAAAACGGA	miRNA	miR-140
t0033385	22	3	AACACAGGGTAGAACCACGGAT	miRNA	miR-140
t0033396	21	3	ACCACAGTGTAGAACCACGGC	miRNA	miR-140
t0033402	24	3	TACTACAGGGTAGAACCACGGACG	miRNA	miR-140
t0033446	24	3	TACCACAGGGTAGAACCACGCACA	miRNA	miR-140
t0033461	24	3	TACCACAGGGTAGAACCACTGACA	miRNA	miR-140
t0033564	24	3	CACCACAGGGTAGAACCACGGACG	miRNA	miR-140
t0033629	23	3	TACCACAGGGTAGAACACCGGAC	miRNA	miR-140
t0033667	24	3	TACCACAGGGTAGAACCACGGAGG	miRNA	miR-140
t0033678	22	3	ACCACAGGGTAGAACCTCGGAT	miRNA	miR-140
t0033695	21	3	AACACAGGGTAGAAACACGGA	miRNA	miR-140
t0033709	22	3	ACCCCAGGGTAGAACCACGGAC	miRNA	miR-140
t0033712	21	3	TACCACAGGGAAGAACCACGG	miRNA	miR-140
t0033735	23	3	TACCACAGGGTAGAACCCAGGAC	miRNA	miR-140
t0033869	21	3	ACCACAGGGTAGAACAACGGT	miRNA	miR-140
t0033889	21	3	TACCACAGGGTAGAGCCACGG	miRNA	miR-140
t0033892	23	3	TGCCACAGGGTAGAACCACGGAT	miRNA	miR-140
t0033904	22	3	ACCACAGGGTAGAACCAAGGAG	miRNA	miR-140
t0033921	22	3	TACCACAGTGTAGAACCACGGC	miRNA	miR-140
t0033938	23	3	TACCACAGGGAAGAACCACGGAG	miRNA	miR-140
t0033984	22	3	TACCACAGGGAAGAACAACGGA	miRNA	miR-140
t0033991	23	3	ACCCCAGGGTAGAACCACGGACG	miRNA	miR-140
t0034014	22	3	ACCACAGCGTAGAACCACGGAT	miRNA	miR-140
t0034015	23	3	TACCACAGGGTAGAACCACCGCC	miRNA	miR-140
t0034037	24	3	TACCACAGGGTACAACCACGGACA	miRNA	miR-140
t0034043	22	3	ACCACAGGGTAGAACCTCGGAC	miRNA	miR-140
t0034064	24	3	TACCACAGGGTAGAACCAAGGCCA	miRNA	miR-140
t0034065	21	3	TACCACAGGGTAGAACCATGG	miRNA	miR-140
t0034130	22	3	ACCACAAGGTAGAACCACGGAT	miRNA	miR-140
t0034162	21	3	ACCACAGGGGAGAACCACGGT	miRNA	miR-140
t0034169	22	3	TAACACAGGGTAGAACAACGGA	miRNA	miR-140
t0034194	23	3	ACCACAGGGTAGAACAAAGGACA	miRNA	miR-140
t0034248	23	3	TACCACAGGGTAGAACCACGACA	miRNA	miR-140
t0034256	22	3	ACCTCAGGGTAGAACCACGGAC	miRNA	miR-140
t0034264	22	3	ACCACAGGGTAGAAAAACGGAC	miRNA	miR-140
t0034275	23	3	AACACAGGGTAGAACCACGGACT	miRNA	miR-140
t0034345	22	3	TACCACAGGGTAGAATCACGGC	miRNA	miR-140
t0034412	24	3	TACCACAGGGTAGAACCACCGACG	miRNA	miR-140
t0034422	23	3	ACCACAGGGTAGAACCACGGTCA	miRNA	miR-140
t0034512	23	3	TACCCCAGGGTAGAACCACGGAT	miRNA	miR-140
t0034535	23	3	TACCACAGGGTAGAACCACGCAA	miRNA	miR-140
t0034545	21	3	ACCACAGGGGAGAACCACGGC	miRNA	miR-140
t0034548	22	3	TACAACAGGGTAGAACCAAGGA	miRNA	miR-140
t0034549	23	3	TACCACAGGGTAGAACCATGGAG	miRNA	miR-140
t0034560	23	3	TACCACAGGGTAGAGCCACGGAT	miRNA	miR-140
t0034581	22	3	TACCATAGGGTAGAATCACGGA	miRNA	miR-140
t0034600	21	3	CACCACAGGGTAGAACCACGG	miRNA	miR-140
t0034635	24	3	TACCACAGGGTAGAACAACGGATA	miRNA	miR-140
t0034644	22	3	ACCACACGGTAGAACCACGGAA	miRNA	miR-140
t0034665	21	3	ACCACAGGGTAGTACCACGGA	miRNA	miR-140
t0034681	23	3	TACCACAGGGTAGAAGCACGGAT	miRNA	miR-140
t0034701	22	3	CACCACAGGGTAGAACCCCGGA	miRNA	miR-140
t0034712	22	3	ACCACAGGGTAGAAACAAGGAC	miRNA	miR-140
t0034715	22	3	ACCACAGGCTAGAACCACGGAA	miRNA	miR-140
t0034756	23	3	TACCACAGGGTAGCACCACGGAT	miRNA	miR-140
t0034762	23	3	TACCACAGGGTACAACCACGGAT	miRNA	miR-140
t0034764	23	3	ACCAAAGGGTAGAACCACGGACA	miRNA	miR-140

t0034765	22	3	ACCACAGTGTAGAACCACGGAC	miRNA	miR-140
t0034770	26	3	ACCACAGGGTAGAACCACGGAAGAGA	miRNA	miR-140
t0034786	21	3	TACAACAGGGTAGAACCACGG	miRNA	miR-140
t0034801	21	3	TGCCACAGGGTAGAACCACGG	miRNA	miR-140
t0034856	23	3	ACCACAGGGTAGAACCAAGGACG	miRNA	miR-140
t0034858	23	3	TAACACAGGGTAGAACAACGGAC	miRNA	miR-140
t0034874	22	3	TACCACAGGGGAGAACCCCGGA	miRNA	miR-140
t0034876	22	3	CCCACAGGGTAGAACCACGGAT	miRNA	miR-140
t0034927	23	3	TACACAGGGTAGAACCACGGACA	miRNA	miR-140
t0034935	23	3	TACCACTGGGTAGAACCACGGAA	miRNA	miR-140
t0034967	23	3	ACCACAGGGTAGAAACACGGACT	miRNA	miR-140
t0034970	22	3	ACCACAGGGTAGGACCACGGAT	miRNA	miR-140
t0034975	21	3	CAGTGCAATGTTAAAAGGGAA	miRNA	miR-130a
t0034980	20	3	ACTCGGCGTGGCGTCGGTAG	miRNA	miR-1307
t0035025	24	3	TCTGGGCAACAAAGTGAGAACTGA	miRNA	miR-1285
t0035038	24	3	TCGGATCCGTCTGAGCTTGGGATC	miRNA	miR-127
t0035051	21	3	TAAGGCACGCGGTGAATGTCA	miRNA	miR-124-3p
t0035055	20	3	GTGAGGACTTGGGAGGGGGA	miRNA	miR-1224
t0035078	22	3	GTGAGGACTCGGGAGGGGGAGA	miRNA	miR-1224
t0035132	22	3	AGCAGCATTATACAGGGCTATT	miRNA	miR-107
t0035134	22	3	AGCAGCATTGTAAAGGGCTATT	miRNA	miR-107
t0035136	22	3	AGCAGCATTGCACAGGGCTATA	miRNA	miR-107
t0035140	22	3	AGCAGAATTGTACAGGGCTATT	miRNA	miR-107
t0035146	22	3	AGCAACATTGTACAGGGCTATT	miRNA	miR-107
t0035160	22	3	AGCAGCACTGTACAGGGCTATT	miRNA	miR-107
t0033172	20	3	CCGCACTGTGGGTACTTGAT	miRNA	miR-106b*
t0033993	22	3	CCGCACTGTGGGTAATTGCTGA	miRNA	miR-106b*
t0034485	21	3	ACCGCACTGTGGGGACTTGCT	miRNA	miR-106b*
t0034699	20	3	CCACACTGTGGGTACTTGCT	miRNA	miR-106b*
t0033179	22	3	TACCGCACTGTGGGAACTTGCT	miRNA	miR-106b*
t0033139	21	3	TAAAGCGCTGACAGTGCAGAT	miRNA	miR-106b
t0034593	20	3	TAAAGAGCTGACAGTGCAGA	miRNA	miR-106b
t0033281	20	3	TAAAGTGCTGACAGTGCGGA	miRNA	miR-106b
t0033442	22	3	TAAAGTGCTGACAGTGCAGATC	miRNA	miR-106b
t0033611	21	3	TAAAGTGCTGACAGAGCAGAT	miRNA	miR-106b
t0033771	21	3	AAAAGTGCTGACAGTGCAGAT	miRNA	miR-106b
t0033868	21	3	TGAAGTGCTGACAGTGCAGAT	miRNA	miR-106b
t0033946	20	3	TAAAGTGCTGACCGTGCAGA	miRNA	miR-106b
t0033270	21	3	TAAAGTGCTGACAGTGAAGAA	miRNA	miR-106b
t0033315	20	3	TAAAGTGATGACAGTGCAGA	miRNA	miR-106b
t0033680	21	3	AAAAGTGCTTACAGTGCCGGT	miRNA	miR-106
t0033768	22	3	AAAAGTGCTTACAGTGCAGGGA	miRNA	miR-106
t0034769	23	3	AAAAGTGCTTACAGTGCAGGTCG	miRNA	miR-106
t0033083	22	3	AAAAGTGCTTACAGTACAGGTA	miRNA	miR-106
t0033169	21	3	AAAAGGGCTTACAGTGCAGGT	miRNA	miR-106
t0033285	21	3	AAAAGTGCTTACAGTGCAGGG	miRNA	miR-106
t0033503	21	3	AAAAGCGCTTACAGTGCAGGT	miRNA	miR-106
t0033690	21	3	AGCAGCATTGTACAGGACTAA	miRNA	miR-103a
t0033728	23	3	AGCAGCATTGTAAAGGGCTATGT	miRNA	miR-103a
t0033937	20	3	AGCAGCATTGTACAGTGCTA	miRNA	miR-103a
t0034759	20	3	AGCAGCATTGTACAGGGCCA	miRNA	miR-103a
t0034929	23	3	AGCTGCATTGTACAGGGCTATGA	miRNA	miR-103a
t0034981	26	3	AGCAGCATTGTACAGGACTATGAATC	miRNA	miR-103a
t0033227	22	3	AGCAGCATAGTACAGGGCTATG	miRNA	miR-103a
t0033598	23	3	AGCAGCATGGTACAGGGCTATGA	miRNA	miR-103a
t0033790	22	3	AGCAGCATGGTACAGGGCTATG	miRNA	miR-103a
t0034299	23	3	AGCAGCATTATACAGGGCTATGT	miRNA	miR-103a
t0034687	22	3	AGCTGCATTGTACAGGGCTATG	miRNA	miR-103a

t0034731	23	3	AGCAGCATTGCACAGGGCTATGT	miRNA	miR-103a
t0035174	20	3	AGCAGCATTCTACAGGGCTA	miRNA	miR-103a
t0033153	23	3	AGCAGCATTGTACAGGGCTAGGA	miRNA	miR-103a
t0033155	23	3	AGCAGCAGTGTACAGGGCTATGA	miRNA	miR-103a
t0033276	23	3	AGCAACATTGTACAGGGCTATGT	miRNA	miR-103a
t0033397	23	3	AGCATCATTGTACAGGGCTATGA	miRNA	miR-103a
t0033423	23	3	AGCAGAATTGTACAGGGCTATGT	miRNA	miR-103a
t0033452	21	3	AGCAGCAGTGTACAGGGCTAT	miRNA	miR-103a
t0033524	22	3	AGCAGCATTGTACAGGGCAATG	miRNA	miR-103a
t0033541	23	3	AGCAGCATTGTACAGCGCTATGA	miRNA	miR-103a
t0033656	22	3	GTACAGTACTGCGATAACTGAT	miRNA	miR-101c
t0033665	22	3	TACAGTACTGGGATAACTGACT	miRNA	miR-101c
t0034000	20	3	TACAGTACGGTGATAACTGA	miRNA	miR-101
t0034440	22	3	TACAGTGCTGTGATAACTGAAA	miRNA	miR-101
t0034454	21	3	GTACAGTACTGTGACAACTGA	miRNA	miR-101
t0034566	21	3	GTACAGTATTGTGATAACTGA	miRNA	miR-101
t0034640	23	3	GTACAGTACTGTGATAAATGAAA	miRNA	miR-101
t0034673	21	3	TACAGTACGGTGATAACTGAA	miRNA	miR-101
t0034744	21	3	TACTGTACTGTGATAACTGAA	miRNA	miR-101
t0034913	22	3	TACAGTACTGTGATAACTAAAG	miRNA	miR-101
t0034994	20	3	TACAGGACTGTGATAACTGA	miRNA	miR-101
t0034995	20	3	TAGAGTACTGTGATAACTGA	miRNA	miR-101
t0035024	21	3	TACAGTAGTGTGATAACTGAA	miRNA	miR-101
t0033846	22	3	TACAGTACCGTGATAACTGAAA	miRNA	miR-101
t0034799	22	3	TACGGTACTGTGATAACTGAAG	miRNA	miR-101
t0033159	21	3	TACAGTACTGTGATACCTGAA	miRNA	miR-101
t0033310	20	3	TCCAGTACTGTGATAACTGA	miRNA	miR-101
t0033374	21	3	GTACAGTACTGTGATAGCTGA	miRNA	miR-101
t0033638	26	3	TACCCTGTAGATCCGAATTTGCGATC	miRNA	miR-10
t0033646	21	3	TAAGGTAGTAGTTTGTGCTGT	miRNA	let-7i
t0033710	23	3	TGAGGTAGAAGTTTGTGCTGTTT	miRNA	let-7i
t0033964	21	3	TGAGGTAGTAGTTTGTGATGT	miRNA	let-7i
t0034077	20	3	TGAGGTAGTGGTTTGTGCTG	miRNA	let-7i
t0034119	19	3	TGAGGTAGGAGTTTGTGCT	miRNA	let-7i
t0034237	21	3	TGAGGTAGTAGTTTGTGCTGG	miRNA	let-7i
t0034572	20	3	TGAGGTAGTAGTTTGTGCTC	miRNA	let-7i
t0034636	23	3	TGAGGTAGTAGTTTGTGATGTTT	miRNA	let-7i
t0034670	23	3	TGAGGCAGTAGTTTGTGCTGTTT	miRNA	let-7i
t0034727	21	3	TGAGGTAGTAGTTTATGCTGT	miRNA	let-7i
t0034875	23	3	TGAGGGAGTAGTTTGTGCTGTTG	miRNA	let-7i
t0034955	22	3	TGAGGTAGTAGTTTGTCCTGTT	miRNA	let-7i
t0034997	22	3	TGAGGTAGTAGTTTGTGCCGTT	miRNA	let-7i
t0033062	21	3	TGCGGTAGTAGTTTGTGCTGT	miRNA	let-7i
t0033133	20	3	TGAGGTAGTAGTTTGCGCTG	miRNA	let-7i
t0033229	22	3	TGAGGGAGGAGTTTGTGCTGTT	miRNA	let-7i
t0033470	22	3	TGTGGTAGTAGTTTGTGCTGTT	miRNA	let-7i
t0033498	22	3	TGAGGTAGTGGTTTGTGCTGTT	miRNA	let-7i
t0033640	21	3	TGAGGTACTAGTTTGTACAGT	miRNA	let-7g
t0033884	20	3	TGAGGTAGTAGTTTGTAAAG	miRNA	let-7g
t0033895	22	3	TGAGGTAGTAGTTAGTACAGTT	miRNA	let-7g
t0034101	22	3	TGAGGTAGTTGTTTGTACAGTT	miRNA	let-7g
t0034221	22	3	TGAGGTAGTACTTTGTACAGTT	miRNA	let-7g
t0034394	22	3	TGAGGTAGTAGTTTATACAGTA	miRNA	let-7g
t0034446	21	3	TGAGGTAGTAGTTCGTACAGT	miRNA	let-7g
t0034492	22	3	TGAGGTAGTAGTTTGTACAGGA	miRNA	let-7g
t0034570	21	3	TGAGGTAGAAGTTTGTACAGT	miRNA	let-7g
t0034707	22	3	TGAGGTAGTGGATTGTATAGTA	miRNA	let-7f-5p
t0035139	22	3	TGAGGTAGTAGATTGTATCGGT	miRNA	let-7f-5p

t0035141	22	3 TGAGGTAGTTGATTGTATAGTA	miRNA	let-7f-5p
t0035168	21	3 TGAGGTAGTAGATTGTACGTT	miRNA	let-7f-5p
t0033418	22	3 TGAGGTGGTGGATTGTATAGTT	miRNA	let-7f-5p
t0033530	22	3 TGAGGTAGTAGATTGTCTAGTA	miRNA	let-7f
t0033705	21	3 GAGGTAGTAGATTGTATATTT	miRNA	let-7f
t0034026	22	3 CGAGGTAGTAGATTGCATAGTT	miRNA	let-7f
t0034173	23	3 TGAGGTAGTAGATCGTATAGTTT	miRNA	let-7f
t0034192	22	3 TGCGGTAGTAGATTGTATAGAT	miRNA	let-7f
t0034383	22	3 GGAGGTAGTAGATTGTATAGGT	miRNA	let-7f
t0034387	22	3 TGAGGTCGTAGCTTGTATAGTT	miRNA	let-7f
t0034615	22	3 TGCGGTAGTAGATTGTATAGTA	miRNA	let-7f
t0033476	23	3 TGAGGTAGTAGATTGTATTGTTG	miRNA	let-7f
t0033755	23	3 TGAGGTAGTAGAATGTATAGTTA	miRNA	let-7f
t0033881	23	3 TGAGGTAGTAGATTGGATAGTTT	miRNA	let-7f
t0034310	21	3 CGAGGTAGTAGATTGCATAGT	miRNA	let-7f
t0034409	23	3 TGAGGTAGTCGATTGTATAGTTA	miRNA	let-7f
t0033011	22	3 TGAGGTAGTAGATTGTGTACTT	miRNA	let-7f
t0033013	22	3 CGAGGTAGTAGATTGTATACTT	miRNA	let-7f
t0033035	23	3 TGAGGTAGTTGATTGTATAGTTG	miRNA	let-7f
t0033102	23	3 TAAGGTAGTAGATTGTATAGTTT	miRNA	let-7f
t0033150	22	3 TGAGGTAGTAGCTTGCATAGTT	miRNA	let-7f
t0033338	21	3 TGAGGTAGTAGATTGTGTTGT	miRNA	let-7f
t0033353	23	3 TGAGGTAGTAGATTGTAAAGTTA	miRNA	let-7f
t0033357	23	3 TCAGGTAGTAGATTGTATAGTTG	miRNA	let-7f
t0033370	22	3 TGAGGTAGTAGATTGCATAGGT	miRNA	let-7f
t0033404	22	3 CGAGGTGGTAGATTGTATAGTT	miRNA	let-7f
t0033435	20	3 TGAGGTAGTAGAGTGTATAG	miRNA	let-7f
t0033591	22	3 TGGGGTAGTAGATTGTGTAGTT	miRNA	let-7f
t0033726	22	3 TGAGGAAGTAGATTGTTTAGTT	miRNA	let-7f
t0033751	22	3 TGAGGTCGTCGATTGTATAGTT	miRNA	let-7f
t0033795	23	3 TGAGGTAGTAGATTCTATAGTTA	miRNA	let-7f
t0033819	19	3 TGATGTAGTAGATTGTATA	miRNA	let-7f
t0033836	22	3 TGAGGTAGTAGCTTGTGTAGTT	miRNA	let-7f
t0033838	23	3 TGAGGTAGTAGATCGTATAGTTA	miRNA	let-7f
t0033863	20	3 TGAGGTAGTAGATTGTGGAG	miRNA	let-7f
t0033877	22	3 TGAGGTAGTAGATTATATACTT	miRNA	let-7f
t0033912	23	3 TGAGGTAGTAGATTGTTTAGTTG	miRNA	let-7f
t0033982	23	3 TGAGGTAGTAGATTGTCTAGTTA	miRNA	let-7f
t0033989	22	3 TGAGGTAGCAGATTGTATAGTA	miRNA	let-7f
t0034002	18	3 GAGGTAGTAGATTGTATT	miRNA	let-7f
t0034038	22	3 TGGGGTAGTAGATTGTATATTT	miRNA	let-7f
t0034080	22	3 TGGTGTAGTAGATTGTATAGTT	miRNA	let-7f
t0034207	23	3 TGAGCTAGTAGATTGTATAGTTT	miRNA	let-7f
t0034325	21	3 TGAGGTAGTAGATTGTGCAGT	miRNA	let-7f
t0034334	22	3 TGAGGTAGTAGATTGCATAGTG	miRNA	let-7f
t0034369	18	3 TGAGGTAGTAGATTATAT	miRNA	let-7f
t0034475	19	3 TGTGGTAGTAGATTGTATA	miRNA	let-7f
t0034488	27	3 TGAGGTAGTAGATTGTATAGTTAAAAA	miRNA	let-7f
t0034513	21	3 TGAGGTAGTAGCTTGTCTAGT	miRNA	let-7f
t0034532	23	3 TGAGGTAGTAGATTGTATAGATT	miRNA	let-7f
t0034632	21	3 TGAGGTAGGAGATTGTATATT	miRNA	let-7e
t0034646	22	3 TGAGGAAGGAGATTGTATAGTT	miRNA	let-7e
t0034650	22	3 TGAGGTAGGAGATTGTACAGTT	miRNA	let-7e
t0034666	22	3 TGGGGTAGGAGATTGTATAGTT	miRNA	let-7e
t0034704	22	3 TGAGGTAGGAGATTGAATAGTT	miRNA	let-7e
t0034792	21	3 AGAGGTAGTAGGTTGCTTAGT	miRNA	let-7d
t0034900	22	3 AGAGGTTGTAGGTTGCATAGTT	miRNA	let-7d
t0035004	22	3 ATAGGTAGTAGGTTGCATAGTT	miRNA	let-7d

t0035037	20	3 AGAGGTAGTAGGTTGCATGG	miRNA	let-7d
t0035045	21	3 AGAGGTACTAGGTTGCATAGT	miRNA	let-7d
t0035048	21	3 ACAGGTAGTAGGTTGCATAGT	miRNA	let-7d
t0035110	23	3 AGAGGTAGTAGGATGCATAGTTA	miRNA	let-7d
t0035112	21	3 AGAGGTAGTAGGTCGCATAGT	miRNA	let-7d
t0033897	21	3 AGAGGTAGTAAGTTGCATAGT	miRNA	let-7d
t0033952	20	3 AGAGATAGTAGGTTGCATAG	miRNA	let-7d
t0034667	19	3 GAGGTAGTAGGTTGCATAG	miRNA	let-7d
t0034825	19	3 AGAGGAAGTAGGTTGCATA	miRNA	let-7d
t0034959	21	3 AGAGGTAGTAGGTTGCATAGC	miRNA	let-7d
t0033103	20	3 AGAGGTAGTAGGTTGCTTAG	miRNA	let-7d
t0033218	20	3 AGAGGTAGTAGGTTGGATAG	miRNA	let-7d
t0033222	20	3 AGTGGTAGTAGGTTGCATAG	miRNA	let-7d
t0033365	20	3 AGAGGTAGTAGGTTACATAG	miRNA	let-7d
t0033406	19	3 AGAGGTAGTAGGTTGTATA	miRNA	let-7d
t0033527	21	3 TGAGGAAGTAGGTTGTATGGT	miRNA	let-7c
t0033554	21	3 TGAGGCAGTAGGTTGTATGGT	miRNA	let-7c
t0033579	25	3 TGAGGTAGTAGGTTATATGGTTATC	miRNA	let-7c
t0033593	21	3 TGAGGTAGCAGGTTGTATGGT	miRNA	let-7c
t0034147	25	3 TGAGGTATTAGGTTGTATGGTTATC	miRNA	let-7c
t0034186	21	3 TGAGGTAGTAGGGTGTATGGT	miRNA	let-7c
t0034613	24	3 TGAGGTAGTAGGTTGTATGGTTAT	miRNA	let-7c
t0034621	25	3 TGAGGTAGTAGGTTGTATGGTTATA	miRNA	let-7b
t0034838	19	3 TGAGGTAGTAGGTTGTGCG	miRNA	let-7b
t0034963	23	3 TGAGGTAGTAGGTTGTGAGGTTA	miRNA	let-7b
t0035115	23	3 TGAGGTAGTAGGTTGTGTGGGTA	miRNA	let-7b
t0035145	23	3 TGAGGTAGTAGGTTGTGGGGTAT	miRNA	let-7b
t0035177	23	3 TGAGGTAGTAGGTTGTGCGGTTG	miRNA	let-7b
t0033158	21	3 CGAGGCAGTAGGTTGTGTGGT	miRNA	let-7b
t0033364	20	3 TGACGTAGTAGGTTGTGTGG	miRNA	let-7b
t0033531	24	3 TGAGGAAGTAGGTTGTGTGGTTAT	miRNA	let-7b
t0033761	23	3 TGAGGTAGTAGGATGTGTGGTTG	miRNA	let-7b
t0033985	23	3 TGAGGTAGTAGGGTGTGTGGTTG	miRNA	let-7b
t0035011	23	3 GAGGTAGTAGGTTGTGTGGTTAA	miRNA	let-7b
t0035034	23	3 TGAGGTAGTAGGTTGTGTGGAAA	miRNA	let-7b
t0033017	23	3 TGAGGTAGTAGGTTGTGGGGTAA	miRNA	let-7b
t0033124	24	3 TGAGGTAGTAGGTTGCGTGGTTAA	miRNA	let-7b
t0033165	19	3 TGAGGTAGGAGGTTGTGTG	miRNA	let-7b
t0033200	23	3 TGAGGTAGGAGGTTGTGTGGTTG	miRNA	let-7b
t0033258	19	3 TGCGGTAGTAGGTTGTGTG	miRNA	let-7b
t0033319	19	3 TGAGGTAGTAGGTTGTGAG	miRNA	let-7b
t0033320	22	3 TGAGGTAGTAGGTTGTGTGGAC	miRNA	let-7b
t0033408	23	3 TAAGGTAGTAGGTTGTGTGGTTT	miRNA	let-7b
t0033550	22	3 TGAGGTAGTGGGTTGTGGGGTT	miRNA	let-7b
t0033717	24	3 TGAGGTAGTAGGTCGTGTGGTTAT	miRNA	let-7b
t0033778	22	3 TGAGGTAGTAAGTTGTGTGGTA	miRNA	let-7b
t0033821	23	3 TGAGGTAGTACGTTGTGTGGTTA	miRNA	let-7b
t0033823	22	3 TGAGGTAGTTGGTTGTGTGGTA	miRNA	let-7b
t0033842	23	3 AGAGGTAGTAGGTTGTGTGGTTA	miRNA	let-7b
t0033844	23	3 TGAGGTAGTAGGTTGTGTGGGAT	miRNA	let-7b
t0033935	23	3 CGAGGTAGTAGGTTGTGTGGTTT	miRNA	let-7b
t0034055	19	3 TGAGGTAGTGGGTTGTGTG	miRNA	let-7b
t0034057	23	3 TGAGCTAGTAGGTTGTGTGGTTT	miRNA	let-7b
t0034145	23	3 TGAGGTAGTAGGTTGTGTGGCTT	miRNA	let-7b
t0034149	23	3 TGAGGTAGTAGGATGTGTGGTAA	miRNA	let-7b
t0034301	24	3 TGAGGTAGTAGGTTGTGCGGTTAG	miRNA	let-7b
t0034311	26	3 TGAGGTAGTAGATTGTGTGGTTTATC	miRNA	let-7b
t0034374	22	3 TGAGGTAGTAGGTTGTGTGGGG	miRNA	let-7b

t0034426	21	3	TGAGGTAGTAGGGTGTGGGGT	miRNA	let-7b
t0034486	23	3	TGAGGTAGTAAGTTGTGTGGTTA	miRNA	let-7b
t0034493	20	3	TGAGCTAGTAGGTTGTGTGG	miRNA	let-7b
t0034551	22	3	TGAGGTAGTAGCTTGTATAGTA	miRNA	let-7-5p
t0034624	22	3	TGAGGTAGTGGGTTGTATAGTA	miRNA	let-7-5p
t0034660	22	3	TGAGGTAGTAGGTTGTCTAGTA	miRNA	let-7-5p
t0034676	23	3	TGAGGTGGTAGGTTGTATAGTTT	miRNA	let-7
t0034720	20	3	TGATGTAGTAGGTTGTATAG	miRNA	let-7
t0034753	19	3	TTAGGTAGTAGGTTGTATA	miRNA	let-7
t0034803	21	3	TGAGGTAGTAGGTTGTAGAGT	miRNA	let-7
t0034903	22	3	TGAGGTAGGAGGGTGTATAGTT	miRNA	let-7
t0034982	22	3	TGAGGTAGTAGGTTTTATAGTT	miRNA	let-7
t0035019	19	3	TGAGGTCGTAGGTTGTATA	miRNA	let-7
t0035020	23	3	TGAGGTCGTAGGTTGTATAGTTA	miRNA	let-7
t0035049	19	3	TAAGGTAGTAGGTTGTATA	miRNA	let-7
t0035182	24	2	TGAACTGATAGATGCTCGGCTTTT	miRNA	miR-996
t0033273	20	2	CTATACAACTTACTACTTTT	miRNA	miR-98*
t0034413	19	2	TATACAACTTACTACTTTT	miRNA	miR-98*
t0034631	20	2	TGAGGGAGTAAGTTGTATTG	miRNA	miR-98
t0033073	22	2	CACCCGGCTGTGGGCACATGTG	miRNA	miR-941
t0033540	22	2	CAAAGCGCTGTTCGTGCAGGTA	miRNA	miR-93
t0033658	21	2	CAAAGTGCTGTTAGTGCAGGT	miRNA	miR-93
t0033988	22	2	CAAAGTGCTGTTCGAGCAGGTA	miRNA	miR-93
t0034189	23	2	CAAAGTGCTATTCGTGCAGGTAG	miRNA	miR-93
t0034246	23	2	CAAAGTGCTGTTTGTGCAGGTAG	miRNA	miR-93
t0034519	21	2	CAAAGTGCCGTTCGTGCAGGT	miRNA	miR-93
t0034694	22	2	CAAAGTACTGTTCGTGCAGGTA	miRNA	miR-93
t0034949	21	2	AAAGTGCTGTTCGTGCAGGGA	miRNA	miR-93
t0036759	22	2	CGAAGTGCTGTTCGTGCAGGTA	miRNA	miR-93
t0037815	21	2	CAAAGAGCTGTTCGTGCAGGT	miRNA	miR-93
t0038061	21	2	CGAAGTGCTGTTCGTGCAGGT	miRNA	miR-93
t0039573	23	2	CAAAGCGCTGTTCGTGCAGGTAG	miRNA	miR-93
t0038088	22	2	CAAAGTGCTGTTAGTGCAGGTA	miRNA	miR-93
t0035308	23	2	CAAAGTGCTGTTCGTGCAGGTCT	miRNA	miR-93
t0035939	23	2	CAAAGTGATGTTCGTGCAGGTAG	miRNA	miR-93
t0035986	21	2	CAAAGTGCTGTTCGTGTAGGT	miRNA	miR-93
t0036085	21	2	CAAAGTGCTGTTCGTGCAGGA	miRNA	miR-93
t0036105	21	2	CAAAGCGCTGTTCGTGCAGGT	miRNA	miR-93
t0036538	23	2	CAAGGTGCTGTTCGTGCAGGTAG	miRNA	miR-93
t0037064	23	2	CAAAGGGCTGTTCGTGCAGGTAG	miRNA	miR-93
t0037221	23	2	TATTGTACTTGTCCCGGCCTGTA	miRNA	miR-92d
t0037251	22	2	AATTGCACTTGTCCCGGCCTGT	miRNA	miR-92c
t0037835	23	2	TATTGCACTTGTCCCGGCCTGCA	miRNA	miR-92b-3p
t0037876	22	2	TATCGCACTTGTCCCGGCCTGA	miRNA	miR-92b-3p
t0038000	22	2	ATTGCACTTGTCCCGGCCTGTC	miRNA	miR-92b-3p
t0038090	22	2	TATTGCACTTGTACCGGCCTGA	miRNA	miR-92b-3p
t0038538	22	2	TATTGCACTTGTCACGGCCTGA	miRNA	miR-92b-3p
t0039486	22	2	TATTGCACTTGTTCCGGCCTGA	miRNA	miR-92b-3p
t0039972	20	2	AGGGACGGGCCGCGGTGCAG	miRNA	miR-92b*
t0039984	21	2	AGGGACGGGATGCGGTGCAGT	miRNA	miR-92b*
t0040086	20	2	AGGGACGGGACGCGGAGCAG	miRNA	miR-92b*
t0040100	21	2	AGGGACGGGACGCGGTGCAGG	miRNA	miR-92b*
t0040167	22	2	TATTGCACTTGTCCCGGCTTTT	miRNA	miR-92b
t0035855	21	2	TATTGCACTTGTCCCGGCTTT	miRNA	miR-92b
t0039851	22	2	CATTGCACTTGTCCCGGTCTGT	miRNA	miR-92a-3p
t0036668	25	2	TATTGCACTTGTCCCGGCCTGTATC	miRNA	miR-92a
t0037145	23	2	TATTGCACTTGTTCCGGCCTGTA	miRNA	miR-92a
t0037290	21	2	TATTGCACTTGTCCCGGCCCG	miRNA	miR-92a

t0037753	21	2	TATTGCACTTGTCCCGCCTG	miRNA	miR-92a
t0039260	23	2	TATTGCACTTGTCCCAGCCTGTA	miRNA	miR-92a
t0040430	22	2	TATTGCACTTGTCCCGGCCTCT	miRNA	miR-92a
t0035673	22	2	TATTGCACTTGACCCGGCCTGT	miRNA	miR-92a
t0037786	21	2	TATTGCACTTGTCAAGGCCTG	miRNA	miR-92a
t0039960	23	2	TATTGCACTTGTCACGGCCTGTA	miRNA	miR-92a
t0040573	23	2	TATTGCACTTGTCCAGGCCTGTA	miRNA	miR-92a
t0035734	22	2	TATTGGACTTGTCCCGGCCTGT	miRNA	miR-92a
t0037093	23	2	TATTGCACTTGTCCAGGCCTGTT	miRNA	miR-92a
t0039971	22	2	TATTGCGCTTGTCCCGGCCTGT	miRNA	miR-92a
t0035618	20	2	TATTGCACTAGTCCCGGCCT	miRNA	miR-92a
t0036006	21	2	TATTGCCCTTGTCCCGGCCTG	miRNA	miR-92a
t0036231	22	2	TATCGCACTTGTCCCGGCCTGT	miRNA	miR-92a
t0036880	22	2	TATTGAACTTGTCCCGGCCTGT	miRNA	miR-92a
t0037058	21	2	TATTGCACTAGTCCCGGCCTG	miRNA	miR-92a
t0037667	21	2	TATTGCACTTGTCGCGGCCTG	miRNA	miR-92a
t0038058	21	2	TAATGCACTTGTCCCGGCCTG	miRNA	miR-92a
t0038134	21	2	TATTGCACTTGTCCTGGCCTG	miRNA	miR-92a
t0038186	21	2	TATTGCACTTGTCCCGGACTA	miRNA	miR-92a
t0038215	23	2	TATCGCACTTGTCCCGGCCTGTT	miRNA	miR-92a
t0038347	22	2	TATTGCACTTGCCCCGGCCTGT	miRNA	miR-92a
t0038469	21	2	TATTGAACTTGTCCCGGCCTG	miRNA	miR-92a
t0038482	22	2	TATTGCACCTGTCCCGGCCTGT	miRNA	miR-92a
t0038611	22	2	TATTGCACTTGGCCCGGCCTGT	miRNA	miR-92a
t0038622	21	2	TATTGGACTTGTCCCGGCCTG	miRNA	miR-92a
t0038706	21	2	TATTGCAATCGTCCCGGCCTA	miRNA	miR-92
t0038766	20	2	TATTGAACTCGTCCCGGCCT	miRNA	miR-92
t0038898	20	2	TATTGCACTCGTCACGGCCT	miRNA	miR-92
t0038931	22	2	GTAGAGGAGATGGAGCAGGGGA	miRNA	miR-877
t0039208	23	2	GTAGAGGAGATGGAGCAGGGGAA	miRNA	miR-877
t0039413	23	2	TGGAGAGAAAGGAAGTTCATGAA	miRNA	miR-765
t0039622	23	2	TGGAAGACTGGGGATATGTTGTT	miRNA	miR-7-5p
t0039734	26	2	AAGTTGGATTGTGACTGGCACCTTTT	miRNA	miR-753c
t0039870	27	2	AAGTTGGATTGTGACTGGCAAGGGGAA	miRNA	miR-753c
t0039958	22	2	TGCGGGGCTAGGGCTAACAGCC	miRNA	miR-744
t0040331	22	2	TGCGGGGCTAGGGCTAACAGAA	miRNA	miR-744
t0040482	22	2	TGCGGGGCTAGGGCTAAAAGCA	miRNA	miR-744
t0040495	23	2	GAAGACTGAAGTGGAGGAGGGTT	miRNA	miR-739
t0036775	23	2	TGAAGTCTGAAGTGGAGAAGGGT	miRNA	miR-739
t0037512	23	2	TGAGGACTGAAGTGGAGAAGGGT	miRNA	miR-739
t0038216	24	2	GAAGACTGAAGAGGAGAAGGGTTT	miRNA	miR-739
t0035339	23	2	GAAGACTGAAGGGGAGAAGGGTT	miRNA	miR-739
t0039741	24	2	TGAAAGACGATGGTAGTGAGATGA	miRNA	miR-71
t0039583	23	2	TGAAAGACGATGGTAGTGAGATA	miRNA	miR-71
t0039330	26	2	AGGGCGCGCGGGTCGGGGCGGCGGGT	miRNA	miR-638
t0035642	23	2	AGGGCGCGCGGGTCGGGGAGGCG	miRNA	miR-638
t0037695	21	2	TGGGTTTACGTTGGGAGCACT	miRNA	miR-629
t0036521	18	2	TGGGTTTACGTTGGGGGA	miRNA	miR-629
t0037080	20	2	TGGGTTTACGTTGGGAGCAC	miRNA	miR-629
t0038384	20	2	TCTAGTAAGAGTGGCAGTCG	miRNA	miR-628-3p
t0037605	22	2	TATGTCATCGTTGTCATCGTCA	miRNA	miR-598
t0038077	22	2	TACGTCATCGTTGTCATCGTAA	miRNA	miR-598
t0039706	22	2	ATTATGGTTTGCCTGGGGCTGA	miRNA	miR-584
t0040374	22	2	TTATGGTTTGCCTGGGACTGAA	miRNA	miR-584
t0040413	21	2	TTATGGTTTGCCTTGGACTGA	miRNA	miR-584
t0036319	21	2	AAAAGTAATTGCGGTTTTTGC	miRNA	miR-548c-5p
t0039872	22	2	TCTCCCACACCCAAGGCTTGCC	miRNA	miR-532-3p
t0036661	22	2	CCTCCCAAACCCAAGGCTTGCA	miRNA	miR-532-3p

t0037074	22	2	CCTCCCACACCCAAGGCTTGGA	miRNA	miR-532-3p
t0037937	22	2	CCTCCCACACACAAGGCTTGCA	miRNA	miR-532-3p
t0038360	22	2	CCTCACACACCCAAGGCTTGCA	miRNA	miR-532-3p
t0038914	22	2	CCTCCCACACCAAAGGCTTGCA	miRNA	miR-532-3p
t0039976	22	2	TCTGGGCACAGGCGGGTGGACA	miRNA	miR-5107
t0039308	22	2	TCTGGGCCCAGGCGGATGGACA	miRNA	miR-5107
t0040644	23	2	TGGGCACAGGCGGATGGACAGGA	miRNA	miR-5107
t0037717	23	2	TGGGCACAGGCGGATGGACAGGG	miRNA	miR-5107
t0038068	22	2	TCTGGGCACAGGCGGCTGGACA	miRNA	miR-5107
t0039707	20	2	TAGCAGCGGGAACAATTCTG	miRNA	miR-503
t0036115	23	2	TCCTGTGCTGAGCTGCCCCGAGT	miRNA	miR-486-5p
t0035206	22	2	TCCTGTACTGAGCTGCTCCGCG	miRNA	miR-486-5p
t0037114	23	2	TCCTGTACTGAGCTGCTCCGAGT	miRNA	miR-486-5p
t0038143	21	2	TCCTGTACTGAGCTGACACGA	miRNA	miR-486-5p
t0038197	22	2	TCCTGTACTGAGATGCCCTGAG	miRNA	miR-486-5p
t0039224	23	2	TCCCGTACTGAGCTGCCCCGAGT	miRNA	miR-486-5p
t0039751	23	2	TCCTGTACTGGGCTGCCCCGAGT	miRNA	miR-486-5p
t0035281	24	2	TCCTGTACTGAGCTGCCCCGAGCA	miRNA	miR-486-5p
t0037316	22	2	TCCTGTACTGAGCTGCCACGAA	miRNA	miR-486-5p
t0038071	21	2	TCCTGTACTGAGCTGACCAGA	miRNA	miR-486-5p
t0038127	22	2	TCCTGTACTGAGATGCCCCGCG	miRNA	miR-486-5p
t0040435	21	2	TCCTGTACAGAGCTGCCCCGA	miRNA	miR-486-5p
t0035708	22	2	TCCTGTACTGAGCCGCCCCGAA	miRNA	miR-486-5p
t0035319	20	2	TCCTGTACTGACCTGCCCCG	miRNA	miR-486-5p
t0035444	26	2	TCCTGTACTGAGCTGCACCGAGAAGA	miRNA	miR-486-5p
t0035456	23	2	TCCTGTACTGAGCTGCCTCGAGT	miRNA	miR-486-5p
t0035492	22	2	TCCTGTAATGAGCTGCCCAGAG	miRNA	miR-486-5p
t0035535	21	2	TCCTGTACTGAGATGACCCGA	miRNA	miR-486-5p
t0035537	24	2	TCCTGCACTGAGCTGCCCCGAGAG	miRNA	miR-486-5p
t0035574	26	2	TCCTGTACTGAGCTGCCCCGCGATGG	miRNA	miR-486-5p
t0035686	19	2	TCCGTACTGAGCTGCCCCG	miRNA	miR-486-5p
t0035776	23	2	TCCTGTAATGAGCTGCCACGAGA	miRNA	miR-486-5p
t0035951	23	2	TCCTGTACTGAGCTGCTCCGCGA	miRNA	miR-486-5p
t0036098	28	2	TCCTGTACTGAGCTGCCCCGCGACGGCG	miRNA	miR-486-5p
t0036177	23	2	TCCTGTACTGAACTGCCCCGAGT	miRNA	miR-486-5p
t0036266	21	2	TTCTGTACTGAGCTGACCCGA	miRNA	miR-486-5p
t0036289	21	2	TCCTGTATTGAGCTGCCCCGC	miRNA	miR-486-5p
t0036472	28	2	TCCTGTACTGAGCTGCTCCGAGACGGCA	miRNA	miR-486-5p
t0036531	22	2	CCTGTACTGAGCTGCCCCGAGG	miRNA	miR-486-5p
t0036595	20	2	TCCTGTACTGAGCTCCCCCG	miRNA	miR-486-5p
t0036646	21	2	TCCTGTACTGAGATGCACCGA	miRNA	miR-486-5p
t0036660	20	2	TCCTGTACTGAGCTGCCGCG	miRNA	miR-486-5p
t0036752	23	2	TCCTGTACTGAGCTGCCCCGAAC	miRNA	miR-486-5p
t0036781	21	2	TACTGTAATGAGCTGCCCCGA	miRNA	miR-486-5p
t0036792	24	2	TCCTGTACTGAGCTGCCCCGCGAT	miRNA	miR-486-5p
t0036936	23	2	TCCTGTACTCAGCTGCCCCGAGA	miRNA	miR-486-5p
t0036956	23	2	TCCTGTACTGAGCTGCCCAGAGT	miRNA	miR-486-5p
t0036957	21	2	TCCTGTACTGAGTTGCCCCGC	miRNA	miR-486-5p
t0037102	21	2	TCATGTACTGAGATGCCCCGA	miRNA	miR-486-5p
t0037271	28	2	TCCTGTACTGAGCTACCCCGAGACGGCA	miRNA	miR-486-5p
t0037277	23	2	TCGTGTACTGAGCTGCCCCGAGA	miRNA	miR-486-5p
t0037318	22	2	TCCTGTACTGAGCTGACACGAG	miRNA	miR-486-5p
t0037368	22	2	TCCTGTACTGAGATACCCCGAG	miRNA	miR-486-5p
t0037531	23	2	TCCTGTACTGAGCTGTCCCGCGA	miRNA	miR-486-5p
t0037580	26	2	CCCTGTACTGAGCTGCCCCGAGAAGA	miRNA	miR-486-5p
t0037629	21	2	TCATGTACTGAGCTGCCCAGA	miRNA	miR-486-5p
t0037650	20	2	CTGTACTGAGCTGTCCCGAA	miRNA	miR-486-5p
t0037652	21	2	CCTGTACTGAGCTGCCTCGAG	miRNA	miR-486-5p

t0037715	23	2 CCCTGTACTGAGCTGCCCCGAGA	miRNA	miR-486-5p
t0037796	20	2 TCCTGTGCTGAGCTGCCCCG	miRNA	miR-486-5p
t0037805	23	2 TCCTGTACTGCGCTGCCCCGAGT	miRNA	miR-486-5p
t0037836	29	2 TCCTGTACTGAGCTGCCCCGAGGCCGGC/	miRNA	miR-486-5p
t0037885	24	2 TCCTGTACTGAGCTGCCCCGCAGA	miRNA	miR-486-5p
t0038009	22	2 TCCTATACTGAGCTGCCCCGAA	miRNA	miR-486-5p
t0038019	22	2 TCCTATACTGAGCTGACCCGAG	miRNA	miR-486-5p
t0038038	23	2 TCTTGTACTGAGCTGCCCCGAGG	miRNA	miR-486-5p
t0038055	20	2 TCCTGTACTGAGCTGCTCCG	miRNA	miR-486-5p
t0038160	23	2 TCCTGTACGGAGCTGCCCCGAGA	miRNA	miR-486-5p
t0038266	22	2 TCCTGTATTGAGCTGCCCCGCG	miRNA	miR-486-5p
t0038319	22	2 TCCTGTACTGAGCTGCCCCGTT	miRNA	miR-486-5p
t0038336	23	2 TCTTGTACTGAGCTGCCCCAAGA	miRNA	miR-486-5p
t0038502	20	2 TCCTGTACCGAGCTGCCCCG	miRNA	miR-486-5p
t0038506	23	2 GCCTGTACTGAGCTGCCCCGAGA	miRNA	miR-486-5p
t0038619	22	2 TCCTGTACTGAGCTGCGCCGAA	miRNA	miR-486-5p
t0038651	22	2 TCCTGTACTGAGATGCCCCGAT	miRNA	miR-486-5p
t0038671	21	2 TACAGTACTGAGCTGCCCCGA	miRNA	miR-486-5p
t0038884	22	2 TCCTGTACTGAGCTGCAACGAG	miRNA	miR-486-5p
t0039101	26	2 TCCTGTACTGAGCTGCCCCGAGGCCC	miRNA	miR-486-5p
t0039117	23	2 TCCTGTACTGAGCGGCCCCGAGA	miRNA	miR-486-5p
t0039153	23	2 TCCTGTACTGAGCTTCCCCGAGA	miRNA	miR-486-5p
t0039191	21	2 TCCTGTACTGAACTGCCTCGA	miRNA	miR-486-5p
t0039310	22	2 TAGATGTAGGATTGTGTCGGTT	miRNA	miR-4860
t0039376	25	2 TGTAGGATTGTGTCGGCAGATTTTT	miRNA	miR-4860
t0039659	20	2 AATGTAGAATTGTGTCGGCA	miRNA	miR-4860
t0039665	21	2 TAGGATTGTGTCGGCAGATCT	miRNA	miR-4860
t0039697	24	2 TGTAGGATTGTGTCGGCCGATTTT	miRNA	miR-4860
t0039723	21	2 TGGAGAGCAGGGAGCAGGAAG	miRNA	miR-4732-5p
t0039979	21	2 TGCAGAGCAGGGAGCAGGAAG	miRNA	miR-4732-5p
t0040075	21	2 TGTAGAGCAGGGAGGAGGAAG	miRNA	miR-4732-5p
t0040249	22	2 TGTGGAGCAGGGAGCAGGAAGC	miRNA	miR-4732-5p
t0040297	23	2 TGTAGAGCAGGGCGCAGGAAGCA	miRNA	miR-4732-5p
t0040340	23	2 TGTAGAACAGGGAGCAGGAAGCT	miRNA	miR-4732-5p
t0040533	21	2 TGAAGAGCAGGGAGCAGGAAG	miRNA	miR-4732-5p
t0040574	23	2 TGTAGAGCAGGGAGCACGAAGCT	miRNA	miR-4732-5p
t0037275	23	2 TGTAGAGCAGGGGGCAGGAAGCA	miRNA	miR-4732-5p
t0038254	21	2 TGTAGAGCGGGGAGCAGGAAG	miRNA	miR-4732-5p
t0038882	23	2 TGTAGAGCAGGGAGCCGGAAGCT	miRNA	miR-4732-5p
t0039645	23	2 TGTAGAGCAGGGTGCAGGAAGCT	miRNA	miR-4732-5p
t0040490	21	2 TGTAGAGCAGGGAGCAGGACG	miRNA	miR-4732-5p
t0035494	21	2 TGTAGAGCAGGGAGCGGGAAG	miRNA	miR-4732-5p
t0035632	23	2 TGTAGAGCCGGGAGCAGGAAGCT	miRNA	miR-4732-5p
t0035915	23	2 TGTAGAGCAGGGAGCAGGAATCT	miRNA	miR-4732-5p
t0036365	23	2 TGTAGCGCAGGGAGCAGGAAGCT	miRNA	miR-4732-5p
t0036484	26	2 TGTAGAGCAGGGAGCAGGAAGCTGAG	miRNA	miR-4732-5p
t0036557	21	2 TGTAGAGCAGGAAGCAGGAAG	miRNA	miR-4732-5p
t0036743	21	2 TGTAGAGCAGGGAGTAGGAAG	miRNA	miR-4732-5p
t0036776	21	2 TGTGGAGCAGGGAGCAGGAAG	miRNA	miR-4732-5p
t0037488	23	2 TGTAGAGCAGGGAGCAGGAAGAA	miRNA	miR-4732-5p
t0037560	23	2 TGTAGAGCAGGGAGAAGGAAGAT	miRNA	miR-4732-5p
t0037668	21	2 TGTAGAGCAGGGAGCAAGAAG	miRNA	miR-4732-5p
t0038098	21	2 TGTGGAAGTGGTCTGAGGCAT	miRNA	miR-4657
t0038205	26	2 AGGGCTGGGTCGGGCGGGCTGGGGCG	miRNA	miR-4651
t0038455	28	2 AGGGCTGGGTCGGTCGGGCTGGGGGGC(	miRNA	miR-4651
t0038668	26	2 AGGGCTGGGTCGGTCGGGCTGGGGGG	miRNA	miR-4651
t0038709	30	2 AGGGCTGGGTCGGTCGGGCTGGGGCGCC	miRNA	miR-4651
t0039001	19	2 GGCTGGGTCGGTCGGGCTG	miRNA	miR-4651

t0039265	26	2	AGGGCTGGGTCGGCCGGGCTGGGGCG	miRNA	miR-4651
t0039425	27	2	AGGGCTGGGTCGGTCGGGATGGGGCGC	miRNA	miR-4651
t0039630	23	2	TCGACTCTGGATAGCTGGGCTTT	miRNA	miR-4538
t0040067	22	2	TCGACTCTGGATAGCTGGGCTT	miRNA	miR-4538
t0040228	25	2	AGAAGGGTCGGGGCGGCAGGGGCCG	miRNA	miR-4516
t0040314	19	2	GAAGGGTCGGGGCGGCAGG	miRNA	miR-4516
t0040563	20	2	GGAGAAGGGTCGGGGCGGAT	miRNA	miR-4516
t0037257	20	2	TGAGGGAGTAGGTTGTGTGA	miRNA	miR-4510
t0035856	22	2	TGAGGGAGTAGGTTCTGTGGTT	miRNA	miR-4510
t0035974	23	2	TGAGGTAGTAGGGTGTGTGGTAT	miRNA	miR-4510
t0036253	22	2	TGAGGGAATAGGTTGTGTGGTT	miRNA	miR-4510
t0037602	22	2	TGAGGGAGTAGGTTGAGTGGTT	miRNA	miR-4510
t0038271	23	2	TGAGGGAGTAGGTTGTGTGGGTT	miRNA	miR-4510
t0039169	21	2	TGAGGAAGTAGGGTGTGTGGT	miRNA	miR-4510
t0039882	25	2	TGAGGGAGTAGGTTGTGTGGTTATC	miRNA	miR-4510
t0035526	24	2	TGAGGGAGTAGGTTGTGTGGTATC	miRNA	miR-4510
t0036172	21	2	AAACCGTTTCCATTACTGAGT	miRNA	miR-451
t0037998	24	2	AAAGCGTTACCATTACTGAGTTTA	miRNA	miR-451
t0038301	26	2	AAACCGTTAGCATTACTGAGTTTAGT	miRNA	miR-451
t0040502	23	2	AAACCGTTAACATTAATGAGTTT	miRNA	miR-451
t0035718	22	2	AACCGTTACCATTACTGCGTTT	miRNA	miR-451
t0035980	25	2	AAACCGTTACCATTACGGAGTTTAG	miRNA	miR-451
t0036923	19	2	AAACCGTTACCATTACTCA	miRNA	miR-451
t0038316	23	2	AAACAGTTACCATTAATGAGTTT	miRNA	miR-451
t0038675	25	2	AAACCGTTACCATTACTAAGTTTAG	miRNA	miR-451
t0038860	23	2	AAACCGTTACCATCACTGAGTAA	miRNA	miR-451
t0039093	22	2	AAACCGTTACCATCACTGGGTT	miRNA	miR-451
t0039580	24	2	AAACCGTTACCATTGCTGAGTTTA	miRNA	miR-451
t0040587	19	2	AAACCGTTACCATTACCGA	miRNA	miR-451
t0035219	23	2	AAACCTTTACCATTACTGAGGTT	miRNA	miR-451
t0035256	24	2	AAACCGTTACAATTACTGAGTTTA	miRNA	miR-451
t0035408	22	2	AAACCGTTAAAATTACTGAGTT	miRNA	miR-451
t0035423	21	2	AAACCTTTACCATTATTGAGT	miRNA	miR-451
t0035508	22	2	AAACCGTTACCATGACTGAGTT	miRNA	miR-451
t0035717	24	2	AAACCGTTACCACTACTGAGTTTA	miRNA	miR-451
t0035743	24	2	CAACCGTTACCATTACTGAGTTTA	miRNA	miR-451
t0035756	19	2	AAACCGGTACCATTACTGA	miRNA	miR-451
t0035761	22	2	AACCGTTACCATTATTGAGTTT	miRNA	miR-451
t0035829	22	2	AAAACGTTACCATTACTGCGTT	miRNA	miR-451
t0035988	24	2	AGACCGTTACCATTACTGAGTTTA	miRNA	miR-451
t0036005	25	2	AGACCGTTACCATTACTGAGTTTAG	miRNA	miR-451
t0036077	19	2	AAAACGTTACCATTACTGA	miRNA	miR-451
t0036165	23	2	ACACCGTTACCATTACTGAGTTT	miRNA	miR-451
t0036192	21	2	AAACCGTTACCATAACTGAGT	miRNA	miR-451
t0036232	24	2	AAACCGTTACCATTACCGAGTTTA	miRNA	miR-451
t0036369	22	2	AAAACGTTACAATTACTGAGTT	miRNA	miR-451
t0036408	24	2	AAACCGTTACCATTACTGAGTTTC	miRNA	miR-451
t0036470	26	2	GAACCGTTACCATTACTGAGTTTAGT	miRNA	miR-451
t0036536	23	2	AAACCGTTCCCATTACTGAGTTT	miRNA	miR-451
t0036563	19	2	AAACCGTTAGCATTACTGA	miRNA	miR-451
t0036575	21	2	AAACCGTTACCATTATTAAGT	miRNA	miR-451
t0036644	24	2	AAACCGTTGCCATTACTGAGTTTA	miRNA	miR-451
t0036657	22	2	AAACCGATACCATTATTGAGTT	miRNA	miR-451
t0036673	22	2	AAACATTACCATTACTGAGTTT	miRNA	miR-451
t0036679	24	2	AAATCGTTACCATTACTGAGTTTA	miRNA	miR-451
t0036725	25	2	AAACTGTTACCATTACTGAGTTTAG	miRNA	miR-451
t0036773	24	2	AAACCGTTACCATTACAGAGTTTA	miRNA	miR-451
t0036780	21	2	AAACCGTTACCAGTACTGAGT	miRNA	miR-451

t0036808	25	2	AAGCCGTTACCATTACTGAGTTTAG	miRNA	miR-451
t0036860	21	2	AAACCTTTACCATTACTGAGA	miRNA	miR-451
t0036998	26	2	AAACCGTTACCATTACTGCGTTTAGT	miRNA	miR-451
t0037121	19	2	AGACCGTTACCATTACTGA	miRNA	miR-451
t0037164	23	2	AAACCTTTACAATTACTGAGTTT	miRNA	miR-451
t0037167	24	2	AAACCGTTACCATCACTGAGTTTA	miRNA	miR-451
t0037236	20	2	AAACCGTTCCCATTACTGAG	miRNA	miR-451
t0037265	22	2	AAACTGTTACCATTATTGAGTT	miRNA	miR-451
t0037273	25	2	AAACCGATACCATTACTGAGTTTAG	miRNA	miR-451
t0037375	21	2	AAACCGTTACCCTTACTGCGT	miRNA	miR-451
t0037379	23	2	AAACCGTTACCATAACTGAGTTT	miRNA	miR-451
t0037389	20	2	AAACCGTTAACATTAATGAG	miRNA	miR-451
t0037468	23	2	AAACCTTTAGCATTACTGAGTTT	miRNA	miR-451
t0037506	23	2	AAACCGTTACCATTACTGAGTGT	miRNA	miR-451
t0037507	21	2	AAACCTTTACCATTACTGCGT	miRNA	miR-451
t0037571	22	2	AAACCCTTACCATTACTGAGAT	miRNA	miR-451
t0037609	19	2	AAACCGTTACCATTGCTGA	miRNA	miR-451
t0037653	25	2	AAACCGTTACCATTACTGGGTTTAA	miRNA	miR-451
t0037670	26	2	AAACCATTACCATTACTGAGTTTAGT	miRNA	miR-451
t0037675	24	2	AAACCGTTACCATTACTGAGTCTA	miRNA	miR-451
t0037845	23	2	AAACCGTTACCAGTACTGAGTTT	miRNA	miR-451
t0037882	26	2	AAACCGTTACCATTAATGAGTTTAGT	miRNA	miR-451
t0038029	26	2	AAACCGTTACCATTACCGAGTTTAGT	miRNA	miR-451
t0038056	22	2	AAACAGTTACCATTACTCAGTT	miRNA	miR-451
t0038131	22	2	AAACCTTTACCATTACTGCGTT	miRNA	miR-451
t0038166	25	2	AAACCGTTACCCTTACTGAGTTTAG	miRNA	miR-451
t0038206	19	2	AAACCGTTATTATTACTGA	miRNA	miR-451
t0038253	21	2	AAACCGTCACCATTATTGAGT	miRNA	miR-451
t0038281	21	2	AAACCGTTAACATTAATGAGT	miRNA	miR-451
t0038297	21	2	AAACAGTTAACATTACTGAGT	miRNA	miR-451
t0038314	23	2	AAACCGTTACCATTACTGAGTCA	miRNA	miR-451
t0038379	25	2	AAACCGTTACCATTGCTGAGTTTAG	miRNA	miR-451
t0038498	24	2	GAACCGTTACCATTACTGAGTTTA	miRNA	miR-451
t0038525	21	2	AAAAAGTTACCATTACTGAGT	miRNA	miR-451
t0038537	22	2	AAACAGTTACCATTAATGAGTT	miRNA	miR-451
t0038734	22	2	AAACCGTTAACATTACTCAGTT	miRNA	miR-451
t0038793	21	2	AAACCGTTACCATTATTGAGC	miRNA	miR-451
t0038857	19	2	AAACCGTTACCATTATTGA	miRNA	miR-451
t0038872	25	2	AAACCGTTACCATTAATGAGTTTAG	miRNA	miR-451
t0038940	20	2	AAACCGTTACAATTAATGAG	miRNA	miR-451
t0038954	27	2	AAAAAAACCGTTACCATTACTGAGTTT	miRNA	miR-451
t0039005	21	2	AAACCTTTACCATTAATGAGT	miRNA	miR-451
t0039162	20	2	ATACCGTTACCATTACTGAG	miRNA	miR-451
t0039165	22	2	AACCGTTACCATTACTGAGGTT	miRNA	miR-451
t0039195	24	2	AAACCGTTACCATTACTGAGCTTA	miRNA	miR-451
t0039275	25	2	AAACCGTCACCATTACTGAGTTTAG	miRNA	miR-451
t0039403	20	2	AATCCGTTACCATTACTGAG	miRNA	miR-451
t0039415	20	2	AAACCGTTACCAGTACTGAG	miRNA	miR-451
t0039520	21	2	AAACCGTTGCCATTATTGAGT	miRNA	miR-451
t0039614	23	2	AACCCGTTACCATTACTGAGTTT	miRNA	miR-451
t0039681	21	2	ATACCGTTACCATTACTGAGT	miRNA	miR-451
t0039719	19	2	AAACCGTTGCCATTACTGA	miRNA	miR-451
t0039730	26	2	AAACCGTTACCATTACTGTGTTTAGT	miRNA	miR-451
t0039809	22	2	AAACCGTTACCAGTACTGAGTT	miRNA	miR-451
t0039897	23	2	AAACCGTTAACATTACTGAGTAA	miRNA	miR-451
t0039952	23	2	TTCGGGGTCTGGGCGCGGCGAGG	miRNA	miR-4508
t0040027	22	2	TTCGGGGTCTGGGCGCGGCGCT	miRNA	miR-4508
t0040029	20	2	TTCGGGGGCTGGGCGCGGCG	miRNA	miR-4508

t0040196	20	2 TTCGGGGTCTGGGAGCGGCG	miRNA	miR-4508
t0040238	20	2 GGCACGGGCTGGGCGAGGCT	miRNA	miR-4486
t0040246	21	2 GGGTGCGGGCCGGCGGGGTTT	miRNA	miR-4466
t0040278	19	2 GGGTGCGGGCCGGCGGGGG	miRNA	miR-4466
t0040311	22	2 TCAAATCCGAGTCACGGCACCA	miRNA	miR-4454
t0040432	22	2 AGTAGCATTGTACAGGGCTATT	miRNA	miR-4289
t0040508	21	2 AGAAGCATTGTACAGGGTTAT	miRNA	miR-4289
t0040519	22	2 AGCGGCATTGTACAGGGCTATT	miRNA	miR-4289
t0040577	21	2 AGAAGCATTGTACAGGGATAT	miRNA	miR-4289
t0040595	20	2 CGCAGCATTGTACAGGGCTA	miRNA	miR-4289
t0035647	20	2 AGAAGCATTGGACAGGGCTA	miRNA	miR-4289
t0035778	20	2 AGAAGCATTGTACAGGGTTA	miRNA	miR-4289
t0039233	18	2 AGAAGCATTGTACAGGGC	miRNA	miR-4289
t0040128	22	2 ATGACACGATCACTCCCGTTGC	miRNA	miR-425-5p
t0038148	21	2 AAGCAGCAATTCATGTTTTGA	miRNA	miR-424
t0035369	21	2 CGGCAGCAATTCATGTTTTGA	miRNA	miR-424
t0035519	20	2 CAGCAGCGATTCATGTTTTG	miRNA	miR-424
t0038388	20	2 CAGCAGCAATTCATGTTTTC	miRNA	miR-424
t0037022	23	2 TGAGGGGCAGAGGGCGAGGCTTT	miRNA	miR-423-5p
t0037232	21	2 TGAGGGGCAGAGAGAGAGATT	miRNA	miR-423-5p
t0037995	20	2 TGAGGGGCAGAAAGCGAGAC	miRNA	miR-423-5p
t0038302	22	2 TGAGTGGCAGAGAGCGAGAATT	miRNA	miR-423-5p
t0038998	19	2 CGAGGGGCAGAGAGCGAGA	miRNA	miR-423-5p
t0039124	23	2 TGAGGGGCAGAGAGCGGGACTAT	miRNA	miR-423-5p
t0039175	21	2 TGAGGGGCAGAGAGAAAGACT	miRNA	miR-423-5p
t0040330	23	2 TGAGGGGCAGAGCGCGAGAATTT	miRNA	miR-423-5p
t0039402	21	2 TGAGGGGCAGAGAGCAAGATT	miRNA	miR-423-5p
t0038054	23	2 TGAGGGGCAGACAGCGAGAATTT	miRNA	miR-423-5p
t0038487	23	2 TGAGGGGCAGAGAACGAGACTTG	miRNA	miR-423-5p
t0039690	19	2 TGAGGGGCAAAGAGCGAGA	miRNA	miR-423-5p
t0039966	24	2 TGAGGGGCAGAGAGCGAGACTTCT	miRNA	miR-423-5p
t0035261	23	2 TGAGGGGGAGAGAGCGAGAATTT	miRNA	miR-423-5p
t0035469	23	2 TGAGAGGCAGAGAGCGAGAATTT	miRNA	miR-423-5p
t0035489	21	2 TGAGCGGCAGAGAGCGAGCCT	miRNA	miR-423-5p
t0035564	20	2 TGAGGGGCAGAGGGCGAGAC	miRNA	miR-423-5p
t0035601	23	2 TGAGGGGCAGAGAGCGCGAATTT	miRNA	miR-423-5p
t0035652	24	2 TGATGGGCAGAGAGCGAGACTTTT	miRNA	miR-423-5p
t0035663	24	2 TGAGGGGCAGAGAGCGAGACTCTA	miRNA	miR-423-5p
t0035748	21	2 TGGGGGCAGAGAGCGAGACTT	miRNA	miR-423-5p
t0035833	24	2 TGAGGGGCAGAGAGCGAGACTTTC	miRNA	miR-423-5p
t0035902	24	2 TGAGGGGCAGAGAGCGGGACTTTA	miRNA	miR-423-5p
t0036102	23	2 AGAGGGGCAGAGAGCGAGAATTT	miRNA	miR-423-5p
t0036181	24	2 TGAGGGGCAGAGAGCGGGACTTAA	miRNA	miR-423-5p
t0036191	19	2 TGTGGGGCAGAGAGCGAGA	miRNA	miR-423-5p
t0036309	23	2 TGAGGGGCAGAGAGCGAGCTTTT	miRNA	miR-423-5p
t0036419	19	2 TGAGGGGCAGAGAGTGAGA	miRNA	miR-423-5p
t0036546	23	2 TGCGGGGCAGAGAGAGAGACTTT	miRNA	miR-423-5p
t0036728	24	2 TGAGGGGCAGAGAGCGAGGCTTAA	miRNA	miR-423-5p
t0036783	21	2 TGAGGGGCAGAGAGCGCGAAT	miRNA	miR-423-5p
t0036835	21	2 TGAGGGGCAGAGAGCGAGGCG	miRNA	miR-423-5p
t0036940	23	2 TGAGGGGCAGCGCGCGAGACTTT	miRNA	miR-423-5p
t0036997	23	2 TGAGGTGCAGAGAGAGAGACTTT	miRNA	miR-423-5p
t0037006	24	2 GGAGGGGCAGAGAGCGAGACTTTT	miRNA	miR-423-5p
t0037024	23	2 TGAGGGGCAGAGAGCGAGACCTA	miRNA	miR-423-5p
t0037177	23	2 TGAGGGGTAGAGAGCGAGAATTT	miRNA	miR-423-5p
t0037180	20	2 AGAGGGGCAGAGAGCGAGAC	miRNA	miR-423-5p
t0037203	24	2 TGAGGGGCAGAGAACGAGACTTTT	miRNA	miR-423-5p
t0037206	21	2 TGAGGGGCAGAGATCGGGACT	miRNA	miR-423-5p

t0037543	21	2	TGAGGGGTAGAGAGCGAGAAT	miRNA	miR-423-5p
t0037561	20	2	TGAGGGGCAGGGAGCGAGAC	miRNA	miR-423-5p
t0037666	20	2	TGAGGGGCAGAGAGCAAGAC	miRNA	miR-423-5p
t0037699	19	2	TGAGGGGCAGCGAGCGAGA	miRNA	miR-423-5p
t0037721	24	2	TGAGGGGCGGAGAGCGAGACTTTA	miRNA	miR-423-5p
t0037768	23	2	TGAGGGGCAGAGAGCCAGACTTA	miRNA	miR-423-5p
t0037898	21	2	TGAGGGGCAGAGAGCGAGCCA	miRNA	miR-423-5p
t0037956	24	2	TGAGGGGCAGAGAGCGAGAATTTA	miRNA	miR-423-5p
t0037969	19	2	TGAGGAGCAGAGAGCGAGA	miRNA	miR-423-5p
t0038014	23	2	TGAGGGGCAGAGAGAGAGGCTTT	miRNA	miR-423-5p
t0038123	23	2	TGAGGGGCAGAGGGAGAGACTTT	miRNA	miR-423-5p
t0038132	23	2	TGAGGGGCAGCGAGCGAGAATTT	miRNA	miR-423-5p
t0038194	24	2	TGAGGGGAAGAGAGCGAGAATTTT	miRNA	miR-423-5p
t0038315	24	2	TGAGGGGCAGATAGCGAGACTTTA	miRNA	miR-423-5p
t0038365	20	2	TGAGGGGCAAAGAGCGAGAC	miRNA	miR-423-5p
t0038503	21	2	TGAGGGGCAGAGCGCGCGACT	miRNA	miR-423-5p
t0038554	24	2	TGAGGGGCAGAGAGCGAGAATTAA	miRNA	miR-423-5p
t0038724	23	2	TGAGGGGTAGAGAGTGAGACTTT	miRNA	miR-423-5p
t0038748	20	2	TGAGGGGCAGAGAGCGACAC	miRNA	miR-423-5p
t0038885	21	2	TGAGGGGCAGAGAGCGAGCAT	miRNA	miR-423-5p
t0038929	19	2	TGAGGGGGAGAGAGCGAGA	miRNA	miR-423-5p
t0039196	24	2	TGAGGGGTAGAGAGCGAGACTTTT	miRNA	miR-423-5p
t0039263	24	2	TGAGGGGCAGAAAGCGAGACTTTA	miRNA	miR-423-5p
t0039380	21	2	TGAGGGGCAGAGCGCGAGATT	miRNA	miR-423-5p
t0039388	22	2	TGAGGGGCAGAGAGAGAGTCTT	miRNA	miR-423-5p
t0039416	24	2	TGAGGGGCAGAGAGAGAGAATTTT	miRNA	miR-423-5p
t0039440	23	2	TGAGAGGCAGAGAGCGAGATTTT	miRNA	miR-423-5p
t0039472	23	2	TGAGGGGCAGAGAACGAGCCTTT	miRNA	miR-423-5p
t0039515	23	2	TGAGGGGCAGAGTGCGAGACTTA	miRNA	miR-423-5p
t0039528	23	2	TGAGGGGCAGAGAGCGAAAATTT	miRNA	miR-423-5p
t0039644	22	2	TGAGGGGCAGAGAGAGGGACTT	miRNA	miR-423-5p
t0039679	21	2	TGAGGTGCAGAGAGCGAGACA	miRNA	miR-423-5p
t0039778	24	2	TGAGGTGCAGAGAGCGAGACTTTA	miRNA	miR-423-5p
t0039797	24	2	TGAGGGGCAGAGAACGAGACTTAA	miRNA	miR-423-5p
t0039830	23	2	TGAGGGGCAGCGAGCGAGACTTA	miRNA	miR-423-5p
t0039838	23	2	TGAGGGGCAGAGAGCCAGAATTT	miRNA	miR-423-5p
t0039866	23	2	TGAGGGGCAGAGAGTAAGACTTT	miRNA	miR-423-5p
t0039930	21	2	TGAGGGGCAGATAGAGAGACT	miRNA	miR-423-5p
t0039948	21	2	TGAGGGGCAGAGAGAGAGGCT	miRNA	miR-423-5p
t0039985	24	2	TGAGGGGCAGAGAGCGCGACTTTT	miRNA	miR-423-5p
t0039995	18	2	CGAGGGGCAGAGAGCGAG	miRNA	miR-423-5p
t0040081	22	2	CTGGACTTAGAGTCAGAAGGCA	miRNA	miR-422a
t0040116	23	2	TGGAACTCTGATATTGGCAGGGA	miRNA	miR-4001g-3p
t0040172	22	2	TGGACTCGCAGGAACAGGCCTT	miRNA	miR-4001b-5p
t0040179	21	2	TGGACTCGCAGGAACAGGCCA	miRNA	miR-4001b-5p
t0040224	30	2	AGAACTGAAACGGACAAGGGGAATCCGA	miRNA	miR-3896-3p
t0040266	26	2	GAGCCGGCGGCGGCGGCCAATAGAGC	miRNA	miR-3885-5p
t0040387	23	2	CGGCGGGGGCGGGGGTGGGCGGC	miRNA	miR-3885-5p
t0040409	20	2	GGCGGCGGCGTCGGCGGCGG	miRNA	miR-3885-5p
t0040440	25	2	CGGCGGCGGCGACTCTGGACGAGAG	miRNA	miR-3885-5p
t0040441	20	2	GCGGCGTCGGCGGCGGCGGG	miRNA	miR-3885-5p
t0040539	19	2	GGCGGCGGCGGCGGGGGGG	miRNA	miR-3885-5p
t0040556	25	2	GGGGCCGGCGGCGGCGGCGACTCTG	miRNA	miR-3885-5p
t0040575	22	2	TCGGGGCGGCGGCGGCGGCGGA	miRNA	miR-3885-5p
t0036454	21	2	CGGCGGCGGCGGGGGCGGCGG	miRNA	miR-3885-5p
t0036258	23	2	TCGGGGCGGCGGCGGCGGTGGCG	miRNA	miR-3885-5p
t0036453	22	2	CGGCGGCGTCGGCGGCGGCGGG	miRNA	miR-3885-5p
t0038031	26	2	GGAGGGCGGCGGCGGCGGCGGGGGGG	miRNA	miR-3885-5p

t0040071	24	2	TGGTAGACTATGGAACGGAGGATC	miRNA	miR-379-5p
t0035907	21	2	ACTGGACTTGGAGGCAGAAGG	miRNA	miR-378f
t0036294	23	2	ACTGGACTTGGAGTCAGAAGGGT	miRNA	miR-378f
t0036434	23	2	ACTGGACTTGGAGCCAGAAGGCG	miRNA	miR-378f
t0036513	23	2	ACTGGACTTGGAGTCAGAAGGAG	miRNA	miR-378c
t0036869	22	2	CTGGACTTGGAGTCAGAAGGAA	miRNA	miR-378c
t0037292	22	2	TTGGACTTGGAGTCAGAAGGAA	miRNA	miR-378b
t0037927	23	2	ACTGGACTTGGAGTCAGAAGCCA	miRNA	miR-378
t0039039	23	2	ACTGGACTTGGAGTCAGAAGTCA	miRNA	miR-378
t0039566	25	2	ACTGGGCTTGGAGTCAGAAGGCATC	miRNA	miR-378
t0039779	21	2	ACTGGACTTGGAGTAAGAAGG	miRNA	miR-378
t0040307	22	2	ACTGGACTTGGAGTCAGCAGGC	miRNA	miR-378
t0040510	21	2	ACTGGACTTGGAGTCAAAAGG	miRNA	miR-378
t0036184	25	2	ACTGGACTTGGAGTCAGACGGCATC	miRNA	miR-378
t0036213	25	2	ACTGGACTTGGAGTCAGAAGGCATA	miRNA	miR-378
t0037104	28	2	ACTGTACTTGGAGTCAGAAGGCTATATC	miRNA	miR-378
t0038006	23	2	ACTGGACTTGGAGTCAGCAGGCG	miRNA	miR-378
t0035273	23	2	AATGGACTTGGAGTCAGAAGGCA	miRNA	miR-378
t0037913	23	2	ACTGGACTTGGAGTCACAAGGCA	miRNA	miR-378
t0035556	26	2	CTGGACTTGGAGTCAGAAGGCATATC	miRNA	miR-378
t0035234	22	2	ATATAATACAACCTGCTAGGTG	miRNA	miR-374
t0035382	22	2	ATATAATACAAACTGCTAAGTG	miRNA	miR-374
t0036050	22	2	ATATAATACAACCTGCTGAGTG	miRNA	miR-374
t0037314	20	2	ATATAATACAACCTGATAAG	miRNA	miR-374
t0037903	22	2	ATATAATAAAACCTGCTAAGTG	miRNA	miR-374
t0038258	22	2	ATATAATACAACCTGCTAAGCG	miRNA	miR-374
t0038267	21	2	AGGGACTTTCAGGGGCAGATG	miRNA	miR-365*
t0039353	19	2	AATTGTACGGTATCCATCT	miRNA	miR-363
t0039713	22	2	AATTGCATGGTATCCATCTGTA	miRNA	miR-363
t0039907	22	2	AATTGCACGGAATCCATCTGTA	miRNA	miR-363
t0040084	22	2	TCTCTCGGCTCCTCGCGGCTTT	miRNA	miR-3615
t0040272	25	2	TTGAAGACTGAAGGGGAGAAGGGTT	miRNA	miR-3526
t0040549	26	2	AACTTTGAAGACTGAAGTGGAGCAGG	miRNA	miR-3526
t0037084	27	2	AACTTTGTAGACTGAAGTGGAGAAGGG	miRNA	miR-3526
t0037940	25	2	TTGAAGACCGAAGTGGAGAAGGGTT	miRNA	miR-3526
t0039889	29	2	AACTTTAAAGACTGAAGTGGAGAAGGGT	miRNA	miR-3526
t0040158	22	2	TGAAGACTGAAGTGGAGCAGGG	miRNA	miR-3526
t0040325	27	2	AACTCTGAAGACTGAAGTGGAGAAGGG	miRNA	miR-3526
t0040585	24	2	TGAAGACTGAAGCGGAGAAGGGTT	miRNA	miR-3526
t0040234	28	2	AACCTTGAAGACTGAAGTGGAGAAGGGT	miRNA	miR-3526
t0036284	28	2	AACTTTCAAGACTGAAGTGGAGAAGGGT	miRNA	miR-3526
t0036901	30	2	AACTTTGAAGACTGAAGGGGAGAAGGGT	miRNA	miR-3526
t0040487	20	2	AACTTTGAAGACTGAAGGGG	miRNA	miR-3526
t0039394	22	2	AACTTTGAAGACTGAAGCGGAG	miRNA	miR-3526
t0035367	20	2	AACTTTGAAGGCCGAAGTGG	miRNA	miR-3526
t0035372	22	2	AACTTTGAAGACTGAAGTGGCG	miRNA	miR-3526
t0035392	24	2	CTTTGAAGACTGAAGTGGAGCAGG	miRNA	miR-3526
t0035639	28	2	AACTTTGAAGACTGTAGTGGAGAAGGGT	miRNA	miR-3526
t0035831	27	2	TGAAGACTGAAGGGGAGAAGGGTTTCT	miRNA	miR-3526
t0036260	28	2	AACTTTGAAGACTGAAGTGAAGAAGGGT	miRNA	miR-3526
t0036311	27	2	AACTTTGGAGACTGAAGTGGAGAAGGG	miRNA	miR-3526
t0036474	24	2	CTTGAAGACTGAAGTGGCGAAGGG	miRNA	miR-3526
t0036492	26	2	AACTTTGAAGACTGAAGCGGAGAAGG	miRNA	miR-3526
t0036552	28	2	GACTTTGAAGACTGAAGTGGAGAAGGGT	miRNA	miR-3526
t0036738	28	2	AACTTTGAAGACTGAAGTGGACAAGGGT	miRNA	miR-3526
t0036975	30	2	AACTTTGAAGACTGAAGTGGAGAAGGGG	miRNA	miR-3526
t0037071	27	2	AACCTTGAAGACTGAAGTGGAGAAGGG	miRNA	miR-3526
t0037204	28	2	AACTTTGAAGACTGGAGTGGAGAAGGGT	miRNA	miR-3526

t0037238	24	2	TGAAGACTAAAGTGGAGAAGGGTT	miRNA	miR-3526
t0037305	26	2	TGAAGACTGAAGTGGAGAAGGGGTTC	miRNA	miR-3526
t0037625	28	2	AACTTTGAAGACTGCAGTGGAGAAGGGT	miRNA	miR-3526
t0037735	23	2	TGAAGACTGAAGTGGAGACGGGT	miRNA	miR-3526
t0037936	24	2	TGAAGACTGAAGTGGAGCAGGGTT	miRNA	miR-3526
t0038139	25	2	TGAAGACTGAAGTGGAGAAGGGATT	miRNA	miR-3526
t0038161	24	2	TTGAAGACTGAAGTGGGGAAGGGT	miRNA	miR-3526
t0038180	27	2	AACTTTGAAGACTGAAGTAGAGAAGGG	miRNA	miR-3526
t0038420	25	2	TGAAGACTGAAGCGGAGAAGGGTTT	miRNA	miR-3526
t0038508	24	2	TTGAAGACTGAAGCGGAGAAGGGT	miRNA	miR-3526
t0038759	20	2	TGAAGACTGAAGGGGAGAAG	miRNA	miR-3526
t0038819	26	2	TTGAAGACTGAAGTGGAGAAGGGGTT	miRNA	miR-3526
t0038889	23	2	AGTAGAATCCAGGACGCGCGTTT	miRNA	miR-3503
t0038916	21	2	AGTGGAATCCAGGACGCGCGT	miRNA	miR-3503
t0038978	21	2	AGGAGATCGGTAGTAGATTGT	miRNA	miR-3500
t0039266	21	2	AGGAGATCGGTGGTAGATTGT	miRNA	miR-3500
t0039516	21	2	TCAAAATCGTTGGCAATGGCT	miRNA	miR-3495
t0039536	22	2	GATAGAAGACTGCCGCTCGTTT	miRNA	miR-3494
t0039565	21	2	ATAGAAGACTGCCGCTCGTTT	miRNA	miR-3494
t0039917	21	2	TCGATAGAAGACTGCCGCTCT	miRNA	miR-3494
t0040077	20	2	TGCAGAAATTTCGTCAGACA	miRNA	miR-3492
t0040197	22	2	TCCGTGCTGAGATTTCGTCATT	miRNA	miR-3492
t0040362	20	2	CCTCGAACTGTTGTGGCCAT	miRNA	miR-3487
t0040376	21	2	TCCTCGAACTGTTGTGGCCAA	miRNA	miR-3487
t0040576	25	2	TCTCACACAGAAATCGCACCCGGAT	miRNA	miR-342-3p
t0038925	22	2	CCTCACACAGAAATCGCACCCG	miRNA	miR-342-3p
t0039789	22	2	TCTCGCACAGAAATCGCACCCG	miRNA	miR-342-3p
t0036721	23	2	TCTCACACAGAAATCGCACCCGC	miRNA	miR-342-3p
t0039855	25	2	TCTCACACAGAAATCGCACCCGGCA	miRNA	miR-342-3p
t0040253	24	2	TCTCACACAGAAATCGCCCCCGTC	miRNA	miR-342-3p
t0037237	24	2	TCTCACACAGAAATCGCAACCGTC	miRNA	miR-342-3p
t0039478	25	2	TCTCACACAGAAATAGCACCCGTCT	miRNA	miR-342-3p
t0040005	23	2	TCTTACACAGAAATCGCACCCGT	miRNA	miR-342-3p
t0035739	23	2	TCTCAGACAGAAATCGCACCCGT	miRNA	miR-342-3p
t0039133	23	2	TCACACACAGAAATCGCACCCGT	miRNA	miR-342-3p
t0035309	23	2	TCTCACACAGAAATCGCACACGT	miRNA	miR-342-3p
t0039329	23	2	TCTCACATAGAAATCGCACCCGT	miRNA	miR-342-3p
t0035792	23	2	ACTCACACAGAAATCGCACCCGT	miRNA	miR-342-3p
t0035808	22	2	TCTCACACAGGAATCGCACCCG	miRNA	miR-342-3p
t0036244	22	2	TCTCACACAGAAATCGAACCAG	miRNA	miR-342-3p
t0036881	22	2	TCTCAAACAGAAATCGCACCCG	miRNA	miR-342-3p
t0037023	22	2	TTATAAAGCAATGAGACTGCTT	miRNA	miR-340-5p
t0037171	21	2	TTATAAAGCAATGAGAATGAT	miRNA	miR-340-5p
t0037234	21	2	TTATAAAGCAATGTGACTGAT	miRNA	miR-340-5p
t0037249	24	2	CAGCAGCAATTCATGTTTTGGATC	miRNA	miR-322
t0037600	21	2	AAGACTGAAGTGGAGAAGGGA	miRNA	miR-320e
t0037688	22	2	AAGACTGAAGTGGAGAAGGGGT	miRNA	miR-320e
t0037910	22	2	TAAAGCTGGGTTGAGAGGGCGT	miRNA	miR-320d
t0038342	23	2	TAAAGCTGGGTTGAGAGGGCGTA	miRNA	miR-320d
t0039025	22	2	AAAGCTGGGTTGAGAGGGCGGA	miRNA	miR-320d
t0039676	22	2	AAAAGCTGGTTTGAGAGGGCGC	miRNA	miR-320d
t0039963	23	2	AAAAGCTGGGTTGAGAGGACGTA	miRNA	miR-320d
t0040089	21	2	AAAAGATGGGTTGAGAGGGAG	miRNA	miR-320d
t0040428	23	2	AAAGGCTGGGTTGAGAGGGCGTA	miRNA	miR-320d
t0039217	24	2	AAAAGCTGGGTTGAGAGGGAGACA	miRNA	miR-320d
t0040274	23	2	AAAAGCTGGGTTGCGAGGGAGAA	miRNA	miR-320d
t0040636	22	2	AAAAGCTGGGTTGAGAGGGCTG	miRNA	miR-320d
t0038567	23	2	AAAAGCTGGGTCGAGAGGGCGTT	miRNA	miR-320d

t0036143	22	2	AAAAGATGGGTTGAGAGGGCGG	miRNA	miR-320d
t0039784	22	2	AAAAGCTCGGTTGAGAGGGCGT	miRNA	miR-320d
t0035380	21	2	AAAGCTGGGTTGAGAGGGGGT	miRNA	miR-320d
t0035437	23	2	AAAAGCTGGGTTGAGAGGGAGAC	miRNA	miR-320d
t0035513	23	2	AAAAGCTGGGTTGAGAGGGAGCA	miRNA	miR-320d
t0035825	21	2	AAAGCTGGGTTGAGAGGGCGG	miRNA	miR-320d
t0035996	22	2	AAAGCTGGGATGAGAGGGCGTA	miRNA	miR-320d
t0036113	23	2	AAAAGCTGGGTTGAGTGGGCGTT	miRNA	miR-320d
t0036203	22	2	AAAAGTTGGGTTGAGAGGGAGA	miRNA	miR-320d
t0036267	22	2	AAAAGCTGGGTTGAAAGGGAGA	miRNA	miR-320d
t0036270	24	2	AAAAGCTGGGTTGAGAGGGCGCGA	miRNA	miR-320d
t0036444	24	2	AAAAGCTGGGTTGAGAGGGAGAAG	miRNA	miR-320d
t0036621	22	2	AAAGCTGGGTTGAGAGGGAGAA	miRNA	miR-320d
t0036667	21	2	AAAAGTTGGGTTGAGGGGCGA	miRNA	miR-320d
t0036794	24	2	AAAAGCTGGGTTGAGAGGGCGCAG	miRNA	miR-320d
t0036827	23	2	AAAAGCTGGGTTGAGAAGGCGTT	miRNA	miR-320d
t0036937	23	2	AAAAGCTGGGTTGAGAGGGCTAG	miRNA	miR-320d
t0036943	22	2	ATAAGCTGGGTTGAGAGGGCGT	miRNA	miR-320d
t0036955	23	2	AAAAGCTGGATTGAGAGGGCGTT	miRNA	miR-320d
t0037350	23	2	AAAAGCTGGGTTGAGATGGCGTA	miRNA	miR-320d
t0037487	23	2	AAAAGCTGGGTTAAGAGGGCGTT	miRNA	miR-320d
t0037582	22	2	AAAAGCTGAGTTGAGAGGGCAT	miRNA	miR-320d
t0037588	22	2	AAAAGCTGGGTTGAGAGGGAGC	miRNA	miR-320d
t0037719	23	2	AAAAGCTGGGTTGAGAGGGCCAA	miRNA	miR-320d
t0037965	22	2	AAAGCTGGGTTGAGAGGGAGTA	miRNA	miR-320d
t0038114	23	2	AAAAGCTGGGTTGTGAGGGCGTT	miRNA	miR-320d
t0038122	23	2	AAAAGCTGGGTTGAGAGGGAATT	miRNA	miR-320d
t0038189	23	2	AAAAGGTGGGTTGAGAGGGCGTA	miRNA	miR-320d
t0038328	22	2	AAAAGCAGGGTTGAGAGGGCGT	miRNA	miR-320d
t0038437	23	2	AAAAGCTGGGTTCAGAGGGCGTA	miRNA	miR-320d
t0038531	21	2	AAAAGCTGGGGTGAGAGGGCA	miRNA	miR-320b
t0038714	21	2	AAAAGCTGGGTTGAGCGGGCA	miRNA	miR-320b
t0038845	22	2	AAAAGCTGGGCTGAGAGGGCAA	miRNA	miR-320b
t0038846	22	2	AAAAGCTGGGTTGAAAGGGCAA	miRNA	miR-320b
t0038947	21	2	AAAAGCTGGGTTGAAAGGGCA	miRNA	miR-320b
t0039066	21	2	AAAAGCTGAGTTGAGAGGGCG	miRNA	miR-320a
t0039135	23	2	AAAAGCTGGCTTGAGAGGGCGAT	miRNA	miR-320a
t0039366	21	2	AAAAGCTGGGTTGAGACGGCG	miRNA	miR-320a
t0039457	21	2	AAAGCTGGGTTGAGCGGGCGA	miRNA	miR-320a
t0039806	23	2	AAAAGCAGGGTTGAGAGGGCGAA	miRNA	miR-320a
t0040061	23	2	AAAATCTGGGTTGAGAGGGCGAA	miRNA	miR-320a
t0040225	21	2	AAAAGCTGGGTTGAGAAGGCG	miRNA	miR-320a
t0040268	23	2	AAAAGCTGGGTTAAGAGGGCGAG	miRNA	miR-320a
t0035701	21	2	AAAGATGGGTTGAGAGGGCGA	miRNA	miR-320a
t0039318	21	2	AAAAGCTGGGTTGAGAGAGCG	miRNA	miR-320a
t0039375	20	2	AAAGCTGGGTTGAGAAGGCG	miRNA	miR-320a
t0040105	23	2	AAAAGATGGGTTGAGAGGGCGAG	miRNA	miR-320a
t0040424	23	2	AAACGCTGGGTTGAGAGGGCGAT	miRNA	miR-320a
t0035737	23	2	AAAAGCTGGGTTGAAAGGGCGAG	miRNA	miR-320a
t0035762	22	2	AAAGCTGGGTTGAGAGGGCGAC	miRNA	miR-320a
t0035882	21	2	AAAAGCTGGGTTGCGAGGGCG	miRNA	miR-320a
t0035990	23	2	AAAAGCTGGGTTGACAGGGCGAA	miRNA	miR-320a
t0036195	22	2	AAAAGCTGGGTTGATAGGGCGA	miRNA	miR-320a
t0036248	23	2	AAAAGGTGGGTTGAGAGGGCGAT	miRNA	miR-320a
t0036397	23	2	AAAAGCTGGGTTGATAGGGCGAA	miRNA	miR-320a
t0036515	20	2	AAGGCTGGGTTGAGAGGGCG	miRNA	miR-320a
t0036600	23	2	AAAAGCGGGGTTGAGAGGGCGAT	miRNA	miR-320a
t0036849	21	2	ACAAGCTGGGTTGAGAGGGCG	miRNA	miR-320a

t0036911	21	2	AAAGCTGGGATGAGAGGGCGA	miRNA	miR-320a
t0037161	20	2	AAAGCTGGGGTGAGAGGGCG	miRNA	miR-320a
t0037307	24	2	AAAAGCTGGGGTGAGAGGGCGAAA	miRNA	miR-320a
t0037372	20	2	AAAGATGGGTTGAGAGGGCG	miRNA	miR-320a
t0037993	23	2	AAAACCTGGGTTGAGAGGGCGAT	miRNA	miR-320a
t0038119	23	2	AATCTGAGAAGGTGCACAAGGTT	miRNA	miR-3200-5p
t0038169	23	2	AATCTGAGAAGGGGCACAAGGTT	miRNA	miR-3200-5p
t0038457	22	2	TCTGGGAGGTTGTAGAAGTGGA	miRNA	miR-3192
t0038646	20	2	TGGAAGGGAGACGGCCAGAG	miRNA	miR-3190
t0038844	21	2	CGGGGAGAGAACGCAGGGACG	miRNA	miR-3175
t0039072	23	2	GGCTGGTCCGAGTGCAGTGGGGA	miRNA	miR-3135b
t0039253	22	2	GGCTGGTCCGAGTGCAGGGGTG	miRNA	miR-3135b
t0039717	25	2	GGCTGGTCCGAGTGCAGTGGTGGTT	miRNA	miR-3135b
t0039962	23	2	GGCTGGTCCGAGGGCAGTGGTGA	miRNA	miR-3135b
t0040270	25	2	GGCTGGTCCGAGTGCAGTGGGGTTT	miRNA	miR-3135b
t0040310	21	2	TGGGACAAACGGACGGTTTTT	miRNA	miR-3122
t0040514	22	2	TATTGCACTTATCCCGGCCTGA	miRNA	miR-311a
t0040547	20	2	TATTGCACTTATCCCGGCCT	miRNA	miR-311a
t0038227	26	2	TGTAAACATCCTTGACTGGACGCATC	miRNA	miR-30e-5p
t0040553	21	2	TGTAAACATCCTACACTCTCC	miRNA	miR-30c
t0037306	21	2	TGTAAACATCATACACTCTCA	miRNA	miR-30c
t0038250	24	2	TGTAAACATCATACACTCTCAGCT	miRNA	miR-30c
t0040611	24	2	TGTAAACATCCTACACTCTCCGCA	miRNA	miR-30c
t0036866	22	2	TGTAAACATCCCACACTCAGCT	miRNA	miR-30b
t0037157	22	2	TGTAAACATCCTACAATCAGCT	miRNA	miR-30b
t0037196	26	2	TGTAAACATCCTCGACTGGAAGCATA	miRNA	miR-30a-5p
t0038406	23	2	GCGGGTCGGGGGGCGGGGCGGGC	miRNA	miR-2981
t0039143	19	2	CGGGTCGGGGGGCGGGGCG	miRNA	miR-2981
t0039964	21	2	CGGCGGCGGGCGGCGGAGGGG	miRNA	miR-2885
t0035387	23	2	CGGTCGGCGGGCGGCGGGGCGGG	miRNA	miR-2885
t0037682	23	2	CGGCGGCAGGCGGCGGGGGGGCC	miRNA	miR-2885
t0039978	20	2	GGCGGATGTAGTCAAGTGGA	miRNA	miR-270
t0035332	20	2	GGCGGATGTAGCCAAGCGGA	miRNA	miR-270
t0038539	20	2	GGCGTATGTAGCCAAGTGGA	miRNA	miR-270
t0038830	20	2	GGCGGATGTAGCCCAGTGGA	miRNA	miR-270
t0039343	22	2	GGCGGATGTAGCCAAGTGGAGA	miRNA	miR-270
t0039152	20	2	GGAGGATGTAGCCAAGTGGA	miRNA	miR-270
t0039199	21	2	TTCAAGTAATTCAGGGTAGGT	miRNA	miR-26b
t0036974	22	2	TTCAAGTAATTCAGGACAGGTT	miRNA	miR-26b
t0037100	21	2	TTCAAGTATTTCAGGATAGGT	miRNA	miR-26b
t0039245	21	2	TTCAAGCAATTCAGGATAGGT	miRNA	miR-26b
t0036574	22	2	TTAAAGTAATTCAGGATAGGTT	miRNA	miR-26b
t0037037	22	2	TTCAAGTATTTCAGGATAGGTT	miRNA	miR-26b
t0038468	21	2	TTCAAGTAATTGAGGATAGGT	miRNA	miR-26b
t0035616	21	2	TTCAAGTAATTCAGGATAGAT	miRNA	miR-26b
t0035709	22	2	TTCAAGTAATTCCGGATAGGTT	miRNA	miR-26b
t0038460	22	2	TTCAAGTAATTCAGGCTAGGTT	miRNA	miR-26b
t0038535	21	2	TTCAAGGAATTCAGGATAGGT	miRNA	miR-26b
t0039453	22	2	TTCAAGTAATTCGGGATAGGTT	miRNA	miR-26b
t0040243	21	2	TTCAAGTAATTCAAGATAGGT	miRNA	miR-26b
t0035420	22	2	TTCAAGTAGTTCAGGATAGGTT	miRNA	miR-26b
t0035555	21	2	TTCAAGTAATTCTGGATAGGT	miRNA	miR-26b
t0036394	21	2	TTCAAGTAATTCAGGCTAGGT	miRNA	miR-26b
t0036703	22	2	TTCAAGTAATTCAGGATGGGTT	miRNA	miR-26b
t0036745	21	2	TTCAAATAATTCAGGATAGGT	miRNA	miR-26b
t0036927	21	2	TTCAAGTAACTCAGGATAGGT	miRNA	miR-26b
t0037112	22	2	TTCAAGTAATCCAGCATAGGCT	miRNA	miR-26a
t0037166	22	2	TTCAAATAATCCAGGATAGGCT	miRNA	miR-26a

t0037920	21	2	TTCAAGTAATCCAGGATAGGT	miRNA	miR-26a
t0038039	22	2	TTGAAGTAATCCAGGATAGGCT	miRNA	miR-26a
t0038440	21	2	TCAAGTAATCCAGGATGGGCT	miRNA	miR-26a
t0039534	22	2	TTCAAGTAGTCCAGGATAGGCT	miRNA	miR-26a
t0039544	21	2	TTCAAGTAATCCATGATAGGC	miRNA	miR-26a
t0039571	21	2	TTCAAGTAATCCAGGATAGAC	miRNA	miR-26a
t0039720	21	2	TTCAAGTAATACAGGATAGGC	miRNA	miR-26a
t0040041	22	2	TCCAAGTAATCCAGGATAGGCA	miRNA	miR-26a
t0040214	25	2	TTCAAGTAATCCAGGATAGGCTATT	miRNA	miR-26a
t0040540	22	2	TTCAAGTAATCCAGGTTAGGCT	miRNA	miR-26a
t0040578	21	2	TTCAAGTAATCCAGGATCGGC	miRNA	miR-26a
t0035222	21	2	TGAGGTAGGAGGGTGTGTGGT	miRNA	miR-265
t0035375	20	2	TGAGGGAGGAGGGTGTGTGG	miRNA	miR-265
t0035661	22	2	TGAGGGAGGAGGGTGTGTGGTT	miRNA	miR-265
t0035688	21	2	AGGCGGAGACTTGGGAAATTG	miRNA	miR-25*
t0035707	21	2	AGGTGGAGACTTGGGCAATTG	miRNA	miR-25*
t0035731	21	2	AGGCGGAGTCTTGGGCAATTG	miRNA	miR-25*
t0035960	23	2	AGGCGGAGACTTGGGAAATTGCT	miRNA	miR-25*
t0036900	21	2	AGGAGGAGACTTGGGCAATTG	miRNA	miR-25*
t0038398	20	2	GGCGGAGACTTGGGCAATTG	miRNA	miR-25*
t0038551	21	2	AGGCGGAGGCTTGGGCAATTG	miRNA	miR-25*
t0039248	22	2	CATTGCATTTGTCTCGGGCTGA	miRNA	miR-25
t0039512	20	2	CATTGAACTTGTCTCGGTCT	miRNA	miR-25
t0040450	22	2	CATTGCACTGGTCTCGGTCTGA	miRNA	miR-25
t0036504	22	2	AATTGCACTTGTCTCGGTCTGC	miRNA	miR-25
t0039108	22	2	CATTGCACTTGTCTAGGTCTGT	miRNA	miR-25
t0040438	23	2	CATTGCACTTGTCTCGGTCTGCA	miRNA	miR-25
t0035937	22	2	CATTGCACTTGTCTCGGTATGT	miRNA	miR-25
t0035975	23	2	CATTGCACTTATCTCGGTCTGAA	miRNA	miR-25
t0036525	21	2	CCTTGCACTTGTCTCGGTCTG	miRNA	miR-25
t0038238	20	2	CATTGCACTTGTCTCGGTAT	miRNA	miR-25
t0039502	19	2	CATTGCACTTGTCTCGGGC	miRNA	miR-25
t0039596	22	2	CATTGAACTTGTCTCGGTCTGT	miRNA	miR-25
t0040065	22	2	CATTGCACTAGTCTCGGTCTGA	miRNA	miR-25
t0035271	22	2	CATTGCACTTGCCTCGGTCTGT	miRNA	miR-25
t0035396	19	2	TTGCACTTGTCTCGGTCTG	miRNA	miR-25
t0035397	23	2	CATTGCACTTGTCTCGGTATGAA	miRNA	miR-25
t0035416	22	2	CATTGCACTTGTCTCGCTCTGA	miRNA	miR-25
t0035544	22	2	GATTGCACTTGTCTCGGTCTGT	miRNA	miR-25
t0035715	22	2	CATTGTACTTGTCTCGGTCTGT	miRNA	miR-25
t0035786	21	2	CATTGCACCTGTCTCGGTCTG	miRNA	miR-25
t0036645	21	2	TGGCTCAGTTCAGCAGGAAAA	miRNA	miR-24b
t0036690	23	2	TGGCTCAGTTTAGCAGGAACAGT	miRNA	miR-24
t0037126	23	2	TGGCTCAGTTCAGCAGGGACAGT	miRNA	miR-24
t0037155	19	2	TGGCTCAGTTCAGCAGGCA	miRNA	miR-24
t0037964	22	2	TGACTCAGTTCAGCAGGAACAG	miRNA	miR-24
t0037990	22	2	TGGCTAAGTTCAGCAGGAACAG	miRNA	miR-24
t0038165	19	2	ATCACATTGCAAGGGATTA	miRNA	miR-23b
t0038326	19	2	ATCACATTGCCGGGGATTA	miRNA	miR-23b
t0038685	19	2	ATCACATTGCCGGGGATTT	miRNA	miR-23a
t0038818	20	2	ATCACATTGCCAGGGCTTTA	miRNA	miR-23a
t0039488	22	2	ATCACATTGCCAGGGATTTCCC	miRNA	miR-23a
t0039757	22	2	ATCACATTGCCAGGGATTTCCG	miRNA	miR-23a
t0040459	19	2	ATCACATTACCAGGGATTT	miRNA	miR-23a
t0036256	19	2	ATCACCTTGCCAGGGATTT	miRNA	miR-23a
t0035675	19	2	ATAACATTGCCAGGGATTT	miRNA	miR-23a
t0036129	19	2	ATCACATTGCCAGGAATTT	miRNA	miR-23a
t0036400	19	2	ATCGCATTGCCAGGGATTT	miRNA	miR-23a

t0037482	19	2	AACACATTGCCAGGGATTT	miRNA	miR-23a
t0038147	19	2	ATGACATTGCCAGGGATTT	miRNA	miR-23a
t0037479	20	2	ATCACATTGCCAGGAATTTC	miRNA	miR-23a
t0038908	19	2	ATCACATTGCCAGGGTTTT	miRNA	miR-23a
t0035353	20	2	AAGCTGCCAGTTGAAGAAAT	miRNA	miR-22-3p
t0035788	21	2	AGCTACATCTGGCTTCTGGGT	miRNA	miR-222
t0036961	21	2	AGCTAAATCTGGCTACTGGGT	miRNA	miR-222
t0036970	23	2	AGCTACATCTGGCTACTGGGGCA	miRNA	miR-222
t0037481	21	2	CAGCTACATTGTCTGCTGGGG	miRNA	miR-221
t0038657	22	2	AGCTACATTGTCTGCTGGGGTA	miRNA	miR-221
t0038878	21	2	GACTGGGGCGGCACATCTGTT	miRNA	miR-219-2-3p
t0039043	21	2	TTGACTGGGGCGGGACATCTG	miRNA	miR-219-2-3p
t0039144	22	2	TGACTGGGGCGGCACATCTGTT	miRNA	miR-219-2-3p
t0039389	23	2	TGACTGGGGCGGGACATCTGTTA	miRNA	miR-219-2-3p
t0039911	25	2	GACTGGGGCGGTACATCTGTTCAAT	miRNA	miR-219-2-3p
t0039969	24	2	TGACTGGGGCGGGACATCTGTTAT	miRNA	miR-219-2-3p
t0040461	22	2	TGATTGTCCATTCGCATTTCTT	miRNA	miR-219
t0040640	23	2	AGTGAGATTGTTGCATATTTACT	miRNA	miR-2162-5p
t0035599	21	2	AGTGAGATTGTTGCATATTTT	miRNA	miR-2162-5p
t0036878	25	2	ATGACCTATGATTTGACAGAAAATC	miRNA	miR-215
t0038596	25	2	ATGACCTATGATTTGACAGACCATC	miRNA	miR-215
t0035251	24	2	TTGGGGAAACGGCCGCTGAGGGAG	miRNA	miR-2110
t0040399	21	2	TAGCTTATCAGAATGATGTTG	miRNA	miR-21
t0035719	21	2	TAGCTTATCAGTCTGATGTTG	miRNA	miR-21
t0036112	21	2	TAGCTTATCAGACTCATGTTG	miRNA	miR-21
t0038823	21	2	TCGCTTATCAGACTGATGTTG	miRNA	miR-21
t0039418	21	2	TAGCTTATTAGACTGATGTTG	miRNA	miR-21
t0040313	21	2	TAGCTTATAAGACTGATGTTG	miRNA	miR-21
t0040434	21	2	TAGCTTATCAGACTGGTGTTG	miRNA	miR-21
t0039771	22	2	TAGCTTATCAGGCTGATGTTGA	miRNA	miR-21
t0036291	26	2	TAGCTTATCAGACTGATGTTGCCATC	miRNA	miR-21
t0040463	23	2	CAAAGTGCCCATAGTGCAGGTAG	miRNA	miR-20b
t0036791	25	2	CGGAATGTAAGGAAGTGTGTGGATC	miRNA	miR-206
t0039483	19	2	TTTATGCATATACTTCTTT	miRNA	miR-202-5p
t0035586	21	2	TTTCCTATGCATATACTTCTT	miRNA	miR-202*
t0035294	22	2	TAAAGCGCTTATAGTGCAGGTA	miRNA	miR-20
t0035999	24	2	TAAAGTGCTTATAGTGCAGGAAGA	miRNA	miR-20
t0036250	23	2	ATAAAGTGCTTATAGTGCAGGAA	miRNA	miR-20
t0036392	23	2	TAAAGTGCTTATAGGGCAGGTAG	miRNA	miR-20
t0037053	21	2	TAAAGTGCTTATAGTGCCGGT	miRNA	miR-20
t0037199	23	2	TAAAGTGCTTATAGTGCAGGGAG	miRNA	miR-20
t0038690	22	2	TAAAGTGCTTATAGGGCAGGTA	miRNA	miR-20
t0040097	22	2	TAAAGTGCTTATAGTGCAGGAA	miRNA	miR-20
t0040242	22	2	TAAAGTGCTTACAGTGCAGGTA	miRNA	miR-20
t0036095	25	2	TGGAATGTAAAGAAGTATGTATATA	miRNA	miR-1a
t0039270	21	2	TAGGGAGTTTCCTGTTGTTGG	miRNA	miR-196b
t0035900	22	2	TAGGGAGTTTCCTGTTGTTGGA	miRNA	miR-196b
t0035457	18	2	TGAGGTAGGGTGTATAGT	miRNA	miR-1961
t0036814	20	2	TAGCAGCACATAAATATTCG	miRNA	miR-195
t0037503	23	2	TGTAACAGCAAATCCATGTGGAA	miRNA	miR-194
t0037563	22	2	TGGGGCTTTGCGGGCGAGATGA	miRNA	miR-193a-5p
t0038725	20	2	CTGACCTATGAATTAACAGC	miRNA	miR-192
t0038729	21	2	GTGACCTATGAATTGACAGCC	miRNA	miR-192
t0039003	21	2	TGATCTATGAATTGACAGCCA	miRNA	miR-192
t0039240	23	2	TGACCTATGAATTGACGGCCAGT	miRNA	miR-192
t0039537	21	2	CTGACCTATGAATCGACAGCC	miRNA	miR-192
t0040265	21	2	TGACCTATGAATTGATAGCCA	miRNA	miR-192
t0040579	22	2	CCGACCTATGAATTGACAGCCA	miRNA	miR-192

t0036858	21	2	CTGACCTGTGAATTGACAGCC	miRNA	miR-192
t0038218	23	2	TGCCCTATGAATTGACAGCCAGA	miRNA	miR-192
t0036344	21	2	CTGACCTATGAATTGACCGCT	miRNA	miR-192
t0038178	21	2	CTGACCTATCAATTGACAGCC	miRNA	miR-192
t0040631	24	2	TGACCTATGAATTGACAGACAGAA	miRNA	miR-192
t0038739	24	2	CTGACCTATGAATTGACAGCCCGT	miRNA	miR-192
t0035385	23	2	TGACCTATGAATTGAAAGCCAGT	miRNA	miR-192
t0035418	20	2	TGACCTATGAATTGATAGCC	miRNA	miR-192
t0035578	21	2	CTGACCTATGAATTGACAGTC	miRNA	miR-192
t0035607	21	2	TGACCTATGAATTGACCGCCA	miRNA	miR-192
t0035963	20	2	CGACCTATGAATTGACAGCC	miRNA	miR-192
t0036298	21	2	CTGACCTATGAATTGACGGCC	miRNA	miR-192
t0036691	20	2	TGACCTATGAATTGACAACC	miRNA	miR-192
t0036737	23	2	CAGCGGAATCCCAAAAGCAGCTG	miRNA	miR-191
t0036985	21	2	CAACGGAATCACAAAAGCAGC	miRNA	miR-191
t0037016	23	2	CGACGGAATCCCAAAAGCAGCTG	miRNA	miR-191
t0037780	22	2	CAACGGAATCCCAAAAGAAGAT	miRNA	miR-191
t0037890	23	2	AACGAAATCCCAAAAGCAGCTGA	miRNA	miR-191
t0038243	20	2	CAACGGAATCCCAACAGCAG	miRNA	miR-191
t0038317	23	2	CCACGGAATCCCAAAAGCAGCTG	miRNA	miR-191
t0038550	23	2	CAACGAAATCCCAAAAGCAGCTG	miRNA	miR-191
t0038564	21	2	AAGGGAATCCCAAAAGCAGCT	miRNA	miR-191
t0039170	24	2	CAACGGAATCACAAAAGCAGCTGA	miRNA	miR-191
t0039529	22	2	CAACGGAATCCCAAAAGCGGCT	miRNA	miR-191
t0040473	22	2	AACGCAATCCCAAAAGCAGCTG	miRNA	miR-191
t0040630	23	2	CAACGGAATCCCAAAAGTAGCTG	miRNA	miR-191
t0035235	22	2	CAACGGAATACCAAAAGCAGCA	miRNA	miR-191
t0035242	23	2	CAACGGAATCCCAAAAGCAACTG	miRNA	miR-191
t0035684	24	2	CAACGGGATCCCAAAAGCAGCTGA	miRNA	miR-191
t0035750	20	2	CAACGGCATCCCAAAAGCAG	miRNA	miR-191
t0035881	22	2	CAACGGAATCCCAAAATCAGCT	miRNA	miR-191
t0035910	22	2	AACGGAATCTCAAAAGCCGCTG	miRNA	miR-191
t0035968	23	2	CAATGGAATCCCAAAAGCAGCTG	miRNA	miR-191
t0036159	19	2	CAACAGAATCCCAAAAGCA	miRNA	miR-191
t0036286	21	2	GACGGAATCCCAAAAGCAGCT	miRNA	miR-191
t0036317	23	2	CAACGGAATCCCAAAACCAGCTG	miRNA	miR-191
t0036420	23	2	CAACGGAATCCCAAGAGCAGCTG	miRNA	miR-191
t0036438	22	2	CAACGGAATCGCAAAAGCAGCT	miRNA	miR-191
t0036502	20	2	CAAAGGAATCCCAAAAGCAG	miRNA	miR-191
t0036710	22	2	AATGGAATCCCAAAAGCAGCTG	miRNA	miR-191
t0036712	24	2	CAATGGAATCCCAAAAGCAGCTGA	miRNA	miR-191
t0036823	21	2	CAACGGAATCCCAAAAACAGC	miRNA	miR-191
t0036857	22	2	AACGGAATCCTAAAAGCAGCTG	miRNA	miR-191
t0036891	21	2	CAAAGGAATCCCAAAAGCAGC	miRNA	miR-191
t0036904	22	2	CAACGGAATCCCAAAACCAGCT	miRNA	miR-191
t0036938	22	2	AACGGAATCCCAAAAGTAGCTG	miRNA	miR-191
t0036967	22	2	CAACGGAATCCGAAAAGCAGCT	miRNA	miR-191
t0037007	22	2	GAACGGAATCCCAAAAGCAGCT	miRNA	miR-191
t0037033	22	2	AACGGAATCCCAAAAGCAGCTT	miRNA	miR-191
t0037170	21	2	CAACGGAATCCCAAAAGGAGC	miRNA	miR-191
t0037395	22	2	AACGGAATCCCAAGAGCAGCTG	miRNA	miR-191
t0037485	22	2	CAACGGAATCCCAAAAGCAGGT	miRNA	miR-191
t0037519	22	2	CAACGGAATCCCAAAAGCATCT	miRNA	miR-191
t0037638	23	2	CAACTGAATCCCAAAAGCAGCTG	miRNA	miR-191
t0037710	23	2	AACGGAATCCCAAAAACAGCTGA	miRNA	miR-191
t0037877	24	2	CAACGGAATCCCAAAAGCAGCGGA	miRNA	miR-191
t0037916	23	2	CAACGGAATCCCAGAAGCAGCTG	miRNA	miR-191
t0038052	21	2	AACGGAATCACAAAAGCAGCT	miRNA	miR-191

t0038204	22	2	CAACGGAATCCCAAAGGCAGCT	miRNA	miR-191
t0038466	22	2	CAAGGGAATCCCAAAAGCAGCT	miRNA	miR-191
t0038500	22	2	CAACGGAATACAAAAAGCAGCT	miRNA	miR-191
t0038541	23	2	CAACGGAATCCCAAAAGCAGCTC	miRNA	miR-191
t0038556	23	2	CAACGGAATCCCTAAAGCAGCTG	miRNA	miR-191
t0038647	22	2	CAAAGGAATCCCAAAAGCAGCA	miRNA	miR-191
t0038802	23	2	AAACGGAATCCCAAAAGCAGCTG	miRNA	miR-191
t0038804	19	2	CAATGGAATCCCAAAAGCA	miRNA	miR-191
t0038997	23	2	AATGGAATCCCAAAAGCAGCTGA	miRNA	miR-191
t0039094	23	2	AACGGAATCCCACAAGCAGCTGA	miRNA	miR-191
t0039212	24	2	CACCGGAATCCCAAAAGCAGCTGA	miRNA	miR-191
t0039297	22	2	CAACGGAATCCCAAAAGCTGCT	miRNA	miR-191
t0039301	23	2	CAACGGAATCCCAAACGCAGCTG	miRNA	miR-191
t0039303	23	2	CAACGGAATCCCATAAGCAGCTG	miRNA	miR-191
t0039317	21	2	TAAGGTGCATCTAGTGCCGTT	miRNA	miR-18b
t0039616	21	2	AGCAGGGGCTGGCTTTACACT	miRNA	miR-185*
t0039931	21	2	AGGGGTCGGCTTTCCTCTGGT	miRNA	miR-185*
t0039956	20	2	TGGAGAGAAATGCAGTTCCT	miRNA	miR-185
t0040023	24	2	TGGAGAGAGAAAGGCAGTTCCTGA	miRNA	miR-185
t0040098	22	2	TGGAGAGAAAGCCAGTTACTGA	miRNA	miR-185
t0040173	20	2	GGGAGAGAAAGGCAGTTCCT	miRNA	miR-185
t0040301	23	2	TGGAGAGAAAGGCAGTTGCTGAT	miRNA	miR-185
t0040491	22	2	TAGAGAGAAAGGGAGTTCCTGA	miRNA	miR-185
t0040499	23	2	TGGAGAGAAAGGCAATTCCTGAG	miRNA	miR-185
t0040529	23	2	TGGAGAGAAAGGCAGTTCGTGAT	miRNA	miR-185
t0040570	20	2	TGGAGAGCAAGGCAGTTCCT	miRNA	miR-185
t0040615	24	2	TGGAGAGAAAGGCAGTTCCTGGAA	miRNA	miR-185
t0039183	22	2	TGGAGAGAAAGGCAGCTCCTGT	miRNA	miR-185
t0038405	24	2	TGGAGAGAAAGGAAGTTCCTGAAG	miRNA	miR-185
t0038899	20	2	TGGAGAGAAAGGCAGTCCCT	miRNA	miR-185
t0035209	21	2	TGGAGAGAAAGGAATTTCCTG	miRNA	miR-185
t0035217	22	2	TGGAGAGAAAGACAGTTCCTGC	miRNA	miR-185
t0035326	22	2	TGGAGAGAAAGGGAGTTCATGA	miRNA	miR-185
t0035407	23	2	CTGGAGAGAAAGGAAGTTCCTGA	miRNA	miR-185
t0035472	23	2	TGAAGAGAAAGGCAGTTCCTGAT	miRNA	miR-185
t0035496	21	2	GGAGAGAAAGGAAGTTCCTGA	miRNA	miR-185
t0035516	21	2	TGGAGAGAAAGGAAGTTGCTG	miRNA	miR-185
t0035600	23	2	TGGAGAGAAAAGCAGTTCCTGAG	miRNA	miR-185
t0035784	22	2	TGGAGAGAAAGGCATTTCCTGC	miRNA	miR-185
t0035802	22	2	TGGAGAGAAAGGAAGTTCTTGA	miRNA	miR-185
t0035838	22	2	TGGAGAGAAAGGAAGTTCCTAA	miRNA	miR-185
t0035863	20	2	TGGAGAGAAAGGCACTTCCT	miRNA	miR-185
t0036046	21	2	TGGAGAGAAAGGCAGTTCTTT	miRNA	miR-185
t0036047	24	2	TGGAGAGAAAGGCAGTTCCTAAAA	miRNA	miR-185
t0036084	23	2	TGGAGAGGAAGGCAGTTCCTGAG	miRNA	miR-185
t0036202	22	2	TAGAGAGAAAGGCAGTTCCCGA	miRNA	miR-185
t0036341	23	2	TGGAGAGAAAGGCTGTTCCTGAA	miRNA	miR-185
t0036462	20	2	TGGAGAGAAAGGCAGTACCT	miRNA	miR-185
t0036507	23	2	TGGCGAGAAAGGCAGTTCCTGAG	miRNA	miR-185
t0036526	21	2	TGGAGAGAAAGGCAGTTAATG	miRNA	miR-185
t0036548	22	2	TGGAGGGAAAGGCAGTTCCTTA	miRNA	miR-185
t0036584	22	2	TGGAGAGAAGGGCAGTTCCTGT	miRNA	miR-185
t0036790	22	2	TGGAGAGAACGGCAGTTCCTGC	miRNA	miR-185
t0036800	20	2	TGGGAGAAAGGCAGTTCCTG	miRNA	miR-185
t0036865	22	2	TGGCGAGAAAGGCAGTTTCTGA	miRNA	miR-185
t0037031	22	2	TGGAGAGATAGGCATTTCCTGA	miRNA	miR-185
t0037050	23	2	TGGAGAGAAAGGCGGTTACTGAA	miRNA	miR-185
t0037120	23	2	TGGAGAGATAGGCAGTTCCTGAT	miRNA	miR-185

t0037186	22	2	TGGAGAGAAAGGCAGTTCATGC	miRNA	miR-185
t0037228	22	2	TGGAGATAAAGGCAGTTCCTGG	miRNA	miR-185
t0037298	23	2	GGGAGAGAAAGGCAGTTCCTGAG	miRNA	miR-185
t0037371	22	2	TAGAGAGAAAGGAAGTTCCTGA	miRNA	miR-185
t0037407	22	2	TGGAGAGAAAAGCAGTTACTGA	miRNA	miR-185
t0037696	21	2	TGGAGAGAAAGGAAGTTCCTA	miRNA	miR-185
t0037740	22	2	TGGAGAGAAAGGCAGTTACTTA	miRNA	miR-185
t0037743	20	2	TGAAGAGAAAGGCAGTTCCT	miRNA	miR-185
t0037797	20	2	TGGAGAGAACGGCAGTTCCT	miRNA	miR-185
t0037959	22	2	TGGAGAGAAGGGCAGTTTCTGA	miRNA	miR-185
t0038072	23	2	TGGAGAGAAATGCAGTTCCTGAT	miRNA	miR-185
t0038381	20	2	TGGAGAGAAAGGCAGTTCGT	miRNA	miR-185
t0038494	23	2	TGGAGAGAAAGTCAGTTCCTGAT	miRNA	miR-185
t0038621	23	2	TGGAGAGAAAGGCACTTCCTGAT	miRNA	miR-185
t0038747	22	2	TGGCGAGAAAGGCAGTTCCTGC	miRNA	miR-185
t0038764	23	2	TGGAGAGAAAGGCAGTGCCTGAA	miRNA	miR-185
t0039049	25	2	TGGAGAGAAAGGAAGTTCCTGAAAA	miRNA	miR-185
t0039051	22	2	TGGAGGGAAAGGCAGTTCCTGC	miRNA	miR-185
t0039064	24	2	TGGAGAGAAAGGCAGTTACTGAAA	miRNA	miR-185
t0039214	23	2	TGGAGAGAAAGTCAGTTCCTGAA	miRNA	miR-185
t0039216	21	2	TGGAGAGAAGGGAAGTTCCTG	miRNA	miR-185
t0039300	22	2	TGGAGATAAAGGCAGTTCCTGC	miRNA	miR-185
t0039306	20	2	TGGAGAGAAAGGCTGTTCCT	miRNA	miR-185
t0039319	22	2	TGGAGAGAAAGGGAGTTTCTGA	miRNA	miR-185
t0039438	22	2	TGGAGAGAAAGGAATTTCCTGA	miRNA	miR-185
t0039548	21	2	TGGAGAGAAAGGAAGGTCCTG	miRNA	miR-185
t0039561	22	2	TGGAGAGAAAGGGAGTTCCTGC	miRNA	miR-185
t0039576	22	2	TGGAGAGCAAGGCAGTTCCTGC	miRNA	miR-185
t0039600	22	2	TGGAGAAAAAGGAAGTTCCTGA	miRNA	miR-185
t0039621	22	2	TGGAGAGAAAGGCATTCCCTGA	miRNA	miR-185
t0039649	22	2	TGGAGGGAAAGGCAGTTCCTGT	miRNA	miR-185
t0039746	22	2	TGGAGAGAAAGGGAGTTACTGA	miRNA	miR-185
t0039750	22	2	TGGAGAGGAAGGCAGTTCCTGT	miRNA	miR-185
t0039880	23	2	TGGAAAGAAAGGCAGTTCCTGAG	miRNA	miR-185
t0039916	21	2	TGGAGAGCAAGGCAGTTACTG	miRNA	miR-185
t0039965	20	2	AAGGAAGATAGAACAGGTCT	miRNA	miR-1839
t0039981	22	2	AGGGTAGATAGAACAGGTCTTG	miRNA	miR-1839
t0040104	21	2	AGGTAGGTAGAACAGGTCTTG	miRNA	miR-1839
t0040109	21	2	AGGTAGATAGAACAGGTCTCG	miRNA	miR-1839
t0040134	22	2	AAGGTAGATAGAACAGGTCCTG	miRNA	miR-1839
t0040165	21	2	AGGTAGATAGAACAGGACTTG	miRNA	miR-1839
t0040221	22	2	AGGTAGATAGAACAGGACTTGT	miRNA	miR-1839
t0040367	22	2	AAGGTAGATAGAGCAGGTCTTG	miRNA	miR-1839
t0040414	23	2	AAGGTAGATAGAAAAGGTCTTGA	miRNA	miR-1839
t0040436	22	2	AGGTGGATAGAACAGGTCTTGT	miRNA	miR-1839
t0040524	22	2	AGGAAGATAGAACAGGTCTTGA	miRNA	miR-1839
t0040593	23	2	AAGGTAGATAGAACAGGCCTTGA	miRNA	miR-1839
t0040596	22	2	AAGGTAGATAGAACAGGTCTTA	miRNA	miR-1839
t0035195	18	2	AAGGCAGATAGAACAGGT	miRNA	miR-1839
t0035931	22	2	AAGGTAGTTAGAACAGGTCTTG	miRNA	miR-1839
t0036589	22	2	AAGGTAGATAGAAAAGGTCTTG	miRNA	miR-1839
t0036753	22	2	TGAGGAAGTAGATTGTGTGGTT	miRNA	miR-1827
t0037062	19	2	TGAGGGAGTAGATTGTAGT	miRNA	miR-1827
t0037128	22	2	TGAGGGAGTAGATTGTAGAGTT	miRNA	miR-1827
t0037874	22	2	TGAGGAAGTAGATTGTATATTT	miRNA	miR-1827
t0037897	21	2	TAGGGAGTAGATTGTATAGTT	miRNA	miR-1827
t0038483	21	2	TGAGGGAGAAGGTTGTATAGT	miRNA	miR-1827
t0038604	26	2	TGAGGGAGTAGGTTGTATGGTTTATC	miRNA	miR-1827

t0038736	22	2	TGAGGGAGTAGGATGTATAGTT	miRNA	miR-1827
t0039290	22	2	TGAGGGAGTCGATTGTATAGTT	miRNA	miR-1827
t0039743	21	2	TGAGGGAGTAGATTGCATAGT	miRNA	miR-1827
t0039946	21	2	TGAGGGAGTAGGTTGTATTGT	miRNA	miR-1827
t0040208	20	2	TGAGGGGGTAGATTGTATAG	miRNA	miR-1827
t0040617	21	2	TGAGGGAGTAGATTGTATACT	miRNA	miR-1827
t0035295	19	2	GAGGGAGTAGATTGTATAG	miRNA	miR-1827
t0035316	22	2	TGAGGGAGTAGATTGTATAGCT	miRNA	miR-1827
t0035357	22	2	TGAGGGAGTAGTTTGTATAGTT	miRNA	miR-1827
t0035359	20	2	TGCGGTAGTAGATTGTATTT	miRNA	miR-1827
t0035404	23	2	TGAGGGAGTAGATTGTATAGTAA	miRNA	miR-1827
t0035753	23	2	TGAGGGAGTAGGTTGTATAGTTT	miRNA	miR-1827
t0036007	26	2	TGAGGGAGTAGATTGTATAGTTACGT	miRNA	miR-1827
t0036180	20	2	TGAGGGAGTAGATTGTACAG	miRNA	miR-1827
t0036384	21	2	TGAGGGAGTAGATTGTATAGG	miRNA	miR-1827
t0036480	23	2	TGAGGGAGTAGTTTGTACAGTAA	miRNA	miR-1827
t0036493	20	2	TGAGGGAGTAGGTTGTATGG	miRNA	miR-1827
t0036695	21	2	TGAGGAAGTAGATTGTATGGT	miRNA	miR-1827
t0037041	22	2	TGAGGGAGTAGATTGTATTGTT	miRNA	miR-1827
t0037311	21	2	TGAGGGAGTAGATTGTATCGT	miRNA	miR-1827
t0037390	24	2	TGAGGGAGTAGATTGTATAGTTGA	miRNA	miR-1827
t0037435	22	2	TGAGGCAGTAGATTGTATATTT	miRNA	miR-1827
t0037459	20	2	TGAGGGAGTAGGTTGTAGTT	miRNA	miR-1827
t0037575	22	2	TGAGGGAGTAGATTGTATAGAA	miRNA	miR-1827
t0037599	22	2	CTGAGGGAGTAGGTTGTATAGT	miRNA	miR-1827
t0037724	22	2	TGAGGGAGGAGATTGTGTAGTT	miRNA	miR-1827
t0037758	22	2	TGAGGAAGTAGATTGTATGGTT	miRNA	miR-1827
t0037817	22	2	TGAGAGAGTAGATTGTATAGTT	miRNA	miR-1827
t0037900	22	2	TGAGGGAGTAGATTGTAAAGTT	miRNA	miR-1827
t0038044	21	2	TGAGGGAATAGGTTGTATAGT	miRNA	miR-1827
t0038392	22	2	TGGGGGAGTAGGTTGTATAGTT	miRNA	miR-1827
t0038571	22	2	TGAGGGAGTTGATTGTATAGTT	miRNA	miR-1827
t0038803	22	2	TGAGGCAGTAGATTGTATACTT	miRNA	miR-1827
t0038809	18	2	TGAGGGAGTAGATTGTAG	miRNA	miR-1827
t0038888	22	2	TGAGGGAGTAGGTTGTATCGTT	miRNA	miR-1827
t0038984	22	2	TGAGGGAGTAGTTTGTACAGTA	miRNA	miR-1827
t0039120	22	2	TTTGGCAATGGGAGAACTCACA	miRNA	miR-182
t0039879	24	2	TTTGGCAATGGTAGAAATCACACT	miRNA	miR-182
t0039942	28	2	TTTGGCAATGGTAGAACTCACCCCGATC	miRNA	miR-182
t0040038	23	2	TTTGGCAATGGTAGAACTCACCC	miRNA	miR-182
t0040095	22	2	TTTGGCAATGGTAGAACTCCCA	miRNA	miR-182
t0040217	24	2	TTTGGCAATGGTAGAACTCACCCT	miRNA	miR-182
t0040286	20	2	AACATTCATTGCTATCGGTG	miRNA	miR-181b
t0040321	22	2	ACATTCATTGCTGTCGGTGGGG	miRNA	miR-181b
t0040398	21	2	AACATTCAACGCTGTCGGGGA	miRNA	miR-181a
t0040416	21	2	AACATTCAACGCTGTCGGTGC	miRNA	miR-181a
t0040477	24	2	AACATTCAACGCTGTCGGTGACTC	miRNA	miR-181a
t0040532	20	2	AACATTCAACGCTGGCGGTG	miRNA	miR-181a
t0040638	23	2	AACATTCAACGCTGTCGGAGAGT	miRNA	miR-181a
t0036016	22	2	AACATTAAACGCTGTCGGTGAG	miRNA	miR-181a
t0037083	19	2	AACATTCAACGCTGTAGGT	miRNA	miR-181a
t0037498	22	2	CAAAGTGCTTACAGTGCAGGTC	miRNA	miR-17a
t0038153	23	2	CAAAGTGCTCATAGTGCAGGTCG	miRNA	miR-17a
t0039748	24	2	TCGGGGCGGCGGCGGCGGCGGGGG	miRNA	miR-1777a
t0040351	19	2	TCGGGGCGGCGGCGGCGGC	miRNA	miR-1777a
t0039624	22	2	TCGGGGCGGCGGCGGCGGGGGG	miRNA	miR-1777a
t0039807	18	2	TCGGGGCGGCGGCGGGGG	miRNA	miR-1777a
t0035524	20	2	TCGGGGCGGCGGCGGAGGCG	miRNA	miR-1777a

t0036185	23	2	ACTGCAGTGAAGGCACTTGGAGA	miRNA	miR-17-3p
t0036246	22	2	ACTGCAGTGAAGGAACTTGTAG	miRNA	miR-17*
t0036535	22	2	ACTGAAGTGAAGGCACTTGTAG	miRNA	miR-17*
t0036639	19	2	ACTGCAGTGAAGGAACTTG	miRNA	miR-17*
t0037872	24	2	CAAAGTGCTTACAGTGCAGGCAGA	miRNA	miR-17
t0039877	22	2	CAAAGTGCTTACAGAGCAGGTA	miRNA	miR-17
t0037281	21	2	CAAAGTGCTTATAGTGCAGGT	miRNA	miR-17
t0039465	22	2	CAAAGTGCTTACAGTGTAGGTA	miRNA	miR-17
t0035858	23	2	CAAAGTGCTTACAGTGCAGGAAG	miRNA	miR-17
t0036872	22	2	CAAAGTGCTTATAGTGCAGGTA	miRNA	miR-17
t0038806	24	2	CAAAGTGCTTACAGGGCAGGTAGA	miRNA	miR-17
t0039822	23	2	CAAAGGGCTTACAGTGCAGGTAG	miRNA	miR-17
t0040072	24	2	CAAAGTGCTTACAGTGCAGGTAGC	miRNA	miR-17
t0038617	23	2	CAAAGTGCTTACAGTGCAGGGAG	miRNA	miR-17
t0035803	22	2	TAGCAGCACGTTAATATTGGCA	miRNA	miR-16b
t0039137	24	2	TAGCAGCACGTAAATATTGGAAAA	miRNA	miR-16b
t0039620	22	2	TAGCAGCACGTAAATATTGGTT	miRNA	miR-16b
t0035667	22	2	TAGCAGCATGTAAATATTGGCA	miRNA	miR-16b
t0035683	22	2	TAGCAGCACGTAAATATTGACA	miRNA	miR-16b
t0036120	22	2	TAGCAGCACGGAAATATTGGCT	miRNA	miR-16b
t0036594	22	2	TAGCAGAACGTAAATATTGGCC	miRNA	miR-16b
t0039262	21	2	TACAGCACGTAAATATTGGCT	miRNA	miR-16b
t0039346	22	2	TAGCAACACGTAAATATTGGCA	miRNA	miR-16b
t0039798	22	2	TAGCAGCGCGTAAATATTGGCT	miRNA	miR-16b
t0039812	22	2	TAGCAGCACGTAAATATCGGCA	miRNA	miR-16b
t0040154	22	2	TAGCAGCACGTAAATATTAGCA	miRNA	miR-16b
t0040262	22	2	TAGCAGCAAGTAAATATTGGCA	miRNA	miR-16b
t0036906	28	2	GGCGGGGAGCGGTCGGGCGGCGGCGGT	miRNA	miR-1607
t0037169	25	2	GGCGGGGAGCGGTCGGGCGGCGGGG	miRNA	miR-1607
t0037421	19	2	TAGCAGCACGTAAATATTT	miRNA	miR-16
t0037770	24	2	TGGCAGCACGTAAATATTGGCGAA	miRNA	miR-16
t0038561	22	2	TAGCGGCACGTAAATATTGGAG	miRNA	miR-16
t0038631	20	2	TAGCAGGACGTAAATATTGG	miRNA	miR-16
t0039112	22	2	TAGCCGCACGTAAATATTGGAG	miRNA	miR-16
t0039395	22	2	TAGCAGCACGTAGATATTGTCG	miRNA	miR-16
t0039428	22	2	TAGCAGCACGTCAATATTGGAG	miRNA	miR-16
t0039617	23	2	TATCAGCACGTAAATATTGGCGA	miRNA	miR-16
t0040030	22	2	TAGCAGCATGTAAATATTGTCG	miRNA	miR-16
t0040119	19	2	TAGCAGCACGCAAATATTG	miRNA	miR-16
t0040174	22	2	TAGCAGCACGTAAATACTGCCG	miRNA	miR-16
t0037046	22	2	TAGCCGCACGTAAATATTGCCG	miRNA	miR-16
t0038697	21	2	TAGCAGCAAGTAAATATTGGT	miRNA	miR-16
t0035312	22	2	TAGAAACACGTAAATATTGGCG	miRNA	miR-16
t0035398	23	2	TAGCAGCACGTAACTATTGGCGT	miRNA	miR-16
t0035431	19	2	TAGCAGCACGGAAATATTG	miRNA	miR-16
t0035441	19	2	TAGCAGCCCGTAAATATTG	miRNA	miR-16
t0035449	23	2	TAGCAGCCCGTAAATATTGGCGA	miRNA	miR-16
t0035462	24	2	AATAGCAGCACGTAAATATTGGAG	miRNA	miR-16
t0035493	21	2	AGTAGCACGTAAATATTGGCG	miRNA	miR-16
t0035510	19	2	TAGCCGCACGTAAATATTG	miRNA	miR-16
t0035559	19	2	TAGCAGCACGTACATATTG	miRNA	miR-16
t0035596	22	2	TAGCAGCACGTAAATATTGAAG	miRNA	miR-16
t0035836	19	2	TAACAGCACGTAAATATTG	miRNA	miR-16
t0035840	23	2	TAGCAGCACGTGAATATTGGCGA	miRNA	miR-16
t0035852	23	2	TAGCACCACGTAAATATTGGCGA	miRNA	miR-16
t0035971	19	2	CAGCAGCACGTAAATATTG	miRNA	miR-16
t0036019	24	2	TAGCAGCACGTAAATATTGGTGAA	miRNA	miR-16
t0036178	20	2	TTGCAGCACGTAAATATTGG	miRNA	miR-16

t0036235	22	2	TAGCAGCACGTCAATATTGTCG	miRNA	miR-16
t0036242	22	2	TAGCAGCACGTAAATATTATCG	miRNA	miR-16
t0036281	23	2	TAGGAGCACGTAAATATTGGCGT	miRNA	miR-16
t0036339	21	2	TAGCAGCACGTAAATATTGCA	miRNA	miR-16
t0036465	20	2	TAGCAGCACGTATATATTGG	miRNA	miR-16
t0036607	22	2	TAGCAGCACGTAAATATTAGAG	miRNA	miR-16
t0036643	22	2	TAGCAGCATGTAAATATTGCCG	miRNA	miR-16
t0036682	23	2	TACCAGCACGTAAATATTGGCGA	miRNA	miR-16
t0036839	22	2	TAGCAGAACGTAAATATTGGTG	miRNA	miR-16
t0036889	21	2	AGCAGCACGTAAGTATTGGCG	miRNA	miR-16
t0037178	22	2	TAGTAGCACGTAAATATTGTCG	miRNA	miR-16
t0037213	22	2	AAGCAGTACGTAAATATTGGCG	miRNA	miR-16
t0037227	19	2	TAGCAGCGCGTAAATATTG	miRNA	miR-16
t0037229	20	2	TAGCAGCACGTTAATATTGG	miRNA	miR-16
t0037255	23	2	TAGCAGCACGTAAATATAGGCGA	miRNA	miR-16
t0037317	23	2	TAGCAGCAGGTAAATATTGGCGA	miRNA	miR-16
t0037592	19	2	TAGCATCACGTAAATATTG	miRNA	miR-16
t0037615	21	2	TAGCAGAACGTAAATATTGGC	miRNA	miR-16
t0037620	23	2	TAGCAGCAAGTAAATATTGGCGA	miRNA	miR-16
t0037662	23	2	TAGCAGCGCGTAAATATTGGCGT	miRNA	miR-16
t0037685	23	2	ATAGGAGCACGTAAATATTGGCG	miRNA	miR-16
t0037706	22	2	TAGCAGCGCGTAAATATTGTCG	miRNA	miR-16
t0037945	21	2	AGCAGAACGTAAATATTGGCG	miRNA	miR-16
t0038060	22	2	TAGCAGCAAGTAAATATTGTCG	miRNA	miR-16
t0038129	23	2	TAGCAGCACGTGAATATTGGCGT	miRNA	miR-16
t0038241	23	2	TAGCAGCACCTAAATATTGGCGA	miRNA	miR-16
t0038418	22	2	TAGCAGCACGTAAATATTCGAG	miRNA	miR-16
t0038453	21	2	TAGCAGTACGTAAATATTGGT	miRNA	miR-16
t0038505	21	2	AGCAGCACGTAAATATTAGCG	miRNA	miR-16
t0038520	20	2	TAGCAGCACGTAAATATTCA	miRNA	miR-16
t0038588	22	2	TAGCAGAACGTAAATATTGGGG	miRNA	miR-16
t0038629	22	2	TAGCAGAACGTAAATATTTGCG	miRNA	miR-16
t0038663	19	2	TAGGAGCACGTAAATATTG	miRNA	miR-16
t0038682	23	2	TAGCAGCACGTCAATATTGGCGT	miRNA	miR-16
t0038692	22	2	TAGCAGCACGTCAATATTGCCG	miRNA	miR-16
t0038760	23	2	TAGCAGCACGTAAAAATTGGCGA	miRNA	miR-16
t0038787	21	2	TAGCACCACGTAAATATTGGC	miRNA	miR-16
t0038867	21	2	AGCAGCACGGAAATATTGGCG	miRNA	miR-16
t0038894	22	2	TAGCAGCATGTAAATATTGACG	miRNA	miR-16
t0038922	20	2	TATCAGCACGTAAATATTGG	miRNA	miR-16
t0038951	19	2	TAGCAGGACGTAAATATTG	miRNA	miR-16
t0038968	24	2	TAGCAGCACGGAAATATTGGCGAA	miRNA	miR-16
t0038974	22	2	TAGCCGTACGTAAATATTGGCG	miRNA	miR-16
t0038987	22	2	TAGCAGTATGTAAATATTGGCG	miRNA	miR-16
t0039019	24	2	TAGCAGCATGTAAATATTGGCGAA	miRNA	miR-16
t0039068	22	2	TAGCGGCACGTAAATATTGTCG	miRNA	miR-16
t0039125	20	2	TGCAGCACGTAAATATTGGC	miRNA	miR-16
t0039157	22	2	TAGCAGCACGTAAACATTGTCG	miRNA	miR-16
t0039311	22	2	TAGAAGCACGTAAATATTGGTG	miRNA	miR-16
t0039314	23	2	ATAGCAGCACGTAAATATTGGAG	miRNA	miR-16
t0039315	23	2	TAGCAGCACGTAAATATGGGCGA	miRNA	miR-16
t0039390	21	2	AGCAGCACGTAAATATTTGCG	miRNA	miR-16
t0039592	20	2	TAGCAGCACCTAAATATTGG	miRNA	miR-16
t0039619	22	2	TAGCAGCATGTAAGTATTGGCG	miRNA	miR-16
t0039625	23	2	TAGCAGCACGTAAATGTTGGCGT	miRNA	miR-16
t0039642	19	2	TGGCAGCACGTAAATATTG	miRNA	miR-16
t0039677	22	2	TAGGAGCACGTAAATATTGTCG	miRNA	miR-16
t0039692	19	2	TAGCAGCATGTAAATATTG	miRNA	miR-16

t0039696	22	2	TAGCAGAACGTAGATATTGGCG	miRNA	miR-16
t0039703	18	2	TAGCGGCACATCATGGTT	miRNA	miR-15b
t0039888	20	2	TAGAAGCACATCATGGGTTA	miRNA	miR-15b
t0039894	19	2	TAGAAGCAAATCATGGTTT	miRNA	miR-15b
t0039992	19	2	TAGCAGAGCATCATGGTTT	miRNA	miR-15b
t0040026	21	2	TAGCAGCACATCATGGTTAAA	miRNA	miR-15b
t0040059	21	2	TAGCAGCACATCATGGTTTCC	miRNA	miR-15b
t0040093	19	2	TAGCAGCAAATCATGGGTT	miRNA	miR-15b
t0040182	21	2	TAGCAGCCCATCATGGTTTAC	miRNA	miR-15b
t0040296	22	2	TAGCAGCACATAATGGTTTATG	miRNA	miR-15b
t0040366	19	2	TAGCAGCACATCATGATTT	miRNA	miR-15b
t0040460	18	2	TAGCATCACATCATGGTT	miRNA	miR-15b
t0040527	19	2	TAGCAGCACATCAAGGTTT	miRNA	miR-15b
t0040618	20	2	TAGCAGCACATCATGGTATA	miRNA	miR-15b
t0035581	22	2	TAGCAGCACTTCATGGTTTACA	miRNA	miR-15b
t0035842	19	2	TAGCAGCACATCATGGTAT	miRNA	miR-15b
t0035865	21	2	TAGCAGCAAATCATGGTTTAC	miRNA	miR-15b
t0035883	21	2	TAGCAGCACACCATGGTTTAC	miRNA	miR-15b
t0035979	19	2	CAGCAGCACATCATGGTTA	miRNA	miR-15b
t0036049	19	2	TAGCAGCGCATCATGGTTA	miRNA	miR-15b
t0036078	20	2	TAGAAGCACATAATGGTTTA	miRNA	miR-15b
t0036363	20	2	TAGCAGCACATCATGGGTTC	miRNA	miR-15b
t0036370	22	2	TAGCAGCACAGCATGGTTTACA	miRNA	miR-15b
t0036379	22	2	TAGCAGCACATCATGGGTTATA	miRNA	miR-15b
t0036561	18	2	TGGCAGCACATCATGGTT	miRNA	miR-15b
t0036610	21	2	TAGCAGCACATTATGGTTTAC	miRNA	miR-15b
t0036861	22	2	TAGCAGCACATCATGGTATACA	miRNA	miR-15b
t0036873	18	2	TAGCAGCACCTCATGGTT	miRNA	miR-15b
t0037055	22	2	TAGCAGCTCATCATGGTTTACA	miRNA	miR-15b
t0037107	19	2	TAGCAGGAAATCATGGTTT	miRNA	miR-15b
t0037139	21	2	TAGCAGCACATCATGGATTAC	miRNA	miR-15b
t0037201	21	2	TAGAAGCACATCATGGTTTAC	miRNA	miR-15b
t0037205	18	2	TAGCAGGACATCATGGTT	miRNA	miR-15b
t0037474	21	2	TAGCAGCACATCGTGGTTTAC	miRNA	miR-15b
t0037950	21	2	TAGCATCACATCATGGTTTAA	miRNA	miR-15b
t0038015	20	2	TAGAAGCAAATCATGGTTTA	miRNA	miR-15b
t0038063	22	2	TAGCAGCACATCATGGTTTAGA	miRNA	miR-15b
t0038327	22	2	TAGCAGAACATCATGGGTTACA	miRNA	miR-15b
t0038650	22	2	TAGCAGCACATAATGGTTCGTG	miRNA	miR-15a
t0038781	21	2	TAGCCGCACATAATGGTTTGT	miRNA	miR-15a
t0038919	21	2	TAGCAGCACATAATGGTTCGT	miRNA	miR-15a
t0039056	21	2	TAGCAGAACATAATGGGTTGT	miRNA	miR-15a
t0039202	21	2	TAGCAGCAGATAATGGTTTGT	miRNA	miR-15a
t0039288	22	2	TAGCGGCACATAATGGTTTGTG	miRNA	miR-15a
t0039810	21	2	TAGCAGCACATAATGGTGTGT	miRNA	miR-15a
t0039848	20	2	TAGCAGCACATAATGGGCTG	miRNA	miR-15a
t0039980	22	2	TAGCAGCACATAATGGTTTGGA	miRNA	miR-15a
t0040188	22	2	TAGAAGCACATAATGGTTTGTG	miRNA	miR-15a
t0040203	19	2	CAGCAGCACATAATGGTTT	miRNA	miR-15a
t0040339	22	2	TAGCAGAACATAATGGTTTACA	miRNA	miR-15a
t0040624	19	2	AAGCAGCACATAATGGTTT	miRNA	miR-15a
t0035304	22	2	TAGCAGCACATAATGATTTGTG	miRNA	miR-15a
t0035443	23	2	ATAGCAGCACATAATGGTTTTTG	miRNA	miR-15a
t0035594	22	2	TAGCAACACATAATGGTTTGTG	miRNA	miR-15a
t0035679	18	2	TAGCCGCACATAATGGTT	miRNA	miR-15a
t0035730	20	2	TAGCAGCACATAATGGGGTG	miRNA	miR-15a
t0035820	21	2	TCGCAGCACATAATGGTTTGT	miRNA	miR-15a
t0035921	19	2	TAGCAGCACGTAATGGTTT	miRNA	miR-15a

t0036109	19	2	TAGCAACACATAATGGTTT	miRNA	miR-15a
t0036700	19	2	TAGCAGCACATAATGGCTT	miRNA	miR-15a
t0036838	20	2	TAGCAGCACATAATGGTATG	miRNA	miR-15a
t0036929	18	2	TAGCAGCGCATAATGGTT	miRNA	miR-15a
t0036952	20	2	TAGCTGCACATAATGGTTTG	miRNA	miR-15a
t0037042	21	2	TAGCAGCACACAATGGGTTGT	miRNA	miR-15a
t0037068	22	2	TAGCAGCACATAGTGGTTTGTG	miRNA	miR-15a
t0037077	20	2	TAGCAGAACATAATGGGTTG	miRNA	miR-15a
t0037085	21	2	TAGCAGGACATAATGGTTTGT	miRNA	miR-15a
t0037344	22	2	TAGGAGCACATAATGGTTTGTG	miRNA	miR-15a
t0037875	19	2	TAGCAGCACATAATGGTTA	miRNA	miR-15a
t0037978	18	2	TAGCAGCACATAATGGTA	miRNA	miR-15a
t0038299	20	2	TAGCAGGACATAATGGTTTG	miRNA	miR-15a
t0038407	24	2	TCAGTGCATGACAGAACTTGGCTC	miRNA	miR-152
t0038445	21	2	TCTCCTAACCCTTGTACCAGT	miRNA	miR-150
t0038627	22	2	TCTCCCAACCCTTGAACCAGTG	miRNA	miR-150
t0038652	22	2	TCTCCCAACCCTTGTAGCAGTG	miRNA	miR-150
t0039211	21	2	TCAGGGCATCACAGAACTTTG	miRNA	miR-148b-3p
t0039230	23	2	TCAGTGCATCACAGAACTTTGCA	miRNA	miR-148b-3p
t0039281	21	2	ACAGTGCACTACAGAACTTTG	miRNA	miR-148a
t0039361	21	2	TCAGTGCACCACAGAACTTTG	miRNA	miR-148a
t0039363	21	2	TCAGTGAACTACAGAACTTTG	miRNA	miR-148a
t0039787	27	2	TGAGAACTGAATTCCATAGGCTGGATC	miRNA	miR-146b
t0040181	23	2	GGATATCATCATATATTGTAAGT	miRNA	miR-144*
t0040213	22	2	GGATATCATCATATACTGGAAG	miRNA	miR-144*
t0040252	23	2	GGATATAATCATATACTGTAAGT	miRNA	miR-144*
t0036821	21	2	GGATCTCATCATATACTGTAA	miRNA	miR-144*
t0036603	21	2	GGATATCATCATATAGTGTAA	miRNA	miR-144*
t0038536	22	2	GGATATCATCATATACTGTTAG	miRNA	miR-144*
t0038993	22	2	GGATATCATAATATACTGTAAG	miRNA	miR-144*
t0035815	22	2	GGATATTATCATATACTGTAAG	miRNA	miR-144*
t0036466	22	2	GGATATCACCATATACTGTAAG	miRNA	miR-144*
t0037256	20	2	GGATATAATCATATACTGTA	miRNA	miR-144*
t0038648	21	2	TACAGTATAGATGATGTCCTA	miRNA	miR-144
t0040021	20	2	TACAGGATAGATGATGTACT	miRNA	miR-144
t0039035	20	2	TACAGTATAGATGTTGTACT	miRNA	miR-144
t0035619	20	2	CACAGTATAGATGATGTACT	miRNA	miR-144
t0035648	18	2	TACAGAATAGATGATGTA	miRNA	miR-144
t0036443	18	2	CACAGTATAGATGATGTA	miRNA	miR-144
t0036688	18	2	TACAGTATAGATGACGTA	miRNA	miR-144
t0037894	18	2	TATAGTATAGATGATGTA	miRNA	miR-144
t0038303	18	2	TACAGTATAGGTGATGTA	miRNA	miR-144
t0039400	19	2	CTGCAGTATAGATGATGTA	miRNA	miR-144
t0040157	18	2	TACAGTATAGATGATGAA	miRNA	miR-144
t0040335	18	2	TACGGTATAGATGATGTA	miRNA	miR-144
t0040401	25	2	TACAGTATAGATGATGTAAAAAAAA	miRNA	miR-144
t0035241	19	2	CTACATTATAGATGATGTA	miRNA	miR-144
t0035265	19	2	CTACAGTATAGCTGATGTA	miRNA	miR-144
t0035401	19	2	CTACAGTATAGATGATGTG	miRNA	miR-144
t0036560	22	2	TACAGTATAGATGATGTACTTG	miRNA	miR-144
t0037030	18	2	TACAGTATTGATGATGTA	miRNA	miR-144
t0037216	22	2	TACAGTATAGATGACGTACTAT	miRNA	miR-144
t0037328	19	2	CTACAGTATAGATGACGTA	miRNA	miR-144
t0037413	19	2	CTACAGTATAGATGCTGTA	miRNA	miR-144
t0037822	23	2	TGAGATGAAGCACTGTAGCTATA	miRNA	miR-143
t0038171	23	2	TGAGATGAAGCACAGTAGCTATC	miRNA	miR-143
t0038886	23	2	TGAGATGAAGCACTGTAGTTATC	miRNA	miR-143
t0038963	23	2	TGAGATGAAGAACTGTAGCTATC	miRNA	miR-143

t0039138	23	2	TGAGATGAAGCACTGGAGCTATC	miRNA	miR-143
t0039181	23	2	TGAGATGGAGCACTGTAGCTATC	miRNA	miR-143
t0039373	23	2	TGGGATGAAGCACTGTAGCTATC	miRNA	miR-143
t0039652	23	2	CGAGATGAAGCACTGTAGCTATC	miRNA	miR-143
t0039876	23	2	TGAGATGAAGCACTATAGCTATC	miRNA	miR-143
t0039903	19	2	CATAAAGAAGAAAGCACTA	miRNA	miR-142-5p
t0039929	19	2	CATAAAGCAGAAAGCACTA	miRNA	miR-142-5p
t0040126	21	2	CATAAAGTAGAAAGCATTACT	miRNA	miR-142-5p
t0040402	19	2	CATAAAGTAGAAAGAACTA	miRNA	miR-142-5p
t0035687	20	2	ACCATAAAGTAGAAAGCACT	miRNA	miR-142-5p
t0036476	20	2	CATAAAGTAGAAAGTACTAC	miRNA	miR-142-5p
t0036699	21	2	CATAAAGTAGAAAGCACTAAT	miRNA	miR-142-5p
t0036930	22	2	GTGGTGTTTCCTACTTTATGGA	miRNA	miR-142-3p
t0037132	22	2	GTAGTGTTTCCTACTTTCTGGA	miRNA	miR-142-3p
t0038173	22	2	GTAGGGTTTCCTACTTTATGGA	miRNA	miR-142-3p
t0039335	21	2	GGAGTGTTTCCTACTTTATGG	miRNA	miR-142*
t0039423	20	2	CCCATAAAGTAGCAAGCACT	miRNA	miR-142
t0039522	20	2	CCCATAAAGTAGAAAGCCTT	miRNA	miR-142
t0035440	21	2	CCCATAAAGTAGAAGGCACTA	miRNA	miR-142
t0035495	20	2	CCCATCAAGTAGAAAGCACT	miRNA	miR-142
t0036205	18	2	CCCATAAAGTAGAAAGAA	miRNA	miR-142
t0036917	20	2	CCCATAAAGTAGAACGCACT	miRNA	miR-142
t0037119	21	2	CCCATAAAGTAAAAAGCACTA	miRNA	miR-142
t0038712	21	2	CCAATAAAGTAGAAAGCACTA	miRNA	miR-142
t0039940	21	2	CCCATAAAGTGGAAAGCACTA	miRNA	miR-142
t0035917	19	2	CCCATAAAGTAGAAAGCCC	miRNA	miR-142
t0038151	20	2	CCCATAAAGTAGAAGGCACT	miRNA	miR-142
t0040177	21	2	CCCATAAAGTAGGAAGCACTA	miRNA	miR-142
t0036558	21	2	CCCATAAAGTAGAAAGCAATA	miRNA	miR-142
t0035233	21	2	CCCACAAAGTAGAAAGCACTA	miRNA	miR-142
t0035438	21	2	ACCACAGGGTAGAATCATGGA	miRNA	miR-140
t0036590	24	2	TACCACAGGGTAGAACTACGGCCA	miRNA	miR-140
t0038649	23	2	TACGACAGGGTAGAACCACGGAT	miRNA	miR-140
t0038776	21	2	ACCAAAGGGTAGAAACACGGA	miRNA	miR-140
t0038883	21	2	ACCACAGGCTAGAAACACGGA	miRNA	miR-140
t0038917	24	2	ACCACAGGGTAGAACCACGGCCGA	miRNA	miR-140
t0039071	22	2	ACCACAGGGTAGTACCACGGAT	miRNA	miR-140
t0039116	22	2	ACCACAGGGGAGAACCACGGCT	miRNA	miR-140
t0039221	21	2	ACAACAGGGTAGAAACACGGA	miRNA	miR-140
t0039244	22	2	ACCACAGGGTAGAACCACTGAC	miRNA	miR-140
t0039247	20	2	ATCACAGGGTAGAACCACGG	miRNA	miR-140
t0039383	22	2	ACCACAGGGTAAAACCACGGAT	miRNA	miR-140
t0039794	22	2	ACCACTGGGTAGAACCACGGAC	miRNA	miR-140
t0035204	23	2	TACCACAGGGTAGAACAACGGCC	miRNA	miR-140
t0035231	24	2	TACCACAGGGTAGTACCACGGACA	miRNA	miR-140
t0035249	23	2	ACCCCAGGGTAGAACCACGGACA	miRNA	miR-140
t0035288	22	2	ACCACAGGGTAGTACCACGGAA	miRNA	miR-140
t0035290	23	2	TACCACAGGGAAGAACCACGGAT	miRNA	miR-140
t0035337	23	2	ACCACAGGGTGGAACCACGGATA	miRNA	miR-140
t0035390	20	2	CTACAGGGTAGAACCACGGA	miRNA	miR-140
t0035450	23	2	TACCACAGGGTAGGACCACGGAT	miRNA	miR-140
t0035464	24	2	TACCACAGGGAAGAACCACGGACG	miRNA	miR-140
t0035501	26	2	ACCACAGGGTAGAACCACGGCCAAGA	miRNA	miR-140
t0035504	20	2	ACCACAGGGTAGAACCACGA	miRNA	miR-140
t0035557	23	2	TACCACAGGGTTGAACCACGGAT	miRNA	miR-140
t0035565	22	2	ACCACAGGGTAGAAGCACGGAA	miRNA	miR-140
t0035570	22	2	ACCACCGGGTAGAACCACGGAA	miRNA	miR-140
t0035631	22	2	ACCACAGGGTAGCACTACGGAA	miRNA	miR-140

t0035666	22	2	TACCACAGGGCAGAACCCCGGA	miRNA	miR-140
t0035805	22	2	ACCACAGGGGAGAACAACGGAC	miRNA	miR-140
t0035895	22	2	TACCACAGGGAAGAACCCCGGA	miRNA	miR-140
t0035924	24	2	TACCACAGGGTAGAACCAAGGAAA	miRNA	miR-140
t0035958	24	2	TACCGCAGGGTAGAACCACGGAAA	miRNA	miR-140
t0036014	23	2	TACCACAGGCTAGAAACACGGAC	miRNA	miR-140
t0036018	23	2	ACCACAGGGTAGGACCACGGACA	miRNA	miR-140
t0036028	24	2	TACTACAGGGTAGAACCACGGAAA	miRNA	miR-140
t0036045	22	2	ACCACAGGGTAGAACGACGGAA	miRNA	miR-140
t0036081	22	2	TACCACAGGGTGGAACCCCGGA	miRNA	miR-140
t0036087	23	2	TACCACAGGGGAGAACCACGGAG	miRNA	miR-140
t0036094	24	2	TACCACAGGGTAGAATCCCGGACA	miRNA	miR-140
t0036099	24	2	ACCACAGGGGAGAACCACGGAAGA	miRNA	miR-140
t0036103	22	2	ACCACAGGGGACAACCACGGAC	miRNA	miR-140
t0036108	22	2	ACCACAGGGTAGAACCAGGAAA	miRNA	miR-140
t0036118	20	2	CCACAGGGTAGAACCCCGGA	miRNA	miR-140
t0036121	23	2	ACCACAGGGTAGAACCACGGCAG	miRNA	miR-140
t0036130	22	2	ACAACAGGGTAGAAACACGGAA	miRNA	miR-140
t0036135	24	2	TACCACAGGGTAGAACCAAGGAAT	miRNA	miR-140
t0036142	21	2	CCACAGGGTAGAAACACGGAA	miRNA	miR-140
t0036154	22	2	TCCACAGGGTAGAACCACGGAC	miRNA	miR-140
t0036187	22	2	TACCATAGGGTAGAACCCCGGA	miRNA	miR-140
t0036238	23	2	TACTACAGGGTAGAACCACGGAG	miRNA	miR-140
t0036271	21	2	TACCACAGGGTAGAACCACCG	miRNA	miR-140
t0036402	22	2	ACCACAGGCTAGAACCACGGAC	miRNA	miR-140
t0036508	21	2	ACCACAGGGAAGAACCACGGT	miRNA	miR-140
t0036547	22	2	TACTACAGGGTAGAACCCCGGA	miRNA	miR-140
t0036555	21	2	ACCACAGTGTAGAAACACGGA	miRNA	miR-140
t0036615	21	2	ACCACAGGGTAGAACCCCGGT	miRNA	miR-140
t0036622	23	2	TACCACAGGGTAGAAACAAGGAA	miRNA	miR-140
t0036626	22	2	TACCACAGGGTAGAACAACGGT	miRNA	miR-140
t0036635	23	2	TACCACAGGGTAGAATCCCGGAC	miRNA	miR-140
t0036653	21	2	ACCACAGGGAAGAACAACGGA	miRNA	miR-140
t0036749	24	2	TACCACAGGGTAGAAAAACGGACA	miRNA	miR-140
t0036756	23	2	ACCACAGGGTAGAACCACGGGTA	miRNA	miR-140
t0036948	20	2	CCACGGGGTAGAACCACGGA	miRNA	miR-140
t0037078	20	2	CCACAGGGTAGAACCATGGA	miRNA	miR-140
t0037115	22	2	TACACAGGGTAGAACCACGGAA	miRNA	miR-140
t0037135	22	2	ACCACAGGGTAGAACCACTGAA	miRNA	miR-140
t0037187	23	2	ACTACAGGGTAGAACCACGGACA	miRNA	miR-140
t0037220	24	2	TACCACAGGGTAGAACCACGGCAT	miRNA	miR-140
t0037279	22	2	ACCACAGGGTATAACCACGGAA	miRNA	miR-140
t0037283	24	2	TACCACAGGGGAGAACCACGGAAA	miRNA	miR-140
t0037326	23	2	TACCACAGGGTAGTACCACGGCT	miRNA	miR-140
t0037331	22	2	CCACAGGGTAGAACAACGGACA	miRNA	miR-140
t0037343	24	2	TACCACAGGGTAGAACCCAGGACA	miRNA	miR-140
t0037348	23	2	TACCACAGGGCAGAACTACGGAA	miRNA	miR-140
t0037386	23	2	TACCACAGGGTAGAAACCCGGAC	miRNA	miR-140
t0037400	22	2	TACAACAGGGTAGAACTACGGA	miRNA	miR-140
t0037402	24	2	TACCACAGGGTAGGACCACGGAAA	miRNA	miR-140
t0037405	23	2	TACCACAGGGTAGAACCAAGGCC	miRNA	miR-140
t0037453	24	2	TACCACAGGGTAGAACTACGGAAA	miRNA	miR-140
t0037494	21	2	ACCACAGGGTAGAACACCGGA	miRNA	miR-140
t0037502	23	2	TACCACAGGGTAGAACACCGGAT	miRNA	miR-140
t0037522	21	2	AACACAGGGTAGAACAACGGA	miRNA	miR-140
t0037528	22	2	ACGACAGGGTAGAACCACGGAA	miRNA	miR-140
t0037535	22	2	ACCACAGGGTAGAACCTCGGAA	miRNA	miR-140
t0037604	22	2	ACCACAGGGTAGACCCACGGAT	miRNA	miR-140

t0037616	22	2	ACCACAGGGTAGAATCACGGCC	miRNA	miR-140
t0037659	23	2	ACCACAGGGTAGAACCAAGGACT	miRNA	miR-140
t0037739	24	2	TACCACAGGGTAGAACCACGGATG	miRNA	miR-140
t0037759	20	2	ACCACGGGGTAGAACCACGG	miRNA	miR-140
t0037767	23	2	TAGCACAGGGTAGAACCACGGAT	miRNA	miR-140
t0037776	22	2	ACCACAGGGTAGAACAAAGGAA	miRNA	miR-140
t0037790	22	2	ACCACAGGTTAGAACCACGGAC	miRNA	miR-140
t0037826	23	2	TACCACAGGGTAGAACCCCGGTA	miRNA	miR-140
t0037849	22	2	TACCACAGGGGATAACCACGGA	miRNA	miR-140
t0037902	24	2	TACCACAGGGTCGAACCACGGACA	miRNA	miR-140
t0037918	22	2	ACCACTGGGTAGAACCACGGAT	miRNA	miR-140
t0037921	23	2	ACCAAAGGGTAGAACCACGGAAT	miRNA	miR-140
t0037941	22	2	ACCACAGAGTAGAACCACGGAT	miRNA	miR-140
t0037961	24	2	TACCATAGGGTAGAACCACGGACG	miRNA	miR-140
t0037963	23	2	ACCACAGGGTAGAACAACGGAAA	miRNA	miR-140
t0037974	24	2	TACCACAGGGTAGAACCCCGGATC	miRNA	miR-140
t0038065	22	2	ACCACAGGGGAGAACCACGGCC	miRNA	miR-140
t0038103	22	2	TACCACAGGGTAGAACCGCGGC	miRNA	miR-140
t0038107	22	2	TACCAAAGGGTAGAAACACGGA	miRNA	miR-140
t0038183	22	2	TACCACAGGCTAGAACCCCGGA	miRNA	miR-140
t0038220	22	2	ACCACAGGGTAGAGCCACGGAA	miRNA	miR-140
t0038305	23	2	TACCACAAGGTAGAACCACGGCC	miRNA	miR-140
t0038318	24	2	TACCACAGGGTAGAAACACGGAAT	miRNA	miR-140
t0038378	22	2	ACCACAGGGTAGAACCACGCAA	miRNA	miR-140
t0038410	23	2	ACCACAGGGTAGAAACACGGACG	miRNA	miR-140
t0038431	23	2	ACCACAGGGTAGAACCAAGGAAG	miRNA	miR-140
t0038450	22	2	TACCACAGGGGAGAACTACGGA	miRNA	miR-140
t0038486	24	2	TACCTCAGGGTAGAACCACGGAAA	miRNA	miR-140
t0038523	23	2	TACCACAGGGTAGAAACACGGCC	miRNA	miR-140
t0038524	22	2	TACCACAGGGTAGCACCCCGGA	miRNA	miR-140
t0038546	23	2	ACCACGGGGTAGAACCACGGACT	miRNA	miR-140
t0038563	21	2	ACCACAGGGGAGAACCAAGGA	miRNA	miR-140
t0038636	21	2	ACCACAGGGGAGAACTACGGA	miRNA	miR-140
t0038664	22	2	TACCACAGGGTAGAACTAAGGA	miRNA	miR-140
t0038742	20	2	ACCACAGGGTAGAACCACCG	miRNA	miR-140
t0038765	21	2	ACCACAGGGTAGAACTAAGGA	miRNA	miR-140
t0038772	22	2	CCACAGGGTAGAACCAAGGACA	miRNA	miR-140
t0038788	21	2	ACACAGGGTAGAACCACGGAC	miRNA	miR-140
t0038807	23	2	ACCACAGGGTAGAGCCACGGACA	miRNA	miR-140
t0038849	22	2	ACCACAGGGCAGAACCACGGAG	miRNA	miR-140
t0039010	24	2	TACGACAGGGTAGAACCACGGACA	miRNA	miR-140
t0039033	24	2	TACCACAGGGCAGAACCACGGAAA	miRNA	miR-140
t0039134	23	2	ACCACAAGGTAGAACCACGGACG	miRNA	miR-140
t0039210	22	2	ACCACAGGGTATAACCACGGAT	miRNA	miR-140
t0039219	24	2	TACCACAGGGTAGAACCAGGGAAA	miRNA	miR-140
t0039251	24	2	TACCACCGGGTAGAACCACGGATA	miRNA	miR-140
t0039274	23	2	TTCCACAGGGTAGAACCACGGAC	miRNA	miR-140
t0039321	24	2	TACCACAGGGTAGAACTCCGGACA	miRNA	miR-140
t0039327	22	2	TACAACAGGGTAGAACCCCGGA	miRNA	miR-140
t0039371	25	2	TACCACAGGGTAGAACAACGGACAA	miRNA	miR-140
t0039392	23	2	ACCGCAGGGTAGAACCACGGACT	miRNA	miR-140
t0039401	21	2	CCACAGGGTAGAACAACGGAT	miRNA	miR-140
t0039408	22	2	TACCACAGGGTGGAACTACGGA	miRNA	miR-140
t0039409	24	2	TACCAGAGGGTAGAACCACGGCCA	miRNA	miR-140
t0039424	24	2	TACCACAGGGCAGAACCACGGACG	miRNA	miR-140
t0039470	21	2	ACCACAGGGTAGAACTACAGA	miRNA	miR-140
t0039471	23	2	TGCCACAGGGTAGAACCCCGGAC	miRNA	miR-140
t0039509	23	2	TACCACAGGGTAGAAACAAGGAT	miRNA	miR-140

t0039558	22	2	TACCATAGGGTAGAACCACGGC	miRNA	miR-140
t0039564	23	2	ACCACAGGGTAGAACCTCGGACT	miRNA	miR-140
t0039610	22	2	TCCACAGGGTAGAACCACGGAA	miRNA	miR-140
t0039638	20	2	ACCACACGGTAGAACCACGG	miRNA	miR-140
t0039654	22	2	ACCACAGGGTCGAACCACGGAC	miRNA	miR-140
t0039671	22	2	TATCACAGGGTAGAACCACGGC	miRNA	miR-140
t0039686	20	2	ACCACAGGGTAGAACCGCGG	miRNA	miR-140
t0039739	24	2	TACCACAGGGTAGAACAACGGACG	miRNA	miR-140
t0039742	21	2	GCCACAGGGTAGAACTACGGA	miRNA	miR-140
t0039747	24	2	TACCATAGGGTAGAACCAAGGACA	miRNA	miR-140
t0039768	22	2	TATCACAGGGTAGAACCCCGGA	miRNA	miR-140
t0039774	21	2	TACCACAGGGTAGAACCACAG	miRNA	miR-140
t0039786	22	2	ACCACAGGGTAGAACCACGGGG	miRNA	miR-140
t0039859	23	2	ACCACAGGGAAGAACCACGGAAA	miRNA	miR-140
t0039862	21	2	TACCACAGGGTAGAACCACGA	miRNA	miR-140
t0039891	20	2	TACCACAGGGTAGAACAACG	miRNA	miR-140
t0039923	22	2	TACCACAGGGTAGAACACCGGA	miRNA	miR-140
t0040000	23	2	ACCACAGGGAACAACCACGGACA	miRNA	miR-140
t0040020	23	2	TACCACAGGGCAGAACAACGGAC	miRNA	miR-140
t0040035	24	2	TACCACAGGGTAGAACCAAGGACT	miRNA	miR-140
t0040114	23	2	ACCACAGGGTAGAAACACGGATA	miRNA	miR-140
t0040133	23	2	ACCACAGGGCAGAACCACGGACG	miRNA	miR-140
t0040144	20	2	ACCACAGGGTAGGACCACGG	miRNA	miR-140
t0040161	21	2	TACCACAGGGTAGTACCACGG	miRNA	miR-140
t0040171	22	2	ACCACAGGGTACAACCACGGAT	miRNA	miR-140
t0040175	22	2	TAACACAGGGTAGAACCAAGGA	miRNA	miR-140
t0040215	21	2	ACCAAAGGGTAGAACAACGGA	miRNA	miR-140
t0040216	24	2	TACCACAGGGTAGAAACACGGAAA	miRNA	miR-140
t0040227	21	2	ACTACAGGGTAGAACCCCGGA	miRNA	miR-140
t0040279	23	2	TACCACAGGGTAGAAACCCGGAA	miRNA	miR-140
t0040294	20	2	ACCACAGGGTAGAACCAGGA	miRNA	miR-140
t0040309	22	2	ACCACAGGGTAGAACCACGTAC	miRNA	miR-140
t0040312	22	2	AGCACAGGGTAGAACCACGGAA	miRNA	miR-140
t0040368	22	2	TACCACAGGGGAGAACCACGGT	miRNA	miR-140
t0040370	22	2	TACCACGGGGTAGAACTACGGA	miRNA	miR-140
t0040407	21	2	ACCACAGGGTGGAATCACGGA	miRNA	miR-140
t0040449	22	2	CAGTGCAATGATGAAAAGGCAT	miRNA	miR-130b
t0040485	21	2	CAGAGCAATGTTAAAAGGGCA	miRNA	miR-130a
t0040497	21	2	CGGTGCAATGTTAAAAGGGCA	miRNA	miR-130a
t0040512	22	2	CAGTGCGATGTTAAAAGGGCAT	miRNA	miR-130a
t0040543	20	2	ACTCGGCGTGGCGTCGGCCG	miRNA	miR-1307
t0040559	20	2	ACTCGGCGTGGAGTCGGTCG	miRNA	miR-1307
t0040560	22	2	ACTCGGCGTGGCGTCGGTAGTG	miRNA	miR-1307
t0040592	18	2	TCCTGTGAGGTTGGCATT	miRNA	miR-1294
t0040605	24	2	TCTGGGCACCAAAGTGAGACCTGA	miRNA	miR-1285
t0040619	21	2	TCACAGTGAACCGGTCTATTT	miRNA	miR-128
t0040627	21	2	TCCCTGAGACTGATAATTGCT	miRNA	miR-125b
t0040637	22	2	CGGATGAACAAAGAAAGTGGTT	miRNA	miR-1255b
t0040651	21	2	GTGGGCGGGGGCAGGGGTGTG	miRNA	miR-1228*
t0037804	21	2	GTGAGGACTCGGGAGGAGGAG	miRNA	miR-1224
t0035342	20	2	GTAAGGACTCGGGAGGTGGA	miRNA	miR-1224
t0037987	20	2	GTGAGGACTCGGGAGGCGGA	miRNA	miR-1224
t0040469	22	2	TGGAGTGTGACAATGGGGTTTG	miRNA	miR-122
t0035354	26	2	TACCCTGTAGAACCGAATTTGTGATC	miRNA	miR-10d
t0037516	20	2	AGCAGCATTGTACAGGACTT	miRNA	miR-107b
t0038276	22	2	AGCAGCCTTGTACAGGGCTATA	miRNA	miR-107
t0035967	22	2	AGCAGCATTGTGCAGGGCTATT	miRNA	miR-107
t0035223	22	2	AGCGGCATTGTACAGGGCTATA	miRNA	miR-107

t0040141	22	2	AGCAGCGTTGTACAGGGCTATT	miRNA	miR-107
t0037772	22	2	AGCAGCATTGTACAAGGCTATT	miRNA	miR-107
t0036310	22	2	AGCAGCATTGTACAGGGCTCTA	miRNA	miR-107
t0036988	20	2	CCGCACTGTGGGTAATTGCT	miRNA	miR-106b*
t0036041	20	2	CCGCACTGTGGGAACTTGCT	miRNA	miR-106b*
t0036568	22	2	TACCGCAATGTGGGTACTTGCT	miRNA	miR-106b*
t0037027	22	2	TACCGCACTGTGGGTACTTGAT	miRNA	miR-106b*
t0039463	22	2	TACCGCACTGTAGGTACTTGCT	miRNA	miR-106b*
t0035973	24	2	CCGCACTGTGGGTACTTGCTGCAA	miRNA	miR-106b*
t0040016	22	2	TAAAGTGCTGACAGAGCAGATA	miRNA	miR-106b
t0036981	21	2	TAAAGTGCTGACAGTGCGGAA	miRNA	miR-106b
t0038108	20	2	TAAAGTGTTGACAGTGCAGA	miRNA	miR-106b
t0038363	21	2	TAAAGTACTGACAGTGCAGAT	miRNA	miR-106b
t0039607	21	2	TAAAGTGCTGACAGTGGAGAT	miRNA	miR-106b
t0039759	21	2	TAAAGTGCTGAGAGTGCAGAT	miRNA	miR-106b
t0040295	19	2	TAAAGTGCTGACAGTGCCG	miRNA	miR-106b
t0035216	20	2	TAAAGTGCTGCCAGTGCAGA	miRNA	miR-106b
t0036995	20	2	TAGAGTGCTGACAGTGCAGA	miRNA	miR-106b
t0037455	21	2	TAAAGTGCTGAAAGTGCAGAT	miRNA	miR-106b
t0037774	20	2	TAAAGTGCCGACAGTGCAGA	miRNA	miR-106b
t0038261	20	2	TACAGTGCTGACAGTGCAGA	miRNA	miR-106b
t0039524	23	2	AAAAGTGCTTACAGAGCAGGTAG	miRNA	miR-106
t0036207	22	2	AAAAGTGATTACAGTGCAGGTA	miRNA	miR-106
t0036221	21	2	AAAAGTGCTTACAGCGCAGGT	miRNA	miR-106
t0037086	23	2	AAAAGTGCTTACAGTGCCGGTAG	miRNA	miR-106
t0037103	21	2	AAAAGTGCTTACAGTGAAGGT	miRNA	miR-106
t0037209	21	2	AAAAGTGCTTACAGTGGAGGT	miRNA	miR-106
t0037286	23	2	AGAAGTGCTTACAGTGCAGGTAG	miRNA	miR-106
t0037358	21	2	AGCAGCAATGTACAGGGCTAT	miRNA	miR-103a
t0037851	20	2	AGCAGCATTTTACAGGGCTA	miRNA	miR-103a
t0037981	21	2	AGCAGCATTGTACAGGGATAA	miRNA	miR-103a
t0038027	23	2	AGCAGCGTTGTACAGGGCTATGT	miRNA	miR-103a
t0038669	19	2	AGCAGCATTGGACAGGGCT	miRNA	miR-103a
t0038755	23	2	AGCAGCATTGTACAGGGCTACGT	miRNA	miR-103a
t0035970	23	2	AGCAGCATAGTACAGGGCTATGA	miRNA	miR-103a
t0036034	23	2	AGGAGCATTGTACAGGGCTATGT	miRNA	miR-103a
t0037949	23	2	AGCAGCATTGTACAGGGCAATGA	miRNA	miR-103a
t0038391	23	2	AGCAGCTTTGTACAGGGCTATGA	miRNA	miR-103a
t0038892	23	2	AGCAGCATTGTATAGGGCTATGT	miRNA	miR-103a
t0039476	21	2	AGCAGCATTGTACAGGGCTAC	miRNA	miR-103a
t0039601	24	2	TAGCAGCATTGTACAGGGCTATGA	miRNA	miR-103a
t0035244	23	2	AGCAGCATCGTACAGGGCTATGT	miRNA	miR-103a
t0035436	22	2	AGAAGAATTGTACAGGGCTATG	miRNA	miR-103a
t0035481	24	2	AAGCAGCATTGTACAGGGCTCTGA	miRNA	miR-103a
t0035550	24	2	AGCAGGATTGTACAGGGCTATGAA	miRNA	miR-103a
t0035552	22	2	AGAAACATTGTACAGGGCTATG	miRNA	miR-103a
t0035682	20	2	AGCAGCAATGTACAGGGCTA	miRNA	miR-103a
t0035913	23	2	AGCAGCATTGTACCGGGCTATGT	miRNA	miR-103a
t0035943	23	2	AGCAGCATTGTACTGGGCTATGT	miRNA	miR-103a
t0035965	24	2	AGAAGCATTGTACAGGGCTATGAA	miRNA	miR-103a
t0035997	21	2	AGCAGCATTGTTCAGGGCTAT	miRNA	miR-103a
t0036040	23	2	AGCAGCATTTTACAGGGCTATGA	miRNA	miR-103a
t0036222	23	2	AGCAGCATTGTGCAGGGCTATGT	miRNA	miR-103a
t0036709	19	2	AGCAGCATTGTAAAGGGCT	miRNA	miR-103a
t0036946	24	2	AGCAGCATTGTACCGGGCTATGAT	miRNA	miR-103a
t0037048	23	2	AGCAGCATTGTACAGGTCTATGA	miRNA	miR-103a
t0037352	20	2	AGCAGCTTTGTACAGGGCTA	miRNA	miR-103a
t0037510	22	2	AGAAGCATTGTAAAGGGCTATG	miRNA	miR-103a

t0037811	20	2	AGCAGCATTGTAGAGGGCTA	miRNA	miR-103a
t0037846	22	2	AGCAGCTTTGTACAGGGCTATG	miRNA	miR-103a
t0037864	22	2	TAGCAGCATTGTACAGGGCTAT	miRNA	miR-103a
t0038443	23	2	AGCGGCATTGTACAGGGCTATGT	miRNA	miR-103a
t0038548	23	2	AGCAGCCTTGTACAGGGCTATGT	miRNA	miR-103a
t0038677	21	2	GAACAGTACTGTGATAACTGA	miRNA	miR-101c
t0038813	22	2	CACAGTACTGTGATAACTGATT	miRNA	miR-101c
t0038972	21	2	CAGTACTGTGATAACTGAAGA	miRNA	miR-101c
t0039107	26	2	GGACAGTACTGTGATAACTGACGGGT	miRNA	miR-101c
t0039149	22	2	CACAGTACTGTGATAACTGAAA	miRNA	miR-101c
t0039187	22	2	TACAGTACTGTGATAAATGACT	miRNA	miR-101c
t0039496	22	2	TACAGTACTATGATAACTGAAA	miRNA	miR-101b
t0039811	22	2	GTACAGTCCTGTGATAACTGAA	miRNA	miR-101
t0039868	22	2	GTACAGTACGGTGATAACTGAA	miRNA	miR-101
t0039914	22	2	TACAGTACTGAGATAACTGAAG	miRNA	miR-101
t0040192	21	2	GTACAGTACTGTGGTAACTGA	miRNA	miR-101
t0040395	22	2	TACAGTACTGTGATAGCTGAAA	miRNA	miR-101
t0040583	22	2	TACAATACTGTGATAACTGAAG	miRNA	miR-101
t0036144	21	2	GTACAGTACTGTGTTAACTGA	miRNA	miR-101
t0036684	22	2	GTACTGTACTGTGATAACTGAA	miRNA	miR-101
t0037852	22	2	GTACCGTACTGTGATAACTGAA	miRNA	miR-101
t0038366	22	2	TATAGTACTGTGATAACTGAAG	miRNA	miR-101
t0039278	20	2	TACCGTACTGTGATAACTGA	miRNA	miR-101
t0040199	23	2	GTACAGTACTGTGATAACTGCAA	miRNA	miR-101
t0037622	26	2	TACAGTACTGTGATAACTGAAGTGGC	miRNA	miR-101
t0035196	21	2	GTACAGTACTGTCATAACTGA	miRNA	miR-101
t0035228	22	2	GTACAGTACTGTGATCACTGAA	miRNA	miR-101
t0035259	21	2	TACAGTACTGTGATAACAGAA	miRNA	miR-101
t0035351	20	2	TACAGTACTGTGATAGCTGA	miRNA	miR-101
t0035478	21	2	TACAGTACTGTGATATCTGAA	miRNA	miR-101
t0035543	22	2	TACAGTACTGTGACAACTGAAA	miRNA	miR-101
t0035588	21	2	GTACAGTACTGTGATGACTGA	miRNA	miR-101
t0035617	22	2	GTACAGTACTGTGATAACTGTA	miRNA	miR-101
t0035721	22	2	TACGGTACTGTGATAACTGAAA	miRNA	miR-101
t0035772	22	2	TACAGTACTGTGATAATTGAAG	miRNA	miR-101
t0035797	22	2	TACAGTACTGTAATAACTGAAG	miRNA	miR-101
t0035991	21	2	GTACAGTACTGTGATACCTGA	miRNA	miR-101
t0036053	23	2	ATACAGTACTGTGATAACTGAAT	miRNA	miR-101
t0036064	22	2	TACAGTGCTGTGATAACTGAAG	miRNA	miR-101
t0036166	20	2	TACAGTACTGTGATAACTTA	miRNA	miR-101
t0036353	20	2	TACAGTACTGTGATGACTGA	miRNA	miR-101
t0036562	25	2	TACAGTACTGTGATAACTGAAAAAA	miRNA	miR-101
t0036735	23	2	GTACAGTACTGTGGTAACTGAAA	miRNA	miR-101
t0036787	22	2	GTACAGTACTCTGATAACTGAA	miRNA	miR-101
t0037246	20	2	TACAGTACTGAGATAACTGA	miRNA	miR-101
t0037762	22	2	GTACAGTACTGTGATAACAGAA	miRNA	miR-101
t0037766	21	2	GTACAGTACTGTGATAACTGT	miRNA	miR-101
t0037867	22	2	TACAGTACTGTGATAATTGAAA	miRNA	miR-101
t0037977	22	2	TACAGTACTGAGATAACTGAAA	miRNA	miR-101
t0038059	20	2	TACAGTAGTGTGATAACTGA	miRNA	miR-101
t0038092	22	2	TACAGTCCTGTGATAACTGAAA	miRNA	miR-101
t0038182	20	2	TACAGTGCTGTGATAACTGA	miRNA	miR-101
t0038491	21	2	GTAAAGTACTGTGATAACTGA	miRNA	miR-101
t0038731	20	2	TACAGTACTGTGAAAACTGA	miRNA	miR-101
t0039018	20	2	TACAGTACTGTGCTAACTGA	miRNA	miR-101
t0039021	21	2	GTACAATACTGTGATAACTGA	miRNA	miR-101
t0039267	24	2	TACAGTACTGTGATAAATGAAGAA	miRNA	miR-101
t0039313	23	2	ATACAGTACTGTGATAACTGAAA	miRNA	miR-101

t0039412	25	2	TGGAATGTAAAGAAGTGGGTATATC	miRNA	miR-1
t0039712	25	2	TGGAATGTAAAGAAGTGTGTATGTC	miRNA	miR-1
t0039725	22	2	TGAGGTAGTAGATTGAATACTT	miRNA	let-7k
t0039749	23	2	TGAGGTAGTAGATTGAATAGTTT	miRNA	let-7k
t0039847	22	2	TCAGGTAGTAGTTTGTGCTGTT	miRNA	let-7i
t0039947	20	2	TGAGGTAGTAGTTTGTGCTA	miRNA	let-7i
t0039975	20	2	TGAGGTAGTAGCTTGTGCTG	miRNA	let-7i
t0040008	20	2	TGAGGGAGTAGTTTGTGCTT	miRNA	let-7i
t0040244	21	2	TGAGGTAGTAGTTTGTGCCGT	miRNA	let-7i
t0040269	20	2	TGACGTAGTAGTTTGTGCTG	miRNA	let-7i
t0040282	19	2	TGAGGTAGTAGATTGTGCT	miRNA	let-7i
t0040505	21	2	TGAGGTAGTAGTTTTTGCTGT	miRNA	let-7i
t0040536	20	2	TGAGGTAGTAGTTTCTGCTG	miRNA	let-7i
t0038826	18	2	TGAGGGAGTAGTTTGTGC	miRNA	let-7i
t0039905	21	2	TGAGGTAGTAGTTTCTGCTGT	miRNA	let-7i
t0037105	22	2	TGAGGTAGTAGTTTGTGCTGCT	miRNA	let-7i
t0039077	20	2	TGAGCTAGTAGTTTGTGCTG	miRNA	let-7i
t0035215	21	2	CGAGGTAGTAGTTTGTGCTGT	miRNA	let-7i
t0035226	23	2	TGAGGGAGTAGTTTGTGCTGTAA	miRNA	let-7i
t0035330	23	2	TGAGGTAGCAGTTTGTGCTGTTA	miRNA	let-7i
t0035582	22	2	TGAGGTAGTAGTGTGTGCTGTT	miRNA	let-7i
t0035690	23	2	TGAGGTACTAGTTTGTGCTGTTT	miRNA	let-7i
t0036022	23	2	TGGGGTAGTAGTTTGTGCTGTTA	miRNA	let-7i
t0036194	22	2	TGAGGTAGTACTTTGTGCTGTT	miRNA	let-7i
t0036259	20	2	TGAGGTAGTAGTTTGGGCTG	miRNA	let-7i
t0036405	20	2	TGAGGTAGTAGTCTGTGCTG	miRNA	let-7i
t0036421	21	2	TGAGGTAGTAGTTCGTGCTGT	miRNA	let-7i
t0036587	19	2	TGGGGTAGTAGTTTGTGCT	miRNA	let-7i
t0036694	19	2	TGAGGTAGTAGTTTGTGGT	miRNA	let-7i
t0037117	23	2	TGAGGTAGTAGTTTGTGCTGGTT	miRNA	let-7i
t0037427	22	2	TGAGTTAGTAGTTTGTGCTGTT	miRNA	let-7i
t0037442	23	2	TGAGGAAGTAGTTTGTGCTGTTA	miRNA	let-7i
t0037565	22	2	TGAGGTAGTAGTTTGTGCTGAT	miRNA	let-7i
t0037658	23	2	TGGGGTAGTAGTTTGTGCTGTTT	miRNA	let-7i
t0037691	21	2	TGAGGTAGCAGTTTGTGCTGT	miRNA	let-7i
t0037742	19	2	TGAGGTAGTAGATTGTGGT	miRNA	let-7i
t0038144	22	2	TGAGGTAGTAGTTTCTGCTGTT	miRNA	let-7i
t0038283	20	2	TGGGGTAGTAGTTTGTGCTG	miRNA	let-7i
t0038473	19	2	TGAGGTAGTAGCTTGTGCT	miRNA	let-7i
t0038767	20	2	CGAGGTAGTAGTTTGTGCTG	miRNA	let-7i
t0038824	23	2	TGAGGTGGTAGTTTGTGCTGTTT	miRNA	let-7i
t0038828	20	2	TGAGGTAGTAGTTTGTACCG	miRNA	let-7g
t0038949	22	2	TGAGGTAGTAGTTGGTACAGTT	miRNA	let-7g
t0039014	23	2	TGAGGAAGTAGTTTGTACAGTTA	miRNA	let-7g
t0039399	21	2	GAGGGAGTAGTTTGTACAGTT	miRNA	let-7g
t0039475	23	2	TGAGGGAGTAGTTTGTACAGTTG	miRNA	let-7g
t0039545	22	2	TGAGGTAGGAGTTTGTGCAGTT	miRNA	let-7g
t0039640	20	2	TGAGGTAGGAGTTTGTACAG	miRNA	let-7g
t0039864	21	2	TGTGGTAGTAGTTTGTACAGT	miRNA	let-7g
t0040149	22	2	TGAGGTAGTAGTTTGTACCGTA	miRNA	let-7g
t0040302	22	2	TGAGGGAGTAGTTTGTGCAGTT	miRNA	let-7g
t0040306	23	2	TGAGGTAGTAGTTTGTAAAGTTA	miRNA	let-7g
t0040342	20	2	TGAGGTAGAAGTTTGTACAG	miRNA	let-7g
t0040419	23	2	TGAGGTAATAGTTTGTACAGTTA	miRNA	let-7g
t0036155	22	2	TGAGGTAGTAGTGTGTACAGTT	miRNA	let-7g
t0036261	21	2	TGAGGTAGTAGTTTGAACAGT	miRNA	let-7g
t0036315	21	2	TGAGGTAATAGTTTGTACAGT	miRNA	let-7g
t0036373	22	2	TGAGGTAGTAGTTTGTACAGTG	miRNA	let-7g

t0036509	21	2	TGAGGTAGTAATTTGTACAGT	miRNA	let-7g
t0036768	20	2	TGAGGTAGTAGATTGTAGGT	miRNA	let-7f-5p
t0036914	20	2	CGAGGTAGTAGATTGTATTT	miRNA	let-7f-5p
t0036951	20	2	TGAGGTAGCAGATTGTATTT	miRNA	let-7f-5p
t0037439	18	2	TGAGGTAGTGGATTGTAT	miRNA	let-7f-5p
t0037496	22	2	TGAGGTAGTCGATTGTATCGTT	miRNA	let-7f-5p
t0037567	22	2	TGAGGTAGTGGATTGTATTGTT	miRNA	let-7f-5p
t0037705	22	2	TGAGGTAGTGGATTGTATATTT	miRNA	let-7f-5p
t0038575	21	2	TGAGGTAGTGGATTGTATAGG	miRNA	let-7f-5p
t0038626	21	2	TGAGGTAGTAGATTGTATCGG	miRNA	let-7f-5p
t0038926	22	2	TGAGGTAGTAGATTGTATAGAG	miRNA	let-7f-5p
t0039141	23	2	TGAGGTAGTAGATTGTATGGTAA	miRNA	let-7f-5p
t0040168	22	2	TGAGGTAGTAGATTGTATCCTT	miRNA	let-7f-5p
t0040241	22	2	TGAGGTAGTCGATTGTATAGGT	miRNA	let-7f-5p
t0035697	21	2	TGAGGTAGTGGATTGTATAGA	miRNA	let-7f-5p
t0035795	20	2	TAAGGTAGTAGATTGTATTT	miRNA	let-7f-5p
t0036316	22	2	TGAGGTAGTGGTTTGTATAGTT	miRNA	let-7f-5p
t0036530	22	2	TGAGGAAGTGGATTGTATAGTT	miRNA	let-7f-5p
t0037091	20	2	TGAGGTAGTAGATTGTACGT	miRNA	let-7f-5p
t0037266	18	2	TGAGGTAGTGGATTGTAG	miRNA	let-7f-5p
t0037274	24	2	TGAGGTAGTAGATTGTATAGTGGC	miRNA	let-7f
t0037901	22	2	TGGGGTAGTAGATTGTATAGCT	miRNA	let-7f
t0038008	22	2	TGAGGTAGCAGATTGTATAGCT	miRNA	let-7f
t0038062	23	2	TGAGGTAGTAGATTGTATAGATG	miRNA	let-7f
t0038126	23	2	TGAGGTAATAGATTGTATAGTTG	miRNA	let-7f
t0038389	21	2	GTAGTAGATTGTATAGTATGA	miRNA	let-7f
t0038465	21	2	TGAGGTAGTAGATTGTTTGGT	miRNA	let-7f
t0039179	21	2	TGCGGTAGTAGCTTGTATAGT	miRNA	let-7f
t0039198	23	2	TGTGGTAGTAGATTGTATAGTTG	miRNA	let-7f
t0039803	22	2	TGAGCTAGTAGATTGTATAGTA	miRNA	let-7f
t0039934	22	2	TGAGCTAGTAGATTGTGTAGTT	miRNA	let-7f
t0040384	22	2	TGAGGTAGAAGATTGTATACTT	miRNA	let-7f
t0040496	22	2	TGAGGTAGTAGCTTGTAAAGTT	miRNA	let-7f
t0035214	24	2	CGAGGTAGTAGATTGTATAGTTAA	miRNA	let-7f
t0035257	23	2	TGAGGTAGTAGATTGTATTGTTT	miRNA	let-7f
t0035321	22	2	TGAGGTGGTAGATTGTATAGGT	miRNA	let-7f
t0035428	18	2	TGCGGTAGTAGATTGTAT	miRNA	let-7f
t0035474	21	2	TTGAGGAAGTAGATTGTATAG	miRNA	let-7f
t0035488	23	2	TGAGGTAGTAGATTTTATAGTTA	miRNA	let-7f
t0035512	21	2	GAGGTAGTAGATTGTATGGTT	miRNA	let-7f
t0035529	22	2	TGAGGTAGTATATTGTATAGGT	miRNA	let-7f
t0035554	23	2	TAAGGTAGTAGATTGTATAGTTG	miRNA	let-7f
t0035606	22	2	TGAGGTAGAAGATTGTATAGGT	miRNA	let-7f
t0035651	22	2	TGTGGTAGTAGATTGTATAGGT	miRNA	let-7f
t0035653	21	2	ATGAGGTAGTAGATTGTCTAG	miRNA	let-7f
t0035656	23	2	CTGAGGTAGTAGATTGTATAGTA	miRNA	let-7f
t0035677	24	2	TGAGGTAGTAGATTGTATGGTTGA	miRNA	let-7f
t0035777	22	2	TGAGGTAGTAGATTGTACATTT	miRNA	let-7f
t0035799	20	2	GAGGTAGTAGATTGTATCGT	miRNA	let-7f
t0035871	22	2	TGCGGTAGTAGATTGTATAGGT	miRNA	let-7f
t0035878	21	2	TGAGGTAGTAGCTTGTGTAGT	miRNA	let-7f
t0035905	22	2	TGAGGTTGTAGATTGTTTAGTT	miRNA	let-7f
t0035916	21	2	TGAGGTACTAGATTGTGTAGT	miRNA	let-7f
t0035942	22	2	TGAAGTAGTAGATTGTATAGTA	miRNA	let-7f
t0036029	22	2	TGGGGTAGTAGATTGCATAGTT	miRNA	let-7f
t0036091	22	2	TGAGGTAGTAGCTTGTATATTT	miRNA	let-7f
t0036116	21	2	TGAGGTAGTATATTGTATAGG	miRNA	let-7f
t0036134	22	2	TGAGGCAGTAGATTATATAGTT	miRNA	let-7f

t0036282	22	2	TGAGGTAGAAGATTGTATATTT	miRNA	let-7f
t0036313	22	2	TGAGGTAGCAGATTGTATAGGT	miRNA	let-7f
t0036318	22	2	CGGGGTAGTAGATTGTATAGTT	miRNA	let-7f
t0036361	23	2	TGAGGTAGTAGATTGTATAATTA	miRNA	let-7f
t0036367	20	2	AAGGTAGTAGATTGTATAGT	miRNA	let-7f
t0036416	23	2	TGAGGTAGTCGATTGTATAGTTG	miRNA	let-7f
t0036439	22	2	TGAGGTAGTAGCTTGTATAGAT	miRNA	let-7f
t0036501	21	2	TGAGGTAGTAGATTGCATAGA	miRNA	let-7f
t0036540	21	2	TAGGTAGTAGATTGTATCGTT	miRNA	let-7f
t0036567	23	2	TGAAGTAGTAGATTGTATAGTTT	miRNA	let-7f
t0036579	23	2	TGAGCTAGTAGATTGTATAGTTA	miRNA	let-7f
t0036580	23	2	AGAGGTAGTAGATTGTATAGTTA	miRNA	let-7f
t0036633	22	2	TGGGGTAGTAGCTTGTATAGTT	miRNA	let-7f
t0036704	22	2	TGCGGTAGTAGATTGTCTAGTT	miRNA	let-7f
t0036727	21	2	TGAGGTAGTAGCTTCTATAGT	miRNA	let-7f
t0036730	22	2	TGAGGCAGTAGATTGCATAGTT	miRNA	let-7f
t0036758	18	2	CGAGGTAGTAGATTGTAG	miRNA	let-7f
t0036769	22	2	TGAGGTGGTAGATTATATAGTT	miRNA	let-7f
t0036786	22	2	TGAGGTAGCAGATTGTTTAGTT	miRNA	let-7f
t0036913	22	2	TGAGGTAGTAGACTGTATAGGT	miRNA	let-7f
t0036925	22	2	TGCGGTCGTAGATTGTATAGTT	miRNA	let-7f
t0036986	20	2	TGAGGTAGTAGATTGCGTAG	miRNA	let-7f
t0037013	23	2	TGAGGTAGTATATTGTATAGTTG	miRNA	let-7f
t0037028	22	2	TGAGGAAGCAGATTGTATAGTT	miRNA	let-7f
t0037034	22	2	TGAGGTAGTAGATTGTAAAGTA	miRNA	let-7f
t0037092	20	2	TGAGGTGGTAGATTGTGTAG	miRNA	let-7f
t0037188	22	2	TGCGGTAGTAGATTGTTTAGTT	miRNA	let-7f
t0037226	22	2	TGAGGTGGAAGATTGTATAGTT	miRNA	let-7f
t0037319	22	2	TGAGGTAGTAGATTGGAGAGTT	miRNA	let-7f
t0037363	22	2	TGAGATAGTAGATTGTGTAGTT	miRNA	let-7f
t0037364	21	2	TGGGGTAGTAGATTGTATTGT	miRNA	let-7f
t0037406	23	2	TGAGGTAGTAGATTGTATAGATA	miRNA	let-7f
t0037490	18	2	TGAGGTAGTAGAATGTAG	miRNA	let-7f
t0037499	22	2	TTGAGGAAGTAGATTGTATAGT	miRNA	let-7f
t0037566	22	2	TGAGGTAGTAGATTGGATAGGT	miRNA	let-7f
t0037633	22	2	TGAGGTAGTAGATTGTCTGGTT	miRNA	let-7f
t0037634	22	2	TGAGGTAATAGATTGTATAGTA	miRNA	let-7f
t0037645	23	2	TCAGGTAGTAGATTGTATAGTTA	miRNA	let-7f
t0037792	21	2	GAGGTAGTTGATTGTATAGTT	miRNA	let-7f
t0037806	18	2	TGAGGTAGAAGATTGTAG	miRNA	let-7f
t0037818	22	2	TGAGGAAGTAGATTGCATAGTT	miRNA	let-7f
t0037834	21	2	TGAGGTAGTAGTTTGTATTGT	miRNA	let-7f
t0037892	22	2	TGAGGTAGTAGATTGGGTAGTT	miRNA	let-7f
t0037893	21	2	GAGGTAGTAGATTGTATAGTC	miRNA	let-7f
t0037947	22	2	CGAGGTAGTAGATTGTGTAGTT	miRNA	let-7f
t0038007	22	2	TGAGGTAGTAGATTGTGTAGGT	miRNA	let-7f
t0038020	21	2	TGAGGTAGTCGCTTGTATAGT	miRNA	let-7f
t0038051	23	2	TGAGGTAGTATATTGTATAGTTA	miRNA	let-7f
t0038067	23	2	TGAGGTAGAAGATTGTATAGTAA	miRNA	let-7f
t0038115	23	2	TGAGGTAGTAGTTTGTATAGTTT	miRNA	let-7f
t0038158	22	2	TGAGGTGGTAGATTGTATGGTT	miRNA	let-7f
t0038187	22	2	TAAGGTAGTAGATTGTATATTT	miRNA	let-7f
t0038193	22	2	TGAGGTGGTAGATCGTATAGTT	miRNA	let-7f
t0038199	18	2	TGAGGTAGTAGCTTGTAT	miRNA	let-7f
t0038232	22	2	TGGGGTAGTAGATTGTATAGGT	miRNA	let-7f
t0038285	23	2	TGAGATAGTAGATTGTATAGTTT	miRNA	let-7f
t0038341	21	2	GAGGTCGTAGATTGTATAGTT	miRNA	let-7f
t0038390	22	2	TGAGGTAGTAGATTGTCTAGGT	miRNA	let-7f

t0038507	22	2	TGATGTAGTAGATTGTATGGTT	miRNA	let-7f
t0038597	19	2	TGAGGTAGTAGATCGTATG	miRNA	let-7f
t0038628	23	2	TGAGGTAGTAGATTGTATAGCTC	miRNA	let-7f
t0038640	23	2	TGAGGTAGTACATTGTATAGTTT	miRNA	let-7f
t0038644	22	2	TGAGGTACTAGATTGTATAGCT	miRNA	let-7f
t0038719	23	2	TGAGGTAGTAGATTATATAGTTT	miRNA	let-7f
t0038723	22	2	TGAGGCAGCAGATTGTATAGTT	miRNA	let-7f
t0038795	19	2	TGAGGTAGTAGAATGTATA	miRNA	let-7f
t0038838	22	2	TGAGGAAGTAGATTGGATAGTT	miRNA	let-7f
t0038879	23	2	TCAGGTAGTAGATTGTATAGTTT	miRNA	let-7f
t0038923	22	2	TGAGGTAGTCGATTGGATAGTT	miRNA	let-7f
t0038948	21	2	TGAGGTAGTAGATTGCATATT	miRNA	let-7f
t0038965	21	2	TGAGGAAGTAGATTGTGTAGT	miRNA	let-7f
t0038989	22	2	TGGGGTAGTAGATTGTATACTT	miRNA	let-7f
t0039027	21	2	TGGGGTAGTAGATTGTATAGC	miRNA	let-7f
t0039075	22	2	TGAGGTAGCAGATTGTATATTT	miRNA	let-7f
t0039096	22	2	TGAGGTCGTAGATTGTATAGTA	miRNA	let-7f
t0039128	22	2	AGAGGTAGTAGATTGTATAGTA	miRNA	let-7f
t0039190	23	2	TGAGGTAGTAGATTGTATCGTTT	miRNA	let-7f
t0039197	22	2	TGAGGTAGTAGATCGTATGGTT	miRNA	let-7f
t0039231	23	2	TGAAGTAGTAGATTGTATAGTTG	miRNA	let-7f
t0039258	22	2	TGAGGTCGTAGATTGTATAGAT	miRNA	let-7f
t0039284	21	2	TGAGGTAGTAGCTTGTATCGT	miRNA	let-7f
t0039309	19	2	TGAGGTAGTCGATTGTATA	miRNA	let-7f
t0039324	23	2	TGAGGTCGTAGATTGTATAGTTA	miRNA	let-7f
t0039396	22	2	TGAGGTACTAGATTGTATAGTA	miRNA	let-7f
t0039485	22	2	TGAGGTAGTAGATTATATAGTA	miRNA	let-7f
t0039507	19	2	GAGGTAGTAGATTATATAG	miRNA	let-7f
t0039578	23	2	TGAGGTAGTAGCTTGTATAGTTT	miRNA	let-7f
t0039593	22	2	TTGAGGCAGTAGATTGTATAGT	miRNA	let-7f
t0039597	22	2	TTAGGTGGTAGATTGTATAGTT	miRNA	let-7f
t0039653	23	2	GGAGGTAGTAGATTGTATAGTTA	miRNA	let-7f
t0039661	23	2	TGAGGTAGTAAATTGTATAGTTG	miRNA	let-7f
t0039869	23	2	CTGAGGTAGAAGATTGTATAGTT	miRNA	let-7f
t0039875	23	2	TGAGGTAGTAGATTCTATAGTTG	miRNA	let-7f
t0039957	22	2	TGAGGTAGTAGATTACATAGTT	miRNA	let-7f
t0039987	22	2	TGAGGTCGTAGATTGTGTAGTT	miRNA	let-7f
t0040036	22	2	TGAGGTGGGAGATTGTATAGTT	miRNA	let-7e
t0040090	21	2	TGAGGTAGGAGAGTGTATAGT	miRNA	let-7e
t0040111	21	2	TGAGGTCGGAGATTGTATAGT	miRNA	let-7e
t0040178	20	2	TGAGGTAGGAGATTGTATTT	miRNA	let-7e
t0040205	22	2	TGAGATAGGAGATTGTATAGTT	miRNA	let-7e
t0040338	18	2	TGAGGTAGGAGATTGTAG	miRNA	let-7e
t0040355	22	2	TGAGGTAGGAGATTGTATAGTA	miRNA	let-7e
t0040426	21	2	TGAGGTAGGAGATTGTGTAGT	miRNA	let-7e
t0040437	19	2	AGAGGTAGTAGGTTGAATA	miRNA	let-7d
t0040447	21	2	AGAGGTAGTACGTTGCATAGT	miRNA	let-7d
t0040580	22	2	AGAGGTAGTAGGTTGCATAATT	miRNA	let-7d
t0040586	23	2	AGAGGAAGTAGGTTGCATAGTTT	miRNA	let-7d
t0040634	22	2	AGAGGTAGTGGGTTGCATAGTA	miRNA	let-7d
t0035371	21	2	AGAGGTAGTAGGGTGCATAGA	miRNA	let-7d
t0036025	22	2	AGATGTAGTAGGTTGCATAGTT	miRNA	let-7d
t0036918	22	2	AGAGGTAGTAGGTTTCATAGTT	miRNA	let-7d
t0037261	19	2	AGGGGTAGTAGGTTGCATA	miRNA	let-7d
t0037451	23	2	AGAGGGAGTAGGTTGCATAGTTA	miRNA	let-7d
t0038353	19	2	AGAGGTAGTAGATTGCATA	miRNA	let-7d
t0038642	20	2	CGAGGTAGTAGGTTGCATAG	miRNA	let-7d
t0038897	24	2	AGAGGTAGTAGGTTGCATAGTAGA	miRNA	let-7d

t0035225	22	2	AGAGGTAGTAGCTTGCATAGTT	miRNA	let-7d
t0035278	21	2	AGATGTAGTAGGTTGCATAGT	miRNA	let-7d
t0036158	23	2	AGAGGTAGTAGGGTGCATAGTTT	miRNA	let-7d
t0036418	21	2	AGAGGTAGTAGGTTTCATAGT	miRNA	let-7d
t0036485	21	2	AGAGGGAGTAGGGTGCATAGT	miRNA	let-7d
t0036577	19	2	AGAGGTAGTAGGTCGCATA	miRNA	let-7d
t0036665	23	2	AGAGGGAGTAGGTTGCATAGTTT	miRNA	let-7d
t0036884	23	2	AGAGGTAGTAGGTTGAATAGTTA	miRNA	let-7d
t0037061	20	2	AGAGGTAGTAGGTTGCAAAG	miRNA	let-7d
t0037280	19	2	AGAGGTAGTAGGATGCATA	miRNA	let-7d
t0037282	19	2	GGAGGTAGTAGGTTGCATA	miRNA	let-7d
t0037325	21	2	AGACGTAGTAGGTTGCATAGT	miRNA	let-7d
t0037478	25	2	TGAGGTAGTAGGATGTATGGTTATC	miRNA	let-7c
t0037558	25	2	TGAGGTAGTAGCTTGTATGGTTATC	miRNA	let-7c
t0037694	22	2	TGAGGTAGTAGGTTGCATGGTT	miRNA	let-7c
t0038047	22	2	TGAGGAAGTAGGTTGTATGGTT	miRNA	let-7c
t0038201	21	2	TGAGGTAGTAGCTTGTATGGT	miRNA	let-7c
t0039446	25	2	CGAGGTAGTAGGTTGTATGGTTATC	miRNA	let-7c
t0039568	25	2	TGAGGTAGTAGGTTGCATGGTTATC	miRNA	let-7c
t0039647	22	2	TGAGGTAGTAGGTTGGATGGTT	miRNA	let-7c
t0039651	22	2	TGAGGTAGTAGGTTATATGGTT	miRNA	let-7c
t0039795	22	2	CGAGGTAGTAGGTTGTATGGTT	miRNA	let-7c
t0040347	22	2	TGAGGTTGTAGATTGTATGGTT	miRNA	let-7c
t0040500	21	2	TAAGGTAGTAGGTTGTATGGT	miRNA	let-7c
t0040612	21	2	TGAGGTAGTAGGCTGTATGGT	miRNA	let-7c
t0035340	25	2	TGGGGTAGTAGGTTGTATGGTTATC	miRNA	let-7c
t0036354	23	2	TGAGGTAGTAGGTTGTGAGGTTT	miRNA	let-7b
t0036381	19	2	TGTGGTAGTAGGTTGTGTG	miRNA	let-7b
t0036585	21	2	TGAGGTACTAGATTGTGTGGT	miRNA	let-7b
t0036693	24	2	TGAGGTAGTAGGTTATGTGGTTAA	miRNA	let-7b
t0037380	24	2	TGGGGTAGTAGGTTGTGTGGTTAG	miRNA	let-7b
t0037989	23	2	TGAGGTAGGAGGTTGTGTGGTTT	miRNA	let-7b
t0038345	23	2	TGAGGTAGTAGGTTGTATGGCTC	miRNA	let-7b
t0038858	24	2	TGAGGTAGTAGGTTGTGTGGGTAG	miRNA	let-7b
t0038875	24	2	TGAGGTAGTAGGTTGTGTGGTCAT	miRNA	let-7b
t0039009	19	2	TGAGCTAGTAGGTTGTGTG	miRNA	let-7b
t0039362	22	2	TGAGGCAGTAGGCTGTGTGGTT	miRNA	let-7b
t0039491	22	2	TGGGGTGGTAGGTTGTGTGGTT	miRNA	let-7b
t0040136	21	2	TGAGGTAGTAGGTTGTTGGGT	miRNA	let-7b
t0035200	22	2	TGAGGTAGTAGGTTGCGTAGTT	miRNA	let-7b
t0035236	23	2	TGAGGTAGTAGGTCGTGTGGTTT	miRNA	let-7b
t0035253	22	2	TGAGGAAGTCGGTTGTGTGGTT	miRNA	let-7b
t0035283	24	2	TGAGGTAGAAGGTTGTGTGGTTAA	miRNA	let-7b
t0035286	23	2	TGAGGTAGTAGGTTGTGTGGATA	miRNA	let-7b
t0035386	24	2	TGGGGTAGTAGGTTGTGTGGTTAA	miRNA	let-7b
t0035447	22	2	TGAGGTAGTAGGCTGTGGGGTT	miRNA	let-7b
t0035541	21	2	TAAGGTAGTAGGTTGTGTGGA	miRNA	let-7b
t0035766	23	2	TGAGGTCGTAGGTTGTGTGGTTT	miRNA	let-7b
t0035768	24	2	TGAGGTAGTAGGTTGTGGGGTTGA	miRNA	let-7b
t0035827	21	2	TGGGTAGTAGGTTGTGTGGTT	miRNA	let-7b
t0035854	25	2	TGAGGTAGTAGGTTGTGTGGTTTTC	miRNA	let-7b
t0035861	23	2	TGAGGTAGTAGGTTGTATGGTTG	miRNA	let-7b
t0035932	24	2	TGAGGTAGTAGGTTGGGTGGTTAA	miRNA	let-7b
t0035953	23	2	TGAGGTAGTAGGTTGCGTGGTAT	miRNA	let-7b
t0035995	20	2	TGAGGTAGTTGGTTGTGTGG	miRNA	let-7b
t0036026	23	2	TGAGGTAATAGGTTGTGTGGTTA	miRNA	let-7b
t0036190	24	2	TGAGGTAGTAGGTTGTGTGGTTGG	miRNA	let-7b
t0036210	24	2	TGAGGTAGTAGGTTGTGTGGTTGC	miRNA	let-7b

t0036227	24	2	TGAGGTAATAGGTTGTGTGGTTAA	miRNA	let-7b
t0036241	22	2	TGAGGTAGTAGGTCGTGTGGTA	miRNA	let-7b
t0036358	23	2	TGAGGTACTAGGTTGTGTGGTTT	miRNA	let-7b
t0036372	22	2	TGACGTAGTAGGTTGTGTGGTA	miRNA	let-7b
t0036377	24	2	TGAGGTAGGAGGTTGTGTGGTTAA	miRNA	let-7b
t0036393	23	2	TGAGGTAGTAGGTTGGGTGGTTA	miRNA	let-7b
t0036473	25	2	TGAGGTAGTAGGTTGTGGGGTTAGA	miRNA	let-7b
t0036494	22	2	TGAGGACGTAGGTTGTGTGGTT	miRNA	let-7b
t0036701	23	2	TGAGGTAGTAGGTTGGGTGGTTT	miRNA	let-7b
t0036760	22	2	TGAGGTAGTAGCTTGTGGGGTT	miRNA	let-7b
t0036887	22	2	TGAGGTAGTGGGTTGTGTGTTT	miRNA	let-7b
t0036949	24	2	TGAGGTGGTAGGTTGTGTGGTTAT	miRNA	let-7b
t0037001	23	2	TGAGGTAGTAGGTTGTGTGGGTG	miRNA	let-7b
t0037136	20	2	TGAGGTAGTAGGTTGTTTGG	miRNA	let-7b
t0037270	19	2	TGAGGTAATAGGTTGTGTG	miRNA	let-7b
t0037320	23	2	TGAGGTAGTAGGTTGTGTGATTA	miRNA	let-7b
t0037414	25	2	TGAGGAAGTAGGTTGTGTGGTTATC	miRNA	let-7b
t0037449	18	2	TGTGGTAGTAGGTTGTGT	miRNA	let-7b
t0037457	24	2	TGAGGTAGCAGGTTGTGTGGTTAT	miRNA	let-7b
t0037508	25	2	CGAGGTAGTAGGTTGTGTGGTTATC	miRNA	let-7b
t0037542	23	2	TGAGGTAGTAGGTTATGTGGTTT	miRNA	let-7b
t0037552	24	2	TGAGGTAGTAGGTTGTGTGGGGAT	miRNA	let-7b
t0037585	22	2	TGACGTAGTCGGTTGTGTGGTT	miRNA	let-7b
t0037655	22	2	TGAGGTAGTAGGATGTGGGGTT	miRNA	let-7b
t0037684	22	2	TGAGGTAGTAGGTTGTGTGATA	miRNA	let-7b
t0037690	24	2	TGAGGAAGTAGGTTGTGTGGTAGA	miRNA	let-7b
t0037726	24	2	TGAGGTAGTAGGTTGTGTGGTCTC	miRNA	let-7b
t0037754	21	2	TTAGGTAGTAGGTTGTGTAGT	miRNA	let-7b
t0037795	19	2	TGAGGTCGTAGGTTGTGTG	miRNA	let-7b
t0037809	23	2	TGGGGTAGTAGGTTGTGTGGTAA	miRNA	let-7b
t0037859	24	2	TTAGGTAGTAGGTTGTGTGGTTAA	miRNA	let-7b
t0037942	23	2	TGAGGTAGTCGGTTGTGTGGTTA	miRNA	let-7b
t0037943	23	2	TGCGGTAGTAGGTTGTGTGGTTA	miRNA	let-7b
t0038030	23	2	TGAGGTAGTAGGTTGTCTGGTTA	miRNA	let-7b
t0038033	20	2	TGAGGTAGTAGGTTTTGTGG	miRNA	let-7b
t0038089	25	2	TGAGGTAGTAGGTTGTATGGTTATT	miRNA	let-7b
t0038091	21	2	TGAGGTAGTAGGGTGTGCGGT	miRNA	let-7b
t0038100	24	2	TGAGGTAGGAGGTTGTGTGGTTAG	miRNA	let-7b
t0038105	22	2	TGAAGTAGTAGGTTGTGTGGTA	miRNA	let-7b
t0038154	24	2	TGAGGTAGTAGGTTGTGTGGGATC	miRNA	let-7b
t0038260	22	2	TGAGGTTGTAGGTTGTGTGGTA	miRNA	let-7b
t0038272	23	2	TGAGGTAGTAGGTTGAGTGGTTA	miRNA	let-7b
t0038306	24	2	TGAGGTAGTAGGGTGTGTGGTTAG	miRNA	let-7b
t0038430	24	2	TAAGGTAGTAGGTTGTGTGGTTAA	miRNA	let-7b
t0038488	23	2	TGAAGTAGTAGGTTGTGTGGTTA	miRNA	let-7b
t0038552	24	2	TGAGGTAGTAGGTTGTGGGGTTAG	miRNA	let-7b
t0038569	24	2	TGAGGTAGGAGGTTGTGTGGTTAT	miRNA	let-7b
t0038582	24	2	TGCGGTAGTAGGTTGTGTGGTTAA	miRNA	let-7b
t0038653	22	2	TGAGGTAGTAGTTTGTGGGGTT	miRNA	let-7b
t0038686	21	2	TGAGGTAGTAGGCTGTGTGGA	miRNA	let-7b
t0038749	26	2	TGAGGTAGTAGGTTGTGTGGTAAATC	miRNA	let-7b
t0038856	24	2	TGTGGTAGTAGGTTGTGTGGTTAA	miRNA	let-7b
t0038862	25	2	TGAGGTAGTAGGTTGTATGGTTACC	miRNA	let-7b
t0038863	21	2	TGAGGTAGTAGGTTGTGCAGT	miRNA	let-7b
t0038912	24	2	CGAGGTAGTAGGTTGTGTGGTTAT	miRNA	let-7b
t0039024	22	2	TGAGGTAGTAGGGTGTGTAGTT	miRNA	let-7b
t0039174	23	2	TGAGGTAGTAGGTTGTGTGGAAT	miRNA	let-7b
t0039177	22	2	TGAGGCAGTAGGTTGTGGGGTT	miRNA	let-7b

t0039209	23	2	TGAGGAAGTAGGTTGTGTGGTAA	miRNA	let-7b
t0039255	23	2	TGAGGTAGTAGATTGTGTGGTAA	miRNA	let-7b
t0039298	19	2	TGAGGTAGAAGGTTGTGTG	miRNA	let-7b
t0039359	23	2	TGAGGTAGTAGGTTGGGTGGTTG	miRNA	let-7b
t0039411	22	2	TGAGGTAGGAGGTTGTGTAGTT	miRNA	let-7b
t0039480	23	2	TGAGCTAGTAGGTTGTGTGGTTA	miRNA	let-7b
t0039510	22	2	TGAGGTAGTAGGTTGTGTTGTA	miRNA	let-7b
t0039560	21	2	TGAGGAAGTAGGTTGTGTGTT	miRNA	let-7b
t0039569	21	2	TGAGGTAGTAGGTTGCGTAGT	miRNA	let-7b
t0039599	23	2	TGAGGTAGTAGGTTGTGCGGTAA	miRNA	let-7b
t0039660	21	2	TTAGGTAGTAGGTTGTGGGGT	miRNA	let-7b
t0039693	25	2	TGAGGTAGTAGGTTGTGTGGTTATA	miRNA	let-7b
t0039705	23	2	TGAGGTAGTAGGTTGTATGGTTC	miRNA	let-7b
t0039722	22	2	TGAGGTAGTCGGTTGTGTGGTA	miRNA	let-7b
t0039785	22	2	TGAGGTAGTAGGGTGTGGGGTT	miRNA	let-7b
t0039790	26	2	TGAGGTAGTAGGTTGTGTGGTAGATC	miRNA	let-7b
t0039804	24	2	TGAGGTTGTAGGTTGTGTGGTTAG	miRNA	let-7b
t0039832	22	2	TGTGGTAGTAGGTTGTGTGGTA	miRNA	let-7b
t0039881	20	2	TGTGGTAGTAGGTTGTGTGG	miRNA	let-7b
t0039884	21	2	CGAGGTAGTAGGTTGTGTGTT	miRNA	let-7b
t0039953	23	2	TGAGGTAGTGGGTTGTGTGGTAT	miRNA	let-7b
t0039970	25	2	TGAGGTAGTAGGTTGTATGGTTTTC	miRNA	let-7b
t0040001	22	2	TGAGGTAGTAGGTTGTGGAGTT	miRNA	let-7b
t0040019	22	2	TGAGGTAGTAGGTTGTATAGGA	miRNA	let-7-5p
t0040042	24	2	TGAGGTAGTAGGGTGTATGGTATC	miRNA	let-7-5p
t0040055	23	2	TGAGGTAGTAGGTTGTATGGTAA	miRNA	let-7-5p
t0040079	22	2	TGAGGTAGCAGGTTGTATAGTA	miRNA	let-7-5p
t0040094	22	2	TGAGGTAGTAGGTTGTATAGGC	miRNA	let-7-5p
t0040146	22	2	TGAGGTAGTAGTTTGTATAGTA	miRNA	let-7-5p
t0040160	20	2	TGAGGTAGGAGGGTGTATAG	miRNA	let-7
t0040259	21	2	TGAGGTAGTATGGTGTATAGT	miRNA	let-7
t0040260	19	2	TGAGGTAGGAGGTTGTATA	miRNA	let-7
t0040332	23	2	TGAGGTAGTGGGTTGTATAGTTG	miRNA	let-7
t0040493	19	2	TGAGGTAGTAGGATGTATA	miRNA	let-7
t0040503	19	2	TGTGGTAGTAGGTTGTATA	miRNA	let-7
t0040600	22	2	TGAGGTAGTAGGTTGTAGAGTT	miRNA	let-7
t0035280	19	2	TGAGGTAGTAGGTCGTATA	miRNA	let-7
t0035812	23	2	TGGGGTAGTAGGTTGTATAGTTG	miRNA	let-7
t0036831	23	2	TGAGGAAGTAGGTTGTATAGTTT	miRNA	let-7
t0038141	19	2	TGAGGTAGTGGGTGGTATA	miRNA	let-7
t0038452	22	2	TGACGTAGTAGGTTGTATAGTT	miRNA	let-7
t0040641	23	2	TGAGGAAGTAGGTTGTATAGTTA	miRNA	let-7
t0035602	18	2	TGAGGTAGTAGGGTGTAG	miRNA	let-7
t0035650	22	2	TGAGGTAGTAGGTAGTATAGTT	miRNA	let-7
t0035658	23	2	TAAGGTAGTAGGTTGTATAGTTT	miRNA	let-7
t0035700	20	2	TGAGGTAGTACGTTGTATAG	miRNA	let-7
t0035726	23	2	TGCGGTAGTAGGTTGTATAGTTT	miRNA	let-7
t0036017	23	2	CGAGGTAGTAGGTTGTATAGTTG	miRNA	let-7
t0036157	19	2	TGCGGTAGTAGGTTGTATA	miRNA	let-7
t0036164	23	2	TGAGGTAGCAGGTTGTATAGTTT	miRNA	let-7
t0036375	23	2	TGAGGTAGTAGGTTGTTTAGTTG	miRNA	let-7
t0036413	22	2	TGGGGCAGTAGGTTGTATAGTT	miRNA	let-7
t0037174	23	2	TGAGGTAATAGGTTGTATAGTTG	miRNA	let-7
t0037340	20	2	TGAGGTATTAGGTTGTATAG	miRNA	let-7
t0037370	23	2	TGAGGTAGTGGGTTGTATAGTTT	miRNA	let-7
t0037909	21	2	TGAGGTGGGAGGTTGTATAGT	miRNA	let-7
t0037932	24	1	AAAAGTTAGTGGTCCGAAGTTTTA	miRNA	miR-9c
t0038049	26	1	TCTTTGGTTATCCAGCTGTATGAATC	miRNA	miR-9a

t0038164	26	1	TCTTTGGTTATCTAGCTATATGAATC	miRNA	miR-9a
t0038287	22	1	CACCCGTAGAACCGAACTTGAG	miRNA	miR-99b
t0038313	22	1	CACCAGTAGAACCGACCTTGCG	miRNA	miR-99b
t0038375	22	1	CACCCGTAGAACCGACCTTGAG	miRNA	miR-99b
t0038673	25	1	CACCCGTAGAACCGACCTCGCGATC	miRNA	miR-99b
t0038680	24	1	ACCCGTAGAACCGACCTTGCGATC	miRNA	miR-99b
t0039057	26	1	AACCCGTAGATCCGAACTTGTGAATC	miRNA	miR-99a
t0039299	20	1	AACCCGCAGATCCGATCTTG	miRNA	miR-99a
t0040040	24	1	AACCCGTAGATACGATCTTGTATC	miRNA	miR-99a
t0040048	22	1	TGAACTGATGGATGCTCGGCTT	miRNA	miR-996
t0040120	21	1	AACTGATAGATGCTCGGCAAT	miRNA	miR-996
t0044426	23	1	TGAACTGATAGATGCTCGGCTTT	miRNA	miR-996
t0064659	24	1	CTGAACTGATAGATGCTCGGCTTT	miRNA	miR-996
t0068392	22	1	TGAACTGATAGATGCTCGGCTT	miRNA	miR-996
t0046242	21	1	AAGCCTCGACAACTTACTACT	miRNA	miR-98*
t0047986	22	1	CTATACGACTTACTACTTTCCT	miRNA	miR-98*
t0061933	21	1	TATACAACTTACTACTTTACT	miRNA	miR-98*
t0089025	21	1	TATACAACTTACTAATTTCAT	miRNA	miR-98*
t0091962	19	1	CTATACCACTTACTACTTT	miRNA	miR-98*
t0057674	20	1	CTATAAAACTTACTACTTTC	miRNA	miR-98*
t0079328	20	1	TGAGGTAATAAGTTGTATTG	miRNA	miR-98
t0091759	22	1	TGCGGTAGTAAGTTGTATTGTT	miRNA	miR-98
t0055920	20	1	TGAGGTAGTCAGTTGTATTG	miRNA	miR-98
t0072593	22	1	TGAGGTAGTAAGTTGTATAGGT	miRNA	miR-98
t0085277	23	1	TGAGGTAGTAGGTTGTATTGATC	miRNA	miR-98
t0090353	21	1	TGAGGGAGTAAGTTGTATTGT	miRNA	miR-98
t0093429	20	1	TGAGGAAGTAAGTTGTATTG	miRNA	miR-98
t0043533	21	1	TGAGGTAGTGAGTTGTATTGT	miRNA	miR-98
t0054593	22	1	TGAGGTAGTAAGTTGTGTGGAA	miRNA	miR-98
t0062959	21	1	TGAGGTAGTAGGTTGTATTGC	miRNA	miR-98
t0066294	22	1	TGAGGTAGGAAGTTGTATTGTT	miRNA	miR-98
t0067733	19	1	TGAGGTGGTAGGTTGTAGT	miRNA	miR-965-5p
t0084995	20	1	GGTGGAAGGGTGTAGGCATG	miRNA	miR-965-5p
t0058130	23	1	GGTGGAAGGTTGTAGGCATGTCT	miRNA	miR-965-5p
t0063971	20	1	AAGTGGAAGGTTGTAGGCAT	miRNA	miR-965-5p
t0064986	24	1	TCCAGGGGGAAGGTTGTAGGCATG	miRNA	miR-965-5p
t0066299	21	1	GGGGGAAGGTTGTAGGCATGT	miRNA	miR-965-5p
t0066353	23	1	AGGTGGAAGGTTGTAGGCATGGA	miRNA	miR-965-5p
t0067755	23	1	CAGGGGGAAGGTTGTAGGCATGT	miRNA	miR-965-5p
t0076401	20	1	AGGTGGGAGGTTGTAGGCAT	miRNA	miR-965-5p
t0079824	20	1	GTGGAAGGTTGTGGGCATGT	miRNA	miR-965-5p
t0081681	22	1	CAGGGGGAAGGTTGTAGGCATG	miRNA	miR-965-5p
t0082874	19	1	CAGGTGGAAGGGTGTAGGC	miRNA	miR-965-5p
t0092713	22	1	AGGTGGAAGGGTGTAGGCATGT	miRNA	miR-965-5p
t0043352	23	1	AGGGGGAAGGTTGTAGGCATGTA	miRNA	miR-965-5p
t0046125	20	1	GTGGAAGGGTGTAGGCATGT	miRNA	miR-965-5p
t0047750	20	1	AGGTGGAAGGGTGTAGGCAT	miRNA	miR-965-5p
t0048835	21	1	CAATCATGTGCAGTGACAATA	miRNA	miR-96*
t0055862	21	1	TTTGGCAATAGCACATTTTTG	miRNA	miR-96
t0059454	22	1	TTCCCTGTTTTGGCCATGTGTG	miRNA	miR-942
t0062329	22	1	CACCCGGCTGTGAGCACATGTG	miRNA	miR-941
t0063209	21	1	CACCCGGCTGTGTGCACCTGT	miRNA	miR-941
t0064703	23	1	CACCCGGCTGTGCGCACATGTGT	miRNA	miR-941
t0067432	23	1	CACCCGGCTGTGGGCACATGTGA	miRNA	miR-941
t0069586	23	1	CACCCGGCTGTGTGCACATGGGA	miRNA	miR-941
t0076959	26	1	TCTATTCCGTAGTGCATTGCAGAATC	miRNA	miR-932-5p
t0082046	26	1	CCAATTCCGTAGTGCATTGCAGAATC	miRNA	miR-932-5p
t0082371	26	1	CAAAGTGCTGTTCGTGCCGGTAGATC	miRNA	miR-93

t0083971	23	1	CAAAGTGCTGTTCGTGCAGGCAG	miRNA	miR-93
t0092494	24	1	CAAAGTGCTGTTCATGCAGGTAGA	miRNA	miR-93
t0048521	19	1	CAAAGCGCTGTTCGTGCAG	miRNA	miR-93
t0080916	23	1	CAACGTGCTGTTCGTGCAGGTAG	miRNA	miR-93
t0077083	20	1	CAAAGTGCTGTTCGTGAAGG	miRNA	miR-93
t0043917	22	1	CAAAGTGCTGTTCGTGCACGTA	miRNA	miR-93
t0068032	23	1	TAAAGTGCTGTTCGTGCAGGTAG	miRNA	miR-93
t0070551	22	1	CAAAGTGCTGTTCATGCAGGTA	miRNA	miR-93
t0073323	22	1	CAAAGTGCTGTCCGTGCAGGTA	miRNA	miR-93
t0079507	23	1	CAAAGTGTTGTTCGTGCAGGTAG	miRNA	miR-93
t0068295	21	1	CAAAGTGCTATTCGTGCAGGT	miRNA	miR-93
t0073827	22	1	CAAAGTGATGTTCGTGCAGGTA	miRNA	miR-93
t0041481	21	1	CAAAGTGCTGTTCGTACAGGT	miRNA	miR-93
t0041530	23	1	CAAAGTGCTTTTCGTGCAGGGAG	miRNA	miR-93
t0041925	21	1	CAAAGTGCAGTTCGTGCAGGT	miRNA	miR-93
t0043211	20	1	CAAAGTGCCGTTCGTGCAGG	miRNA	miR-93
t0043765	20	1	CAAAGTGCTGTTCGTGGAGG	miRNA	miR-93
t0044311	22	1	CAAAGGGCTGTTCGTGCAGTTA	miRNA	miR-93
t0044850	21	1	CAAAGTGCTGTTTGTGCAGGT	miRNA	miR-93
t0048530	23	1	CAAAGTGCTGTTCCTGCAGGTAG	miRNA	miR-93
t0050427	22	1	CAAGGTGCTGTTCGTGCAGGTA	miRNA	miR-93
t0050612	22	1	CAAAGTGCTGTTCGGGCAGGGA	miRNA	miR-93
t0050630	22	1	CAAAGTGCTGTTCGTGAAGGTA	miRNA	miR-93
t0051042	21	1	CAAAGTGCTGTTCGAGCAGGT	miRNA	miR-93
t0051299	21	1	AAAGAGCTGTTCGTGCAGGTA	miRNA	miR-93
t0052100	23	1	CAAAGTGCTGTTCGTGGAGGTAG	miRNA	miR-93
t0052120	23	1	CAAAGTGCTGTTCGCGCAGGTAG	miRNA	miR-93
t0053373	21	1	CAAAGTGATGTTCGTGCAGGT	miRNA	miR-93
t0054190	19	1	CAAAGTGCTGTTCGTGCCG	miRNA	miR-93
t0054260	23	1	CAAAGTGCTGTTCGTGCAGGCAA	miRNA	miR-93
t0054548	22	1	CAGAGTGCTGTTCGTGCAGGTA	miRNA	miR-93
t0056548	23	1	CAAAGTGCTGTTCGGGCAGGTAT	miRNA	miR-93
t0058905	21	1	CACAGTGCTGTTCGTGCAGGT	miRNA	miR-93
t0059241	21	1	CAAAGTGCTGTTCGTGCAGGC	miRNA	miR-93
t0059986	23	1	CAAAGTGCTGTTCGTACAGGTAT	miRNA	miR-93
t0061470	22	1	CAAAGTGCTGATCGTGCAGGTA	miRNA	miR-93
t0061961	21	1	CAAAGTGCTGTTCGTGCGGGT	miRNA	miR-93
t0062023	23	1	CAAAGTGCTTTTCGTGCAGGAAG	miRNA	miR-93
t0062573	22	1	CAAAGTGCTGTTCGTGCCGGTT	miRNA	miR-93
t0062607	22	1	CAAAGTGCGGTTCGTGCAGGTA	miRNA	miR-93
t0064634	23	1	CAAAGTGCTGTTAGTGCAGGTAT	miRNA	miR-93
t0065991	23	1	CAAAGTGGTGTTCGTGCAGGTAG	miRNA	miR-93
t0066226	21	1	CAAAGTGCTGTTCGGGCAGGT	miRNA	miR-93
t0066313	22	1	CAAAGTGCTGTTCATGCAGGTG	miRNA	miR-93
t0066370	23	1	CACAGTGCTGTTCGTGCAGGTAG	miRNA	miR-93
t0066490	21	1	CAAAGTACTGTTCGTGCAGGT	miRNA	miR-93
t0066672	23	1	CAAAGCGCTGTTCGTGCAGGCAG	miRNA	miR-93
t0066757	23	1	CGAAGTGCTGTTCGTGCAGGTAG	miRNA	miR-93
t0067565	21	1	CAAAGTGCTGTTCGGGAAGGT	miRNA	miR-93
t0068489	21	1	CAAAGTGCTGCTCGTGCAGGT	miRNA	miR-93
t0068642	24	1	CAAAGTGCTGTTCGAGCAGGTAGA	miRNA	miR-93
t0069471	22	1	CAAAGTGCTGTTCGGGCAGGTC	miRNA	miR-93
t0069643	22	1	CAAAGTGCCGTTCGTGCAGGTA	miRNA	miR-93
t0070529	20	1	CAAAGTGCTGTTCGTGCCGG	miRNA	miR-93
t0074022	22	1	CAAAGTGCTGTTCGTGCGGGTA	miRNA	miR-93
t0074027	22	1	CAAAGAGCTGTTCGTGCAGGTA	miRNA	miR-93
t0074182	20	1	CAAAGTGCTGTTCGGGCAGG	miRNA	miR-93
t0074599	23	1	CAAAGTCCTGTTCGTGCAGGTAG	miRNA	miR-93

t0075248	22	1	CAAAGTGCTGTTCGTACAGGTA	miRNA	miR-93
t0075504	22	1	CCAAGTGCTGTTCGTGCAGGTA	miRNA	miR-93
t0077865	22	1	TAAAGTGCTGTTCGTGCAGGTA	miRNA	miR-93
t0078109	22	1	CTAAGTGCTGTTCGTGCAGGTA	miRNA	miR-93
t0078943	24	1	TATTGTACTTGTCCCGGCCTGTAG	miRNA	miR-92d
t0079650	20	1	ATTGCACTTGTCCCGGGCTG	miRNA	miR-92c
t0079890	19	1	ATTGCACTTGTCACGGCCT	miRNA	miR-92c
t0080775	22	1	ATTGAACTTGTCCCGGCCTGTT	miRNA	miR-92c
t0083274	19	1	ATTGCACTTGTACCGGCCT	miRNA	miR-92c
t0084377	19	1	ATTGCACTTGTTCAGGCCT	miRNA	miR-92c
t0084933	22	1	AATTGCATTTGTCCCGGCCTGT	miRNA	miR-92c
t0085357	21	1	GATTGCACTTGTCCCGGCCTG	miRNA	miR-92c
t0086908	22	1	TATTGCACTTGTCCCGGCTGGA	miRNA	miR-92b-3p
t0087163	22	1	TATTGCACTTGTCTCGGCCTGA	miRNA	miR-92b-3p
t0088809	22	1	ATTGCACCTGTCCCCGCCTGTA	miRNA	miR-92b-3p
t0089811	22	1	TATTGCACTTGTCCCGGACTGA	miRNA	miR-92b-3p
t0092877	22	1	TATTGCAATTGTCCCGGCCTGA	miRNA	miR-92b-3p
t0069500	22	1	ATTGCAATTGTCCCGGCCTGTA	miRNA	miR-92b-3p
t0043889	22	1	TATTGCACTTGTCCCTGCCTGA	miRNA	miR-92b-3p
t0053376	23	1	TATTGCATTTGTCCCGGCCTGAA	miRNA	miR-92b-3p
t0058766	22	1	ATTGCACCTGTCCCGGCCTGGA	miRNA	miR-92b-3p
t0061357	24	1	TATTGCATTTGTCCCGGCCTGAAA	miRNA	miR-92b-3p
t0061573	22	1	TATTGCACTTGTCCCGGCTTGG	miRNA	miR-92b-3p
t0074440	23	1	TATTGCACTTGTCCAGGCCTGGA	miRNA	miR-92b-3p
t0082914	22	1	TATTGCTCTTGTCCCGGCCTGA	miRNA	miR-92b-3p
t0041948	22	1	ATTGCACTTGTCCCGGCCTGGA	miRNA	miR-92b-3p
t0048322	22	1	TATTGCATTTGTCCCGGCCTGG	miRNA	miR-92b-3p
t0050495	35	1	ATTGAACTTGTCCCGGCCTGTAATACTCT[illegible]	miRNA	miR-92b-3p
t0051829	22	1	TATTGCACTTGTCCTGGCCTGA	miRNA	miR-92b-3p
t0056181	22	1	TATTGCACTTGTCCCGGCTCGG	miRNA	miR-92b-3p
t0058300	22	1	ATTGCACTTGTCCCAGCCTGTA	miRNA	miR-92b-3p
t0062152	23	1	TATTGCACTTGTCTTGGCCTGGA	miRNA	miR-92b-3p
t0063084	22	1	ATTGCACTTATCCCGGCCTGTA	miRNA	miR-92b-3p
t0064409	20	1	AGGGCCGGGACGCGGTGCAA	miRNA	miR-92b*
t0068252	20	1	AGGGACGGGACGCGGTGCCG	miRNA	miR-92b*
t0071948	20	1	AGGGACGGGACGAGGTGCAG	miRNA	miR-92b*
t0073032	20	1	AGGGACGGGCCGCGGTGCAA	miRNA	miR-92b*
t0073143	22	1	AGGGATGGGACGCGGTGCAGTA	miRNA	miR-92b*
t0074062	21	1	AGGGACGGGACGCGGTGAAGT	miRNA	miR-92b*
t0081699	20	1	AGGGACGGGACGCGGGGCAG	miRNA	miR-92b*
t0081806	21	1	AGGGACGGTACGCGGTGCAGT	miRNA	miR-92b*
t0082345	22	1	AGGGACGGGACGCAGTGCAGTG	miRNA	miR-92b*
t0085265	22	1	AGGGACGGGACGCGGTGCAGCG	miRNA	miR-92b*
t0090553	21	1	CGGGACGGGACGCGGTGCAGT	miRNA	miR-92b*
t0092230	22	1	AGGGACGGGACGCGGTGCACTG	miRNA	miR-92b*
t0092475	23	1	AGGGACGGGACGCGGGGCAGTGT	miRNA	miR-92b*
t0040720	21	1	AGGGACAGGACGCGGTGCAGT	miRNA	miR-92b*
t0041055	21	1	AGGGCCGGGACGCGGTGCAGT	miRNA	miR-92b*
t0042019	20	1	AGGGACGGGAGGCGGTGCAA	miRNA	miR-92b*
t0047447	21	1	AGGGACGGGACGCGGGGCAGT	miRNA	miR-92b*
t0056613	23	1	AGGGACGGGACACGGTGCAGTGA	miRNA	miR-92b*
t0057107	21	1	AGGGACGGGACGCTGTGCAGT	miRNA	miR-92b*
t0058360	23	1	AGGGACGGGACGAGGTGAAGTGA	miRNA	miR-92b*
t0058651	21	1	AGGGACGGGACGCGGTGCCGT	miRNA	miR-92b*
t0058821	21	1	GGGACGGGACGCGGTGCAGGA	miRNA	miR-92b*
t0061062	21	1	AGGGAAGGGACGCGGTGCAGT	miRNA	miR-92b*
t0061269	21	1	AGGGACGGGACGAGGTGCAGT	miRNA	miR-92b*
t0061365	21	1	AGGGACGGGACGCGGTGCGGT	miRNA	miR-92b*

t0063646	21	1	AGGGACGGGACGCAGTGCAGT	miRNA	miR-92b*
t0065026	20	1	AGGGACGGGAAGCGGAGCAG	miRNA	miR-92b*
t0066458	20	1	AGGGACGGGAAGCGGTGCAG	miRNA	miR-92b*
t0067390	19	1	AGGGACGGGACGCGGTGTA	miRNA	miR-92b*
t0071401	22	1	AGGGACGGGACGCGGTGCTGTG	miRNA	miR-92b*
t0072300	20	1	AGGGACGGGGCGCGGTGCAG	miRNA	miR-92b*
t0073737	20	1	AGGGGCGGGACGCGGTGCAG	miRNA	miR-92b*
t0074141	20	1	AGGGACGGGACGCGATGCAG	miRNA	miR-92b*
t0074170	23	1	AGGGACGGGGCGCGGTGCAGTAG	miRNA	miR-92b*
t0074707	22	1	AGGGACGGGTCGCGGTGCAGTG	miRNA	miR-92b*
t0074849	20	1	AGGGACGGGCCGCGGTGCCG	miRNA	miR-92b*
t0076198	22	1	AGGGACGGGACGCGGTGCAGTC	miRNA	miR-92b*
t0077433	21	1	AGGGACGGGACGCGATGCAGT	miRNA	miR-92b*
t0078627	21	1	TATTGCACTTGTCCCGGACTT	miRNA	miR-92b
t0079695	22	1	TATTGCACTTGTCCCAGCCTTA	miRNA	miR-92b
t0080578	22	1	TATTGCACTTGTCTCGGCCTTT	miRNA	miR-92b
t0081264	23	1	TATTGCACTTGTCCCGGCCTTTT	miRNA	miR-92b
t0081611	21	1	TATTGCACTTGTCCCTGCCTT	miRNA	miR-92b
t0082889	21	1	TGTTGCACTTGTCCCGGCCTT	miRNA	miR-92b
t0083069	21	1	TATTGCACTTGTACCGGCCTT	miRNA	miR-92b
t0083630	22	1	TATTGCACTTGGCCCGGCATTT	miRNA	miR-92b
t0083923	21	1	TATTGCCCTTGTCTCGGCCTT	miRNA	miR-92b
t0084573	22	1	TATTGCACTTGTCCCGGCTGTC	miRNA	miR-92b
t0085295	21	1	ATTGCACTTGTCCCGGCTTTT	miRNA	miR-92b
t0085401	21	1	CATTGCACTTGTCCCGGCCAA	miRNA	miR-92a-3p
t0090438	22	1	CATTGCACTTGTCACGGCCTGT	miRNA	miR-92a-3p
t0045001	21	1	CATTGCACTTGTCCCGGCCTG	miRNA	miR-92a-3p
t0045114	20	1	AGGTTGGGATCGGTTGCGAT	miRNA	miR-92a-1*
t0047577	23	1	AGGTTGGGATCGGTTGCAATTCT	miRNA	miR-92a-1*
t0050135	23	1	AGGTTGGGATCGGTTGCCATGCT	miRNA	miR-92a-1*
t0055451	21	1	TATTGCACTTGTCCCTGCCTG	miRNA	miR-92a
t0063352	21	1	TATTGCACTTGTCTCAGCCTG	miRNA	miR-92a
t0074771	23	1	TATTACACTTGTCCCGGCCTGTT	miRNA	miR-92a
t0074823	22	1	TATTGCCCTTGTCCCGGCCTAT	miRNA	miR-92a
t0081234	20	1	TATTGCACCTGTCCCGGCCT	miRNA	miR-92a
t0085563	22	1	TATTGCATTTGTCCCGGGCTGT	miRNA	miR-92a
t0089776	21	1	TATTGCTCTTGTCCCGGCCTG	miRNA	miR-92a
t0047556	21	1	TATTGCACTTGTCCCGGCCAC	miRNA	miR-92a
t0077230	20	1	TATTGGACTTGTCCCGGCCT	miRNA	miR-92a
t0078679	20	1	TATTGCACTTGTCCTGGCCT	miRNA	miR-92a
t0047356	23	1	TATTGCACTTGTCCCGGACTGTA	miRNA	miR-92a
t0070050	23	1	TCTTGCACTTGTCCCGGACTGTA	miRNA	miR-92a
t0076074	22	1	TATTGCACTTGTCCCTGCCTGT	miRNA	miR-92a
t0040727	20	1	TATTACACTTGTCCCGGCAT	miRNA	miR-92a
t0041447	24	1	TATTGCACTTGTCCCGTCCTGTAG	miRNA	miR-92a
t0042477	23	1	TATTGCACTTGTCCCGGCATGTA	miRNA	miR-92a
t0043232	20	1	TATTGCACTTGTCCCGACCT	miRNA	miR-92a
t0043275	22	1	TATTGCAATTGTCCCGGCCTAT	miRNA	miR-92a
t0044282	23	1	TATTGCACTTGTACCGGCCTGTA	miRNA	miR-92a
t0044988	19	1	TATTGCACTTGTTCCGGCC	miRNA	miR-92a
t0045391	23	1	TATTGCACTTGTCCCGGCATGTT	miRNA	miR-92a
t0045928	20	1	TATTGCACTTGTCCCGGACT	miRNA	miR-92a
t0045960	21	1	TATTGCACGTGTCCCGGCCTG	miRNA	miR-92a
t0046123	22	1	TATTGCAGTTGTCCCGGCCTGT	miRNA	miR-92a
t0046140	23	1	TATTGCACCTGTCCCGGCCTGTT	miRNA	miR-92a
t0046324	21	1	TATTGCACTTGTACCGGCCTA	miRNA	miR-92a
t0046421	21	1	TATTGCACCTGTCCCGGCCTG	miRNA	miR-92a
t0046827	19	1	TATTGCACTGGTCCCGGCC	miRNA	miR-92a

t0046930	22	1	TATTGCACTTGTGCCGGCCTGT	miRNA	miR-92a
t0049330	21	1	TATTTCACTTGTCCCGGCCTG	miRNA	miR-92a
t0050060	23	1	TATTGCATTTGTCCCGGCCTGTT	miRNA	miR-92a
t0050333	22	1	TATTGCGCTTGTCCTGGCCTGT	miRNA	miR-92a
t0051091	20	1	TATTGCACTTGACCCGGCCT	miRNA	miR-92a
t0052104	21	1	TATTGCACTTGTCCCAGCCTG	miRNA	miR-92a
t0052381	21	1	TATTGCACATGTCCCGGCCTG	miRNA	miR-92a
t0052911	21	1	TATTGCGCTTGTCCCGGCCTG	miRNA	miR-92a
t0053346	20	1	TATTGCACTTGTCTCGGCCT	miRNA	miR-92a
t0053616	21	1	TATAGCACTTGTCCCGGCCTG	miRNA	miR-92a
t0055261	19	1	TATTGCACTTGTACCGGCC	miRNA	miR-92a
t0055381	19	1	TATTGCAATTGTCCCGGCC	miRNA	miR-92a
t0055958	19	1	TATTGCACTTGTCACGGCC	miRNA	miR-92a
t0057955	23	1	TATTGCACTTGTACCGGCCTGTT	miRNA	miR-92a
t0058532	20	1	TATTACACTTGTCCCGGCCT	miRNA	miR-92a
t0058595	20	1	TATTGCACTTGTCCCGGACA	miRNA	miR-92a
t0060195	22	1	TCTTGCACTTGTCCCGGCCTGT	miRNA	miR-92a
t0060766	23	1	TATTGCACTTGTCCCGACCTGTA	miRNA	miR-92a
t0061361	19	1	TATTACACTTGTCCCGGCC	miRNA	miR-92a
t0062277	20	1	TGTTGCACTTGTCCCGGCCT	miRNA	miR-92a
t0063569	21	1	TATTGCATTTGTCCCGGGCTG	miRNA	miR-92a
t0063868	20	1	TATTGCACTTGGCCCGGCCT	miRNA	miR-92a
t0064032	22	1	TATTGCATTTGTCCCGGACTGT	miRNA	miR-92a
t0064333	22	1	TATTGCACTTGTCCCGGGGTGT	miRNA	miR-92a
t0066478	23	1	TATTGAACTTGTCCCGGCCTGTA	miRNA	miR-92a
t0067352	21	1	TATTGCACTTGTCCCGACCTA	miRNA	miR-92a
t0068889	19	1	TATTGCCCTTGTCCCGGCC	miRNA	miR-92a
t0068896	22	1	TATTGCACTTGTCTCGGCATGT	miRNA	miR-92a
t0069323	21	1	TATTGCACTTGTTCCGGCCTA	miRNA	miR-92a
t0069483	20	1	TATTGCACTTGTCCAGGACT	miRNA	miR-92a
t0070110	23	1	TATTGCCCTTGTCCCGGCCTGTA	miRNA	miR-92a
t0070799	23	1	TATTGCATTTGTCCAGGCCTGTA	miRNA	miR-92a
t0072541	20	1	TATTGCACTTGTCCCGGCTT	miRNA	miR-92a
t0074645	21	1	TATCGCACTTGTCCCGGCCTG	miRNA	miR-92a
t0074660	19	1	TATTGCACTTCTCCCGGCC	miRNA	miR-92a
t0075334	23	1	TATTGCACTTGTCCCGGCCCGTA	miRNA	miR-92a
t0076627	20	1	TATTGCACTTGTCCAGGCCT	miRNA	miR-92a
t0076798	22	1	ATATTGCACTTGTCCCGGCTTG	miRNA	miR-92a
t0077493	23	1	TATTGCACTTGTCCCGGGCTGTA	miRNA	miR-92a
t0079272	23	1	TATCGCACTTGTCCCGGCCTGTA	miRNA	miR-92a
t0079299	22	1	TATTGCACTTGTCCCGGCCCGT	miRNA	miR-92a
t0079682	19	1	TATTGCACTTGTCCCGGAC	miRNA	miR-92a
t0080100	22	1	TATTGCACTTGTCCCGCCCTGT	miRNA	miR-92a
t0080429	22	1	TATTGCACTTGTACAGGCCTGT	miRNA	miR-92a
t0080862	20	1	TATAGCACTTGTCCCGGCCT	miRNA	miR-92a
t0080930	20	1	TACTGCACTTGTCCCGGCCT	miRNA	miR-92a
t0081863	23	1	TATTGGACTTGTCCCGGCCTGTA	miRNA	miR-92a
t0083073	22	1	TACTGCACTTGTCCCGGCCTGT	miRNA	miR-92a
t0084644	21	1	TATTGCACTTGACCCGGCCTG	miRNA	miR-92a
t0084777	22	1	TATTGCATTTGTCTCGGCCTGT	miRNA	miR-92a
t0085105	21	1	TATTGCACTTTTCCCGGCCTG	miRNA	miR-92a
t0085227	22	1	ATATTGCACTTGTCCAGGCCTG	miRNA	miR-92a
t0085512	21	1	TATTGCACTTGTCCAGGCATG	miRNA	miR-92a
t0085700	22	1	GGTGGGAGGCTGTGAAGCGGTG	miRNA	miR-920
t0086086	19	1	TATTGCACTCGTCACGGCC	miRNA	miR-92
t0086808	22	1	TATTGCACTCGTCCCGGCAAAA	miRNA	miR-92
t0087140	21	1	TATTGCACTCGTCCCGGCATT	miRNA	miR-92
t0087230	22	1	TATTGAACTCGTCCCGGCCTCT	miRNA	miR-92

t0087253	21	1	TATTGAACTCGTCCCGGCCAT	miRNA	miR-92
t0087534	21	1	TATTCCACTCGTCCCGGCCAA	miRNA	miR-92
t0087749	21	1	TATTGCACTCGTCCCGGCATC	miRNA	miR-92
t0088112	21	1	TATTGCAATCGTCCCGGCCTT	miRNA	miR-92
t0088907	22	1	TACTGCACTCGTCCCGGCCTAA	miRNA	miR-92
t0090018	22	1	TATTGCACTCGTACCGGCCTCT	miRNA	miR-92
t0090746	22	1	TATTGCACTCGTTCCGGCCATT	miRNA	miR-92
t0092217	21	1	TATTGCTCTCGTCCCGGCCTA	miRNA	miR-92
t0051331	21	1	TATTGGACTCGTCCCGGCCTA	miRNA	miR-92
t0040655	22	1	TATTGCACTCGCCCCGGCCTAA	miRNA	miR-92
t0044447	23	1	TATTGCACTCGTCACGGCCTCCA	miRNA	miR-92
t0044849	22	1	TATTGCACTCGTCCCGGCAATA	miRNA	miR-92
t0045272	20	1	TATTGCACTCGTCCTGGCCA	miRNA	miR-92
t0045739	22	1	TATTGCACTCGTCACGGCCTTA	miRNA	miR-92
t0046880	23	1	CTATTGCACTTGTCCCGGCCTAA	miRNA	miR-92
t0047514	20	1	AATTGCACTCGTCCCGGCCT	miRNA	miR-92
t0048466	21	1	TATTGCACTCGTCCCGGCCTC	miRNA	miR-92
t0049982	19	1	TATTGTACTCGTCCCGGCC	miRNA	miR-92
t0050653	19	1	TATTGCACTCGTCCAGGCC	miRNA	miR-92
t0051214	22	1	TATTGCACTAGTCCCGGCCATT	miRNA	miR-92
t0054048	22	1	TATTGAACTCGTCCCGGCCTAA	miRNA	miR-92
t0055218	22	1	TATTGCACTCGTCCAGGCCTCT	miRNA	miR-92
t0055253	20	1	TATTGCACTCGTCCCGGCCG	miRNA	miR-92
t0055910	22	1	TATTGCACTCGTCCCGCCCTCA	miRNA	miR-92
t0056363	22	1	TATTGCACTCGTCCGGGCCTTA	miRNA	miR-92
t0057643	22	1	TATTGCACTTGTCCCGGCCTAC	miRNA	miR-92
t0057840	19	1	TTGCACTCGTCCCGGCATC	miRNA	miR-92
t0058346	20	1	TATTGCACTCGTCCCGGTCA	miRNA	miR-92
t0058567	22	1	TATTGCACTCGTCACGGACTCT	miRNA	miR-92
t0060617	23	1	TATTGCACTCGTCCCGGCAAAGA	miRNA	miR-92
t0062093	23	1	TATTGGACTCGTCCCGGCCTATT	miRNA	miR-92
t0062485	22	1	TATTGCACCCGTCCCGGCCTTA	miRNA	miR-92
t0063358	23	1	TATTGCACTTGTCCCGGCCTAAG	miRNA	miR-92
t0064138	22	1	TATTGCACTCGTCCCGGGCTAT	miRNA	miR-92
t0065267	21	1	TATTGCACTCGTCCCGGCCTG	miRNA	miR-92
t0066308	22	1	TATTGCACTCGTCCCGGCCTAC	miRNA	miR-92
t0067397	20	1	TATTGCACTCGTCACGGCCA	miRNA	miR-92
t0067997	21	1	TATCGCACTCGTCCCGGCCTA	miRNA	miR-92
t0068023	20	1	TATTGCACTCGTCCCGGACA	miRNA	miR-92
t0068349	19	1	TTGCACTCGTACCGGCCTC	miRNA	miR-92
t0069814	22	1	TATTGCACTCGTCCCGACCTAA	miRNA	miR-92
t0070842	22	1	TATTGCACTCGTCCCAGCCTCT	miRNA	miR-92
t0073264	21	1	TATTGCGCTCGTCCCGGCCTA	miRNA	miR-92
t0074136	22	1	TATTGCAATCGTCCCGGCCAAT	miRNA	miR-92
t0074172	22	1	TATTGCACTTGTCCCGGCCACA	miRNA	miR-92
t0075039	20	1	TATTGCACTCGTACCGGCCT	miRNA	miR-92
t0075514	21	1	TATTGCACTCGTCACGGCCTA	miRNA	miR-92
t0076237	22	1	TATTGCAATCGTCCCGGCCTCT	miRNA	miR-92
t0076324	22	1	TATTGCACTCGTCCCGGCCACA	miRNA	miR-92
t0076661	18	1	GGGCAGGGGGTGTAGTGG	miRNA	miR-885-3p
t0078430	20	1	GGGCAGGGGGTGTAGTGGTA	miRNA	miR-885-3p
t0079573	18	1	GGGCACGTGGTGTAGTGG	miRNA	miR-885
t0080284	18	1	CCGGTGGTGTAGCGGTAT	miRNA	miR-885
t0080710	20	1	GGGCAGGTGGTGCAGTGGTA	miRNA	miR-885
t0080811	23	1	GTAGAGGAGATGGCGCAGGGGCC	miRNA	miR-877
t0083357	22	1	TAGAGGAGATGGAGCAGGGGAA	miRNA	miR-877
t0083926	20	1	AGAGGAAATGGCGCAGGGGA	miRNA	miR-877
t0086987	22	1	GTAGCGGCGATGGCGCAGGGGA	miRNA	miR-877

t0088176	22	1	GTAGAGGAGATGGCGCCGGGGA	miRNA	miR-877
t0089371	20	1	GTAGAGGCGATGGCGCAGGG	miRNA	miR-877
t0089467	20	1	GTAGAGGAGATGGCGAAGGA	miRNA	miR-877
t0092071	23	1	TGGAGGAGATGGCGCAGGGGACT	miRNA	miR-877
t0056867	22	1	GTAGAGGAGTTGGCGCAGGGGA	miRNA	miR-877
t0059665	23	1	TAGACGAGATGGCGCAGGGGACT	miRNA	miR-877
t0056480	22	1	AGAGGAGATGGCGCAGTGGACT	miRNA	miR-877
t0069350	20	1	GTAGAGGGGATGGCGCAGGA	miRNA	miR-877
t0092644	21	1	TAGAGGAGATGGCGCAGGTAA	miRNA	miR-877
t0041972	22	1	TAGAGGAGATGGCACAGGGGAA	miRNA	miR-877
t0042133	22	1	GTAGAGGAGCTGGCGCAGGGGA	miRNA	miR-877
t0043892	22	1	TAGAGGAGATAGCGCAGGGGAA	miRNA	miR-877
t0047868	22	1	TAGAGGAAATGGCGCAGGGGAC	miRNA	miR-877
t0048475	21	1	AGAGGAGATGGAGCAGGGGAA	miRNA	miR-877
t0057349	26	1	GTAGAGGAGATGGCGCAGGGGAATTT	miRNA	miR-877
t0059565	21	1	GTAGAGGAGATGGCGCAAGGG	miRNA	miR-877
t0059894	24	1	GTAGAGGAGATGGAGCAGGGGACT	miRNA	miR-877
t0060582	22	1	AGAGGAGATGGCGCAGGGGGCT	miRNA	miR-877
t0061372	22	1	TAGAGGAGATGGCGCAGGGGTA	miRNA	miR-877
t0069123	22	1	AGGGGAGATGGCGCAGGGGACA	miRNA	miR-877
t0069252	22	1	TAGAGGAGATGGGGCAGGGGAA	miRNA	miR-877
t0070159	22	1	GTGAGCAAAGTTTCAGGTGTGT	miRNA	miR-87
t0076625	25	1	TGGAAGACTAGTGATTTTGTTCATC	miRNA	miR-7b
t0077304	24	1	GTGGGCCTGACGTGGAGCTGGCTC	miRNA	miR-770-3p
t0080185	20	1	TGGAGAGAAAGGAAGTTTGA	miRNA	miR-765
t0081004	21	1	TGGAGAGAAAGGAAGTGTTTG	miRNA	miR-765
t0082138	22	1	TGGAGAGAAAGGAAGTTAATGA	miRNA	miR-765
t0082164	22	1	TGGAGAGAAAGGAAGTTCTTGC	miRNA	miR-765
t0082458	22	1	TGGAGAGAAAGGAAGTTTATGA	miRNA	miR-765
t0083872	18	1	TGGAGAGAAAGGAAGTTT	miRNA	miR-765
t0087548	19	1	TGGAGAGAAAGGGAGTTGA	miRNA	miR-765
t0088971	22	1	TGGAGAGAAAGGCAGTTAATGG	miRNA	miR-765
t0092899	22	1	TGAAGAGAAAGGCAGTTTATGT	miRNA	miR-765
t0093110	21	1	TGGAGAGAAAGGCAGTTTATG	miRNA	miR-765
t0051536	19	1	TGGAGAGAAAGGCAGTTTA	miRNA	miR-765
t0088078	21	1	TGGAGAGAAAGGAAGTTTATG	miRNA	miR-765
t0087233	21	1	TGGAGAGAAAGGAAGTTTCTG	miRNA	miR-765
t0042469	23	1	TGGAAGACTGGTGATATGTTGTA	miRNA	miR-7-5p
t0053737	21	1	TGGAAGACTGGGGATATGTTT	miRNA	miR-7-5p
t0055842	23	1	AAGTTAGATTGTGATTGGCATTT	miRNA	miR-753c
t0076371	21	1	TTTGTATTGTGACTGGCATGT	miRNA	miR-753c
t0080077	26	1	AAGTTGGATTGTGAGTGGCACATTTT	miRNA	miR-753c
t0081623	24	1	AATTTGGATTATGACTGGCATTTT	miRNA	miR-753c
t0082794	20	1	AAGTTAGATTGTGATTGGCA	miRNA	miR-753c
t0083852	20	1	AAGTTGGATTGTGACTGGCA	miRNA	miR-753c
t0088593	26	1	AAGTTGGATTGTGACTGGCATATTCT	miRNA	miR-753c
t0088841	27	1	AAGTTGGATTGTGACTGGCAAGCGGAA	miRNA	miR-753c
t0089454	25	1	AAGTTGGATTGTGACTGGTATTTTT	miRNA	miR-753c
t0090159	24	1	AAGTTGGATTGTGACTGGCATTAT	miRNA	miR-753c
t0093060	26	1	AAGTTAGATTGTGACTGGCACATTTT	miRNA	miR-753c
t0047185	23	1	TTTGTATTGTGACTGGCATGTTT	miRNA	miR-753c
t0057673	24	1	TTTGTATTGTGACTGGCAAGTTTT	miRNA	miR-753c
t0042943	22	1	ATTTGTATTGTGACTGGCATTT	miRNA	miR-753c
t0043728	26	1	AAGTTGGATTGTGACTGGCATATTTT	miRNA	miR-753c
t0044464	24	1	AATTTGGATTGTGACTGGCATTTT	miRNA	miR-753c
t0046956	25	1	AAGTTGGATTGTGACTGGCAATTTT	miRNA	miR-753c
t0047835	22	1	AAGTTGGATTGTGACTAGCGGA	miRNA	miR-753c
t0054938	24	1	TGCAGGGCTAGGGCTAACAGCATC	miRNA	miR-744

t0056459	22	1	TGAGGGGCTAGGGCTAACAGCA	miRNA	miR-744
t0057160	24	1	TGCGGGGCTAGGGCTAAAAGCATC	miRNA	miR-744
t0062958	22	1	TGTGGGGCTAGGGCTAACAGCA	miRNA	miR-744
t0069571	20	1	TGCGGGGATAGGGCTAACAG	miRNA	miR-744
t0071062	22	1	TGCGGGGCTAGGGCCAACAGCA	miRNA	miR-744
t0073055	19	1	TGCGGGGCTAGGGCTAAAA	miRNA	miR-744
t0076789	24	1	TGCGGGGCTAGGGCTAACAGCCGA	miRNA	miR-744
t0078068	24	1	TGCGGAGCTAGGGCTAACAGCATC	miRNA	miR-744
t0081849	22	1	CGGGGCTAGGGCTAAAAGCAGA	miRNA	miR-744
t0090335	24	1	AAAGATGCCACGCTATGTAGAATA	miRNA	miR-741-3p
t0090479	24	1	AAAGATGACACGCTATGTAGAATC	miRNA	miR-741-3p
t0092922	24	1	GAAGATGCCACGCTATGTAGAATC	miRNA	miR-741-3p
t0040886	23	1	GGAAGACTGAAGTGGAGAAGGGT	miRNA	miR-739
t0049328	22	1	GAAGCCTGAAGTGGAGAAGGGT	miRNA	miR-739
t0057586	22	1	GAAGACTGAGGTGGAGAAGGGT	miRNA	miR-739
t0059757	21	1	AAGACTGAAGGGGAGAAGGGT	miRNA	miR-739
t0063804	24	1	GAAGACTGAAGTAGAGAAGGGTTT	miRNA	miR-739
t0071540	22	1	AAGACTGAAGTGGGGAAGGGTT	miRNA	miR-739
t0073510	22	1	GAAGAATGAAGTGGAGAAGGGT	miRNA	miR-739
t0080067	23	1	GAAGACTGAAGTGGGGAAGGGTT	miRNA	miR-739
t0083476	25	1	GTGAAGACTGAAGTGGAGAAGGGTT	miRNA	miR-739
t0086485	21	1	AAGACTGCAGTGGAGAAGGGT	miRNA	miR-739
t0045409	24	1	GAAGACTGAAGCGGAGAAGGGTTT	miRNA	miR-739
t0048977	25	1	CGAAGACTGAAGTGGAGAAGGGTTT	miRNA	miR-739
t0091711	22	1	GAAGACTGAAGCGGAGAAGGGT	miRNA	miR-739
t0043240	24	1	TGGAGACTGAAGTGGAGAAGGGTT	miRNA	miR-739
t0043469	22	1	GAAGACTGAAGGGGATAAGGGT	miRNA	miR-739
t0045464	23	1	GAAGATTGAAGTGGAGAAGGGTT	miRNA	miR-739
t0046340	20	1	AAGACTGAAGTAGAGAAGGG	miRNA	miR-739
t0048219	21	1	GAAGACTGAAGTAGAGAAGGG	miRNA	miR-739
t0048775	24	1	TGAAGATTGAAGGGGAGAAGGGTT	miRNA	miR-739
t0050098	31	1	GAAGACTGAAGTGGAGAAGGGTTTATTGT	miRNA	miR-739
t0050232	23	1	TCAAGACTGAAGTGGAGAAGGGT	miRNA	miR-739
t0050652	20	1	CGAAGACTGAAGTGGAGAAG	miRNA	miR-739
t0053936	21	1	GATGACTGAAGTGGAGAAGGG	miRNA	miR-739
t0055530	20	1	GAAGTCTGAAGTGGAGAAGG	miRNA	miR-739
t0056171	24	1	GAAGATTGAAGTGGAGAAGGGTTT	miRNA	miR-739
t0056793	20	1	AAGACTGAAGGGGAGAAGGG	miRNA	miR-739
t0057628	23	1	TGATGACTGAAGTGGCGAAGGGT	miRNA	miR-739
t0059055	24	1	TGAGGATTGAAGTGGAGAAGGGTT	miRNA	miR-739
t0059452	22	1	AAGGCTGAAGTGGAGAAGGGTT	miRNA	miR-739
t0061011	25	1	TGCAGACTGAAGTGGAGAAGGGTTT	miRNA	miR-739
t0064362	23	1	TGAAGGCTGAAGTGGAGAAGGGT	miRNA	miR-739
t0065898	22	1	GAAGACTAAAGTGGATAAGGGT	miRNA	miR-739
t0066810	21	1	GAAGACTGAAGCGGAGAAGGG	miRNA	miR-739
t0069104	23	1	TAAGACTGAAGTGGAGAAGGGTT	miRNA	miR-739
t0070148	22	1	GAAGACTGAAGTGGGGAAGGGT	miRNA	miR-739
t0073103	23	1	GAAGAATGAAGTGGAGAAGGGTT	miRNA	miR-739
t0073202	23	1	GAAGACTGGAGTGGAGAAGGGTT	miRNA	miR-739
t0074809	26	1	CTCTGAAGACTGAAGTGGAGAAGGGT	miRNA	miR-739
t0075309	22	1	GAAGACTGAAGAGGAGAAGGGT	miRNA	miR-739
t0076104	24	1	GAAGACTGAAGTCGAGAAGGGTTT	miRNA	miR-739
t0076883	22	1	GACGACTGAAGTGGAGAAGGGT	miRNA	miR-739
t0077013	23	1	AAGACTGAAGTGGAGAAGGGTTT	miRNA	miR-739
t0080292	23	1	GAGGACTGAAGTGGAGAAGGGTT	miRNA	miR-739
t0081277	23	1	CAAGACTGAAGTGGAGAAGGGTT	miRNA	miR-739
t0081651	22	1	AAGACTTGAGTAGTGAGACGCT	miRNA	miR-71b-5p
t0081987	23	1	TGAAAGACGATGGGAGTGAGATG	miRNA	miR-71

t0082484	23	1	AAAGACGATGGTAGTGAGATGTT	miRNA	miR-71
t0082662	24	1	TGAAAGACGATGGTAGTGAGATGT	miRNA	miR-71
t0084358	23	1	TGAAAGACGATGGTAGTGAGCTG	miRNA	miR-71
t0084483	22	1	TGAAAGACGATTGTAGTGAGAT	miRNA	miR-71
t0084662	22	1	TGAAAGACGATGGTAGTGAGAA	miRNA	miR-71
t0087171	24	1	TGGACGACTAGTGATTTTGTTGTT	miRNA	miR-7
t0087369	24	1	TGGAAGACTAGTGATTTTGTTGGT	miRNA	miR-7
t0088888	24	1	TGGAAGACTAGTGCTTTTGTTGTT	miRNA	miR-7
t0089927	23	1	ACTCGCTAGGGAAAATGATTGGA	miRNA	miR-664*
t0091550	22	1	ACCGGCTAGGGAAAATGATTGG	miRNA	miR-664*
t0092636	22	1	ACTGGCTAGGGCAAATGATTGG	miRNA	miR-664*
t0084885	26	1	CTGGCTGGGGAAAATGATTGGATATT	miRNA	miR-664*
t0059709	23	1	ACTGGCTAGGGAAAACGATTGGA	miRNA	miR-664*
t0077539	26	1	GCTGGCTGGGGAAAATGATTGGAATC	miRNA	miR-664*
t0086266	23	1	ACTGGCTAGGGAAAATGATTTGA	miRNA	miR-664*
t0086623	23	1	ACTGGCTAGGGAAAATGATTGGG	miRNA	miR-664*
t0088428	21	1	TACCCATTGCATATCGGAGGT	miRNA	miR-660
t0090351	21	1	TACCCATTGCATATCTGAGTT	miRNA	miR-660
t0068452	19	1	TACCTATTGCATATCGGAG	miRNA	miR-660
t0073458	21	1	TACCCATTGTATATCGGAGTT	miRNA	miR-660
t0091498	21	1	TACCCATTGCATATCGGGGTT	miRNA	miR-660
t0044279	22	1	TACCCATTCCATATCGGAGTTG	miRNA	miR-660
t0051574	21	1	AATGGCGCCCCTAGGGTTGTG	miRNA	miR-652
t0055101	21	1	AATGGCGCCACTAGGGGTGTG	miRNA	miR-652
t0069023	21	1	AGGGCGCGCGGGGCGGGGCGG	miRNA	miR-638
t0075685	25	1	AGGGCGCGCGGGTCGGGGCGACGGA	miRNA	miR-638
t0080634	26	1	AGGGCGCGTGGGTCGGGGCGGCGGAC	miRNA	miR-638
t0081573	26	1	AGGGCGCGTGGGTCGGGGAGGCGGCC	miRNA	miR-638
t0081793	25	1	AGGGCGTGCGGGTCGGGGCGGCGGT	miRNA	miR-638
t0044580	26	1	AGGGCGCGCGGGTCGGGGCGGCGGAT	miRNA	miR-638
t0044826	25	1	AGGGCGCGCGGGTCGGGGCGGCGGG	miRNA	miR-638
t0065376	26	1	AGGGCGCGTGGGGCGGGGCGGCGGCC	miRNA	miR-638
t0078013	18	1	TCGGTTTACGTTGGGAGA	miRNA	miR-629
t0080227	19	1	AGGGTTTACGTTGGGAGAA	miRNA	miR-629
t0083225	19	1	CGGGTTTACGTTGGGAGAA	miRNA	miR-629
t0055637	19	1	TGGGTTTACGCTGGGAGAA	miRNA	miR-629
t0063400	19	1	TGGGTTTACGTTGGGAGGA	miRNA	miR-629
t0052671	18	1	TGGGATTACGTTGGGAGA	miRNA	miR-629
t0053867	19	1	TGGGTTTGCGTTGGGAGAA	miRNA	miR-629
t0063911	19	1	TGGGATTACGTTGGGAGAA	miRNA	miR-629
t0083495	18	1	TGGGTTTACGTTGGGAGC	miRNA	miR-629
t0084863	18	1	TGGGTTTAAGTTGGGAGA	miRNA	miR-629
t0089297	18	1	TGGGTTTACGTTGGGAGG	miRNA	miR-629
t0090603	20	1	TCTAGTAAGAGTGGCAGGAG	miRNA	miR-628-3p
t0092963	21	1	TTCTAGTAAGAGTGGCAGGGG	miRNA	miR-628-3p
t0054756	20	1	TCTAGTAAGAGTGGCAGACG	miRNA	miR-628-3p
t0056934	20	1	TCTAGTAAGAGTGACAGTCG	miRNA	miR-628-3p
t0059248	20	1	TCTAGTAAGAGTGGCAGGGG	miRNA	miR-628-3p
t0060199	23	1	GACTATAGAAATTTCCCCCTGAA	miRNA	miR-625*
t0063263	20	1	GACTATAGAAGTTTCCCCCT	miRNA	miR-625*
t0065351	20	1	TAGTACCAGGACCTTGTGTT	miRNA	miR-624*
t0075109	22	1	TAGTACCAGTACATTGTGTTCA	miRNA	miR-624*
t0089050	21	1	AAGTACCAGTACCTTGTGTTC	miRNA	miR-624*
t0091386	22	1	AAGTCATTGGAGGGGTTGAGCA	miRNA	miR-616
t0092293	19	1	TATGTCATCGTTGTCATCG	miRNA	miR-598
t0092427	21	1	TACGTCATCGTTGTCATTGTC	miRNA	miR-598
t0048824	20	1	TACGTCATCGCTGTCATCGT	miRNA	miR-598
t0053556	22	1	TACGTCATCGCTGTCATCGTCA	miRNA	miR-598

t0062074	22	1	TACGGCATCGTTGTCATCGTCA	miRNA	miR-598
t0063408	25	1	TACGTCATCGTCGTCATCGTCAATT	miRNA	miR-598
t0093415	22	1	TACGACATCGTTGTCATCGTCA	miRNA	miR-598
t0043012	22	1	TACGTCATCGTTGTTATGGTCA	miRNA	miR-598
t0044156	22	1	TACGTCCTCGTTGTCATCGTCA	miRNA	miR-598
t0051531	22	1	AACGTCATCGTTGTCATCGTCA	miRNA	miR-598
t0063669	22	1	TAAGTCATCGTTGTCATCGTCA	miRNA	miR-598
t0089098	24	1	TACGTCATCGTTGTCATCGTCCTT	miRNA	miR-598
t0071325	19	1	TACGTCATCGTTGTAATCG	miRNA	miR-598
t0041087	22	1	TACGTCATCGTTGTCATGGTCA	miRNA	miR-598
t0045020	22	1	TACGTCATCGTTGTAATCGTCA	miRNA	miR-598
t0047378	22	1	TACGTCATCGTTGTCATCGCCA	miRNA	miR-598
t0049338	21	1	AACGTCATCGTTGTCATCGTC	miRNA	miR-598
t0056334	22	1	TACGTCATCGTTATCATCGTCA	miRNA	miR-598
t0057844	21	1	TACCTCATCGTTGTCATCGTC	miRNA	miR-598
t0060853	22	1	TACGTCATCGTTGTCATCATCA	miRNA	miR-598
t0061489	22	1	TACGTAATCGTTGTCATCGTCA	miRNA	miR-598
t0063382	21	1	TACGTCATCGTTGTCATCGAC	miRNA	miR-598
t0064152	21	1	GAGCTTATTCATAAAAGTGAA	miRNA	miR-590-5p
t0064206	22	1	GAGCTTATTCATAAAAGGGCAA	miRNA	miR-590-5p
t0068474	21	1	GAGCTTATTAATAAAAGTGCA	miRNA	miR-590-5p
t0071075	22	1	GAGCTTATTCATAAAAGGGCAG	miRNA	miR-590-5p
t0071534	22	1	AGAACAAATACCGGTTCCCAGA	miRNA	miR-589*
t0079624	21	1	TGAGAATCACGTCTGCTCTGA	miRNA	miR-589
t0080169	21	1	TTATGGTTTGCCTGAGACTGA	miRNA	miR-584
t0080476	21	1	TTATGGTTTGTCTGGGACTGA	miRNA	miR-584
t0081082	21	1	TTATGGTTTGCCTGGGAATGA	miRNA	miR-584
t0084346	21	1	TTATGGTTTACCTGGGACTGA	miRNA	miR-584
t0085452	21	1	TTATGGATTGCCTGGGACTGA	miRNA	miR-584
t0089693	21	1	TTATGGTTTGCCTGGGGCTGT	miRNA	miR-584
t0090650	21	1	TTATGGTTTGACTGGGACTGA	miRNA	miR-584
t0052193	21	1	TTATGGTTTGCCTGGGCCTGA	miRNA	miR-584
t0054392	21	1	TTCTGGTTTGCCTGGGACTGA	miRNA	miR-584
t0072764	21	1	TTATGGTTTGCCTGGGACTGC	miRNA	miR-584
t0088788	21	1	TTATGGTTTGTCTGGGGCTGA	miRNA	miR-584
t0077220	21	1	TTATGCTTTGCCTGGGACTGA	miRNA	miR-584
t0092769	22	1	ATTATGGTTTGCCTGGGACTGC	miRNA	miR-584
t0043659	21	1	TTATGGTCTGCCTGGGACTGA	miRNA	miR-584
t0044583	22	1	TTATGGTTTGCCTGGGACTGCT	miRNA	miR-584
t0048192	19	1	GAGATGTGGAAAAATTGGA	miRNA	miR-576-3p
t0049805	22	1	AAAGTAATTGCAGTTTTTGACT	miRNA	miR-548w
t0056972	21	1	CAAAGGTGATCGTGGTTTTTG	miRNA	miR-548t
t0061108	19	1	CAAAAGTGCTCGTGGTTTT	miRNA	miR-548t
t0062065	19	1	CAAAAGGGATCGTGGTTTT	miRNA	miR-548t
t0065293	21	1	AGAAGTATTTGCGGGTTTTGT	miRNA	miR-548l
t0066033	21	1	AAAAGGATTTGCGGGTTTTGT	miRNA	miR-548l
t0075361	22	1	AAACGTACTTGCGGATTTTGCT	miRNA	miR-548k
t0081760	22	1	AAAAGTACTTGCTGATTTTGCT	miRNA	miR-548k
t0083792	19	1	AAAGTAATCATGGTCTTTG	miRNA	miR-548j
t0088443	20	1	AAAAACTGAGACTACTATTG	miRNA	miR-548e
t0092504	22	1	AAAAACTGAGACTACTTTCGCA	miRNA	miR-548e
t0093022	22	1	AAAGTGATTGTGGTCTTTGTCA	miRNA	miR-548d-5p
t0064075	22	1	AAACGGCAATGATTTTTGTACA	miRNA	miR-548al
t0059857	21	1	AAAAGGAATTGCGGTTTTTGA	miRNA	miR-548ak
t0060309	20	1	AAAAGTAATTGCGGTTTTTG	miRNA	miR-548ak
t0068337	19	1	AACCACAATTACCTTTGCA	miRNA	miR-548aa
t0090453	18	1	TACAGTTTGATAACTGAA	miRNA	miR-542-3p
t0061764	24	1	AAGGGATTCTGATGTTGGTCAATC	miRNA	miR-541

t0069474	23	1	AGGGGATTCTGATGTTGGTCATC	miRNA	miR-541
t0050193	19	1	CGGGAAGGAATTCTGATAT	miRNA	miR-541
t0051596	21	1	CATGCCTTGAGGGTAGGACCG	miRNA	miR-532-5p
t0053482	23	1	CATGCCTTGAGTGTAGGACCGTC	miRNA	miR-532-5p
t0044496	21	1	CATGCCTTGAGTGTAGGAACG	miRNA	miR-532-5p
t0073449	22	1	CATGCCTTGAGTGTAGGACAGT	miRNA	miR-532-5p
t0055220	22	1	CATGCCCTGAGTGTAGGACCGT	miRNA	miR-532-5p
t0053265	23	1	CATGCCTTGAGTGTAGGCCCGTA	miRNA	miR-532-5p
t0046759	23	1	CATGCCTTGAGTGTAGGACTGTT	miRNA	miR-532-5p
t0069620	22	1	CATGCCTTGAGAGTAGGACCGT	miRNA	miR-532-5p
t0065798	19	1	CCCACACCCAAGGCTTGAA	miRNA	miR-532-3p
t0077657	21	1	CCTCCCACACCTAAGGCTTGC	miRNA	miR-532-3p
t0060901	21	1	CTCCCACACCCAAGGCTTGCC	miRNA	miR-532-3p
t0062672	22	1	CCTCCCACGCCCAAGGCTTGCA	miRNA	miR-532-3p
t0073318	22	1	GCTCCCACACCCAAGGCTTGCA	miRNA	miR-532-3p
t0046110	22	1	CCTCCCACACCCAAGGGTTGCA	miRNA	miR-532-3p
t0061352	22	1	CCTCCCACACCCAAAGCTTGCA	miRNA	miR-532-3p
t0064517	22	1	CCTCCCACACTCAAGGCTTGCC	miRNA	miR-532-3p
t0066924	21	1	TTCCCACACACAAGGCTTGCA	miRNA	miR-532-3p
t0072500	21	1	CCTCCCACACACAAGGCTTGC	miRNA	miR-532-3p
t0084668	21	1	CCTCCCACACCAAAGGCTTGC	miRNA	miR-532-3p
t0085713	22	1	CCTCCCACACTCAAGGCTTGGA	miRNA	miR-532-3p
t0090903	22	1	CCTTCCACACCCAAGGCTTGCA	miRNA	miR-532-3p
t0044594	22	1	CCTCCCACACCCAAGGCTTCCA	miRNA	miR-532-3p
t0045231	21	1	CCTCCCACACCCAAGGCTTTC	miRNA	miR-532-3p
t0048841	22	1	CCTCCCACACGCAAGGCTTGCA	miRNA	miR-532-3p
t0049165	22	1	CTTCCCCCACCCAAGGCTTGCA	miRNA	miR-532-3p
t0049196	22	1	CCTACCACACCCAAGGCTTGCA	miRNA	miR-532-3p
t0050322	22	1	CCTCCCACACAAAAGGCTTGCA	miRNA	miR-532-3p
t0051215	20	1	CCTCCCACACCTAAGGCTTG	miRNA	miR-532-3p
t0056011	22	1	CCTCCCAGACCCAAGGCTTGCA	miRNA	miR-532-3p
t0057503	22	1	CCTCCCACACCCAAGACTTGCA	miRNA	miR-532-3p
t0059150	22	1	CCACCCACACCCAAGGCTTGCA	miRNA	miR-532-3p
t0060181	22	1	ACTCCCACACCCAAGGCTTGCT	miRNA	miR-532-3p
t0062961	22	1	CATCCCACACCCAAGGCTTGCA	miRNA	miR-532-3p
t0063462	20	1	CCTCCCACACCCATGGCTTG	miRNA	miR-532-3p
t0064296	23	1	ACCTCCCGCACCCAAGGCTTGCA	miRNA	miR-532-3p
t0064328	22	1	CCTCCCACACCCAAGTCTTGCA	miRNA	miR-532-3p
t0064485	22	1	ACTCCCACACCCAAGGCTTGCA	miRNA	miR-532-3p
t0065551	20	1	CATCCCACACCCAAGGCTTG	miRNA	miR-532-3p
t0067490	20	1	CCTCTCACACCCAAGGCTTG	miRNA	miR-532-3p
t0069044	20	1	CCTCCCGCACCCAAGGCTTG	miRNA	miR-532-3p
t0071159	28	1	TGGTGCGGACCAGGGGCATCCGACTGTT	miRNA	miR-5109
t0074341	29	1	CTGGGGCGGACCAGGGGAATCCGACTGT	miRNA	miR-5109
t0077026	22	1	AGTTATAGCATCTGGATGGTTT	miRNA	miR-5108
t0077583	22	1	TCTGGGCACAGACGGATGGACA	miRNA	miR-5107
t0078814	20	1	TGGGCACAGGCGGATGGACC	miRNA	miR-5107
t0079127	24	1	TCTGGGCAAAGGCGGATGGACAGA	miRNA	miR-5107
t0079177	20	1	TCTGGGCACCGGCGGATGGA	miRNA	miR-5107
t0082498	24	1	TCTGGGCACAGGCGGATGGGCAGA	miRNA	miR-5107
t0084467	22	1	TCTGGGCACGGGCGGATGGACA	miRNA	miR-5107
t0084963	22	1	TCTGGGCACAGGAGGATGGACA	miRNA	miR-5107
t0088020	21	1	TCTGGGCACAGGCGGGTGGAC	miRNA	miR-5107
t0089077	22	1	TCTGGGGACAGGCGGATGGACA	miRNA	miR-5107
t0091777	21	1	TCTGGGCACAGGCGGATGGAG	miRNA	miR-5107
t0046968	23	1	TCTGGGCACAGGCGGATGGACCA	miRNA	miR-5107
t0081167	23	1	TGGGCACAGGCGGATGGGCAGGT	miRNA	miR-5107
t0061786	22	1	TCTGGCCACAGGCGGATGGACA	miRNA	miR-5107

t0041471	22	1	TCTCGGCACAGGCGGATGGACA	miRNA	miR-5107
t0042689	24	1	TCTGGACACAGGCGGATGGACAGA	miRNA	miR-5107
t0043912	23	1	TGGGCACAGGCGGATGGACGGGT	miRNA	miR-5107
t0044892	24	1	TCTGGGCACAGGAGGATGGACAGA	miRNA	miR-5107
t0045789	24	1	TCTGGGCAAAGGCGGATGGACAGT	miRNA	miR-5107
t0047894	24	1	TATGGGCACAGGCGGATGGACAGA	miRNA	miR-5107
t0052487	23	1	TCTGGGCACAGCCGGATGGACAA	miRNA	miR-5107
t0052572	24	1	TCTGGGCACCGGCGGATGGACAGT	miRNA	miR-5107
t0053680	22	1	TCTGGGCACAGGCGAATGGACA	miRNA	miR-5107
t0054192	21	1	TCTGGGCACAGGCGGATGGAA	miRNA	miR-5107
t0056935	20	1	TGGGCACAGGCGGAGGGACA	miRNA	miR-5107
t0058850	22	1	TCTGTGCACAGGCGGATGGACA	miRNA	miR-5107
t0061421	22	1	TCTGGGTACAGGCGGATGGACA	miRNA	miR-5107
t0061517	22	1	TCGAATCCCAGCGGTGCCTCCC	miRNA	miR-5100
t0063572	22	1	TCGAATCCCAGCGGAGCCTCCA	miRNA	miR-5100
t0065019	21	1	GGGAGCAAGGAAGTATTGATG	miRNA	miR-505*
t0069801	23	1	GGAAGCCAGGAAGTATTGATGTT	miRNA	miR-505*
t0069907	22	1	GGGAGGGCAGGGCAGGGTTTCC	miRNA	miR-504*
t0071638	21	1	TAGCAGTGGGAACAGTTCTAA	miRNA	miR-503
t0072436	21	1	TAGCAGCGGGAACAGCTCTGA	miRNA	miR-503
t0073619	20	1	TAGCAGCGGAAACAGTTCTA	miRNA	miR-503
t0076029	19	1	TAGCAGCGGGAACAGTTTT	miRNA	miR-503
t0078497	21	1	TAGCAGCGGGAACAGTTCCGA	miRNA	miR-503
t0079033	21	1	TAGCAGCGGGAATAGTTCTGA	miRNA	miR-503
t0079212	21	1	TAGCAGCGGGAAAAGTTCTGA	miRNA	miR-503
t0084739	18	1	AATCCTTGATATCTGGGT	miRNA	miR-500b
t0070103	26	1	CTTGCTATCTGGGCATGATACTATGA	miRNA	miR-500b
t0077242	21	1	TTAGGACTTGCAGTGATGTTT	miRNA	miR-499-5p
t0041301	23	1	TGGTAAGACTGTAGTTGATGTTT	miRNA	miR-499
t0081538	22	1	GTAAGACTGTAGTTGATGTTTT	miRNA	miR-499
t0063060	22	1	GGTAAGACTGTAGTTGATGTTT	miRNA	miR-499
t0044283	23	1	TAAGACTGTAGTTGATGTTATTT	miRNA	miR-499
t0053395	21	1	TAAGACTGTAGTTGATGTTAT	miRNA	miR-499
t0057789	22	1	AAACAAACATGGCGCACTTCTT	miRNA	miR-495
t0076361	22	1	AAACAAACATGGGGCACTTCTT	miRNA	miR-495
t0079371	23	1	TCCTGTACTTAGCTGCCCCGAGT	miRNA	miR-486-5p
t0092706	22	1	TCCTGTACTGTGTTGCCCCGAG	miRNA	miR-486-5p
t0093427	23	1	TCTTGTACTGAGCTGCCCCGGGA	miRNA	miR-486-5p
t0051986	28	1	TCCTGTACTGAGCTGCCCCAAGACGGCT	miRNA	miR-486-5p
t0053336	28	1	CCCTGTACTGAGCTGCCCCGAGACGGGA	miRNA	miR-486-5p
t0051311	19	1	TGTACTGAGATGCCCCGAG	miRNA	miR-486-5p
t0044885	24	1	TCCTGTACTGCGCTGCCCCGAGAA	miRNA	miR-486-5p
t0063380	21	1	TTCTGTACTGAGTTGCCCCGA	miRNA	miR-486-5p
t0065827	26	1	TCCTGTACTGAGCTGCCCAGCGCAAG	miRNA	miR-486-5p
t0083844	21	1	TCCTGTACTGAACTGCCACGA	miRNA	miR-486-5p
t0092902	21	1	TCCTGTACTGAGCTGACCCGT	miRNA	miR-486-5p
t0062890	22	1	TCCTGTACTGCGCTGCCCCGAA	miRNA	miR-486-5p
t0067280	21	1	TCCTGTACTGCGCTGCCCCAA	miRNA	miR-486-5p
t0040872	20	1	TCTGTACTGAGCTGACCCGA	miRNA	miR-486-5p
t0040920	24	1	TCCTGTACTGAACTGCCCCGAGAA	miRNA	miR-486-5p
t0040979	29	1	TCCTGTACTGAGCTGCCCCGAGGCGGCC(	miRNA	miR-486-5p
t0041061	20	1	CTGTACTGAGCTGCCCCGGG	miRNA	miR-486-5p
t0041120	24	1	TCCTGTACTGAGCTGACCCGAGGA	miRNA	miR-486-5p
t0041217	29	1	TCCTGTACCGAGCTGCCCCGAGACGGCT(	miRNA	miR-486-5p
t0041219	23	1	CCCTGTACTGAGCTGCCCCGAGT	miRNA	miR-486-5p
t0041341	21	1	TCTTGTACTGAGCTGCCACGA	miRNA	miR-486-5p
t0041412	23	1	TCCTGAACTGAGCTGCCCCGAGT	miRNA	miR-486-5p
t0041432	20	1	TCCTGTACTGAGCTGCCCGG	miRNA	miR-486-5p

t0041472	18	1	TCCTGTACTGAGGTGCCC	miRNA	miR-486-5p
t0041595	22	1	TTCTGTACTGAGCTGCCCAGAG	miRNA	miR-486-5p
t0041602	18	1	TCCTGTACTGCGCTGCCC	miRNA	miR-486-5p
t0041607	21	1	TACTGTACTGAGCTGCTCCGA	miRNA	miR-486-5p
t0041709	26	1	TCCTGTACTGAGATGCCCCGAGAAGA	miRNA	miR-486-5p
t0041911	21	1	TCTTGTTCTGAGCTGCCCCGA	miRNA	miR-486-5p
t0042016	22	1	TTCTGTACTGAGATGCCCCGAG	miRNA	miR-486-5p
t0042141	20	1	TCCTGTCCTGAGCTGCCCCG	miRNA	miR-486-5p
t0042267	23	1	TCCTGTACTGAGCTGCCACGAGG	miRNA	miR-486-5p
t0042341	23	1	TCCTGTACTGAGCTGCCCCGTGA	miRNA	miR-486-5p
t0042414	24	1	TCCTGTACTGAGCTGCCCCGAGCT	miRNA	miR-486-5p
t0042550	21	1	TCCTGTACTGCGCTCCCCCGA	miRNA	miR-486-5p
t0042692	20	1	TCCTGTACTGAGCTGCCAAG	miRNA	miR-486-5p
t0042768	22	1	TACTGTACTGAGCTGCCCCGAA	miRNA	miR-486-5p
t0042998	24	1	TCCTGTAATGAGCTGCCCCGAGAT	miRNA	miR-486-5p
t0043009	22	1	TCCTGTACTGGGCTGCCCTGAG	miRNA	miR-486-5p
t0043104	22	1	TCCTGTACTGAGCTGCCCCAAA	miRNA	miR-486-5p
t0043382	22	1	ACCTGTACTGAGCTGCTCCGAG	miRNA	miR-486-5p
t0043490	23	1	TCTTGTACTGAGGTGCCCCGAGA	miRNA	miR-486-5p
t0043515	23	1	TTCTGTACTGAGCTGCCCCGCGT	miRNA	miR-486-5p
t0043822	24	1	TCCTGTACTGAGCTGTCCCGAGAA	miRNA	miR-486-5p
t0043957	22	1	TCCTGTAATGAGCTGCCCCGAT	miRNA	miR-486-5p
t0043963	20	1	TCCTGTACTGAGGTGCCCCG	miRNA	miR-486-5p
t0044392	22	1	TTCCGTACTGAGCTGCCCCGAG	miRNA	miR-486-5p
t0044462	26	1	TCCTGTACTGAGCTGCCCCAAGATGG	miRNA	miR-486-5p
t0044478	23	1	TCCTGTACTTAGCTGCCCCGAGA	miRNA	miR-486-5p
t0044505	28	1	TCCTGTACTGAGCTGCCCCGCGACTGCA	miRNA	miR-486-5p
t0044832	20	1	TTGTATTGAGCTGCCCCGAG	miRNA	miR-486-5p
t0045212	25	1	TCCTGTACTGAGCTGACCCGAGAGA	miRNA	miR-486-5p
t0045242	22	1	TCCTGTGCTGAGCTGCTCCGAG	miRNA	miR-486-5p
t0045344	21	1	TCTTGCACTGAGCTGCCCCGA	miRNA	miR-486-5p
t0045467	24	1	TCCTGTACTGAGATGCCCCGAGAT	miRNA	miR-486-5p
t0045718	24	1	TCCTGTACTGAGCTGCCTCGAGAT	miRNA	miR-486-5p
t0045821	20	1	CTGTGCTGAGCTGCCCCGAG	miRNA	miR-486-5p
t0045964	23	1	TTCTGTACTGAGCTGCCCCGAGG	miRNA	miR-486-5p
t0046084	23	1	TCCTGTACTGAGCTACCCCGAGT	miRNA	miR-486-5p
t0046118	22	1	TCCTGTACTTAGCTCCCCCGAG	miRNA	miR-486-5p
t0046130	28	1	ACCTGTACTGAGCTGCCCCGAGCGGCAT	miRNA	miR-486-5p
t0046332	19	1	CCCTGTACTGAGCTGCCCC	miRNA	miR-486-5p
t0046442	21	1	TCTTGTACTGAGCTGCACCGA	miRNA	miR-486-5p
t0046610	22	1	TCATGTACTAAGCTGCCCCGAG	miRNA	miR-486-5p
t0046658	19	1	TTGTACTGAGCTGCACCGA	miRNA	miR-486-5p
t0047024	25	1	TCCTGTACTGAGCTGCCCCGAGGGG	miRNA	miR-486-5p
t0047473	22	1	TCCTGTCCTGAGCTGTCCCGAG	miRNA	miR-486-5p
t0047519	24	1	TCCTGTACTGAGCTGCCCCGGGAT	miRNA	miR-486-5p
t0047731	25	1	TGTACTGAGCTGCCCCGAGACGGCA	miRNA	miR-486-5p
t0047974	20	1	TCCGGTACTGAGCTGTCCCG	miRNA	miR-486-5p
t0047985	21	1	ACCTGTACTGAGCTGCCCCAA	miRNA	miR-486-5p
t0048010	22	1	TCCTGTAATGAGCTGCCACGAG	miRNA	miR-486-5p
t0048057	21	1	TCCTGTACTGCGCTGTCCCGA	miRNA	miR-486-5p
t0048271	22	1	TCCAGTACTGAACTGCCCCGAG	miRNA	miR-486-5p
t0048286	26	1	TCCTGTACTGAGCTGCCACGAGACGG	miRNA	miR-486-5p
t0048343	28	1	TCCTGAACTGAGCTGCCCCGAGCCCGCA	miRNA	miR-486-5p
t0048373	20	1	TTGCACTGAGCTGCCCCGAG	miRNA	miR-486-5p
t0048390	23	1	TCCGGTACTGAGCTGCCCCGAGT	miRNA	miR-486-5p
t0048614	22	1	TCCTGTACTGAGCTGCCCCGCC	miRNA	miR-486-5p
t0048831	23	1	TCCTGTACTGAGCTGCACCGCGA	miRNA	miR-486-5p
t0048843	29	1	TCCTGTACTGAGCTGCCCCGAGACGGAA(	miRNA	miR-486-5p

t0049054	21	1	TCCTGTACTGCGCTGCCCCGT	miRNA	miR-486-5p
t0049058	23	1	TCCTGTCCTGAGCTGCCCCGAGT	miRNA	miR-486-5p
t0049089	22	1	TCCTGTACTGAGATGTCCCGAG	miRNA	miR-486-5p
t0049186	21	1	TCCTGTACTGAGCTGCTCCGC	miRNA	miR-486-5p
t0049305	22	1	TCCTGTACTGTGCTGTCCCGAG	miRNA	miR-486-5p
t0049342	20	1	CTGTACTGAGCCGCCCCGAG	miRNA	miR-486-5p
t0049593	29	1	TCCTGTACTGAGCTGCCCCGTGATGAGCG	miRNA	miR-486-5p
t0049699	22	1	TCCTGTACTGCGCTGTCCCGAG	miRNA	miR-486-5p
t0049704	23	1	TCCTGTACTGAGCTGCCCCGTGC	miRNA	miR-486-5p
t0049736	26	1	TCCTGTACTAAGCTGCCCCGAGGTCC	miRNA	miR-486-5p
t0049907	23	1	TCCTGTACTGAGCTGGCCCGAGT	miRNA	miR-486-5p
t0049983	21	1	TCCTGTACTGAGATGCCTCGA	miRNA	miR-486-5p
t0050095	22	1	TCCTGTATTGAGCTGCCCCGAT	miRNA	miR-486-5p
t0050112	22	1	TCCTGTACTGAGCTGCACCGAT	miRNA	miR-486-5p
t0050179	21	1	TCCTGTACCGAGCTGCCCCGC	miRNA	miR-486-5p
t0050235	23	1	TCCTCTACTGAGCTGCCCCGAGG	miRNA	miR-486-5p
t0050451	27	1	TCTATACTGAGCTGCCCCGAGACGGCA	miRNA	miR-486-5p
t0050472	21	1	TCCTGTAATGAGCTGTCCCGA	miRNA	miR-486-5p
t0050571	23	1	TCCTGTACTGAGATGCCCCGCGT	miRNA	miR-486-5p
t0050801	28	1	TCCTGCACTGAGCTGCCCTGAGACGGCT	miRNA	miR-486-5p
t0051057	20	1	TCCTGTACTGAGCGGCCCCG	miRNA	miR-486-5p
t0051061	24	1	TCCTGTACTGAGCTGCCCCGGGGA	miRNA	miR-486-5p
t0051433	19	1	CTATACTGAGCTGCCCCGA	miRNA	miR-486-5p
t0051975	18	1	CTGTACTGAGCTGACCCG	miRNA	miR-486-5p
t0052134	22	1	TCTTGTACTGAGCTGCCCAGAG	miRNA	miR-486-5p
t0052150	21	1	TCCCGTACTGAGCTGCCCCGT	miRNA	miR-486-5p
t0052200	21	1	TCCTGGACTGAGCTGACCCGA	miRNA	miR-486-5p
t0052414	22	1	TCCTGTACTGAGCCGCCCCGAC	miRNA	miR-486-5p
t0052425	21	1	TACTGTACTGAGATGCCCCGA	miRNA	miR-486-5p
t0053001	20	1	TCCTGTACTGAGCTGACACG	miRNA	miR-486-5p
t0053032	18	1	TCCTGTACTGAGCTGCGC	miRNA	miR-486-5p
t0053157	22	1	TCCTGTACTGAGCTGGACCGAG	miRNA	miR-486-5p
t0053309	19	1	TTGTACTGAGCTGCTCCGA	miRNA	miR-486-5p
t0053333	22	1	TCCTGTACTAAGCTGCCCCGAA	miRNA	miR-486-5p
t0053539	19	1	TGTACTGAGCTGCCCCGCA	miRNA	miR-486-5p
t0053559	20	1	TCTTATACTGAGCTGCCCCG	miRNA	miR-486-5p
t0053956	21	1	TGCTGTACTGAGCTGCTCCGA	miRNA	miR-486-5p
t0054277	28	1	TCCTGTACTGAGCTGCCCCGAGACGCCG	miRNA	miR-486-5p
t0054575	21	1	TCCTGTACTGAGCCGCCCCGC	miRNA	miR-486-5p
t0054605	24	1	TCCTGTACTGAGCTACCCCGAGTT	miRNA	miR-486-5p
t0054995	20	1	CTGTACTGCGCTGCCCCGAG	miRNA	miR-486-5p
t0055225	22	1	TCCTGTACTGTGCTGCCCCGAT	miRNA	miR-486-5p
t0055820	20	1	TCCTGTACTAAGCTGCCCCG	miRNA	miR-486-5p
t0055978	24	1	TCCTGTACTGAGCAGCCCCGAGAA	miRNA	miR-486-5p
t0056056	22	1	TCCTGTACTGACCTGCCCCGAA	miRNA	miR-486-5p
t0056074	22	1	TCCTGGACTGAGCTGACCCGAG	miRNA	miR-486-5p
t0056469	23	1	TCCTGTACTGAGCTGCCGCGAGA	miRNA	miR-486-5p
t0056580	21	1	TCCTGTACTGAGCGGCCCCAA	miRNA	miR-486-5p
t0056618	22	1	TCCTGTAATAAGCTGCCCCGAG	miRNA	miR-486-5p
t0056711	22	1	CCCTGTACTGAGCTGCCCCGAT	miRNA	miR-486-5p
t0056756	22	1	GCCTGTACTGAGCTGCCCCGCG	miRNA	miR-486-5p
t0056896	27	1	TCCTGTACTGAGCTGCCGCGACGCTAC	miRNA	miR-486-5p
t0056973	19	1	TGTACTGAGGTGCCCCGAA	miRNA	miR-486-5p
t0056976	22	1	TCCTGTACCGAGCTGCCCCGAC	miRNA	miR-486-5p
t0057022	22	1	TCCTGTACTGAGCTGGCCAGAG	miRNA	miR-486-5p
t0057106	22	1	TCCTGTCCTGAGCTGCCCCGAA	miRNA	miR-486-5p
t0057187	28	1	TCCTGTACTGAGCTGCCCCCAGACGGCT	miRNA	miR-486-5p
t0057516	21	1	TCCGGTACTGGGCTGCCCCGA	miRNA	miR-486-5p

t0057531	24	1	ACCTGTACTGAGCTGCCCCGAGAA	miRNA	miR-486-5p
t0057680	23	1	TCCTGTACAGAGCTGCCCCGAGT	miRNA	miR-486-5p
t0057681	26	1	TCCTGTACTGAGTTGCCCCGAGCAAG	miRNA	miR-486-5p
t0057824	24	1	TCCTGTACTGAGCTGCCCCGCGTA	miRNA	miR-486-5p
t0057983	22	1	TCCTGTACTGAGCTGTCACGAG	miRNA	miR-486-5p
t0058178	18	1	TTGTAATGAGCTGCCCCG	miRNA	miR-486-5p
t0058284	22	1	TCCTGTACTGAGCTGCTACGAG	miRNA	miR-486-5p
t0058492	20	1	CTGTACTGAGCTGCCACGAG	miRNA	miR-486-5p
t0058622	22	1	TCCCGTACTTAGCTGCCCCGAG	miRNA	miR-486-5p
t0058701	24	1	TCCTATACTGAGCTGCCCCGAGAT	miRNA	miR-486-5p
t0058732	22	1	TCCTGTAATGAGCTGCCCGGAG	miRNA	miR-486-5p
t0058893	22	1	TCCTGTACTAAGCTGCCCTGAG	miRNA	miR-486-5p
t0058956	22	1	TCCTGTACCGAGCTGCCCCGAA	miRNA	miR-486-5p
t0058968	23	1	TCCGGTACTGAGCTGCCCCGAGA	miRNA	miR-486-5p
t0059353	21	1	TCCTGTACTGTGCTGCCCCGT	miRNA	miR-486-5p
t0059459	23	1	TCCTGTACTGAGCTGCCCAGAGG	miRNA	miR-486-5p
t0059833	22	1	TCCTGTACTGAGTTGCCCCAAG	miRNA	miR-486-5p
t0059904	24	1	TCCTGTACTGAGCTGCCCCGAGGG	miRNA	miR-486-5p
t0060066	22	1	TCCTGTACTGAGCTGGCCCGAT	miRNA	miR-486-5p
t0060213	25	1	CCCTGTACTGAGCTGCCCCGAGAGA	miRNA	miR-486-5p
t0060521	24	1	TCCTGTACTGAGCTGCCCCAAGAG	miRNA	miR-486-5p
t0060864	22	1	TCCTGCACTGAGCTGCCCCGAA	miRNA	miR-486-5p
t0060992	20	1	TCCTGTAATAAGCTGCCCCG	miRNA	miR-486-5p
t0060998	22	1	TCCTGTACTAAGCTGCCCCGCG	miRNA	miR-486-5p
t0061019	21	1	TACTGTACTGAGCTGCCCCGT	miRNA	miR-486-5p
t0061113	29	1	TCCTGTACTGAGCTGCCCCGAGCTCGGCC	miRNA	miR-486-5p
t0061487	22	1	TCTGTACTGAGCTGCCCCGAGT	miRNA	miR-486-5p
t0061649	22	1	TCCTGTACTTAGCTGCCCCGCG	miRNA	miR-486-5p
t0061670	21	1	TCATGTACTGAGCTGCCCCGC	miRNA	miR-486-5p
t0062188	26	1	TCCTGTACTGAGCTGCCACGAGGTCC	miRNA	miR-486-5p
t0062295	21	1	ATCCTGTACTGAGATGCCCCG	miRNA	miR-486-5p
t0062735	21	1	TCTTGTACTGAGATGCCCCGA	miRNA	miR-486-5p
t0062917	28	1	TTCTGTACTGAGCTGCCCCGAGCCCGCA	miRNA	miR-486-5p
t0062926	21	1	TCCTGTACTGAGCTGACTCGA	miRNA	miR-486-5p
t0063031	21	1	GCCTGTACTGAGCTGCCGCGA	miRNA	miR-486-5p
t0063205	21	1	TCCTCTACTGAGCGGCCCCGA	miRNA	miR-486-5p
t0063356	29	1	TCCTGTACTGAGCTGCCCCGAGGCGGCAA	miRNA	miR-486-5p
t0063357	22	1	TCCTGTAATGAGCTGACCCGAG	miRNA	miR-486-5p
t0063390	20	1	TCCTGTAATGAGCTGCCCAG	miRNA	miR-486-5p
t0063429	20	1	CTGGACTGAGCTGCCCCGAG	miRNA	miR-486-5p
t0063515	22	1	TCCTGTACTGAGCTGCCACGGG	miRNA	miR-486-5p
t0063531	24	1	TCCTGTACTAAGCTGCCCCGAGAT	miRNA	miR-486-5p
t0063604	24	1	TCCTGTACTGAGCTGCCCCGTGAA	miRNA	miR-486-5p
t0063873	24	1	TCCTGTACTGAGCTGCCCTGAGAA	miRNA	miR-486-5p
t0063914	23	1	TCCTGTACTGATCTGCCCCGAGA	miRNA	miR-486-5p
t0064111	28	1	TCCTGTACTGAGCTGACCCGAGACGGCA	miRNA	miR-486-5p
t0064359	23	1	TCTTGTACTGAGCTGGCCCGAGA	miRNA	miR-486-5p
t0064416	21	1	TCCTGTACTGAGCTGTCCAGA	miRNA	miR-486-5p
t0064873	22	1	TCCTGTACTGAGTTGCCCCGCG	miRNA	miR-486-5p
t0064952	23	1	TCCTGTACTGAGCTCCCCCGCGT	miRNA	miR-486-5p
t0065197	23	1	TCCTGTACTGACCTGCCCCGAGT	miRNA	miR-486-5p
t0065449	26	1	TCCTGTACTGAGCTGCCACGAGCAAG	miRNA	miR-486-5p
t0065466	28	1	TCCTGTACTGAGCTGCCCCGAGACTGAA	miRNA	miR-486-5p
t0065574	21	1	TCTTGTACTGAGCTGCCCAGA	miRNA	miR-486-5p
t0065579	22	1	TCTTGTACTGAGCTGCCCCGAA	miRNA	miR-486-5p
t0065658	20	1	TCCTGTACTGAGCTGCCATG	miRNA	miR-486-5p
t0065757	24	1	TCCTGTACTGAGCTGTCCCGAGGA	miRNA	miR-486-5p
t0065847	24	1	TCCTGTACTGAGCTGCCCCGAAAA	miRNA	miR-486-5p

t0065857	27	1	TTCTGTACTGAGCTACCCCGAGACGGC	miRNA	miR-486-5p
t0066198	27	1	TCCTGTACTGAGCTGCCCCGAGCTGAT	miRNA	miR-486-5p
t0066298	23	1	TCATGTACTGAGCTGCCCCGAGG	miRNA	miR-486-5p
t0066380	24	1	TCCTGTACTGAGCTGCCCCGAGCG	miRNA	miR-486-5p
t0066445	27	1	TCCTGTACTGAGCTGCCCCGACGCTAA	miRNA	miR-486-5p
t0066631	20	1	TCCTGTACTGAGCTGCCCCT	miRNA	miR-486-5p
t0066657	21	1	ACCTGTACTGAGCTCCCCCGA	miRNA	miR-486-5p
t0067042	28	1	TCCTGTACTGAGCTGCCCCGAGCGGAAT	miRNA	miR-486-5p
t0067273	21	1	TTCTGTACTGAGCTGCCCAGA	miRNA	miR-486-5p
t0067359	24	1	TCCTGTACTGAGCTGACCCGAGAA	miRNA	miR-486-5p
t0067516	23	1	TCCTGTACTGAGCTGCCTCGAGC	miRNA	miR-486-5p
t0067533	20	1	TCCTGTACTGAGCCGCCCCG	miRNA	miR-486-5p
t0067881	28	1	TCATGTACTGAGCTGCCCCGAGACTGCA	miRNA	miR-486-5p
t0068010	23	1	TCTTGTACTGCGCTGCCCCGAGA	miRNA	miR-486-5p
t0068069	22	1	TCCTGTACTGAGATGCCACGAG	miRNA	miR-486-5p
t0068324	28	1	TACTGTACTGAGCTGCCCCGAGACGGAA	miRNA	miR-486-5p
t0068578	23	1	TCCTGTAATGAGCTGCCCCGAAA	miRNA	miR-486-5p
t0068639	23	1	TCTTGTACTGAGCTGCCCCCAGA	miRNA	miR-486-5p
t0068678	22	1	ACCTGTACTGAGCTGCCCCGAT	miRNA	miR-486-5p
t0068812	28	1	TCCTGTACTGAGCTGCCCCGAGAAGGCA	miRNA	miR-486-5p
t0068848	29	1	TCCTGTACTGAGCTGCCCCGAGACGGCC(	miRNA	miR-486-5p
t0068887	22	1	TCCTGTACTGAGCTGTACCGAG	miRNA	miR-486-5p
t0068917	29	1	TCCTGTACCGAGCTGCCCCGAGATGAGC(	miRNA	miR-486-5p
t0068985	20	1	TACTGTACTGAGCTGCCCCG	miRNA	miR-486-5p
t0068989	23	1	TCCTGTACTGAGCTGTCCCGAGT	miRNA	miR-486-5p
t0069025	28	1	TCCTGTACTGACCTGCCCCGAGACGGCA	miRNA	miR-486-5p
t0069121	23	1	TCCTGTAGTGAGCTGCCCCGAGA	miRNA	miR-486-5p
t0069222	24	1	TCCTGTACTGAGCTGCCTCGAGAA	miRNA	miR-486-5p
t0069501	22	1	TCATGTACTGAGCTGCCCCGCG	miRNA	miR-486-5p
t0069792	23	1	TCCTGTACTGAGCTGCCCTGAAA	miRNA	miR-486-5p
t0069996	21	1	TCTTGTCCTGAGCTGCCCCGA	miRNA	miR-486-5p
t0070029	22	1	TTCTGGACTGAGCTGCCCCGAG	miRNA	miR-486-5p
t0070248	20	1	TTGTACTGAGCTGCACCGAG	miRNA	miR-486-5p
t0070275	23	1	TCCTGTACTAAGCTGCCCCGCGT	miRNA	miR-486-5p
t0070288	21	1	TTCTGCACTGAGCTGCCCCGA	miRNA	miR-486-5p
t0070343	28	1	TCTTGTACTGAGCTGCCCCGAGACGGCA	miRNA	miR-486-5p
t0070374	21	1	TCCTGTACTGAGCGGCCTCGA	miRNA	miR-486-5p
t0070450	21	1	TCCTGTAATGAGCTGCACCGA	miRNA	miR-486-5p
t0070468	28	1	TCCTGTACTGAGCTGCCCCGAGGCGGTA	miRNA	miR-486-5p
t0070471	22	1	TCCTGTACTGAGCTGTCCAGAG	miRNA	miR-486-5p
t0070848	19	1	TTGTACTGAGCTGCCCCTA	miRNA	miR-486-5p
t0070880	26	1	TCCTGTACTGAGTTGCCCCGCGAAGA	miRNA	miR-486-5p
t0071148	23	1	TCCTCTACTGAGCTGCCCCGGGT	miRNA	miR-486-5p
t0071532	23	1	TCCTGGACTGAGCTGCCCCGAGT	miRNA	miR-486-5p
t0071548	19	1	TCCTGTACTGAGCTGCGCG	miRNA	miR-486-5p
t0071613	22	1	TCCTGTACTGAGCTCCCCCGCG	miRNA	miR-486-5p
t0071689	22	1	TCCTGTACTGAGCTGCACCGAA	miRNA	miR-486-5p
t0071808	26	1	TCCTGTACTGAGCTGCCCCAAGGTCC	miRNA	miR-486-5p
t0071836	19	1	TGTACTGAGCTGCCCCAAG	miRNA	miR-486-5p
t0071860	28	1	TCCTGTACTGAGCTGCCCCGCGATGGCG	miRNA	miR-486-5p
t0072068	20	1	CTGTACTGAGCTACCCCGAG	miRNA	miR-486-5p
t0072142	28	1	TCCTGTACTGAGCTGCCCCAAGACTGCA	miRNA	miR-486-5p
t0072266	22	1	TCTTATACTGAGCTGCCCCGAG	miRNA	miR-486-5p
t0072342	29	1	TCCTGTACTGAGCTGCCCCGAGACGGCA/	miRNA	miR-486-5p
t0072431	22	1	TCTTGTACTGAGATGCCCCGAG	miRNA	miR-486-5p
t0072636	21	1	TCCTGTACTGCGCTGCTCCGA	miRNA	miR-486-5p
t0072906	22	1	ATCCTGTACTGAGCGGCCACGA	miRNA	miR-486-5p
t0072940	28	1	TCCTGTACTGCGCTGCCCCGAGCCCGCA	miRNA	miR-486-5p

t0072964	26	1	TCCTGTACTGAGCTGCCCCGACGGGC	miRNA	miR-486-5p
t0072981	21	1	TCTTGTACTAAGCTGCCCCGA	miRNA	miR-486-5p
t0073001	21	1	TTCTGGACTGAGCTGCCCCGA	miRNA	miR-486-5p
t0073043	22	1	TCCTGTATTGAGCTGGCCCGAG	miRNA	miR-486-5p
t0073088	28	1	TCCTGTACTCAGCTGCCCCGAGACGGTA	miRNA	miR-486-5p
t0073284	23	1	TCCAGTACTGAGCTGCCCCGAGA	miRNA	miR-486-5p
t0073336	23	1	TCTTGTAATGAGCTGCCCCGAGA	miRNA	miR-486-5p
t0073443	23	1	TCCTGTACTGAGCTGCCCCGATT	miRNA	miR-486-5p
t0073761	28	1	TCCTGTACTGAGCTGCCCCGAGCTACTC	miRNA	miR-486-5p
t0073844	28	1	TCCTGTACTGAGCTGCTTCGAGACGGCA	miRNA	miR-486-5p
t0073849	21	1	TCCTGTACTGAGATGCCCTGA	miRNA	miR-486-5p
t0074043	18	1	TGTACTGAGCTGCCCCGC	miRNA	miR-486-5p
t0074057	21	1	CTGTACTGAGCTGCACCGAGA	miRNA	miR-486-5p
t0074124	22	1	TCCTGTACAGAGCTGCCCCGAT	miRNA	miR-486-5p
t0074243	21	1	TTGTACTGAGCCGCCCCGAGA	miRNA	miR-486-5p
t0074279	24	1	TCCTGTACTGAGCTGCCCCGATGG	miRNA	miR-486-5p
t0074340	21	1	TCCTGTACTGAGCTGCACAGA	miRNA	miR-486-5p
t0074476	21	1	TCCTGTACTGAGCTGCCCTGC	miRNA	miR-486-5p
t0074529	28	1	TCCTGTACTGAGCTGCCCCGACGCTCGG	miRNA	miR-486-5p
t0074784	22	1	TCCTGGACTGAGCTGCCCCGAA	miRNA	miR-486-5p
t0074867	24	1	TCCTGTACTGATCTGCCCCGAGAT	miRNA	miR-486-5p
t0075596	22	1	TCCTGTAATGAGCCGCCCCGAG	miRNA	miR-486-5p
t0075644	21	1	TCCTGTACTGAGCGGCCACGA	miRNA	miR-486-5p
t0075649	23	1	TCCTGTACTGAGCTGCACCGAGG	miRNA	miR-486-5p
t0075934	23	1	TCCTGTACTGAGCTGCCCCAAGT	miRNA	miR-486-5p
t0076010	25	1	TCCTGTAATGAGCTGCCCCGAGATA	miRNA	miR-486-5p
t0076045	26	1	TCCCGTACTGAGCTGCCCCGAGCAAG	miRNA	miR-486-5p
t0076172	22	1	TCTTGCACTGAGCTGCCCCGAG	miRNA	miR-486-5p
t0076181	23	1	TCCTGTACTGAGCTGCCCGGAGA	miRNA	miR-486-5p
t0076351	22	1	TACTGTACTGAGCTGCCGCGAG	miRNA	miR-486-5p
t0076394	22	1	TCCTGTACTGAGCTGCCCTGAA	miRNA	miR-486-5p
t0076497	22	1	TCCTGTCCTGAGCTGACCCGAG	miRNA	miR-486-5p
t0076741	23	1	TCCTGTACTGAGCTGCACAGAGT	miRNA	miR-486-5p
t0076952	23	1	TCTTGTACTGAGCTGCGCCGAGT	miRNA	miR-486-5p
t0077025	18	1	TCCTGTACTGAGCTGACC	miRNA	miR-486-5p
t0077098	23	1	TCCTGTACTGAGCTCCCCCGTGT	miRNA	miR-486-5p
t0077135	22	1	TCCTGTACTGGGCTGTCCCGAG	miRNA	miR-486-5p
t0077203	22	1	TCCTGTACTGAGTTGTCCCGAG	miRNA	miR-486-5p
t0077333	25	1	TCCTGTAATGAGCTGCCCCGAGGAA	miRNA	miR-486-5p
t0077441	23	1	TCCTGTATTGAGCTGCCCCGCGA	miRNA	miR-486-5p
t0077518	21	1	TCCTGTACTGAGCTGCGGCGA	miRNA	miR-486-5p
t0077586	20	1	CTGTACTGAGCTGCCCCGCG	miRNA	miR-486-5p
t0077623	23	1	TCCTGTACTGAGCTGGACCGAGA	miRNA	miR-486-5p
t0077898	23	1	TCCTGTACTGTGCTGCCCCGAGA	miRNA	miR-486-5p
t0077918	28	1	TCCTGTACTAAGCTGCCCCGAGACGGCA	miRNA	miR-486-5p
t0077925	21	1	TCCTGTACGGAGCTGTCCCGA	miRNA	miR-486-5p
t0077944	22	1	ACCTGTACTGAGCTGCCCTGAG	miRNA	miR-486-5p
t0078218	25	1	TCCTGTGCTGAGCTGCCCCGAGTCC	miRNA	miR-486-5p
t0078450	23	1	ACCTGTACTGAGCTGCCCCGAAA	miRNA	miR-486-5p
t0078697	26	1	TCCTGTACTGAGATGCCCCGAGCGCG	miRNA	miR-486-5p
t0078741	24	1	TCCAGTACTGAGCTGCCCCGAGAT	miRNA	miR-486-5p
t0078953	23	1	TCCTGTGCTGAGCTGCCCCGAGC	miRNA	miR-486-5p
t0079516	22	1	TCCTGTACTGAGCTGACCTGAG	miRNA	miR-486-5p
t0079755	29	1	TCCTGTACTGAGCTGCCCCGCGATGAGC[illegible]	miRNA	miR-486-5p
t0080053	26	1	TCCTGTACTGAGCTGCCCAGAGACGG	miRNA	miR-486-5p
t0080218	26	1	TCCTGTACTGAGCTGCCCCGAGGGCC	miRNA	miR-486-5p
t0080231	21	1	TCTTGTACTGCGCTGCCCCGA	miRNA	miR-486-5p
t0080427	23	1	TCCTGTACTGAGATGCCCCGAGT	miRNA	miR-486-5p

t0080740	22	1	TCCTGTACTGAGCTCCCCCGAC	miRNA	miR-486-5p
t0080747	21	1	TCCTGTACTGAACTGGCCCGA	miRNA	miR-486-5p
t0080795	26	1	TCCTGTACTGAGCTGCCCAGAGGTCC	miRNA	miR-486-5p
t0080855	21	1	TCCTGTACTGAGCTGAACCGA	miRNA	miR-486-5p
t0081414	23	1	TCCTATACTGAGCTGTCCCGAGA	miRNA	miR-486-5p
t0081502	20	1	CTGTACTGAGCTGCCCCGAT	miRNA	miR-486-5p
t0081755	21	1	TCCTGTACCGAGCTGCCTCGA	miRNA	miR-486-5p
t0081944	23	1	TCCTGTACTGAGCTGACCCGAGG	miRNA	miR-486-5p
t0081973	22	1	TCATGTACTGAGCTGCCCCGAT	miRNA	miR-486-5p
t0082092	23	1	TCTTGTACTGAGCTGCCCCGCGA	miRNA	miR-486-5p
t0082298	21	1	TCCTGTACTGAGCTGCCTCGC	miRNA	miR-486-5p
t0082513	28	1	TCCTGTACTGAGCTGCCCCGAGACGGAA	miRNA	miR-486-5p
t0082570	22	1	TACTGTACTGAGCTGCCCCGAT	miRNA	miR-486-5p
t0082602	23	1	TCCTCTACTGAGCTGCCCCGAGA	miRNA	miR-486-5p
t0082695	19	1	TCCTGTACTGAACTGCCCC	miRNA	miR-486-5p
t0082919	22	1	TCATGTACTGAGCTGACCCGAG	miRNA	miR-486-5p
t0082929	22	1	TCCTGTACTGAGCTGCCCCAAT	miRNA	miR-486-5p
t0082991	22	1	TCTTGTACTGAGCTGCCCCGAT	miRNA	miR-486-5p
t0083112	22	1	TCCTGTACTGAGCCGCCCCGCG	miRNA	miR-486-5p
t0083260	20	1	TCCTGTACTGAGTTGCCCCG	miRNA	miR-486-5p
t0083320	24	1	TCCTGTACTGAGCTGTCCCGAGTT	miRNA	miR-486-5p
t0083326	23	1	TCCTGTACTCAGCTGCCCCGAGT	miRNA	miR-486-5p
t0083841	21	1	TCGTGTACTGAGCTGCCTCGA	miRNA	miR-486-5p
t0083893	25	1	TCCTGTACTGAGATGCCCCGACGGT	miRNA	miR-486-5p
t0084085	19	1	TCCTGTACTGAGCTGCCCG	miRNA	miR-486-5p
t0084189	22	1	TCCTGTACTGAGCGGTCCCGAG	miRNA	miR-486-5p
t0084232	28	1	TCCTGTACTGAGCTGCCCCGAGACCGCC	miRNA	miR-486-5p
t0084493	22	1	TTCTGTACTGAGCTGCCCCCAG	miRNA	miR-486-5p
t0084772	21	1	TCCTGTACTGAGCTGCAACGA	miRNA	miR-486-5p
t0084838	21	1	TCATGTAATGAGCTGCCCCGA	miRNA	miR-486-5p
t0085238	26	1	TCCTGTACTGAGCTGCCCCGACATGG	miRNA	miR-486-5p
t0085389	20	1	TACTGTACTGAGCTGACCCG	miRNA	miR-486-5p
t0085492	23	1	TCCTGTACTGAGATGCACCGAGA	miRNA	miR-486-5p
t0085552	23	1	TACTGTACTGAGCTGCACCGAGA	miRNA	miR-486-5p
t0085941	26	1	TCCTATACTGAGCTGCCCCGAGATGG	miRNA	miR-486-5p
t0085993	22	1	TTCTGTACTGAGCTCCCCCGAG	miRNA	miR-486-5p
t0086360	22	1	TCCTGTACTGGGCTGCCCCGAA	miRNA	miR-486-5p
t0086442	24	1	TCCTGTACTGAGCTGCCCCGCCGT	miRNA	miR-486-5p
t0086650	25	1	TCCTGTACTGAGCTGCCCCCACGGT	miRNA	miR-486-5p
t0086723	24	1	TCCTGTACTGAGCTGCCCCGGGAA	miRNA	miR-486-5p
t0086779	22	1	TCTTGTAATGAGCTGCCCCGAG	miRNA	miR-486-5p
t0086896	19	1	TTGTACTGAGCTGCCACGA	miRNA	miR-486-5p
t0087026	21	1	TCCTGTGCTGAGCAGCCCCGA	miRNA	miR-486-5p
t0087037	19	1	TTGTACTGAGCTGCCCCGG	miRNA	miR-486-5p
t0087510	28	1	TCCTGTACTGAGCTGCCCCGAGCGGCTC	miRNA	miR-486-5p
t0087912	23	1	CTGTACTGAGCTGCCCCGAGAAG	miRNA	miR-486-5p
t0088004	27	1	TCTGTACTGAGCTGCCCCGAGACGGCA	miRNA	miR-486-5p
t0088359	21	1	TCCTGTACTGAGCTGTCACGA	miRNA	miR-486-5p
t0088436	19	1	CTGTACTGAGCTGCCCCGC	miRNA	miR-486-5p
t0088447	22	1	TCCTGTACTGAGCTGCCTCGAA	miRNA	miR-486-5p
t0088448	22	1	TCCTGTAATGAGCTGCCCCGAA	miRNA	miR-486-5p
t0088692	22	1	TCCTGTTCTGCGCTGCCCCGAG	miRNA	miR-486-5p
t0088817	22	1	TCTTGTACTAAGCTGCCCCGAG	miRNA	miR-486-5p
t0088847	22	1	TTCTGTACTGAGTTGCCCCGAG	miRNA	miR-486-5p
t0088902	23	1	TCCTGTACTGCGATGCCCCGAGA	miRNA	miR-486-5p
t0089075	22	1	TTCTATACTGAGCTGCCCCGAG	miRNA	miR-486-5p
t0089190	21	1	TCCTGTACTGAGCTGCTCAGA	miRNA	miR-486-5p
t0089247	21	1	TCCTGTACTGCGCTGCCCAGA	miRNA	miR-486-5p

t0089385	21	1	TCCTGTACTGAACTGCCCTGA	miRNA	miR-486-5p
t0089423	23	1	ACCTGTACTGAGCTGCCCCGAGT	miRNA	miR-486-5p
t0089431	20	1	TCATGTACTGAGCTGCCCAG	miRNA	miR-486-5p
t0089615	22	1	TCCTGTACTGAGGTGCACCGAG	miRNA	miR-486-5p
t0089641	22	1	TCCTGTACTGAGCTCCACCGAG	miRNA	miR-486-5p
t0089912	23	1	TCCTGTCCTGAGCTGCCCAGAGA	miRNA	miR-486-5p
t0089940	22	1	TCCTGTTCTGAGCTGCCCCGAT	miRNA	miR-486-5p
t0090292	21	1	TCTTGAACTGAGCTGCCCCGA	miRNA	miR-486-5p
t0090313	22	1	TCCTGTACTGAGCTGCTCCCAG	miRNA	miR-486-5p
t0090681	21	1	TCCTGTAATGAGCTGCCCCCA	miRNA	miR-486-5p
t0090710	23	1	TCTTGTACTGAGCTGACCCGAGA	miRNA	miR-486-5p
t0090825	21	1	CGGGGCAGCTCAGTACGGGAT	miRNA	miR-486-3p
t0090893	21	1	CGGGGCAGCTCAGTACCGGAT	miRNA	miR-486-3p
t0091195	21	1	CGGGGCAGCTCACTACAGGAT	miRNA	miR-486-3p
t0091249	21	1	CGGGGCAGCTAAGTACAGGAT	miRNA	miR-486-3p
t0091274	21	1	GGGGGCAGCTCAGTACAGGAT	miRNA	miR-486-3p
t0091310	20	1	GTAGGATTGTGTCGGCCGAT	miRNA	miR-4860
t0091651	23	1	TAGGATTGTGTCGGCAGATCTTT	miRNA	miR-4860
t0091749	20	1	AGGATTGTGTCGGGAGATCA	miRNA	miR-4860
t0091818	22	1	AGATGTAGGATTGTGTCGGCAT	miRNA	miR-4860
t0091929	25	1	ATGTAGGATTGTGTCGGGAGTTTTT	miRNA	miR-4860
t0092011	23	1	GTAGGATTGTGTCGGTAGATATT	miRNA	miR-4860
t0092336	21	1	TAGGATTGTGTCGGTAGGTCA	miRNA	miR-4860
t0092549	21	1	TAGAATTGTGTCGGCAGATCT	miRNA	miR-4860
t0068361	25	1	GGATTGTGTCGGCAGATCAGATTTT	miRNA	miR-4860
t0079342	22	1	TAAATGTAGAATTGTGTCGGTC	miRNA	miR-4860
t0085635	22	1	TAGAATTGTGTCGGCAGATCTC	miRNA	miR-4860
t0086681	23	1	TAGAATTGTGTCGGCAGATCATT	miRNA	miR-4860
t0088997	23	1	TAGATGTAGGATTGTGTCGGTTC	miRNA	miR-4860
t0053584	21	1	TGTAGGATTGTGTCGGTAGAT	miRNA	miR-4860
t0054380	23	1	GTAGAATTGTGTCGGCAGATTAT	miRNA	miR-4860
t0056057	24	1	GGATTGTGTCGGCAGATCAGTTTC	miRNA	miR-4860
t0058808	23	1	TAGATGTAGGATTGTGTCGGTAT	miRNA	miR-4860
t0060570	22	1	TGTAGGATTGTGTCGGTAGATT	miRNA	miR-4860
t0061214	21	1	TAAATGTAGAATTGTGTCGGT	miRNA	miR-4860
t0063399	20	1	TAGATGTAGGATTGTGTCGG	miRNA	miR-4860
t0064420	25	1	AGATGTAGGATTGTGTCGGTTTATT	miRNA	miR-4860
t0069221	23	1	TGTAGGATTGTGTCGGGAGATTT	miRNA	miR-4860
t0069795	24	1	TGTAGGATTGTGTCGGTAGACTTT	miRNA	miR-4860
t0070654	23	1	AGATGTAGGATTGTGTCGGTTTA	miRNA	miR-4860
t0071109	21	1	TCAGGCTCAGTCCCCTCCCGC	miRNA	miR-484
t0075421	22	1	TCAGGCTCAGTCCCCTCCCGTT	miRNA	miR-484
t0082354	20	1	AGGACAGGAAAGAGAAGGAG	miRNA	miR-483
t0083537	22	1	AGAGTCGGCGACGACGCCAGTT	miRNA	miR-4785
t0084555	22	1	AGAGTCGGCGACGCCGCCCGTT	miRNA	miR-4785
t0084897	21	1	TCGGGCGGAGGTGGTTGCGTG	miRNA	miR-4750
t0085361	20	1	CTCGGGCGGAGGGGGTTGAG	miRNA	miR-4750
t0088468	21	1	ACAGGCAAAGGGATATTTACA	miRNA	miR-4742-5p
t0089956	25	1	TGTAGAGCAGGGCGCAGGAAGCTGA	miRNA	miR-4732-5p
t0090629	22	1	AGCTGTAGAGCAGGGAGCAGGG	miRNA	miR-4732-5p
t0090700	21	1	TGTAGAGCAGGGAGCATGAAG	miRNA	miR-4732-5p
t0091806	23	1	TGTAGAGCAGGGAGCAGGAGGCT	miRNA	miR-4732-5p
t0092511	24	1	TGTAGAGCAGGCAGCAGGAAGCTG	miRNA	miR-4732-5p
t0074649	24	1	TGTAGAGCAGGGAGAAGGAAGCTG	miRNA	miR-4732-5p
t0078880	23	1	TGCAGAGCAGGGAGTAGGAAGCT	miRNA	miR-4732-5p
t0081966	22	1	TGTAGAGCAGGGCGCAGGAAGC	miRNA	miR-4732-5p
t0052806	24	1	TGTAGAGCAGGGAGCAGGAAGATG	miRNA	miR-4732-5p
t0055226	23	1	TGTAGAGCAGGGAGCAGGAAGGA	miRNA	miR-4732-5p

t0069505	23	1	TGTAAAGCAGGGAGCAGGAAGCA	miRNA	miR-4732-5p
t0082899	23	1	TGTAGATCAGGGAGCAGGAAGCT	miRNA	miR-4732-5p
t0086101	23	1	TGTAGAGCAGGGAGCAGGGAGCA	miRNA	miR-4732-5p
t0040924	23	1	TGTAGAGCAGGAAGTAGGAAGCT	miRNA	miR-4732-5p
t0041249	23	1	TGTAGAGCAGGGAGCAGCAAGCT	miRNA	miR-4732-5p
t0041608	22	1	TGTAGAGCAGGGAGCAGGAAAC	miRNA	miR-4732-5p
t0043065	23	1	TGTAGAGCAGGGCGTAGGAAGCT	miRNA	miR-4732-5p
t0043245	19	1	CGTAGAGCAGGGAGCAGGA	miRNA	miR-4732-5p
t0043908	21	1	TGTAGAGCAGTGAGCAGGAAG	miRNA	miR-4732-5p
t0044470	19	1	TGTAGAGCAGGGAGAAGGA	miRNA	miR-4732-5p
t0044507	20	1	TGTAGAGCGGGGAGCAGGAA	miRNA	miR-4732-5p
t0045547	19	1	TGTAGAGCAGGGCGCAGGA	miRNA	miR-4732-5p
t0046252	20	1	TGTAGAGCAGGGCGCAGGAA	miRNA	miR-4732-5p
t0046255	23	1	TGTAGAGCCGGGAGCAGGAAGCA	miRNA	miR-4732-5p
t0046258	23	1	TGTAGAGCACGGAGCAGGAAGCT	miRNA	miR-4732-5p
t0046374	22	1	TGTAGAGCAGGGAGCAGGCAGC	miRNA	miR-4732-5p
t0048789	23	1	TGTAGAGCAGGGAACAGGAAGCT	miRNA	miR-4732-5p
t0049894	25	1	TATAGAGCAGGGAGCAGGAAGCTGA	miRNA	miR-4732-5p
t0050024	23	1	TGTAGAGCAGGGAGCGGGAAGCT	miRNA	miR-4732-5p
t0050487	23	1	TGTAGAGTAGGGAGCAGGAAGTT	miRNA	miR-4732-5p
t0051079	22	1	CGTAGAGCAGGGAGCAGGAAGC	miRNA	miR-4732-5p
t0051137	24	1	TGTAGAGCAGGGAGCGGGAAGCTG	miRNA	miR-4732-5p
t0052015	24	1	TGTAGAGCAGGGGGCAGGAAGCTG	miRNA	miR-4732-5p
t0052919	19	1	TGTAAAGCAGGGAGCAGGA	miRNA	miR-4732-5p
t0052937	23	1	TGTAGAGCAGGGATCAGGAAGCT	miRNA	miR-4732-5p
t0053096	21	1	TGTAGAGCAGGGAGCAGGAAT	miRNA	miR-4732-5p
t0053666	25	1	CGTAGAGCAGGGAGCAGGAAGCTGT	miRNA	miR-4732-5p
t0054002	25	1	TGTAGAGCAGGGAGCAGGAGGCTGA	miRNA	miR-4732-5p
t0055016	22	1	TGTAGAGCAGGGAGCAGCAAGC	miRNA	miR-4732-5p
t0056222	23	1	TGTAGAGTAGGGAGCAGGAAGCT	miRNA	miR-4732-5p
t0057991	22	1	AGCTGCAGAGCAGGGAGCAGGA	miRNA	miR-4732-5p
t0059377	21	1	TGTAGAGCAGGGAGCAGGCAG	miRNA	miR-4732-5p
t0060526	23	1	TGTAGAGCAGGGAGCATGAAGCT	miRNA	miR-4732-5p
t0060817	23	1	TGTAGAGCAGGGAGTAGGAATCT	miRNA	miR-4732-5p
t0060949	22	1	TGTAGAGAAGGGAGCAGGAAGC	miRNA	miR-4732-5p
t0060976	23	1	TGTAGAGCAGGGAGCGGGAAGCA	miRNA	miR-4732-5p
t0061948	23	1	TATAGAGCAGGGAGCAGGAAGCA	miRNA	miR-4732-5p
t0063738	25	1	TGTAGAGCAGGGAGCAGGAAGATGT	miRNA	miR-4732-5p
t0064181	23	1	TGTAGAGCAGGGAGCAGGAATCA	miRNA	miR-4732-5p
t0065608	23	1	TGTAGAGCAGGGAGCAGGTAGCT	miRNA	miR-4732-5p
t0068553	21	1	TGTAGGGCAGGGAGCAGGAAG	miRNA	miR-4732-5p
t0069449	21	1	TGTAGAGCAGAGAGCAGGAAG	miRNA	miR-4732-5p
t0070013	21	1	TGTAGAGCATGGAGCAGGAAG	miRNA	miR-4732-5p
t0071779	20	1	TGTAGAGCCGGGAGCAGGAA	miRNA	miR-4732-5p
t0074080	23	1	TGTAGAGCAGGAAGCAGGAAGCT	miRNA	miR-4732-5p
t0074333	21	1	TGTAGAGCAGGGAACAGGAAG	miRNA	miR-4732-5p
t0076695	24	1	TGTAGAGCAGGGAGCAGGAAGCCG	miRNA	miR-4732-5p
t0076791	24	1	ATGTAGAGCAGGGAGCAAGAAGCA	miRNA	miR-4732-5p
t0077014	22	1	TGTAGAGCAGGGAGCAAGAAGC	miRNA	miR-4732-5p
t0077384	22	1	TGCAGAGCAGGGAGCAGGAAGC	miRNA	miR-4732-5p
t0078567	19	1	TGTAGGGCAGGGAGCAGGA	miRNA	miR-4732-5p
t0079723	21	1	TGTAGAGAAGGGAGCAGGAGG	miRNA	miR-4732-5p
t0080028	22	1	TGTAGAGCAGGGAGCGGGAAGA	miRNA	miR-4732-5p
t0080217	23	1	TGTAGAGCTGGGAGCAGGAAGCT	miRNA	miR-4732-5p
t0080375	23	1	TGTAGAGCAGGGAGAAGGAAGCA	miRNA	miR-4732-5p
t0082166	24	1	TGTAGAGCAGGGAGCAGGAAGCAG	miRNA	miR-4732-5p
t0082534	23	1	TGTAGAGCAGGGAGCAGGAACCT	miRNA	miR-4732-5p
t0082668	24	1	TGTAGAGCAGGGAGCAGGCAGCTG	miRNA	miR-4732-5p

t0086633	21	1	TGTAGAGCAGGGAGCCGGAAG	miRNA	miR-4732-5p
t0086655	21	1	TATAGAGCAGGGAGCAGGAAG	miRNA	miR-4732-5p
t0087166	20	1	TCTCCCTTTCTGCCCTGGCT	miRNA	miR-4685-3p
t0088319	20	1	TCTCCCTTCCTGCCCTGGGT	miRNA	miR-4685-3p
t0088713	20	1	TCTCCCTCCCTGCCCTGGCT	miRNA	miR-4685-3p
t0088859	22	1	ACTCCCTTCCTGCCCTGGCAAA	miRNA	miR-4685-3p
t0090021	20	1	TCTCCCTTCCTGCACTGGCT	miRNA	miR-4685-3p
t0090038	20	1	TCTCCCTTCCTGCCCTGGAT	miRNA	miR-4685-3p
t0090097	21	1	AAGCTATTGTTCAGACTTATG	miRNA	miR-4678
t0090761	20	1	AAGGTATTGTTCTGACTTAT	miRNA	miR-4678
t0090820	20	1	AAGGTATTGTTAAGACTTAT	miRNA	miR-4678
t0091033	21	1	AAGGAATTGTTCAGACTTATG	miRNA	miR-4678
t0092264	20	1	AAGGGATTGTTCAGACTTAT	miRNA	miR-4678
t0092676	21	1	AAGGTATTGTTCAGAATTATG	miRNA	miR-4678
t0092778	23	1	ATATACACACACACATACACAGA	miRNA	miR-466b-2*
t0053884	21	1	TCTTCTTAGACATGGAAACGT	miRNA	miR-4659a-3p
t0058430	21	1	TCTTCTTAGGCATGGCAACGT	miRNA	miR-4659a-3p
t0062111	29	1	AAGGGCTGGGTCGGTCGGGATGGGGCG(	miRNA	miR-4651
t0066171	29	1	AGCGTCGGTCGGGCTGGGGCGCGAAGC(	miRNA	miR-4651
t0071750	25	1	AAGGGCTGGGTCGGTCGGGCAAATC	miRNA	miR-4651
t0080617	30	1	AGGGCTGGGTCGGTCGGGCTGGGGAGC(	miRNA	miR-4651
t0046562	25	1	GGCTGGGTCGGTCGGGCTGGGGGGC	miRNA	miR-4651
t0059200	28	1	AGGGCTGTGTCGGTCGGGCTGGGGCGCG	miRNA	miR-4651
t0060659	30	1	AGGGCTGGGTCGGTCGGGCTGGGGCGCA	miRNA	miR-4651
t0075187	27	1	AGGGCTGGGTCGGTCGGGCTGGGGAGC	miRNA	miR-4651
t0082417	26	1	AGGGCTGGGTCGGTCGGGATGGGGCG	miRNA	miR-4651
t0089520	19	1	AAGGGCTGGGGCGGTCGGG	miRNA	miR-4651
t0078044	28	1	AGGGCTGGGTCGGTCGGGCTGGGGCGGT	miRNA	miR-4651
t0062752	26	1	GGGCTGGGTCGGTCGGGCTGGGGGGC	miRNA	miR-4651
t0068674	29	1	AGGGCTGGGTCGGTCGGGCTGGGGCGAT	miRNA	miR-4651
t0041304	22	1	AAGGGCTGGGTCTGTCGGGCTG	miRNA	miR-4651
t0041722	22	1	AGGGCTGGGTCGGTTGGGCTGG	miRNA	miR-4651
t0041844	23	1	TAAGGGCTGGGCCGGTCGGGCTG	miRNA	miR-4651
t0043594	20	1	TAAGGGCTGAGTCGGTCGGG	miRNA	miR-4651
t0044450	26	1	AGGGCTGGGTCGGACGGGCTGGGGCG	miRNA	miR-4651
t0045973	20	1	AGGGCTGGGGCGGTCGGGCT	miRNA	miR-4651
t0046106	20	1	CCTTGGGATGGGTCGATCGG	miRNA	miR-4651
t0047593	18	1	AGGGCTGGGGCGGTCGGG	miRNA	miR-4651
t0050966	19	1	AAGGGCTGGGTCGGGCGGG	miRNA	miR-4651
t0051189	28	1	AGGGCTGGGGCGGTCGGGCTGGGGCGC(	miRNA	miR-4651
t0052325	25	1	AGGCTGGGTAGGTCGGGCTGGGGCG	miRNA	miR-4651
t0055186	24	1	CTGGGTAGGTCGGGCTGGGGCGCG	miRNA	miR-4651
t0055246	25	1	GCTGGGTCGGTCGGGCTGGGGCGGT	miRNA	miR-4651
t0061161	28	1	AAGGGCTGGGTCGGTCGGGCTGGGGGG(	miRNA	miR-4651
t0061236	26	1	AGGGCGGGGTCGGTCGGGCTGGGGCG	miRNA	miR-4651
t0063765	28	1	AGGGCTGGGTCGGTCGGGCTGGGGAGC(	miRNA	miR-4651
t0064100	28	1	AGGGCTGGGACGGTCGGGCTGGGGCGAT	miRNA	miR-4651
t0064123	22	1	ACTGGGAAGAGGAGCTGTGGGA	miRNA	miR-4646-5p
t0064505	20	1	TGGGAAGAGGGGCTGAGGGA	miRNA	miR-4646-5p
t0065269	20	1	TGGGAAGAGGAGATGAGGGA	miRNA	miR-4646-5p
t0065392	22	1	ACTGGGAAGAGGCGCTGAGGGA	miRNA	miR-4646-5p
t0069697	22	1	ACTGGGAAGAGGGGCTGAGGGA	miRNA	miR-4646-5p
t0070890	22	1	ACTGGGAGGAGGAGCTGAGGGA	miRNA	miR-4646-5p
t0077152	21	1	TGGGAAGAGGAGCTGAGGGAC	miRNA	miR-4646-5p
t0081523	22	1	TAATAACTGCAGATTCAAGTGA	miRNA	miR-4637
t0082627	22	1	AAGCAGCACGTAAATATTGGTG	miRNA	miR-457b
t0085893	22	1	AAGCAGCACGTAAATACTGGCG	miRNA	miR-457b
t0087439	23	1	AAGCAGCACGTAAATATTGGCGT	miRNA	miR-457b

t0087442	21	1	AAGCAGCACGTAAATATTGGA	miRNA	miR-457b
t0093137	21	1	AAGCAGCACGTAAATATTGGC	miRNA	miR-457b
t0041326	22	1	AAGCAGCACGTAAATATTGGAG	miRNA	miR-457b
t0046191	23	1	TAGTGCAATATTGCTTATAGGGG	miRNA	miR-454b
t0063753	24	1	TAGTGCAATATTGCTTATAGGGGT	miRNA	miR-454b
t0064943	22	1	TAGCGCAATATTGCTTATAGGG	miRNA	miR-454
t0077633	23	1	CGACTCTGGATAGCTGGGCTTTT	miRNA	miR-4538
t0079470	24	1	TTCGACTCTGGATAGCTGGGTTTT	miRNA	miR-4538
t0089897	23	1	CGACTCTGGATAACTGGGCTTAT	miRNA	miR-4538
t0047313	23	1	CGACTCTGGATAGCTGGGCTTCT	miRNA	miR-4538
t0042609	23	1	TTCGACTCTGGATAACTGGGTTA	miRNA	miR-4538
t0058901	23	1	TCGACTCTGGATAACTGGGCTAC	miRNA	miR-4538
t0061026	23	1	TTCGACTCTGGATAGCTGGGTTT	miRNA	miR-4538
t0076182	21	1	TCGACTCTGGATAGCTGGGAT	miRNA	miR-4538
t0084889	23	1	TTTCGACTCTGGATAGCTGGTTT	miRNA	miR-4538
t0090250	23	1	CGACTCTGGATAACTGGGCTTTT	miRNA	miR-4538
t0053677	20	1	GACTCTGGATAGCTGGGCTA	miRNA	miR-4538
t0083163	21	1	ATAGCAAGAGAAACATTACCA	miRNA	miR-451b
t0065245	21	1	ATAGCAAGAGGACCATTACCA	miRNA	miR-451b
t0053493	25	1	CGGGGGGAGAAGGGACGGGGCGGCA	miRNA	miR-4516
t0054198	20	1	AGAAGGGTCGGGGCGGGTTT	miRNA	miR-4516
t0054309	20	1	AGAGAAGGGTCGGGGCGGCT	miRNA	miR-4516
t0056736	25	1	CGGGGGGAGGAGGGTCGGGGCGGGA	miRNA	miR-4516
t0057359	21	1	AGAAGGGTCGGGGCGGCAGGG	miRNA	miR-4516
t0068984	26	1	GGGAGAAGGGTCGGGGCGGCCGGGGC	miRNA	miR-4516
t0075333	25	1	GGAGAAGGGTCGGGGCGGCAGGGGC	miRNA	miR-4516
t0076056	20	1	GGAGAAGGGTTGGGGCGGCT	miRNA	miR-4516
t0082420	21	1	GAAGGGTCGGGGCGGCCGTTT	miRNA	miR-4516
t0089523	23	1	GGACAAGGGTCGGGGCGGCAGGG	miRNA	miR-4516
t0093446	21	1	GGGGGGAGAAGGGGCGGGGCG	miRNA	miR-4516
t0068767	22	1	GGGGGGCGAAGGGTCGGGGCGG	miRNA	miR-4516
t0080101	20	1	GAGACGGGTCGGGGCGGGCG	miRNA	miR-4516
t0041539	21	1	GAAGGGTCGGGGAGGCAGTTT	miRNA	miR-4516
t0042747	26	1	CGGGGGGAGAAGGGTCGGGGCGGAAG	miRNA	miR-4516
t0048445	24	1	AGAGAAGGGTCGGGGAGGCAGGGG	miRNA	miR-4516
t0052338	22	1	TGAGGGAGTAGGGTGTGTGGTC	miRNA	miR-4510
t0058082	24	1	TGAGGTAGTAGGGTGTGTGGTAAA	miRNA	miR-4510
t0061977	21	1	TGAGGGAGTAGGTTGTGTAGT	miRNA	miR-4510
t0064639	25	1	TGAGGGAGTAGGTTGTGTGGTTAGA	miRNA	miR-4510
t0070558	21	1	TGAGGGAGTCGGTTGTGTGGT	miRNA	miR-4510
t0071384	23	1	TGAGGGAGTAGGTTGTGGGGTAA	miRNA	miR-4510
t0072247	22	1	TGAGGGAGTAGGTTGTGTGGAG	miRNA	miR-4510
t0073842	21	1	TGAGGGAGTGGGTTGTGTGGT	miRNA	miR-4510
t0074966	22	1	TAAGGGAGTAGGTTGTGTGGTT	miRNA	miR-4510
t0077838	21	1	TGAGGGAGTAGGGTGTGTGTT	miRNA	miR-4510
t0083698	21	1	TGAGGGACTAGGTTGTGTGGT	miRNA	miR-4510
t0090635	24	1	TGAGGGAGTAGGTTGTGTGGTATT	miRNA	miR-4510
t0091404	22	1	TGAGGTAGTAGGGGGTGTGGTA	miRNA	miR-4510
t0043111	21	1	GAGGGAGTAGGTTGTGTGGTA	miRNA	miR-4510
t0043327	24	1	TGAGGGAGTAGGGTGTGTGGTTAA	miRNA	miR-4510
t0043629	22	1	TGAGGGAGTAGGTTGTGTGGAA	miRNA	miR-4510
t0045172	21	1	TGAGGGATTAGGTTGTGTGGT	miRNA	miR-4510
t0045366	21	1	TGAGGGAGTAGGTTGTGCGGT	miRNA	miR-4510
t0045509	24	1	TGAGGGAGTAGGTTGTGTGGTTTT	miRNA	miR-4510
t0045541	24	1	TGAGGTAGTAGGGTGTGTGGTATC	miRNA	miR-4510
t0048257	24	1	TGAGGGAGTAGGGTGTGTGGTTAT	miRNA	miR-4510
t0048437	21	1	GAGGGAGTAGGTTGTGTGGTT	miRNA	miR-4510
t0049930	22	1	TGAGGGACTAGGTTGTGTGGTT	miRNA	miR-4510

t0050968	23	1	TGAGGTAGTAGGGTGTGTGGTAG	miRNA	miR-4510
t0051375	22	1	TGAGGGAGTATGTTGTGTGGTT	miRNA	miR-4510
t0053358	20	1	TGAGGGAGTAGGTTGTGGGG	miRNA	miR-4510
t0053555	21	1	TTGAGGGAGTAGGTTGTGTGG	miRNA	miR-4510
t0054185	20	1	TGAGGGAGTAGGTTATGTGG	miRNA	miR-4510
t0055611	18	1	TGAGGGAGTAGGTTGTGG	miRNA	miR-4510
t0055892	23	1	TGAGGGAGTAGGTTGTGTGGTGA	miRNA	miR-4510
t0057369	22	1	TGAGGGAGTAGGTTGTCTGGTT	miRNA	miR-4510
t0059107	22	1	TGAGGGAGTAGGTTGTGTGGTG	miRNA	miR-4510
t0059453	20	1	TGAGGGAGTAGGTTGTGTAG	miRNA	miR-4510
t0062156	20	1	TGAGGGAGTAGGGTGTGTAG	miRNA	miR-4510
t0063500	22	1	TGAGGGAGTAGCTTGTGTGGTT	miRNA	miR-4510
t0063670	21	1	TGAGGGAGTAGGTTGTGTTGT	miRNA	miR-4510
t0064004	22	1	TGAGGGAGTAGGTTGCGTGGTT	miRNA	miR-4510
t0066155	21	1	TGAGGGAGTAGGTTGTCTGGT	miRNA	miR-4510
t0066257	22	1	TGAGGGAGTAGGTTGTGTCGTT	miRNA	miR-4510
t0066674	22	1	TGAGGGAGTAGGTTGGGTGGTT	miRNA	miR-4510
t0067017	22	1	TGAGGGAGTAGGGTGTGGGGTT	miRNA	miR-4510
t0067692	22	1	TGAGGGAGTAGGTTGTGTGGGA	miRNA	miR-4510
t0067795	22	1	TGCGGGAGTAGGTTGTGTGGTT	miRNA	miR-4510
t0067940	22	1	TGAGGGAGTAGTTTGTGTGGTT	miRNA	miR-4510
t0070257	22	1	TCAGGGAGTAGGTTGTGTGGTA	miRNA	miR-4510
t0071303	21	1	TGAGGGAGTAGGTCGTGTGGT	miRNA	miR-4510
t0075996	21	1	TGAGGGAGTAGGCTGTGTGGT	miRNA	miR-4510
t0076586	22	1	TGAGGGAGTAGGGTGTATGGTT	miRNA	miR-4510
t0079226	21	1	TGAGGGAGTAGTTTGTGTGGT	miRNA	miR-4510
t0079922	20	1	TGAGGGAGTAGTTTGTGTGG	miRNA	miR-4510
t0080797	26	1	TGAGGGAGTAGGTTGTGTGGTTTATC	miRNA	miR-4510
t0082280	25	1	AAACCGCTACCATTACTGAGTTTAG	miRNA	miR-451
t0086526	22	1	AAACCTTTACCATTACCGAGTT	miRNA	miR-451
t0087030	22	1	AAACCGCTACTATTACTGAGTT	miRNA	miR-451
t0087227	23	1	AAACCGTTACAATTACTGAGTAA	miRNA	miR-451
t0089206	21	1	AAACCGTTACCATTAGGGAGT	miRNA	miR-451
t0089998	24	1	AAACCGTTACCACTACTGAGTTTG	miRNA	miR-451
t0090019	25	1	AAACCGTTAACATTACTGAGTTTAA	miRNA	miR-451
t0090609	22	1	AAACCCTTAACATTACTGAGTT	miRNA	miR-451
t0091264	21	1	AAAACGTTACCATTAATGAGT	miRNA	miR-451
t0091807	19	1	AACCCGTTACCATTACTGA	miRNA	miR-451
t0092215	20	1	AAACCCTTACCATTATTGAG	miRNA	miR-451
t0092675	25	1	AAACCGTTACCATTACTGAGTTTAC	miRNA	miR-451
t0092879	22	1	AAACCATTACCATTACTCAGTT	miRNA	miR-451
t0040762	23	1	AAACCGTTGCCATTACTGAGTTA	miRNA	miR-451
t0041159	24	1	AAACCGTTACCATTACTGCGTTTG	miRNA	miR-451
t0041292	22	1	AAACCGTTACCATTATTGGGTT	miRNA	miR-451
t0041484	22	1	AAACTGTTAACATTACTGAGTT	miRNA	miR-451
t0041568	23	1	AAAACTGTTACCATTACTGAGTT	miRNA	miR-451
t0041625	23	1	AAACCGTTACCATTACTGAGTAT	miRNA	miR-451
t0041934	22	1	AAACCGTTGCCATCACTGAGTT	miRNA	miR-451
t0042082	21	1	AAACAGTTACCATTACTCAGT	miRNA	miR-451
t0042098	23	1	AAACCGTTACCATTACCCAGTTT	miRNA	miR-451
t0042211	22	1	AAACCGTTACCATTACTTAGTA	miRNA	miR-451
t0042364	26	1	AAACCGTTACCATTACTGAGATTAGT	miRNA	miR-451
t0042422	20	1	AAACCATTACCATTACTGAC	miRNA	miR-451
t0042721	22	1	AAACCTCTACCATTACTGAGTT	miRNA	miR-451
t0042748	19	1	AAACCATTACCGTTACTGA	miRNA	miR-451
t0042865	20	1	AAACCGTTACCATTATTGAA	miRNA	miR-451
t0042979	23	1	AAAACGTCACCATTACTGAGTTT	miRNA	miR-451
t0043504	23	1	AAATCATTACCATTACTGAGTTT	miRNA	miR-451

t0043557	26	1	GATAAACAGTTACCATTACTGAGTTT	miRNA	miR-451
t0043611	23	1	AAACCTTTACCATTACTGAGCTT	miRNA	miR-451
t0043671	22	1	AAACCGTTACCATTATTGAGCT	miRNA	miR-451
t0043745	23	1	AAACCGTTACCCTTACTGAGTTG	miRNA	miR-451
t0043953	22	1	AAACCGTTACCATTACTCCGTT	miRNA	miR-451
t0044131	20	1	AAACCGTTACCATTATTGGG	miRNA	miR-451
t0044213	19	1	AAACCGTTACCACTACTGA	miRNA	miR-451
t0044262	22	1	AAACCGTTACCATTACTAAGGT	miRNA	miR-451
t0044472	20	1	AAACAGTTATCATTACTGAG	miRNA	miR-451
t0044518	21	1	AAACAGTTACCGTTACTGAGT	miRNA	miR-451
t0044542	21	1	AAACCGTTAGCATTATTGAGT	miRNA	miR-451
t0045113	22	1	AAACCGTTACCATTATTAAGTT	miRNA	miR-451
t0045145	21	1	AAACCGTTAACAGTACTGAGT	miRNA	miR-451
t0045185	21	1	AAACCTTTACCATCACTGAGT	miRNA	miR-451
t0045244	20	1	AAACCGTTAAGATTACTGAG	miRNA	miR-451
t0045297	21	1	AAACCGTTACCATTAATGAGA	miRNA	miR-451
t0045371	23	1	AAACCGTTACCATTACTGGGTTA	miRNA	miR-451
t0045493	22	1	AAACCGTGACCATTACTGAGGT	miRNA	miR-451
t0045867	23	1	AAACCGTTACCATTATTGCGTTT	miRNA	miR-451
t0045877	21	1	AAACCGGTACGATTACTGAGT	miRNA	miR-451
t0046107	24	1	AAACCGTTACCATTAATGAGTAAA	miRNA	miR-451
t0046213	21	1	AAACCGCTACCATTACTCAGT	miRNA	miR-451
t0046339	25	1	AAACCGTTACCATTACTGAGTTGAG	miRNA	miR-451
t0046348	23	1	AAACTGTTACCATTACTGAGTAA	miRNA	miR-451
t0046602	23	1	AAACAGTCACCATTACTGAGTTT	miRNA	miR-451
t0046667	21	1	AAACCGTTACAATTACTGCGT	miRNA	miR-451
t0046841	22	1	GAACCGTTACCATTACTGAGTA	miRNA	miR-451
t0046884	21	1	AAACTGTTACCATTACTCAGT	miRNA	miR-451
t0046904	22	1	AAACCGTCACCACTACTGAGTT	miRNA	miR-451
t0046932	21	1	AAACCGGTACCATTATTGAGT	miRNA	miR-451
t0046933	22	1	AAACCGCTACCATTACCGAGTT	miRNA	miR-451
t0046959	21	1	AAACCGTTACCATTAAGGAGT	miRNA	miR-451
t0047094	18	1	CGTTACCATTACTGAGTT	miRNA	miR-451
t0047101	22	1	CGTTACCATTACTGGGTTAAAC	miRNA	miR-451
t0047237	24	1	AAACCGTCACCATTACTGAGTTAG	miRNA	miR-451
t0047428	21	1	AAACCGTTACCATTATTGCGT	miRNA	miR-451
t0047463	24	1	AAACCGATACCATTACTGAGTTTA	miRNA	miR-451
t0047518	23	1	AAACCGTTACCATTACTGAGACA	miRNA	miR-451
t0047600	22	1	AAACCGTTATCGTTACTGAGTT	miRNA	miR-451
t0047683	27	1	AAACCGTTACCATTACTGAGTTCAAAA	miRNA	miR-451
t0047705	22	1	AAAAAGTTACCATTACTGAGTT	miRNA	miR-451
t0047861	21	1	AAACCTTTACCATTACTGAAT	miRNA	miR-451
t0048260	23	1	AAACCGTACCCATTACTGAGTTT	miRNA	miR-451
t0048562	20	1	AAACCGTGACCATTACTGAG	miRNA	miR-451
t0048565	19	1	CAACCGTTACCATTACTGA	miRNA	miR-451
t0048590	23	1	ACACCGTTAACATTACTGAGTTT	miRNA	miR-451
t0048638	25	1	AAACCGTTAACATTAATGAGTTTAG	miRNA	miR-451
t0048826	21	1	AAACCGTTACCATTACTTAGC	miRNA	miR-451
t0049161	21	1	AACCGTTACCATTACTGCGTT	miRNA	miR-451
t0049166	21	1	AAACCGTTACTATTACTTAGT	miRNA	miR-451
t0049203	25	1	AAACCGTTACCAATACTGAGTTTGG	miRNA	miR-451
t0049239	21	1	AAATCCTTACCATTACTGAGT	miRNA	miR-451
t0049310	22	1	AAACCGTTAACCTTACTGAGTT	miRNA	miR-451
t0049373	21	1	AAACCCTTACCATTACTGAGG	miRNA	miR-451
t0049727	25	1	AGCAAACCGTTACCATTACTGAGTT	miRNA	miR-451
t0049798	21	1	CGTTACAATTACTGAGTAAAC	miRNA	miR-451
t0049957	24	1	AAACCGCTACCATTACTGAGTTTA	miRNA	miR-451
t0050164	23	1	AAACCGTTACCATTAATGAGGTT	miRNA	miR-451

t0050203	23	1	AAACCGTTACCATTACCGAGTTG	miRNA	miR-451
t0050510	26	1	AAACCGTTACCATTACGGAGTTTAGT	miRNA	miR-451
t0050856	23	1	AAACCGTTATCATTACTGAGTCT	miRNA	miR-451
t0051053	21	1	AAACCGTTACCATTAATGCGT	miRNA	miR-451
t0051221	25	1	AAACCGTTACCGTTACTGAGTTTAG	miRNA	miR-451
t0051516	22	1	AAACCTTCACCATTACTGAGTT	miRNA	miR-451
t0051599	25	1	CAACCGTTACCATTACTGAGTTTAG	miRNA	miR-451
t0051657	23	1	AAACCGTCATCATTACTGAGTTT	miRNA	miR-451
t0051704	22	1	AATCCGTTACCATTACTAAGTT	miRNA	miR-451
t0051759	29	1	AAACCGCTACCATTACTGAGTTCGTCGAT	miRNA	miR-451
t0051761	23	1	AAACCGTTACCATTACTGAGATA	miRNA	miR-451
t0051804	21	1	AAACAGTTACCATTACTGAGG	miRNA	miR-451
t0051853	26	1	AAACCGTTACCATTACTGAGTTCAGT	miRNA	miR-451
t0051956	23	1	AAACCGTTATCATTAATGAGTTT	miRNA	miR-451
t0051982	23	1	AAACCTTTACCATTACTGAGATT	miRNA	miR-451
t0052122	25	1	AAACCATTACCATTACTGAGTTACA	miRNA	miR-451
t0052391	22	1	AAACCGTTACCATTATTGAGTG	miRNA	miR-451
t0052534	22	1	AGACCTTTACCATTACTGAGTT	miRNA	miR-451
t0052853	18	1	AAACTGTTACCATTACTG	miRNA	miR-451
t0053045	21	1	AAAACGTTACCATTACTGCGT	miRNA	miR-451
t0053176	22	1	AAACCGTTACCGTTACTCAGTT	miRNA	miR-451
t0053184	22	1	AAACCGTTACCATTAATGAGAT	miRNA	miR-451
t0053626	23	1	AAACCTTTACCATTACCGAGTTT	miRNA	miR-451
t0053748	22	1	AAAACCTTACCATTACTGAGTT	miRNA	miR-451
t0053807	21	1	AAACTGTTACCATTATTGAGT	miRNA	miR-451
t0054053	22	1	AAACCGTTACGATTACTGAGGT	miRNA	miR-451
t0054101	20	1	AGACCGTTACCATTATTGAG	miRNA	miR-451
t0054236	21	1	AACCGTTACCATTACTGAGAT	miRNA	miR-451
t0054387	22	1	AAACCGTTAACTTTACTGAGTT	miRNA	miR-451
t0054515	20	1	AAACCGTTACCATTACGTAG	miRNA	miR-451
t0054565	22	1	AAACCGTTACCCTTACTGAGTA	miRNA	miR-451
t0054757	22	1	AAACCGTTACCATTACTGCGGT	miRNA	miR-451
t0054799	26	1	AAACCGTTACCATTATTGAGTTTAGT	miRNA	miR-451
t0054898	23	1	AAACCCTTACCCTTACTGAGTTT	miRNA	miR-451
t0054956	18	1	AAACAGTTACCATTACTG	miRNA	miR-451
t0054972	22	1	AAACCGTTACCCTTACTGCGTT	miRNA	miR-451
t0055312	21	1	AAACCTTTATCATTACTGAGT	miRNA	miR-451
t0055336	22	1	AAACCGTTACCACTATTGAGTT	miRNA	miR-451
t0055504	19	1	AAACCGTTACCATTACTCG	miRNA	miR-451
t0055640	23	1	AAACCGTTACCATTACTGGTTTT	miRNA	miR-451
t0055674	21	1	AAACCTTTACCATTACTGGGT	miRNA	miR-451
t0055931	22	1	AAACCGTTACTATTACTCAGTT	miRNA	miR-451
t0055990	22	1	AAACCGTTACAATTACTGAGCT	miRNA	miR-451
t0056006	23	1	AAACCTTTACCATTACTGGGTTT	miRNA	miR-451
t0056228	22	1	AAACCGTTACACTTACTGAGTT	miRNA	miR-451
t0056377	27	1	AAACCGTTACCATTACTGAGTCGTAGG	miRNA	miR-451
t0056951	26	1	AAAACGTTACCATTACTGAGTTTAGT	miRNA	miR-451
t0056988	20	1	AAACCGTTACCATTATTAAG	miRNA	miR-451
t0057059	23	1	AAACCTTTACCGTTACTGAGTTT	miRNA	miR-451
t0057258	23	1	AAACCGTTACCATTAATGAGTAA	miRNA	miR-451
t0057357	22	1	TACCTTTACCATTACTGAGTTT	miRNA	miR-451
t0057573	23	1	AAACAGTTAACATTACTGAGTTA	miRNA	miR-451
t0057886	25	1	AAACCGTTACAATTACTGAGTTTAG	miRNA	miR-451
t0057974	20	1	AAACCGTTAACATTACTGAA	miRNA	miR-451
t0058196	19	1	AAACCGTTACCGTTAATGA	miRNA	miR-451
t0058313	21	1	AAACCGTTACCATTAGTGAGG	miRNA	miR-451
t0058467	22	1	AAACCTTTTCCATTACTGAGTT	miRNA	miR-451
t0058578	25	1	AAACCGTTACCATTACTTAGTTTAG	miRNA	miR-451

t0058663	18	1	AAACCGTTACCATTAATG	miRNA	miR-451
t0058769	22	1	AAACGGTTACCATTGCTGAGTT	miRNA	miR-451
t0058825	21	1	AAACCGTTACCGTTACCGAGT	miRNA	miR-451
t0058841	20	1	AAACCGTTACCATTACGGGG	miRNA	miR-451
t0058953	25	1	AACCAGTTACCATTACTGAGTTTAG	miRNA	miR-451
t0059458	20	1	AACCGTTACCATTATTGAGT	miRNA	miR-451
t0059461	21	1	AAACTGTTACCATTACTGAGA	miRNA	miR-451
t0059682	23	1	AAACCGTTACCATTACTGCGGTT	miRNA	miR-451
t0059705	23	1	AAACCGTTGCCATTACTGAGGTT	miRNA	miR-451
t0059842	22	1	AAACCGTTACCGTTACTGGGTT	miRNA	miR-451
t0060460	22	1	AAACCGTTACCATTATTTAGTT	miRNA	miR-451
t0060635	20	1	AAAAAGTTACCATTACTGAG	miRNA	miR-451
t0060751	23	1	AAACCTTTACCATTACTAAGTTT	miRNA	miR-451
t0060945	23	1	GAACCGTTACCATTACTGAGTTA	miRNA	miR-451
t0061048	21	1	AAACCGTTAACATTACTTAGT	miRNA	miR-451
t0061099	22	1	AAACCGTTACCATTGCTGCGTT	miRNA	miR-451
t0061125	22	1	AAACAGTTATCATTACTGAGTT	miRNA	miR-451
t0061428	19	1	AAACCGTTACCAATACTGA	miRNA	miR-451
t0061499	20	1	AAACAGTTACCATTACTTAG	miRNA	miR-451
t0061676	26	1	AAACCGTTACCATCACTGAGTTTAGT	miRNA	miR-451
t0061837	21	1	AAACCGTTAACATTACTGAGA	miRNA	miR-451
t0061883	21	1	AAGCCTTTACCATTACTGAGT	miRNA	miR-451
t0061993	20	1	AAACCGTTACCATTACTAGG	miRNA	miR-451
t0062165	22	1	AAACAGTTACCATTACTGAGTA	miRNA	miR-451
t0062419	23	1	AAACCGTTACCATTACTGAGTAG	miRNA	miR-451
t0062505	19	1	AAACCGTTACCTTTACTGA	miRNA	miR-451
t0062575	23	1	AGACCCTTACCATTACTGAGTTT	miRNA	miR-451
t0062902	21	1	AAACAGTTACCATTACTAAGT	miRNA	miR-451
t0063221	23	1	AAACCGATACCATTACTGAGTAA	miRNA	miR-451
t0063225	23	1	GAACCTTTACCATTACTGAGTTT	miRNA	miR-451
t0063361	22	1	AAACCGTTACCATTATTGCGTT	miRNA	miR-451
t0063499	24	1	AAACCGTTACCATTACTAAGTTTA	miRNA	miR-451
t0063558	23	1	AAACAGTTACCATTACTGAGTAA	miRNA	miR-451
t0063576	23	1	AAACCTTTACCATTACTGACTTT	miRNA	miR-451
t0063613	21	1	AAACCGTTACCATTATTGGGT	miRNA	miR-451
t0063665	27	1	AAACCGTTACCATTACTGAGTTTAGAA	miRNA	miR-451
t0063936	23	1	AAACCGTCACCATTACTGAGTTC	miRNA	miR-451
t0063951	21	1	AAACCGTTACCATTACCGGGT	miRNA	miR-451
t0063991	19	1	AAACGGTTACCATTACTGA	miRNA	miR-451
t0064285	23	1	TAACCGTTACCATTACTGAGTTT	miRNA	miR-451
t0064463	22	1	AAACCGTCACCATTCCTGAGTT	miRNA	miR-451
t0064578	23	1	AAACTGTTACCATTATTGAGTTT	miRNA	miR-451
t0064616	22	1	AAACCGTTACCATTACTGGGAT	miRNA	miR-451
t0064647	20	1	AAATCGTCACCATTACTGAG	miRNA	miR-451
t0065029	22	1	AAACCGTTACCATTACCGGGTT	miRNA	miR-451
t0065178	22	1	AAACGTTACCATTACTGCGTTT	miRNA	miR-451
t0065237	24	1	AAACCTTTACTATTACTGAGTTTA	miRNA	miR-451
t0065645	24	1	AAAACCGTTACCATTGCTGAGTTA	miRNA	miR-451
t0065671	23	1	AAACCGTTACCATTACGGAGTTT	miRNA	miR-451
t0065736	20	1	AAAACTTTACCATTACTGAG	miRNA	miR-451
t0065797	19	1	AAACAGTTACCGTTACTGA	miRNA	miR-451
t0065882	21	1	AAACACTTACCATTACTGAGT	miRNA	miR-451
t0066060	20	1	AAACCGTTACAATTACTGCG	miRNA	miR-451
t0066192	19	1	ATACCGTTACCATTACTGA	miRNA	miR-451
t0066311	26	1	AAATCGTTACCATTACTGAGTTTAGT	miRNA	miR-451
t0066546	21	1	CAACCGTTACCATTACTGAAT	miRNA	miR-451
t0067095	25	1	CCAAAACCGTTACCATTACTGAGTT	miRNA	miR-451
t0067102	22	1	AAACCGTTACCATTATTGAGGT	miRNA	miR-451

t0067310	21	1	AACCGTTACCATTACTGAGGA	miRNA	miR-451
t0067892	20	1	AAACAGTTACCATTACTGAC	miRNA	miR-451
t0068354	23	1	AAACCGTTACAATTAATGAGTTT	miRNA	miR-451
t0068457	22	1	AAACCTTTACCATTACTGGGTT	miRNA	miR-451
t0068461	22	1	AACAGTTACCATTACTGAGTTT	miRNA	miR-451
t0068823	23	1	AAACCGTTACCATTAATGCGTTT	miRNA	miR-451
t0068829	22	1	AAACCGTCACCATTAATGAGTT	miRNA	miR-451
t0068911	26	1	AAACTGTTACCATTACTGAGTTTAGT	miRNA	miR-451
t0068926	19	1	AAACCGTTACCATTACAGA	miRNA	miR-451
t0068982	22	1	AAACCGTCACCATTACTTAGTT	miRNA	miR-451
t0069212	22	1	AAAGCTTTACCATTACTGAGTT	miRNA	miR-451
t0069355	26	1	AAACCTTTAACATTACTGAGTTTAGT	miRNA	miR-451
t0069673	21	1	AAACCGTTACTATTACTGAGG	miRNA	miR-451
t0069807	26	1	AAAACGTTACAATTACTGAGTTTAGT	miRNA	miR-451
t0069850	21	1	AAACCATTGCCATTACTGAGT	miRNA	miR-451
t0069892	21	1	ATCGTTACCATTATTGAGTTT	miRNA	miR-451
t0069906	25	1	AAACCGTTCCCATTACTGAGTTTAG	miRNA	miR-451
t0070070	20	1	AACCCTTTACCATTACTGAG	miRNA	miR-451
t0070118	21	1	AAACCTTTACCATTACTGAGC	miRNA	miR-451
t0070158	22	1	AAACTGTTACTATTACTGAGTT	miRNA	miR-451
t0070322	26	1	AAACCGTTACCATTGCTGAGTTTAGA	miRNA	miR-451
t0070585	21	1	AAACCGTTACCATTGTTGAGT	miRNA	miR-451
t0070648	20	1	AAACAGATACCATTACTGAG	miRNA	miR-451
t0071027	22	1	AAACCTTTACCATTAATGAGTT	miRNA	miR-451
t0071234	23	1	AAACCGTTACCATTACTGAGGTA	miRNA	miR-451
t0071556	21	1	AAATCTTTACCATTACTGAGT	miRNA	miR-451
t0071595	22	1	AAACCGTTACCATTAGTGAGCT	miRNA	miR-451
t0071627	22	1	AAACCGTTACCATTAATGAGCT	miRNA	miR-451
t0071650	24	1	AAACCGTTACCATTACTGAGTATA	miRNA	miR-451
t0071684	23	1	AAACCGTTATCATTACTAAGTTT	miRNA	miR-451
t0072094	22	1	AAACCGTTACAATTACTGAGTA	miRNA	miR-451
t0072122	22	1	AAACTGTTACCATTACTGACTT	miRNA	miR-451
t0072341	21	1	AAACCGCTACCATTAGTGAGT	miRNA	miR-451
t0072356	21	1	AAACCTTTACCAATACTGAGT	miRNA	miR-451
t0072456	22	1	AAACCGCTACCCTTACTGAGTT	miRNA	miR-451
t0072695	22	1	AAACCATTACCATTATTGAGTT	miRNA	miR-451
t0072864	23	1	AAACAGTTACCATTACTGAGTTA	miRNA	miR-451
t0073061	21	1	GAACTGTTACCATTACTGAGT	miRNA	miR-451
t0073473	21	1	AAACCGCTACCATTATTGAGT	miRNA	miR-451
t0073559	22	1	AAACCGTTACCATTACTGCGCT	miRNA	miR-451
t0073577	25	1	AAACCGCTACCATTACTGAGTTTAC	miRNA	miR-451
t0073608	22	1	AAACCGTTACCATTAATGCGTT	miRNA	miR-451
t0073717	26	1	AAACCGTTACCATTACTGAGCTTAGT	miRNA	miR-451
t0073791	27	1	AAACCGTTACCATTACTGCGTCGTCGG	miRNA	miR-451
t0073836	20	1	AAACCTTTAACATTACTGAG	miRNA	miR-451
t0074025	20	1	AAACCTTTACAATTACTGAG	miRNA	miR-451
t0074091	23	1	AAAACGTTAACATTACTGAGTTT	miRNA	miR-451
t0074109	21	1	AAACTGTTACCATTACCGAGT	miRNA	miR-451
t0074309	19	1	AAACCGTTAACGTTACTGA	miRNA	miR-451
t0074424	23	1	AAACATTTACCATTACTGAGTTT	miRNA	miR-451
t0074425	23	1	AAACCTTTACCATTACTGTGTTT	miRNA	miR-451
t0074478	20	1	GAACCGTTACCATTATTGAG	miRNA	miR-451
t0074548	22	1	GAACCGTTAACATTACTGAGTT	miRNA	miR-451
t0074586	18	1	TAACCGTTACCATTACTG	miRNA	miR-451
t0074589	21	1	AAACCTTTACCATTACTAAGT	miRNA	miR-451
t0074701	24	1	AAACCGTTACCATTACTGAGTCAA	miRNA	miR-451
t0074718	20	1	AAACCGTTAAAATTACTGAG	miRNA	miR-451
t0074749	22	1	AAACCTTTACCAATACTGAGTT	miRNA	miR-451

t0075051	21	1	AAACCGTTACTATTATTGAGT	miRNA	miR-451
t0075328	24	1	AAACCGTTACCAATACTGAGTTTA	miRNA	miR-451
t0075383	24	1	CAGAAACCGTTACCATTACTGCGT	miRNA	miR-451
t0075561	22	1	AAACAGTTACCATTACTGAATT	miRNA	miR-451
t0075655	26	1	AAACCGTTACCCTTACTGCGTTTAGT	miRNA	miR-451
t0075847	22	1	AAACCGTTAACATTAATGAGTT	miRNA	miR-451
t0075994	19	1	AAACCGTTAACATTAATGA	miRNA	miR-451
t0076177	22	1	AAACCTTTATCATTACTGAGTT	miRNA	miR-451
t0076426	22	1	AAACTGTCACCATTACTGAGTT	miRNA	miR-451
t0076509	22	1	AAACTGTTACCATTGCTGAGTT	miRNA	miR-451
t0076517	22	1	AAACCGCTACCATTACTGGGTT	miRNA	miR-451
t0076774	23	1	AAACCGTTTCCATTTCTGAGTTT	miRNA	miR-451
t0076887	21	1	AAACAGTTACCATTCCTGAGT	miRNA	miR-451
t0076982	23	1	AAACCGTCAACATTACTGAGTTT	miRNA	miR-451
t0076996	20	1	AAACCGTTAACATTATTGAG	miRNA	miR-451
t0077254	23	1	AAACCGTTACCCTTACTGAGTAA	miRNA	miR-451
t0077313	22	1	AAACCGTTAGCATTATTGAGTT	miRNA	miR-451
t0077318	23	1	AAACAGTTACAATTACTGAGTTT	miRNA	miR-451
t0077391	27	1	AAACCGTTACCATTACTGAGTCCGGGG	miRNA	miR-451
t0077402	21	1	AAAGTTACCATTACTGAGTTT	miRNA	miR-451
t0077451	22	1	AAACCGTCACCATTACTGCGTT	miRNA	miR-451
t0077927	22	1	AAACCGTTATCATTATTGAGTT	miRNA	miR-451
t0078294	21	1	AAAGCGTTAACATTACTGAGT	miRNA	miR-451
t0078820	29	1	AAACCGTTACCATTACTGAGTTGGGGGAC	miRNA	miR-451
t0078938	20	1	AAACCGTTACCACTACCGAG	miRNA	miR-451
t0079152	26	1	AAACCGTTGCCATTACTGAGTTTAGT	miRNA	miR-451
t0079259	29	1	AAACCGTTACCATTACTGAGTTCGTCGCT	miRNA	miR-451
t0079399	23	1	AAACCGTTACCATTACTGACTAA	miRNA	miR-451
t0079430	22	1	AAATCGTTACAATTACTGAGTT	miRNA	miR-451
t0079453	30	1	AAACCGTTACCATTACTGAGGTCGGCACC	miRNA	miR-451
t0079643	20	1	AAACCTTTACCATTACTGCG	miRNA	miR-451
t0079885	25	1	AAACCGTTACCATTACTGAGTTTGG	miRNA	miR-451
t0079938	21	1	AAACCGTTACCATTAGTGGGT	miRNA	miR-451
t0079968	22	1	AAAACGTTACCATTAATGAGTT	miRNA	miR-451
t0080118	23	1	AAACCGTTAACATTACTGAGTGT	miRNA	miR-451
t0080335	20	1	AAACCGTTACCATTAATAAG	miRNA	miR-451
t0080493	22	1	AAACCGTTACCCTTACTCAGTT	miRNA	miR-451
t0080570	22	1	AAACCGTTACTATTACTTAGTT	miRNA	miR-451
t0080591	22	1	AAGCCTTACCATTACTGAGTTT	miRNA	miR-451
t0080660	20	1	AAACCGTTAACATTACTGGG	miRNA	miR-451
t0080730	22	1	AAACCGTTACAATTAATGAGTT	miRNA	miR-451
t0080741	19	1	AAATCGTTAACATTACTGA	miRNA	miR-451
t0080813	19	1	AACCGTTACCATTACTGCG	miRNA	miR-451
t0080814	25	1	AAACCGTTACCATTACTGTGTTTAG	miRNA	miR-451
t0080831	21	1	AAACCGTTACCATTACTTATT	miRNA	miR-451
t0080893	20	1	AAACCGATAACATTACTGAG	miRNA	miR-451
t0081170	28	1	AAACCGTTACCATTACTGAATTGCCCGG	miRNA	miR-451
t0081235	20	1	AAACCTTCACCATTACTGAG	miRNA	miR-451
t0081294	21	1	AAACCGTTAACAATACTGAGT	miRNA	miR-451
t0081785	23	1	GAACCGTTACCATTACTGAGTAA	miRNA	miR-451
t0081825	24	1	AAACCGTCACCATTACTGAGTTTA	miRNA	miR-451
t0081833	22	1	AAACCTTTACCATTACTGAGAT	miRNA	miR-451
t0081852	22	1	AAACCTTTACCATTCCTGAGTT	miRNA	miR-451
t0082339	22	1	AAACAGTTACCATTCCTGAGTT	miRNA	miR-451
t0082566	23	1	AAACCTTCACCATTACTGAGTTT	miRNA	miR-451
t0082634	22	1	AACCGTTACCATCACTGAGTTT	miRNA	miR-451
t0082751	21	1	AAACCGTTACCATTAGTAAGT	miRNA	miR-451
t0083007	22	1	AAACCTTTACCATTACTGAGGT	miRNA	miR-451

t0083021	23	1	AAACCGTTACCATTCCTGGGTTT	miRNA	miR-451
t0083189	21	1	AAACCGTTCCCATTACTGAGT	miRNA	miR-451
t0083341	20	1	AAACCGTTACCATAAATGAG	miRNA	miR-451
t0083664	21	1	AAACCGTTACAATTAATGAGT	miRNA	miR-451
t0083820	24	1	AAAGCTTTACCATTACTGAGTTTA	miRNA	miR-451
t0083958	21	1	AAACCGTTACCATTCCTGCGT	miRNA	miR-451
t0084017	25	1	AAACCGTTACCATTACTGCTTTTAG	miRNA	miR-451
t0084766	22	1	AAACCGTTACCATTCCTTAGTT	miRNA	miR-451
t0084860	19	1	AAACCGTAACCATTACTGA	miRNA	miR-451
t0084936	22	1	AAACAGTTACTATTACTGAGTT	miRNA	miR-451
t0084942	23	1	AAACCGTTAGCATTATTGAGTTT	miRNA	miR-451
t0084974	22	1	AAACCGTTAACATTACTTAGTT	miRNA	miR-451
t0085262	22	1	AAACCTTTAGCATTACTGAGTT	miRNA	miR-451
t0085502	20	1	AAACCGTTATCATTATTGAG	miRNA	miR-451
t0085587	20	1	AAACCGTTGCCATTATTGAG	miRNA	miR-451
t0085624	20	1	GAACCTTTACCATTACTGAG	miRNA	miR-451
t0086021	21	1	AAAACGTTACCATTATTGAGT	miRNA	miR-451
t0086130	22	1	GAACCTTTACCATTACTGAGTT	miRNA	miR-451
t0086356	23	1	AAACCGTTACCGTTATTGAGTTT	miRNA	miR-451
t0086638	19	1	AAACCGTTACCCTTACTGA	miRNA	miR-451
t0086923	25	1	AAACCGTTACCATTACTGAGTTTTG	miRNA	miR-451
t0087239	19	1	AAACCGTTACGATTACTGA	miRNA	miR-451
t0087280	22	1	AAACCGTTACCAATACTGAGGT	miRNA	miR-451
t0087333	22	1	AAACCGTTATCATTAATGAGTT	miRNA	miR-451
t0087463	22	1	AACCCGTTACCATTACTGCGTT	miRNA	miR-451
t0087571	23	1	AAACCGGTACCATTACTGAGTTA	miRNA	miR-451
t0087712	22	1	AAACCGTTAACATTACTGCGTT	miRNA	miR-451
t0088161	22	1	AAACCCTTACCATTACTAAGTT	miRNA	miR-451
t0088222	19	1	AAACCGATACCATTACTGA	miRNA	miR-451
t0088236	20	1	AAACCGTAACCATTACTTAG	miRNA	miR-451
t0088273	21	1	AAACCTTTACCATTACTCAGT	miRNA	miR-451
t0088314	21	1	AAACCGTTATCATTATTGAGT	miRNA	miR-451
t0088477	21	1	AAACCGTTAACATTACTAAGT	miRNA	miR-451
t0088760	23	1	AAATCGTTACCATTACTGAGTTG	miRNA	miR-451
t0088863	21	1	AAACCGTTCCCATTACTGCGT	miRNA	miR-451
t0089329	22	1	AAACACTTACCATTACTGAGTT	miRNA	miR-451
t0089384	23	1	CAACCGTTACAATTACTGAGTTT	miRNA	miR-451
t0089400	21	1	GAACCTTTACCATTACTGAGT	miRNA	miR-451
t0089552	23	1	AAACCGTTACCATTTTTGAGTTT	miRNA	miR-451
t0089598	20	1	AACCGTTACCATTACTGCGT	miRNA	miR-451
t0090022	21	1	AAACCGTGACCATTACTGAGT	miRNA	miR-451
t0090068	23	1	AAAACGTTACCATTAATGAGTTT	miRNA	miR-451
t0090323	23	1	AAACCGTTAACATTACTGAGTTA	miRNA	miR-451
t0090592	22	1	AAACCGTTACCATCACTTAGTT	miRNA	miR-451
t0091172	22	1	AAAACGTTAACATTACTGAGTT	miRNA	miR-451
t0091295	21	1	AAGCCGTTACCATTACTTAGT	miRNA	miR-451
t0091736	23	1	AAACCGTTACCATTACTGCGTTA	miRNA	miR-451
t0091792	24	1	AAACCGTTAACATTAGTGAGTTTA	miRNA	miR-451
t0091941	24	1	TTTTGCGATGTGTTCCTAATGATC	miRNA	miR-450a
t0092006	21	1	TTCGGGGCCTGGGCGCGGCGA	miRNA	miR-4508
t0092038	21	1	TTCGGAGTCTGGGAGCGGCGA	miRNA	miR-4508
t0092147	19	1	TTCGGGGTCTGAGCGCGGA	miRNA	miR-4508
t0092358	21	1	TTCGGGGTCTGAGCGCGGCGA	miRNA	miR-4508
t0092544	23	1	TTCGGGGTCTGGGAGCGGCGAGA	miRNA	miR-4508
t0092566	23	1	TTCGGGGTATGGGCGCGGCGAAT	miRNA	miR-4508
t0092594	20	1	TTCGGGGCCTGGGCGCGGCG	miRNA	miR-4508
t0092673	23	1	TTCGGGGTCTGGGCGCAGCGAAT	miRNA	miR-4508
t0092757	21	1	TTCGGGGTCCGGGCGCGGCGA	miRNA	miR-4508

t0092822	20	1	TTCGGGGTCTGAGCGCGGCG	miRNA	miR-4508
t0092994	23	1	TTCGGGGGCTGGGCGCGGCGAGA	miRNA	miR-4508
t0093265	21	1	CGACCGGCTCCCGGACGGCTG	miRNA	miR-4497
t0064171	21	1	TGGGGCTAGTGATGCAGGAAG	miRNA	miR-4489
t0041247	21	1	TGGGGCTAGCGATGCAGGACG	miRNA	miR-4489
t0045534	22	1	GCTCCCTCTAAGGTCGCTCGGA	miRNA	miR-4469
t0060450	21	1	TGGCGGCGGTAGTTATGGGAT	miRNA	miR-4467
t0067446	21	1	TGGCGGCGGGAGTTATGGGCT	miRNA	miR-4467
t0071883	22	1	GGGTGCGGGCCGGCGGGGGCCT	miRNA	miR-4466
t0076322	20	1	GGGTGCGGGCCGGCGGGGAC	miRNA	miR-4466
t0077935	21	1	CGAATCCGAGTAACGGCACCA	miRNA	miR-4454
t0079344	21	1	TCGAATCCGAGTCCCGGAACC	miRNA	miR-4454
t0082177	22	1	TCGAATCAGAGTAACGGCACCA	miRNA	miR-4454
t0091545	22	1	TCGGATCCGAGTCACGGCACCC	miRNA	miR-4454
t0093401	23	1	CTCGAATCCGAGTCACGGCCCCA	miRNA	miR-4454
t0061569	22	1	TCGAATCCAAGTCACGGCACCA	miRNA	miR-4454
t0057076	22	1	TCGAATCCGAGTAACGGCAACA	miRNA	miR-4454
t0060413	22	1	TCGAATCCGAGTAACGGCCCCA	miRNA	miR-4454
t0054584	22	1	TCGAATCCGAGTCACGGCACAA	miRNA	miR-4454
t0061922	22	1	TCGAATCCGGGTCACGGCACCA	miRNA	miR-4454
t0089426	22	1	TCGAATCCGAGTCACAGCACCA	miRNA	miR-4454
t0081613	22	1	TCGAATCCGAGTCACGGAACCA	miRNA	miR-4454
t0087735	29	1	TTGGTAGAGCTGTAGACTGTAGATCTTTA	miRNA	miR-4451
t0044501	28	1	GGTAGAGCTGAAGACTGTAGATCTTTAG	miRNA	miR-4451
t0053363	21	1	TGGTAGAGCTGAGGACTGTAG	miRNA	miR-4451
t0056012	20	1	CTTGGAGGAGATGCGGTTAT	miRNA	miR-4443
t0068914	18	1	TTTGGAGGAGATGCGGGT	miRNA	miR-4443
t0072582	18	1	TTTGGAGGAGATGAGGTT	miRNA	miR-4443
t0073529	22	1	AATTTTGGAGGAGAAGCGGTTA	miRNA	miR-4443
t0076582	25	1	CCTGAGCTCACACAGAAAGTAGTTA	miRNA	miR-4435
t0078226	22	1	ATGGCTAGAGCTCACACAGAGA	miRNA	miR-4435
t0081533	22	1	ACAGGAGTGGGGGGGGGGACGT	miRNA	miR-4433
t0084643	20	1	AAAGCTGGGCTGAGAGGGCG	miRNA	miR-4429
t0089115	22	1	AAAAGCTTGGCTGAGAGGGCGA	miRNA	miR-4429
t0092470	23	1	AAAAGTTGGGCTGAGAGGGCGAT	miRNA	miR-4429
t0043384	21	1	AAAAGCTAGGCTGAGAGGGCG	miRNA	miR-4429
t0086300	23	1	AAAAGCTGGGCTGAGAGGGCGTC	miRNA	miR-4429
t0090886	24	1	AAAAGCTGGGCTGAGAGGGCGAAA	miRNA	miR-4429
t0043063	21	1	AAAGCTGGGCTGAGAGGGCGA	miRNA	miR-4429
t0068542	22	1	GAAAAGCTGGGCTGAGAGGGCG	miRNA	miR-4429
t0082606	23	1	AAAAGCTGGGCTGAGAGGGCGAG	miRNA	miR-4429
t0088765	21	1	GCAGGCAGAAGGAAGCCGTGG	miRNA	miR-4419b
t0078984	19	1	TGAGAGAAAGGCAGTTGTT	miRNA	miR-4306
t0079616	21	1	TAGAGAGAAAGGCAGTAGTTG	miRNA	miR-4306
t0044300	19	1	TGGAGAGAAAGGCAGTCGA	miRNA	miR-4306
t0050837	22	1	TGGAGAGAAAGGCAGTGGTTGA	miRNA	miR-4306
t0061579	22	1	TGGAGAGAAAGGCAGTAGCTGA	miRNA	miR-4306
t0062334	22	1	TGGAGAGAAAGGCAGTAGTTGC	miRNA	miR-4306
t0065625	22	1	TGGAGAGAAAGGCAGTACTTGA	miRNA	miR-4306
t0067595	23	1	TGGAGAGAAAGGCTGTAGTTGAT	miRNA	miR-4306
t0074211	22	1	TCCCACTGCTTCACTTGACTCG	miRNA	miR-4301
t0077440	26	1	ACTCCCACTGCTTCCCTTGACTAGCC	miRNA	miR-4301
t0086523	26	1	ACTCCCACTGCTTCACTTGCCTAGCC	miRNA	miR-4301
t0090041	21	1	GCCCACTGCTTCACTTGACTA	miRNA	miR-4301
t0091669	19	1	CTCCCACTGATTCACTTGA	miRNA	miR-4301
t0050897	20	1	ACTCTCACTGCTTCACTTGA	miRNA	miR-4301
t0062615	21	1	TCCCACTGCTTCACTTGCCTA	miRNA	miR-4301
t0063346	20	1	AGCTGCATTGTATAGGGCTA	miRNA	miR-4289

t0063901	21	1 AGAAGAATTGTACAGGGCTAT	miRNA	miR-4289
t0065514	21	1 AGAAGCATTGTAAAGGGCTAT	miRNA	miR-4289
t0080374	21	1 AGCTGCATTGTACAGGGCTAT	miRNA	miR-4289
t0081088	19	1 AGTAGCATTGTACAGGGTT	miRNA	miR-4289
t0082393	21	1 AGTACAGGGCTATCGCAGCAT	miRNA	miR-4289
t0043528	22	1 AGAAGCATTGTACAGGGCTCTT	miRNA	miR-4289
t0045469	21	1 GGCAGCATTGTACATGGCTAT	miRNA	miR-4289
t0058193	21	1 GGCAGCATTGTACAGGGCTAA	miRNA	miR-4289
t0069549	21	1 AGTACAGGGCTATAGCCGCAT	miRNA	miR-4289
t0076126	23	1 AGAAGCATTGTACAGGGCTATTT	miRNA	miR-4289
t0077519	21	1 AGCGGCATTGTATTGGGCTAT	miRNA	miR-4289
t0084979	20	1 AGTAGCATTGTACAGGGTTA	miRNA	miR-4289
t0040746	21	1 AGTAGCATTGTACAGGGCTAA	miRNA	miR-4289
t0043458	22	1 AGAAGCATTGTACAGGGCTAAT	miRNA	miR-4289
t0043843	20	1 AGTAGCGTTGTACAGGGCTA	miRNA	miR-4289
t0044165	21	1 AGAAGCATCGTACAGGGCTAT	miRNA	miR-4289
t0045778	21	1 AACAGCATTGTACAGGGCTAA	miRNA	miR-4289
t0046431	22	1 AACAGCATTGTACAGGGCTATT	miRNA	miR-4289
t0048336	20	1 AGAAGCATTGTACAGGGCTC	miRNA	miR-4289
t0049435	21	1 GGCAGCATTGTACTGGGCTAT	miRNA	miR-4289
t0051107	20	1 AGCTGCATTGTACAGGGCTA	miRNA	miR-4289
t0051442	21	1 AGCGGCATTGTACAGGGCTAC	miRNA	miR-4289
t0051922	22	1 AGGAGGATTGTACAGGGCTATT	miRNA	miR-4289
t0053870	21	1 AGTGGCATTGTACAGGGCTAT	miRNA	miR-4289
t0056915	20	1 AGAAACATTGTACAGGGCTA	miRNA	miR-4289
t0058373	23	1 AACAGCATTGTACAGGGCTATTT	miRNA	miR-4289
t0060278	22	1 AGGAGCATTGTACAGGGCTATT	miRNA	miR-4289
t0061007	19	1 GGCAGCATTGTACAGGGCT	miRNA	miR-4289
t0061235	20	1 GTCAGCATTGTACAGGGCTA	miRNA	miR-4289
t0062004	20	1 ATCAGCATTGTCCAGGGCTA	miRNA	miR-4289
t0062321	22	1 AGGAGCATTGTACAGGGCTAGT	miRNA	miR-4289
t0063795	22	1 ATGACACGATCACTCCCGTTGG	miRNA	miR-425-5p
t0064914	23	1 ATGACACGATCACTCCCGTTTAG	miRNA	miR-425-5p
t0065480	22	1 ATGAAACGATCACTCCCGTTGA	miRNA	miR-425-5p
t0067249	22	1 AATGACACGATCACTCCCGTGG	miRNA	miR-425
t0067902	22	1 AATGACACGATCACTCCCGGTG	miRNA	miR-425
t0070680	23	1 AATGACACGATCACTCCAGTTGA	miRNA	miR-425
t0075210	22	1 AATGACATGATCACTCCCGTTG	miRNA	miR-425
t0075518	23	1 AATGATACGATCCCTCCCGTTGA	miRNA	miR-425
t0075910	22	1 AATGACACGATAACTCCCGTTG	miRNA	miR-425
t0077068	23	1 AATGACACGATTACTCCCGTTGA	miRNA	miR-425
t0077413	23	1 AATGACATGATCACTCCCGTTGA	miRNA	miR-425
t0079875	23	1 AATGAAACGATCACTCCCGTTGA	miRNA	miR-425
t0089500	22	1 AATGACACGATCACTACCGTTG	miRNA	miR-425
t0044409	23	1 AATGACCCGATCACTCGCGTTGA	miRNA	miR-425
t0071887	22	1 AATGGCACGATCACTCCCGTTG	miRNA	miR-425
t0077628	23	1 AATGACACGATCACTCCCGATGA	miRNA	miR-425
t0043413	25	1 AATGACACGATCACTTCCGTTGAGA	miRNA	miR-425
t0046197	24	1 AATGACACGATCACTCCTGTTGAG	miRNA	miR-425
t0048481	21	1 AATGACACGATCACTCCCGGT	miRNA	miR-425
t0048666	22	1 AATGAAACGATCACTCCCGTTG	miRNA	miR-425
t0050855	23	1 AATGACACGATAACTCCCGTTGA	miRNA	miR-425
t0051557	22	1 AATGACACGATCATTCCCGTTG	miRNA	miR-425
t0053804	23	1 AATGACACGATCACACCCGTTGA	miRNA	miR-425
t0053952	23	1 AATGACACGATCGCTCCCGTTGA	miRNA	miR-425
t0054939	23	1 AATGACACGATCAATCCCGTTGA	miRNA	miR-425
t0060733	22	1 AATGACACGGTCACTCCCGTTG	miRNA	miR-425
t0061166	25	1 AATGACACGATCAATCCCGTTGAGA	miRNA	miR-425

t0062194	22	1	AATGACACGATCACTCCAGTTG	miRNA	miR-425
t0062333	25	1	AATGACACGATCATTCCCGTTGAGA	miRNA	miR-425
t0062348	21	1	CAAAACGAGAGGCGCTGCTAT	miRNA	miR-424*
t0063712	21	1	CAAAACGTGAGGAGCTGCTAT	miRNA	miR-424*
t0069757	20	1	CAAAGCGTGAGGCGCTGCTA	miRNA	miR-424*
t0071116	20	1	CAAAACGAGAGGCGCTGCTA	miRNA	miR-424*
t0076274	21	1	CAGCAGCATTTCATGTTTTGA	miRNA	miR-424
t0076786	19	1	CAGCAGCACTTCATGTTTT	miRNA	miR-424
t0077895	21	1	CAGCAGCGATTCATGTTTTGA	miRNA	miR-424
t0083969	21	1	CAGCATCAATTCATGTTTTGA	miRNA	miR-424
t0086752	21	1	CAGCAGCAATTCATGTTTTCA	miRNA	miR-424
t0087517	21	1	CAGCAGCAATTCATGGTTTGA	miRNA	miR-424
t0088664	21	1	CAGCAACAATTCATGTTTTGA	miRNA	miR-424
t0089607	23	1	CAGCAGCAATTCATGTTTTGACA	miRNA	miR-424
t0093282	19	1	CAGCAGCAAATCATGGTTT	miRNA	miR-424
t0064067	21	1	CAGCAGAAATTCATGTTTTGA	miRNA	miR-424
t0084987	21	1	CAGCAGTAATTCATGTTTTGA	miRNA	miR-424
t0089109	21	1	CAGCAGCAATTCATGTTTTTA	miRNA	miR-424
t0090199	21	1	CAGCAGCAATTCATGTTTCGA	miRNA	miR-424
t0043328	21	1	CAGCAGCAACTCATGTTTTGA	miRNA	miR-424
t0043392	20	1	CAGCAGCATTTCATGTTTTG	miRNA	miR-424
t0060284	23	1	TGAGGGGCAGAGTGCGAGAGTTT	miRNA	miR-423-5p
t0062006	21	1	TGAGGGGCAGACAGAGAGACT	miRNA	miR-423-5p
t0067022	22	1	TGAGGGGAAGAGGGCGAGACTT	miRNA	miR-423-5p
t0069771	21	1	TGAGAGGCAGAGAGTGAGACT	miRNA	miR-423-5p
t0070324	23	1	TGAGGGGCGGAGCGCGAGACTTT	miRNA	miR-423-5p
t0070587	21	1	TGAGGGGAAGAGAGCGAGACA	miRNA	miR-423-5p
t0070860	22	1	TGAGGGGCAGAGAGAGAGACTG	miRNA	miR-423-5p
t0073399	23	1	TGAGGGGAAGGGAGCGAGACTTT	miRNA	miR-423-5p
t0078537	21	1	CGAGGGGTAGAGAGCGAGACT	miRNA	miR-423-5p
t0081444	23	1	TGAGGGGCAGAGGGCGAGACTTG	miRNA	miR-423-5p
t0086192	22	1	TGAGGGGCAGAGAGCGAGGCCT	miRNA	miR-423-5p
t0086283	21	1	TAAGGGGCAGAGAGCGAGAAT	miRNA	miR-423-5p
t0088659	23	1	TGAGGGGTAGACAGCGAGACTTT	miRNA	miR-423-5p
t0041054	23	1	TGAGGGGCAGAGGGGGAGACTTT	miRNA	miR-423-5p
t0041248	21	1	TGAGGGGCAGAGAGGGAGATT	miRNA	miR-423-5p
t0041357	21	1	TGAGGGACAGAGAGAGAGACT	miRNA	miR-423-5p
t0041392	23	1	TGAGGGGCATAGAGCTAGACTTT	miRNA	miR-423-5p
t0041394	23	1	TGATGGGCAGAGGGCGAGACTTT	miRNA	miR-423-5p
t0041463	23	1	TGAGAGGCAGATAGCGAGACTTT	miRNA	miR-423-5p
t0041696	23	1	TGAAGGGCAGAGAGCGAGATTTT	miRNA	miR-423-5p
t0041742	22	1	TGAGGGGTAGAGACCGAGACTT	miRNA	miR-423-5p
t0041852	23	1	TGAGGGGCAGAGACCGAGGCTTT	miRNA	miR-423-5p
t0041973	23	1	CGAGGGGCAGAGAGCGAGCCTTT	miRNA	miR-423-5p
t0042100	22	1	TGAGGGGCAGAGAGCGAGCATT	miRNA	miR-423-5p
t0042209	42	1	TGAGGGGCAGAGAGCGAGCCTTTGTGTG	miRNA	miR-423-5p
t0042468	21	1	TTAGGGACAGAGAGCGAGACT	miRNA	miR-423-5p
t0042650	23	1	TGAGGGGAAGAGAGCGAGCCTTT	miRNA	miR-423-5p
t0042671	22	1	TGGGGGGCAGAGAGCTAGACTT	miRNA	miR-423-5p
t0042709	22	1	TGAGGGGCAGAAAGAGAGACTT	miRNA	miR-423-5p
t0043087	23	1	TGAGGGGCAAAGGGCGAGACTTT	miRNA	miR-423-5p
t0043194	24	1	TGAGGGACAGATAGCGAGACTTTT	miRNA	miR-423-5p
t0043639	21	1	TGAGGGGCAGAGAGCGGGATT	miRNA	miR-423-5p
t0043682	24	1	TGAGGGCCAGAGAGCGAGACTTTT	miRNA	miR-423-5p
t0043740	19	1	TGAGGGGCAGATAACGAGA	miRNA	miR-423-5p
t0043991	24	1	TGAGGGGCTGAGAGCGAGACTTTT	miRNA	miR-423-5p
t0044238	23	1	TTAGGGGCAGAGAGCGAGCCTTT	miRNA	miR-423-5p
t0044346	18	1	TGAGGGGCAGAGAGGGAG	miRNA	miR-423-5p

t0044348	23	1 TGAGGGGAAAAGAGCGAGACTTT	miRNA	miR-423-5p
t0044383	21	1 AGGGGCAGAGAGCGAGCCTTT	miRNA	miR-423-5p
t0044787	21	1 TGAGTGGCAGAGAGAGAGACT	miRNA	miR-423-5p
t0044814	24	1 TGAGGGGCAGAGAGCGAGACTTCA	miRNA	miR-423-5p
t0044915	21	1 AAGGGCAGAGAGCGAGACTTT	miRNA	miR-423-5p
t0044930	23	1 TGATGGGCAGAGAGAGAGACTTT	miRNA	miR-423-5p
t0045012	27	1 TGAGGGGCAGAGAGCGAGCCTTTAGGT	miRNA	miR-423-5p
t0045311	22	1 TCAGGGGCAGAGAGAGAGACTT	miRNA	miR-423-5p
t0045407	24	1 TGAGGGGCAGAGCGCGAGACTTAT	miRNA	miR-423-5p
t0045483	24	1 TTAGGGGCAGAGAGCGAGACTTTT	miRNA	miR-423-5p
t0045488	19	1 TGAGGGGCAGAGAGCCAGA	miRNA	miR-423-5p
t0045529	22	1 TGAGGGGAAGAGAGTGAGACTT	miRNA	miR-423-5p
t0045568	21	1 TGAGGGGCAGAGAGTGGGACT	miRNA	miR-423-5p
t0045596	23	1 TGAGGGGCAGATAGAGAGACTTT	miRNA	miR-423-5p
t0045901	24	1 TGAGGGGCAGAGAGCGAGAAGTTT	miRNA	miR-423-5p
t0046181	23	1 TGACGGGCAAAGAGCGAGACTTT	miRNA	miR-423-5p
t0046203	23	1 TAAGGGGCAGAGAGCGAGCCTTT	miRNA	miR-423-5p
t0046238	21	1 TGAGTGGCAGAGAGCGGGACT	miRNA	miR-423-5p
t0046271	23	1 TGGGGGGCAGAGAGCGGGACTTT	miRNA	miR-423-5p
t0046275	24	1 TGAGGGGCAGAGAGCGAGTCTTTT	miRNA	miR-423-5p
t0046787	25	1 TGAGGGGCAGAGAGTGAGACTTTTT	miRNA	miR-423-5p
t0047028	24	1 TGAGGGGCAGAGAGCGAGAGTTAA	miRNA	miR-423-5p
t0047267	23	1 TCAGGGGCAGAGAGCGAGAATTT	miRNA	miR-423-5p
t0047760	21	1 AGAGGGGCAGAGAGCGAGGCT	miRNA	miR-423-5p
t0047927	24	1 TGAGGGGCAGAGAGCCAGAATTTT	miRNA	miR-423-5p
t0047934	22	1 TTTGAGGGGCAGAGAGCGAGCC	miRNA	miR-423-5p
t0047951	22	1 TGAGGAGCAGAGCGCGAGACTT	miRNA	miR-423-5p
t0048043	23	1 TGAGGGGCAGAGAAAGAGACTTT	miRNA	miR-423-5p
t0048182	23	1 TGATAGGCAGAGAGCGAGACTTT	miRNA	miR-423-5p
t0048482	21	1 TGAGGGGCAGAGAGAGAGTCT	miRNA	miR-423-5p
t0048578	22	1 AGGGGCAGAGAGCGAGGCTTTT	miRNA	miR-423-5p
t0048854	22	1 TGAGGGTCAGAGAGCGAGACTC	miRNA	miR-423-5p
t0049036	23	1 TGAGGGGCAGAGATCGAGACTCT	miRNA	miR-423-5p
t0049079	23	1 TGAGGGGCAGGGAGCGAGCCTTT	miRNA	miR-423-5p
t0049115	22	1 TGAGGGGAAGAGAGCGACACTT	miRNA	miR-423-5p
t0049262	24	1 TGAGGGGCAGAGGGCGAGACTTTA	miRNA	miR-423-5p
t0049558	21	1 TGAGGGGCAGATAGGGAGACT	miRNA	miR-423-5p
t0049936	23	1 TGAGGGGCAGAGAGCGTGACTTA	miRNA	miR-423-5p
t0050114	23	1 TGAGGGGCAGAGACCGAGACTTG	miRNA	miR-423-5p
t0050187	23	1 TGGGGGGCAGAGAGCGAGAATTT	miRNA	miR-423-5p
t0050221	21	1 TGAGGGGAAGACAGCGAGACT	miRNA	miR-423-5p
t0050248	21	1 AGGGGCAGAGAGCGAGAATTT	miRNA	miR-423-5p
t0050401	21	1 TGAGGGGCAGAGAGCCAGACA	miRNA	miR-423-5p
t0050575	24	1 TGTGGGGCAGAGAGCGAGACTTTT	miRNA	miR-423-5p
t0050637	19	1 TGAGGGGCAGAGTGCGAGA	miRNA	miR-423-5p
t0050712	23	1 TGAGGGGAAGATAGCGAGACTTT	miRNA	miR-423-5p
t0050815	21	1 TGAGGGGGAGAGAGCTAGACT	miRNA	miR-423-5p
t0051159	21	1 TGAGTGGCAGAGAGCAAGACT	miRNA	miR-423-5p
t0051169	23	1 TGTGGGGCAGAGAGCGAGACTTA	miRNA	miR-423-5p
t0051178	21	1 TGAGGGGCAGATGGCGAGACT	miRNA	miR-423-5p
t0051308	23	1 TGAGGGGCAGAGAGCGAGGTTTT	miRNA	miR-423-5p
t0051405	23	1 TGAGAGGCAGAGACCGAGACTTT	miRNA	miR-423-5p
t0051543	23	1 CGAAGGGCAGAGAGCGAGACTTT	miRNA	miR-423-5p
t0051559	22	1 TGACGGGCAGATAGCGAGACTT	miRNA	miR-423-5p
t0051670	21	1 TGAGGGGCAGATAGCGAGTCT	miRNA	miR-423-5p
t0051748	22	1 TTTGAGGGGCCGAGAGCGAGAC	miRNA	miR-423-5p
t0052038	24	1 TGAGGGGCAGAGCGCGAGAATTTT	miRNA	miR-423-5p
t0052053	19	1 TGAGGGGCAGGGAGCGAGA	miRNA	miR-423-5p

t0052488	22	1	TGAGGGGCAGAGGGCGAGAATT	miRNA	miR-423-5p
t0052665	24	1	TGAGGGGCAGAGGGCGAGACTTAT	miRNA	miR-423-5p
t0052756	23	1	TAAGGGGCAGAGAGCGAGATTTT	miRNA	miR-423-5p
t0052839	20	1	TGAGGGACAGAGAGCGAGAC	miRNA	miR-423-5p
t0053314	23	1	TGGGGGGCAGAGAGCGAGACATT	miRNA	miR-423-5p
t0053473	22	1	TGAGGGGCAGAGAGCGAGGCTA	miRNA	miR-423-5p
t0053872	22	1	AGGAGCAGAGAGCGAGACTTTT	miRNA	miR-423-5p
t0054116	21	1	TTGAGGGGCAGAGCGCGAGAC	miRNA	miR-423-5p
t0054235	42	1	TGAGGGGCAGAGAGCGAGACTTTTCGTTT	miRNA	miR-423-5p
t0054390	23	1	TGACGGGCAGAGAGCGAGACTTA	miRNA	miR-423-5p
t0054621	22	1	TGAGCGACAGAGAGCGAGACTT	miRNA	miR-423-5p
t0054965	20	1	TAGGGGCAGAGAGAGAGACT	miRNA	miR-423-5p
t0055396	20	1	AGGGGCAGAGGGCGAGACTT	miRNA	miR-423-5p
t0055419	21	1	TGAGGGGCAGAGAGAGAGCCT	miRNA	miR-423-5p
t0055994	23	1	TGAGGGGCAGACAGCGAGCCTTT	miRNA	miR-423-5p
t0056297	21	1	TGAATGGCAGAGAGCGAGACT	miRNA	miR-423-5p
t0056464	23	1	TGGGGGGCAGAGAGCGAGATTTT	miRNA	miR-423-5p
t0056558	21	1	TGAGGGGAAGAGAGCGGGACT	miRNA	miR-423-5p
t0056674	22	1	AGGGGCAGAGAACGAGACTTTT	miRNA	miR-423-5p
t0056761	24	1	TGAGGGGAAGAGAGCGAGACTTTA	miRNA	miR-423-5p
t0057144	22	1	TGAGAGGCAGAGAGCGAGAATT	miRNA	miR-423-5p
t0057629	21	1	AGGGGCAGAGAGGGAGACTTT	miRNA	miR-423-5p
t0058039	24	1	TGAGGGGAAGAGAGCGAGAATTTA	miRNA	miR-423-5p
t0058061	24	1	TGGGGGGCAGAGAGCGAGACTTGA	miRNA	miR-423-5p
t0058149	23	1	TGAGCAGCAGAGAGCGAGACTTT	miRNA	miR-423-5p
t0058352	21	1	TGAGGGGCAGAGGCCGAGACT	miRNA	miR-423-5p
t0058677	22	1	GGGGGGCAGAGAGCGAGACTTT	miRNA	miR-423-5p
t0058911	23	1	TGAGGGGAAGAGGGCGAGACTTT	miRNA	miR-423-5p
t0059125	23	1	TGAGGGGAAGAAAGCGAGACTTT	miRNA	miR-423-5p
t0059157	22	1	TGAGGGGCGGAGAGCGAGACTA	miRNA	miR-423-5p
t0059251	20	1	TGAGGAGCAGAGAGAGAGAC	miRNA	miR-423-5p
t0059292	23	1	TGAGGGGCAGAGAGCGAGACATA	miRNA	miR-423-5p
t0059300	21	1	TGAGGGGCAGAGAGAGACACT	miRNA	miR-423-5p
t0059387	22	1	TGAGGGGCAGAGAGCGAGGTTT	miRNA	miR-423-5p
t0059742	23	1	TGAGGGGCAGAGCGCGAGGCTTT	miRNA	miR-423-5p
t0059760	23	1	TGAGGGGCAGATAGCGAGGCTTT	miRNA	miR-423-5p
t0059868	21	1	TGAGGGACAGATAGCGAGACT	miRNA	miR-423-5p
t0059911	23	1	TGAGGGGCAGACAGCAAGACTTT	miRNA	miR-423-5p
t0059973	22	1	TGAGGGGAAGAGAGCGAGACTA	miRNA	miR-423-5p
t0060064	23	1	TGAGGGGCAGAGAGGGATACTTT	miRNA	miR-423-5p
t0060126	22	1	GAGGGGAAGAGAGCGAGACTTT	miRNA	miR-423-5p
t0060152	24	1	CTGAGGGGCAGAGAGCGAGCCTTT	miRNA	miR-423-5p
t0060177	20	1	GAGGGGAAGAGAGCGAGACT	miRNA	miR-423-5p
t0060448	21	1	TGAGGGGCAGAGAGCGGGAAT	miRNA	miR-423-5p
t0060465	23	1	TGAGCGGCAGAGAGCGGGACTTT	miRNA	miR-423-5p
t0060515	21	1	TGAAGGGCAGAGAGCGAGAAT	miRNA	miR-423-5p
t0060555	22	1	TGCGGGGCAGAGGGCGAGACTT	miRNA	miR-423-5p
t0060676	24	1	TGAGGGGCAGAGAGCGGGACTTAT	miRNA	miR-423-5p
t0060700	23	1	TGAGGGGCAGGGAGCGACACTTT	miRNA	miR-423-5p
t0060722	23	1	TGAGGGGCGGAGAGCGAGAATTT	miRNA	miR-423-5p
t0060728	23	1	TGAGGGGCAGAGGGCAAGACTTT	miRNA	miR-423-5p
t0060791	22	1	TGAGGGGCAGAGAGCCAGATTT	miRNA	miR-423-5p
t0061191	21	1	TGGGGGGCATAGAGCGAGACT	miRNA	miR-423-5p
t0061345	22	1	TGAGGGGCAGAGGGCTAGACTT	miRNA	miR-423-5p
t0061535	23	1	TGAGGGGAAGAGAGCTAGACTTT	miRNA	miR-423-5p
t0061544	23	1	TGAGGAGCAGAGAGCTAGACTTT	miRNA	miR-423-5p
t0061595	21	1	TGAGGGGCAGCGAGAGAGACT	miRNA	miR-423-5p
t0061684	23	1	TGAGGGGCAGCGAGCGAGACCTT	miRNA	miR-423-5p

t0061696	22	1	TGAGGGACAGAGAGCTAGACTT	miRNA	miR-423-5p
t0061805	22	1	TGAGGGGCAGCCAGCGAGACTT	miRNA	miR-423-5p
t0062061	21	1	TGAGGGGCAGCGAGCGAGAAT	miRNA	miR-423-5p
t0062071	23	1	TGAGGGGGAGAGAGAGAGACTTT	miRNA	miR-423-5p
t0062377	22	1	TGAGGGGAAGAGAGCGAGATTT	miRNA	miR-423-5p
t0062513	24	1	TGAGGGGCAGAGAGCGAGCCTTAT	miRNA	miR-423-5p
t0063198	20	1	AGGCGCAGAGAGCGAGACTT	miRNA	miR-423-5p
t0063291	21	1	TGAGTGGCAGAGAGCGAGAAT	miRNA	miR-423-5p
t0063315	23	1	TGAGGTGCAGAGAGCGGGACTTT	miRNA	miR-423-5p
t0063363	23	1	TAAAGGGCAGAGAGCGAGACTTT	miRNA	miR-423-5p
t0063598	21	1	TGAGGGGCAGAGAGAGCGACT	miRNA	miR-423-5p
t0063822	23	1	TGAGGGGAAGTGAGCGAGACTTT	miRNA	miR-423-5p
t0064249	23	1	TGATGGGTAGAGAGCGAGACTTT	miRNA	miR-423-5p
t0064274	22	1	TGATGGGCAGGGAGCGAGACTT	miRNA	miR-423-5p
t0064436	22	1	TAAGGGCAGAGAGCGAGACTTT	miRNA	miR-423-5p
t0064481	23	1	CGAGGGGCAGAGAGTGAGACTTT	miRNA	miR-423-5p
t0064691	21	1	TGCGGGGCAGAGAGCGAGACA	miRNA	miR-423-5p
t0064980	23	1	TGAGGGGCAGAGAGCAAGATTTT	miRNA	miR-423-5p
t0064992	23	1	TGAGGGGCAGAGTGCGAGAATTT	miRNA	miR-423-5p
t0065009	22	1	TAAGGGGCAGAGAGCGAGACTA	miRNA	miR-423-5p
t0065121	19	1	TGAGGGGCGGAGAGAGAGA	miRNA	miR-423-5p
t0065246	22	1	TGAGTGGCAGAGAGAGAGACTT	miRNA	miR-423-5p
t0065774	23	1	TGAGGGGAAGAGAGCGAGACTTG	miRNA	miR-423-5p
t0065778	23	1	TGAGGGGCAGAGAGCTAGAATTT	miRNA	miR-423-5p
t0065826	21	1	TGAGGGGCAGAGCGAGAGACT	miRNA	miR-423-5p
t0065903	22	1	TGAGGGGTAGAGAGAGAGACTT	miRNA	miR-423-5p
t0065964	23	1	TGAGGGTCAGATAGCGAGACTTT	miRNA	miR-423-5p
t0066025	21	1	TGAGGGGCAGGGAGCGAGATT	miRNA	miR-423-5p
t0066029	21	1	TGAGGGGCAGAGAGCAAGACA	miRNA	miR-423-5p
t0066111	23	1	TGAGGGGCGGAGAGCGAGCCTTT	miRNA	miR-423-5p
t0066189	21	1	TGAGGGGTAGAGGGCGAGACT	miRNA	miR-423-5p
t0066240	23	1	TGAGGGGCAGAGAGACAGACTTT	miRNA	miR-423-5p
t0066356	22	1	GGAGGGGCAGAGAACGAGACTT	miRNA	miR-423-5p
t0066464	21	1	TGAGGGGAAGAGAGCGAGAGT	miRNA	miR-423-5p
t0066766	23	1	CGAGGGGCAGAGAGAGAGACTTT	miRNA	miR-423-5p
t0067013	23	1	TGAGGGCCAGAGAGCGAGAATTT	miRNA	miR-423-5p
t0067287	23	1	TGAGGGCCAGAGAGCGAGACTTA	miRNA	miR-423-5p
t0067552	23	1	TGAGGGGCAGAGAGCTAGACCTT	miRNA	miR-423-5p
t0067880	20	1	TGGGGGGCAGAGAGCGAGAC	miRNA	miR-423-5p
t0068009	22	1	GAGGAGCAGAGAGCGAGACTTA	miRNA	miR-423-5p
t0068277	23	1	TGAGGGCCAGATAGCGAGACTTT	miRNA	miR-423-5p
t0068308	23	1	TGAGGGGTAGAGAGCGAGATTTT	miRNA	miR-423-5p
t0068513	23	1	TGAGGGGAAGAGAGCGAGACTCT	miRNA	miR-423-5p
t0068517	23	1	CGAGGGGCAGAGACCGAGACTTT	miRNA	miR-423-5p
t0068634	23	1	TGAGGGGTAGAGAGAGAGACTTT	miRNA	miR-423-5p
t0068723	23	1	TGAGGGGGAGAGAGCGAGACTTG	miRNA	miR-423-5p
t0068819	20	1	TGAGGCAGAGAGCGAGACTT	miRNA	miR-423-5p
t0068862	24	1	TGAGGGGCAGAGAGTGAGACTTAA	miRNA	miR-423-5p
t0069041	21	1	TGAGGGGCGGAGGGCGAGACT	miRNA	miR-423-5p
t0069207	22	1	GAGGGGCAGAGAGCGAGCCTTA	miRNA	miR-423-5p
t0069422	23	1	TGAGGGGCGGAGAGCGAGTCTTT	miRNA	miR-423-5p
t0069530	22	1	TGAGAGGGAGAGAGCGAGACTT	miRNA	miR-423-5p
t0069618	23	1	TGAGGGGCAGAGAGCAAGCCTTT	miRNA	miR-423-5p
t0069764	24	1	TGAGGGGCAGAGGGCGAGACTTAG	miRNA	miR-423-5p
t0069811	23	1	TGAGGGGCTGAGAGAGAGACTTT	miRNA	miR-423-5p
t0069854	21	1	TGAGTGGGAGAGAGCGAGACT	miRNA	miR-423-5p
t0069933	21	1	TGAGGGGCAGAGAGCTAGAAT	miRNA	miR-423-5p
t0070227	23	1	TGAGGGGCATAGAGCAAGACTTT	miRNA	miR-423-5p

t0070263	23	1	TGAGGGGCAGAGAGTGAGGCTTT	miRNA	miR-423-5p
t0070349	21	1	TGAGGGGCAGAGAGGGAGACA	miRNA	miR-423-5p
t0070510	23	1	TAAGGGGCGGAGAGCGAGACTTT	miRNA	miR-423-5p
t0070517	24	1	TGAGGGGCAGAGAACGAGACTTAC	miRNA	miR-423-5p
t0070625	21	1	TGCGGGGCAGAGCGCGAGACT	miRNA	miR-423-5p
t0070658	21	1	TGAGGGGGAGAGAGCGAGATT	miRNA	miR-423-5p
t0070694	21	1	TGAGGGGCAGAGAGCCAGCCT	miRNA	miR-423-5p
t0070747	22	1	TGAGGGGCAGCGAGCGCGACTT	miRNA	miR-423-5p
t0070789	21	1	TGGGGAGCAGAGAGCGAGACT	miRNA	miR-423-5p
t0070840	21	1	TGAGGTGCGGAGAGCGAGACT	miRNA	miR-423-5p
t0071490	24	1	TGAGGGGCAGAGAGTGAGACTTTC	miRNA	miR-423-5p
t0071889	23	1	TGAGGGGCAGCGAGCGAGCCTTT	miRNA	miR-423-5p
t0071993	24	1	TGAGGGGCAGAGAGGGAGACTTTA	miRNA	miR-423-5p
t0072151	23	1	TGAGGGGCAGAGAGAGAGATTTT	miRNA	miR-423-5p
t0072234	23	1	TGAGTGGCAGAGAGCGGGACTTT	miRNA	miR-423-5p
t0072321	22	1	TGAGGGGCAGAGAGCGAGACCA	miRNA	miR-423-5p
t0072437	18	1	TGAGGGGCGGAGAGCGAG	miRNA	miR-423-5p
t0072559	23	1	AGGGGCAGAGAGCGAGACTTTTA	miRNA	miR-423-5p
t0072599	27	1	TGAGGGGCAGAGTGCGAGACTTTAGGT	miRNA	miR-423-5p
t0072680	23	1	TGAAGGGCAGAGAGCGAGCCTTT	miRNA	miR-423-5p
t0072701	21	1	TGAAGGGCAGAGAGCGGGACT	miRNA	miR-423-5p
t0072738	23	1	TGAGGGGCAGAGAGCCCGACTTT	miRNA	miR-423-5p
t0072825	23	1	TGAGGGGCAGAGAGCGGGACTTC	miRNA	miR-423-5p
t0073083	22	1	TGAGGGGCAGACAGAGAGACTT	miRNA	miR-423-5p
t0073171	22	1	TGAGGGGCAAAGAGCAAGACTT	miRNA	miR-423-5p
t0073253	23	1	TAAGGGGCAGATAGCGAGACTTT	miRNA	miR-423-5p
t0073288	21	1	TGAGGGGCAGAGGGCGAGTCT	miRNA	miR-423-5p
t0073639	21	1	CGAGGGGCAGAGAGCGAGGCT	miRNA	miR-423-5p
t0073794	23	1	AGATGGGCAGAGAGCGAGACTTT	miRNA	miR-423-5p
t0073976	21	1	TGAGGGGCAGATAGCGAGAAT	miRNA	miR-423-5p
t0074167	23	1	TGAGGGGCAGAGAGAGAGACTCT	miRNA	miR-423-5p
t0074201	23	1	TGAGGTGCAGAGAGCGAGACTTG	miRNA	miR-423-5p
t0074782	21	1	TGAGGGGCAGCGGGCGAGACT	miRNA	miR-423-5p
t0074838	20	1	TAAGGGGCAGAGAGCGAGAC	miRNA	miR-423-5p
t0075014	22	1	TGGGGGGCAGAGAGCGAGAATT	miRNA	miR-423-5p
t0075119	21	1	TGAGGGGCAGGGAGCGAGACA	miRNA	miR-423-5p
t0075193	21	1	TAAGGGGCAGAGAGCAAGACT	miRNA	miR-423-5p
t0075214	23	1	TGGGGGGCAGAGAGCTAGACTTT	miRNA	miR-423-5p
t0075238	23	1	TGAGGGGCAGAAAGCGAGAATTT	miRNA	miR-423-5p
t0075240	23	1	TGGGGGGTAGAGAGCGAGACTTT	miRNA	miR-423-5p
t0075568	21	1	TAGGGGCAGAGAGTGAGACTT	miRNA	miR-423-5p
t0075643	23	1	TGAGAGGCAGAGAGCTAGACTTT	miRNA	miR-423-5p
t0075866	25	1	CGAGGGGCAGAGAGCGAGACTTTTT	miRNA	miR-423-5p
t0075953	23	1	TGAGGGGCTGAGAGCGTGACTTT	miRNA	miR-423-5p
t0075993	21	1	GAGGGGCAGAGCGCGAGACTT	miRNA	miR-423-5p
t0076696	22	1	TGGGGGGCAGAGGGCGAGACTT	miRNA	miR-423-5p
t0076721	20	1	TGAGGGGCAGAGAGTGAGAC	miRNA	miR-423-5p
t0076910	22	1	TAAGGGGCAGAGACCGAGACTT	miRNA	miR-423-5p
t0077194	21	1	TGAGGGGCAGAGATCGCGACT	miRNA	miR-423-5p
t0077252	22	1	TAAGGGGCAGAGAGAGAGACTT	miRNA	miR-423-5p
t0077332	20	1	TGAGGGGCAGAGAGAGAGAA	miRNA	miR-423-5p
t0077607	24	1	AGAGGGGCAGAGAGCGAGACTTTT	miRNA	miR-423-5p
t0077610	22	1	TGAGGGGCAGAGAGCGAGCTTT	miRNA	miR-423-5p
t0077624	20	1	TGAGGCGCAGAGAGCGAGAC	miRNA	miR-423-5p
t0077749	22	1	TGATGGCCAGAGAGCGAGACTT	miRNA	miR-423-5p
t0077810	22	1	ATAGGGGCAGAGAGCGAGACTT	miRNA	miR-423-5p
t0078349	23	1	TGAGGGACAGGGAGCGAGACTTT	miRNA	miR-423-5p
t0078579	27	1	TGAGGGGCAGAGAGCGAGACTTTTGTT	miRNA	miR-423-5p

t0078620	23	1	TGCGTGGCAGAGAGCGAGACTTT	miRNA	miR-423-5p
t0078716	22	1	TGAGGGGCAGAGAGAGAGAGTT	miRNA	miR-423-5p
t0078994	22	1	TGAGGGGCCGAGAGAGAGACTT	miRNA	miR-423-5p
t0079035	19	1	TGAGGCGCAGAGAGCGAGA	miRNA	miR-423-5p
t0079172	21	1	TGAGGCGCAGAGAGCGAGCCT	miRNA	miR-423-5p
t0079271	24	1	TCAGGGGCAGAGAGCGAGACTTTT	miRNA	miR-423-5p
t0079322	22	1	TAAGGGGCAGAGAGCTAGACTT	miRNA	miR-423-5p
t0079365	21	1	TGAGGGGCAGAGAGCGTGCCT	miRNA	miR-423-5p
t0079414	18	1	TGAGGGGCAGCGAGCGAG	miRNA	miR-423-5p
t0079531	22	1	TGAGGGGCAGAGAGCGAGATTC	miRNA	miR-423-5p
t0079549	23	1	TGAGGGGCAGAGAGCAAGACCTT	miRNA	miR-423-5p
t0079646	21	1	GGAGGGGCAGATAGCGAGACT	miRNA	miR-423-5p
t0079891	21	1	TGAGGGGCAGAGGGCGAGAAT	miRNA	miR-423-5p
t0079966	21	1	TGAGGGGCAGATAGCGAGAGT	miRNA	miR-423-5p
t0080069	24	1	TGAGGGGCAGAGAGCGAGGCTTTA	miRNA	miR-423-5p
t0080215	24	1	TGAGGGGCAGAGAGAGAGACTTGA	miRNA	miR-423-5p
t0080250	22	1	TGAGGGGCAGAGACCGAGCCTT	miRNA	miR-423-5p
t0080445	23	1	TGAGGGGCATAGCGCGAGACTTT	miRNA	miR-423-5p
t0080477	24	1	TGAGGGGCAGAGAGCGAGAACTTT	miRNA	miR-423-5p
t0080492	23	1	TGAGGGGCAGACAGAGAGACTTT	miRNA	miR-423-5p
t0080497	23	1	TGAGGGGAAGAGAGGGAGACTTT	miRNA	miR-423-5p
t0080672	24	1	TGAGGGGCAGAGTGCGAGACTTGA	miRNA	miR-423-5p
t0080763	18	1	TGAGGGACAGAGAGCGAG	miRNA	miR-423-5p
t0080865	24	1	AGAGGGGCAGAGAGCGAGACTTAG	miRNA	miR-423-5p
t0080987	21	1	TGAGGGGCACAGAGCGAGTCT	miRNA	miR-423-5p
t0081164	21	1	AGGGGCAGAGCGCGAGACTTT	miRNA	miR-423-5p
t0081423	23	1	TGCGGGGCAGAGAGCGAGAATTT	miRNA	miR-423-5p
t0081458	24	1	TGAGAGGCAGAGAGCGAGACTTAG	miRNA	miR-423-5p
t0081539	24	1	TGAGGGGCAGAGAGCGAAACTTTT	miRNA	miR-423-5p
t0081675	23	1	TGAGGGGAAGAGAGCGACACTTT	miRNA	miR-423-5p
t0081903	22	1	GAGGGGCGGAGAGCGAGACTTT	miRNA	miR-423-5p
t0082063	21	1	TGAGTGGCAGAGGGCGAGACT	miRNA	miR-423-5p
t0082099	23	1	TGAGGGGCAGAGGGCTAGACTTT	miRNA	miR-423-5p
t0082118	23	1	TGAGGGGAAGAGAGCCAGACTTT	miRNA	miR-423-5p
t0082331	23	1	TGAAGGGCAGAGAGCGAGAGTTT	miRNA	miR-423-5p
t0082356	21	1	TGGGGGGCAGAGAGCGATACT	miRNA	miR-423-5p
t0082455	21	1	TGAGGGGTAGAGAGCGGGACT	miRNA	miR-423-5p
t0082714	18	1	TGAGGGGCAGAGCGCGAG	miRNA	miR-423-5p
t0082757	23	1	TGGGGGGCAGAGATCGAGACTTT	miRNA	miR-423-5p
t0083039	21	1	TGAGGGTCAGAGAGCGGGACT	miRNA	miR-423-5p
t0083627	21	1	CGAGGGGAAGAGAGCGAGACT	miRNA	miR-423-5p
t0083659	21	1	TGAGGGGCAGAGCGCGAGAAT	miRNA	miR-423-5p
t0083834	23	1	TGAGTGGAAGAGAGCGAGACTTT	miRNA	miR-423-5p
t0083906	22	1	TGAGGGGAAGAGAGCGAGTCTT	miRNA	miR-423-5p
t0083917	23	1	TGAGGGGCAGAGAGGGAGACTTG	miRNA	miR-423-5p
t0083996	24	1	TGAGGGGGAGAGAGCGAGACTTTA	miRNA	miR-423-5p
t0084182	21	1	TGAGGTGCAGAGAGCGAGGCT	miRNA	miR-423-5p
t0084235	23	1	TGAGGGGCATAGAGCGAGACTTA	miRNA	miR-423-5p
t0084252	21	1	TGAGGGGGAGAGAGCGGGACT	miRNA	miR-423-5p
t0084382	18	1	TGAGGGGCAGAGAGAGAG	miRNA	miR-423-5p
t0084599	24	1	TGAGGGGCAGAGAGCGAGATTTAT	miRNA	miR-423-5p
t0084681	23	1	TGAGGGGCAGAGCGCTAGACTTT	miRNA	miR-423-5p
t0084746	19	1	GAGGGGCAGAGAGCGCGAC	miRNA	miR-423-5p
t0084941	21	1	TGAGGGGCAGAGAGCGGGACA	miRNA	miR-423-5p
t0084962	21	1	TGAGGGGCAGAGAGTGAGCCT	miRNA	miR-423-5p
t0085084	24	1	TGAGGAGCAGAGAGCGAGACTTAG	miRNA	miR-423-5p
t0085226	23	1	TGAGGGGCAGAGAGCGAGGGTTT	miRNA	miR-423-5p
t0085383	21	1	TGAGGGGTAGAGAGCGAGACA	miRNA	miR-423-5p

t0085432	23	1	TGAGTGGCAGAGAGAGAGACTTT	miRNA	miR-423-5p
t0085593	21	1	TGAGGGGCAGAGACGGAGACT	miRNA	miR-423-5p
t0085607	19	1	TGCGGGGCAGAGAGCGAGA	miRNA	miR-423-5p
t0085703	23	1	TGAGGGCTAGAGAGCGAGACTTT	miRNA	miR-423-5p
t0085782	23	1	TGATGGGCAGAGAGCGAGACTTC	miRNA	miR-423-5p
t0085827	23	1	TGAGGGGCAGAGAGCCGGACTTT	miRNA	miR-423-5p
t0086023	22	1	GAGGGGCCGAGAGCGAGACTTT	miRNA	miR-423-5p
t0086314	25	1	TGAGGGGCAGAGAGCGAGCCTTAGA	miRNA	miR-423-5p
t0086575	23	1	TTAGGGGAAGAGAGCGAGACTTT	miRNA	miR-423-5p
t0086632	21	1	TGAGGGGCAGGAAGCGAGACT	miRNA	miR-423-5p
t0086671	23	1	TGAGGGGCAGATAGGGAGACTTT	miRNA	miR-423-5p
t0086860	23	1	TGAGGGGCAGACAGCGAGACTTG	miRNA	miR-423-5p
t0086904	22	1	TGAGGGGCAGATAGCGAGAATT	miRNA	miR-423-5p
t0087017	23	1	TGAGGTGCAGAGAGCGAGTCTTT	miRNA	miR-423-5p
t0087043	23	1	TGAGGGGCAGAGGGCGAGACTCT	miRNA	miR-423-5p
t0087049	21	1	TGAGGGGCTGAGAGCGAGCCT	miRNA	miR-423-5p
t0087142	21	1	TGAGGGGCAGATAGCGCGACT	miRNA	miR-423-5p
t0087150	24	1	TGCGGGGCAGAGAGCGAGACTTTT	miRNA	miR-423-5p
t0087210	21	1	TAGGGGCAGGGAGCGAGACTT	miRNA	miR-423-5p
t0087516	22	1	TGAGGGGCAGACAGCGAGCCTT	miRNA	miR-423-5p
t0087629	23	1	TGAGCGGCAGAGGGCGAGACTTT	miRNA	miR-423-5p
t0087846	22	1	TGAGGGGCAGAGAGCGAGAATA	miRNA	miR-423-5p
t0087854	21	1	CGAGGGGCAGAGAGCGAGCCT	miRNA	miR-423-5p
t0087915	23	1	TGACGGGCAGAGGGCGAGACTTT	miRNA	miR-423-5p
t0087965	23	1	TGAGGCGCAGAGAGCGAGACTTA	miRNA	miR-423-5p
t0087985	21	1	AGGGGCAGAGAGAGAGACTTT	miRNA	miR-423-5p
t0088118	22	1	TGAGGGGCAGAGAGAGAGCCTT	miRNA	miR-423-5p
t0088207	23	1	TGAGGGGCAGAGACCGGGACTTT	miRNA	miR-423-5p
t0088217	23	1	TGATGGGAAGAGAGCGAGACTTT	miRNA	miR-423-5p
t0088232	23	1	TGATGGGCAGAGAGCGAGCCTTT	miRNA	miR-423-5p
t0088244	23	1	TGAGGGTCAGGGAGCGAGACTTT	miRNA	miR-423-5p
t0088374	19	1	TGAGGGGCAGAGCGCGATA	miRNA	miR-423-5p
t0088821	22	1	TGAGGGGCAGACAGCGGGACTT	miRNA	miR-423-5p
t0088850	22	1	TGAGGGGCAGAGAGCGGGATTT	miRNA	miR-423-5p
t0088891	23	1	TGAGGGGCAGAGCGAGAGACTTT	miRNA	miR-423-5p
t0089309	23	1	CGAGGGGCAGAAAGCGAGACTTT	miRNA	miR-423-5p
t0089374	42	1	TGAGGGGCAGAGAGCGCGAGTTTTCGTA	miRNA	miR-423-5p
t0089440	23	1	TGAGGGGCAGAGAGCGAGAATTG	miRNA	miR-423-5p
t0089627	23	1	TGAGGGGCCGAGAGCGAGAATTT	miRNA	miR-423-5p
t0089784	21	1	TTGAGGGGCAGAGAGCAAGAC	miRNA	miR-423-5p
t0089890	23	1	TAAGGGGCAGAGAGCGAGACTTG	miRNA	miR-423-5p
t0090219	23	1	TGAGGGGTAGAGAGCTAGACTTT	miRNA	miR-423-5p
t0090336	23	1	TGAGGGGCAGAGAGTGAGACTTC	miRNA	miR-423-5p
t0090368	24	1	TGAGGGGCAGAGGGCGAGACTTAA	miRNA	miR-423-5p
t0090378	21	1	TGAGGGGCAGAGATCGAGAAT	miRNA	miR-423-5p
t0090473	22	1	TGAGGGGCAGAGAACGAGATTT	miRNA	miR-423-5p
t0090513	22	1	TGAGAGCCAGAGAGCGAGACTT	miRNA	miR-423-5p
t0090622	24	1	TGAGGGGCAGAGAGCGAGACTTGG	miRNA	miR-423-5p
t0090640	23	1	TTAGGGGCAGAGAGCGAGACTTA	miRNA	miR-423-5p
t0090644	23	1	TGAGGGGAAGAGAGCGAGATTTT	miRNA	miR-423-5p
t0090652	21	1	TGAGGGGCAGAGAGATAGACT	miRNA	miR-423-5p
t0090771	24	1	TGAGGGGCAGAGAGAGAGACATTT	miRNA	miR-423-5p
t0090902	21	1	TGAGGGGCAGAGAGCGTGAGT	miRNA	miR-423-5p
t0090914	22	1	TGAGGGGCAGAGAGGGAGAATT	miRNA	miR-423-5p
t0090986	24	1	TGAGGGGCAGACAGCGAGACTTAG	miRNA	miR-423-5p
t0091047	24	1	CGAGGGGCAGAGAGCGAGACTTTA	miRNA	miR-423-5p
t0091254	24	1	TGAGGGGCAGAGAGCGCGACTTAA	miRNA	miR-423-5p
t0091260	23	1	TGAGGGGCAGACAGTGAGACTTT	miRNA	miR-423-5p

t0091307	20	1	TGAGGGGCAGAGAGCGAAAC	miRNA	miR-423-5p
t0091537	23	1	TGAGGGGCAGAGAGCGATACTTA	miRNA	miR-423-5p
t0091679	21	1	TGAGGGGCAGAGGGCGAGATT	miRNA	miR-423-5p
t0091858	24	1	TGAGGGGCAGAGAGCGGGACTTGA	miRNA	miR-423-5p
t0092142	23	1	TGAGGAGCATAGAGCGAGACTTT	miRNA	miR-423-5p
t0092281	26	1	TGAGGGGCAGCGAGCGAGACTTTTCT	miRNA	miR-423-5p
t0092380	21	1	TGAGGGGCAGAGAGTCAGACT	miRNA	miR-423-5p
t0092402	25	1	TGAGGGGCAGAGAGCGGGACTTTAT	miRNA	miR-423-5p
t0092416	22	1	TAAGTGGCAGAGAGCGAGACTT	miRNA	miR-423-5p
t0092452	24	1	TGAGGGGCAAAGAGCGAGACTTTT	miRNA	miR-423-5p
t0092477	23	1	TGAGGGGCAGTGAGAGAGACTTT	miRNA	miR-423-5p
t0092498	21	1	AGCTAGGTCTGAGGCCCTTCA	miRNA	miR-423-3p
t0092641	23	1	AGCTCGGTCTGAGGCCCCTCAGT	miRNA	miR-423-3p
t0092750	22	1	ACTGGACTTAGAGTCAGAAGGC	miRNA	miR-422a
t0092774	28	1	GGGGATGTAGATCAGATGGTAGAGCAGA	miRNA	miR-4175-3p
t0092925	21	1	GGGGATGTAGCTCGAATGGTA	miRNA	miR-4175-3p
t0092941	22	1	GGGGATGTAGCTCATATGGGAG	miRNA	miR-4175-3p
t0093147	28	1	GGGGCTGTAGCTCAGATGGGAGAGAGCT	miRNA	miR-4175-3p
t0093191	21	1	GGGGATGTAGATCATATGGTA	miRNA	miR-4175-3p
t0093212	20	1	GGGGATGTAGCTAAGATGGT	miRNA	miR-4175-3p
t0093229	19	1	GGGGATGTAGCTCCGATGG	miRNA	miR-4175-3p
t0093248	24	1	GGGGATGTAGCTCAGATGGTTGAG	miRNA	miR-4175-3p
t0093413	21	1	GGGGATGCAGCTCAGATGGTA	miRNA	miR-4175-3p
t0093538	19	1	GGGGATGTAGATCAGATGG	miRNA	miR-4175-3p
t0048405	32	1	GGGGCTGTAGCTCAGATGGGAGAGAGCC	miRNA	miR-4175-3p
t0052409	21	1	AGACTGTAGTTGGTGCTAATT	miRNA	miR-4152-5p
t0068231	23	1	CTTCCTCCTCCAGCTCGGCGTTT	miRNA	miR-4123-5p
t0046682	24	1	ACTTCCTCCTACAGCTCGGCATTT	miRNA	miR-4123-5p
t0048296	21	1	ACTTCCTCCTCCAGCTCGGCT	miRNA	miR-4123-5p
t0052489	22	1	GAGTGTTGCTCGGTGAACCCCT	miRNA	miR-409-3p
t0064219	22	1	GAATGTTCCTCGGTGAACCCCT	miRNA	miR-409-3p
t0070896	21	1	TGGAACTCTGATATTCGCAGG	miRNA	miR-4001g-3p
t0071503	24	1	CATGGACTCGCAGGAACAGGTTTT	miRNA	miR-4001b-5p
t0084702	20	1	TGGACTCGCAGGAACAGGTC	miRNA	miR-4001b-5p
t0085626	22	1	ATGGACTCGCAGGAACAGGCTA	miRNA	miR-4001b-5p
t0087614	24	1	ATGGACTCGCAGGAACAGGCATTT	miRNA	miR-4001b-5p
t0092076	22	1	TGGACTCGCAGGAACAGGCCTA	miRNA	miR-4001b-5p
t0093216	22	1	TGGACTCGCAGGAAAAGGCCCT	miRNA	miR-4001b-5p
t0067282	21	1	TGGACTCGCAGGAACAGGCCT	miRNA	miR-4001b-5p
t0046216	24	1	ATGGACTCGCAGGAACAGGCTTTT	miRNA	miR-4001b-5p
t0047970	20	1	TGGACTCGCAGGAACAGGCC	miRNA	miR-4001b-5p
t0088565	25	1	TGGACTCGCAGGAACAGGCATATTT	miRNA	miR-4001b-5p
t0062231	26	1	TGGACTCGCAGGAACAGGCCTATTTT	miRNA	miR-4001b-5p
t0072051	19	1	ATAAGGAGAAAGCAATGTA	miRNA	miR-3964
t0069995	22	1	TCGAATCCCACTCCTGACACCC	miRNA	miR-3963
t0047668	21	1	TTAGGTAGTAGTTTGTACAGT	miRNA	miR-3962
t0055681	22	1	AGAGGTAGTAGGTTGTACAGTT	miRNA	miR-3962
t0057654	23	1	TTAGGTAGTAGATTGTACAGTTT	miRNA	miR-3962
t0066293	20	1	TAAGGTAGTAGTTTGTACAG	miRNA	miR-3962
t0068539	22	1	TAAGGCAGTAGTTTGTACAGTT	miRNA	miR-3962
t0069930	22	1	CGAGGTAGTAGTTTGTGCAGTT	miRNA	miR-3962
t0072844	22	1	CGAGGTAGTAGTTTGTACAGTA	miRNA	miR-3962
t0074934	22	1	AGGTAGTAGTTTGTACAGTTTG	miRNA	miR-3962
t0075052	20	1	TGAGTAGTAGTTTGTACAGT	miRNA	miR-3962
t0084101	22	1	CGAGGTGGTAGTTTGTACAGTT	miRNA	miR-3962
t0089532	21	1	TAAGGTAGTAGATTGTACAGT	miRNA	miR-3962
t0067524	22	1	TTAGGTAGTAGTTTGTATAGTT	miRNA	miR-3962
t0051095	22	1	AGAGGTAGTAGCTTGTACAGTT	miRNA	miR-3962

t0043498	22	1	CGAGGTAGTAGATTGTACAGTT	miRNA	miR-3962
t0046194	22	1	TAAGGTAGTAGATTGTACAGTT	miRNA	miR-3962
t0049893	22	1	AGAGGTAGTAGTTTGCACAGTT	miRNA	miR-3962
t0050542	19	1	AGGTAGTAGTTTATACAGT	miRNA	miR-3962
t0051197	22	1	ACAGCCCGGATCCCAGCCCCCT	miRNA	miR-3940-3p
t0051213	25	1	GGAGAAACCTTGGAGCTTCGGCTAT	miRNA	miR-3928
t0055469	22	1	TGTCCTCTAGGGACTGCAGTCG	miRNA	miR-3909
t0067492	23	1	GAACTGAAGCGGACAAGGGGAAT	miRNA	miR-3896-3p
t0069021	27	1	AGAACTGAAACGGACAAGGGGAATACG	miRNA	miR-3896-3p
t0070320	20	1	AACTGAAACGGATAAGGGGA	miRNA	miR-3896-3p
t0075243	22	1	TCGGGGCGGCGGCGGCGGGGGT	miRNA	miR-3885-5p
t0081763	19	1	GGCGGCGGCGGTGGGGGCG	miRNA	miR-3885-5p
t0082397	28	1	CCGGCGGCGGCGGCGACTCTGGAAGCGA	miRNA	miR-3885-5p
t0083032	26	1	GGGGCCGGCGGCGGCGGCGACCCTGG	miRNA	miR-3885-5p
t0086539	26	1	TCGGGGCGGCGGCGGCGGCGGTGGCG	miRNA	miR-3885-5p
t0089065	21	1	GCGGCGTCGGCGGCGGCGGGG	miRNA	miR-3885-5p
t0093223	21	1	CGGCGGCGGCGGAGGAGGAGA	miRNA	miR-3885-5p
t0051697	25	1	ACCCGGGGGGCCGGCGGCGGCGGCG	miRNA	miR-3885-5p
t0090744	21	1	CGGCGGCGTCGGCGGCGGCGG	miRNA	miR-3885-5p
t0087999	23	1	GGGCGGCGGCGGCGGCGGGGGGG	miRNA	miR-3885-5p
t0057191	21	1	GCGGCGGCGGCGGGGGGGTGG	miRNA	miR-3885-5p
t0075790	26	1	CGGCGGCGGCGGGGGCGGCGGGACCG	miRNA	miR-3885-5p
t0085005	22	1	TCGGGGCGGCGGCGGCGGCGTG	miRNA	miR-3885-5p
t0041092	24	1	GGCGGCAGCAGCGGCGGACCATAA	miRNA	miR-3885-5p
t0042916	19	1	GGGCGGCGGCGGGGGGGGA	miRNA	miR-3885-5p
t0043772	24	1	TCGGGGCGGCGGCGGCGGCGGCGG	miRNA	miR-3885-5p
t0043883	27	1	GGAGGGCGGCGGCGGCGGCGGCGGGGC	miRNA	miR-3885-5p
t0044402	28	1	CGGCGGCGGAGGCGACTCTGGACGCGAC	miRNA	miR-3885-5p
t0045168	20	1	GCGGCGGCGGCGGGGGGGTA	miRNA	miR-3885-5p
t0047513	20	1	CGGCGGCGGCGGCGGCGGTG	miRNA	miR-3885-5p
t0047586	22	1	GGCGGCGGCGGCGGGGGGGTGG	miRNA	miR-3885-5p
t0047933	23	1	TCGGAGGGCGGCGGCGGCGGCGG	miRNA	miR-3885-5p
t0048693	25	1	TCGGGGCGGCGGCGGCGGCGGCGGG	miRNA	miR-3885-5p
t0049365	30	1	GGCCGGCGGCGGCGGCGACTCTGGACGC	miRNA	miR-3885-5p
t0049521	22	1	GGCGGCGGCGGCGGGGGTGGGG	miRNA	miR-3885-5p
t0049533	23	1	CGGCGGCGGCGGCGGTGGCGGCG	miRNA	miR-3885-5p
t0050342	23	1	CGGCGGCGTCGGCGGGGGCGGGA	miRNA	miR-3885-5p
t0052059	23	1	CGGCGGCGTCGGCGGCGGCGGGA	miRNA	miR-3885-5p
t0052061	28	1	CGGCGGCGGCGGCGACTCTGGACGGGAC	miRNA	miR-3885-5p
t0052429	18	1	GGGCGGCGGCGGCGGAGG	miRNA	miR-3885-5p
t0053283	22	1	TCGGGGCGGCGGCGGCCGCGGT	miRNA	miR-3885-5p
t0053563	21	1	GGGCGGCGGCGGGGGGGGAAG	miRNA	miR-3885-5p
t0056394	24	1	CGGCTGAGGCGGCGTCGGTAGAGT	miRNA	miR-3885-5p
t0057778	22	1	GGCGGCGGCGGCGGGGGCGGCG	miRNA	miR-3885-5p
t0059510	19	1	CGGCGGCGGCGGGGGTGGG	miRNA	miR-3885-5p
t0059784	22	1	TCAGGGCGGCGGCGGCGGCGGT	miRNA	miR-3885-5p
t0059936	19	1	CGGCGGCGGCGGCGGGACG	miRNA	miR-3885-5p
t0060423	22	1	AGGGCGGCGGCGGCGGCGGGGG	miRNA	miR-3885-5p
t0061423	18	1	CGGCGGCGGCGGGGGTGT	miRNA	miR-3885-5p
t0062949	25	1	CGGCGGCGGCGACTCTGGACGGGAG	miRNA	miR-3885-5p
t0063196	19	1	CGGCGGCGGCGGGGGGGGG	miRNA	miR-3885-5p
t0063229	23	1	CGGCGGGGGCGGGGGCGGGCGGG	miRNA	miR-3885-5p
t0063750	25	1	CGGCGGCGGCGACTCTGGAAGCGAG	miRNA	miR-3885-5p
t0065002	20	1	GGCGGCGGCGGGGGGGGGGG	miRNA	miR-3885-5p
t0065162	26	1	GGAGGGCGGCGGAGGCGGCGGGGGGG	miRNA	miR-3885-5p
t0066541	27	1	AGGGCGGCGGCGGCGGCGGCGGCGGGG	miRNA	miR-3885-5p
t0068348	22	1	GGCGGCGTCGGCGGCGGCGGGA	miRNA	miR-3885-5p
t0068843	19	1	CGGCGGCGGCGGCGGGGGT	miRNA	miR-3885-5p

t0069959	26	1	GGAGGGCGGCGGCGGCGGCGGGGGTG	miRNA	miR-3885-5p
t0070600	23	1	GGGGCCGGCGGCGGCGACTCTGG	miRNA	miR-3885-5p
t0070970	21	1	GGCGGCGTCGGCGGCGGGGGG	miRNA	miR-3885-5p
t0071655	24	1	CGGCGGCGTCGGCGGCGGCGGGAC	miRNA	miR-3885-5p
t0073242	27	1	ACCCGGGGGGCCGGCGGCGGCGGCGAA	miRNA	miR-3885-5p
t0074120	22	1	TCGGGGCGGCGGCGGAGGCGGA	miRNA	miR-3885-5p
t0074345	18	1	GGCGGCGGCGGGGGGGTG	miRNA	miR-3885-5p
t0075962	23	1	TCGGGGCGGCGGCGGCGGCGGAG	miRNA	miR-3885-5p
t0076026	25	1	GGCGGCGGCGGCGGGGGCGGCGCGA	miRNA	miR-3885-5p
t0078590	27	1	ACCCGGGGGGCCGGCGGCGGCGGGGAC	miRNA	miR-3885-5p
t0079950	18	1	GGCGGCGGCGGGGGTGTG	miRNA	miR-3885-5p
t0080006	25	1	GGCGGCGGGGGCGGTGGCATCATAA	miRNA	miR-3885-5p
t0080406	20	1	ATTCGAGACTTACGTTGCGT	miRNA	miR-3850-3p
t0080528	25	1	TATACAAGGGCAAGCTCTCTGTATC	miRNA	miR-381
t0080758	24	1	TGGTAGACTATGGAACGTAGGCTC	miRNA	miR-379-5p
t0080765	23	1	ACTGGACTTGGAGTCAGAAGTCG	miRNA	miR-378f
t0081649	20	1	ACTGGACTTGGAGGCAGAAG	miRNA	miR-378f
t0081759	23	1	ACTGGACTTGGAGAAAGAAGGCT	miRNA	miR-378f
t0081933	23	1	ACTGGACTTGGAGCCAGAAGGCT	miRNA	miR-378f
t0082058	26	1	ACTGGACTTGGAGGCAGAAGGCTATC	miRNA	miR-378f
t0086106	22	1	CTGGACTTGGAGGCAGAAGGCT	miRNA	miR-378f
t0090554	26	1	ACTGGACTTGGAGTCAGAAGGATATC	miRNA	miR-378f
t0092096	22	1	CTGGACTTGGAGACAGAAGGCC	miRNA	miR-378f
t0092826	23	1	ACTGGACTTGGAGGCAGAAGGCT	miRNA	miR-378f
t0093030	24	1	ACTGGACTTGGAGGCAGAAGGATC	miRNA	miR-378f
t0070190	22	1	ACTGGACTTGGAGTAAGAAGGA	miRNA	miR-378c
t0082579	22	1	ACTGGACTTGGAGCCAGAAGGA	miRNA	miR-378c
t0044704	23	1	ACTGGCCTTGGAGTCAGAAGGAG	miRNA	miR-378c
t0044061	22	1	ACTGGACTGGGCGTCAGAAGGA	miRNA	miR-378c
t0048790	25	1	ACTGGACTTGGAGTTAGCAGGAATC	miRNA	miR-378c
t0053592	25	1	ACTAGACTTGGAGTCAGAAGGAATC	miRNA	miR-378c
t0057499	22	1	ACTGGACTTGGAGTCAGCAGGA	miRNA	miR-378c
t0057877	22	1	ACTGGACTTGGAGACAGAAGGA	miRNA	miR-378c
t0060331	22	1	TTGGGCTTGGAGTCAGAAGGCA	miRNA	miR-378b
t0074019	21	1	CGGCGAGTCGGGGTGTTTGGG	miRNA	miR-3787
t0081718	24	1	AAGTAATTGATGATGATCGTGGAT	miRNA	miR-3781
t0082561	22	1	CTGGACCTGGAGTCAGAAGGCT	miRNA	miR-378
t0083964	25	1	ATTGGACTTGGAGTCAGAAGGCATC	miRNA	miR-378
t0066188	23	1	ACTGGCCTTGGAGTCAGAAGGCG	miRNA	miR-378
t0068729	23	1	ACTGGACTTGGAGTCGGAAGGCA	miRNA	miR-378
t0069719	25	1	ACTGGACTTGGAGTCAGAAGGGATC	miRNA	miR-378
t0073769	20	1	ACTGGACTTGGCGTCAGAAG	miRNA	miR-378
t0078217	27	1	ACTGGACTTGGGGTCAGAAGGAATATC	miRNA	miR-378
t0080655	24	1	ACTGGACTTGGAGACAGAAGGCAT	miRNA	miR-378
t0090891	22	1	CTGGACTTGGACTCAGAAGGCC	miRNA	miR-378
t0091613	23	1	ACTGGACTTGGAGTCAGAAGGGA	miRNA	miR-378
t0062694	21	1	ACTGGACTTGGAGTCGGAAGG	miRNA	miR-378
t0064536	20	1	ACTGGACTCGGAGTCAGAAG	miRNA	miR-378
t0045313	21	1	CTGGACTTGGGGTCAGAAGGC	miRNA	miR-378
t0041458	25	1	ACTGGAATTGGAGTCAGAAGGCATC	miRNA	miR-378
t0042590	24	1	ACTGGACTTGGAGACAGAAGGCAA	miRNA	miR-378
t0044934	22	1	ACTGGACTGGGAGTCAGAAGGC	miRNA	miR-378
t0045894	23	1	ACTGGACTTGGAGTCAGAAGACA	miRNA	miR-378
t0046459	25	1	ACTAGACTTGGAGTCAGAAGGCATC	miRNA	miR-378
t0046781	23	1	ACTGGACTTGGAGTCAGGAGGCA	miRNA	miR-378
t0046924	25	1	ACTGGACTTGGAGTCTGAAGGCATC	miRNA	miR-378
t0047321	23	1	ACTGGACTTGGAGTCAAAAGGCG	miRNA	miR-378
t0047754	22	1	ACTGGACTTGGAGTTAGACGGC	miRNA	miR-378

t0047824	24	1	CTGGACTTGGAGCCAGAAGGCATC	miRNA	miR-378
t0048848	22	1	CTGGCCTTGGAGTCAGAAGGCA	miRNA	miR-378
t0050441	23	1	ATTGGACTTGGAGTCAGAAGGCG	miRNA	miR-378
t0051120	25	1	ACTGGACTTGGAGTCAGAAGACATC	miRNA	miR-378
t0052451	23	1	ACTGGACTTGGCGTCAGAAGGCT	miRNA	miR-378
t0052990	23	1	ACTGGACTTGGCGTCAGAAGGCA	miRNA	miR-378
t0053402	23	1	ACTAGACTTGGAGTCAGAAGGCG	miRNA	miR-378
t0053403	23	1	ACTGGAATTGGAGTCAGAAGGCG	miRNA	miR-378
t0053915	25	1	ACTGGACTTGGAGCCAGAAGGCATC	miRNA	miR-378
t0054337	23	1	ACTGGACTTGGAATCAGAAGGCA	miRNA	miR-378
t0054359	23	1	ACTGGACTTGGAATCAGAAGGCG	miRNA	miR-378
t0055423	23	1	ACAGGACTTGGAGTCAGAAGGCA	miRNA	miR-378
t0056390	25	1	ACTGGACTTCGAGTCAGAAGGCATC	miRNA	miR-378
t0057585	23	1	ACTGGACTTGAAGTCAGAAGGCA	miRNA	miR-378
t0058133	23	1	ACTGGACTTGGAGTCAGCAGGCC	miRNA	miR-378
t0058147	23	1	ACTGGACTCGGAGTCAGAAGGCA	miRNA	miR-378
t0058325	23	1	ACTGGACTCGGAGTCAGAAGGTA	miRNA	miR-378
t0058846	21	1	ACTGGTCTTGGAGTCAGAAGG	miRNA	miR-378
t0059578	25	1	ACTGGACTTGGACTCAGAAGGCATC	miRNA	miR-378
t0060777	25	1	ACTGGACTTGGGGTCAGAAGGCATC	miRNA	miR-378
t0061105	25	1	ACTGGTCTTGGAGTCAGAAGGCATC	miRNA	miR-378
t0061241	23	1	ACTAGACTTGGAGTCAGAAGGCT	miRNA	miR-378
t0061360	23	1	ACTGGACTTCGAGTCAGAAGGCA	miRNA	miR-378
t0062734	25	1	ACCGGACTTGGAGTCAGAAGGCATC	miRNA	miR-378
t0063409	25	1	ACTGGACTTGGAGTCAGAAGGAATT	miRNA	miR-378
t0063761	22	1	CTGGACTTGGAGTCAGAAGGGA	miRNA	miR-378
t0063879	24	1	ACTGGACTTGGAGTCAGAAGGAAA	miRNA	miR-378
t0064298	23	1	CTGGACTTGGAGACAGAAGGCAA	miRNA	miR-378
t0064869	23	1	ACTGGTCTTGGAGTCAGAAGGCA	miRNA	miR-378
t0064930	21	1	ACTGGACTTGGGGTCAGAAGG	miRNA	miR-378
t0065277	23	1	ACTGGACTTGGAGTCAAAAGGCA	miRNA	miR-378
t0065932	25	1	CCTGGACTTGGAGTCAGAAGGCATC	miRNA	miR-378
t0066654	25	1	ACTGGACTTGGAGTCAGAAGGCGTC	miRNA	miR-378
t0068735	22	1	ACTGGACTTGGAGTCAGAACGC	miRNA	miR-378
t0070169	25	1	ACTGGACTTGGAGTCAGTAGGCATC	miRNA	miR-378
t0070358	23	1	ACTGGACTTGGAGTAAGAAGGCT	miRNA	miR-378
t0071525	22	1	CTGGACTTGGAGTCGGAAGGCA	miRNA	miR-378
t0071990	23	1	ACTGGACTTTGAGTCAGAAGGCT	miRNA	miR-378
t0073080	21	1	ACTGGACTGGGAGTCGGAAGG	miRNA	miR-378
t0074285	23	1	ACTGGACTTGGAGTCCGAAGGCA	miRNA	miR-378
t0074727	27	1	ACTGGACTTGGAGTCAGAAGGAATATC	miRNA	miR-378
t0075402	23	1	ACTGGATTTGGAGTCAGAAGGCA	miRNA	miR-378
t0076249	25	1	ACTGGACTTGGAGTCAGAAGGCCTC	miRNA	miR-378
t0076291	20	1	ACTGGACTTGGAGTCAGCAG	miRNA	miR-378
t0077215	21	1	ACTGGACTTGGAGACAGAAGG	miRNA	miR-378
t0077646	26	1	ACTGGGCTTGGAGTCAGAAGGTAATC	miRNA	miR-378
t0077929	23	1	ACTGGCCTTGGAGTCAGAAGGCA	miRNA	miR-378
t0080145	21	1	ACTGGACTTGGACTCAGAAGG	miRNA	miR-378
t0080794	21	1	ACTAGACTTGGAGTCAGAAGG	miRNA	miR-378
t0081514	22	1	CTGAACTTGGAGTCAGAAGGCA	miRNA	miR-378
t0082269	24	1	ACTGGACTTGGAGTCAGAAAGCAA	miRNA	miR-378
t0082323	21	1	ACTGGACTTGGAGCCAGAAGG	miRNA	miR-378
t0082965	25	1	ACTGGACTTGGAGTCCGAAGGCATC	miRNA	miR-378
t0083497	25	1	TTTGTTCGTTCGGCTCGCGTGGATC	miRNA	miR-375
t0084812	25	1	TTTGTTCGTTCGGCTCGCGTGAATT	miRNA	miR-375
t0085147	22	1	GTATAATACAACCTGCTAAGTG	miRNA	miR-374c
t0085603	21	1	TTATAATACAACCTGCTAAGT	miRNA	miR-374c
t0085909	20	1	ATAATAAAACCTGCTAAGTG	miRNA	miR-374c

t0087954	23	1	TTATAATACAACCTGATAAGGAA	miRNA	miR-374a
t0089419	22	1	TTATAATACAACCTGATAAGCG	miRNA	miR-374a
t0090782	21	1	TTATAATACAACCTGATCAGT	miRNA	miR-374a
t0090834	23	1	TTATAATACAACCTGATAAGTCA	miRNA	miR-374a
t0091095	27	1	ATATAATAAAACCTGCTAAGTGAAAAA	miRNA	miR-374
t0091393	22	1	ATATAATACAACCTGCCAAGTG	miRNA	miR-374
t0091820	22	1	ATATAATACACCCTGCTAAGTG	miRNA	miR-374
t0093116	23	1	ATATAATACAACCTGCTAAGGGA	miRNA	miR-374
t0059005	22	1	ATATAATACAACCTGCTAAATG	miRNA	miR-374
t0064621	21	1	ATATAATACAACCTGCTCAGT	miRNA	miR-374
t0057639	21	1	ATATAATACAACCCGCTAAGT	miRNA	miR-374
t0059633	21	1	ATAGAATACAACCTGCTAAGT	miRNA	miR-374
t0067061	22	1	ATGTAATACATCCTGCTAAGTG	miRNA	miR-374
t0084369	22	1	ATATAATACAACCTGCTTAGTG	miRNA	miR-374
t0086117	22	1	ATATAATACAGCCTGCTAAGTG	miRNA	miR-374
t0088042	20	1	ATATAATACAACCTGCTCAG	miRNA	miR-374
t0092626	22	1	ATATAATACGACCTGCTAAGTG	miRNA	miR-374
t0042934	21	1	ATATAATATAACCTGCTAAGT	miRNA	miR-374
t0054003	20	1	ATATAATAAAACCTGCTAAG	miRNA	miR-374
t0054132	21	1	ATATAATACAATCTGCTAAGT	miRNA	miR-374
t0055185	22	1	ATATAATGCAACCTGCTAAGTG	miRNA	miR-374
t0055982	27	1	ATATAATACAACCTGCTACGTGAAAAA	miRNA	miR-374
t0056616	20	1	ATATAATACAACCTGCTAGG	miRNA	miR-374
t0058473	22	1	ATATAATACAACCTGCTAAGAG	miRNA	miR-374
t0059116	22	1	ATATAATTCAACCTGCTAAGTG	miRNA	miR-374
t0059514	22	1	ATATAATACAACCTCCTAAGTG	miRNA	miR-374
t0060826	21	1	ATATAATACAACCTGATAAGT	miRNA	miR-374
t0060834	19	1	TGAGGATATGGCAGGGGAG	miRNA	miR-3679-5p
t0064864	23	1	TGAGGATATGGCAGGGAAGGGGT	miRNA	miR-3679-5p
t0069170	21	1	AGGGACTTTCAGGGGCAGCCG	miRNA	miR-365*
t0069415	20	1	AATTGCACGGTACCCATCTG	miRNA	miR-363
t0070483	20	1	TATTGCACGGTATCCATCTG	miRNA	miR-363
t0075586	22	1	AATTGCAAGGTATCCATCTGTA	miRNA	miR-363
t0076709	22	1	AATTGAACGGTATCCATCTGTA	miRNA	miR-363
t0084997	20	1	AATTGCACGGTATACATCTG	miRNA	miR-363
t0086347	22	1	AATTGCCCGGTATCCATCTGTA	miRNA	miR-363
t0087839	19	1	AATTGCACGGTATCGATCT	miRNA	miR-363
t0089360	20	1	AATTGGACGGTATCCATCTG	miRNA	miR-363
t0090128	20	1	AATTGCACTGTATCCATCTT	miRNA	miR-363
t0092556	21	1	ATTGCACGGTATACATCTGTA	miRNA	miR-363
t0046145	18	1	AATTGCACGGTATCAATA	miRNA	miR-363
t0056535	23	1	AATTGCACGGGATCCATCTGTAA	miRNA	miR-363
t0087800	19	1	AATTGCACGGTATCAATCT	miRNA	miR-363
t0043697	22	1	TATTGCACGGTATCCATCTGTA	miRNA	miR-363
t0045253	19	1	AATTGCACGGTACCCATCT	miRNA	miR-363
t0045575	23	1	ATTGAACGGTATCCATCTGTATT	miRNA	miR-363
t0047559	22	1	AATTGCACGGTATCCATCTGCA	miRNA	miR-363
t0047979	22	1	AATTGCACGGTATCAATCTGTA	miRNA	miR-363
t0049616	23	1	AATTGCACGGTATCCATCTGCAA	miRNA	miR-363
t0052303	20	1	AATTGAACGGTATCCATCTG	miRNA	miR-363
t0056182	22	1	AATTGCACGGTATCTATCTGTA	miRNA	miR-363
t0056926	22	1	AATTGCACGGTATACATCTGTA	miRNA	miR-363
t0057236	20	1	AATTGCACGGTATCCACCTG	miRNA	miR-363
t0060590	23	1	AATTGCACGCTATCCATCTGTAG	miRNA	miR-363
t0061623	21	1	AATTGCACGGTATCAATCTGT	miRNA	miR-363
t0062089	20	1	AATTGCAAGGTATCCATCTA	miRNA	miR-363
t0062887	22	1	AATTGCACCGTATCCATCAGTA	miRNA	miR-363
t0062893	19	1	AATTGCACGGGATCCATCT	miRNA	miR-363

t0064176	23	1	AATTGCACGGTGTCCATCTGTAT	miRNA	miR-363
t0067341	21	1	ATTGCACGGGATCCATCTGTA	miRNA	miR-363
t0068540	19	1	AATTGCACGGAATCCATCT	miRNA	miR-363
t0072450	22	1	AATTGCACGGTATCCATCTGAA	miRNA	miR-363
t0073347	23	1	AATCCTCGGAACCTAGGTGTGAG	miRNA	miR-362-5p
t0074680	23	1	AATCCTTGGAACCTAGATGTGAG	miRNA	miR-362-5p
t0081766	23	1	AATCCTTGGAACCTAGGTGTGCG	miRNA	miR-362-5p
t0086177	23	1	CCGGGGGTGGGGTCGGCGGGGGG	miRNA	miR-3621
t0086422	21	1	CCGGGGGTGGGGTAGGCGGAT	miRNA	miR-3621
t0086745	22	1	GGTGATGTCGGCGGTATAGGCG	miRNA	miR-3621
t0086924	19	1	CCGGGGGGGGGGTCGGCGG	miRNA	miR-3621
t0087697	20	1	CCGGGGGTGGGGGCGGCGGG	miRNA	miR-3621
t0091122	19	1	TCTCTCGGCTCCTCGCGGC	miRNA	miR-3615
t0091285	20	1	TCTCTCGGCCCCTCGCGGCT	miRNA	miR-3615
t0091414	20	1	TCTCTCGGCTCCTCGAGGCT	miRNA	miR-3615
t0092075	21	1	TGTTGTACTTTTTTTTTTTGT	miRNA	miR-3613-5p
t0092638	22	1	ACAAAAAAAAAAGCACAACCCT	miRNA	miR-3613-3p
t0048576	23	1	ACAAAAAAAAAAAGCCCAACCCT	miRNA	miR-3613-3p
t0049173	22	1	ACAAAAAACAAAGCCCAACCCT	miRNA	miR-3613-3p
t0061865	24	1	ACAAAAAAAAAAAAAGCCCAACCCT	miRNA	miR-3613-3p
t0050044	22	1	ACAAAAAAAAAAGCCCACCCCT	miRNA	miR-3613-3p
t0050177	21	1	TGAGGATGGATTGCAAGGAAG	miRNA	miR-3605-5p
t0051563	21	1	TGAGGATGGATAGCAAGGCAG	miRNA	miR-3605-5p
t0053148	21	1	TGAGGATGGATAGCACGGAAG	miRNA	miR-3605-5p
t0065773	23	1	GATGAGGATGGATAGCACGGAAG	miRNA	miR-3605-5p
t0082246	22	1	TGAGGTTGGATAGCAAGGAAGT	miRNA	miR-3605-5p
t0087099	21	1	TGAGGATGGATAGCAGGGAAG	miRNA	miR-3605-5p
t0087175	21	1	TGAGGATGGATAGCAAGGGAG	miRNA	miR-3605-5p
t0091203	22	1	TACTAGACTGAAGCTCCTCGAG	miRNA	miR-3586-3p
t0055247	23	1	CTAGACTGAAGCTCCTCGAGGTA	miRNA	miR-3586-3p
t0062898	26	1	AAATTTGAAGACTGAAGTGGAGAAGG	miRNA	miR-3526
t0063891	24	1	TTGAAGACTGAAGTCGAGAAGGGT	miRNA	miR-3526
t0072857	24	1	TGAAGACTGAAGTGGTGAAGGGTT	miRNA	miR-3526
t0075656	28	1	AACTTTGAAGAATGAAGTGGAGAAGGGT	miRNA	miR-3526
t0050390	27	1	AACTTTGAAGACTGAAGTGGAGAACGG	miRNA	miR-3526
t0067949	24	1	CTTTGAAGACTGAAGTGGCGAAGG	miRNA	miR-3526
t0067972	20	1	AACTTTGAAGAATGAAGTGG	miRNA	miR-3526
t0070375	29	1	AACCTTGAAGACTGAAGTGGAGAAGGGT	miRNA	miR-3526
t0075601	24	1	TGAAGACTGAAGTGGCGAAGGGTT	miRNA	miR-3526
t0076153	28	1	TGAAGACTGAAGTGGAGAAGGGGTTCTT	miRNA	miR-3526
t0082171	27	1	AACTTTGAAGACTGAAGTGGTGAAGGG	miRNA	miR-3526
t0049837	27	1	AACTTCGAAGACTGAAGTGGAGAAGGG	miRNA	miR-3526
t0056327	21	1	TGAAGACTGAAGTGAAGAAGG	miRNA	miR-3526
t0041004	26	1	AGCTTTGAAGACTGAAGTGGAGAAGG	miRNA	miR-3526
t0041205	24	1	CTTTGAAGACTGAAGTGGAGACGG	miRNA	miR-3526
t0042054	22	1	TGAAGACTGAAGTGGAAAAGGG	miRNA	miR-3526
t0042185	21	1	AACTTTGAAGACTGAAGAGGA	miRNA	miR-3526
t0042255	29	1	AACTTTGAAGACTGAAGTGGAGTAGGGTT	miRNA	miR-3526
t0042674	28	1	AACTTTGAAGTCTGAAGTGGCGAAGGGT	miRNA	miR-3526
t0042814	28	1	AACTTTGAAGACTGAAGTGGAGCAGGGT	miRNA	miR-3526
t0042947	29	1	AACTTTGAAGACTGAAGTGGCGAAGGGG	miRNA	miR-3526
t0043174	28	1	AACTTTGAAGACTGAAGTGGAGCAGGGG	miRNA	miR-3526
t0043476	24	1	GAAGACTGAAGTGGAGAAGGGCTT	miRNA	miR-3526
t0043780	23	1	TGAAGACTCAAGTGGAGAAGGGT	miRNA	miR-3526
t0043786	22	1	AACTTTGAAGACTGAAGGGGAG	miRNA	miR-3526
t0044370	27	1	AACTTTGAAGATTGAAGTGGAGAAGGG	miRNA	miR-3526
t0044515	23	1	TGAAGACTGAAGTGGAGCAGGGC	miRNA	miR-3526
t0044777	28	1	AACTTCGAAGACTGAAGTGGCGAAGGGT	miRNA	miR-3526

t0044944	21	1	AACTTTGAAGACTAAAGTGGA	miRNA	miR-3526
t0045068	29	1	AACTTTGAAGACTGAAGTGGAGAATGGT	miRNA	miR-3526
t0045140	23	1	TTGAAGACTCAAGTGGAGAAGGG	miRNA	miR-3526
t0045545	28	1	AACTTCGAAGACTGAAGTGGAGAAGGGT	miRNA	miR-3526
t0045609	24	1	TTTGAAGACTGAAGTGGAGGAGGG	miRNA	miR-3526
t0045703	27	1	ACTTTGAGGACTGAAGTGGAGAAGGGT	miRNA	miR-3526
t0045710	22	1	TTGAAGACTGAGGTGGAGAAGG	miRNA	miR-3526
t0045796	28	1	AACTTTGAAGATTGAAGTGGAGAAGGGT	miRNA	miR-3526
t0045915	22	1	TGAAGACTGAAGTAGAGAAGTG	miRNA	miR-3526
t0046148	26	1	CTTTGAAGACTGAAGTGGAGACGGGT	miRNA	miR-3526
t0046321	22	1	AAGACTGAAGTGGAGACGGGTT	miRNA	miR-3526
t0046648	19	1	AACTTTGAAGACTGAAGGG	miRNA	miR-3526
t0046833	26	1	TTGAAGACTGAAGTGGGGAAGGGTTT	miRNA	miR-3526
t0047087	23	1	GAAGACTGAAGTGGAGACGGGTT	miRNA	miR-3526
t0047139	22	1	GAAGACTGAAGTGGAGAAGGGC	miRNA	miR-3526
t0047781	23	1	GAAGACTGAAGTGGAGAAGGGAT	miRNA	miR-3526
t0047877	23	1	TGAAGACTGAAGAGGAGAAGGGT	miRNA	miR-3526
t0047936	26	1	AACTCTGAAGACTGAAGTGGAGAAGG	miRNA	miR-3526
t0048874	21	1	AACTTCGAAGACTGAAGTGGA	miRNA	miR-3526
t0049296	26	1	AACTTTGAAGACTGGAGTGGAGAAGG	miRNA	miR-3526
t0050046	27	1	AACTTTAAAGACTGAAGTGGAGAAGGG	miRNA	miR-3526
t0050139	21	1	AACTTTGGAGACTGAAGTGGA	miRNA	miR-3526
t0050418	27	1	AACTTTGAAGACTGAAGTGGAGAAGGT	miRNA	miR-3526
t0050599	28	1	AACTTTGAAGACTGAAGTGGAGAGGGGT	miRNA	miR-3526
t0051064	29	1	AACTTTGAAGACCGAAGTGGAGAAGGGT	miRNA	miR-3526
t0051224	29	1	AAATTTGAAGACTGAAGTGGAGAAGGGT	miRNA	miR-3526
t0051547	24	1	TGAAGACTGAAGTGGAGACGGGTT	miRNA	miR-3526
t0051772	25	1	TTGAAGACTGAAGTGGAGAAGGGCT	miRNA	miR-3526
t0052136	27	1	AACTTTGAAGACTGACGTGGAGAAGGG	miRNA	miR-3526
t0052264	23	1	GAAGACTGAAGTGGAGAAGGGCT	miRNA	miR-3526
t0052382	27	1	AACTTTGAAGACTGAAGTGGAGAAGAG	miRNA	miR-3526
t0052646	27	1	AACTTTGAAGACTGAAGTGGGGAAGGG	miRNA	miR-3526
t0052946	26	1	AACTTTGAAGACTGAAGAGGAGAAGG	miRNA	miR-3526
t0053214	27	1	AAATTTGAAGACTGAAGTGGAGAAGGG	miRNA	miR-3526
t0054207	28	1	AACTTTGAAGACTGAAGTAGAGAAGGGT	miRNA	miR-3526
t0054471	23	1	TGAAGACTGAAGTGGAGAGGGGT	miRNA	miR-3526
t0054607	21	1	GAAGACTGAAGTGGAGACGGG	miRNA	miR-3526
t0054776	22	1	GAAGACTGAAGTGGAGACGGGT	miRNA	miR-3526
t0054807	29	1	AACTTTGAAGACTGAAGTGGAGAAGGGC	miRNA	miR-3526
t0054854	25	1	TGAAGACTGAAGCGGAGAAGGGGTT	miRNA	miR-3526
t0055327	22	1	GAAGACTGAAGTGGAGAAGAGT	miRNA	miR-3526
t0056089	25	1	TTGAAGACTGGAGTGGAGAAGGGTT	miRNA	miR-3526
t0056172	27	1	TGAAGACTGAAGTGGAGAAGGGATTCT	miRNA	miR-3526
t0056254	26	1	AACTTTGAAGGCTGAAGTGGAGAAGG	miRNA	miR-3526
t0056410	29	1	AACTTTGAAGACTGAAGTGGAGGAGGGT	miRNA	miR-3526
t0056549	21	1	AACTTTGAAAACTGAAGTGGA	miRNA	miR-3526
t0057079	24	1	TTGAAGACTGAAGTTGAGAACGGA	miRNA	miR-3526
t0057330	24	1	TGAAGACTGAAGTGGAGGAGGGTT	miRNA	miR-3526
t0057413	26	1	ACTTTGAAGACTGAAGTGGAGACGGG	miRNA	miR-3526
t0057607	30	1	AACTTTGAAGACTGAAGCGGAGAAGGGT	miRNA	miR-3526
t0057958	21	1	AACTTTGAAGACTGAAGCGGA	miRNA	miR-3526
t0060004	24	1	TTGAAGACTGAAGTGGTGAAGGGT	miRNA	miR-3526
t0060016	27	1	ACTTTGAAGACTGAAGTAGAGAAGGGT	miRNA	miR-3526
t0060305	25	1	TGAAGACTGAAGAGGAGAAGGGTTT	miRNA	miR-3526
t0061244	20	1	TGAAGACTGAAGTGGAGCAG	miRNA	miR-3526
t0061254	23	1	TGAAGACTGAACTGGAGAAGGGT	miRNA	miR-3526
t0061370	20	1	AACTTTGAAGCCTGAAGTGG	miRNA	miR-3526
t0061681	24	1	TGAAGACTGGAGTGGAGAAGGGTT	miRNA	miR-3526

t0061748	23	1	TGAAGACTGGAGGGGAGAAGGGT	miRNA	miR-3526
t0061796	24	1	TTGAAGACTGACGTGGAGAAGGGT	miRNA	miR-3526
t0062123	28	1	AAGTTTGAAGACTGAAGTGGAGAAGGGT	miRNA	miR-3526
t0062134	21	1	AACTTTGAAGGCTGAAGTGGA	miRNA	miR-3526
t0062369	21	1	AACTTTGAAGAATGAAGTGGA	miRNA	miR-3526
t0062378	24	1	TTGAAGACTGAAGGGGAGAAGGGT	miRNA	miR-3526
t0062579	23	1	TGAAGACTGAAGTGGAGGAGGGT	miRNA	miR-3526
t0063435	28	1	AACTTTGAATACTGAAGTGGAGAAGGGT	miRNA	miR-3526
t0063491	24	1	TTGAAGATTGAAGTGGAGAAGGGG	miRNA	miR-3526
t0063652	24	1	CTTTGAAGACTGAAGTGGGGAAGG	miRNA	miR-3526
t0063709	29	1	GACTTTGAAGACTGAAGTGGCGAAGGGT	miRNA	miR-3526
t0064006	24	1	CTTGAAGACTGAAGTGGAGGAGGG	miRNA	miR-3526
t0064931	30	1	ACTTTGGAGACTGAAGTGGAGAAGGGTT	miRNA	miR-3526
t0064972	28	1	AACTTTGAAGACTGAAGTGGAGAAGGAT	miRNA	miR-3526
t0065075	26	1	TTGAAGACTGAAGAGGAGAAGGGTTT	miRNA	miR-3526
t0065772	22	1	TGAAGACTGGAGTGGAGAAGGG	miRNA	miR-3526
t0065841	29	1	AACTTTGAAGACTGAAGCGGAGAAGGGT	miRNA	miR-3526
t0066201	18	1	AACTTTGAAGAGTGAAGT	miRNA	miR-3526
t0066781	29	1	GACTTTGAAGACTGAAGGGGAGAAGGGT	miRNA	miR-3526
t0067700	24	1	TTGAAGCCTGAAGTGGAGAAGGTT	miRNA	miR-3526
t0068428	24	1	GAAGACTGAAGTGGAGAAGGGATT	miRNA	miR-3526
t0068815	27	1	AACTTTGAAGAATGAAGTGGTGAAGGG	miRNA	miR-3526
t0068866	22	1	TGAAGACTGAAGTGGAGACGTG	miRNA	miR-3526
t0069148	27	1	ACTTTGAAGACTGAGGTGGAGAAGGGT	miRNA	miR-3526
t0069256	28	1	AACTTTGAAGTCTGAAGTGGAGAAGGGT	miRNA	miR-3526
t0069956	26	1	AACTTTGAAGACTGAAGTGGGGAAGG	miRNA	miR-3526
t0070096	20	1	AACTTTGAAGACTGAAGCGG	miRNA	miR-3526
t0070121	27	1	GACTTTGAAGACTGAAGTGGAGAAGGG	miRNA	miR-3526
t0070581	27	1	TGAAGACTGAAGGGGAGAAGGGGTTCT	miRNA	miR-3526
t0070703	22	1	GAGGACTGAAGTGGGGAAAGGG	miRNA	miR-3526
t0070794	24	1	GAAGACTGAAGTGGAGACGGGTTT	miRNA	miR-3526
t0070817	28	1	AACTTTGAAGACTAAAGTGGAGAAGGGT	miRNA	miR-3526
t0071206	28	1	AACTTTGAAAACTGAAGTGGAGAAGGGT	miRNA	miR-3526
t0071381	27	1	AACTTTGAAGACTGAAGTGGAGCAGGG	miRNA	miR-3526
t0071448	26	1	CTTTGAAGACTGAAGTGGAGCAGGGT	miRNA	miR-3526
t0071456	26	1	AACTTGGAAGACTGAAGTGGAGAAGG	miRNA	miR-3526
t0071588	19	1	AACTTTGAAGACTGAAGCG	miRNA	miR-3526
t0072615	25	1	TGAAGACTGAAGTGGAGAAAGGTTT	miRNA	miR-3526
t0073090	29	1	AACTTTGAAGACTAAAGTGGAGAAGGGT	miRNA	miR-3526
t0073206	22	1	TGAAGACTGAAGAGGAGAAGGG	miRNA	miR-3526
t0073234	21	1	AACTTTGAAGACCGAAGTGGA	miRNA	miR-3526
t0073321	25	1	TTGAAGACTGAAGTGGGGAAGGGTT	miRNA	miR-3526
t0073383	24	1	TGAGGACTGAAGTGGAGAAGGGTA	miRNA	miR-3526
t0073549	30	1	ACTTTGAAGACTGAAGTGGAGACGGGTTT	miRNA	miR-3526
t0073906	27	1	ACCTTTGAAGACTGAAGTGGAGAAGGG	miRNA	miR-3526
t0074077	26	1	ACTTTGAAGACTGAAGGGGAGAAGGG	miRNA	miR-3526
t0074413	24	1	TGAAGACTGCAGTGGAGAAGGGTT	miRNA	miR-3526
t0074799	28	1	CACTTTGAAGACTGAAGTGGAGAAGGGT	miRNA	miR-3526
t0075021	23	1	TTGAAGACTGAAGTGGAGAGGGT	miRNA	miR-3526
t0075190	23	1	TTGAAGACTGAAGTGGGGAAGGG	miRNA	miR-3526
t0075286	30	1	ACTTTGAAGACTGAAGTGGAGAAGGGGT	miRNA	miR-3526
t0075343	27	1	TGAAGACCGAAGTGGAGAAGGGTTTCT	miRNA	miR-3526
t0075575	21	1	TGAAGACTGAAGTGGTGAAGG	miRNA	miR-3526
t0076140	27	1	AACTTTGAAGCCTGAAGTGGAGAAGGG	miRNA	miR-3526
t0077096	27	1	AACATTGAAGACTGAAGTGGAGAAGGG	miRNA	miR-3526
t0077793	28	1	AAATTTGAAGACTGAAGTGGAGAAGGGT	miRNA	miR-3526
t0078260	24	1	TGAAGACTGAGGTGGAGAAGGGTT	miRNA	miR-3526
t0078265	29	1	AACTTTGAAGACTGACGTGGAGAAGGGT	miRNA	miR-3526

t0078442	26	1	CTTTGAAGAATGAAGTGGAGAAGGGT	miRNA	miR-3526
t0078571	27	1	TGAAGACTGAAGTTGAGGAGGGTTTCT	miRNA	miR-3526
t0078900	20	1	AACTTTGAAGACCGAAGTGG	miRNA	miR-3526
t0078972	20	1	GAAGACTGAAGTGGAGAGGG	miRNA	miR-3526
t0079165	22	1	TGAAGACTGAAGGGGAGAAGGG	miRNA	miR-3526
t0079351	26	1	AACTTTGAGGACTGAAGTGGAGAAGG	miRNA	miR-3526
t0079452	28	1	AACTTTGAGGACTGAAGTGGAGAAGGGT	miRNA	miR-3526
t0079586	25	1	TTGAAGGCTGAAGTGGAGAAGGGTT	miRNA	miR-3526
t0080050	24	1	CTTTGAAGACTGAAGGGGAGAAGG	miRNA	miR-3526
t0080090	28	1	AACTTTGAAGACTGAAGTGGTGAAGGGT	miRNA	miR-3526
t0080199	27	1	TGAAGACTGAAGTGGAGAAGGGCTTCT	miRNA	miR-3526
t0080395	26	1	AACTTTGGAGACTGAAGTGGAGAAGG	miRNA	miR-3526
t0080624	27	1	AACTTTGAGGACTGAAGTGGAGAAGGG	miRNA	miR-3526
t0081283	24	1	TTGAAGACTGAGGTGGAGAAGGGT	miRNA	miR-3526
t0081818	28	1	AACTTTGAAGACTGAAGTGGAGAACGGT	miRNA	miR-3526
t0082034	22	1	AACTTTGAAGACTGAAATGGAG	miRNA	miR-3526
t0082335	25	1	TTGAATACTGAAGTGGAGAAGGGTT	miRNA	miR-3526
t0082366	26	1	CTTTGAAGACTGAAGTGGAGGAGGGT	miRNA	miR-3526
t0082404	26	1	TTGAAGACCGAAGTGGAGAAGGGTTT	miRNA	miR-3526
t0082604	25	1	TGAAGACTGAAGTGGATAAGGGGTT	miRNA	miR-3526
t0082764	21	1	TGAAGACTAAAGTGGAGAAGG	miRNA	miR-3526
t0082791	28	1	AACTTTGAAGACTGCAGGGGAGAAGGGC	miRNA	miR-3526
t0083509	24	1	TTTGAAGACTGAAGGGGAGAAGGG	miRNA	miR-3526
t0083920	27	1	TGAAGACTGAAGTTGAGAAGGGCTTCT	miRNA	miR-3526
t0084180	29	1	AACTTTGAAGACTGAAGGGGAGAAGGGT	miRNA	miR-3526
t0084471	29	1	AACTTTGAAGACTGAACTGGAGAAGGGT	miRNA	miR-3526
t0084621	23	1	TGAAGACTGAAGTGGAGAACGGT	miRNA	miR-3526
t0084803	24	1	TGAAGACTGAAGTGGAGAGGGGTT	miRNA	miR-3526
t0085104	23	1	TGAAGACTGAAGTGGAGAAAGGT	miRNA	miR-3526
t0085327	26	1	AACTTTGAAGACTGAAGTGGTGAAGG	miRNA	miR-3526
t0085745	23	1	TTGAAGACTGAAGCGGAGAAGGG	miRNA	miR-3526
t0086524	28	1	AACTTTGAAGACTGAAGTGGAGAAGGGC	miRNA	miR-3526
t0086545	26	1	CTTTGAAGACTGAAGTGGGGAAGGGT	miRNA	miR-3526
t0086586	20	1	AACTTTGAAGATTGAAGTGG	miRNA	miR-3526
t0087079	26	1	CTTTGAAGACTGAAGCGGAGAAGGGT	miRNA	miR-3526
t0087132	24	1	TGAAGACTGAAGTGGAGAATGGTT	miRNA	miR-3526
t0087218	21	1	GAAGACTGAAGTGGAGAGGGG	miRNA	miR-3526
t0088060	27	1	AACTTTGCAGACTGAAGTGGAGAAGGG	miRNA	miR-3526
t0088127	25	1	TTGAAGACTGAAGTAGAGAAGGGTT	miRNA	miR-3526
t0088200	27	1	TGAAGACTGAAGTGGAGGAGGGTTTCT	miRNA	miR-3526
t0088250	28	1	AACTTTGAAGACTGAAGGGGAGAAGGGA	miRNA	miR-3526
t0088281	27	1	AACTTTGAAGACCGAAGTGGAGAAGGG	miRNA	miR-3526
t0088506	29	1	AACTCTGAAGACTGAAGTGGAGAAGGGT	miRNA	miR-3526
t0088697	23	1	TGAAGACCGAAGTGGAGAAGGGT	miRNA	miR-3526
t0088716	21	1	TGAAGACTGAAGCGGAGAAGG	miRNA	miR-3526
t0089445	25	1	TGAAGACTGGAGTGGAGAAGGGTTT	miRNA	miR-3526
t0089822	27	1	TGAAGACTGAAGTGGAGAAGGGTTTGT	miRNA	miR-3526
t0089847	26	1	ACTTTGAAGACTGAAGTGGGGAAGGG	miRNA	miR-3526
t0090474	21	1	GTAGGAGGTTGCATAGTAGAG	miRNA	miR-352
t0090524	19	1	AGAGTAGTAGGTTGCATAG	miRNA	miR-352
t0090824	20	1	AGAGTAGTAGGTTGCATAGT	miRNA	miR-352
t0090978	21	1	AGAGGTGTAGGTTGCATAGTT	miRNA	miR-352
t0091125	22	1	AAGAGAATAGGGCATGGGTATT	miRNA	miR-3507
t0091343	21	1	TGACTGTCTGGACTCAGTAGT	miRNA	miR-3505
t0091517	20	1	TGACTGTCTGGACACAGTAG	miRNA	miR-3505
t0091671	23	1	TGGAATTCAGGACGCGCGTTTTT	miRNA	miR-3503
t0092210	24	1	AGTGGAATCCAGGACGCGCGTTAT	miRNA	miR-3503
t0092503	22	1	TGGAATCCAGGACGCGCGTTTT	miRNA	miR-3503

t0092819	22	1	AAGTGGAATCCAGGATGCGCTT	miRNA	miR-3503
t0093119	26	1	GTGGAATCCAGGACGCGCGTTATTCT	miRNA	miR-3503
t0093255	24	1	AAGTGGAATCCAGGACGCGCTTTT	miRNA	miR-3503
t0045141	22	1	AAGTGGAATCCAGGACGCGTTT	miRNA	miR-3503
t0061417	20	1	GTGGAATCCAGGACGCGCGT	miRNA	miR-3503
t0071576	20	1	AAGTGGAATCCAGGACGCGC	miRNA	miR-3503
t0091605	20	1	CAAGTGGAATCAAGGACGCG	miRNA	miR-3503
t0079897	21	1	AGCGGAATCCAGGACGCGCGT	miRNA	miR-3503
t0060747	23	1	AGTGGAATCCAGGACGCGCGTTT	miRNA	miR-3503
t0068016	24	1	GTGGAATCCAGGACGCGCGTTTTT	miRNA	miR-3503
t0041176	26	1	AGTGGAATCCAGGACGCGCGTTTTTT	miRNA	miR-3503
t0041544	26	1	AAGTGGAATCCAGGAAGCGCGTGTTT	miRNA	miR-3503
t0044980	22	1	GTGGAATCTAGGACGCGCGTTT	miRNA	miR-3503
t0050054	26	1	AAGTGGAATCCAGGACGCGCGGGTTT	miRNA	miR-3503
t0052499	21	1	GTGACGATCGTACATGTCTTT	miRNA	miR-3502
t0054103	22	1	AACCTTGTAGTTTCGTTGTGTT	miRNA	miR-3501
t0058259	20	1	AGGAGATCGGTAGTAGATTG	miRNA	miR-3500
t0060541	22	1	AGGAGATTGGTGGTAGATTGTT	miRNA	miR-3500
t0062701	23	1	AGGAGATCGGTGGTAGATTGTAT	miRNA	miR-3500
t0069386	26	1	AGGCAGTGTAGTTAGCTGATTGCAAC	miRNA	miR-34c
t0071829	23	1	TGGATGGTGATGCATGGCCGGTT	miRNA	miR-34a
t0072099	21	1	TGGATGGTGATGCATGGTCGT	miRNA	miR-34a
t0077101	22	1	TTCTGAATCCGTGCTGAGATCT	miRNA	miR-3499
t0082002	22	1	TCTGTATCCGTGCTGAGATTCT	miRNA	miR-3499
t0084880	24	1	CAGCATCCGTGCTGAGATTTCGTC	miRNA	miR-3499
t0087414	22	1	TTCTGAATCCGTGCTGAGATAT	miRNA	miR-3499
t0087970	23	1	TTAGACGGTTTGAAACATGGTTA	miRNA	miR-3498
t0048329	21	1	CGCCCGGCGTCTGGACGTTTT	miRNA	miR-3496
t0088800	23	1	CGGCGTCTGGACGTTTGGTTTTT	miRNA	miR-3496
t0052582	20	1	CGCCCGGCGTCTGGACGTTT	miRNA	miR-3496
t0063963	19	1	TGCTCAAAATCGTTGGCAA	miRNA	miR-3495
t0071987	21	1	TCAGAATAGTTTGCAATGGCT	miRNA	miR-3495
t0043464	23	1	CCAGAATCGTTTGCATTGGCTAT	miRNA	miR-3495
t0061910	23	1	CAGAATCGTTTGCAATGGCGTTT	miRNA	miR-3495
t0082391	22	1	TCAGAATCGTTTGCAATGGCTT	miRNA	miR-3495
t0047179	21	1	AGAATCGTTCGCAATGGCGCT	miRNA	miR-3495
t0065013	20	1	GGCTCAAAATCGTTGGCAAT	miRNA	miR-3495
t0089078	23	1	TCAGAATCGTTCGCAATGGCTAT	miRNA	miR-3495
t0092030	22	1	TCAGAATCGTTCGCAATGGCTT	miRNA	miR-3495
t0083503	20	1	CAGCTCAAAATCGTTGGCAA	miRNA	miR-3495
t0045239	25	1	GAATCGTTTGCAATGGCGCATTTTT	miRNA	miR-3495
t0047075	21	1	TAGAAGATTGCCGCTCGTTCT	miRNA	miR-3494
t0082805	20	1	ATAGAAGACTGCCGCTCGTT	miRNA	miR-3494
t0045057	23	1	AGACTGTTGCTCGTTTTGGATTT	miRNA	miR-3494
t0050277	24	1	TCGATAGAAGACTGCCGCTCTTTT	miRNA	miR-3494
t0050964	23	1	ATAGAAGACTGCCGCTCGTTTTG	miRNA	miR-3494
t0051592	23	1	ATAGAAGACTGCCGCTCGTTTTT	miRNA	miR-3494
t0061962	22	1	TATAGAAGACTGCCGCTCGTTT	miRNA	miR-3494
t0064133	21	1	ATAGAAGACTGCCGCTCGTTA	miRNA	miR-3494
t0072416	25	1	AGATTGCCGCTCGTTCTGGATCTTT	miRNA	miR-3494
t0081380	22	1	ACAGAAGACTGCCGCTCATTTA	miRNA	miR-3494
t0086863	21	1	GATAGAAGACTGCCGCTCGTT	miRNA	miR-3494
t0092568	20	1	TATAGAAGACTGCCGCTCGT	miRNA	miR-3494
t0093082	20	1	GATAGAAGACTGCCGCTCGT	miRNA	miR-3494
t0041646	22	1	TCGATAGAAGACTGCCGCTCCT	miRNA	miR-3494
t0042976	24	1	TCCGTGCTGAGATTTCGTCATTTT	miRNA	miR-3492
t0045151	23	1	TCCGTGCTGAGATTTCGTCATTC	miRNA	miR-3492
t0061003	20	1	TTCGTACTGAGATTTCGTCA	miRNA	miR-3492

t0063930	23	1	TCCGTGCTGAGATTTCGTCGTTT	miRNA	miR-3492
t0065878	23	1	TCCGTGCTGAGATTTCGGCATTA	miRNA	miR-3492
t0073777	22	1	TATGACTGAGCGATTACTGGTT	miRNA	miR-3491
t0076974	24	1	ATGACTGAGCGATTACTGGATTTT	miRNA	miR-3491
t0080261	23	1	TATGACTGAGCGATTACTGGATT	miRNA	miR-3491
t0081774	21	1	ACTGAGCGATTACTGGAGTTT	miRNA	miR-3491
t0082441	23	1	TATGACTGAGCGATTACTGGTTT	miRNA	miR-3491
t0082848	22	1	TCTTTTTGGGTTGTGGGGTTAT	miRNA	miR-3490
t0083127	20	1	GTCTTTTCGGGTTGTGGGGT	miRNA	miR-3490
t0089895	23	1	TTTGGGTTGTGGGATAATAATTT	miRNA	miR-3490
t0046780	20	1	CGTCTTTTCGGGTTGTGGGG	miRNA	miR-3490
t0058410	24	1	CCGTAGCTCAGTTGGTCATCATAA	miRNA	miR-3488
t0074123	20	1	GCTCGGTAGCTCAGTTGGGA	miRNA	miR-3488
t0077161	21	1	CGTCCTCGAACTGTTGTGGCT	miRNA	miR-3487
t0082712	21	1	CGCCCTCGAACTGTTGTGGCT	miRNA	miR-3487
t0047103	22	1	TCCTCGAACTGATGTGGCCATT	miRNA	miR-3487
t0065018	23	1	ACCTCGAACGGTTGTGGCCATTT	miRNA	miR-3487
t0067618	23	1	TCCTCGAACTGTTGTGGCCATAT	miRNA	miR-3487
t0071859	23	1	CCTCGAACTGTTGTGGCCATTCA	miRNA	miR-3487
t0082973	20	1	CTCGAACTGTTGTGGCCATG	miRNA	miR-3487
t0055827	20	1	CCCTCGAACTGTTATGGCCA	miRNA	miR-3487
t0076276	23	1	CCCTCGAACTGTTGTGGCCATCT	miRNA	miR-3487
t0089375	23	1	TCGAACTGTTGTGGCCATGGTCT	miRNA	miR-3487
t0092697	24	1	TCCTCGAACTGTTGTGGCCAATTT	miRNA	miR-3487
t0065015	22	1	CGTCCTCGAACTGTTGTGGCTA	miRNA	miR-3487
t0073011	25	1	CCCTCGAACTGTTGTGGCCATTTTT	miRNA	miR-3487
t0042123	25	1	TCCTCGAACTGTTGTGGCCATTTTT	miRNA	miR-3487
t0043315	22	1	ACCTCGAACTGTTGTGGCCATT	miRNA	miR-3487
t0043417	21	1	CCTCGAACTGTTGTGGCCACT	miRNA	miR-3487
t0044151	20	1	TCCTCGAACGGTTGTGGCCA	miRNA	miR-3487
t0044503	22	1	TCCTCGAACTGTTGTGGCCAAT	miRNA	miR-3487
t0044530	24	1	CACGAACTGTTGTGGCCATATTCT	miRNA	miR-3487
t0045124	21	1	CTTCGAACTGTTGTGGCCATT	miRNA	miR-3487
t0046079	24	1	CCTCGAAATGTTGTGGCCATTTTT	miRNA	miR-3487
t0046479	23	1	CTCGAACTGTTGTGGCCATGTTT	miRNA	miR-3487
t0047409	21	1	AGCCCTCGAACTGTTGTGGCA	miRNA	miR-3487
t0048168	26	1	CCCTCGAACTGTTGTGGCCATTTTTT	miRNA	miR-3487
t0049492	22	1	CCTCGAACTGTTGTGGCCATTT	miRNA	miR-3487
t0049651	21	1	TCCTCGAACTGTTGTGGCCTT	miRNA	miR-3487
t0051240	24	1	CTCGAACTGTTGTGGCCATTTTTT	miRNA	miR-3487
t0051392	23	1	CCCTCGAACTGTTGGGGCCATTT	miRNA	miR-3487
t0054262	23	1	TCCTCGAACTGTTGTGGCCATCT	miRNA	miR-3487
t0054767	21	1	CGTCCTCGAACTGTTGTGGCA	miRNA	miR-3487
t0056430	22	1	CGCCCTCGAACTGTTGTGGCTT	miRNA	miR-3487
t0058572	25	1	CCTCGAACTGTTATGGCCATTTTTT	miRNA	miR-3487
t0059701	24	1	CCCTCGAACTGTTGTGGCCATTTT	miRNA	miR-3487
t0061392	23	1	CGTCCTCGAACTGTTGTGGCTAT	miRNA	miR-3487
t0061589	24	1	CCACGAACTGTTGTGGCCACTTTA	miRNA	miR-3487
t0062685	22	1	TCTCGAACTGTTGTGGCCATTT	miRNA	miR-3487
t0065176	20	1	TCCTCGAACTGTTGGGGCCA	miRNA	miR-3487
t0065919	19	1	CTCGAACTGTTGTGGCCAT	miRNA	miR-3487
t0069198	19	1	CACGAACTGTTGTGGCCAC	miRNA	miR-3487
t0073067	22	1	CGTCCTCGAACTGTTGTGGCCT	miRNA	miR-3487
t0074363	21	1	CCCTCGAACTGTTGTGGCCAA	miRNA	miR-3487
t0077485	21	1	ATGACAAAAGGACGCGTTTAT	miRNA	miR-3485-3p
t0078168	20	1	TTTAATGACGAAAGGACGCG	miRNA	miR-3485-3p
t0078254	23	1	TTAATGACAAAAGGACGCGTTTT	miRNA	miR-3485-3p
t0080622	22	1	TTTAATGACGAAAGGACGCGCA	miRNA	miR-3485-3p

t0080993	22	1	ATGCCTGACAACCGTCTACCTT	miRNA	miR-3483-3p
t0081053	23	1	CGGTGATCTTTGTATGGACATTT	miRNA	miR-3481-3p
t0081349	22	1	TATTGCACTTACCTTCGCCTTG	miRNA	miR-3479-3p
t0081988	23	1	TCTCACACGGAAATCGCACCCGT	miRNA	miR-342-3p
t0085441	23	1	TCTCACACAGAAATCGGACCCGT	miRNA	miR-342-3p
t0086941	25	1	TCTCACACAGAAATCGGACCCGTCA	miRNA	miR-342-3p
t0086957	23	1	TCTCACACAGAAACCGCACCCGT	miRNA	miR-342-3p
t0091435	23	1	TCTCACAGAGAAATCGCACCCGT	miRNA	miR-342-3p
t0091573	22	1	TCTCACACAGAAATCGAAACCG	miRNA	miR-342-3p
t0044761	25	1	TCTCACACAGAAATCGCACACGTCA	miRNA	miR-342-3p
t0049703	22	1	TCACACAGAAATCGCAACCGTC	miRNA	miR-342-3p
t0054383	22	1	TTTCACACAGAAATCGCACCCG	miRNA	miR-342-3p
t0072049	24	1	TCTCACACAGAAATCGCACACGTC	miRNA	miR-342-3p
t0065494	23	1	TCTCAAACAGAAATCGCACCCGT	miRNA	miR-342-3p
t0049584	24	1	CCTCACACAGAAATCGCACCCGTC	miRNA	miR-342-3p
t0077862	22	1	TCTCACACAGAAATCGTACCCG	miRNA	miR-342-3p
t0042608	22	1	TCTCACACAGAAATCCCATCCG	miRNA	miR-342-3p
t0042850	25	1	TCTCACACAGAAATCGCACCCGCCA	miRNA	miR-342-3p
t0043069	24	1	TCTCGCACAGAAATCGCACCCGTA	miRNA	miR-342-3p
t0043607	22	1	TCTTACACAGAAATCGCATCCG	miRNA	miR-342-3p
t0044913	24	1	TCACACACAGAAATCGCACCCGTC	miRNA	miR-342-3p
t0046013	24	1	TCTCACACAGAAAACGCACCCGTC	miRNA	miR-342-3p
t0047080	25	1	TCTCTCACAGAAATCGCACCCGTCA	miRNA	miR-342-3p
t0047163	22	1	TCTCACACAGAAATCGCGCCCG	miRNA	miR-342-3p
t0047408	25	1	TCTCACATAGAAATCGCACCCGTCT	miRNA	miR-342-3p
t0048331	23	1	TCTCACACAGAAATCGCGCCCGT	miRNA	miR-342-3p
t0051102	23	1	TCTCATACAGAAATCGCACCCGT	miRNA	miR-342-3p
t0051756	22	1	TCTCACACAGAAATCGCACCCA	miRNA	miR-342-3p
t0052313	22	1	GCTCACACAGAAATCGCACCCG	miRNA	miR-342-3p
t0052627	24	1	TCCCACACAGAAATCGCACCCGTC	miRNA	miR-342-3p
t0054461	25	1	TCTCACTCAGAAATCGCACCCGTCT	miRNA	miR-342-3p
t0054801	21	1	TCTCACACAGCAATCGCACCC	miRNA	miR-342-3p
t0056021	24	1	TCTCACACAGAAATCGCACCAGTC	miRNA	miR-342-3p
t0056775	23	1	TCAAACAGAAATCGCACCCGTCA	miRNA	miR-342-3p
t0057560	25	1	TCTCACACAGAGATCGCACCCGTCA	miRNA	miR-342-3p
t0057663	23	1	TCTCACAAAGAAATCGCACCCGA	miRNA	miR-342-3p
t0057729	18	1	TCTCATACAGAAATCGCG	miRNA	miR-342-3p
t0058707	24	1	TCTCAAACAGAAATCGCACCCGTC	miRNA	miR-342-3p
t0059580	22	1	ACTCACACAGAAATCGCACCCG	miRNA	miR-342-3p
t0060125	23	1	TGTCACACAGAAATCGCACCCGT	miRNA	miR-342-3p
t0060302	22	1	TCTCACTCAGAAATCGCACCCG	miRNA	miR-342-3p
t0060326	24	1	TCTCACCCAGAAATCGCACCCGTC	miRNA	miR-342-3p
t0063138	25	1	TCTCACACAGAAATCGCACACGTCT	miRNA	miR-342-3p
t0063854	23	1	TTTCACACAGAAATCGCACCCGT	miRNA	miR-342-3p
t0063863	25	1	TCTCACACAGAAATCGCAACCGTCT	miRNA	miR-342-3p
t0063880	22	1	TCTCACACAGAAATCGCCTCCG	miRNA	miR-342-3p
t0063962	22	1	TCTCACACAGAAATCGGACCCG	miRNA	miR-342-3p
t0063979	23	1	TCTCACACAGAAATCGCACCAGT	miRNA	miR-342-3p
t0064933	24	1	TCTCACAAAGAAATCGCACCCGTT	miRNA	miR-342-3p
t0066669	23	1	TCACACAGAAATCGCAACCGTCA	miRNA	miR-342-3p
t0066972	22	1	TCACACAGAAATCGCACCAGTC	miRNA	miR-342-3p
t0067614	22	1	TCTCACACAGACATCGCAACCG	miRNA	miR-342-3p
t0067830	22	1	TCTCACACAGAAATCGCACTCG	miRNA	miR-342-3p
t0068033	23	1	TCTCACACTGAAATCGCACCCGT	miRNA	miR-342-3p
t0068151	23	1	TATCACACAGAAATCGCACCCGT	miRNA	miR-342-3p
t0068309	24	1	TCACACAGAAATCGAACCCGTCAT	miRNA	miR-342-3p
t0068559	25	1	TCCCACACAGAAATCGCACCCGTCA	miRNA	miR-342-3p
t0069061	25	1	TCTCACACAGAAATCGCACCTGTCT	miRNA	miR-342-3p

t0069189	25	1	TCTCACACAGAAATCGTACCCGTCT	miRNA	miR-342-3p
t0069880	23	1	TCACACAGAAATAGCACCCGTAA	miRNA	miR-342-3p
t0070011	22	1	TCACACACAGAAATCGCACCCG	miRNA	miR-342-3p
t0071004	24	1	TCTCACACAGAGATCGCACCCGTC	miRNA	miR-342-3p
t0071115	22	1	TCTTACACAGAAATCGCACCCG	miRNA	miR-342-3p
t0072432	22	1	TCTAACACAGAAATCGCACCCG	miRNA	miR-342-3p
t0072671	23	1	TCTCACGCAGAAATCGCACCCGA	miRNA	miR-342-3p
t0072840	23	1	TCTCACACAGAAATCGCAACCGT	miRNA	miR-342-3p
t0073128	24	1	TCTCACACAGATATCGCACCCGTC	miRNA	miR-342-3p
t0073164	22	1	TCACACAGAAATCGCACTCGTC	miRNA	miR-342-3p
t0074562	22	1	TCTCACACAGAAATGGCACCCG	miRNA	miR-342-3p
t0074899	25	1	TCTCACACAGAAATCGCATCCATCA	miRNA	miR-342-3p
t0075235	23	1	TCTCACACAGAAATCTCACCCGT	miRNA	miR-342-3p
t0075750	23	1	TCACACAGAAATCGCACCCGACA	miRNA	miR-342-3p
t0076747	24	1	TCTCACACAGAAATCGAACCCGTT	miRNA	miR-342-3p
t0077218	23	1	TCTCACACAGAAATCGAACCCGA	miRNA	miR-342-3p
t0077248	25	1	TCTCACACAGAAATCGCACCCATCT	miRNA	miR-342-3p
t0078277	25	1	TCTCACACAGAAATCGCACCAGTCT	miRNA	miR-342-3p
t0079572	23	1	TCTCACACAGAAATCGTACCCGT	miRNA	miR-342-3p
t0080038	23	1	TCTCACACAGAAATCGCAGCCGT	miRNA	miR-342-3p
t0080133	23	1	TCTCTCACAGAAATCGCACCCGT	miRNA	miR-342-3p
t0080676	24	1	TCTCACATAGAAATCGCACCCGTC	miRNA	miR-342-3p
t0081189	25	1	TCTCACACAGAAATCGCACCCGACA	miRNA	miR-342-3p
t0081282	23	1	TCACACAGAAATAGCACCCGTCA	miRNA	miR-342-3p
t0081722	22	1	TCTCACACAGCAATCGCACCCG	miRNA	miR-342-3p
t0082122	22	1	TCTCACAGAGAAATCGCACCCG	miRNA	miR-342-3p
t0082541	22	1	TCTCACACGGAAATCGCACCCG	miRNA	miR-342-3p
t0082649	22	1	TCTCACACAGAAAACGCACCCG	miRNA	miR-342-3p
t0082967	23	1	TCTCACACAGAAATCGAAACCGT	miRNA	miR-342-3p
t0083081	23	1	TCACACAGGAATCGCACCCGTCA	miRNA	miR-342-3p
t0084145	22	1	TCTCACACAGAAAGCGCACCCG	miRNA	miR-342-3p
t0084367	23	1	ACTCACACAGAAATCGTACCCGT	miRNA	miR-342-3p
t0084876	25	1	TCTCACACAGAAATCACACCCGTCT	miRNA	miR-342-3p
t0085203	24	1	TCTCACAAAGAAATCGCACCCGTC	miRNA	miR-342-3p
t0085672	24	1	TCTCACACAGGAATCGCACCCGTC	miRNA	miR-342-3p
t0086166	25	1	TCTCACCCAGAAATCGCACCCGTCA	miRNA	miR-342-3p
t0086288	23	1	TCTCACACAGAAAACGCACCCGT	miRNA	miR-342-3p
t0086434	22	1	TTATAAAGCAATGAGACTGACT	miRNA	miR-340-5p
t0088223	22	1	TTATGAAGCAATGAGACTGATT	miRNA	miR-340-5p
t0089539	21	1	TTATAAAGCAGTGAGACTGAT	miRNA	miR-340-5p
t0089583	21	1	TTATAAAGCAATGAGACTGAC	miRNA	miR-340-5p
t0090341	21	1	TTATAAAGCAATGAGACTGGT	miRNA	miR-340-5p
t0091169	19	1	TTATAAAGCAGTGAGACTG	miRNA	miR-340-5p
t0091478	20	1	TTATAAAGCAATGAGAGTGA	miRNA	miR-340-5p
t0092022	22	1	TTAAAAAGCAATGAGACTGATT	miRNA	miR-340-5p
t0092043	22	1	TTATAAAGCAATGGGACTGATT	miRNA	miR-340-5p
t0092102	20	1	TTACAAAGCAATGAGACTGA	miRNA	miR-340-5p
t0092237	20	1	ATAAAGCAATGAGACTGCTT	miRNA	miR-340-5p
t0092694	20	1	TTATAAAGCAATGAGCCTGA	miRNA	miR-340-5p
t0093419	21	1	TTATAAAGCAATGACACTGAT	miRNA	miR-340-5p
t0041711	21	1	TTATAAAGCGATGAGACTGAT	miRNA	miR-340-5p
t0044444	22	1	TTATAAAGCAATGAGACTAATT	miRNA	miR-340-5p
t0044862	20	1	ATAAAGCAATGAGATTGATT	miRNA	miR-340-5p
t0046622	22	1	TTATAAAGCAATGAGACCGATT	miRNA	miR-340-5p
t0048778	21	1	TTATAAAGCAATAAGACTGAT	miRNA	miR-340-5p
t0049403	22	1	TTATAAAGCAATGAGAATGATT	miRNA	miR-340-5p
t0049411	20	1	TTATAAAGCGATGAGACTGA	miRNA	miR-340-5p
t0049515	21	1	TTATAAAGCAATGGGACTGAT	miRNA	miR-340-5p

t0051604	22	1	GATTATAAAGCAATGAGATTGA	miRNA	miR-340-5p
t0051716	22	1	TTACAAAGCAATGAGACTGATT	miRNA	miR-340-5p
t0051810	19	1	CTATAAAGCAATGAGACTG	miRNA	miR-340-5p
t0054606	20	1	TCATAAAGCAATGAGACTGA	miRNA	miR-340-5p
t0059199	20	1	TTATAAAGCAATGAGATTGA	miRNA	miR-340-5p
t0062330	22	1	TTATAAAGCAATGAGATTGATA	miRNA	miR-340-5p
t0062478	21	1	TTATAAAGCAATGAGACTGCT	miRNA	miR-340-5p
t0065453	21	1	TTACAAAGCAATGAGACTGAT	miRNA	miR-340-5p
t0066703	22	1	TTATAAAGCAATGAGACGGATT	miRNA	miR-340-5p
t0071508	21	1	TCATAAAGCAATGAGACTGAT	miRNA	miR-340-5p
t0072757	22	1	TTGTAAAGCAATGAGACTGATT	miRNA	miR-340-5p
t0072793	19	1	TCCCTGTCCTCAAGGAGCT	miRNA	miR-339b
t0074101	19	1	TCCCTGTTCTCCAGGAGCT	miRNA	miR-339b
t0074609	21	1	TGAGCGCCTCGACGACAGAGC	miRNA	miR-339-3p
t0077061	20	1	TGAGCGCCTCGACGACAGAG	miRNA	miR-339-3p
t0079765	21	1	TGAGAGCCTAGACGACAGAGA	miRNA	miR-339
t0082057	20	1	TGAGAGCCTCGACGACAGAG	miRNA	miR-339
t0083029	21	1	GCCCCTGGGCCTATCCTAGCA	miRNA	miR-331
t0085381	22	1	GCCCCTGGGCCTATCCTAGAAT	miRNA	miR-331
t0086060	22	1	GCCCCTGGGACTATCCTAGAAA	miRNA	miR-331
t0086120	21	1	GCCCCTGGGCCTATCCTAGAC	miRNA	miR-331
t0087572	23	1	GCAAATCACACGGCCTGCAGAGA	miRNA	miR-330-3p
t0090197	23	1	GCAAAGCACACGGCCTGCAGCGT	miRNA	miR-330-3p
t0090301	23	1	GCCAAGCACACGGCCTGCAGAGA	miRNA	miR-330-3p
t0065650	22	1	GCAAAGCACACGGCCTGCAGCG	miRNA	miR-330-3p
t0079508	23	1	GCAAAGCACACGGCCTGCAAAGA	miRNA	miR-330-3p
t0077431	23	1	GCAAAGCACCCGGCCTGCAGAGA	miRNA	miR-330-3p
t0089961	19	1	TCTCTGGGACTGTGTCTTA	miRNA	miR-330
t0044991	20	1	GCATCCCCTAGGGCATTGGT	miRNA	miR-324-5p
t0048401	22	1	CGCATCCCCTAGGGCATTGGGG	miRNA	miR-324-5p
t0050188	21	1	CGCATCCCCTAGGGCTTTGGT	miRNA	miR-324-5p
t0050635	22	1	CGCATCCCCTAGAGCATTGGTG	miRNA	miR-324-5p
t0066844	21	1	ACTGCCCCAGGTGCCGCTGGT	miRNA	miR-324-3p
t0085896	24	1	AAACATGAAGCGCTGCAACACATC	miRNA	miR-322*
t0054803	20	1	AAAGACTGAAGTGGAGAAGG	miRNA	miR-320e
t0062393	24	1	TAAAGACTGAAGTGGAGAAGGGTT	miRNA	miR-320e
t0063383	23	1	TAAAGACTGAAGTGGAGAAGGGT	miRNA	miR-320e
t0069977	25	1	TAAAGACTGAAGTGGAGAAGGGTTT	miRNA	miR-320e
t0077832	23	1	AAAAGCTGGGTTGAGCGGGAGAA	miRNA	miR-320d
t0080607	23	1	ATAAGCTGGGTTGAGAGGGCGTT	miRNA	miR-320d
t0083730	22	1	AAAGCTGGGTTGAGAGGGCGCT	miRNA	miR-320d
t0050513	23	1	AAACGCTGGGTTGAGAGGGGGAT	miRNA	miR-320d
t0080953	23	1	GAAAGCTGGGTTGAGAGGGCGGT	miRNA	miR-320d
t0083054	22	1	AAAAGCTGGGTTAAGAGGGCAT	miRNA	miR-320d
t0085103	24	1	AAAAGCTGGGTTGAGAGGGGGGAT	miRNA	miR-320d
t0057679	23	1	AAAAGCTGGGTTGAGAAGGCGTA	miRNA	miR-320d
t0088030	23	1	AAAAGCTGGGTTGAGAGGGCGGC	miRNA	miR-320d
t0052400	22	1	AAAAGCTGGGTTGAGAGGCGAG	miRNA	miR-320d
t0054617	24	1	ATAAGCTGGGTTGAGAGGGCGTTG	miRNA	miR-320d
t0070344	23	1	GAAAAGCTGGGTTGAGAGGGGGT	miRNA	miR-320d
t0077930	23	1	AAAAGCTGGGTTTAGAGGGCGTA	miRNA	miR-320d
t0041338	23	1	AAAAGCTGCTTTGAGAGGGCGGT	miRNA	miR-320d
t0041521	22	1	AAAGCTGGGTTGGGAGGGCGTA	miRNA	miR-320d
t0041899	24	1	AAAAGCTGGGTTGAGAGGACGAAA	miRNA	miR-320d
t0042519	25	1	AAAAGCTGGGTTGAGAGGGCGCTAT	miRNA	miR-320d
t0042766	23	1	AAAAGCTGGGTTGAGAGGGCTTT	miRNA	miR-320d
t0042855	22	1	AACAGCTGGGTTGAGAGGGCGG	miRNA	miR-320d
t0043094	23	1	AAAAGCTGGGTCGAGAGGGCGGA	miRNA	miR-320d

t0043998	24	1	AAAAGCTGGGTTGAGAGGGTAAAA	miRNA	miR-320d
t0044331	23	1	AAAGGCTGGGTTGAGAGGGCGGT	miRNA	miR-320d
t0044641	22	1	AAAAGCTGGGTTGAGAGGACAA	miRNA	miR-320d
t0044770	21	1	AAAGCTGGGTTGAGAGGGTGA	miRNA	miR-320d
t0045201	21	1	AAGCTGGGTTGAGAGGGCGTC	miRNA	miR-320d
t0045262	22	1	AAAAGTTGGGTTGAGAGGGTGA	miRNA	miR-320d
t0045302	22	1	AAAAGCTGGGTTGAGAGGAGTT	miRNA	miR-320d
t0045533	23	1	AAAAGCTGGGTTGAGAGGGCACG	miRNA	miR-320d
t0045572	22	1	AAAAGCTGGGTTGCGAGGGAGT	miRNA	miR-320d
t0046045	21	1	AAAGCTGGGTTGAGGGGGCGT	miRNA	miR-320d
t0046124	21	1	AAAGCTGGGATGAGAGGGCGT	miRNA	miR-320d
t0046273	22	1	ACAAGCTGGGTTGAGAGGGGGA	miRNA	miR-320d
t0046367	23	1	AAAAGCTGGGGTGAGAGGGCTTA	miRNA	miR-320d
t0046473	22	1	AAAAGCTTGGTTGAGAGGGAGT	miRNA	miR-320d
t0046532	24	1	AAAAGCTGGGTTGAGAGGGCGCGT	miRNA	miR-320d
t0046715	23	1	AAAAGCTGGGTCGAGAGGGAGAA	miRNA	miR-320d
t0046726	24	1	AAAAGCTGGGCTGAGAGGGCATAA	miRNA	miR-320d
t0047335	23	1	AAAAGCTGGGTTGAGAGGGCTGA	miRNA	miR-320d
t0047337	24	1	GAAAAGCTGGGGTGAGAGGGCGTA	miRNA	miR-320d
t0047475	22	1	GAAAAGTTGGGTTGAGAGGACT	miRNA	miR-320d
t0047583	22	1	AAAAGTTGGGTTGAGAGGCGAA	miRNA	miR-320d
t0047665	22	1	AAAAGCGGGGTTGAGAGGGCAT	miRNA	miR-320d
t0047669	23	1	AAAAGCTGGGTTGAGAGGGGGAG	miRNA	miR-320d
t0047720	22	1	AAAAGCTGGGGTGAGAGGGCAT	miRNA	miR-320d
t0048123	22	1	AAAAGCTGGGTTCAGAGGGCGC	miRNA	miR-320d
t0049023	22	1	AAAAGCTGGGTTAAGAGGGAGT	miRNA	miR-320d
t0049156	23	1	AAAAGCTGGGTTGAGAGGTCGAT	miRNA	miR-320d
t0049170	23	1	AAGAGCTGGGTTAAGAGGGCGTA	miRNA	miR-320d
t0049421	24	1	AAAAGCTGGGTTGAGAGGGTGAAT	miRNA	miR-320d
t0049767	23	1	GAAAAGCTGGGTTGAGAGGGCGC	miRNA	miR-320d
t0049903	24	1	AAAAGCTCGGTTGAGAGGGCGTTT	miRNA	miR-320d
t0049961	22	1	AAAAGCTGGGTTGAAAGGGCAT	miRNA	miR-320d
t0050161	21	1	AAAAGCTGGGGTGAGAGGGTT	miRNA	miR-320d
t0050339	23	1	GAAAGCTGGGTTGAGAGGGCGCA	miRNA	miR-320d
t0050479	22	1	AGAAGCTGGGTTGAGAGGCGAA	miRNA	miR-320d
t0051261	22	1	AAAGCTGGGGTGAGAGGGCGTA	miRNA	miR-320d
t0051815	22	1	AAAAGATGGGTTGAGAGGGCAT	miRNA	miR-320d
t0051937	23	1	AAAAGCTAGGTTGAGAGGGCGGA	miRNA	miR-320d
t0052044	22	1	AAAAGTTGGGTTGAGTGGGCGT	miRNA	miR-320d
t0052162	23	1	AAAAAACTGGGTTGAGAGGGCTTA	miRNA	miR-320d
t0052282	23	1	AAAAGCTGGGTTGAGAGGGCCGA	miRNA	miR-320d
t0052379	24	1	GAAAAGCTGGGTTGAGAGGGAGTA	miRNA	miR-320d
t0052583	22	1	AAAAACTGGGTTGAGAGGGCGC	miRNA	miR-320d
t0053232	23	1	AAAAACTGGTTTGAGAGGGCGTA	miRNA	miR-320d
t0053294	20	1	AAAGCTAGGTTGAGAGGGCC	miRNA	miR-320d
t0053386	23	1	AAAAGCTGGGTTGCGAGGGCGTG	miRNA	miR-320d
t0053441	21	1	AAAAGCTGGGTTGAGCGGGTA	miRNA	miR-320d
t0053689	23	1	AAAAGCTGGGTTGAAAGGGAGAA	miRNA	miR-320d
t0053851	25	1	AAAAGCTGGGTTGAGAGGGCGCAAG	miRNA	miR-320d
t0054551	23	1	AAAAGCTGCGTTGAGAGGGCGTA	miRNA	miR-320d
t0054845	23	1	AAAAGCTGGGTTCAGAGGGAGAA	miRNA	miR-320d
t0055196	23	1	AAAAGATGGGTTGAGAGGGGGAA	miRNA	miR-320d
t0055265	24	1	AAAAGCTGGGTTGAGAGGGAGAGC	miRNA	miR-320d
t0055505	24	1	AAACGCTGGGTTGAGAGGGCGTTT	miRNA	miR-320d
t0055779	23	1	AAGAGCTGGGTTGAGAGGGCGTT	miRNA	miR-320d
t0055826	24	1	AAAAGCTGGGTTGAGAGGGGGATC	miRNA	miR-320d
t0055991	23	1	AAAAGCTGGCTTGAGAGGGCGTA	miRNA	miR-320d
t0056059	22	1	AAAAGCTGGGTTGAGAGCGCGT	miRNA	miR-320d

t0056391	22	1	AAAAGCCGGGTTGAGAGGGCAT	miRNA	miR-320d
t0056519	23	1	AAAAGCTGGGTTGAGAGGGCTCA	miRNA	miR-320d
t0056791	24	1	AAAAGCTGGGTTGAGAGGGTGTAA	miRNA	miR-320d
t0057085	23	1	AAAAGCTGGGTTGAGAGGGAGTG	miRNA	miR-320d
t0057532	22	1	AAAGCTGGGGTGAGAGGGCGTT	miRNA	miR-320d
t0057911	21	1	AAAAGATGGGTTGAGAGGGCT	miRNA	miR-320d
t0058148	23	1	AAAAGCTGGGTTGAGAGGGAGCT	miRNA	miR-320d
t0058188	23	1	AAAGCTGGGGTGAGAGGGCGTTT	miRNA	miR-320d
t0058463	22	1	AAAAGCTGGGTTGAGAGGGTGC	miRNA	miR-320d
t0058508	21	1	GAAAAGCTGGGTTGAGAGGCA	miRNA	miR-320d
t0058757	22	1	AAAAGGTGGGTTGAGAGGGCGT	miRNA	miR-320d
t0058772	21	1	AAAGATGGGTTGAGAGGGCGT	miRNA	miR-320d
t0059094	22	1	AAAGCTGGGTTGAGAGGGCATA	miRNA	miR-320d
t0060139	22	1	AAAAGCTGGGTTAAGAGGCGTT	miRNA	miR-320d
t0060329	24	1	GAAAAGCTGGGTTGAGAGGGCGCA	miRNA	miR-320d
t0060411	23	1	AAAAGCCGGGTTGAGAGGGCGCA	miRNA	miR-320d
t0060458	25	1	TCTACAAGCTGGGTTGAGAGGGCGT	miRNA	miR-320d
t0060627	23	1	AGAAGCTGGGGTGAGAGGGCGTT	miRNA	miR-320d
t0060671	22	1	AAAGCTGGGTTGAGAGGGCGGT	miRNA	miR-320d
t0060914	24	1	CAAAAGCTGGGTTGGGAGGGCGTA	miRNA	miR-320d
t0061005	22	1	AAAAGCTGGGTTGAGATGGAGA	miRNA	miR-320d
t0061429	23	1	AAAAGATGGGTTGAGAGGGCATA	miRNA	miR-320d
t0061438	23	1	AAAAGCTGGGTTGAGAGCGCGCA	miRNA	miR-320d
t0061459	22	1	AAAAGCTGGGTAGAGAGGGCGT	miRNA	miR-320d
t0061853	22	1	AAAAGCTGGGTTGCGAGGGCTA	miRNA	miR-320d
t0062309	22	1	AAAAGCTGGGTTGAGAGGGCTC	miRNA	miR-320d
t0062810	25	1	TCTAAAAGCTGGGTTGAGAGGGAGT	miRNA	miR-320d
t0063087	23	1	AAAAGCTGGTTTGAGAGGGCGCA	miRNA	miR-320d
t0063485	22	1	AAAGCTGGGCTGAGAGGGGGAA	miRNA	miR-320d
t0063568	22	1	AAGAGCTGGGTTGAGTGGGCGT	miRNA	miR-320d
t0063726	22	1	AAAAGCTGGGTTGCGAGGGCGG	miRNA	miR-320d
t0063772	22	1	AAAGCTGGGTTGAGAGGGAGAT	miRNA	miR-320d
t0063816	22	1	AAAAGCTCGGTTGAGAGGGCGC	miRNA	miR-320d
t0063837	22	1	AAAAGCTGGGTTGGGAGGCGTT	miRNA	miR-320d
t0063881	24	1	AAAAGCTGGGTTGTGAGGGCGTAT	miRNA	miR-320d
t0064454	23	1	AAAAGCTGGGTTGAGAGAGAGAT	miRNA	miR-320d
t0064514	22	1	AAAAGCTAGGTTGAGAGGGCGG	miRNA	miR-320d
t0064623	23	1	AAAAGCTGGGTTGAGAGTGCGTT	miRNA	miR-320d
t0065102	23	1	AAAAGCTGCGTTGAGAGGGCGCT	miRNA	miR-320d
t0065636	23	1	AAAAGCTGGGTTGAGAGGGAAAT	miRNA	miR-320d
t0065667	22	1	AATAGCTGGGTTGAGAGGGCGT	miRNA	miR-320d
t0065907	22	1	AAAAGCTGGGTTGTGAGGGGGA	miRNA	miR-320d
t0066261	23	1	AAAAGCTGGGTTGAGAGGCGAAT	miRNA	miR-320d
t0066715	23	1	AAAAGCTAGGTTGAGAGGGCGCC	miRNA	miR-320d
t0066984	22	1	AAAAGCTGGGTTGAGCGGGCGC	miRNA	miR-320d
t0067001	23	1	AAAAGCTGGGTTGAAAGGGCGTG	miRNA	miR-320d
t0067732	24	1	AAAAGCTGGGTTGAGAGGGAGGAT	miRNA	miR-320d
t0067889	23	1	AAAAGCTGGGTTGAGAGGCCGTA	miRNA	miR-320d
t0067891	22	1	AAAAGCTGGGTTGAGGGGGCGG	miRNA	miR-320d
t0067944	23	1	AAAAGCTGGGTTGAGAGGTCGTT	miRNA	miR-320d
t0067988	22	1	AAAAGCTGGGTTGAGTGGGCGG	miRNA	miR-320d
t0067998	23	1	GAAAAGCTGGGTTGAGAGGGAGA	miRNA	miR-320d
t0068243	23	1	AACAGCTGGGTTGAGAGGGCGGT	miRNA	miR-320d
t0068321	25	1	AAAAGCTGGGTTGAGAGGGAGTTAA	miRNA	miR-320d
t0068453	22	1	AACAGCTGGGTTGAGCGGGCGT	miRNA	miR-320d
t0068691	24	1	AAAAGCTGGGTTGAGAGGGGGAAT	miRNA	miR-320d
t0068822	22	1	AAAAGCTGGGGTGGGAGGGCGT	miRNA	miR-320d
t0068976	23	1	AAAAGATGGGTTGAGAGGGAGAT	miRNA	miR-320d

t0069295	23	1	AAACGCTGGGTTGAGAGGGAGAA	miRNA	miR-320d
t0069454	22	1	AAAGCTGGGTTGAGAGGGCGTC	miRNA	miR-320d
t0069576	23	1	AAAACTGGGTTGAGAGGGCGTTA	miRNA	miR-320d
t0069610	22	1	AAAAGCTGGGTTGAGAGGGATA	miRNA	miR-320d
t0069722	21	1	AAAAGCTGGGTCGAGAGGGAA	miRNA	miR-320d
t0070064	23	1	GAAAGCTGGGTTGAAAGGGCGTA	miRNA	miR-320d
t0070130	24	1	GAAAGCTGGGTTGAGAGGGCGTTA	miRNA	miR-320d
t0070221	21	1	AAAAGCTGGGTTGAGGGGCGC	miRNA	miR-320d
t0070245	22	1	GAAAAGCTGGGTTGAGAGGGGG	miRNA	miR-320d
t0070271	23	1	AAAAGCTGACTTGAGAGGGCGTA	miRNA	miR-320d
t0070295	24	1	AAAAGCTGGGTTGAGAGGGGGATT	miRNA	miR-320d
t0071502	24	1	AAAAGCTGGGGTGAGAGGGGGAAT	miRNA	miR-320d
t0071811	23	1	AAAAGCTGGGTAGAGAGGGCGTA	miRNA	miR-320d
t0072224	22	1	AAAAGCTGGATTGAGAGGGAGA	miRNA	miR-320d
t0072283	23	1	AAAAGCTGGGTTGAGAGGGTGGA	miRNA	miR-320d
t0072607	22	1	AAAAGCTTGGTTGAGAGGGAGA	miRNA	miR-320d
t0072781	22	1	AAAAGCGGGGTTGAGAGGGCGT	miRNA	miR-320d
t0073704	23	1	AAAAGCTGGTTTGAGAGGGCATA	miRNA	miR-320d
t0073964	23	1	AAAAGCTGGGGTGAGAGGGGGTT	miRNA	miR-320d
t0074557	24	1	AAAAGCTGGGTTGAGAGGGTGGTA	miRNA	miR-320d
t0074998	23	1	AAAAGATGGGTTGAGAGGGAGTA	miRNA	miR-320d
t0076382	22	1	AAAAGCTGGGTTGAGATGGGGA	miRNA	miR-320d
t0076676	24	1	AAAAGCTAGGTTGAGAGGGCGGTT	miRNA	miR-320d
t0076807	23	1	AAAAGCTGGCTTGAGAGGGCGGT	miRNA	miR-320d
t0076821	22	1	AAAGCTGGGTTGAGGGGGCGTT	miRNA	miR-320d
t0076892	23	1	AAAGCTGGGTTGAGAGGGCGCAT	miRNA	miR-320d
t0077421	21	1	AAAAGCTGGGTTGAGAAGGAG	miRNA	miR-320d
t0077743	21	1	AAAAGCTGGGTTGAGGGCGTA	miRNA	miR-320d
t0077788	22	1	AAAAGCTGGGATGAGAGGGAGT	miRNA	miR-320d
t0078677	24	1	AAAGCTGGGTTGAGAGGGAGAAAT	miRNA	miR-320d
t0078992	23	1	AAAAGCTGGGTTGAGTGGGCGTA	miRNA	miR-320d
t0079004	24	1	AAAAGCTGGGTTGAGACGGCGTAA	miRNA	miR-320d
t0079181	23	1	AAAAGCTGGGTTGAGAGGGAGGT	miRNA	miR-320d
t0079460	23	1	AAAAGCTGGGTGGAGAGGGCGTA	miRNA	miR-320d
t0080195	22	1	AAAAGCTGTGTTGAGAGGGCGT	miRNA	miR-320d
t0080233	24	1	AAAAGCTGGGTTGAGAGGACGAAT	miRNA	miR-320d
t0080316	22	1	GAAAGCTGGGTTGAGAGGGCGC	miRNA	miR-320d
t0080358	21	1	AAAGCTGGGATGAGAGGGCAT	miRNA	miR-320d
t0080669	22	1	AAAAACTGGGTTGAGAGGGCGG	miRNA	miR-320d
t0081084	23	1	AAAAGCGGGGTTGAGAGGGCGTT	miRNA	miR-320d
t0081368	22	1	AAAGCTGGGGTGAGAGGGCGTC	miRNA	miR-320d
t0081496	22	1	AAAAGCTGGGTTGACAGGGCAT	miRNA	miR-320d
t0081616	23	1	AAAAGATGGTTTGAGAGGGCGTA	miRNA	miR-320d
t0081991	23	1	TAAAGCTGGGTTGAGAGGGAGAT	miRNA	miR-320d
t0082224	22	1	AAAAGCTCGGTTGAGAGGGAGA	miRNA	miR-320d
t0082900	23	1	AAAGGCTAGGTTGAGAGGGCGTA	miRNA	miR-320d
t0083005	20	1	AAAAGCTGGGTTGAGGGCGA	miRNA	miR-320d
t0083233	21	1	AAAAGCTGGGTTGAGAGGCGT	miRNA	miR-320d
t0084094	23	1	AAAAGCTGGGTTGAGAGGACGAT	miRNA	miR-320d
t0084459	23	1	AAAAGCTGGGTTGAGAGGGGATA	miRNA	miR-320d
t0084476	20	1	AAAAGCTGGGTTGAGAGGCA	miRNA	miR-320d
t0084509	22	1	AAAAGCTGGGTTGAGAGGGACT	miRNA	miR-320d
t0084511	23	1	AAAAGATGGGTTGAAAGGGCGTT	miRNA	miR-320d
t0084831	23	1	AAAAGCTGGGTTTAGAGGGCGCT	miRNA	miR-320d
t0085075	22	1	AAAAGCTGGGTTGATAGGGCGT	miRNA	miR-320d
t0085114	22	1	AAAAGCTGGGTTGAGATGGAGT	miRNA	miR-320d
t0085164	23	1	GAAAGCTGGGTTGAGAGGGCATA	miRNA	miR-320d
t0085266	22	1	AAAAGCTGGGTTGGGAGGGCAT	miRNA	miR-320d

t0085625	22	1	AAAAGCTGGTTTGAGAGGGCTA	miRNA	miR-320d
t0085734	23	1	GAAAAGCTGGGTTGAGAGGGCGG	miRNA	miR-320d
t0085797	20	1	AAAGCTGGGTTGAGAAGGAG	miRNA	miR-320d
t0085908	22	1	CAAGCTGGGTTGAGAGGGCGTT	miRNA	miR-320d
t0086068	23	1	AAAAGCTGTGTTGAGAGGGCGTA	miRNA	miR-320d
t0086161	23	1	AAAAGCTGGGTTGAGAGGTCGAA	miRNA	miR-320d
t0086264	21	1	AAAGCTGGGTTGAGAGGCCGA	miRNA	miR-320d
t0086665	22	1	AAAGCTGGGTTGAGAGGGTGTA	miRNA	miR-320d
t0086818	24	1	AAAAGCTGGGTTGAGAGGGCGGCA	miRNA	miR-320d
t0086883	23	1	AAAAGTTGGGTTGAGAGGGAGAC	miRNA	miR-320d
t0087084	24	1	GAAAAGATGGGTTGAGAGGGCGTT	miRNA	miR-320d
t0087109	23	1	AAAAGCTGGGTTGACAGGGCGTT	miRNA	miR-320d
t0087177	24	1	AAAAGCTGGGGTGAGAGGGCGTTT	miRNA	miR-320d
t0087457	23	1	AAAAGCTGTGTTGAGAGGGCGGT	miRNA	miR-320d
t0087565	23	1	AAAAGATGCGTTGAGAGGGCGTT	miRNA	miR-320d
t0087678	23	1	AAAAGCTGGGTTAAGAGGGCGGT	miRNA	miR-320d
t0088804	22	1	AAAAGCTGGGTTGAGAGGCGTA	miRNA	miR-320d
t0088854	21	1	AAGGCTGGGTTGAGAGGGCGT	miRNA	miR-320d
t0089010	22	1	AAAAGCTGGGTTGAGAGGCGCA	miRNA	miR-320d
t0089272	23	1	AAAAGCTGGGTTGAGCGGGCGGT	miRNA	miR-320d
t0089551	23	1	AAAAGATGGGTTGAGAGGGAGAA	miRNA	miR-320d
t0089578	22	1	AAAGCTGGGTTGAGATGGCGTT	miRNA	miR-320d
t0089686	22	1	AAAAGCTGGGTTGAGAGGGGTT	miRNA	miR-320d
t0089712	23	1	AAAAGCTGGTTTGCGAGGGCGTA	miRNA	miR-320d
t0090052	23	1	AAAAGCTGGGTTGAGTGGGCTAA	miRNA	miR-320d
t0090152	24	1	AAAAGCTGGGTTGAGAGGGAGAGA	miRNA	miR-320d
t0090252	23	1	AAAAGCTCGGTTGAGAGGGCGTT	miRNA	miR-320d
t0090460	22	1	AAAAGCTGGGTTGAGATGGCGC	miRNA	miR-320d
t0090944	24	1	AAAAGCTGGGTTGAGAGGGGGAAG	miRNA	miR-320d
t0091102	21	1	AAAGCTGGGTTGAGAGGGAAA	miRNA	miR-320d
t0091309	23	1	AAAAGCTGGGTTGAGAGGGAGGA	miRNA	miR-320d
t0091409	22	1	AAAAGTCGGGTTGAGAGGGCGG	miRNA	miR-320c
t0091485	21	1	AAAATCGGGTTGAGAGGGCGA	miRNA	miR-320c
t0091540	22	1	AAAAGTCGGGTTGAGAGGGCGC	miRNA	miR-320c
t0091847	21	1	GAAAGCCGGGTTGAGAGGGCG	miRNA	miR-320c
t0091865	22	1	AAAAGGCGGGTTGAGAGGGCGA	miRNA	miR-320c
t0092046	22	1	AAAAGTCGGGTCGAGAGGGCGT	miRNA	miR-320c
t0092194	22	1	AAAAGTCGGGTTGAGAGGGAGA	miRNA	miR-320c
t0092299	23	1	AAAAGCTGGGTTCAGAGGGCAAT	miRNA	miR-320b
t0092448	23	1	AAAAGCTGGGTTGAAAGGGCAAA	miRNA	miR-320b
t0092852	23	1	AAAAGCTGGGTTGAGCGGGCAAA	miRNA	miR-320b
t0092859	22	1	AAAGCTGGGCTGAGAGGGCAAA	miRNA	miR-320b
t0093056	21	1	AAAAGCTGGGTTGAGGGGGCA	miRNA	miR-320b
t0093442	22	1	AAAAGCTAGGTTGAGAGGGCAA	miRNA	miR-320b
t0047479	22	1	AAAAGCTGGGTTAAGAGGGCAA	miRNA	miR-320b
t0052113	22	1	AAAAGCTGGGTTGAGAGAGCAA	miRNA	miR-320b
t0066321	22	1	AAAAGATGGGTTGAGAGGGCAA	miRNA	miR-320b
t0079293	24	1	AAAAGCTGGGTTGAGAGGGCAAAT	miRNA	miR-320b
t0080632	22	1	AAAAGCTGGGTAGAGAGGGCAA	miRNA	miR-320b
t0081860	22	1	AAAAGCCGGGTTGAGAGGGCAA	miRNA	miR-320b
t0087195	22	1	AAAAGTTGGGTTGAGAGGGCAA	miRNA	miR-320b
t0042431	23	1	AAAAGCTGGGTTGAGCGGGCAAT	miRNA	miR-320b
t0045727	21	1	AAAAGCTGGGTTGATAGGGCA	miRNA	miR-320b
t0045811	20	1	AAAGATGGGTTGAGAGGGCA	miRNA	miR-320b
t0046260	20	1	AAAGCTGGGTTGAAAGGGCA	miRNA	miR-320b
t0049775	21	1	AAAAGCTGGGTTGCGAGGGCA	miRNA	miR-320b
t0050677	23	1	AAAGGCTGGGTTGAGAGGGCAAT	miRNA	miR-320b
t0050755	21	1	AAAAGCTGGGTTGAGAGAGCA	miRNA	miR-320b

t0050977	23	1	AAAAGTTGGGTTGAGAGGGCAAA	miRNA	miR-320b
t0053013	22	1	AAAAGCTGGGTTGAGGGGGCAA	miRNA	miR-320b
t0057489	22	1	AAAAGCTGGATTGAGAGGGCAA	miRNA	miR-320b
t0058170	22	1	AAAAGCTGGGTCGAGAGGGCAA	miRNA	miR-320b
t0060143	23	1	AAAAGCTGGGTTGAGAGGGCAAC	miRNA	miR-320b
t0066049	22	1	AGAAGCTGGGTTGAGAGGGCAA	miRNA	miR-320b
t0066706	22	1	AAAAGCTGTGTTGAGAGGGCAA	miRNA	miR-320b
t0068861	25	1	AAAAGCTGGGTTGAGAGGGCAATAT	miRNA	miR-320b
t0069240	20	1	AAAAGCTGGGTTGAAAGGGC	miRNA	miR-320a
t0071495	21	1	AAAAGTTGGGGTGAGAGGGCG	miRNA	miR-320a
t0073943	22	1	GAAAAGCTGGGTTGCGAGGGCG	miRNA	miR-320a
t0074515	24	1	AAAAGCTGGGTTGAGCGGGCGATA	miRNA	miR-320a
t0075433	22	1	GAAAAGCTGGGTTGAGCGGGCG	miRNA	miR-320a
t0076920	22	1	AAAAGATGCGTTGAGAGGGCGA	miRNA	miR-320a
t0082980	24	1	AAAAGCTGGATTGAGAGGGCGAGA	miRNA	miR-320a
t0086473	20	1	AAAGCTGCGTTGAGAGGGCG	miRNA	miR-320a
t0087035	24	1	AAAAGCTGGGTTCAGAGGGCGATT	miRNA	miR-320a
t0087977	25	1	AAAAACTGGGTTGAGAGGGCGAAGT	miRNA	miR-320a
t0088522	24	1	AAAAGCTGGGGTGAGAGGGCGAAG	miRNA	miR-320a
t0090476	21	1	GAAGCTGGGTTGAGAGGGCGA	miRNA	miR-320a
t0090624	22	1	GAAAAGCTGGGTTGAGAGAGCG	miRNA	miR-320a
t0040776	24	1	AAAAGCTGGGTTGAAAGGGCGAAA	miRNA	miR-320a
t0041414	23	1	AAAAGCTCGGTTGAGAGGGCGAT	miRNA	miR-320a
t0041627	24	1	AGAAGCTGGGTTGAGAGGGCGAAT	miRNA	miR-320a
t0042013	23	1	AAAAGCTGGTTTAAGAGGGCGAA	miRNA	miR-320a
t0042186	21	1	AGAAGCTGGGTTGAGAGGGCG	miRNA	miR-320a
t0042189	19	1	AACAGGGTTGAGAGGGCGA	miRNA	miR-320a
t0042716	22	1	AAAAGCTGGGGTGATAGGGCGA	miRNA	miR-320a
t0043400	23	1	AAAAACTGGGTTGAGAGGGCGAG	miRNA	miR-320a
t0043770	23	1	AAAATCTGTGTTGAGAGGGCGAA	miRNA	miR-320a
t0044084	22	1	GAAAAGCTAGGTTGAGAGGGCG	miRNA	miR-320a
t0044632	23	1	AAAAGCTGGGTTGAGAAGGCGAT	miRNA	miR-320a
t0045320	25	1	AAAAGCTGGGGTGAGAGGGCGATTT	miRNA	miR-320a
t0045359	21	1	AAAAGCTGGCTTGAGAGGGCG	miRNA	miR-320a
t0045962	23	1	AAAAGCTGGGTTGAGCGGGCGAG	miRNA	miR-320a
t0048498	25	1	CGAAAAACTGGGTTGAGAGGGCGAA	miRNA	miR-320a
t0048555	21	1	AAAATCTGGGTTGAGAGGGCG	miRNA	miR-320a
t0048734	24	1	AAAAGGTGGGTTGAGAGGGCGAAT	miRNA	miR-320a
t0048923	21	1	AAAAGCTGGGTGGAGAGGGCG	miRNA	miR-320a
t0049222	22	1	AAACGATGGGTTGAGAGGGCGA	miRNA	miR-320a
t0050505	24	1	AAAAGCTGGGTTGAGAGAGCGATA	miRNA	miR-320a
t0050819	23	1	GAAAAGCTGGGATGAGAGGGCGA	miRNA	miR-320a
t0051327	23	1	AAAAGCTGGGTTGAGAGCGCGAG	miRNA	miR-320a
t0052016	23	1	AGAAGCTGGGTTGAGAGGGCGAG	miRNA	miR-320a
t0052039	21	1	ATAAGCTGGGTTGAGAGGGCG	miRNA	miR-320a
t0052137	23	1	AAGAGCTGGGTTGAGAGGGCGAG	miRNA	miR-320a
t0052688	21	1	AAAAGCCGGATTGAGAGGGCG	miRNA	miR-320a
t0053524	21	1	CAAAGCTGGGTTGAGAGGGCG	miRNA	miR-320a
t0053892	21	1	AAAGCTAGGTTGAGAGGGCGA	miRNA	miR-320a
t0055442	24	1	CAAAAGCTGGGTTGAGAGGGCGAA	miRNA	miR-320a
t0058449	21	1	AAAGCTGGGTCGAGAGGGCGA	miRNA	miR-320a
t0059554	23	1	AAAAGCTGGGTCGAGATGGCGAA	miRNA	miR-320a
t0059751	23	1	AAAAGTTGGGTTGAGAGGGCGAG	miRNA	miR-320a
t0059864	23	1	AAAAGCTGGGTTGATAGGGCGAT	miRNA	miR-320a
t0059882	20	1	AAAGCTGGGTTGCGAGGGCG	miRNA	miR-320a
t0060363	22	1	AAAGCTGGGTTGAGAAGGCGAT	miRNA	miR-320a
t0061939	23	1	AAAGCTGGGTTGAGAGAGCGATG	miRNA	miR-320a
t0062110	24	1	AAAAGCTGGGTTGGGAGGGCGAAT	miRNA	miR-320a

t0062300	20	1	AAAGCTGGGTTGAGCGGGCG	miRNA	miR-320a
t0062445	23	1	AAAAGCTGGGTGGAGAGGGCGAT	miRNA	miR-320a
t0063245	23	1	AAAAGCTGGGTTGAGGGGGCGAG	miRNA	miR-320a
t0064608	23	1	AAAAGCTGGGTAGAGAGGGCGAA	miRNA	miR-320a
t0064719	21	1	AATAGCTGGGTTGAGATGGCG	miRNA	miR-320a
t0064892	23	1	TAAAGCTGGGTTGAGAGGGCGAA	miRNA	miR-320a
t0065007	24	1	AAAAGCTGGGGTGAGAGGGCGAAT	miRNA	miR-320a
t0066935	21	1	AAAAACTGGGTTGAGATGGCG	miRNA	miR-320a
t0067173	22	1	AAAAGCTCGGTTGAGTGGGCGA	miRNA	miR-320a
t0067498	20	1	AAAACTGGGTTGAGAGGGCG	miRNA	miR-320a
t0067886	23	1	GAAAAGCTGGGTTGAGAGAGCGA	miRNA	miR-320a
t0068526	23	1	AAAAGCTGGGTGGAGAGGGCGAA	miRNA	miR-320a
t0068581	24	1	AAAAGCTGGGTTGAGATGGCGAAT	miRNA	miR-320a
t0068665	21	1	AAAGCTGGATTGAGAGGGCGA	miRNA	miR-320a
t0068722	22	1	AAAAGTAGGGTTGAGAGGGCGA	miRNA	miR-320a
t0068980	23	1	AAAAGCTGGGATGAGAGGGCGAG	miRNA	miR-320a
t0069268	21	1	AAAGCTGGGTTGGGAGGGCGA	miRNA	miR-320a
t0069596	21	1	AAAACCTGGGTTGAGAGGGCG	miRNA	miR-320a
t0069686	21	1	AAGAGCTGGGTTGAGAGGGCG	miRNA	miR-320a
t0070737	22	1	AAAAGCTGGGTCGAGATGGCGA	miRNA	miR-320a
t0071341	20	1	AGAGCTGGGTTGAGAGGGCG	miRNA	miR-320a
t0071404	23	1	AAACGTTGGGTTGAGAGGGCGAC	miRNA	miR-320a
t0072307	20	1	AAAGCTGGGTTGAGAGAGCG	miRNA	miR-320a
t0072902	22	1	AAAGCTGGGTCGAGAGGGCGAT	miRNA	miR-320a
t0072950	22	1	AACAGCTGGGTTGAGATGGCGA	miRNA	miR-320a
t0073477	22	1	AAGAGCTGAGTTGAGAGGGCGA	miRNA	miR-320a
t0073545	22	1	AAAGCTGGGTTGGGAGGGCGAA	miRNA	miR-320a
t0073618	23	1	AAAAGCTGGGTTGGGAGGGCGAC	miRNA	miR-320a
t0074422	21	1	AAAGCTGGGTTGAGGGGGCGA	miRNA	miR-320a
t0074879	24	1	AAAAGCTGGGTCGAGAGGGCGACA	miRNA	miR-320a
t0075015	21	1	AACGCTGGGTTGAGAGGGCGA	miRNA	miR-320a
t0075082	22	1	AAAGGCTGGGTTGAGTGGGCGA	miRNA	miR-320a
t0075253	23	1	GAAAAGCTGGGTTGAGCGGGCGA	miRNA	miR-320a
t0075675	23	1	AAAAAGCTGGGTTGAGAGGGCGA	miRNA	miR-320a
t0076299	24	1	AAAAGATGGGTTGAGAGGGCGAAA	miRNA	miR-320a
t0076702	20	1	TAAGCTGGGTTGAGAGGGCG	miRNA	miR-320a
t0076909	23	1	AAAGGCTGGGGTGAGAGGGCGAA	miRNA	miR-320a
t0077069	23	1	AAAAGCTGGGTTTAGAGGGCGAA	miRNA	miR-320a
t0078104	21	1	AAACGCTGGGTTGAGAGGGCG	miRNA	miR-320a
t0078525	24	1	AAAAGCCGGGTTGAGAGGGCGAAA	miRNA	miR-320a
t0078779	23	1	AAAAGCGGGGTTGAGAGGGCGAA	miRNA	miR-320a
t0078781	21	1	AATAGCTGGGTTGAGAGGGCG	miRNA	miR-320a
t0079324	23	1	AAAAGCAGGGTTGAGAGGGCGAG	miRNA	miR-320a
t0079378	23	1	AAAAGCTGGGTTGAGAGTGCGAA	miRNA	miR-320a
t0080576	24	1	GAAAGCTGGGTTGAGAGGGCGATT	miRNA	miR-320a
t0080818	21	1	AAAACTGGGTTGAGAGGGCGA	miRNA	miR-320a
t0080933	21	1	AAAAGCGGGGTTGAGAGGGCG	miRNA	miR-320a
t0081636	21	1	AAAAGCTGGGTTGACAGGGCG	miRNA	miR-320a
t0081919	26	1	CTGGAAGAGCTGGGTTGAGAGGGCGA	miRNA	miR-320a
t0081921	21	1	AAAAGCTGGGTTGAGTGGGCG	miRNA	miR-320a
t0082332	24	1	AAAAGCTGGGTTGCGAGGGCGAAT	miRNA	miR-320a
t0082673	21	1	AAGCCGGGTTGCGAGGGCGAA	miRNA	miR-320a
t0083773	23	1	AAAAGCTGTGTTGAGAGGGCGAG	miRNA	miR-320a
t0084081	22	1	AAAAGCTAGGTTGAGATGGCGA	miRNA	miR-320a
t0084642	21	1	AAAGCTGGGTTGTGAGGGCGA	miRNA	miR-320a
t0085170	25	1	CCAAAAAGCTGGGTTGAGCGGGCGA	miRNA	miR-320a
t0085261	22	1	AAAACTGGGTTGAGAGGGCGAA	miRNA	miR-320a
t0085414	21	1	AAAGCTGGGGTGAGAGGGCGA	miRNA	miR-320a

t0085742	24	1	GAAAAGCTGGGTTGAGGGGGCGAT	miRNA	miR-320a
t0085945	21	1	AAAGCTGGGTTGAGAGAGCGA	miRNA	miR-320a
t0085950	24	1	AAAAGCTGGGTTGAGAGCGCGAAT	miRNA	miR-320a
t0086030	22	1	AAAAGTTGGGTTGGGAGGGCGA	miRNA	miR-320a
t0086985	24	1	ACAAGCTGGGTTGAGAGGGCGAAT	miRNA	miR-320a
t0087020	23	1	AAAAGCTGGGTCGAGAGGGCGAG	miRNA	miR-320a
t0087393	23	1	AAACGCTGCGTTGAGAGGGCGAT	miRNA	miR-320a
t0088092	23	1	AAAAATTGGGTTGAGAGGGCGAA	miRNA	miR-320a
t0088276	23	1	AAAAGCTTGTTTGAGAGGGCGAA	miRNA	miR-320a
t0088290	24	1	AAGAGCTGGGTTGAGAGGGCGAAT	miRNA	miR-320a
t0088831	23	1	AAAAGCTGGGTTGAGAAGGCGAC	miRNA	miR-320a
t0089182	20	1	AAAGCTGGGTTGGGAGGGCG	miRNA	miR-320a
t0089427	21	1	GAAGGGGGAAGAGCTTTAATG	miRNA	miR-3202
t0089742	23	1	AATCCGAGAAGGCGCACAAGGTT	miRNA	miR-3200-5p
t0089866	23	1	AATCTGAGAAGGCGCACATGGTT	miRNA	miR-3200-5p
t0090345	24	1	AATCTGAGAAGGTGCACAAGGTTT	miRNA	miR-3200-5p
t0091063	22	1	AATCTGAGAAGGCGTACAAGGT	miRNA	miR-3200-5p
t0091112	23	1	AATCTGAGAAGGCGCAGAAGGTT	miRNA	miR-3200-5p
t0091574	23	1	AATCTGAGAAGGCGCCCAAGGTT	miRNA	miR-3200-5p
t0091817	23	1	AATTTGAGAAGGCGCACAAGGTT	miRNA	miR-3200-5p
t0092182	24	1	AATCTGAGAAGGCGCACAAGGATT	miRNA	miR-3200-5p
t0092205	24	1	AATATGAGAAGGCGCACAAGGTTT	miRNA	miR-3200-5p
t0092876	26	1	GAATCTGAGAAGGCGCACAAGGGAGA	miRNA	miR-3200-5p
t0093124	24	1	AATCTGAGAAGGCGCACAAGGGTT	miRNA	miR-3200-5p
t0093477	22	1	AATCTGAGAAGGAGCACAAGGT	miRNA	miR-3200-5p
t0082309	23	1	AATCTGCGAAGGCGCACAAGGGT	miRNA	miR-3200-5p
t0041020	23	1	GAATCTGAGAAGGAGCACAAGGT	miRNA	miR-3200-5p
t0042965	23	1	AATCTGAGAAGGCGAACAAGGTT	miRNA	miR-3200-5p
t0048964	23	1	AATCTGAGAGGGCGCACAAGGTT	miRNA	miR-3200-5p
t0049066	22	1	AATCAGAGAAGGCGCACAAGGT	miRNA	miR-3200-5p
t0053683	25	1	AATCCGAGAAGGCGCACAAGGTTTG	miRNA	miR-3200-5p
t0054357	25	1	AATCTGAGAAGGCGCACCAGGTTTG	miRNA	miR-3200-5p
t0054988	24	1	AATCTGAGAAGGCGCACAAGGAAA	miRNA	miR-3200-5p
t0055638	24	1	CATCTGAAAAGGCGCACAAGGTTT	miRNA	miR-3200-5p
t0056550	23	1	AATCTGAGAAGGCGCTCAAGGTT	miRNA	miR-3200-5p
t0057963	23	1	AATCTGGGAAGGCGCACAAGGTT	miRNA	miR-3200-5p
t0065824	23	1	AATCTGAGAAGGCGCACAAGGCA	miRNA	miR-3200-5p
t0066077	23	1	AATCTGAGATGGCGCACAAGGTT	miRNA	miR-3200-5p
t0067204	22	1	ACCTTGCGCTACTCAGGGCTGT	miRNA	miR-3200-3p
t0073183	21	1	CAACTTGCGCTACTCAGGTCT	miRNA	miR-3200-3p
t0074644	20	1	CACCTTGCGTTACTCAGGTC	miRNA	miR-3200-3p
t0079823	21	1	CACCTTGCGCTACTCGGGTCT	miRNA	miR-3200-3p
t0081021	21	1	CACTTTGCGCTACTCAGGTCT	miRNA	miR-3200-3p
t0081721	20	1	CACCTTGCGCTACTCAGGCC	miRNA	miR-3200-3p
t0082521	22	1	CACCTTGCGATACTCAGGTCTG	miRNA	miR-3200-3p
t0083769	21	1	CACCTTGGGCTACTCAGGTCT	miRNA	miR-3200-3p
t0084339	22	1	GTGGAGTCCTGGGGAATGGAGC	miRNA	miR-3198
t0084534	22	1	CTCCTGCGTAGGATCTGAGGCA	miRNA	miR-3193
t0085071	22	1	CTCCTGCGGAGGATCTGAGGAA	miRNA	miR-3193
t0089279	22	1	CTCCTGCGTAGGCTCTGAGGAA	miRNA	miR-3193
t0090319	22	1	TCCGGGAGGTTGTAGCAGTGGA	miRNA	miR-3192
t0049356	22	1	TCTGGGAGTTTGTAGAAGTGGA	miRNA	miR-3192
t0050186	22	1	TCTGGGAGGTTGTAGCAGTGGC	miRNA	miR-3192
t0056668	22	1	TCTGGGAGGTTGTAGCAGAGGA	miRNA	miR-3192
t0058293	21	1	TCTGGGAGGTTGTAGTAGTGG	miRNA	miR-3192
t0068150	22	1	TCTGGGAGGTTGTAGTAGTGGA	miRNA	miR-3192
t0081758	22	1	TCTGGGAGGTTGTAGCCGTGGA	miRNA	miR-3192
t0083692	21	1	TCTGGGAGATTGTAGCAGTGA	miRNA	miR-3192

t0086999	22	1	TCTGGGAGGTTGTAGCGGTGGA	miRNA	miR-3192
t0065635	22	1	TCTGGGAGGTTGGAGCAGTGGA	miRNA	miR-3192
t0053880	20	1	GTGGAAGGTAGACGGCCCGA	miRNA	miR-3190
t0064035	21	1	TGAAAGGTAGACGGCCAGAGA	miRNA	miR-3190
t0073491	21	1	GTGGAAGGTAGACGGCCAGGG	miRNA	miR-3190
t0043968	20	1	GTGGAAGGGAGACGGCCAGA	miRNA	miR-3190
t0045770	21	1	TGGAAGGGAGACGGCCAGAGA	miRNA	miR-3190
t0046886	24	1	GTGGAAGGTAGACGGCCAGAGCGA	miRNA	miR-3190
t0054144	21	1	TGGAAGGTAGACGGCCAGAGC	miRNA	miR-3190
t0054660	21	1	GTGGAAGGTAGACGGGCAGAG	miRNA	miR-3190
t0058374	20	1	TGGAAGGTAGACGGCCCGAG	miRNA	miR-3190
t0074577	22	1	GTGGAAGGTAGACAGACAGAGA	miRNA	miR-3190
t0077163	20	1	GGGAAGGTAGACGGCCAGAG	miRNA	miR-3190
t0080903	21	1	TGGAAGGAAGACGGCCAGAGA	miRNA	miR-3190
t0092332	23	1	TGGAAGGTAGACGGACAGAGAGA	miRNA	miR-3190
t0041981	20	1	TGGAAAGTAGACGGCCAGAG	miRNA	miR-3190
t0042752	21	1	TGGAAGGTAGACGGGCAGAGA	miRNA	miR-3190
t0044005	21	1	GTGGAAGGTAGACGGACAGAG	miRNA	miR-3190
t0044627	22	1	TGTGGAAGGTAGACGGCCAGAG	miRNA	miR-3190
t0047505	22	1	TGGAAGGTAGACGACCAGAGTT	miRNA	miR-3190
t0050780	21	1	TGGAAGGTAGACAGCCAGAGA	miRNA	miR-3190
t0056905	22	1	TAGAAGGGGGGAAATTTAAACG	miRNA	miR-3179
t0057307	22	1	TAGGAGGGGTGAAATTTAAACG	miRNA	miR-3179
t0058645	21	1	CGGGGAGAGAACGCAGTGGCG	miRNA	miR-3175
t0058759	21	1	CGGGGAGAGAACGCAGTGACT	miRNA	miR-3175
t0066556	22	1	TGTGACTTTAAGGGAAATGGAG	miRNA	miR-3164
t0072301	21	1	TATGAAATGAGGGCAGTAAGA	miRNA	miR-3163
t0075490	22	1	GAGGAGATCGTCGAGGTTGGCC	miRNA	miR-3150b-3p
t0076496	19	1	TGAGGAGATCGTCGAGGTT	miRNA	miR-3150b-3p
t0084507	19	1	TGAGGAGATTGTCGAGGTT	miRNA	miR-3150b-3p
t0084908	23	1	CATGCTAGGATAGAGAGAATGGG	miRNA	miR-3146
t0085090	21	1	AGCTTTTGGGAATTCAGGAAG	miRNA	miR-3140-3p
t0089722	21	1	AGCTTTTGGGAATGCAGGTAG	miRNA	miR-3140-3p
t0093252	22	1	TGTGGACAGTGAGGTAGAAGGA	miRNA	miR-3138
t0045327	23	1	TGTGGACAGTGAGGTAGAGGGCG	miRNA	miR-3138
t0050818	22	1	TGTGGACAGTGAGGAAGAGGGA	miRNA	miR-3138
t0066504	23	1	TGTGGACAGTGAGGGAGAGGGAG	miRNA	miR-3138
t0076459	24	1	TGTGGACAGTGAGGTAGAGGGCGA	miRNA	miR-3138
t0067751	20	1	GGCTGGTCCGAGGGCAGGGG	miRNA	miR-3135b
t0092120	22	1	GGCTGGGCCGAGTGCAGTGGTG	miRNA	miR-3135b
t0049706	23	1	GGCTGGTCCGAGTGCAGGGGTGT	miRNA	miR-3135b
t0055547	22	1	GGCTGGTCCGAGTGGAGTGGTG	miRNA	miR-3135b
t0072568	24	1	GACTGGTCCGAGTGCAGTGGTGTT	miRNA	miR-3135b
t0072454	22	1	GGCTGGTCTGAGTGCCGTGGTG	miRNA	miR-3135b
t0049271	23	1	GACTGGTCCGAGTGCAGTGGTGT	miRNA	miR-3135b
t0063451	23	1	GGCTGGTACGAGTGCAGTGGTGT	miRNA	miR-3135b
t0041173	23	1	GGCTGGTCCGAGTGCCGTGGTGT	miRNA	miR-3135b
t0042685	24	1	GGCTGGTCCGAATGCAGTGGTGTT	miRNA	miR-3135b
t0066359	22	1	GACTGGTCCGAGTGCAGTGGTG	miRNA	miR-3135b
t0070801	26	1	GGCTGGTCCGAGTGCAGTGGGGTTTA	miRNA	miR-3135b
t0086271	22	1	GGCTGGTCCGAGGGCAGTGGTG	miRNA	miR-3135b
t0048493	24	1	GGCTGGGCCGAGTGCAGTGGGGTT	miRNA	miR-3135b
t0052458	24	1	GGCTGGTCCGAGTGCCGTGGGGTT	miRNA	miR-3135b
t0052909	23	1	GGCTGGTCCGAGGGCAGTGGTGT	miRNA	miR-3135b
t0054271	24	1	GGCTAGTCCGAGTGCAGTGGTGTT	miRNA	miR-3135b
t0055188	24	1	GGCTGGTCCGAGTGCAGGGGGGTT	miRNA	miR-3135b
t0055243	20	1	TGGGAAGAGGAGCTCAGGGA	miRNA	miR-3132
t0056507	23	1	GCAGGTTGTGTAGAGTTGGCTTT	miRNA	miR-3129-5p

t0057635	18	1	TATTGCACTCACGGCCTG	miRNA	miR-311-3p
t0063579	19	1	TATTGCTGTCACGGCCTGT	miRNA	miR-311-3p
t0064523	23	1	CGGAGCAGCTCAGTACAGGAAGA	miRNA	miR-3107*
t0069007	23	1	CGGGGCAGCTCAGTACAGGAAGG	miRNA	miR-3107*
t0069220	23	1	CGAGGCAGCTCAGTACAGGAAGA	miRNA	miR-3107*
t0072240	22	1	AGCTTATGGTGGATTGTCGTTT	miRNA	miR-3101*
t0072888	20	1	AGCTTATAGCGGATTGTCGT	miRNA	miR-3101*
t0076106	23	1	AGCTTATGGTGGATTGTCGTTTT	miRNA	miR-3101*
t0080614	20	1	GAGCTTATGGTGGATTGTCG	miRNA	miR-3101*
t0084048	22	1	TGCTTATGGTGGATTGTCGTTT	miRNA	miR-3101*
t0088940	21	1	TATTGCACTCATCCCGACCAT	miRNA	miR-310
t0080315	23	1	GGAGGCAGATGTTCGCAAGACTG	miRNA	miR-31
t0080439	23	1	CTTTCAGTCGGAAGTTTACAGCA	miRNA	miR-30e*
t0073623	25	1	CTTTCAGTCGGATGTTTCCAGCATC	miRNA	miR-30e*
t0075874	20	1	ATTTCAGTCGGATGTTTACA	miRNA	miR-30e*
t0041105	20	1	CTTCCAGTCGGATGTTTACA	miRNA	miR-30e*
t0078451	23	1	CTTTCAGTCGGATGTTTCCAGCA	miRNA	miR-30e*
t0087693	26	1	TGTAAACATCCTTGACTGGAAGAATC	miRNA	miR-30e
t0045110	24	1	TGTAAACATCCTTGACTGGAAGAT	miRNA	miR-30e
t0052135	24	1	TGTAAACATCCTTCACTGGAAGCT	miRNA	miR-30e
t0055864	26	1	TGTAAACATCCTTGACTGGAAGCCTC	miRNA	miR-30e
t0077938	23	1	TGTAAACATCCCTGACTGGAAGA	miRNA	miR-30e
t0092705	26	1	TGTAAACATCCTTGACTGGAAGGATC	miRNA	miR-30e
t0060000	24	1	TGTAAACATCCCCGACAGGAAGCT	miRNA	miR-30d
t0067757	27	1	TGTAAACATCGTCGACTGGAAGCTATC	miRNA	miR-30d
t0040895	27	1	TGTAAACCTCCCCGACTGGAAGCTATC	miRNA	miR-30d
t0049432	24	1	TGTAGACATCCCCGACTGGAAGCT	miRNA	miR-30d
t0070477	23	1	TGTAAACATCCCCGACTGGACGA	miRNA	miR-30d
t0078121	20	1	TGAAAACATCCCCGACTGGA	miRNA	miR-30d
t0091564	22	1	TGTACACATCCCCGACTGGAAG	miRNA	miR-30d
t0042795	20	1	TGTAAACATCCCAGACTGGA	miRNA	miR-30d
t0062437	21	1	TGTAAACATACCCGACTGGAA	miRNA	miR-30d
t0067054	23	1	TGTAAACATCCCCGCCTGGAAGC	miRNA	miR-30d
t0069507	24	1	TGTAAACATCCACGACTGGAAGCT	miRNA	miR-30d
t0069889	24	1	GGTAAACATCCCCGACTGGAAGCT	miRNA	miR-30d
t0077088	25	1	TGTAAACATCCCCAACTGGAAGATC	miRNA	miR-30d
t0044451	24	1	TGTAAACATCCCCGACTGGAAGAT	miRNA	miR-30d
t0045656	22	1	TATAAACATCCCCGACTGGAAG	miRNA	miR-30d
t0047822	24	1	TGCAAACATCCCCGACTGGAAGCT	miRNA	miR-30d
t0047995	24	1	TGAAAACATCCCCGACTGGAAGCT	miRNA	miR-30d
t0053943	24	1	TGTAAACATCCCCGACTGGACGCT	miRNA	miR-30d
t0056680	26	1	TGTAAACATCCCCGACTGGAAGCATT	miRNA	miR-30d
t0058746	24	1	TGTAAACACCCCCGACTGGAAGCT	miRNA	miR-30d
t0062610	24	1	TGTAAACATTCCCGACTGGAAGCT	miRNA	miR-30d
t0064772	24	1	TGTAAACATCCCCGACTGGAAGTT	miRNA	miR-30d
t0068260	26	1	TGTAAACATCCCCGACTGGAAGCATA	miRNA	miR-30d
t0069647	26	1	TGTAAACATCCCCGACTGGAAGAATA	miRNA	miR-30d
t0070719	21	1	TGTAAACATCCCTGACTGGAA	miRNA	miR-30d
t0070864	24	1	TGAAAACATCCTACACTCTCAGCT	miRNA	miR-30c
t0071546	21	1	TATAAACATCCTACACTCTCA	miRNA	miR-30c
t0076318	24	1	TGTAAACATCCTACACTATCAGCT	miRNA	miR-30c
t0079151	21	1	TGTACACATCCTACACTCTCA	miRNA	miR-30c
t0079447	23	1	TGTAAACATCCTACACTATCAGC	miRNA	miR-30c
t0084204	20	1	TGTAAACATCATACACTCTC	miRNA	miR-30c
t0086224	24	1	TGTAAAAATCCTACACTCTCAGCT	miRNA	miR-30c
t0086824	23	1	TGTAAACATCCTACACTCTCCGC	miRNA	miR-30c
t0087815	21	1	TGTAAACATACTACACTCTCA	miRNA	miR-30c
t0087913	26	1	TGTAAACATCCTACACTCTCCGCATC	miRNA	miR-30c

t0089684	24	1	TGTAGACATCCTACACTCTCAGCT	miRNA	miR-30c
t0092150	23	1	TTTCGGTCGGATGTTTACAGTTC	miRNA	miR-30b-3p
t0092361	20	1	TTCAGTCGGATGTTTAGAGA	miRNA	miR-30b-3p
t0044936	23	1	TTTTCAGTCGGATGTTTACGGTA	miRNA	miR-30b-3p
t0047459	23	1	TTTCAGACGGATGTTTACAGTTC	miRNA	miR-30b-3p
t0052436	20	1	CTTTAAGTCGGATGTTTATA	miRNA	miR-30b-3p
t0055270	21	1	TTTCAGTCGGATATTTACAGT	miRNA	miR-30b-3p
t0060445	24	1	CTTTCAGTCGGATGTTTGCAGATC	miRNA	miR-30b-3p
t0063487	21	1	CTTTCAGTCGGATGTTTAAAG	miRNA	miR-30b-3p
t0063982	20	1	CTGGGAGGAGGATGTTTACT	miRNA	miR-30b*
t0066027	20	1	CTGGGAGGGGGATGTTTACT	miRNA	miR-30b*
t0073686	32	1	TGTAAACATCCTACACTCAGCTAGACATG	miRNA	miR-30b
t0080667	22	1	TGTAAACATCCTACACTAAGCT	miRNA	miR-30b
t0085674	22	1	TGTAAACATCCTACACTCAGCA	miRNA	miR-30b
t0052252	22	1	TGAAAACATCCTACACTCAGCT	miRNA	miR-30b
t0054609	22	1	TGTAAACATCTTACACTCAGCG	miRNA	miR-30b
t0062114	22	1	TGTAAACATCCTACACTCAGCC	miRNA	miR-30b
t0064713	22	1	TGTAAACATCCTACACACAGCT	miRNA	miR-30b
t0069683	22	1	TCTAAACATCCTACACTCAGCT	miRNA	miR-30b
t0069790	22	1	TGTAAACATCCTACACTCAGAT	miRNA	miR-30b
t0087226	32	1	TGTAAACATCCTACACTCAGCTAGACATG	miRNA	miR-30b
t0089821	22	1	TGTAAACATCCTACACTCCGCT	miRNA	miR-30b
t0046961	26	1	TGTAAACATCCTCGACTAGAAGCATC	miRNA	miR-30a-5p
t0056115	26	1	TGTAAACATCCCCGACTGGACGCATC	miRNA	miR-30a-5p
t0041954	26	1	TGTAAACATCCTCGACTGGACGCATC	miRNA	miR-30a-5p
t0062423	26	1	TGTAAACATCCCCGACTGGAAGAATC	miRNA	miR-30a-5p
t0065170	26	1	TGTAGACATCCTCGACTGGAAGCATC	miRNA	miR-30a-5p
t0066807	26	1	TGTAAACATCCCCGACTGGATGCATC	miRNA	miR-30a-5p
t0072464	26	1	TGTAAACATCCTCGACTGGAAGCGTC	miRNA	miR-30a-5p
t0077192	26	1	TGTAAACATCCTCGACTGGAAGCATT	miRNA	miR-30a-5p
t0077708	26	1	TGTACACATCCTCGACTGGAAGCATC	miRNA	miR-30a-5p
t0084323	21	1	GTAAACATCCTCGACTGGAAG	miRNA	miR-30a-5p
t0092587	26	1	TGTAAACATCCCCGTCTGGAAGCATC	miRNA	miR-30a-5p
t0092747	26	1	TATAAACATCCTCGACTGGAAGCATC	miRNA	miR-30a-5p
t0092917	26	1	TGTAAGCATCCTCGACTGGAAGCATC	miRNA	miR-30a-5p
t0042293	26	1	TGTAAACATCCTCGGCTGGAAGCATC	miRNA	miR-30a-5p
t0044784	26	1	TGTAAACATCCTCGACTGGAAGCATG	miRNA	miR-30a-5p
t0045252	26	1	TGTAAACATCCTCGACTGGAAGCCTC	miRNA	miR-30a-5p
t0046830	25	1	CTTTCAGTCGGATGTTTGCAGCCTC	miRNA	miR-30a*
t0051463	25	1	ATTTCAGTCGGATGTTTGCAGCATC	miRNA	miR-30a*
t0051723	21	1	TTTCAGTCGGATGTTTGCAGT	miRNA	miR-30a*
t0064226	22	1	TGTAAACATCCTTGACTGGATC	miRNA	miR-30a
t0064496	22	1	TGTAAACATCCTCGACTGGATA	miRNA	miR-30a
t0077530	22	1	GGTAAACATCCTCGACTGGATC	miRNA	miR-30a
t0079230	20	1	TGTAAACTGGTATGCTCTCA	miRNA	miR-3048
t0080657	21	1	GCCTGTAAACTGGCATGCTCT	miRNA	miR-3048
t0080713	21	1	CTGTAAACTGGTATGCTCTCT	miRNA	miR-3048
t0086140	22	1	CTGTAAACTGGTATGCTCTCTA	miRNA	miR-3048
t0087326	23	1	CAGTGCAATAGAATTGTCAAAGC	miRNA	miR-301b-3p
t0090915	24	1	CAGTGCAATAGTATTGTCTAAGCA	miRNA	miR-301b-3p
t0091723	22	1	TATCACAGTTCTGCTTAGGTGA	miRNA	miR-2d-3p
t0047435	24	1	ACCCTTGTTCGACTGTGCTGTTTC	miRNA	miR-2c-5p
t0086821	24	1	ACCCTTGTTCGACTGTGATGTTTT	miRNA	miR-2c-5p
t0092850	22	1	TATCACAGCCCTGCTTGGGACA	miRNA	miR-2b
t0059392	22	1	TAGCATTATCTGAAATCGGGTT	miRNA	miR-29e
t0073497	21	1	TAGCATTATCTGAAATCGGGT	miRNA	miR-29e
t0089252	22	1	TAGCACCATCTGAAATCGGTAC	miRNA	miR-29d
t0047319	22	1	CTAGCAACATCTGAAATCGGTT	miRNA	miR-29d

t0074032	23	1	TAGCACCATCTGAAATCGGTATC	miRNA	miR-29d
t0076750	21	1	TAGCACCATTTGAAGTCGGTT	miRNA	miR-29c
t0080086	22	1	TAGCACCATCTGAAATCGGGTA	miRNA	miR-29c
t0061914	22	1	TAGCACCATTTGAAATCTGGTA	miRNA	miR-29c
t0081635	22	1	TAGCACCATCTGAAATCTGTTA	miRNA	miR-29c
t0062750	22	1	CAGCACCATTTGAAATCGGTTA	miRNA	miR-29c
t0059032	22	1	TACCACCATCTGAAATCGGTTA	miRNA	miR-29c
t0066875	22	1	TAGCACCATCTGAAATAGGTTA	miRNA	miR-29c
t0055478	22	1	TAGCACCATTTGAAACCGGTTA	miRNA	miR-29c
t0063993	21	1	TAGCACCCTTTGAAATCGGTT	miRNA	miR-29c
t0084811	22	1	TAGCAAAATTTGAAATCGGTTA	miRNA	miR-29c
t0071953	24	1	TAGCACCATCTGAAATCGGTTATC	miRNA	miR-29c
t0077789	22	1	TAGCACCATTTGAAATCGGGTA	miRNA	miR-29c
t0088751	21	1	TAGCCCCATTTGAAATCGGTT	miRNA	miR-29c
t0041116	22	1	CAGCACCATCTGAAATCGGTTA	miRNA	miR-29c
t0042021	21	1	TAGCACCATTTGAAATAAGTG	miRNA	miR-29b
t0050174	21	1	CGCGGGGCGGGGGGCGGGGCG	miRNA	miR-2981
t0050334	31	1	CCGCGCGCGGGTCGGGGGCGGGGCGG	miRNA	miR-2981
t0050561	26	1	CGCGGGTCGGGGGGTGGGGCGGACTG	miRNA	miR-2981
t0060390	23	1	CGCGGGTCGAGGGGCGGGGCGGA	miRNA	miR-2981
t0063764	23	1	CGCGGGGCGGGGGGCGGGGCGGA	miRNA	miR-2981
t0068001	24	1	GCGCGGGACGGGGGGAGGGGCGGA	miRNA	miR-2981
t0072894	20	1	GCGCGCGGGGCGGGGGGCGG	miRNA	miR-2981
t0085093	23	1	CGCGGGTCGGGGGGAGGGGCGGA	miRNA	miR-2981
t0086230	22	1	CGGGTCGGGGGGCGGGGAGGAC	miRNA	miR-2981
t0086777	27	1	CGCGGGTCGGGGGGCGGGGAGGACTGT	miRNA	miR-2981
t0090121	21	1	CGCGGGACGGGGGGCGGGGCG	miRNA	miR-2981
t0093142	31	1	CGCGCGCGGGTCGGGGGGCGGGGAGGA	miRNA	miR-2981
t0048609	31	1	CCGCGCGCGGGTCGGGGGGAGGGGCGG	miRNA	miR-2981
t0049842	31	1	CGCGCGCGGGTCGGGGGGAGGGGCGGA	miRNA	miR-2981
t0053968	23	1	GCGGGTCGGGGGGCGGGGCGGCC	miRNA	miR-2981
t0054411	27	1	CGCGGGGCGGGGGGCGGGGCGGACTGT	miRNA	miR-2981
t0055092	21	1	CGCGGGTCGGGGGGAGGGGCG	miRNA	miR-2981
t0059672	23	1	GAGGGTTGGGTGGAGGCTCTCTC	miRNA	miR-296-3p
t0059815	23	1	GAGGGTTGGGTGGAGGTTCTATC	miRNA	miR-296-3p
t0061176	23	1	GAGGGGTGGGTGGAGGCTCTATC	miRNA	miR-296-3p
t0061304	19	1	AGGGGGCGGGGCGGACTGT	miRNA	miR-2885
t0070518	24	1	CGGCGGGCGGCGGGGCGGGGCGGT	miRNA	miR-2885
t0071994	18	1	CGGCGGGCGGCGGGGCGG	miRNA	miR-2885
t0073054	19	1	GGGGGGCGGGACGGACTGT	miRNA	miR-2885
t0075935	21	1	GCGGCGGCGGTCGGCGGGGGG	miRNA	miR-2885
t0076991	19	1	GGGGGGCGGGGCGGACTGG	miRNA	miR-2885
t0077526	23	1	CGGTCGGCGGGGGGCGGGGCGGG	miRNA	miR-2885
t0085270	23	1	CGGCGGGCGGCGGGGCGGGGCGG	miRNA	miR-2885
t0088832	23	1	AAGGAGCTCACAGTCTATTGATC	miRNA	miR-28-5p
t0089506	24	1	ACTAGATTGTGAGCTCCTGGAGAT	miRNA	miR-28-3p
t0070406	24	1	TTCACAGTGGCTAAATTGTGCATC	miRNA	miR-27b
t0072269	24	1	TTCACAGTGGCTAAGTTCTGAATC	miRNA	miR-27a-3p
t0083351	24	1	TTCACAGTGGCTAAGTTCTGCATT	miRNA	miR-27a-3p
t0043214	24	1	TTCACAGTGGCTAAGTTCCGCATC	miRNA	miR-27a-3p
t0044797	21	1	GATGCGTATCGTTTAGTCAAT	miRNA	miR-279d*
t0059213	20	1	GATGCGTATCGTTTAGTCAA	miRNA	miR-279d*
t0068874	22	1	CGACTAGATATACACTCATCCA	miRNA	miR-279b
t0074401	20	1	TGATCCGACTCGAAGGACCA	miRNA	miR-2779
t0079005	21	1	TAAATGCATTTTCTGGCCCGT	miRNA	miR-277
t0086255	24	1	TAGGAACTTCATACCGTGGTCATC	miRNA	miR-276a-3p
t0086969	27	1	TGGTAACCCCGAACCACCGTTGGCATC	miRNA	miR-2765
t0066748	27	1	TGGTAACTCAGAACCACCGTTGGCATC	miRNA	miR-2765

t0090919	27	1	TGGTAAATCCGAACCACCGTTGGCATC	miRNA	miR-2765
t0091120	27	1	TGGTAACTCCGAACCACCGTTGACATC	miRNA	miR-2765
t0053281	20	1	GGCGGATGTAACCAAGTGGA	miRNA	miR-270
t0063000	20	1	GGCGGATGTCGCCAAGTGGA	miRNA	miR-270
t0074604	20	1	GGCGGATGTAGCCAATTGGA	miRNA	miR-270
t0061374	20	1	GGCGGATGTAGCTAAGTGGA	miRNA	miR-270
t0065637	20	1	GGCGGATGAAGCCAAGTGGA	miRNA	miR-270
t0045635	23	1	GGCGGATGTAGCCAAGTGGAGCC	miRNA	miR-270
t0056798	23	1	GGAGGATGTAGCCAAGTGGATTA	miRNA	miR-270
t0050711	20	1	GGCGGATGTAGCCAAGTGAA	miRNA	miR-270
t0050643	20	1	GGCGGATGTAGCAAAGTGGA	miRNA	miR-270
t0047648	20	1	GGCGGATGGAGCCAAGTGGA	miRNA	miR-270
t0064500	22	1	TTTAAGTAATTCAGGATAGGTT	miRNA	miR-26b
t0067271	22	1	TTCAAGTAATTCAGGATAGCTT	miRNA	miR-26b
t0084856	22	1	TTCAAGTAATTCAAGATAGGTT	miRNA	miR-26b
t0046444	21	1	TTCAAGTAATTAAGGATAGGT	miRNA	miR-26b
t0046921	22	1	TTCAAGTGATTCAGGATAGGTT	miRNA	miR-26b
t0050700	21	1	TTCAAGTAATTCAGGTTAGGT	miRNA	miR-26b
t0054426	21	1	TTCAAGTAGTTCAGGATAGGT	miRNA	miR-26b
t0070424	21	1	TTCTAGTAATTCAGGATAGGT	miRNA	miR-26b
t0071708	22	1	TTCAAGTAATTTAGGATAGGTA	miRNA	miR-26b
t0073007	21	1	TTTAAGTGATTCAGGATAGGT	miRNA	miR-26b
t0083891	22	1	TTCATGTAATTCAGGATAGGTT	miRNA	miR-26b
t0089080	22	1	TTCAAGTAAATCAGGATAGGTT	miRNA	miR-26b
t0092181	21	1	TTCAAGTAAGTCAGGATAGGT	miRNA	miR-26b
t0042200	21	1	ATCAAGTAATTCAGGATAGGT	miRNA	miR-26b
t0042262	20	1	TTCAAGTAATTCAGAATAGG	miRNA	miR-26b
t0043198	24	1	TTCAAGTAATTCAGGATCGGTTGA	miRNA	miR-26b
t0044353	22	1	TTCAAGGAATTCAGGATAGGTA	miRNA	miR-26b
t0046476	23	1	CTCAAGTAATTCAGGATAGGTTA	miRNA	miR-26b
t0048225	22	1	TTCAAGTAATTGAGGATAGGTA	miRNA	miR-26b
t0048901	22	1	TTCAGGTAATTCAGGATAGGTT	miRNA	miR-26b
t0049245	22	1	TTCAAGTACTTCAGGATAGGTT	miRNA	miR-26b
t0052863	21	1	CTCAAGTAATTCAGGATAGGT	miRNA	miR-26b
t0053021	22	1	TTCAAGTAATTCAGGATAGGCC	miRNA	miR-26b
t0054104	21	1	TTCCAGTAATTCAGGATAGGT	miRNA	miR-26b
t0055177	22	1	TTCAAGTAATCCAGGATAGGTA	miRNA	miR-26b
t0055756	21	1	TTAAAGTAATTCAGGATAGGT	miRNA	miR-26b
t0056203	22	1	TCCAAGTAATTCAGGATAGGTT	miRNA	miR-26b
t0057308	21	1	TTCATGTAATTCAGGATAGGT	miRNA	miR-26b
t0057714	22	1	TTCAAGTAATTCAGGATATGTT	miRNA	miR-26b
t0058125	20	1	TTCAAGTAATTAAGGATAGG	miRNA	miR-26b
t0058140	22	1	TTCAAGTAATTCATGATAGGTT	miRNA	miR-26b
t0063745	21	1	TTCAAGTAATTCAGCATAGGT	miRNA	miR-26b
t0064611	23	1	TTCAAGTAATTCAGGAGAGGTTA	miRNA	miR-26b
t0067073	21	1	TTCAAGTAATTCAGGATGGGT	miRNA	miR-26b
t0067391	21	1	TTGAAGTAATTCAGGATAGGT	miRNA	miR-26b
t0070526	22	1	TTCAAGTAACTCAGGATAGGTT	miRNA	miR-26b
t0070913	21	1	TTTAAGTAATTCAGGATAGGT	miRNA	miR-26b
t0071029	23	1	TTCAAGTAATTCAGGATGGGTTT	miRNA	miR-26b
t0072384	24	1	CTCAAGTAATTCAGGATAGGTAGA	miRNA	miR-26b
t0073369	21	1	TTCGAGTAATTCAGGATAGGT	miRNA	miR-26b
t0074512	22	1	TTCAAGTAATTTAGGATAGGTT	miRNA	miR-26b
t0075170	21	1	TTCAAGTAATTTAGGATAGGT	miRNA	miR-26b
t0078149	21	1	TTCACGTAATTCAGGATAGGT	miRNA	miR-26b
t0079096	22	1	TTCAAGTAATTCAGGATAGGAA	miRNA	miR-26b
t0079469	22	1	TCAAGGAATTCAGGATAGGTTG	miRNA	miR-26b
t0080679	22	1	TTCAAGTAACCTAGGATAGGCT	miRNA	miR-26a

t0081001	22	1	TTCAAGTAATCCAGGATAAGCA	miRNA	miR-26a
t0082338	23	1	TTCAAGGAATCCAGGATAGGCTT	miRNA	miR-26a
t0083290	20	1	TTCAAGTAATCCCGGATAGG	miRNA	miR-26a
t0083387	24	1	TTCAAGTAATCCAGGACAGGCTAG	miRNA	miR-26a
t0084051	20	1	TTCAAATAATCCAGGATAGG	miRNA	miR-26a
t0084340	21	1	TCAAGTGATCCAGGATAGGCT	miRNA	miR-26a
t0085610	21	1	TTCAAGTAATCCGGGATAGGC	miRNA	miR-26a
t0085834	22	1	TTCAAGTACTCCAGGATCGGCT	miRNA	miR-26a
t0086756	20	1	AAGTAATCCAGGATAGGCTT	miRNA	miR-26a
t0086785	22	1	TTCGAGTAATCCAGGATAGGCG	miRNA	miR-26a
t0088007	21	1	TCAAGTAATCCACGATAGGCT	miRNA	miR-26a
t0091756	20	1	TTCAAGTAATACAGGATAGG	miRNA	miR-26a
t0043610	22	1	TTCAGGTAATCCAGGATAGGCA	miRNA	miR-26a
t0045274	22	1	TTCAAGTAATCCAGGATCGGAT	miRNA	miR-26a
t0046040	22	1	TCAAGTAATCCAGGATAGGTTG	miRNA	miR-26a
t0046099	21	1	TTCAAGTAGTCCAGGATAGGC	miRNA	miR-26a
t0046488	22	1	TTCAAGAAATCTAGGATAGGCT	miRNA	miR-26a
t0048834	20	1	TTCAAGGAATCCAGGATAGG	miRNA	miR-26a
t0049145	22	1	TTCAAGTATTCCAGGATAGGCT	miRNA	miR-26a
t0052769	21	1	TTCAAGTAATCCCGGATAGGC	miRNA	miR-26a
t0054870	21	1	TTCAAGTAACCCAGGATAGGC	miRNA	miR-26a
t0056216	22	1	TTCAAGTAATCCTGGATAGGCA	miRNA	miR-26a
t0056494	25	1	TTCAAGTAATCCAGGATCGGCTAGA	miRNA	miR-26a
t0057275	23	1	TTCAAGTAATACAGGATAGGCTA	miRNA	miR-26a
t0058236	21	1	TTCAAGTAATCCAAGATAGGC	miRNA	miR-26a
t0058629	22	1	TTCAAGTAGTCCAGGATAGGCA	miRNA	miR-26a
t0058736	22	1	TTCAAGTAATCCACGATAGGCT	miRNA	miR-26a
t0058823	22	1	TTCAAGAAATCCAGGATAGGCA	miRNA	miR-26a
t0059348	23	1	TTCGAGTAATCCAGGATAGGATC	miRNA	miR-26a
t0059374	22	1	GTCAAGTAATCCAGGATAGGCT	miRNA	miR-26a
t0060123	23	1	CTTCAAGTAATCCAGGATCGGCT	miRNA	miR-26a
t0061197	21	1	TTCAAGTAATCCAGGATTGGC	miRNA	miR-26a
t0061762	20	1	TTCAAGTAATCCAGGATCGG	miRNA	miR-26a
t0062425	23	1	TTCAAGTAATCCAGGATAGGAAT	miRNA	miR-26a
t0063333	20	1	TTCAAGTAATCCAGAATAGG	miRNA	miR-26a
t0064346	22	1	TTCAAGTAATCCAGGAGAGGCT	miRNA	miR-26a
t0065126	22	1	TTCAAGTCATCCAGGATAGGCT	miRNA	miR-26a
t0066465	21	1	TTTAAGTAATCCAGGATAGGC	miRNA	miR-26a
t0067037	19	1	AAGTAATACAGGATAGGCT	miRNA	miR-26a
t0067262	22	1	ATCAAGTAATCCAGGATAGGCT	miRNA	miR-26a
t0067570	21	1	TCAAGTAATCTAGGATAGGCT	miRNA	miR-26a
t0069317	23	1	TTCAAGTAATCTGGGATAGGCTT	miRNA	miR-26a
t0069398	22	1	TTCAAGTAATCCAGGAGAGGCA	miRNA	miR-26a
t0069711	22	1	TTCAAGTAATCCAGGCTAGGCT	miRNA	miR-26a
t0071447	21	1	TTCAAGTAATCCAGGGTAGGC	miRNA	miR-26a
t0073915	23	1	TTCAAGTAATCCAGGATAGGCCA	miRNA	miR-26a
t0077229	21	1	CTCAAGTAATCCAGGATAGGC	miRNA	miR-26a
t0077986	22	1	TTCAAGTAATCAAGGATAGGCA	miRNA	miR-26a
t0078243	22	1	CTCAAGTAATCCAGGATAGGCA	miRNA	miR-26a
t0078873	22	1	TTCAAGTAATCCAGGATAGGGA	miRNA	miR-26a
t0079034	24	1	TTCAAGTAATCCAGGATAGACTAA	miRNA	miR-26a
t0080705	22	1	TGCAAGTAATCCAGGATAGGCT	miRNA	miR-26a
t0081791	22	1	TTCAAGGAATCCAGGATAGCCT	miRNA	miR-26a
t0082583	25	1	TTCAAGTAATCCAGGATAGGCTATA	miRNA	miR-26a
t0083123	22	1	TTCAAGTAAACCAGGATAGGCT	miRNA	miR-26a
t0083595	22	1	TTCACGTAATCCAGGATAGGCT	miRNA	miR-26a
t0084047	22	1	TTCAACTAATCCAGGATAGGCT	miRNA	miR-26a
t0084853	20	1	TTCAAGTAATCCAGGAAAGG	miRNA	miR-26a

t0085351	22	1	TTCAAGTAATCCAGGATAGACT	miRNA	miR-26a
t0085822	22	1	TTCAAGTAATCCAGGATCGGCC	miRNA	miR-26a
t0085916	22	1	TTCAAGTAATCGAGGATAGGCT	miRNA	miR-26a
t0085926	24	1	TGAGGTAGGAGGGTGTGTGGTTAG	miRNA	miR-265
t0086119	20	1	TGAGGGAGGAGGTTGTGTGG	miRNA	miR-265
t0086345	22	1	TGAGGGAGGAGGGTGTGTGGTA	miRNA	miR-265
t0086520	20	1	TGAGGGAGGAGGTTGTGTAG	miRNA	miR-265
t0086786	21	1	TGAGGGAGGAGGTTGTGTAGT	miRNA	miR-265
t0086891	20	1	TGAGGGAGGAGGGTGTATAG	miRNA	miR-265
t0087246	21	1	TGAGGGAGGAGGATGTGTCGT	miRNA	miR-265
t0087329	22	1	TGAGGGAGGAGGGTGTATAGTT	miRNA	miR-265
t0087798	25	1	TGAGGGAGGAGGTTGTGTGGTTATA	miRNA	miR-265
t0089929	25	1	AATGGCACTGGAAGAATTCACGGTC	miRNA	miR-263a-5p
t0091457	24	1	TTAAGTAGTAGTGCCGGAGAGATC	miRNA	miR-252b
t0092107	24	1	TTAAGTAGTAGGGCCGTAGAGATC	miRNA	miR-252b
t0092805	23	1	AGGCGGCGACTTGGGCAATTGCT	miRNA	miR-25*
t0042771	23	1	AGGCAGAGACTTGGGCAATTGCT	miRNA	miR-25*
t0055711	21	1	AAGCGGAGACTTGGGCAATTG	miRNA	miR-25*
t0061463	23	1	AGACGGAGACTTGGGCAATTGCT	miRNA	miR-25*
t0067332	21	1	AGGCGGCGACTTGGGCAATTG	miRNA	miR-25*
t0071157	23	1	AGGAGGAGACTTGGGCAATTGCT	miRNA	miR-25*
t0080870	21	1	AGGCGCAGACTTGGGCAATTG	miRNA	miR-25*
t0083003	20	1	AGGCGGAGACTTAGGCAATT	miRNA	miR-25*
t0086994	23	1	AGGAGGAGAATTGGGCAATTGAT	miRNA	miR-25*
t0090428	21	1	AGGCGGAGCCTTGGGCAATTG	miRNA	miR-25*
t0050569	21	1	GGGCGGAGACTTGGGCAATTG	miRNA	miR-25*
t0049259	21	1	AGGCGAAGACTTGGGCAATTG	miRNA	miR-25*
t0066286	21	1	AGGCGGAGAATTGGGCAATTG	miRNA	miR-25*
t0043563	21	1	AGGCGGAAACTTGGGCAATTG	miRNA	miR-25*
t0044519	21	1	AGGGGGAGACTTGGGCAATTG	miRNA	miR-25*
t0044589	23	1	AGGCGGAGACTTGGGGAATTGCT	miRNA	miR-25*
t0045266	21	1	AGGCGGAGACTTGGGTAATTG	miRNA	miR-25*
t0045840	23	1	AGGCGGAGACTTTGGCAATTGCT	miRNA	miR-25*
t0047074	21	1	AGGCAGAGACTTGGGCAATTG	miRNA	miR-25*
t0050620	23	1	AGGCGGAGGCTTGGGCAATTGCT	miRNA	miR-25*
t0052029	21	1	AGCCGGAGACTTGGGCAATTG	miRNA	miR-25*
t0060218	23	1	AGGCGGAGACTTGGGCAATTTCT	miRNA	miR-25*
t0060736	20	1	AGGCGGAGGCTTGGGCAATT	miRNA	miR-25*
t0061071	23	1	AGGCGGAGACTCGGGCAATTGCT	miRNA	miR-25*
t0063589	22	1	AGGCGGAGCCTTGGGCAATTGA	miRNA	miR-25*
t0065403	22	1	AGGAGGAGACTTGGGCAATTGA	miRNA	miR-25*
t0065944	21	1	AGGCGGGGACTTGGGCAATTG	miRNA	miR-25*
t0066219	21	1	AGGCGAAGGCTTGGGCAATTG	miRNA	miR-25*
t0067469	22	1	CATTGCACTTGACTCGGTATGA	miRNA	miR-25
t0068221	21	1	CATTGCAATTGTATCGGTCTG	miRNA	miR-25
t0074300	22	1	CATTGCAATTGTCTCGGTGTGA	miRNA	miR-25
t0075440	22	1	AATTGCACTTGTCTCGGTCTGT	miRNA	miR-25
t0076771	27	1	CATTGCACTTGTCTCGGTCTGGCGGGA	miRNA	miR-25
t0077673	19	1	CATTGCGCTTGTCTCGGTC	miRNA	miR-25
t0078712	22	1	TATTGCACTTGTCTCGGTCTGC	miRNA	miR-25
t0080462	21	1	CATTACACTTGTCTCGGTTTG	miRNA	miR-25
t0087131	27	1	CCTTGCACTTGTCTCGGTCTGGCGGGC	miRNA	miR-25
t0089546	23	1	CATTGCACTTGTCTCGGGCTGTA	miRNA	miR-25
t0090147	22	1	CCTTGCACTTGTCTCGGCCTGA	miRNA	miR-25
t0091840	19	1	GATTGCACTTGTCTCGGTC	miRNA	miR-25
t0092282	27	1	CATTGCACTTGCCTCGGTCTGAAGTAG	miRNA	miR-25
t0040945	35	1	CATTGCACTTGTCTCGGCCTGAATACTCT(	miRNA	miR-25
t0041015	22	1	CATTGCACCTGTCTCGGTCTGT	miRNA	miR-25

t0041265	21	1	CATTGCAGTTGTCTCGGGCTG	miRNA	miR-25
t0041578	20	1	CATTGCCCTTGTCTCGGTCT	miRNA	miR-25
t0042542	22	1	CATTGTACTTGTCTCGGTCTTA	miRNA	miR-25
t0042561	22	1	CATTGCACTTGTATCGGTCTGT	miRNA	miR-25
t0042935	21	1	CATTGCACTTGTCTCGTTCTG	miRNA	miR-25
t0044272	22	1	CATTGCATTTGTTTCGGTCTGA	miRNA	miR-25
t0044884	22	1	CATTGCACTTCTCTCGGTCTGT	miRNA	miR-25
t0045283	21	1	CATTGCACTTGTCTAGGTATG	miRNA	miR-25
t0045420	22	1	CATTGAATTTGTCTCGGTCTGA	miRNA	miR-25
t0045803	22	1	CAATCCACTTGTCTCGGTCTGA	miRNA	miR-25
t0046700	23	1	CATTGCACTTGTCCCGGTCTGAA	miRNA	miR-25
t0046843	19	1	CATTGTACTTGTCTCGGTC	miRNA	miR-25
t0048094	21	1	CATTGCTCTTGTCTCGGTCTG	miRNA	miR-25
t0048925	20	1	ATTGCACTTGTCTCAGTCTG	miRNA	miR-25
t0049611	19	1	CATTGCACTTATCTCGGTC	miRNA	miR-25
t0050414	22	1	CATTGCACTTGTCTCGGTCTTC	miRNA	miR-25
t0050912	21	1	ATTGCACTTGTATCGGTCTGA	miRNA	miR-25
t0051114	19	1	CACTGCACTTGTCTCGGTC	miRNA	miR-25
t0052070	21	1	CATTGCACTTGCCTCGGTCTT	miRNA	miR-25
t0053127	22	1	CATTGCACTTGTCTCGGTGTGT	miRNA	miR-25
t0053311	21	1	CATTGCACTGGTCTCGGTCTG	miRNA	miR-25
t0053529	21	1	CATTATACTTGTCTCGGTCTG	miRNA	miR-25
t0053597	22	1	CATTGCACTTGTCTCGGTCAGA	miRNA	miR-25
t0053628	22	1	CATTGCTCTTGTCTCGGTCTGT	miRNA	miR-25
t0053693	21	1	CATTGCACTTGACTAGGTCTG	miRNA	miR-25
t0053800	22	1	CATTGCCCTTGTCTCGGGCTGA	miRNA	miR-25
t0054164	21	1	CATTGCACGTGTCTCGGTCTG	miRNA	miR-25
t0054427	23	1	CATTGCACTTGTCTCGGTCTGCT	miRNA	miR-25
t0055652	23	1	CATTGCACTTGTCTCGGTCAGAA	miRNA	miR-25
t0055782	23	1	CATCGCACTTGTCTCGGTCTGAA	miRNA	miR-25
t0056298	21	1	ATTGCACTTGTCTCGGGCTGA	miRNA	miR-25
t0056902	20	1	CATTGCACTAGTCTCGGTCT	miRNA	miR-25
t0057234	21	1	CATTGTACTTGTCTCGGTATG	miRNA	miR-25
t0057295	22	1	CATTGCGCTTGTCTCGGGCTGA	miRNA	miR-25
t0057453	22	1	CATTGCAATTGTCTCGGGCTGA	miRNA	miR-25
t0057561	19	1	CTTTGCACTTGTCTCGGTC	miRNA	miR-25
t0057650	21	1	CATTGTACTTGTCCCGGTCTG	miRNA	miR-25
t0057943	23	1	CATTGCACTTGTCTCGGGCTGAT	miRNA	miR-25
t0057957	22	1	CATTGTACTTGTCTCGGGCTGA	miRNA	miR-25
t0058930	21	1	AATTGCACTTGTATCGGTCTG	miRNA	miR-25
t0059181	22	1	CATTGCACGTGTCTCGGTCTGA	miRNA	miR-25
t0059257	22	1	CATTGCACTTGTCTCTGTCTGC	miRNA	miR-25
t0059441	22	1	CATTGCCTTTGTCTCGGTCTGA	miRNA	miR-25
t0059874	23	1	CATTGCCCTTGTCTCGGTCTGAA	miRNA	miR-25
t0060514	22	1	CATTTAACTTGTCTCGGTCTGA	miRNA	miR-25
t0060527	22	1	CATTGCACTTGTCTAGGGCTGA	miRNA	miR-25
t0060530	22	1	CATTGCACATGTCTCGGGCTGA	miRNA	miR-25
t0060863	22	1	CATTGCATTTGCCTCGGTCTGA	miRNA	miR-25
t0061600	19	1	CATTGCACTTGTCTCAGTC	miRNA	miR-25
t0061877	22	1	CACTGCACTTGTCTCGGTCTGT	miRNA	miR-25
t0062558	23	1	CATTACACTTGTCTCGGTCTGAA	miRNA	miR-25
t0062593	22	1	TATTGCAATTGTCTCGGTCTGA	miRNA	miR-25
t0062611	18	1	CATTGCACTTGTCTCGGG	miRNA	miR-25
t0063330	22	1	TATTGCACTTGTCTCAGTCTGA	miRNA	miR-25
t0064092	22	1	CATTACACTTGTCTCGGCCTGA	miRNA	miR-25
t0064121	22	1	CATCGCACTTGTCTCGGTCTTA	miRNA	miR-25
t0064995	22	1	CATTGCACTTGGCTCGGTCTGT	miRNA	miR-25
t0065910	21	1	CATTGAACTTGTCTCGGGCTG	miRNA	miR-25

t0066208	21	1	CATTGCACTTGTCTCGGGCTA	miRNA	miR-25
t0066290	23	1	CATTGCACTTGTCTCGGGCTGTT	miRNA	miR-25
t0066720	22	1	CATTGTACTCGTCTCGGTCTGA	miRNA	miR-25
t0067006	23	1	CATTGCACTTGTCCCGGTCTGAT	miRNA	miR-25
t0067068	20	1	ATTACACTTGTCTCGGTCTG	miRNA	miR-25
t0067246	23	1	CGTTGCACTTGTCTCGGTCTGAA	miRNA	miR-25
t0068302	21	1	ACTGCACTTGTCTCGGTCTGA	miRNA	miR-25
t0068412	21	1	CATTGCACTAGTCTCGGTCTG	miRNA	miR-25
t0068468	23	1	CATTGCAATTGTCTCGGTCTGAA	miRNA	miR-25
t0068562	22	1	CATTGCAGTTGTCTCGGTCTGA	miRNA	miR-25
t0069057	23	1	CATTGCAATTGTCTCGGTCTGAG	miRNA	miR-25
t0069574	22	1	CATTGCAATTGTCTCGGTCTCA	miRNA	miR-25
t0069775	22	1	CATTGCACTTGTCTCGGTCGGA	miRNA	miR-25
t0070066	22	1	CATTGCAATTGTCTCGGTCTGC	miRNA	miR-25
t0071562	23	1	CATTGCACTTGTCTCAGTCTGTA	miRNA	miR-25
t0072303	42	1	CATTGCACTTGTCTCGGTCTGACGGGGCT	miRNA	miR-25
t0072352	23	1	CATTGCACTTGTCTTGGTCTGAA	miRNA	miR-25
t0072855	22	1	CATTGCACTTGGCTCGGGCTGA	miRNA	miR-25
t0072985	29	1	CATTGCACTTGTCTCGGCCTGAAAATCAT	miRNA	miR-25
t0075483	22	1	CATTGCATTTGTCTCGGCCTGA	miRNA	miR-25
t0076202	21	1	CATTGCAATTGTCTCGGACTG	miRNA	miR-25
t0076653	21	1	CATTTCACTTGTCTCGGTCTG	miRNA	miR-25
t0076895	21	1	ATTGCACTTGTCTCGGTCCGA	miRNA	miR-25
t0077889	20	1	CATTGCACTTGTCTGGGTTT	miRNA	miR-25
t0077892	22	1	CATTGCACTTGTTTCGGTCTGC	miRNA	miR-25
t0079140	19	1	CATTGCACTAGTCTCGGTC	miRNA	miR-25
t0079819	21	1	CATTGCACTTGTCACGGTCTG	miRNA	miR-25
t0080012	22	1	CATTGTAATTGTCTCGGTCTGA	miRNA	miR-25
t0080412	23	1	CATTGTACTTGTCTCGGTCTGAA	miRNA	miR-25
t0080691	22	1	CATTGCAATTGTCTCGGTATGA	miRNA	miR-25
t0080733	22	1	CATTGCAGTTGTCTCGGTGTGA	miRNA	miR-25
t0081309	22	1	CGTTGCATTTGTCTCGGTCTGA	miRNA	miR-25
t0081398	27	1	AATTGCACTTGTCTCGGTCTGGCGGGC	miRNA	miR-25
t0081546	21	1	CATTGCACTTGTCTTGGGCTG	miRNA	miR-25
t0081587	18	1	CGTTGCACTTGTCTCGGT	miRNA	miR-25
t0081739	21	1	ATCGCACTTGTCTCGGTCTGA	miRNA	miR-25
t0082126	22	1	CATTGCACTTGTCTCAGTCTGT	miRNA	miR-25
t0082469	35	1	CATTGCACTTGTCTCGGGCTGCAGGAATT	miRNA	miR-25
t0082686	21	1	CATTGTACTTGTCTCAGTCTG	miRNA	miR-25
t0083230	22	1	CATTGAACTTGTCTCGGGCTGA	miRNA	miR-25
t0084426	22	1	CATTGCTCTTGTCTCGGCCTGA	miRNA	miR-25
t0084629	21	1	CATTGCACTTGTCTCGGTCTC	miRNA	miR-25
t0085288	22	1	CATTGCAATTGTCTAGGTCTGA	miRNA	miR-25
t0086341	21	1	CATTGCACTCGTCTCGGTCTA	miRNA	miR-25
t0086373	26	1	TGGCTCAGTTCAGCAGGAACCGTATC	miRNA	miR-24b
t0086612	23	1	TGGCTCAGTTCAGCAGGAATAGT	miRNA	miR-24b
t0086694	24	1	TGACTCAGTTCAGCAGGAACAGAA	miRNA	miR-24b
t0087328	25	1	TGGCTCAGTTCAGCAGGAACAGATG	miRNA	miR-24b
t0088095	24	1	TGGCTCAGTTAAGCAGGAACAGAA	miRNA	miR-24b
t0088279	25	1	TGGCTCAGTTCAGCAGGCACAGATC	miRNA	miR-24b
t0089513	21	1	TGGCTCAGTTCAGCAGGACCA	miRNA	miR-24b
t0089992	23	1	TGGCTCAGTTCCGCAGGAAAAGT	miRNA	miR-24b
t0090380	24	1	TGGCTTAGTTCAGCAGGAACAGAA	miRNA	miR-24b
t0090784	23	1	TGGCTCAGTTCAGCAGGAACAGC	miRNA	miR-24b
t0091143	26	1	TGGCTCAGTTCAGCAGTACCAGAATC	miRNA	miR-24b
t0091471	24	1	TGGCTCAGTTCAGCAGGAACAGGT	miRNA	miR-24b
t0092457	25	1	TGGCTCAGTTCAGCAGGAACAGGTC	miRNA	miR-24b
t0042034	23	1	TGGCTCAGTTCAGCAGGAACGGT	miRNA	miR-24b

t0042416	22	1	GACTCAGTTCAGCAGGAACAGA	miRNA	miR-24b
t0043153	21	1	TGGCTCAGTTCAGCAGGAGCA	miRNA	miR-24b
t0047166	25	1	CGGCTCAGTTCAGCAGGAACAGATC	miRNA	miR-24b
t0048869	22	1	TGGCTCAGTTCAGCAGGAACAC	miRNA	miR-24b
t0057723	23	1	TGGCTCAGTTCAGCAGGCAAAGT	miRNA	miR-24b
t0058308	21	1	TGGCTCAGTTCAGCAGGAATA	miRNA	miR-24b
t0062588	24	1	TGGCTCAGTTCAGCAGGAACAGGA	miRNA	miR-24b
t0067878	26	1	GGCTCTAAGGGCTGGGTCGGGCGGGC	miRNA	miR-2487
t0068238	20	1	GATTGGCTCTGAGGGCTGGG	miRNA	miR-2487
t0068276	24	1	GGCTCTAAGGGCTGGGGCGGTCGG	miRNA	miR-2487
t0073176	21	1	TATTTTTTTGTAGGTCGTTTT	miRNA	miR-2421
t0073518	23	1	TGGCTCAGTTCAGAAGGAACAGT	miRNA	miR-24
t0073830	26	1	TGGCACAGTTCAGCAGGAACAGTATC	miRNA	miR-24
t0074158	23	1	TGGCTCAGTTAAGCAGGAACAGT	miRNA	miR-24
t0076480	21	1	TGGCTAAGTTCAGCAGGAACA	miRNA	miR-24
t0078280	24	1	TGGCTAAGTTCAGCAGGAACAGTG	miRNA	miR-24
t0079914	23	1	TGGCTCAGTTCTGCAGGAACAGT	miRNA	miR-24
t0082418	22	1	TGGCTCCGTTCAGCAGGAACAG	miRNA	miR-24
t0083086	23	1	TGGCTCAGCTCAGCAGGAACAGT	miRNA	miR-24
t0087016	23	1	TGGCTCGGTTCAGCAGGAACAGT	miRNA	miR-24
t0059769	21	1	TGGCTCAGTTCCGCAGGAACA	miRNA	miR-24
t0060950	23	1	CGGCTCAGTTCAGCAGGAACAGT	miRNA	miR-24
t0093250	24	1	TGGCTCAGTTCAGCCGGAACAGTG	miRNA	miR-24
t0069391	19	1	TCAGTTCAGCAGGGACAGT	miRNA	miR-24
t0041440	23	1	TAGCTCAGTTCAGCAGGAACAGT	miRNA	miR-24
t0045767	22	1	TGGCTCAGTCCAGCAGGAACAG	miRNA	miR-24
t0046007	23	1	TGGATCAGTTCAGCAGGAACAGT	miRNA	miR-24
t0046498	22	1	TGGCTCAGTTCAGCAAGAACAG	miRNA	miR-24
t0048995	24	1	TGGCTCGGTTCAGCAGGAACAGTT	miRNA	miR-24
t0052082	22	1	TGGCTCAGTTGAGCAGGAACAG	miRNA	miR-24
t0054897	22	1	TGGCTCAGTTCGGCAGGAACAG	miRNA	miR-24
t0059279	22	1	TGCCTCAGTTCAGCAGGAACAG	miRNA	miR-24
t0060373	23	1	TGGCTCAGATCAGCAGGAACAGT	miRNA	miR-24
t0060707	18	1	TGGTTCAGTTCAGCAGGA	miRNA	miR-24
t0060997	22	1	TGGCTCAGCTCAGCAGGAACAG	miRNA	miR-24
t0062461	23	1	TGGCTCCGTTCAGCAGGAACAGT	miRNA	miR-24
t0063248	23	1	AGGCTCAGTTCAGCAGGAACAGT	miRNA	miR-24
t0064018	22	1	TGGCTCAATTCAGCAGGAACAG	miRNA	miR-24
t0065240	22	1	TGGCTCAGTTCAACAGGAACAG	miRNA	miR-24
t0067896	23	1	TGGCTCAGTTCAGTAGGAACAGT	miRNA	miR-24
t0069017	19	1	TGGCTCAGTTCCGCAGGAA	miRNA	miR-24
t0071373	18	1	TGGCTCAGTTCAGCAGGC	miRNA	miR-24
t0073722	21	1	TGACTCAGTTCAGCAGGAACA	miRNA	miR-24
t0073798	23	1	TGGCTCAGTTCCGCAGGAACAGT	miRNA	miR-24
t0074220	24	1	TGGCTTAGTTCAGCAGGAACAGTT	miRNA	miR-24
t0074931	23	1	TGGCTCAGTTCAGGAGGAACAGT	miRNA	miR-24
t0075107	23	1	TTGGGTTCCTGGAATGCTGATTT	miRNA	miR-23b*
t0075446	21	1	TGGGTTCCTGGCATGCTGCTT	miRNA	miR-23b*
t0076086	20	1	TGGGATCCTGGCATGCTGAT	miRNA	miR-23b*
t0076813	20	1	ATCACATTGCAAGGGATTAC	miRNA	miR-23b
t0081600	19	1	ATCACATTGCCAGGGCTTA	miRNA	miR-23b
t0082684	20	1	ATTACATTGCCAGGGATTAC	miRNA	miR-23b
t0083848	19	1	ATCACATTGCCAGGAATTA	miRNA	miR-23b
t0084438	24	1	ATCACATTGCCAGGGATTACCCCT	miRNA	miR-23b
t0085416	20	1	ATCACATTGCCCGGGATTAA	miRNA	miR-23b
t0086502	19	1	ATCACATTGCCAGGGACTA	miRNA	miR-23b
t0087703	20	1	ATCACATTGACAGGGATTAC	miRNA	miR-23b
t0089145	19	1	ATAACATTGCCAGGGATTA	miRNA	miR-23b

t0092178	21	1	ATCACATCGCCAGGGATTTCA	miRNA	miR-23a
t0048967	21	1	AGCACATTGCCAGGGATTTCG	miRNA	miR-23a
t0060158	21	1	ATCAAATTGCCAGGGATTTCC	miRNA	miR-23a
t0073207	20	1	ATCACGTTGCCAGGGATTTC	miRNA	miR-23a
t0049083	20	1	ATCACATTGTCAGGGATTTA	miRNA	miR-23a
t0051777	19	1	ATCTCATTGCCAGGGATTT	miRNA	miR-23a
t0055011	19	1	ATCAGATTGCCAGGGATTT	miRNA	miR-23a
t0056471	20	1	ATCACATTGCCAGGGAGTTC	miRNA	miR-23a
t0057419	22	1	ATCACATTGCCAGGGATTTACA	miRNA	miR-23a
t0059468	20	1	ATCACGTTGCCAGGGATTTA	miRNA	miR-23a
t0063717	20	1	ATCACCTTGCCAGGGATTTC	miRNA	miR-23a
t0082983	20	1	ATCACATCGCCAGGGATTTC	miRNA	miR-23a
t0088612	19	1	ATCACATTGCAAGGGCTTT	miRNA	miR-23a
t0040904	23	1	ATCACATTGCCAGGGCTTTCCAA	miRNA	miR-23a
t0041704	18	1	TCACATTGCCAGGGATTT	miRNA	miR-23a
t0043627	20	1	ATCACATTGCCAGGGATTCC	miRNA	miR-23a
t0045799	19	1	ATCACATTGCCAAGGATTT	miRNA	miR-23a
t0046867	21	1	ATCACATAGCCAGGGATTTCA	miRNA	miR-23a
t0047228	21	1	ATCACATTGCCAGGGATTTAC	miRNA	miR-23a
t0048785	21	1	AATCATATTGCCAGGGATTTA	miRNA	miR-23a
t0050527	19	1	GTCACATTGCCAGGGATTT	miRNA	miR-23a
t0051144	21	1	ATAACATTGCCAGGGATTTAA	miRNA	miR-23a
t0051758	20	1	ATCACATTGCCAGGGCTTTT	miRNA	miR-23a
t0052037	20	1	ATCACATTGCCAGGGGTTTC	miRNA	miR-23a
t0054634	21	1	ATCACATTGCAAGGGATTTCC	miRNA	miR-23a
t0054753	20	1	ATCACATTGCCAGGGACTTC	miRNA	miR-23a
t0055366	19	1	ATCACGTTGCCAGGGATTT	miRNA	miR-23a
t0055491	19	1	ATCACATTGCCAGGGATGT	miRNA	miR-23a
t0057146	20	1	ATCACATTGCTAGGGATTTC	miRNA	miR-23a
t0057444	20	1	ATCACATTGCCAAGGATTTC	miRNA	miR-23a
t0057657	22	1	ATAACATTGACAGGGATTTCCA	miRNA	miR-23a
t0057950	20	1	ATCCCATTGCCAGGGATTTA	miRNA	miR-23a
t0058302	20	1	ATTACATTGCCAGGGATTTT	miRNA	miR-23a
t0059358	19	1	ACCACATTGCCAGGGATTT	miRNA	miR-23a
t0063135	20	1	ATCACATTGACAGGGATTTT	miRNA	miR-23a
t0064025	19	1	ATCAAATAGCCAGGGATTT	miRNA	miR-23a
t0065811	20	1	ATCACATTGCCAGGGTTTTA	miRNA	miR-23a
t0066691	19	1	ATCACATTGCCAGAGATTT	miRNA	miR-23a
t0067409	23	1	ATCACATTGCCAGGGATTTCTAA	miRNA	miR-23a
t0067762	21	1	ATCACATTGCCAGAGATTTCC	miRNA	miR-23a
t0067763	21	1	GTCACATTGCCAGGGATTTCA	miRNA	miR-23a
t0067809	19	1	ATCACATTTCCAGGGATTT	miRNA	miR-23a
t0067849	20	1	ATCAAATTGCCAGGGATTTA	miRNA	miR-23a
t0069983	22	1	ATCACCTTGCCAGGGATTTCCA	miRNA	miR-23a
t0070014	18	1	TTCACATTGCCAGGGATT	miRNA	miR-23a
t0071467	20	1	ATCCCATTGCCAGGGATTTC	miRNA	miR-23a
t0071672	22	1	ATCACATTGCCAGGGCTTTCCA	miRNA	miR-23a
t0073320	19	1	ATCACTTTGCCAGGGATTT	miRNA	miR-23a
t0073625	22	1	ATCACATTGCCAGGGGTTTCCA	miRNA	miR-23a
t0074797	19	1	TTCACATTGCCAGGGATTT	miRNA	miR-23a
t0075012	20	1	ATCACATTGCCAGGGGTTTT	miRNA	miR-23a
t0075348	18	1	ATCACATAGCCAGGGATT	miRNA	miR-23a
t0076093	20	1	ATCACATTGCCAGGGATTCA	miRNA	miR-23a
t0076834	19	1	ATCACATTGCTAGGGATTT	miRNA	miR-23a
t0076913	20	1	ATCACATTGCCAGGGATTTG	miRNA	miR-23a
t0077981	23	1	ATCACATTGCCAGGGATTTCCCA	miRNA	miR-23a
t0078835	20	1	ATCACTTTGCCAGGGATTTC	miRNA	miR-23a
t0078855	19	1	ATCACATTGCCCGGGATTT	miRNA	miR-23a

t0079434	18	1	ATCATATTGCCAGGGATT	miRNA	miR-23a
t0080121	20	1	ACCACATTGCCAGGGATTTC	miRNA	miR-23a
t0082945	22	1	TAGGAGGGGGTGTGATAGGATC	miRNA	miR-2392
t0083088	20	1	TATTGCACTCGCCCCGGCCA	miRNA	miR-235
t0083155	20	1	TATTGCACTCGGCCCGGCCA	miRNA	miR-235
t0083588	18	1	CGGCGGCGTCGGCGGCGG	miRNA	miR-2305
t0087559	23	1	CGGCGGCGTCGGCGGCGGGGGGA	miRNA	miR-2305
t0087828	21	1	CGGTGGCGGCGGCGGCGGGGG	miRNA	miR-2305
t0088338	20	1	CGGCGGCGGCGGCGGGGGGG	miRNA	miR-2305
t0088730	22	1	CGGCGGTGGCGGCGGCGGGGGG	miRNA	miR-2305
t0089417	22	1	CGGTGGCGGCGGCGGCGGGGGC	miRNA	miR-2305
t0090079	19	1	AAAGATTGGTTTTGGTTTT	miRNA	miR-2284c
t0090861	23	1	AAGCAGCTGTAGAGTATGCCTGG	miRNA	miR-2278
t0092139	21	1	AAGCTGCCAGTTGAAGAATTT	miRNA	miR-22-3p
t0092243	20	1	AAGCTACCAGTTGAAGAACT	miRNA	miR-22-3p
t0066922	19	1	AAGCTGCCAGTTGCAGAAC	miRNA	miR-22-3p
t0041395	18	1	AAACTGCCATTTGAAGAA	miRNA	miR-22-3p
t0050609	20	1	GAGCTGCCAGTTGAAGAACT	miRNA	miR-22-3p
t0053865	19	1	AGGCTGCCAGTTGAAGAAC	miRNA	miR-22-3p
t0057319	19	1	AAGCTGCCAGTTGAAGACC	miRNA	miR-22-3p
t0059365	23	1	AAGCTGACAGTTGAAGAACTGTA	miRNA	miR-22-3p
t0063802	19	1	AAGCTGCTAGTTGAAGAAC	miRNA	miR-22-3p
t0071034	22	1	AAGCTGCCAGTTGAAGGACTGT	miRNA	miR-22-3p
t0072603	22	1	ACGCTGCCAGTTGAAGAACTGT	miRNA	miR-22-3p
t0054892	20	1	AAGCTGCCAGTTGAAGATCT	miRNA	miR-22-3p
t0073022	19	1	AAGCTGCCAGTTGAAGGAC	miRNA	miR-22-3p
t0049691	22	1	TGTCAGTTCGTCAAATACCCCA	miRNA	miR-223
t0057627	22	1	TGTCAGTTTGTCAAATCCCCCA	miRNA	miR-223
t0057678	22	1	TGTCAGTTTGTCAAATAACCCA	miRNA	miR-223
t0061409	22	1	TGTCAGGTTGTCAAATACCCCA	miRNA	miR-223
t0066612	22	1	TGTCAGTTTGTCTAATACCCCA	miRNA	miR-223
t0072243	21	1	AGCTACATCTGGCTAATGGGT	miRNA	miR-222
t0077746	21	1	AGCTACATCTGGCTAGTGGGT	miRNA	miR-222
t0080558	21	1	AGCTACATCCGGCTACTGGGT	miRNA	miR-222
t0080942	22	1	AGATACATCTGGCTACTGGGAA	miRNA	miR-222
t0081598	23	1	AGCTACATCTGGCTACTGGGCCT	miRNA	miR-222
t0085219	21	1	AGCTACAACTGGCTACTGGGT	miRNA	miR-222
t0087342	21	1	AGCTACATCTGGCTCCTGGGT	miRNA	miR-222
t0088122	21	1	AGTTACATCTGGCTACTGGGT	miRNA	miR-222
t0041175	21	1	AGCTACACCTGGCTACTGGGT	miRNA	miR-222
t0041850	21	1	AGCTACATCTGGCTACTGGGC	miRNA	miR-222
t0050088	23	1	AGCTACATCTGGCTACTGGGTTT	miRNA	miR-222
t0054063	23	1	AGCTACATCTGGCTACTGGGTCC	miRNA	miR-222
t0061664	22	1	AGCTGCATCTGGCTACTGGGTA	miRNA	miR-222
t0043595	22	1	AGCTACATTTGGCTACTGGGAA	miRNA	miR-222
t0047506	21	1	ACCTGACATACAATGTAGATT	miRNA	miR-221*
t0052245	23	1	AAACTGGCATACAATGTAGATTT	miRNA	miR-221*
t0053241	22	1	ACCTGGCATACAATGTGGATTT	miRNA	miR-221*
t0055308	19	1	ACCTGGCATGCAATGTAGA	miRNA	miR-221*
t0060986	22	1	ACCTGGCATAAAATGTAGATTT	miRNA	miR-221*
t0061609	21	1	AACTGGCATACAATGTAGATT	miRNA	miR-221*
t0063818	21	1	ACATGGCATCCAATGTAGATT	miRNA	miR-221*
t0064224	21	1	AGCTACACTGTCTGCTGGGTT	miRNA	miR-221
t0072215	22	1	AGCTACATTGTCCGCTGGGTTA	miRNA	miR-221
t0073359	21	1	AGCTACATTGTATGCTGGGTT	miRNA	miR-221
t0078378	23	1	AGCTACATTGTCTGCTGGGGTAA	miRNA	miR-221
t0081809	20	1	AGCTACATTGTCTGCTGGGG	miRNA	miR-221
t0092153	22	1	AGCTACATTATCTGCTGGGTTT	miRNA	miR-221

t0047432	20	1	AGTTCTTCAGTGGCAAGGTT	miRNA	miR-22*
t0057449	21	1	AGTTCTTGAGTGGCAAGCTTT	miRNA	miR-22*
t0057921	23	1	GACTGGGGCGGTACATCTGTTAC	miRNA	miR-219-2-3p
t0068216	26	1	TGACTGGGGCGGTACATCTGTTCAAT	miRNA	miR-219-2-3p
t0071762	25	1	GACTGGGGCGGAACATCTGTTAAAT	miRNA	miR-219-2-3p
t0074112	24	1	TTGACTGGGGCGGACATCTGTTA	miRNA	miR-219-2-3p
t0083263	23	1	GACTGGGGCGGAACATCTGTTAA	miRNA	miR-219-2-3p
t0052407	20	1	TGACTGGGGCGGAACATCTG	miRNA	miR-219-2-3p
t0059749	20	1	GACTGTGGCGGGACATCTGT	miRNA	miR-219-2-3p
t0065402	18	1	TTGACTGGGGCGGGACAT	miRNA	miR-219-2-3p
t0066637	24	1	TGACTGGGGCGGAACATCTGTTAT	miRNA	miR-219-2-3p
t0080167	21	1	TTGACTGGGGCGGAACATCTG	miRNA	miR-219-2-3p
t0093074	19	1	TGACTGGGGCGGGACATCT	miRNA	miR-219-2-3p
t0052094	22	1	GACTGGGGCGGAACATCTGTTA	miRNA	miR-219-2-3p
t0052265	28	1	TGACTGGGGCGGGACATCTGTTAAATAT	miRNA	miR-219-2-3p
t0041876	20	1	TGGGGCGGCACATCTGTTAA	miRNA	miR-219-2-3p
t0042524	22	1	TGACTGAGGCGGTACATCTGTT	miRNA	miR-219-2-3p
t0044196	20	1	GACTGAGGCGGTACATCTGT	miRNA	miR-219-2-3p
t0045046	27	1	GACTGGGGCGGAACATCTGTTAAATAT	miRNA	miR-219-2-3p
t0045982	25	1	GACTGGGGCGGAACATCTGTTAAAG	miRNA	miR-219-2-3p
t0047470	29	1	TGACTGGGGCGGTACATCTGTTAAATATT	miRNA	miR-219-2-3p
t0049470	19	1	ACTGGGGCGGGACATCTGT	miRNA	miR-219-2-3p
t0052779	20	1	TGATTGGGGCGGGACATCTG	miRNA	miR-219-2-3p
t0053523	20	1	GACTGCGGCGGTACATCTGT	miRNA	miR-219-2-3p
t0056651	25	1	TTGACTGGGGCGGAACATCTGTTAA	miRNA	miR-219-2-3p
t0058176	21	1	GACTGGGGCGGGACATCTTTT	miRNA	miR-219-2-3p
t0060732	24	1	TGACTGGGGCGGAACATCTGTTAA	miRNA	miR-219-2-3p
t0065405	20	1	TGACTGGGGCGGCACATCTG	miRNA	miR-219-2-3p
t0066955	19	1	GACTGGGGCGGCACATCTG	miRNA	miR-219-2-3p
t0068152	20	1	GACTGTGGCGGTACCTCTGT	miRNA	miR-219-2-3p
t0071233	21	1	TGACTGAGGCGGTACATCTGT	miRNA	miR-219-2-3p
t0074846	24	1	TGACTGGGGCGGGACATCTGTTAA	miRNA	miR-219-2-3p
t0075606	21	1	AGAGTTGAGTCTGGACGCCTT	miRNA	miR-219-1-3p
t0075642	21	1	AGAGTTGAGTCTGGATGTCTT	miRNA	miR-219-1-3p
t0077550	23	1	TGATTGTCCATTCGCATTTCTTG	miRNA	miR-219
t0077833	20	1	TAATCGGACGAAATTTCTAA	miRNA	miR-2178
t0084469	25	1	TAATCTCAGCTGGAAACTGGGAATC	miRNA	miR-216a
t0084943	20	1	TGAGAGATTGTTGCATATTT	miRNA	miR-2162-5p
t0085173	19	1	AGTGAGATTGTTGCATCTA	miRNA	miR-2162-5p
t0085691	23	1	AGTGAGATTGTTGCATATTTACA	miRNA	miR-2162-5p
t0085858	19	1	ATTATGCAACGTTTCACTC	miRNA	miR-2162-3p
t0087908	21	1	TATTATGCAACGTTTCACTCT	miRNA	miR-2162-3p
t0087929	25	1	ATGACCTATGATTTGGCAGACAATC	miRNA	miR-215
t0090170	25	1	ATGACCTATGATTTGACCGACAATC	miRNA	miR-215
t0090382	25	1	ATGACCTATGATTTGACAGCCAATC	miRNA	miR-215
t0087032	25	1	ATGACTTATGATTTGACAGACAATC	miRNA	miR-215
t0088045	25	1	ATGACCTATAATTTGACAGACAATC	miRNA	miR-215
t0042417	25	1	ATGACCTATGATTTGACAGACAATA	miRNA	miR-215
t0069245	25	1	ATTACCTATGATTTGACAGACAATC	miRNA	miR-215
t0055353	25	1	ATGACCTATGACTTGACAGACAATC	miRNA	miR-215
t0043846	22	1	TTGGGGAAACGGCCGCTGCGAG	miRNA	miR-2110
t0087386	21	1	TTGGAGAAACGGCCGCTGAGT	miRNA	miR-2110
t0089477	23	1	TTGGGGAAAAGGCCGCGGAGTGG	miRNA	miR-2110
t0057451	21	1	TTGGGGAAACGGCCGCTGAGG	miRNA	miR-2110
t0091751	25	1	TTGGGGAAACGGCCGCTGAGGGAGA	miRNA	miR-2110
t0044434	21	1	TTGGGGAAACGGCCGCTGAGC	miRNA	miR-2110
t0053454	21	1	TTGGGAAAACGGCCGCTGAGT	miRNA	miR-2110
t0055390	22	1	TTGGAGAAACGGCCGCTGAGTG	miRNA	miR-2110

t0056189	25	1	TTGGGGAAACGGCCGCTGAGTGGGA	miRNA	miR-2110
t0069497	22	1	TTGGGAAAACGGCCGCTGAGTG	miRNA	miR-2110
t0089654	22	1	TTGGGGAAACGGCCGCTGAGAG	miRNA	miR-2110
t0090922	22	1	TTGGGGAAACAGCCGCTGAGTG	miRNA	miR-2110
t0091059	23	1	TTGGGGAAACGGTCGCTGAGTGA	miRNA	miR-2110
t0044751	25	1	TTGGGGAAACGGCCGCTGAGAGAGA	miRNA	miR-2110
t0045563	21	1	TTGGGGAAACGGCCGCTGGGT	miRNA	miR-2110
t0046854	25	1	TTGGGGAAACGGCCGATGAGTGAGT	miRNA	miR-2110
t0048458	23	1	TTGGGGAAACGGCCGATGAGTGA	miRNA	miR-2110
t0048626	23	1	TTGAGGAAACGGCCGCTGAGTGA	miRNA	miR-2110
t0049172	21	1	TTGGGGAAACTGCCGCTGAGT	miRNA	miR-2110
t0052064	21	1	TTGGGGAAAAGGCCGCTGAGT	miRNA	miR-2110
t0058087	22	1	TTGGGGAAACGGCCACTGAGTG	miRNA	miR-2110
t0058338	22	1	TTGGGGAAAAGGCCGCTGAGTG	miRNA	miR-2110
t0059850	21	1	TTGGGGAAACGGCCGCTGCGT	miRNA	miR-2110
t0060261	24	1	TTGGGGAAACGGCCGCTGGGTGAG	miRNA	miR-2110
t0062057	25	1	TTGGGGCAACGGCCGCTGAGTGAGT	miRNA	miR-2110
t0063336	21	1	TTGGGGAAACGGCCGCCGAGT	miRNA	miR-2110
t0063942	25	1	TTGGGCAAACGGCCGCTGAGTGAGA	miRNA	miR-2110
t0067156	22	1	TTGGGGCAACGGCCGCTGAGTG	miRNA	miR-2110
t0073163	25	1	TTGGGGACACGGCCGCTGAGTGAGA	miRNA	miR-2110
t0073523	23	1	TTGGGGAAACGGCCGCTGAGAGA	miRNA	miR-2110
t0075645	23	1	TTGGGGAAACAGCCGCTGAGTGA	miRNA	miR-2110
t0075898	21	1	TTGGGGAAACGGCCGCTAAGT	miRNA	miR-2110
t0078651	21	1	TTGGGGAAACGACCGCTGAGT	miRNA	miR-2110
t0080777	24	1	TTGGGGAAACGGCCACTGAGTGAG	miRNA	miR-2110
t0082450	21	1	TTGGGAAACGGCCGCTGAGTG	miRNA	miR-2110
t0082645	21	1	TTATTTTTGTCATCCTTCGCC	miRNA	miR-211
t0083074	23	1	CTGTGCGCGTGACAGCGGCTGAT	miRNA	miR-210
t0083399	20	1	CTGTACGTGTGACAGCGGCT	miRNA	miR-210
t0083666	25	1	TAGCTTATCAGGCTGATGTTGAATC	miRNA	miR-21
t0083889	22	1	CAGCTTATCAGACTGATGTTGA	miRNA	miR-21
t0084424	24	1	TAGCTTATCAGACTGATGTTGCCA	miRNA	miR-21
t0084433	21	1	TAGCTTATCAGACTGATGCTG	miRNA	miR-21
t0085392	22	1	TAGCTTATCAGACTGATGTTTA	miRNA	miR-21
t0086838	25	1	TAGCTTATCAGACTGATGTTGACTC	miRNA	miR-21
t0087330	26	1	TAGCTTATCAGAATGATGTTGACATC	miRNA	miR-21
t0088399	21	1	TAGCTCATCAGACTGATGTTG	miRNA	miR-21
t0091128	26	1	TAGCTTATCAGACTGATGTTAACATC	miRNA	miR-21
t0092633	24	1	TAGCTTATCAGACTGATGTTGCTC	miRNA	miR-21
t0065249	22	1	TAGCTTATAAGACTGATGTTGA	miRNA	miR-21
t0062055	26	1	TAGCTTATCAGATTGATGTTGACATC	miRNA	miR-21
t0073120	22	1	TAGCTTATCAGACTGACGTTGA	miRNA	miR-21
t0041041	22	1	TAGCTTTTCAGACTGATGTTGA	miRNA	miR-21
t0041073	20	1	TAGCTTATCCGACTGATGTT	miRNA	miR-21
t0041413	22	1	TAGCCTATCAGACTGATGTTGA	miRNA	miR-21
t0042828	21	1	TAGCTTATCAGAGTGATGTTG	miRNA	miR-21
t0042885	26	1	TAGCTTACCAGACTGATGTTGCCATC	miRNA	miR-21
t0043626	22	1	TAGCTTATCAGACTGCTGTTGA	miRNA	miR-21
t0044066	21	1	TAGCCTATCAGACTGATGTTG	miRNA	miR-21
t0045781	21	1	TAGCTTATCAGACTGATATTG	miRNA	miR-21
t0045927	21	1	TAGCTTATCAAACTGATGTTG	miRNA	miR-21
t0046944	21	1	TAGCTTATCAGATTGATGTTG	miRNA	miR-21
t0048028	22	1	TAGCTTATGAGACTGATGTTGA	miRNA	miR-21
t0049688	24	1	TAGCTTATCACACTTATGTTGATC	miRNA	miR-21
t0051127	26	1	TAGCTTACCAGACTGATGTTGACATA	miRNA	miR-21
t0051195	21	1	TAGCTTATCAGACTGATGGTG	miRNA	miR-21
t0051258	23	1	TCGCTTATCAGACTGATGTTGAC	miRNA	miR-21

t0051344	24	1	TAGCTTATCAGACTGACGTTGATC	miRNA	miR-21
t0051747	27	1	TAGCTTATCAGACTGATGTTGCCTATC	miRNA	miR-21
t0051781	22	1	TAGCTTATCAGCCTGATGTTGA	miRNA	miR-21
t0053349	22	1	TAGCTTATCAGACCGATGTTGA	miRNA	miR-21
t0054244	21	1	TAACTTATCAGACTGATGTTG	miRNA	miR-21
t0055168	21	1	TAGCTTATCAGACTGATGTTA	miRNA	miR-21
t0055475	20	1	TAGCTCATCAGACTGATGTT	miRNA	miR-21
t0057070	23	1	TAGCTTATTAGACTGATGTTGAC	miRNA	miR-21
t0059670	26	1	TAGCTTATCAGACTGATGTTGAGATC	miRNA	miR-21
t0059766	21	1	CAGCTTATCAGACTGATGTTG	miRNA	miR-21
t0062526	26	1	TAGCTTATCAAACTGATGTTGACATC	miRNA	miR-21
t0063192	21	1	TAGCTTATCAGCCTGATGTTG	miRNA	miR-21
t0063261	25	1	TACCTTATCAGACTGATGTTGAATC	miRNA	miR-21
t0065036	27	1	TAGCTTATCAGACTGATGTTGACCATC	miRNA	miR-21
t0066648	22	1	TAGCTTATCGGACTGATGTTGA	miRNA	miR-21
t0067044	21	1	TAGCTTATCGGACTGATGTTG	miRNA	miR-21
t0067658	21	1	TAGCTTATCTGACTGATGTTG	miRNA	miR-21
t0068351	22	1	TAGCTTATCAGACTAATGTTGA	miRNA	miR-21
t0069118	26	1	TAGCTTATCAGACCGATGTTGACATC	miRNA	miR-21
t0070270	24	1	TAGCTTATCAGTCTGATGTTGATC	miRNA	miR-21
t0070549	21	1	TAGCTTATCAGACCGATGTTG	miRNA	miR-21
t0071110	26	1	TAGCTTATCAGACTGATGTTGACATA	miRNA	miR-21
t0071359	21	1	TAGCTTACCAGACTGATGTTG	miRNA	miR-21
t0071932	21	1	TACCTTATCAGACTGATGTTG	miRNA	miR-21
t0073199	21	1	TAGCTTATCAGACTGATTTTG	miRNA	miR-21
t0075749	22	1	TGGCTTATCAGACTGATGTTGA	miRNA	miR-21
t0077483	26	1	TAGCTTATTAGACTGATGTTGACATC	miRNA	miR-21
t0077694	24	1	TAGCTTATCAGACTGATGTTGCTT	miRNA	miR-21
t0080796	23	1	TAGCTTATCAGAATGATGTTGAC	miRNA	miR-21
t0081247	20	1	ACTGTAGTATGGGCAATTTT	miRNA	miR-20b*
t0082408	22	1	TACTGTAGTATGGGCAATTTTT	miRNA	miR-20b*
t0083379	22	1	TACTGTAGTATGGGCACCTTTT	miRNA	miR-20b*
t0083819	23	1	CAAAGTGCTCACAGTGCAGGTAG	miRNA	miR-20b
t0084738	23	1	CAAGGTGCTCATAGTGCAGGTAG	miRNA	miR-20b
t0086961	24	1	CAAAGTGCTCATAGGGCAGGTAGT	miRNA	miR-20b
t0089702	24	1	CAAAGTGCTCATAGTGTAGGTAGA	miRNA	miR-20b
t0090039	23	1	CAAAGTGCTCATAGGGCAGGTAG	miRNA	miR-20b
t0090227	22	1	CAAAGTGCTCAAAGTGCAGGTA	miRNA	miR-20b
t0090245	23	1	CAAAGGGCTCATAGTGCAGGTAG	miRNA	miR-20b
t0091673	22	1	CAAAGTGCTCACAGTGCAGGTA	miRNA	miR-20b
t0092975	23	1	CAAAGTGCTCATAGTGCAGGAAG	miRNA	miR-20b
t0093005	25	1	TAGAATGTAAGGAAGTGTGTGGATC	miRNA	miR-206
t0045570	25	1	TGGAATGTAAGGAAGTGTATGGATC	miRNA	miR-206
t0048066	25	1	TGGAATGTAAGGAAGTGTGTGGGTC	miRNA	miR-206
t0053258	25	1	TGAAATGTAAGGAAGTGTGTGGATC	miRNA	miR-206
t0040798	25	1	TGGAATGTAATGAAGTGGGTGGATC	miRNA	miR-206
t0042336	24	1	TGGAATGTAAGGAAGTGTGTGATT	miRNA	miR-206
t0044153	25	1	TGGAATGCAAGGAAGTGTGTGGATC	miRNA	miR-206
t0052103	25	1	TGGAATGTAAGGAAGTGCGTGGATC	miRNA	miR-206
t0053276	25	1	TGGAATGTAAGGAAGCGTGTGGATC	miRNA	miR-206
t0055737	25	1	TGGAATGTAAGGAAGTGAGTGGATC	miRNA	miR-206
t0071947	25	1	TGGAATGTAAGGAAGTGTGTGGCTC	miRNA	miR-206
t0078761	25	1	TGGAATGTAAGGGAGTGTGTGGATC	miRNA	miR-206
t0086335	25	1	AGGAATGTAAGGAAGTGTGTGGATC	miRNA	miR-206
t0042199	25	1	TGGAATGTAAGGCAGTGTGTGGATC	miRNA	miR-206
t0043818	25	1	TGAAATGTTTAGGACCACTAGTCTC	miRNA	miR-203
t0048330	21	1	TTCCTGTGCATATACTTCTTT	miRNA	miR-202-5p
t0048905	22	1	TTTCCTATGCTTATACTTCTTT	miRNA	miR-202*

t0053004	26	1	TAATACTGCCGGGAAATGATGGAATC	miRNA	miR-200c
t0057028	26	1	TAATACTGCCGGGTAATGATGGAATT	miRNA	miR-200c
t0059145	27	1	TAATACTGCCTGGCAATGATGACTATC	miRNA	miR-200b
t0065172	23	1	TAAAGTGCTTATAGTCCAGGTAT	miRNA	miR-20
t0067160	23	1	TAGAGTGCTTATAGTGCAGGTAG	miRNA	miR-20
t0073823	21	1	TAAAGTGTTTATAGTGCAGGT	miRNA	miR-20
t0075442	23	1	TAAAGTGCTTATAGCGCAGGTAA	miRNA	miR-20
t0080720	24	1	TAAAGTGCTTATAGTGTAGGTAGA	miRNA	miR-20
t0084677	22	1	TAAAGTGATTATAGTGCAGGTA	miRNA	miR-20
t0092464	22	1	TATAGTGCTTATAGTGCAGGTA	miRNA	miR-20
t0058639	24	1	TAAAGTGCTTATAGTGAAGGTAGC	miRNA	miR-20
t0073654	23	1	TAAAGTGCTTATAGTGCGGGTAT	miRNA	miR-20
t0060952	24	1	TAGAGTGCTTATAGTGCAGGTAGA	miRNA	miR-20
t0065799	20	1	TAAAGTGCTTATAGGGCAGG	miRNA	miR-20
t0092115	22	1	TAAAGTGCCTATAGTGCAGGTA	miRNA	miR-20
t0079202	23	1	TAAAGGGCTTATAGTGCAGGTAA	miRNA	miR-20
t0041510	21	1	TAAAGTGCTTATAGGGCAGGT	miRNA	miR-20
t0041532	23	1	TAAAGTGCTTACAGTGCAGGTAA	miRNA	miR-20
t0042703	24	1	TAAAGTGCTTATAGAGCAGGTAGA	miRNA	miR-20
t0042822	23	1	TAAAGTGCTTATAGTGCAGGTCA	miRNA	miR-20
t0044217	22	1	TAAAGTGCTTATAGTGCAGGTC	miRNA	miR-20
t0050619	23	1	TAAAGTGCCTATAGTGCAGGTAA	miRNA	miR-20
t0050812	24	1	TAAAGTGCTTATAGGGCAGGTAGA	miRNA	miR-20
t0051658	22	1	TAAAGTGATTATAGTGCCGGTA	miRNA	miR-20
t0052295	24	1	TAAAGTGCTCATAGTGCAGGTAGA	miRNA	miR-20
t0055217	21	1	TAAAGTGCTTATAGTGCAGGG	miRNA	miR-20
t0058111	23	1	TAAAGTGCTTATAGTGCGGGTAA	miRNA	miR-20
t0059373	21	1	TAAAGTGCTTATACTGCAGGT	miRNA	miR-20
t0061046	23	1	TAAAGTGCTTATAGTGAAGGTAG	miRNA	miR-20
t0062794	21	1	TAAAGGGCTTATAGTGCAGGT	miRNA	miR-20
t0065619	22	1	TAAAGTGCTTATAGTGCCGGTA	miRNA	miR-20
t0068826	23	1	TAAAGTGCTTATAGTGCGGGTAG	miRNA	miR-20
t0068951	24	1	TAAAGGGCTTATAGTGCAGGTAGA	miRNA	miR-20
t0070557	25	1	TGGAATGTAAAGAAGTATGGGGATC	miRNA	miR-1b
t0075201	25	1	TGGAATGTAAAGAAGTATGTAAATC	miRNA	miR-1a
t0076082	25	1	TGGAATGTAAAGAAGTATGAACATC	miRNA	miR-1a
t0077099	25	1	TGGACTGTAAAGAAGTATGTACATC	miRNA	miR-1a
t0079067	23	1	TGGAATGTAAGGAAGTATGTATC	miRNA	miR-1a
t0081528	24	1	TGAATGTAAAGAAGTATGTATGTC	miRNA	miR-1a
t0084036	25	1	TGGAATGTAAAGAAGTATGAATATC	miRNA	miR-1a
t0084240	25	1	TGGAATGTAAAGAAGTATGTCCATC	miRNA	miR-1a
t0086733	23	1	TGGAATGTAAAGAAGTATGTTTC	miRNA	miR-1a
t0087928	25	1	TGGAATGTAAAGCAGTATGTACATC	miRNA	miR-1a
t0092094	25	1	TGGAATGTAAAGAAGTATATACATC	miRNA	miR-1a
t0092634	25	1	TGGAATATAAAGAAGTATGTACATC	miRNA	miR-1a
t0093479	25	1	TGGAATGTAAAGAAGTATGTCTATC	miRNA	miR-1a
t0045440	24	1	TGAATGTAAAGAAGAATGTATATC	miRNA	miR-1a
t0044162	25	1	TGGGATGTAAAGAAGTATGTACATC	miRNA	miR-1a
t0045891	23	1	TGGAATGTAAAGAAGGATGTATC	miRNA	miR-1a
t0050501	25	1	TGGAATGTAAAGAAGTATGCATATC	miRNA	miR-1a
t0054322	22	1	TGTGCAAAGCCATGCAAAACTG	miRNA	miR-19b
t0056307	23	1	TGTGAAAATCCATGCAAAACTGA	miRNA	miR-19b
t0061740	20	1	TGTGCAAATCCATGCAACAC	miRNA	miR-19b
t0064162	21	1	TGTGAAAATCCATGCAAAACT	miRNA	miR-19b
t0065703	22	1	TGCGCAAATCCATGCAAAACTG	miRNA	miR-19b
t0069337	21	1	TGTGCAAATCCATGCAACACT	miRNA	miR-19b
t0084156	21	1	TGCGCAAATCCATGCAAAACT	miRNA	miR-19b
t0088555	23	1	TGTGCAAATTTATGCAAAACTGA	miRNA	miR-19a

t0089882	21	1	ACAGTAGTCTGCACATTGGGT	miRNA	miR-199-3p
t0090417	23	1	TCCTGCTCTCCTTGCTGTAGTTT	miRNA	miR-1976
t0091897	22	1	TAGGTAGTTTCCTGTTGTTGGC	miRNA	miR-196b
t0091947	22	1	TAGGCAGTTTCCTGTTGTCGGA	miRNA	miR-196b
t0092310	25	1	TAGGAAGTTTCCTGTTGTTGGGATC	miRNA	miR-196b
t0042302	21	1	TAGGAAGTTTCCTGTTGTTGG	miRNA	miR-196b
t0048531	24	1	TAGGTAGTTTCATGTTGTTGGCTC	miRNA	miR-196a
t0053782	19	1	TGAGGTAGCTTGTATAGTT	miRNA	miR-1961
t0076129	18	1	TAAGGTAGATTGTATAGT	miRNA	miR-1961
t0079095	19	1	TGAAGTAGGTTGTATAGTT	miRNA	miR-1961
t0080387	20	1	TAGCAGAACATAAATATTGG	miRNA	miR-195
t0080961	22	1	TAGAAGCACATAAATATTGGCG	miRNA	miR-195
t0054263	22	1	TAGCAGCACATAAATACTGGCA	miRNA	miR-195
t0078489	21	1	TAGCAGCACATAAATATTGGA	miRNA	miR-195
t0045287	22	1	TAGCAGCACATAAATATTGCCA	miRNA	miR-195
t0042050	21	1	TAGCAGCACATAAATATTGCG	miRNA	miR-195
t0046391	23	1	TGTAACGGCAACTCCATGTGGAA	miRNA	miR-194
t0053393	23	1	TGTAACAGCAACTCCATGGGGAA	miRNA	miR-194
t0069010	23	1	TGTAACAGCAACTCCATGCGGAA	miRNA	miR-194
t0093431	23	1	TGTAACAGAAACTCCATGTGGAA	miRNA	miR-194
t0052380	23	1	TGTAAAAGCAACTCCATGTGGAA	miRNA	miR-194
t0054528	23	1	TGTAACAGCAACTCCATGAGGAA	miRNA	miR-194
t0065606	23	1	TGTAACAGTAACTCCATGTGGAA	miRNA	miR-194
t0046044	23	1	TATAACAGCAACTCCATGTGGAA	miRNA	miR-194
t0047884	23	1	TGAAACAGCAACTCCATGTGGAA	miRNA	miR-194
t0050864	22	1	TGGGACTTTGCGGGCGAGATGA	miRNA	miR-193a-5p
t0055809	22	1	TGGGTCTTTGCGGGCGAGTTGA	miRNA	miR-193a-5p
t0075377	22	1	TGGGTCTTTGCGGGCGCGATGA	miRNA	miR-193a-5p
t0091974	21	1	TGGGTCTTTGCAGGCGAGATG	miRNA	miR-193a-5p
t0040736	21	1	CTGACCTATGAATTTACAGCC	miRNA	miR-192
t0045893	21	1	CTGACCTATGAATTGACAGGC	miRNA	miR-192
t0059211	21	1	TGACCTATGAATTGGCAGCCA	miRNA	miR-192
t0076951	21	1	CTGACCTATGAATTGACTGCA	miRNA	miR-192
t0078526	21	1	CTGACCCATGAATTGACAGCC	miRNA	miR-192
t0081867	21	1	CTGACCTATGAATTGAGAGCC	miRNA	miR-192
t0083304	23	1	TGACCTATGAATTGACACCCAGT	miRNA	miR-192
t0092297	21	1	CTGGCCTATGAATTGACAGCT	miRNA	miR-192
t0093490	21	1	CTGAACTATGAATTGACAGCC	miRNA	miR-192
t0052516	21	1	TGACCTACGAATTGACAGCCA	miRNA	miR-192
t0060147	23	1	CTGACCTATGAATTAACAGCATC	miRNA	miR-192
t0072061	22	1	CTGACCTATGAATTGACCGCCG	miRNA	miR-192
t0072190	20	1	CTGACTTATGAATTGACAGC	miRNA	miR-192
t0040666	25	1	CTGACCTATGAATTGACAACCAGAA	miRNA	miR-192
t0040840	21	1	CTGACCTATGAATTGAAAGCA	miRNA	miR-192
t0041293	25	1	CTGACATATGAATTGACAGACAGAA	miRNA	miR-192
t0041639	23	1	TGACCTATGAATTGACAGCCAGG	miRNA	miR-192
t0041745	21	1	AGACCTATGAATTGACAGCCA	miRNA	miR-192
t0042425	23	1	TGACCTATGAATTGACACCCAGA	miRNA	miR-192
t0042695	23	1	TGACCTACGAATTGACAGCCAGA	miRNA	miR-192
t0042980	21	1	CTGATCTATGAATTGACAGCA	miRNA	miR-192
t0044251	21	1	TGACCTATGAATTGACAGCTC	miRNA	miR-192
t0044499	20	1	TGACCTATAAATTGACAGCC	miRNA	miR-192
t0045049	21	1	GGACCTATGAATTGACAGCCA	miRNA	miR-192
t0045393	22	1	CTGACCTATGAATTGACGGCCA	miRNA	miR-192
t0045759	22	1	TGACCTATGAATTGACAGCGAG	miRNA	miR-192
t0046159	22	1	CTGACCTAAGAATTGACAGCCA	miRNA	miR-192
t0046178	20	1	CTGACCTATGAATCGACAGC	miRNA	miR-192
t0046267	20	1	TGACCTATGCATTGACAGCC	miRNA	miR-192

t0046768	22	1	TGACCTATGAATTGACACCCAG	miRNA	miR-192
t0046822	21	1	TGACCTATGGATTGACAGCTA	miRNA	miR-192
t0047984	20	1	AGACCTATGAATTGACAGCC	miRNA	miR-192
t0048277	24	1	TGACCTATGAATTGACAGCCAGCA	miRNA	miR-192
t0049016	24	1	CTGACCTATGAATTGACAGCCCTC	miRNA	miR-192
t0050944	24	1	TGAACTATGAATTGACAGCCAGAA	miRNA	miR-192
t0051164	21	1	TAACCTATGAATTGACAGCCA	miRNA	miR-192
t0051318	21	1	CTGACCAATGAATTGACAGCA	miRNA	miR-192
t0051439	23	1	TGACCTATGAAATGACAGCCAGT	miRNA	miR-192
t0051609	21	1	CTGGCCTATGAATTGACAGCC	miRNA	miR-192
t0052028	21	1	CTGACCTATGAATTGGCAGCA	miRNA	miR-192
t0052081	21	1	TGACCTATGAATTGACAGACA	miRNA	miR-192
t0052825	24	1	TGACCTATGAATTGACAGCAAGTA	miRNA	miR-192
t0053459	21	1	CCTGACCTCTGAATTGACAGC	miRNA	miR-192
t0054123	21	1	CTGAGCTATGAATTGACAGCC	miRNA	miR-192
t0054669	22	1	TGACCTATGAATTGAAAGCCAG	miRNA	miR-192
t0057140	20	1	TGACCTATGAATTAACAGCT	miRNA	miR-192
t0058792	21	1	CTGACCTATGATTTGACAGCC	miRNA	miR-192
t0058877	25	1	CTGACCTATGAATTGACAGCCCGTA	miRNA	miR-192
t0059120	23	1	CTGACCTATGAATTGACCGCCAT	miRNA	miR-192
t0059194	21	1	CGACCTATGAATTGACAGCCA	miRNA	miR-192
t0059281	24	1	TGACCTATGAATTGACAGCCCGTA	miRNA	miR-192
t0059329	21	1	CTGACCTACGAATTGACAGCA	miRNA	miR-192
t0059824	20	1	CTGACCTATGAATTGCCAGC	miRNA	miR-192
t0060030	23	1	TGACCTATGAATTGACAGCAAGT	miRNA	miR-192
t0061424	20	1	TGACCTATGAATTGACACCC	miRNA	miR-192
t0061578	21	1	CAGACCTATGAATTGACAGCC	miRNA	miR-192
t0061814	20	1	CTGACCTATGAATTCACAGC	miRNA	miR-192
t0062509	21	1	CTGACCTATGAATTGACAACC	miRNA	miR-192
t0062609	23	1	TGACCTATGAATTGACAGACAGT	miRNA	miR-192
t0062839	23	1	TGAGCTATGAATTGACAGCCAGT	miRNA	miR-192
t0063127	23	1	TGACCTATGAATTGGCAGCCAGT	miRNA	miR-192
t0063344	21	1	TGACCTATGAACTGACAGCCA	miRNA	miR-192
t0063947	24	1	TGACCTATGAATTGACAGTCAAAA	miRNA	miR-192
t0064168	24	1	CTGACCTATGAACTGACAGCCAGT	miRNA	miR-192
t0064603	20	1	TGACCTATGAACTGACAGCC	miRNA	miR-192
t0064987	24	1	TGACCTATAAATTGACAGCCAGAT	miRNA	miR-192
t0065116	21	1	CTGACATATGAATTGACAGCC	miRNA	miR-192
t0065577	20	1	TTGACCTATGAATTGACAGC	miRNA	miR-192
t0065871	23	1	TGACCTATGAATTAACAGCCAGA	miRNA	miR-192
t0066119	22	1	CTGACCTATGAATTGAAAGCCA	miRNA	miR-192
t0066448	21	1	CTGACCTATGAAATGACAGCC	miRNA	miR-192
t0067121	21	1	CTGACCTATGAATTGACACCC	miRNA	miR-192
t0067225	23	1	TCACCTATGAATTGACAGCCAGA	miRNA	miR-192
t0067978	21	1	CTGACCTATGAGTTGACAGCC	miRNA	miR-192
t0069242	22	1	TCTGACCTATGAATTGAAAGCC	miRNA	miR-192
t0069522	23	1	CGACCTATGAATTGACAGCCAGT	miRNA	miR-192
t0070201	21	1	TGACCTATGAATTGAAAGCCA	miRNA	miR-192
t0070634	23	1	CTAACCTATGAATTGACAGCCAT	miRNA	miR-192
t0071225	21	1	CTCACCTATGAATTGACAGCA	miRNA	miR-192
t0071446	22	1	CTGACCTATGAATTGAAAGACA	miRNA	miR-192
t0071761	22	1	CTGACCTATGAATTAACAGCCG	miRNA	miR-192
t0072093	25	1	CTGACCTATGAATTGACAGCCCGTG	miRNA	miR-192
t0072328	22	1	CTGACCTCTGAATTGACAGCTA	miRNA	miR-192
t0072744	22	1	CTGACCTATGAATTGACAGGCA	miRNA	miR-192
t0072969	22	1	CTGACCTATGAATTAACAGCCA	miRNA	miR-192
t0074066	20	1	TGACCTATGAATTGACAGAA	miRNA	miR-192
t0074106	23	1	TGACCTATGAGTTGACAGCCAGA	miRNA	miR-192

t0074282	21	1	CTGACCTATGAATTGATAGCC	miRNA	miR-192
t0075009	20	1	CTGACCTATGAATTGAAAGC	miRNA	miR-192
t0075074	22	1	CTGACCTATGAACTGACAGCCA	miRNA	miR-192
t0075150	21	1	CTGACCTATGAATTGACAGGG	miRNA	miR-192
t0075438	24	1	CTGACCTACGAATTGACAGCCAGT	miRNA	miR-192
t0075883	20	1	CTGACCTATGAATTGACGGC	miRNA	miR-192
t0076159	21	1	CTGAGCTACGAATTGACAGCC	miRNA	miR-192
t0076366	21	1	CTGACCTATGAATTGCCCGCC	miRNA	miR-192
t0077982	20	1	ATGACCTATGAATTGACAGC	miRNA	miR-192
t0079119	23	1	TGACCTATGAATTGACGGCCAGG	miRNA	miR-192
t0079179	24	1	CTGACCTATGACTTGACAGCCAGT	miRNA	miR-192
t0079519	21	1	CGACCTATGAATTGACAGCTA	miRNA	miR-192
t0079666	21	1	CTGACCTATGATTTGACAGCA	miRNA	miR-192
t0081308	20	1	TGACCTATGAATTGACCGTC	miRNA	miR-192
t0081761	20	1	TGACCTATGAATTGATCGCC	miRNA	miR-192
t0081980	22	1	TGACCTATGTATTGACAGCCAG	miRNA	miR-192
t0082400	20	1	TGACCTATGAATTGAAAGCT	miRNA	miR-192
t0082507	21	1	CTGACCTATGAATTGACAGAT	miRNA	miR-192
t0083381	23	1	TGACCTATGAATTGACAGCCGGT	miRNA	miR-192
t0083450	24	1	CTGACCTATGAATTCACAGCCAGT	miRNA	miR-192
t0083577	21	1	CTGACCTATAAATTGACAGCT	miRNA	miR-192
t0083817	21	1	TAACCTATGAATTGACAGCTA	miRNA	miR-192
t0084480	22	1	CTGACCTATGAATTGACAGTCA	miRNA	miR-192
t0084560	24	1	TGACCTATGAATTAACAGCCAGTA	miRNA	miR-192
t0084733	21	1	CTGACCTATGAAGTGACAGCC	miRNA	miR-192
t0084828	20	1	CTGACCTATGAATTGATAGC	miRNA	miR-192
t0085059	23	1	CTGACCTATGACTTGACAGCCAG	miRNA	miR-192
t0085536	20	1	TGACCTATCAATTGACAGCC	miRNA	miR-192
t0085772	20	1	TGACCTGTGAATTGACAGCC	miRNA	miR-192
t0086214	21	1	TGACCTGTGAATTGACAGCTA	miRNA	miR-192
t0086401	20	1	TGACCTATGAATTTACAGCC	miRNA	miR-192
t0086461	22	1	TGACCTATGAATTGACAGCCAT	miRNA	miR-192
t0086672	24	1	TGCCCTATGAATTGACAGCCAGAA	miRNA	miR-192
t0087300	23	1	CAACGGAATCCCAAAATCAGCTG	miRNA	miR-191
t0088104	23	1	AACGGAATCTCAAAAGCAGCTGT	miRNA	miR-191
t0088277	20	1	CAACGGGATCCCAAAAGCAG	miRNA	miR-191
t0089220	20	1	CAACGGAAACCCAAAAGCAG	miRNA	miR-191
t0089618	21	1	AACGGAATCCCCAAAGCAGCT	miRNA	miR-191
t0089881	23	1	AACGGAACCCCAAAAGCAGCTGA	miRNA	miR-191
t0089959	21	1	AACGGAATCCCAAAAGGAGCT	miRNA	miR-191
t0090226	23	1	AACGGAATCCCAAAAGAAGCTGT	miRNA	miR-191
t0090332	23	1	ACAACGGAATCCCAAAAGAAGCT	miRNA	miR-191
t0090343	20	1	CAACGGAATCCAAAAAGCAC	miRNA	miR-191
t0091379	24	1	CAACGGAATCCCACAAGCAGCTGT	miRNA	miR-191
t0092288	23	1	CAACGTAATCCCAAAAGCAGCTG	miRNA	miR-191
t0092484	21	1	CAACGGAACCCCAAAAGCAGC	miRNA	miR-191
t0040880	21	1	CAACGGAATCCCAAAATCAGC	miRNA	miR-191
t0041350	21	1	AACGGAATCCCAAACGCAGCT	miRNA	miR-191
t0041739	23	1	CAACGGAATCCAAAAAGAAGCTG	miRNA	miR-191
t0041866	24	1	CAACGGAATCCCAAAAACAGCTGT	miRNA	miR-191
t0041914	22	1	AACGGAATCCCAAAAGAAGCAG	miRNA	miR-191
t0042112	21	1	ACGGAATCCCAAAAGCAGCTG	miRNA	miR-191
t0042635	22	1	CCACGGAATCCCAAAAGAAGCT	miRNA	miR-191
t0043005	22	1	CAACGGAGTCCCGAAAGCAGCT	miRNA	miR-191
t0043381	19	1	CAACGGAATCCCACAAGCA	miRNA	miR-191
t0043561	22	1	AACCGAATCCCAAAAGCAGCTG	miRNA	miR-191
t0043721	24	1	CAACGGCATCCCAAAAGCAGCTGT	miRNA	miR-191
t0043750	22	1	CAAAGGAATCCCAAAAGAAGCT	miRNA	miR-191

t0044332	23	1	AACCGAATCCCAAAAGCAGCTGA	miRNA	miR-191
t0044485	22	1	AACGGAATCCCGAAAGCAGCTG	miRNA	miR-191
t0044523	22	1	AAGGGAATCCCAAAAGCAGCTG	miRNA	miR-191
t0045061	20	1	CACCGGAATCCCAAAAGCAG	miRNA	miR-191
t0045704	22	1	CAACGGAATCCAAAAAGCCGCT	miRNA	miR-191
t0045843	22	1	AACGGAATCCCAAAAGCGGCTG	miRNA	miR-191
t0045976	21	1	AACGGAATCCCAAAAACAGCT	miRNA	miR-191
t0046189	24	1	CAACGGAATCCAAAAAGCAGCTGT	miRNA	miR-191
t0046331	24	1	CAACGGAATCCCAAAAGCAGCCGT	miRNA	miR-191
t0046352	23	1	CAACGGAATCCCAAAAGCATCTG	miRNA	miR-191
t0046878	24	1	CAACGGAATCCCAAAAGCGGCTGA	miRNA	miR-191
t0046913	22	1	AACGGAATCCCAAAAGCAGCAG	miRNA	miR-191
t0046916	22	1	AACGGAATTCCAAAAGCCGCTG	miRNA	miR-191
t0047282	24	1	CAACGGAATACCAAAAGCAGCTGT	miRNA	miR-191
t0047413	20	1	CAACGGAATCCCAAACGCAG	miRNA	miR-191
t0047464	24	1	CAACGGAATGCCAAAAGCAGCTGA	miRNA	miR-191
t0047883	22	1	AACGGAATCCCAAATGCAGCTG	miRNA	miR-191
t0048311	21	1	AACGGAATCCCAAAAGCAGGT	miRNA	miR-191
t0048527	21	1	CCACGGAATCCCAAAAGCAGC	miRNA	miR-191
t0048690	20	1	AAAGGAATCCCAAAAGCAGC	miRNA	miR-191
t0048791	22	1	CAACGGAATACCAAAAGAAGCT	miRNA	miR-191
t0049022	22	1	AACGGAGTCCCAAAAGCAGCTG	miRNA	miR-191
t0049274	21	1	AACGGAATCCCAAGAGCAGGT	miRNA	miR-191
t0049457	23	1	CAACGGAATCGCAAAAGCAGCTG	miRNA	miR-191
t0049590	21	1	CAAAGGAATCCCAAAAGCAGA	miRNA	miR-191
t0049820	22	1	CAATGGAATCCTAAAAGCAGCT	miRNA	miR-191
t0049887	22	1	CAAAGGAATCACAAAAGCAGCT	miRNA	miR-191
t0050215	23	1	AACGGAATCCCAGAAGCAGCTGA	miRNA	miR-191
t0050562	22	1	AACGGAATCCCACAAGCAGCTG	miRNA	miR-191
t0051096	22	1	AACGGACTCCCAAAAGCAGCTG	miRNA	miR-191
t0051448	24	1	CAACGGAATCCCAATAGCAGCTGT	miRNA	miR-191
t0051470	24	1	CAAGGGAATCCCAAAAGCAGCTGA	miRNA	miR-191
t0051548	24	1	CAACGGAATCCCAAAAGCAGTTGA	miRNA	miR-191
t0051567	26	1	CAACGGACTCCCAAAAGCAGCATAAA	miRNA	miR-191
t0051847	22	1	AACGGCATCCCAAAAGCAGCTG	miRNA	miR-191
t0051852	23	1	AACAGAATCCCAAAAGCAGCTGA	miRNA	miR-191
t0051889	22	1	AACGGAATTCCAAAAGCAGATG	miRNA	miR-191
t0052473	22	1	CAACGGAATCCTAAAAGAAGCT	miRNA	miR-191
t0052872	23	1	CAACGGAATCCCAAAAGAAGCTT	miRNA	miR-191
t0053099	21	1	CAACGGAATCCCGAAAGCAGC	miRNA	miR-191
t0053466	21	1	AACGGAATCGCAAAAGCAGCT	miRNA	miR-191
t0053723	23	1	CAACGGAATCCCAAAAGCAGGTG	miRNA	miR-191
t0053836	20	1	CAATGGAATCCCAAAAGCAG	miRNA	miR-191
t0053898	22	1	CAACGGACTCCCAAAAGCAGTT	miRNA	miR-191
t0054711	19	1	CAACGGAATTCTAAAAGCA	miRNA	miR-191
t0054760	23	1	CAACGGAATCCCAAAAGCAGACG	miRNA	miR-191
t0054820	22	1	AACGGAATCTCAAAAGTAGCTG	miRNA	miR-191
t0054847	22	1	AAACGGAATCCAAAAAGCAGCT	miRNA	miR-191
t0054912	24	1	CAACGGAATCCCAAAAGCAGTTGT	miRNA	miR-191
t0054937	20	1	AACGGAATCCCAAGAGCAGC	miRNA	miR-191
t0055094	22	1	AACGGAATGCCAAAAGCAGCTG	miRNA	miR-191
t0055688	24	1	CAACGGGATCCCAAAAGCAGCTGT	miRNA	miR-191
t0055866	22	1	AACGGAATCCCAGAAGCAGCTG	miRNA	miR-191
t0056146	22	1	AACGGAATCCCAAAAACAGCTG	miRNA	miR-191
t0056300	22	1	CAACGGAATGCCAAAAGCAGCT	miRNA	miR-191
t0056341	21	1	CAACGGAATCCCAAAAGAAGA	miRNA	miR-191
t0056357	24	1	CAACGAAATCCCAAAAGCAGCTAA	miRNA	miR-191
t0056402	23	1	CAACGGAATCCCAAAAGGAGCTG	miRNA	miR-191

t0056407	21	1	CAACGGAATCCCAAAAGCAAC	miRNA	miR-191
t0056723	19	1	CAACGGAATCCCAGAAGCA	miRNA	miR-191
t0056911	24	1	CAACGGAATCCCAAAAGCCGCTGT	miRNA	miR-191
t0057043	20	1	CAACGGAATCCCAAAAGTAG	miRNA	miR-191
t0057385	20	1	AACGGAATACCCAAAGCAGC	miRNA	miR-191
t0057750	24	1	AACGGAATCCCAAAAGAAGCTGAA	miRNA	miR-191
t0057986	21	1	AACGGAATACCAAAAGCAGCT	miRNA	miR-191
t0058462	20	1	CAACGGAATTCCAAAAGAAG	miRNA	miR-191
t0060132	19	1	CAACGGAATTGCAAAAGCA	miRNA	miR-191
t0060168	23	1	CGACGGAATCTCAAAAGCAGCTG	miRNA	miR-191
t0060327	22	1	AACGGAATCCCAAAAGAAGCTT	miRNA	miR-191
t0060639	24	1	AAACGGAATCCCAAAAGCAGCTGA	miRNA	miR-191
t0061387	22	1	CAACGGAATCCAAAAAGCAGAT	miRNA	miR-191
t0061630	21	1	CAACAGAATCCCAAAAGCAGC	miRNA	miR-191
t0063776	23	1	CAACGGAATCCCAACAGCAGCTG	miRNA	miR-191
t0064165	24	1	AAACGGAATCCCAAAAGCAGCTGT	miRNA	miR-191
t0065104	22	1	CAACGGAATCCCAAAAGAAGCA	miRNA	miR-191
t0065229	24	1	CAACGGAATCCCAAAAGCAGCTGC	miRNA	miR-191
t0065380	22	1	AACGAAATCCCAAAAGCAGCTG	miRNA	miR-191
t0065616	23	1	AACGGAATTCCAAAAGCAGCTGA	miRNA	miR-191
t0066186	22	1	CAACGGAATCCCAAAAGCAGAG	miRNA	miR-191
t0066314	24	1	CAACGGAATCCTAAAAGCAGCTGA	miRNA	miR-191
t0066317	21	1	AACGGAATCCCAAAAGCAGAT	miRNA	miR-191
t0066779	19	1	CAACGGAATACCAAAAGCA	miRNA	miR-191
t0066980	20	1	CAACGGAATCCCGAAAGCAG	miRNA	miR-191
t0067298	22	1	AACGGAATCCCAAAAGGAGCTG	miRNA	miR-191
t0067628	24	1	CACCGGAATCCCAAAAGCAGCTGT	miRNA	miR-191
t0067671	24	1	CAACGGAATCTCAAAAGCAGCCGA	miRNA	miR-191
t0067822	23	1	AACGGAATCCCAAAAGCAGCTGG	miRNA	miR-191
t0068022	23	1	GAACGGAATCCCAAAAGCAGCTG	miRNA	miR-191
t0068025	24	1	CATCGGAATCCCAAAAGCAGCTGA	miRNA	miR-191
t0068153	22	1	AACGGAATCCCAAAAGGAGCTC	miRNA	miR-191
t0068166	21	1	AACGCAATCCCAAAAGCAGCT	miRNA	miR-191
t0068423	23	1	AACGCAATCCCAAAAGCAGCTGA	miRNA	miR-191
t0068945	21	1	AACGGCATCCCAAAAGCAGCT	miRNA	miR-191
t0069092	20	1	CAACGGAACCCCAAAAGCAG	miRNA	miR-191
t0069475	24	1	CAACGGAATACAAAAAGCAGCTGT	miRNA	miR-191
t0070195	25	1	CAACGGAATCCCAAAACCAGCTGTG	miRNA	miR-191
t0070328	20	1	CAACGGAATCCCACAAGCAG	miRNA	miR-191
t0070371	22	1	CAACGGAATCCCAATAGCAGCT	miRNA	miR-191
t0070373	24	1	CAACGGAATCCCAAAAGGAGCTGA	miRNA	miR-191
t0070749	24	1	CAATGGAATCCCAAAAGCAGCTGT	miRNA	miR-191
t0072168	22	1	CATCGGAATCCTAAAAGCAGCT	miRNA	miR-191
t0072567	22	1	CAACAGAATCCAAAAAGCAGCT	miRNA	miR-191
t0072691	22	1	CAACGGAATCCCAAACGAAGCT	miRNA	miR-191
t0072953	22	1	TACCGGAATCCCAAAAGCAGCT	miRNA	miR-191
t0073000	21	1	CAACGGAATCCCCAAAGCAGC	miRNA	miR-191
t0073036	20	1	CAACGCAATCCCAAAAGCAG	miRNA	miR-191
t0073214	24	1	CAAAGGAATCCCAAAAGCAGCTGA	miRNA	miR-191
t0073425	23	1	CAACGGAATCTCAAGAGCAGCTG	miRNA	miR-191
t0073459	23	1	CAACGGAATTCCACAAGCAGCTG	miRNA	miR-191
t0073918	22	1	CAACGGAATACCAAAAGCAACT	miRNA	miR-191
t0074149	22	1	CAACGGACTCCCAAAAGCCGCT	miRNA	miR-191
t0074665	23	1	AACGGAATCCCAAAAGCAGCTGC	miRNA	miR-191
t0074962	19	1	CAACAGAATCCAAAAAGCA	miRNA	miR-191
t0075041	21	1	CAGCGGAATCCCAAAAGCAGC	miRNA	miR-191
t0075070	22	1	CAACCGAATCCCAAAAGCAGCT	miRNA	miR-191
t0075481	22	1	CAACTGAATCCCAAAAGCAGCT	miRNA	miR-191

t0075690	21	1	CAACGGAATCCCAAACGCAGC	miRNA	miR-191
t0075987	24	1	CAACGGAATCACAAAAGCAGCTGT	miRNA	miR-191
t0076285	21	1	AACAGAATCCCAAAAGCAGCT	miRNA	miR-191
t0076666	23	1	CAACGGAATCCTAAAAGCAGCTT	miRNA	miR-191
t0076736	22	1	TAACGGAATCCCAAAAGCAGTT	miRNA	miR-191
t0076971	22	1	AACTGAATCCCAAAAGCAGCTG	miRNA	miR-191
t0078064	22	1	CACGGAATCCCAAAAGCAGCTG	miRNA	miR-191
t0078191	24	1	CAACGGAATCCAAAAAGCAGCTGA	miRNA	miR-191
t0078341	22	1	AAACGGAATACCAAAAGCAGCT	miRNA	miR-191
t0078541	22	1	AACGGAATCCCAAAAGCAGCCG	miRNA	miR-191
t0078834	20	1	CAACGGAATCTCAAAAGCAG	miRNA	miR-191
t0078911	20	1	CAACGGAATCGCAAAAGCAG	miRNA	miR-191
t0079104	22	1	CAACGGAATCCCAAAAGACGCT	miRNA	miR-191
t0079895	22	1	CAACGTAATCCCAAAAGAAGCT	miRNA	miR-191
t0080040	19	1	CCACGGAATTCCAAAAGCA	miRNA	miR-191
t0080275	22	1	CTACGGAATCCCAAAAGCAGCT	miRNA	miR-191
t0080825	20	1	CAACGGAATCCTAAAAGCAG	miRNA	miR-191
t0081023	22	1	ATCGGAATCCCAAAAGCAGCTG	miRNA	miR-191
t0081166	24	1	CAACGGAATCCCCAAAGCAGCTGA	miRNA	miR-191
t0081257	21	1	AACGGAATCCCAGAAGCAGCT	miRNA	miR-191
t0081467	21	1	AACGGAATCCCAAAAGCGGCT	miRNA	miR-191
t0081705	26	1	CAACGGAATCCCAAAAGAAGCTGATC	miRNA	miR-191
t0082130	24	1	CAACGGAACCCCAAAAGCAGCTGA	miRNA	miR-191
t0082428	24	1	CAACGGACTCCCAAAAGCAGCTGA	miRNA	miR-191
t0082560	20	1	AACGGAATCCCAAAAGCCGC	miRNA	miR-191
t0082821	21	1	AACGGAATCCCGAAAGCAGCT	miRNA	miR-191
t0082941	21	1	CAACGGAATACCAAAAGCAGA	miRNA	miR-191
t0082956	19	1	CAACGGAATCCCAAAAGCC	miRNA	miR-191
t0083047	23	1	AACGGAATCCAAAAAGCAGCTGA	miRNA	miR-191
t0083330	24	1	TAACGGAATCCCAAAAGCAGCTGA	miRNA	miR-191
t0083606	24	1	CAACGCAATCTCAAAAGCAGCTGA	miRNA	miR-191
t0083887	22	1	AACGGAATCTCAAAAGAAGCTG	miRNA	miR-191
t0084630	24	1	CAACGGAATCCCAAAAGACGCTGA	miRNA	miR-191
t0084751	24	1	CAACGGAATCCCAAAAGCAGCTGG	miRNA	miR-191
t0085341	24	1	CAACGGAATCCAAAAAGTAGCTGT	miRNA	miR-191
t0085409	24	1	CAACGGAATCCCAAAAGCCGCTGA	miRNA	miR-191
t0085578	19	1	AACGGAATCCCCAAAGCAG	miRNA	miR-191
t0085595	23	1	CAACGGTATCCCAAAAGCAGCTG	miRNA	miR-191
t0086034	24	1	CAACGGAATTCCAAAAGAAGCTGT	miRNA	miR-191
t0086147	24	1	CGACGGAATCCCAAAAGCAGCTGT	miRNA	miR-191
t0086543	22	1	CAACGGATTCCCAAAAGCAGCT	miRNA	miR-191
t0086677	23	1	GACGGAATCCCAAAAGCAGCTGA	miRNA	miR-191
t0086851	23	1	AACGGAATCCCAAAAGCCGCTGT	miRNA	miR-191
t0086898	24	1	CAACAGAATCCCAAAAGCAGCTGT	miRNA	miR-191
t0087193	23	1	AACGGAATCCCAAACGCAGCTGA	miRNA	miR-191
t0087245	21	1	AACGGAATCCCAAATGCAGCT	miRNA	miR-191
t0087502	24	1	CAACGGAATCCCAAAAGCAGATGT	miRNA	miR-191
t0087594	22	1	CAACAGAATCACAAAAGCAGCT	miRNA	miR-191
t0087777	22	1	AACGGAATCCCAAACGCAGCTG	miRNA	miR-191
t0087871	19	1	AACGGAATCCCAAAAGTAG	miRNA	miR-191
t0088724	21	1	AACGGAATCCCACAAGCAGCT	miRNA	miR-191
t0088783	22	1	AGCGGAATCCCAAAAGCAGCTG	miRNA	miR-191
t0088931	24	1	CAACGGAATCCTAAAAGCAGCTGT	miRNA	miR-191
t0088988	19	1	CAACGGAATCCCAAACGCA	miRNA	miR-191
t0089192	22	1	AACGGAATCCAAAAAGCAGCTG	miRNA	miR-191
t0089394	23	1	CAAAGGAATCCCAAAAGCAGCTT	miRNA	miR-191
t0089758	22	1	AACGGAATCCCAAAAGCAGCGG	miRNA	miR-191
t0089768	21	1	CGGCGGGGACGGCGACTGGTC	miRNA	miR-1908

t0090538	23	1	CAGTGACCAGACATATCCCTTTT	miRNA	miR-190-3p
t0091079	21	1	TAAGGTGCATCTAGTGGAGTT	miRNA	miR-18b
t0091201	22	1	TAAGGTGCATCTAGTGCCGTTA	miRNA	miR-18b
t0091267	22	1	TAAGGGGCATCTAGTGCAGTTA	miRNA	miR-18b
t0091866	21	1	TAAGGTGCATCTAGGGGAGTT	miRNA	miR-18b
t0092382	21	1	ACTGCCCTAAGTGCTCCTTCT	miRNA	miR-18a*
t0092386	21	1	TAAGGTGCATCTAGAGCAGAT	miRNA	miR-18a
t0092999	20	1	TAAGGTGCATCTAGTGCGGA	miRNA	miR-18a
t0093253	21	1	TAAGGTGCATCTAGTACAGAT	miRNA	miR-18a
t0093287	22	1	CAAAGAATTCTACTTTTGGGCT	miRNA	miR-186
t0093323	21	1	CCAAAGAATTCTCCTTTCGGG	miRNA	miR-186
t0093496	20	1	CAAAGAATTCTCCTTTTGAA	miRNA	miR-186
t0069600	20	1	CAAAGAATTATCCTTTTGGA	miRNA	miR-186
t0077681	20	1	TAAAGAATTCTCCTTTTGGG	miRNA	miR-186
t0042632	19	1	CAAAGAATTCTCCTTTTGG	miRNA	miR-186
t0048513	22	1	CAGGGGCCGGCTTTCCTCTGGT	miRNA	miR-185*
t0069319	18	1	AGCAGGGGCTGGCTTTAC	miRNA	miR-185*
t0077414	21	1	AGGGGCTGGCTTTACTCTGGT	miRNA	miR-185*
t0090540	21	1	AGGGGCTGGATTTCCTCTGGT	miRNA	miR-185*
t0062768	20	1	AGGGGCTGGCTTTTATCTGG	miRNA	miR-185*
t0064675	21	1	AGGGGCTGGCTTTCCTCCGGT	miRNA	miR-185*
t0072804	21	1	GGGGGCTGGCTTTCCTCTGGT	miRNA	miR-185*
t0049253	21	1	TGGAGAGAGAGGCATTTCCTG	miRNA	miR-185
t0051454	21	1	GGAGAGAAAGGCAGTTACTGA	miRNA	miR-185
t0052775	23	1	CTGGAGGGAAAGGCAGTTCCTGA	miRNA	miR-185
t0057041	23	1	TGGAGAGACAGGCAGTTCCTGAT	miRNA	miR-185
t0069723	22	1	TGGAGAGAAGGGCAGTTCCTAA	miRNA	miR-185
t0083568	22	1	TGGAGCGAAAGGAAGTTCCTGA	miRNA	miR-185
t0049952	22	1	TGGAGAGAAAGGTAGTTCTTGA	miRNA	miR-185
t0050945	23	1	TGGAGAGAAAGGAAGTTACTGAT	miRNA	miR-185
t0053138	22	1	TGGAGTGAAAGGCAGTTCCTGG	miRNA	miR-185
t0063662	21	1	TGGGGTGAAAGGCAGTTCCTG	miRNA	miR-185
t0080189	22	1	TGGAGATAAAGGCAGTTCTTGA	miRNA	miR-185
t0084258	23	1	TCGAGAGAAAGGCCGTTCCTGAG	miRNA	miR-185
t0092637	24	1	TGGAGAGAAAGGGAGTTCCTGAAA	miRNA	miR-185
t0040665	22	1	TGGAGAGAAAGGCTGTTCATGA	miRNA	miR-185
t0041228	22	1	TGGAGAGAAAGGCAGTTCCTCT	miRNA	miR-185
t0041389	23	1	TGGAGAGAAAGGCAGTTCAGGAA	miRNA	miR-185
t0041768	24	1	TGGAGAGAAAGGCAGTTCCCGAAA	miRNA	miR-185
t0041921	19	1	TGGAGGGAAAGGCAGTTCC	miRNA	miR-185
t0041939	22	1	TGGAGCGTAAGGCAGTTCCTGA	miRNA	miR-185
t0042212	22	1	TGGAGACAAAGGCAATTCCTGA	miRNA	miR-185
t0042407	22	1	TGGGGAGAAAGGCAGTTTCTGA	miRNA	miR-185
t0042409	23	1	TGGAAAGAAAGGAAGTTCCTGAT	miRNA	miR-185
t0042806	20	1	TGGAGAGAAAGGCAGTTACA	miRNA	miR-185
t0043150	23	1	TTGAGAGAACGGCAGTTCCTGAA	miRNA	miR-185
t0043247	23	1	TGGAGAGAAAGGAAGTTCCTAAA	miRNA	miR-185
t0043360	24	1	TGAAGAGAAAGGCAGTTCCTGAAT	miRNA	miR-185
t0043547	22	1	TGGAGTGAAAGGCAGTTCCTGC	miRNA	miR-185
t0043695	24	1	TGGAGAGAAAGGCAGTTCCTGCAG	miRNA	miR-185
t0043737	22	1	TGGAGTGAAAGGCAGTTCCTGT	miRNA	miR-185
t0043768	22	1	TGGAGAGCAAGGCATTTCCTGA	miRNA	miR-185
t0043850	21	1	TGAGAGAAAGGCAGTTCCTTA	miRNA	miR-185
t0043893	22	1	TAGAGAGAAAGGCGGTTCCTGA	miRNA	miR-185
t0044054	22	1	TGGAGGGAAAGGCAGTTACTGA	miRNA	miR-185
t0044088	24	1	TGGAGAGGAAGGCAGTTCCTGAGT	miRNA	miR-185
t0044305	23	1	TGGAGAGACAGGCAGTTACTGAA	miRNA	miR-185
t0044924	25	1	TGGAGAGAAAGGCAGTTCCTGCAAA	miRNA	miR-185

t0045205	25	1	TGGAGAGAAAGGCAATTCCTGAAAA	miRNA	miR-185
t0045355	23	1	TTGAGCGAAAGGCAGTTCCTGAA	miRNA	miR-185
t0045388	23	1	TGGAGAGAAAGGCAGTTCATGGA	miRNA	miR-185
t0045531	22	1	TGGAGAGAAAGGCAGTTCTTTA	miRNA	miR-185
t0045684	22	1	TGGAAAAAAAGGCAGTTCCTGA	miRNA	miR-185
t0045955	22	1	TGGAGAGAAAGATAGTTCCTGA	miRNA	miR-185
t0045994	19	1	CGGAGAGAAAGGCAGTTCC	miRNA	miR-185
t0046072	22	1	TGGAGAGAAAGGCAGTTTCGGA	miRNA	miR-185
t0046080	22	1	CGGAGAAAAAGGCAGTTCCTGA	miRNA	miR-185
t0046093	20	1	TGGAGAGAAAGACAGTTCCG	miRNA	miR-185
t0046109	24	1	TGGAGAGAAAGGCAGTTCGTGAGA	miRNA	miR-185
t0046560	27	1	TGGAGAGATAGGCAGTTCCTGAACGTT	miRNA	miR-185
t0047230	20	1	TCGAGAGAGAGGCAGTTCCT	miRNA	miR-185
t0047476	22	1	TGGAGAGAAACACAGTTCCTGA	miRNA	miR-185
t0047483	22	1	TGGAGAGAAAGGCCGGTCCTGA	miRNA	miR-185
t0047484	25	1	TCGAGAGAAAGGCAGTTCCTGAATC	miRNA	miR-185
t0047677	22	1	TAGAGAGAAAGGCAGTTACTGA	miRNA	miR-185
t0047785	23	1	TGGAGAGAAAGGCAGTTAATGAA	miRNA	miR-185
t0047815	18	1	TGGAGGGAAAGGCAGTTG	miRNA	miR-185
t0047919	22	1	TGGAGAGAAAGGCATTTACTGA	miRNA	miR-185
t0048588	23	1	TGGACAGAAAGGCAGTTCCTGAT	miRNA	miR-185
t0048592	21	1	GGAGAGAAAAGCAGTTCCTGA	miRNA	miR-185
t0048877	22	1	TGGAGAGAAAGGCAGGTCCTGT	miRNA	miR-185
t0048972	22	1	TGGAGAGAAAGGGAGTTCCTTA	miRNA	miR-185
t0048973	22	1	TGTAGAGAAAGGCAATTCCTGA	miRNA	miR-185
t0049126	24	1	TGGAGAGAAACGCAGTTCCTGAAA	miRNA	miR-185
t0049266	22	1	TGAAGAGAAAGGCAGTTCCTGT	miRNA	miR-185
t0049404	23	1	TGGAGAGAGAGGCAGTTTCTGAA	miRNA	miR-185
t0049516	22	1	TGGAGAGAGATGCAGTTCCTGA	miRNA	miR-185
t0049552	22	1	TAGGGAGAAAGGCAGTTCCTGA	miRNA	miR-185
t0049850	20	1	TCGAGAGAAAGGCAGTTCCT	miRNA	miR-185
t0049925	22	1	TGGAGAGAAAAGCAGTTCCTGC	miRNA	miR-185
t0049958	22	1	CGGAGAGAAAAGCAGTTCCTGA	miRNA	miR-185
t0050152	24	1	TGGAGAGAAAGGCAGTTTCTGAAT	miRNA	miR-185
t0050167	24	1	TGGGGAGAAAGGCAGTTCCTGAGT	miRNA	miR-185
t0050343	22	1	TGGGAGAAAGGCAGTTCCTGAA	miRNA	miR-185
t0050469	22	1	TGGAGAGAAAGGCAGTTACTGC	miRNA	miR-185
t0050729	23	1	TGGAGAGAAAGGAAGTTCCTGTA	miRNA	miR-185
t0050923	22	1	TGGAGAGAAAGGCAGTTTCTCA	miRNA	miR-185
t0051259	22	1	TGGAGAGAAAACCAGTTCCTGA	miRNA	miR-185
t0051508	21	1	TGGAGAGAAAGGAAGTTCGTG	miRNA	miR-185
t0051608	23	1	TTGGAGAGAAAGGCAATTCCTGA	miRNA	miR-185
t0051842	22	1	TGCAGAGAAAGGCAGTTCTTGA	miRNA	miR-185
t0051933	22	1	TGGAGAAAAAGGGAGTTCCTGA	miRNA	miR-185
t0051983	23	1	TGGAGAGAAAGGCAGTTTCTGAG	miRNA	miR-185
t0052244	23	1	ATGGAGAGAAAGGGAGTTCCTGA	miRNA	miR-185
t0052249	22	1	TAGAGAGAAAGGCAGTTCCTGC	miRNA	miR-185
t0052270	22	1	TGGATAGAAAGGCAGCTCCTGA	miRNA	miR-185
t0052356	23	1	TGGAGAGAAAGGCAGTTCCTTGA	miRNA	miR-185
t0052700	26	1	TGGAGAGAAAGGCAGTTCCTTAAGGT	miRNA	miR-185
t0052902	22	1	TCGAGAGAAACGCAGTTCCTGA	miRNA	miR-185
t0053120	23	1	ATGGAGAGGAAGGCAGTTCCTGA	miRNA	miR-185
t0053197	22	1	TGGAGAGAAAGGAAGCTCCTGA	miRNA	miR-185
t0053222	23	1	TGGAGAGAAAGGCAGCTCTTGAA	miRNA	miR-185
t0053289	22	1	TGGAGAGAAAGTAAGTTCCTGA	miRNA	miR-185
t0053300	22	1	TGGACAGCAAGGCAGTTCCTGA	miRNA	miR-185
t0053361	22	1	TGGAGAGAAAGGCAGTCCTTGA	miRNA	miR-185
t0054005	22	1	TGGAGTGAAAGGAAGTTCCTGA	miRNA	miR-185

t0054054	23	1	TGGAGAGAAAGGGAGTTCCTGTG	miRNA	miR-185
t0054154	18	1	ATGGAGAGGAAGGCAGTT	miRNA	miR-185
t0055067	24	1	TGGAGAGAAAGACAGTTCCTGAAA	miRNA	miR-185
t0055078	22	1	TGTAGAGAAAGGCAGTTCCTTA	miRNA	miR-185
t0055096	21	1	TGTAGAGAAAGGCAGTTCCTT	miRNA	miR-185
t0055193	24	1	TGGAGAGAAAGGTAGTTCCTGAAA	miRNA	miR-185
t0055267	23	1	TGGAGACAAAGGCAGTTCCTGAT	miRNA	miR-185
t0055544	20	1	TGGAGAGAAAGGCAGCTCCT	miRNA	miR-185
t0055845	21	1	TGGAGAGAAAGACAGTTCATG	miRNA	miR-185
t0055880	22	1	TGGAGGGAAAGGCAGTTCCTAA	miRNA	miR-185
t0055957	18	1	TGGAGAGAACGGCAGTTT	miRNA	miR-185
t0056035	19	1	TGGAGAGGAAGGCAGTTCC	miRNA	miR-185
t0056068	22	1	GGGAGAGAAAGACAGTTCCTGA	miRNA	miR-185
t0056118	24	1	TGGAGAGAAAGGCAGTTCATGAAT	miRNA	miR-185
t0056292	22	1	TGGAGAGAGAGGCAGTTCATGA	miRNA	miR-185
t0056514	23	1	TGGAGAGAAAGGCGGTTCCTGGT	miRNA	miR-185
t0056554	24	1	TGGAGAGAAAGGCAGTTCTTGAAA	miRNA	miR-185
t0056586	23	1	TGGAGAGACAAGCAGTTCCTGAA	miRNA	miR-185
t0056995	23	1	TGGAGAGAAAGGCAGTTCTTGTA	miRNA	miR-185
t0057443	22	1	TGGAGAGAAAGGCTGCTCCTGA	miRNA	miR-185
t0057702	21	1	TGGAGAGAAAGGCCGTTCCTA	miRNA	miR-185
t0058440	21	1	TGAAGAGAAAGGCATTTCCTG	miRNA	miR-185
t0058447	22	1	TCGAGAGAAAGGCAGTTTCTGA	miRNA	miR-185
t0058586	22	1	TGGAGCGAAAGGCAGTTCATGA	miRNA	miR-185
t0058796	23	1	TGGAGAGAAAGGCAGTTATTGAA	miRNA	miR-185
t0058848	18	1	TGGAGAGAAAAGCAGTTG	miRNA	miR-185
t0059019	23	1	TGGAGAGAAAGGTAGTTCCTGAG	miRNA	miR-185
t0059313	22	1	TGGAGAGAAATGAAGTTCCTGA	miRNA	miR-185
t0059408	24	1	TGGTGAGAAAGGCAGTTCCTGAAA	miRNA	miR-185
t0059430	18	1	TAGAGAGAAAGGCAGTTG	miRNA	miR-185
t0059596	22	1	TGAAGAGAAAGGCAGTTCCTGC	miRNA	miR-185
t0060020	23	1	TTGAGAGAAAGGCCGTTCCTGAA	miRNA	miR-185
t0060538	23	1	CGGAGAGAAAGACAGTTCCTGAA	miRNA	miR-185
t0060568	23	1	TGGAGATAAAGGCAGTTCCTGAT	miRNA	miR-185
t0060572	21	1	TGGAGAGAACGGCAGTTTCTG	miRNA	miR-185
t0060599	25	1	TGGCGAGAAAGGCAGTTCCTGATGG	miRNA	miR-185
t0060850	22	1	TGGAGAGAAGGGCAGTTCATGA	miRNA	miR-185
t0061069	23	1	TGGAGAGAAAGGCAGTTCCTTTA	miRNA	miR-185
t0061348	23	1	TGGAGAGAAAGGCCGTTCCTGCA	miRNA	miR-185
t0061425	23	1	TGAAGAGAAAGGCAGTTCCTGAG	miRNA	miR-185
t0061486	22	1	TGAAGACAAAGGCAGTTCCTGA	miRNA	miR-185
t0061566	24	1	TGGAGAGAAAGGCAGCTCCTGAAT	miRNA	miR-185
t0061815	23	1	GGGAGAGAAAGGCAGTTGCTGAA	miRNA	miR-185
t0062264	19	1	AGAGAAAGGAAGTTCCTGA	miRNA	miR-185
t0062483	20	1	TGGTGAGAAAGGCAGTTCCT	miRNA	miR-185
t0062521	22	1	TGGAGAGAAAGCCAGTTCCTGC	miRNA	miR-185
t0062620	23	1	TGGAGATAAAGGCAGTTCCTGAA	miRNA	miR-185
t0062671	22	1	TGGAGAGAAAGGCAGTTCCTAC	miRNA	miR-185
t0062741	23	1	TGGAGAGAAGGGCAGTTCCTGCA	miRNA	miR-185
t0062843	22	1	TGGAGAGAAGGGCAGTTACTGA	miRNA	miR-185
t0063184	21	1	TGGAGAGAAAGGCCGTTACTG	miRNA	miR-185
t0063388	18	1	TGGAGAGAAAGGCAGTGC	miRNA	miR-185
t0063478	22	1	TAGAGAGAAAGGCATTTCCTGA	miRNA	miR-185
t0063540	23	1	TGGAGAAAAAGGCAGTTCCTGCG	miRNA	miR-185
t0063617	22	1	TGCAGAGCAAGGCAGTTCCTGA	miRNA	miR-185
t0063747	22	1	TGAGAGAAAGGCAGTTCCTGAT	miRNA	miR-185
t0063801	23	1	ATGGAAAGAAAGGCAGTTCCTGA	miRNA	miR-185
t0063808	18	1	CGGAGAGAAAGGCAGTTG	miRNA	miR-185

t0063858	22	1	TGGAGAGAAAGGCAGATCCTGC	miRNA	miR-185
t0063867	22	1	TGAGAGAAATGCAGTTCCTGAA	miRNA	miR-185
t0063929	23	1	TGGAGAGAAAGGCCGTTCCTGAG	miRNA	miR-185
t0063967	22	1	TGGAGAGAACGGAAGTTCCTGA	miRNA	miR-185
t0064038	22	1	TGGAGAGAAAGGAAATTCCTGA	miRNA	miR-185
t0064222	23	1	TGGAGAGAAAGGCAGTTCCTCCA	miRNA	miR-185
t0064444	23	1	TGGAGAGAAAGGCCGTTCATGAG	miRNA	miR-185
t0064529	22	1	TGGAGAGAAAGGTAGTTACTGA	miRNA	miR-185
t0064785	18	1	TGGGGAGAAAGGCAGTTC	miRNA	miR-185
t0064793	22	1	TGGAGAGAAACGAAGTTCCTGA	miRNA	miR-185
t0064880	21	1	TGGAGAGAAAGGCGGTTTCTG	miRNA	miR-185
t0065025	22	1	TGGATAGAAAGGCAGTTCCTGT	miRNA	miR-185
t0065101	24	1	TGGAAAGAAAGGCAGTTCCTGAAA	miRNA	miR-185
t0065142	22	1	TGGAGAGAAAGGTTGTTCCTGA	miRNA	miR-185
t0065190	24	1	TGGAGAGAAAGGCAGTTCCTGCGA	miRNA	miR-185
t0065284	22	1	TGGAGAGTAAGGCAGTTCTTGA	miRNA	miR-185
t0065610	22	1	TGGAGAGAAAGGCAATTACTGA	miRNA	miR-185
t0065763	20	1	CGGAGAGAAGGGCAGTTCCT	miRNA	miR-185
t0065766	23	1	TGGTGAGAAAGGCAGTTCCTGAG	miRNA	miR-185
t0065796	21	1	TGGAGGGAAAGGCAGTTCTTG	miRNA	miR-185
t0066084	22	1	TGGATAGAAAGGCAGTTACTGA	miRNA	miR-185
t0066151	22	1	TGGAGAGAAAGGCGGATCCTGA	miRNA	miR-185
t0066161	22	1	TGGAGAGAGAGGCAGTTGCTGA	miRNA	miR-185
t0066179	20	1	TGGGGAGAAAGGCCGTTCCT	miRNA	miR-185
t0066510	21	1	TGGAGCGAAAGGAAGTTCCTG	miRNA	miR-185
t0066861	23	1	TGGAGAGAAAGGCAGTTCCTCAT	miRNA	miR-185
t0067004	22	1	TGGAGAGAAAGGCAGTTCCCGT	miRNA	miR-185
t0067034	19	1	TAGAGAGAAAGGCAGTTCC	miRNA	miR-185
t0067242	22	1	TGGGGAGAAAGGCAGTTCTTGA	miRNA	miR-185
t0067270	22	1	TGGAGAGACAGGCCGTTCCTGA	miRNA	miR-185
t0067728	21	1	GGAGAGAAAGGCAGTTCCTGC	miRNA	miR-185
t0067741	19	1	TGGAGAGAAATGCAGTTCC	miRNA	miR-185
t0068111	22	1	TGGAGAGAAACGCAGTTCCTGC	miRNA	miR-185
t0068245	23	1	TGGAGAGTAAGGCAGTTCCTGAG	miRNA	miR-185
t0068676	22	1	TGGAGAGAAAAGCAGTTCCTGT	miRNA	miR-185
t0069063	22	1	TGGAGAGAAAGGCAGTACCTTA	miRNA	miR-185
t0069112	23	1	TGGAGCGAAAGGCAGGTCCTGAT	miRNA	miR-185
t0069264	23	1	TGGAGAGAACGGCAGTTTCTGAT	miRNA	miR-185
t0069503	22	1	TGGAGACAAAGGCAGTTTCTGA	miRNA	miR-185
t0069698	22	1	TGGGGAGAAAGGCAGTTCCTGT	miRNA	miR-185
t0069774	20	1	TGGAGACAAAGGCAGTTCCT	miRNA	miR-185
t0070193	22	1	TGGAGAGATAGGAAGTTCCTGA	miRNA	miR-185
t0070316	22	1	TGGAGAGAAAGGCAGTTCATGG	miRNA	miR-185
t0070469	21	1	TGGAGGGAAAGGCAGTTACTG	miRNA	miR-185
t0071030	20	1	TGGAGAGATAGGCAGTTCCT	miRNA	miR-185
t0071489	22	1	TGGAGAGAAAGGCAGCTTCTGA	miRNA	miR-185
t0071549	24	1	TGGAGAGAAAGGCAGTTCATGAAA	miRNA	miR-185
t0071567	23	1	TGGAGAGAAAGGCGGTTCCCGAA	miRNA	miR-185
t0071635	22	1	TGGATAGAAAGGAAGTTCCTGA	miRNA	miR-185
t0072001	21	1	TGGAGATAAAGGCAGTTCCTG	miRNA	miR-185
t0072057	22	1	TGGAGAGGAAGTCAGTTCCTGA	miRNA	miR-185
t0072445	20	1	TGGAGAGAAAGGAAGTTACT	miRNA	miR-185
t0072561	22	1	TGGAGAGAAAGGCAGTACCTGT	miRNA	miR-185
t0072624	22	1	TGGAGACAAAGGGAGTTCCTGA	miRNA	miR-185
t0072775	22	1	TGGAGAGAAAGGCAGTTGCTGT	miRNA	miR-185
t0072907	22	1	TGGAGAGAAAGGTAGTTCCTTA	miRNA	miR-185
t0072976	22	1	TGGAGAGAAAGGTAGTTCCTGC	miRNA	miR-185
t0073226	22	1	GGGAGAGAAAGGCAGTTCCTTA	miRNA	miR-185

t0073381	23	1	GTGGAGGGAAAGGCAGTTCCTGA	miRNA	miR-185
t0073525	22	1	TGGAGAGAAAGGCAGTTCGTGT	miRNA	miR-185
t0073543	22	1	TGGAGATAAAGGGAGTTCCTGA	miRNA	miR-185
t0073615	22	1	CGGAGAGAAAGGCAGTTCCTGC	miRNA	miR-185
t0073616	22	1	TGGAGAGAAAGAAAGTTCCTGA	miRNA	miR-185
t0073821	20	1	TGGAGAGAAAGGGAGTTCAT	miRNA	miR-185
t0073831	22	1	TGGAGGGAAAGGTAGTTCCTGA	miRNA	miR-185
t0073954	22	1	TGGAGAGAAAGGCAGCTCCTGG	miRNA	miR-185
t0074305	21	1	TGGAGAGAAAGGAGGTTCCTG	miRNA	miR-185
t0074411	22	1	TGAAGAGAAAGGAAGTTCCTGA	miRNA	miR-185
t0074527	23	1	ATGGAGAGAAAGGCAGTTCCTAA	miRNA	miR-185
t0074657	22	1	TGGGGAGAAAGGCCGTTCCTGA	miRNA	miR-185
t0074770	20	1	TGGAGAGAAAGAAAGTTCCT	miRNA	miR-185
t0074873	21	1	TGAGAGAAAGGCAGTTTCTGA	miRNA	miR-185
t0075223	19	1	TGGGGAGAAAGGCAGTTCC	miRNA	miR-185
t0075288	22	1	TGGAGAGAAAGGCAGTACCCGA	miRNA	miR-185
t0075374	22	1	TGGAGAGAGAGGCAGTCCCTGA	miRNA	miR-185
t0075506	20	1	AGGAGAAAGGCAGTTCCTGA	miRNA	miR-185
t0075545	21	1	TGGAGAAAAAGGCAGTTCATG	miRNA	miR-185
t0075738	24	1	TGAGAGAAAGGCAGTTCCTGAAAG	miRNA	miR-185
t0076135	23	1	TGGAGGGAAAGGCAGTTCCTTAA	miRNA	miR-185
t0076166	22	1	GGGAGAGAAAGGCAGTTCCTGT	miRNA	miR-185
t0076313	24	1	TGGAGTGAAAGGCAGTTCCTGAGT	miRNA	miR-185
t0076314	20	1	TGGAGAGAAAGTCAGTTCCT	miRNA	miR-185
t0076679	23	1	TGGAGAGAAAGGCAGTTCGTGAG	miRNA	miR-185
t0076784	22	1	TGGGGAGGAAGGCAGTTCCTGA	miRNA	miR-185
t0076792	27	1	TCGAGAGAAAGGCAGTTCCTGAACGTT	miRNA	miR-185
t0076923	24	1	TGGAGAGAAAGGCAGCTCCTGAAA	miRNA	miR-185
t0077429	22	1	TGGAGGGAAAGGCAGCTCCTGA	miRNA	miR-185
t0077435	23	1	TGGAGAGAAAGGCAGTTCATGCG	miRNA	miR-185
t0077481	18	1	TGGAGAGAAAGCCAGTTG	miRNA	miR-185
t0077489	21	1	CGGAGAGAAAGGCAGTTCTTG	miRNA	miR-185
t0077636	23	1	TGGACAGAAAGGGAGTTCCTGAA	miRNA	miR-185
t0077742	22	1	TGGAGAGGAAGGCAGTTCCTGC	miRNA	miR-185
t0078172	22	1	TGGGGAGAAAGGAAGTTCCTGA	miRNA	miR-185
t0079105	23	1	TGGAGGGAAAGGCAGTTCCTGCT	miRNA	miR-185
t0079498	21	1	TGGAGAGAAAGGTAGTTCCTA	miRNA	miR-185
t0079821	23	1	TGGAGAGCAAGGCAGTTCATGAT	miRNA	miR-185
t0080262	24	1	TGGAGAGAAAGGCAGTTCGTGATT	miRNA	miR-185
t0080608	19	1	TGGAGAGAAAGGAAGTTCC	miRNA	miR-185
t0081098	23	1	TGGAGAGAAAGCCAGTTCCTGAG	miRNA	miR-185
t0081127	22	1	TGGAGAAAAAGGCAGTTCATGA	miRNA	miR-185
t0081436	22	1	TGGAGGGATAGGCAGTTCCTGA	miRNA	miR-185
t0081756	22	1	TGGAGAGAAAGGCAGGTCCTTA	miRNA	miR-185
t0081907	22	1	TGGAGAGAAAGGTAGTTCCTGT	miRNA	miR-185
t0081959	22	1	TGGAGAGGAAGGCAGTTTCTGA	miRNA	miR-185
t0082160	22	1	GGGAGGGAAAGGCAGTTCCTGA	miRNA	miR-185
t0082687	23	1	TTGAGAGAAAGGCAGTTCCTGAT	miRNA	miR-185
t0082748	25	1	TGGAGTGAAAGGCAGTTCCTGAAAA	miRNA	miR-185
t0082981	22	1	TGGAGAGAAAGGAAGTTCGTGA	miRNA	miR-185
t0083187	22	1	TGGAGGGAAAGGCAGTTCATGA	miRNA	miR-185
t0083200	23	1	TGGGGAGAAAGGAAGTTCCTGAA	miRNA	miR-185
t0083430	23	1	TGTAGAGAAAGGCAGTTCCTGAT	miRNA	miR-185
t0083564	24	1	GGGAGAGAAAGGCAGTTCCTGAAA	miRNA	miR-185
t0083976	23	1	TGGCGAGAAAGGCAGTTCCTGCT	miRNA	miR-185
t0084080	24	1	TGGAGAGAAAGGCAGTTCCAGAAT	miRNA	miR-185
t0084091	22	1	TGGAGATAAAAGCAGTTCCTGA	miRNA	miR-185
t0084284	22	1	TGGAGAGAAAGTCAGTTCCTGT	miRNA	miR-185

t0084313	23	1	TGGAGACAAAGGAAGTTCCTGAA	miRNA	miR-185
t0084517	22	1	TGGAGAGAAAGGCAGTTCTTGC	miRNA	miR-185
t0084527	22	1	TGGAGAGGAAGGCCGTTCCTGA	miRNA	miR-185
t0084719	21	1	TGGAGAGAAAGGCAATTGCTG	miRNA	miR-185
t0084736	23	1	TGGAGAGAAGGGAAGTTCCTGAT	miRNA	miR-185
t0084836	23	1	TGGAGGGAAACGCAGTTCCTGAT	miRNA	miR-185
t0084953	25	1	AGGAGAGAAAGGCAGTTCCTGAAAA	miRNA	miR-185
t0085176	23	1	TGGAGAGAAAGGCAGTTCCTGCC	miRNA	miR-185
t0085557	23	1	TGGAGAGAAAGGCAGTTTCTGCT	miRNA	miR-185
t0086036	22	1	TGGAGAGAAAGGCTGTTACTGA	miRNA	miR-185
t0086198	23	1	TGTAGAGAAAGGCAGTTCCTGCA	miRNA	miR-185
t0086242	22	1	TGGAGAGGAAGGTAGTTCCTGA	miRNA	miR-185
t0086331	23	1	TGGAGGGAAAGGCAGTTCCTTAG	miRNA	miR-185
t0086647	22	1	TGGGGAGAAAGGCAGTTCCTGC	miRNA	miR-185
t0086978	22	1	TGGAGAGAAAAGCATTTCCTGA	miRNA	miR-185
t0087061	23	1	CGGAGAGAAAGGAAGTTCCTGAG	miRNA	miR-185
t0087116	23	1	AGGAGAGAAAGGAAGTTCCTGAG	miRNA	miR-185
t0087217	20	1	TGGAGACAAAGGCAGTTCCG	miRNA	miR-185
t0087823	25	1	TGGGGAGAAAGGCAGTTCCTGAAAA	miRNA	miR-185
t0087876	22	1	TGGAGAGAAAGGAAGTTCCTGG	miRNA	miR-185
t0087880	22	1	CGGAGAGAAAGGGAGTTCCTGA	miRNA	miR-185
t0087925	20	1	TGGAAAGAAAGGCAGTTCCT	miRNA	miR-185
t0088076	22	1	TTGAGAGAAAGGAAGTTCCTGA	miRNA	miR-185
t0088196	18	1	TGGGGAGAAAGGCAGTTG	miRNA	miR-185
t0088325	22	1	CGGAGATAAAGGCAGTTCCTGA	miRNA	miR-185
t0088406	23	1	TGGAGAGAAAGGCAGTTAATGAT	miRNA	miR-185
t0088481	22	1	TGGGGAGAAAGGCAGTTCATGA	miRNA	miR-185
t0088561	20	1	TGGAGAGACAGGCAGTTCCT	miRNA	miR-185
t0088668	18	1	TGGAGGGAAAGGCAGTTC	miRNA	miR-185
t0088738	21	1	TGGAGAGAAAGGCAGTTATTG	miRNA	miR-185
t0088762	18	1	TGGAGAGAAAGGCAGATC	miRNA	miR-185
t0089043	23	1	TGGAGAGAAAGGCAGCTCCTGAG	miRNA	miR-185
t0089128	22	1	TGGAGAGAACGGCAGTTACTGA	miRNA	miR-185
t0089172	18	1	TGGAGAGAAAGGCAGGTG	miRNA	miR-185
t0089262	23	1	TGGAGAGCAAGGCAGTTCCTGGA	miRNA	miR-185
t0089296	22	1	TGAAGCGAAAGGCAGTTCCTGA	miRNA	miR-185
t0089573	24	1	TGGAGAGAAAGGCAGTTCCTGCAT	miRNA	miR-185
t0089644	22	1	ATGGAGAGAAAGGCCGTTCCTG	miRNA	miR-185
t0089666	20	1	GGAGAGAAAGGCAGGTCCTG	miRNA	miR-185
t0089680	22	1	TGGAGAGAGAGGCAGTTCCTGT	miRNA	miR-185
t0090026	22	1	TGGAGATAACGGCAGTTCCTGA	miRNA	miR-185
t0090314	22	1	TGGAGAGAAAGGCAGTTACTAA	miRNA	miR-185
t0090410	22	1	TAGAGAGCAAGGCAGTTCCTGA	miRNA	miR-185
t0090657	20	1	TGGAGAGAAAGCCAGTTCCT	miRNA	miR-185
t0091115	21	1	GGAGAGAAAGGCAGTTCCCGA	miRNA	miR-185
t0091151	20	1	TGGAGAGAAAGGCAGATCCA	miRNA	miR-185
t0091166	21	1	TGGAGAGAAGGGCAGTTCCTA	miRNA	miR-185
t0091294	22	1	CGGAGAGAAAGGAAGTTCCTGA	miRNA	miR-185
t0091362	22	1	TGGAGAGAAAGGCAGTTCATGT	miRNA	miR-185
t0091385	22	1	TGGAGAGAATGGCAGTTCCTTA	miRNA	miR-185
t0091533	22	1	TGGAGAGAAAGGCAGTTCCTCC	miRNA	miR-185
t0091724	25	1	TGGCCGGAGAACTGATAAGGGCATC	miRNA	miR-184-3p
t0091791	25	1	TAGACGGAGAACTGATAAGGGCATC	miRNA	miR-184-3p
t0091951	25	1	TGGACGGAGAACTGATAAGGGCGTC	miRNA	miR-184-3p
t0092229	25	1	TGGACGGGGAACTGATAAGGGCATC	miRNA	miR-184-3p
t0092307	25	1	TGGACGGAGCACTGATAAGGGAATC	miRNA	miR-184-3p
t0092397	25	1	TGGACGGAGAGCTGATAAGGGTATC	miRNA	miR-184-3p
t0092421	24	1	TGGACGGTGAACTGATAAGGGCTT	miRNA	miR-184-3p

t0092564	25	1	TGGACGGAGAACTGATAAGGGAATT	miRNA	miR-184-3p
t0092762	25	1	TGGACGGAGAACTGATAAGGGAATA	miRNA	miR-184-3p
t0092931	22	1	TGGACGGAGAACTGATTGGGGC	miRNA	miR-184-3p
t0092955	25	1	TGGACGGAGAACTGATAAGGGGATC	miRNA	miR-184-3p
t0092962	27	1	TGGACGGAGAACTGATAAGGGTATATC	miRNA	miR-184-3p
t0092977	25	1	TGGACGGAGAACTGATACGGGCATC	miRNA	miR-184-3p
t0040807	25	1	TGGACGGAGAACTGATAAGGGCATA	miRNA	miR-184-3p
t0042959	25	1	TGGAAGGAGAACTGATAAGGGAATC	miRNA	miR-184-3p
t0043122	25	1	TGGACGGAGGACTGATAAGGGAATC	miRNA	miR-184-3p
t0053378	23	1	TAGACGGAGAACTGATAAGGGCA	miRNA	miR-184-3p
t0060099	24	1	TGGACGGAGAACTGATTAGGGATC	miRNA	miR-184
t0065630	22	1	TGGACGGAGAACTGATAAGGGA	miRNA	miR-184
t0066870	24	1	TGGACGGAGAACTGATAAGGGATT	miRNA	miR-184
t0072955	24	1	TGGAAGGAGAACTGATAAGGGATC	miRNA	miR-184
t0075033	24	1	TGGACGGAGAACTGATGAGGGATC	miRNA	miR-184
t0079384	20	1	TGGACGGAGAATTGATAGGG	miRNA	miR-184
t0079849	23	1	TGGACGGAGAACTGATACGGGAA	miRNA	miR-184
t0083092	24	1	TGCACGGAGAACTGATAAGGGATC	miRNA	miR-184
t0084277	20	1	TGGACGGCGAACTGATAGGG	miRNA	miR-184
t0084937	21	1	AGACCTACTTGTCTACCAACA	miRNA	miR-1839-3p
t0089625	21	1	AGACCTACTTATCTACCCACA	miRNA	miR-1839-3p
t0089759	21	1	AGACCTACTTATTTACCAACC	miRNA	miR-1839-3p
t0092301	22	1	AGACCTACTTATCTCCCAACAG	miRNA	miR-1839-3p
t0044513	21	1	AAACCTACTTATCTACCAACA	miRNA	miR-1839-3p
t0045625	21	1	AGACCTAGTTATCTACCAACA	miRNA	miR-1839-3p
t0046807	23	1	AAGGAAGATAGAACAGGTCTTGA	miRNA	miR-1839
t0050262	20	1	AAGGTAGATAGGACAGGTCT	miRNA	miR-1839
t0050420	22	1	AAGGTAGATGGAACAGGTCTTG	miRNA	miR-1839
t0063893	22	1	AGGGAGATAGAACAGGTCTTGT	miRNA	miR-1839
t0078381	22	1	AAGGTAGATAGAACAGGACTTG	miRNA	miR-1839
t0078920	21	1	AGGTAGATAGAATAGGTCTTG	miRNA	miR-1839
t0091472	22	1	AGGTAGATAGAACAGGTCTTGG	miRNA	miR-1839
t0048476	22	1	AAGGTAGCTAGAACAGGTCTTG	miRNA	miR-1839
t0050724	21	1	AGGTAGAAAGAACAGGTCTTG	miRNA	miR-1839
t0053018	23	1	AAAGGTAGATAGAACAGATCTTG	miRNA	miR-1839
t0055372	22	1	AAGGTAGATAGCACAGGTTTTG	miRNA	miR-1839
t0066230	22	1	AGGTAGATAGAAAAGGTCTTGT	miRNA	miR-1839
t0077625	20	1	AAGGTAGATAGAACAGGCCT	miRNA	miR-1839
t0041702	21	1	AGGTAGATATAACAGGTCTTG	miRNA	miR-1839
t0042119	21	1	AGGGAGATAGAACAGGGCTTG	miRNA	miR-1839
t0043551	23	1	AAAGGTAGATAGAACAGGTCCTG	miRNA	miR-1839
t0045176	22	1	AGGTAGATAGAACGGGTCTTGT	miRNA	miR-1839
t0045903	21	1	AGGGAGATACAACAGGTCTTG	miRNA	miR-1839
t0046193	23	1	AGGTAGATAGAACAGGGCTTGTT	miRNA	miR-1839
t0046449	22	1	AGGTAGATAGAACAGGTCTTAA	miRNA	miR-1839
t0046529	21	1	AGGTAGATAGACCAGGTCTTG	miRNA	miR-1839
t0047304	21	1	AGGTAGATAGAAGAGGTCTTG	miRNA	miR-1839
t0047308	20	1	AGTAGATAGAACAGGTCTTG	miRNA	miR-1839
t0047311	21	1	AAGGTAGATAGAACAGGGCTT	miRNA	miR-1839
t0048012	22	1	AAGGCAGATAGAACAGGTTTTG	miRNA	miR-1839
t0050257	23	1	AAGGTGGATAGAACAGGTCTTGA	miRNA	miR-1839
t0050704	24	1	AAAGGTAGATAGAACAGGGCTTGA	miRNA	miR-1839
t0050869	22	1	AAGGTAGATAGAACACGTCTTG	miRNA	miR-1839
t0050995	23	1	AAAGGTAGATAGGACAGGTCTTG	miRNA	miR-1839
t0051675	22	1	AAGGTAGATAGCACAGGTCTTG	miRNA	miR-1839
t0052878	23	1	AGGTAAATAGAACAGGTCTTGTT	miRNA	miR-1839
t0053062	23	1	AAGGTAAATAGAACAGGTCTTGA	miRNA	miR-1839
t0053656	22	1	AAGGAAGATAGAACAAGTCTTG	miRNA	miR-1839

t0053930	22	1	AGGTAGATAGAATAGGTCTTGT	miRNA	miR-1839
t0054517	21	1	AGGTAGATAGAACAGATCTTG	miRNA	miR-1839
t0054694	22	1	AAGGTAGATTGAACAGGTCTTG	miRNA	miR-1839
t0055634	22	1	AGGTAGATAGAACAGGACTTGA	miRNA	miR-1839
t0055901	22	1	AAGGGAGATAGAACAGGTTTTG	miRNA	miR-1839
t0056742	22	1	AAGGTAGATAGAACCGGTCTTG	miRNA	miR-1839
t0057154	22	1	AAGGAAGATAGAACATGTCTTG	miRNA	miR-1839
t0057754	20	1	AAGGTAGATAGAACCGGTCT	miRNA	miR-1839
t0058355	21	1	TGGTAGATAGAACAGGTCTTG	miRNA	miR-1839
t0060644	22	1	AAGGTAGATACAACAGGTCTTG	miRNA	miR-1839
t0060721	20	1	AAGGTAGATAAAACAGGTCT	miRNA	miR-1839
t0060724	22	1	AAGGTAGATAGAACAGTTCTTG	miRNA	miR-1839
t0060917	23	1	AAAGGTAGATAGAACAGGTCTGG	miRNA	miR-1839
t0061305	22	1	AGGTGGATAGAACAGGTCTTGA	miRNA	miR-1839
t0064795	20	1	AAGGTAGATAGACCAGGTCT	miRNA	miR-1839
t0064928	22	1	AGGTAGATAGAACAGGTCTAGT	miRNA	miR-1839
t0066270	22	1	AAGGTCGATAGAACAGGTCTTG	miRNA	miR-1839
t0067213	22	1	AAGGTGGATAGAACAGGTCTTG	miRNA	miR-1839
t0067760	21	1	AGGTAGATAGAACCGGTCTTA	miRNA	miR-1839
t0069325	22	1	AAGGTAGATAGAACAGGTCATG	miRNA	miR-1839
t0069605	21	1	AGGTAGCTAGAACAGGTCTTG	miRNA	miR-1839
t0070024	21	1	AAGTAGATAGAACAGGTCTTG	miRNA	miR-1839
t0070334	22	1	AAGGTAGATAGGACAGGTCTTG	miRNA	miR-1839
t0070754	18	1	AAGGTAGATAGAACCGGT	miRNA	miR-1839
t0071121	21	1	AGGTAGATAGGACAGGTCTTG	miRNA	miR-1839
t0071366	22	1	AAGGTAGATAGAACATGTCTTG	miRNA	miR-1839
t0071649	22	1	AAGGTAGAAAGAACAGGTCTTG	miRNA	miR-1839
t0072011	24	1	AAAGGGAGATAGAACAGGTCTTGA	miRNA	miR-1839
t0073020	22	1	AGGTAGATAGAACTGGTCTTGT	miRNA	miR-1839
t0073401	21	1	AGGTAGATAGAACAGGTCATG	miRNA	miR-1839
t0075032	22	1	AGGTAGATAGAACAGGTCTTGC	miRNA	miR-1839
t0076516	21	1	AGGTAGATAAAACAGGTCTTG	miRNA	miR-1839
t0076755	22	1	AAAGTAGATAGAACAGGTCTTG	miRNA	miR-1839
t0076881	21	1	AGGGAGATAGAACAGGTCTTA	miRNA	miR-1839
t0076964	22	1	AAGATAGATAGAACAGGTCTTG	miRNA	miR-1839
t0077968	22	1	AGGTAAATAGAACAGGTCTTGT	miRNA	miR-1839
t0078462	18	1	AAGGGAGATAGAACAGGT	miRNA	miR-1839
t0079408	22	1	AGATAGATAGAACAGGTCTTGT	miRNA	miR-1839
t0079544	23	1	AAGGCAGATAGAACAGGTCTTGA	miRNA	miR-1839
t0081632	21	1	AGGTAGATAGAACGGGTCTTG	miRNA	miR-1839
t0081678	22	1	AAGGTAGATAGGACAGGTTTTG	miRNA	miR-1839
t0082488	23	1	AAAGGTAGATAGATCAGGTTTTG	miRNA	miR-1839
t0083272	21	1	AAGGGAGATAGAACAGGTCTT	miRNA	miR-1839
t0083657	20	1	TGAATTACCGAAGGGCCCTA	miRNA	miR-183*
t0084522	20	1	TGAATTACCGAAGGGACATA	miRNA	miR-183*
t0084822	19	1	TATGGAACTGGTAGAATTA	miRNA	miR-183
t0085394	19	1	TATGGCACTGGTAGAACTC	miRNA	miR-183
t0088184	21	1	TATGGCACTGGTAGAATTCCC	miRNA	miR-183
t0088903	22	1	TGTGGCACTGGTAGAATTCACT	miRNA	miR-183
t0089907	28	1	TATGGCACTTGTAGAATTCACTAAACCG	miRNA	miR-183
t0091447	24	1	TATGGCACTGGTAGAATCCACATC	miRNA	miR-183
t0092119	27	1	ATGGCACTGGTAGAATTCCCTTCGTAT	miRNA	miR-183
t0092319	20	1	CATGGCACTGGTAGAATTCA	miRNA	miR-183
t0092384	22	1	TATGGCACTGGTAGAATTAACT	miRNA	miR-183
t0092957	20	1	TATGGCACTGGTAGAATTAA	miRNA	miR-183
t0093129	20	1	TATGGCACCGGTAGAATTCA	miRNA	miR-183
t0055114	24	1	ATGGCACTGGTAGAATTCACTACC	miRNA	miR-183
t0092422	22	1	TGAGGGAGTACATTGTATAGTT	miRNA	miR-1827

t0041422	22	1	TGAGGAAGTAGATTGTATAGTC	miRNA	miR-1827
t0044889	21	1	TGAGGGAGTAGATTGTTAGTT	miRNA	miR-1827
t0049438	22	1	TGAGGGAGTAGATTGTATAGTC	miRNA	miR-1827
t0068435	22	1	TGAGGGAGTATATTGTATAGTT	miRNA	miR-1827
t0068838	22	1	TGAGGAAGAAGATTGTATAGGA	miRNA	miR-1827
t0077039	23	1	TGAGGGAGTAGATTGTATACTTG	miRNA	miR-1827
t0080125	20	1	TGGGAGTAGATTGTATAGTT	miRNA	miR-1827
t0084931	22	1	TGAGGGAGAAGATTGGATAGTT	miRNA	miR-1827
t0085310	21	1	TGAGGGAGTAGAGTGTATAGT	miRNA	miR-1827
t0087518	21	1	AGGGAGTAGATTGTATAGTTG	miRNA	miR-1827
t0087819	21	1	CGAGGGAGTAGGTTGTATAGT	miRNA	miR-1827
t0089974	22	1	TGAGGGAGTGGGTTGTATAGTT	miRNA	miR-1827
t0040786	20	1	TGAGGGAGTAGATTGTGTCG	miRNA	miR-1827
t0040855	21	1	TGAGGGAGTAGATCGTATAGT	miRNA	miR-1827
t0041108	22	1	TGGGGGGGTAGATTGTATAGTT	miRNA	miR-1827
t0041541	23	1	TGAGGGAGTAGGTTGTATAGTAA	miRNA	miR-1827
t0041717	22	1	TGAGGGAGTAGATTGTGCAGTT	miRNA	miR-1827
t0041752	22	1	TGAGGGAGTAGACTGTATAGTT	miRNA	miR-1827
t0042574	21	1	TGGGGGAGTAGGTTGTATAGT	miRNA	miR-1827
t0042944	20	1	TGAGGGACTAGATTGTATAG	miRNA	miR-1827
t0043144	24	1	TGAGGGAGTAGGTTGTATGGTTAG	miRNA	miR-1827
t0043210	19	1	TGAGGGAGTAGATTGCAGT	miRNA	miR-1827
t0043681	20	1	TGAGGAAGTAGATTGTATTT	miRNA	miR-1827
t0044421	22	1	ATGAGGGAGTAGATTGTATAGT	miRNA	miR-1827
t0045033	21	1	TGAGGGAGTAGATTGTAGTTT	miRNA	miR-1827
t0045054	22	1	TGTGGGAGTAGATTGTATAGTT	miRNA	miR-1827
t0045081	21	1	TGAGGGAGTAGATTTTATAGT	miRNA	miR-1827
t0045207	22	1	CTGAGGGAGTAGATTGTATAGT	miRNA	miR-1827
t0045743	24	1	TGAGGGAGTAGATTGTATAGTTAT	miRNA	miR-1827
t0045810	19	1	TGGGGGAGTAGATTGTATA	miRNA	miR-1827
t0047344	22	1	TAAGGGAGTAGATTGTATAGTT	miRNA	miR-1827
t0047748	23	1	TGAGGGAGTAGATTGAATAGTTG	miRNA	miR-1827
t0047879	20	1	TGAGGGAGTAGAGTGTATAG	miRNA	miR-1827
t0048807	19	1	TGGGGAGTAGATTGTATAG	miRNA	miR-1827
t0048828	23	1	TGAGGCAGTAGATTGTATAGGTG	miRNA	miR-1827
t0048986	26	1	TGAGGGAGTAGGTTGTATGGTTATCT	miRNA	miR-1827
t0049286	21	1	TGAGGGAGTAGATTGTATGTT	miRNA	miR-1827
t0049770	20	1	TGAGGAAGTAGATTGTATAC	miRNA	miR-1827
t0050728	23	1	TGAGGGAGTAGATTGTATAGTTC	miRNA	miR-1827
t0051085	21	1	TGAGGGAGTAGATCGTATAGA	miRNA	miR-1827
t0051458	20	1	TGAGGGAGTAGATTGTGGAG	miRNA	miR-1827
t0051468	21	1	TGAGGGAGTAGATTGGATAGG	miRNA	miR-1827
t0052159	20	1	AGGCAGTAGATTGTATAGTG	miRNA	miR-1827
t0053474	23	1	TGAGGGAGTAGGTTGTATGGTTA	miRNA	miR-1827
t0054038	21	1	TGAGGGAGTAGATTGTAGAGT	miRNA	miR-1827
t0054170	21	1	TGAGGGAGTAGATTGTATGGT	miRNA	miR-1827
t0054777	20	1	TGAGGGAGTAGATTGTATAT	miRNA	miR-1827
t0055200	20	1	TGAGGGAGTAGATTGGATAG	miRNA	miR-1827
t0055369	20	1	TGAGGGAGTAGTTTGTGTTG	miRNA	miR-1827
t0055384	22	1	TTGAGGGAGTAGATTGTATAGT	miRNA	miR-1827
t0056552	21	1	TGAGGGAATAGATTGAATAGT	miRNA	miR-1827
t0056656	24	1	TGAGGGAGTAGGTTGTATGGAATC	miRNA	miR-1827
t0057072	19	1	TAGGGAGTAGATTGTATAG	miRNA	miR-1827
t0057198	20	1	TGAGGGAGTAGATTGCATGT	miRNA	miR-1827
t0057361	22	1	TGAGGGAGTAGATTGTGCTGTT	miRNA	miR-1827
t0057782	21	1	TGAGAGAGTAGATTGTATAGT	miRNA	miR-1827
t0058605	25	1	TGAGGGAGTAGGTTGTATGGCTATC	miRNA	miR-1827
t0058650	21	1	TGAGGGAGGAGATTGTATTGT	miRNA	miR-1827

t0059002	21	1	TGAGGAAGTAGATTGTATAAT	miRNA	miR-1827
t0059643	21	1	TGAGGGAGTTGATTGTATAGT	miRNA	miR-1827
t0059686	22	1	GAGGGAGTAGATTGTATAGTTT	miRNA	miR-1827
t0060294	25	1	TGAGGGAGTAGATTGTATAGTACGG	miRNA	miR-1827
t0060468	24	1	TGAGGGAGTAGATTGTATAGTTGT	miRNA	miR-1827
t0060749	20	1	TGAGGGAGCAGATTGTATAG	miRNA	miR-1827
t0060795	20	1	TGGGGTAGTAGATTGTATTT	miRNA	miR-1827
t0061318	20	1	TGAGGGAGTAGATTGAATAC	miRNA	miR-1827
t0061783	20	1	TGGGGGAGTAGATTGTATAG	miRNA	miR-1827
t0061826	21	1	TGAGGGAGTAGATAGTATAGT	miRNA	miR-1827
t0063186	22	1	TGAGGGAGTAGGTTGTATATTT	miRNA	miR-1827
t0063681	22	1	TGAGGGAGTAGATTGGCTAGTT	miRNA	miR-1827
t0064813	22	1	TGAGGAAGTAGATTGTATAGTG	miRNA	miR-1827
t0064918	24	1	TGAGGGAGGAGATTGTATAGTTAA	miRNA	miR-1827
t0065506	23	1	TGAGGGGGTAGATTGTATAGTTA	miRNA	miR-1827
t0065534	23	1	TGAGGGAGGAGATTGTATAGTTT	miRNA	miR-1827
t0066184	22	1	TGAGGGAGTAGATGGTATAGTT	miRNA	miR-1827
t0066533	22	1	TGAGGGAGTAGTTTGTACACTT	miRNA	miR-1827
t0066693	18	1	TGAGGGAGTAGATTATAG	miRNA	miR-1827
t0066989	21	1	TCAGGGAGTAGATTGTATAGT	miRNA	miR-1827
t0067610	22	1	TGAGGGACTAGATTGTATAGTT	miRNA	miR-1827
t0068119	22	1	TGAGGGGTTAGATTGTATAGTT	miRNA	miR-1827
t0068773	21	1	TGGGGTAGTAGATTGTATGTT	miRNA	miR-1827
t0068806	25	1	TGAGGGAGTAGATTGTATAGTTATC	miRNA	miR-1827
t0069392	23	1	CTGAGGGAGTAGATTGTATAGTT	miRNA	miR-1827
t0069856	21	1	TGAGGGAGTAGATTGTATAAC	miRNA	miR-1827
t0070572	20	1	TGCGGGAGTAGATTGTATAG	miRNA	miR-1827
t0070641	20	1	TGAGGGAGTAGGTTGGATAG	miRNA	miR-1827
t0070836	20	1	TGAGGGAGTAGATTGTATGT	miRNA	miR-1827
t0070987	20	1	TGAGGGAAGAGATTGTATAG	miRNA	miR-1827
t0071113	18	1	TGAGGGAGTAGGTTGTAT	miRNA	miR-1827
t0071482	27	1	TGAGGGAGTAGATTGTATAGTGGCCGA	miRNA	miR-1827
t0071483	20	1	TGAGGGAGTAGTTTGTATAG	miRNA	miR-1827
t0071753	20	1	TGAGGGAGTAGATTGTACTT	miRNA	miR-1827
t0072457	21	1	TGAGGGAGTAGTTTGTACAGG	miRNA	miR-1827
t0072973	21	1	TGAGGGAGTAGATTGTAATGT	miRNA	miR-1827
t0073352	21	1	TGAGGGAGTAGGTTGTTTAGT	miRNA	miR-1827
t0073385	22	1	TGACGGAGTAGATTGTATAGTT	miRNA	miR-1827
t0073413	22	1	TGAGGGAGTAGATAGTATAGTT	miRNA	miR-1827
t0073772	22	1	TGCGGGAGTAGGTTGTATAGTT	miRNA	miR-1827
t0073825	21	1	TGAGGGAGTAGATTGTGTGGT	miRNA	miR-1827
t0074776	20	1	TGAGGGAGTAGATTGCATAG	miRNA	miR-1827
t0075120	22	1	TGAGGCAGTAGATTGTATCGTT	miRNA	miR-1827
t0075153	19	1	TGAGGGAGTAGATTGTATT	miRNA	miR-1827
t0075980	22	1	TGAGGGTGTAGATTGTATAGTT	miRNA	miR-1827
t0076211	20	1	TGAGGGAGTAGATTGTGCAG	miRNA	miR-1827
t0076592	24	1	TGAGGGAGTAGGTTGTATGGTATC	miRNA	miR-1827
t0077015	22	1	TGAGGGAGTAGTTTGTGTTGTT	miRNA	miR-1827
t0078498	22	1	TGAGGAAGAAGATTGTATAGGT	miRNA	miR-1827
t0078665	21	1	TGAGGTCGTAGATTGTATGTT	miRNA	miR-1827
t0078672	19	1	TGAGGGAGTAGGTTGTAGT	miRNA	miR-1827
t0079049	22	1	TGAGGGAGTAGATTCTATAGTT	miRNA	miR-1827
t0079099	21	1	GAGGAAGTAGATTGTATAGGT	miRNA	miR-1827
t0080304	21	1	TGACGGAGTAGATTGTATAGT	miRNA	miR-1827
t0080415	21	1	TAAGGGAGTAGATTGTATAGT	miRNA	miR-1827
t0080714	21	1	TGAGGGAGTAGCTTGTATAGT	miRNA	miR-1827
t0083554	20	1	TGAAGTAGTAGATTGTATGT	miRNA	miR-1827
t0084052	22	1	TGAGGCAGTAGATTGTATAGCT	miRNA	miR-1827

t0084895	24	1 TGAGGGAGTAGATTGTATAGTTAA	miRNA	miR-1827
t0085284	20	1 TGAGGGAGTAGATTCTATAG	miRNA	miR-1827
t0086468	20	1 TGAGGGAGTAGATTTTATAG	miRNA	miR-1827
t0086684	21	1 TGAGGGAGTTGGTTGTATAGT	miRNA	miR-1827
t0087885	22	1 TGAGGGAGTAGATTGAATAGTA	miRNA	miR-1827
t0088080	20	1 TGAGGGAGTAGATTGTATTG	miRNA	miR-1827
t0088711	22	1 TGAGGAAGTAGATTGTATTGTT	miRNA	miR-1827
t0089056	22	1 GGAGGGAGTAGATTGTATAGTT	miRNA	miR-1827
t0089269	21	1 TGAGGGAGTAGATTGGATAGT	miRNA	miR-1827
t0089486	22	1 TGAGGGAGTAGATTGTATAGAT	miRNA	miR-1827
t0089720	20	1 AGGGAGTAGATTGTATAGTT	miRNA	miR-1827
t0089830	21	1 TGAGGGAGAAGATTGGATAGT	miRNA	miR-1827
t0089984	22	1 TTTGGCAATGGTAGAAATCACA	miRNA	miR-182
t0090176	20	1 TTTGGCATTGGTAGAACTCA	miRNA	miR-182
t0090338	28	1 TTTGGCAATGGTAGAACTCACACCGATA	miRNA	miR-182
t0090434	22	1 TTTGACAATGGTAGAACTCACA	miRNA	miR-182
t0090939	20	1 TTTAGCAATGGTAGAACTCA	miRNA	miR-182
t0090960	27	1 TTTGGCAATGGTAGAACTCACACTAAA	miRNA	miR-182
t0091674	27	1 TTTGGCAATGGTAGAAATCACACTGGA	miRNA	miR-182
t0091752	22	1 TTTGGCAATGGTAGAATTCACA	miRNA	miR-182
t0092069	20	1 TTTGGCAATGGTAGGACTCA	miRNA	miR-182
t0092372	22	1 TTTGGCAATGGTAGAACTTACA	miRNA	miR-182
t0092608	24	1 TTTGGCAATGGAAGAACTTACACT	miRNA	miR-182
t0092610	27	1 TTTGGCAATGGTAGAACTCACCCTGAT	miRNA	miR-182
t0093525	20	1 TTTGGCAATGGTAGAACCCA	miRNA	miR-182
t0044828	22	1 TTTGGCAATGGTGGAACTCACA	miRNA	miR-182
t0046600	29	1 TTTGGCAATGGTAGAAATCACACTGGATC	miRNA	miR-182
t0048996	23	1 TTTGGCAATGGTAGAACTCAAAC	miRNA	miR-182
t0049755	20	1 TTTGGCAATGGAAGAATTCA	miRNA	miR-182
t0050035	22	1 TGTGGCAATGGTAGAACTCACA	miRNA	miR-182
t0054396	24	1 TTTAGCAATGGTAGAACTCACACT	miRNA	miR-182
t0056991	22	1 TTTGGCAATGCTAGAACTCACA	miRNA	miR-182
t0062474	24	1 TTTGGCAATGGTAGAACTAACACT	miRNA	miR-182
t0066157	22	1 ACATTCATTGCTGTCGGTGGGC	miRNA	miR-181b
t0070878	22	1 ACATTCATTGCTGTCGGGGGGG	miRNA	miR-181b
t0078312	26	1 AACATTCATTGCTGTCGGTGGGAATC	miRNA	miR-181b
t0079029	23	1 AACATTCATTGCTGTCGGGGGGT	miRNA	miR-181b
t0080778	20	1 AACATTCATTGATGTCGGTG	miRNA	miR-181b
t0081043	23	1 AACATTCATTGCTGTCGGTGGGC	miRNA	miR-181b
t0082001	24	1 AACATTCATTGTTGTCGGTGGCTC	miRNA	miR-181b
t0082476	19	1 AACATTCATTGGTGTCGGT	miRNA	miR-181b
t0087051	23	1 ACATTCATTGCTGTCGGTGGGGT	miRNA	miR-181b
t0087668	20	1 TCATCACATTCATTACTGTC	miRNA	miR-181b
t0091445	19	1 AACATTCATTGCTGGCGGT	miRNA	miR-181b
t0091676	24	1 AACATTCATTGTTGTCGGTGGGGA	miRNA	miR-181b
t0092203	23	1 AACATTCATTGCTGTCGGTGGGG	miRNA	miR-181b
t0044514	22	1 ACATTCATTGCTGTCGGGGGGT	miRNA	miR-181b
t0047687	22	1 AACATTCATTGCTGTCGGGGGG	miRNA	miR-181b
t0049644	23	1 AACATTCATTGCCGTCGGTGGGA	miRNA	miR-181b
t0050220	24	1 AACATTCACTGCTGTCGGTGGATC	miRNA	miR-181b
t0052095	23	1 GCATTCATTGCTGTCGGTGGGTT	miRNA	miR-181b
t0053831	22	1 ACATTCATTGCTGTCGGCGGGT	miRNA	miR-181b
t0053881	23	1 AACATTCATTGCTGACGGTGGGT	miRNA	miR-181b
t0053988	24	1 AACATTCATTGCTGTCGGGGGATC	miRNA	miR-181b
t0059469	20	1 GACATTCATTGCTGTCGGTG	miRNA	miR-181b
t0060977	19	1 ACCACTGACTGTTGACTGT	miRNA	miR-181a-2*
t0061467	20	1 ACCACTGACCCTTGACTGTA	miRNA	miR-181a-2*
t0062997	20	1 AACACTGACCGTTGACTGTA	miRNA	miR-181a-2*

t0065218	19	1	ACCACTGACCGCTGACTGT	miRNA	miR-181a-2*
t0065253	19	1	ATCACTGACCGTTGACTGT	miRNA	miR-181a-2*
t0065746	18	1	ACCACTGAACGTTGACTG	miRNA	miR-181a-2*
t0066384	44	1	ACCACTGACCGTTGACTGTAACTTTGAGC	miRNA	miR-181a-2*
t0069285	19	1	ACCACTGAACGTTGACTGT	miRNA	miR-181a-2*
t0070605	20	1	ACCACTGACCGGTGACTGTA	miRNA	miR-181a-2*
t0083257	22	1	ATCATTCAACGCTGTCGGTGAG	miRNA	miR-181a
t0085507	24	1	AACATTCAACGCTGTCGGTGAATA	miRNA	miR-181a
t0089131	25	1	AACATTCAACGTTGTCGGTGAGTTT	miRNA	miR-181a
t0092052	23	1	AACATTCAACGCTGTCGGGGAGT	miRNA	miR-181a
t0046288	24	1	AACATTCAACGCTGTCGGCGAGTT	miRNA	miR-181a
t0046513	24	1	AACATTCAACGCTGACGGTGAATA	miRNA	miR-181a
t0049417	22	1	AACATTCAACGCTGTCGGCGAG	miRNA	miR-181a
t0051901	22	1	AACATTCAACGATGTCGGGGAG	miRNA	miR-181a
t0060325	22	1	AACATTCAACGCTGGCGGTGAG	miRNA	miR-181a
t0064624	22	1	AACATTCAACGCTGTCGGTGAC	miRNA	miR-181a
t0079762	22	1	AACATTCAACGCTGTTGGTGAG	miRNA	miR-181a
t0091175	23	1	AACATTCAACGCTGTCGGAGAGG	miRNA	miR-181a
t0091776	20	1	AACATTCAACGCTGCCGGTG	miRNA	miR-181a
t0041333	21	1	AACATTCAACGCTGTCGGTGG	miRNA	miR-181a
t0043798	24	1	AACATTCCACGCTGTCGGTGAGTT	miRNA	miR-181a
t0044569	21	1	AACATTCAACACTGTCGGTTA	miRNA	miR-181a
t0046958	20	1	AACATTAAACGCTGTCGGTG	miRNA	miR-181a
t0047098	22	1	AACATTCAACACTGTCGGTGAG	miRNA	miR-181a
t0047795	19	1	AACATTCAACGCTGGCGGT	miRNA	miR-181a
t0049371	22	1	AACATTGAACGCTGTCGGTGAG	miRNA	miR-181a
t0050814	22	1	ACATTCAACGCTGTCGGGGAGT	miRNA	miR-181a
t0052008	22	1	AACATTCAACGCTGTAGGTGAG	miRNA	miR-181a
t0052218	23	1	AACATTCAACGCTGTCGGTGCGT	miRNA	miR-181a
t0055620	19	1	AACATTCAAAGCTGTCGGT	miRNA	miR-181a
t0056100	21	1	AACATTCAACGCTGTCAGTGA	miRNA	miR-181a
t0057196	24	1	AACATTCAACGCTGTCGGTGCGTT	miRNA	miR-181a
t0058206	22	1	AACCTTCAACGCTGTCGGTGAG	miRNA	miR-181a
t0058225	22	1	ACATTCAACGCTGTCGGCGAGT	miRNA	miR-181a
t0062873	19	1	AACACTCAACGCTGTCGGT	miRNA	miR-181a
t0066466	21	1	AACATTCAACGCTGTCGGTTC	miRNA	miR-181a
t0068743	19	1	GACATTCAACGCTGTCGGT	miRNA	miR-181a
t0069481	19	1	AACATTCAACGCTGTCGGG	miRNA	miR-181a
t0070418	19	1	AACATTCAACGCTGTCGGA	miRNA	miR-181a
t0071147	22	1	AAAATTCAACGCTGTCGGTGAG	miRNA	miR-181a
t0071681	22	1	AACATTCAACGCCGTCGGTGAG	miRNA	miR-181a
t0074928	22	1	AACATTCAACGCTGTCGGTTAG	miRNA	miR-181a
t0075089	20	1	AACATTCAGCGCTGTCGGTG	miRNA	miR-181a
t0077536	22	1	AATATTCAACGCTGTCGGTGAG	miRNA	miR-181a
t0077764	19	1	AACATTCAACGATGTCGGT	miRNA	miR-181a
t0078337	24	1	CAAAGTGCTTATAGTGCAGGTAAA	miRNA	miR-17a
t0080159	24	1	CAAAGTGCTTACAGTGCAGGTCGA	miRNA	miR-17a
t0080495	23	1	TCGGGGAGGCGGCGGCGGCGGGG	miRNA	miR-1777a
t0083137	21	1	TCGGGGCGGCGGCGGCGGTGG	miRNA	miR-1777a
t0083602	20	1	TCGGGGCGGCGGCGGCGGAG	miRNA	miR-1777a
t0084862	20	1	TCGGGGCGGCGGCGGTGGCG	miRNA	miR-1777a
t0086948	18	1	TCGGGGCGCCGGCGGCGG	miRNA	miR-1777a
t0087023	20	1	TCGGGGCGGCGGTGGCGGCG	miRNA	miR-1777a
t0087733	18	1	TCGGGGCGGCGGTGGCGG	miRNA	miR-1777a
t0088059	20	1	TCGGGGCGGAGGCGGAGGCG	miRNA	miR-1777a
t0089005	19	1	TCGGGGCGGCGGCGGGGGC	miRNA	miR-1777a
t0091955	24	1	CGGGTCGGGGCGGCGGCGGCGGCG	miRNA	miR-1777a
t0092171	23	1	TCGGGGCGGAGGCGGAGGCGGGG	miRNA	miR-1777a

t0066630	21	1	TCGGGGCGGCGGCGGCGGGGG	miRNA	miR-1777a
t0075043	25	1	TCGGGGCGGCGGCGGCGGCGGGGGC	miRNA	miR-1777a
t0044211	29	1	TCGGGGCGGCGGCGGCGGCGGGGGCGG	miRNA	miR-1777a
t0044458	23	1	TAGGGACGGGAGCGCGCGGGGGA	miRNA	miR-1763
t0048429	22	1	TCGGTGTATGTGCTTGGCTATC	miRNA	miR-1755
t0055585	22	1	TTGGTGTATGAGCTTGGCTATC	miRNA	miR-1755
t0057847	22	1	ACTGCAGTGAAGGCACTTGTAA	miRNA	miR-17-3p
t0060269	22	1	ACTGCAGTGAAGGCACTTGTAT	miRNA	miR-17-3p
t0063014	21	1	ACTGCAGTGAAGGCACATGTA	miRNA	miR-17*
t0071332	20	1	ACTGCAGTGGAGGCACTTGT	miRNA	miR-17*
t0074907	23	1	ACTGCAGTGAAGGAACTTGTAGA	miRNA	miR-17*
t0077411	23	1	ACTGCAGTGAAGGCGCTTGTAGA	miRNA	miR-17*
t0077921	22	1	ACTACAGTGAAGGCACTTGTAG	miRNA	miR-17*
t0078235	19	1	ACTGTAGTGAAGGCACTTG	miRNA	miR-17*
t0080759	19	1	GAAGTGAAGGCACTTGTAG	miRNA	miR-17*
t0089355	19	1	ACTGCGGTGAAGGCACTTG	miRNA	miR-17*
t0087968	23	1	ACTGCAGGGAAGGCACTTGTAGA	miRNA	miR-17*
t0047732	23	1	ACTCCAGTGAAGGCACTTGTAGA	miRNA	miR-17*
t0054696	22	1	ACTGCAATGAAGGCACTTGTAG	miRNA	miR-17*
t0062542	22	1	ACTGCAGTGAAGGCCCTTGTAG	miRNA	miR-17*
t0079707	19	1	ACTGCAGTGAAGGCACTCG	miRNA	miR-17*
t0041101	19	1	ACTGCAGTGAAGGCAATTG	miRNA	miR-17*
t0046597	19	1	ACTGCCGTGAAGGCACTTG	miRNA	miR-17*
t0049219	23	1	ACTGCAGTGAAGCCACTTGTAGA	miRNA	miR-17*
t0052715	23	1	ACTGCAGAGAAGGCACTTGTAGA	miRNA	miR-17*
t0054299	20	1	ACTGCAGTGAAGGCCCTTGT	miRNA	miR-17*
t0054422	23	1	ACTGCGGTGAAGGCACTTGTAGA	miRNA	miR-17*
t0057967	22	1	AAAGTGCTTACAGTGCCGGTAG	miRNA	miR-17
t0064063	24	1	CAAAGTGCTTACAGTGCGGGTAGT	miRNA	miR-17
t0073674	23	1	CAAAGTGCTTACAGTGCACGTAG	miRNA	miR-17
t0075418	23	1	CAAAGTGCTTGCAGTGCAGGTAG	miRNA	miR-17
t0075554	23	1	CAAGGTGCTTACAGTGCAGGTAG	miRNA	miR-17
t0076694	22	1	AAAGTGCTTAGAGTGCAGGTAG	miRNA	miR-17
t0080914	21	1	TCAAAGTGCTTACAGGGCAGG	miRNA	miR-17
t0082497	24	1	CAAAGTGCTTACAGTGCAGGAAGA	miRNA	miR-17
t0086334	22	1	AAAGTGATTACAGTGCAGGTAG	miRNA	miR-17
t0088467	24	1	CAAGGTGCTTACAGTGCAGGTAGA	miRNA	miR-17
t0092057	23	1	CAAAGTACTTACAGTGCAGGTAG	miRNA	miR-17
t0093070	21	1	CAAAGTGCTTATACTGCAGGT	miRNA	miR-17
t0093474	22	1	AAAGCGCTTACAGTGCAGGTAG	miRNA	miR-17
t0041124	24	1	CAAAGAGCTTACAGTGCAGGTAGA	miRNA	miR-17
t0041880	24	1	CAAAGTGATTACAGTGCAGGTAGA	miRNA	miR-17
t0043651	22	1	GAAAGTGCTTACAGTGCAGGTA	miRNA	miR-17
t0043731	22	1	CAAAGCGCTTACAGAGCAGGTA	miRNA	miR-17
t0044393	22	1	CAAAGTGCTTACAGGGCAGGTA	miRNA	miR-17
t0044606	21	1	CAAAGAGCTTACAGTGCAGGT	miRNA	miR-17
t0044825	20	1	CAAAGTACTTACAGTGCAGG	miRNA	miR-17
t0045259	22	1	ACAAAGTGCTTACAGTGCCGGT	miRNA	miR-17
t0045809	22	1	CAAAGTGCTTACGGTGCAGGTA	miRNA	miR-17
t0052129	23	1	CAAAGTGCTTACAGTGCGGGTAG	miRNA	miR-17
t0058419	22	1	CAAAGTGCTTACCGTGCAGGTA	miRNA	miR-17
t0058548	21	1	CAAAGTGCTTACAGCGCAGGT	miRNA	miR-17
t0058847	21	1	CAAAGTGCTTACAGGGCAGGT	miRNA	miR-17
t0059346	21	1	CAAAGTGCATACAGTGCAGGT	miRNA	miR-17
t0061760	24	1	CAAAGTGTTTACAGTGCAGGTAGA	miRNA	miR-17
t0061888	22	1	AAAGTGCTTACAGTGCAGGGAG	miRNA	miR-17
t0062349	23	1	CAAAGAGCTTACAGTGCAGGTAG	miRNA	miR-17
t0065980	23	1	CAAAGTGCTTATAGTGCAGGTAG	miRNA	miR-17

t0068867	23	1	CAAAGTGCTTACAGTGCAAGTAG	miRNA	miR-17
t0069476	22	1	ACAGTGCTTACAGTGCAGGTAG	miRNA	miR-17
t0069627	22	1	CAAAGTGCTTACAGTGAAGCTA	miRNA	miR-17
t0070098	22	1	CAAAGGGCTTACAGTGCAGGTA	miRNA	miR-17
t0070126	22	1	ACAAAGTGCTTACACTGCAGGT	miRNA	miR-17
t0070191	23	1	CAAAGTGCTTACAGGGCAGGTAG	miRNA	miR-17
t0073763	24	1	CAAAGTGCTTGCAGTGCAGGTAGA	miRNA	miR-17
t0074625	24	1	CAAAATGCTTACAGTGCAGGTAGA	miRNA	miR-17
t0074733	23	1	CAAAGTGCTTACAATGCAGGTAG	miRNA	miR-17
t0075961	22	1	AAGGTGCTTACAGTGCAGGTAG	miRNA	miR-17
t0077247	24	1	CAAAGCGCTTACAGTGCAGGTAGA	miRNA	miR-17
t0077890	22	1	TAGTAGCATGTAAATATTGGCG	miRNA	miR-16c
t0078809	22	1	TAACAGCATGTAAATATTGGCG	miRNA	miR-16c
t0079810	19	1	TAGCACGTAAATATTGGAG	miRNA	miR-16c
t0081811	19	1	TAGCACATAAATATTGGCG	miRNA	miR-16c
t0082156	19	1	CAGCATGTAAGTATTGGCG	miRNA	miR-16c
t0083916	19	1	TAGCATGTAAATATTGGAG	miRNA	miR-16c
t0084414	19	1	TAGCACGTAAATATAGGCG	miRNA	miR-16c
t0086295	19	1	TAGCACGAAAATATTGGCG	miRNA	miR-16c
t0086570	19	1	TAGCACGTAAATATTGACG	miRNA	miR-16c
t0086636	22	1	TAGCAGCACGCAAATATTGGCA	miRNA	miR-16b
t0087262	22	1	TAGCAGGACGTAAATATTTGCA	miRNA	miR-16b
t0090077	24	1	TAGCAGCACGTAAATATTGGCTCT	miRNA	miR-16b
t0091911	22	1	TAGCAGCACGTAAGTATTGGCT	miRNA	miR-16b
t0040723	27	1	TAGCAGCACGTAAATATTCGCAATAAC	miRNA	miR-16b
t0048542	22	1	TAGCAGCACGTATATATTGGCT	miRNA	miR-16b
t0051425	22	1	TAGCAGCACGTAAATATTCGCA	miRNA	miR-16b
t0053442	26	1	TAGCAGCACGTAAATATTGCTCGTGT	miRNA	miR-16b
t0061528	22	1	TCGCAGCACGTAAATATTGGCT	miRNA	miR-16b
t0063787	24	1	TAGCAGCACGTAAATATTGGCATT	miRNA	miR-16b
t0075324	25	1	TAGCAGCACGTAAATATTGGCCAAA	miRNA	miR-16b
t0078017	22	1	TAGCGGCACGTAAATATTGGCA	miRNA	miR-16b
t0085282	22	1	TAGCAGCACGTAAGTATTGGCA	miRNA	miR-16b
t0041459	22	1	TGGCAGCACGTAAATATTGGCA	miRNA	miR-16b
t0042423	23	1	AAGCAGCACGTAAATATTGGCTT	miRNA	miR-16b
t0042842	22	1	TAGCAGCACGTCAATATTGGCA	miRNA	miR-16b
t0044116	22	1	TAGCAGCACATAAATATTGGCT	miRNA	miR-16b
t0046363	22	1	TAGCAGCACGTAAATTTTGGCA	miRNA	miR-16b
t0049602	22	1	TAGCAGCAGGTAAATATTGGCC	miRNA	miR-16b
t0051527	22	1	TAACAGCACGTAAATATTGGCT	miRNA	miR-16b
t0053024	22	1	TAGCAGCACGTAAACATTGGCA	miRNA	miR-16b
t0053165	22	1	TAGCAGAACGTAAATATTGGAA	miRNA	miR-16b
t0054331	22	1	TAGCAGCACGTAAATATTGGAT	miRNA	miR-16b
t0055427	22	1	TAGCAGCACGGAAATATTGGCA	miRNA	miR-16b
t0056805	23	1	TAGCAGCACGTAAATATTGGCCC	miRNA	miR-16b
t0059203	22	1	TAGCAGCACGTAAATGTTGGCA	miRNA	miR-16b
t0061172	22	1	TAGCAGCACGTACATATTGGCT	miRNA	miR-16b
t0061341	22	1	TAGCAGCACGTGAATATTGGCA	miRNA	miR-16b
t0061722	22	1	TAGCAGCACGTAAATATCGGCC	miRNA	miR-16b
t0062101	22	1	TAGCAGCACGTAAATATTGGTC	miRNA	miR-16b
t0062408	22	1	CAGCAGCACGTAAATATTGGCA	miRNA	miR-16b
t0064190	23	1	ATAGCAGCACGTAAATATTGGCT	miRNA	miR-16b
t0064635	22	1	TAGCAGTACGTAAATATTGGCA	miRNA	miR-16b
t0064910	22	1	TAGCAGCACGTAAACATTGGCT	miRNA	miR-16b
t0065047	22	1	TAGCATCACGTAAATATTGGCT	miRNA	miR-16b
t0065076	22	1	TAGCAGCACATAAATATTGGCC	miRNA	miR-16b
t0065457	24	1	TAGCAGCACGTAAATATTGGCAAG	miRNA	miR-16b
t0065548	22	1	TAGCAGCACGTAAATATTGGGA	miRNA	miR-16b

t0065948	22	1	TAGCAGCACGTAAATATGGGCA	miRNA	miR-16b
t0066038	22	1	TAGCAGCACGTAAATCTTGGCA	miRNA	miR-16b
t0067053	22	1	TAGCAGCACGTAACTATTGGCA	miRNA	miR-16b
t0070452	27	1	TAGCAGCACGTAAATATTGGCCATAAC	miRNA	miR-16b
t0071544	22	1	TAGGAGCACGTAAATATTGGCC	miRNA	miR-16b
t0073360	22	1	TAGCTGCACGTAAATATTGGCA	miRNA	miR-16b
t0074378	22	1	TAGCAGCACGTAACTATTGGCC	miRNA	miR-16b
t0076307	22	1	TAGCAGCACGTAAATACTGGCT	miRNA	miR-16b
t0077076	22	1	TAGCACCACGTAAATATTGGCA	miRNA	miR-16b
t0079159	22	1	TAGCAGCACGTTAATATTGGCC	miRNA	miR-16b
t0079907	22	1	AAGCGGCGATGGCGGAGCTGCA	miRNA	miR-1636
t0081392	25	1	AAGCGGCGATGGCGGGGCTGAATTA	miRNA	miR-1636
t0082709	25	1	AAGCGGCGATGGCGGAGCTGGATTA	miRNA	miR-1636
t0084162	20	1	AAGCGGCGATGGCGGGGCTG	miRNA	miR-1636
t0084198	22	1	AAGCGGCGATGGCGGAGATGAA	miRNA	miR-1636
t0085857	24	1	GAAGCGGCGATGGCGGAGCTGGAA	miRNA	miR-1636
t0086276	20	1	AAGCGGCGATGGCGGAGCCG	miRNA	miR-1636
t0087256	24	1	GAAGCGGCGATGGCGGGGCTGAAA	miRNA	miR-1636
t0089356	22	1	CAGCGGCGCTGGCGGGGCTGAA	miRNA	miR-1636
t0091657	24	1	GAAGCGGCGATGGCGGAGATGAAA	miRNA	miR-1636
t0091775	22	1	AAGCGGCGATGGCGGCGCTGAA	miRNA	miR-1636
t0092195	24	1	GAAGCGGCGATGGCGGAGCGGAAA	miRNA	miR-1636
t0092584	22	1	AAGCGGCGATGGCGGAGCTTAA	miRNA	miR-1636
t0042319	20	1	CCAATATTACTATGCTGCTT	miRNA	miR-16-2*
t0047589	20	1	CCAATATTACTGGGCTGCTT	miRNA	miR-16-2*
t0051009	24	1	GCGGGGCGCGGTCGGGCGGCGGCG	miRNA	miR-1607
t0052205	19	1	TGGGGGCGGGGGGCGGTCG	miRNA	miR-1607
t0056583	26	1	TGGGGGCGGGGAGCGGGCGGGCGGTT	miRNA	miR-1607
t0064613	29	1	GGGGGCGGGGAGCGGTCGGGCGGCGGA	miRNA	miR-1607
t0068000	27	1	GGCGGGGAGCGGGCCGGCGGCGGCGGG	miRNA	miR-1607
t0070511	27	1	GGCGGGGAGCGGGCGGGCGGCGGCGGG	miRNA	miR-1607
t0073684	26	1	CTGGGGGCGGGGAGCGGCCGGGCGGC	miRNA	miR-1607
t0074222	28	1	GGCGGGGAGCGGTCGGGCGGCGGCGGG	miRNA	miR-1607
t0078327	26	1	GGGGCGGGGAGCGGTTGGGCGGCGGC	miRNA	miR-1607
t0080036	26	1	GGGGCGGGGAGCGGTCGGGCGGCGGG	miRNA	miR-1607
t0085089	26	1	GCTGGGGGCGGGGAGCGGTCGGGGGG	miRNA	miR-1607
t0063826	26	1	GGGGGCGGGGAGCGGTCGGGCGGGGG	miRNA	miR-1607
t0067483	18	1	GGGGGCGGGGAGAGGTCG	miRNA	miR-1607
t0046483	27	1	GGGCGGGGAGCGGTCGGGCGGCGGCGG	miRNA	miR-1607
t0047205	25	1	GGGGGCGGGGAGCGGTCGGGCGGCG	miRNA	miR-1607
t0048364	30	1	GGGGGCGGGGAGCGGTAGGGCGGCGGC	miRNA	miR-1607
t0052341	21	1	GCGGGGAGCGGTTGGGGGGCG	miRNA	miR-1607
t0053244	24	1	GCGGGGAGCGGTCGGGCAGCGGCG	miRNA	miR-1607
t0057552	28	1	GGCGGGGAGCGGTCGGGCGGCGGCGGT	miRNA	miR-1607
t0060295	28	1	GGGGCGGGGAGCGGCCGGGCGGCGGCG	miRNA	miR-1607
t0060482	27	1	GGCGGGGGGCGGTCGGGCGGCGGCGGT	miRNA	miR-1607
t0060573	22	1	GGGGGCGGGGAGCGGTAGGGCG	miRNA	miR-1607
t0062086	27	1	GCGGCGGGGAGCGGTCGGGCGGCGGCG	miRNA	miR-1607
t0062730	26	1	TGGGGGCGGGGAGCGGGCGGGCGGCG	miRNA	miR-1607
t0066653	26	1	TGGGGGCGGGGAGCGGTAGGGAGGCG	miRNA	miR-1607
t0067231	24	1	GGGGCGGGGAGCGGGCGGGCGGCG	miRNA	miR-1607
t0071911	26	1	TGGGGGCGGGGAGCGGTCGGGCGGGT	miRNA	miR-1607
t0072386	24	1	GGGGCGGGGGGCGGTCGGGCGGCG	miRNA	miR-1607
t0072600	22	1	TAGCGGAACGTAAATATTGGCG	miRNA	miR-16
t0072801	21	1	AGCAGCACGTGAATATTGGCG	miRNA	miR-16
t0075113	21	1	TAGCAGCACGTAAATAATGGT	miRNA	miR-16
t0075921	24	1	TAGCAGCACGTAAATATTCGCGTA	miRNA	miR-16
t0081745	24	1	TAGCAGCACGTAAACATTGGCGAA	miRNA	miR-16

t0083191	22	1	TATCAGCACGGAAATATTGGCG	miRNA	miR-16
t0083583	24	1	TAGCACCACGTAAATATTGGCGAA	miRNA	miR-16
t0084163	22	1	TTGAAGCACGTAAATATTGGCG	miRNA	miR-16
t0086173	22	1	TCGCAGCACGTAAATCTTGGCG	miRNA	miR-16
t0089216	23	1	TAGCAGCACGTACATATTGGCGA	miRNA	miR-16
t0089837	22	1	TAGCAGCGCTTAAATATTGGCG	miRNA	miR-16
t0090035	22	1	TAGCAGGACGTAAATATTGGTG	miRNA	miR-16
t0091933	20	1	TAGCAGCACGTAAATACTGG	miRNA	miR-16
t0040688	22	1	TAGCAGCACGCAAATATTGACG	miRNA	miR-16
t0040708	21	1	AGCAGCACGTAAATGTTGGCG	miRNA	miR-16
t0041001	24	1	TAGCAGCACATAAATATTGGCGTA	miRNA	miR-16
t0041109	22	1	TAGCAGCACGTAATTATTGGTG	miRNA	miR-16
t0041162	24	1	TAGGAGCACGTAAATATTGGCGTA	miRNA	miR-16
t0041368	21	1	AGCAGTACGTAAATATTGGCG	miRNA	miR-16
t0041470	22	1	TAGCAGCACGGAAATCTTGGCG	miRNA	miR-16
t0041516	23	1	TAGCAGCATGTAAATATTGGCGT	miRNA	miR-16
t0041895	22	1	TAGCAGCACGTAAATACTGGTG	miRNA	miR-16
t0041936	21	1	TAGAAGCACGTAAATATTGGA	miRNA	miR-16
t0042076	20	1	TAGCTGCACGTAAATATTGG	miRNA	miR-16
t0042155	23	1	TAGCAGCACGTAAATATAGGCGT	miRNA	miR-16
t0042167	23	1	TAGCATCACGTAAATATTGGCGA	miRNA	miR-16
t0042226	31	1	TAGCAGCACGTAAATATTGGCGAAAAAA[illegible]	miRNA	miR-16
t0042361	22	1	TAGCAGCAAGTAAATTTTGGCG	miRNA	miR-16
t0042371	22	1	TCGCAGCACGTACATATTGGCG	miRNA	miR-16
t0042499	21	1	TAGCAGTACGTAAATATTGGC	miRNA	miR-16
t0042548	22	1	TAGCAGCACGTAAATGTTTGCG	miRNA	miR-16
t0042816	24	1	TAGGAGCACGTAAATATTGGCGAA	miRNA	miR-16
t0043303	20	1	TAGCAGCAAGTAAAAATTGG	miRNA	miR-16
t0043532	20	1	AAGCAGGACGTAAATATTGG	miRNA	miR-16
t0043687	23	1	ATAGCAGCACGTAAATATTAGCG	miRNA	miR-16
t0043948	22	1	TAGAAGCACGTTAATATTGGCG	miRNA	miR-16
t0044268	22	1	TAGCAGCATGTAAATATTAGCG	miRNA	miR-16
t0044293	20	1	TAGCAACACGTAACTATTGG	miRNA	miR-16
t0044578	22	1	TAGCAGCACATAAATATTAGCG	miRNA	miR-16
t0044631	22	1	TAGCAGTACGTAAATATTCGCG	miRNA	miR-16
t0044822	24	1	ATAGCAGCACATAAATATTGGCGT	miRNA	miR-16
t0044882	21	1	AGCAGCACGTAAATATTGGGG	miRNA	miR-16
t0044919	22	1	TAGAAGCACGGAAATATTGGCG	miRNA	miR-16
t0045052	22	1	TAGCAGAACGTAACTATTGGCG	miRNA	miR-16
t0045194	22	1	TAGCAGCACGTATATATTGCCG	miRNA	miR-16
t0045501	23	1	ATAGCAGCTCGTAAATATTGGCG	miRNA	miR-16
t0045525	22	1	TAGCAGCACGTAAGTATTGTCG	miRNA	miR-16
t0045636	20	1	TCGCAGCACGTAAATATTGG	miRNA	miR-16
t0045653	25	1	TAGCAGCACGTAAATATTGGTGTAA	miRNA	miR-16
t0045715	22	1	TAGCCGCACGTAAATCTTGGCG	miRNA	miR-16
t0045726	23	1	TAGCAGCACGTAAATATTCGCGA	miRNA	miR-16
t0045760	24	1	TGGCAGCACGTAAATATTGGCGTT	miRNA	miR-16
t0045776	22	1	TAGCAGCACGCAAATATTCGCG	miRNA	miR-16
t0045898	24	1	TAGCAGCACGTAATTATTGGCGAA	miRNA	miR-16
t0045922	20	1	TAGCAGAACGTAAATATTTG	miRNA	miR-16
t0046023	20	1	TACCAACACGTAAATATTGG	miRNA	miR-16
t0046033	24	1	TAGCAGCGCGTAAATATTGGCGAG	miRNA	miR-16
t0046564	22	1	TAGCAGCGCGTAAATATTGCCG	miRNA	miR-16
t0046817	22	1	TAGCAGTACGGAAATATTGGCG	miRNA	miR-16
t0047053	23	1	TAGCAGCACGTAAATATTGGTGT	miRNA	miR-16
t0047224	24	1	TAGCAGCAAGTAAATATTGGCGAA	miRNA	miR-16
t0047251	22	1	TTGCAGCACGGAAATATTGGCG	miRNA	miR-16
t0047336	22	1	TAGCAGCACGCAACTATTGGCG	miRNA	miR-16

t0047488	24	1	TAGCAGCACGTAAATATTGACGAA	miRNA	miR-16
t0047604	20	1	TAGCAGCACGTAAAGATTGG	miRNA	miR-16
t0047756	19	1	CAGCACGTAAATATTGGCG	miRNA	miR-16
t0047945	22	1	TGGCAGCACGTAAATATTGTCG	miRNA	miR-16
t0048262	24	1	TAGCAACACGTAAATATTGGCGTA	miRNA	miR-16
t0048317	22	1	TAGCAGGATGTAAATATTGGCG	miRNA	miR-16
t0048428	22	1	CAGCTGCACGTAAATATTGGCG	miRNA	miR-16
t0048504	22	1	TAGCAGCAGGCAAATATTGGCG	miRNA	miR-16
t0049097	22	1	CAGCAGCACGTAAATATTGTCG	miRNA	miR-16
t0049217	22	1	TAGCAGCAGGTAAATATTGGTG	miRNA	miR-16
t0049337	22	1	TAGCAGTAGGTAAATATTGGCG	miRNA	miR-16
t0049388	22	1	TAGCAGCACGTAAATATTACCG	miRNA	miR-16
t0049635	22	1	TAGCAGCACGTACATATTGGAG	miRNA	miR-16
t0049885	22	1	TAGCAGCAAGTAAATATTTGCG	miRNA	miR-16
t0049927	20	1	GCAGCACGTAAATATTGGAG	miRNA	miR-16
t0049989	22	1	TAGCATCACGTAAATATTGGGG	miRNA	miR-16
t0050340	20	1	TAGCAGCACGTAACCATTGG	miRNA	miR-16
t0050631	20	1	GCAGCACGTAAACATTGGCG	miRNA	miR-16
t0050879	25	1	TAGCAGCACGTAAATATTGACGAAA	miRNA	miR-16
t0050937	20	1	TAGCAGCACGTAAAAAATGG	miRNA	miR-16
t0050940	22	1	TAGCAGGAAGTAAATATTGGCG	miRNA	miR-16
t0051005	20	1	TACAGCACGTAAATATTGGC	miRNA	miR-16
t0051083	24	1	TAGCAGCACGTAAATGTTGGCGAA	miRNA	miR-16
t0051177	23	1	TAGCAGCACGTAAATATTGACGA	miRNA	miR-16
t0051774	22	1	TAGCAGTACGTAAATATTGCCG	miRNA	miR-16
t0051848	25	1	GAGTAGCAGCACGTAACTATTGGCG	miRNA	miR-16
t0051849	22	1	TAGGAGCAAGTAAATATTGGCG	miRNA	miR-16
t0051890	22	1	TAAGAGCACGTAAATATTGGCG	miRNA	miR-16
t0051973	23	1	TAGCAGCACGTAAACATTGGCGA	miRNA	miR-16
t0052118	22	1	TAGCAGCATGTAAATATAGGCG	miRNA	miR-16
t0052156	25	1	TAGAAGCACGTAAATATTGGCGACA	miRNA	miR-16
t0052484	21	1	TAGCAGCAAGTAAATATTGGC	miRNA	miR-16
t0052838	22	1	TAGCAGCACGTAACTATTGGAG	miRNA	miR-16
t0052897	24	1	TAGCAGCACGTAAATATTGGAGAT	miRNA	miR-16
t0053002	23	1	ATAGCAGCAAGTAAATATTGGCG	miRNA	miR-16
t0053015	23	1	ATAGCAGCACGTAAATAATGGCG	miRNA	miR-16
t0053082	22	1	TAGCAGAACGTACATATTGGCG	miRNA	miR-16
t0053083	22	1	GAGGAGCACGTAAATATTGGCG	miRNA	miR-16
t0053273	22	1	TAACAGCACGTAAATATTTGCG	miRNA	miR-16
t0053387	22	1	TAGCAGCACGTAAATATCGTCG	miRNA	miR-16
t0053655	21	1	AGCAGCACGTAACTATTGGCG	miRNA	miR-16
t0053674	22	1	TAGCAGCACGTCAATGTTGGCG	miRNA	miR-16
t0053766	22	1	TAGCAGCACGAAAATATTTGCG	miRNA	miR-16
t0053848	21	1	AGCCGCCCGTAAATATTGGCG	miRNA	miR-16
t0054227	24	1	TAGCAGCATGTAAATATTGGCGTT	miRNA	miR-16
t0054382	24	1	TAGCAGTAGGTAAATATTGGCGAT	miRNA	miR-16
t0054409	23	1	ATAGCAGCACGTAAGTATTGGCG	miRNA	miR-16
t0054431	21	1	AGCAGCACCTAAATATTGGCG	miRNA	miR-16
t0054583	18	1	TAGCAGAACGTAAATATT	miRNA	miR-16
t0054695	21	1	TAGCGGCACGTAAATATTTGC	miRNA	miR-16
t0054738	21	1	GAGCAGCACGTAAATATTGGC	miRNA	miR-16
t0054741	22	1	TAGCAGCACGTACATATTGTCG	miRNA	miR-16
t0055323	23	1	TAGCAGCACGTAAACATTGGCGT	miRNA	miR-16
t0055838	24	1	TAGCAGAACGTAAATATTGGCGAG	miRNA	miR-16
t0055844	24	1	TAGCAGCACGTCAATATTGGCGAG	miRNA	miR-16
t0055921	23	1	TAGCAGCACGTAAATTTTGGCGT	miRNA	miR-16
t0055941	22	1	TAGCAGCCCGTAAATATTGGTG	miRNA	miR-16
t0056196	22	1	TAGCAGCACTTAAATATTGGAG	miRNA	miR-16

t0056374	22	1	TAGCAGTACGTCAATATTGGCG	miRNA	miR-16
t0056797	22	1	TAGCAGCACCTAAATATTGTCG	miRNA	miR-16
t0056875	22	1	TAGCAGCACGTAAATCTTGGAG	miRNA	miR-16
t0057034	22	1	TAGGAGCACGTAAATGTTGGCG	miRNA	miR-16
t0057281	22	1	GAGCAGCACGTAAATATTGGAG	miRNA	miR-16
t0057292	22	1	TAGCAGACCGTAAATATTGGCG	miRNA	miR-16
t0057389	22	1	TAGCAGCCCGGAAATATTGGCG	miRNA	miR-16
t0057434	22	1	TAGAAGCACGTAAATAATGGCG	miRNA	miR-16
t0057456	22	1	TAGCAGCATGTAAATAATGGCG	miRNA	miR-16
t0057488	23	1	TAGCAACACGTAAATATTGGCGT	miRNA	miR-16
t0057609	23	1	TAGCAGCACGTACCTATTGGCGC	miRNA	miR-16
t0057641	21	1	AGCAGCCCGTAAATATTGGCG	miRNA	miR-16
t0057644	44	1	TAGCAGCACGTAAATATTGGCGTGGTTTG	miRNA	miR-16
t0057889	20	1	TGCAGCACGTAAATGTTGGC	miRNA	miR-16
t0057926	25	1	TAGCAGCACGTAAATATGGGCGAAA	miRNA	miR-16
t0057969	19	1	TAGCAGCACGTAACTATTG	miRNA	miR-16
t0058071	22	1	TAGCAGCACGTAAATAATGCCG	miRNA	miR-16
t0058429	24	1	TAGCAACACGTAAATATTGGCGAA	miRNA	miR-16
t0058633	22	1	TAGCAGCATGTGAATATTGGCG	miRNA	miR-16
t0058799	22	1	TAGCATCACGTAACTATTGGCG	miRNA	miR-16
t0058900	21	1	TGCAGCACATAAATATTGGCG	miRNA	miR-16
t0059049	24	1	TAGCAGCACGTAAATAATGGCGAA	miRNA	miR-16
t0059146	22	1	TAGCAGCACGTAAATACCGGCG	miRNA	miR-16
t0059391	22	1	TAGTAGCACGTAAATATTGCCG	miRNA	miR-16
t0059713	19	1	TAGCAGCACGTGAATATGG	miRNA	miR-16
t0059976	22	1	TAGCAGCAAGTAAATATTGGGG	miRNA	miR-16
t0060106	22	1	TAGCAGCATGTAAACATTGGCG	miRNA	miR-16
t0060262	25	1	GAATAGCAGCACGTAAATATTGGCG	miRNA	miR-16
t0060341	24	1	TTTTTAGCACCACGTAAATATTGG	miRNA	miR-16
t0060729	22	1	TAGCAGAACGCAAATATTGGCG	miRNA	miR-16
t0060822	22	1	TAGAAGCACGTAAATATAGGCG	miRNA	miR-16
t0060921	23	1	ATAGCAGCACGTAGATATTGGTG	miRNA	miR-16
t0061118	19	1	TAGCAGCACGTAAATATCG	miRNA	miR-16
t0061471	24	1	TAGCAGCACGTAAATATCGGCGAA	miRNA	miR-16
t0061512	24	1	AATAGCAGCACGTAAATCTTGGCG	miRNA	miR-16
t0061638	22	1	TAGAAGCACGTAAATATTGGGG	miRNA	miR-16
t0061660	23	1	TAGCAGCACGTACATATTGGCGT	miRNA	miR-16
t0061765	23	1	ATGGCAGCACGTAAATATTGGCG	miRNA	miR-16
t0061850	19	1	TAGCAGCACGTAAATATTA	miRNA	miR-16
t0061976	24	1	ATAGCAGCACGTAAATATTGGAGA	miRNA	miR-16
t0062170	21	1	TAGCAGCATGTAAATATTTGC	miRNA	miR-16
t0062220	22	1	TAGAAGTACGTAAATATTGGCG	miRNA	miR-16
t0062708	20	1	GAGCAGCACGTAAATATTGG	miRNA	miR-16
t0062749	21	1	TAGCAGCACGTAAGTATTGGC	miRNA	miR-16
t0062850	24	1	TAGAAGCAAGTAAATATTGGCGAA	miRNA	miR-16
t0063151	23	1	TAGCAGCACGTAAATACTGGCGT	miRNA	miR-16
t0063623	21	1	AGCAGCACGCAAATATTGGCG	miRNA	miR-16
t0063680	22	1	TAGCAGCACGTGAATATTGACG	miRNA	miR-16
t0063848	24	1	TAGCAGCACGTAAATATTAGCGAA	miRNA	miR-16
t0063861	23	1	ATAGCAGCACGAAAATATTGGCG	miRNA	miR-16
t0064252	20	1	TAGCAGCAAGTAAATATGGG	miRNA	miR-16
t0064265	20	1	TAGCAGCACGTAAAAATTAG	miRNA	miR-16
t0064315	22	1	TAGCAGCTCGAAAATATTGGCG	miRNA	miR-16
t0064336	20	1	TAGCAGCACGTAAATATCCG	miRNA	miR-16
t0064470	24	1	TAGCAGCACGTAAATATTGGGGTA	miRNA	miR-16
t0064526	22	1	TAGCCGCAAGTAAATATTGGCG	miRNA	miR-16
t0064707	22	1	TATCAGCACGTAGATATTGGCG	miRNA	miR-16
t0064773	25	1	TAGCAGCATGTAAATATTGGCGAAA	miRNA	miR-16

t0064966	24	1	TAGAAGCACGTAAATATTGGCGAG	miRNA	miR-16
t0065166	22	1	TAGGAGAACGTAAATATTGGCG	miRNA	miR-16
t0065450	19	1	TAGCAGCACCTAAATATTG	miRNA	miR-16
t0065647	22	1	TAGCAGCAAGTAAACATTGGCG	miRNA	miR-16
t0065923	22	1	TAGCAGCACGTAAATATCCGCG	miRNA	miR-16
t0066174	23	1	CTAGCAGCACGTAAATATTGGGG	miRNA	miR-16
t0066363	23	1	TAGTAGCACGAAAATATTGGCGT	miRNA	miR-16
t0066542	19	1	TAGCAGCACGTAAGTATTG	miRNA	miR-16
t0066553	23	1	TAGAAGAACGTAAATATTGGCGA	miRNA	miR-16
t0066701	23	1	TAGCAGTACGTAAATATTGGCGT	miRNA	miR-16
t0067008	22	1	TAGAAGCAGGTAAATATTGGCG	miRNA	miR-16
t0067228	21	1	TAGCAGCACGTAAATATGGGC	miRNA	miR-16
t0067301	23	1	TAGCCGCACGTAAATATTGGCGT	miRNA	miR-16
t0067309	21	1	TAGCAGAACGTAAATATTGGA	miRNA	miR-16
t0067846	23	1	TAGCAGCACGTAAATACTGGCGA	miRNA	miR-16
t0067868	24	1	TAGCAGCACGTAAAAATTGGCGAA	miRNA	miR-16
t0067869	22	1	TAGCAGCACGTAAAAATTGGGG	miRNA	miR-16
t0067895	20	1	TAGCAGCACGGAAAAATTGG	miRNA	miR-16
t0068336	21	1	TAGCAGCACGTTAATATTGGC	miRNA	miR-16
t0068420	22	1	TAGAAGCACGCAAATATTGGCG	miRNA	miR-16
t0068485	22	1	CAGCAGTACGTAAATATTGGCG	miRNA	miR-16
t0068603	23	1	TAGCAGCACGTAAATATGGGCGT	miRNA	miR-16
t0068733	22	1	TAGCAGCTCGGAAATATTGGCG	miRNA	miR-16
t0068778	22	1	TAGCAGTAAGTAAATATTGGCG	miRNA	miR-16
t0068943	20	1	TAGCGGCACGTAAATATTCG	miRNA	miR-16
t0068995	21	1	AGCAGCACGTAAATATTGACG	miRNA	miR-16
t0069076	22	1	TAGCAGCAAGTAAATATTGGTG	miRNA	miR-16
t0069556	26	1	TAGCAGCCCGTAAATATTGGCGAAAA	miRNA	miR-16
t0069570	21	1	TGGCAGCACGTAAATATTGGC	miRNA	miR-16
t0069846	22	1	TGGCAGCCCGTAAATATTGGCG	miRNA	miR-16
t0070069	25	1	TAGCAGCACGTAAATATTGACGACA	miRNA	miR-16
t0070147	21	1	CGCAGCACGTAAATATTGGCG	miRNA	miR-16
t0070204	22	1	TAGCAGCACGTGAATATTGTCG	miRNA	miR-16
t0070273	25	1	TAGCAGCATGTAAATATTGGCGGAA	miRNA	miR-16
t0070361	22	1	TGGCGGCACGTAAATATTGGCG	miRNA	miR-16
t0070849	22	1	TAGCAGCACGTAGATATTGGAG	miRNA	miR-16
t0070903	24	1	TAGCAGCACGTAAATATCGGCGTT	miRNA	miR-16
t0071058	25	1	TAGCAGCACGCAAATATTGGCGAAA	miRNA	miR-16
t0071074	21	1	TAGCAGCACGGAAATATTGGC	miRNA	miR-16
t0071331	22	1	TAGCCGCGCGTAAATATTGGCG	miRNA	miR-16
t0071452	23	1	GAGCAGCACGTAAATATTGGCGA	miRNA	miR-16
t0071617	22	1	TAACAGCACGTAAATATTGCCG	miRNA	miR-16
t0071632	23	1	TAGCAGCACGTAAATATTGGGGA	miRNA	miR-16
t0072361	23	1	TAGCAGCACGTAAGTATTGGCGA	miRNA	miR-16
t0072381	22	1	TAGCAGCAAGTAAAAATTGGCG	miRNA	miR-16
t0072506	25	1	TAGAAGCACGTAAATATTGGCGAAA	miRNA	miR-16
t0072527	22	1	AAGCACCACGTAAATATTGGCG	miRNA	miR-16
t0072784	22	1	TAGCAGCACGTAGATATTGGTG	miRNA	miR-16
t0073097	22	1	TAGCGGCACGTAAATATTGACG	miRNA	miR-16
t0073223	22	1	TAGCAGCACGTTAATATTGGTG	miRNA	miR-16
t0073499	23	1	TAGCAGGACGTAAATATTGGCGT	miRNA	miR-16
t0073633	22	1	TAGCAGCCCGTAAATCTTGGCG	miRNA	miR-16
t0073745	20	1	TGGCAGCACGGAAATATTGG	miRNA	miR-16
t0074014	22	1	TAGCATAACGTAAATATTGGCG	miRNA	miR-16
t0074186	22	1	TAGGAGCACGGAAATATTGGCG	miRNA	miR-16
t0074543	22	1	TAGCAGCCCGTAAGTATTGGCG	miRNA	miR-16
t0074850	24	1	ATAGAAGCACGTAAATATTGGCGT	miRNA	miR-16
t0074957	21	1	CTAGCAGCACGTCAATATTGG	miRNA	miR-16

t0075019	25	1	GAGTAGCAGCACGTAAACATTGGCG	miRNA	miR-16
t0075072	21	1	AGCAGCACGAAAATATTGGCG	miRNA	miR-16
t0075177	24	1	TAGCAGCACGTAAATATTGGCGCA	miRNA	miR-16
t0075195	22	1	TAGCAGTGCGTAAATATTGGCG	miRNA	miR-16
t0075332	25	1	AAATAGAAGCACGTAAATATTGGCG	miRNA	miR-16
t0075550	23	1	TAGCAGCACGAAAATATTGGCGT	miRNA	miR-16
t0075724	22	1	TAGCAGCACGTAGATGTTGGCG	miRNA	miR-16
t0075984	26	1	TAGCGGCACGTAAATATTGGCGTTAT	miRNA	miR-16
t0076000	21	1	AGCAGCACGTCAATATTGGCG	miRNA	miR-16
t0076150	23	1	ATAGCAGCACCTAAATATTGGCG	miRNA	miR-16
t0076212	22	1	TAGCAACACGTAAATATTGCCG	miRNA	miR-16
t0076315	25	1	ATAACAGCACGTAAATATTGGCGAA	miRNA	miR-16
t0077047	22	1	TAGCAGGACGGAAATATTGGCG	miRNA	miR-16
t0077284	25	1	GAGGAGCAGCACGTAAATATTGGCG	miRNA	miR-16
t0077314	20	1	TAGCAGCCCTTAAATATTGG	miRNA	miR-16
t0077419	20	1	ATAGCAGCACGTAAATATTG	miRNA	miR-16
t0077622	23	1	TAGCGGCACGTAAATATTGGCGT	miRNA	miR-16
t0077897	22	1	TAGCAGCAAGTAAATATGGGCG	miRNA	miR-16
t0077979	21	1	TAGCGGCACGTAAATATTGCC	miRNA	miR-16
t0078043	22	1	TAGCAGCACGTACAAATTGGCG	miRNA	miR-16
t0078128	22	1	TAGCAGCACGTAAATATTGTTG	miRNA	miR-16
t0078195	22	1	TAGCAGCACGTAAACATTGCCG	miRNA	miR-16
t0078315	21	1	TAGCAGAACGTAAATATTGGT	miRNA	miR-16
t0078486	21	1	TAGAAGCACGTAAATATTGGT	miRNA	miR-16
t0078506	22	1	TAGCAGCAAGTAAATCTTGGCG	miRNA	miR-16
t0078556	23	1	TAGCAGCACGTAAATCTTGGCGT	miRNA	miR-16
t0078619	23	1	ATAGCAGCACGTAAATACTGGCG	miRNA	miR-16
t0079175	23	1	TAGCAGCACGTAGATATTGGCGT	miRNA	miR-16
t0079690	23	1	TAGCAGCACGTAAATGTTGGCGA	miRNA	miR-16
t0079844	22	1	TAGCAGCACGTAAGTATTGGAG	miRNA	miR-16
t0080146	19	1	TAGTAGCACGTAAATATTG	miRNA	miR-16
t0080155	23	1	TAGCAGCACGTAAATATTGGGGC	miRNA	miR-16
t0080252	20	1	TAGCAGCACGTAAATAGTCG	miRNA	miR-16
t0080257	22	1	TAGAAGCACGTCAATATTGGCG	miRNA	miR-16
t0080515	24	1	TAGCAGCATGTAAATATTGGCGAT	miRNA	miR-16
t0080602	20	1	TAGCAGCGCGTAAATATTCG	miRNA	miR-16
t0080729	22	1	TAGCAGTACGTAAATAATGGCG	miRNA	miR-16
t0080826	22	1	TAGCAACAAGTAAATATTGGCG	miRNA	miR-16
t0080879	22	1	TGGCAGCACGTAAATATTGGTG	miRNA	miR-16
t0080895	22	1	TAGCAGCACGTAGATATTGCCG	miRNA	miR-16
t0081229	22	1	TAGCAGTTCGTAAATATTGGCG	miRNA	miR-16
t0081388	22	1	TAGCAGTACGTAAATATTGGTG	miRNA	miR-16
t0081409	22	1	TAGCAGCCCTTAAATATTGGCG	miRNA	miR-16
t0081490	21	1	TAGCAGCACGTGAATATTGGC	miRNA	miR-16
t0081789	22	1	TAGCAGCACGTAAATATTCTCG	miRNA	miR-16
t0081948	22	1	TAGCAGCCCGTAACTATTGGCG	miRNA	miR-16
t0082037	21	1	GGCAGCACGTAAATATTGGCG	miRNA	miR-16
t0082172	22	1	TAGCAGCACGCACATATTGGCG	miRNA	miR-16
t0082242	23	1	TAGCAGCCCGTAAATATTGGCGT	miRNA	miR-16
t0082243	21	1	AGCAGCACGTAATTATTGGCG	miRNA	miR-16
t0082515	23	1	TAGCAGCACGTAATTATTGGCGA	miRNA	miR-16
t0082656	22	1	TAGCAGAACGTAAATATTCGCG	miRNA	miR-16
t0082745	22	1	TATCAGCACGTAAATAGTGGCG	miRNA	miR-16
t0083382	25	1	TGGCAGCACGTAAATATTGGCGAAA	miRNA	miR-16
t0083384	24	1	TAGGAGCACGGAAATATTGGCGTA	miRNA	miR-16
t0083491	22	1	TAGCAGCACGTAAATTTTGGTG	miRNA	miR-16
t0083983	23	1	TAGCAGCACGTAGATATTGGCGA	miRNA	miR-16
t0084292	23	1	TAGCAGCACGTTAATATTGGCGT	miRNA	miR-16

t0084418	22	1	TAGCGGCACGTAAGTATTGGCG	miRNA	miR-16
t0084594	22	1	TAGCAGCACGAAAATATCGGCG	miRNA	miR-16
t0084698	22	1	TAGCAGCACGCAAATATTGGTG	miRNA	miR-16
t0084732	22	1	TAGCAGCTTGTAAATATTGGCG	miRNA	miR-16
t0085060	22	1	TAGCAGTACGTAAATATTGACG	miRNA	miR-16
t0085127	23	1	TAGCAGCACGTATATATTGGCGT	miRNA	miR-16
t0085225	22	1	TAGCAGCACGTAAATCTTGGTG	miRNA	miR-16
t0085255	22	1	TAGCAGCTCGTAAATATTTGCG	miRNA	miR-16
t0085471	22	1	TAGCAGCAAGTAAATGTTGGCG	miRNA	miR-16
t0085487	22	1	TAGCAGCCCGTAAATATTGGGG	miRNA	miR-16
t0085645	23	1	TAACAGCACGTAAATATTGGCGT	miRNA	miR-16
t0085770	21	1	CAGCAGCACGTAAATATTGGC	miRNA	miR-16
t0086028	23	1	CTAGCAGCCCGTAAATATTGGCG	miRNA	miR-16
t0086291	21	1	TAGCAGCACGTAAATATTAGA	miRNA	miR-16
t0086614	19	1	TAGCAGCACGTAAACATTG	miRNA	miR-16
t0086718	22	1	TAGCAGCACTTAAATATTCGCG	miRNA	miR-16
t0086887	21	1	AGCCGCACGTAAATATTGGCG	miRNA	miR-16
t0087047	22	1	TAGCAGCCCGTAAATATTGGAG	miRNA	miR-16
t0087127	22	1	TATCAGTACGTAAATATTGGCG	miRNA	miR-16
t0087237	21	1	CAGCAGCACGTAAATATTGGT	miRNA	miR-16
t0087573	20	1	TAGGAGCACGTAAATATTGA	miRNA	miR-16
t0087890	22	1	CAGCAGCACGTAAGTATTGGCG	miRNA	miR-16
t0087955	23	1	TAGCAGCACGTAAATATTCGCGT	miRNA	miR-16
t0088023	22	1	TAGCAGTACGCAAATATTGGCG	miRNA	miR-16
t0088187	23	1	TAGCAGCACGTAAATATTGACGT	miRNA	miR-16
t0088993	26	1	CAAGTAGCAGCACGTAAATATTGGAG	miRNA	miR-16
t0089302	23	1	TAGCAGCACGTAATTATTGGCGT	miRNA	miR-16
t0089435	22	1	TAGCCGCACGTACATATTGGCG	miRNA	miR-16
t0089629	22	1	TAGCAGCCCGTAAATATTTGCG	miRNA	miR-16
t0089669	22	1	TAGCAGCACGTCAATATTGGTG	miRNA	miR-16
t0089809	23	1	TAGCAGCACGTCAATATTGGCGA	miRNA	miR-16
t0089853	22	1	TAGAAGCACGTGAATATTGGCG	miRNA	miR-16
t0089864	22	1	TAGCAGCGCGTAAATATTGGAG	miRNA	miR-16
t0090045	22	1	GTAGCAGCACGTAAATATTGGC	miRNA	miR-16
t0090288	22	1	TAGCAGAACGTAAATCTTGGCG	miRNA	miR-16
t0090499	22	1	TAGCAGCCTGTAAATATTGGCG	miRNA	miR-16
t0090531	20	1	TACCAGCACGTATATATTGG	miRNA	miR-16
t0090757	24	1	TAGCAGTACGTAAATATTGGCGTT	miRNA	miR-16
t0090801	22	1	TAGCAGAACGTAAAAATTGGCG	miRNA	miR-16
t0090851	23	1	ATAGCAGCGCGTAAATATTGGCG	miRNA	miR-16
t0090858	22	1	TAGCAGCAAGTGAATATTGGCG	miRNA	miR-16
t0090962	22	1	AGCAGCACGTAAATATTGGCGC	miRNA	miR-16
t0091007	24	1	GTAGCAGCCCGTAAATATTGGCGT	miRNA	miR-16
t0091192	23	1	ATAGCAGAACGTAAATATTGGCG	miRNA	miR-16
t0091204	22	1	TAGCATCACGTAAATATTGTCG	miRNA	miR-16
t0091429	22	1	TGGCAGCACGTAAATATTCGCG	miRNA	miR-16
t0091510	19	1	TAGCAGCACGTCAATATTG	miRNA	miR-16
t0091829	19	1	TACCAGCACGTAAATATTG	miRNA	miR-16
t0091997	22	1	TAGCAGCACGTAAGGATTGGCG	miRNA	miR-16
t0092113	23	1	ATAGCAGCACGTCAATATTGGCG	miRNA	miR-16
t0092145	21	1	TAGCCGCCCATCATGGTTTTA	miRNA	miR-15c
t0092279	22	1	TAGCAGCAAATCATGGTTTTCA	miRNA	miR-15c
t0092370	22	1	TAGCGGCACATCATGGTTTTCA	miRNA	miR-15c
t0092413	22	1	TAGCAGCACATCATGGGTTTCA	miRNA	miR-15c
t0092474	20	1	TAGAAGCACATCATGGTTTT	miRNA	miR-15c
t0092478	20	1	TAGCAGAAGATCATGGTTTT	miRNA	miR-15c
t0092657	22	1	TAGCAGCACATCATGGTTATCC	miRNA	miR-15c
t0092766	22	1	TAGCAGAACATCATGGTTTTCA	miRNA	miR-15c

t0092779	20	1	TAGCGGCACATCATGGTTTT	miRNA	miR-15c
t0093002	20	1	TAGACGCACATCATGGTTTT	miRNA	miR-15c
t0093303	22	1	TAGAAGCACATCATGGTTTTCA	miRNA	miR-15c
t0093340	20	1	TAGCCGCAAATCATGGTTTT	miRNA	miR-15c
t0093342	22	1	TAGCAGCACATTATGGTTTTAA	miRNA	miR-15c
t0044815	20	1	TAGCAGCCCATCATGGTTTT	miRNA	miR-15c
t0044843	21	1	TAGCAGCAAATCATGGTTTTA	miRNA	miR-15c
t0048084	20	1	TAGCAGCGCATCATGGTTTT	miRNA	miR-15c
t0049433	20	1	TAGTAGCACATCATGGTTTT	miRNA	miR-15c
t0049715	22	1	TAGCAGAACATCATGGTTATCA	miRNA	miR-15c
t0049801	22	1	CGAATCATTATTTGCTGCTCTC	miRNA	miR-15b*
t0055033	19	1	CGAATCATTATTTGCTGAT	miRNA	miR-15b*
t0059405	18	1	AGCGGCACATCATGGTTT	miRNA	miR-15b
t0062410	19	1	CAGCCGCACATCATGGTTT	miRNA	miR-15b
t0068209	22	1	AATAGAAGCACATCATGGTTTA	miRNA	miR-15b
t0068759	19	1	TACCAGCACATTATGGTTT	miRNA	miR-15b
t0071289	21	1	ACTAGCAGCGCATCATGGTTT	miRNA	miR-15b
t0074756	18	1	TAGCAGCACATCATGCTC	miRNA	miR-15b
t0077447	20	1	TAGTAGCATATCATGGTTTA	miRNA	miR-15b
t0077963	19	1	TAGCAGACCATCATGGTTT	miRNA	miR-15b
t0080958	22	1	TAGCAGTACATCATGGTTTACC	miRNA	miR-15b
t0082457	21	1	TAGCAGCGCATCATGGTTTAA	miRNA	miR-15b
t0088149	19	1	TAGTAGCACATCATGCTTT	miRNA	miR-15b
t0074593	19	1	TAGCAGCACATCAGGGTTT	miRNA	miR-15b
t0085436	22	1	TAGCGGCACATCTTGGTTTACA	miRNA	miR-15b
t0040969	19	1	TAGCAGCACGTTATGGTTT	miRNA	miR-15b
t0041376	19	1	TAGCAGCCGATCATGGTTT	miRNA	miR-15b
t0041514	20	1	TAGCAGCACCTCATGGGTTA	miRNA	miR-15b
t0041889	22	1	TAGCAGCACATCATGGGTTACC	miRNA	miR-15b
t0042002	21	1	AGCAGCACATCATGGTTTACA	miRNA	miR-15b
t0042379	19	1	TAGCGGCACATCATGATTT	miRNA	miR-15b
t0043833	24	1	TAGCAGCACATCATGGGTTAAGTT	miRNA	miR-15b
t0043924	21	1	TAGCAGCACATAATGGTTTAC	miRNA	miR-15b
t0044015	22	1	TAGAAGCACAACATGGTTTACA	miRNA	miR-15b
t0045173	18	1	TAGCAGCAAATCATGTTT	miRNA	miR-15b
t0045209	20	1	TAGCAGCACATTATGGATTA	miRNA	miR-15b
t0045692	20	1	TAGCAGCACATCATGGGGTA	miRNA	miR-15b
t0045780	25	1	TAGCAGCACATAATGGTTTACAATA	miRNA	miR-15b
t0045822	20	1	GCAGCACATCATGGTTTACG	miRNA	miR-15b
t0046011	19	1	TAGTAGCACATCATGGGTT	miRNA	miR-15b
t0046943	18	1	TAGCAGCACATCATGGCT	miRNA	miR-15b
t0047013	20	1	TAGCAGCACATAATGATTTA	miRNA	miR-15b
t0047114	18	1	TACCAGCACATCATGGTT	miRNA	miR-15b
t0047160	22	1	TAGCCGCACATCATGGTTTACC	miRNA	miR-15b
t0047554	19	1	TAGAAGCACATCATGGGTT	miRNA	miR-15b
t0047736	30	1	TAGCAGCACATCATGGTTTACCAATCATG	miRNA	miR-15b
t0048802	19	1	TAGCAGCCCATCATGGTTA	miRNA	miR-15b
t0049529	24	1	AAAATAGAAGCACATCATGGTTTA	miRNA	miR-15b
t0049964	20	1	TAGAAGCACATGATGGTTTA	miRNA	miR-15b
t0050144	22	1	TAGCAGCACATTATGTTTTACA	miRNA	miR-15b
t0050176	19	1	AAGCAGCACATCATGGTTC	miRNA	miR-15b
t0050850	19	1	TAGCAGCACAGCATGGGTT	miRNA	miR-15b
t0050885	18	1	TAGCAGCACATCATGGTC	miRNA	miR-15b
t0050953	24	1	TAGCAGCATATCATGGTTTAAGTT	miRNA	miR-15b
t0051044	19	1	TATCAGCACATCATGGATT	miRNA	miR-15b
t0051330	23	1	TAGCAGCACATCATGGATTACAA	miRNA	miR-15b
t0051345	21	1	TAGCAGCACATCATGGTTACG	miRNA	miR-15b
t0051525	19	1	TAGAAGCCCATCATGGTTT	miRNA	miR-15b

t0051637	20	1	TAGCAGCAGATCATGGGTTA	miRNA	miR-15b
t0051677	21	1	TACCAGCACATCATGGTTTAC	miRNA	miR-15b
t0051899	21	1	TAGCAGCACATCATGTTTTAC	miRNA	miR-15b
t0051959	22	1	TAGCAGCACATCATGCTTTACC	miRNA	miR-15b
t0052049	19	1	TAGCAGGATATCATGGTTT	miRNA	miR-15b
t0052191	20	1	AAGCAGCACATCATGGGTTA	miRNA	miR-15b
t0052605	20	1	TACCAGCACATCGTGGTTTA	miRNA	miR-15b
t0052686	22	1	TAGCAACACATCATGTTTTACA	miRNA	miR-15b
t0053972	22	1	TAGCAGCACATCATGGTTTGTG	miRNA	miR-15b
t0054119	21	1	TAGCAGAACATCATGGTTTAA	miRNA	miR-15b
t0054487	24	1	TAGCAGAACATCATGGTTTACAAA	miRNA	miR-15b
t0054499	18	1	TAGCAGCTCATCATGGTT	miRNA	miR-15b
t0054527	30	1	TAGCAGCACATCATGGTTTACAAATCATG	miRNA	miR-15b
t0054947	21	1	AATAGCAGCACATCATGGGTT	miRNA	miR-15b
t0054948	22	1	TAGCAGCACATCATGGTTTAAC	miRNA	miR-15b
t0055113	22	1	TGGCAGAACATCATGGTTTACA	miRNA	miR-15b
t0055457	20	1	TAGCAGTACATCATGGGTTA	miRNA	miR-15b
t0055659	22	1	TAGCAGAAAATCATGGTTTACA	miRNA	miR-15b
t0055706	18	1	TTGCAGCACATCATGGTT	miRNA	miR-15b
t0055850	21	1	TAGCAGCACATCATGGCTTAC	miRNA	miR-15b
t0057223	19	1	TAGTAGCACATCATGGTTA	miRNA	miR-15b
t0058279	22	1	TAGCAGCACATCATGGTTAACA	miRNA	miR-15b
t0058289	26	1	TTGGTAGCAGCACATCATGGTTTACA	miRNA	miR-15b
t0059026	19	1	TAGCAGCAGATCATGGATT	miRNA	miR-15b
t0059386	28	1	TAGCAGCATATCATGGTTTACATGCCAC	miRNA	miR-15b
t0059419	22	1	TAGCAGAACATCATGGTTTACC	miRNA	miR-15b
t0059501	20	1	CAGCAGCACATTATGGTTTA	miRNA	miR-15b
t0060487	19	1	TAGCAGCACATCATGGTGT	miRNA	miR-15b
t0060625	22	1	TAGCAGCATATCATGGTTTACC	miRNA	miR-15b
t0060628	20	1	TAGAAGCACATCATGGATTA	miRNA	miR-15b
t0061149	22	1	TAGTAGCACATCATGGTTTATA	miRNA	miR-15b
t0061261	20	1	TAGCAGCACATCATGCTTTA	miRNA	miR-15b
t0062075	20	1	GAGAAGCACATCATGGTTTA	miRNA	miR-15b
t0062180	21	1	TAGCAGCACATCATGGCTTAA	miRNA	miR-15b
t0062352	22	1	TAGGAGCACATCATGGTTTATA	miRNA	miR-15b
t0062528	19	1	TAGCAACACATTATGGTTT	miRNA	miR-15b
t0063719	21	1	TCGCAGCACATCATGGTTTAA	miRNA	miR-15b
t0064071	19	1	TAGAAGCAGATCATGGTTT	miRNA	miR-15b
t0064499	21	1	TAGCAGCACATCATGGGTTAA	miRNA	miR-15b
t0064788	20	1	TAGCAGAATATCATGGTTTA	miRNA	miR-15b
t0065265	21	1	TAGCAGCACATCATGGTTGAA	miRNA	miR-15b
t0065786	20	1	TAGCAGCACATAATGGATTA	miRNA	miR-15b
t0066334	21	1	TAGCAACACATCATGGTTTAC	miRNA	miR-15b
t0066640	18	1	AGCAGGACATCATGGTTT	miRNA	miR-15b
t0066937	20	1	TAGCAGCACTTCATGGGTTA	miRNA	miR-15b
t0067261	22	1	TAGCAGAACATCATGGTTTAAA	miRNA	miR-15b
t0067675	19	1	TAGCAGCGCATCATGGGTT	miRNA	miR-15b
t0067996	20	1	TAGCGGCATATCATGGTTTA	miRNA	miR-15b
t0068088	19	1	TAGCATCACATCATGGTTA	miRNA	miR-15b
t0069286	18	1	AGCAGTACATCATGGTTT	miRNA	miR-15b
t0069318	22	1	TAGCAGCACATCATGGGTCACA	miRNA	miR-15b
t0069732	21	1	TAGCAGTACATCATGGTTTAC	miRNA	miR-15b
t0069883	30	1	TAGCAGCAAATCATGGTTTACAAATTATG[illegible]	miRNA	miR-15b
t0070570	19	1	TAGCGGCACATTATGGTTT	miRNA	miR-15b
t0070943	19	1	TAGGAGCACATCATGGTTA	miRNA	miR-15b
t0070997	22	1	TAGCAGCGCATTATGGTTTACA	miRNA	miR-15b
t0071398	20	1	TAGCAGCATATAATGGTTTA	miRNA	miR-15b
t0071760	21	1	TAGCGGCACATCATGGTTTAC	miRNA	miR-15b

t0072150	19	1	TAGCACCACATCATGGTTA	miRNA	miR-15b
t0072986	20	1	TAGCGGCACATCATGGGTTA	miRNA	miR-15b
t0073245	22	1	TAGCAGTACGTCATGGTTTACA	miRNA	miR-15b
t0074026	20	1	TAGCAGACCATCATGGTTTA	miRNA	miR-15b
t0074050	22	1	TAGCAGCACATCATTGTTTACC	miRNA	miR-15b
t0074426	19	1	TAGCAGCACATCGTGGTTA	miRNA	miR-15b
t0074619	20	1	TAGAAACACATCATGGTTTA	miRNA	miR-15b
t0074676	18	1	AGCATCACATCATGGTTT	miRNA	miR-15b
t0075176	18	1	TAGCAGCACTTCATGGTT	miRNA	miR-15b
t0075564	22	1	TAGCAGCACATAATGGTTTATT	miRNA	miR-15b
t0075877	22	1	TAGCAGTACATCCTGGTTTACA	miRNA	miR-15b
t0075925	20	1	TAGCAGAAAATCATGGTTTA	miRNA	miR-15b
t0076470	23	1	TAGCAGCACATCATGGTTTAAAT	miRNA	miR-15b
t0076942	21	1	TAGCAGCGCATCATGGTTTAC	miRNA	miR-15b
t0077233	20	1	TAGCGGCACTTCATGGTTTA	miRNA	miR-15b
t0077243	22	1	TAGCAGCACATCAGGGTTTACA	miRNA	miR-15b
t0077291	19	1	TAGAATCACATCATGGTTT	miRNA	miR-15b
t0077687	22	1	TACCAGCACATCATGGTTTACA	miRNA	miR-15b
t0077841	19	1	AAGCAGCACATTATGGTTT	miRNA	miR-15b
t0078145	20	1	TAGCAGCATATCATGGGTTA	miRNA	miR-15b
t0078290	18	1	TAGTAGCACATCATGGTT	miRNA	miR-15b
t0078296	18	1	TAGAAGCACATTATGGTT	miRNA	miR-15b
t0078945	19	1	TAGCAGCACATGATGCTTT	miRNA	miR-15b
t0079137	20	1	TAGCAGCACATCAAGGGTTA	miRNA	miR-15b
t0079837	20	1	TAGCAGCATATCATGGATTA	miRNA	miR-15b
t0080031	19	1	TGGCAGCACATCATGGTTA	miRNA	miR-15b
t0080355	20	1	TAGCAGCACATAATGGGTTA	miRNA	miR-15b
t0080664	20	1	TAGCAGCACATCAGGGTTTA	miRNA	miR-15b
t0081515	20	1	TTGCAGCACATCATGGTTTA	miRNA	miR-15b
t0081618	19	1	TAGCAAAACATCATGGTTT	miRNA	miR-15b
t0081642	19	1	TAGCGGCCCATCATGGTTT	miRNA	miR-15b
t0081740	22	1	TAGCAGCCCATCATGTTTTACA	miRNA	miR-15b
t0082491	19	1	TAGAAGCATATCATGGTTT	miRNA	miR-15b
t0082795	22	1	TAGCAGCACATTATGGTTTACC	miRNA	miR-15b
t0082963	19	1	TAGCAGCGCATTATGGTTT	miRNA	miR-15b
t0083161	20	1	TAGCAGCACATCAATGTTTA	miRNA	miR-15b
t0083505	22	1	TAGTAGCATCACATCATGGTTT	miRNA	miR-15b
t0085493	19	1	TAGCCGGACATCATGGTTT	miRNA	miR-15b
t0085553	22	1	TAGAAGCACATCATGGTTTAAA	miRNA	miR-15b
t0085647	22	1	TAGCAGCAAATAATGGTTTACA	miRNA	miR-15b
t0086226	19	1	TAGCAGCACGTCATGGTTA	miRNA	miR-15b
t0086241	22	1	TAGCAGCATATCATGGGTTACA	miRNA	miR-15b
t0086657	22	1	TAGCTGCACATCATGGTTTACA	miRNA	miR-15b
t0086717	22	1	TAGAAGCACATCATGGGTTACA	miRNA	miR-15b
t0087033	18	1	TAGCAACACATCATGGTT	miRNA	miR-15b
t0087103	21	1	TAGTAGCACATCATGGTTTAC	miRNA	miR-15b
t0087456	19	1	TAGCAGTAAATCATGGTTT	miRNA	miR-15b
t0087490	20	1	TAGCCGCATATCATGGTTTA	miRNA	miR-15b
t0087685	19	1	TAGCAGAACATCATGGATT	miRNA	miR-15b
t0087773	20	1	AAGCAGCATATCATGGTTTA	miRNA	miR-15b
t0087960	22	1	TAGCAGCACATGATGGTTTACA	miRNA	miR-15b
t0088111	21	1	TAGCAGCACATCCTGGTTTGT	miRNA	miR-15b
t0088225	20	1	TAGCAGCACATCGTGGGTTA	miRNA	miR-15b
t0088568	22	1	TAGCAGAACATCATGTTTTACA	miRNA	miR-15b
t0088986	21	1	AATAGCAGCACCTCATGGTTT	miRNA	miR-15b
t0090082	21	1	TAGCAGCACATAATGGCTTTT	miRNA	miR-15a
t0090131	19	1	TTGCAGCACATAATGGGTT	miRNA	miR-15a
t0090291	21	1	GAGCAGCACATAATGGTTTGT	miRNA	miR-15a

t0090309	21	1	TAGCAGCACCTAATGGCTTGT	miRNA	miR-15a
t0091453	18	1	TAGCAGCACATACTGGTT	miRNA	miR-15a
t0092186	23	1	TAGCAGCACACAATGGTTTGTGG	miRNA	miR-15a
t0092228	20	1	TAGCAGCACATAATGGGTTT	miRNA	miR-15a
t0092476	20	1	TAGCAGCACACAATGATTTG	miRNA	miR-15a
t0092592	19	1	TAGCAGCACATAACGGTTT	miRNA	miR-15a
t0092709	20	1	TAGCAGCACATAATGGTGTG	miRNA	miR-15a
t0092788	20	1	TAGTAGAACATAATGGTTTG	miRNA	miR-15a
t0093451	19	1	TGGCAGCACATAATGGTTT	miRNA	miR-15a
t0093521	20	1	TAGTAGTACATAATGGTTTG	miRNA	miR-15a
t0041030	20	1	TAGAAGCACATAATGGGTTG	miRNA	miR-15a
t0041574	22	1	TAGCAGTACATAATGGTTTACA	miRNA	miR-15a
t0042421	22	1	TAGCAGCACATAATGGTTAGTG	miRNA	miR-15a
t0042767	20	1	TAACAGCACATAATGGGTTG	miRNA	miR-15a
t0042780	18	1	TAGCAGCACATATTGGTT	miRNA	miR-15a
t0043081	19	1	TCGCAGCACATAATGGTTT	miRNA	miR-15a
t0043105	23	1	ATAGCAGCACATAATGGTTTGGG	miRNA	miR-15a
t0043756	22	1	TAGCAGCACATGATGGTTTGTG	miRNA	miR-15a
t0043928	20	1	TAGAAGCACATACTGGTTTG	miRNA	miR-15a
t0044592	19	1	TAGCAGCACATACTGGGTT	miRNA	miR-15a
t0045117	24	1	TAGCAGCACATAATGGTTTTTGGA	miRNA	miR-15a
t0045246	20	1	TAGCAGCACATAACGTTTTG	miRNA	miR-15a
t0046074	19	1	TAGGAGCACATAATGGTTT	miRNA	miR-15a
t0046192	23	1	TAGCAGCACATAATGGTTTGTCA	miRNA	miR-15a
t0046438	20	1	TAGAAGCAAATAATGGTTTG	miRNA	miR-15a
t0046561	19	1	TAGCAGCACATAGTGGTTT	miRNA	miR-15a
t0047016	19	1	TAGCAGAACATAATGGTTA	miRNA	miR-15a
t0047153	19	1	TAGCAGCACATAAAGGTTT	miRNA	miR-15a
t0047708	22	1	TAGCAGAACATAATGGTTTGTT	miRNA	miR-15a
t0047767	18	1	TAGCGGCACATAATGGTT	miRNA	miR-15a
t0047949	19	1	TAGCAGCACATAATGGTAC	miRNA	miR-15a
t0048285	23	1	TAGCAGCACGTAATGGTTTGTGG	miRNA	miR-15a
t0048963	19	1	TAGCAGCACATACTGGTTT	miRNA	miR-15a
t0049622	22	1	TAGCAGCATATAATGGTTTGTG	miRNA	miR-15a
t0049705	19	1	TACCAGCACATAATGGTTT	miRNA	miR-15a
t0050862	20	1	TAGAAGCACACAATGGTTTG	miRNA	miR-15a
t0051355	22	1	TGGCAGCACATAATGGTTTGTG	miRNA	miR-15a
t0051851	42	1	TAGCAGCACATAATGGTTTGTCGTATGAG	miRNA	miR-15a
t0053447	22	1	TAGCAGCACATAATGGTTTGCA	miRNA	miR-15a
t0054127	22	1	TAGTAGCAGCACATAATGGTTT	miRNA	miR-15a
t0054136	22	1	TAGCAGCCCATAATGGTTTGTG	miRNA	miR-15a
t0056758	22	1	TAGCAGCACATAATGGCTTGTG	miRNA	miR-15a
t0056888	20	1	TAGCAGCACATAAATGTTTG	miRNA	miR-15a
t0057135	22	1	TAGCAGCATATAATGGTTTGTA	miRNA	miR-15a
t0057493	22	1	TAGCAGCACATGATGGTTTGTT	miRNA	miR-15a
t0057577	22	1	TAGCAGCTCATAATGGTTTGTG	miRNA	miR-15a
t0059021	21	1	AGCAGCACATAATGGTTTGGG	miRNA	miR-15a
t0059688	23	1	TAGCAGCACATAATGGTTTGGGA	miRNA	miR-15a
t0060017	22	1	CAGCAGCACATAATGGTTTGTA	miRNA	miR-15a
t0060282	22	1	TAGAAGCACATAATGGTTTTTG	miRNA	miR-15a
t0060391	22	1	TAGTAGCACATAATGGTTTGTG	miRNA	miR-15a
t0060840	22	1	ATAGCAGCACGTAATGGTTTGT	miRNA	miR-15a
t0062339	24	1	TAGCAGCACATAATGGGTTGTGGA	miRNA	miR-15a
t0062722	22	1	TAGCAGCACATAATGGTTGGTG	miRNA	miR-15a
t0063503	18	1	TAGCAGCACATAATGGTC	miRNA	miR-15a
t0064716	19	1	TATCAGCACATAATGGTTT	miRNA	miR-15a
t0065033	21	1	TAGCAGAACATAATGGTTTAC	miRNA	miR-15a
t0065215	21	1	TAGCAGCACATAATGGGATGT	miRNA	miR-15a

t0065271	24	1	TAGCAGGACATAATGGTTTGTGAA	miRNA	miR-15a
t0065317	19	1	TAGCAGCTCATAATGGTTG	miRNA	miR-15a
t0065322	22	1	TAGCAGCACATACTGGTTTGTG	miRNA	miR-15a
t0065740	18	1	TAGCAGCACGTAATGGTG	miRNA	miR-15a
t0065880	21	1	TAGCAGCACATAATGGGGTGT	miRNA	miR-15a
t0066713	18	1	TAGCAGCACATAATGCTG	miRNA	miR-15a
t0066744	23	1	TAGCAGCACATAATGGTTTGGGG	miRNA	miR-15a
t0066961	20	1	CAGCAGCACATAATGGGTTG	miRNA	miR-15a
t0067248	19	1	TAGCAGCACATAATAGTTT	miRNA	miR-15a
t0067329	22	1	CAGCAGCACATAATGGTTTGTG	miRNA	miR-15a
t0067651	21	1	TAGCAGCACATAATGGTATGT	miRNA	miR-15a
t0067677	19	1	TAGCGGCACATAATGGTTG	miRNA	miR-15a
t0067777	22	1	TAGCAGCACATAATGGTTGGGG	miRNA	miR-15a
t0068203	20	1	TAGCAGCACATAAGGGTTTG	miRNA	miR-15a
t0068301	20	1	TAGCGGCGCATAATGGTTTG	miRNA	miR-15a
t0068507	22	1	ATAGCAGAACATAATGGTTTGT	miRNA	miR-15a
t0068993	22	1	TAGCAGCACATAATGGGTTGTT	miRNA	miR-15a
t0069378	26	1	GAGTTAGCAGCACATAATGGTTTTTG	miRNA	miR-15a
t0070564	21	1	TTGCAGCACATAATGGTTTGT	miRNA	miR-15a
t0070676	23	1	ATAGCAGAACATAATGGTTTGTG	miRNA	miR-15a
t0071348	20	1	TAGAAGCATATAATGGTTTG	miRNA	miR-15a
t0072019	23	1	TAGCAGCACATAATGGGTTGTGG	miRNA	miR-15a
t0072513	22	1	TAGCATCACATAATGGTTTGTG	miRNA	miR-15a
t0072692	19	1	TAGTAGCACATAATGGTTG	miRNA	miR-15a
t0072795	21	1	TAGCTGCACATAATGGTTTGT	miRNA	miR-15a
t0072952	22	1	TAGCCGCACATAATGGTTTGTG	miRNA	miR-15a
t0072954	20	1	TAGCAGAACATAATGGTTTT	miRNA	miR-15a
t0073073	20	1	TAGCAGAATATAATGGTTTG	miRNA	miR-15a
t0073178	20	1	TTGCAGCACATAATGGTTTG	miRNA	miR-15a
t0073476	19	1	TAGAAGCACATAATGGTTG	miRNA	miR-15a
t0073910	22	1	TAGCACCACATAATGGTTTGTG	miRNA	miR-15a
t0073953	18	1	TAGCAGCACATAATGGGG	miRNA	miR-15a
t0075936	19	1	TAGCAGCACATAATGGTTC	miRNA	miR-15a
t0076225	18	1	TAGCAGGACATAATGGTT	miRNA	miR-15a
t0076674	20	1	TAGAAGAACATAATGGTTTG	miRNA	miR-15a
t0078100	20	1	TAGCAGAACCTAATGGTTTG	miRNA	miR-15a
t0078206	21	1	TAGCAGAACATAATGGATTGT	miRNA	miR-15a
t0078300	21	1	TAGCAGCACATAATTGTTTGT	miRNA	miR-15a
t0078410	18	1	AGCAGCACATAATGGTTT	miRNA	miR-15a
t0080094	24	1	TAGCAGCACGTAATGGTTTGTGGA	miRNA	miR-15a
t0080828	20	1	TGGCAGAACATAATGGTTTG	miRNA	miR-15a
t0080910	20	1	TAGCAGAAAATAATGGTTTG	miRNA	miR-15a
t0081200	22	1	TAGCAGCACACAATGATTTGTG	miRNA	miR-15a
t0082343	20	1	TAGCCGCACATAATGGGTTG	miRNA	miR-15a
t0082950	20	1	TAGCATCACATAATGGGTTG	miRNA	miR-15a
t0083570	20	1	TAGCATCACATAACGGTTTG	miRNA	miR-15a
t0083652	19	1	TAGCAGCCCATAATGGTTT	miRNA	miR-15a
t0083929	19	1	TAGCAGCGCATAATGGTTG	miRNA	miR-15a
t0084547	20	1	TCGCAGCACATAATGGGTTG	miRNA	miR-15a
t0084952	19	1	TAACAGCACATAATGGTTT	miRNA	miR-15a
t0086195	21	1	TAGCAGCACATATTGGTTTGT	miRNA	miR-15a
t0086696	20	1	TAGCAGCACATAATGGTTGG	miRNA	miR-15a
t0086922	20	1	TAGCAGCACCTAATGGGTTG	miRNA	miR-15a
t0087696	20	1	AAGCAGAACATAATGGTTTG	miRNA	miR-15a
t0088543	22	1	TAGCAGGACATAATGGTTTGTG	miRNA	miR-15a
t0089031	20	1	TAGCCGCATATAATGGTTTG	miRNA	miR-15a
t0089698	19	1	TAGCAGCGCATAATGGTTT	miRNA	miR-15a
t0089800	23	1	AATCTCCTTCGAATGCCACTTTT	miRNA	miR-1596

t0090016	20	1	AACACCTCCTTCGAATGCCA	miRNA	miR-1596
t0090110	20	1	ACCCGTCGGCTGTCAGCGGA	miRNA	miR-1557
t0090917	18	1	CCCGTCGGCTGTCAGCGG	miRNA	miR-1557
t0091015	25	1	ACCCGTCGGCTGTCAGCGGACTGCT	miRNA	miR-1557
t0091319	21	1	TCAGGGCATGACAGAACTTGG	miRNA	miR-152
t0091547	24	1	TCAGTGCATGACATAACTTGGATC	miRNA	miR-152
t0091616	24	1	TCAGTGCATGACAGAACTTGGATA	miRNA	miR-152
t0091739	22	1	TCAGTGCATGACAGAACTTGGG	miRNA	miR-152
t0091786	22	1	TCAGTACATGACAGAACTTGGT	miRNA	miR-152
t0092241	22	1	TCAGGGCATGACAGAACTTGGT	miRNA	miR-152
t0092534	22	1	TTAGTGCATGACAGAACTTGGT	miRNA	miR-152
t0092621	22	1	TCAGTGCATGACAGGACTTGGT	miRNA	miR-152
t0046316	22	1	TTGAGGAGCTCACAGTCTAGTA	miRNA	miR-151-5p
t0089248	21	1	TCGAGGAGCTCACAGTCAAGT	miRNA	miR-151-5p
t0051589	20	1	TCGAGGAGCTCACAGTCCAG	miRNA	miR-151-5p
t0085431	21	1	TCGAGGAGCACACAGTCTAGT	miRNA	miR-151-5p
t0089204	21	1	TCGAGGAGCTCACAGCCTAGT	miRNA	miR-151-5p
t0042637	21	1	TCGGGGAGCTCACAGTCTAGT	miRNA	miR-151-5p
t0042690	22	1	TCGAGGAGCTCACAGTCTAGTC	miRNA	miR-151-5p
t0043848	22	1	CTAGACTGAAGCTCCTTGCGGA	miRNA	miR-151-3p
t0053909	25	1	CTAGACCGAAGCTCCTTGAGGAAGA	miRNA	miR-151-3p
t0057944	24	1	CTAGACTGAAGCTCCTTGCGGAAG	miRNA	miR-151-3p
t0074494	24	1	CTTGACTGAAGCTCCTTGAGGAAG	miRNA	miR-151-3p
t0075459	25	1	CTAGACTGAAGCTCCCTGAGGAAGA	miRNA	miR-151-3p
t0080081	20	1	CTAGACTGAAGATCCTTGAG	miRNA	miR-151-3p
t0052692	25	1	CTAGACTGAAGCTCCTTGAGTAAGA	miRNA	miR-151-3p
t0058688	20	1	CTAGACTAAAGCTCCTTGAG	miRNA	miR-151-3p
t0059095	20	1	CTAGACTGAGGCTTCTTGAG	miRNA	miR-151-3p
t0071687	20	1	CCAGACTGAAGCTCCTTGAG	miRNA	miR-151-3p
t0072743	25	1	CTAGACTGAGGCTCCTTGAGAAATC	miRNA	miR-151-3p
t0074358	23	1	CTAGACTGAAGCTCCTTGAGGGA	miRNA	miR-151-3p
t0075659	22	1	GGAAGATCTTGTAGCATGGTTT	miRNA	miR-1503
t0048675	21	1	GAAGATCTTGTAGCATGGTAT	miRNA	miR-1503
t0049909	21	1	CCAGGAAGATCTTGAAGCATA	miRNA	miR-1503
t0055081	24	1	CTGGTACAGGCCTGGGGGACAATC	miRNA	miR-150*
t0057699	22	1	TCTCCCAACCCTTGGACCAGTG	miRNA	miR-150
t0057873	21	1	TCTCGCAACCCTTGTACCAGT	miRNA	miR-150
t0060900	22	1	TCTCCAAACCCTTGTACCAGTG	miRNA	miR-150
t0065925	22	1	CCTCCCAACCCTTGTACCAGTG	miRNA	miR-150
t0075747	22	1	TCTCCCAACTCTTGTACCAGTA	miRNA	miR-150
t0079617	22	1	TCTCCCAATCCTTGTACCAGTG	miRNA	miR-150
t0084598	22	1	TTTCCCAACCCTTGTACCAGTG	miRNA	miR-150
t0090799	21	1	TCTCACAACCCTTGTACCAGT	miRNA	miR-150
t0092797	22	1	TCTCCCAACCCTCGTACCAGTA	miRNA	miR-150
t0082270	23	1	TCTGCCAACCCTTGTACCAGTGT	miRNA	miR-150
t0083040	22	1	TCTCCCAACCCTTGTACCAGAG	miRNA	miR-150
t0083446	23	1	TCTCCCAACCCTTGAACCAGTGT	miRNA	miR-150
t0042740	21	1	TCTCCCAACCCTTGTACCAGC	miRNA	miR-150
t0042530	22	1	TCTCACAACCCTTGTACCAGTG	miRNA	miR-150
t0042679	21	1	TCTCCCAACCCTTGTACCGGT	miRNA	miR-150
t0044160	22	1	TCTCCCAACCATTGTACCAGTG	miRNA	miR-150
t0047233	21	1	TCTCCCAACCCTTGTACCAGA	miRNA	miR-150
t0050264	23	1	TCTCCCAACCCTTGTACCAGAGT	miRNA	miR-150
t0052297	21	1	TCTCCCAACCCTTGTACAAGT	miRNA	miR-150
t0055449	23	1	TCTCCCAACCCTTGGACCAGTGA	miRNA	miR-150
t0057428	21	1	TCTCCAAACCCTTGTACCAGT	miRNA	miR-150
t0059499	20	1	TCTCCCAACCCTTGTATCAG	miRNA	miR-150
t0060404	21	1	TCTCCCAACCCTTATACCAGT	miRNA	miR-150

t0061174	22	1	TCTCCCAAACCTTGTACCAGTG	miRNA	miR-150
t0062081	21	1	TCTTCCAACCCTTGTACCAGT	miRNA	miR-150
t0062952	21	1	TCTCCCAACCCTTGGACCAGT	miRNA	miR-150
t0064848	21	1	TATCCCAACCCTTGTACCAGT	miRNA	miR-150
t0066869	18	1	TATTGCTGTACCGGCCTG	miRNA	miR-1492
t0066974	20	1	TATTGCTGTACCGGCCTGTA	miRNA	miR-1492
t0068649	18	1	TATTGCACTACCGGCCTG	miRNA	miR-1492
t0070982	21	1	TCAGTGCATCACCGAACTTTG	miRNA	miR-148b-3p
t0072983	21	1	TCAGTGCGTCACAGAACTTTG	miRNA	miR-148b-3p
t0073060	21	1	TCAGTGTATCACAGAACTTTG	miRNA	miR-148b-3p
t0076727	21	1	TAAGTGCATCACAGAACTTTG	miRNA	miR-148b-3p
t0078469	27	1	TCAGTGCATCACAGAACTTTGTATATC	miRNA	miR-148b-3p
t0080717	19	1	TCAGTGCTTCACAGAACTT	miRNA	miR-148b-3p
t0081652	21	1	TCAGTGCATCACAGAACTTTA	miRNA	miR-148b-3p
t0086443	18	1	TCAGAGCATCACAGAACT	miRNA	miR-148b-3p
t0090465	21	1	TCAGTGAATCACAGAACTTTG	miRNA	miR-148b-3p
t0092738	21	1	TCAGCGCATCACAGAACTTTG	miRNA	miR-148b-3p
t0057949	18	1	TCAGTGCATCACATAACT	miRNA	miR-148b-3p
t0084820	20	1	TCAGGGCATCACAGAACTTT	miRNA	miR-148b-3p
t0092972	19	1	CAGTGCACTACAGAAGTTT	miRNA	miR-148a
t0051277	25	1	TCAGTGCACTACAGAAATTTGGTAG	miRNA	miR-148a
t0057490	25	1	TCAGTGCACTACAGAACTTTGGATC	miRNA	miR-148a
t0061599	23	1	TCAGTGCACTACAGAACTTTGGA	miRNA	miR-148a
t0062213	23	1	TCAGTGCACTCCAGAACTTTGAA	miRNA	miR-148a
t0062322	21	1	TCAGTGCACTACAGAACCTTG	miRNA	miR-148a
t0070544	22	1	TCAGGGCACTACAGAACTTTGT	miRNA	miR-148a
t0071493	18	1	TCAGTGCACTCCAGAACT	miRNA	miR-148a
t0071715	18	1	TCAGTCCACTACAGAACT	miRNA	miR-148a
t0075600	25	1	TCAGTGCACTACGGAACTTTGTATC	miRNA	miR-148a
t0083596	22	1	TCAATGCACTACAGAACTTTGA	miRNA	miR-148a
t0083714	25	1	TCGGTGCACTACAGAACTTTGTATC	miRNA	miR-148a
t0090806	21	1	TAAGTGCACTACAGAACTTTG	miRNA	miR-148a
t0040957	21	1	CCAGTGCACTACAGAACTTTG	miRNA	miR-148a
t0041037	22	1	TCAGTGTACTACAGAACTTTGA	miRNA	miR-148a
t0042083	25	1	TCAGTGCACTACAGAACTTTGCGTG	miRNA	miR-148a
t0044149	21	1	TTAGTGCACTACAGAACTTTG	miRNA	miR-148a
t0044355	21	1	TCAGTACACTACAGAACTTTG	miRNA	miR-148a
t0044810	20	1	TCAGTGCACTACAGAGCTTT	miRNA	miR-148a
t0045548	21	1	TCAGTGCACTACAGAACTTTT	miRNA	miR-148a
t0049813	22	1	CCAGTGCACTACAGAACTTTGA	miRNA	miR-148a
t0052440	25	1	TCAGTGCACTACAGGACTTTGTATC	miRNA	miR-148a
t0052856	19	1	TCAGTACACTACAGAACTT	miRNA	miR-148a
t0053638	20	1	TCAGTGAACTACAGAACTTT	miRNA	miR-148a
t0062816	23	1	TCAGAGCACTACAGAACTTTGAA	miRNA	miR-148a
t0064938	20	1	TCAGTACATTACAGAACTTT	miRNA	miR-148
t0065638	23	1	TGAGAACTGAATTCCATAGACTG	miRNA	miR-146c
t0066169	24	1	TGAGAACTGAATTCCATAGGCATC	miRNA	miR-146b-5p
t0066760	26	1	TGAGAACTGAATTCCATGGGTTTATC	miRNA	miR-146b-5p
t0067485	25	1	TGAGAACTGAATTCCATAGGCTGGA	miRNA	miR-146b
t0067603	22	1	CGAGAACTGAATTCCATAGGCT	miRNA	miR-146b
t0073150	27	1	TGAGAACTGAATTCCATAGGTTGTATC	miRNA	miR-146b
t0074734	26	1	TGAGAACTGAATTCCATAGGCTGATC	miRNA	miR-146b
t0077022	23	1	TGAGAACTGAATTCCATCGGCTG	miRNA	miR-146b
t0079918	27	1	TGAGAACTGAATTCCATCGGCTGTATC	miRNA	miR-146b
t0081438	27	1	TGAGAACTGAGTTCCATAGGCTGTATC	miRNA	miR-146b
t0090208	23	1	GGATATCATCATACACTGTAAGT	miRNA	miR-144*
t0092481	22	1	GGATATCATCATATCCTGTAAG	miRNA	miR-144*
t0057240	23	1	GGATATCATCATATACTATAAGT	miRNA	miR-144*

t0075104	20	1	AGATATCATCATATACTGTA	miRNA	miR-144*
t0062960	21	1	GGATATCATCATAAACTGTAA	miRNA	miR-144*
t0065966	23	1	GGATATCATCATATGCTGTAAGT	miRNA	miR-144*
t0053593	20	1	GGATATCATCATATATTGTA	miRNA	miR-144*
t0054355	23	1	GGGTATCATCATATACTGTAAGT	miRNA	miR-144*
t0059859	24	1	GGATATAATCATATACTGTAAGTT	miRNA	miR-144*
t0066343	21	1	GGATACCATCATATACTGTAA	miRNA	miR-144*
t0080894	21	1	GGATATCATCATACACTGTAA	miRNA	miR-144*
t0083573	21	1	GGATATCATCATATACTGTAG	miRNA	miR-144*
t0090501	21	1	TGATATCATCATATACTGTAA	miRNA	miR-144*
t0040991	23	1	GGATATCAATATATACTGTAAGT	miRNA	miR-144*
t0042206	23	1	GGATACCATCATATACTGTAAGT	miRNA	miR-144*
t0042572	22	1	GGATACCATCATATACTGTAAG	miRNA	miR-144*
t0043388	20	1	GGATATCATCATATACTATA	miRNA	miR-144*
t0044559	23	1	GGATATCATTATATACTGTCAGT	miRNA	miR-144*
t0045437	23	1	GGATATCAACATATACTGTAAGT	miRNA	miR-144*
t0045850	23	1	GGATAACATCATATACTGTAAGT	miRNA	miR-144*
t0049099	23	1	GGATATCATGATATACTGTAAGT	miRNA	miR-144*
t0049834	21	1	GAATATCATCATATACTGTAA	miRNA	miR-144*
t0051002	23	1	GGATATCATCATATACTGTATGT	miRNA	miR-144*
t0051298	20	1	GGATATCATCATATACTGAA	miRNA	miR-144*
t0052207	24	1	GGATATCATCATACACTGTAAGTT	miRNA	miR-144*
t0052510	21	1	GGATATCATCATATACCGTAA	miRNA	miR-144*
t0053041	22	1	GGATATCATCATATACTGTAGG	miRNA	miR-144*
t0053348	23	1	GGATATCATTATATACTGTACGT	miRNA	miR-144*
t0053888	24	1	GATATCATCATATACTGTAAGTTT	miRNA	miR-144*
t0053921	18	1	GGATATCATCATAAACTG	miRNA	miR-144*
t0055162	23	1	GGATATCATCATATACTGGAAGT	miRNA	miR-144*
t0057487	20	1	GGATCTCATCATATACTGTA	miRNA	miR-144*
t0058503	24	1	GGATATCATCATATACTGCAAGTT	miRNA	miR-144*
t0059139	22	1	CGATATCATCATATACTGTAAG	miRNA	miR-144*
t0059728	23	1	GGATATCATCATATACTGAAAGT	miRNA	miR-144*
t0060060	22	1	GGATATCATCGTATACTGTAAG	miRNA	miR-144*
t0060264	22	1	GGATATCATCATATACTGCAAG	miRNA	miR-144*
t0061056	22	1	GTATATCATCATATACTGTAAG	miRNA	miR-144*
t0062971	22	1	GCATATCATCATATACTGTAAG	miRNA	miR-144*
t0063043	23	1	GGATATCATCATCTACTGTAAGT	miRNA	miR-144*
t0064042	22	1	GGTTATCATCATATACTGTAAG	miRNA	miR-144*
t0064990	21	1	GGATATCATCATATAATGTAA	miRNA	miR-144*
t0065517	23	1	GGATATCATCATGTACTGTAAGT	miRNA	miR-144*
t0068327	21	1	GGATAACATCATATACTGTAA	miRNA	miR-144*
t0069984	23	1	GGATATCATCATATAATGTAAGT	miRNA	miR-144*
t0071258	23	1	GGATATCATCATATACTGTAACT	miRNA	miR-144*
t0073329	22	1	TACAGTATAGATGATGTATTAT	miRNA	miR-144
t0075477	21	1	TATAGTATAGATGATGTACTA	miRNA	miR-144
t0082624	20	1	AATACAGTATAGATGAAGTA	miRNA	miR-144
t0084988	21	1	ATACAGTTTAGATGATGTACT	miRNA	miR-144
t0085804	19	1	ATACAGTATAGATGATGCA	miRNA	miR-144
t0086337	19	1	TACAGGATAGATGATGTAA	miRNA	miR-144
t0086750	22	1	TACAGTATAGATGATGTAATAT	miRNA	miR-144
t0087473	18	1	CTACAGTATAGATGGTGT	miRNA	miR-144
t0090689	21	1	TACAGTATAGATGATGTACCA	miRNA	miR-144
t0092385	21	1	TACAGTATAGATGATGGAATT	miRNA	miR-144
t0092496	20	1	TACAGTATAGATGATGTCAA	miRNA	miR-144
t0093497	19	1	CTATAGTATAGATGATGTA	miRNA	miR-144
t0093543	18	1	TTCAGTATAGATGATGTA	miRNA	miR-144
t0041620	22	1	TACGGTATAGATGATGTACTAA	miRNA	miR-144
t0042372	19	1	CTACAGTATATATGATGTA	miRNA	miR-144

t0042684	18	1	TACAGCATAGATGATGTA	miRNA	miR-144
t0042893	21	1	ACAGTATAGATGTTGTACTTT	miRNA	miR-144
t0043011	19	1	TACAGTATAGATGAGGTAA	miRNA	miR-144
t0043856	23	1	TACAGTATAGATGATGTCCATAA	miRNA	miR-144
t0046876	21	1	GACAGTATAGATGATGTACTT	miRNA	miR-144
t0047272	19	1	CTACAGTATAGATGATATA	miRNA	miR-144
t0047369	19	1	CTACAGTGTAGATGATGTA	miRNA	miR-144
t0047843	20	1	CTACAGTATAGATGATGCAT	miRNA	miR-144
t0048224	18	1	TACAGTATAGATGGTGTA	miRNA	miR-144
t0048708	18	1	CTACAGTATGGATGATGT	miRNA	miR-144
t0048875	21	1	TACAGTGTAGATGATGTACTT	miRNA	miR-144
t0050166	18	1	TGCAGTATAGATGATGTA	miRNA	miR-144
t0050924	19	1	CTACCGTATAGATGATGTA	miRNA	miR-144
t0051250	20	1	TACAGTGTAGATGATGGACT	miRNA	miR-144
t0051665	21	1	TACAGTATAGATGATGTAAAC	miRNA	miR-144
t0052055	19	1	GTACAGTATAGATGATGTA	miRNA	miR-144
t0052717	19	1	CTACAGTATAGATGTTGTA	miRNA	miR-144
t0053675	19	1	CTACAGTATAGATGATTTA	miRNA	miR-144
t0054345	22	1	TACAGTATAGATAATGTACTAT	miRNA	miR-144
t0054399	18	1	TACAGTATAAATGATGTA	miRNA	miR-144
t0055147	19	1	TACAGTATAGATGATGAAC	miRNA	miR-144
t0056246	23	1	TACAGTAAAGATGATGTACATAA	miRNA	miR-144
t0056821	20	1	TACAGTATGGATGATGTACT	miRNA	miR-144
t0056974	18	1	TAAAGTATAGATGATGTA	miRNA	miR-144
t0057497	18	1	TACAGGATAGATGATGTA	miRNA	miR-144
t0057682	20	1	TGCAGTATAGATGATGTACT	miRNA	miR-144
t0058476	20	1	CACAGTATAGATGATGTATT	miRNA	miR-144
t0058829	19	1	CTACAGGATAGATGATGTA	miRNA	miR-144
t0059722	18	1	TACAGTATACATGATGTA	miRNA	miR-144
t0060267	20	1	TACAGTATAGTTGATGTACT	miRNA	miR-144
t0060351	18	1	TACAGTATAGAAGATGTA	miRNA	miR-144
t0061012	22	1	TACAGTATAGACGATGTACTAT	miRNA	miR-144
t0061705	23	1	TACAGTATAGATGATGTAAATAA	miRNA	miR-144
t0061743	20	1	TACAGTATAGATGACGTACT	miRNA	miR-144
t0061979	22	1	TACAGTATAGATGATGTCCTAA	miRNA	miR-144
t0062715	19	1	CTACAGTTTAGATGATGTA	miRNA	miR-144
t0063158	22	1	TACAGTATAGATGGTGTACTAT	miRNA	miR-144
t0064530	19	1	CTACAGCATAGATGATGTA	miRNA	miR-144
t0064831	20	1	ATACAGTATAGCTGATGTAA	miRNA	miR-144
t0065087	21	1	ATACGGTATAGATGATGTACT	miRNA	miR-144
t0065294	20	1	TACAGTATATATGATGTACT	miRNA	miR-144
t0066052	22	1	TACAGTATAGATGATGTACCAT	miRNA	miR-144
t0066203	19	1	TACAGCATAGATGATGTAG	miRNA	miR-144
t0066778	19	1	TCCAGTATAGATGATGTAA	miRNA	miR-144
t0068382	22	1	TACAGTATAGATGATGCACTTT	miRNA	miR-144
t0070399	21	1	TACAGTATAGATAATGTACTT	miRNA	miR-144
t0070681	20	1	TACAGTATAGATGATATTCT	miRNA	miR-144
t0070876	20	1	TACAGTGTAGATGATGTAAT	miRNA	miR-144
t0070985	21	1	TACAGTATAGATGATGGACTT	miRNA	miR-144
t0071444	20	1	TACAGTATAGATGATATACT	miRNA	miR-144
t0073790	19	1	TACAGTATAGATGTTGTAC	miRNA	miR-144
t0074055	18	1	TACACTATAGATGATGTA	miRNA	miR-144
t0074349	22	1	AATTACAGTATAGATGATGTAA	miRNA	miR-144
t0074703	20	1	TACAGTATAAATGATGTACT	miRNA	miR-144
t0075821	19	1	TACAGTATAGATGATGTCC	miRNA	miR-144
t0075977	21	1	TACAGTGTAGATGATGTACTA	miRNA	miR-144
t0078267	19	1	CTACGGTATAGATGATGTA	miRNA	miR-144
t0078520	22	1	TACAGTACAGATGATGTACTTT	miRNA	miR-144

t0080494	19	1	TAGAGTATAGATGATGTAC	miRNA	miR-144
t0081679	18	1	TACAGTATATATGATGTA	miRNA	miR-144
t0083278	25	1	TACAGTATAGATGACGTACTAAAAA	miRNA	miR-144
t0084278	19	1	CTACAGTATAGAAGATGTA	miRNA	miR-144
t0084308	21	1	TACAGTATGGATGATGTACTT	miRNA	miR-144
t0084956	19	1	CTACAGTATAGAGGATGTA	miRNA	miR-144
t0085437	21	1	TACAGTAGAGATGATGTTTTT	miRNA	miR-144
t0085549	20	1	TACAGTATAGATAATGTACT	miRNA	miR-144
t0086256	23	1	TGAGGTGAAGCACTGTAGCTATC	miRNA	miR-143
t0087991	25	1	TGAGATGAAGCACTGTAGCTCTAAC	miRNA	miR-143
t0089307	23	1	TGAGATAAAGCACTGTAGCTATC	miRNA	miR-143
t0089439	25	1	TGAGATGAAGCACTGTAGCTATATC	miRNA	miR-143
t0089721	23	1	TGAGATGAAGCAGTGTAGCTATC	miRNA	miR-143
t0089859	23	1	CGAGATGAAGCACTGTACCTATC	miRNA	miR-143
t0090447	23	1	TGAGATTAAGCACTGCAGCTATC	miRNA	miR-143
t0090815	23	1	TGAGATGAAGCATTGTAGCTCTC	miRNA	miR-143
t0090938	23	1	GGAGATGAAGCACTGTAGCTATC	miRNA	miR-143
t0091422	23	1	TGAGATGAAGCACTGTAGCTGTC	miRNA	miR-143
t0091838	23	1	TGAGATGATGCACTGTAGCTATC	miRNA	miR-143
t0093063	23	1	TGAGATGAAGCACTGTAGCTACC	miRNA	miR-143
t0093240	23	1	TGAGATGAAGCACTGTAGCACTC	miRNA	miR-143
t0041719	23	1	TGAGATGAAGGACTGTAGCTATC	miRNA	miR-143
t0043146	23	1	TGAAATGAAGCACTGTAGCTATC	miRNA	miR-143
t0049148	23	1	TGAGATGAAGCACTGTAGCGATC	miRNA	miR-143
t0049420	23	1	TGAGATGAAGCACTGTAGCTAAC	miRNA	miR-143
t0054946	23	1	TGAGATGAAGCACTGTAGGTATC	miRNA	miR-143
t0055818	23	1	TGAGACGAAGCACTGTAGCTATC	miRNA	miR-143
t0058388	23	1	TGAGATGAAGCACTGTGGCTATC	miRNA	miR-143
t0059796	26	1	TGAGATGAAGCACTGTAGCTCTAATA	miRNA	miR-143
t0060451	23	1	TGAGATGAAGCGCTGTAGCTATC	miRNA	miR-143
t0061494	23	1	TCAGATGAAGCACTGTAGCTATC	miRNA	miR-143
t0061739	23	1	TGAGATTAAGCACTGTAGCTGTC	miRNA	miR-143
t0064186	23	1	TGAGATGAAGCACTGTAGCCATC	miRNA	miR-143
t0065550	23	1	TGAGATGAAGTACTGTAGCTATC	miRNA	miR-143
t0067016	23	1	TGAGATGAATCACTGTAGCTATC	miRNA	miR-143
t0067528	19	1	CATAAAGTAGAAATCACTA	miRNA	miR-142-5p
t0072483	20	1	CATAAAGTAGAAAGCGCTAC	miRNA	miR-142-5p
t0074790	21	1	CATAAAGTAGTAAGCACTACT	miRNA	miR-142-5p
t0076184	22	1	ATAAAGTAGAAAGCACTACTAC	miRNA	miR-142-5p
t0076756	21	1	CATAAAGTGGAAAGCACCACT	miRNA	miR-142-5p
t0079733	21	1	CATAAAGTAGGAAGCACTACT	miRNA	miR-142-5p
t0080647	20	1	CATAAAGTAGAAAGAACTAC	miRNA	miR-142-5p
t0081157	19	1	CATAAAGTAGAAAGCGCTA	miRNA	miR-142-5p
t0084520	18	1	CATAAAGTAGAAAGCACC	miRNA	miR-142-5p
t0085380	21	1	ACCATAAAGTAGAAAGCACTA	miRNA	miR-142-5p
t0087475	18	1	TCCATAAAGTAGGAAGCA	miRNA	miR-142-5p
t0091549	20	1	CATAAAGTGGAAAGCACTAG	miRNA	miR-142-5p
t0092161	19	1	TATAAAGTAGAAAGCACTA	miRNA	miR-142-5p
t0045864	21	1	CATAAAGAAGAAAGCACTACT	miRNA	miR-142-5p
t0049122	21	1	CATAAAGGAGAAAGCACCACT	miRNA	miR-142-5p
t0049929	21	1	CATAAAGTAGAAAGCACCACC	miRNA	miR-142-5p
t0051342	21	1	CATAAAGGAGAAAGCACTACT	miRNA	miR-142-5p
t0051839	22	1	ATAAAGTAGAAAGAAATACTAA	miRNA	miR-142-5p
t0053303	21	1	CACATAAAGTAGAAAGCACTA	miRNA	miR-142-5p
t0053840	19	1	CATAAAGTAGAAAGAAATA	miRNA	miR-142-5p
t0054585	21	1	GTGGTGTTTCCTACTTTATGG	miRNA	miR-142-3p
t0054875	22	1	GTAGTGTTTCCTACTTTATAGT	miRNA	miR-142-3p
t0057833	21	1	GTAGTGTTTATTACTTTATGG	miRNA	miR-142-3p

t0065323	21	1 GTAGGGTTTCCTACTTTATGG	miRNA	miR-142-3p
t0066279	22	1 GTAGTGGTTCCTACTTTATGGT	miRNA	miR-142-3p
t0069859	22	1 GTAGTATTTCCTACTTTATGGA	miRNA	miR-142-3p
t0072062	21	1 GTAGAGTTTCCTACTTTATGG	miRNA	miR-142-3p
t0075907	22	1 GTAGTGTTTCCGACTTTATGGA	miRNA	miR-142-3p
t0087472	21	1 GTAGTGTTTCTTACTTTATGG	miRNA	miR-142-3p
t0087934	21	1 GTAGCGTTTCCTACTTTATGG	miRNA	miR-142-3p
t0088879	21	1 GTAGTGTTCCCTACTTTATGG	miRNA	miR-142-3p
t0090137	23	1 GTAGGGTTTCCTACTTTATGGCA	miRNA	miR-142-3p
t0092984	21	1 GTAGTGTTTCCTACTTTAAGG	miRNA	miR-142-3p
t0045971	18	1 CCCATAAAGTAGGAAGCA	miRNA	miR-142
t0046284	19	1 CCCATAAAGTAGCAAGAAC	miRNA	miR-142
t0047490	19	1 CCCATGAAGTAGAAAGCAC	miRNA	miR-142
t0049524	20	1 CCTATAAAGTAGAAAGCAAT	miRNA	miR-142
t0052242	21	1 CCCATAAAGTAGAAAGCACTT	miRNA	miR-142
t0054581	22	1 CCCATAAAGTAGAAAGCAATAC	miRNA	miR-142
t0056479	19	1 CCCATAAAGTAGAAAACAC	miRNA	miR-142
t0057812	20	1 CCATAAAGTAGAAAGCACTA	miRNA	miR-142
t0069432	22	1 CCTATAAAGTAGAAAGCACTAT	miRNA	miR-142
t0073891	22	1 CCCACAAAGTAGAAAGCACTAA	miRNA	miR-142
t0078902	20	1 CCCATAAAGTAGAAAACACT	miRNA	miR-142
t0083791	22	1 CCCATAAAGTAGATAGCACTAA	miRNA	miR-142
t0092736	18	1 CCCATAAAGTAGCAAGCA	miRNA	miR-142
t0041417	20	1 CCCATAAAGAAGAAAACACT	miRNA	miR-142
t0045182	20	1 CCCATAGAGTAGAAAGCACT	miRNA	miR-142
t0045853	21	1 CCCATAAAGTAGCAAGCACTA	miRNA	miR-142
t0045987	20	1 CCCATAAAGCAGAACGCACT	miRNA	miR-142
t0048149	20	1 CCCATAAAGTAGAAAGCACA	miRNA	miR-142
t0049345	22	1 CCCATAAAGTAGAAAGAACTAT	miRNA	miR-142
t0049623	20	1 CCCATAAGGTAGAAAGCACT	miRNA	miR-142
t0050660	20	1 CCCATAAAGTAGAAAGAATT	miRNA	miR-142
t0050706	22	1 CCCAAAAAGTAGAAAGCACTAA	miRNA	miR-142
t0051349	20	1 CCCATAAACTAGAAAGCACT	miRNA	miR-142
t0055360	20	1 CCCATAAAGTAGAAACCACT	miRNA	miR-142
t0056686	18	1 CCCATGAAGTAGAAAGCA	miRNA	miR-142
t0057201	20	1 CCCTTAAAGTAGAAAGCACT	miRNA	miR-142
t0061419	22	1 CCCATAAAGTAGAAAGAACTAG	miRNA	miR-142
t0062224	19	1 CCTATAAAGTAGAAAGCAC	miRNA	miR-142
t0062256	20	1 CCTATCAAGTAGAAAGCACT	miRNA	miR-142
t0064449	18	1 CCCGTAAAGTAGAAAGCA	miRNA	miR-142
t0065057	21	1 CCCATAAAGTAGAAAGATCTA	miRNA	miR-142
t0065423	23	1 CCCATAAAGTAGAAAGCACTCGA	miRNA	miR-142
t0066521	22	1 CCCATAAAGTGGAAAGCACTAT	miRNA	miR-142
t0067371	19	1 CCCAAAAAGTAGAAAGCAC	miRNA	miR-142
t0068740	20	1 CCCATATAGTAGAAAGCACT	miRNA	miR-142
t0069672	19	1 CCCACAAAGTAGAAAGCAC	miRNA	miR-142
t0069788	21	1 CCCATAAAGTAGAACGCACTA	miRNA	miR-142
t0070705	20	1 CCCATAAAGTAGAAAGCGCT	miRNA	miR-142
t0071542	25	1 CCCATAAAGTAGAAAGCACTGCATC	miRNA	miR-142
t0074563	21	1 CCCATAAAGTAGAACGAACTA	miRNA	miR-142
t0074608	20	1 CATAAAGTAGAAAGCACTCA	miRNA	miR-142
t0074890	21	1 CCCTATAAAGTAGAAAGCACT	miRNA	miR-142
t0075609	20	1 CCCACAAAGTAGAAAGCACT	miRNA	miR-142
t0075733	20	1 CCCATAAAGTATAAAGCCCT	miRNA	miR-142
t0076030	20	1 CCCATAAAGTAGGAAGCACT	miRNA	miR-142
t0076543	21	1 CCCATAAAGTACAAAGCACTA	miRNA	miR-142
t0077397	23	1 TACCACTGGGTAGAATCACGGAT	miRNA	miR-140
t0080540	24	1 TACCACAGGGTACAACCAAGGACG	miRNA	miR-140

t0081117	22	1	TAACACAGGGTAGAACTACGGA	miRNA	miR-140
t0081156	23	1	ACCACAGGGTAGAATCACGGAAA	miRNA	miR-140
t0084327	22	1	TACAACAAGGTAGAACCACGGA	miRNA	miR-140
t0084775	23	1	ACCCCAGGGTAGAACCACGGACT	miRNA	miR-140
t0088404	23	1	ACCACAGGGTAGGACCACGGACG	miRNA	miR-140
t0088808	21	1	CCACAGGGGAGAACCACGGAC	miRNA	miR-140
t0090354	24	1	TACCGCAGGGTAGAACCACGGACT	miRNA	miR-140
t0091489	23	1	TACCACAGGGTGGAACCACAGAA	miRNA	miR-140
t0093046	22	1	ACAACAGGGTAGAACCACGGAG	miRNA	miR-140
t0093148	22	1	TACCACAGGGTCGAACTACGGA	miRNA	miR-140
t0093195	23	1	ACCACAGGGTAGAACAACGGAAT	miRNA	miR-140
t0040675	21	1	ACCAAAGGGTAGAACCAAGGA	miRNA	miR-140
t0040690	24	1	TGCCACAGGGTAGAACCACGGACG	miRNA	miR-140
t0040811	23	1	ACCACAGGGTAGAAACACGGAAG	miRNA	miR-140
t0040876	23	1	ACCACGGGGTAGAACCACGGACG	miRNA	miR-140
t0041069	22	1	ACCACAGGGTAGAACCAGGGCA	miRNA	miR-140
t0041132	24	1	TACCACAGGGTAGAACCCCGGTAA	miRNA	miR-140
t0041239	21	1	ACCACGGGGAAGAACCACGGA	miRNA	miR-140
t0041310	22	1	TACCACAGGGCAGAACTACGGA	miRNA	miR-140
t0041344	24	1	TACCACAGGGTAGAATCACAGACA	miRNA	miR-140
t0041444	23	1	TACCACAGGGCAGCACCACGGAC	miRNA	miR-140
t0041487	22	1	TACCACATGGTAGAACCACGGC	miRNA	miR-140
t0041641	22	1	ACCACAGGGTAGAAGCACGGAC	miRNA	miR-140
t0041725	23	1	ACCACAGGGTAGCACCATGGACG	miRNA	miR-140
t0041832	23	1	ACCACATGGTAGAACCACGGACT	miRNA	miR-140
t0041837	21	1	ACCACAGGGAAGAACTACGGA	miRNA	miR-140
t0041900	24	1	TACCACAGGGTAGAAACACGGACT	miRNA	miR-140
t0041953	22	1	TACCACAGGGTAGAAACACGGT	miRNA	miR-140
t0042032	22	1	ACGACAGGGTAGAACCACGGAT	miRNA	miR-140
t0042124	21	1	CCACAGGGTACAACCACGGAT	miRNA	miR-140
t0042278	24	1	TACCACAGGGTAGAAACACGGATA	miRNA	miR-140
t0042403	23	1	ACCACAGGGTAGGACCACGGACT	miRNA	miR-140
t0042405	24	1	TACCACAGGGTACAACCATGGACG	miRNA	miR-140
t0042444	23	1	ACCACAGGGTAGAAGCACGGACA	miRNA	miR-140
t0042516	24	1	TAACACAGGGTAGAACCACGGACG	miRNA	miR-140
t0042525	22	1	TACCACAGGGTAGAACCAAGGC	miRNA	miR-140
t0042528	21	1	CCACAGGGTAGAACCACGGCT	miRNA	miR-140
t0042712	22	1	CCACAGGGTAGAACCCCGGACT	miRNA	miR-140
t0042738	22	1	ACCACAGGCTAGAACCACGGAT	miRNA	miR-140
t0042797	23	1	ACCACAGGGTAGAACTACGGGCA	miRNA	miR-140
t0042874	23	1	GCCACAGGGTAGAACCAAGGACA	miRNA	miR-140
t0042936	21	1	CCACAGGGTCGAACCACGGAT	miRNA	miR-140
t0042953	23	1	ACAACAGGGTAGAACCAAGGACG	miRNA	miR-140
t0042986	24	1	TACCACAGGGGAGAACAACGGACA	miRNA	miR-140
t0043000	22	1	TACCACAGGGAAGAACTACGGA	miRNA	miR-140
t0043033	23	1	TACCAGAGGGTAGAACCACGGAA	miRNA	miR-140
t0043160	21	1	ACCACAGAGTAGAACTACGGA	miRNA	miR-140
t0043251	21	1	ACTACGGGGTAGAACCACGGA	miRNA	miR-140
t0043391	24	1	TACCACAGGGTAGAACCACGGCGT	miRNA	miR-140
t0043399	22	1	TACCACAGGGTATAACTACGGA	miRNA	miR-140
t0043462	19	1	CACAGGGTAGAACAACGGA	miRNA	miR-140
t0043468	21	1	ACCACAGGGTAAAACAACGGA	miRNA	miR-140
t0043544	22	1	ACCACAGGGTCGAACCACGGAG	miRNA	miR-140
t0043558	24	1	TACCACAGGGTAGACCCACGGACA	miRNA	miR-140
t0043729	23	1	TAACACAGGGTAGAACAACGGAA	miRNA	miR-140
t0043792	23	1	TACCACAGGGTAGAACCAAGAAC	miRNA	miR-140
t0043884	23	1	TACCACAGGGTAGATCCAAGGAA	miRNA	miR-140
t0043890	23	1	ACCACAGGGTAGAAAAACGGACA	miRNA	miR-140

t0043895	21	1	TACCACAGGGTGGAACCACGG	miRNA	miR-140
t0043984	22	1	ACCACAGGGTAGAGCCACGGAG	miRNA	miR-140
t0043992	21	1	ACTACAGGGTAGAAACACGGA	miRNA	miR-140
t0044017	23	1	TAACAAAGGGTAGAACCACGGAA	miRNA	miR-140
t0044094	24	1	TACCACAGGGGAGAACCACGGATC	miRNA	miR-140
t0044224	24	1	CTACCACAGGGTAGAACCACGGCC	miRNA	miR-140
t0044310	22	1	TAGCACAGGGTAGAACCCCGGA	miRNA	miR-140
t0044334	22	1	AACACAGGGTAGAAACACGGAA	miRNA	miR-140
t0044459	21	1	ACCACAGGGCAGAACTACGGA	miRNA	miR-140
t0044568	22	1	GCCACAGGGTAGAACCACGGAG	miRNA	miR-140
t0044601	23	1	TACCACACGGTAGAACCCCGGAC	miRNA	miR-140
t0044626	23	1	ACCACAGGGTAGAACCCCGGAAA	miRNA	miR-140
t0044652	22	1	TACCACAAGGTAGAACAACGGA	miRNA	miR-140
t0044706	22	1	TACCCTAGGGTAGAACCACGGA	miRNA	miR-140
t0044738	24	1	TACCACAGGGTAGAACCCCGGGCA	miRNA	miR-140
t0044791	23	1	TCCACAGGGTAGAACCACGGACA	miRNA	miR-140
t0045111	24	1	ACAACAGGGTAGAACCACGGAAGA	miRNA	miR-140
t0045202	22	1	TACCACAGGGTAGAACTGCGGA	miRNA	miR-140
t0045217	23	1	TACCACAGGGTAGAAATACGGAC	miRNA	miR-140
t0045238	22	1	ACCACAGGGTAGAAGCACGGAT	miRNA	miR-140
t0045292	24	1	TACCACAGGGTAGAACCACGGCTA	miRNA	miR-140
t0045349	24	1	TACCACTGGGTAGAACCACGGACT	miRNA	miR-140
t0045421	20	1	CCACAGGGTAAAACCACGGA	miRNA	miR-140
t0045429	21	1	ACCACAGGGTAGAACTATGGA	miRNA	miR-140
t0045608	23	1	TACCACAGGGTCGAACTACGGAA	miRNA	miR-140
t0045616	26	1	TACCACAGGGTAGAACAACGGACAAT	miRNA	miR-140
t0045657	23	1	ACAACAGGGTAGAACCACGGCCT	miRNA	miR-140
t0045725	23	1	TACCACAGGGCAGAACCACGGCC	miRNA	miR-140
t0045751	23	1	ACCACAGGGTAGAACCCCGGACG	miRNA	miR-140
t0045866	21	1	ACCACAAGTTAGAACCACGGA	miRNA	miR-140
t0045972	23	1	TACCATAGGGTAGAACCACGGCA	miRNA	miR-140
t0046064	21	1	GCCACAGGGGAGAACCACGGA	miRNA	miR-140
t0046210	24	1	TACCATAGGGTAGAACCACGGAAA	miRNA	miR-140
t0046262	21	1	ACCACAGGGTATAAACACGGA	miRNA	miR-140
t0046265	23	1	TACCACAGGGCAGAACCACGGCA	miRNA	miR-140
t0046308	21	1	ACCACAGGGCAGAATCACGGA	miRNA	miR-140
t0046441	22	1	ACCACAGGATAGAACCACGGAT	miRNA	miR-140
t0046525	21	1	ACAACAGGGTAGAACCAAGGA	miRNA	miR-140
t0046527	21	1	ACCACAGGGATGAACCACGGA	miRNA	miR-140
t0046574	22	1	ACCACAGGGTAGAACCGCGGAG	miRNA	miR-140
t0046588	21	1	TACCACAGGGTAGCACCACGG	miRNA	miR-140
t0046589	23	1	TACCACAGGGGAGAACCCCGGAC	miRNA	miR-140
t0046698	21	1	ACCACAGGGTAGAACCTCGGC	miRNA	miR-140
t0046875	23	1	TACAACAGGGTAGAACCACGGAG	miRNA	miR-140
t0046899	23	1	ACCACAGGGAAGAACCACGGACC	miRNA	miR-140
t0046941	23	1	ACCACAGGGTAGAACCACAGATA	miRNA	miR-140
t0046985	23	1	AACACAGGGTAGAACCACGGCCG	miRNA	miR-140
t0047283	22	1	TACCGTAGGGTAGAACCACGGA	miRNA	miR-140
t0047334	24	1	TACCACAGGGGAGAACCAAGGACT	miRNA	miR-140
t0047393	23	1	ATCACAGGGTAGAACCACGGACA	miRNA	miR-140
t0047441	23	1	TAACACAGGGTAGAAACACGGAC	miRNA	miR-140
t0047631	24	1	TACCACAGGGTAGAACCACGGCGA	miRNA	miR-140
t0047653	24	1	TACCACAGGGTAGAATCACGGCCG	miRNA	miR-140
t0047993	24	1	TACCAAAGGGTAGAACCACGGCCA	miRNA	miR-140
t0048006	23	1	ACCACAGGCTAGAACCACGGACT	miRNA	miR-140
t0048029	21	1	ACCACAGGTTAGAACCACGGC	miRNA	miR-140
t0048035	21	1	CCACATGGTAGAACTACGGAT	miRNA	miR-140
t0048070	23	1	ACCACAGGGTAGACCCACGGACT	miRNA	miR-140

t0048357	24	1	TACAATAGGGTAGAACCACGGACA	miRNA	miR-140
t0048387	23	1	ACCCCAGGGTAGAACCACGGATA	miRNA	miR-140
t0048489	24	1	TACCACAGGGTAGAACCACGCAGA	miRNA	miR-140
t0048529	24	1	ACCACAGGGTAGAACAACGGACAA	miRNA	miR-140
t0048573	21	1	CTACAGGGTAGAACCACGGAA	miRNA	miR-140
t0048658	22	1	ACCACAGGGAAAAACCACGGAC	miRNA	miR-140
t0048669	21	1	ATCACAGGGTAGAACCACGGC	miRNA	miR-140
t0048676	22	1	ACCACAGGGTAGAACTACGAAT	miRNA	miR-140
t0048714	23	1	ACCAAAGGGTAGAACCACGGACT	miRNA	miR-140
t0048735	23	1	ACCACAGGGTATAACCACGGACG	miRNA	miR-140
t0048813	23	1	AGCACAGGGTAGAACCACGGAGT	miRNA	miR-140
t0048827	20	1	CCACAGGGTATAACCACGGA	miRNA	miR-140
t0048837	23	1	TACCACCGGGTAGAACCACGGCC	miRNA	miR-140
t0049017	24	1	TACCACAGGGTACAACCACGGACT	miRNA	miR-140
t0049131	22	1	TACCACAAGGTAGAACCAAGGA	miRNA	miR-140
t0049190	22	1	ACAACAGGGTAGAACAACGGAT	miRNA	miR-140
t0049200	20	1	CCACAGGGAAGAACCACGGA	miRNA	miR-140
t0049243	21	1	TACCACAGAGTAGAACCACGG	miRNA	miR-140
t0049366	24	1	TACCACAGGGGACAACCACGGACG	miRNA	miR-140
t0049379	22	1	TACCACAGGGTAGAACTACTGA	miRNA	miR-140
t0049427	23	1	ACCAAAGGGTAGAACAACGGAAG	miRNA	miR-140
t0049459	22	1	AACACAGGGTAGAACCAAGGAT	miRNA	miR-140
t0049625	24	1	TACCACAGGGTCGAACCACGGAAA	miRNA	miR-140
t0049696	23	1	TACCACAGGGTAGATCCACGGAT	miRNA	miR-140
t0049752	21	1	ACCAAAGGGTAGAACCACGGC	miRNA	miR-140
t0049756	22	1	ACCACAGGGTCGAACCACGGCC	miRNA	miR-140
t0049773	24	1	TACCACAGAGTAGAACCACGGACT	miRNA	miR-140
t0049868	22	1	TACCACAGTGTAGAACCACCGA	miRNA	miR-140
t0049872	23	1	TACAACAGGGTAGAACAACGGAC	miRNA	miR-140
t0049980	24	1	TACCAAAGGGTAGAACCACGGAAA	miRNA	miR-140
t0049988	22	1	TACCACAGGGTAGGACTACGGA	miRNA	miR-140
t0050010	23	1	TACCAGAGGGTAGAACCACGGCA	miRNA	miR-140
t0050075	24	1	TACGACAGGGTAGAACCACGGTCA	miRNA	miR-140
t0050078	23	1	TACCACACGGTAGAACCACGGAT	miRNA	miR-140
t0050190	22	1	ACCACCGGGTAGAACAACGGAC	miRNA	miR-140
t0050344	21	1	ACCACAGGGTAGACCCACGGC	miRNA	miR-140
t0050473	21	1	ACCACAGGGTAGAACAACGGC	miRNA	miR-140
t0050549	22	1	TACCACAGGGTATAACCTCGGA	miRNA	miR-140
t0050673	23	1	TACCAAAGGGTAGAACCACGAAC	miRNA	miR-140
t0050901	20	1	ACCACAGGGTAGAACCAGGG	miRNA	miR-140
t0050996	23	1	TACCACATGGTAGAACCACGGCC	miRNA	miR-140
t0051140	26	1	ACCACAGGGTAGAACCACGGAAGATA	miRNA	miR-140
t0051172	23	1	TACCAGAGGGTAGAACCACGGGC	miRNA	miR-140
t0051247	23	1	ACCACAGGGTAGAACCACTGACA	miRNA	miR-140
t0051255	23	1	TCCACAGGGTAGAACCACGGAAA	miRNA	miR-140
t0051289	23	1	TACCCCAGGGTAGAACCCCGGAA	miRNA	miR-140
t0051358	22	1	TACCGCAGGGTAGAACTACGGA	miRNA	miR-140
t0051399	23	1	TACCACAGGGTAGAGCTACGGAA	miRNA	miR-140
t0051576	22	1	TACCACAGGGTAGAGCCACGGT	miRNA	miR-140
t0051590	22	1	TAACACAGGGTAGAATCACGGA	miRNA	miR-140
t0051791	26	1	TACCACAGGGTAGAACCACGGCCAGA	miRNA	miR-140
t0051805	21	1	ATCACAGGGTAGAACTACGGA	miRNA	miR-140
t0051831	24	1	TACCACATGGTGGAACCACGGAAA	miRNA	miR-140
t0051878	23	1	TACCACAGGGTAGAACCCCGGCG	miRNA	miR-140
t0051962	23	1	TACCATAGGGTAGAACCACGGGC	miRNA	miR-140
t0052012	23	1	TACCACAGGGTAGAAACACGGTA	miRNA	miR-140
t0052048	23	1	TACCACAGGGTAGACACACGGAC	miRNA	miR-140
t0052106	23	1	TACCACAGGGTAGAAACACCGAA	miRNA	miR-140

t0052287	21	1	ACCACAGGGTAGAATAACGGA	miRNA	miR-140
t0052335	23	1	TACCACAGGTTAGAACCACGGAA	miRNA	miR-140
t0052394	22	1	ACCACAGGGTTGAACCACGGAC	miRNA	miR-140
t0052447	23	1	ATCACAGGGTAGAACCACGGACT	miRNA	miR-140
t0052555	24	1	TACCACAGGGTAGAACCATGGACG	miRNA	miR-140
t0052666	23	1	ACCACAGGGTAGAACCATGGACT	miRNA	miR-140
t0052732	22	1	ACCACAGGGTAGAAGCACGGAG	miRNA	miR-140
t0052737	22	1	ACCACAGGGTAGCACAACGGAC	miRNA	miR-140
t0052765	23	1	TACCACAGGGTAGAACCACGAAG	miRNA	miR-140
t0052802	23	1	TACCATAGGGTAGAACCCCGGAA	miRNA	miR-140
t0052823	22	1	CACCACAGGGTAGAACCAAGGA	miRNA	miR-140
t0052876	23	1	TACACAGGGTAGAACCACGGACG	miRNA	miR-140
t0052923	27	1	ACCACAGGGTAGAACCACGGACGAATC	miRNA	miR-140
t0052938	22	1	TACCACAGGATAGAACCCCGGA	miRNA	miR-140
t0052950	21	1	ACCACATGGTAGAACAACGGA	miRNA	miR-140
t0052955	20	1	CCACAGGGTAGAACCACGGC	miRNA	miR-140
t0052970	20	1	CACAGGGTAGAACCACGGTA	miRNA	miR-140
t0052986	24	1	TATCACAGGGTAGAACCACGGCAA	miRNA	miR-140
t0053179	20	1	ACCACAGGGTAGAACTGCGG	miRNA	miR-140
t0053340	24	1	TACCACAGGGTAGAACCACGACAA	miRNA	miR-140
t0053417	22	1	TACCACAGGGCAGAACCACGGT	miRNA	miR-140
t0053530	21	1	ACCACAGGGCAGAACCAAGGA	miRNA	miR-140
t0053667	23	1	TACCACAGGGTAGAAACACGGCA	miRNA	miR-140
t0053719	24	1	ACCACAGGGTAGAACCGCGGAAGA	miRNA	miR-140
t0053731	20	1	CCACAGGGTAGACCCACGGA	miRNA	miR-140
t0053811	22	1	ACCACAGGGTACAACCAAGGAC	miRNA	miR-140
t0053816	23	1	TACCACAGGGGAGAACCACGGCA	miRNA	miR-140
t0053821	22	1	ACTACAGGGTAGAACCACGGAG	miRNA	miR-140
t0053901	23	1	CACCACAGGGTAGAACCCCGGAC	miRNA	miR-140
t0053966	24	1	TACCACAGGGTGGAACCACGGACG	miRNA	miR-140
t0054023	22	1	AACACAGGGTAGAAACACGGAT	miRNA	miR-140
t0054057	23	1	ACCACAGGGTAGAACAACGGACG	miRNA	miR-140
t0054085	22	1	ACCACCGGGTAGAACCACGGCT	miRNA	miR-140
t0054137	22	1	ACCACAGGGTAGAACCACGCAC	miRNA	miR-140
t0054226	23	1	ACCACAGGGTAGAACCACGGTCT	miRNA	miR-140
t0054406	21	1	CCACAGGATAGAACCACGGAT	miRNA	miR-140
t0054423	21	1	CCACAGGGTATAACCACGGAA	miRNA	miR-140
t0054424	23	1	ACCACAGGGGAGAACCACGGATA	miRNA	miR-140
t0054446	23	1	TACGACAGGGTAGAACTACGGAC	miRNA	miR-140
t0054555	24	1	ACCACAGGGTAAAACCACGGAGGA	miRNA	miR-140
t0054589	23	1	TACAACAGGGTAGAACCAAGGAG	miRNA	miR-140
t0054764	21	1	CCACAGGGTAGAACCACGGCC	miRNA	miR-140
t0054863	22	1	TACCGCAGGGTAGAACCACGGC	miRNA	miR-140
t0054980	21	1	ACCACAGGCTAGAACCCCGGA	miRNA	miR-140
t0055021	22	1	ACAACAGGGTAGAACCCCGGAA	miRNA	miR-140
t0055030	21	1	ACCACAGGGAAGAACCAAGGA	miRNA	miR-140
t0055072	23	1	ACCACAGGGTGGAACCACGGACT	miRNA	miR-140
t0055118	21	1	ACCACAGGGGAGAACCATGGA	miRNA	miR-140
t0055244	22	1	TACCACAGGGTAGCACCACGGT	miRNA	miR-140
t0055264	20	1	ACCACAGGATAAAACCACGG	miRNA	miR-140
t0055326	22	1	ACCACATGGAAGAACCACGGAA	miRNA	miR-140
t0055446	22	1	ACCACAGGGTAGAACCACCGAG	miRNA	miR-140
t0055508	24	1	TACCACAGGGAAGAACCACGGAAA	miRNA	miR-140
t0055668	22	1	ACAACAGGGTAGAACCAAGGAC	miRNA	miR-140
t0055699	22	1	ACCACAGTGTAGAACCGCGGAA	miRNA	miR-140
t0055710	23	1	GACAACAGGGTAGAACCACGGAA	miRNA	miR-140
t0055760	23	1	TACCACGGGGTAGAACCACGGCA	miRNA	miR-140
t0055766	23	1	TACCACAGGGTAGAACTACGGCA	miRNA	miR-140

t0055768	23	1	TACCGCAGGGTAGAACCACGGAG	miRNA	miR-140
t0055801	24	1	TACCACAGGGTAGAACCACGTCCG	miRNA	miR-140
t0055868	23	1	TACAACAGGGTAGCACCACGGAA	miRNA	miR-140
t0055933	21	1	TATCACAGGGTAGAACCACGG	miRNA	miR-140
t0056019	22	1	ACAACAGGGTAGAACCACGGTC	miRNA	miR-140
t0056023	26	1	ACCACAGGGGAGAACCACGGACGGTG	miRNA	miR-140
t0056084	22	1	TACCAAAGGGAAGAACCACGGA	miRNA	miR-140
t0056110	23	1	TACCACCGGGTAGAACTACGGAA	miRNA	miR-140
t0056227	22	1	TACCGCACGGTAGAACCACGGA	miRNA	miR-140
t0056239	23	1	TACCACCGGGTAGAACCCCGGAC	miRNA	miR-140
t0056441	24	1	TACCACAGGGTAGAACCACGGGAA	miRNA	miR-140
t0056467	22	1	ACCCCAGGGTAGAACCACGGAT	miRNA	miR-140
t0056472	24	1	TACCACAGGGTAGAACACCGGACA	miRNA	miR-140
t0056544	22	1	ACCTCAGGGTAGAACCACGGAT	miRNA	miR-140
t0056569	22	1	ACCACGGGGTAGAACCGCGGAC	miRNA	miR-140
t0056590	23	1	TACAACAGGGTAGAACAACGGAA	miRNA	miR-140
t0056728	22	1	TACCGCAGGGGAGAACCACGGA	miRNA	miR-140
t0056807	23	1	TACCACAGGGTAGAAGCACGGAG	miRNA	miR-140
t0056891	23	1	TACCACATGGTAGAACCACGGAG	miRNA	miR-140
t0056944	21	1	CCACAGGGTAGAATCACGGAT	miRNA	miR-140
t0056956	25	1	TACCACAGGGGAGAACCACGGACAA	miRNA	miR-140
t0057030	23	1	TACAACAGGGTAGAAACACGGAA	miRNA	miR-140
t0057065	25	1	TACCACAGGGTAGAACCACGGGTAA	miRNA	miR-140
t0057242	22	1	TACCACAGGGTGGAACCACAGA	miRNA	miR-140
t0057339	22	1	ACCACAGGGTAGAACAAAGGAC	miRNA	miR-140
t0057548	23	1	TACCACAGGGTAGAACTACGGCT	miRNA	miR-140
t0057567	23	1	TACCACAGGGTAGAAACAAGGAG	miRNA	miR-140
t0057604	21	1	ACGACAGGGTAGAACCACGGC	miRNA	miR-140
t0057616	25	1	TACCACAGGGTAGAACCACGGACAC	miRNA	miR-140
t0057666	23	1	TACCACAGGGTATAACCCCGGAA	miRNA	miR-140
t0057685	20	1	ACCACAGGGTAGAACCATGG	miRNA	miR-140
t0057745	23	1	TACCACAGGGTAGGACCATGGAC	miRNA	miR-140
t0057773	21	1	ACCACAGGGAAGAACCACGTA	miRNA	miR-140
t0057879	22	1	ACCACAGGGGAGAACCACGGCG	miRNA	miR-140
t0057909	23	1	ACCACAGGGCAGAACCACGGACT	miRNA	miR-140
t0057971	22	1	TACCACAGAGTAGAAACACGGA	miRNA	miR-140
t0058064	21	1	ACCACAGGGTAGAACCAAGAA	miRNA	miR-140
t0058094	24	1	TACCACAGGGTAGAAACAAGGACG	miRNA	miR-140
t0058312	20	1	ACCACATGGTAGAACCACGG	miRNA	miR-140
t0058402	23	1	GACCACAGGGTAGAATCACGGAC	miRNA	miR-140
t0058477	22	1	CACCACAGGGTAGAACCACGGC	miRNA	miR-140
t0058502	23	1	ACAACAGGGTAGAACAACGGACG	miRNA	miR-140
t0058565	22	1	TCCCACCGGGTAGAACCACGGA	miRNA	miR-140
t0058630	24	1	TACCACAGGGTAGAACAACGGACT	miRNA	miR-140
t0058654	22	1	ACCACAGGGTAGAAACAAGGAA	miRNA	miR-140
t0058744	21	1	ACCACAGGTTAGGACCACGGA	miRNA	miR-140
t0058751	22	1	ACCACAGGGTAGAACTGCGGAT	miRNA	miR-140
t0058803	24	1	TAACACAGGGTACAACCACGGACG	miRNA	miR-140
t0058810	23	1	ACCACAGGGTACAACCACGGAAA	miRNA	miR-140
t0058811	21	1	CCCACAGGGTAGAACAACGGA	miRNA	miR-140
t0058884	25	1	ACCACAGGGTAGAACCACGGCCATC	miRNA	miR-140
t0059084	24	1	TACCACGGGGTAGAACCACGGAAT	miRNA	miR-140
t0059328	22	1	ACCACAGGGTAGAACGACGGAT	miRNA	miR-140
t0059338	23	1	ACCACAGGGAAGAACCACGGAGA	miRNA	miR-140
t0059396	28	1	AACACAGGGTAGAACAACGGACAAAAAA	miRNA	miR-140
t0059675	21	1	ACCGCAGGGTAGAACTACGGA	miRNA	miR-140
t0059730	20	1	ACCACAGGATAGAAACACGG	miRNA	miR-140
t0059741	22	1	TACCACAGGGTAGAGCTACGGA	miRNA	miR-140

t0059901	22	1	TACCACAGGGTATAACCACAGA	miRNA	miR-140
t0059972	24	1	TACCACAGGGTAGAACCACGTAAT	miRNA	miR-140
t0060035	21	1	ACCACAAGGTAGACCCACGGA	miRNA	miR-140
t0060061	22	1	ACCACAGGGTAGAACCACCGAA	miRNA	miR-140
t0060187	23	1	ACCACAGGGTAGAACAACGGAAG	miRNA	miR-140
t0060251	22	1	ACCACAAGGTAGAACCAAGGAA	miRNA	miR-140
t0060268	23	1	AACCACAGGGTAGAACCACGGGT	miRNA	miR-140
t0060280	23	1	TACCATAGGGTAGAACCACGGAG	miRNA	miR-140
t0060367	22	1	ACCACAGGGAAGAACCAAGGAT	miRNA	miR-140
t0060638	22	1	ACCACAGGGTAAAACCACGGAG	miRNA	miR-140
t0060654	20	1	ACCACAGGGTAGAACCACTG	miRNA	miR-140
t0060855	22	1	ACCACAGGGAAGAACAACGGAC	miRNA	miR-140
t0060906	21	1	ACCACAGGGTAGAAACCCGGA	miRNA	miR-140
t0060980	22	1	CCCACAGGGTAGAACCACGGAA	miRNA	miR-140
t0060995	21	1	ACCACACGGTAGAATCACGGA	miRNA	miR-140
t0061080	23	1	TACCACAGGGTACAACCCCGGAA	miRNA	miR-140
t0061107	23	1	TACCACAGGGTCGAACCACGGAT	miRNA	miR-140
t0061131	23	1	ACCACAGGGTAGAGCCACGGACT	miRNA	miR-140
t0061159	23	1	TACCACCTGGTAGAACCACGGAA	miRNA	miR-140
t0061170	22	1	ACCACAAGGTAGAACCACGGAG	miRNA	miR-140
t0061180	23	1	TACCACAGGGGAGAACCACGGCC	miRNA	miR-140
t0061258	20	1	CCACAGGGCAGAACCACGGA	miRNA	miR-140
t0061329	22	1	ATCACAGGGAAGAACCACGGAA	miRNA	miR-140
t0061385	23	1	CACCACAGGGTAGAGCCACGGAA	miRNA	miR-140
t0061410	23	1	ACCACAGGTTAGAACCACGGACT	miRNA	miR-140
t0061457	21	1	ACCACAGGGGAGAATCACGGA	miRNA	miR-140
t0061466	21	1	ACCACAGTGTAGAACCAGGGA	miRNA	miR-140
t0061520	23	1	ACCACAGGGTAGAACCACGCAGA	miRNA	miR-140
t0061721	26	1	ACCACAGGGTAGAACCACGGCCGGCC	miRNA	miR-140
t0061729	20	1	TACCACAGGGGAGAACCACG	miRNA	miR-140
t0061778	22	1	CCACAGGGTAGAAAAACGGACA	miRNA	miR-140
t0061818	24	1	AACCACAGGGTAGAACCACGGACG	miRNA	miR-140
t0061878	23	1	ACCACAGGGTAGAACCACGGAAC	miRNA	miR-140
t0061892	24	1	TACGACAGGGTAGAACCACGGAAA	miRNA	miR-140
t0061920	23	1	TACCACAGGGTAGAATCACGGCA	miRNA	miR-140
t0061941	23	1	ACCACAGGGTAGAACCAAGGAAT	miRNA	miR-140
t0061959	20	1	CCACAGGATAGAACCACGGA	miRNA	miR-140
t0061970	23	1	ACCACAGGGGAGAACCACGGATT	miRNA	miR-140
t0062007	24	1	ACAACAGGGTAGAACCACGGCAGA	miRNA	miR-140
t0062125	23	1	ACCACAGGGTAGAAACACGGACC	miRNA	miR-140
t0062151	23	1	TACCACAGGGGAGAATCACGGAC	miRNA	miR-140
t0062182	23	1	ACCACAGGGAAGAACCACGGATG	miRNA	miR-140
t0062257	23	1	ACCACAGGGTAGCACCACGGACA	miRNA	miR-140
t0062375	21	1	ACCACAGGGTAGAATCCCGGA	miRNA	miR-140
t0062438	23	1	TACCACAGGGTAGAACCATGGCC	miRNA	miR-140
t0062502	24	1	TAGCACAGGGTAGAACCACGGACG	miRNA	miR-140
t0062621	22	1	TACCACATGGTAGAACAACGGA	miRNA	miR-140
t0062644	22	1	ACCACAGGGTACAACCACGGCC	miRNA	miR-140
t0062676	23	1	CACCACAGGGTAGAACCACGGCC	miRNA	miR-140
t0062783	23	1	ACCACAGGGTAGAACCACGGGTT	miRNA	miR-140
t0062896	22	1	ATCACAGGGTAGAACCACGGAG	miRNA	miR-140
t0062930	24	1	TACCACAGGGTAAAACAACGGACA	miRNA	miR-140
t0063033	23	1	TACCAAAGGGTAGAACCAAGGAT	miRNA	miR-140
t0063128	23	1	ACCACAGGGTAGAACCACAGAAA	miRNA	miR-140
t0063286	24	1	TACTACAGGGTAGAACCACAGACA	miRNA	miR-140
t0063300	22	1	ACCACAGGGTAGAATTACGGAG	miRNA	miR-140
t0063528	23	1	TACTACAGGGTAGAACTACGGAA	miRNA	miR-140
t0063586	23	1	TACCACAGAGTAGAACCACGGAG	miRNA	miR-140

t0063618	24	1	TACCACAGGGTAGAACCACGCAGT	miRNA	miR-140
t0063677	23	1	TACCACAGGGAAGAACCACAGAA	miRNA	miR-140
t0063794	22	1	ACCACAGAGAAGAACCACGGAA	miRNA	miR-140
t0063836	22	1	TACCGCAGGGTAGAACCCCGGA	miRNA	miR-140
t0064195	22	1	TACCACAAGGTAGAACCACGGT	miRNA	miR-140
t0064288	22	1	ACCACAGGGTACAAACACGGAC	miRNA	miR-140
t0064453	22	1	ACCACAGGGTAGAAACACGGCA	miRNA	miR-140
t0064477	23	1	ACCACGGGGTAGAACCACGGAAG	miRNA	miR-140
t0064528	24	1	TACCACAGGGTAAAACCACGGACG	miRNA	miR-140
t0064711	24	1	TATCACAGGGTAGAACCACGGAAT	miRNA	miR-140
t0064766	23	1	TATCACAGGGTAGAACCCCGGAC	miRNA	miR-140
t0064784	25	1	TACCACAGGGTAGAACCACGGCCAA	miRNA	miR-140
t0064845	22	1	ACCACAGGGTAGAACCCCGGCC	miRNA	miR-140
t0064948	21	1	CCACAGGGTTGAACCACGGAT	miRNA	miR-140
t0065031	21	1	CCACAGGGTAGAACCAAGGAA	miRNA	miR-140
t0065058	24	1	TACCACAGGGTAGAACCACCGATA	miRNA	miR-140
t0065086	22	1	TACCACAGGGTAGAACCCCGGG	miRNA	miR-140
t0065156	24	1	TACCACGGGGTAGAACCACGGAAA	miRNA	miR-140
t0065340	23	1	TACCACAAGGTAGAACCACGGAG	miRNA	miR-140
t0065394	23	1	TACCACAAGGTAGAACCAAGGAA	miRNA	miR-140
t0065404	22	1	TGCCACAGCGTAGAACCACGGA	miRNA	miR-140
t0065498	23	1	TACCACAGGGTAGAACAAGGGAT	miRNA	miR-140
t0065526	21	1	ACCACAGGGTAGAAACAAGGA	miRNA	miR-140
t0065552	24	1	ACCACAGGGTAGAACCATGGAATC	miRNA	miR-140
t0065768	22	1	ACCACAGGGCAGAATCACGGAT	miRNA	miR-140
t0065775	23	1	TACCACAGGGTAGAACTACGGCG	miRNA	miR-140
t0065861	23	1	ACCACAGGGTAGAACCAGGGACG	miRNA	miR-140
t0065969	23	1	TACCACAGGGTACAACCACGGAG	miRNA	miR-140
t0066102	24	1	TACCACAGGGGAGAACCACGGAGA	miRNA	miR-140
t0066163	22	1	TGCCACAGGGTAGAACTACGGA	miRNA	miR-140
t0066301	24	1	TACCACAGGGTAGAACCACGGCTC	miRNA	miR-140
t0066360	23	1	ACCACAGGGGAGAACCACGGAAT	miRNA	miR-140
t0066537	24	1	TACGACAGGGTAGAACCACGGACG	miRNA	miR-140
t0066552	25	1	TACCACAGGGTAGAAACACGGACAA	miRNA	miR-140
t0066576	23	1	TACCACAGGGTATAAGCACGGAA	miRNA	miR-140
t0066609	20	1	CCACAGGGTAGAACCTCGGA	miRNA	miR-140
t0066679	21	1	TAGCACAGGGTAGAACCACGG	miRNA	miR-140
t0066702	21	1	ACCACAGGGTTGAACAACGGA	miRNA	miR-140
t0066773	22	1	ACCAGAGGGTAGAACCACGGAA	miRNA	miR-140
t0066801	23	1	TACAACAGGGTAGAACCAAGGAC	miRNA	miR-140
t0066817	22	1	TACCACAGGGGAGAACCACGGG	miRNA	miR-140
t0066898	22	1	TACCACAAGGTAGAACTACGGA	miRNA	miR-140
t0066901	24	1	TACCACAGGGGAGAACCAAGGACA	miRNA	miR-140
t0066910	21	1	ACCATAGGGTAGAACCACGGG	miRNA	miR-140
t0066965	23	1	TACCACAGGGTAGATCTACGGAA	miRNA	miR-140
t0067043	24	1	TACCACAGGGTAGAACCGCGGACG	miRNA	miR-140
t0067152	23	1	CTACCACAGGGTAGAACAACGGA	miRNA	miR-140
t0067299	21	1	ACCACAGGGTAGAACAAGGGA	miRNA	miR-140
t0067317	23	1	ACCTCAGGGTAGAACCACGGACA	miRNA	miR-140
t0067342	22	1	ACCACAGGGTTGAACCACGGAT	miRNA	miR-140
t0067449	23	1	TACCCAAGGGTAGAACCACGGAC	miRNA	miR-140
t0067495	23	1	ACCACAGGGTAGAACCGCGGACG	miRNA	miR-140
t0067542	20	1	CAACAGGGTAGAACAACGGA	miRNA	miR-140
t0067550	23	1	ACAACAGGGTAGAACCACGGATG	miRNA	miR-140
t0067569	23	1	TACCACAGGGTAGAACCCCGGGC	miRNA	miR-140
t0067615	24	1	TACCATAGGGTAGAACCACAGACA	miRNA	miR-140
t0067642	23	1	TACCACAGGGGAGAACCCCGGAA	miRNA	miR-140
t0067657	22	1	ACCACTGGGTAGAACCACGGAG	miRNA	miR-140

t0067884	23	1	TCCCACAGGGTAGAACCATGGAC	miRNA	miR-140
t0067942	21	1	ACCACAGGGAAGAACCATGGA	miRNA	miR-140
t0068029	24	1	TACCACGGGGTAGAACCCCGGACA	miRNA	miR-140
t0068112	22	1	TACAACAGGGAAGAACCACGGA	miRNA	miR-140
t0068304	22	1	ACCAAAGGGTAGAACAACGGAC	miRNA	miR-140
t0068312	25	1	ACCACAAGGTAGAACCACGGACAGA	miRNA	miR-140
t0068425	21	1	CCACAGGGTAGAACAACGGAC	miRNA	miR-140
t0068584	24	1	TACCACAGGGTGGAACCACGGAAA	miRNA	miR-140
t0068621	23	1	ACCACAGGGTAGAACCACGGCGA	miRNA	miR-140
t0068635	24	1	TACCACAGGGTAGAAACCCGGACA	miRNA	miR-140
t0068827	23	1	TACCACAGGGTAGAACCACTGAT	miRNA	miR-140
t0068898	23	1	ACCACAGGTTAGAACCACGGACA	miRNA	miR-140
t0069015	22	1	AACACAGGGTAGAACCACGGAG	miRNA	miR-140
t0069024	23	1	TACCACAGGGTAGAACCCCCGAA	miRNA	miR-140
t0069056	23	1	TACCACAGGGGAGAATCACGGAA	miRNA	miR-140
t0069066	20	1	TACCACAGGGTAGAACCCCG	miRNA	miR-140
t0069160	24	1	ATCACAGGGTAGAACCACGGATAA	miRNA	miR-140
t0069247	23	1	TACCACCGGGTAGAACCACGGCA	miRNA	miR-140
t0069433	24	1	TACCACAGGGTAGAAGCACGGACA	miRNA	miR-140
t0069434	21	1	CTACAGGGTAGAACCACGGAC	miRNA	miR-140
t0069479	21	1	ACCACAGGGGATAACCACGGA	miRNA	miR-140
t0069496	23	1	TACCACAGGGTAGAAAAACGGAC	miRNA	miR-140
t0069565	22	1	ACCACAGGGGAGAACAACGGAA	miRNA	miR-140
t0069601	24	1	TACTACAGGGTAGAACCACGGATA	miRNA	miR-140
t0069613	23	1	TACCACATGGTAGAACCACGGCT	miRNA	miR-140
t0069724	22	1	TACTACAGGGTAGAAACACGGA	miRNA	miR-140
t0069813	24	1	ACCACAGGGGAGAACCACGGAATC	miRNA	miR-140
t0070199	23	1	TACCACAGGGAAGAACAACGGAA	miRNA	miR-140
t0070234	24	1	TACCAAAGGGTAGAACCACAGACA	miRNA	miR-140
t0070367	26	1	ACCACAGGGTAGAACCACGGACGGGG	miRNA	miR-140
t0070389	23	1	TACCACAGGGTAGAACCCCCGAC	miRNA	miR-140
t0070407	23	1	TACCACAGGATAGAACCAAGGAC	miRNA	miR-140
t0070543	22	1	ACCACAGGGTAGAACAACGGAG	miRNA	miR-140
t0070594	21	1	ACCACAGGGGAGAACAACGGA	miRNA	miR-140
t0070692	24	1	TACCACAGGGTCGAACCACGGAAG	miRNA	miR-140
t0070765	22	1	ACCACAGGGTAGAATCAAGGAC	miRNA	miR-140
t0070785	23	1	ACCACAGGGAAGAACCACGGACG	miRNA	miR-140
t0070824	24	1	GACCACAGGGTAGAACCACGGACG	miRNA	miR-140
t0070874	22	1	TACCACAGGGTAGAACTACGGG	miRNA	miR-140
t0070940	22	1	TACCACAGGGTAGCACCACGGC	miRNA	miR-140
t0070947	24	1	ACCACAGGGTAAAACCACGGACAA	miRNA	miR-140
t0071006	24	1	TACCACAGGGGAGAACCACGGAGT	miRNA	miR-140
t0071085	21	1	TCACAGGGTAGAACCACGGAT	miRNA	miR-140
t0071114	21	1	TACTACAGGGTAGAACCACGG	miRNA	miR-140
t0071141	23	1	TACCACAGGGTAGAACCACGCAT	miRNA	miR-140
t0071164	20	1	TACCACAGGGTAGGACCACG	miRNA	miR-140
t0071187	24	1	TACCACAGGGTAGGACCACGGACG	miRNA	miR-140
t0071243	22	1	ACCACAGGGTAGAACTACGGTT	miRNA	miR-140
t0071266	22	1	TACCAGAGGGTATAACCACGGA	miRNA	miR-140
t0071268	23	1	TACAACAGGGTAGAACCATGGAC	miRNA	miR-140
t0071279	21	1	ACCACAGGGTAGAACCACAGC	miRNA	miR-140
t0071301	23	1	TACCAAAGGGTAGAACAACGGAC	miRNA	miR-140
t0071302	22	1	TAACAAAGGGTAGAACCACGGA	miRNA	miR-140
t0071361	22	1	CCACAGGGAAGAACCACGGACT	miRNA	miR-140
t0071414	22	1	ACCACAGGGTAGAACCACGTAA	miRNA	miR-140
t0071491	21	1	ACCACAAGGTAGAACTACGGA	miRNA	miR-140
t0071842	23	1	ACCACAGGGGAGAACCACGGATG	miRNA	miR-140
t0071848	23	1	ACCACAGGGTAGAACAACGGATA	miRNA	miR-140

t0071861	20	1	TCACAGGGGAGAACCACGGA	miRNA	miR-140
t0071928	24	1	TACCACAGGGTAGAACAACGGAAT	miRNA	miR-140
t0072004	22	1	ACCACAGGGTAGAACCACGCAT	miRNA	miR-140
t0072131	22	1	TACCACTGGGTAGAACTACGGA	miRNA	miR-140
t0072172	24	1	TACCATAGGGTAGAACCACGGAAT	miRNA	miR-140
t0072255	19	1	CACAGAGTAGAACCACGGA	miRNA	miR-140
t0072268	22	1	ACCACAGGGTAGAGCTACGGAT	miRNA	miR-140
t0072412	21	1	TTCACAGGGTAGAACCACGGA	miRNA	miR-140
t0072509	21	1	ACCACAGAGTAGAACAACGGA	miRNA	miR-140
t0072570	20	1	ACCACAGGTTAGAACCACGG	miRNA	miR-140
t0072730	23	1	TACCAGAGGGTAGAACCACGGAT	miRNA	miR-140
t0072741	23	1	AACACAGGGTAGAAACACGGACA	miRNA	miR-140
t0072762	21	1	ACCACAGGGTAGATCAACGGA	miRNA	miR-140
t0072826	23	1	ACCACAGGGAAGAACCACGGATT	miRNA	miR-140
t0072878	21	1	ACCACAGTGTAGAGCCACGGA	miRNA	miR-140
t0073038	21	1	ACCACGGGGTAGAACTACGGA	miRNA	miR-140
t0073208	23	1	TACCGCAGGGTAGAAACACGGAC	miRNA	miR-140
t0073236	22	1	AGAACAGGGTAGAACCACGGAT	miRNA	miR-140
t0073335	22	1	ACCACAGGGGAGAACTACGGAA	miRNA	miR-140
t0073439	23	1	TACCACAGGGTAGAACCAGGAAA	miRNA	miR-140
t0073495	23	1	CACCACAGGGTAGAATCACGGAC	miRNA	miR-140
t0073872	23	1	TACCACAGAGTAGAACCTCGGAT	miRNA	miR-140
t0073925	23	1	ACCAGAGGGTAGAACCACGGACT	miRNA	miR-140
t0073951	23	1	TACCATAGGGTAGAACAACGGAA	miRNA	miR-140
t0073956	21	1	ATCACATGGTAGAACCACGGA	miRNA	miR-140
t0073967	21	1	ACAAAAGGGTAGAACCACGGA	miRNA	miR-140
t0074008	22	1	TACCACACGGTACAACCACGGA	miRNA	miR-140
t0074016	22	1	ACCACAGGGTGGAACCACGGAG	miRNA	miR-140
t0074063	22	1	TACCACAAGGTAGAAACACGGA	miRNA	miR-140
t0074159	23	1	ACCACAAGGTAGAACCACGGACT	miRNA	miR-140
t0074171	24	1	TACCACAGGGCAGAACCACGGATC	miRNA	miR-140
t0074526	23	1	ACCACAGGGTAGAACCTCGGACA	miRNA	miR-140
t0074568	23	1	TACCACAGGGGAGAACAACGGAC	miRNA	miR-140
t0074656	23	1	TAACACAGGGTAGAACCAAGGAC	miRNA	miR-140
t0074662	24	1	TACCACAGGGTAGAACCGCGGACT	miRNA	miR-140
t0074788	24	1	TACCACAGGGTAGAGCCACGGAAA	miRNA	miR-140
t0074792	20	1	CCACAGGGTAGAACTACGGC	miRNA	miR-140
t0074839	24	1	TAACACAGGGTAGAACCACGGAAA	miRNA	miR-140
t0074944	22	1	ACCACAGGGTAGAACTAAGGAC	miRNA	miR-140
t0075085	22	1	ACCAAAGGGTAGAATCACGGAC	miRNA	miR-140
t0075131	24	1	TACCAGAGGGTAGAACCACGGATA	miRNA	miR-140
t0075281	23	1	TACCAGAGGGTAGAACCCCGGAC	miRNA	miR-140
t0075302	26	1	ACCACAGGGTAGAACCACGGAAAAGA	miRNA	miR-140
t0075450	22	1	ACCACAGGGTAGAACAAAGGAT	miRNA	miR-140
t0075487	20	1	CCACAGGGGAGAACCACGGT	miRNA	miR-140
t0075509	21	1	CCCCCAGGGTAGAACCACGGA	miRNA	miR-140
t0075522	24	1	CACCACAGGGTAGAACCACGGAAG	miRNA	miR-140
t0075525	24	1	GCCACAGGGTAGAACCACGGACAA	miRNA	miR-140
t0075585	22	1	TACCACAGCGTAGAACCAAGGA	miRNA	miR-140
t0075632	22	1	TACCACAGGGTAGAATCAAGGA	miRNA	miR-140
t0075633	22	1	TACCACAAGGTAGAACCCCGGA	miRNA	miR-140
t0075762	23	1	ACCAGAGGGTAGAACCACGGACA	miRNA	miR-140
t0075772	22	1	ACCACAGGGTAGCACCACGGAT	miRNA	miR-140
t0075878	23	1	ACCACAGGGGAGAACCACGGAAA	miRNA	miR-140
t0075879	21	1	ACCACAGGGTAGCAACACGGA	miRNA	miR-140
t0075944	23	1	TACCACAGGGTCGAACAACGGAC	miRNA	miR-140
t0075998	25	1	ACCACAGGGTAGAAACACGGACATC	miRNA	miR-140
t0076007	22	1	TACCACAGGGTAGAACCACACA	miRNA	miR-140

t0076098	23	1	ACCACAGGGAAGAACTACGGACA	miRNA	miR-140
t0076354	22	1	TGCCACAGTGTAGAACCACGGA	miRNA	miR-140
t0076494	22	1	TACCACAGTGTAGAACTACGGA	miRNA	miR-140
t0076600	22	1	TATCACAGGGTAGAAACACGGA	miRNA	miR-140
t0076608	21	1	ACCACAGGGTAGAACAACGGG	miRNA	miR-140
t0076681	22	1	ACCACAGGGTAGAAACACGGGC	miRNA	miR-140
t0076739	22	1	TACCACGGGGGAGAACCACGGA	miRNA	miR-140
t0076914	24	1	TACCACAGGCTAGAACCACGGACA	miRNA	miR-140
t0076943	23	1	ACCACAGGGGAGAACCACGGACT	miRNA	miR-140
t0077085	22	1	ACAAAAGGGTAGAACCACGGAT	miRNA	miR-140
t0077143	23	1	TACCACAGGGTAGAAACAGGGAC	miRNA	miR-140
t0077168	23	1	ACTACAGGGTAGAACCACGGACT	miRNA	miR-140
t0077246	24	1	TACCACAGGGTAGTACCACGGAAA	miRNA	miR-140
t0077262	22	1	ACCAAAGGGTAGAACAACGGAA	miRNA	miR-140
t0077310	21	1	TACCACAAGGTAGAACCACGG	miRNA	miR-140
t0077418	20	1	AACACAGGGTAGAACCACGG	miRNA	miR-140
t0077649	23	1	TACCACAAGGTAGAAACACGGAC	miRNA	miR-140
t0077671	22	1	ACCACAGGGTCGAACCACGGAT	miRNA	miR-140
t0077715	20	1	CCGCAGGGTAGAACCACGGA	miRNA	miR-140
t0077894	22	1	CACCACAGGGTAGAACCACGGT	miRNA	miR-140
t0078029	21	1	CCACAGGGTAGGACCACGGAC	miRNA	miR-140
t0078088	23	1	ACCACAGGGTAGAACCAGGGACA	miRNA	miR-140
t0078164	22	1	ACCACAGGTTAGAACCACGGAT	miRNA	miR-140
t0078171	23	1	TACCACACGGTAGAACCACGGAG	miRNA	miR-140
t0078177	21	1	ATCACAGGGTAGAACCAAGGA	miRNA	miR-140
t0078355	23	1	TACCACATGGTAGAACCCCGGAC	miRNA	miR-140
t0078607	23	1	TACCGCAGGGTAGAACCCCGGAC	miRNA	miR-140
t0078642	23	1	ACCACAGGGTAGAACCGCGGATT	miRNA	miR-140
t0078767	23	1	TACCACAGGGTAGATCCCCGGAA	miRNA	miR-140
t0078874	23	1	ACCACAGGGTAGAATCACGGCCA	miRNA	miR-140
t0078991	22	1	TACCACCGGGTAGAAACACGGA	miRNA	miR-140
t0079083	23	1	ACCACAGGGTCGAACCACGGACG	miRNA	miR-140
t0079111	20	1	ACCAAAGGGTAGAACCACGG	miRNA	miR-140
t0079168	22	1	TAACACAGGGAAGAACCACGGA	miRNA	miR-140
t0079208	23	1	TACCACAGGGAAGAACCAAGGAC	miRNA	miR-140
t0079209	22	1	CCACAGGGTAGAACCACAGACA	miRNA	miR-140
t0079240	21	1	ACCACATGGTAGAACCACGGC	miRNA	miR-140
t0079275	23	1	TACCACAGGGGAGAACCACTGAA	miRNA	miR-140
t0079339	24	1	AACAACAGGGTAGAACCACGGACG	miRNA	miR-140
t0079439	24	1	TACAACAGGGTAGAACCAAGGAAG	miRNA	miR-140
t0079471	22	1	ACCACAGGGTAGAACTACGGCC	miRNA	miR-140
t0079688	22	1	TACCACAGCGTAGAACGACGGA	miRNA	miR-140
t0079697	23	1	TACCACAAGGGAGAACCACGGAC	miRNA	miR-140
t0079790	22	1	TACCACAGTGTAGAACCAAGGA	miRNA	miR-140
t0079794	22	1	TACCACAGGGTTGAACCACGGC	miRNA	miR-140
t0079838	22	1	TACCGCAGGGTAGAACCACGGT	miRNA	miR-140
t0079868	21	1	CCAAAGGGTAGAACCACGGAT	miRNA	miR-140
t0079877	20	1	ACCACAGGGTAAAACCACGG	miRNA	miR-140
t0080074	23	1	TACCACAGGGGATAACCACGGAA	miRNA	miR-140
t0080176	21	1	ACCACAGTTTAGAACCACGGA	miRNA	miR-140
t0080214	22	1	ACCACAGGGTAGGATCACGGAA	miRNA	miR-140
t0080276	23	1	ACCACAGGGTAAAACCACGGACG	miRNA	miR-140
t0080279	23	1	ACAACAGGGGAGAACCACGGAAG	miRNA	miR-140
t0080313	22	1	TACCACAGGGTAGAACCCCAGA	miRNA	miR-140
t0080400	23	1	TACCACGGGGTAGAACAACGGAC	miRNA	miR-140
t0080465	20	1	TCACAGGGTAGAACCAAGGA	miRNA	miR-140
t0080546	23	1	TAACACAGGGTAGAACCAAGGAA	miRNA	miR-140
t0080564	20	1	ACCACAGGGTAGAAAAACGG	miRNA	miR-140

t0080802	22	1	TACCACAGGGTAGAATCACGGG	miRNA	miR-140
t0081039	21	1	ACCACAGGGGAGAAACACGGA	miRNA	miR-140
t0081055	24	1	TACCACGGGGTAGAACCACGGATA	miRNA	miR-140
t0081202	22	1	ATCACAGGGTAGAACCACAGAA	miRNA	miR-140
t0081291	20	1	ACTACAGGGTAGAACCACGG	miRNA	miR-140
t0081316	22	1	ACTACAGGGTAGAACTACGGAT	miRNA	miR-140
t0081451	22	1	TACCACGGGGTAGAACCCCGGA	miRNA	miR-140
t0081526	22	1	ACCACAGGGTAGAACAACGGCT	miRNA	miR-140
t0081585	22	1	ACCACAGGGTAGAACTATGGAA	miRNA	miR-140
t0081591	21	1	ACCAAAGGGTAGAACTACGGA	miRNA	miR-140
t0081604	21	1	ACCACAGGGCAGATCCACGGA	miRNA	miR-140
t0081648	22	1	TACCACAGGGTAGAACAAGGGA	miRNA	miR-140
t0081820	22	1	ACCACAGGGTATAACCACGGCC	miRNA	miR-140
t0081832	24	1	TACCACAGGGGAGAAACACGGAGA	miRNA	miR-140
t0081940	23	1	TATCACAGGGTAGAACCACGGCA	miRNA	miR-140
t0081971	24	1	TACCGCAGGGTAGAATCACGGACA	miRNA	miR-140
t0082065	22	1	TACCACAGGGTAGAACAACGGC	miRNA	miR-140
t0082195	24	1	CACCACAGGGTACAACCACGGACG	miRNA	miR-140
t0082225	23	1	TACCACAGGGTAGAACTAAGGAA	miRNA	miR-140
t0082367	21	1	ACCACAAGGTAGAATCACGGA	miRNA	miR-140
t0082439	23	1	TACCACATGGCAGAACCACGGAC	miRNA	miR-140
t0082483	20	1	ACCACAGGGTAGAACCACGC	miRNA	miR-140
t0082527	24	1	TACCACAGAGTAGAACCACGGTCA	miRNA	miR-140
t0082556	25	1	TACCACAGGGTAGAACCACGGGAAA	miRNA	miR-140
t0082563	22	1	GCAACAGGGTAGAACCACGGAT	miRNA	miR-140
t0082642	23	1	TACCACAGGATAGAACCCCGGAA	miRNA	miR-140
t0082654	22	1	TACCACAGGGTAGAATCACTGA	miRNA	miR-140
t0082820	22	1	TATCACAGGGGAGAACCACGGA	miRNA	miR-140
t0082960	24	1	TACCACAGGGTAGAACCCCGGACC	miRNA	miR-140
t0083037	22	1	ACCACAGGGTAGAACTACGGCA	miRNA	miR-140
t0083084	22	1	ACCACATGGTAGAACCACGGAC	miRNA	miR-140
t0083089	23	1	ACCACAGGGTAGAACCTCGGAAA	miRNA	miR-140
t0083156	23	1	TACCACAGAGTATAACCACGGAA	miRNA	miR-140
t0083193	22	1	TACCACAGGGTAAAACTACGGA	miRNA	miR-140
t0083322	22	1	TACCACAGGGTAGAACCCTGGA	miRNA	miR-140
t0083432	21	1	ACCACAGTGTAGAACCACGGT	miRNA	miR-140
t0083506	22	1	ACCACAGGGTAGAAAAACGGAA	miRNA	miR-140
t0083519	23	1	TACCACAGGGTAGAACCGTGGAA	miRNA	miR-140
t0083591	23	1	ACCACAGGGTAGAACCACGGCAT	miRNA	miR-140
t0083605	23	1	TATCACAGGGTAGAACCACGGAG	miRNA	miR-140
t0083856	26	1	ACCACAGGGTAGAACCACGGCCGGAT	miRNA	miR-140
t0083918	21	1	ACCACGGGGTAGAACCACTGA	miRNA	miR-140
t0083938	22	1	AACACAGGGTAGAACCAGGGAT	miRNA	miR-140
t0083939	24	1	TACCATAGGGTAGAACAACGGACA	miRNA	miR-140
t0084053	20	1	CCCACAGGGTAGAACCACGG	miRNA	miR-140
t0084083	27	1	ACCACAGGGTAGAACCCCGGACACCGG	miRNA	miR-140
t0084087	24	1	TACCACAGGGTAGAATCACGGCCA	miRNA	miR-140
t0084304	22	1	ACCACCGGGTAGAACCACGGAG	miRNA	miR-140
t0084372	24	1	TACCCCAGGGTAGAACCACGGAAA	miRNA	miR-140
t0084413	23	1	ACCACAGGGTAGATCCACGGACA	miRNA	miR-140
t0084427	21	1	ACAACAGGGGAGAACCACGGA	miRNA	miR-140
t0084569	22	1	ACCACAGGGTAGAGCTACGGAA	miRNA	miR-140
t0084583	23	1	GACCACAGGGTAGAACCACGGAT	miRNA	miR-140
t0084658	24	1	TACCACAGGGCAGAACTACGGACA	miRNA	miR-140
t0084864	23	1	TCCCACAGGGTAGAACCACGGAC	miRNA	miR-140
t0085073	21	1	ACCATAGGGTAGAACCACGGC	miRNA	miR-140
t0085136	22	1	TACCACAGGGTAGACCCACGGC	miRNA	miR-140
t0085146	20	1	CCCCAGGGTAGAACCACGGA	miRNA	miR-140

t0085331	24	1	TACCAGAGGGTAGTACCACGGACA	miRNA	miR-140
t0085373	23	1	TACCACGGGGTAGAACCACGGAG	miRNA	miR-140
t0085384	22	1	ACCACAGGGTAGAAAAACGGAT	miRNA	miR-140
t0085419	24	1	TACCACAGTGTAGAACCACGGAAA	miRNA	miR-140
t0085550	22	1	AGCACAGGGTAGAACCACGGAT	miRNA	miR-140
t0085585	24	1	TACAACAGGGTAGAACCACGGAAA	miRNA	miR-140
t0085725	24	1	TACCACAGGGTAGAACCACAGAAT	miRNA	miR-140
t0085785	22	1	CCACAGGGTAGAACCACGGCCT	miRNA	miR-140
t0085892	24	1	TACCCCAGGGTAGAACCACGGAAT	miRNA	miR-140
t0085895	24	1	TACCACAGGGTAGAACCCCGGAGT	miRNA	miR-140
t0085904	20	1	ACACAGGGTAGAACCCCGGA	miRNA	miR-140
t0085914	22	1	TACCACAGGGTAGAACCCCGCA	miRNA	miR-140
t0085915	23	1	TACCACAGGGTAGAAAGACGGAC	miRNA	miR-140
t0085958	20	1	CCACAGGGTGGAACCACGGA	miRNA	miR-140
t0086003	22	1	TACCACAGGGTAGGATCACGGA	miRNA	miR-140
t0086026	21	1	TACCACAGGGTAGAACCACGC	miRNA	miR-140
t0086044	24	1	TACCACAGAGTAGAACCACGGAAA	miRNA	miR-140
t0086182	21	1	ATCACAGGGTAGAACCACGGG	miRNA	miR-140
t0086190	21	1	ACCTCAGGGGAGAACCACGGA	miRNA	miR-140
t0086213	24	1	TACCATAGGGTAGAACCACGGACT	miRNA	miR-140
t0086251	24	1	TACCATAGGGTAGAACCACGGATA	miRNA	miR-140
t0086312	20	1	CCACAGGGTAGAACCGCGGA	miRNA	miR-140
t0086327	23	1	TACCACAGGGTAGAACTAAGGAC	miRNA	miR-140
t0086487	21	1	ACCACAGGGTAGAACCGCGGC	miRNA	miR-140
t0086584	24	1	TACCACAGGGTAGAACCACGGGCT	miRNA	miR-140
t0086695	21	1	CCACAGGGTAAAACCACGGAT	miRNA	miR-140
t0086704	23	1	TACCACAGGCTAGAACCAAGGAA	miRNA	miR-140
t0086938	23	1	ACCACAGGGTAGACCCACGGACA	miRNA	miR-140
t0087036	23	1	ACCACAGGGGAGAACCGCGGACA	miRNA	miR-140
t0087105	23	1	TACCACAGCGTAGAACCACGGAT	miRNA	miR-140
t0087119	22	1	ACCACAGGGTAGAACGACGGAC	miRNA	miR-140
t0087125	21	1	ACCACAGGGTAAAACTACGGA	miRNA	miR-140
t0087203	21	1	ACCACAGGGTAGACCCCCGGA	miRNA	miR-140
t0087241	22	1	TACCACAGGCTAGAACCACGGG	miRNA	miR-140
t0087255	22	1	TACCACTGGGTAGAACAACGGA	miRNA	miR-140
t0087316	23	1	ACCGCAGGGTAGAACCACGGACA	miRNA	miR-140
t0087431	21	1	ACCACAGGGTATAACCACCGA	miRNA	miR-140
t0087504	23	1	TACCACAGGGTAGAACCACGGGG	miRNA	miR-140
t0087604	22	1	TACCACAGGGAAGAAACACGGA	miRNA	miR-140
t0087633	24	1	AACCAGAGGGTAGAACCACGGACA	miRNA	miR-140
t0087780	22	1	ACTACAGGGTATAACCACGGAC	miRNA	miR-140
t0087855	22	1	TACCACAGTGTAGAACAACGGA	miRNA	miR-140
t0087899	22	1	ACCACAGGGTAGAAATACGGAT	miRNA	miR-140
t0087924	22	1	TACCATAGCGTAGAACCACGGA	miRNA	miR-140
t0087988	22	1	TACCACAGGGTAGACCCCCGGA	miRNA	miR-140
t0088082	24	1	TACCACAGGATAGAACCACGGAAA	miRNA	miR-140
t0088284	22	1	TACCACAGAGTAGAACCACGGC	miRNA	miR-140
t0088318	22	1	ACCACAGGGTAGAAACACGGCC	miRNA	miR-140
t0088367	24	1	TACCACAGGGTAGAACCACGGTCG	miRNA	miR-140
t0088371	21	1	ACAACAGTGTAGAACCACGGA	miRNA	miR-140
t0088441	23	1	TACCACCGGGTAGAACCCCGGAA	miRNA	miR-140
t0088494	23	1	CCCCACAGGGTAGAACCACGGAC	miRNA	miR-140
t0088495	24	1	TACCACAGGGTAGAACCAGGGAAT	miRNA	miR-140
t0088520	24	1	TACCACCGGGTAGAACCCCGGACA	miRNA	miR-140
t0088572	23	1	TACCACAGGATAGAACTACGGAA	miRNA	miR-140
t0088608	21	1	ACCACAGGGTTAAACCACGGA	miRNA	miR-140
t0088619	23	1	ACCATAGGGTAGAACCACGGACT	miRNA	miR-140
t0088687	24	1	ACCACAGGGTAGAACAACGGATGA	miRNA	miR-140

t0088702	23	1	TACCACAGGGTGGAACCACGGAG	miRNA	miR-140
t0088937	23	1	AGCACAGGGTAGAACCACGGACA	miRNA	miR-140
t0088950	21	1	ACCACAGGGTGGAACTACGGA	miRNA	miR-140
t0088978	24	1	TACCACAGGGTAGAATCACGAACG	miRNA	miR-140
t0089004	23	1	ACCACAAGGTAGAACCACGGCAG	miRNA	miR-140
t0089150	21	1	ACCACGGCGTAGAACCACGGA	miRNA	miR-140
t0089151	23	1	TACCACAGGGTCGAACCACGGAG	miRNA	miR-140
t0089160	24	1	TTCCACAGGGTAGAACCACGGACA	miRNA	miR-140
t0089188	23	1	TACCACAGGGTAGAACTACGGTT	miRNA	miR-140
t0089244	23	1	TACCACAGGGTAGTACCCCGGAT	miRNA	miR-140
t0089249	23	1	ACCACAGGGTGGAACCACGGAGA	miRNA	miR-140
t0089286	22	1	ACCACAGGGATGAACCACGGAA	miRNA	miR-140
t0089361	23	1	TACGACAGGGTAGAACCCCGGAC	miRNA	miR-140
t0089522	23	1	TACCAAAGGGTAGAACCAAGGAC	miRNA	miR-140
t0089582	24	1	CACCACAGGGTAGAACCACGGAAA	miRNA	miR-140
t0089622	24	1	ACCACAGGGTAGAACCAAGGAAGA	miRNA	miR-140
t0089662	21	1	ACCACAGGCTAGAATCACGGA	miRNA	miR-140
t0089679	23	1	TACCACAGGGCAGAACCAAGGAA	miRNA	miR-140
t0090076	23	1	ACCACGGGGTAGAACCACGCACA	miRNA	miR-140
t0090127	20	1	CCACAGCGTAGAACCACGGA	miRNA	miR-140
t0090330	21	1	ACCACAGGGTATACCCACGGA	miRNA	miR-140
t0090415	23	1	TACCACAGGGTAGAACTCCGGAC	miRNA	miR-140
t0090528	23	1	TACCACAGGGTAGAAGCCCGGAC	miRNA	miR-140
t0090552	22	1	ACCACAGGGTTGAACCGCGGAA	miRNA	miR-140
t0090617	23	1	TACCACAGGCTAGAACCACGGGC	miRNA	miR-140
t0090885	22	1	CCACAGGGTAGAAACACGGACA	miRNA	miR-140
t0090934	24	1	ACCACAGGGCAGAACCACGGAAGA	miRNA	miR-140
t0090975	23	1	TAACACAGGGTAGAACCACGGAG	miRNA	miR-140
t0091014	23	1	TACCACAAGGTAGAACCACGGCT	miRNA	miR-140
t0091084	23	1	TACCACAAGGTAGAACCCCGGAC	miRNA	miR-140
t0091426	22	1	ACCACAGGGCAGAACCACAGAC	miRNA	miR-140
t0091460	22	1	CCACAGGGTAGAATCACGGAAA	miRNA	miR-140
t0091467	21	1	ACAACAAGGTAGAACCACGGA	miRNA	miR-140
t0091637	21	1	ACCACAGGGTAGCACCACGGC	miRNA	miR-140
t0091686	22	1	ACCATAGGGTAGAATCACGGAC	miRNA	miR-140
t0091729	23	1	TACCACAGGGTAGTACCACGGAC	miRNA	miR-140
t0091761	23	1	TACCAAAGGGTAGAACCACGGAT	miRNA	miR-140
t0092103	21	1	TACCACAGGGTAGAACTACGA	miRNA	miR-140
t0092219	24	1	TACCACAGGGTAGGACCACGGACT	miRNA	miR-140
t0092257	21	1	ACCACAGGGTAGAATCACAGA	miRNA	miR-140
t0092304	24	1	TACCACAGAGTAGAACCACGGACG	miRNA	miR-140
t0092340	20	1	ACCACAGGGTAGAGCCACGG	miRNA	miR-140
t0092500	22	1	ACCACTGGGTAGAACCACGGAA	miRNA	miR-140
t0092537	22	1	ACCACAGGGTAGAACCATGGAG	miRNA	miR-140
t0092542	21	1	ACCACAGTGTAGAACCAAGGA	miRNA	miR-140
t0092553	23	1	ACCACTGGGTAGAACCACGGACA	miRNA	miR-140
t0092578	22	1	TACCACAGTGTAGAACCACGGT	miRNA	miR-140
t0092597	23	1	ACCACAGGGTTGAACCACGGACA	miRNA	miR-140
t0092614	23	1	ACCACAGGGTAGAACCACGAACA	miRNA	miR-140
t0092639	22	1	TACCACAGGGTAGAATAACGGA	miRNA	miR-140
t0092650	21	1	ACCAAAAGGTAGAACCACGGA	miRNA	miR-140
t0092693	22	1	TACCACACGGGAGAACCACGGA	miRNA	miR-140
t0092809	23	1	TACCACAGGGTAGAACCTCGGCT	miRNA	miR-140
t0092835	20	1	ACCACAGAGTAGAACCACGG	miRNA	miR-140
t0092871	24	1	CACCACAGGGTAGAACCCCGGACA	miRNA	miR-140
t0092914	26	1	TGGAATGTAAAGAAGTATGGACCATC	miRNA	miR-1-3p
t0092918	22	1	TGGAATGAAAAGAAGTATGGAG	miRNA	miR-1-3p
t0092937	25	1	TGGAATGTAAAGAAGTATGGAGCTC	miRNA	miR-1-3p

t0092958	25	1	TGGAATGTAAAGAAGTATGGTGATC	miRNA	miR-1-3p
t0093040	22	1	TGGAATGGAAAGAAGTATGGAG	miRNA	miR-1-3p
t0093067	21	1	TGGAGTGTAAAGAAGTATGGA	miRNA	miR-1-3p
t0093088	21	1	TGGAATGTAGAGAAGTATGGA	miRNA	miR-1-3p
t0093167	22	1	TGGAATGTAAAGAAGGATGGAG	miRNA	miR-1-3p
t0093231	25	1	TGGAATGTAAAGAAGTATGGACATC	miRNA	miR-1-3p
t0093319	24	1	TGGAATGTAAAGAAGTATGTAATA	miRNA	miR-1-3p
t0093337	22	1	TGGAACGTAAAGAAGTATGGAG	miRNA	miR-1-3p
t0093461	25	1	TGGAGACGCGGCCCTGTTGGGGATC	miRNA	miR-139*
t0093498	20	1	TCTACAGAGCTATGTGACAT	miRNA	miR-139
t0040656	21	1	CAGTGCAATGTTAAAAAGGCA	miRNA	miR-130d
t0040660	22	1	CAGTGCAATGTTAAAAAGGCAT	miRNA	miR-130d
t0043346	21	1	GAGTGCAATGATGAAAGGGCA	miRNA	miR-130b
t0049018	21	1	CGGTGCAATGATGAAAGGGCA	miRNA	miR-130b
t0054861	22	1	CAGTGCAATGATGTAAGGGCAT	miRNA	miR-130b
t0054954	21	1	CAGTGCAATGATGAAAGGGAA	miRNA	miR-130b
t0061505	22	1	CAGTGCAATGATGAAAGGGCTT	miRNA	miR-130b
t0064060	21	1	CAGTGCAATGATGAGAGGGCA	miRNA	miR-130b
t0073129	22	1	CAGTGCAATGATGCAAGGGCAT	miRNA	miR-130b
t0074884	22	1	CAGTGCAATAATGAAAGGGCAT	miRNA	miR-130b
t0077739	21	1	CAGTGCAATGGTGAAAGGGCA	miRNA	miR-130b
t0088683	23	1	CAGTGCAATGATGAAAGGGAATA	miRNA	miR-130b
t0061151	23	1	CAGTGCAATGATGAAAGGACATT	miRNA	miR-130b
t0055306	21	1	CAGGGCAATGATGAAAGGGCA	miRNA	miR-130b
t0090348	22	1	CAGTACAATGATGAAAGGGCAT	miRNA	miR-130b
t0042347	22	1	CATTGCAATGATGAAAGGGCAT	miRNA	miR-130b
t0043526	22	1	CAGGGCAATGATGAAAGGGCAT	miRNA	miR-130b
t0044230	21	1	CAGTGAAATGTTAAAAGGGCA	miRNA	miR-130a
t0048468	21	1	CAGTGTAATGTTAAAAGGGAA	miRNA	miR-130a
t0052304	23	1	CAGTGCAATGTTAAAAGGGCCTA	miRNA	miR-130a
t0053236	23	1	CAGTGCCATGTTAAAAGGGCCTT	miRNA	miR-130a
t0060318	22	1	CAGTGAAATGTTAAAAGGGCAT	miRNA	miR-130a
t0063037	21	1	CAGTGCAATGTCAAAAGGGCA	miRNA	miR-130a
t0063968	22	1	CAGTGCAATGTTAACAGGGCAA	miRNA	miR-130a
t0066446	22	1	CAGTGCAATGATAAAAGGGCAT	miRNA	miR-130a
t0076076	22	1	CAGTGCAATGTTAAAATGGCAT	miRNA	miR-130a
t0077347	21	1	CAGTGCAATGCTAAAAGGGCA	miRNA	miR-130a
t0085831	21	1	CAGTGCAATGTTGAAAGGGCA	miRNA	miR-130a
t0086646	22	1	CAGTGCAATGTTAAAAGGCCAT	miRNA	miR-130a
t0089534	22	1	CAGAGCAATGTTAAAAGGGCAT	miRNA	miR-130a
t0040870	22	1	CAGTACAATGTTAAAAGGGCAT	miRNA	miR-130a
t0042737	22	1	CAGTGCCATGTTAAAAGGGCAT	miRNA	miR-130a
t0044932	22	1	CAGTGCAATGCTAAAAGGGCAT	miRNA	miR-130a
t0045667	23	1	GCAGTGCAATGTTAAAAGGGCCT	miRNA	miR-130a
t0046856	22	1	CAGTGCAACGTTAAAAGGGCAT	miRNA	miR-130a
t0049180	22	1	CAGTGCAATGTTAAAAGGACAT	miRNA	miR-130a
t0050207	22	1	CAGTGCAATGTTGAAAGGGCAT	miRNA	miR-130a
t0050793	21	1	CAGTGCAATGATAAAAGGGCA	miRNA	miR-130a
t0051534	21	1	CAGGGCAATGTTAAAAGGGCA	miRNA	miR-130a
t0051940	21	1	CAGTGCAATGTTAAAAGGGTA	miRNA	miR-130a
t0060105	22	1	CAGTGCAGTGTTAAAAGGGCAT	miRNA	miR-130a
t0060540	20	1	CAGTGCAATGTTAAAAGGGT	miRNA	miR-130a
t0062161	22	1	TAGTGCAATGTTAAAAGGGCAT	miRNA	miR-130a
t0062525	21	1	AAGTGCAATGTTAAAAGGGCA	miRNA	miR-130a
t0064949	22	1	CAGTCCAATGTTAAAAGGGCAT	miRNA	miR-130a
t0072076	22	1	CAGTGTAATGTTAAAAGGGCAT	miRNA	miR-130a
t0076250	22	1	CCGTGCAATGTTAAAAGGGCAT	miRNA	miR-130a
t0077222	20	1	ACTCGGCGTGGCGTCGGTCT	miRNA	miR-1307

t0077355	21	1	CTCGTCGTGGCGTCGGTCGTG	miRNA	miR-1307
t0077533	20	1	ACTCGGAGTGGCGTCGGTCG	miRNA	miR-1307
t0081188	19	1	ACTCGGCGTGGCGTGGGTC	miRNA	miR-1307
t0081255	19	1	ACTCGGCGGGGCGTCGGTC	miRNA	miR-1307
t0082724	22	1	ACTCGGCGTGGCGTCGGTTGTT	miRNA	miR-1307
t0084098	21	1	CTCGGCGTGGAGTCGGTCGTG	miRNA	miR-1307
t0086689	21	1	CTCGGCGTGGCGTAGGTCGTG	miRNA	miR-1307
t0086789	20	1	ACTCGGCGTGGCGCCGGTCG	miRNA	miR-1307
t0089164	19	1	ACTCGGCGTGGCGTCGGGC	miRNA	miR-1307
t0090339	22	1	ACTCGACGTGGCGTCGGTCGTG	miRNA	miR-1307
t0090494	20	1	ACTCGGCGTGGCGCCGGGCG	miRNA	miR-1307
t0091785	22	1	ACTCGGCGTGGCGTCGGGCGTG	miRNA	miR-1307
t0041203	21	1	TTCGGCGTGGCGTCGGTCGTG	miRNA	miR-1307
t0045629	22	1	ACTCGGCGTGGCGTCGGTCGAG	miRNA	miR-1307
t0045731	22	1	ACTCGGCGTGGCGTCGGTCGGG	miRNA	miR-1307
t0048950	19	1	CTCGGCGTGGTGTCGGTCG	miRNA	miR-1307
t0051487	20	1	ACTCGGCGTGGCGTCGGTCA	miRNA	miR-1307
t0052501	22	1	ACTCGGCGTGGCATCGGTCGTG	miRNA	miR-1307
t0053492	20	1	CTCGGCGTGGCGTCGGGCGT	miRNA	miR-1307
t0053542	20	1	ACTCGGCGTGGCGTCAGTCG	miRNA	miR-1307
t0053634	21	1	ACTCGGCGTGGCGTCGGGCGT	miRNA	miR-1307
t0053708	20	1	ACTCGGCGTGGCATCGGTCG	miRNA	miR-1307
t0055019	20	1	ATTCGGCGTGGCGTCGGGCG	miRNA	miR-1307
t0057046	21	1	CTCGACGTGGCGTCGGTCGTG	miRNA	miR-1307
t0059144	22	1	CTCGGCGTGGCGTCGGTAGTGG	miRNA	miR-1307
t0060215	21	1	ACTCGGCGTGGCGTCGGTAGT	miRNA	miR-1307
t0062892	19	1	ACTCAGCGTGGCGTCGGTC	miRNA	miR-1307
t0066115	20	1	GCTCGGCGTGGCGTCGGTCG	miRNA	miR-1307
t0067971	22	1	ACTCGGCGTGGGGTCGGTCGTT	miRNA	miR-1307
t0067986	19	1	ACTCGGCGTGGCGTCGGTA	miRNA	miR-1307
t0069550	21	1	TTTGAGGCTACCGTGAGATGT	miRNA	miR-1304
t0070154	21	1	TTCGAGGCTACAGTGAGATTT	miRNA	miR-1304
t0072610	21	1	TTTGAGGCTACAGTGAGATGG	miRNA	miR-1304
t0073201	21	1	TTTGAGGCTACAGTGAGGTGT	miRNA	miR-1304
t0073372	23	1	CGGTTCGAGGCTACAGTGAGATT	miRNA	miR-1304
t0076420	20	1	TTTAGAGACGGGGTCTTGGT	miRNA	miR-1303
t0078448	23	1	TAGGGCCCTGTCTCCATCTCCTT	miRNA	miR-1296
t0083874	19	1	TGTGAGGTTGGCATCGTTG	miRNA	miR-1294
t0085946	19	1	TGTGCGGTTGGCATTGTTG	miRNA	miR-1294
t0086138	20	1	TGTGCGGTTGGCATTGTTGT	miRNA	miR-1294
t0086315	19	1	TGTGAGGTTGGCCTTGTTG	miRNA	miR-1294
t0086667	20	1	TGTGAGGTTGGGATTGTTGT	miRNA	miR-1294
t0086690	19	1	AGTGAGGTTGGCATTGTTG	miRNA	miR-1294
t0042251	20	1	TATGAGGTTGGCATTGTTGT	miRNA	miR-1294
t0054292	19	1	TGTGAGGTTGCCATTGTTG	miRNA	miR-1294
t0080588	20	1	TGTGAGGTTGGAATTGTTGT	miRNA	miR-1294
t0087818	20	1	TGTGAGGGTGGCATTGTTGT	miRNA	miR-1294
t0093550	19	1	TATGAGGTTGGCATTGTTG	miRNA	miR-1294
t0085673	19	1	TGTGAGGGTGGCATTGTTG	miRNA	miR-1294
t0041334	19	1	TGTGAGGTTGGCGTTGTTG	miRNA	miR-1294
t0041169	20	1	TGTGAGGTTGACATTGTTGT	miRNA	miR-1294
t0049449	19	1	TGCGAGGTTGGCATTGTTG	miRNA	miR-1294
t0053516	20	1	ATGGATTTTTGGCGCAGGGA	miRNA	miR-1290
t0059584	24	1	CTTTTTGTGGTCTGGGCTTGCATC	miRNA	miR-129
t0061321	24	1	CTTTTTGCGGTCTGGGATTGCATC	miRNA	miR-129
t0062533	24	1	CTTTTTGCGGTCTGGGCTTGTATC	miRNA	miR-129
t0063557	24	1	TCTAGGCAACAAAGTGAGACCTGA	miRNA	miR-1285a
t0068941	23	1	TCCGGGCAACAAAGTGAGACCTA	miRNA	miR-1285

t0071377	23	1	TCTGGGCAACAAAGGGAGACCTA	miRNA	miR-1285
t0073312	21	1	TCTGGGTAACAAAGTGAGACC	miRNA	miR-1285
t0075197	24	1	TCTGGGCAACATAGTGAGACCTGA	miRNA	miR-1285
t0079229	24	1	TCTGGGCAACAAAGTGGGACCTGA	miRNA	miR-1285
t0082880	24	1	TCTGGACAACAAAGTGAGACCTGA	miRNA	miR-1285
t0087348	20	1	TCTGGGCAACAAAGTGAAAC	miRNA	miR-1285
t0090774	20	1	TCTGGGCAAAAAAGTGAGAC	miRNA	miR-1285
t0091365	19	1	TCACAGTGAACCGGTCTAT	miRNA	miR-128
t0054550	20	1	TCGCAGTGAACCGGTCTCTT	miRNA	miR-128
t0063633	21	1	TCACAGTGGACCGGTCTCTTT	miRNA	miR-128
t0069798	20	1	TCACAGTGAACCGGTTTCTT	miRNA	miR-128
t0050269	21	1	TCACAGCGAACCGGTCTCTTG	miRNA	miR-128
t0043852	20	1	TCACAGTGAACCAGTCTCTT	miRNA	miR-128
t0044977	21	1	TCACAGTGAACCGGGCTCTTT	miRNA	miR-128
t0046022	20	1	TCACAGTGATCCGGTCTCTT	miRNA	miR-128
t0059777	21	1	TCACAGTGAACCGATCTCTTT	miRNA	miR-128
t0067010	20	1	TAGGACTGTGCATATCATCT	miRNA	miR-1278
t0071568	20	1	TACGTAGATATATATGTATT	miRNA	miR-1277
t0077691	21	1	GCGGGGGAGAGGCTGTAATTT	miRNA	miR-1275
t0083064	21	1	TGAGAGGCAGAGGTTGCAGGG	miRNA	miR-1273f
t0040791	23	1	TGAGAGGCAGAGGTTGCCGTGGA	miRNA	miR-1273f
t0054343	21	1	TGGGAGGCCGAGGTTGCAGTG	miRNA	miR-1273f
t0054817	25	1	GGCGACAAAACGAGACCCTGGCAAA	miRNA	miR-1273c
t0058105	23	1	CTGGAGATATGGAAGAGCTGTGG	miRNA	miR-1270
t0074372	22	1	CTGGAGATATGGCAGAGCTGTG	miRNA	miR-1270
t0076598	23	1	ACTGGAGACATGGAAGAGCTGTG	miRNA	miR-1270
t0082918	23	1	ACTGGAGATATGGAAGAGCTGGG	miRNA	miR-1270
t0084452	24	1	TCGGAACCGTCTGAGCTTGGTATC	miRNA	miR-127
t0092276	24	1	TCGGATCCGTCCGAGCTTGGCATC	miRNA	miR-127
t0057338	24	1	TCGGATCCGTCCGAGCTTGGTATC	miRNA	miR-127
t0059590	24	1	TCGGATCCGTCTGAGATTGGTATC	miRNA	miR-127
t0083864	21	1	ATCCTACCGCTGCCACCAAAA	miRNA	miR-1260b
t0063790	20	1	ATCCCACCGCTGCCACCACG	miRNA	miR-1260b
t0070018	21	1	ATCCCACCTCTGCCACCAAAA	miRNA	miR-1260b
t0071648	21	1	ATCCCACCACTGCCACCAAAA	miRNA	miR-1260b
t0066484	21	1	ATCCCACCGCTGCCACAAAGA	miRNA	miR-1260b
t0060176	20	1	ATCCCACCGCTGCCACGAAG	miRNA	miR-1260b
t0073420	20	1	ATCCCAACGCTGCCACCAAA	miRNA	miR-1260
t0081625	20	1	ATCCCAACGCTGCCACCAAG	miRNA	miR-1260
t0089691	26	1	CTCGTACCGTGAGTAATCATGCGATC	miRNA	miR-126
t0049500	25	1	TCGTACCGTGAGTGATAATGCAATC	miRNA	miR-126
t0051992	25	1	TCGTACCGTGAGTAATAATGCAATA	miRNA	miR-126
t0077055	24	1	CGTACCGTGAGTAATAATGCGCTC	miRNA	miR-126
t0085953	25	1	ACGTACCGTGAGTAATAATGCGATC	miRNA	miR-126
t0052553	25	1	TCGTACCGTGAGTAATAATGCGCTC	miRNA	miR-126
t0053384	25	1	TCGTACCGTGAGTGATAATGCGATC	miRNA	miR-126
t0059332	23	1	GTACCGTGAGTAATAATGCGATC	miRNA	miR-126
t0060497	25	1	TCGTACCGTGAGGAATAATGCGATC	miRNA	miR-126
t0078576	25	1	TCGTACCGTGAGGACTAATGCGATC	miRNA	miR-126
t0090165	25	1	TCGTACCGTGGGTAATAATGCGATC	miRNA	miR-126
t0053074	26	1	CCCGTACCGTGAGTAATAATGCGATC	miRNA	miR-126
t0068640	42	1	TCCCTGAGACCCTAACTTGTGAGTCTGGG	miRNA	miR-125b-5p
t0045740	22	1	AGTCATTGACTTCTCTCTAGCT	miRNA	miR-1256
t0048912	22	1	AGGCATTGACTTCTCACTAGAT	miRNA	miR-1256
t0049055	22	1	CGGCTGAGCAAAGAAAGTGGTT	miRNA	miR-1255b
t0054208	22	1	TGGATGAGCAAAGAAAGTGGTT	miRNA	miR-1255b
t0061530	22	1	CGGATGAGCAAAGAAAGAGGTT	miRNA	miR-1255b
t0062458	22	1	CGGATGAGCAAAGAAAGTGGGT	miRNA	miR-1255b

t0066747	21	1	CGGATGAGAAAAGAAAGTGGT	miRNA	miR-1255b
t0079121	21	1	CGGATGCGCAAAGAAAGTGGT	miRNA	miR-1255b
t0081370	22	1	CGGATGACCAAAGAAAGTGGTT	miRNA	miR-1255b
t0082705	22	1	AGCCTGGAAGCTGGAGCCTGCC	miRNA	miR-1254
t0083218	21	1	TAGGCACGCTGTGAAGAGACA	miRNA	miR-124e
t0083608	20	1	TAAGACTCGGTGGATGCTCA	miRNA	miR-124b
t0046537	20	1	GTGGGCGGGGGCAGGTGGGT	miRNA	miR-1228*
t0045102	21	1	GTGGGCGGGGGCAGGTGTGGG	miRNA	miR-1228*
t0069508	20	1	GTGGGCGGGGGCAGGTATGT	miRNA	miR-1228*
t0049247	21	1	GGGGGCGGGGGCAGGTGTGGG	miRNA	miR-1228*
t0050984	21	1	CGTGCCACCCTTTTTCCCAGT	miRNA	miR-1227
t0057149	21	1	GTGAGGACTCGGGAGGTGAGA	miRNA	miR-1224-5p
t0065341	21	1	CCCCCTCACCCTCTCCTGCAG	miRNA	miR-1224-3p
t0077385	23	1	CTTCCCCACCCTCTCCTGCAGGA	miRNA	miR-1224-3p
t0078129	21	1	GTGAGGACTCGGGAAGTGGAG	miRNA	miR-1224
t0086202	20	1	GCGAGGACTTGGGAGGTGGA	miRNA	miR-1224
t0085837	20	1	GCGAGGACTCGGGAGGTGGA	miRNA	miR-1224
t0070993	19	1	GTGGGGACTCGGGAGGTGG	miRNA	miR-1224
t0041758	22	1	GTGAGGACTCGGGAGGTGGAGC	miRNA	miR-1224
t0057574	20	1	GTGAGGATTCGGGAGGTGGA	miRNA	miR-1224
t0057710	19	1	GTGATGACTCGGGAGGTGG	miRNA	miR-1224
t0067147	21	1	GGGAGGACTCGGGAGGTGGAG	miRNA	miR-1224
t0092579	21	1	GGGAGGACTCGGGAGGGGGAG	miRNA	miR-1224
t0090618	21	1	GTGAGGACTCGGGAGGCGGAG	miRNA	miR-1224
t0082206	21	1	GTGAGGACTCGGGAGGTTGAA	miRNA	miR-1224
t0071479	24	1	TGGAGGGTGACAATGGTGTTTATC	miRNA	miR-122
t0072201	21	1	TGGAGTGTGACAATGGGGTTT	miRNA	miR-122
t0043412	22	1	TGGAGTGTGGCAATGGTGTTTG	miRNA	miR-122
t0052725	26	1	TGGAGTGTGACAATGGTGTTTGTTTC	miRNA	miR-122
t0054698	24	1	TTGAGTGTGACAATGGTGTTTATC	miRNA	miR-122
t0062027	21	1	TGGAGTGTGACAATGGAGTTT	miRNA	miR-122
t0069154	24	1	TGGAGTGTGACAATGGTGTTTCTC	miRNA	miR-122
t0070968	24	1	TGGAGTGAGACAATGGTGTTTATC	miRNA	miR-122
t0076155	20	1	TTTCCGGCTCGCGTGGGCGT	miRNA	miR-1180
t0078083	20	1	TTTCCGGCTCGCGTGGGCGC	miRNA	miR-1180
t0082496	25	1	ACCCTGTAGAACCGAATTTGTGATC	miRNA	miR-10d
t0086491	25	1	TACCCTGTAGAACCGAATTTTTATC	miRNA	miR-10b
t0092216	24	1	ACCCTGTAGAACCGAATTTGTATC	miRNA	miR-10b
t0049698	25	1	TACCCTGTAGATCCAAATTTGTATC	miRNA	miR-10a
t0050012	25	1	TACCCTGAAGATCCGAATTTGTATC	miRNA	miR-10a
t0051225	25	1	TACCCTGTAGATCCGAATTTTTATC	miRNA	miR-10a
t0054270	25	1	TACCCTGTAGATCCGAATTTGGATC	miRNA	miR-10a
t0059543	25	1	TACCCTGTAGATCCGATTTTGTATC	miRNA	miR-10a
t0063331	22	1	TACTCTGTAGATCCGAATTTGT	miRNA	miR-10a
t0074591	25	1	TACCCTGTAGATCCGAATTTGAATC	miRNA	miR-10a
t0091410	25	1	TACCCTGTAGATCCGAATTTGCATC	miRNA	miR-10a
t0081175	21	1	TACCCTATAGATCCGAATTTG	miRNA	miR-10a
t0082641	25	1	TACACTGAAGATCCGAATTTGTATC	miRNA	miR-10a
t0081544	21	1	AGAAGCATTGTACAGGGCATT	miRNA	miR-107b
t0058974	22	1	AGCAGTATTGTACAGGGCTTTG	miRNA	miR-107b
t0059132	21	1	AGCAGCATTGTACAGGGGTTT	miRNA	miR-107b
t0042978	20	1	AGCAGCATAGTACAGGGCTT	miRNA	miR-107b
t0045016	20	1	AGCAGCATTGTGCAGGGCTT	miRNA	miR-107b
t0047900	20	1	AGCAGCATTGTACAGGGATT	miRNA	miR-107b
t0050568	21	1	AGCAGCATTGTACAGGGATTT	miRNA	miR-107b
t0053321	22	1	AGCAGCATTGTACAGGGCTTTT	miRNA	miR-107b
t0060874	20	1	AGCAGCATTGTACAGGGGAT	miRNA	miR-107b
t0062999	23	1	AACAGCATTGTACAGGGCTATAA	miRNA	miR-107

t0064956	23	1	AGCAGCATTGTACAGGGTTATAA	miRNA	miR-107
t0069518	23	1	AGCAGCATTGTACAGGGATATCT	miRNA	miR-107
t0081310	22	1	AGCAGCTTTGTACAGGGCTATT	miRNA	miR-107
t0057355	25	1	GGCAGCATTGTACAGGGCTATAAAC	miRNA	miR-107
t0058444	22	1	AGCAGCATTGTAGAGGGCTATT	miRNA	miR-107
t0060330	22	1	AGCAGCATTGTATAGGGCTCTT	miRNA	miR-107
t0076463	22	1	GGCAGCATTGTACAGGGCTATA	miRNA	miR-107
t0082409	24	1	AGCAGCATTGTACAGGGCTATAAT	miRNA	miR-107
t0082421	22	1	AGGAGCATTGTACAGGGCTATA	miRNA	miR-107
t0086784	22	1	AGCAGCATAGTACAGGGCTATA	miRNA	miR-107
t0090516	22	1	AGCAGCATTGTACAGGGGTATT	miRNA	miR-107
t0092015	23	1	AGCAGCATTGTACAGGGCTATAG	miRNA	miR-107
t0041083	23	1	AGTAGCATTGTACAGGGCTATAA	miRNA	miR-107
t0044966	22	1	AGCAGCATTGTACCGGGCTATT	miRNA	miR-107
t0045072	23	1	AGCAGCATTGGACAGGGCTATAA	miRNA	miR-107
t0047131	22	1	AGCACCATTGTACAGGGCTAGT	miRNA	miR-107
t0048601	22	1	AGCAGCATTGTACAGGACTATT	miRNA	miR-107
t0054029	22	1	AGCAGCATTGTACAAGGCTATA	miRNA	miR-107
t0054493	22	1	AGCAGCATTGTCCAGGGCTATT	miRNA	miR-107
t0056366	22	1	AGCAGCACTGTACAGGGCTATA	miRNA	miR-107
t0056405	22	1	AGCAGCATTGTACATGGCTATT	miRNA	miR-107
t0058235	23	1	AGAAGCATTGTACAGGGCTATAA	miRNA	miR-107
t0058927	22	1	AGCAGCATTGTACAGGGCAATT	miRNA	miR-107
t0061500	22	1	AGCAGCCTTGTACAGGGCTATT	miRNA	miR-107
t0066217	23	1	AGCAGCATGGTACAGGGCTATAA	miRNA	miR-107
t0066752	22	1	AGCAGGATTGTACAGGGCTATA	miRNA	miR-107
t0066824	22	1	AGCAGGATTGTACAGGGCTATT	miRNA	miR-107
t0070217	23	1	ACCAGCATTGTACAGGGCTATAA	miRNA	miR-107
t0070319	23	1	AGCAGCATTGTACAAGGCTATAA	miRNA	miR-107
t0071688	22	1	AGCAGCATTGTACGGGGCTATT	miRNA	miR-107
t0073683	23	1	AGCGGCATTGTACAGGGCTATAA	miRNA	miR-107
t0073687	22	1	AGCAGCATTGTGCAGGGCTATA	miRNA	miR-107
t0076733	20	1	CAGCATTGTACAGGGCTATC	miRNA	miR-107
t0077961	22	1	AGCACCATTGTACAGGGCTATT	miRNA	miR-107
t0080940	21	1	CCGCACTGTGGGTACTTGCTT	miRNA	miR-106b*
t0083706	22	1	TACCGCACTGTGGGTACTTACT	miRNA	miR-106b*
t0084031	21	1	ACCGCGCTGTGGGTACTTGCT	miRNA	miR-106b*
t0085063	24	1	CCGCACTGTGGGTACTTGCTGAGA	miRNA	miR-106b*
t0085099	21	1	TACCGCACTGTGGGTAATTGT	miRNA	miR-106b*
t0088728	22	1	CCGCACTGTGGGGACTTGCTGA	miRNA	miR-106b*
t0089494	20	1	CCGCACCGTGGGTACTTGCT	miRNA	miR-106b*
t0089537	22	1	CCGCACTGTGGGTACTTGCTGG	miRNA	miR-106b*
t0090561	29	1	TACCGCACTGTGGGTAATTGCTAAAAAGC	miRNA	miR-106b*
t0090864	21	1	ACCGCACTGTGGGTACTTGTT	miRNA	miR-106b*
t0090976	23	1	CCGCACTGGGGGTACTTGCTGAA	miRNA	miR-106b*
t0092136	22	1	TACCGCACTGTGGGCACTTGCA	miRNA	miR-106b*
t0092751	21	1	ATCGCACTGTGGGTACTTGCT	miRNA	miR-106b*
t0040846	23	1	CCGCACTGTGGGTACTTACTGCA	miRNA	miR-106b*
t0041321	21	1	CCGCACTGTGGGTAATTGCTG	miRNA	miR-106b*
t0041527	22	1	TACCGCCCTGTGGGTACTTGCT	miRNA	miR-106b*
t0042045	20	1	CCGCGCTGTGGGTACTTGAT	miRNA	miR-106b*
t0042346	29	1	TACCGCACTGTGGGTACTTGCTAAAAAAC	miRNA	miR-106b*
t0043748	20	1	TACCGCACTGTGGGTACTTT	miRNA	miR-106b*
t0047723	20	1	CCGCGCTGTGGGTACTTGCT	miRNA	miR-106b*
t0047960	21	1	ACCGCACTGTGGGCACTTGCT	miRNA	miR-106b*
t0047978	20	1	ACGCACTGTGGGTACTTGCT	miRNA	miR-106b*
t0048985	21	1	GCCGCACTGTGGGTACTTGCT	miRNA	miR-106b*
t0049369	20	1	CCGCACTGTGGGTACTCGCT	miRNA	miR-106b*

t0053633	21	1	ACCGCACTGTGGGTACTTGGT	miRNA	miR-106b*
t0053783	22	1	CCGAACTGTGGGTACTTGCTGA	miRNA	miR-106b*
t0054441	20	1	CCGCACTGTGGGTACTTACT	miRNA	miR-106b*
t0055137	22	1	GCGCACTGTGGGTACTTGCTGA	miRNA	miR-106b*
t0055190	23	1	CCGCACTGCGGGTACTTGCTGAA	miRNA	miR-106b*
t0057128	20	1	CCGAACTGTGGGTACTTGCT	miRNA	miR-106b*
t0059136	22	1	TACCGCACTGAGGGTACTTGCT	miRNA	miR-106b*
t0060063	23	1	CCGCACTGTGGGGACTTGCTGCA	miRNA	miR-106b*
t0060083	22	1	TACCGCACTGGGGGGACTTGCT	miRNA	miR-106b*
t0060111	21	1	ACCGCACTGTGGGTACTGGCT	miRNA	miR-106b*
t0060979	22	1	TACCGCACTGTGGGTAATTGCT	miRNA	miR-106b*
t0062015	24	1	CCGCACTGTGGGTCCTTGCTGCGA	miRNA	miR-106b*
t0062844	21	1	ACCGCACTGTGGGTGCTTGCT	miRNA	miR-106b*
t0063210	22	1	TACCACACTGTGGGTACTTGCT	miRNA	miR-106b*
t0063904	22	1	TACCGCACCGTGGGTACTTGCT	miRNA	miR-106b*
t0066719	21	1	TACCGCACTGTGGGTACTTCT	miRNA	miR-106b*
t0067025	21	1	CCGCACTGTGGGGACTTGCTG	miRNA	miR-106b*
t0067635	22	1	CCGCACTGTGGGAACTTGCTGA	miRNA	miR-106b*
t0068087	22	1	TACCGAACTGTGGGTACTTGCT	miRNA	miR-106b*
t0068721	22	1	TACCGCACTGTGGGTACTTGCC	miRNA	miR-106b*
t0068852	20	1	CCGCACTGTGGGTACATGCT	miRNA	miR-106b*
t0069951	20	1	CCGCACTGTAGGTACTTGCT	miRNA	miR-106b*
t0070238	21	1	AACGCACTGTGGGTACTTGCT	miRNA	miR-106b*
t0070292	23	1	TACCGCACTGTGGGTAATTGCTA	miRNA	miR-106b*
t0070829	20	1	GCGCACTGTGGGTAATTGCT	miRNA	miR-106b*
t0070841	21	1	CCGCACTGTGGGCACTTGCTG	miRNA	miR-106b*
t0071177	20	1	CCGCACTGTGGGCACTTGCT	miRNA	miR-106b*
t0071374	20	1	CCGCACTGGGGGTACTTGCT	miRNA	miR-106b*
t0071412	23	1	TACCGCACTGTGGGGACTTGCTA	miRNA	miR-106b*
t0076138	22	1	CCGCACTGTGGGTACTTGCTGC	miRNA	miR-106b*
t0077815	24	1	TAAAGTGCTGACAGCGCAGATAGA	miRNA	miR-106b
t0078114	20	1	TAAAGTGCTGACAGTGGAGA	miRNA	miR-106b
t0078635	21	1	TAAAGTGCCGACAGTGCAGAT	miRNA	miR-106b
t0079603	20	1	TAAAATGCTGACAGTGCAGA	miRNA	miR-106b
t0080955	24	1	TAAAGAGCTGACAGTGCAGATAGT	miRNA	miR-106b
t0083560	23	1	TAAAGTGCTGACAGTGCCGATAG	miRNA	miR-106b
t0084512	20	1	TAAAGTGCTGACAGTGCAGC	miRNA	miR-106b
t0084884	20	1	TAAAGTGCTGACAGTGCCTA	miRNA	miR-106b
t0085240	21	1	TAAAGTGCTGACAGTGTAGAT	miRNA	miR-106b
t0086607	20	1	TGAAGTGCTGACAGTGCAGA	miRNA	miR-106b
t0090841	23	1	TAAAGTGCTGACAGAGCAGATAG	miRNA	miR-106b
t0091376	22	1	TAAAGTGCTGACAGTGCCGATG	miRNA	miR-106b
t0091968	22	1	TAAAGTGCTGACAGTGCCGATT	miRNA	miR-106b
t0041384	20	1	TAAAGTGCTGACAGTGCAGG	miRNA	miR-106b
t0041766	20	1	AAAGAGCTGACAGTGCAGAT	miRNA	miR-106b
t0043442	23	1	TAAAGTGCTGACAGTGCAGATCA	miRNA	miR-106b
t0043923	21	1	TAAAGTGCTGACAATGCAGAT	miRNA	miR-106b
t0044104	23	1	TAAAGTGCTGACAGTGCCGATAA	miRNA	miR-106b
t0044693	23	1	TAAAGTGCTGACACTGCAGATAT	miRNA	miR-106b
t0044710	22	1	TAAAGTGCTGACAGGGCAGAAA	miRNA	miR-106b
t0045135	22	1	TAAAGTGCTAACAGTGCAGATT	miRNA	miR-106b
t0045280	21	1	TAAAGTGCTGACAGAGCAGAA	miRNA	miR-106b
t0045603	20	1	TAAAGTGCTGACAGTGCAAA	miRNA	miR-106b
t0046067	21	1	TAAAGTGCTGATAGTGCAGAT	miRNA	miR-106b
t0046282	20	1	TAAAGTGCTGAAAGTGCAGA	miRNA	miR-106b
t0046852	21	1	TAAAGTGCTGACAGTACAGAT	miRNA	miR-106b
t0046901	20	1	TAAAGTGCTGACAATGCAGA	miRNA	miR-106b
t0047358	20	1	TAAAGTGCTGACAGTGCTGA	miRNA	miR-106b

t0047738	23	1	TAAAGTGCTGACAGTGCCGATGA	miRNA	miR-106b
t0048104	21	1	TAAGGTGCTGACAGTGCAGAT	miRNA	miR-106b
t0050300	21	1	TAAAGTGCTGACGGTGCAGAA	miRNA	miR-106b
t0052025	24	1	TAAAGTGCTGACAGTGCCGATAGA	miRNA	miR-106b
t0053025	20	1	TAAACTGCTGACAGTGCAGA	miRNA	miR-106b
t0053132	22	1	TAAAGTACTGACAGTGCAGATA	miRNA	miR-106b
t0053519	20	1	TAAAGTGCTGGCAGTGCAGA	miRNA	miR-106b
t0058115	22	1	TAAAGGGCTGACAGTGCAGATA	miRNA	miR-106b
t0058158	20	1	TAAAGGGCTGACAGGGCAGA	miRNA	miR-106b
t0058592	21	1	TAAAGTGCTGACAGTGGAGAA	miRNA	miR-106b
t0058908	24	1	TAAAGTGCTGACAGCGCAGATAGT	miRNA	miR-106b
t0059533	21	1	TAAAGTGTTGACAGTGCAGAT	miRNA	miR-106b
t0061059	22	1	TAAAGTGCTTACAGTGCAGATA	miRNA	miR-106b
t0061290	22	1	TAAAGTGCTAACAGTGCAGATA	miRNA	miR-106b
t0061969	20	1	TAAGGTGCTGACAGTGCAGA	miRNA	miR-106b
t0062980	20	1	TAAAGTGCAGACAGTGCAGA	miRNA	miR-106b
t0063189	20	1	TAAAGTGCTGAGAGTGCAGA	miRNA	miR-106b
t0063480	22	1	TAAAGTGCTGACAGGGCAGATT	miRNA	miR-106b
t0063749	21	1	TAAAGTGCTGACAGTGCAAAT	miRNA	miR-106b
t0063970	22	1	TAAAGTGCTGATAGTGCAGATA	miRNA	miR-106b
t0064128	20	1	TAAAGTGCTGACAGTACAGA	miRNA	miR-106b
t0065872	22	1	TAAAGTGCTGACAGTGAAGATG	miRNA	miR-106b
t0066134	22	1	TAGAGTGCTGACAGTGCAGATA	miRNA	miR-106b
t0066427	19	1	TAAGTGCTGACAGTGCAGA	miRNA	miR-106b
t0067104	22	1	TAAAGTGCTGACAGTGCCGATC	miRNA	miR-106b
t0069127	20	1	TAAAGTGCTGATAGGGCAGA	miRNA	miR-106b
t0069250	22	1	TAAAGTGCTGACAGAGCAGAAA	miRNA	miR-106b
t0069424	22	1	TAAAGTGCTGACAGGGCAGATG	miRNA	miR-106b
t0072353	21	1	TAAACTGCTGACAGTGCAGAT	miRNA	miR-106b
t0073446	22	1	ATAAAGTGCTGACCGTGCAGAT	miRNA	miR-106b
t0073949	22	1	TAAAGTGCTGAGAGTGCAGATA	miRNA	miR-106b
t0074105	20	1	TAAAGTGCTGACATTGCAGA	miRNA	miR-106b
t0074428	21	1	TAAAGTGCAGACAGTGCAGAT	miRNA	miR-106b
t0074534	20	1	TAACGTGCTGACAGTGCAGA	miRNA	miR-106b
t0075929	20	1	TAAAGTGCTGACAGTGCAGT	miRNA	miR-106b
t0076788	20	1	TAAAGTGCGGACAGTGCAGA	miRNA	miR-106b
t0077082	21	1	TAAAGTGCTGTCAGTGCAGAT	miRNA	miR-106b
t0077172	22	1	ATAAAGGGCTGACAGTGAAGAA	miRNA	miR-106b
t0077577	20	1	TAAAGTGCTGACAGGGAAGA	miRNA	miR-106b
t0077740	21	1	TAAAGTGCTGACAGCGCAGAA	miRNA	miR-106b
t0078666	20	1	TAAAGTACTGACAGTGCAGA	miRNA	miR-106b
t0078748	21	1	TAAAGAGCTGACAGTGCAGAA	miRNA	miR-106b
t0081381	23	1	AAAAGTGCTTACAGTGAAGGTAG	miRNA	miR-106
t0083022	21	1	AAAAGTGCTTACAGTGCAGGA	miRNA	miR-106
t0083231	21	1	AAAAGTGCTCACAGTGCAGGT	miRNA	miR-106
t0083285	23	1	AAAAGTGCTTACAGTGCAGGCAG	miRNA	miR-106
t0083336	22	1	AAAAGTACTTACAGTGCAGGTA	miRNA	miR-106
t0083851	21	1	AGAAGTGCTTACAGTGCAGGT	miRNA	miR-106
t0085180	24	1	AAAAGTGCTTACAGTGCGGGTAGA	miRNA	miR-106
t0087267	22	1	AAAAGTGCTTACAGTGCAGGTC	miRNA	miR-106
t0090281	22	1	AAAAGTGCCTACAGTGCAGGTA	miRNA	miR-106
t0090594	23	1	AAAAGTGATTACAGTGCAGGTAG	miRNA	miR-106
t0093198	22	1	AAAAGTGCTTACAGTGAAGGTA	miRNA	miR-106
t0093438	21	1	AAAAAATGCTTACAGTGCAGGT	miRNA	miR-106
t0093489	22	1	AAAGGGCTTACAGTGCAGGTAA	miRNA	miR-106
t0046905	22	1	AAAAGTGCTTAAAGTGCAGGTA	miRNA	miR-106
t0047343	22	1	AAAAGTGCTTACAGTGCCGGTC	miRNA	miR-106
t0052392	22	1	AAAAGTGCTTACAGCGCAGGTA	miRNA	miR-106

t0052418	22	1	AAAAGTGCTTACAGTGCAAGTA	miRNA	miR-106
t0052834	21	1	AAAAGTGCTTACAGTGCAGGC	miRNA	miR-106
t0053873	23	1	AAAAGTGCTTACAGTGCTGGTAG	miRNA	miR-106
t0054092	23	1	AAAAGTACTTACAGTGCAGGTAG	miRNA	miR-106
t0056427	22	1	AAAAGTGCTTACAGGGCAGGTA	miRNA	miR-106
t0060709	23	1	AACAGTGCTTACAGTGCCGGTAG	miRNA	miR-106
t0060786	22	1	AAAAGAGCTTACAGTGCAGGAA	miRNA	miR-106
t0060823	22	1	AAAAGTGCTTACAGTGCCGGTA	miRNA	miR-106
t0063044	21	1	AAAAGTGATTACAGTGCAGGT	miRNA	miR-106
t0071102	26	1	ACCCTGTAGATCCGAGTTTGTGAATC	miRNA	miR-10-5p
t0079002	21	1	ACCCTGTGGATCCGAATTTGT	miRNA	miR-10-5p
t0079164	22	1	AGCTTCTTTACAGGGCTGCCTT	miRNA	miR-103a-2*
t0079710	25	1	AGCAGCATTGTACAGGGCTATGAGA	miRNA	miR-103a
t0079832	22	1	AGCAGCATAGTACAGGGTTATG	miRNA	miR-103a
t0081505	23	1	AGCAGCATTGTACAGGGCGAAGA	miRNA	miR-103a
t0082009	21	1	AGCAGCATCGTACAGGGCTAA	miRNA	miR-103a
t0084269	21	1	AGCAGCATTTTACAGTGCTAT	miRNA	miR-103a
t0085222	23	1	AGCAGCATTGTCCAGGGCTATGT	miRNA	miR-103a
t0085588	19	1	AGCAGCATTGTACAAGGCA	miRNA	miR-103a
t0085789	21	1	CAGCATTGTACAGGGCTATGC	miRNA	miR-103a
t0086451	20	1	AAGAAGCATTGTACAGGGCT	miRNA	miR-103a
t0087536	24	1	AGCAGCATTGTACAGTGCTATGAA	miRNA	miR-103a
t0049941	22	1	AGCAGTATTGTGCAGGGCTATG	miRNA	miR-103a
t0073578	22	1	CAGGAGCATTGTACAGGGCTAT	miRNA	miR-103a
t0080303	22	1	AGCAGCATTTTACAGAGCTATG	miRNA	miR-103a
t0040751	25	1	AGCACCATTGTACAGGGCTATGAAA	miRNA	miR-103a
t0041145	24	1	AGCAGCATTGTACAGGGCTAAGAA	miRNA	miR-103a
t0041931	22	1	CGCAGCATTGTAAAGGGCTATG	miRNA	miR-103a
t0042340	21	1	CAGCATTGTACAGGCCTATGA	miRNA	miR-103a
t0042573	20	1	AGCAGCATTGTAAAGGGCTC	miRNA	miR-103a
t0042660	22	1	GCAGCATTGTACAGGGATATGT	miRNA	miR-103a
t0043794	21	1	AGCAGCATTGTATAGGGTTAT	miRNA	miR-103a
t0044437	19	1	AGCAGAATTGTACAGGGCT	miRNA	miR-103a
t0045149	22	1	AGCAGCATTGTAGAGGGATATG	miRNA	miR-103a
t0045197	21	1	AGCACCATTGTACAAGGCTAT	miRNA	miR-103a
t0045798	19	1	AGCAGCATTGTACAGGGGT	miRNA	miR-103a
t0046028	23	1	ACCAGCATTGTGCAGGGCTATGA	miRNA	miR-103a
t0046756	22	1	AGAAGCAATGTACAGGGCTATG	miRNA	miR-103a
t0047425	19	1	AGCAGGATTGTACAGGGCT	miRNA	miR-103a
t0047724	23	1	AGCAGCATTGTACAGGGCTGTGT	miRNA	miR-103a
t0047889	20	1	AGCAGCATTGCACATGGCTA	miRNA	miR-103a
t0048479	22	1	AGCAGCATTGTACAGCCCTATG	miRNA	miR-103a
t0049001	18	1	AGCAGCATTGTATAGGGC	miRNA	miR-103a
t0049024	23	1	AGCAGTATTGTACAGGGCTATGC	miRNA	miR-103a
t0049573	23	1	AGCAGCATTGTACCTGGCTATGA	miRNA	miR-103a
t0050391	23	1	GGCAGCATTGTACAGGGCTATGT	miRNA	miR-103a
t0050433	19	1	AGCAGCATTGTACCGGGCT	miRNA	miR-103a
t0050530	20	1	AGCAGCATTGTACACGGCTC	miRNA	miR-103a
t0051198	21	1	AGCAGCTTTGTACCGGGCTAT	miRNA	miR-103a
t0051578	23	1	AGCAGAATTGTAAAGGGCTATGA	miRNA	miR-103a
t0051735	23	1	GGCAGCATTATACAGGGCTATGA	miRNA	miR-103a
t0051840	21	1	AGCAGTATTGTACCGGGCTAT	miRNA	miR-103a
t0052427	23	1	AGCAGCATTGTACGGGGCTATGT	miRNA	miR-103a
t0052511	23	1	AGCAGCATTGAAAAGGGCTATGA	miRNA	miR-103a
t0052520	24	1	AGCAGCATTGTATAGGGCTATGAA	miRNA	miR-103a
t0053122	24	1	AGCAGGATTGTACAGGGCTATGAT	miRNA	miR-103a
t0053355	24	1	AACAGCATTGTACAGGGCTATGAG	miRNA	miR-103a
t0053547	22	1	GCAGCACTGTACAGGGCTATGA	miRNA	miR-103a

t0053886	21	1	AGCAGAATTGTACAGGGCTAA	miRNA	miR-103a
t0054525	23	1	ACCAGCATTGTACAGGGCTATGT	miRNA	miR-103a
t0055120	19	1	AGCAGCATTGTACAGGGCG	miRNA	miR-103a
t0055249	24	1	AGCAGAATTGCACAGGGCTATGAA	miRNA	miR-103a
t0055408	22	1	AACAGCATTGTACAGGGCTACG	miRNA	miR-103a
t0055938	18	1	AGCAGCCTTGTACAGGGC	miRNA	miR-103a
t0056060	18	1	AGCAGCATTGTACAGGGA	miRNA	miR-103a
t0056220	24	1	AAAGCAGCATTGTACAGGGCTCTG	miRNA	miR-103a
t0056375	23	1	AGCAGCATTGTACAGGGCGATGA	miRNA	miR-103a
t0056443	23	1	AGCAGCAGTGTACAGGGCTAGGA	miRNA	miR-103a
t0056820	21	1	CAGCATTGTATAGGGCTATGA	miRNA	miR-103a
t0056961	21	1	AAGCAGCCTTGTACAGGGCTA	miRNA	miR-103a
t0057124	21	1	AGCAGCATTGTACAGGGTTAA	miRNA	miR-103a
t0057707	22	1	TGCAGCATTGTACATGGCTATG	miRNA	miR-103a
t0057724	23	1	AGCAGCATTGCACAGTGCTATGA	miRNA	miR-103a
t0058272	23	1	AGCACCATTGTACAGGGCCATGA	miRNA	miR-103a
t0058771	22	1	AGCAACATTGTACAGGGTTATG	miRNA	miR-103a
t0059011	22	1	AGCAGCATTGTACAAGGATATG	miRNA	miR-103a
t0059308	23	1	AGCAGCATTGTACATGGCTCTGA	miRNA	miR-103a
t0059560	23	1	AGCAGCATTGTACAGGACTATGT	miRNA	miR-103a
t0060069	21	1	AGCAGCATTGGACAGGGCTAA	miRNA	miR-103a
t0061654	23	1	TAGCAGCATTGTACAGGGCTATG	miRNA	miR-103a
t0061821	21	1	AGCAGCATAGTACAGGGCTAT	miRNA	miR-103a
t0062072	20	1	CAGCATTGTACAGGCCTATG	miRNA	miR-103a
t0062288	24	1	AGCAGCATTGCACAGGGCTATGAT	miRNA	miR-103a
t0062724	23	1	AGCAGCATTGTACAAGGCTATGT	miRNA	miR-103a
t0062848	22	1	AGCAGAATTGTACAGGGATATG	miRNA	miR-103a
t0063667	21	1	AGCAGCATCGTACAGGGCTAG	miRNA	miR-103a
t0063966	37	1	AGCAGCATTGTACAGGGCTATGACATACT	miRNA	miR-103a
t0064764	19	1	AGCAGCATTGTACAAGGCT	miRNA	miR-103a
t0064882	24	1	AGCAGCATTGTACAGGGATATGAA	miRNA	miR-103a
t0065280	23	1	AGCAGCATTGGACAGGGCTATGT	miRNA	miR-103a
t0065337	25	1	AAGCAGCATTGTACAGGGATATGAA	miRNA	miR-103a
t0065409	24	1	AGCAGAATTGTACAGGGCTATGAA	miRNA	miR-103a
t0065860	22	1	AGAAGTATTGTACAGGGCTATG	miRNA	miR-103a
t0067096	24	1	AGCAGCATTGTACAGGGCTCTGAA	miRNA	miR-103a
t0067473	19	1	AGCAGCATTATACAGGGCT	miRNA	miR-103a
t0067861	21	1	AGCAGCATTGTAACGGGCTAT	miRNA	miR-103a
t0068983	23	1	AGCAGCATTGTACAGGGCTCTGT	miRNA	miR-103a
t0068994	22	1	AGCAGCATTCTGCAGGGCTATG	miRNA	miR-103a
t0069097	22	1	GCAGTATTGTACAGGGCTATGA	miRNA	miR-103a
t0069443	18	1	AGCAGCATTGTCCAGGGC	miRNA	miR-103a
t0069493	23	1	AACAGCATTCTACAGGGCTATGA	miRNA	miR-103a
t0069972	24	1	AGCAGCATTGTACACGGCTATGAA	miRNA	miR-103a
t0070376	21	1	AGCAGCATTGTAAAGGGCTAA	miRNA	miR-103a
t0070725	23	1	AGAAGCATTGTACAGGCCTATGA	miRNA	miR-103a
t0070857	22	1	AGCAGCATTGAACCGGGCTATG	miRNA	miR-103a
t0072012	22	1	AGCAGAATTGTACAAGGCTATG	miRNA	miR-103a
t0072400	24	1	AGCAGCATTGTACGGGGCTATGAA	miRNA	miR-103a
t0072489	23	1	AGCAGCATTGTACAGGCCTATGT	miRNA	miR-103a
t0072612	23	1	AACAGCATTGTACAGGGCTATGT	miRNA	miR-103a
t0072652	21	1	AGCAGCATTGAACAGGACTAT	miRNA	miR-103a
t0073123	23	1	AGCAGCATTTTACAGGGCTATGT	miRNA	miR-103a
t0073191	23	1	AGCAGCATTGAACAGGGCTATGT	miRNA	miR-103a
t0073200	23	1	AGCAGCATTGTTCAGGGCTATGA	miRNA	miR-103a
t0073393	20	1	AGCAGCATAGTACAGGGCTA	miRNA	miR-103a
t0073563	22	1	AGCAGCAGTGTACAGGGCTATG	miRNA	miR-103a
t0073698	25	1	AGCAACATTGTACAGGGCTATGAGC	miRNA	miR-103a

t0074409	23	1	AAGCAGCATTGTACAGAGCTATG	miRNA	miR-103a
t0074661	26	1	AGCAGCATTGTACCGGGCTATGAATC	miRNA	miR-103a
t0075017	35	1	AGCAGCATTGTACAGGGCTATGAAACTCT	miRNA	miR-103a
t0075340	21	1	AAGCAGCATTATACAGGGCTA	miRNA	miR-103a
t0075375	21	1	CAGCATTGCACAGGGCTATGA	miRNA	miR-103a
t0075407	19	1	AGCAGCATTGTAAAGGGCA	miRNA	miR-103a
t0075456	22	1	AGAAGCATTGTACAGGGATATG	miRNA	miR-103a
t0077437	23	1	AGAAGCATTGGACAGGGCTATGA	miRNA	miR-103a
t0077705	24	1	AGCAGCATTGTACAGGGCCATGAT	miRNA	miR-103a
t0077828	21	1	AAGAAGCATTGTACAGGGCTA	miRNA	miR-103a
t0078236	21	1	AGCAGCACTGTACAGGGCTAG	miRNA	miR-103a
t0078424	23	1	AGCAGCATTCTACAGGGCTATGT	miRNA	miR-103a
t0078680	19	1	AGCAGCATTGTACAGCGCT	miRNA	miR-103a
t0078958	21	1	AGCAGCATTGAACAGGGATAT	miRNA	miR-103a
t0079515	20	1	AGCAGCATTGTACAGGGCGA	miRNA	miR-103a
t0079701	23	1	AGCAGCCTTGTCCAGGGCTATGA	miRNA	miR-103a
t0080255	21	1	AGCAGCATGGTACAGGGCTAT	miRNA	miR-103a
t0080907	22	1	AGCAGCATTGTACACGGATATG	miRNA	miR-103a
t0081441	22	1	AGCCGCATTGTCCAGGGCTATG	miRNA	miR-103a
t0081749	21	1	AGCAGCATTGTCCAGGGATAT	miRNA	miR-103a
t0081845	21	1	AGCAGCATTGTACAGGGCAAT	miRNA	miR-103a
t0081885	22	1	AGAAGCTTTGTACAGGGCTATG	miRNA	miR-103a
t0082211	23	1	AGCAGCATTGTACAGGGGTATGT	miRNA	miR-103a
t0082451	22	1	AGCAGCATTCTACAGGGTTATG	miRNA	miR-103a
t0082552	24	1	CGCAGCATTGTACAGGGCTATGAA	miRNA	miR-103a
t0082636	20	1	AGCAGTAATGTACAGGGCTA	miRNA	miR-103a
t0082729	22	1	AGAAGCATTGGACAGGGCTATG	miRNA	miR-103a
t0083043	21	1	AGCAGCATTGTACAGTGCTGT	miRNA	miR-103a
t0084177	23	1	AGCAGCATTGTACAGGGCCATGT	miRNA	miR-103a
t0084704	24	1	AGCAGCATCGTACAGGGCTATGTA	miRNA	miR-103a
t0085078	23	1	AGCAGCATTGGACATGGCTATGA	miRNA	miR-103a
t0085687	24	1	AGCAGCGTTGTACAGGGCTATGAA	miRNA	miR-103a
t0086365	24	1	AGCAGCATTGTACAGGACTATGAT	miRNA	miR-103a
t0086873	20	1	AGCAGCATTGTACAGGCCTA	miRNA	miR-103a
t0086879	22	1	AGCAGCATTGTACCGTGCTATG	miRNA	miR-103a
t0087223	18	1	AGCAGCATTGTAAAGGGC	miRNA	miR-103a
t0087360	22	1	CAGCAGCATTGTACAGGGCTAT	miRNA	miR-103a
t0087394	21	1	AGCAGCATCGGACAGGGCTAT	miRNA	miR-103a
t0087791	24	1	AAAGCAGCATTGTACAGGGATATG	miRNA	miR-103a
t0088083	22	1	AACAGCATTGCACAGGGCTATG	miRNA	miR-103a
t0088208	21	1	TAGAGTACTGTGATAACTGAT	miRNA	miR-101c
t0088269	22	1	CACAGTACTGTGATAACTGAAG	miRNA	miR-101c
t0089637	21	1	TACAGTACTGGGATAACTGAT	miRNA	miR-101c
t0089745	19	1	CACAGTACTGTGATAACTG	miRNA	miR-101c
t0090435	22	1	GGACAGTACTGGGATAACTGAA	miRNA	miR-101c
t0090458	22	1	TACGGTACTGTGATAACTGACA	miRNA	miR-101c
t0090950	22	1	TACAGAACTGTGATAACTGATT	miRNA	miR-101c
t0091261	22	1	GAAAAGTACTGTGATAACTGAA	miRNA	miR-101c
t0091737	22	1	CACAGTACTGTGATAACTGAAT	miRNA	miR-101c
t0092872	19	1	CAGTACTGTGGTAACTGAA	miRNA	miR-101c
t0093153	22	1	GTACGGTACTGTGATAACTGAG	miRNA	miR-101c
t0093510	22	1	TACAGTACTCTGATAACTGACT	miRNA	miR-101c
t0093556	19	1	CAGGACTGTGATAACTGAA	miRNA	miR-101c
t0040802	22	1	TAGAGTACTGTGATAACTGATT	miRNA	miR-101c
t0042152	22	1	GTACAGTAATGTGATAACTGAT	miRNA	miR-101c
t0042249	22	1	TACAGTACTGTGGTAACTGACT	miRNA	miR-101c
t0044285	22	1	TACAGTACTGTGATAACTGAGG	miRNA	miR-101c
t0044708	22	1	GACAGTACTGTGATAACTGATA	miRNA	miR-101c

t0045198	22	1	TAGAGTACTGTGATAACTGATA	miRNA	miR-101c
t0046003	19	1	CAGTACTGTGATAACGGAA	miRNA	miR-101c
t0046240	22	1	TACAGTACTGTGATCACTGACT	miRNA	miR-101c
t0046895	19	1	CAGTACTGGGATAACTGAA	miRNA	miR-101c
t0047041	22	1	GGACAGTACTGTGATAACTGAT	miRNA	miR-101c
t0047291	22	1	GTACAGAACTGTGATAACTGAC	miRNA	miR-101c
t0047896	22	1	TACAGTACTGCGATAACTGACT	miRNA	miR-101c
t0048796	22	1	TACAGTCCTGTGATAACTGACT	miRNA	miR-101c
t0049138	22	1	TACAGTACTGTAATAACTGACC	miRNA	miR-101c
t0049546	22	1	GTACAGTACTGTGATAAATGAT	miRNA	miR-101c
t0050104	21	1	AAAGTACTGTGATAACTGAAG	miRNA	miR-101c
t0050354	21	1	TACAGTACTGTGATAAATGAT	miRNA	miR-101c
t0050466	22	1	TACAGTACTGTTATAACTGATA	miRNA	miR-101c
t0051588	22	1	TACAGGACTGTGATAACTGACT	miRNA	miR-101c
t0054648	21	1	GACAGTACTGTGATAACTGAA	miRNA	miR-101c
t0054793	21	1	TACAGTACTGTGTTAACTGAT	miRNA	miR-101c
t0055370	19	1	CAGTCCTGTGATAACTGAA	miRNA	miR-101c
t0055429	22	1	GTACAGTACTGTTATAACTGAC	miRNA	miR-101c
t0057023	21	1	TACAGTACTGTGATCACTGAT	miRNA	miR-101c
t0058652	22	1	TGCAGTACTGTGATAACTGATA	miRNA	miR-101c
t0063925	25	1	TACAGTACTGTGATAACTGACTATT	miRNA	miR-101c
t0064009	19	1	CAGCACTGTGATAACTGAA	miRNA	miR-101c
t0064157	25	1	TACAGTACTGTGATAGCTGACGATC	miRNA	miR-101c
t0064589	19	1	CAGTACTGTGATAAATGAA	miRNA	miR-101c
t0065335	21	1	TACAGTACTGTGGTAACTGAT	miRNA	miR-101c
t0065382	22	1	GAACAGTAATGTGATAACTGAA	miRNA	miR-101c
t0066254	22	1	TACAGTACTGTGATAACTGCTT	miRNA	miR-101c
t0067107	21	1	TACAGTATTGTGATAACTGAC	miRNA	miR-101c
t0068051	22	1	TACAGTACTGTGATAACTGCCT	miRNA	miR-101c
t0069973	21	1	TACAGTGCTGTGATAACTGAT	miRNA	miR-101c
t0071507	22	1	GTACAGTACTGTGATAACTGCT	miRNA	miR-101c
t0072941	26	1	GTACAGTACTGTGATAACTGACCCCA	miRNA	miR-101c
t0072975	22	1	GTACAGGACTGTGATAACTGAT	miRNA	miR-101c
t0073624	22	1	TACAGTATTGTGATAACTGACT	miRNA	miR-101c
t0075219	22	1	GTACAGTACTGTGATACCTGAT	miRNA	miR-101c
t0076387	21	1	TACAGTACTGTGATAACTGCT	miRNA	miR-101c
t0077209	22	1	TACAGTACTGTGATAACTAACT	miRNA	miR-101c
t0077667	19	1	CAGTACTGCGATAACTGAA	miRNA	miR-101c
t0078289	22	1	GTACAGTCCTGTGATAACTGAC	miRNA	miR-101c
t0078713	19	1	ACAGCACTGTGATAACTGA	miRNA	miR-101c
t0079407	22	1	TACAGTACTGTGATAACCGACT	miRNA	miR-101c
t0079920	21	1	ACAGTACTATGATAACTGAAG	miRNA	miR-101b
t0080516	20	1	TACAGTATTATGATAACTGA	miRNA	miR-101b
t0080897	22	1	GTACAGTACTATGATAACTGAG	miRNA	miR-101b
t0081180	22	1	GTACAGTACTATGATAAGTGAA	miRNA	miR-101b
t0081405	22	1	TACAGTAATGTGATAACTGAAT	miRNA	miR-101
t0082250	21	1	TATAGTACTGTGATTACTGAA	miRNA	miR-101
t0082548	21	1	GTACAGTACTTTGATAACTGC	miRNA	miR-101
t0083674	27	1	TACAGTACTGTGATAGCTGAAGAAATC	miRNA	miR-101
t0084123	23	1	TACAGTACTGTAATAACTGAAGA	miRNA	miR-101
t0084168	22	1	TACAGTAATGTGATAACTGAAG	miRNA	miR-101
t0087889	23	1	TACAGTACTGTGATAACGGAAGA	miRNA	miR-101
t0091121	22	1	TACAGTACTGTGTTAACTGAAA	miRNA	miR-101
t0093109	22	1	TACAGTACTGTGAGAACTGAAG	miRNA	miR-101
t0045614	22	1	TCCAGTACTGTGATAACTGAAA	miRNA	miR-101
t0065393	22	1	TACAGTACTGTGATAACAGAAG	miRNA	miR-101
t0081819	19	1	TATAGTACTGTGATAACTG	miRNA	miR-101
t0091701	27	1	GTACAGTACTGTGATAACTGAATCGGC	miRNA	miR-101

t0041140	19	1	TCCAGTACTGTGATAACTG	miRNA	miR-101
t0041593	26	1	TACAGTACTGTGATAGCTGAAGTATC	miRNA	miR-101
t0041663	29	1	GTACAGTACTGTGCTAACTGAACTCTGAA	miRNA	miR-101
t0042106	22	1	TACAGTACTGCGATAACTGTAG	miRNA	miR-101
t0043493	22	1	GTACAGTACTGTGAAAACTGAA	miRNA	miR-101
t0044077	23	1	GTACAGTACTGTGACAACTGAAA	miRNA	miR-101
t0045788	21	1	GTACAGTACTGTGATATCTGA	miRNA	miR-101
t0045916	27	1	TACGGTACTGTGATAACTGAAGAGGGA	miRNA	miR-101
t0047226	21	1	TTACAGTACTGTGATAACTGA	miRNA	miR-101
t0048137	21	1	GTACACTACTGTGATAACTGA	miRNA	miR-101
t0048210	21	1	TACAGTTCTGTGATAACTGAA	miRNA	miR-101
t0048627	23	1	TCGTACAGTACTGTGATAACTGA	miRNA	miR-101
t0048747	23	1	TACAGTACTGTGGTAACTGAAGA	miRNA	miR-101
t0049405	19	1	TACAGGACTGTGATAACTG	miRNA	miR-101
t0049477	42	1	GTACAGTACTGTGATAACTGAACCGGCTC	miRNA	miR-101
t0050040	21	1	TACAGTAATGTGATAAATGAA	miRNA	miR-101
t0051160	22	1	TACAGTACTGTGATAACTAAAT	miRNA	miR-101
t0051876	21	1	TACAGTAATGTGATAATTGAA	miRNA	miR-101
t0052321	22	1	TACAGTACTGTGATCACTGAAG	miRNA	miR-101
t0053277	21	1	TAAAGTACTGTGATAAATGAA	miRNA	miR-101
t0053278	20	1	TACAGTACTGTGATAAGTGA	miRNA	miR-101
t0053812	21	1	TACAGTACTGTGATAACTGTA	miRNA	miR-101
t0053894	20	1	TACAGTACTGTGATACCTGA	miRNA	miR-101
t0054088	23	1	GTACAGTACTGTGGTAACTGAAT	miRNA	miR-101
t0054341	22	1	GTACAGTACTGTGATTACTGAA	miRNA	miR-101
t0054578	20	1	TACAGTACTGTGATAACAGA	miRNA	miR-101
t0055170	21	1	TACAGGACTGGGATAACTGAA	miRNA	miR-101
t0055562	22	1	GTACAGTACTGTGAGAACTGAA	miRNA	miR-101
t0055642	22	1	TACAGTACTGTGATGACTGAAG	miRNA	miR-101
t0056005	22	1	GTACAGTACTGTGATAAATGCA	miRNA	miR-101
t0056187	21	1	TACAGTACTGTGATGAATGAA	miRNA	miR-101
t0057246	22	1	GTAGAGTACTGTGATAACTGAA	miRNA	miR-101
t0057461	22	1	TACAGTACTGTGGTAACTGAAA	miRNA	miR-101
t0057940	23	1	GTACACTACTGTGATAACTGAAA	miRNA	miR-101
t0057984	22	1	TGCAGTACTGTGATAACTGAAA	miRNA	miR-101
t0058934	21	1	GTACAGTACAGTGATAACTGA	miRNA	miR-101
t0059551	21	1	TACAGTAATGCGATAACTGAA	miRNA	miR-101
t0059844	22	1	TACAGTACTGTGATAACTGGAG	miRNA	miR-101
t0059950	23	1	GTACAGTACCGTGATAACTGAAA	miRNA	miR-101
t0061744	21	1	GTACAGTACTGTGCTAACTGA	miRNA	miR-101
t0062626	21	1	TAAAGTACTGTGATCACTGAA	miRNA	miR-101
t0063318	25	1	TACAGTACTGTCATAGCTGAAGATC	miRNA	miR-101
t0063650	21	1	GTACATTACTGTGATAACTGA	miRNA	miR-101
t0063744	21	1	GTACAGTACTGTGATAAGTGA	miRNA	miR-101
t0063763	22	1	TACAGTACTGTGTTAACTGAAG	miRNA	miR-101
t0063892	26	1	TACAGTACTGTGATAACTGAATCCAA	miRNA	miR-101
t0063949	27	1	TACAGTACTGTGTTAACTGAACACGGC	miRNA	miR-101
t0064088	22	1	TACAGTACTGTGAAAACTGAAT	miRNA	miR-101
t0064730	22	1	TACAGTAATGTGATAACTCAAG	miRNA	miR-101
t0064741	21	1	TACAGTACAGTGATAACTGAA	miRNA	miR-101
t0065049	22	1	TGCAGTACTGTTATAACTGAAG	miRNA	miR-101
t0065348	22	1	TACAGGACTGTGATAACTGAAA	miRNA	miR-101
t0066066	23	1	GTACAGTACTGGGATAACTGAAC	miRNA	miR-101
t0066307	22	1	TACAGTACTGTGGTAACTGAAG	miRNA	miR-101
t0066410	22	1	TAAAGTACTGTGATAACTGAAA	miRNA	miR-101
t0066468	21	1	GTCCAGTACTGTGATAACTGA	miRNA	miR-101
t0066578	21	1	TGCAGTACTGTGATACCTGAA	miRNA	miR-101
t0066858	22	1	TACACTACTGTGATAACTGAAA	miRNA	miR-101

t0066864	22	1	TACAGAACTGTGATAACTGAAG	miRNA	miR-101
t0066949	22	1	TACAGGACTGTGATAACTGAAG	miRNA	miR-101
t0067101	22	1	GTACAGTTCTGTGATAACTGAA	miRNA	miR-101
t0067168	22	1	TACAGTACTGTGATATCTGAAG	miRNA	miR-101
t0068171	22	1	TACAGTACGGTGATAACTGAAG	miRNA	miR-101
t0068329	22	1	TACAGTACTGTGATACCTGAAG	miRNA	miR-101
t0068924	21	1	GTACAGTACTGTGATAACAGA	miRNA	miR-101
t0069167	25	1	TACAGTACTGTGATAACTGAATATC	miRNA	miR-101
t0069546	22	1	TACAGTACTGTAATAACTGAAA	miRNA	miR-101
t0069779	22	1	GTACAGTACTGTCATAACTGAA	miRNA	miR-101
t0070936	22	1	TACAGTCCTGTGATAACTGAAG	miRNA	miR-101
t0072204	22	1	GTACAGTACTTTGATAACTGAA	miRNA	miR-101
t0072216	22	1	TACAGTACTGTGATAACTGGAT	miRNA	miR-101
t0072382	20	1	TACAGTACTGTTATAACTGA	miRNA	miR-101
t0072613	25	1	TACGGTACTGTGATAACTGAAGTCC	miRNA	miR-101
t0072810	25	1	TACAGTACTGTGATAACTGAAGGCC	miRNA	miR-101
t0073579	21	1	GTTCAGTACTGTGATAACTGA	miRNA	miR-101
t0074115	22	1	TACAGTACTGTGCTAACTGAAT	miRNA	miR-101
t0074646	20	1	TAAAGTACTGTGATAAATGA	miRNA	miR-101
t0074826	23	1	GTACAGAACTGTGATAACTGAAA	miRNA	miR-101
t0076308	22	1	GTACAGTACTGTGATAACGGAA	miRNA	miR-101
t0077338	22	1	TACAGTACTGTGATAACTAAAA	miRNA	miR-101
t0077720	22	1	TACGTTACTGTGATAACTGAAG	miRNA	miR-101
t0077846	22	1	TACAGTACTGTCATAACTGAAG	miRNA	miR-101
t0078037	21	1	GTACAGTACTGTGATAACCGA	miRNA	miR-101
t0078046	23	1	TACAGTACTGGGATAACTGAAGA	miRNA	miR-101
t0078510	21	1	GTACAGTACTGTAATAACTGA	miRNA	miR-101
t0078771	23	1	TGCAGTACTGTGATAACTGAATA	miRNA	miR-101
t0079173	28	1	TACAGTACTGTGATAACTGAAACAAGCA	miRNA	miR-101
t0079355	22	1	TACAGTACTGCGATAACTGAAT	miRNA	miR-101
t0079618	22	1	TAAAGTACTGTGATAACTGAAT	miRNA	miR-101
t0079704	22	1	TACCGTACTGTGATAACTGAAG	miRNA	miR-101
t0080130	22	1	TACAGTACTGTGATAAATCAAG	miRNA	miR-101
t0080615	20	1	TTCAGTACTGTGATAACTGA	miRNA	miR-101
t0080695	21	1	GTACAGTACTGTGAAAACTGA	miRNA	miR-101
t0081468	22	1	TACAGTATTGTGATAAATGAAA	miRNA	miR-101
t0081814	23	1	TACAGTACCGTGATAACTGAAGA	miRNA	miR-101
t0082068	22	1	TACAGTACTGCGATAACTGAAA	miRNA	miR-101
t0082214	21	1	TCCAGTACTGTGCTAACTGAA	miRNA	miR-101
t0082248	21	1	GTACTGTACTGTGATAACTGA	miRNA	miR-101
t0082364	22	1	TACAGTGCTGTGATAACTGAAT	miRNA	miR-101
t0082406	20	1	TACAGTACTCTGATAACTGA	miRNA	miR-101
t0082412	22	1	TACAGTAATGTGATAACTGAAA	miRNA	miR-101
t0082444	22	1	TACAGCACTGTGATAACTGAAT	miRNA	miR-101
t0082883	22	1	TCCAGTACTGTGATAACTGAAG	miRNA	miR-101
t0083168	20	1	TACAGTACTGTGATAACGGA	miRNA	miR-101
t0083178	21	1	TACAGTACTCTGATAACTGAA	miRNA	miR-101
t0083518	25	1	TACAGTACTGTGATAGCTGAAGATT	miRNA	miR-101
t0083765	22	1	TACAGTACTGTGATAACGGAAG	miRNA	miR-101
t0083866	20	1	GTACAGCACTGTGATAACTG	miRNA	miR-101
t0085292	22	1	TACAGTACTGAGATAACTGAAT	miRNA	miR-101
t0085648	20	1	TACATTAATGTGATAACTGA	miRNA	miR-101
t0085651	21	1	GTAGAGTACTGTGATAACTGA	miRNA	miR-101
t0086411	21	1	TACAGCACTGGGATAACTGAA	miRNA	miR-101
t0086659	20	1	TACAGTACTGTGATAACTGT	miRNA	miR-101
t0086683	42	1	GTACAGTACTGTGATAACTGAACCGGCTC	miRNA	miR-101
t0086839	19	1	TACATTACTGTGATAACTG	miRNA	miR-101
t0087350	21	1	TACGGTATTGTGATAACTGAA	miRNA	miR-101

t0087840	21	1	ATCCAGTACTGTGATAACTGA	miRNA	miR-101
t0088031	23	1	TACAGTACTGTGATGACTGAAGA	miRNA	miR-101
t0088151	20	1	TACAGTACTGTGAGAACTGA	miRNA	miR-101
t0088302	44	1	GTACAGTACTGTGATAACTGAATCGGCTC	miRNA	miR-101
t0089322	24	1	AACCCGTAGATCCGAACTTGTCTC	miRNA	miR-100
t0089790	24	1	TACCCTGTAGAACCGAATTTGTTC	miRNA	miR-10
t0089901	26	1	TACCTTGTAGATCCGAATTTGTGATC	miRNA	miR-10
t0090392	25	1	TACCCTGTAGATCCGAATTTGTTTC	miRNA	miR-10
t0090431	26	1	ACCCTGTAGAACCGAATTTGTGTATC	miRNA	miR-10
t0090442	26	1	TACCCTGTAGATCAGAATTTGTGATC	miRNA	miR-10
t0090897	25	1	TACCCTGTAGATCCGAATTTGTACC	miRNA	miR-10
t0090974	26	1	TACCCTGTAGATCCGAATTTGTGCTC	miRNA	miR-10
t0091027	25	1	TGGAATGTAAAGAAGTGTGGACATC	miRNA	miR-1
t0091417	25	1	TGGAATGTAAAGAAGCGTGTATATC	miRNA	miR-1
t0091799	23	1	TGGAATGTAAAGAAGTGTGTATA	miRNA	miR-1
t0093053	25	1	TGGAATGAAAAGAAGTGTGTACATC	miRNA	miR-1
t0093386	25	1	TGGAATGTAAAGAAGTGTGTACATA	miRNA	miR-1
t0078758	25	1	TGGAATGTAAAGAAGTGTGTATATA	miRNA	miR-1
t0053882	25	1	TGGAATGTAATGAAGTGTGTATATC	miRNA	miR-1
t0059636	25	1	TGGAATGTAAAGAAGTGTGCACATC	miRNA	miR-1
t0060179	25	1	TAGAATGTAAAGAAGTGTGTACATC	miRNA	miR-1
t0060828	25	1	TGGAATGTAAAGAAGTGGGTGTATC	miRNA	miR-1
t0064058	25	1	TGGAATGTAAAGACGTGTGTATATC	miRNA	miR-1
t0071032	25	1	TGGAATGTAAAGAAGTGTGTAAATC	miRNA	miR-1
t0082505	25	1	TGGAATGTAAAGAAGTGTGTCTATC	miRNA	miR-1
t0042025	25	1	TGGAATGTAAAGGAGTGTGTACATC	miRNA	miR-1
t0043308	25	1	TGGAATGTAAAGAAGTGCGTACATC	miRNA	miR-1
t0046955	25	1	TGGAATGTAAAGAAGTGTGCATATC	miRNA	miR-1
t0047403	25	1	TGGAATGGAAAGAAGTGTGTATATC	miRNA	miR-1
t0052532	25	1	CGGAATGTAAAGAAGTGTGTACATC	miRNA	miR-1
t0058222	25	1	TGGAATGTAAAGAAGTGTGTACATT	miRNA	miR-1
t0062990	25	1	TGGAATGTAAAGAAGGGTGTATATC	miRNA	miR-1
t0064875	25	1	TGGAATGAAAAGAAGTGTGTATATC	miRNA	miR-1
t0065345	25	1	TGGAATGTAAAGAAGTGTGTATAAC	miRNA	miR-1
t0067939	25	1	TGAGGTAGTAGATTGAATTGTTATC	miRNA	let-7k
t0069301	18	1	TGAGGTAGTAGATTGAAG	miRNA	let-7k
t0070926	21	1	TAGGTAGTAGATTGAATAGTT	miRNA	let-7k
t0074455	18	1	TGAGGTAGTAGATTGAAT	miRNA	let-7k
t0078031	22	1	TGAGGTACTAGATTGAATAGTT	miRNA	let-7k
t0080391	21	1	GAGGTAGTAGATTGAATAGTT	miRNA	let-7k
t0081486	21	1	TGAGGTAGTAGATGGAATAGT	miRNA	let-7k
t0082588	22	1	TGAGCTAGTAGATTGAATAGTT	miRNA	let-7k
t0083778	20	1	TGAGGTAGTAGATTGAGTAG	miRNA	let-7k
t0084951	22	1	TGAGGTAGTAGATTGAATGGTT	miRNA	let-7k
t0085012	20	1	TGAGGTAGTAGATTGAACAG	miRNA	let-7k
t0090480	20	1	TGAGGTAGTAGATTGAATAA	miRNA	let-7k
t0091470	21	1	TGAGGTAGTAGATTGAATATT	miRNA	let-7k
t0042420	21	1	TGAGGTAGAAGATTGAATAGT	miRNA	let-7k
t0042633	21	1	AGAGGTAGTAGATTGAATAGT	miRNA	let-7k
t0043117	20	1	TGAGATAATAGATTGAATAG	miRNA	let-7k
t0044155	21	1	TGAGGTAGTAGATTGAATAGA	miRNA	let-7k
t0047474	23	1	TGAGGTAGTAGATTGAATAGTAA	miRNA	let-7k
t0048574	22	1	AGAGGTAGTAGATTGAATAGTT	miRNA	let-7k
t0049719	22	1	TGAGGTAGTAGATTGAGTAGTT	miRNA	let-7k
t0051227	20	1	TGAGGTAGTAGATTGAATTT	miRNA	let-7k
t0052683	23	1	TGAGGTAGAAGATTGAATAGTTG	miRNA	let-7k
t0052813	22	1	TGAGGTAGTAGATTGAATATTT	miRNA	let-7k
t0053582	22	1	TAAGGTAGTAGATTGAATAGTT	miRNA	let-7k

t0055260	21	1	TGAGGTAATAGATTGAATAGT	miRNA	let-7k
t0055879	20	1	GAGGTAGTAGATTGAATAGT	miRNA	let-7k
t0057063	21	1	TGAGGTAGTAGAATGAATAGT	miRNA	let-7k
t0057861	21	1	TGAGGTGGTAGATTGAATAGT	miRNA	let-7k
t0059009	22	1	TGAGGTAGTAGATTAAATAGTT	miRNA	let-7k
t0061992	22	1	TGATGTAGTAGATTGAATAGTT	miRNA	let-7k
t0064895	20	1	TGAGGAAGTAGATTGAATAG	miRNA	let-7k
t0069192	21	1	TGAGGTAGTAGATTGAATAGG	miRNA	let-7k
t0069272	20	1	TGAGGTAGTAGATTTTGCAG	miRNA	let-7j
t0074339	19	1	TGAGGTAGTAGATTGTTCG	miRNA	let-7j
t0076778	23	1	TGAGGTAGTAGATTGTTCAGAGG	miRNA	let-7j
t0080921	22	1	TGATGTAGTAGTTTGTGCTGTT	miRNA	let-7i
t0083534	19	1	TCAGGTAGTAGTCTGTGCT	miRNA	let-7i
t0083767	18	1	TGAGGTAGTAGTTTGTGG	miRNA	let-7i
t0085081	19	1	TGAGTTAGTCGTTTGTGCT	miRNA	let-7i
t0086137	23	1	TGAGGTAGTAGTTTGTGGTGTTT	miRNA	let-7i
t0089526	22	1	TGAGGTAGGAGCTTGTGCTGTT	miRNA	let-7i
t0089681	20	1	TGAGGTAGTAGTTTGGGCTT	miRNA	let-7i
t0091528	22	1	TGAGGTAGTAGTTTTTGCTGTT	miRNA	let-7i
t0092157	21	1	TGAGGTAGTAATTTGTGCTGT	miRNA	let-7i
t0093309	22	1	TGAGGGAGTAGTTTGTGCTGTA	miRNA	let-7i
t0043966	20	1	GGAGGTAGTAGTTTGTGCTG	miRNA	let-7i
t0059788	22	1	TGAGGTAGTATTTTGTGCTGTT	miRNA	let-7i
t0083239	25	1	TGCGGTAGTAGTTTGTGCTGTTAGA	miRNA	let-7i
t0040760	25	1	TGAGGTAGTAGTTTGTGCTGTTAGG	miRNA	let-7i
t0041261	21	1	TGAGGTAGTCGTTTGTGCTGT	miRNA	let-7i
t0041467	23	1	TGAGGTAGTAGTATGTGCTGTTA	miRNA	let-7i
t0042332	22	1	TGAGGTAGGAGTTTGTGGTGTT	miRNA	let-7i
t0042385	23	1	TGAGGTAGTAGTTTATGCTGTAA	miRNA	let-7i
t0043511	21	1	TGAGGTAGTAGTCTGTGCTGT	miRNA	let-7i
t0043609	22	1	TGAGGGGGTAGTTTGTGCTGTT	miRNA	let-7i
t0044564	21	1	TCAGGTAGTAGTTTGTGCTGT	miRNA	let-7i
t0045007	20	1	TAAGGTAGTAGTTTGTGTTG	miRNA	let-7i
t0045131	22	1	TGAGGTATTAGTTTGTGCTGTT	miRNA	let-7i
t0045161	21	1	TGAGGTCGTAGTTTGTGCTGT	miRNA	let-7i
t0045538	23	1	TGAGGCAGTAGTTTGTGCTGTTA	miRNA	let-7i
t0046146	21	1	TGTGGTAGTAGTTTGTGCTGT	miRNA	let-7i
t0046309	20	1	TGAGGTAGTAGTTTGTGGTG	miRNA	let-7i
t0046536	22	1	TGAGGTGGAAGTTTGTGCTGTT	miRNA	let-7i
t0046832	20	1	TGAGGTACTAGTTTGTGCTG	miRNA	let-7i
t0047010	20	1	TCAGGTAGTAGTTTGTGCTG	miRNA	let-7i
t0047038	23	1	TGAGGAAGTAGTTTGTGCTGTTG	miRNA	let-7i
t0047528	22	1	TGAGGTAATAGTTTGTGCTGTT	miRNA	let-7i
t0047950	19	1	TGACGTAGTAGTTTGTGCT	miRNA	let-7i
t0049364	23	1	TGAGGTCGTAGTTTGTGCTGTTA	miRNA	let-7i
t0049937	23	1	TGAGATAGTAGTTTGTGCTGTTA	miRNA	let-7i
t0051206	20	1	TGAGATAGTAGTTTGTGCTG	miRNA	let-7i
t0051978	19	1	AGAGGTAGTAGTTTGTGCT	miRNA	let-7i
t0052004	19	1	TGAGGAAGTAGTTTGTGCT	miRNA	let-7i
t0052189	23	1	TGAGGTATTAGTTTGCGCTGTTT	miRNA	let-7i
t0052386	22	1	TGAGGGAATAGTTTGTGCTGTT	miRNA	let-7i
t0052763	23	1	TGAGGTAGTCGTTTGTGCTGTTA	miRNA	let-7i
t0053503	21	1	TGAGGTAGTACTTTGTGCTGT	miRNA	let-7i
t0053591	23	1	TGAGGTCGTAGTTTGTGCTGTTT	miRNA	let-7i
t0053630	21	1	TGAGATAGTAGTTTGTGCTGT	miRNA	let-7i
t0053700	19	1	TGAGGTAGTTGTTTGTGCT	miRNA	let-7i
t0055003	23	1	TAAGGTAGTAGTTTGTGCTGTTA	miRNA	let-7i
t0055598	23	1	TGAGGTAATAGTTTGTGCTGTAA	miRNA	let-7i

t0056412	23	1	TGAGGTAGCAGTTTGTGCTGTTG	miRNA	let-7i
t0056465	20	1	TGCGGTAGTAGTTTGTGCTG	miRNA	let-7i
t0057175	22	1	TGAGAGAGTAGTTTGTGCTGTT	miRNA	let-7i
t0058445	21	1	TGAGGTAGTAGTTTGAGCTGT	miRNA	let-7i
t0058723	20	1	TGAGGTAGTAGTTTGTGATT	miRNA	let-7i
t0058754	23	1	TGAGGTAGTGGTTTGTGCTGTTA	miRNA	let-7i
t0059054	20	1	TGTGGTAGTAGTTTGTGCTG	miRNA	let-7i
t0059954	19	1	TGAGGTAGAAGTTTGTGCT	miRNA	let-7i
t0060226	21	1	TGAGGTAGTAGTTAGTGCTGT	miRNA	let-7i
t0061061	23	1	TGAGGTAGTAGATTGTGCTGTTT	miRNA	let-7i
t0061164	20	1	TGAGGTAGTCGTTTGTGCTG	miRNA	let-7i
t0061177	26	1	TGAGGTAGGAGTTTGTGCTGTTAATC	miRNA	let-7i
t0061375	20	1	TGGGGAAGTAGTTTGTGCTG	miRNA	let-7i
t0061452	24	1	TGAGGTAGTAGTTTGTGTTGTAGG	miRNA	let-7i
t0061639	22	1	TGAGGGAGTAGTTTGTGCTCTT	miRNA	let-7i
t0061816	19	1	TGAGGTAGTAGTTTGTGCC	miRNA	let-7i
t0062019	21	1	TGAGGTTGTAGTTTGTGCTGT	miRNA	let-7i
t0062265	23	1	TGAGGTAGTAGTTTGTGCTTTAA	miRNA	let-7i
t0062823	20	1	TGGGGTAGTAGTTTTTGCTG	miRNA	let-7i
t0063324	20	1	TGAGGTGGTAGTTTGTGTTG	miRNA	let-7i
t0064030	19	1	TGAGGTAGTAGTTTGGGCT	miRNA	let-7i
t0064742	21	1	TGAGGGGGTAGTTTGTGCTGT	miRNA	let-7i
t0064748	19	1	TGAGATAGTAGTTTGTGCT	miRNA	let-7i
t0065167	22	1	TGAGGTAGTAGTTTGTGCCGTA	miRNA	let-7i
t0065936	23	1	TGAGGCGGTAGTTTGTGCTGTTA	miRNA	let-7i
t0066471	23	1	TTAGGTAGCAGTTTGTGCTGTTT	miRNA	let-7i
t0066726	26	1	TGATGTAGTAGTTTGTGCTGTTAATC	miRNA	let-7i
t0067983	19	1	TGAGGTAGTGGTTTGTGCT	miRNA	let-7i
t0068020	26	1	TGAGGGAGTAGTTTGTGCTGTTAATC	miRNA	let-7i
t0068236	26	1	TGAGGTAGTAGTTTGCGCTGTTAATC	miRNA	let-7i
t0068725	21	1	TGAGGTACTAGTTTGTGCTGT	miRNA	let-7i
t0068882	22	1	TGAGGTAGTAGTTTGGGCTGTA	miRNA	let-7i
t0069237	22	1	TGAGGTAGTAGTTAGTGCTGTT	miRNA	let-7i
t0069305	22	1	TGAGGTAGTAGTCTGTGCTGTA	miRNA	let-7i
t0069425	23	1	TGAGGGAGTAGTTTGTGCTGCTT	miRNA	let-7i
t0069437	19	1	CGAGGTAGTAGTTTGTGCT	miRNA	let-7i
t0071181	22	1	TGGGGTAGTAGTTTGTGCTGTA	miRNA	let-7i
t0071394	26	1	TGAGGCAGTAGTTTGTGCTGTTAATC	miRNA	let-7i
t0071768	23	1	CGAGGTAGTAGTTTGTGCTGTTA	miRNA	let-7i
t0072096	21	1	TGAGGTAGTAGTTTGGGCTGT	miRNA	let-7i
t0072157	22	1	TGAGGAGGTAGTTTGTGCTGTT	miRNA	let-7i
t0072978	23	1	TGAGGTAATAGTTTGTGCTGTTT	miRNA	let-7i
t0072992	22	1	TGAGGTAGTAGCTTGTGCTGTG	miRNA	let-7i
t0073307	23	1	TGAGGTAGTAGTTTATGCTGTTA	miRNA	let-7i
t0073996	22	1	TGAGGTAGGAGTTTGCGCTGTT	miRNA	let-7i
t0074168	21	1	TGAGGTAGTAGTTTGTCCTGT	miRNA	let-7i
t0074185	20	1	TGAGGTAGTAGATTGTGCTT	miRNA	let-7i
t0074694	23	1	TGAGCTAGTAGTTTGTGCTGTTT	miRNA	let-7i
t0075726	22	1	TGAGGTAGTAGTTTGTGCTGGA	miRNA	let-7i
t0075971	26	1	TGAGGTAGTAGTTTGTGCTGTTTATC	miRNA	let-7i
t0076423	21	1	TGAGGTAGTATTTTGTGCTGT	miRNA	let-7i
t0077166	22	1	TGAGGTAGTAGTTTGGGATGTT	miRNA	let-7i
t0077299	19	1	GGAGGTAGTAGTTTGTGCT	miRNA	let-7i
t0078264	22	1	TGAAGCAGTAGTTTGTGCTGTT	miRNA	let-7i
t0078705	22	1	TGACGTAGTAGTTTGTGCTGTT	miRNA	let-7i
t0079235	21	1	TGAGGTAGTAGTATGTGCTGT	miRNA	let-7i
t0079280	19	1	TGAGGTAGTAGTTTATGCT	miRNA	let-7i
t0079491	27	1	TGAGGTAGTAGTTTGTGCTGTTAAATC	miRNA	let-7i

t0079903	21	1	TGAGGCAGTAGTTTGTGCTTT	miRNA	let-7i
t0080772	22	1	TGAGGTACTAGTTTGTGCTGTG	miRNA	let-7i
t0081238	23	1	TGAGGTAGTAGTTTGTGCTATTA	miRNA	let-7i
t0082812	19	1	TGAGGTAGTAGTTTGAGCT	miRNA	let-7i
t0083059	23	1	TGAGGTAGTAGTTTTTGCTGTTG	miRNA	let-7i
t0084003	19	1	TGCGGTAGTAGTTTGTGCT	miRNA	let-7i
t0084056	23	1	TGAGGTAGTAGTTTGTGCTGTCA	miRNA	let-7i
t0084351	23	1	TGAGGTAGTGGTTTGTGCTGTTT	miRNA	let-7i
t0084617	19	1	TGAGGTAGCAGTTTGTGCT	miRNA	let-7i
t0084984	25	1	TGAGGTAGTAGATTGTGCTGTTAGA	miRNA	let-7i
t0085042	21	1	TGAGCTAGTAGTTTGTGCTGT	miRNA	let-7i
t0085685	23	1	CGAGGTAGTAGTTTGTGCTGTTG	miRNA	let-7i
t0086016	23	1	TGAGGTAGTAGTTTGTGTTGTTA	miRNA	let-7i
t0086413	21	1	TGATGTAGTAGTTTGTGCTGT	miRNA	let-7i
t0087429	22	1	TGAGGAAGTAGTTTGTGCTGTA	miRNA	let-7i
t0088427	20	1	TGGGGTAGTAGTTTGTGCTT	miRNA	let-7i
t0088772	23	1	TGTGGTAGTAGTTTGTGCTGTTT	miRNA	let-7i
t0089039	20	1	TGAGGGAGTAGTTTTTGCTG	miRNA	let-7i
t0089554	22	1	TGAGGTAGTAGTTCGCGTGGTT	miRNA	let-7h
t0089576	23	1	TGAGGTAGTAATTTGTACAGTTG	miRNA	let-7g
t0090542	21	1	GAGGAAGTAGTTTGTATAGTT	miRNA	let-7g
t0091248	24	1	TCAGGTAGTAGTTTGTACAGTTAA	miRNA	let-7g
t0091469	22	1	TGAGGTAGTAGTTTGTGCAGGT	miRNA	let-7g
t0091690	21	1	TGAGGTAGTAGTTTGTACAAT	miRNA	let-7g
t0091758	23	1	TGAGGTGGTAGTTTGTACAGTTA	miRNA	let-7g
t0092128	22	1	TGAGGTAGTATTTTGTACCGTT	miRNA	let-7g
t0092173	23	1	TGAGATAGTAGTTTGTACAGTTA	miRNA	let-7g
t0092387	22	1	TGAGGTAGTAGCTTGTACAGGT	miRNA	let-7g
t0092507	23	1	TCAGGTAGTAGTTTGTACAGTTA	miRNA	let-7g
t0093458	21	1	GAGGAAGTAGTTTGTACAGTT	miRNA	let-7g
t0093506	25	1	TGAGGTAGTAGTTTGTACAGTTTTC	miRNA	let-7g
t0069978	22	1	TGAGGTAATAGTTTGTACAGAT	miRNA	let-7g
t0040771	23	1	GGAGGTAGTAGTTTGTACAGTTA	miRNA	let-7g
t0042117	21	1	TGACGTAGTAGTTTGTACAGT	miRNA	let-7g
t0042221	22	1	TGAGCTGGTAGTTTGTACAGTT	miRNA	let-7g
t0042287	21	1	TGAGGTAGTAGTGTGTACAGT	miRNA	let-7g
t0042390	20	1	TGAGGTAGTAGCTTGTACAG	miRNA	let-7g
t0042727	22	1	TGAGGTAGTAGTTTGTACTGTA	miRNA	let-7g
t0043260	22	1	TGAGGTAGTAGCTTTTACAGTT	miRNA	let-7g
t0044275	20	1	TGAGGTAGCAGTTTGTACAG	miRNA	let-7g
t0044952	19	1	TGAGGTGGTAGTTTGTACA	miRNA	let-7g
t0045247	21	1	TGAGGCAGTAGTTTGTATAGT	miRNA	let-7g
t0046111	21	1	TGAGGTAGTAGTTTGTACACT	miRNA	let-7g
t0046415	26	1	TGAGGTAGTAGTTTGTACAGTTAATC	miRNA	let-7g
t0046962	20	1	TGAGGTAGTAGTTCGTACAG	miRNA	let-7g
t0047367	22	1	TGAGGTAGTAGTTTGTGAAGTT	miRNA	let-7g
t0047480	22	1	TGAGGAGGTAGTTTGTACAGTT	miRNA	let-7g
t0047966	23	1	TGAGGTAATAGTTTGTACAGTTT	miRNA	let-7g
t0048560	22	1	TGAGGGAGTAGTTTGGACAGTT	miRNA	let-7g
t0049883	24	1	TGGGGTAGTAGTTTGTACAGTTGA	miRNA	let-7g
t0050034	23	1	TGGGGTAGTAGTTTGTACAGTTT	miRNA	let-7g
t0050091	22	1	TGAGTTAGTAGTTTATACAGTT	miRNA	let-7g
t0050591	23	1	TGAGGAAGTAGTTTGTACAGTTT	miRNA	let-7g
t0051135	20	1	TGAGGGAGAAGTTTGTACAG	miRNA	let-7g
t0053139	21	1	TGAGGTTGTAGTTTGTACAGT	miRNA	let-7g
t0053808	23	1	TGAGGTAGTAGTTCGTACAGTTA	miRNA	let-7g
t0055371	22	1	TGAGGCAGTAGTTTGTATAGTT	miRNA	let-7g
t0055967	19	1	TGAGGTAGTAGTTTGTACT	miRNA	let-7g

t0056362	21	1	TGAGGTAGTCGTTTGTACAGG	miRNA	let-7g
t0057256	21	1	TGAGGTCGTAGTTTGTACAGT	miRNA	let-7g
t0059244	23	1	TGAGGTAGTAGTGTGTACAGTTA	miRNA	let-7g
t0059360	22	1	TGAGGTCGTCGTTTGTACAGTT	miRNA	let-7g
t0059966	22	1	TGAGTTAGCAGTTTGTACAGTT	miRNA	let-7g
t0060734	21	1	TGAGGTAGTAGTTTGTAACGT	miRNA	let-7g
t0060750	22	1	TGAGGGGGTAGTTTGTACAGTT	miRNA	let-7g
t0061018	22	1	TGAGGTAGAAGTATGTACAGTT	miRNA	let-7g
t0061344	23	1	TGAGGTAGGAGTTTGTACAGTTG	miRNA	let-7g
t0061362	22	1	TGAGGTGGTAGTTTGTAAAGTT	miRNA	let-7g
t0061774	21	1	TGAGGCAGTAGTTTGTAAAGT	miRNA	let-7g
t0061972	22	1	TGCGGTAGTAGTTTTTACAGTT	miRNA	let-7g
t0063039	23	1	TGAGGTAGTAGTTTATACAGTTA	miRNA	let-7g
t0063220	23	1	TGAGGAAGTAGTTTGTACAGTTG	miRNA	let-7g
t0063297	22	1	TGAGGTGGTCGTTTGTACAGTT	miRNA	let-7g
t0063688	21	1	TGAGGCAGTAGTTTGTGCAGT	miRNA	let-7g
t0063853	21	1	TGAGCTAGTAGTTTGTACAGT	miRNA	let-7g
t0064182	19	1	TGCGGTAGTAGTTTGTACA	miRNA	let-7g
t0064215	22	1	TGAGGGAGTAGTTTGAACAGTT	miRNA	let-7g
t0065542	22	1	TGAGGTATTAGTTCGTACAGTT	miRNA	let-7g
t0065927	23	1	TGAGGTAGCAGTTTGTACAGTTA	miRNA	let-7g
t0067910	23	1	TGAGATAGTAGTTTGTACAGTTG	miRNA	let-7g
t0068082	20	1	TGAGGTAGTAGTTTATACAG	miRNA	let-7g
t0068632	23	1	TGAGGTGGTAGTTTGTACAGTTG	miRNA	let-7g
t0069141	22	1	TGAGGTAGCGGTTTGTACAGTT	miRNA	let-7g
t0069516	20	1	TGAGGAAGTAGTTTGTACAG	miRNA	let-7g
t0070989	20	1	TGAGGAAGTAGTTTGTATAG	miRNA	let-7g
t0071316	23	1	TGAGGTAGTAGTTTATACAGTTG	miRNA	let-7g
t0072187	21	1	TGAGGTAGTAGTTTGTACTTT	miRNA	let-7g
t0072641	21	1	TGAGGTAGTAGTTTGTTCAGT	miRNA	let-7g
t0073846	22	1	TGAAGTAGTAGTTCGTACAGTT	miRNA	let-7g
t0075182	23	1	CGAGGTAGTAGTTTGTACAGTTA	miRNA	let-7g
t0075369	23	1	TGAGGTAGTAGCTTGTACAGTTT	miRNA	let-7g
t0075397	23	1	TGAGGTAGTAGTTTGTACTGTTT	miRNA	let-7g
t0075712	20	1	GAGGTAGTAGTTTGTACAGT	miRNA	let-7g
t0075781	22	1	TGGGGTAGTAGTTTGCACAGTT	miRNA	let-7g
t0076498	22	1	TGAGTTAGTAGTTTGTACAGAT	miRNA	let-7g
t0076646	19	1	TGAGGTAGTAGTTTGTTCA	miRNA	let-7g
t0076785	22	1	TGAGGTAGTAGTTTGTGGAGTT	miRNA	let-7g
t0078454	19	1	TGAGGTAGTAGTTTGAACA	miRNA	let-7g
t0078484	20	1	TGAGATAGTAGTTTGTACAG	miRNA	let-7g
t0078622	24	1	TGAGGAAGTAGTTTGTACAGTTGA	miRNA	let-7g
t0078987	21	1	TGGGGTGGTAGTTTGTACAGT	miRNA	let-7g
t0079242	23	1	TGAGGTAGTAGTTTGTACTGTAG	miRNA	let-7g
t0079509	22	1	TGAGTTAGTAGTTTGTAGAGTT	miRNA	let-7g
t0079842	23	1	TGAGGTAGTAGTTTGTACCGTTA	miRNA	let-7g
t0079965	21	1	TGAGGTAGTAGTATGTACAGT	miRNA	let-7g
t0080350	22	1	TGAGGGAGTATTTTGTACAGTT	miRNA	let-7g
t0081945	23	1	ATGAGGTAGTAGTTTGTACAGTT	miRNA	let-7g
t0082434	22	1	TGAGGTGGTAGTTTGTATAGTT	miRNA	let-7g
t0083055	22	1	TGAGGCAGTAGTTTGTACCGTT	miRNA	let-7g
t0083228	22	1	TGAGGTAGTCGTTTGTGCAGTT	miRNA	let-7g
t0083882	21	1	CGAGGTAGTGGATTGTATAGT	miRNA	let-7f-5p
t0084930	22	1	TGAGGTAGTCGATTGTATAGCT	miRNA	let-7f-5p
t0085793	23	1	TGAGGTAGTGGGTTGTATGGATC	miRNA	let-7f-5p
t0086007	22	1	TGAGGGGGTGGATTGTATAGTT	miRNA	let-7f-5p
t0086325	22	1	TGTGGTAGTGGATTGTATAGTT	miRNA	let-7f-5p
t0086993	20	1	AGGTAGTAGATTGTATAGGG	miRNA	let-7f-5p

t0087252	21	1	TGAGGTAGTAGATTGTATGGA	miRNA	let-7f-5p
t0087530	22	1	TGAGGTAGTAGATTGTATCGGG	miRNA	let-7f-5p
t0088461	22	1	TGAGGTAGTAGATTGTAGTTTT	miRNA	let-7f-5p
t0089211	21	1	TGGGGTAGTGGATTGTTTGGT	miRNA	let-7f-5p
t0089862	21	1	AGAGGTAGTAGATTGTATCCT	miRNA	let-7f-5p
t0090384	22	1	TGAGGTAGTGGGTTGTATATTT	miRNA	let-7f-5p
t0091441	22	1	TGAGGTAGTAGATTGTATTGGT	miRNA	let-7f-5p
t0042028	22	1	AGAGGTAGTAGATTGTATGGGT	miRNA	let-7f-5p
t0042210	22	1	TGAGGTGGTGGATTGTGTAGTT	miRNA	let-7f-5p
t0043293	20	1	GAGGTAGTGGATTGTATAGT	miRNA	let-7f-5p
t0044049	19	1	TGAGGTAGTGGATTGTAGT	miRNA	let-7f-5p
t0044200	22	1	CGAGGTAGTGGATTGTATAGTT	miRNA	let-7f-5p
t0044318	21	1	TGAGGTAGTGGATTGTATATT	miRNA	let-7f-5p
t0044870	21	1	TAGGTAGTGGATTGTATAGTT	miRNA	let-7f-5p
t0045256	23	1	TGAGGTAGTAGATTGTATAGGAA	miRNA	let-7f-5p
t0047885	22	1	TGAGGTAGTGGACTGTATAGTT	miRNA	let-7f-5p
t0049594	24	1	TGAGGTAGTGGATTGTATAGTTAA	miRNA	let-7f-5p
t0050359	22	1	TGGGGTAGTGGATTGTGTAGTT	miRNA	let-7f-5p
t0050553	22	1	TGAGGTAGTGGCTTGTATAGTT	miRNA	let-7f-5p
t0051312	22	1	TGAGGTAGTAGATTGTATACAA	miRNA	let-7f-5p
t0051510	21	1	TGAGGTAGTGGGTTGTATAGA	miRNA	let-7f-5p
t0051730	22	1	TGAGGTAGTTGATTGTATTGTT	miRNA	let-7f-5p
t0052417	20	1	TGAGGTAGTAGATAGTATGT	miRNA	let-7f-5p
t0052768	20	1	TGAGGTAGTACATTGTATTT	miRNA	let-7f-5p
t0053436	22	1	AGAGGTAGTGGATTGTATAGTT	miRNA	let-7f-5p
t0053640	22	1	TGAGTTAGTGGATTGTATAGTT	miRNA	let-7f-5p
t0055060	22	1	TGAGGTAGTAGATTGTATCGTA	miRNA	let-7f-5p
t0055414	22	1	TGAGGTAGTAGATTGTATGGGT	miRNA	let-7f-5p
t0056498	22	1	TGAGGTAGTGGGTTGTATTGTT	miRNA	let-7f-5p
t0057756	21	1	TGAGGTAGTGGATTGGATAGT	miRNA	let-7f-5p
t0058177	20	1	TGAGGTAGTAGATTGGATTT	miRNA	let-7f-5p
t0058676	20	1	TGAGGTAGTAGATCGTATGT	miRNA	let-7f-5p
t0058749	20	1	TGAGGTAGTAGAGTGTATTT	miRNA	let-7f-5p
t0059567	20	1	TGAGGTAGTAGATTGTATGC	miRNA	let-7f-5p
t0059923	22	1	TGAGGTAGTAGATTGTATGGTC	miRNA	let-7f-5p
t0060167	22	1	TGAGGTAGTGGATTGTATAGTC	miRNA	let-7f-5p
t0060481	24	1	TGAGGTAGTGGATTGTATAGTTAG	miRNA	let-7f-5p
t0061279	22	1	TGAGGTAGTCGATTGTATAGTA	miRNA	let-7f-5p
t0061323	44	1	TGAGGTAGTAGATTGTTTGGTGGTGTGGG	miRNA	let-7f-5p
t0062803	22	1	TGCGGTAGTGGATTGTATAGTT	miRNA	let-7f-5p
t0062988	22	1	TGAGGTAGTAGATTGTATGTTT	miRNA	let-7f-5p
t0063542	21	1	TGAGGTAGTTGATTGTATAGG	miRNA	let-7f-5p
t0065020	22	1	TGAGGTAGTGGATTGTATCGTT	miRNA	let-7f-5p
t0065555	20	1	TGAGGCAGTGGATTGTATAG	miRNA	let-7f-5p
t0066013	22	1	TGGGGTAGTGGGTTGTATAGTT	miRNA	let-7f-5p
t0066129	20	1	TGAGGTAGTAGACTGTATTT	miRNA	let-7f-5p
t0066333	20	1	TGAGGTAGTAGATCGTATTT	miRNA	let-7f-5p
t0066893	21	1	TGAGGTACTGGATTGTATAGT	miRNA	let-7f-5p
t0066953	21	1	TGAGGTAGTCGATCGTATAGA	miRNA	let-7f-5p
t0067235	23	1	TGAGGTAGTGGATTGTATGGTTA	miRNA	let-7f-5p
t0067974	24	1	TGAGGTAGTAGATTGTATAGAATC	miRNA	let-7f-5p
t0068563	43	1	TGAGGTAGTGGATTGTTTGGTTTGGTTTG(	miRNA	let-7f-5p
t0069451	22	1	TGAGGTAGTGGATTTTATAGTT	miRNA	let-7f-5p
t0070814	21	1	TGAGGTAGTGGTTTGTATAGT	miRNA	let-7f-5p
t0071134	21	1	TGAGGCAGTGGATTGTATAGT	miRNA	let-7f-5p
t0071758	26	1	TGAGGTAGTGGGTTGTATGGTTTATC	miRNA	let-7f-5p
t0072380	22	1	TGAGGTAGTAGATTGTATGGGG	miRNA	let-7f-5p
t0072939	22	1	TGAGGTAGTGGATTGTATACTT	miRNA	let-7f-5p

t0073689	22	1 TGATGTAGTGGGTTGTATGGTT	miRNA	let-7f-5p
t0074095	21	1 TGGGGTAGTGGATTGTATAGT	miRNA	let-7f-5p
t0077169	21	1 GGAGGTAGTAGATTGTATATA	miRNA	let-7f-5p
t0077228	21	1 TGAGGTAGTGGATTGTTAGTT	miRNA	let-7f-5p
t0077540	22	1 TGAGGTAGTGGATTGTATAGTG	miRNA	let-7f-5p
t0078090	19	1 GGATTAAGGCAGTGGATTG	miRNA	let-7f-5p
t0078463	23	1 ATGAGGTAGTAGATTGTATAGGG	miRNA	let-7f-5p
t0078468	22	1 TGAGGTAGTAGATTGTATTGCG	miRNA	let-7f-5p
t0078613	22	1 TGAGGTAGCGGATTGTATAGTT	miRNA	let-7f-5p
t0080899	22	1 TGAGGTAGTAGATTGTATAATA	miRNA	let-7f-5p
t0081029	20	1 TGAGGTAGTCGATTGTATCG	miRNA	let-7f-5p
t0082856	20	1 TGAGGTAGTCGATTGTAGTT	miRNA	let-7f-5p
t0083997	22	1 TGAGGTAGTAGATTGTAGAGTA	miRNA	let-7f
t0084194	22	1 TGAGTTAGTAGATTGTATAGTA	miRNA	let-7f
t0085810	21	1 TGAGGTAGTAGATTATATTGT	miRNA	let-7f
t0086063	22	1 TGAGGTAGTCGATTGTCTAGTT	miRNA	let-7f
t0086517	21	1 GAGATAGTAGATTGTATAGTT	miRNA	let-7f
t0087379	22	1 TGAGGTAGTAGAGTTTATAGTT	miRNA	let-7f
t0088990	22	1 TGGGGTAATAGATTGTATAGTT	miRNA	let-7f
t0089541	22	1 TGAAGTAGTAGATCGTATAGTT	miRNA	let-7f
t0089577	23	1 GGAGGTAGTAGATTGTATAGTTG	miRNA	let-7f
t0090136	22	1 TTGAGGTTGTAGATTGTATAGT	miRNA	let-7f
t0091178	22	1 TGAGGTGGTAGACTGTATAGTT	miRNA	let-7f
t0092737	20	1 AGGTAGTAGATTGTAAAGTG	miRNA	let-7f
t0093414	21	1 TGAGGTAGAAGATTATATAGT	miRNA	let-7f
t0040789	20	1 TGCGGTAGTAGATTGTCTAG	miRNA	let-7f
t0040843	22	1 TGAGGTAGTAGATTATATTGTT	miRNA	let-7f
t0041033	20	1 TGAGGTAGTCGATTGTGTAG	miRNA	let-7f
t0041082	22	1 TGAGGTATTAGATTGTATAGGT	miRNA	let-7f
t0041084	22	1 TGAGGTAGTAGATCGTATACTT	miRNA	let-7f
t0041088	21	1 GAGGTAGTAGACTGTATAGTT	miRNA	let-7f
t0041127	23	1 TGAGGTAGTAGATTCTATAGTAA	miRNA	let-7f
t0041171	22	1 AGAGGTAGTAGATTGTATAGGT	miRNA	let-7f
t0041186	22	1 TGAGGTAGTCGCTTGTATAGTT	miRNA	let-7f
t0041202	22	1 TGAGGTAGTATATTGTACAGTT	miRNA	let-7f
t0041232	20	1 TGAGGTAGTAGATTGGTTAG	miRNA	let-7f
t0041319	22	1 AGAGCTAGTAGATTGTATAGTT	miRNA	let-7f
t0041456	24	1 TGAGGTAGTAAATTGTATAGTTTG	miRNA	let-7f
t0041557	22	1 TGAGGTAGTAGATTGCACAGTT	miRNA	let-7f
t0041594	23	1 TGAGGTCGTAGCTTGTATAGTTT	miRNA	let-7f
t0041622	21	1 TCAGGTAGTAGATTGTTTAGT	miRNA	let-7f
t0041657	21	1 AGGTAGTAGATTGTATAGATG	miRNA	let-7f
t0041686	22	1 TGAGATAGTAGATTGTATTGTT	miRNA	let-7f
t0041697	21	1 GAGGTAGTAGCTTGTATAGTT	miRNA	let-7f
t0041989	22	1 TCCGGTAGTAGATTGTATAGTT	miRNA	let-7f
t0042297	22	1 AGAGGCAGTAGATTGTATAGTT	miRNA	let-7f
t0042388	21	1 TGAGGTAGTAGATTGTGTAGG	miRNA	let-7f
t0042443	23	1 TGAGGTAGTAGATTGTACAGTGT	miRNA	let-7f
t0042493	23	1 TGAGGTAGTAGATTGGATAGTTG	miRNA	let-7f
t0042754	22	1 TGAGGCAGTAGATTGTGTAGTT	miRNA	let-7f
t0042757	22	1 TGGGGTCGTAGATTGTATAGTT	miRNA	let-7f
t0042862	22	1 TGTGGTAGTAGATTGTTTAGTT	miRNA	let-7f
t0042925	20	1 TGAGGCAGTAGATTGGATAG	miRNA	let-7f
t0042995	21	1 TGAGGTAGAAGATTGTATAGG	miRNA	let-7f
t0043053	22	1 TGAGGTATTAGAATGTATAGTT	miRNA	let-7f
t0043155	22	1 TGAGCTAGTAGATTGTATACTT	miRNA	let-7f
t0043185	21	1 TGGGGTTGTAGATTGTATAGT	miRNA	let-7f
t0043379	20	1 TGAGGTAGTACATTGTATCG	miRNA	let-7f

t0043439	20	1	TGAGCAAGTAGATTGTATAG	miRNA	let-7f
t0043543	21	1	TGAGGTAGTAGATCGTTTAGT	miRNA	let-7f
t0043625	23	1	ATGAGGTAGTAGATTGTATGGTT	miRNA	let-7f
t0043643	22	1	TAAGGTAGTAGATTCTATAGTT	miRNA	let-7f
t0043661	19	1	TGCGGTAGTAGCTTGTATA	miRNA	let-7f
t0043826	22	1	TCAGGTAGTAGCTTGTATAGTT	miRNA	let-7f
t0043842	22	1	TGAGGCAGTAGATTGGATAGTT	miRNA	let-7f
t0043867	22	1	TGAGGTAATAGATTGTACAGTT	miRNA	let-7f
t0043869	22	1	TGCGGTAGTAGATTGTATCGTT	miRNA	let-7f
t0043879	22	1	TGAGATAGTAGATTGTATAGGT	miRNA	let-7f
t0043996	21	1	TGGGGTATTAGATTGTATAGT	miRNA	let-7f
t0044000	24	1	TGAGGTAGTCGATTGTATAGTTAA	miRNA	let-7f
t0044042	22	1	TGAGGTAGTAGATCGTGTAGTT	miRNA	let-7f
t0044256	22	1	TGAGATAGTAGATTTTATAGTT	miRNA	let-7f
t0044491	20	1	TGAGGTAGTAGATTGTGAAG	miRNA	let-7f
t0044531	22	1	TGAGGTAGTTGATTGTAAAGTT	miRNA	let-7f
t0044677	22	1	TGCGGCAGTAGATTGTATAGTT	miRNA	let-7f
t0044680	22	1	TGAGGTAGTAGATTGTACTGTT	miRNA	let-7f
t0044743	22	1	TGTGGTAGTAGATTGTATACTT	miRNA	let-7f
t0044779	22	1	TTAGGTAGTAGATTGTATAGCT	miRNA	let-7f
t0044986	22	1	GAGGTAGCAGATTGTATAGTTG	miRNA	let-7f
t0045132	22	1	TGAGGTAGTAGTTTCTATAGTT	miRNA	let-7f
t0045466	22	1	TGAGGTAGTATATTGTATATTT	miRNA	let-7f
t0045476	24	1	TGAGGTAGTAGATTGTATCGTTAA	miRNA	let-7f
t0045775	22	1	TGAGGTAGTAGACTGCATAGTT	miRNA	let-7f
t0046082	21	1	TGAGGTAGTAGATTGTGGAGT	miRNA	let-7f
t0046170	21	1	TGAGGCAGTAGATTGTACAGT	miRNA	let-7f
t0046241	21	1	TGAGGTAGTAGATTGTTAAGT	miRNA	let-7f
t0046393	21	1	TGAGGTCGTAGATTGTATCGT	miRNA	let-7f
t0046466	24	1	TGAGGTAGAAGATTGTATAGTTGA	miRNA	let-7f
t0046596	21	1	TGAGGTAGCAGATTGTATAGA	miRNA	let-7f
t0046656	22	1	TGAGGTTGTAGATTGTATAGAT	miRNA	let-7f
t0046679	20	1	AGGTACTAGATTGTATAGTG	miRNA	let-7f
t0046736	21	1	TGAGGTAGTAGAATGCATAGT	miRNA	let-7f
t0046783	23	1	TGACGTAGTAGATTGTATAGTTT	miRNA	let-7f
t0046810	21	1	TGAGGCAGTAGATTGCATAGT	miRNA	let-7f
t0046839	21	1	TGAGGTAGTAGATTGTACCGT	miRNA	let-7f
t0046853	19	1	TTAGGTAGTAGATTGTATA	miRNA	let-7f
t0047027	22	1	TTAGGTAGTAGCTTGTATAGTT	miRNA	let-7f
t0047036	20	1	GAGGTAGTAGATTGTATAGG	miRNA	let-7f
t0047047	19	1	TGTAGTAGATTGTATAGTC	miRNA	let-7f
t0047077	24	1	TGAGGTTGTAGATTGTATAGTTAG	miRNA	let-7f
t0047315	21	1	TTGAGGAAGTAGATTGTAAAG	miRNA	let-7f
t0047575	21	1	TAAGGTAGCAGATTGTATAGT	miRNA	let-7f
t0047606	22	1	TGCGGTGGTAGATTGTATAGTT	miRNA	let-7f
t0047780	22	1	CTGAGGAAGTAGATTGTATAGT	miRNA	let-7f
t0047814	23	1	TGAGGTAGTAGACTGTATAGTTA	miRNA	let-7f
t0048067	22	1	CGAGGTAGTAGATTGTATAGGT	miRNA	let-7f
t0048118	22	1	TGAGGTAGTAGCTTGGATAGTT	miRNA	let-7f
t0048233	22	1	CGAGGCAGTAGATTGTATAGTT	miRNA	let-7f
t0048258	20	1	TAGGGTAGTAGATTGTATAG	miRNA	let-7f
t0048349	21	1	GTGAGGTAGTAGATTGTATAG	miRNA	let-7f
t0048472	20	1	TAGGTAGTAGATTGTATAGA	miRNA	let-7f
t0048478	22	1	TGAGGTAGTAGATTGTGTAGTG	miRNA	let-7f
t0048543	22	1	TGAGGTAGTAGATTCTATAGGT	miRNA	let-7f
t0048600	21	1	TGCGGTAGTAGATTGTATAGG	miRNA	let-7f
t0048649	22	1	TGAGGTAGTAGTTTGTATATTT	miRNA	let-7f
t0048850	20	1	TGAGGTAGTAGATTGTGTAT	miRNA	let-7f

t0048962	22	1	TGATGTAGTAGATTGTATAGCT	miRNA	let-7f
t0049289	22	1	TGAGGTAGTAGTTTGTATAGGT	miRNA	let-7f
t0049380	22	1	TGAGGTAGTCGTTTGTATAGTT	miRNA	let-7f
t0049400	22	1	AAAGGTAGTAGATTGTATAGTT	miRNA	let-7f
t0049519	21	1	TGAGGTAGTAGATTGTGTAGC	miRNA	let-7f
t0049659	24	1	TGAGGTAGTAGACTGTATAGTTAA	miRNA	let-7f
t0049771	20	1	TGAGGTAGTAGATTGTGTTG	miRNA	let-7f
t0050134	22	1	TAGGGTAGTAGATTGTATAGTT	miRNA	let-7f
t0050145	19	1	TGAGGTAGTACATTGTATA	miRNA	let-7f
t0050201	21	1	GCGGTAGTAGATTGTATAGTT	miRNA	let-7f
t0050541	22	1	TAAGGTAGTAGATCGTATAGTT	miRNA	let-7f
t0050740	18	1	TGAGCTAGTAGATTGTAG	miRNA	let-7f
t0050906	21	1	TAAGATAGTAGATTGTATAGT	miRNA	let-7f
t0050981	23	1	TGATGTAGTAGATTGTATAGTTG	miRNA	let-7f
t0051395	24	1	TGAGGTAGTAGATTGTATAGTAAG	miRNA	let-7f
t0051423	22	1	TGAGGTAGTAGACTGTATAGCT	miRNA	let-7f
t0051480	21	1	TGAAGTGGTAGATTGTATAGT	miRNA	let-7f
t0051681	22	1	TGAGGTAGCAGATTGTATAGAT	miRNA	let-7f
t0051966	20	1	TGAGGAAGTAGATTGTGTAG	miRNA	let-7f
t0052133	23	1	TGAGGTAGTAGAATGTATAGTTG	miRNA	let-7f
t0052175	22	1	TGAGGTATTAGATTGTGTAGTT	miRNA	let-7f
t0052326	22	1	TTAGGTAGTAGATTGTTTAGTT	miRNA	let-7f
t0052330	22	1	TGAGGTCGCAGATTGTATAGTT	miRNA	let-7f
t0052350	22	1	TGACGTAGTAGATTGTACAGTT	miRNA	let-7f
t0052626	22	1	TGAGGTAGTAGATTATGTAGTT	miRNA	let-7f
t0052655	25	1	TCAGGTAGTAGATTGTATAGTTACA	miRNA	let-7f
t0052741	22	1	TGATGTAGTAGATTGTATACTT	miRNA	let-7f
t0053140	22	1	TCAGGTGGTAGATTGTATAGTT	miRNA	let-7f
t0053183	24	1	TGAGGTAGTAGATTGTATTGTTTG	miRNA	let-7f
t0053235	21	1	GTGGTAGTAGATTGTATAGTT	miRNA	let-7f
t0053380	22	1	CGAGGTAGTAGATTGTATGGTT	miRNA	let-7f
t0053471	22	1	TGAGGTAGTAGATCGCATAGTT	miRNA	let-7f
t0053754	22	1	TGTGGTAGTAGATTGTATAGTA	miRNA	let-7f
t0054022	21	1	TGAGGTAGTAGATGGTATGGT	miRNA	let-7f
t0054252	20	1	TTAGGTAGTAGATTGTGTAG	miRNA	let-7f
t0054344	22	1	TGAGGTAGTAGATTGCGTAGTT	miRNA	let-7f
t0054508	22	1	TGAGGTAGTATCTTGTATAGTT	miRNA	let-7f
t0054586	24	1	TGAGGTAGTAGACTGTATAGTTGA	miRNA	let-7f
t0054631	21	1	TGTGGTAGTAGATTGTGTAGT	miRNA	let-7f
t0054645	21	1	TGAGGTAGTAGATTGTGTCGT	miRNA	let-7f
t0054787	23	1	TGAGGCGGTAGATTGTATAGTTG	miRNA	let-7f
t0054815	21	1	TGAGGTAGTAGATCGTATAGG	miRNA	let-7f
t0054900	22	1	TGAGGTAGTAGATTGTGTCGTT	miRNA	let-7f
t0054993	23	1	TGAGGTAGTAGATTGTATAGTGG	miRNA	let-7f
t0055009	23	1	TTGAGGTAGTAGAATGTATAGTT	miRNA	let-7f
t0055100	21	1	TTGCGGAAGTAGATTGTATAG	miRNA	let-7f
t0055111	21	1	TGAGGCAGTAGATTGTGTAGT	miRNA	let-7f
t0055130	22	1	TGAAGTAGTCGATTGTATAGTT	miRNA	let-7f
t0055262	21	1	TTGAGGTAGTAGATTGGATAG	miRNA	let-7f
t0055356	20	1	TGAGGTAGCAGATTGTATAT	miRNA	let-7f
t0055375	22	1	TGAGGTAGTAGATTGTACGGTT	miRNA	let-7f
t0055556	24	1	TGAGGTAGTAGATTGGATAGTTGA	miRNA	let-7f
t0055648	21	1	TGAGGTAGTAGATTGCCTAGT	miRNA	let-7f
t0055651	21	1	TGAGGTAGTAGTTTGTATAGG	miRNA	let-7f
t0055691	22	1	TGAAGTAGTAGATTGTATACTT	miRNA	let-7f
t0055858	20	1	TGAGGTAGTAGATTGTGTAA	miRNA	let-7f
t0055911	22	1	AGAGGTTGTAGATTGTATAGTT	miRNA	let-7f
t0056007	21	1	TGAGGTCGTAGATTGTCTAGT	miRNA	let-7f

t0056233	22	1	TGAGGTAGTAGATTGTTTAATT	miRNA	let-7f
t0056415	22	1	AGAGGTGGTAGATTGTATAGTT	miRNA	let-7f
t0056420	22	1	TGAGCTAGTAGATTGTATAGGT	miRNA	let-7f
t0056429	22	1	TGAGGCCGTAGATTGTATAGTT	miRNA	let-7f
t0056497	22	1	TAAGGTAGTAGATTGTATACTT	miRNA	let-7f
t0056596	22	1	TAAGGTAGTAGACTGTATAGTT	miRNA	let-7f
t0056643	22	1	TGGGGTAGTAGATTGTATTGTT	miRNA	let-7f
t0056794	21	1	TGACGTACTAGATTGTATAGT	miRNA	let-7f
t0056895	22	1	TGAGGTAACAGATTGTATAGTT	miRNA	let-7f
t0057141	21	1	AGTGGTAGTAGATTGTATAGT	miRNA	let-7f
t0057148	22	1	ATGAGGTAGTAGATTATATAGT	miRNA	let-7f
t0057553	21	1	AGAGGTGGTAGATTGTATAGT	miRNA	let-7f
t0057660	24	1	TGAGGTAGTAGATTGTATAGTATA	miRNA	let-7f
t0057853	22	1	TGAGGTAGTAGATTGTGTAGTC	miRNA	let-7f
t0057855	20	1	CGAGGTAGTAGATCGTATAG	miRNA	let-7f
t0057900	22	1	TTATGTAGTAGATTGTATAGTT	miRNA	let-7f
t0058298	21	1	TGAGGTATTCGATTGTATAGT	miRNA	let-7f
t0058408	18	1	TGAGGTAGTAGATTGTGG	miRNA	let-7f
t0058499	22	1	TGAGGTAGTACATTGTATAGTA	miRNA	let-7f
t0058923	24	1	TGAGGTAGTAGATAGTATAGTTAA	miRNA	let-7f
t0058929	21	1	GAGGTAGTAGATCGTATAGTT	miRNA	let-7f
t0058941	22	1	TGAGGTAGTAGATTGTCTCGTT	miRNA	let-7f
t0059029	21	1	TAAGGTAGTAGATTGTGTAGT	miRNA	let-7f
t0059179	21	1	AGGGGTAGTAGATTGTATAGT	miRNA	let-7f
t0059283	24	1	TGAGGTAGAAGATTGTATAGTTTG	miRNA	let-7f
t0059306	22	1	TGCGGAAGTAGATTGTATAGTT	miRNA	let-7f
t0059323	18	1	AGGTAGTAGATTGTGTAG	miRNA	let-7f
t0059341	22	1	TGAGGTAATATATTGTATAGTT	miRNA	let-7f
t0059466	24	1	TTTGAGGCAGTAGATTGTATAGTT	miRNA	let-7f
t0059491	22	1	TGAGGCAGTAGATTGTACAGTT	miRNA	let-7f
t0059495	20	1	TGCGGTAGTAGATTGGATAG	miRNA	let-7f
t0059610	23	1	TGAGGTAGTAGATTGTACAGTTT	miRNA	let-7f
t0059787	21	1	CCAGGTAGTAGATTGTATAGT	miRNA	let-7f
t0059808	21	1	TGAGGTGGTAGCTTGTATAGT	miRNA	let-7f
t0059926	22	1	TGAGTTAGTATATTGTATAGTT	miRNA	let-7f
t0060178	23	1	TGAGGTCGTAGATTGTATAGTAT	miRNA	let-7f
t0060198	22	1	TGAGGTACTAGATTATATAGTT	miRNA	let-7f
t0060453	23	1	TGAGGTAGTCGATTGTATAGTTT	miRNA	let-7f
t0060588	19	1	AGGTAGTAGATTGTCTAGT	miRNA	let-7f
t0060605	22	1	ATGAGGTAGTAGATTGGATAGT	miRNA	let-7f
t0060645	24	1	TGGGGTAGTAGATTGTATAGTTAA	miRNA	let-7f
t0060701	21	1	TTAGGTAGTAGATTGTATAGA	miRNA	let-7f
t0060810	21	1	TGTGGTAGTAGTTTGTATAGT	miRNA	let-7f
t0060816	23	1	TGAGGTAGTAGATTGTATACTTA	miRNA	let-7f
t0060877	21	1	TGAGGTAGTAGCTTATATAGT	miRNA	let-7f
t0060907	21	1	TAGGAAGTAGATTGTATAGTT	miRNA	let-7f
t0061102	22	1	TTTGAGGTAGTAGATTGTATCG	miRNA	let-7f
t0061189	23	1	TGAGGTAGTAGATAGTATAGTTT	miRNA	let-7f
t0061395	21	1	TGAGGTAGTAGAATGTATATT	miRNA	let-7f
t0061625	41	1	TGAGGTAGTAGATTGTATAGTTTTGTTTGC	miRNA	let-7f
t0061675	22	1	TGAGGTAGTATATTGCATAGTT	miRNA	let-7f
t0061720	22	1	TGAGGTAATAGATTGTATACTT	miRNA	let-7f
t0061839	22	1	TGAGGTAATAGATTGTTTAGTT	miRNA	let-7f
t0061890	21	1	GAGGTAATAGATTGTATAGTT	miRNA	let-7f
t0062266	22	1	TGAGGTAGTAGACTGTATAGTG	miRNA	let-7f
t0062388	22	1	TGAGATAGTAGATTGTATAATT	miRNA	let-7f
t0062544	20	1	TGAGGTGGAAGATTGTATAG	miRNA	let-7f
t0062576	21	1	TTGAGGTAGTAGATTGCATAG	miRNA	let-7f

t0062589	21	1 GAGGTAGTAGATTGTATTGTT	miRNA	let-7f
t0062670	22	1 CTAGGTAGTAGATTGTATAGTT	miRNA	let-7f
t0062761	22	1 TTAGGTAGTATATTGTATAGTT	miRNA	let-7f
t0062762	21	1 TGAGGTGGCAGATTGTATAGT	miRNA	let-7f
t0062861	21	1 TGAGGTTGTAGATTGTATAGA	miRNA	let-7f
t0063001	21	1 CGAGGTAGTAGATCGTATAGT	miRNA	let-7f
t0063011	22	1 TTAGGTAGTAGATTATATAGTT	miRNA	let-7f
t0063047	20	1 TGGAGTAGTAGATTGTATAG	miRNA	let-7f
t0063074	23	1 TGACGTAGTAGATTGTATAGTTA	miRNA	let-7f
t0063157	22	1 TGAGGTAGAAGATTATATAGTT	miRNA	let-7f
t0063293	21	1 TGAGGTGGTAGATTGCATAGT	miRNA	let-7f
t0063458	44	1 TGAGGTAGTAGATTGTATAGTTTGGTTTG(	miRNA	let-7f
t0063603	22	1 TGAGGTAGCAGATTGTCTAGTT	miRNA	let-7f
t0063621	20	1 TGAGGTAGTAGATTGGGTAG	miRNA	let-7f
t0063678	21	1 TGGGGTAGTAGATTGTATCGT	miRNA	let-7f
t0063699	21	1 GAGGTAGTAGATTGTACAGTT	miRNA	let-7f
t0063708	22	1 CGAGGTAGTAGATTGTAAAGTT	miRNA	let-7f
t0063823	24	1 TGAGGTAGTAGATTGTATAGTTCA	miRNA	let-7f
t0063838	22	1 TGACGTAGTAGATTGTATAGTA	miRNA	let-7f
t0063843	21	1 CGCGGTAGTAGATTGTATAGT	miRNA	let-7f
t0063955	21	1 TGAGGTAGTAGCTTGTATACT	miRNA	let-7f
t0064129	22	1 TGAGGTAGTAGAATGTATAGTA	miRNA	let-7f
t0064318	21	1 TGAGGTAGTAGATTGTCTTGT	miRNA	let-7f
t0064392	22	1 CGAGGTAGTAGATTGTATAGTG	miRNA	let-7f
t0064457	22	1 TGAGGTAGTAGATTATATATTT	miRNA	let-7f
t0064462	22	1 GTTTGAGGTAGTAGATTGTATC	miRNA	let-7f
t0064482	22	1 TCAGGTAGTAGATTGTATGGTT	miRNA	let-7f
t0064512	44	1 TGAGGTAGTAGATTGTATAGTTTCGTATA(	miRNA	let-7f
t0064569	20	1 TGGTAGTAGATTGGATAGTT	miRNA	let-7f
t0064723	21	1 TGACGTAGTAGCTTGTATAGT	miRNA	let-7f
t0064871	22	1 TGACGTAGTAGATTGTATAGTG	miRNA	let-7f
t0064957	23	1 ATGAGGAAGTAGATTGTATAGTT	miRNA	let-7f
t0065059	22	1 TGAGGCGGTAGATTGTATAGTT	miRNA	let-7f
t0065084	21	1 TGAGGTAGTAGATTGGATGGT	miRNA	let-7f
t0065120	22	1 ATGAGGAAGTAGATTGTATAGT	miRNA	let-7f
t0065282	22	1 TGAGGAAGAAGATTGTATAGTT	miRNA	let-7f
t0065304	19	1 TGAAGTAGTAGATTGTATA	miRNA	let-7f
t0065390	23	1 TCAGGTAGTAGATTGTATAGTAA	miRNA	let-7f
t0065426	22	1 TGAGGTAGTAGCTTGTCTAGTT	miRNA	let-7f
t0065520	22	1 TTGAGGTAGTAGATTGGATAGT	miRNA	let-7f
t0065660	22	1 CTGAGGTGGTAGATTGTATAGT	miRNA	let-7f
t0065845	22	1 TGAGCTAGTAGATTGTATAGCT	miRNA	let-7f
t0065999	23	1 TGAGGTAGTAGATCGTATAGTTG	miRNA	let-7f
t0066196	18	1 TGAGGCAGTAGATTGTAT	miRNA	let-7f
t0066204	22	1 TGAGGTAGTCGATTGTGTAGTT	miRNA	let-7f
t0066255	23	1 ATGAGGTAGTAGATTGGATAGTT	miRNA	let-7f
t0066394	23	1 TGGGGTAGTAGATTGTATAGTAA	miRNA	let-7f
t0066412	21	1 AGAGGTAGTAGATTGTATAGA	miRNA	let-7f
t0066443	22	1 TGACATAGTAGATTGTATAGTT	miRNA	let-7f
t0066963	22	1 TGATGTAGTAGATTGTATAGGT	miRNA	let-7f
t0067072	21	1 TTGAGGTTGTAGATTGTATAG	miRNA	let-7f
t0067119	22	1 TGAGACAGTAGATTGTATAGTT	miRNA	let-7f
t0067187	20	1 CAGGTAGTAGATTGTATCGT	miRNA	let-7f
t0067276	21	1 TTAGGTAGTAGATTGTATCGT	miRNA	let-7f
t0067405	22	1 GGAGGTGGTAGATTGTATAGTT	miRNA	let-7f
t0067428	21	1 TGAGGTAGTAGATTATATAAT	miRNA	let-7f
t0067481	22	1 TGAGGCAGTAGCTTGTATAGTT	miRNA	let-7f
t0067786	28	1 TGAGGTAGTAGATTGCATAGTTGGTAGT	miRNA	let-7f

t0067839	22	1	TGAGGTGGCAGATTGTATAGTT	miRNA	let-7f
t0067938	22	1	TGAGCTAGTAGATTGTATATTT	miRNA	let-7f
t0067941	26	1	TGAGGCAGTAGATTGTATAGTTAAAA	miRNA	let-7f
t0067967	22	1	TGGGGTAGTAGATTGTATAGAT	miRNA	let-7f
t0068076	22	1	TCAGGTAGTCGATTGTATAGTT	miRNA	let-7f
t0068094	25	1	TGAGGTAGTAGATTGTATAGTTGGG	miRNA	let-7f
t0068143	19	1	CAGGTAGTAGATTGTATAG	miRNA	let-7f
t0068293	22	1	TGCGGTAGTATATTGTATAGTT	miRNA	let-7f
t0068376	21	1	TGAGGTAGTAGATTCTGTAGT	miRNA	let-7f
t0068554	21	1	TTGAGGTAGTAGACTGTATAG	miRNA	let-7f
t0068681	21	1	TGAGCTAGTAGATTGTATCGT	miRNA	let-7f
t0068709	23	1	TGAGGTAGTAGATTGTATGGTTT	miRNA	let-7f
t0068973	21	1	TGAGGTAGTAGATCATATAGT	miRNA	let-7f
t0069035	20	1	GAGGTAGTAGACTGTATAGT	miRNA	let-7f
t0069065	20	1	TGCGGTAGTAGATTGTTTAG	miRNA	let-7f
t0069119	20	1	TGGGGTAGTAGATTGTATCG	miRNA	let-7f
t0069206	21	1	GAGGTAGTAGATTGCATAGTT	miRNA	let-7f
t0069467	22	1	CGAGGTAGTAGACTGTATAGTT	miRNA	let-7f
t0069634	22	1	TAAGGTAATAGATTGTATAGTT	miRNA	let-7f
t0069714	23	1	TGAGGTATTAGATTGTATAGTTA	miRNA	let-7f
t0069787	21	1	CGAGGTAGTAGATTGTATAAT	miRNA	let-7f
t0069823	22	1	CGAGGTAGTAGATTCTATAGTT	miRNA	let-7f
t0069866	22	1	TGATGTACTAGATTGTATAGTT	miRNA	let-7f
t0069903	21	1	GAGGTAGTAGTTTGTATAGTT	miRNA	let-7f
t0069987	22	1	TGATGTAGAAGATTGTATAGTT	miRNA	let-7f
t0070054	22	1	TGATGTAGTAGATTGTGTAGTT	miRNA	let-7f
t0070207	22	1	TGAGGTAGTAGATAGTATAGAT	miRNA	let-7f
t0070279	20	1	TGAGGTAGCAGATTGTGTAG	miRNA	let-7f
t0070338	22	1	TGAGGTGGTAGATTGTACAGTT	miRNA	let-7f
t0070387	22	1	TGAGGTAGTAGATTATACAGTT	miRNA	let-7f
t0070408	22	1	CGAGGTAGTAGATTGTATATTT	miRNA	let-7f
t0070409	21	1	TGAGGTCGTAGATTGTATACT	miRNA	let-7f
t0070428	22	1	TTGGGTAGTAGATTGTATAGTT	miRNA	let-7f
t0070497	22	1	TTAGGAAGTAGATTGTATAGTT	miRNA	let-7f
t0070527	22	1	TAAGGAAGTAGATTGTATAGTT	miRNA	let-7f
t0070539	24	1	TGAGGTAGTAGATTGTGTAGTTAA	miRNA	let-7f
t0070550	21	1	TGAGGTAGTAGCTTGTATTGT	miRNA	let-7f
t0070643	41	1	TGAGGTAGTAGATTGTTTGGTTTGGTTTGC	miRNA	let-7f
t0070722	22	1	ATGAGGTAGAAGATTGTATAGT	miRNA	let-7f
t0070761	22	1	TGAGGTAGTAGCTTGTATACTT	miRNA	let-7f
t0070770	22	1	TGAGGTGGTAGATTGTATCGTT	miRNA	let-7f
t0070811	22	1	TAAGGTAGCAGATTGTATAGTT	miRNA	let-7f
t0070976	21	1	TGGGGTAGTAGATTGTGTAGT	miRNA	let-7f
t0071042	21	1	TGAGATAGTAGATTGTATTGT	miRNA	let-7f
t0071156	20	1	GAGGTGGTAGATTGTATAGT	miRNA	let-7f
t0071255	21	1	TTGGGTAGTAGATTGTATAGT	miRNA	let-7f
t0071298	23	1	TGAGGTGGTATATTGTATAGTTA	miRNA	let-7f
t0071309	22	1	TGGGGTAGTATATTGTATAGTT	miRNA	let-7f
t0071436	22	1	TGAGGAAGTAAATTGTATAGTT	miRNA	let-7f
t0071449	21	1	GAGGTAGTAGATTGTATAGAT	miRNA	let-7f
t0071639	22	1	TGAGGTGGTAGATTGTGTAGTT	miRNA	let-7f
t0071710	22	1	GGAGGTAGTAGATTGTATAATT	miRNA	let-7f
t0071729	21	1	TGAGGTAGTAGATTGTCCAGT	miRNA	let-7f
t0071830	22	1	AGAGGTAGTAGACTGTATAGTT	miRNA	let-7f
t0072052	21	1	GTAGTAGTTTGTATAGTTGAG	miRNA	let-7f
t0072053	23	1	TGAGGTAGTAGATTGTGTAGTAA	miRNA	let-7f
t0072067	18	1	CGAGGTAGTAGATTGTAT	miRNA	let-7f
t0072104	22	1	TAAGGTAGTAGATTGTATAGAT	miRNA	let-7f

t0072114	22	1	TGAGGTGATAGATTGTATAGTT	miRNA	let-7f
t0072267	22	1	ATGAGGTAGTAGATTGTCTAGT	miRNA	let-7f
t0072275	22	1	TGTGGTAGTAGATTGTATTGTT	miRNA	let-7f
t0072315	22	1	GGAGGTAGTAGATTGTATAGTA	miRNA	let-7f
t0072447	22	1	TGAGGTATTAGATTGTATCGTT	miRNA	let-7f
t0072679	24	1	TGAGGTAGTAGATTGTATAGTTAC	miRNA	let-7f
t0072686	20	1	AGGAAGTAGATTGTATAGTT	miRNA	let-7f
t0072901	22	1	TAAGGCAGTAGATTGTATAGTT	miRNA	let-7f
t0073304	22	1	TGAGGAAGTAGATTTTATAGTT	miRNA	let-7f
t0073315	21	1	TGAGGTAGTAGCTTGGATAGT	miRNA	let-7f
t0073474	25	1	TGAGGTAGTAGATTATATAGTCGTC	miRNA	let-7f
t0073637	21	1	TGATGTAGTAGCTTGTATAGT	miRNA	let-7f
t0073638	22	1	TGAGGTAGTAGATTGTCTAGAT	miRNA	let-7f
t0073994	22	1	TGATGTAGTAGCTTGTATAGTT	miRNA	let-7f
t0074225	23	1	TGAGGTCGTAGATTGTATAGTTT	miRNA	let-7f
t0074230	23	1	CTGAAGTAGTAGATTGTATAGTT	miRNA	let-7f
t0074283	22	1	TGAGGTGGTAGATTGTATAGTC	miRNA	let-7f
t0074312	23	1	ACGAGGTAGTAGATTGTATAGTT	miRNA	let-7f
t0074403	21	1	TGAGGAACTAGATTGTATAGT	miRNA	let-7f
t0074407	21	1	TGAGGTAGTAGATTGCGTAGT	miRNA	let-7f
t0074532	22	1	TGAGGTAGTAGATAGTATCGTT	miRNA	let-7f
t0074726	22	1	TGAGGTGGTAGATTGTATACTT	miRNA	let-7f
t0074888	23	1	AGAGGTAGTAGATTGTATAGTTG	miRNA	let-7f
t0075264	22	1	TGAGGTAGTAGAATGCATAGTT	miRNA	let-7f
t0075321	21	1	TGAGGTAGTAGATTCTAGAGT	miRNA	let-7f
t0075426	25	1	TGAGGTAGTAGATTGTGTAGTTGTG	miRNA	let-7f
t0075498	22	1	TGTGGTAGTAGATTGTATGGTT	miRNA	let-7f
t0075584	20	1	TCAGGTAGTAGATTTTATAG	miRNA	let-7f
t0075591	24	1	TGAGGTAGTAGATTGTATAGTTCG	miRNA	let-7f
t0076049	21	1	GAGGTAGAAGATTGTATAGTT	miRNA	let-7f
t0076067	22	1	GAGGTAGTAGATTGTATCGTTT	miRNA	let-7f
t0076097	21	1	TGAGGTAGTAGTTTCTATAGT	miRNA	let-7f
t0076190	22	1	TGAGGTAGTAGATTGTGTAGCT	miRNA	let-7f
t0076226	18	1	TGGGGTAGTAGATTGTAT	miRNA	let-7f
t0076293	22	1	TGAGGTAGCAGACTGTATAGTT	miRNA	let-7f
t0076553	20	1	TTAGGCAGTAGATTGTATAG	miRNA	let-7f
t0076663	21	1	TGAGGTAGCAGATTGTATCGT	miRNA	let-7f
t0076802	22	1	TGAGGAAGTAGAATGTATAGTT	miRNA	let-7f
t0076937	21	1	TGAGGTAGTAGATTGGATTGT	miRNA	let-7f
t0076941	22	1	TGAGGTAGTAGATTGTACAGTC	miRNA	let-7f
t0077130	22	1	TGAGGAAGTAGATTATATAGTT	miRNA	let-7f
t0077173	21	1	TGAGGTAGTAGTTTGTGTAGT	miRNA	let-7f
t0077308	21	1	TGAGGTAGTAGCTTGTATATT	miRNA	let-7f
t0077392	21	1	TGAGGTAGTAGCTTGTATAGC	miRNA	let-7f
t0077417	21	1	GAGGTAGTAGATTGTATAGCT	miRNA	let-7f
t0077462	20	1	TGAGGTAGTAGATCATATAG	miRNA	let-7f
t0077523	22	1	CGAGGTCGTAGATTGTATAGTT	miRNA	let-7f
t0077736	23	1	TTGAGGTAGTAGATTGTATATTT	miRNA	let-7f
t0077946	22	1	TGCGGTAGTCGATTGTATAGTT	miRNA	let-7f
t0078003	24	1	TGCGGTAGTAGATTGTATAGTTGA	miRNA	let-7f
t0078383	19	1	TGAGGTCGTAGATTGTATA	miRNA	let-7f
t0078501	22	1	TGGGGTAGTAGATTGTTTAGTT	miRNA	let-7f
t0078585	22	1	TGAGGAAGTAGATTGTAAAGTT	miRNA	let-7f
t0078601	21	1	TGAGCTAGTAGATTGTACAGT	miRNA	let-7f
t0078723	20	1	TGAGGTGGTACATTGTATAG	miRNA	let-7f
t0078797	22	1	TGAAGTAGTAGTTTGTATAGTT	miRNA	let-7f
t0078808	22	1	TGAGGTAGTAGAGTGTATAGTA	miRNA	let-7f
t0078866	22	1	CGAGGTAGTACATTGTATAGTT	miRNA	let-7f

t0078914	25	1	TGAGGTAGTAGATTGTATAGTTTTG	miRNA	let-7f
t0079211	22	1	TGAGGTAGTAGATTATATAGGT	miRNA	let-7f
t0079803	22	1	TGAGGAAGTAGATTGTACAGTT	miRNA	let-7f
t0079807	22	1	CTGAGGTAGTAGATTGTATAGC	miRNA	let-7f
t0079836	22	1	TGAGGTAGTAGATCATATAGTT	miRNA	let-7f
t0079839	21	1	TGAGGTAGTAGTTTGGATAGT	miRNA	let-7f
t0079881	22	1	TGAGGTAGAAGATTGTACAGTT	miRNA	let-7f
t0079949	24	1	ATGAGGCAGTAGATTGTATAGTTT	miRNA	let-7f
t0080232	20	1	GAGGTAGTAGCTTGTATAGT	miRNA	let-7f
t0080521	22	1	TCAGGAAGTAGATTGTATAGTT	miRNA	let-7f
t0080830	21	1	TGAGGTAGCAGAATGTATAGT	miRNA	let-7f
t0081033	18	1	TGAGCTAGTAGATTGTAT	miRNA	let-7f
t0081071	21	1	TGAGGTAGTAGATCGTATTGT	miRNA	let-7f
t0081415	22	1	TGAGGTAGTAGCTTGTATTGTT	miRNA	let-7f
t0081454	21	1	TGAGGAAGAAGATTGTATAGT	miRNA	let-7f
t0081481	24	1	TGAGGTAGTAGCTTGTATAGTTAA	miRNA	let-7f
t0081542	22	1	TGAGGTAGTATATTGTAAAGTT	miRNA	let-7f
t0081662	19	1	GGAGGTAGCAGATTGTATA	miRNA	let-7f
t0081724	22	1	TGAGGTAGAAGTTTGTATAGTT	miRNA	let-7f
t0081734	23	1	ATGAGGTGGTAGATTGTATAGTT	miRNA	let-7f
t0081746	21	1	TGAGGTAGAAGATTGTATTGT	miRNA	let-7f
t0081797	21	1	TGAGGTCGTAGATTGTATAGC	miRNA	let-7f
t0081817	22	1	TGAGGTAATAGATTGTATGGTT	miRNA	let-7f
t0082045	21	1	TGAGGTAGCAGATTGTATAGG	miRNA	let-7f
t0082064	20	1	TGAGGTCGTAGCTTGTATAG	miRNA	let-7f
t0082095	22	1	ATGAGGTAGTAGATTGTATAGG	miRNA	let-7f
t0082259	22	1	TGAGGTGGTAGATTGTATATTT	miRNA	let-7f
t0082308	21	1	TGAGGTTGTAGATTGTATAGG	miRNA	let-7f
t0082340	22	1	TGAGGTAGTAGATTGTCCAGTT	miRNA	let-7f
t0082506	20	1	GAGGAAGTAGATTGGATAGT	miRNA	let-7f
t0082520	22	1	TGGGGTAGAAGATTGTATAGTT	miRNA	let-7f
t0082592	23	1	TGAGGTAATAGATTGTATAGTTT	miRNA	let-7f
t0082618	21	1	TGAGGTGGTAGATTGTATAGA	miRNA	let-7f
t0082825	22	1	TGAGGTAGTAGAGGGTATAGTT	miRNA	let-7f
t0082855	22	1	TGAGGTATTAGATTGTACAGTT	miRNA	let-7f
t0083017	22	1	TGAGGAAGTAGACTGTATAGTT	miRNA	let-7f
t0083035	25	1	TGAGGTAGTAGATTCTATAGTTATC	miRNA	let-7f
t0083077	22	1	TGAGGTAGTAGATTGTTTAGAT	miRNA	let-7f
t0083103	23	1	CTGAGGAAGTAGATTGTATAGTT	miRNA	let-7f
t0083121	22	1	TGAGGTAGTAGATTGTACAGTG	miRNA	let-7f
t0083145	19	1	TGAGGTAGTAGATCGTATT	miRNA	let-7f
t0083361	22	1	TGAGGTAGTAGATTGTTTATTT	miRNA	let-7f
t0083390	22	1	TGGGGTAGTAGATTGTATAGTA	miRNA	let-7f
t0083400	21	1	TGATGTAGTAGATTGTATCGT	miRNA	let-7f
t0083510	18	1	TGAGGTAGTAGATTGCAG	miRNA	let-7f
t0083544	23	1	TGAGGTAGTAAATTGTATAGTTT	miRNA	let-7f
t0083555	21	1	TAAGGTAGTAGATCGTATAGT	miRNA	let-7f
t0083574	22	1	TGGGGTAGCAGATTGTATAGTT	miRNA	let-7f
t0083772	28	1	TGAGGTAGTAGATTGTATAGTCGAACGG	miRNA	let-7f
t0083839	22	1	TTGAGGTAGTAGATTGTCTAGT	miRNA	let-7f
t0084001	22	1	CTGAGGCAGTAGATTGTATAGT	miRNA	let-7f
t0084160	22	1	GAGGCAGTAGATTGTATAGTTA	miRNA	let-7f
t0084335	22	1	TGAGGTAGTAGTTTGCATAGTT	miRNA	let-7f
t0084365	21	1	CGAGGTTGTAGATTGTATAGT	miRNA	let-7f
t0084465	26	1	TGAGGTAGTAGATTGTATAGTTAATC	miRNA	let-7f
t0084495	20	1	TAAGGAAGTAGATTGTATAG	miRNA	let-7f
t0084498	22	1	TGATGTAGTAGAATGTATAGTT	miRNA	let-7f
t0084568	22	1	CGAGGTAGTTGATTGTATAGTT	miRNA	let-7f

t0084619	21	1	TAAGGTAGTAGATTATATAGT	miRNA	let-7f
t0084697	21	1	TGTGGTAGTAGATTGTATCGT	miRNA	let-7f
t0085038	21	1	TGAGGTAGTAGATTGGATAGG	miRNA	let-7f
t0085334	20	1	AGGTAGCAGATTGTATAGTT	miRNA	let-7f
t0085364	22	1	TGGGGTAGTAGATTATATAGTT	miRNA	let-7f
t0085369	21	1	TAGGTAGTAGATTATATAGTT	miRNA	let-7f
t0085535	22	1	TGAGGTAGTAGCTTCTATAGTT	miRNA	let-7f
t0085571	22	1	TGAGGTAGAAGATTGTATCGTT	miRNA	let-7f
t0085751	20	1	GAGGCAGTAGATTGTATAGT	miRNA	let-7f
t0085842	44	1	TGAGGTAGTAGATTGTATAGTTTCGTTTG[illegible]	miRNA	let-7f
t0085912	22	1	TGATGTAGTAGATTTTATAGTT	miRNA	let-7f
t0086257	21	1	TGGGGTAGTAGATTGTATACT	miRNA	let-7f
t0086286	20	1	TGAGGTTGTAGATTGTATAT	miRNA	let-7f
t0086592	22	1	TGAGGTAGTAGTTTGGATAGTT	miRNA	let-7f
t0086597	22	1	GGAAGTAGTAGATTGTATAGTT	miRNA	let-7f
t0086628	21	1	TGAGGTAGTAGTCTGTATAGT	miRNA	let-7f
t0086691	20	1	TGGGGAAGTAGATTGTATAG	miRNA	let-7f
t0086721	22	1	TGTGGAAGTAGATTGTATAGTT	miRNA	let-7f
t0086727	22	1	TGAGATAGCAGATTGTATAGTT	miRNA	let-7f
t0086747	21	1	AAGGTAGTAGATTGTATAGTT	miRNA	let-7f
t0086827	22	1	TGAGGTAGTAGAGTGTATAGGT	miRNA	let-7f
t0086890	21	1	TTAAGGTAGTAGATTGTATAG	miRNA	let-7f
t0087001	21	1	GTTGAGGCAGTAGATTGTATA	miRNA	let-7f
t0087059	23	1	TGAGCTAGTAGATTGTATAGTTG	miRNA	let-7f
t0087152	22	1	TGAGGTCGTAGATTGTATAGCT	miRNA	let-7f
t0087198	21	1	TGAGGTAGTAGATTGTTTAGG	miRNA	let-7f
t0087264	22	1	CGAGGTAGTAGATTATATAGTT	miRNA	let-7f
t0087276	20	1	TTAGGTAGTAGATTGTCTAG	miRNA	let-7f
t0087282	22	1	GAGGTAGTAGATTGTATCGTTG	miRNA	let-7f
t0087321	21	1	TGAGGTAGTAGATTGTGTAGA	miRNA	let-7f
t0087436	22	1	TGTGATAGTAGATTGTATAGTT	miRNA	let-7f
t0087537	21	1	TTGAGGTAGTAGATTGTATAT	miRNA	let-7f
t0087546	21	1	ATAGGTAGTAGATTGTATAGT	miRNA	let-7f
t0087590	21	1	TTGAGGTATTAGATTGTATAG	miRNA	let-7f
t0087609	22	1	TGAGGTACTAGATTGTGTAGTT	miRNA	let-7f
t0087751	20	1	TAGGTAGTAGCTTGTATAGT	miRNA	let-7f
t0088026	22	1	TGCGGTAGTAGACTGTATAGTT	miRNA	let-7f
t0088070	22	1	TGAGGTAGAAGATTTTATAGTT	miRNA	let-7f
t0088096	22	1	TGAGGCAGAAGATTGTATAGTT	miRNA	let-7f
t0088107	19	1	TGAGCTAGTATATTGTATA	miRNA	let-7f
t0088113	22	1	CGAGGTAGTAGATTGTATAGCT	miRNA	let-7f
t0088243	21	1	TGAGGTAGTAGATTGTCTCGT	miRNA	let-7f
t0088322	21	1	TGAGGTAGTAGCTTGTATAGG	miRNA	let-7f
t0088376	22	1	TTGAGGTGGTAGATTGTATAGT	miRNA	let-7f
t0088476	22	1	TGTGGTAGTAGATTGTATAGAT	miRNA	let-7f
t0088514	21	1	TGAGGTAGTAGATGGGATAGT	miRNA	let-7f
t0088606	22	1	TGAGGTGGTAGATTGTTTAGTT	miRNA	let-7f
t0088750	22	1	TGAGGTAGTAGATTGCATAGCT	miRNA	let-7f
t0088949	22	1	TGAGGTAGTAGATTGGATAGAT	miRNA	let-7f
t0088980	21	1	TTGAGCTAGTAGATTGTATAG	miRNA	let-7f
t0089202	22	1	GAGGTAGTAGATTGGATAGTTA	miRNA	let-7f
t0089265	23	1	TGAGGTAGTAGATAGTATAGTTG	miRNA	let-7f
t0089328	22	1	TGAGGTTATAGATTGTATAGTT	miRNA	let-7f
t0089549	20	1	TGAGGAAGTAGATTATATAG	miRNA	let-7f
t0089602	22	1	TGAGGTAGTAGATTGTCTAGCT	miRNA	let-7f
t0089709	22	1	TGAGGTAGAAGATTGCATAGTT	miRNA	let-7f
t0089743	23	1	TGAGATAGTAGATTGTATAGTTG	miRNA	let-7f
t0089777	22	1	TGGGGTAGTAGACTGTATAGTT	miRNA	let-7f

t0089869	23	1	ATGAGGTAGAAGATTGTATAGTG	miRNA	let-7f
t0089952	21	1	TGATGTAGTAGATTGCATAGT	miRNA	let-7f
t0090031	22	1	TGAGGTCGTAGATTGTATAGGT	miRNA	let-7f
t0090073	21	1	TGAGGTAGTAGATCGTATAGC	miRNA	let-7f
t0090141	19	1	TGACGTAGTAGATTGTATA	miRNA	let-7f
t0090212	22	1	TGAGGTAGTAAATTTTATAGTT	miRNA	let-7f
t0090333	22	1	TGAGGTAGTATATTGTATAGTA	miRNA	let-7f
t0090545	22	1	TGAGCTAGTAGATTGTACAGTT	miRNA	let-7f
t0090580	23	1	ATGCGGTAGTAGATTGTATAGTT	miRNA	let-7f
t0091086	22	1	TGAGGTAGTAGACTATATAGTT	miRNA	let-7f
t0091093	19	1	CGAGGTAGTAGATTGTATT	miRNA	let-7f
t0091119	20	1	TTAGGTAGTAGATTGTAAAG	miRNA	let-7f
t0091209	22	1	TGAGGTAGTAGTTTGTTTAGTT	miRNA	let-7f
t0091363	21	1	GGTAGTAGATTGTATAGTTGG	miRNA	let-7f
t0091601	20	1	TGAGGAACTAGATTGTATAG	miRNA	let-7f
t0091697	23	1	TGAGGTAGTAGAATGTATAGTTT	miRNA	let-7f
t0091771	19	1	TGAGGTAGTAGATGGTATA	miRNA	let-7f
t0091940	22	1	TGAGTTAGTAGATTGTACAGTT	miRNA	let-7f
t0091959	22	1	TGCGGTAGTAGATTGTATGGTT	miRNA	let-7f
t0092000	23	1	AGAGGTAGTAGATTGTATAGTTT	miRNA	let-7f
t0092133	21	1	GAGGTTGTAGATTGTATAGTT	miRNA	let-7f
t0092179	20	1	TGAGGTAGGAGAATGTATAG	miRNA	let-7e
t0092325	20	1	TGAGGTAGGAGATTGGATAG	miRNA	let-7e
t0092354	22	1	TGAGGTAGGAGATCGTATAGTT	miRNA	let-7e
t0092596	21	1	TGAGGTAGGAGATTGAATAGT	miRNA	let-7e
t0092773	22	1	TGAGGTAGGAGATTCTATAGTT	miRNA	let-7e
t0092804	23	1	TGAGGTAGGAGATTGTATAGTAA	miRNA	let-7e
t0092900	22	1	TGAGGTTGGAGCTTGTATAGTT	miRNA	let-7e
t0093238	21	1	TGAGGTAGGAGATTGTATTGT	miRNA	let-7e
t0093267	21	1	TGAGGTAGGAGATTGGATAGT	miRNA	let-7e
t0093271	20	1	TGAGGAAGGAGATTGTATAG	miRNA	let-7e
t0093350	22	1	TGAGGGGGGAGATTGTATAGTT	miRNA	let-7e
t0093371	21	1	TGAGGTAGGAGATTGTACAGT	miRNA	let-7e
t0093553	20	1	TGAGGTAGGAGATCGTATAG	miRNA	let-7e
t0042320	22	1	TGAGGTAGGAGATTGTATCGTT	miRNA	let-7e
t0042427	20	1	TGAGGTAGGAGATTGTAGAT	miRNA	let-7e
t0043816	22	1	TGAGGTAGGAGTTTGTATAGTT	miRNA	let-7e
t0047399	20	1	TGCGGTAGGAGATTGTATAG	miRNA	let-7e
t0047615	22	1	TGAGGTAGGAGATTGTTTAGTT	miRNA	let-7e
t0049273	21	1	TGAGGGGGGAGATTGTATAGT	miRNA	let-7e
t0051913	21	1	TGAGGTAGGAGATTGTATAGG	miRNA	let-7e
t0054030	20	1	TGAGGTAGGAGATTGTACAG	miRNA	let-7e
t0055099	22	1	TGAGGTAGGAGATTGTATTGTT	miRNA	let-7e
t0056955	21	1	TGAGGTAGGAGTTTGTATCGT	miRNA	let-7e
t0058376	23	1	TGAGGTAGGAGATTGTATAGTGA	miRNA	let-7e
t0059429	20	1	TGAGGTAGGAGATTGTGTAG	miRNA	let-7e
t0060202	26	1	TGGGGTAGGAGGTTGTATAGTTGATC	miRNA	let-7e
t0063143	22	1	TGAGGTAGGGGATTGTATAGTT	miRNA	let-7e
t0063219	21	1	TGAGGCAGGAGATTGTATAGT	miRNA	let-7e
t0065012	21	1	GAGGTAGGAGATTGTATAGTT	miRNA	let-7e
t0066525	20	1	TGAGGTAGGAGATTGCATAG	miRNA	let-7e
t0066911	20	1	TTAGGTAGGAGATTGTATAG	miRNA	let-7e
t0068083	22	1	TTAGGTAGGAGATTGTATAGTT	miRNA	let-7e
t0068624	22	1	TGAAGTAGGAGATTGTATAGTT	miRNA	let-7e
t0069517	22	1	TGAGGTAGGAGATTGTATATTT	miRNA	let-7e
t0069528	25	1	TGAGGTAGGAGATTGAATAGTTATC	miRNA	let-7e
t0070230	22	1	TGAGGTAGGAGATTGGATAGTT	miRNA	let-7e
t0071028	22	1	TGAGGTAGGAAATTGTATAGTT	miRNA	let-7e

t0072197	22	1	TGAGGTAGGAGATTGTATACTT	miRNA	let-7e
t0072581	22	1	TGAGGCAGGAGATTGTATAGTT	miRNA	let-7e
t0074375	23	1	TTGAGGTAGGAGATTGTATAGTT	miRNA	let-7e
t0074777	20	1	TGAGGTAGGAGATTGAATAG	miRNA	let-7e
t0076032	21	1	TGAGGTAGGAGATTGTATACT	miRNA	let-7e
t0076384	20	1	AGGTAGGAGGTTGTATAGTT	miRNA	let-7e
t0078153	22	1	TGAGGTAGGAGATTGTAGAGTT	miRNA	let-7e
t0079352	20	1	CTATACGACCTGCTGACTTT	miRNA	let-7d*
t0079717	21	1	AAAGGAAGTAGGTTGCATAGT	miRNA	let-7d
t0080845	22	1	AGCGGTAGTAGGTTGCATCGTT	miRNA	let-7d
t0082307	22	1	AGAGGTAGTAGGATGCATGGTT	miRNA	let-7d
t0083335	19	1	AGAGGTACTAGGTTGCATA	miRNA	let-7d
t0083547	24	1	AGAGGTAGTAGGTTGCGTAGTTCT	miRNA	let-7d
t0084519	20	1	AGAGGTAGTAGGCTGCATAA	miRNA	let-7d
t0085523	20	1	AGAGGGAGGAGGTTGCATAG	miRNA	let-7d
t0088824	23	1	AGAGGTAGTAGGTTGCATACTTA	miRNA	let-7d
t0089621	21	1	AGAGGTAGTAGCTTGCATAGT	miRNA	let-7d
t0092510	22	1	AGAGGAAGTAGGTTGCATAGTA	miRNA	let-7d
t0092927	20	1	ATAGGTAGTGGGTTGCATAG	miRNA	let-7d
t0093232	19	1	AGAGGTAGTAGGTTGCAAA	miRNA	let-7d
t0091903	22	1	AGAGGTCGTATGTTGCATAGTT	miRNA	let-7d
t0040949	20	1	AGAGGTAGTAGGGTGCAAAG	miRNA	let-7d
t0041008	25	1	AGAGGTAGTAGGGTGCATAGTTAAA	miRNA	let-7d
t0041065	19	1	AGAGGTAATAGGTTGCATA	miRNA	let-7d
t0041191	23	1	AGAGGTAGTAGGTTGCATAGCTA	miRNA	let-7d
t0042373	23	1	AGAGGTAGTAGGTTGCTTAGTTA	miRNA	let-7d
t0042406	23	1	AGAGGTAGTAGGTTTCATAGTTA	miRNA	let-7d
t0042776	23	1	AGAGGTAGTAGGTTGCATAGATG	miRNA	let-7d
t0043163	20	1	AGAGCTAGTAGGTTGCATAG	miRNA	let-7d
t0044125	22	1	AGACGTGGTAGGTTGCATAGTT	miRNA	let-7d
t0044620	25	1	AGAGGTAGTAGGGTGCATAGTTATC	miRNA	let-7d
t0045941	25	1	AGAGGTAGTAGGTTGCATAGTTAAC	miRNA	let-7d
t0046400	22	1	AGAGGTAGTAGGTTGCATAGGG	miRNA	let-7d
t0046512	20	1	AGAGGTAGGAGGGTGCATAG	miRNA	let-7d
t0047066	22	1	AGAGGGAGTAGGATGCATAGTT	miRNA	let-7d
t0047099	22	1	AGAGGAAGTAGGTTGCATAGAT	miRNA	let-7d
t0048072	19	1	AGAGGTAGTAGGTTGCGTA	miRNA	let-7d
t0048420	20	1	AGACGTAGTAGGTTGCATAG	miRNA	let-7d
t0048566	20	1	ACAGGTAGTAGGTTGCATAG	miRNA	let-7d
t0048710	23	1	AGAGGTAGTAGGTTGTATAGTTT	miRNA	let-7d
t0049127	23	1	AGAGGTAGTAGGTTGCATAGTTC	miRNA	let-7d
t0049221	21	1	AGAGGTAGTAGGTGGCATAGT	miRNA	let-7d
t0049587	18	1	AGAGGTAGTAGGTCGCAT	miRNA	let-7d
t0050041	21	1	AGCGGTAGTAGGTTGAATAGT	miRNA	let-7d
t0050345	22	1	AGACGTAGTAGGTTGCATAGTT	miRNA	let-7d
t0050470	22	1	AGAGGGGGTAGGTTGCATAGTT	miRNA	let-7d
t0050874	21	1	AGAGGTAGTGGGTTGTATAGT	miRNA	let-7d
t0051419	22	1	AAGAGGTATTAGGTTGCATAGT	miRNA	let-7d
t0051636	22	1	AGAGGTAGTAGGTTGCGTAGTA	miRNA	let-7d
t0052584	20	1	AGAGGTAGTAGGTAGCATAG	miRNA	let-7d
t0052611	23	1	AGAGGTGGTAGGTTGCATAGTTG	miRNA	let-7d
t0053271	21	1	TAGAGGTAGTAGGTTGCCTAG	miRNA	let-7d
t0053837	20	1	AGAGGGAGTAGGGTGCATAG	miRNA	let-7d
t0054000	21	1	TAGAGGAAGTAGGTTGCATAG	miRNA	let-7d
t0054069	21	1	AGAGGTATTAGGTTGCATAGT	miRNA	let-7d
t0055754	22	1	AGAGGTAGTCGGTTGCATAGTA	miRNA	let-7d
t0055885	24	1	AGAGGTAGTAGGCTGCATAGTTAA	miRNA	let-7d
t0056093	22	1	AGGGGTAGTAGGTTGCATAGTA	miRNA	let-7d

t0056541	22	1	AGAGGTAGTAGGTTGCGTGGTT	miRNA	let-7d
t0057045	22	1	AGAGGTAGTAGGTTGCATAGAC	miRNA	let-7d
t0057506	22	1	AGCGGCAGTAGGTTGCATAGTT	miRNA	let-7d
t0057610	18	1	AGATGTAGTAGGTTGCAT	miRNA	let-7d
t0057937	20	1	AGAGGGAGAAGGTTGCATAG	miRNA	let-7d
t0058469	19	1	AGAGGTAGTAGGGTGCATA	miRNA	let-7d
t0059083	20	1	ATAGGTAGTAGGGTGCATAG	miRNA	let-7d
t0059097	19	1	AGAGGTAGTAGGCTGCATA	miRNA	let-7d
t0059250	20	1	AGAGGTAGTCGGTTGCATAG	miRNA	let-7d
t0059327	21	1	ACAGGTAGTAGTTTGCATAGT	miRNA	let-7d
t0059963	24	1	AGAGGTAGTAGGTTCCATAGTTAT	miRNA	let-7d
t0060339	21	1	AGAGGTAGTAGGTTGGATAGA	miRNA	let-7d
t0060344	19	1	AGAGGCAGTAGGTTGCATA	miRNA	let-7d
t0061496	21	1	AGAGGGAGTAGGTTGAATAGT	miRNA	let-7d
t0062010	21	1	TGGAGGTAGTAGGTTGCATAG	miRNA	let-7d
t0062155	21	1	AGAGGTAGTAGGTTGCATATT	miRNA	let-7d
t0062261	21	1	GAGGAAGTAGGTTGCATAGTT	miRNA	let-7d
t0062742	23	1	AGAGGTAGTAGGCTGCATAGTTT	miRNA	let-7d
t0062825	21	1	AGAGGGAGTAGGTTGCATAGA	miRNA	let-7d
t0063716	22	1	AGAGGTAGTAGGTTGCAAAGTA	miRNA	let-7d
t0064096	19	1	AGAGGTAGTAGGTTGCCTA	miRNA	let-7d
t0064221	22	1	AGAGGTGGTAGGTTGCATAGGT	miRNA	let-7d
t0064676	22	1	AGAGGGAGAAGGTTGCATAGTT	miRNA	let-7d
t0065952	21	1	AGAGCTAGTAGGTTGCATAGT	miRNA	let-7d
t0066480	23	1	AGAGGGAGTAGGTTGCATAGTTG	miRNA	let-7d
t0066689	19	1	AGAGGTAGTAGGTTGCATC	miRNA	let-7d
t0066774	24	1	AGAGGTAGTAGGTTGCGTAGTTAT	miRNA	let-7d
t0067137	22	1	AGAAGTAGTAGGTTGCATAGTA	miRNA	let-7d
t0067250	21	1	AGAGGTAGTGGGTTGCATAGA	miRNA	let-7d
t0067914	25	1	AGAGGTAGTAGGATGCATAGTTATC	miRNA	let-7d
t0068747	19	1	AGAGGTGGTAGGTTGCATA	miRNA	let-7d
t0070242	22	1	AGAGGTAGTAGGTGGCATAGTT	miRNA	let-7d
t0070813	20	1	AGAGGTAGTAGTTTGCGTAG	miRNA	let-7d
t0071170	23	1	AGAGGTAGTAGGGTGCATAGTTG	miRNA	let-7d
t0071466	22	1	AGAGGTAGTAGGATGCATAGTA	miRNA	let-7d
t0071547	19	1	AGAGGTAGTGGGTTGCATA	miRNA	let-7d
t0071558	20	1	GAGGCAGTAGGTTGCATAGT	miRNA	let-7d
t0072250	22	1	AGCGGTAGTCGGTTGCATAGTT	miRNA	let-7d
t0072263	19	1	AGAGGTAGTAGGTTACATA	miRNA	let-7d
t0073362	20	1	AGAGGTAGTAGGTTGCATAC	miRNA	let-7d
t0074663	20	1	AAAGGTAGTAGGTTGCATAG	miRNA	let-7d
t0075293	20	1	AGAGGTAGTAGGTTGCATAT	miRNA	let-7d
t0075715	23	1	AGAGGTAGTAGGTTACATAGTTG	miRNA	let-7d
t0076174	22	1	ACAGGTAGTAGGGTGCATAGTT	miRNA	let-7d
t0077213	23	1	AGAGGTAGTAGGTTGCGTAGTTG	miRNA	let-7d
t0077339	20	1	AGAGGGGGTAGGTTGCATAG	miRNA	let-7d
t0077472	21	1	GGTAGTAGGTTGCATAGTTGA	miRNA	let-7d
t0077697	24	1	AGAGGGAGTAGGTTGCATAGTTTT	miRNA	let-7d
t0078008	21	1	AGAGGTAGTAGGGTGCACAGT	miRNA	let-7d
t0078052	21	1	AGAGGTTGTAGGTTGCATAGT	miRNA	let-7d
t0078660	22	1	AGAGGGAGTAGGTTGCATAGTA	miRNA	let-7d
t0078978	21	1	AGAGGAAGTAGGTTGCATAAT	miRNA	let-7d
t0081219	25	1	AGAGGCAGTAGGTTGCATAGTTATC	miRNA	let-7d
t0082041	23	1	AGAGGTAGTAGGTTGCATAGGTA	miRNA	let-7d
t0082302	22	1	AGAGGTAGCAGGTTGCATAGTA	miRNA	let-7d
t0082954	21	1	AGAGGTAGCAGGTTGTATAGT	miRNA	let-7d
t0083722	22	1	AGAGGTAGGAGGGTGCATAGTT	miRNA	let-7d
t0083928	23	1	AGACGTAGTAGGTTGCATAGTTG	miRNA	let-7d

t0084731	26	1	AAGAGGTAGTAGGATGCATAGTAATT	miRNA	let-7d
t0085199	23	1	AGAGGTAGTAGGCTGCATAGTTG	miRNA	let-7d
t0085242	22	1	AGAGGGAGGAGGTTGCATAGTT	miRNA	let-7d
t0086515	22	1	AGAGGTAGTAGGATGCATAGGT	miRNA	let-7d
t0087231	21	1	AGAGGTAGTAGGATGTATAGT	miRNA	let-7d
t0088254	23	1	AGAGGTAGTAGGTTGCGTAGTTT	miRNA	let-7d
t0088360	21	1	AGAGGAAGTAGGTTCCATAGT	miRNA	let-7d
t0089305	25	1	TGAGGTAGTAGGCTGTATGGTTATC	miRNA	let-7c
t0090046	22	1	TGAGGTAGTAGGTTGTATGGAT	miRNA	let-7c
t0090191	22	1	TGAGGTAGTTGGTTATATGGTT	miRNA	let-7c
t0090234	25	1	TGAGGTAGTAGGTTGAATGGTTATC	miRNA	let-7c
t0090566	22	1	TGAGGTAGTAGGTTGTATGGGT	miRNA	let-7c
t0090734	25	1	TGAGGTTGTAGGTTGTATGGTTATC	miRNA	let-7c
t0090811	21	1	TGAGGTAGTAGGTTGTATGGA	miRNA	let-7c
t0091290	25	1	TGAGGTAGTAGGTTCTATGGTTATC	miRNA	let-7c
t0091773	25	1	TGAGGCAGTAGGTTGTATGGTTATC	miRNA	let-7c
t0092296	22	1	TTAGGTAGTAGGTTGTATGGTT	miRNA	let-7c
t0092867	20	1	TGCGGTAGTAGGTTGTATGG	miRNA	let-7c
t0093158	25	1	TGAGGTAGTAGGTTGTACGGTTATC	miRNA	let-7c
t0093310	22	1	TGAGGTGGTTGGTTGTATGGTT	miRNA	let-7c
t0041693	21	1	TGAGGTAGTAGGTTGCATGGT	miRNA	let-7c
t0042691	21	1	TGGGGTAGTAGGTTGTATGGT	miRNA	let-7c
t0044087	25	1	TGAGGTAGTAGGTCGTATGGTTATC	miRNA	let-7c
t0045644	22	1	TGCGGTAGTAGGTTGTATGGTT	miRNA	let-7c
t0045793	22	1	TGAGGTAGTTGGTTGTATGGTT	miRNA	let-7c
t0048245	21	1	TGAGATAGTAGGTTGTATGTT	miRNA	let-7c
t0048744	22	1	TGAGGTAGTAGGTTGTATGTTT	miRNA	let-7c
t0050015	22	1	TGAGGTGGTGGGTTGTATGGTT	miRNA	let-7c
t0052347	19	1	TGAGGTAGTAGGATGTATG	miRNA	let-7c
t0052792	25	1	TGAGGTAGGAGGTTGTATGGTTATC	miRNA	let-7c
t0052820	22	1	TGAGGTGGTAGGTTGTATGGTT	miRNA	let-7c
t0053360	22	1	TGAGGTAATAGGTTGTATGGTT	miRNA	let-7c
t0054455	22	1	TGAGGTAGTATGTTGTATGGTT	miRNA	let-7c
t0054587	20	1	TGAGGTAGTCGGTTGTATGG	miRNA	let-7c
t0059103	25	1	TTAGGTAGTAGGTTGTATGGTTATC	miRNA	let-7c
t0059761	20	1	TGAGGTAGTAGGGTGTATGG	miRNA	let-7c
t0061262	21	1	TGAGGTAGTAGGTTGTAAGGT	miRNA	let-7c
t0061924	24	1	GAGGTAGTAGGTTGTATGGTTATC	miRNA	let-7c
t0063120	25	1	TGACGTAGTAGGTTGTATGGTTATC	miRNA	let-7c
t0063874	25	1	TGAGGTAGTCGGTTGTATGGTTATC	miRNA	let-7c
t0066862	22	1	TGAGGCAGTAGGTTGTATGGTT	miRNA	let-7c
t0067238	20	1	TGAGGTAGTAGCTTGTATGT	miRNA	let-7c
t0067407	20	1	TCAGGTAGTAGGTTGTATGG	miRNA	let-7c
t0069680	21	1	TGAGGTAGTAGTTTGTATGGT	miRNA	let-7c
t0070383	20	1	TGAGGTAGTAGGTTATATGG	miRNA	let-7c
t0071441	19	1	CTATACAACCTGCTGCCTT	miRNA	let-7b*
t0073950	19	1	CTATGCAACCTACTGCCTT	miRNA	let-7b*
t0076994	20	1	CTATACAACATACTGCCTTC	miRNA	let-7b*
t0078376	21	1	CTATACAACATACTGCCTTCC	miRNA	let-7b*
t0078864	21	1	TGGGGTAGTGGGTTGTGTGGT	miRNA	let-7b
t0078892	23	1	TGAGGTAGTAGATTGTGTGGTAT	miRNA	let-7b
t0080296	21	1	TGAGGTATTAGGTTGTGCGGT	miRNA	let-7b
t0080789	26	1	TGAGGTAGTGGGTTGTGTGGTAAGAA	miRNA	let-7b
t0081075	21	1	TGAGGTAGTAGGCTGAGTGGT	miRNA	let-7b
t0083981	21	1	TGAGGCAGTAGGTTGTGTGGA	miRNA	let-7b
t0084457	24	1	TGAGGAAGTAGGTAGTGTGGTTAA	miRNA	let-7b
t0090918	21	1	TGAGGTAGTAGGTCGTGTAGT	miRNA	let-7b
t0092104	22	1	TGAGGTGGTCGGTTGTGTGGTT	miRNA	let-7b

t0055167	22	1	TGAGGTAGTAGGTTGTTTGGAT	miRNA	let-7b
t0060825	22	1	TGAGGTAGTAGGTTGTCCGGTT	miRNA	let-7b
t0065460	22	1	TGAGGTGGTAGGTTGTGGGGTT	miRNA	let-7b
t0076170	21	1	TGAGGTAGTGGGTTGTGGGGT	miRNA	let-7b
t0040696	18	1	TGCGGTAGTAGGTTGTGT	miRNA	let-7b
t0040719	23	1	TGACGTAGTAGGTTGTGTGGTTA	miRNA	let-7b
t0040822	22	1	TGAGGTAGTAGGTTCTGTGATT	miRNA	let-7b
t0040823	25	1	TGAGGTAGTAGGTTGTGTGGTTATT	miRNA	let-7b
t0040845	23	1	TGAGGTAGCAGGTTGTGTGGTTG	miRNA	let-7b
t0040875	22	1	TGAGATAGTAGGTTGTGTGGAT	miRNA	let-7b
t0041155	22	1	TGAGGTAGTAGATTGTGAGGTT	miRNA	let-7b
t0041237	22	1	TGAGATAGTAGGTTGTGTGGTA	miRNA	let-7b
t0041642	23	1	TGAGGTAGTAAGTTGTGTGGTTG	miRNA	let-7b
t0041730	22	1	TGAGGTAGTATGTTGTGTGGTA	miRNA	let-7b
t0041853	22	1	TGAGGTAGTAGGTTGTGTGCAT	miRNA	let-7b
t0041873	24	1	TGAGGTAGTAGATTGTGTGGTTAT	miRNA	let-7b
t0041997	24	1	TGAGGCAGTAGGTTGTGTGGTTAG	miRNA	let-7b
t0042057	22	1	TAAGGTAGTAGGTCGTGTGGTT	miRNA	let-7b
t0042065	22	1	TGAGGTAGTATATTGTGTGGTT	miRNA	let-7b
t0042099	24	1	TGAGGTAGTAGGTTGTGAGGTTAA	miRNA	let-7b
t0042394	24	1	TGAGGTAGAAGGTTGTGTGGTTGA	miRNA	let-7b
t0042415	22	1	TGAGGTAGTAGGTTGTGCGTTT	miRNA	let-7b
t0042428	21	1	TGAGGTAGTAGGATGTGTGTT	miRNA	let-7b
t0042501	25	1	TGAGGCAGTAGGTTGTGTGGTTAGA	miRNA	let-7b
t0042556	21	1	GAGGTAGAAGGTTGTGTGGTT	miRNA	let-7b
t0042839	20	1	TGAGGCAGTAGGTTGTGTGA	miRNA	let-7b
t0042921	22	1	TGAGCTAGTAGGTTGTGTGGTA	miRNA	let-7b
t0043049	24	1	TGAGGTAGTAGGTTGTGTGGGTTT	miRNA	let-7b
t0043079	25	1	TGAGGTAGTAGGTTGTGTGGGGAGT	miRNA	let-7b
t0043188	23	1	TGAGGTAGTAGGCTGTGTGGTTG	miRNA	let-7b
t0043479	22	1	TGAGGTACTAGGGTGTGTGGTT	miRNA	let-7b
t0043529	44	1	TGAGGTGGTTGGTTGTGTGGTTGGTTTGG(	miRNA	let-7b
t0043536	22	1	TGAGGTAGTAGGTTGTGCGGTC	miRNA	let-7b
t0043624	23	1	TGAGGAAGTAGGTTGTGTGGTAT	miRNA	let-7b
t0043733	22	1	TAAGGTAGTAGGTTATGTGGTT	miRNA	let-7b
t0043778	42	1	TGAGGTAGTGGGTTGTGTGGTTTCGTTTG(	miRNA	let-7b
t0043814	21	1	TGAGGTAGTATGTTGTGTGTT	miRNA	let-7b
t0043931	23	1	TGACGTAGTAGGTTGTGTGGTTT	miRNA	let-7b
t0043959	23	1	TGAGGTAGTAGGTAGTGTGGTTA	miRNA	let-7b
t0044240	23	1	TAAGGTAGTAGGTTGTGTGGTAA	miRNA	let-7b
t0044342	22	1	TGAGGTCGTAGGTTGTGTGTTT	miRNA	let-7b
t0044667	25	1	TGAGGAAGTAGGTTGTGTGGTTAGT	miRNA	let-7b
t0044713	22	1	TGAGGTAGTAGGTTGTGTCGTA	miRNA	let-7b
t0045208	22	1	TGAGGTAGTTGGTTGTGGGGTT	miRNA	let-7b
t0045275	23	1	TGTGGTAGTAGGTTGTGTGGTAT	miRNA	let-7b
t0045288	22	1	TGCGGTAGTAGGTTGGGTGGTT	miRNA	let-7b
t0045353	22	1	TGAGGTAGGAGGTTGTGTGGGT	miRNA	let-7b
t0045386	24	1	TGGGGTAGTAGGTTGTGTGGTTTA	miRNA	let-7b
t0045387	23	1	AGAGGTAGTAGGTTGTGTGGTTG	miRNA	let-7b
t0045454	22	1	TGAGGTCGTAGGTTGTGTGGGT	miRNA	let-7b
t0045520	21	1	TGAGGTAGTTGGTTGTGTGTT	miRNA	let-7b
t0045666	23	1	TGAGGTAGAAGGTTGTGTGGTAT	miRNA	let-7b
t0045709	22	1	TGAGGGCGTAGGTTGTGTGGTT	miRNA	let-7b
t0045719	25	1	TGAGGTAGTTGGTTGTGTGGTTATC	miRNA	let-7b
t0045815	22	1	TGAGGTAGTAGGTTCTGTGGTA	miRNA	let-7b
t0045831	44	1	TGAGGTAGTAGGTTGTGTGGTTTGGTTTG(	miRNA	let-7b
t0045837	20	1	TGAGGTAGTAGGTTGTCTTG	miRNA	let-7b
t0045855	25	1	TGAGGTAGTAGGTCGTGTGGTTATT	miRNA	let-7b

t0046214	24	1	TAAGGTAGTAGGTTGTGTGGTTAT	miRNA	let-7b
t0046268	26	1	TGAGGTCGTAGGTTGTGTGGTTAGAT	miRNA	let-7b
t0046342	21	1	TGAGGAAGTAGGTTGTGTAGT	miRNA	let-7b
t0046394	24	1	TGAGGTAGTAGGTTGTTTGGTTAT	miRNA	let-7b
t0046482	24	1	TGAGGTAGTAGGTTGTGCGGTTAT	miRNA	let-7b
t0046533	25	1	TGAGGTAGCAGGTTGTGTGGTTATC	miRNA	let-7b
t0046633	24	1	TGAGCTAGTAGGTTGTGTGGTTAA	miRNA	let-7b
t0046763	19	1	TGAGGTAGTAGGTTGCGTG	miRNA	let-7b
t0046777	24	1	TGAGGTAGTAGGTTGTATGGTTTC	miRNA	let-7b
t0046785	21	1	TGAGGTACCAGGTTGTGTGGT	miRNA	let-7b
t0046887	22	1	TGAGGTAGTAGTTTGTCTGGTT	miRNA	let-7b
t0046926	24	1	TGAGGTAGTAGGTTGTGTGGTTCT	miRNA	let-7b
t0047031	21	1	TGAGGTAGTAGGGTGTGTGGA	miRNA	let-7b
t0047265	24	1	TGAGGTAGTAGGATGTGTGGTTAA	miRNA	let-7b
t0047397	21	1	TGAGGTAGTAGGTTGTGGGGG	miRNA	let-7b
t0047478	22	1	TGAGGTAGCAGGTTGTGTGGGT	miRNA	let-7b
t0047558	21	1	TGAGGTAGTGGGTTGTTTGGT	miRNA	let-7b
t0047694	25	1	TGAGGTAGTAGGGTGTGTGGTTAGA	miRNA	let-7b
t0047809	24	1	TGAGGTACTAGGTTGTGTGGTTAT	miRNA	let-7b
t0047811	19	1	TGAGGTAGTAGGTTTTGTG	miRNA	let-7b
t0047831	42	1	TGAGGTGGTAGGTTGTGTGGTTTTGTTTG(	miRNA	let-7b
t0048018	22	1	TAAGGTAGTAGGTTGTGCGGTT	miRNA	let-7b
t0048129	20	1	TGAGGTAGTAGGTAGTGTGG	miRNA	let-7b
t0048474	21	1	TGCGGCAGTAGGTTGTGTGGT	miRNA	let-7b
t0048607	22	1	TAAGGTAGTAGGTTGTGAGGTT	miRNA	let-7b
t0048642	24	1	TGAGGTAGTAGGTTGTGTGATTGA	miRNA	let-7b
t0048656	21	1	TGAGGTACTAGGTTGTGGGGT	miRNA	let-7b
t0049057	21	1	TGAGATAGTAGGGTGTGTGGT	miRNA	let-7b
t0049084	22	1	TGTGGTAGTCGGTTGTGTGGTT	miRNA	let-7b
t0049238	21	1	TGACGTAGTGGGTTGTGTGGT	miRNA	let-7b
t0049556	21	1	TGAGCTAGAAGGTTGTGTGGT	miRNA	let-7b
t0049575	23	1	TAAGGTAGTAGGTTGTGTGGTAT	miRNA	let-7b
t0049609	21	1	TGGGGTAGTAGATTGTGTGGT	miRNA	let-7b
t0049666	25	1	TGAGGTAGTAGGTTGTGTGGTTGTC	miRNA	let-7b
t0049679	23	1	TGAGGTAGTGGGTTGTGTGGTTG	miRNA	let-7b
t0049758	22	1	TGAGGTAGTAGGTTATGTAGTT	miRNA	let-7b
t0049816	21	1	TGAGGCAGTAGGTTGTGTGTT	miRNA	let-7b
t0049886	25	1	TGAGGTAGTAGGTTGTGTGGTTGGA	miRNA	let-7b
t0050066	24	1	TGAGGTAGTAGGTTGTGTGGCTAA	miRNA	let-7b
t0050458	24	1	TGAGGTAGTAGATTGTGTGGTTAA	miRNA	let-7b
t0050667	24	1	TGAGGAAGTAGGTTGTGTGGTTAG	miRNA	let-7b
t0050683	22	1	TGAGGTAGTAGGTTGTTTGGTA	miRNA	let-7b
t0050703	22	1	TAAGGAAGTAGGTTGTGTGGTT	miRNA	let-7b
t0050963	22	1	TGAGGTAGTTGGTTGTGTGGGT	miRNA	let-7b
t0051136	25	1	TGAGGTAGTAGGTTGTATGGTTGTC	miRNA	let-7b
t0051149	24	1	TGAGGTAGTAGGTTGCGTGGTTAG	miRNA	let-7b
t0051325	21	1	TGAGGTAGTAGGTTGTGCGGA	miRNA	let-7b
t0051887	19	1	TGAGGTAGTACGTTGTGTG	miRNA	let-7b
t0051970	23	1	TGAGGTAGTAGGTTGCGTGGTAG	miRNA	let-7b
t0052010	22	1	TGTGGTAGCAGGTTGTGTGGTT	miRNA	let-7b
t0052063	23	1	TGAGGTAGTCGGTTGTGTGGTAT	miRNA	let-7b
t0052071	21	1	TGAGGTAGTAGGTTGCGTGGA	miRNA	let-7b
t0052165	42	1	TGAGGTAGTAGGTTGTGTGGTTTGTGTGT(	miRNA	let-7b
t0052253	22	1	ATGAGGTAGTAGGTTGTGTGGT	miRNA	let-7b
t0052595	42	1	TGAGGTAGTAGGTTGTGTGGTTTGTTTGG(	miRNA	let-7b
t0052833	22	1	TGTGGTAGTTGGTTGTGTGGTT	miRNA	let-7b
t0052880	23	1	CGAGGTAGTAGGTTGTGTGGATC	miRNA	let-7b
t0052891	21	1	TGAGGCAGTAGGTAGTGTGGT	miRNA	let-7b

t0052943	22	1	TGGGGTAGTAGGTTGTGTGTTT	miRNA	let-7b
t0052958	24	1	TGAGGTAGTAGGTTGTGTAGTTAG	miRNA	let-7b
t0053129	19	1	TGAGGTAGTTGGTTGTGTG	miRNA	let-7b
t0053234	23	1	TGAGGTAGTAGGTTATGTGGTTG	miRNA	let-7b
t0053371	22	1	TGAGGTAGTAGGGTGTGCGGTT	miRNA	let-7b
t0053435	21	1	TGAGGCTGTAGGTTGTGTGGT	miRNA	let-7b
t0053598	19	1	GAGGTAGTAGGTTGTGTGG	miRNA	let-7b
t0053654	23	1	TGAGGTAGTCGGTTGTGTGGTTG	miRNA	let-7b
t0053775	19	1	TGAGGTAGTAGGTTGCGTT	miRNA	let-7b
t0053778	20	1	TGAGGTAGTAGGGTGTGTAG	miRNA	let-7b
t0053805	24	1	TGAGGTAGTAGGTTGTGTGGATAA	miRNA	let-7b
t0053846	24	1	TGAGGTAGTAGGTTGTGTGGTAGG	miRNA	let-7b
t0053855	23	1	TGGGGTAGTAGGTTGTGTGGTTG	miRNA	let-7b
t0054498	22	1	TGGGGAAGTAGGTTGTGTGGTT	miRNA	let-7b
t0054582	22	1	TGAGTTAGCAGGTTGTGTGGTT	miRNA	let-7b
t0054690	23	1	TGAGGTAGTAGGTTGTGTGGCTC	miRNA	let-7b
t0054744	22	1	TGAGGTACTAGGTTGTGTGGTA	miRNA	let-7b
t0054991	18	1	TGTAGTAGGTTGCGTGGT	miRNA	let-7b
t0055004	24	1	CTGAGGAAGTAGGTTGTGTGGTTA	miRNA	let-7b
t0055232	21	1	TGAGGTAGTAGGCCGTGTGGT	miRNA	let-7b
t0055233	22	1	TGAGGTAGTAGCTTTTGTGGTT	miRNA	let-7b
t0055305	21	1	TGAGGTAGTAGGGTGTGTAGT	miRNA	let-7b
t0055330	22	1	TGAGGTAGTAGGTTGTGCGGTG	miRNA	let-7b
t0055362	24	1	TGAGGTAGTAGGTTGTGAGGTAAA	miRNA	let-7b
t0055437	21	1	TTAGGTAGTAGGGTGTGTGGT	miRNA	let-7b
t0055477	21	1	TGAGGTAGTAGGTCGTGTGTT	miRNA	let-7b
t0055516	22	1	TGAGGAAGTAGGTTGTGTGGGT	miRNA	let-7b
t0055522	26	1	TGAGGTAGTAGGTTGTGCGGTTTATC	miRNA	let-7b
t0055581	22	1	TGAGGTAGTAGGTTGTCTCGTT	miRNA	let-7b
t0055721	23	1	TGAGGTAGTAGGTTGTGTGATTG	miRNA	let-7b
t0055802	23	1	TGAGGTTGTAGGTTGTGTGGTTT	miRNA	let-7b
t0056184	22	1	TGAGGAAGTAGTTTGTGTGGTT	miRNA	let-7b
t0056397	21	1	TGGGGTAGTAGGTTGTCTGGT	miRNA	let-7b
t0056539	22	1	TGAGGTAGTAGGGTATGTGGTT	miRNA	let-7b
t0056703	22	1	TGAGGTAGTAGATTGTGGGGTT	miRNA	let-7b
t0056765	21	1	TGAGGTAGTAGGTTGTGGAGT	miRNA	let-7b
t0056969	24	1	TGAGGTAGTAGGTTGTATGGTTGA	miRNA	let-7b
t0057051	24	1	TGAGGTAGTGGGTTGTGTGGTTAG	miRNA	let-7b
t0057159	23	1	TGAGGTAGTAGGTTGTGAGGTAA	miRNA	let-7b
t0057502	24	1	TGAGGAAGTAGGTTGTGTGGTTGA	miRNA	let-7b
t0057739	22	1	TGAGGTCGTAGGTTGTGTGGTA	miRNA	let-7b
t0057781	19	1	TGAGGTAGTGGGTTGTGTA	miRNA	let-7b
t0057966	23	1	CGAGGTAGTAGGTTGTGTGGTAA	miRNA	let-7b
t0058527	21	1	CGAGGTAGTAGGTTGTGTTGT	miRNA	let-7b
t0058563	22	1	TGAGATAGTAGGTAGTGTGGTT	miRNA	let-7b
t0058741	22	1	TGGGGTAGTAGGTTGTGTAGTT	miRNA	let-7b
t0058789	22	1	TGAGGCAGTAGGTTGTGTGATT	miRNA	let-7b
t0058807	24	1	TGGGGTAGTAGGTTGTGGGGTTAT	miRNA	let-7b
t0058882	23	1	TGAGGTAGTAGGTTCTGTGGTAA	miRNA	let-7b
t0058946	21	1	TGAGGTAGTAGCTTGTGTGGC	miRNA	let-7b
t0058978	21	1	TGAGGTCGTAGGTTGTGGGGT	miRNA	let-7b
t0058995	22	1	TGAGGTATTAGGGTGTGTGGTT	miRNA	let-7b
t0059038	22	1	TGAGGTAGTAGGTTGTGGGGTG	miRNA	let-7b
t0059069	22	1	TGAGGTAGTAGCTTGTGTGGGT	miRNA	let-7b
t0059081	41	1	TGAGGTAGTTGGTTGTGTAGTTTGGTTTG(	miRNA	let-7b
t0059331	21	1	TGACGTAGTAGGTTGTGTAGT	miRNA	let-7b
t0059347	24	1	TGAGGTAATAGGTTGTGTGGTTAT	miRNA	let-7b
t0059540	22	1	TGAGGTACTAGGTTGGGTGGTT	miRNA	let-7b

t0059631	21	1	TGAGCTAGTAGTTTGTGTGGT	miRNA	let-7b
t0059745	23	1	TGAGGTAGTAAGTTGTGTGGTTT	miRNA	let-7b
t0059818	23	1	TGTGGTAGTAGGTTGTGTGGTTA	miRNA	let-7b
t0059895	23	1	TGAGCTAGTAGGTTGTGTGGTTG	miRNA	let-7b
t0060144	23	1	TGAGGTAGTAGGTCGTGTGGTGA	miRNA	let-7b
t0060207	23	1	TGAGATATTAGGTTGTGTGGTTA	miRNA	let-7b
t0060242	23	1	TGAGGTAGTAGGTTGTGTGGTGC	miRNA	let-7b
t0060273	22	1	TGAGGTAGTAGTTTGTGTGATT	miRNA	let-7b
t0060350	23	1	TGAGGTAGTAGTTTGTGTGGTAT	miRNA	let-7b
t0060414	21	1	TGAGGTGGTAGTTTGTGTGGT	miRNA	let-7b
t0060457	21	1	TGAGGCAGTAGGTTGTGTAGT	miRNA	let-7b
t0060494	44	1	TGAGGTAGTGGGTTGTGTGGTTTGGTTTG(	miRNA	let-7b
t0060500	20	1	TGAGGTAGTAGGCTGTGGGG	miRNA	let-7b
t0060631	24	1	TGAGGTAGGAGGTTGTGTGGTATC	miRNA	let-7b
t0060636	21	1	TGAGGTAGTATGTTGTGTAGT	miRNA	let-7b
t0060973	22	1	TGAGGTACTAGGTTGCGTGGTT	miRNA	let-7b
t0060982	21	1	TCAGGTAGTATGTTGTGTGGT	miRNA	let-7b
t0061140	24	1	TGTGGTAGTAGGTTGTGTGGTTAT	miRNA	let-7b
t0061234	23	1	TGAGGAAGTAGGTTGTGTGGTAG	miRNA	let-7b
t0061303	25	1	TGAAGTAGTAGGTTGTGTGGTAGTC	miRNA	let-7b
t0061426	22	1	TGAGGTAGTAGGGGGTGTGGTT	miRNA	let-7b
t0061628	23	1	TGAGGTAGTAGGTTGTGTGGGAA	miRNA	let-7b
t0061633	25	1	TGAGGTAGTAGGTTGTGTGGTCATC	miRNA	let-7b
t0061702	24	1	TGAGGTAGTAGGTTGTGTGGCTAG	miRNA	let-7b
t0061795	22	1	AGAGGTGGTAGGTTGTGTGGTT	miRNA	let-7b
t0062036	18	1	GGAGGTAGTAGGTTGTGT	miRNA	let-7b
t0062047	22	1	TGAGGTCGTGGGTTGTGTGGTT	miRNA	let-7b
t0062108	21	1	TGAGGTAGTAAGTTGTGTGGA	miRNA	let-7b
t0062208	24	1	TGAGGTAGTAGGTTGTGTGGTTCG	miRNA	let-7b
t0062212	21	1	TGATCTAGTAGGTTGTGTGGT	miRNA	let-7b
t0062405	25	1	TGAGGTAGTAGGTTGTGTGGTTAGG	miRNA	let-7b
t0062541	23	1	TGAGCTAGTAGGTTGTGTGGTAT	miRNA	let-7b
t0062641	25	1	TGAGGTAGTAGGTTGTGTGGATATC	miRNA	let-7b
t0062812	22	1	TGAGGTAGTGGGTTATGTGGTT	miRNA	let-7b
t0062856	18	1	TGAGGTAGTAGGTTGTCT	miRNA	let-7b
t0063029	21	1	TGCGGTAGTAGGTTGTGGGGT	miRNA	let-7b
t0063303	21	1	TGAGGTTGTAGGTTGGGTGGT	miRNA	let-7b
t0063326	24	1	TGAGGTAGTAGGTAGTGTGGTTAA	miRNA	let-7b
t0063461	22	1	TGAGGTAGTAGGTGGTGTGGTC	miRNA	let-7b
t0063533	22	1	TGAGCTAGTAGGGTGTGTGGTT	miRNA	let-7b
t0063728	23	1	TGTGGTAGTAGGTTGTGTGGTTT	miRNA	let-7b
t0063888	24	1	TGAGGTACTAGGTTGTGTGGTTAA	miRNA	let-7b
t0064095	22	1	TGAGGTAGTAGGCCGTGTGGTT	miRNA	let-7b
t0064210	23	1	TGAGGTAATAGGTTGTGTGGTAA	miRNA	let-7b
t0064289	22	1	TGAGGTATTAGGTTGTGCGGTT	miRNA	let-7b
t0064351	22	1	TGGGGTAGTAGGGTGTGTGGTT	miRNA	let-7b
t0064352	23	1	TGAGGTAGTAGGGTGTGCGGTTT	miRNA	let-7b
t0064509	21	1	TGAGGTAGTAGGGTGTCTGGT	miRNA	let-7b
t0064543	22	1	TGAGTTAGTAGGTTGTGCGGTT	miRNA	let-7b
t0064662	24	1	GGAGGTAGTAGGTTGTGTGGTTAT	miRNA	let-7b
t0065109	24	1	TGACGTAGTAGGTTGTGTGGTTAA	miRNA	let-7b
t0065206	21	1	TGAGGTAGTAGGTTGCTTGGT	miRNA	let-7b
t0065214	25	1	TGCGGTAGTAGGTTGTGTGGTGAGT	miRNA	let-7b
t0065288	23	1	TGAGGTAGCAGGTTGTGTGGTAA	miRNA	let-7b
t0065609	25	1	TGAGGTAGTAGGTTGTGCGGTTATC	miRNA	let-7b
t0065702	25	1	TGAGGTAGTAGGTTGTGGGGTTATC	miRNA	let-7b
t0065751	20	1	TGGGTAGTAGGTTGTGTGGT	miRNA	let-7b
t0066011	22	1	TGAGGTAGTAGGTTGTGGGGGT	miRNA	let-7b

t0066085	24	1	GTTGAGGTAGTAGGATGTGTGGTT	miRNA	let-7b
t0066117	22	1	TGGGGCAGTAGGTTGTGTGGTT	miRNA	let-7b
t0066128	23	1	TGAGGTAGTAGGTTATGTGGTAT	miRNA	let-7b
t0066518	25	1	TGAGGTAGTAGGCTGTGTGGTTAGA	miRNA	let-7b
t0066646	23	1	TGAGGTAGTAGGTTGTGTGGAGC	miRNA	let-7b
t0066746	20	1	TGGGGTAGTAGGTTGTGTAG	miRNA	let-7b
t0066936	22	1	CGAGGTAGTAGGTTGTGTAGTT	miRNA	let-7b
t0067185	26	1	TGAGGTAGTAGGTTGTATGGGTAATC	miRNA	let-7b
t0067507	24	1	TGAGGTAGTAGGATGTGTGGTATC	miRNA	let-7b
t0067536	23	1	TGAGGTAGTAAGTTGTGTGGTAA	miRNA	let-7b
t0067577	23	1	CGAGGTAGTAGGTTGTGTGGTAG	miRNA	let-7b
t0067833	24	1	TGAGGTAGTAGGTTGTGTGGGAAA	miRNA	let-7b
t0068108	22	1	TGCGGTAGTAGGTTGTGGGGTT	miRNA	let-7b
t0068178	21	1	TGAGGCAGTTGGTTGTGTGGT	miRNA	let-7b
t0068185	22	1	TGAGATAGTAGGGTGTGTGGTT	miRNA	let-7b
t0068371	22	1	TAAGGTAGTAGGGTGTGTGGTT	miRNA	let-7b
t0068416	21	1	TGAGGTAGTGGGTTGTGTCGT	miRNA	let-7b
t0068431	21	1	TGAGGTGGTAGGTTGTGGGGT	miRNA	let-7b
t0068449	23	1	TGAGGTGGTAGGTTGTGTGGTAT	miRNA	let-7b
t0068637	25	1	TGAGGTAGTAGGTTGTGCGGTTGAT	miRNA	let-7b
t0068903	21	1	TGAGGTAGCAGGTTGTGTGGA	miRNA	let-7b
t0069083	24	1	TGAGGTAGGAGGTTGTGTGGTAGA	miRNA	let-7b
t0069120	24	1	TGAGGTAGTAGGTTGTGGGGTATC	miRNA	let-7b
t0069271	21	1	TGGGGTAGTAGGTTGTGTGTT	miRNA	let-7b
t0069327	21	1	TGAGGTACTAGGTTGTGTGTT	miRNA	let-7b
t0069399	24	1	TGAGGTTGTAGGTTGTGTGGTTAA	miRNA	let-7b
t0069486	21	1	TAAGGTAGTAGGTTGGGTGGT	miRNA	let-7b
t0069557	21	1	TGAGGTATCAGGTTGTGTGGT	miRNA	let-7b
t0069655	24	1	TGAGGTAGTAGGGTGTGTGGTTAT	miRNA	let-7b
t0069760	23	1	TGAGGTAGTTGGTTGTGTGGTAT	miRNA	let-7b
t0069804	19	1	TGAAGTAGTAGGTTGTGTG	miRNA	let-7b
t0069855	23	1	TGCGGTAGTAGGTTGTGTGGTAA	miRNA	let-7b
t0070040	24	1	TGCGGTAGTAGGTTGTGTGGTTAG	miRNA	let-7b
t0070106	24	1	TGAGATAGTAGGTTGTGTGGTTAT	miRNA	let-7b
t0070141	22	1	TGAGGCAGTAGGATGTGTGGTT	miRNA	let-7b
t0070162	21	1	TGAGGTGGTAGGTTGTGTGTT	miRNA	let-7b
t0070249	22	1	TGAGGAAGAAGGTTGTGTGGTT	miRNA	let-7b
t0070370	21	1	TGAGGAAGTAGGTTGGGTGGT	miRNA	let-7b
t0070419	21	1	TGAGGTGGTGGGTTGTGTGGT	miRNA	let-7b
t0070443	24	1	TGAGGTAGTAGGTTATGTGGTTGA	miRNA	let-7b
t0070596	21	1	TGGGGTGGTAGGTTGTGTGGT	miRNA	let-7b
t0070784	42	1	TGAGGTAGTTGGTTGTGTGGTTTGGTTTG(	miRNA	let-7b
t0070795	21	1	TGAGGCATTAGGTTGTGTGGT	miRNA	let-7b
t0070910	21	1	TGAGGTAGTAGGATGTGTGGA	miRNA	let-7b
t0070950	22	1	TGAGGAAGTAGGTTGTCTGGTT	miRNA	let-7b
t0070974	22	1	TGAGGTAGTCGGTTGTGGGGTT	miRNA	let-7b
t0071169	22	1	AAAGGTAGTAGGTTGTGTGGTT	miRNA	let-7b
t0071598	22	1	TAAGGTAGTAGGTTGTGGGGTT	miRNA	let-7b
t0071744	20	1	TGAGATAGTAGGTTGTGTGA	miRNA	let-7b
t0071896	21	1	TGAGGTAGTGAGTTGTGTGGT	miRNA	let-7b
t0071902	23	1	TGAGGTGGTAGGTTGTGTGGTAA	miRNA	let-7b
t0071954	21	1	GAGGTAGTAGGTTGCGTGGTT	miRNA	let-7b
t0072103	21	1	TGAGTTAATAGGTTGTGTGGT	miRNA	let-7b
t0072139	22	1	TGAGGTAGTAGGTCGTGGGGTT	miRNA	let-7b
t0072160	23	1	TGAGGTAGTAGGTTGTCTGGTTT	miRNA	let-7b
t0072214	22	1	TGCGTTAGTAGGTTGTGTGGTT	miRNA	let-7b
t0072312	22	1	TGAGGTAGTAGGTTGTGGGGCT	miRNA	let-7b
t0072688	21	1	TGAGGTAGCAGGTTGTGTTGT	miRNA	let-7b

t0072756	23	1	TGAGGTAGAAGGTTGTGTGGTTT	miRNA	let-7b
t0072832	19	1	AGAGGTAGTAGGTTGTGTG	miRNA	let-7b
t0072861	21	1	CCAGGTAGTAGGTTGTGTGGT	miRNA	let-7b
t0072882	20	1	AGAGGTAGTAGGTTGTGTGG	miRNA	let-7b
t0072896	20	1	GAGGAAGTAGGTTGTGTGGT	miRNA	let-7b
t0073364	22	1	TGAGGTAGTAGGTTGTGGGTTT	miRNA	let-7b
t0073370	24	1	TGAGGTAGTAGGTTGGGTGGTTGA	miRNA	let-7b
t0073448	21	1	GAGGTAGGAGGTTGTGTGGTT	miRNA	let-7b
t0073553	21	1	TAAGGTAGTAGGTTGTGCGGT	miRNA	let-7b
t0073581	24	1	TGAGGTAGTAGGTTATGTGGTTAT	miRNA	let-7b
t0073591	24	1	TGAGGTAGCAGGTTGTGTGGTTTT	miRNA	let-7b
t0073709	24	1	TGAGGTAGTAGGGTGTGTGGTTAA	miRNA	let-7b
t0073764	27	1	TGAGGTAGTAGGTTGTGTGGGAATATC	miRNA	let-7b
t0073820	24	1	TGAGGTGGTAGGTTGTGTGGTTAG	miRNA	let-7b
t0073911	22	1	TGTGGTAGTAGGTTGTTTGGTT	miRNA	let-7b
t0073929	22	1	TGAGGTAGTAGGTCGTGTAGTT	miRNA	let-7b
t0073936	22	1	TGAGGTAGTAGGTTGTGGGGAT	miRNA	let-7b
t0074122	24	1	TGAGGTAGTAGGCTGTGTGGTTAG	miRNA	let-7b
t0074130	23	1	TGAGGTAGTAGGTTGTGAGGTTG	miRNA	let-7b
t0074212	27	1	TGAGGTAGTAGGTTGTGTGGTTATATA	miRNA	let-7b
t0074219	22	1	TGAGGTGGTGGGTTGTGTGGTT	miRNA	let-7b
t0074269	23	1	TGAGGTAGTATGTTGTGTGGTTT	miRNA	let-7b
t0074288	21	1	TGAGGTAGTAGGTTCTGTGGA	miRNA	let-7b
t0074430	23	1	TGAGGTATTAGGTTGTGTGGTTA	miRNA	let-7b
t0074535	21	1	TGAGGTAGTAAGTTGTGGGGT	miRNA	let-7b
t0074554	27	1	TGAGGTAGTAGGTTGTGTGGTTGTATC	miRNA	let-7b
t0074597	20	1	TGAGGTAGTAGGCGGTGTGG	miRNA	let-7b
t0074634	21	1	TTAGGTAGTAGTTTGTGTGGT	miRNA	let-7b
t0074824	21	1	TGAGGTAGTAGGTTGTGCGTT	miRNA	let-7b
t0074856	22	1	TGAGGTAGAAGGTTGTGGGGTT	miRNA	let-7b
t0074872	23	1	TGAGGTAATAGGTTGTGTGGTTG	miRNA	let-7b
t0075458	25	1	TGAGGCAGTAGGTTGTGTGGTTATC	miRNA	let-7b
t0075647	21	1	TGAGGTAGTAGGGGGTGTGGT	miRNA	let-7b
t0075819	22	1	TAAGGTAGGAGGTTGTGTGGTT	miRNA	let-7b
t0075870	21	1	TGATGTAGTAGGCTGTGTGGT	miRNA	let-7b
t0075912	22	1	TGAGGAAGTAGGTTGTGTGTTT	miRNA	let-7b
t0075920	22	1	TGAGGTGGTAGGTTGTGCGGTT	miRNA	let-7b
t0076113	23	1	TGAGGTAGTAGGTTGTGCGGTAT	miRNA	let-7b
t0076395	21	1	TGAGCTAGTAGGTTGTGGGGT	miRNA	let-7b
t0076427	23	1	TGAGGTAGTAGGTTCTGTGGTTA	miRNA	let-7b
t0076488	21	1	CGAGGTAGTAGGTTGTGTAGT	miRNA	let-7b
t0076535	21	1	TGAGGTAGTAGGTTGTGTGTA	miRNA	let-7b
t0076546	20	1	TTAGGTAGTAGGTTGTGTGG	miRNA	let-7b
t0076656	22	1	TGAGGTAGTAGGGTGGGTGGTT	miRNA	let-7b
t0076687	42	1	TGAGGTGGTTGGTTGTGTGGTTTGGTTTG(	miRNA	let-7b
t0076740	25	1	TGAGGTAGTAGGTTGTGGGGTAGTC	miRNA	let-7b
t0076925	26	1	TGAGGTAGTAGGTTGTGTGGTCTATC	miRNA	let-7b
t0077390	21	1	TGAGGTAGTAGGATGTGGGGT	miRNA	let-7b
t0077575	27	1	TGAGGAAGTAGGTTGTATGGTTGAATC	miRNA	let-7b
t0077663	21	1	TGAGGTAGGAGGTTGTGGGGT	miRNA	let-7b
t0077727	22	1	TGGGGTATTAGGTTGTGTGGTT	miRNA	let-7b
t0077738	22	1	TGAGGTGGTAGGTTGTGTGTTT	miRNA	let-7b
t0077976	22	1	TGAGGTGGTAGTTTGTGTGGTT	miRNA	let-7b
t0078113	19	1	TGAGATAGTAGGTTGTGTG	miRNA	let-7b
t0078189	26	1	CGAGGTAGTAGGTTGTGTGGTTAAAA	miRNA	let-7b
t0078205	22	1	TGAGGTTGTAGGTTGTGGGGTT	miRNA	let-7b
t0078285	21	1	TGAGGTAGCAGGTTGTGTAGT	miRNA	let-7b
t0078382	20	1	TGAGGTAGTAGGTTGTGCAG	miRNA	let-7b

t0078527	19	1	TGTAGTAGGTTGTGTGGGT	miRNA	let-7b
t0078559	25	1	TGAGGTAGTAGGGTGTGTGGTTATC	miRNA	let-7b
t0078659	26	1	TGAGGTAGCAGGTTGTATGGTTAATC	miRNA	let-7b
t0078919	25	1	TGAGGTAGTAGGCTGTGTGGTTGAT	miRNA	let-7b
t0078952	23	1	CTGAGGTAGTAGGTTGTGTGGTA	miRNA	let-7b
t0079097	21	1	TGAGGTAGCAGGTTGTGGGGT	miRNA	let-7b
t0079134	22	1	TGAGGAAGTAGGTTGTTTGGTT	miRNA	let-7b
t0079191	18	1	CGAGGTAGTAGGTTGTGT	miRNA	let-7b
t0079238	22	1	CTGAGGAAGTAGGTTGTGTGGT	miRNA	let-7b
t0079298	25	1	TGAGGTAGTAGATTGTGTGGTTAAA	miRNA	let-7b
t0079458	20	1	TGAGGTTGTAGGTTGTGTGG	miRNA	let-7b
t0079639	26	1	TGAGGTAGTAGGTCGTATGGTTTATC	miRNA	let-7b
t0079851	21	1	TGAGGAGGTAGGTTGTGTGGT	miRNA	let-7b
t0079927	22	1	TGAGGTAGTAGGTTGTGAGGTA	miRNA	let-7b
t0079932	26	1	TGAGGTAGTAGGTTGTGTGGTTCATC	miRNA	let-7b
t0079975	19	1	TGAGGTAGTAGGATGTGTG	miRNA	let-7b
t0080144	21	1	TAAGGTAATAGGTTGTGTGGT	miRNA	let-7b
t0080211	25	1	TGAGGTAGTAGGATGTGTGGTTAAA	miRNA	let-7b
t0080228	22	1	TGAGGTAGTAGGTTGTCTGGGT	miRNA	let-7b
t0080373	22	1	TAGGTAGTAGGTTGCGTGGTTA	miRNA	let-7b
t0080431	22	1	CGAGGTAGTGGGTTGTGTGGTT	miRNA	let-7b
t0080442	23	1	TGAGGTAGTAGGTGGTGTGGTTA	miRNA	let-7b
t0080450	25	1	TAAGGTAGTAGGTTGTGTGGTTTAA	miRNA	let-7b
t0080769	23	1	GGAGGTAGTAGGTTGTGTGGTTA	miRNA	let-7b
t0080868	24	1	TGAGGTAGTAGGCTGTGTGGTTAA	miRNA	let-7b
t0080992	25	1	TGAGGTAGTAGGTTGTATGGCTATA	miRNA	let-7b
t0081251	44	1	TGAGGTAGTAGGTTGTGTGGTTCGTCTGG	miRNA	let-7b
t0081298	22	1	TGAGGTAGTAGGGTGCGTGGTT	miRNA	let-7b
t0081685	21	1	GAGGTAGTACGTTGTGTGGTT	miRNA	let-7b
t0081738	21	1	TGAGGTAGTAGGCTGTGGGGT	miRNA	let-7b
t0081754	22	1	TGAGGAAGTAGGTAGTGTGGTT	miRNA	let-7b
t0081801	22	1	TGAGGTAGTAGGTTGGGGGGTT	miRNA	let-7b
t0081858	24	1	TGAGGTAGTAGGTTGTGCGGTTGA	miRNA	let-7b
t0081897	24	1	TGGGGTAGTAGGTTGTGTGGTAAT	miRNA	let-7b
t0082026	22	1	TGTGGTAGTAGGTTGTGTGGGT	miRNA	let-7b
t0082112	28	1	TGAGGTAGTAGGTTGTATGGTTAAGATC	miRNA	let-7b
t0082163	22	1	TGAGGTAGTAGGTTGTGTATTT	miRNA	let-7b
t0082203	23	1	TGAGGCAGTAGGTTGTGTGGTTG	miRNA	let-7b
t0082432	21	1	TGAGGAAGGAGGTTGTGTGGT	miRNA	let-7b
t0082481	23	1	TGAGGTAGTAGGTCGTGTGGTAT	miRNA	let-7b
t0082486	23	1	TGAGGTAGTAGGTTGAGTGGTTT	miRNA	let-7b
t0082677	21	1	TGAGGTAGTAGGTTGGGGGGT	miRNA	let-7b
t0082718	20	1	TGAGGTAGTAGGTTGTGCGA	miRNA	let-7b
t0083457	24	1	TGAGGTAGTAGGTTGTTTGGTTAA	miRNA	let-7b
t0083541	25	1	TGAGGTAGTAGGTTGTGTGGGTATC	miRNA	let-7b
t0083612	21	1	TGAGTTACTAGGTTGTGTGGT	miRNA	let-7b
t0083634	21	1	CGAGGTAGTGGGTTGTGTGGT	miRNA	let-7b
t0084063	21	1	TGCGGTAGTAGGTTGTGTGGA	miRNA	let-7b
t0084124	20	1	TGAGGTAGTAGGTTGCGTAG	miRNA	let-7b
t0084352	21	1	TGAGGCAGTAAGTTGTGTGGT	miRNA	let-7b
t0084449	21	1	TGAGATAGTAGGTTGTGTAGT	miRNA	let-7b
t0084556	19	1	GGAGGTAGTAGGTTGTGTG	miRNA	let-7b
t0084782	22	1	TGAGGTTGTTGGTTGTGTGGTT	miRNA	let-7b
t0084793	22	1	TGAAGTAGTAGGTTGTGTGGGT	miRNA	let-7b
t0084874	21	1	TGGGGTAGTAGGTTGTGGGGT	miRNA	let-7b
t0085054	21	1	TGTGGTAGTTGGTTGTGTGGT	miRNA	let-7b
t0085067	21	1	TGAGGTAATAGGTTGTGTGGA	miRNA	let-7b
t0085082	25	1	TGAGGTAGTGGGTTGTGTGGTTAAA	miRNA	let-7b

t0085198	23	1	TGAGGTAGTAGGTTATGTGGTAA	miRNA	let-7b
t0085237	22	1	TGAGGTGGTAGGTGGTGTGGTT	miRNA	let-7b
t0085387	24	1	TGAGGTAGTAGGTTGTGTGCTTAA	miRNA	let-7b
t0085556	23	1	TGAGGTAGAAGGTTGTGTAGTTT	miRNA	let-7b
t0085649	41	1	TGAGGTAGTAGGTTGTGTGGTTTCGTTTG(	miRNA	let-7b
t0085656	23	1	TGGGGTAGAAGGTTGTGTGGTTA	miRNA	let-7b
t0085688	23	1	TGAGGTAGTAGGTTGTGTGATTT	miRNA	let-7b
t0085903	24	1	TGAGGTAGTAGATTGTGTGGTTAG	miRNA	let-7b
t0086091	21	1	TGAGGAAGTAGGATGTGTGGT	miRNA	let-7b
t0086152	23	1	TGAGGTAGTAGGCTGTGTGGTAT	miRNA	let-7b
t0086217	25	1	TGAGGTTGTAGGGTGTGTGGTTATC	miRNA	let-7b
t0086421	22	1	TGAGGTAGTAGGCTGTGTGGTG	miRNA	let-7b
t0086467	23	1	CGAGGTAGTAGGTTGTGTGGTAT	miRNA	let-7b
t0086601	20	1	TGAGGGCGTAGGTTGTGTGG	miRNA	let-7b
t0086775	42	1	TGAGGTAGTGGGTTGTGTGGTTTGGTTTG(	miRNA	let-7b
t0086882	24	1	TGGGGTAGTAGGTTGTATGGTTAG	miRNA	let-7b
t0087123	25	1	TGTGGTAGTAGGTTGTGTGGTTATC	miRNA	let-7b
t0087136	22	1	TGTGGTTGTAGGTTGTGTGGTT	miRNA	let-7b
t0087214	24	1	TGAGGTAGTAGGTTGTGTGGTATG	miRNA	let-7b
t0087263	24	1	TGAGGTAGTAGGTTGTGTGGCTTT	miRNA	let-7b
t0087610	19	1	TGAGGTAGTAGATTGTGTG	miRNA	let-7b
t0087896	22	1	TGAGGTAGTAAGTTGTGTGGAT	miRNA	let-7b
t0088075	24	1	TGAGGTAGTAGGTTGTGTGGTTTC	miRNA	let-7b
t0088081	24	1	TGAGGTATTAGGTTGTGTGGTTAA	miRNA	let-7b
t0088167	22	1	TGAGGCAGGAGGTTGTGTGGTT	miRNA	let-7b
t0088253	22	1	TGAGATAGTAGGTTGTGTGTTT	miRNA	let-7b
t0088586	21	1	TGAGGTGGTAGGTTGTGTGGC	miRNA	let-7b
t0088685	23	1	TGCGGTAGTAGGTTGTGTGGTAT	miRNA	let-7b
t0088781	20	1	TGAGGCAGTAGGTTGTGCGG	miRNA	let-7b
t0088964	24	1	TGAGGTCGTAGGTTGTGTGGTTAT	miRNA	let-7b
t0089443	27	1	TGAGGCAGTAGGTTGTGTGGTAATATC	miRNA	let-7b
t0089488	21	1	TGAGGTAGTAGGTTGTGTGCA	miRNA	let-7b
t0089553	23	1	TGAGGTAGCAGGTTGTGTGGTAT	miRNA	let-7b
t0089594	24	1	TGAGGTAGTAGGTTCTGTGGTTAG	miRNA	let-7b
t0089663	23	1	TAAGGTAGTAGGTTGTGTGGTTG	miRNA	let-7b
t0089935	26	1	TGAGGTAATAGGTTGTGTGGTAAGAA	miRNA	let-7b
t0089972	22	1	TGAGGTAGTCAGTTGTGTGGTT	miRNA	let-7b
t0090033	22	1	TCAGGTAGTAGGTTGTGTGGTA	miRNA	let-7b
t0090060	24	1	TGAGGTAGTAGGTTGTGCGGTTTT	miRNA	let-7b
t0090089	23	1	TGAGGAAGTAGGTTGTGTGGTTG	miRNA	let-7b
t0090143	24	1	TGAGGTAGTAGGTCGTGTGGTTAG	miRNA	let-7b
t0090158	23	1	TGAGGTCGTAGGTTGTGTGGTTA	miRNA	let-7b
t0090287	22	1	TGGGGTAGTAGGTTGTTTGGTT	miRNA	let-7b
t0090293	22	1	TGATGTAGTAGGGTGTGTGGTT	miRNA	let-7b
t0090396	25	1	TGAGGTAGTAGGTTGGGTGGTTATC	miRNA	let-7b
t0090515	21	1	TGAGGAAGTAGGTCGTGTGGT	miRNA	let-7b
t0090546	24	1	TGCGGTAGTAGGTTGTGTGGTTAT	miRNA	let-7b
t0090557	21	1	TGAGGTAGGAGGTTGTGTGGG	miRNA	let-7b
t0090739	20	1	TGAGGAAGTAGGTTGTGTGT	miRNA	let-7b
t0090773	24	1	TGAGGTAGTAGGTTGTGTGGAAAA	miRNA	let-7b
t0090827	22	1	TGAGGTAGGAGGTTGGGTGGTT	miRNA	let-7b
t0090874	23	1	TGAGGTGGTAGGTTGTGTGGTTG	miRNA	let-7b
t0091246	22	1	TGAGGTAGTAGGTTCTGCGGTT	miRNA	let-7b
t0091311	22	1	TGAGGTAGGAGGTTGTGGGGTT	miRNA	let-7b
t0091335	18	1	TGAGGTGGTAGGTTGTGT	miRNA	let-7b
t0091446	21	1	TGAGGAAGTAGGTTGTGGGGT	miRNA	let-7b
t0091466	22	1	TGAAGTAGTAGATTGTGTGGTT	miRNA	let-7b
t0091689	22	1	TGAGGTAGTATGTTGTGTAGTT	miRNA	let-7b

t0091826	19	1	TGAGGTAGGAGGTGTGGTT	miRNA	let-7a
t0091975	18	1	TGAGGTAGCAGGTGTGGT	miRNA	let-7a
t0092056	21	1	TGAGGTAGTAGGTTGGGTGTT	miRNA	let-7a
t0092222	22	1	TGAGGTAGTAGATTTGATAGGT	miRNA	let-7a
t0092453	23	1	TGAGGTAGTAGATTTGGATAGTT	miRNA	let-7a
t0092456	19	1	TGAGGTAGTAGTTGTGGTG	miRNA	let-7a
t0092783	20	1	TGAGGTAGTAGGTTGGGGGT	miRNA	let-7a
t0092790	22	1	TGAGGTAGTAGGTTCTGTGGAA	miRNA	let-7a
t0092950	24	1	TGGGGTAGTAGGTTGTATAGTATC	miRNA	let-7-5p
t0093243	24	1	TGAGGTAGGAGGTTGTATGGTATC	miRNA	let-7-5p
t0093249	22	1	TGAGGTCGTAGGTTGTATAGTG	miRNA	let-7-5p
t0093304	25	1	TGAGGTAGTAGGTTGTATGGTCATC	miRNA	let-7-5p
t0093410	22	1	TGAGGTAGTAGGTTGTATGATC	miRNA	let-7-5p
t0041290	22	1	TGAGGTAGTAGGTTGTATCGTA	miRNA	let-7-5p
t0043686	24	1	TGAGGTAGTAGGTTGCATAGTATC	miRNA	let-7-5p
t0053134	22	1	TGAGGTAGTAGGTTGTATTGTA	miRNA	let-7-5p
t0060225	23	1	TGAGGAAGTAGGTTGTATAGTAT	miRNA	let-7-5p
t0069769	22	1	TGAGGTAGTAGGTTGTATGGTG	miRNA	let-7-5p
t0080393	24	1	TGAGGTAGTAGGTTGTATAGTAAA	miRNA	let-7-5p
t0086158	24	1	TTAGGTAGTAGGTTGTATGGTATC	miRNA	let-7-5p
t0088001	23	1	TGAGGTAGTAGGTTGTATAGTGT	miRNA	let-7-5p
t0040950	22	1	TGAGGTGGTAGGTTGTATAGTA	miRNA	let-7-5p
t0042237	24	1	TGAGGTAGTAGGCTGTATAGTATC	miRNA	let-7-5p
t0042241	24	1	TGAGGTAGTAGGTTGTATGGTGTC	miRNA	let-7-5p
t0043208	22	1	TGAGGTAGTAGGTTGCATAGTA	miRNA	let-7-5p
t0045820	23	1	TAAGGTAGTAGGTTGTATAGTAA	miRNA	let-7-5p
t0046950	24	1	TGAGGTAGTAGCTTGTATAGTATC	miRNA	let-7-5p
t0047485	21	1	TGGGTAGTAGGTTGTATAGTA	miRNA	let-7-5p
t0048794	22	1	TGTGGTAGTAGGTTGTATAGTA	miRNA	let-7-5p
t0049012	24	1	TGAGGTAGTAGGTCGTATAGTATC	miRNA	let-7-5p
t0050690	23	1	TGAGGTAGTAGGTTGTATAGTCA	miRNA	let-7-5p
t0052323	22	1	TGAGGTAGTAGGTTGTACAGTA	miRNA	let-7-5p
t0053679	26	1	TGAGGTAGTCGGTTGTATGGTAAATC	miRNA	let-7-5p
t0055413	22	1	TGAGGTAGTAGTTTGTAAAGTA	miRNA	let-7-5p
t0058031	22	1	CGAGGTAGTAGGTTGTATAGTA	miRNA	let-7-5p
t0059734	22	1	TGAGGTAGTAGGTTGTGTAGAA	miRNA	let-7-5p
t0060878	24	1	TGAGGTAGTAGGTTGTATGGTCTC	miRNA	let-7-5p
t0061220	24	1	TGAGGTAGTAGGCTGTATGGTATC	miRNA	let-7-5p
t0062746	25	1	TGAGGTAGTAGCTTGTATAGTCCGG	miRNA	let-7-5p
t0063543	23	1	TGAGGTGGTAGGTTGTATAGTAA	miRNA	let-7-5p
t0064001	25	1	TTGAGGTAGTAGGTTGTATGGTATC	miRNA	let-7-5p
t0064560	23	1	TGAGGTAGTAGGTTGTATGGTAT	miRNA	let-7-5p
t0065222	22	1	TGAGGAAGTAGGTTGTATAGTA	miRNA	let-7-5p
t0065897	23	1	TGAGGTAGTAGGTTGTACAGTAA	miRNA	let-7-5p
t0066310	22	1	TGAGGTAGTAGGCTGTATAGTG	miRNA	let-7-5p
t0068038	22	1	TGAGGTAGTAGGGTGTATAGTG	miRNA	let-7-5p
t0068093	22	1	TGAGGTAGTAGGTTGTATGGTC	miRNA	let-7-5p
t0071390	23	1	TGAGGTAGTAGGTTGTGTAGTAA	miRNA	let-7-5p
t0072023	24	1	TGAGGTAGTAGGTTGTATGGTATA	miRNA	let-7-5p
t0076930	23	1	TGAGGTAGTAGGTCGTATAGTTT	miRNA	let-7
t0077066	19	1	TGAGGTAGTAGGTAGTATA	miRNA	let-7
t0081924	21	1	TGAGGGGGTAGGTTGTATAGT	miRNA	let-7
t0083134	22	1	TGATGTAGTAGGCTGTATAGTT	miRNA	let-7
t0083749	21	1	GTGAGGAAGTAGGTTGTATAG	miRNA	let-7
t0084221	23	1	TGAGGTGGTAGGTTGTATAGTTG	miRNA	let-7
t0085066	22	1	AGACGTAGTAGGTTGTATAGTT	miRNA	let-7
t0085559	22	1	TGAGGTAGTAGGTTGTACAGGT	miRNA	let-7
t0085869	20	1	TGAGGTAGTAGGTTGTAGAG	miRNA	let-7

t0086131	22	1	TGCGGTAGTAGGCTGTATAGTT	miRNA	let-7
t0089290	21	1	TGAGGCAGTAGGATGTATAGT	miRNA	let-7
t0091266	22	1	TGAGGTAGTAGGGTCTATAGTT	miRNA	let-7
t0091682	21	1	TGGGGAAGTAGGTTGTATAGT	miRNA	let-7
t0040868	23	1	TGAGGTAGCAGGTTGTATAGTTG	miRNA	let-7
t0041434	23	1	CTGAGGAAGTAGGTTGTATAGTT	miRNA	let-7
t0041673	20	1	TGAGGTAGTAGGTGGTATAG	miRNA	let-7
t0042263	23	1	TGAGGTAGTCGGTTGTATAGTTA	miRNA	let-7
t0042404	22	1	TGAGGTAGTAGGTTGGATCGTT	miRNA	let-7
t0042482	22	1	TGAGGTAGTAGGTTGTACAGAT	miRNA	let-7
t0043142	19	1	TGAGGTAGTAGGTTGTATC	miRNA	let-7
t0043418	21	1	TGGGTAGTAGGTTGTATAGTT	miRNA	let-7
t0043521	23	1	TGAGGTAGTAGGTTGTACAGTTT	miRNA	let-7
t0043622	21	1	TAAGGTAGTAGGTCGTATAGT	miRNA	let-7
t0044380	23	1	TGAGGTAGTAGGCTGTATAGTTT	miRNA	let-7
t0044453	23	1	TGAGGTAGTAGGTTGTATCGTTT	miRNA	let-7
t0044647	20	1	TGAGGTAGTAGGGTGGATAG	miRNA	let-7
t0044668	23	1	TGAGGTAGTACGTTGTATAGTTA	miRNA	let-7
t0045487	25	1	TGAGGTAGTAGGTTGTATAGTTGTC	miRNA	let-7
t0045507	21	1	TGAGATAGTAGGTTGAATAGT	miRNA	let-7
t0045617	22	1	TGAGGTAGCAGGTTCTATAGTT	miRNA	let-7
t0046423	21	1	TGAGGTAGTAGGACGTATAGT	miRNA	let-7
t0046493	23	1	TGAGGTAGTGGGTTGTATAGTTA	miRNA	let-7
t0046604	23	1	TGAGGCAGTAGGTTGTATAGTTT	miRNA	let-7
t0046840	22	1	TGAGATTGTAGGTTGTATAGTT	miRNA	let-7
t0047274	23	1	TGGGGTAGTAGGTTGTATAGTTT	miRNA	let-7
t0047333	18	1	TGAGGTAGTAGGGTGTAT	miRNA	let-7
t0047354	20	1	TGAGGTAGTAGGATGTATAC	miRNA	let-7
t0047433	21	1	TGAGGTAGTGGGTTGTTTAGT	miRNA	let-7
t0047801	22	1	CTGAGGAAGTAGGTTGTATAGT	miRNA	let-7
t0047810	23	1	TGAGGTAGGAGGTTGTATAGTTT	miRNA	let-7
t0048557	20	1	GAGGAAGTAGGTTGTATAGT	miRNA	let-7
t0048634	25	1	TGAGGTAGTAGGTTGTATAGTTATA	miRNA	let-7
t0048886	23	1	TGAGGTAGTAGGTTGTATAGGTA	miRNA	let-7
t0048910	20	1	TGGGGTAGTAGGTTGTATCG	miRNA	let-7
t0048929	21	1	TGCGGTAGTAGGTTGCATAGT	miRNA	let-7
t0049132	20	1	TGAGGTTGTAGGTTGTATAG	miRNA	let-7
t0049234	20	1	TGAGGTAGGAGGTCGTATAG	miRNA	let-7
t0049284	21	1	TGAGGTAGTAGGGTGTATACT	miRNA	let-7
t0049633	19	1	TGAGGTAATAGGTTGTATA	miRNA	let-7
t0049657	21	1	GTGAGGTCGTAGGTTGTATAG	miRNA	let-7
t0049760	20	1	CGAGGTAGTAGGTTGTATAA	miRNA	let-7
t0050032	21	1	TGCGGTAGTAGGTTGTATATT	miRNA	let-7
t0050051	20	1	CGGGGTAGTAGGTTGTATAG	miRNA	let-7
t0050296	23	1	TGCGGTAGTAGGTTGTATAGTTG	miRNA	let-7
t0050477	22	1	TGAGGAAGTAGGCTGTATAGTT	miRNA	let-7
t0050481	22	1	TGAGATAGTAGGTTGTATTGTT	miRNA	let-7
t0050764	21	1	TAAGGTAGTTGGTTGTATAGT	miRNA	let-7
t0050925	21	1	TGAGATAGTAGGTTGTAAAGT	miRNA	let-7
t0051105	22	1	TGAGGTAGTAAGCTGTATAGTT	miRNA	let-7
t0051199	21	1	GAGGAAGTAGGTTGTATAGTT	miRNA	let-7
t0051428	23	1	TGAGGTAGTAGGTTCTATAGTTG	miRNA	let-7
t0051699	21	1	CGGGGTAGTAGGTTGTATAGT	miRNA	let-7
t0051799	21	1	TGAGGTGGTGGGTTGTATAGT	miRNA	let-7
t0052444	19	1	TGACGTAGTAGGTTGTATA	miRNA	let-7
t0053114	24	1	TGAGGAAGTAGGTTGTATAGTTAA	miRNA	let-7
t0053151	26	1	TGAGGTAGTAGGGTGTATAGTTAATC	miRNA	let-7
t0053463	21	1	TGAGGTAGTTGGTTATATAGT	miRNA	let-7

t0054635	22	1	TGAGGTAGGAGGTTGTACAGTT	miRNA	let-7
t0054758	19	1	TGAGGTAGAAGGTTGTATA	miRNA	let-7
t0054923	20	1	TGAGGTAGTAGGGTCTATAG	miRNA	let-7
t0055854	23	1	TGAGGTAGTAGGTTGGATAGTTA	miRNA	let-7
t0056140	19	1	TGAAGTAGTAGGTTGTATA	miRNA	let-7
t0056229	20	1	TGAGGAAGTAGGTTGTACAG	miRNA	let-7
t0056598	23	1	TGAGGTAGTAGGCTGTATAGTTA	miRNA	let-7
t0057375	22	1	GGAGGTAGTATGTTGTATAGTT	miRNA	let-7
t0057435	20	1	GAGGTAGTATGTTGTATAGC	miRNA	let-7
t0057708	23	1	TAAGGTAGTAGGTTGTATAGTTG	miRNA	let-7
t0058230	21	1	TGAGGTAGTAGGGTGTATAGA	miRNA	let-7
t0058570	25	1	TGAGGCAGTAGGTTGTATAGTTATC	miRNA	let-7
t0058611	21	1	TGAGGTAGAAGGATGTATAGT	miRNA	let-7
t0058718	22	1	TGACGTACTAGGTTGTATAGTT	miRNA	let-7
t0058815	23	1	TGAGGTAGTAGGCTGTATAGTTG	miRNA	let-7
t0059254	22	1	TGCGGTAGTAGGTTGTTTAGTT	miRNA	let-7
t0059443	21	1	TGCGGAAGTAGGTTGTATAGT	miRNA	let-7
t0059790	22	1	TGAAGTACTAGGTTGTATAGTT	miRNA	let-7
t0060299	25	1	TGAGGTAGTAGGTTGTAAAGTTATC	miRNA	let-7
t0060508	19	1	TGAGATAGTAGGTTGTATA	miRNA	let-7
t0060813	20	1	TGGGGTAGTAGGTTGTATAA	miRNA	let-7
t0061414	21	1	TGAGGTAGTAGGTTTTATTGT	miRNA	let-7
t0061608	21	1	TGAGGTAGGAGGTTGGATAGT	miRNA	let-7
t0062171	23	1	TCAGGTAGTAGGTTGTATAGTTA	miRNA	let-7
t0062205	25	1	TGAGGTAGTAGGGTGTATAGTTATC	miRNA	let-7
t0062702	19	1	TGAGGTAGTAGGTTATATA	miRNA	let-7
t0063212	20	1	TGCGGTAGTAGGCTGTATAG	miRNA	let-7
t0063460	21	1	GGAGGTGGTAGGTTGTATAGT	miRNA	let-7
t0063498	23	1	TGAGGTAGTAGGGTGTATAGTTT	miRNA	let-7
t0063596	20	1	GAGGTAGTAGGTTGTATAGC	miRNA	let-7
t0064017	25	1	TGGGGTAGTAGGTTGTATAGTTATC	miRNA	let-7
t0065053	18	1	TGAGGTAGTGGGTTGTAT	miRNA	let-7
t0066555	22	1	TGAGGGGGTAGGTTGTATAGTT	miRNA	let-7
t0066914	21	1	TGAGTTAGTAGGTTGTACAGT	miRNA	let-7
t0067170	22	1	TGTGGTTGTAGGTTGTATAGTT	miRNA	let-7
t0067343	21	1	TGCGGTCGTAGGTTGTATAGT	miRNA	let-7
t0067346	23	1	TGAGGTAGTAAGTTGTATAGTTG	miRNA	let-7
t0067465	22	1	TGAGGTAGCAGGCTGTATAGTT	miRNA	let-7
t0067716	22	1	TGAGGTAGTAGGTTGTACCGTT	miRNA	let-7
t0067803	22	1	TGAGGAAGTAGGTTGGATAGTT	miRNA	let-7
t0068575	23	1	TGAGGCAGTAGGTTGTATAGTTA	miRNA	let-7
t0069144	21	1	TGTGGTAGTAGGTTGTTTAGT	miRNA	let-7
t0069472	21	1	TGAGGTAGTAGGGTGAATAGT	miRNA	let-7
t0069756	25	1	TGAGGTAGTAGGTTGTATAGTTACA	miRNA	let-7
t0070533	22	1	TGAGGTAGTAGGTTGTATATCT	miRNA	let-7
t0070755	23	1	TGAGGCAGTAGGTTGTATAGTTG	miRNA	let-7
t0071288	20	1	TGAGGTAGTAGGGTGAATAG	miRNA	let-7
t0071674	20	1	TGAGGTAGTAGGGTGTCTAG	miRNA	let-7
t0071938	22	1	TGGGGTAGTAGGTTGTACAGTT	miRNA	let-7
t0071965	22	1	TGAGGTAGTCGGTTGTATATTT	miRNA	let-7
t0072041	42	1	TGAGGTAGTAGGTTGTATTGTTTGTGTGT(	miRNA	let-7
t0072408	21	1	TGACGTACTAGGTTGTATAGT	miRNA	let-7
t0072569	20	1	GAGGCAGTAGGTTGTATAGT	miRNA	let-7
t0072588	23	1	TGAGGTAGGAGGTTGTATATTTG	miRNA	let-7
t0072930	19	1	TGAGGTACTAGGTTGTATA	miRNA	let-7
t0073269	21	1	TGAGGTAGTATGTTTTATAGT	miRNA	let-7
t0073886	19	1	TGAGGTAGTAGGTTGAATA	miRNA	let-7
t0075688	22	1	TGAGGCAGTAGGTTGTATATTT	miRNA	let-7

t0076195	21	1	GAGGTAGTGGGTTGTATAGTT	miRNA	let-7
t0077179	20	1	TGAGGTGGTAGGTTGTATAA	miRNA	let-7
t0077257	21	1	TGAGCTGGTAGGTTGTATAGT	miRNA	let-7
t0077967	19	1	TGAGGTAGTAGGTTGGATA	miRNA	let-7
t0078096	23	1	TGAGGTAGTAGGATGTATAGTTT	miRNA	let-7
t0078240	21	1	TGAGGTAGTAGGATATATAGT	miRNA	let-7
t0078404	22	1	CGAGGTAGTAGGTTGTATTGTT	miRNA	let-7
t0078918	21	1	TGGGGTAGTAGGTTGTTTAGT	miRNA	let-7
t0078948	19	1	TGAGGTAGTAGGTTCTATA	miRNA	let-7
t0079054	20	1	TGAGGTAGTAGGTTGTAGAT	miRNA	let-7
t0079113	21	1	TGAGGCAGTAGGGTGTATAGT	miRNA	let-7
t0079386	19	1	TGAGGTAGTATGTTGTATA	miRNA	let-7
t0080087	22	1	TAAGGAAGTAGGTTGTATAGTT	miRNA	let-7
t0080173	24	1	TGAGGTAGTAGGTTGTATAGTTTC	miRNA	let-7
t0080277	21	1	CGAGGTAGTAGGTCGTATAGT	miRNA	let-7
t0080554	23	1	TGAGGTAGTAGGTTGTATAGCTT	miRNA	let-7
t0080606	22	1	TGAGGAAGTTGGTTGTATAGTT	miRNA	let-7
t0081047	21	1	TGAGGTGGAAGGTTGTATAGT	miRNA	let-7
t0081280	22	1	TGGGGTAGTAGGTTGTATATTT	miRNA	let-7
t0081657	20	1	TAAGGTGGTAGGTTGTATAG	miRNA	let-7
t0081732	18	1	TGAGGTTGTAGGTTGTAT	miRNA	let-7
t0082472	23	1	TGAGGTAGTAGGTTGTAAAGTTA	miRNA	let-7
t0082699	19	1	TGAGGTAGTTGGTTGTATA	miRNA	let-7
t0082787	25	1	TGAGGTAATAGGTTGTATAGTTATC	miRNA	let-7
t0082970	22	1	GTTTGAGGCAGTAGGTTGTATA	miRNA	let-7
t0083356	22	1	TGAAGTAGTAGGCTGTATAGTT	miRNA	let-7
t0083395	21	1	TGAGGTAGCAGGCTGTATAGT	miRNA	let-7
t0083529	22	1	TGAGCTAGCAGGTTGTATAGTT	miRNA	let-7
t0083642	21	1	GGAGGTAGTAGGTTGGATAGT	miRNA	let-7
t0083736	22	1	TGAGGTAGTAGGTTGAATTGTT	miRNA	let-7
t0084491	19	1	TGAGGTAGTAGGTTGTACA	miRNA	let-7
t0085336	22	1	TGAGGTAGAGGGTTGTATAGTT	miRNA	let-7
t0085402	23	1	TGAGGTAGTAGGATGTATAGTTG	miRNA	let-7
t0085611	19	1	TGAGGTAGTAGGTTGCATA	miRNA	let-7
t0085763	23	1	TGAGGTCGTAGGTTGTATAGTTG	miRNA	let-7
t0085775	23	1	TGAGGTAGTAGGTTGTATTGTTG	miRNA	let-7
t0086513	22	1	TGAGGTTGTAGGTTGTATATTT	miRNA	let-7
t0087909	20	1	TGAGGGGGTAGGTTGTATAG	miRNA	let-7
t0088153	23	1	TGAGGTAGTAGGATGTATAGTTA	miRNA	let-7
t0088251	23	1	TGTGGTAGTAGGTTGTATAGTTT	miRNA	let-7
t0088355	22	1	CTGAGGTAGTAGGTTGTCTAGT	miRNA	let-7
t0088576	21	1	TGAGGTCGTAGGTTGTCTAGT	miRNA	let-7
t0088755	23	1	TGAGGTAGTAGGTTGTATAGATT	miRNA	let-7
t0089263	18	1	TGAGGTATTAGGTTGTAT	miRNA	let-7
t0089387	23	1	TGAGGTAGTTGGTTGTATAGTTA	miRNA	let-7
t0089457	23	1	TGAGCTAGTAGGTTGTATAGTTT	miRNA	let-7
t0089475	23	1	TGAGGTAGCAGGTTGTATAGTTA	miRNA	let-7
t0090399	21	1	TGCGGTAGTAGGCTGTATAGT	miRNA	let-7
t0090489	22	1	TGGGGTAGTAGGATGTATAGTT	miRNA	let-7
t0090692	20	1	TGGGGTAGTAGGTTGTTTAG	miRNA	let-7
t0090967	22	1	TGAGGTGGTAGGTTGTACAGTT	miRNA	let-7
t0091046	22	1	TGAGATCATTGCGAAGGCCACT	miRNA	bantam
t0091183	22	1	TGAGATCATTGTGAAGGACACT	miRNA	bantam

nRBCs

Seq ID	Length	Read Count	Seq	Category	Annotation
t0000001	21	230509	AAACCGTTACCATTACTGAGT	miRNA	hsa-mir-451a
t0000003	22	174024	TTTGGCAATGGTAGAACTCACA	miRNA	hsa-mir-182

t0000005	24	159790	TTTGGCAATGGTAGAACTCACACT	miRNA	hsa-mir-182
t0000006	22	136475	TAGCAGCACGTAAATATTGGCG	miRNA	hsa-mir-16-2
t0000007	22	126146	AAACCGTTACCATTACTGAGTT	miRNA	hsa-mir-451a
t0000008	22	119769	TGAGGTAGTAGTTTGTGCTGTT	miRNA	hsa-let-7i
t0000009	22	100272	TATTGCACTTGTCCCGGCCTGT	miRNA	hsa-mir-92a-2
t0000010	22	91019	TGAGGTAGTAGATTGTATAGTT	miRNA	hsa-let-7f-2
t0000011	20	80084	TCCTGTACTGAGCTGCCCCG	miRNA	hsa-mir-486
t0000012	21	76791	TGAGGTAGTAGGTTGTATAGT	miRNA	hsa-let-7a-3
t0000013	23	67678	AAACCGTTACCATTACTGAGTTT	miRNA	hsa-mir-451a
t0000014	22	57560	AACATTCAACGCTGTCGGTGAG	miRNA	hsa-mir-181a-1
t0000015	22	56715	CATTGCACTTGTCTCGGTCTGA	miRNA	hsa-mir-25
t0000016	22	52930	TGAGGTAGTAGGTTGTATAGTT	miRNA	hsa-let-7a-3
t0000017	19	47538	AGCAGCATTGTACAGGGCT	miRNA	hsa-mir-107
t0000018	21	38124	TGAGGGGCAGAGAGCGAGACT	miRNA	hsa-mir-423
t0000020	20	31976	CCCATAAAGTAGAAAGCACT	miRNA	hsa-mir-142
t0000021	22	27859	TTCAAGTAATCCAGGATAGGCT	miRNA	hsa-mir-26a-2
t0000022	22	26767	AAGCTGCCAGTTGAAGAACTGT	miRNA	hsa-mir-22
t0000023	21	25774	TATTGCACTTGTCCCGGCCTG	miRNA	hsa-mir-92a-2
t0000025	20	24468	TTTGGCAATGGTAGAACTCA	miRNA	hsa-mir-182
t0000027	23	20255	GGATATCATCATATACTGTAAGT	miRNA	hsa-mir-144
t0000028	20	18060	CATTGCACTTGTCTCGGTCT	miRNA	hsa-mir-25
t0000029	23	17256	TATTGCACTTGTCCCGGCCTGTT	miRNA	hsa-mir-92a-1
t0000030	20	16205	TAGCAGCACATAATGGTTTG	miRNA	hsa-mir-15a
t0000033	23	15009	CAACGGAATCCCAAAAGCAGCTG	miRNA	hsa-mir-191
t0000036	22	13538	TACAGTACTGTGATAACTGAAG	miRNA	hsa-mir-101-2
t0000037	21	13340	CATTATTACTTTTGGTACGCG	miRNA	hsa-mir-126
t0000038	21	13017	TAGCAGCACGTAAATATTGGC	miRNA	hsa-mir-16-2
t0000039	23	12866	AACATTCAACGCTGTCGGTGAGT	miRNA	hsa-mir-181a-1
t0000040	20	12719	CCGCACTGTGGGTACTTGCT	miRNA	hsa-mir-106b
t0000042	21	12475	CTGACCTATGAATTGACAGCC	miRNA	hsa-mir-192
t0000043	21	12292	CAAAGTGCTGTTCGTGCAGGT	miRNA	hsa-mir-93
t0000044	20	12180	ACCAATATTACTGTGCTGCT	miRNA	hsa-mir-16-2
t0000045	20	11887	AAACCGTTACCATTACTGAG	miRNA	hsa-mir-451a
t0000046	22	11790	TACCGCACTGTGGGTACTTGCT	miRNA	hsa-mir-106b
t0000047	24	11697	TGTAAACATCCTTGACTGGAAGCT	miRNA	hsa-mir-30e
t0000048	21	11601	TGAGGTAGTAGTTTGTGCTGT	miRNA	hsa-let-7i
t0000049	20	11453	CTGTACTGAGCTGCCCCGAG	miRNA	hsa-mir-486
t0000050	21	9552	TGAGGTAGTAGTTTGTACAGT	miRNA	hsa-let-7g
t0000051	22	9447	AGCAGCATTGTACAGGGCTATG	miRNA	hsa-mir-103a-1
t0000053	21	8892	CATAAAGTAGAAAGCACTACT	miRNA	hsa-mir-142
t0000056	23	8820	TTTGGCAATGGTAGAACTCACAC	miRNA	hsa-mir-182
t0000057	21	8776	CATTGCACTTGTCTCGGTCTG	miRNA	hsa-mir-25
t0000059	22	8647	GGATATCATCATATACTGTAAG	miRNA	hsa-mir-144
t0000060	21	8060	TACAGTACTGTGATAACTGAA	miRNA	hsa-mir-101-2
t0000062	23	7574	TGAGGGGCAGAGAGCGAGACTTT	miRNA	hsa-mir-423
t0000063	19	7460	TGAGGTAGTAGGTTGTATA	miRNA	hsa-let-7a-3
t0000067	21	7318	TTTGGCAATGGTAGAACTCAC	miRNA	hsa-mir-182
t0000069	23	7313	TCCTGTACTGAGCTGCCCCGAGG	miRNA	hsa-mir-486
t0000071	22	7121	TGAGGTAGTAGTTTGTACAGTT	miRNA	hsa-let-7g
t0000072	21	6888	AAGCTGCCAGTTGAAGAACTG	miRNA	hsa-mir-22
t0000073	21	6323	AGCAGCATTGTACAGGGCTAT	miRNA	hsa-mir-107
t0000076	23	6022	AGCAGCATTGTACAGGGCTATGA	miRNA	hsa-mir-103a-1
t0000077	21	5978	ACCAATATTACTGTGCTGCTT	miRNA	hsa-mir-16-2
t0000078	19	5900	TCCTGTACTGAGCTGCCCC	miRNA	hsa-mir-486
t0000082	22	5693	TGAGGGGCAGAGAGCGAGACTT	miRNA	hsa-mir-423
t0000083	20	5391	TACAGTATAGATGATGTACT	miRNA	hsa-mir-144
t0000086	21	5117	GTACAGTACTGTGATAACTGA	miRNA	hsa-mir-101-2
t0000088	24	5067	TGTAAACATCCCCGACTGGAAGCT	miRNA	hsa-mir-30d

t0000091	20	4986	TGAGGTAGTAGGTTGTATAG	miRNA	hsa-let-7a-3
t0000094	26	4800	TTTGGCAATGGTAGAACTCACACTGG	miRNA	hsa-mir-182
t0000096	22	4800	GTACAGTACTGTGATAACTGAA	miRNA	hsa-mir-101-2
t0000097	20	4799	TGAGGTAGTAGTTTGTGCTG	miRNA	hsa-let-7i
t0000100	20	4699	TGAGGTAGTAGATTGTATAG	miRNA	hsa-let-7f-2
t0000101	22	4613	TGTGCAAATCCATGCAAAACTG	miRNA	hsa-mir-19b-2
t0000102	22	4324	TAGCTTATCAGACTGATGTTGA	miRNA	hsa-mir-21
t0000104	22	4277	TCAGTGCACTACAGAACTTTGT	miRNA	hsa-mir-148a
t0000106	22	4263	CTAGACTGAAGCTCCTTGAGGA	miRNA	hsa-mir-151a
t0000110	22	4147	TGTAAACATCCTACACTCAGCT	miRNA	hsa-mir-30b
t0000111	24	4088	TGTAAACATCCTACACTCTCAGCT	miRNA	hsa-mir-30c-1
t0000112	22	3919	CAAAGAATTCTCCTTTTGGGCT	miRNA	hsa-mir-186
t0000116	20	3867	AGCAGCATTGTACAGGGCTA	miRNA	hsa-mir-107
t0000118	26	3855	AAACCGTTACCATTACTGAGTTTAGT	miRNA	hsa-mir-451a
t0000121	19	3814	TATTGCACTTGTCCCGGCC	miRNA	hsa-mir-92a-2
t0000123	21	3767	AACATTCAACGCTGTCGGTGA	miRNA	hsa-mir-181a-1
t0000124	21	3726	AGAGGTAGTAGGTTGCATAGT	miRNA	hsa-let-7d
t0000126	19	3715	CTGTACTGAGCTGCCCCGA	miRNA	hsa-mir-486
t0000127	19	3661	TGAGGTAGTAGATTGTATA	miRNA	hsa-let-7f-2
t0000128	19	3506	CATTGCACTTGTCTCGGTC	miRNA	hsa-mir-25
t0000129	21	3455	TAGCAGCACATAATGGTTTGT	miRNA	hsa-mir-15a
t0000130	22	3396	ATCCTGTACTGAGCTGCCCCGA	miRNA	hsa-mir-486
t0000132	18	3172	AGCAGCATTGTACAGGGC	miRNA	hsa-mir-107
t0000133	25	3170	TTTGGCAATGGTAGAACTCACACTG	miRNA	hsa-mir-182
t0000136	23	3139	CAAAGTGCTGTTCGTGCAGGTAG	miRNA	hsa-mir-93
t0000142	18	3119	TTTGGCAATGGTAGAACT	miRNA	hsa-mir-182
t0000143	18	3003	TGAGGTAGTAGTTTGTGC	miRNA	hsa-let-7i
t0000145	20	2913	TATTGCACTTGTCCCGGCCT	miRNA	hsa-mir-92a-2
t0000146	21	2912	TTCAAGTAATCCAGGATAGGC	miRNA	hsa-mir-26a-2
t0000148	22	2893	TAGCAGCACATCATGGTTTACA	miRNA	hsa-mir-15b
t0000150	24	2789	AAACCGTTACCATTACTGAGTTTA	miRNA	hsa-mir-451a
t0000151	18	2711	TCCTGTACTGAGCTGCCC	miRNA	hsa-mir-486
t0000152	21	2674	CATGCCTTGAGTGTAGGACCG	miRNA	hsa-mir-532
t0000154	22	2648	TGAGGTAGTAGGTTGTGTGGTT	miRNA	hsa-let-7b
t0000158	22	2616	CAAAGTGCTGTTCGTGCAGGTA	miRNA	hsa-mir-93
t0000159	20	2540	TACAGTACTGTGATAACTGA	miRNA	hsa-mir-101-2
t0000162	21	2512	TGAGGTAGTAGGTTGTGTGGT	miRNA	hsa-let-7b
t0000164	21	2422	TTCAAGTAATTCAGGATAGGT	miRNA	hsa-mir-26b
t0000165	19	2411	GTACAGTACTGTGATAACT	miRNA	hsa-mir-101-2
t0000167	25	2346	AAACCGTTACCATTACTGAGTTTAG	miRNA	hsa-mir-451a
t0000170	21	2342	TAAAGTGCTTATAGTGCAGGT	miRNA	hsa-mir-20a
t0000171	20	2315	CATGCCTTGAGTGTAGGACC	miRNA	hsa-mir-532
t0000172	20	2284	GGATATCATCATATACTGTA	miRNA	hsa-mir-144
t0000173	21	2172	CGGGGCAGCTCAGTACAGGAT	miRNA	hsa-mir-486
t0000176	20	2170	TGACCTATGAATTGACAGCC	miRNA	hsa-mir-192
t0000177	22	2161	TTCAAGTAATTCAGGATAGGTT	miRNA	hsa-mir-26b
t0000179	23	2037	GTAAACATCCTTGACTGGAAGCT	miRNA	hsa-mir-30e
t0000180	21	1887	CAAAGTGCTTACAGTGCAGGT	miRNA	hsa-mir-17
t0000181	22	1857	AAAAGCTGGGTTGAGAGGGCGA	miRNA	hsa-mir-320a
t0000185	21	1845	CAAAGAATTCTCCTTTTGGGC	miRNA	hsa-mir-186
t0000192	19	1830	GGATATCATCATATACTGT	miRNA	hsa-mir-144
t0000195	20	1804	CGGGGCAGCTCAGTACAGGA	miRNA	hsa-mir-486
t0000196	22	1787	AGAGGTAGTAGGTTGCATAGTT	miRNA	hsa-let-7d
t0000197	21	1767	CTAGACTGAAGCTCCTTGAGG	miRNA	hsa-mir-151a
t0000200	22	1748	AACGGAATCCCAAAAGCAGCTG	miRNA	hsa-mir-191
t0000202	20	1739	TAAAGTGCTGACAGTGCAGA	miRNA	hsa-mir-106b
t0000203	21	1707	TCGAGGAGCTCACAGTCTAGT	miRNA	hsa-mir-151a
t0000205	20	1594	TTCACAGTGGCTAAGTTCTG	miRNA	hsa-mir-27b

t0000207	21	1540	CAACACCAGTCGATGGGCTGT	miRNA	hsa-mir-21
t0000209	21	1442	ATTGCACTTGTCCCGGCCTGT	miRNA	hsa-mir-92a-2
t0000220	22	1419	CAACGGAATCCCAAAAGCAGCT	miRNA	hsa-mir-191
t0000227	22	1351	CATGCCTTGAGTGTAGGACCGT	miRNA	hsa-mir-532
t0000235	19	1349	TGAGGTAGTAGTTTGTGCT	miRNA	hsa-let-7i
t0000239	22	1340	TGAGGTAGTAAGTTGTATTGTT	miRNA	hsa-mir-98
t0000246	23	1321	TGAGGTAGTAGATTGTATAGTTT	miRNA	hsa-let-7f-2
t0000247	20	1320	TTCAAGTAATTCAGGATAGG	miRNA	hsa-mir-26b
t0000248	20	1285	AATTGCACGGTATCCATCTG	miRNA	hsa-mir-363
t0000249	21	1259	TAGCTTATCAGACTGATGTTG	miRNA	hsa-mir-21
t0000250	22	1227	TGTGCAAATCTATGCAAAACTG	miRNA	hsa-mir-19a
t0000252	21	1198	TCAGGCTCAGTCCCCTCCCGA	miRNA	hsa-mir-484
t0000255	22	1187	GATATCATCATATACTGTAAGT	miRNA	hsa-mir-144
t0000260	22	1145	ACCACAGGGTAGAACCACGGAC	miRNA	hsa-mir-140
t0000265	20	1143	GTACAGTACTGTGATAACTG	miRNA	hsa-mir-101-2
t0000266	18	1104	TACAGTATAGATGATGTA	miRNA	hsa-mir-144
t0000271	23	1020	TACAGTACTGTGATAACTGAAGA	miRNA	hsa-mir-101-2
t0000273	19	1004	TTTGGCAATGGTAGAACTC	miRNA	hsa-mir-182
t0000280	23	981	TAGTGCAATATTGCTTATAGGGT	miRNA	hsa-mir-454
t0000293	22	977	ACTGGACTTGGAGTCAGAAGGC	miRNA	hsa-mir-378a
t0000299	23	922	ATCCTGTACTGAGCTGCCCCGAG	miRNA	hsa-mir-486
t0000305	20	918	TGTAAACATCCCCGACTGGA	miRNA	hsa-mir-30d
t0000306	21	916	GGATATCATCATATACTGTAA	miRNA	hsa-mir-144
t0000315	21	862	AAAAGCTGGGTTGAGAGGGCG	miRNA	hsa-mir-320a
t0000318	21	844	ACCGCACTGTGGGTACTTGCT	miRNA	hsa-mir-106b
t0000320	21	829	TAAAGTGCTGACAGTGCAGAT	miRNA	hsa-mir-106b
t0000329	18	814	CATAAAGTAGAAAGCACT	miRNA	hsa-mir-142
t0000331	21	811	CTACAGTATAGATGATGTACT	miRNA	hsa-mir-144
t0000335	20	712	TGAGGGGCAGAGAGCGAGAC	miRNA	hsa-mir-423
t0000336	22	670	AATGACACGATCACTCCCGTTG	miRNA	hsa-mir-425
t0000339	21	640	TCAGTGCACTACAGAACTTTG	miRNA	hsa-mir-148a
t0000365	20	638	ATTGCACTTGTCCCGGCCTG	miRNA	hsa-mir-92a-2
t0000373	22	636	TGTAAACATCCCCGACTGGAAG	miRNA	hsa-mir-30d
t0000385	18	625	AAACCGTTACCATTACTG	miRNA	hsa-mir-451a
t0000387	19	620	TACAGTACTGTGATAACTG	miRNA	hsa-mir-101-2
t0000388	22	613	CAAAGTGCTTACAGTGCAGGTA	miRNA	hsa-mir-17
t0000393	23	607	TATTGCACTTGTCCCGGCCTGTG	miRNA	hsa-mir-92a-2
t0000395	21	594	ACTGGACTTGGAGTCAGAAGG	miRNA	hsa-mir-378a
t0000397	22	584	CTTATCAGATTGTATTGTAATT	miRNA	hsa-mir-374a
t0000400	22	583	TATGGCACTGGTAGAATTCACT	miRNA	hsa-mir-183
t0000406	22	582	TAAAGTGCTTATAGTGCAGGTA	miRNA	hsa-mir-20a
t0000412	21	574	CCCATAAAGTAGAAAGCACTA	miRNA	hsa-mir-142
t0000413	23	569	TGACCTATGAATTGACAGCCAGT	miRNA	hsa-mir-192
t0000415	20	559	TGAGGTAGTAGTTTGTACAG	miRNA	hsa-let-7g
t0000418	19	545	AAACCGTTACCATTACTGA	miRNA	hsa-mir-451a
t0000422	21	543	CAGTGCAATGTTAAAAGGGCA	miRNA	hsa-mir-130a
t0000428	21	543	AATTGCACGGTATCCATCTGT	miRNA	hsa-mir-363
t0000433	22	542	ATTGCACTTGTCCCGGCCTGTT	miRNA	hsa-mir-92a-1
t0000435	20	539	TAGCTTATCAGACTGATGTT	miRNA	hsa-mir-21
t0000436	19	527	TACAGTATAGATGATGTAC	miRNA	hsa-mir-144
t0000438	23	524	AGCTCGGTCTGAGGCCCCTCAGT	miRNA	hsa-mir-423
t0000441	21	509	TTATAAAGCAATGAGACTGAT	miRNA	hsa-mir-340
t0000448	20	501	CAGTGCAATGTTAAAAGGGC	miRNA	hsa-mir-130a
t0000452	22	499	TACTAGACTGAAGCTCCTTGAG	miRNA	hsa-mir-151a
t0000466	24	494	GGATATCATCATATACTGTAAGTT	miRNA	hsa-mir-144
t0000470	22	487	CACTAGATTGTGAGCTCCTGGA	miRNA	hsa-mir-28
t0000472	23	486	TAAAGTGCTTATAGTGCAGGTAG	miRNA	hsa-mir-20a
t0000477	23	485	TAGCAGCACGTAAATATTGGCGT	miRNA	hsa-mir-16-2

t0000482	22	459	TACCCATTGCATATCGGAGTTG	miRNA	hsa-mir-660
t0000483	19	452	CCAATATTACTGTGCTGCT	miRNA	hsa-mir-16-2
t0000484	22	450	TGTAACAGCAACTCCATGTGGA	miRNA	hsa-mir-194-2
t0000500	23	447	CAGTGCAATAGTATTGTCAAAGC	miRNA	hsa-mir-301a
t0000502	23	445	GATATCATCATATACTGTAAGTT	miRNA	hsa-mir-144
t0000504	24	441	CAAAGTGCTGTTCGTGCAGGTAGT	miRNA	hsa-mir-93
t0000506	21	440	CCTGTACTGAGCTGCCCCGAG	miRNA	hsa-mir-486
t0000507	22	434	TACCACAGGGTAGAACCACGGA	miRNA	hsa-mir-140
t0000516	19	424	TGTACTGAGCTGCCCCGAG	miRNA	hsa-mir-486
t0000517	23	415	CAAAGTGCTTACAGTGCAGGTAG	miRNA	hsa-mir-17
t0000521	24	401	CAACGGAATCCCAAAAGCAGCTGT	miRNA	hsa-mir-191
t0000530	20	397	AGCTACATTGTCTGCTGGGT	miRNA	hsa-mir-221
t0000536	23	389	TTGGCAATGGTAGAACTCACACT	miRNA	hsa-mir-182
t0000544	19	389	TGAGGTAGTAGTTTGTACA	miRNA	hsa-let-7g
t0000546	21	388	CAAAGTGCTCATAGTGCAGGT	miRNA	hsa-mir-20b
t0000555	22	384	TAGCAGCACATAATGGTTTGTG	miRNA	hsa-mir-15a
t0000557	23	379	AAAAGCTGGGTTGAGAGGGCGAA	miRNA	hsa-mir-320a
t0000558	20	376	TGAGGTAGTAGGTTGTGTGG	miRNA	hsa-let-7b
t0000563	21	369	TGAGGTAGTAAGTTGTATTGT	miRNA	hsa-mir-98
t0000569	20	368	CCAATATTACTGTGCTGCTT	miRNA	hsa-mir-16-2
t0000573	21	366	AGCTACATTGTCTGCTGGGTT	miRNA	hsa-mir-221
t0000581	19	356	TATTGCACTCGTCCCGGCC	miRNA	hsa-mir-92b
t0000584	20	356	CTAGACTGAAGCTCCTTGAG	miRNA	hsa-mir-151a
t0000587	19	353	CATTATTACTTTTGGTACG	miRNA	hsa-mir-126
t0000601	20	343	TAGCAGCACGTAAATATTGG	miRNA	hsa-mir-16-2
t0000602	20	339	TTCAAGTAATCCAGGATAGG	miRNA	hsa-mir-26a-2
t0000606	21	337	TGTAAACATCCTACACTCAGC	miRNA	hsa-mir-30b
t0000620	21	334	GCCCTGACCTGTCCTGTTCTG	miRNA	hsa-mir-4732
t0000627	21	332	CAGTGCAATGATGAAAGGGCA	miRNA	hsa-mir-130b
t0000629	20	331	CATAAAGTAGAAAGCACTAC	miRNA	hsa-mir-142
t0000632	20	329	AAGCTGCCAGTTGAAGAACT	miRNA	hsa-mir-22
t0000636	20	327	AAAAGCTGGGTTGAGAGGGC	miRNA	hsa-mir-320b-2
t0000637	23	326	TGTAAACATCCTACACTCTCAGC	miRNA	hsa-mir-30c-1
t0000640	19	326	AATGGCGCCACTAGGGTTG	miRNA	hsa-mir-652
t0000647	20	325	AGAGGTAGTAGGTTGCATAG	miRNA	hsa-let-7d
t0000649	22	324	ACCAATATTACTGTGCTGCTTT	miRNA	hsa-mir-16-2
t0000650	19	323	CGAATCATTATTTGCTGCT	miRNA	hsa-mir-15b
t0000651	23	315	TAGCTTATCAGACTGATGTTGAC	miRNA	hsa-mir-21
t0000652	27	309	TTTGGCAATGGTAGAACTCACACTGGT	miRNA	hsa-mir-182
t0000653	20	306	TATTGCACTCGTCCCGGCCT	miRNA	hsa-mir-92b
t0000663	18	297	TGAGGTAGTAGGTTGTAT	miRNA	hsa-let-7c
t0000670	23	295	TGAGGTAGTAGATTGTATAGTTG	miRNA	hsa-let-7f-1
t0000675	19	294	CCCATAAAGTAGAAAGCAC	miRNA	hsa-mir-142
t0000693	23	288	TGTGCAAATCCATGCAAAACTGA	miRNA	hsa-mir-19b-2
t0000695	22	286	TTATAAAGCAATGAGACTGATT	miRNA	hsa-mir-340
t0000698	19	286	CATAAAGTAGAAAGCACTA	miRNA	hsa-mir-142
t0000703	21	280	TGACCTATGAATTGACAGCCA	miRNA	hsa-mir-192
t0000705	20	276	CTACAGTATAGATGATGTAC	miRNA	hsa-mir-144
t0000706	22	274	TCAAAGTGCTTACAGTGCAGGT	miRNA	hsa-mir-17
t0000713	21	274	ATCCTGTACTGAGCTGCCCCG	miRNA	hsa-mir-486
t0000719	20	268	TGAGGTAGTAAGTTGTATTG	miRNA	hsa-mir-98
t0000725	22	268	TCGTACCGTGAGTAATAATGCG	miRNA	hsa-mir-126
t0000726	24	266	TGAGGGGCAGAGAGCGAGACTTTT	miRNA	hsa-mir-423
t0000738	21	266	CACCAATATTACTGTGCTGCT	miRNA	hsa-mir-16-2
t0000739	24	265	AATGACACGATCACTCCCGTTGAG	miRNA	hsa-mir-425
t0000746	19	263	TTCAAGTAATCCAGGATAG	miRNA	hsa-mir-26a-2
t0000747	20	255	CAAAGTGCTGTTCGTGCAGG	miRNA	hsa-mir-93
t0000750	18	252	TACAGTACTGTGATAACT	miRNA	hsa-mir-101-2

t0000751	22	252	TCTCCCAACCCTTGTACCAGTG	miRNA	hsa-mir-150
t0000764	20	246	TGTAAACATCCTTGACTGGA	miRNA	hsa-mir-30e
t0000769	20	237	CTGACCTATGAATTGACAGC	miRNA	hsa-mir-192
t0000770	22	237	TCAGGCTCAGTCCCCTCCCGAT	miRNA	hsa-mir-484
t0000777	20	235	TAAAGTGCTTATAGTGCAGG	miRNA	hsa-mir-20a
t0000792	20	234	ATAAAGTAGAAAGCACTACT	miRNA	hsa-mir-142
t0000793	21	231	AAGGGCTTCCTCTCTGCAGGA	miRNA	hsa-mir-3158-2
t0000795	20	230	CAAAGTGCTTACAGTGCAGG	miRNA	hsa-mir-17
t0000800	23	229	ACCACAGGGTAGAACCACGGACA	miRNA	hsa-mir-140
t0000809	21	220	TGTAAACATCCCCGACTGGAA	miRNA	hsa-mir-30d
t0000817	18	218	TGAGGTAGTAGATTGTAT	miRNA	hsa-let-7f-2
t0000820	22	215	AACATTCATTGCTGTCGGTGGG	miRNA	hsa-mir-181b-2
t0000843	21	215	ACCACAGGGTAGAACCACGGA	miRNA	hsa-mir-140
t0000851	23	214	TATGGCACTGGTAGAATTCACTG	miRNA	hsa-mir-183
t0000857	22	212	AACATTCAACCTGTCGGTGAGT	miRNA	hsa-mir-181c
t0000859	21	211	CGTACCGTGAGTAATAATGCG	miRNA	hsa-mir-126
t0000861	18	208	CTGTACTGAGCTGCCCCG	miRNA	hsa-mir-486
t0000869	23	207	TACCACAGGGTAGAACCACGGAC	miRNA	hsa-mir-140
t0000873	21	206	AGCAGCACGTAAATATTGGCG	miRNA	hsa-mir-16-2
t0000880	20	200	AACATTCAACGCTGTCGGTG	miRNA	hsa-mir-181a-1
t0000881	19	197	TTATGGTTTGCCTGGGACT	miRNA	hsa-mir-584
t0000884	20	191	TTCACAGTGGCTAAGTTCCG	miRNA	hsa-mir-27a
t0000906	21	191	TTGGCAATGGTAGAACTCACA	miRNA	hsa-mir-182
t0000922	20	190	CAACGGAATCCCAAAAGCAG	miRNA	hsa-mir-191
t0000934	21	189	TCAAGTAATCCAGGATAGGCT	miRNA	hsa-mir-26a-2
t0000937	22	188	ATCAACAGACATTAATTGGGCG	miRNA	hsa-mir-421
t0000940	19	184	CTACAGTATAGATGATGTA	miRNA	hsa-mir-144
t0000942	23	179	TGAGGTAGTAGGTTGTATAGTTT	miRNA	hsa-let-7a-3
t0000946	22	177	CAAAGTGCTCATAGTGCAGGTA	miRNA	hsa-mir-20b
t0000959	21	177	TGTGCAAATCCATGCAAAACT	miRNA	hsa-mir-19b-2
t0000980	20	177	CTTTCAGTCGGATGTTTACA	miRNA	hsa-mir-30e
t0000990	23	175	TACTAGACTGAAGCTCCTTGAGG	miRNA	hsa-mir-151a
t0000993	22	175	TCTCACACAGAAATCGCACCCG	miRNA	hsa-mir-342
t0000994	21	170	ATATAATACAACCTGCTAAGT	miRNA	hsa-mir-374b
t0000999	21	169	AACATTCAACCTGTCGGTGAG	miRNA	hsa-mir-181c
t0001001	22	167	TAATGGTAATGGTTCTCTTGCT	miRNA	hsa-mir-451a
t0001020	21	166	AAAAGTGCTTACAGTGCAGGT	miRNA	hsa-mir-106a
t0001022	23	164	CCGCACTGTGGGTACTTGCTGCT	miRNA	hsa-mir-106b
t0001028	20	157	CCTGTACTGAGCTGCCCCGA	miRNA	hsa-mir-486
t0001031	20	156	TCGAGGAGCTCACAGTCTAG	miRNA	hsa-mir-151b
t0001043	23	150	TACCCATTGCATATCGGAGTTGT	miRNA	hsa-mir-660
t0001074	19	149	TAGCTTATCAGACTGATGT	miRNA	hsa-mir-21
t0001078	22	147	TCGAGGAGCTCACAGTCTAGTA	miRNA	hsa-mir-151a
t0001101	21	147	TGTAAACATCCTACACTCTCA	miRNA	hsa-mir-30c-1
t0001102	19	144	CAAAACGTGAGGCGCTGCT	miRNA	hsa-mir-424
t0001108	23	144	CAAAGTGCTCATAGTGCAGGTAG	miRNA	hsa-mir-20b
t0001110	21	141	GATATCATCATATACTGTAAG	miRNA	hsa-mir-144
t0001132	22	140	TAGTGCAATATTGCTTATAGGG	miRNA	hsa-mir-454
t0001133	20	140	TATTGCACATTACTAAGTTG	miRNA	hsa-mir-32
t0001154	18	139	TGTACTGAGCTGCCCCGA	miRNA	hsa-mir-486
t0001161	20	138	TATGGAAAGACTTTGCCACT	miRNA	hsa-mir-3688-2
t0001163	23	138	TGTGCAAATCTATGCAAAACTGA	miRNA	hsa-mir-19a
t0001165	24	137	TAGTGCAATATTGCTTATAGGGTT	miRNA	hsa-mir-454
t0001171	24	137	TAGCTTATCAGACTGATGTTGACT	miRNA	hsa-mir-21
t0001176	22	136	TGACCTATGAATTGACAGCCAG	miRNA	hsa-mir-192
t0001178	19	134	ACCAATATTACTGTGCTGC	miRNA	hsa-mir-16-2
t0001181	18	134	GGATATCATCATATACTG	miRNA	hsa-mir-144
t0001185	21	133	CAACGGAATCCCAAAAGCAGC	miRNA	hsa-mir-191

t0001187	20	131	AAGCTCGGTCTGAGGCCCCT	miRNA	hsa-mir-423
t0001191	20	131	ATGGTAATGGTTCTCTTGCT	miRNA	hsa-mir-451a
t0001199	18	130	ACCAATATTACTGTGCTG	miRNA	hsa-mir-16-2
t0001209	20	130	TAGCAGCACATCATGGTTTA	miRNA	hsa-mir-15b
t0001210	19	129	TAGCAGCACATCATGGTTT	miRNA	hsa-mir-15b
t0001215	22	128	AACCGTTACCATTACTGAGTTT	miRNA	hsa-mir-451a
t0001216	23	126	TGTAGTGTTTCCTACTTTATGGA	miRNA	hsa-mir-142
t0001218	21	125	TACCCTGTAGATCCGAATTTG	miRNA	hsa-mir-10a
t0001235	20	124	AACCGTTACCATTACTGAGT	miRNA	hsa-mir-451a
t0001244	21	123	ATGGCACTGGTAGAATTCACT	miRNA	hsa-mir-183
t0001257	22	122	AATTGCACGGTATCCATCTGTA	miRNA	hsa-mir-363
t0001259	21	122	AGCTCGGTCTGAGGCCCCTCA	miRNA	hsa-mir-423
t0001269	21	121	GCTGACTCCTAGTCCAGGGCT	miRNA	hsa-mir-345
t0001270	23	121	TGTAAACATCCTTGACTGGAAGC	miRNA	hsa-mir-30e
t0001273	23	120	TGTAAACATCCCCGACTGGAAGC	miRNA	hsa-mir-30d
t0001281	22	119	ATGGCACTGGTAGAATTCACTG	miRNA	hsa-mir-183
t0001285	19	118	CCGCACTGTGGGTACTTGC	miRNA	hsa-mir-106b
t0001288	21	117	ATTGCACTTGTCTCGGTCTGA	miRNA	hsa-mir-25
t0001295	20	116	CTGTGCGTGTGACAGCGGCT	miRNA	hsa-mir-210
t0001297	22	116	AGTGTTTCCTACTTTATGGATG	miRNA	hsa-mir-142
t0001305	22	115	CGGGGCAGCTCAGTACAGGATA	miRNA	hsa-mir-486
t0001313	23	113	TCTCACACAGAAATCGCACCCGT	miRNA	hsa-mir-342
t0001315	18	113	GTACAGTACTGTGATAAC	miRNA	hsa-mir-101-2
t0001326	21	111	TTATGGTTTGCCTGGGACTGA	miRNA	hsa-mir-584
t0001333	21	110	AACCGTTACCATTACTGAGTT	miRNA	hsa-mir-451a
t0001339	20	110	TATGGCACTGGTAGAATTCA	miRNA	hsa-mir-183
t0001357	20	107	ACTGGACTTGGAGTCAGAAG	miRNA	hsa-mir-378c
t0001364	21	106	CTTATCAGATTGTATTGTAAT	miRNA	hsa-mir-374a
t0001365	22	102	AGCTCGGTCTGAGGCCCCTCAG	miRNA	hsa-mir-423
t0001393	22	102	CAGTGCAATAGTATTGTCAAAG	miRNA	hsa-mir-301a
t0001404	19	100	TGTAAACATCCTACACTCT	miRNA	hsa-mir-30c-1
t0001440	20	98	TCAGGCTCAGTCCCCTCCCG	miRNA	hsa-mir-484
t0001444	19	98	TGAGGGGCAGAGAGCGAGA	miRNA	hsa-mir-423
t0001468	22	97	ATGAGGTAGTAGATTGTATAGT	miRNA	hsa-let-7f-2
t0001491	24	96	CTGACCTATGAATTGACAGCCAGT	miRNA	hsa-mir-192
t0001493	19	96	TCACAGTGAACCGGTCTCT	miRNA	hsa-mir-128-2
t0001496	20	96	TCAGTGCACTACAGAACTTT	miRNA	hsa-mir-148a
t0001509	21	96	TTATAATACAACCTGATAAGT	miRNA	hsa-mir-374a
t0001515	20	95	CAGTGCAATGATGAAAGGGC	miRNA	hsa-mir-130b
t0001518	23	94	ATGCACCTGGGCAAGGATTCTGA	miRNA	hsa-mir-500a
t0001520	18	94	TAGCAGCACATCATGGTT	miRNA	hsa-mir-15b
t0001531	20	92	TCTCTCGGCTCCTCGCGGCT	miRNA	hsa-mir-3615
t0001536	23	91	TGAGAACTGAATTCCATAGGCTG	miRNA	hsa-mir-146b
t0001539	23	89	TGTAGAGCAGGGAGCAGGAAGCT	miRNA	hsa-mir-4732
t0001561	22	89	CTGAGGTAGTAGTTTGTACAGT	miRNA	hsa-let-7g
t0001566	20	88	TTATGGTTTGCCTGGGACTG	miRNA	hsa-mir-584
t0001602	18	88	TCGAGGAGCTCACAGTCT	miRNA	hsa-mir-151b
t0001607	23	87	TGAGGTAGTAGTTTGTGCTGTTG	miRNA	hsa-let-7i
t0001617	19	86	AGAGGTAGTAGGTTGCATA	miRNA	hsa-let-7d
t0001626	21	85	TGTAAACATCCTTGACTGGAA	miRNA	hsa-mir-30e
t0001647	19	85	TCAGTGCACTACAGAACTT	miRNA	hsa-mir-148a
t0001658	20	84	CTGCGCAAGCTACTGCCTTG	miRNA	hsa-let-7i
t0001677	21	84	CTGGACTTGGAGTCAGAAGGC	miRNA	hsa-mir-378a
t0001684	22	84	CGCATCCCCTAGGGCATTGGTG	miRNA	hsa-mir-324
t0001688	21	83	TCTCCCAACCCTTGTACCAGT	miRNA	hsa-mir-150
t0001693	18	82	TGAGGTAGTAAGTTGTAT	miRNA	hsa-mir-98
t0001699	20	81	CTATACGACCTGCTGCCTTT	miRNA	hsa-let-7d
t0001710	23	81	AACATTCATTGCTGTCGGTGGGT	miRNA	hsa-mir-181b-[illegible]

t0001728	21	80	AGCTGCCAGTTGAAGAACTGT	miRNA	hsa-mir-22
t0001735	20	79	ACTCGGCGTGGCGTCGGTCG	miRNA	hsa-mir-1307
t0001756	21	78	TGAGAACCACGTCTGCTCTGA	miRNA	hsa-mir-589
t0001769	21	78	TCTGGGCAACAAAGTGAGACC	miRNA	hsa-mir-1285-2
t0001784	20	77	GAGGTAGTAGATTGTATAGT	miRNA	hsa-let-7f-2
t0001792	21	77	TTTAGTAATGGTAATGGTTCT	miRNA	hsa-mir-451a
t0001803	21	76	CGAATCATTATTTGCTGCTCT	miRNA	hsa-mir-15b
t0001822	21	75	TACCGCACTGTGGGTACTTGC	miRNA	hsa-mir-106b
t0001829	19	74	TAAAGTGCTGACAGTGCAG	miRNA	hsa-mir-106b
t0001833	20	72	CAAAGAATTCTCCTTTTGGG	miRNA	hsa-mir-186
t0001848	20	72	TCGTACCGTGAGTAATAATG	miRNA	hsa-mir-126
t0001876	22	72	CTGTGCGTGTGACAGCGGCTGA	miRNA	hsa-mir-210
t0001916	24	72	TAAAGTGCTTATAGTGCAGGTAGT	miRNA	hsa-mir-20a
t0001917	21	71	ACTGCAGTGAAGGCACTTGTA	miRNA	hsa-mir-17
t0001922	22	70	TGTAAACATCCTTGACTGGAAG	miRNA	hsa-mir-30e
t0001925	18	70	TCAGTGCACTACAGAACT	miRNA	hsa-mir-148a
t0001946	23	70	TCAGTGCACTACAGAACTTTGTC	miRNA	hsa-mir-148a
t0001956	22	69	GTAGTGTTTCCTACTTTATGGA	miRNA	hsa-mir-142
t0001967	19	69	TTCAAGTAATTCAGGATAG	miRNA	hsa-mir-26b
t0001968	21	68	TGCGGGGCTAGGGCTAACAGC	miRNA	hsa-mir-744
t0001970	22	68	TGAGACCTCTGGGTTCTGAGCT	miRNA	hsa-mir-769
t0001976	24	66	CAAAGTGCTTACAGTGCAGGTAGT	miRNA	hsa-mir-17
t0002000	23	66	AATGACACGATCACTCCCGTTGA	miRNA	hsa-mir-425
t0002005	22	65	AAAAGTGCTTACAGTGCAGGTA	miRNA	hsa-mir-106a
t0002058	23	64	CATGCCTTGAGTGTAGGACCGTT	miRNA	hsa-mir-532
t0002061	21	63	ACTAGACTGAAGCTCCTTGAG	miRNA	hsa-mir-151a
t0002074	22	63	TGTCAGTTTGTCAAATACCCCA	miRNA	hsa-mir-223
t0002089	21	63	TACTAGACTGAAGCTCCTTGA	miRNA	hsa-mir-151a
t0002108	24	62	TACTAGACTGAAGCTCCTTGAGGA	miRNA	hsa-mir-151a
t0002110	19	61	CATGCCTTGAGTGTAGGAC	miRNA	hsa-mir-532
t0002119	21	61	TAGCAGCACATCATGGTTTAC	miRNA	hsa-mir-15b
t0002157	22	60	CTGACCTATGAATTGACAGCCA	miRNA	hsa-mir-192
t0002163	23	60	AACGGAATCCCAAAAGCAGCTGT	miRNA	hsa-mir-191
t0002175	22	60	TTATAATACAACCTGATAAGTG	miRNA	hsa-mir-374a
t0002193	22	60	TACCCTGTAGATCCGAATTTGT	miRNA	hsa-mir-10a
t0002194	20	60	TGAGATGAAGCACTGTAGCT	miRNA	hsa-mir-143
t0002200	23	59	CTGAGGTAGTAGTTTGTGCTGTT	miRNA	hsa-let-7i
t0002202	22	58	TCAGTGCATCACAGAACTTTGT	miRNA	hsa-mir-148b
t0002213	18	58	TAGCTTATCAGACTGATG	miRNA	hsa-mir-21
t0002228	19	57	ATTGCACGGTATCCATCTG	miRNA	hsa-mir-363
t0002276	18	57	ATTGCACTTGTCCCGGCC	miRNA	hsa-mir-92a-2
t0002277	20	56	ACCACTGACCGTTGACTGTA	miRNA	hsa-mir-181a-2
t0002296	20	56	ATGCCTTGAGTGTAGGACCG	miRNA	hsa-mir-532
t0002302	18	56	GTTACCATTACTGAGTTT	miRNA	hsa-mir-451a
t0002314	22	56	TAAACATCCTTGACTGGAAGCT	miRNA	hsa-mir-30e
t0002332	21	55	ACTGTGCGTGTGACAGCGGCT	miRNA	hsa-mir-210
t0002335	23	55	AAGCTGCCAGTTGAAGAACTGTT	miRNA	hsa-mir-22
t0002338	24	55	AGCAGCATTGTACAGGGCTATGAA	miRNA	hsa-mir-103a-1
t0002342	18	55	GGTAGTAGATTGTATAGT	miRNA	hsa-let-7f-2
t0002351	24	55	AAAAGCTGGGTTGAGAGGGCGAAA	miRNA	hsa-mir-320a
t0002362	21	55	ATCAACAGACATTAATTGGGC	miRNA	hsa-mir-421
t0002366	24	54	AACATTCAACGCTGTCGGTGAGTT	miRNA	hsa-mir-181a-1
t0002368	22	53	AAGGGCTTCCTCTCTGCAGGAC	miRNA	hsa-mir-3158-2
t0002369	19	53	AACATTCAACGCTGTCGGT	miRNA	hsa-mir-181a-1
t0002382	22	53	AAAAGTACTTGCGGATTTTGCT	miRNA	hsa-mir-548k
t0002414	23	53	CCCATAAAGTAGAAAGCACTACT	miRNA	hsa-mir-142
t0002415	22	53	CCCATAAAGTAGAAAGCACTAC	miRNA	hsa-mir-142
t0002417	18	52	CATTGCACTTGTCTCGGT	miRNA	hsa-mir-25

t0002423	22	52	ACATTCATTGCTGTCGGTGGGT	miRNA	hsa-mir-181b-2
t0002429	22	52	TTCACAGTGGCTAAGTTCTGCA	miRNA	hsa-mir-27b
t0002437	21	52	ACATTCAACGCTGTCGGTGAG	miRNA	hsa-mir-181a-1
t0002441	19	52	TATGGCACTGGTAGAATTC	miRNA	hsa-mir-183
t0002443	19	51	CCATAAAGTAGAAAGCACT	miRNA	hsa-mir-142
t0002444	21	51	CTGTGCGTGTGACAGCGGCTG	miRNA	hsa-mir-210
t0002449	19	50	TAGCAGCACGTAAATATTG	miRNA	hsa-mir-16-2
t0002472	19	50	TCTGGGCAACAAAGTGAGA	miRNA	hsa-mir-1285-2
t0002490	22	49	ACTAGACTGAAGCTCCTTGAGG	miRNA	hsa-mir-151a
t0002504	23	49	CAGTGCAATGTTAAAAGGGCATT	miRNA	hsa-mir-130a
t0002514	19	49	ACTGCAGTGAAGGCACTTG	miRNA	hsa-mir-17
t0002549	20	47	TGTAAACATCCTACACTCAG	miRNA	hsa-mir-30b
t0002563	20	46	AAAGTGCTGTTCGTGCAGGT	miRNA	hsa-mir-93
t0002572	23	46	CTGAGGTAGTAGTTTGTACAGTT	miRNA	hsa-let-7g
t0002636	22	46	CACCAATATTACTGTGCTGCTT	miRNA	hsa-mir-16-2
t0002682	19	45	TTCACAGTGGCTAAGTTCC	miRNA	hsa-mir-27a
t0002686	21	45	TAGCACCATTTGAAATCGGTT	miRNA	hsa-mir-29c
t0002703	22	45	AAAGTGCTGTTCGTGCAGGTAG	miRNA	hsa-mir-93
t0002744	23	45	GAACATTCAACGCTGTCGGTGAG	miRNA	hsa-mir-181a-1
t0002746	21	45	CTTTCAGTCGGATGTTTACAG	miRNA	hsa-mir-30e
t0002755	21	45	AACGGAATCCCAAAAGCAGCT	miRNA	hsa-mir-191
t0002759	21	44	ATGCCTTGAGTGTAGGACCGT	miRNA	hsa-mir-532
t0002760	22	44	CAGTGCAATGTTAAAAGGGCAT	miRNA	hsa-mir-130a
t0002765	23	44	TGAGGTAGTAGGTTGTGTGGTTT	miRNA	hsa-let-7b
t0002783	21	44	GAGGTAGTAGTTTGTGCTGTT	miRNA	hsa-let-7i
t0002789	21	44	AGGGGCTGGCTTTCCTCTGGT	miRNA	hsa-mir-185
t0002804	20	44	CATTATTACTTTTGGTACGC	miRNA	hsa-mir-126
t0002806	22	43	TGTAAACATCCTACACTCTCAG	miRNA	hsa-mir-30c-1
t0002821	22	43	ATATAATACAACCTGCTAAGTG	miRNA	hsa-mir-374b
t0002832	20	43	CAAAGTGCTCATAGTGCAGG	miRNA	hsa-mir-20b
t0002860	18	42	TGAGGTAGTAGTTTGTAC	miRNA	hsa-let-7g
t0002867	20	42	ATTATTACTTTTGGTACGCG	miRNA	hsa-mir-126
t0002892	18	42	CTACAGTATAGATGATGT	miRNA	hsa-mir-144
t0002935	21	42	CCGCACTGTGGGTACTTGCTG	miRNA	hsa-mir-106b
t0002951	23	42	TAGGTAGTTTCCTGTTGTTGGGA	miRNA	hsa-mir-196b
t0002963	22	42	TAAAGTGCTGACAGTGCAGATA	miRNA	hsa-mir-106b
t0002965	23	41	CTAGCAGCACGTAAATATTGGCG	miRNA	hsa-mir-16-2
t0002970	20	41	AATGGCGCCACTAGGGTTGT	miRNA	hsa-mir-652
t0002972	19	41	TAGCAGCACATAATGGTTT	miRNA	hsa-mir-15a
t0002976	21	41	GAGGTAGTAGATTGTATAGTT	miRNA	hsa-let-7f-2
t0002980	21	41	TTTGGCACTAGCACATTTTTG	miRNA	hsa-mir-96
t0002997	20	41	TTGCACTTGTCCCGGCCTGT	miRNA	hsa-mir-92a-2
t0003003	23	40	ACTGCTGAGCTAGCACTTCCCGA	miRNA	hsa-mir-93
t0003015	21	40	AATGGCGCCACTAGGGTTGTG	miRNA	hsa-mir-652
t0003027	18	39	AATTGCACGGTATCCATC	miRNA	hsa-mir-363
t0003044	20	39	TCACAGTGAACCGGTCTCTT	miRNA	hsa-mir-128-2
t0003058	25	39	TTGGCAATGGTAGAACTCACACTGG	miRNA	hsa-mir-182
t0003080	22	39	CCTCCCACACCCAAGGCTTGCA	miRNA	hsa-mir-532
t0003082	24	38	GAAGTTCTGTTATACACTCAGGCT	miRNA	hsa-mir-148b
t0003108	22	38	CTTTCAGTCGGATGTTTACAGC	miRNA	hsa-mir-30e
t0003130	22	38	CTCGGGGCAGCTCAGTACAGGA	miRNA	hsa-mir-486
t0003142	23	38	TACCCTGTAGATCCGAATTTGTG	miRNA	hsa-mir-10a
t0003168	19	37	ATTGCACTTGTCTCGGTCT	miRNA	hsa-mir-25
t0003171	18	37	AGCACGTAAATATTGGCG	miRNA	hsa-mir-16-2
t0003177	22	37	CACCTTGCGCTACTCAGGTCTG	miRNA	hsa-mir-3200
t0003199	21	37	AAAGAATTCTCCTTTTGGGCT	miRNA	hsa-mir-186
t0003209	21	37	TGTCTTACTCCCTCAGGCACA	miRNA	hsa-mir-550a-3
t0003219	19	37	TGACCTATGAATTGACAGC	miRNA	hsa-mir-192

t0003220	22	36	GAACATTCAACGCTGTCGGTGA	miRNA	hsa-mir-181a-1
t0003237	21	36	CCAATATTACTGTGCTGCTTT	miRNA	hsa-mir-16-2
t0003241	24	36	AAGCTCGGTCTGAGGCCCCTCAGT	miRNA	hsa-mir-423
t0003278	24	36	TGTAAACATCCTCGACTGGAAGCT	miRNA	hsa-mir-30a
t0003285	22	36	ACATTCAACGCTGTCGGTGAGT	miRNA	hsa-mir-181a-1
t0003293	18	36	TATTGCACTTGTCCCGGC	miRNA	hsa-mir-92a-2
t0003302	21	36	TATTGCACTCGTCCCGGCCTC	miRNA	hsa-mir-92b
t0003303	21	36	TCAAAGTGCTTACAGTGCAGG	miRNA	hsa-mir-17
t0003304	22	36	TACCCTGTAGAACCGAATTTGT	miRNA	hsa-mir-10b
t0003319	21	35	TGTGCAAATCTATGCAAAACT	miRNA	hsa-mir-19a
t0003322	21	35	TGAGGTAGTAGGTTGTATGGT	miRNA	hsa-let-7c
t0003341	20	35	TGTGCAAATCCATGCAAAAC	miRNA	hsa-mir-19b-2
t0003373	20	35	CGAATCATTATTTGCTGCTC	miRNA	hsa-mir-15b
t0003380	21	35	ACTCGGCGTGGCGTCGGTCGT	miRNA	hsa-mir-1307
t0003396	23	35	TGAGGTAGTAGTTTGTACAGTTT	miRNA	hsa-let-7g
t0003397	20	35	TCTCCCAACCCTTGTACCAG	miRNA	hsa-mir-150
t0003405	22	35	TGATATGTTTGATATATTAGGT	miRNA	hsa-mir-190a
t0003418	32	35	TTTGGCAATGGTAGAACTCACACTGGTGA	miRNA	hsa-mir-182
t0003419	21	34	CTCGGCGTGGCGTCGGTCGTG	miRNA	hsa-mir-1307
t0003433	21	34	TGAGATGAAGCACTGTAGCTC	miRNA	hsa-mir-143
t0003437	19	34	GGTAGTAGATTGTATAGTT	miRNA	hsa-let-7f-2
t0003475	21	34	CACTAGATTGTGAGCTCCTGG	miRNA	hsa-mir-28
t0003481	21	34	TGGCTCAGTTCAGCAGGAACA	miRNA	hsa-mir-24-2
t0003506	20	33	TAGCACCATTTGAAATCGGT	miRNA	hsa-mir-29c
t0003530	19	33	CTAGACTGAAGCTCCTTGA	miRNA	hsa-mir-151a
t0003546	21	33	TACGTCATCGTTGTCATCGTC	miRNA	hsa-mir-598
t0003590	21	32	AATGACACGATCACTCCCGTT	miRNA	hsa-mir-425
t0003617	19	32	ATTGCACTTGTCCCGGCCT	miRNA	hsa-mir-92a-2
t0003634	21	32	TCACAGTGAACCGGTCTCTTT	miRNA	hsa-mir-128-2
t0003645	20	32	ATTGCACGGTATCCATCTGT	miRNA	hsa-mir-363
t0003662	23	32	TCAAAGTGCTTACAGTGCAGGTA	miRNA	hsa-mir-17
t0003664	19	32	CAATGGTAGAACTCACACT	miRNA	hsa-mir-182
t0003676	24	32	TACCACAGGGTAGAACCACGGACA	miRNA	hsa-mir-140
t0003719	18	31	CGAATCATTATTTGCTGC	miRNA	hsa-mir-15b
t0003722	19	31	GGTAGTAGGTTGTATAGTT	miRNA	hsa-let-7a-3
t0003739	21	31	CTGTACTGAGCTGCCCCGAGG	miRNA	hsa-mir-486
t0003757	21	31	CTATACGACCTGCTGCCTTTC	miRNA	hsa-let-7d
t0003783	20	31	AAAAGTGCTTACAGTGCAGG	miRNA	hsa-mir-106a
t0003795	22	30	TGCGGGGCTAGGGCTAACAGCA	miRNA	hsa-mir-744
t0003800	18	30	TAGCAGCACATAATGGTT	miRNA	hsa-mir-15a
t0003821	18	30	TTCAAGTAATTCAGGATA	miRNA	hsa-mir-26b
t0003893	24	30	TAGCAGCACGTAAATATTGGCGTT	miRNA	hsa-mir-16-1
t0003912	20	30	TGTAAACATCCTACACTCTC	miRNA	hsa-mir-30c-1
t0003926	21	29	TGTAACAGCAACTCCATGTGG	miRNA	hsa-mir-194-2
t0003938	18	29	GCAATGGTAGAACTCACA	miRNA	hsa-mir-182
t0003950	22	29	ACAAAAAAAAAAGCCCAACCCT	miRNA	hsa-mir-3613
t0003970	21	29	TAAGGTGCATCTAGTGCAGAT	miRNA	hsa-mir-18a
t0003998	20	29	TAAGGTGCATCTAGTGCAGA	miRNA	hsa-mir-18a
t0004004	20	29	TCAAGTAATCCAGGATAGGC	miRNA	hsa-mir-26a-2
t0004011	22	29	TCTGTGAGACCAAAGAACTACT	miRNA	hsa-mir-4677
t0004050	23	28	ATGGCACTGGTAGAATTCACTGT	miRNA	hsa-mir-183
t0004067	24	28	TAGCAGCACGTAAATATTGGCGTA	miRNA	hsa-mir-16-2
t0004091	20	28	ATTCTAATTTCTCCACGTCT	miRNA	hsa-mir-576
t0004112	23	28	AACATTCAACCTGTCGGTGAGTT	miRNA	hsa-mir-181c
t0004119	21	28	CTATACAATCTACTGTCTTTC	miRNA	hsa-let-7a-3
t0004121	23	28	TTTGGCACTAGCACATTTTTGCT	miRNA	hsa-mir-96
t0004135	21	28	TATGGCACTGGTAGAATTCAC	miRNA	hsa-mir-183
t0004137	21	28	ACAGTACTGTGATAACTGAAG	miRNA	hsa-mir-101-2

t0004169	22	27 GTGAGGTAGTAGGTTGTATAGT	miRNA	hsa-let-7a-3
t0004173	18	27 GTAGTAGTTTGTGCTGTT	miRNA	hsa-let-7i
t0004211	22	27 GAAAAGCTGGGTTGAGAGGGCG	miRNA	hsa-mir-320a
t0004218	20	27 TACCCTGTAGATCCGAATTT	miRNA	hsa-mir-10a
t0004225	21	27 ACATTCATTGCTGTCGGTGGG	miRNA	hsa-mir-181b-[illegible]
t0004231	22	27 TGAGAACTGAATTCCATGGGTT	miRNA	hsa-mir-146a
t0004277	21	27 GAGGTAGTAGGTTGTATAGTT	miRNA	hsa-let-7a-3
t0004291	20	27 CAGAGTCTCGTTCTGTTGCC	miRNA	hsa-mir-1273c
t0004294	20	27 AATGACACGATCACTCCCGT	miRNA	hsa-mir-425
t0004318	23	26 ATGAGGTAGTAGATTGTATAGTT	miRNA	hsa-let-7f-2
t0004340	24	26 TGAGAACTGAATTCCATAGGCTGT	miRNA	hsa-mir-146b
t0004355	22	26 CATAAAGTAGAAAGCACTACTA	miRNA	hsa-mir-142
t0004366	23	26 AATTGCACGGTATCCATCTGTAA	miRNA	hsa-mir-363
t0004380	19	26 AAGCTGCCAGTTGAAGAAC	miRNA	hsa-mir-22
t0004388	23	26 ATGACACGATCACTCCCGTTGAG	miRNA	hsa-mir-425
t0004400	22	26 TGAGAACTGAATTCCATAGGCT	miRNA	hsa-mir-146b
t0004413	21	26 CCACTGCCCCAGGTGCTGCTG	miRNA	hsa-mir-324
t0004421	18	26 AATGGTAGAACTCACACT	miRNA	hsa-mir-182
t0004424	21	26 TAATGGTAATGGTTCTCTTGC	miRNA	hsa-mir-451a
t0004446	22	25 ACCCTGTAGATCCGAATTTGTG	miRNA	hsa-mir-10a
t0004449	18	25 GCACTTGTCCCGGCCTGT	miRNA	hsa-mir-92a-2
t0004491	19	25 GTGCATTGCTGTTGCATTG	miRNA	hsa-mir-33b
t0004547	23	25 TTCAAGTAATCCAGGATAGGCTG	miRNA	hsa-mir-26a-2
t0004560	19	25 CGTTACCATTACTGAGTTT	miRNA	hsa-mir-451a
t0004571	20	25 TCTTCTCTGTTTTGGCCATG	miRNA	hsa-mir-942
t0004578	20	25 CTATACAACCTACTGCCTTC	miRNA	hsa-let-7b
t0004588	22	25 AGCTACATTGTCTGCTGGGTTT	miRNA	hsa-mir-221
t0004589	20	25 GAGGTAGTAGGTTGTATAGT	miRNA	hsa-let-7a-3
t0004621	20	24 ACCCTGTAGATCCGAATTTG	miRNA	hsa-mir-10a
t0004638	18	24 GTACTGTGATAACTGAAG	miRNA	hsa-mir-101-2
t0004644	19	24 GGTAGTAGTTTGTGCTGTT	miRNA	hsa-let-7i
t0004695	20	24 CACCCGGCTGTGTGCACATG	miRNA	hsa-mir-941-4
t0004737	19	24 GATATCATCATATACTGTA	miRNA	hsa-mir-144
t0004780	20	24 TCTGGGCAACAAAGTGAGAC	miRNA	hsa-mir-1285-[illegible]
t0004785	22	24 TCTCTCGGCTCCTCGCGGCTCG	miRNA	hsa-mir-3615
t0004790	20	24 AAAGCTGGGTTGAGAGGGCG	miRNA	hsa-mir-320a
t0004817	21	23 TTGCACTTGTCCCGGCCTGTT	miRNA	hsa-mir-92a-1
t0004825	20	23 GCCCTGACCTGTCCTGTTCT	miRNA	hsa-mir-4732
t0004855	18	23 GCACTTGTCTCGGTCTGA	miRNA	hsa-mir-25
t0004866	20	23 AACCCGTAGATCCGAACTTG	miRNA	hsa-mir-100
t0004889	18	23 AACATTCAACGCTGTCGG	miRNA	hsa-mir-181a-1
t0004891	20	23 AGCTCGGTCTGAGGCCCCTC	miRNA	hsa-mir-423
t0004896	23	23 GAAGTTCTGTTATACACTCAGGC	miRNA	hsa-mir-148b
t0004912	20	23 TTATAAAGCAATGAGACTGA	miRNA	hsa-mir-340
t0004922	24	23 TTCAAGTAATCCAGGATAGGCTGT	miRNA	hsa-mir-26a-2
t0004930	22	23 CTATACGACCTGCTGCCTTTCT	miRNA	hsa-let-7d
t0004932	18	23 GGTAGTAGGTTGTATAGT	miRNA	hsa-let-7a-3
t0004960	22	23 TGAGGTAGTAGGTTGTATGGTT	miRNA	hsa-let-7c
t0005015	20	23 TCTCCCTTCCTGCCCTGGCT	miRNA	hsa-mir-4685
t0005016	22	22 ACTGCCCTAAGTGCTCCTTCTG	miRNA	hsa-mir-18a
t0005046	20	22 ATATAATACAACCTGCTAAG	miRNA	hsa-mir-374b
t0005053	20	22 AGCTGCCAGTTGAAGAACTG	miRNA	hsa-mir-22
t0005060	21	22 ACCCTATCAATATTGTCTCTG	miRNA	hsa-mir-454
t0005106	23	22 CTCGTACCGTGAGTAATAATGCG	miRNA	hsa-mir-126
t0005141	20	22 GAGGGGCAGAGAGCGAGACT	miRNA	hsa-mir-423
t0005190	20	22 TAACGCATAATATGGACATG	miRNA	hsa-mir-3912
t0005197	21	22 CCTGTTCTCCATTACTTGGCT	miRNA	hsa-mir-26b
t0005221	21	22 AAAAGCTGGGTTGAGAGGGCA	miRNA	hsa-mir-320b-[illegible]

t0005227	18	21	GCAGCATTGTACAGGGCT	miRNA	hsa-mir-107
t0005254	23	21	TAAGGTGCATCTAGTGCAGATAG	miRNA	hsa-mir-18a
t0005261	19	21	TGTAAACATCCTTGACTGG	miRNA	hsa-mir-30e
t0005316	22	21	ACTGCTGAGCTAGCACTTCCCG	miRNA	hsa-mir-93
t0005388	20	21	CAACACCAGTCGATGGGCTG	miRNA	hsa-mir-21
t0005392	22	21	ATTGCACTTGTCCCGGCCTGTG	miRNA	hsa-mir-92a-2
t0005403	19	21	TTCACAGTGGCTAAGTTCT	miRNA	hsa-mir-27b
t0005436	24	21	TTGGCAATGGTAGAACTCACACTG	miRNA	hsa-mir-182
t0005478	22	20	TATTGCACTCGTCCCGGCCTCC	miRNA	hsa-mir-92b
t0005505	19	20	CTTATCAGATTGTATTGTA	miRNA	hsa-mir-374a
t0005519	24	20	TCTCACACAGAAATCGCACCCGTC	miRNA	hsa-mir-342
t0005522	23	20	GAAAAGCTGGGTTGAGAGGGCGA	miRNA	hsa-mir-320a
t0005530	22	20	TGGCAATGGTAGAACTCACACT	miRNA	hsa-mir-182
t0005597	22	20	CTCGAGGAGCTCACAGTCTAGT	miRNA	hsa-mir-151a
t0005629	22	20	GTAGAGGAGATGGCGCAGGGGA	miRNA	hsa-mir-877
t0005632	18	20	TATGGCACTGGTAGAATT	miRNA	hsa-mir-183
t0005652	21	20	ATTGCACGGTATCCATCTGTA	miRNA	hsa-mir-363
t0005695	20	19	ATTATGGTTTGCCTGGGACT	miRNA	hsa-mir-584
t0005702	24	19	TATGGCACTGGTAGAATTCACTGT	miRNA	hsa-mir-183
t0005724	20	19	TGGCAATGGTAGAACTCACA	miRNA	hsa-mir-182
t0005780	20	19	CCACTGCCCCAGGTGCTGCT	miRNA	hsa-mir-324
t0005782	22	19	GTAAACATCCTTGACTGGAAGC	miRNA	hsa-mir-30e
t0005787	22	19	TGTAGTGTTTCCTACTTTATGG	miRNA	hsa-mir-142
t0005798	23	19	CAAAGAATTCTCCTTTTGGGCTT	miRNA	hsa-mir-186
t0005841	18	19	CCGTTACCATTACTGAGT	miRNA	hsa-mir-451a
t0005843	18	19	CCCATAAAGTAGAAAGCA	miRNA	hsa-mir-142
t0005886	24	19	TGATATGTTTGATATATTAGGTTG	miRNA	hsa-mir-190a
t0005899	21	19	TGAGGTAGGAGGTTGTATAGT	miRNA	hsa-let-7e
t0005915	19	19	CTATACGACCTGCTGCCTT	miRNA	hsa-let-7d
t0005927	21	19	TTTGGGACTGATCTTGATGTC	miRNA	hsa-mir-3913-2
t0005935	22	19	AAAGTTCTGAGACACTCCGACT	miRNA	hsa-mir-148a
t0006013	22	19	TCTGGGCAACAAAGTGAGACCT	miRNA	hsa-mir-1285-2
t0006018	22	18	CACGCTCATGCACACACCCACA	miRNA	hsa-mir-574
t0006024	23	18	TAAAGTGCTGACAGTGCAGATAG	miRNA	hsa-mir-106b
t0006049	19	18	ATGCCTTGAGTGTAGGACC	miRNA	hsa-mir-532
t0006058	21	18	CACCTTGCGCTACTCAGGTCT	miRNA	hsa-mir-3200
t0006173	20	18	GCTGACTCCTAGTCCAGGGC	miRNA	hsa-mir-345
t0006188	21	18	AATGCACCTGGGCAAGGATTC	miRNA	hsa-mir-502
t0006243	19	18	CTTTCAGTCGGATGTTTAC	miRNA	hsa-mir-30e
t0006265	19	18	TCTGTGAGACCAAAGAACT	miRNA	hsa-mir-4677
t0006273	21	18	AAAGCTGGGTTGAGAGGGCGA	miRNA	hsa-mir-320a
t0006282	20	18	GCAGCACGTAAATATTGGCG	miRNA	hsa-mir-16-2
t0006287	21	18	CAGCATTGTACAGGGCTATGA	miRNA	hsa-mir-103a-1
t0006309	23	18	ACCAATATTACTGTGCTGCTTTA	miRNA	hsa-mir-16-2
t0006313	22	17	CCAATATTACTGTGCTGCTTTA	miRNA	hsa-mir-16-2
t0006321	21	17	GAAAAGCTGGGTTGAGAGGGC	miRNA	hsa-mir-320b-2
t0006341	19	17	AAGTAATCCAGGATAGGCT	miRNA	hsa-mir-26a-2
t0006395	20	17	GATATCATCATATACTGTAA	miRNA	hsa-mir-144
t0006416	19	17	CGGGGCAGCTCAGTACAGG	miRNA	hsa-mir-486
t0006444	22	17	TAGCACCATTTGAAATCGGTTA	miRNA	hsa-mir-29c
t0006490	18	17	CGTTACCATTACTGAGTT	miRNA	hsa-mir-451a
t0006505	20	17	TATCATCATATACTGTAAGT	miRNA	hsa-mir-144
t0006526	19	17	TTGGCAATGGTAGAACTCA	miRNA	hsa-mir-182
t0006564	23	17	GCCCTGACCTGTCCTGTTCTGCC	miRNA	hsa-mir-4732
t0006586	21	17	CCACAGGGTAGAACCACGGAC	miRNA	hsa-mir-140
t0006598	23	17	ACTAGACTGAAGCTCCTTGAGGA	miRNA	hsa-mir-151a
t0006624	21	17	CTATACAACCTACTGCCTTCC	miRNA	hsa-let-7b
t0006631	21	17	TAGCACCATCTGAAATCGGTT	miRNA	hsa-mir-29a

t0006635	18	16	TATTGCACTCGTCCCGGC	miRNA	hsa-mir-92b
t0006689	24	16	TAGCAGCACATAATGGTTTGTGGA	miRNA	hsa-mir-15a
t0006698	21	16	TGAGAACTGAATTCCATGGGT	miRNA	hsa-mir-146a
t0006740	21	16	CGGGGCCGTAGCACTGTCTGA	miRNA	hsa-mir-128-1
t0006766	19	16	AAAGCTGGGTTGAGAGGGC	miRNA	hsa-mir-320b-[illegible]
t0006774	22	16	AAGCTCGGTCTGAGGCCCCTCA	miRNA	hsa-mir-423
t0006802	22	16	TCTTCTCTGTTTTGGCCATGTG	miRNA	hsa-mir-942
t0006811	24	16	TAAAGTGCTGACAGTGCAGATAGT	miRNA	hsa-mir-106b
t0006828	21	16	TCGGGGCAGCTCAGTACAGGA	miRNA	hsa-mir-486
t0006845	21	16	GGCAATGGTAGAACTCACACT	miRNA	hsa-mir-182
t0006919	24	16	GATATCATCATATACTGTAAGTTT	miRNA	hsa-mir-144
t0006934	21	16	TGAGCGCCTCGACGACAGAGC	miRNA	hsa-mir-339
t0006955	21	16	TACCACAGGGTAGAACCACGG	miRNA	hsa-mir-140
t0006993	20	16	CTTATCAGATTGTATTGTAA	miRNA	hsa-mir-374a
t0007038	18	15	TACCGCACTGTGGGTACT	miRNA	hsa-mir-106b
t0007050	25	15	AATGACACGATCACTCCCGTTGAGT	miRNA	hsa-mir-425
t0007102	21	15	CTGGCCCTCTCTGCCCTTCCG	miRNA	hsa-mir-328
t0007126	20	15	TAATGGTAATGGTTCTCTTG	miRNA	hsa-mir-451a
t0007177	20	15	TGGCTCAGTTCAGCAGGAAC	miRNA	hsa-mir-24-2
t0007211	23	15	ACTGTGCGTGTGACAGCGGCTGA	miRNA	hsa-mir-210
t0007231	21	15	CACCCGGCTGTGTGCACATGT	miRNA	hsa-mir-941-4
t0007234	21	15	AAGCTCGGTCTGAGGCCCCTC	miRNA	hsa-mir-423
t0007271	20	15	TACTAGACTGAAGCTCCTTG	miRNA	hsa-mir-151a
t0007328	22	15	TGTAGAGCAGGGAGCAGGAAGC	miRNA	hsa-mir-4732
t0007356	22	15	ATGGCCAGAGCTCACACAGAGG	miRNA	hsa-mir-4435-[illegible]
t0007383	21	15	CAGCAGCAATTCATGTTTTGA	miRNA	hsa-mir-424
t0007429	22	14	ACTCGGCGTGGCGTCGGTCGTG	miRNA	hsa-mir-1307
t0007454	20	14	TGAGAACCACGTCTGCTCTG	miRNA	hsa-mir-589
t0007530	21	14	AAAGTGCTGTTCGTGCAGGTA	miRNA	hsa-mir-93
t0007647	22	14	CAGTGCAATGATGAAAGGGCAT	miRNA	hsa-mir-130b
t0007656	19	14	AGCTCGGTCTGAGGCCCCT	miRNA	hsa-mir-423
t0007709	20	14	TACCCATTGCATATCGGAGT	miRNA	hsa-mir-660
t0007733	19	14	TGAGGTAGTAGGTTGTGTG	miRNA	hsa-let-7b
t0007783	23	14	AAAAGTGCTTACAGTGCAGGTAG	miRNA	hsa-mir-106a
t0007809	23	14	TTAATGCTAATCGTGATAGGGGT	miRNA	hsa-mir-155
t0007823	22	14	TGGAGAGAAAGGCAGTTCCTGA	miRNA	hsa-mir-185
t0007849	21	14	TACAGTATAGATGATGTACTA	miRNA	hsa-mir-144
t0007860	19	14	CCGTTACCATTACTGAGTT	miRNA	hsa-mir-451a
t0007900	18	14	TAAAGTGCTGACAGTGCA	miRNA	hsa-mir-106b
t0007917	18	14	TGAGGTAGTAGGTTGTGT	miRNA	hsa-let-7b
t0007922	20	13	CTTTCAGTCAGATGTTTGCT	miRNA	hsa-mir-30d
t0008018	18	13	TCATCATATACTGTAAGT	miRNA	hsa-mir-144
t0008049	21	13	ATGACACGATCACTCCCGTTG	miRNA	hsa-mir-425
t0008070	21	13	ACAGTAGTCTGCACATTGGTT	miRNA	hsa-mir-199b
t0008074	23	13	TAATCCTTGCTACCTGGGTGAGA	miRNA	hsa-mir-500b
t0008105	22	13	CAACACCAGTCGATGGGCTGTC	miRNA	hsa-mir-21
t0008123	20	13	GAGGTAGTAGTTTGTACAGT	miRNA	hsa-let-7g
t0008226	19	13	CAAAGAATTCTCCTTTTGG	miRNA	hsa-mir-186
t0008304	20	13	TGAGCGCCTCGACGACAGAG	miRNA	hsa-mir-339
t0008307	21	13	CGCATCCCCTAGGGCATTGGT	miRNA	hsa-mir-324
t0008324	19	13	GGCAATGGTAGAACTCACA	miRNA	hsa-mir-182
t0008369	20	13	TACGTCATCGTTGTCATCGT	miRNA	hsa-mir-598
t0008371	27	13	CTGGATTCAAGTAATCCAGGATAGGCT	miRNA	hsa-mir-26a-2
t0008424	22	13	CTGAGGGGCAGAGAGCGAGACT	miRNA	hsa-mir-423
t0008477	21	13	ATATCATCATATACTGTAAGT	miRNA	hsa-mir-144
t0008490	20	13	GACTATAGAACTTTCCCCCT	miRNA	hsa-mir-625
t0008499	21	13	TACCCATTGCATATCGGAGTT	miRNA	hsa-mir-660
t0008530	23	13	TGTAACAGCAACTCCATGTGGAA	miRNA	hsa-mir-194-2

t0008544	20	13	CTATACAATCTACTGTCTTT	miRNA	hsa-let-7a-3
t0008578	23	13	TACAGTACTGTGATAACTGAAGG	miRNA	hsa-mir-101-1
t0008647	19	12	CGTACCGTGAGTAATAATG	miRNA	hsa-mir-126
t0008649	19	12	AGGGGCAGAGAGCGAGACT	miRNA	hsa-mir-423
t0008677	21	12	AATGGTAATGGTTCTCTTGCT	miRNA	hsa-mir-451a
t0008719	18	12	TTCAAGTAATCCAGGATA	miRNA	hsa-mir-26a-2
t0008827	24	12	CAGTGCAATAGTATTGTCAAAGCA	miRNA	hsa-mir-301a
t0008829	19	12	AGGTAGTAGATTGTATAGT	miRNA	hsa-let-7f-2
t0008875	23	12	GTAGTGTTTCCTACTTTATGGAT	miRNA	hsa-mir-142
t0008879	19	12	TGAGGTAGTAAGTTGTATT	miRNA	hsa-mir-98
t0008921	20	12	GCAATGGTAGAACTCACACT	miRNA	hsa-mir-182
t0008930	21	12	GTAAACATCCTTGACTGGAAG	miRNA	hsa-mir-30e
t0008970	20	12	AGTGCAATGATGAAAGGGCA	miRNA	hsa-mir-130b
t0008998	23	12	GTACAGTACTGTGATAACTGAAG	miRNA	hsa-mir-101-2
t0009059	22	12	TGAGGTAGGAGGTTGTATAGTT	miRNA	hsa-let-7e
t0009065	18	12	GTAGTAGATTGTATAGTT	miRNA	hsa-let-7f-2
t0009102	21	12	TTGGGACTGATCTTGATGTCT	miRNA	hsa-mir-3913-2
t0009195	19	11	CTCGAGGAGCTCACAGTCT	miRNA	hsa-mir-151b
t0009208	22	11	CTGCCCTGGCCCGAGGGACCGA	miRNA	hsa-mir-874
t0009255	22	11	TACGTCATCGTTGTCATCGTCA	miRNA	hsa-mir-598
t0009403	21	11	TGGCGGCGGTAGTTATGGGCT	miRNA	hsa-mir-4467
t0009404	23	11	ACCCTATCAATATTGTCTCTGCT	miRNA	hsa-mir-454
t0009468	21	11	TAGACTGAAGCTCCTTGAGGA	miRNA	hsa-mir-151a
t0009566	20	11	CCGTTACCATTACTGAGTTT	miRNA	hsa-mir-451a
t0009567	21	11	TTCACCACCTTCTCCACCCAG	miRNA	hsa-mir-197
t0009575	22	11	TTGGCAATGGTAGAACTCACAC	miRNA	hsa-mir-182
t0009577	19	11	ACCGTTACCATTACTGAGT	miRNA	hsa-mir-451a
t0009583	19	11	CTGACCTATGAATTGACAG	miRNA	hsa-mir-192
t0009591	20	11	CTGGACTTGGAGTCAGAAGG	miRNA	hsa-mir-378a
t0009600	21	11	TTTCAGTCGGATGTTTACAGC	miRNA	hsa-mir-30e
t0009606	21	11	TCAAGAGCAATAACGAAAAAT	miRNA	hsa-mir-335
t0009607	22	11	TTTCCGGCTCGCGTGGGTGTGT	miRNA	hsa-mir-1180
t0009622	20	11	CAGTACTGTGATAACTGAAG	miRNA	hsa-mir-101-2
t0009636	24	11	TCAAAGTGCTTACAGTGCAGGTAG	miRNA	hsa-mir-17
t0009649	25	11	TTCAAGTAATCCAGGATAGGCTGTT	miRNA	hsa-mir-26a-2
t0009701	21	11	TTCTAGTAAGAGTGGCAGTCG	miRNA	hsa-mir-628
t0009707	18	11	AATATTACTGTGCTGCTT	miRNA	hsa-mir-16-2
t0009833	23	11	TTGTGCAAATCTATGCAAAACTG	miRNA	hsa-mir-19a
t0009914	18	11	GTACTGAGCTGCCCCGAG	miRNA	hsa-mir-486
t0009925	21	11	GAACATTCAACGCTGTCGGTG	miRNA	hsa-mir-181a-1
t0009933	21	11	TGATATGTTTGATATATTAGG	miRNA	hsa-mir-190a
t0009935	22	11	CTAGCACCATTTGAAATCAGTG	miRNA	hsa-mir-29b-2
t0010027	19	11	CAAAGTGCTGTTCGTGCAG	miRNA	hsa-mir-93
t0010066	20	11	TAGCACCATCTGAAATCGGT	miRNA	hsa-mir-29a
t0010100	19	10	TGTAAACATCCCCGACTGG	miRNA	hsa-mir-30d
t0010114	22	10	ATGCACCTGGGCAAGGATTCTG	miRNA	hsa-mir-500a
t0010123	23	10	CCGGTCCCAGGAGAACCTGCAGA	miRNA	hsa-mir-4746
t0010143	24	10	CAAAGTGCTCATAGTGCAGGTAGT	miRNA	hsa-mir-20b
t0010173	21	10	TTCTCTGTTTTGGCCATGTGT	miRNA	hsa-mir-942
t0010175	23	10	AATCCTTGGAACCTAGGTGTGAG	miRNA	hsa-mir-362
t0010249	22	10	TCCCCCAGGTGTGATTCTGATT	miRNA	hsa-mir-361
t0010281	21	10	TCTTCTCTGTTTTGGCCATGT	miRNA	hsa-mir-942
t0010305	22	10	AACCCGTAGATCCGAACTTGTG	miRNA	hsa-mir-100
t0010409	20	10	AGCAGCACGTAAATATTGGC	miRNA	hsa-mir-16-2
t0010432	22	10	GAAGTTCTGTTATACACTCAGG	miRNA	hsa-mir-148b
t0010440	21	10	ACCCTGTAGATCCGAATTTGT	miRNA	hsa-mir-10a
t0010463	20	10	TCTACTGTAGTATGGGCACT	miRNA	hsa-mir-20b
t0010542	22	10	TTGAGAATGATGAATCATTAGG	miRNA	hsa-mir-580

t0010567	21	10	TGTCAGTTTGTCAAATACCCC	miRNA	hsa-mir-223
t0010610	22	10	ACTCTTTCCCTGTTGCACTACT	miRNA	hsa-mir-130b
t0010615	23	10	AGAGTTGAGTCTGGACGTCCCGA	miRNA	hsa-mir-219-1
t0010630	23	10	ACAGGGTAGAACCACGGACAGGA	miRNA	hsa-mir-140
t0010632	22	10	CATCCCTTGCATGGTGGAGGGT	miRNA	hsa-mir-188
t0010651	21	10	TATGGAAAGACTTTGCCACTC	miRNA	hsa-mir-3688-2
t0010656	19	10	AGTTCTTCAGTGGCAAGCT	miRNA	hsa-mir-22
t0010729	22	10	CTGAGGTAGTAGTTTGTGCTGT	miRNA	hsa-let-7i
t0010755	22	10	TGTGACAATAGAGATGAACATG	miRNA	hsa-mir-4504
t0010850	20	10	ACAGTACTGTGATAACTGAA	miRNA	hsa-mir-101-2
t0010854	22	10	AATCTGAGAAGGCGCACAAGGT	miRNA	hsa-mir-3200
t0010879	19	10	TCAGTGCATCACAGAACTT	miRNA	hsa-mir-148b
t0010889	20	10	TGTAAACATCCTCGACTGGA	miRNA	hsa-mir-30a
t0010894	20	10	AAGCAGCATTGTACAGGGCT	miRNA	hsa-mir-107
t0010903	21	10	ATGGTAATGGTTCTCTTGCTA	miRNA	hsa-mir-451a
t0010986	20	10	ACCGCACTGTGGGTACTTGC	miRNA	hsa-mir-106b
t0011013	23	10	CACTAGATTGTGAGCTCCTGGAG	miRNA	hsa-mir-28
t0011020	20	9	TGTGCAAATCTATGCAAAAC	miRNA	hsa-mir-19a
t0011038	18	9	CAGTACTGTGATAACTGA	miRNA	hsa-mir-101-2
t0011068	20	9	AAAGTGCTTACAGTGCAGGT	miRNA	hsa-mir-106a
t0011180	20	9	TGTCTTACTCCCTCAGGCAC	miRNA	hsa-mir-550a-3
t0011222	20	9	AGGTAGTAGTTTGTGCTGTT	miRNA	hsa-let-7i
t0011228	19	9	TATACGACCTGCTGCCTTT	miRNA	hsa-let-7d
t0011229	18	9	TGCCAGTTGAAGAACTGT	miRNA	hsa-mir-22
t0011293	18	9	ATCATCATATACTGTAAG	miRNA	hsa-mir-144
t0011350	23	9	TTCAAGTAATTCAGGATAGGTTG	miRNA	hsa-mir-26b
t0011353	20	9	TGCGGGGCTAGGGCTAACAG	miRNA	hsa-mir-744
t0011365	22	9	TAGCACCATTTGAAATCAGTGT	miRNA	hsa-mir-29b-2
t0011419	22	9	AGGATGAGCAAAGAAAGTAGAT	miRNA	hsa-mir-1255a
t0011512	23	9	AGCTTCTTTACAGTGCTGCCTTG	miRNA	hsa-mir-103a-2
t0011538	19	9	CAACACCAGTCGATGGGCT	miRNA	hsa-mir-21
t0011547	22	9	TACAGTAGTCTGCACATTGGTT	miRNA	hsa-mir-199b
t0011571	23	9	TCAGGCTCAGTCCCCTCCCGATA	miRNA	hsa-mir-484
t0011576	23	9	CATTATTACTTTTGGTACGCGCT	miRNA	hsa-mir-126
t0011578	18	9	CTAGACTGAAGCTCCTTG	miRNA	hsa-mir-151a
t0011647	24	9	TCCTGTACTGAGCTGCCCCGAGGC	miRNA	hsa-mir-486
t0011708	18	9	GTAGTAGGTTGTATAGTT	miRNA	hsa-let-7a-3
t0011744	22	9	ATTATTACTTTTGGTACGCGCT	miRNA	hsa-mir-126
t0011762	23	9	CATCCTGTACTGAGCTGCCCCGA	miRNA	hsa-mir-486
t0011777	18	9	TACTAGACTGAAGCTCCT	miRNA	hsa-mir-151a
t0011858	24	9	CATTGCACTTGTCTCGGTCTGACA	miRNA	hsa-mir-25
t0011889	21	9	AGGGGCAGAGAGCGAGACTTT	miRNA	hsa-mir-423
t0011990	22	9	ACCACTGACCGTTGACTGTACC	miRNA	hsa-mir-181a-2
t0012047	19	9	TTTAGTAATGGTAATGGTT	miRNA	hsa-mir-451a
t0012059	20	9	CCACCTCCCCTGCAAACGTC	miRNA	hsa-mir-1306
t0012086	20	9	ATTGCACTTGTCTCGGTCTG	miRNA	hsa-mir-25
t0012089	23	9	CTGTGCGTGTGACAGCGGCTGAT	miRNA	hsa-mir-210
t0012133	21	9	ACTGCCCTAAGTGCTCCTTCT	miRNA	hsa-mir-18a
t0012147	20	8	AGGGCTTCCTCTCTGCAGGA	miRNA	hsa-mir-3158-2
t0012186	22	8	AGTGCAATATTGCTTATAGGGT	miRNA	hsa-mir-454
t0012250	23	8	TGAGAACTGAATTCCATGGGTTG	miRNA	hsa-mir-146a
t0012283	19	8	ACCACTGACCGTTGACTGT	miRNA	hsa-mir-181a-2
t0012290	22	8	TTTCTTCTTAGACATGGCAGCT	miRNA	hsa-mir-4659b
t0012366	20	8	CTATACAATCTATTGCCTTC	miRNA	hsa-let-7f-1
t0012393	22	8	AGGACCTTCCCTGAACCAAGGA	miRNA	hsa-mir-659
t0012425	21	8	AGTTCTTCAGTGGCAAGCTTT	miRNA	hsa-mir-22
t0012444	20	8	ATGGCACTGGTAGAATTCAC	miRNA	hsa-mir-183
t0012504	19	8	AGCATTGTACAGGGCTATG	miRNA	hsa-mir-103a-1

t0012523	21	8	TACCCTGTAGAACCGAATTTG	miRNA	hsa-mir-10b
t0012763	20	8	TGGAGAGAAAGGCAGTTCCT	miRNA	hsa-mir-185
t0012788	20	8	TTGGCAATGGTAGAACTCAC	miRNA	hsa-mir-182
t0012796	21	8	AATGCACCCGGGCAAGGATTC	miRNA	hsa-mir-501
t0012904	22	8	TTTGGGACTGATCTTGATGTCT	miRNA	hsa-mir-3913-2
t0012980	22	8	TATGGAAAGACTTTGCCACTCT	miRNA	hsa-mir-3688-2
t0012984	19	8	CAAAAGCAATCGCGGTTTT	miRNA	hsa-mir-548e
t0013020	20	8	GTGAGGTAGTAGGTTGTATA	miRNA	hsa-let-7a-3
t0013046	18	8	TTCACAGTGGCTAAGTTC	miRNA	hsa-mir-27b
t0013047	21	8	CCAAAACTGCAGTTACTTTTG	miRNA	hsa-mir-548o-2
t0013117	19	8	CAAAGTGCTTACAGTGCAG	miRNA	hsa-mir-17
t0013128	21	8	TTCACAGTGGCTAAGTTCTGC	miRNA	hsa-mir-27b
t0013147	23	8	AACCGTTACCATTACTGAGTTTA	miRNA	hsa-mir-451a
t0013170	20	8	TTATAATACAACCTGATAAG	miRNA	hsa-mir-374a
t0013233	23	8	AATCTGAGAAGGCGCACAAGGTT	miRNA	hsa-mir-3200
t0013312	19	8	CACCAATATTACTGTGCTG	miRNA	hsa-mir-16-2
t0013314	19	8	AGGTAGTAGGTTGTATAGT	miRNA	hsa-let-7a-3
t0013361	18	8	CCGCACTGTGGGTACTTG	miRNA	hsa-mir-106b
t0013466	21	8	TAACACTGTCTGGTAAAGATG	miRNA	hsa-mir-141
t0013569	20	8	AAAGAATTCTCCTTTTGGGC	miRNA	hsa-mir-186
t0013637	21	8	GTAGTGTTTCCTACTTTATGG	miRNA	hsa-mir-142
t0013648	21	8	TATTGCACATTACTAAGTTGC	miRNA	hsa-mir-32
t0013703	24	8	ATGACACGATCACTCCCGTTGAGT	miRNA	hsa-mir-425
t0013720	21	7	CAGAGTCTCGTTCTGTTGCCC	miRNA	hsa-mir-1273c
t0013780	20	7	ATCCTGTACTGAGCTGCCCC	miRNA	hsa-mir-486
t0013787	22	7	AGGGGGATGGCAGAGCAAAATT	miRNA	hsa-mir-5010
t0013842	23	7	AGCTACATTGTCTGCTGGGTTTC	miRNA	hsa-mir-221
t0013930	18	7	TGTAACAGCAACTCCATG	miRNA	hsa-mir-194-2
t0013940	20	7	AGGTAGTAGATTGTATAGTT	miRNA	hsa-let-7f-2
t0013973	25	7	CGTGTATTTGACAAGCTGAGTTGGA	miRNA	hsa-mir-223
t0013984	22	7	CGAATCATTATTTGCTGCTCTA	miRNA	hsa-mir-15b
t0014054	19	7	ATCACATTGCCAGGGATTT	miRNA	hsa-mir-23a
t0014062	20	7	CAAGTAATCCAGGATAGGCT	miRNA	hsa-mir-26a-2
t0014111	20	7	CTGGGAGAGGGTTGTTTACT	miRNA	hsa-mir-30c-1
t0014170	20	7	TTGCACTTGTCTCGGTCTGA	miRNA	hsa-mir-25
t0014230	21	7	GTGAGGTAGTAGGTTGTATAG	miRNA	hsa-let-7a-3
t0014265	21	7	CTGCGCAAGCTACTGCCTTGC	miRNA	hsa-let-7i
t0014285	22	7	TAACGCATAATATGGACATGTT	miRNA	hsa-mir-3912
t0014311	20	7	TTTCCGGCTCGCGTGGGTGT	miRNA	hsa-mir-1180
t0014313	20	7	AAAAGTAATTGCGGTTTTTG	miRNA	hsa-mir-548au
t0014338	21	7	TTATCAGAATCTCCAGGGGTA	miRNA	hsa-mir-361
t0014370	21	7	AAAGATTCTGAGCAATAACCT	miRNA	hsa-mir-4678
t0014384	23	7	GTGAGGTAGTAGGTTGTATAGTT	miRNA	hsa-let-7a-3
t0014458	20	7	ACTAGACTGAAGCTCCTTGA	miRNA	hsa-mir-151a
t0014472	21	7	AGCTACATCTGGCTACTGGGT	miRNA	hsa-mir-222
t0014478	20	7	TTAAAACTTTAAGTGTGCCT	miRNA	hsa-mir-5582
t0014519	23	7	ACTGCCCTAAGTGCTCCTTCTGG	miRNA	hsa-mir-18a
t0014537	20	7	TAGTACCAGTACCTTGTGTT	miRNA	hsa-mir-624
t0014544	21	7	CCTCCGTGTTACCTGTCCTCT	miRNA	hsa-mir-3605
t0014545	18	7	AGAGGTAGTAGGTTGCAT	miRNA	hsa-let-7d
t0014575	23	7	TAGCAGCACATAATGGTTTGTGG	miRNA	hsa-mir-15a
t0014584	22	7	AAAAGCTGGGTTGAGAGGGCAA	miRNA	hsa-mir-320b-2
t0014590	22	7	ATGAGGTAGTAGGTTGTATAGT	miRNA	hsa-let-7a-1
t0014620	23	7	AAGCTCGGTCTGAGGCCCCTCAG	miRNA	hsa-mir-423
t0014676	22	7	CGGATGAGCAAAGAAAGTGGTT	miRNA	hsa-mir-1255b-
t0014710	24	7	AGATAACTATACAACCTACTGCCT	miRNA	hsa-let-7b
t0014725	21	7	TCGACCGGACCTCGACCGGCT	miRNA	hsa-mir-1307
t0014734	20	7	AGGTAGTAGGTTGTATAGTT	miRNA	hsa-let-7a-3

t0014750	21	7	ATTATGGTTTGCCTGGGACTG	miRNA	hsa-mir-584
t0014754	19	7	CAGCACGTAAATATTGGCG	miRNA	hsa-mir-16-2
t0014904	19	7	TGTCTTACTCCCTCAGGCA	miRNA	hsa-mir-550a-3
t0014949	21	7	TGGAGAGAAAGGCAGTTCCTG	miRNA	hsa-mir-185
t0014989	19	7	TGTCAGTTTGTCAAATACC	miRNA	hsa-mir-223
t0015043	20	7	TAGACTGAAGCTCCTTGAGG	miRNA	hsa-mir-151a
t0015112	21	7	GAGGTAGTAGGTTGCATAGTT	miRNA	hsa-let-7d
t0015143	22	7	TTTCAGTCAGATGTTTGCTGCT	miRNA	hsa-mir-30d
t0015205	20	7	CTGCAGTGAAGGCACTTGTA	miRNA	hsa-mir-17
t0015218	19	7	CGCACTGTGGGTACTTGCT	miRNA	hsa-mir-106b
t0015238	21	7	TTTAGGATAAGCTTGACTTTT	miRNA	hsa-mir-651
t0015259	19	7	ACCATCGACCGTTGATTGT	miRNA	hsa-mir-181a-1
t0015266	22	7	TCTGACCTATGAATTGACAGCC	miRNA	hsa-mir-192
t0015291	22	7	ATGGCATCGTCCCCTGGTGGCT	miRNA	hsa-mir-4642
t0015300	18	7	ATGAGGTAGTAGATTGTA	miRNA	hsa-let-7f-2
t0015323	23	7	GATGAGGATGGATAGCAAGGAAG	miRNA	hsa-mir-3605
t0015329	19	7	AGCTACATTGTCTGCTGGG	miRNA	hsa-mir-221
t0015340	22	7	CTGCGCAAGCTACTGCCTTGCT	miRNA	hsa-let-7i
t0015392	21	7	ACCGTTACCATTACTGAGTTT	miRNA	hsa-mir-451a
t0015540	23	7	GTAGCTTATCAGACTGATGTTGA	miRNA	hsa-mir-21
t0015571	21	7	ATAGATCTGGATTGGAACCCA	miRNA	hsa-mir-607
t0015631	22	7	ACCGCACTGTGGGTACTTGCTG	miRNA	hsa-mir-106b
t0015715	21	7	GTCAGTTTGTCAAATACCCCA	miRNA	hsa-mir-223
t0015770	22	6	ACTAGATTGTGAGCTCCTGGAG	miRNA	hsa-mir-28
t0015847	23	6	TCAGTTATCACAGTGCTGATGCT	miRNA	hsa-mir-101-1
t0015851	18	6	GCACTGTGGGTACTTGCT	miRNA	hsa-mir-106b
t0015868	20	6	AACATTCAACCTGTCGGTGA	miRNA	hsa-mir-181c
t0015887	22	6	GCCCTGACCTGTCCTGTTCTGC	miRNA	hsa-mir-4732
t0015889	19	6	AGCAGCACATAATGGTTTG	miRNA	hsa-mir-15a
t0016080	18	6	AAGTAATCCAGGATAGGC	miRNA	hsa-mir-26a-2
t0016082	18	6	AGTACTGTGATAACTGAA	miRNA	hsa-mir-101-2
t0016146	22	6	TGGCTCAGTTCAGCAGGAACAG	miRNA	hsa-mir-24-2
t0016257	18	6	CCAATATTACTGTGCTGC	miRNA	hsa-mir-16-2
t0016270	24	6	AACATTCATTGCTGTCGGTGGGTT	miRNA	hsa-mir-181b-2
t0016281	22	6	TGAGCGCCTCGACGACAGAGCC	miRNA	hsa-mir-339
t0016307	22	6	CCACAGGGTAGAACCACGGACA	miRNA	hsa-mir-140
t0016349	18	6	CACCCGGCTGTGTGCACA	miRNA	hsa-mir-941-4
t0016428	21	6	TAGTGCAATATTGCTTATAGG	miRNA	hsa-mir-454
t0016454	20	6	TCCCTGTCCTCCAGGAGCTC	miRNA	hsa-mir-339
t0016504	21	6	CGGATGAGCAAAGAAAGTGGT	miRNA	hsa-mir-1255b-
t0016631	22	6	ACTGTGCGTGTGACAGCGGCTG	miRNA	hsa-mir-210
t0016674	19	6	TTGCACTTGTCCCGGCCTG	miRNA	hsa-mir-92a-2
t0016709	20	6	AATGCACCCGGGCAAGGATT	miRNA	hsa-mir-501
t0016826	22	6	CATATGGAAAGACTTTGCCACT	miRNA	hsa-mir-3688-2
t0016829	20	6	AACACACCTATTCAAGGATT	miRNA	hsa-mir-362
t0016856	20	6	CTAGGTATGGTCCCAGGGAT	miRNA	hsa-mir-331
t0016878	21	6	CTGTACAGGCCACTGCCTTGC	miRNA	hsa-let-7g
t0016975	21	6	TCGAGGAGCTCACAGTCTAGA	miRNA	hsa-mir-151b
t0017144	19	6	ATCATCATATACTGTAAGT	miRNA	hsa-mir-144
t0017259	22	6	TTATGGTTTGCCTGGGACTGAG	miRNA	hsa-mir-584
t0017369	21	6	ACGGAATCCCAAAAGCAGCTG	miRNA	hsa-mir-191
t0017374	22	6	TTAATTTTTTGTTTCGGTCACT	miRNA	hsa-mir-4775
t0017391	20	6	GAGGTAGTAGTTTGTGCTGT	miRNA	hsa-let-7i
t0017429	20	6	AAAAGTAATTGTGGTTTTTG	miRNA	hsa-mir-548ae-
t0017439	23	6	CATTGCACTTGTCTCGGTCTGAC	miRNA	hsa-mir-25
t0017572	23	6	ACTGGCCTGGGACTACCGGGGGT	miRNA	hsa-mir-3176
t0017585	21	6	TCGTACCGTGAGTAATAATGC	miRNA	hsa-mir-126
t0017637	21	6	TTGGGGAAACGGCCGCTGAGT	miRNA	hsa-mir-2110

t0017705	21	6	TATCATCATATACTGTAAGTT	miRNA	hsa-mir-144
t0017762	19	6	TTATAAAGCAATGAGACTG	miRNA	hsa-mir-340
t0017789	24	6	TTGTGCAAATCTATGCAAAACTGA	miRNA	hsa-mir-19a
t0017812	18	6	TTATCAGACTGATGTTGA	miRNA	hsa-mir-21
t0017821	22	6	TCAAGTAATCCAGGATAGGCTG	miRNA	hsa-mir-26a-2
t0017929	20	6	ACTGCAGTGAAGGCACTTGT	miRNA	hsa-mir-17
t0018000	21	6	GTGCAAATCCATGCAAAACTG	miRNA	hsa-mir-19b-2
t0018020	21	6	GTAGTACCAGTACCTTGTGTT	miRNA	hsa-mir-624
t0018054	20	6	AACATTCATTGCTGTCGGTG	miRNA	hsa-mir-181b-2
t0018100	19	6	GAGGTAGTAGTTTGTGCTG	miRNA	hsa-let-7i
t0018258	27	6	TAGCTTATCAGACTGATGTTGACTGTT	miRNA	hsa-mir-21
t0018276	23	6	AGAGGTAGTAGGTTGCATAGTTT	miRNA	hsa-let-7d
t0018290	23	6	ATTCTAATTTCTCCACGTCTTTG	miRNA	hsa-mir-576
t0018360	18	6	CTATACGACCTGCTGCCT	miRNA	hsa-let-7d
t0018372	21	6	TTCACAGTGGCTAAGTTCCGC	miRNA	hsa-mir-27a
t0018418	19	6	ATCCTGTACTGAGCTGCCC	miRNA	hsa-mir-486
t0018422	20	6	ACAGTAGTCTGCACATTGGT	miRNA	hsa-mir-199b
t0018427	22	6	AAAGCTGGGTTGAGAGGGCGAA	miRNA	hsa-mir-320a
t0018455	21	6	CGGTTTGAGGCTACAGTGAGA	miRNA	hsa-mir-1304
t0018467	22	6	TCGGGGCAGCTCAGTACAGGAT	miRNA	hsa-mir-486
t0018541	18	5	ATTGCACTTGTCTCGGTC	miRNA	hsa-mir-25
t0018603	23	5	TTCACCACCTTCTCCACCCAGCA	miRNA	hsa-mir-197
t0018617	19	5	ACTGCCCCAGGTGCTGCTG	miRNA	hsa-mir-324
t0018686	20	5	AGGTAGTAGTTTGTACAGTT	miRNA	hsa-let-7g
t0018842	23	5	CAAAAGTAATTGCGGTCTTTGGT	miRNA	hsa-mir-548j
t0018852	22	5	TAAGGTGCATCTAGTGCAGATA	miRNA	hsa-mir-18a
t0018964	20	5	TACCACAGGGTAGAACCACG	miRNA	hsa-mir-140
t0019016	19	5	AAAAGTAATTGCGGTTTTT	miRNA	hsa-mir-548au
t0019044	19	5	TCAGGCTCAGTCCCCTCCC	miRNA	hsa-mir-484
t0019057	20	5	ACCACAGGGTAGAACCACGG	miRNA	hsa-mir-140
t0019133	21	5	AAAGTGCTTACAGTGCAGGTA	miRNA	hsa-mir-106a
t0019162	20	5	CTCCGTTTGCCTGTTTCGCT	miRNA	hsa-mir-1468
t0019167	21	5	TTGCAGCTGCCTGGGAGTGAC	miRNA	hsa-mir-1301
t0019174	20	5	ACCGTTACCATTACTGAGTT	miRNA	hsa-mir-451a
t0019180	22	5	GCCCTGTGGACTCAGTTCTGGT	miRNA	hsa-mir-146b
t0019194	21	5	TGTTCCTCTGTCTCCCAGACT	miRNA	hsa-mir-4326
t0019218	21	5	CAGCTACATTGTCTGCTGGGT	miRNA	hsa-mir-221
t0019340	21	5	TGTGCTTGCTCGTCCCGCCCG	miRNA	hsa-mir-636
t0019486	24	5	TTAATGCTAATCGTGATAGGGGTT	miRNA	hsa-mir-155
t0019659	20	5	CCACAGGGTAGAACCACGGA	miRNA	hsa-mir-140
t0019713	24	5	TTCAAGTAATTCAGGATAGGTTGT	miRNA	hsa-mir-26b
t0019789	18	5	TAGCAGCACGTAAATATT	miRNA	hsa-mir-16-2
t0019948	24	5	AATCCTTGGAACCTAGGTGTGAGT	miRNA	hsa-mir-362
t0019955	18	5	TTATGGTTTGCCTGGGAC	miRNA	hsa-mir-584
t0019969	21	5	TCAACAGACATTAATTGGGCG	miRNA	hsa-mir-421
t0020046	22	5	CTGGCCCTCTCTGCCCTTCCGT	miRNA	hsa-mir-328
t0020065	21	5	GCAGCATTGTACAGGGCTATG	miRNA	hsa-mir-103a-1
t0020094	21	5	CCTCCCACACCCAAGGCTTGC	miRNA	hsa-mir-532
t0020113	22	5	AAAGTGCTTACAGTGCAGGTAG	miRNA	hsa-mir-106a
t0020222	23	5	TGTCTCCCAACCCTTGTACCAGT	miRNA	hsa-mir-150
t0020288	20	5	ATCACATTGCCAGGGATTTC	miRNA	hsa-mir-23a
t0020306	23	5	GTAAACATCCTACACTCTCAGCT	miRNA	hsa-mir-30c-1
t0020410	20	5	AATGCACCTGGGCAAGGATT	miRNA	hsa-mir-502
t0020436	19	5	TGTAAACATCCTACACTCA	miRNA	hsa-mir-30b
t0020469	22	5	TATACAAGGGCAAGCTCTCTGT	miRNA	hsa-mir-381
t0020518	23	5	CCCCCAGGTGTGATTCTGATTTG	miRNA	hsa-mir-361
t0020550	19	5	TACGTCATCGTTGTCATCG	miRNA	hsa-mir-598
t0020560	22	5	TGCCCTGTGGACTCAGTTCTGG	miRNA	hsa-mir-146b

t0020609	20	5	CTGAGGTAGTAGTTTGTACA	miRNA	hsa-let-7g
t0020645	21	5	CCAGTGGGGCTGCTGTTATCT	miRNA	hsa-mir-194-2
t0020684	24	5	TATTGCACTTGTCCCGGCCTGTGG	miRNA	hsa-mir-92a-2
t0020733	20	5	AGTTCTTCAGTGGCAAGCTT	miRNA	hsa-mir-22
t0020809	22	5	ACTGGCCTGGGACTACCGGGGG	miRNA	hsa-mir-3176
t0020919	21	5	TAATTTTAGATCTGGTCTGCT	miRNA	hsa-mir-4771-2
t0020946	21	5	TCTAGGTATGGTCCCAGGGAT	miRNA	hsa-mir-331
t0020986	23	5	TGCCCTGTGGACTCAGTTCTGGT	miRNA	hsa-mir-146b
t0021015	22	5	GAGGGGCAGAGAGCGAGACTTT	miRNA	hsa-mir-423
t0021026	19	5	GCCCTGACCTGTCCTGTTC	miRNA	hsa-mir-4732
t0021078	20	5	CTAGATTGTGAGCTCCTGGA	miRNA	hsa-mir-28
t0021092	23	5	CTGACCTATGAATTGACAGCCAG	miRNA	hsa-mir-192
t0021212	19	5	TGCGGGGCTAGGGCTAACA	miRNA	hsa-mir-744
t0021227	19	5	GTTACCATTACTGAGTTTA	miRNA	hsa-mir-451a
t0021287	23	5	AGATAACTATACAACCTACTGCC	miRNA	hsa-let-7b
t0021295	22	5	TCGGATCCGTCTGAGCTTGGCT	miRNA	hsa-mir-127
t0021297	22	5	AGCTACATCTGGCTACTGGGTC	miRNA	hsa-mir-222
t0021302	24	5	TAGGTAGTTTCCTGTTGTTGGGAT	miRNA	hsa-mir-196b
t0021327	19	5	CAACGGAATCCCAAAAGCA	miRNA	hsa-mir-191
t0021334	21	5	AATCCTTGCTATCTGGGTGCT	miRNA	hsa-mir-502
t0021370	22	5	TTCTCTGTTTTGGCCATGTGTG	miRNA	hsa-mir-942
t0021428	20	5	TTCTCTGTTTTGGCCATGTG	miRNA	hsa-mir-942
t0021487	21	5	AGCTTATCAGACTGATGTTGA	miRNA	hsa-mir-21
t0021548	22	5	ATTGCACGGTATCCATCTGTAA	miRNA	hsa-mir-363
t0021645	20	5	ATGAGGTAGTAGATTGTATA	miRNA	hsa-let-7f-2
t0021684	21	5	GTTCACAGTGGCTAAGTTCTG	miRNA	hsa-mir-27b
t0021749	21	5	TAGCACCATTTGAAATCAGTG	miRNA	hsa-mir-29b-2
t0021850	19	5	AACATTCATTGCTGTCGGT	miRNA	hsa-mir-181b-2
t0021893	20	5	ACTGCCCCAGGTGCTGCTGG	miRNA	hsa-mir-324
t0021905	21	5	TCTCCCTTCCTGCCCTGGCTA	miRNA	hsa-mir-4685
t0021977	21	5	GTGAGGACTCGGGAGGTGGAG	miRNA	hsa-mir-1224
t0022049	25	5	AACCGTTACCATTACTGAGTTTAGT	miRNA	hsa-mir-451a
t0022102	18	5	TGGCAATGGTAGAACTCA	miRNA	hsa-mir-182
t0022255	19	5	TTTAGGATAAGCTTGACTT	miRNA	hsa-mir-651
t0022286	23	5	AGGATGAGCAAAGAAAGTAGATT	miRNA	hsa-mir-1255a
t0022437	22	5	ATATCATCATATACTGTAAGTT	miRNA	hsa-mir-144
t0022474	18	5	CAAAGTGCTGTTCGTGCA	miRNA	hsa-mir-93
t0022587	21	4	TCTCACACAGAAATCGCACCC	miRNA	hsa-mir-342
t0022642	18	4	TGCTGTTCGTGCAGGTAG	miRNA	hsa-mir-93
t0022853	18	4	CACTAGATTGTGAGCTCC	miRNA	hsa-mir-28
t0022956	22	4	AAGGAGCTCACAGTCTATTGAG	miRNA	hsa-mir-28
t0023151	21	4	ATTCTAATTTCTCCACGTCTT	miRNA	hsa-mir-576
t0023200	20	4	TGAGTGTGTGTGTGTGAGTG	miRNA	hsa-mir-574
t0023287	19	4	TACCCATTGCATATCGGAG	miRNA	hsa-mir-660
t0023381	22	4	TGAGCTAAATGTGTGCTGGGAC	miRNA	hsa-mir-610
t0023385	21	4	GTAAACATCCTACACTCAGCT	miRNA	hsa-mir-30b
t0023455	19	4	AGTGCAATGATGAAAGGGC	miRNA	hsa-mir-130b
t0023459	22	4	ATGCTGACATATTTACTAGAGG	miRNA	hsa-mir-628
t0023472	22	4	TGATATGTTTGATATTGGGTTG	miRNA	hsa-mir-190b
t0023576	21	4	TGAGAACTGAATTCCATAGGC	miRNA	hsa-mir-146b
t0023613	22	4	GCTCGGTCTGAGGCCCCTCAGT	miRNA	hsa-mir-423
t0023696	21	4	TCCCTGAGACCCTTTAACCTG	miRNA	hsa-mir-125a
t0023729	21	4	TCAGTGCATCACAGAACTTTG	miRNA	hsa-mir-148b
t0023756	22	4	TTGTGCAAATCTATGCAAAACT	miRNA	hsa-mir-19a
t0023787	19	4	ATTGCACTCGTCCCGGCCT	miRNA	hsa-mir-92b
t0023861	21	4	ATGGCCAGAGCTCACACAGAG	miRNA	hsa-mir-4435-2
t0023873	19	4	ACATTCATTGCTGTCGGTG	miRNA	hsa-mir-181b-2
t0023980	21	4	TAATTTTATGTATAAGCTAGT	miRNA	hsa-mir-590

t0024058	20	4	CCTCCCACACCCAAGGCTTG	miRNA	hsa-mir-532
t0024113	21	4	CGAAAACAGCAATTACCTTTG	miRNA	hsa-mir-570
t0024213	21	4	GCCCCTGGGCCTATCCTAGAA	miRNA	hsa-mir-331
t0024359	20	4	AAAGTTCTGAGACACTCCGA	miRNA	hsa-mir-148a
t0024384	23	4	CACCAATATTACTGTGCTGCTTT	miRNA	hsa-mir-16-2
t0024473	20	4	ATGCACCTGGGCAAGGATTC	miRNA	hsa-mir-502
t0024649	21	4	CCACCTCCCCTGCAAACGTCC	miRNA	hsa-mir-1306
t0024671	21	4	CTCCTGACTCCAGGTCCTGTG	miRNA	hsa-mir-378a
t0024776	21	4	AGGGGGATGGCAGAGCAAAAT	miRNA	hsa-mir-5010
t0024981	20	4	TTAGTAATGGTAATGGTTCT	miRNA	hsa-mir-451a
t0025071	21	4	AGTGCAATGATGAAAGGGCAT	miRNA	hsa-mir-130b
t0025095	20	4	GTTACCATTACTGAGTTTAG	miRNA	hsa-mir-451a
t0025138	21	4	CTGAGGTAGTAGTTTGTACAG	miRNA	hsa-let-7g
t0025294	20	4	GTGAGGACTCGGGAGGTGGA	miRNA	hsa-mir-1224
t0025340	22	4	TCCCTGAGACCCTTTAACCTGT	miRNA	hsa-mir-125a
t0025419	22	4	CCGTTACCATTACTGAGTTTAG	miRNA	hsa-mir-451a
t0025435	22	4	AAAAGTTATTGCGGTTTTGGCT	miRNA	hsa-mir-548at
t0025475	19	4	TCAGTTATCACAGTGCTGA	miRNA	hsa-mir-101-1
t0025839	22	4	CGTGTATTTGACAAGCTGAGTT	miRNA	hsa-mir-223
t0025987	19	4	TGAGGTAGTAGGTTGTATG	miRNA	hsa-let-7c
t0026155	20	4	AGTGTTTCCTACTTTATGGA	miRNA	hsa-mir-142
t0026187	23	4	CCGTTACCATTACTGAGTTTAGT	miRNA	hsa-mir-451a
t0026257	20	4	TTTAGTAATGGTAATGGTTC	miRNA	hsa-mir-451a
t0026357	21	4	TCAGTTATCACAGTGCTGATG	miRNA	hsa-mir-101-1
t0026526	21	4	ATAACATTGTAAAGCGCTTCT	miRNA	hsa-mir-3143
t0026595	24	4	TGTAGAGCAGGGAGCAGGAAGCTG	miRNA	hsa-mir-4732
t0026696	21	4	CCATCGGGAATGTCGTGTCCG	miRNA	hsa-mir-425
t0026744	19	4	CCAAAACTGCAGTTACTTT	miRNA	hsa-mir-548o-2
t0026746	18	4	CAAATCTATGCAAAACTG	miRNA	hsa-mir-19a
t0026856	21	4	TCACACAGAAATCGCACCCGT	miRNA	hsa-mir-342
t0026967	19	4	AATGCACCTGGGCAAGGAT	miRNA	hsa-mir-502
t0027024	25	4	AAAAGCTGGGTTGAGAGGGCGAAAA	miRNA	hsa-mir-320a
t0027098	19	4	AACCGTTACCATTACTGAG	miRNA	hsa-mir-451a
t0027129	24	4	ATGCACCTGGGCAAGGATTCTGAG	miRNA	hsa-mir-500a
t0027276	23	4	TTTTGTGTCTCCCATTCCCCAGA	miRNA	hsa-mir-5010
t0027343	21	4	ATTGTCAGGTAGTGATAGGAT	miRNA	hsa-mir-4999
t0027420	21	4	AATCTGAGAAGGCGCACAAGG	miRNA	hsa-mir-3200
t0027589	22	4	TTGCACTTGTCTCGGTCTGACA	miRNA	hsa-mir-25
t0027714	21	4	TGTAGAGCAGGGAGCAGGAAG	miRNA	hsa-mir-4732
t0027894	22	4	CACTGGACTTGGAGTCAGAAGG	miRNA	hsa-mir-378a
t0027919	21	4	TCAAGTAATTCAGGATAGGTT	miRNA	hsa-mir-26b
t0027970	19	4	GCACTTGTCCCGGCCTGTT	miRNA	hsa-mir-92a-1
t0028217	24	4	GAACATTCAACGCTGTCGGTGAGT	miRNA	hsa-mir-181a-1
t0028296	22	4	TTCTGGATAACATGCTGAAGCT	miRNA	hsa-mir-5706
t0028312	19	4	TGAGATGAAGCACTGTAGC	miRNA	hsa-mir-143
t0028376	21	4	TCTCTCGGCTCCTCGCGGCTC	miRNA	hsa-mir-3615
t0028496	19	4	AATGACACGATCACTCCCG	miRNA	hsa-mir-425
t0028586	19	4	ATAGATCTGGATTGGAACC	miRNA	hsa-mir-607
t0028625	24	4	TAGTAGGTTGTATAGTTTGGGGCT	miRNA	hsa-let-7a-3
t0028658	23	4	TCCCTGTCCTCCAGGAGCTCACG	miRNA	hsa-mir-339
t0028705	22	4	GCAGTGCAATGATGAAAGGGCA	miRNA	hsa-mir-130b
t0028806	22	4	ACAGGGTAGAACCACGGACAGG	miRNA	hsa-mir-140
t0028917	21	4	TAGAGGAGATGGCGCAGGGGA	miRNA	hsa-mir-877
t0029039	21	4	ATTGCACTCGTCCCGGCCTCC	miRNA	hsa-mir-92b
t0029042	22	4	CTGCCCTAGTCTAGCTGAAGCT	miRNA	hsa-mir-3157
t0029090	22	4	CTCCGTTTGCCTGTTTCGCTGA	miRNA	hsa-mir-1468
t0029235	20	4	TTCTGCCTCTGTCCAGGTCC	miRNA	hsa-mir-5001
t0029261	22	4	CAGGGGCTGGCTTTCCTCTGGT	miRNA	hsa-mir-185

t0029410	22	4	ATGACACGATCACTCCCGTTGA	miRNA	hsa-mir-425
t0029466	18	4	TATCATCATATACTGTAA	miRNA	hsa-mir-144
t0029624	20	4	AGGGGCTGGCTTTCCTCTGG	miRNA	hsa-mir-185
t0029649	20	4	TGAGAACTGAATTCCATAGG	miRNA	hsa-mir-146b
t0029654	19	4	AATTGCACGGTATCCATCT	miRNA	hsa-mir-363
t0029745	19	4	GCATTGTACAGGGCTATGA	miRNA	hsa-mir-103a-1
t0029766	20	4	ACATTCAACGCTGTCGGTGA	miRNA	hsa-mir-181a-1
t0029769	24	4	TGAGGTAGTAGATTGTATAGTTGT	miRNA	hsa-let-7f-1
t0029786	21	4	ATGCACCTGGGCAAGGATTCT	miRNA	hsa-mir-500a
t0029913	21	4	AAAGCTCGGTCTGAGGCCCCT	miRNA	hsa-mir-423
t0029964	21	4	GTGCAAATCTATGCAAAACTG	miRNA	hsa-mir-19a
t0029968	20	4	AAAGATTCTGAGCAATAACC	miRNA	hsa-mir-4678
t0030047	22	4	TCGCGGTTTGTGCCAGATGACG	miRNA	hsa-mir-579
t0030073	19	4	TCGAGGAGCTCACAGTCTA	miRNA	hsa-mir-151b
t0030159	18	4	GCATTGTACAGGGCTATG	miRNA	hsa-mir-103a-1
t0030184	21	4	AGTGCCTGAGGGAGTAAGAGC	miRNA	hsa-mir-550a-2
t0030233	22	4	ACATTATTACTTTTGGTACGCG	miRNA	hsa-mir-126
t0030294	20	4	TGCTGGATCAGTGGTTCGAG	miRNA	hsa-mir-1287
t0030353	19	4	AACGGAATCCCAAAAGCAG	miRNA	hsa-mir-191
t0030442	19	4	TATCATCATATACTGTAAG	miRNA	hsa-mir-144
t0030460	21	4	AACATTCATTGCTGTCGGTGG	miRNA	hsa-mir-181b-2
t0030570	24	4	TCAGTGCACTACAGAACTTTGTCT	miRNA	hsa-mir-148a
t0030614	19	4	CTATACAGTCTACTGTCTT	miRNA	hsa-let-7f-2
t0030647	23	3	TTTTGCAATATGTTCCTGAATAT	miRNA	hsa-mir-450b
t0030712	20	3	TTCACCACCTTCTCCACCCA	miRNA	hsa-mir-197
t0030776	25	3	TAGTGCAATATTGCTTATAGGGTTT	miRNA	hsa-mir-454
t0031027	21	3	TGACCGATTTCTCCTGGTGTT	miRNA	hsa-mir-29c
t0031300	19	3	AGTGTTTCCTACTTTATGG	miRNA	hsa-mir-142
t0031315	21	3	AACTGGTTGAACAACTGAACC	miRNA	hsa-mir-582
t0031544	21	3	GTAGCAGCACATAATGGTTTG	miRNA	hsa-mir-15a
t0031561	23	3	CAGTGCAATGATATTGTCAAAGC	miRNA	hsa-mir-301b
t0031611	23	3	TTGAGGTAGTAGGTTGTATAGTT	miRNA	hsa-let-7a-2
t0031669	22	3	TAACTGGTTGAACAACTGAACC	miRNA	hsa-mir-582
t0031872	22	3	TTTCTTCTTAGACATGGCAACG	miRNA	hsa-mir-4659a
t0031912	20	3	TAAGGTGCATCTAGTGCAGT	miRNA	hsa-mir-18b
t0031991	19	3	CACTAGATTGTGAGCTCCT	miRNA	hsa-mir-28
t0031997	22	3	CTGCATTATGAGCACTTAAAGT	miRNA	hsa-mir-20a
t0032013	24	3	ACTGTGCGTGTGACAGCGGCTGAT	miRNA	hsa-mir-210
t0032042	20	3	TACTGCCCTAAATGCCCCTT	miRNA	hsa-mir-18b
t0032503	21	3	TGTGAGGTTGGCATTGTTGTC	miRNA	hsa-mir-1294
t0032509	20	3	CCTGTTCTCCATTACTTGGC	miRNA	hsa-mir-26b
t0032653	22	3	TAACAGTCTACAGCCATGGTCG	miRNA	hsa-mir-132
t0032865	22	3	TAGTGGATGATGGAGACTCGGT	miRNA	hsa-mir-3691
t0033276	22	3	ATGGTAATGGTTCTCTTGCTAT	miRNA	hsa-mir-451a
t0033287	20	3	CTCGGCGTGGCGTCGGTCGT	miRNA	hsa-mir-1307
t0033373	21	3	TCCCCCAGGTGTGATTCTGAT	miRNA	hsa-mir-361
t0033389	20	3	GAAAGACATAGGATAGAGTC	miRNA	hsa-mir-641
t0033449	19	3	AATATTACTGTGCTGCTTT	miRNA	hsa-mir-16-2
t0033503	20	3	AAGGGCTTCCTCTCTGCAGG	miRNA	hsa-mir-3158-2
t0033538	23	3	TGAGCGCCTCGACGACAGAGCCG	miRNA	hsa-mir-339
t0033632	23	3	TAGCACCATTTGAAATCAGTGTT	miRNA	hsa-mir-29b-2
t0033861	22	3	CATCGGGAATGTCGTGTCCGCC	miRNA	hsa-mir-425
t0034086	22	3	CTGGACTTGGAGTCAGAAGGCC	miRNA	hsa-mir-378a
t0034213	20	3	CGAGGAGCTCACAGTCTAGT	miRNA	hsa-mir-151a
t0034224	21	3	TGAGGATGGATAGCAAGGAAG	miRNA	hsa-mir-3605
t0034410	21	3	TCGAATCCCAGCGGTGCCTCC	miRNA	hsa-mir-3676
t0034568	22	3	CAGTGGTTTTACCCTATGGTAG	miRNA	hsa-mir-140
t0034608	18	3	TCAGAACAAATGCCGGTT	miRNA	hsa-mir-589

t0034611	20	3 AGCCTGGAAGCTGGAGCCTG	miRNA	hsa-mir-1254-2
t0034986	24	3 AGTTTTGCAGGTTTGCATCCAGCT	miRNA	hsa-mir-19b-1
t0035053	21	3 TGTAGTGTTTCCTACTTTATG	miRNA	hsa-mir-142
t0035131	24	3 TATTGCACTTGTCCCGGCCTGTTG	miRNA	hsa-mir-92a-1
t0035426	23	3 AGGCGGAGACTTGGGCAATTGCT	miRNA	hsa-mir-25
t0035912	20	3 CCAAAACTGCAGTTACTTTT	miRNA	hsa-mir-548o-2
t0035964	21	3 AGGACCTTCCCTGAACCAAGG	miRNA	hsa-mir-659
t0036128	19	3 AAAACCGTCTAGTTACAGT	miRNA	hsa-mir-1537
t0036155	19	3 GTGCATTGTAGTTGCATTG	miRNA	hsa-mir-33a
t0036159	22	3 TGAGATGAAGCACTGTAGCTCA	miRNA	hsa-mir-143
t0036219	20	3 CACTTGTAATGGAGAACACT	miRNA	hsa-mir-4473
t0036300	21	3 CAAAAGCAATCGCGGTTTTTG	miRNA	hsa-mir-548e
t0036341	22	3 AGCAGCATTGTACAGGGCTATC	miRNA	hsa-mir-107
t0036778	20	3 CACTAGATTGTGAGCTCCTG	miRNA	hsa-mir-28
t0037092	22	3 TAGCAGCACAGAAATATTGGCA	miRNA	hsa-mir-195
t0037154	22	3 ATTCTAATTTCTCCACGTCTTT	miRNA	hsa-mir-576
t0037272	22	3 ACGCATAATATGGACATGTTAT	miRNA	hsa-mir-3912
t0037500	25	3 AGCTTCTTTACAGTGCTGCCTTGTA	miRNA	hsa-mir-103a-2
t0037598	21	3 GTTCCTGCTGAACTGAGCCAG	miRNA	hsa-mir-3074
t0037623	20	3 TCAGTGCATCACAGAACTTT	miRNA	hsa-mir-148b
t0037628	20	3 ACTGGAGATATGGAAGAGCT	miRNA	hsa-mir-1270-2
t0037930	23	3 AATGGCGCCACTAGGGTTGTGCA	miRNA	hsa-mir-652
t0038054	19	3 TGAGCGCCTCGACGACAGA	miRNA	hsa-mir-339
t0038139	18	3 TGCACTTGTCCCGGCCTG	miRNA	hsa-mir-92a-2
t0038260	21	3 CAGCCCGGATCCCAGCCCACT	miRNA	hsa-mir-3940
t0038268	21	3 CTTCCTCGTCTGTCTGCCCCA	miRNA	hsa-mir-1238
t0038326	21	3 TCTGACCTATGAATTGACAGC	miRNA	hsa-mir-192
t0038471	22	3 GCACCATTTGAAATCGGTTATG	miRNA	hsa-mir-29c
t0038604	20	3 ATATCATCATATACTGTAAG	miRNA	hsa-mir-144
t0038651	18	3 ATTATTACTTTTGGTACG	miRNA	hsa-mir-126
t0038776	22	3 AGTGCCTGAGGGAGTAAGAGCC	miRNA	hsa-mir-550a-2
t0038841	31	3 TTTGGCAATGGTAGAACTCACACTGGTGA	miRNA	hsa-mir-182
t0038948	22	3 GAGCGCCTCGACGACAGAGCCG	miRNA	hsa-mir-339
t0039024	18	3 GAGGTAGTAGGTTGTATA	miRNA	hsa-let-7a-3
t0039220	19	3 GGGGCAGCTCAGTACAGGA	miRNA	hsa-mir-486
t0039288	21	3 TCTAGTAAGAGTGGCAGTCGA	miRNA	hsa-mir-628
t0039401	18	3 TCAGTGCATCACAGAACT	miRNA	hsa-mir-148b
t0039438	24	3 TGGGGAGCTGAGGCTCTGGGGGTG	miRNA	hsa-mir-939
t0039473	19	3 TAAAGTGCTTATAGTGCAG	miRNA	hsa-mir-20a
t0039501	19	3 ATTCAACGCTGTCGGTGAG	miRNA	hsa-mir-181a-1
t0039548	21	3 CTGACTGAATAGGTAGGGTCA	miRNA	hsa-mir-3136
t0039706	21	3 TCAGTAAATGTTTATTAGATG	miRNA	hsa-mir-545
t0039763	21	3 CCTGACCTGTCCTGTTCTGCC	miRNA	hsa-mir-4732
t0040038	21	3 GCCCTGTGGACTCAGTTCTGG	miRNA	hsa-mir-146b
t0040079	21	3 AGGCGGAGACTTGGGCAATTG	miRNA	hsa-mir-25
t0040115	21	3 TAGTGTTTCCTACTTTATGGA	miRNA	hsa-mir-142
t0040507	20	3 GCTGGTTTCACATGGTGGCT	miRNA	hsa-mir-29b-2
t0040557	22	3 AATGCACCCGGGCAAGGATTCT	miRNA	hsa-mir-501
t0040577	20	3 CGCATCCCCTAGGGCATTGG	miRNA	hsa-mir-324
t0040619	18	3 CATGCCTTGAGTGTAGGA	miRNA	hsa-mir-532
t0040758	18	3 GTAGTAGTTTGTACAGTT	miRNA	hsa-let-7g
t0040759	19	3 TACCCTGTAGAACCGAATT	miRNA	hsa-mir-10b
t0040891	18	3 GAATCCCAAAAGCAGCTG	miRNA	hsa-mir-191
t0040935	21	3 AGACTGAAGCTCCTTGAGGAC	miRNA	hsa-mir-151a
t0041018	23	3 AACATTCATTGTTGTCGGTGGGT	miRNA	hsa-mir-181d
t0041068	20	3 TCTCACACAGAAATCGCACC	miRNA	hsa-mir-342
t0041227	22	3 TGTGGGACTTCTGGCCTTGACT	miRNA	hsa-mir-3922
t0041952	19	3 TGTATGGCACTGGTAGAAT	miRNA	hsa-mir-183

t0042118	20	3	AGTGCAATGTTAAAAGGGCA	miRNA	hsa-mir-130a
t0042310	19	3	TGCACTTGTCCCGGCCTGT	miRNA	hsa-mir-92a-2
t0042450	23	3	TCAAGAGCAATAACGAAAAATGT	miRNA	hsa-mir-335
t0042513	20	3	GCTACATTGTCTGCTGGGTT	miRNA	hsa-mir-221
t0042634	18	3	ATCACATTGCCAGGGATT	miRNA	hsa-mir-23b
t0042875	22	3	TGTTCCTCTGTCTCCCAGACTC	miRNA	hsa-mir-4326
t0042887	23	3	CCTCAGTAAATGTTTATTAGATG	miRNA	hsa-mir-545
t0043057	22	3	CCTGTACTGAGCTGCCCCGAGG	miRNA	hsa-mir-486
t0043194	20	3	TATAAAATGAGGGCAGTAAG	miRNA	hsa-mir-3163
t0043298	22	3	TTTTACTGTTCCCTCCTAGAGT	miRNA	hsa-mir-4779
t0043393	23	3	AGTGCAATATTGCTTATAGGGTT	miRNA	hsa-mir-454
t0043429	23	3	AGCTACATCTGGCTACTGGGTCT	miRNA	hsa-mir-222
t0043506	21	3	TGGGTTTACGTTGGGAGAACT	miRNA	hsa-mir-629
t0043908	24	3	ATATCATCATATACTGTAAGTTTG	miRNA	hsa-mir-144
t0044000	20	3	TGTACTGAGCTGCCCCGAGG	miRNA	hsa-mir-486
t0044070	22	3	ATAACTATACAATCTACTGTCT	miRNA	hsa-let-7a-3
t0044151	20	3	CAACAAATCACAGTCTGCCA	miRNA	hsa-mir-7-1
t0044344	18	3	GTGAGGTAGTAGGTTGTA	miRNA	hsa-let-7a-3
t0044394	20	3	ATGGCCAGAGCTCACACAGA	miRNA	hsa-mir-4435-2
t0044410	21	3	TTACACAGCTGGACAGAGGCA	miRNA	hsa-mir-4672
t0044457	20	3	TAGATCTGGATTGGAACCCA	miRNA	hsa-mir-607
t0044530	21	3	ACCCATTGCATATCGGAGTTG	miRNA	hsa-mir-660
t0044586	18	3	TCGTACCGTGAGTAATAA	miRNA	hsa-mir-126
t0044611	26	3	TTGGCAATGGTAGAACTCACACTGGT	miRNA	hsa-mir-182
t0044813	22	3	ATTATGGTTTGCCTGGGACTGA	miRNA	hsa-mir-584
t0044872	21	3	TCAGGACACTTCTGAACTTGG	miRNA	hsa-mir-5000
t0045055	21	3	GAGGGGCAGAGAGCGAGACTT	miRNA	hsa-mir-423
t0045236	19	3	AAAGAATTCTCCTTTTGGG	miRNA	hsa-mir-186
t0045407	20	3	TTGGGACTGATCTTGATGTC	miRNA	hsa-mir-3913-2
t0045688	22	3	TTGAGAACTGAATTCCATGGGT	miRNA	hsa-mir-146a
t0045767	22	3	TCCCTGAGACCCTAACTTGTGA	miRNA	hsa-mir-125b-2
t0045946	23	3	TGATATGTTTGATATATTAGGTT	miRNA	hsa-mir-190a
t0045997	22	3	GGCAATGGTAGAACTCACACTG	miRNA	hsa-mir-182
t0045999	21	3	GTTACCATTACTGAGTTTAGT	miRNA	hsa-mir-451a
t0046131	22	3	TAATCCTTGCTACCTGGGTGAG	miRNA	hsa-mir-500b
t0046252	22	3	CACCCGGCTGTGTGCACATGTG	miRNA	hsa-mir-941-4
t0046321	18	3	CTGACCTATGAATTGACA	miRNA	hsa-mir-192
t0046854	19	3	ATTCTAATTTCTCCACGTC	miRNA	hsa-mir-576
t0046885	20	3	CCTGACTGTTGCCGTCCTCC	miRNA	hsa-mir-943
t0046897	22	3	ACTGCAGTGAAGGCACTTGTAG	miRNA	hsa-mir-17
t0046913	20	3	TTATCAGAATCTCCAGGGGT	miRNA	hsa-mir-361
t0047025	20	3	GCAGCATTGTACAGGGCTAT	miRNA	hsa-mir-107
t0047074	20	3	TCTAGTAAGAGTGGCAGTCG	miRNA	hsa-mir-628
t0047232	25	3	GGGGTGCTATCTGTGATTGAGGGAC	miRNA	hsa-mir-342
t0047648	22	3	TATATATATATATGTACGTATG	miRNA	hsa-mir-1277
t0047743	23	3	AAAGTGCTGTTCGTGCAGGTAGT	miRNA	hsa-mir-93
t0047799	21	3	TCAGTAAGTGGCACTCTGTCT	miRNA	hsa-mir-4797
t0047866	25	3	AGGGGTGCTATCTGTGATTGAGGGA	miRNA	hsa-mir-342
t0047905	21	3	AGGATGAGCAAAGAAAGTAGA	miRNA	hsa-mir-1255a
t0047945	22	3	ATAGGACTCATATAGTGCCAGG	miRNA	hsa-mir-3117
t0048020	22	3	CTAGCAGCACGTAAATATTGGC	miRNA	hsa-mir-16-2
t0048132	22	3	GACTATAGAACTTTCCCCCTCA	miRNA	hsa-mir-625
t0048263	18	3	ATGGTAGAACTCACACTG	miRNA	hsa-mir-182
t0048269	21	3	CAGTGCAATAGTATTGTCAAA	miRNA	hsa-mir-301a
t0048393	22	3	CTCCTGACTCCAGGTCCTGTGT	miRNA	hsa-mir-378a
t0048431	23	3	TTTACGTTGGGAGAACTTTTACG	miRNA	hsa-mir-629
t0048542	24	2	TGAGAACTGAATTCCATGGGTTGT	miRNA	hsa-mir-146a
t0048632	21	2	CCATGCCTTGAGTGTAGGACC	miRNA	hsa-mir-532

t0048848	23	2	TACTGCCCTAAATGCCCCTTCTG	miRNA	hsa-mir-18b
t0048967	22	2	ATGCCTTGAGTGTAGGACCGTT	miRNA	hsa-mir-532
t0049126	20	2	GGGGCAGAGAGCGAGACTTT	miRNA	hsa-mir-423
t0049493	21	2	ATCAGGGCTTGTGGAATGGGA	miRNA	hsa-mir-3127
t0050171	20	2	ACTGCAATGTAAGCACTTCT	miRNA	hsa-mir-106a
t0050327	18	2	TGCACTTGTCTCGGTCTG	miRNA	hsa-mir-25
t0050784	18	2	TAAAGTGCTTATAGTGCA	miRNA	hsa-mir-20a
t0051084	21	2	AGGGGTGCTATCTGTGATTGA	miRNA	hsa-mir-342
t0051209	23	2	TTATCAGACTGATGTTGACTGTT	miRNA	hsa-mir-21
t0051283	21	2	TAAGGTGCATCTAGTGCAGTT	miRNA	hsa-mir-18b
t0051461	22	2	AACATTCATTGTTGTCGGTGGG	miRNA	hsa-mir-181d
t0051463	21	2	ACTCAAAACCCTTCAGTGACT	miRNA	hsa-mir-616
t0051482	25	2	TACCGCACTGTGGGTACTTGCTGCT	miRNA	hsa-mir-106b
t0051598	22	2	TATAGATCTGGATTGGAACCCA	miRNA	hsa-mir-607
t0051834	22	2	TAACACTGTCTGGTAAAGATGG	miRNA	hsa-mir-141
t0051869	19	2	ATAGGACTCATATAGTGCC	miRNA	hsa-mir-3117
t0051950	20	2	TGGCGGCGGTAGTTATGGGC	miRNA	hsa-mir-4467
t0051971	23	2	ACTGGGAAGAGGAGCTGAGGGAC	miRNA	hsa-mir-4646
t0052116	22	2	TTTGGTCCCCTTCAACCAGCTG	miRNA	hsa-mir-133a-2
t0052117	21	2	GAATCATTATTTGCTGCTCTA	miRNA	hsa-mir-15b
t0052168	25	2	TAGCAGCACATAATGGTTTGTGGAT	miRNA	hsa-mir-15a
t0052884	20	2	TTTGGGACTGATCTTGATGT	miRNA	hsa-mir-3913-2
t0052926	22	2	TAAAACTTTAAGTGTGCCTAGG	miRNA	hsa-mir-5582
t0052930	18	2	AGGTAGTAGATTGTATAG	miRNA	hsa-let-7f-2
t0053021	21	2	ATAAAGTAGAAAGCACTACTA	miRNA	hsa-mir-142
t0053186	20	2	GCTTATCAGACTGATGTTGA	miRNA	hsa-mir-21
t0053198	22	2	TCAGAACAAATGCCGGTTCCCA	miRNA	hsa-mir-589
t0053295	20	2	TACCGCACTGTGGGTACTTG	miRNA	hsa-mir-106b
t0053415	21	2	GTAGAGGAGATGGCGCAGGGG	miRNA	hsa-mir-877
t0053430	24	2	TTGGGGAAACGGCCGCTGAGTGAG	miRNA	hsa-mir-2110
t0053624	19	2	TGTAGTGTTTCCTACTTTA	miRNA	hsa-mir-142
t0053640	18	2	GCACTGGTAGAATTCACT	miRNA	hsa-mir-183
t0053884	22	2	TAGACTGAAGCTCCTTGAGGAC	miRNA	hsa-mir-151a
t0053908	18	2	TGTTTCCTACTTTATGGA	miRNA	hsa-mir-142
t0053921	21	2	TCTCCAGTATTAACTGTGCTG	miRNA	hsa-mir-16-1
t0054160	22	2	TCAAGAGCAATAACGAAAAATG	miRNA	hsa-mir-335
t0054321	18	2	GTTGTACTTTTTTTTTTG	miRNA	hsa-mir-3613
t0054385	19	2	ACAGTATAGATGATGTACT	miRNA	hsa-mir-144
t0054420	20	2	CTGGTACAGGCCTGGGGGAC	miRNA	hsa-mir-150
t0054442	19	2	CTATACAATCTACTGTCTT	miRNA	hsa-let-7a-3
t0054501	21	2	AGGCCAAAGGAAGAGAACAGA	miRNA	hsa-mir-4753
t0054513	22	2	CATCCTGTACTGAGCTGCCCCG	miRNA	hsa-mir-486
t0054728	22	2	GGACCTTCCCTGAACCAAGGAA	miRNA	hsa-mir-659
t0054837	22	2	ATAGTAGACCGTATAGCGTACG	miRNA	hsa-mir-411
t0054915	23	2	CTGTGCAAATCCATGCAAAACTG	miRNA	hsa-mir-19b-2
t0054959	20	2	TTCCTATGCATATACTTCTT	miRNA	hsa-mir-202
t0055247	20	2	CAGCAGCAATTCATGTTTTG	miRNA	hsa-mir-424
t0055452	23	2	AAAGCTGGGTTGAGAGGGCGAAA	miRNA	hsa-mir-320a
t0055707	21	2	CGAGGAGCTCACAGTCTAGTA	miRNA	hsa-mir-151a
t0055805	21	2	TTTTGTGTCTCCCATTCCCCA	miRNA	hsa-mir-5010
t0056026	22	2	CCAAAGAATTCTCCTTTTGGGC	miRNA	hsa-mir-186
t0056357	20	2	ACCATCGACCGTTGATTGTA	miRNA	hsa-mir-181a-1
t0056958	21	2	TTGTGCAAATCTATGCAAAAC	miRNA	hsa-mir-19a
t0057020	33	2	TTTGGCAATGGTAGAACTCACACTGGTGA	miRNA	hsa-mir-182
t0057573	19	2	TAGTACCAGTACCTTGTGT	miRNA	hsa-mir-624
t0057597	20	2	AAGGAGCTCACAGTCTATTG	miRNA	hsa-mir-28
t0057887	19	2	CAATATTACTGTGCTGCTT	miRNA	hsa-mir-16-2
t0057891	22	2	GAGGTAGTAGATTGTATAGTTT	miRNA	hsa-let-7f-2

t0057942	23	2	AGGGGTGCTATCTGTGATTGAGG	miRNA	hsa-mir-342
t0058105	19	2	ATGGTAGAACTCACACTGG	miRNA	hsa-mir-182
t0058667	19	2	GAGGTAGTAGATTGTATAG	miRNA	hsa-let-7f-2
t0058999	20	2	AAAAGTACTTGCGGATTTTG	miRNA	hsa-mir-548k
t0059528	22	2	AAAGGTAATTGTGGTTTCTGCC	miRNA	hsa-mir-548ag-
t0059551	22	2	AGGGGCAGAGAGCGAGACTTTT	miRNA	hsa-mir-423
t0059659	20	2	GAGGTAGTAGGTTGTGTGGT	miRNA	hsa-let-7b
t0059917	20	2	TGTGGGACTTCTGGCCTTGA	miRNA	hsa-mir-3922
t0059921	23	2	CTCGGGGCAGCTCAGTACAGGAT	miRNA	hsa-mir-486
t0059961	22	2	AGTAGCAGCACATAATGGTTTG	miRNA	hsa-mir-15a
t0060098	21	2	AACGGCAATGACTTTTGTACC	miRNA	hsa-mir-548al
t0060138	20	2	AGGGGGAAAGTTCTATAGTC	miRNA	hsa-mir-625
t0060314	18	2	CATCATATACTGTAAGTT	miRNA	hsa-mir-144
t0060426	23	2	AAGTTCTGTTATACACTCAGGCT	miRNA	hsa-mir-148b
t0060435	22	2	TCAGGACACTTCTGAACTTGGA	miRNA	hsa-mir-5000
t0060452	22	2	ACAGCCCGGATCCCAGCCCACT	miRNA	hsa-mir-3940
t0060546	21	2	TTTCTCCTAAGGCAGTCCCTG	miRNA	hsa-mir-3199-2
t0060578	22	2	TTGCAGCTGCCTGGGAGTGACT	miRNA	hsa-mir-1301
t0060902	22	2	ATTTGGGACTGATCTTGATGTC	miRNA	hsa-mir-3913-2
t0061093	20	2	GGCAATGGTAGAACTCACAC	miRNA	hsa-mir-182
t0061425	19	2	CTATACAACCTACTGCCTT	miRNA	hsa-let-7b
t0061502	18	2	ATATCATCATATACTGTA	miRNA	hsa-mir-144
t0061744	20	2	CAGTAAGTGGCACTCTGTCT	miRNA	hsa-mir-4797
t0062147	20	2	ATTCAACGCTGTCGGTGAGT	miRNA	hsa-mir-181a-1
t0062156	19	2	TCAAGTAATTCAGGATAGG	miRNA	hsa-mir-26b
t0062383	21	2	AAACCATCGACCGTTGATTGT	miRNA	hsa-mir-181a-1
t0062490	21	2	AAAAGTTATTGCGGTTTTGGC	miRNA	hsa-mir-548at
t0062862	22	2	TTTGTTCGTTCGGCTCGCGTGA	miRNA	hsa-mir-375
t0062897	20	2	ATGACACGATCACTCCCGTT	miRNA	hsa-mir-425
t0063093	20	2	AGTGCACTACAGAACTTTGT	miRNA	hsa-mir-148a
t0063633	18	2	TGTAAACATCCCCGACTG	miRNA	hsa-mir-30d
t0063950	21	2	CTGAGATGAAGCACTGTAGCT	miRNA	hsa-mir-143
t0064070	20	2	GAGGAGCTCACAGTCTAGTA	miRNA	hsa-mir-151a
t0064355	20	2	TGGCACTGGTAGAATTCACT	miRNA	hsa-mir-183
t0064429	23	2	CGGGGCCGTAGCACTGTCTGAGA	miRNA	hsa-mir-128-1
t0064611	20	2	ACTGCTGAGCTAGCACTTCC	miRNA	hsa-mir-93
t0064616	21	2	ACTGCCCTAAATGCCCCTTCT	miRNA	hsa-mir-18b
t0064934	23	2	AACCCGTAGATCCGAACTTGTGG	miRNA	hsa-mir-100
t0065026	22	2	TTGGGGAAACGGCCGCTGAGTG	miRNA	hsa-mir-2110
t0065191	19	2	CGCATCCCCTAGGGCATTG	miRNA	hsa-mir-324
t0065303	20	2	TTAAGACTTGCAGTGATGTT	miRNA	hsa-mir-499a
t0065446	21	2	TGTTGTACTTTTTTTTTTGTT	miRNA	hsa-mir-3613
t0065772	23	2	ATTGCACTTGTCCCGGCCTGTTG	miRNA	hsa-mir-92a-1
t0065788	18	2	ACTGGACTTGGAGTCAGA	miRNA	hsa-mir-378d-1
t0065815	25	2	CTGTAAACATCCTTGACTGGAAGCT	miRNA	hsa-mir-30e
t0066113	21	2	CCAGTATTAACTGTGCTGCTG	miRNA	hsa-mir-16-1
t0066616	23	2	TGTAAACATCCTACACTCAGCTG	miRNA	hsa-mir-30b
t0066627	22	2	GAAGAACTGTTGCATTTGCCCT	miRNA	hsa-mir-4511
t0066652	24	2	TACCCATTGCATATCGGAGTTGTG	miRNA	hsa-mir-660
t0067186	21	2	AAGCAGCATTGTACAGGGCTA	miRNA	hsa-mir-107
t0067402	19	2	AACCGTGGCTTTCGATTGT	miRNA	hsa-mir-132
t0067558	20	2	TTTCAGATAACAGTATTACA	miRNA	hsa-mir-3942
t0067573	20	2	TTTAGGATAAGCTTGACTTT	miRNA	hsa-mir-651
t0067746	23	2	AGCGCGGGCTGAGCGCTGCCAGT	miRNA	hsa-mir-2277
t0067963	22	2	CTAAAGTGCTGACAGTGCAGAT	miRNA	hsa-mir-106b
t0068136	23	2	TTTGGGACTGATCTTGATGTCTG	miRNA	hsa-mir-3913-2
t0068257	19	2	CTGCCAGTTGAAGAACTGT	miRNA	hsa-mir-22
t0068587	19	2	CTGAGGTAGTAGTTTGTGC	miRNA	hsa-let-7i

t0068685	19	2	CTGTGCGTGTGACAGCGGC	miRNA	hsa-mir-210
t0069338	22	2	TTGAGGTAGTAGGTTGTATAGT	miRNA	hsa-let-7a-2
t0069496	19	2	TGGCAATGGTAGAACTCAC	miRNA	hsa-mir-182
t0069756	20	2	ATAGATCTGGATTGGAACCC	miRNA	hsa-mir-607
t0069934	20	2	ACCTCCTGTGTGCATGGATT	miRNA	hsa-mir-660
t0070301	21	2	CTCTACTGTAGTATGGGCACT	miRNA	hsa-mir-20b
t0070459	20	2	GTGCCTGAGGGAGTAAGAGC	miRNA	hsa-mir-550a-2
t0070578	19	2	AGTGTGCGGAAATGCTTCT	miRNA	hsa-mir-147b
t0070579	19	2	CAGGCCATATTGTGCTGCC	miRNA	hsa-mir-15a
t0070586	22	2	GAATCTGAGAAGGCGCACAAGG	miRNA	hsa-mir-3200
t0070589	23	2	GTAGAGGAGATGGCGCAGGGGAC	miRNA	hsa-mir-877
t0070601	21	2	CTAGCACCATCTGAAATCGGT	miRNA	hsa-mir-29a
t0070713	19	2	GTACTGTGATAACTGAAGA	miRNA	hsa-mir-101-2
t0070878	20	2	CCCATCCGCGCTCTGACTCT	miRNA	hsa-mir-937
t0071433	18	2	TGACCTATGAATTGACAG	miRNA	hsa-mir-215
t0071577	22	2	GAATGTTGCTCGGTGAACCCCT	miRNA	hsa-mir-409
t0071652	22	2	GTAGCAGCACATAATGGTTTGT	miRNA	hsa-mir-15a
t0072118	22	2	GTAGCTTATCAGACTGATGTTG	miRNA	hsa-mir-21
t0072126	25	2	AGCTACATCTGGCTACTGGGTCTCT	miRNA	hsa-mir-222
t0072148	21	2	TAACTGTACAGGCCACTGCCT	miRNA	hsa-let-7g
t0072323	21	2	GTGAATTACCGAAGGGCCATA	miRNA	hsa-mir-183
t0072336	21	2	TACAGTAGTCTGCACATTGGT	miRNA	hsa-mir-199b
t0072662	20	2	AGGGGCAGAGAGCGAGACTT	miRNA	hsa-mir-423
t0072665	19	2	GCAATGGTAGAACTCACAC	miRNA	hsa-mir-182
t0072673	20	2	TGAGGTAGTAGGTTGTATGG	miRNA	hsa-let-7c
t0072676	19	2	AACAGACATTAATTGGGCG	miRNA	hsa-mir-421
t0072689	20	2	GTGTCAGTTTGTCAAATACC	miRNA	hsa-mir-223
t0072730	27	2	ATACAACCTGATAAGTGTTATAGCACT	miRNA	hsa-mir-374a
t0073384	19	2	AAAAGTACTTGCGGATTTT	miRNA	hsa-mir-548k
t0073612	21	2	AAAACCGTCTAGTTACAGTTG	miRNA	hsa-mir-1537
t0073697	24	2	GTATTGCACTTGTCCCGGCCTGTT	miRNA	hsa-mir-92a-1
t0073780	21	2	CAAAAAAAAAAGCCCAACCCT	miRNA	hsa-mir-3613
t0073886	19	2	TTTCAGTCGGATGTTTACA	miRNA	hsa-mir-30e
t0073961	22	2	CAGTTATCACAGTGCTGATGCT	miRNA	hsa-mir-101-1
t0074171	21	2	TGCACGGCACTGGGGACACGT	miRNA	hsa-mir-3177
t0074324	20	2	TCCTCCTGCCCTCCTTGCTG	miRNA	hsa-mir-1976
t0074634	18	2	TCACAGTGAACCGGTCTC	miRNA	hsa-mir-128-2
t0074910	19	2	TCCCTGAGACCCTAACTTG	miRNA	hsa-mir-125b-2
t0075062	22	2	GCAGTGCAATGTTAAAAGGGCA	miRNA	hsa-mir-130a
t0075138	22	2	AGGAGCTCAACAGATGCCTGTT	miRNA	hsa-mir-3139
t0075214	22	2	TAGTACCAGTACCTTGTGTTCA	miRNA	hsa-mir-624
t0075479	23	2	TTACACAGCTGGACAGAGGCACG	miRNA	hsa-mir-4672
t0075500	20	2	CAAGTAATTCAGGATAGGTT	miRNA	hsa-mir-26b
t0075544	24	2	TGTGCAAATCCATGCAAAACTGAT	miRNA	hsa-mir-19b-2
t0075662	23	2	TCAGAACAAATGCCGGTTCCCAG	miRNA	hsa-mir-589
t0076001	19	2	CCCTCTCACCACTGCCCTC	miRNA	hsa-mir-1229
t0076203	24	2	TCTAGCAGCACGTAAATATTGGCG	miRNA	hsa-mir-16-2
t0076324	22	2	CAGCATTGTACAGGGCTATGAA	miRNA	hsa-mir-103a-1
t0076606	19	2	TCACAGTGGCTAAGTTCTG	miRNA	hsa-mir-27b
t0076628	24	2	TGTGCAAATCTATGCAAAACTGAT	miRNA	hsa-mir-19a
t0076851	20	2	CAACAGACATTAATTGGGCG	miRNA	hsa-mir-421
t0076928	20	2	CCATAAAGTAGAAAGCACTA	miRNA	hsa-mir-142
t0076999	22	2	TTCTAGTAAGAGTGGCAGTCGA	miRNA	hsa-mir-628
t0077156	21	2	TATAAAATGAGGGCAGTAAGA	miRNA	hsa-mir-3163
t0077329	22	2	GAGGTAGTAGATTGTATAGTTG	miRNA	hsa-let-7f-1
t0077549	18	2	TATATATATATATGTACG	miRNA	hsa-mir-1277
t0077804	18	2	GGTAGTAGTTTGTGCTGT	miRNA	hsa-let-7i
t0078241	19	2	TGCACTTGTCTCGGTCTGA	miRNA	hsa-mir-25

t0078279	20	2	TAGTACTGTGCATATCATCT	miRNA	hsa-mir-1278
t0078293	22	2	GAAACCGTTACCATTACTGAGT	miRNA	hsa-mir-451a
t0080041	23	2	GTAAACATCCCCGACTGGAAGCT	miRNA	hsa-mir-30d
t0080137	18	2	ACACGATCACTCCCGTTG	miRNA	hsa-mir-425
t0080457	24	2	ACCACAGGGTAGAACCACGGACAG	miRNA	hsa-mir-140
t0080639	20	2	AAAGACATAGGATAGAGTCA	miRNA	hsa-mir-641
t0080770	22	2	ACCCATTGCATATCGGAGTTGT	miRNA	hsa-mir-660
t0081420	23	2	CTGGAGATATGGAAGAGCTGTGT	miRNA	hsa-mir-1270-2
t0081680	22	2	TATAAAATGAGGGCAGTAAGAC	miRNA	hsa-mir-3163
t0081694	23	2	TTAGCAGCACGTAAATATTGGCG	miRNA	hsa-mir-16-1
t0081961	22	2	CTGCTGAGCTAGCACTTCCCGA	miRNA	hsa-mir-93
t0082037	21	2	TTCTGCCTCTGTCCAGGTCCT	miRNA	hsa-mir-5001
t0082083	24	2	CTGAGGGGCAGAGAGCGAGACTTT	miRNA	hsa-mir-423
t0082507	27	2	AAACCGTTACCATTACTGAGTTTAGTA	miRNA	hsa-mir-451a
t0082539	22	2	GCAGCATTGTACAGGGCTATGA	miRNA	hsa-mir-103a-1
t0082664	18	2	CTGCGCAAGCTACTGCCT	miRNA	hsa-let-7i
t0082795	24	2	TGTTCCTCTGTCTCCCAGACTCTG	miRNA	hsa-mir-4326
t0082894	24	2	TGAGGTAGTAGATTGTATAGTTTT	miRNA	hsa-let-7f-2
t0082900	21	2	GAGGTAGTAGTTTGTACAGTT	miRNA	hsa-let-7g
t0083067	21	2	CAGTAGTCTGCACATTGGTTA	miRNA	hsa-mir-199b
t0083071	23	2	GCATTGCACTTGTCTCGGTCTGA	miRNA	hsa-mir-25
t0083226	20	2	GGGGCAGCTCAGTACAGGAT	miRNA	hsa-mir-486
t0083882	18	2	CATTATTACTTTTGGTAC	miRNA	hsa-mir-126
t0084019	20	2	ACAAAAAAAAAAGCCCAACC	miRNA	hsa-mir-3613
t0084059	19	2	TCAAAGTGCTTACAGTGCA	miRNA	hsa-mir-17
t0084274	20	2	AACCACTGACCGTTGACTGT	miRNA	hsa-mir-181a-2
t0084517	21	2	CAGTACTGTGATAACTGAAGA	miRNA	hsa-mir-101-2
t0084607	22	2	AAAGACATAGGATAGAGTCACC	miRNA	hsa-mir-641
t0084995	22	2	ATCTCCAGTATTAACTGTGCTG	miRNA	hsa-mir-16-1
t0085000	22	2	TTCTAGGTATGGTCCCAGGGAT	miRNA	hsa-mir-331
t0085862	21	2	TCCCTGAGACCCTAACTTGTG	miRNA	hsa-mir-125b-2
t0086092	21	2	ACTCCAGCCCCACAGCCTCAG	miRNA	hsa-mir-766
t0086195	21	2	TTATCAGATTGTATTGTAATT	miRNA	hsa-mir-374a
t0086700	21	2	TTCCTGCTGAACTGAGCCAGT	miRNA	hsa-mir-3074
t0086915	20	2	AGCTACATCTGGCTACTGGG	miRNA	hsa-mir-222
t0087189	22	2	CGGGGCCGTAGCACTGTCTGAG	miRNA	hsa-mir-128-1
t0087224	19	2	ACCGTGGCTTTCGATTGTT	miRNA	hsa-mir-132
t0087312	20	2	AAAGTGCTTATAGTGCAGGT	miRNA	hsa-mir-20a
t0087321	22	2	AATGCACCTGGGCAAGGATTCT	miRNA	hsa-mir-500a
t0087600	22	2	AGTGCAATAGTATTGTCAAAGC	miRNA	hsa-mir-301a
t0087718	19	2	GTATGGCACTGGTAGAATT	miRNA	hsa-mir-183
t0088153	23	2	CATAAAGTAGAAAGCACTACTAA	miRNA	hsa-mir-142
t0088241	20	2	CACGCTCATGCACACACCCA	miRNA	hsa-mir-574
t0088444	22	2	AGCCTGGAAGCTGGAGCCTGCA	miRNA	hsa-mir-1254-2
t0089131	20	2	TCTTTTCTTTGAGACTCACT	miRNA	hsa-mir-627
t0089742	20	2	TGTGAGGTTGGCATTGTTGT	miRNA	hsa-mir-1294
t0090008	21	2	CTCGTACCGTGAGTAATAATG	miRNA	hsa-mir-126
t0090120	22	2	TAGGTAGTTTCCTGTTGTTGGG	miRNA	hsa-mir-196b
t0090182	23	2	TAGCAGCACATCATGGTTTACAT	miRNA	hsa-mir-15b
t0090315	20	2	AGGGCCCCCCCTCAATCCTG	miRNA	hsa-mir-296
t0091004	19	2	ACCGCACTGTGGGTACTTG	miRNA	hsa-mir-106b
t0091045	22	2	AGCAGCACGTAAATATTGGCGT	miRNA	hsa-mir-16-2
t0091492	22	2	CCTGGACACCGCTCAGCCGGCC	miRNA	hsa-mir-4638
t0091580	19	2	TCTCTCGGCTCCTCGCGGC	miRNA	hsa-mir-3615
t0091596	23	2	TGTCAGTTTGTCAAATACCCCAA	miRNA	hsa-mir-223
t0091650	23	2	ATTATTACTTTTGGTACGCGCTG	miRNA	hsa-mir-126
t0091667	19	2	GAGGAGCTCACAGTCTAGT	miRNA	hsa-mir-151a
t0091822	19	2	ACAGGGTAGAACCACGGAC	miRNA	hsa-mir-140

t0091840	19	2 AGGCTCAGTCCCCTCCCGA	miRNA	hsa-mir-484
t0092415	24	2 TCCCCCAGGTGTGATTCTGATTTG	miRNA	hsa-mir-361
t0092417	22	2 CTGGGAGAGGGTTGTTTACTCC	miRNA	hsa-mir-30c-1
t0092924	20	2 GGTACAGTACTGTGATAACT	miRNA	hsa-mir-101-2
t0092965	23	2 TCATATACTGTAAGTTTGCGATG	miRNA	hsa-mir-144
t0093796	21	2 TTCTTCTTAGACATGGCAGCT	miRNA	hsa-mir-4659b
t0093973	21	2 ATAGGACTCATATAGTGCCAG	miRNA	hsa-mir-3117
t0094348	22	2 GTACAAAAGCAATCGCGGTTTT	miRNA	hsa-mir-548e
t0094392	20	2 CAAAAGTAATTGCGGTCTTT	miRNA	hsa-mir-548ap
t0094438	22	2 CAACAAATCACAGTCTGCCATA	miRNA	hsa-mir-7-1
t0094966	19	2 ATATCATCATATACTGTAA	miRNA	hsa-mir-144
t0095095	20	2 TGCCTGAGGGAGTAAGAGCC	miRNA	hsa-mir-550a-2
t0095116	19	2 AAAAGCTGGGTTGAGAGGG	miRNA	hsa-mir-320c-2
t0095145	23	2 AGGACCTTCCCTGAACCAAGGAA	miRNA	hsa-mir-659
t0095183	20	2 ATTGCACTCGTCCCGGCCTC	miRNA	hsa-mir-92b
t0095622	22	2 CCGCACTGTGGGTACTTGCTGC	miRNA	hsa-mir-106b
t0095727	25	2 TCAAAGTGCTTACAGTGCAGGTAGT	miRNA	hsa-mir-17
t0095967	22	2 TACAGTATAGATGATGTACTAG	miRNA	hsa-mir-144
t0096165	21	2 AAAGTGCTTATAGTGCAGGTA	miRNA	hsa-mir-20a
t0096284	22	1 ATTGTCAGGTAGTGATAGGATT	miRNA	hsa-mir-4999
t0096399	24	1 TGTCAGTTTGTCAAATACCCCAAG	miRNA	hsa-mir-223
t0096481	23	1 AGAATTGCGTTTGGACAATCAGT	miRNA	hsa-mir-2964a
t0097095	20	1 ACAACAGTGCCAACCTCACA	miRNA	hsa-mir-1294
t0097546	19	1 TACTGCCCTAAGTGCTCCT	miRNA	hsa-mir-18a
t0097754	25	1 CTGTAAACATCCTACACTCTCAGCT	miRNA	hsa-mir-30c-2
t0097985	19	1 AGTGCAATGTTAAAAGGGC	miRNA	hsa-mir-130a
t0098758	22	1 TCTGTATTCTCCTTTGCCTGCA	miRNA	hsa-mir-4742
t0098873	24	1 TGAAGTTCTGTTATACACTCAGGC	miRNA	hsa-mir-148b
t0099340	20	1 CAGTGGTTTTACCCTATGGT	miRNA	hsa-mir-140
t0099469	18	1 TGTCAGTTTGTCAAATAC	miRNA	hsa-mir-223
t0099740	23	1 ATCAGGGCTTGTGGAATGGGAAG	miRNA	hsa-mir-3127
t0100045	22	1 CTGTACTGAGCTGCCCCGAGGC	miRNA	hsa-mir-486
t0100113	19	1 ATGACACGATCACTCCCGT	miRNA	hsa-mir-425
t0100309	19	1 CAGTACTGTGATAACTGAA	miRNA	hsa-mir-101-2
t0100573	22	1 CACACAGGTTATAGATCTGGAT	miRNA	hsa-mir-607
t0100804	22	1 ACCCTGTAGAACCGAATTTGTG	miRNA	hsa-mir-10b
t0100813	20	1 AGCAGCACATAATGGTTTGT	miRNA	hsa-mir-15a
t0100901	21	1 CTATACAATCTATTGCCTTCC	miRNA	hsa-let-7f-1
t0101573	22	1 TTAGCCAATTGTCCATCTTTAG	miRNA	hsa-mir-4662a
t0101941	19	1 TGAGGTAGGAGGTTGTATA	miRNA	hsa-let-7e
t0101985	21	1 TGGCCCGGCGACGTCTCACGG	miRNA	hsa-mir-4745
t0102475	20	1 GTCAGTTTGTCAAATACCCC	miRNA	hsa-mir-223
t0102933	24	1 AAGCAGCATTGTACAGGGCTATGA	miRNA	hsa-mir-103a-1
t0103264	22	1 ACTACAGTATAGATGATGTACT	miRNA	hsa-mir-144
t0103331	20	1 CAAAACGTGAGGCGCTGCTA	miRNA	hsa-mir-424
t0103334	21	1 TAAATAGAGTAGGCAAAGGAC	miRNA	hsa-mir-3121
t0103387	19	1 TACCGTGAGTAATAATGCG	miRNA	hsa-mir-126
t0103690	25	1 TGATATGTTTGATATATTAGGTTGT	miRNA	hsa-mir-190a
t0104155	20	1 AACCCGTAGATCCGATCTTG	miRNA	hsa-mir-99a
t0104343	18	1 ACATTCATTGCTGTCGGT	miRNA	hsa-mir-181b-2
t0104929	19	1 CTCGGCGTGGCGTCGGTCG	miRNA	hsa-mir-1307
t0105833	22	1 TTTGGCACTAGCACATTTTTGC	miRNA	hsa-mir-96
t0106275	20	1 AATGGTAATGGTTCTCTTGC	miRNA	hsa-mir-451a
t0106653	19	1 AGTACTGTGATAACTGAAG	miRNA	hsa-mir-101-2
t0107382	19	1 TTCACCACCTTCTCCACCC	miRNA	hsa-mir-197
t0107866	22	1 CCCTGACCTGTCCTGTTCTGCC	miRNA	hsa-mir-4732
t0108141	18	1 GATATCATCATATACTGT	miRNA	hsa-mir-144
t0108506	21	1 GACTATAGAACTTTCCCCCTC	miRNA	hsa-mir-625

t0108735	21	1	ATGGAAAGACTTTGCCACTCT	miRNA	hsa-mir-3688-2
t0108774	23	1	AGCTTATCAGACTGATGTTGACT	miRNA	hsa-mir-21
t0109174	22	1	TCGACCGGACCTCGACCGGCTC	miRNA	hsa-mir-1307
t0109312	18	1	TTATAAAGCAATGAGACT	miRNA	hsa-mir-340
t0109336	22	1	AGGTAGTAGATTGTATAGTTGT	miRNA	hsa-let-7f-1
t0109922	18	1	TCGAATCCCAGCGGTGCC	miRNA	hsa-mir-3676
t0109926	20	1	AAAGACTCTGCAAGATGCCT	miRNA	hsa-mir-4432
t0110469	23	1	ACATATGGAAAGACTTTGCCACT	miRNA	hsa-mir-3688-2
t0110522	21	1	CTAGATTGTGAGCTCCTGGAG	miRNA	hsa-mir-28
t0110727	20	1	TGAGGAGATCGTCGAGGTTG	miRNA	hsa-mir-3150b
t0110988	22	1	CCACTGCCCCAGGTGCTGCTGG	miRNA	hsa-mir-324
t0111500	21	1	AACTCTGACCCCTTAGGTTGA	miRNA	hsa-mir-4714
t0111655	20	1	TTACCATTACTGAGTTTAGT	miRNA	hsa-mir-451a
t0111666	22	1	AAAAGTTATTGTGGTTTTTGCT	miRNA	hsa-mir-548ac
t0111669	21	1	AAAAGTACTTGCGGATTTTGC	miRNA	hsa-mir-548k
t0112003	22	1	CAAGCTTGTATCTATAGGTATG	miRNA	hsa-mir-100
t0112112	21	1	GTGCATATTTACTTTAGGATG	miRNA	hsa-mir-559
t0112420	18	1	CAATGGTAGAACTCACAC	miRNA	hsa-mir-182
t0113069	20	1	TCTGGCTGTTGTGGTGTGCA	miRNA	hsa-mir-3064
t0113155	19	1	TTTCAGTCAGATGTTTGCT	miRNA	hsa-mir-30d
t0113522	24	1	CAGTGCAATGATATTGTCAAAGCA	miRNA	hsa-mir-301b
t0114035	20	1	CTACTGCTGAGCTAGCACTT	miRNA	hsa-mir-93
t0114476	20	1	AACGGAATCCCAAAAGCAGC	miRNA	hsa-mir-191
t0114505	21	1	TTTCCGGCTCGCGTGGGTGTG	miRNA	hsa-mir-1180
t0115429	24	1	TAATCCTTGCTACCTGGGTGAGAG	miRNA	hsa-mir-500b
t0115812	18	1	GGTAGTAGGTTGCATAGT	miRNA	hsa-let-7d
t0116383	22	1	TGTCCTCTAGGGCCTGCAGTCT	miRNA	hsa-mir-3909
t0116603	21	1	ATGACCTATGAATTGACAGAC	miRNA	hsa-mir-215
t0116714	20	1	TGAATTACCGAAGGGCCATA	miRNA	hsa-mir-183
t0117686	24	1	GGGAGCCAGGAAGTATTGATGTTT	miRNA	hsa-mir-505
t0117732	22	1	TACCCAGTCTCCGGTGCAGCCT	miRNA	hsa-mir-3130-2
t0117995	19	1	AAGTAATTCAGGATAGGTT	miRNA	hsa-mir-26b
t0118010	23	1	TGGAAGACTAGTGATTTTGTTGT	miRNA	hsa-mir-7-3
t0118259	20	1	CGGCGGGGACGGCGATTGGT	miRNA	hsa-mir-1908
t0118541	23	1	AATCCTTGCTATCTGGGTGCTAG	miRNA	hsa-mir-502
t0118923	20	1	GCAGCACATCATGGTTTACA	miRNA	hsa-mir-15b
t0118968	21	1	TGACCTATGAATTGACAGACA	miRNA	hsa-mir-215
t0119413	21	1	TAGCAGCACAGAAATATTGGC	miRNA	hsa-mir-195
t0120578	20	1	ATTTAGGCGTCAGACTACCT	miRNA	hsa-mir-5696
t0120627	19	1	CTCGGTCTGAGGCCCCTCA	miRNA	hsa-mir-423
t0120919	20	1	ACCATCGACCGTTGAGTGGA	miRNA	hsa-mir-181c
t0120973	21	1	TAACTATACGACCTGCTGCCT	miRNA	hsa-let-7d
t0121012	19	1	CTTATCAGACTGATGTTGA	miRNA	hsa-mir-21
t0121199	20	1	AGTGCTCATAGTGCAGGTAG	miRNA	hsa-mir-20b
t0121522	21	1	AGAGGAGATGGCGCAGGGGAC	miRNA	hsa-mir-877
t0121540	19	1	AGGTGACTGTCCTATGTCT	miRNA	hsa-mir-641
t0121936	23	1	AAACTCTACTTGTCCTTCTGAGT	miRNA	hsa-mir-618
t0121998	20	1	AGATAACTATACAACCTACT	miRNA	hsa-let-7b
t0122553	22	1	CGGCCCCACGCACCAGGGTAAG	miRNA	hsa-mir-874
t0122745	22	1	TTTTTCATTATTGCTCCTGACC	miRNA	hsa-mir-335
t0122824	20	1	CCCTGTGGACTCAGTTCTGG	miRNA	hsa-mir-146b
t0123041	23	1	CAACACCAGTCGATGGGCTGTCT	miRNA	hsa-mir-21
t0123139	21	1	TCAGCAAACATTTATTGTGTG	miRNA	hsa-mir-545
t0123418	19	1	AAAGTGCTGACAGTGCAGA	miRNA	hsa-mir-106b
t0123569	21	1	TTTTAGAGACGGGGTCTTGCT	miRNA	hsa-mir-1303
t0123805	21	1	ATCACATTGCCAGGGATTACC	miRNA	hsa-mir-23b
t0124147	20	1	CTATACAGTCTACTGTCTTT	miRNA	hsa-let-7f-2
t0124375	18	1	TGTACTTTTTTTTTTGTT	miRNA	hsa-mir-3613

t0124866	19	1	ACCACAGGGTAGAACCACG	miRNA	hsa-mir-140
t0125215	23	1	GAAAAGCTGGGTTGAGAGGGCAA	miRNA	hsa-mir-320b-2
t0125667	24	1	TGACCTATGAATTGACAGCCAGTG	miRNA	hsa-mir-192
t0125719	21	1	CTGTGAGGTTGGCATTGTTGT	miRNA	hsa-mir-1294
t0125766	21	1	TGTTCCTGCTGAACTGAGCCA	miRNA	hsa-mir-3074
t0125963	21	1	ACAGGGTAGAACCACGGACAG	miRNA	hsa-mir-140
t0126171	19	1	TCGTACCGTGAGTAATAAT	miRNA	hsa-mir-126
t0126321	21	1	TCCCTGTCCTCCAGGAGCTCA	miRNA	hsa-mir-339
t0126790	20	1	CGCATAATATGGACATGTTA	miRNA	hsa-mir-3912
t0126918	22	1	TAGAGGAGATGGCGCAGGGGAC	miRNA	hsa-mir-877
t0127298	22	1	CAGTGCAATGATATTGTCAAAG	miRNA	hsa-mir-301b
t0128569	21	1	CCGGTCCCAGGAGAACCTGCA	miRNA	hsa-mir-4746
t0129068	22	1	ACCGTTACCATTACTGAGTTTA	miRNA	hsa-mir-451a
t0129567	23	1	CCTCCGTGTTACCTGTCCTCTAG	miRNA	hsa-mir-3605
t0129598	23	1	CCACTAGATTGTGAGCTCCTGGA	miRNA	hsa-mir-28
t0129601	21	1	CAGTTATCACAGTGCTGATGC	miRNA	hsa-mir-101-1
t0129691	23	1	TGTTCCTCTGTCTCCCAGACTCT	miRNA	hsa-mir-4326
t0129931	21	1	AGGCTGGTGCAAAAGTAATGG	miRNA	hsa-mir-548q
t0130045	22	1	CAATGTTGGAATCCTCGCTAGA	miRNA	hsa-mir-4781
t0130137	23	1	TCGGTTATCATGGTACCGATGCT	miRNA	hsa-mir-101-2
t0130363	24	1	ATCCTGTACTGAGCTGCCCCGAGG	miRNA	hsa-mir-486
t0130703	19	1	TCCCTGTCCTCCAGGAGCT	miRNA	hsa-mir-339
t0131001	21	1	ATATGGAAAGACTTTGCCACT	miRNA	hsa-mir-3688-2
t0131221	20	1	GAATGGCGCCACTAGGGTTG	miRNA	hsa-mir-652
t0131239	23	1	TAGTGTTTCCTACTTTATGGATG	miRNA	hsa-mir-142
t0131427	25	1	TTTTGGCAATGGTAGAACTCACACT	miRNA	hsa-mir-182
t0131854	20	1	TAAGGGGTGTATGGCAGATG	miRNA	hsa-mir-3936
t0132379	22	1	TTCACCACCTTCTCCACCCAGC	miRNA	hsa-mir-197
t0132492	21	1	AGGGCCCCCCCTCAATCCTGT	miRNA	hsa-mir-296
t0132564	21	1	TTTCTATTTCTCAGTGGGGCT	miRNA	hsa-mir-4482-2
t0133516	18	1	ATGGCGCCACTAGGGTTG	miRNA	hsa-mir-652
t0133791	25	1	AATCTGAGAAGGCGCACAAGGTTTG	miRNA	hsa-mir-3200
t0133874	19	1	GCTGCGCTTGGATTTCGTC	miRNA	hsa-mir-191
t0134063	18	1	ACCACTGACCGTTGACTG	miRNA	hsa-mir-181a-2
t0134324	23	1	TTAGGGTCATACCCCATCTTGGA	miRNA	hsa-let-7f-2
t0134328	23	1	CAAAGACTGCAATTACTTTTGCG	miRNA	hsa-mir-548u
t0135252	21	1	AACAAATCACAGTCTGCCATA	miRNA	hsa-mir-7-1
t0135377	21	1	CAAAACGTGAGGCGCTGCTAT	miRNA	hsa-mir-424
t0135921	19	1	CACTGTTTCACCACTGGCT	miRNA	hsa-mir-4676
t0137416	18	1	TGTAAACATCCTACACTC	miRNA	hsa-mir-30c-1
t0137531	18	1	CTGGAGATATGGAAGAGC	miRNA	hsa-mir-1270-2
t0137710	18	1	TAATGGTAATGGTTCTCT	miRNA	hsa-mir-451a
t0137820	24	1	CAGTGCAATGTTAAAAGGGCATTG	miRNA	hsa-mir-130a
t0138816	21	1	AACGCATAATATGGACATGTT	miRNA	hsa-mir-3912
t0138830	20	1	ACACCAATATTACTGTGCTG	miRNA	hsa-mir-16-2
t0139077	20	1	GCTGCGCTTGGATTTCGTCC	miRNA	hsa-mir-191
t0139487	29	1	GATATCATCATATACTGTAAGTTTGCGAT	miRNA	hsa-mir-144
t0139580	24	1	AACCGTTACCATTACTGAGTTTAG	miRNA	hsa-mir-451a
t0140447	22	1	TAATCCTTGCTATCTGGGTGCT	miRNA	hsa-mir-502
t0140458	20	1	TATGGTTTGCCTGGGACTGA	miRNA	hsa-mir-584
t0140476	23	1	GGGGTGCTATCTGTGATTGAGGG	miRNA	hsa-mir-342
t0140661	20	1	TCTGCCTCTGTCCAGGTCCT	miRNA	hsa-mir-5001
t0141033	20	1	GCCCTGTGGACTCAGTTCTG	miRNA	hsa-mir-146b
t0141577	19	1	AAAAGTATTTGCGGGTTTT	miRNA	hsa-mir-548l
t0141821	19	1	AGGTAGTAGTTTGTGCTGT	miRNA	hsa-let-7i
t0142288	23	1	ATATCATCATATACTGTAAGTTT	miRNA	hsa-mir-144
t0142426	18	1	CAGTTATCACAGTGCTGA	miRNA	hsa-mir-101-1
t0143003	19	1	GCAGCATTGTACAGGGCTA	miRNA	hsa-mir-107

t0143164	18	1	ATGGTAATGGTTCTCTTG	miRNA	hsa-mir-451a
t0143224	18	1	CCGTAGATCCGATCTTGT	miRNA	hsa-mir-99a
t0143304	26	1	CGTGTATTTGACAAGCTGAGTTGGAC	miRNA	hsa-mir-223
t0143613	22	1	AGGGGTGCTATCTGTGATTGAG	miRNA	hsa-mir-342
t0143673	21	1	ACTAGATTGTGAGCTCCTGGA	miRNA	hsa-mir-28
t0143686	21	1	TCTGGATTGGAACCCAGGGAG	miRNA	hsa-mir-607
t0143749	21	1	GAGCGCCTCGACGACAGAGCC	miRNA	hsa-mir-339
t0143761	20	1	GTAGTGTTTCCTACTTTATG	miRNA	hsa-mir-142
t0144558	18	1	TGTAAACATCCTTGACTG	miRNA	hsa-mir-30e
t0144622	21	1	AAGGAGCTCACAGTCTATTGA	miRNA	hsa-mir-28
t0145166	21	1	AAAAACTGAGACTACTTTTGC	miRNA	hsa-mir-548e
t0145353	23	1	ACTGCATTATGAGCACTTAAAGT	miRNA	hsa-mir-20a
t0145579	23	1	TCTAGTAAGAGTGGCAGTCGAAG	miRNA	hsa-mir-628
t0146196	25	1	TGTAAACATCCTTGACTGGAAGCTG	miRNA	hsa-mir-30e
t0146290	24	1	AAAGTGCTGTTCGTGCAGGTAGTG	miRNA	hsa-mir-93
t0146562	18	1	ACCTCCTGTGTGCATGGA	miRNA	hsa-mir-660
t0147397	21	1	TTGGTGCAAAAGTAATTGTGG	miRNA	hsa-mir-548ad
t0147527	20	1	TACGCGCAGACCACAGGATG	miRNA	hsa-mir-3939
t0148322	19	1	AATGCACCCGGGCAAGGAT	miRNA	hsa-mir-501
t0148518	19	1	AAAACGTGAGGCGCTGCTA	miRNA	hsa-mir-424
t0148956	24	1	GGGGTGCTATCTGTGATTGAGGGA	miRNA	hsa-mir-342
t0149754	23	1	TCTTTGGTTATCTAGCTGTATGA	miRNA	hsa-mir-9-3
t0150125	20	1	TGCCCTGCCTGTTTTCTCCT	miRNA	hsa-mir-3173
t0150166	22	1	AGTAAGTGGCACTCTGTCTTCT	miRNA	hsa-mir-4797
t0150237	20	1	CAAAAGCAATCGCGGTTTTT	miRNA	hsa-mir-548e
t0150742	18	1	ACGCATAATATGGACATG	miRNA	hsa-mir-3912
t0151296	18	1	GCCCTGACCTGTCCTGTT	miRNA	hsa-mir-4732
t0152062	22	1	CTAGCACCATCTGAAATCGGTT	miRNA	hsa-mir-29a
t0152110	23	1	CTGGTTTCACATGGTGGCTTAGA	miRNA	hsa-mir-29b-2
t0152143	19	1	TCACAGTGGCTAAGTTCCG	miRNA	hsa-mir-27a
t0152202	19	1	TTCTTCTTAGACATGGCAA	miRNA	hsa-mir-4659a
t0152274	19	1	GTGCTTACAGTGCAGGTAG	miRNA	hsa-mir-106a
t0152682	19	1	CAAGTAATCCAGGATAGGC	miRNA	hsa-mir-26a-2
t0152715	20	1	CCTCCTGCCCTCCTTGCTGT	miRNA	hsa-mir-1976
t0152791	23	1	TTATAAAGCAATGAGACTGATTG	miRNA	hsa-mir-340
t0152825	21	1	TTCTAGGTATGGTCCCAGGGA	miRNA	hsa-mir-331
t0153303	19	1	TCCCTGAGACCCTTTAACC	miRNA	hsa-mir-125a
t0153569	20	1	TACTGCAGTGAAGGCACTTG	miRNA	hsa-mir-17
t0154176	20	1	GCTGCCAGTTGAAGAACTGT	miRNA	hsa-mir-22
t0154714	22	1	TGCACAGCAAGTGTAGACAGGC	miRNA	hsa-mir-3120
t0154788	21	1	TGGCAATGGTAGAACTCACAC	miRNA	hsa-mir-182
t0156143	21	1	ACGTTGGCTCTGGTGGTGATG	miRNA	hsa-mir-1306
t0156454	18	1	ATCCTGTACTGAGCTGCC	miRNA	hsa-mir-486
t0156544	20	1	ACGGGTTAGGCTCTTGGGAG	miRNA	hsa-mir-125b-1
t0157001	22	1	TAAACATCCTACACTCTCAGCT	miRNA	hsa-mir-30c-1
t0157052	21	1	CCAAAGGTGAATTTTTTGGGA	miRNA	hsa-mir-186
t0157185	21	1	TTCTCCCAACGTAAGCCCAGC	miRNA	hsa-mir-629
t0157793	22	1	AACTGGCCTACAAAGTCCCAGT	miRNA	hsa-mir-193a
t0158340	24	1	AGTGCAATATTGCTTATAGGGTTT	miRNA	hsa-mir-454
t0158569	23	1	TTATGGTTTGCCTGGGACTGAGG	miRNA	hsa-mir-584
t0158672	20	1	CAAAAACTGTAATTACTTTT	miRNA	hsa-mir-548f-4
t0159169	23	1	AGGTTGGGATCGGTTGCAATGCT	miRNA	hsa-mir-92a-1
t0159599	21	1	CGGCCCCACGCACCAGGGTAA	miRNA	hsa-mir-874
t0159668	25	1	AGTGCAATATTGCTTATAGGGTTTT	miRNA	hsa-mir-454
t0159677	22	1	CTTAGCAGGTTGTATTATCATT	miRNA	hsa-mir-374b
t0159808	21	1	TACTGCAGTGAAGGCACTTGT	miRNA	hsa-mir-17
t0160466	21	1	ATCACATTGCCAGGGATTTCC	miRNA	hsa-mir-23a
t0160471	20	1	TTTGCCACACTGCAACACCT	miRNA	hsa-mir-3064

t0160776	20	1	GTACAAAAGCAATCGCGGTT	miRNA	hsa-mir-548e
t0160966	21	1	CAGTGGTTTTACCCTATGGTA	miRNA	hsa-mir-140
t0161289	18	1	TGAGGGGCAGAGAGCGAG	miRNA	hsa-mir-423
t0161680	23	1	TAGCACCATTTGAAATCGGTTAT	miRNA	hsa-mir-29c
t0161693	26	1	AAGGAAACCGTTACCATTACTGAGTT	miRNA	hsa-mir-451a
t0162078	20	1	TGTTGTACTTTTTTTTTTGT	miRNA	hsa-mir-3613
t0162382	19	1	TATATATATATATGTACGT	miRNA	hsa-mir-1277
t0162462	21	1	AGCTTCTTTACAGTGCTGCCT	miRNA	hsa-mir-103a-2
t0162571	22	1	CTAGCGGGGATTCCAATATTGG	miRNA	hsa-mir-4781
t0162706	18	1	TAATCCTTGCTACCTGGG	miRNA	hsa-mir-500b
t0162738	20	1	CCTCCGTGTTACCTGTCCTC	miRNA	hsa-mir-3605
t0162821	20	1	AGCATTGTACAGGGCTATGA	miRNA	hsa-mir-103a-1
t0163541	21	1	TAACTGGTTGAACAACTGAAC	miRNA	hsa-mir-582
t0163565	19	1	AACATTCATTGTTGTCGGT	miRNA	hsa-mir-181d
t0163716	20	1	AACCATCGACCGTTGATTGT	miRNA	hsa-mir-181a-1
t0163888	22	1	AGGCAGTGTATTGCTAGCGGCT	miRNA	hsa-mir-449c
t0163921	20	1	GTAGTACCAGTACCTTGTGT	miRNA	hsa-mir-624
t0163983	25	1	TGAGGTAGTAGATTGTATAGTTGTG	miRNA	hsa-let-7f-1
t0164052	22	1	GCAAAGCACACGGCCTGCAGAG	miRNA	hsa-mir-330
t0164079	23	1	TGTGCTTGCTCGTCCCGCCCGCA	miRNA	hsa-mir-636
t0164316	21	1	AGAATTGCGTTTGGACAATCA	miRNA	hsa-mir-2964a
t0164359	19	1	TCCCCCAGGTGTGATTCTG	miRNA	hsa-mir-361
t0164419	18	1	TAGGTAGTTTCCTGTTGT	miRNA	hsa-mir-196b
t0164451	20	1	TATACAAGGGCAAGCTCTCT	miRNA	hsa-mir-381
t0164885	23	1	CAGTACTGTGATAACTGAAGAAT	miRNA	hsa-mir-101-2
t0165429	19	1	GCTGCCAGTTGAAGAACTG	miRNA	hsa-mir-22
t0165468	21	1	TATACGACCTGCTGCCTTTCT	miRNA	hsa-let-7d
t0165482	19	1	CAGCTGCCTGGGAGTGACT	miRNA	hsa-mir-1301
t0165509	26	1	AAAGTATTGCACTTGTCCCGGCCTGT	miRNA	hsa-mir-92a-2
t0165889	23	1	TTTTGGCAATGGTAGAACTCACA	miRNA	hsa-mir-182
t0165899	24	1	TCAGAACAAATGCCGGTTCCCAGA	miRNA	hsa-mir-589
t0166177	22	1	GCTCTGACTTTATTGCACTACT	miRNA	hsa-mir-301a
t0166179	21	1	TGAGGAGATCGTCGAGGTTGG	miRNA	hsa-mir-3150b
t0166442	21	1	AACCCGTAGATCCGAACTTGT	miRNA	hsa-mir-100
t0166646	20	1	AAAAGTAATCACAGTTTTTG	miRNA	hsa-mir-548f-1
t0166704	21	1	GCAGTGCAATGATGAAAGGGC	miRNA	hsa-mir-130b
t0166761	21	1	ATGGCATCGTCCCCTGGTGGC	miRNA	hsa-mir-4642
t0166793	19	1	TCTTTACAGTGCTGCCTTG	miRNA	hsa-mir-103a-1
t0167101	20	1	TACTGCCCTAAGTGCTCCTT	miRNA	hsa-mir-18a
t0167165	20	1	CTGTACAGGCCACTGCCTTG	miRNA	hsa-let-7g
t0167248	21	1	TTTTGCAATATGTTCCTGAAT	miRNA	hsa-mir-450b
t0167942	20	1	TGATATGTTTGATATATTAG	miRNA	hsa-mir-190a
t0168382	19	1	ATGGCACTGGTAGAATTCA	miRNA	hsa-mir-183
t0168440	22	1	AGTTGTTCGTGGTGGATTCGCT	miRNA	hsa-mir-382
t0168747	20	1	TCTGTATTCTCCTTTGCCTG	miRNA	hsa-mir-4742
t0168860	18	1	TTCAACGCTGTCGGTGAG	miRNA	hsa-mir-181a-1
t0168947	23	1	AAAGTGCTTATAGTGCAGGTAGT	miRNA	hsa-mir-20a
t0169160	21	1	TGAGACCTCTGGGTTCTGAGC	miRNA	hsa-mir-769
t0169181	19	1	TAGTGCAATATTGCTTATA	miRNA	hsa-mir-454
t0169228	21	1	CAAAAACCGCAATTATTTTTG	miRNA	hsa-mir-548k
t0169416	18	1	TCTCCCAACCCTTGTACC	miRNA	hsa-mir-150
t0169801	21	1	AGCAGCACATCATGGTTTACA	miRNA	hsa-mir-15b
t0169960	18	1	TTTAGGATAAGCTTGACT	miRNA	hsa-mir-651
t0170007	19	1	CAGTGGTTTTACCCTATGG	miRNA	hsa-mir-140
t0170393	23	1	TGACACGATCACTCCCGTTGAGT	miRNA	hsa-mir-425
t0170405	20	1	TGTCTCCCAACCCTTGTACC	miRNA	hsa-mir-150
t0170562	20	1	TCAGAACAAATGCCGGTTCC	miRNA	hsa-mir-589
t0171144	23	1	GTCCAGTTTTCCCAGGAATCCCT	miRNA	hsa-mir-145

t0171212	18	1	TCTAGTAAGAGTGGCAGT	miRNA	hsa-mir-628
t0171431	23	1	TAATGCTAATCGTGATAGGGGTT	miRNA	hsa-mir-155
t0171782	19	1	TTTCTTCTTAGACATGGCA	miRNA	hsa-mir-4659b
t0171827	19	1	AACACAAGGTATTGGTATT	miRNA	hsa-mir-624
t0171847	21	1	TGGAAGACTAGTGATTTTGTT	miRNA	hsa-mir-7-3
t0172017	23	1	CCTCTCACCACTGCCCTCCCACA	miRNA	hsa-mir-1229
t0172170	20	1	ACTGCCCTAAGTGCTCCTTC	miRNA	hsa-mir-18a
t0172555	20	1	TTAATTTTTTGTTTCGGTCA	miRNA	hsa-mir-4775
t0173332	19	1	ATGCACCTGGGCAAGGATT	miRNA	hsa-mir-502
t0173356	19	1	TAGACTGAAGCTCCTTGAG	miRNA	hsa-mir-151a
t0173745	19	1	ATAACATTGTAAAGCGCTT	miRNA	hsa-mir-3143
t0173950	21	1	CTGAGGTAGTAGTTTGTGCTG	miRNA	hsa-let-7i
t0174295	20	1	ACATATGGAAAGACTTTGCC	miRNA	hsa-mir-3688-2
t0174362	23	1	CCAAAGAATTCTCCTTTTGGGCT	miRNA	hsa-mir-186
t0174383	20	1	GTTCACAGTGGCTAAGTTCT	miRNA	hsa-mir-27b
t0175077	19	1	CCATGACGTCACAGAGGCT	miRNA	hsa-mir-4757
t0175135	21	1	GACGTTGGCTCTGGTGGTGAT	miRNA	hsa-mir-1306
t0175425	21	1	GACTCCAAGAAGAATCTAGAC	miRNA	hsa-mir-5695
t0175663	23	1	TCGTACCGTGAGTAATAATGCGC	miRNA	hsa-mir-126
t0175684	23	1	TCTCTGGGCCTGTGTCTTAGGCT	miRNA	hsa-mir-330
t0175723	21	1	ACCACTGACCGTTGACTGTAC	miRNA	hsa-mir-181a-2
t0176115	18	1	AAGTAATTCAGGATAGGT	miRNA	hsa-mir-26b
t0176149	21	1	TACCCAGTCTCCGGTGCAGCC	miRNA	hsa-mir-3130-2
t0176387	20	1	TGAGAACTGAATTCCATGGG	miRNA	hsa-mir-146a
t0177275	18	1	CAGTATAGATGATGTACT	miRNA	hsa-mir-144
t0177524	19	1	GTGCACTACAGAACTTTGT	miRNA	hsa-mir-148a
t0177683	22	1	TGGGGCGGAGCTTCCGGAGGCC	miRNA	hsa-mir-3180-5
t0177811	20	1	TATCAGATTGTATTGTAATT	miRNA	hsa-mir-374a
t0178077	21	1	TGAATGGCGCCACTAGGGTTG	miRNA	hsa-mir-652
t0178190	22	1	TAGTACTGTGCATATCATCTAT	miRNA	hsa-mir-1278
t0178877	23	1	GATTGTTGTACTTTTTTTTTTGT	miRNA	hsa-mir-3613
t0179307	24	1	AAAAGCTGGGTTGAGAGGGCAAAA	miRNA	hsa-mir-320b-2
t0179551	20	1	AGCAATACTGTTACCTGAAA	miRNA	hsa-mir-3942
t0179760	21	1	AGGGCTTCCTCTCTGCAGGAC	miRNA	hsa-mir-3158-2
t0180017	21	1	ACTATAGAACTTTCCCCCTCA	miRNA	hsa-mir-625
t0180179	18	1	TCAGGCTCAGTCCCCTCC	miRNA	hsa-mir-484
t0180948	22	1	TGTAAACATCCTCGACTGGAAG	miRNA	hsa-mir-30a
t0181519	23	1	ACATTCAACGCTGTCGGTGAGTT	miRNA	hsa-mir-181a-1
t0181696	21	1	CGTTACCATTACTGAGTTTAG	miRNA	hsa-mir-451a
t0181910	21	1	TGTCCTCTAGGGCCTGCAGTC	miRNA	hsa-mir-3909
t0181986	22	1	CCACTTGGATCTGAAGGCTGCC	miRNA	hsa-mir-3614
t0182022	22	1	GTCATTTTTGTGATCTGCAGCT	miRNA	hsa-mir-153-1
t0182371	21	1	CTCGGTCTGAGGCCCCTCAGT	miRNA	hsa-mir-423
t0182463	20	1	TGCTCTGACTTTATTGCACT	miRNA	hsa-mir-301a
t0182749	22	1	TATCAGATTGTATTGTAATTGT	miRNA	hsa-mir-374a
t0183222	20	1	CTCACTGAACAATGAATGCA	miRNA	hsa-mir-181b-1
t0183902	19	1	CATCGGGAATGTCGTGTCC	miRNA	hsa-mir-425
t0183915	19	1	GAATCATTATTTGCTGCTC	miRNA	hsa-mir-15b
t0184320	23	1	CTGGTGCAAAAGTAATGGCGGTT	miRNA	hsa-mir-548q
t0184360	22	1	ACTTATCAGATTGTATTGTAAT	miRNA	hsa-mir-374a
t0184912	20	1	CTCGTACCGTGAGTAATAAT	miRNA	hsa-mir-126
t0186216	20	1	TGTCAGTTTGTCAAATACCC	miRNA	hsa-mir-223
t0186376	22	1	TAGCAGCGGGAACAGTTCTGCA	miRNA	hsa-mir-503
t0186635	18	1	GTAAACATCCTTGACTGG	miRNA	hsa-mir-30e
t0186927	20	1	ACGCATAATATGGACATGTT	miRNA	hsa-mir-3912
t0186958	19	1	TCTGTATTCTCCTTTGCCT	miRNA	hsa-mir-4742
t0186974	22	1	AGACTGAAGCTCCTTGAGGACA	miRNA	hsa-mir-151a
t0187153	25	1	GTACAGTACTGTGATAACTGAAGAA	miRNA	hsa-mir-101-2

t0187420	25	1	CAACGGAATCCCAAAAGCAGCTGTT	miRNA	hsa-mir-191
t0187713	20	1	ATGGGGCTTCTGTAGAGATT	miRNA	hsa-mir-3675
t0188355	24	1	GGCTGAGGTAGTAGTTTGTGCTGT	miRNA	hsa-let-7i
t0188660	20	1	TAACATTGTAAAGCGCTTCT	miRNA	hsa-mir-3143
t0188825	18	1	TACTGTAGTATGGGCACT	miRNA	hsa-mir-20b
t0189012	21	1	CTATACAACTTACTACTTTCC	miRNA	hsa-mir-98
t0189470	24	1	TACCCTGTAGATCCGAATTTGTGT	miRNA	hsa-mir-10a
t0190038	21	1	TAATGCCCCTAAAAATCCTTA	miRNA	hsa-mir-365b
t0190955	20	1	AAAGCTGGGTTGAGAGGGCA	miRNA	hsa-mir-320b-2
t0191392	23	1	GTGAGGTAGTAGATTGTATAGTT	miRNA	hsa-let-7f-1
t0191480	24	1	GTAAACATCCTTGACTGGAAGCTG	miRNA	hsa-mir-30e
t0191618	21	1	AGCGCCTCGACGACAGAGCCG	miRNA	hsa-mir-339
t0191674	18	1	GTGCTCATAGTGCAGGTA	miRNA	hsa-mir-20b
t0191966	20	1	CATCGGGAATGTCGTGTCCG	miRNA	hsa-mir-425
t0192159	19	1	TGTTCCTCTGTCTCCCAGA	miRNA	hsa-mir-4326
t0193004	19	1	ACCCTGTAGATCCGAATTT	miRNA	hsa-mir-10a
t0193511	20	1	ACCCTGTAGAACCGAATTTG	miRNA	hsa-mir-10b
t0193649	21	1	AGTGCAATAGTATTGTCAAAG	miRNA	hsa-mir-301a
t0193746	21	1	ATCGGGAATGTCGTGTCCGCC	miRNA	hsa-mir-425
t0193772	20	1	CACAGGGTAGAACCACGGAC	miRNA	hsa-mir-140
t0194455	22	1	GTGAGTCTCTAAGAAAAGAGGA	miRNA	hsa-mir-627
t0195668	21	1	AGTGTTTCCTACTTTATGGAT	miRNA	hsa-mir-142
t0195827	19	1	TATCAGACTGATGTTGACT	miRNA	hsa-mir-21
t0195934	19	1	TACCCTGTAGATCCGAATT	miRNA	hsa-mir-10a
t0196018	22	1	TCACACAGAAATCGCACCCGTC	miRNA	hsa-mir-342
t0196286	22	1	ATCAAGGATCTTAAACTTTGCC	miRNA	hsa-mir-561
t0196506	20	1	CATCCTGTACTGAGCTGCCC	miRNA	hsa-mir-486
t0197182	23	1	ATGGTAATGGTTCTCTTGCTATA	miRNA	hsa-mir-451a
t0197829	21	1	GATAACTATACAATCTACTGT	miRNA	hsa-let-7a-3
t0198678	23	1	TCGAGGAGCTCACAGTCTAGTAT	miRNA	hsa-mir-151a
t0198766	22	1	TGTATGGCACTGGTAGAATTCA	miRNA	hsa-mir-183
t0198858	23	1	CTTCACCACCTTCTCCACCCAGC	miRNA	hsa-mir-197
t0199018	18	1	TACCACAGGGTAGAACCA	miRNA	hsa-mir-140
t0199584	21	1	GCCCCGGGCAGTGTGATCATC	miRNA	hsa-mir-5587
t0199670	25	1	TGAGAACTGAATTCCATAGGCTGTG	miRNA	hsa-mir-146b
t0199996	18	1	TATAGGACTCATATAGTG	miRNA	hsa-mir-3117
t0200657	23	1	ACATTCATTGCTGTCGGTGGGTT	miRNA	hsa-mir-181b-2
t0200778	21	1	AAATTGCACGGTATCCATCTG	miRNA	hsa-mir-363
t0200833	18	1	ATCAGACTGATGTTGACT	miRNA	hsa-mir-21
t0201005	22	1	TATTGCACATTACTAAGTTGCA	miRNA	hsa-mir-32
t0201028	23	1	GTATTGCACTTGTCCCGGCCTGT	miRNA	hsa-mir-92a-2
t0201464	18	1	TCAAAGTGCTTACAGTGC	miRNA	hsa-mir-17
t0201469	20	1	GAGGTAGTAGGTTGCATAGT	miRNA	hsa-let-7d
t0201514	21	1	TCAGAACAAATGCCGGTTCCC	miRNA	hsa-mir-589
t0202330	19	1	CCTGTACTGAGCTGCCCCG	miRNA	hsa-mir-486
t0202733	20	1	GCCCCTGGGCCTATCCTAGA	miRNA	hsa-mir-331
t0203307	19	1	TACCGCACTGTGGGTACTT	miRNA	hsa-mir-106b
t0203494	19	1	TTGTGGCTGGTCATGAGGC	miRNA	hsa-mir-4474
t0203725	21	1	TAGGTAGTTTCCTGTTGTTGG	miRNA	hsa-mir-196b
t0203758	20	1	AGGTAGTAGGTTGCATAGTT	miRNA	hsa-let-7d
t0204185	21	1	AAGTGCTGTTCGTGCAGGTAG	miRNA	hsa-mir-93
t0204200	19	1	ATAAAGTAGAAAGCACTAC	miRNA	hsa-mir-142
t0204335	19	1	CATCCTGTACTGAGCTGCC	miRNA	hsa-mir-486
t0204666	20	1	TTCTAATTTCTCCACGTCTT	miRNA	hsa-mir-576
t0204710	21	1	TCGGATCCGTCTGAGCTTGGC	miRNA	hsa-mir-127
t0206078	18	1	CAAAGTGCTCATAGTGCA	miRNA	hsa-mir-20b
t0206845	23	1	CGCATCCCCTAGGGCATTGGTGT	miRNA	hsa-mir-324
t0206945	22	1	GTGAGGTAGTAGATTGTATAGT	miRNA	hsa-let-7f-1

t0207200	19	1	ACAGTGAGGTAGAGGGAGT	miRNA	hsa-mir-3138
t0207585	20	1	CAGCATTGTACAGGGCTATG	miRNA	hsa-mir-103a-1
t0207738	23	1	ACTGGACTTGGAGTCAGAAGGCC	miRNA	hsa-mir-378a
t0207838	21	1	CCTCTCACCACTGCCCTCCCA	miRNA	hsa-mir-1229
t0207904	25	1	AACATTCAACGCTGTCGGTGAGTTT	miRNA	hsa-mir-181a-1
t0208092	21	1	CTAAAGTGCTTATAGTGCAGG	miRNA	hsa-mir-20a
t0208336	20	1	TCACCACCTTCTCCACCCAG	miRNA	hsa-mir-197
t0208613	18	1	CTATACAATCTACTGTCT	miRNA	hsa-let-7a-3
t0209184	20	1	TTTTCTTCTTAGACATGGCA	miRNA	hsa-mir-4659b
t0210513	22	1	AGGGGGAAAGTTCTATAGTCCT	miRNA	hsa-mir-625
t0210628	25	1	GATGAGGATGGATAGCAAGGAAGCC	miRNA	hsa-mir-3605
t0210727	18	1	TCAGTTATCACAGTGCTG	miRNA	hsa-mir-101-1
t0210785	21	1	ATGAGGTAGTAGGTTGTATAG	miRNA	hsa-let-7a-1
t0210799	19	1	CGGGGCCGTAGCACTGTCT	miRNA	hsa-mir-128-1
t0211590	20	1	ACTTAGCAGGTTGTATTATC	miRNA	hsa-mir-374b
t0211591	19	1	CCACCTCCCCTGCAAACGT	miRNA	hsa-mir-1306
t0211645	24	1	GGAACATTCAACGCTGTCGGTGAG	miRNA	hsa-mir-181a-2
t0212006	23	1	AAAGCTGCCAGTTGAAGAACTGT	miRNA	hsa-mir-22
t0212699	21	1	TTCATTGGACTGCTGATGGCC	miRNA	hsa-mir-4529
t0212795	20	1	AGGGAGCTGTAGAGCAGGGA	miRNA	hsa-mir-4732
t0212997	23	1	CGGCCCCACGCACCAGGGTAAGA	miRNA	hsa-mir-874
t0213046	20	1	TGCCTGAGGGAGTAAGAGTC	miRNA	hsa-mir-550a-3
t0213290	20	1	GAAGAACTGTTGCATTTGCC	miRNA	hsa-mir-4511
t0213493	21	1	GCTTTCAGTCGGATGTTTACA	miRNA	hsa-mir-30e
t0213617	22	1	AAAAGTAATTGTGGATTTTGCT	miRNA	hsa-mir-548ab
t0213771	22	1	ACAAAAGCAATCGCGGTTTTTG	miRNA	hsa-mir-548e
t0213813	19	1	CTATACAACTTACTACTTT	miRNA	hsa-mir-98
t0213905	20	1	ACGCCCTTCCCCCCCTTCTT	miRNA	hsa-mir-1249
t0213943	22	1	ACCATCGACCGTTGATTGTACC	miRNA	hsa-mir-181a-1
t0214046	23	1	AAAGTGCTTACAGTGCAGGTAGT	miRNA	hsa-mir-17
t0214067	20	1	TGAGGTAGGAGGTTGTATAG	miRNA	hsa-let-7e
t0214282	21	1	TCACACCTGCCTCGCCCCCCA	miRNA	hsa-mir-1228
t0214370	18	1	ACTGGCATTAGTGGGACT	miRNA	hsa-mir-5588
t0214468	19	1	AGGTAGTAGTTTGTACAGT	miRNA	hsa-let-7g
t0214881	21	1	CTAAAGTGCTGACAGTGCAGA	miRNA	hsa-mir-106b
t0214983	20	1	CACCTTGCGCTACTCAGGTC	miRNA	hsa-mir-3200
t0215197	22	1	AAGAGGTAGTAGGTTGCATAGT	miRNA	hsa-let-7d
t0215857	19	1	AAAGTGCTGTTCGTGCAGG	miRNA	hsa-mir-93
t0216060	24	1	TACAGTACTGTGATAACTGAAGGA	miRNA	hsa-mir-101-1
t0216220	23	1	AACGCATAATATGGACATGTTAT	miRNA	hsa-mir-3912
t0216250	19	1	TATTGCACATTACTAAGTT	miRNA	hsa-mir-32
t0216422	23	1	AAGAGGTAGTAGGTTGCATAGTT	miRNA	hsa-let-7d
t0216954	23	1	ACAACAGTGCCAACCTCACAGGA	miRNA	hsa-mir-1294
t0217095	22	1	ATGAGGATGGATAGCAAGGAAG	miRNA	hsa-mir-3605
t0217210	19	1	AAAGTGCTTACAGTGCAGG	miRNA	hsa-mir-106a
t0217411	20	1	TGGGACTGATCTTGATGTCT	miRNA	hsa-mir-3913-2
t0218178	18	1	AGTAATCCAGGATAGGCT	miRNA	hsa-mir-26a-2
t0218266	22	1	TCTCCCTTCCTGCCCTGGCTAG	miRNA	hsa-mir-4685
t0218280	19	1	GCTACATTGTCTGCTGGGT	miRNA	hsa-mir-221
t0218467	23	1	CTAGACTGAAGCTCCTTGAGGAC	miRNA	hsa-mir-151a
t0218573	21	1	ACACCAGTCGATGGGCTGTCT	miRNA	hsa-mir-21
t0218637	22	1	CGGGTAGAGAGGGCAGTGGGAG	miRNA	hsa-mir-197
t0218728	19	1	TCTCCCAACCCTTGTACCA	miRNA	hsa-mir-150
t0219034	19	1	AAGTGCTGACAGTGCAGAT	miRNA	hsa-mir-106b
t0219157	22	1	TCTTTTCTTTGAGACTCACTAC	miRNA	hsa-mir-627
t0219570	21	1	CTCCGTTTGCCTGTTTCGCTG	miRNA	hsa-mir-1468
t0219706	24	1	ATGGCACTGGTAGAATTCACTGTG	miRNA	hsa-mir-183
t0219908	19	1	TACTGTAGTATGGGCACTT	miRNA	hsa-mir-20b

t0220357	20	1	GTGCAAATCCATGCAAAACT	miRNA	hsa-mir-19b-2
t0221266	21	1	GCAGCACGTAAATATTGGCGT	miRNA	hsa-mir-16-2
t0221444	21	1	TAGTTCTTCAGTGGCAAGCTT	miRNA	hsa-mir-22
t0221541	20	1	GGAGGAACCTTGGAGCTTCG	miRNA	hsa-mir-3928
t0221903	20	1	TGCAAATCCATGCAAAACTG	miRNA	hsa-mir-19b-2
t0223159	20	1	TCAAAGTGCTTACAGTGCAG	miRNA	hsa-mir-17
t0223163	18	1	CACCTTGCGCTACTCAGG	miRNA	hsa-mir-3200
t0223344	23	1	TCCTCTTTTCTTTGAGACTCACT	miRNA	hsa-mir-627
t0223946	19	1	CCTCTCACCACTGCCCTCC	miRNA	hsa-mir-1229
t0224341	20	1	AACTATACAATCTATTGCCT	miRNA	hsa-let-7f-1
t0224392	22	1	CAAAACGTGAGGCGCTGCTATA	miRNA	hsa-mir-424
t0224432	21	1	AAGAGGTATAGGGCATGGGAA	miRNA	hsa-mir-202
t0224457	18	1	CGTAGATCCGAACTTGTG	miRNA	hsa-mir-100
t0224645	22	1	CGTCAACACTTGCTGGTTTCCT	miRNA	hsa-mir-505
t0225028	22	1	TAGCACCATCTGAAATCGGTTA	miRNA	hsa-mir-29a
t0226237	18	1	TATCAGACTGATGTTGAC	miRNA	hsa-mir-21
t0227115	18	1	CAAATCCATGCAAAACTG	miRNA	hsa-mir-19b-2
t0227632	22	1	AAAAGTAATTGTGGTTTTTGCC	miRNA	hsa-mir-548d-2
t0227773	18	1	TTGCACGGTATCCATCTG	miRNA	hsa-mir-363
